# Supplementary material for: Safety and parasite clearance of artemisinin-resistant Plasmodium falciparum infection: A pilot and a randomised volunteer infection study in Australia
Source: PLoS Med. 2020 Aug 21;17(8):e1003203. doi: 10.1371/journal.pmed.1003203 (PMC7444516; doi:10.1371/journal.pmed.1003203)
Supplement: S2 Text — (PDF) [file pmed.1003203.s002.pdf]

S2 Text. Eligibility criteria for the comparative study

Participant inclusion criteria

Participants eligible for inclusion in this study must fulfil **all** of the following criteria:

Demography

1. Adult (male and non-pregnant, non-lactating female) participants between 18 and 55 years of age inclusive, who do not live alone (from inoculation day until at least the end of the Malarone® treatment) and will be contactable and available for the duration of the trial and contactable up to 2 weeks following the End of Study visit (approximately 6 weeks).
2. Body weight minimum 50 kg, body mass index between 18 and 32 kg/m^2, inclusive.

Health status

3. Certified as healthy by a comprehensive clinical assessment (detailed medical history and complete physical examination).
4. Vital signs after 5 minutes resting in supine position:

   90 mmHg ≤ systolic blood pressure (SBP) ≤ 140 mmHg,

   50 mmHg ≤ diastolic blood pressure (DBP) ≤ 90 mmHg,

   40 bpm ≤ heart rate (HR) ≤ 100 bpm.
5. Normal standard 12-lead ECG after 5 minutes resting in supine position, QTcF≤450 msec with absence of second or third degree atrioventricular block or abnormal T wave morphology at screening and at pre-inoculation on inoculation day.
6. Laboratory parameters within the normal range, unless the Investigator considers an abnormality to be clinically irrelevant for healthy participants enrolled in this clinical investigation in accordance with approved clinically acceptable laboratory ranges documented prior to study start. More specifically, serum creatinine, hepatic transaminase enzymes (aspartate aminotransferase, alanine aminotransferase), and total bilirubin (unless the participant has documented Gilbert syndrome) should not exceed the approved acceptable ranges and hemoglobin must be equal or higher than the lower limit of the normal range.
7. Heterosexual women of childbearing potential should be surgically sterile or using an insertable, injectable, transdermal or combination oral contraceptive approved by the TGA combined with a barrier contraceptive for the duration of the study, and have negative results on a serum pregnancy test done before inoculation. Abstinent, heterosexual female participants must agree to start a double method if they start a sexual relationship during the study. Adequate contraception does not apply to participants of childbearing potential with same sex partners (abstinence from penile-vaginal intercourse), when this is their preferred and usual lifestyle. Female participants with same sex partners must not be planning *in vitro* fertilization within the required contraception period.

   Women of non-childbearing potential who will not require contraception during the study are defined as: post-menopausal (spontaneous amenorrhea for ≥ 12 months, or spontaneous amenorrhea for 6-12 months and follicle-stimulating hormone (FSH) ≥ 40 IU/mL; either should be together with the absence of oral contraceptive use for > 12 months).

Regulations

8. Having given written informed consent prior to undertaking any study-related procedure.

Participant exclusion criteria

Participants fulfilling **any** of the following criteria will not be eligible for inclusion in this study:

Medical history and clinical status

1. Any history of malaria or participation in a previous malaria challenge study.
2. Must not have travelled to or lived (>2 weeks) in a malaria-endemic region during the past 12 months or planned travel to a malaria-endemic region during the course of the study (for endemic regions see https://map.ox.ac.uk/country-profiles/#!/). Bali is not considered a malaria-endemic region.
3. Participation in any investigational product study within the 12 weeks preceding the study (i.e. there should be 12 weeks between the last dose of a previous investigational product and malaria parasite inoculation, at the Investigator’s discretion).
4. Has evidence of increased cardiovascular disease risk (defined as >10%, 5 year risk for those greater than 35 years of age, as determined by the Australian Absolute Cardiovascular Disease Risk Calculator (http://www.cvdcheck.org.au/)). Risk factors include sex, age, systolic blood pressure (mm/Hg), smoking status, total and HDL cholesterol (mmol/L), and reported diabetes status.
5. Symptomatic postural hypotension at screening, irrespective of the decrease in blood pressure, or asymptomatic postural hypotension defined as a decrease in systolic blood pressure ≥20 mmHg within 2-3 minutes when changing from supine to standing position.
6. History of splenectomy.
7. Presence or history of drug hypersensitivity, or allergic disease diagnosed by an allergist/immunologist and/or treated by a physician for allergy or history of a severe allergic reaction, anaphylaxis or convulsions following any vaccination or infusion.
8. Presence of current or suspected serious chronic diseases such as cardiac or autoimmune disease (HIV or other immuno-deficiencies), insulin-dependent and non-insulin dependent diabetes (excluding glucose intolerance if exclusion criterion 4 is met), progressive neurological disease, severe malnutrition, acute or progressive hepatic disease, acute or progressive renal disease, porphyria, psoriasis, rheumatoid arthritis, asthma, epilepsy, or obsessive compulsive disorder.
9. History of malignancy of any organ system (other than localised basal cell carcinoma of the skin or *in situ* cervical cancer), treated or untreated, within 5 years of screening, regardless of whether there is evidence of local recurrence or metastases.
10. Participants with history of schizophrenia, bi-polar disease, or other severe (disabling) chronic psychiatric diagnosis including depression or receiving psychiatric drugs or who has been hospitalised within the past 5 years prior to enrolment for psychiatric illness, history of suicide attempt, or confinement for danger to self or others.
11. History of serious psychiatric condition that may affect participation in the study or preclude compliance with the protocol, including but not limited to past or present psychoses, disorders requiring lithium, a history of attempted or planned suicide, more than one previous episode of major depression, any previous single episode of major depression lasting for or requiring treatment for more than 6 months, or any episode of major depression during the 5 years preceding screening.

    The Beck Depression Inventory will be used as an objective tool for the assessment of depression at screening. In addition to the conditions listed above, participants with a score of 20 or more on the Beck Depression Inventory and/or a response of 1, 2 or 3 for item 9 of this inventory (related to suicidal ideation) will not be eligible for participation. These participants will be referred to a general practitioner or medical specialist as appropriate. Participants with a Beck score of 17 to 19 may be enrolled at the discretion of the Investigator if they do not have a history of the psychiatric conditions mentioned in this criterion and their

mental state is not considered to pose additional risk to the health of the participant or to the execution of the study and interpretation of the data gathered.

12. Frequent headaches and/or migraines, recurrent nausea, and/or vomiting (more than twice a month).
13. Presence of acute infectious disease or fever (e.g. sublingual temperature ≥38.5°C) within the 5 days prior to inoculation with malaria parasites.
14. Evidence of acute illness within the 4 weeks prior to screening that the Investigator deems may compromise participant safety.
15. Significant inter-current disease of any type, in particular liver, renal, cardiac, pulmonary, neurologic, rheumatologic, or autoimmune disease by history, physical examination, and/or laboratory studies including urinalysis.
16. Participant has a clinically significant disease or any condition or disease that might affect drug absorption, distribution or excretion (e.g. gastrectomy, diarrhea).
17. Blood donation of any volume within 1 month before inclusion, or participation in any research study involving blood sampling (more than 450 mL/unit of blood), or blood donation to Australian Red Cross Blood Service (Blood Service) or other blood bank during the 8 weeks prior to the treatment drug dose in the study.
18. Participant unwilling to defer blood donations to the Blood Service for at least 6 months.
19. Medical requirement for intravenous immunoglobulin or blood transfusions.
20. Participant who has ever received a blood transfusion.
21. History or presence of alcohol abuse (alcohol consumption more than 40 g per day) or drug habituation, or any prior intravenous usage of an illicit substance.
22. Tobacco use of more than 5 cigarettes or equivalent per day, and unable to stop smoking for the duration of the clinical unit confinement.

Interfering substances

23. Any vaccination within the last 28 days.
24. Any corticosteroids, anti-inflammatory drugs, immunomodulators or anticoagulants. Any participant currently receiving or having previously received immunosuppressive therapy (including systemic steroids, adrenocorticotrophic hormone or inhaled steroids) at a dose or duration associated with hypothalamic-pituitary-adrenal axis suppression (e.g. 1 mg/kg/day prednisone, chronic use of inhaled high potency corticosteroids such as budesonide 800 μg/day or fluticasone 750 μg, or equivalent).
25. Any recent (<6 weeks) or current systemic therapy with an antibiotic or drug with potential antimalarial activity (e.g. chloroquine, piperaquine phosphate, benzodiazepine, flunarizine, fluoxetine, tetracycline, azithromycin, clindamycin, doxycycline etc.).
26. Ingestion of any poppy seeds within the 24 hours prior to the screening blood test (participants will be advised by phone not to consume any poppy seeds in this time period).
27. Excessive consumption of beverages or food containing xanthine bases including Red Bull, chocolate, coffee etc. (more than 400 mg caffeine per day, equivalent to more than 4 cups of coffee per day).
28. Unwillingness to abstain from consumption of grapefruit or Seville oranges from inoculation day until end of Malarone® treatment.
29. Unwillingness to abstain from consumption of quinine containing foods/beverages such as tonic water and lemon bitter, from inoculation day until end of Malarone® treatment.

30. Use of prescription drugs or non-prescription drugs or herbal supplements (such as St John's Wort), within 14 days or 5 half-lives (whichever is longer) prior to the malaria challenge agent inoculation. As an exception, ibuprofen (preferred) may be used at doses of up to 1.2 g/day, or paracetamol at doses of up to 4 g/day after discussion with the Investigator. Limited use of other non-prescription medications or dietary supplements, not believed to affect participant safety or the overall results of the study, may be permitted on a case-by-case basis following approval by the Sponsor in consultation with the Investigator. Participants are requested to refrain from taking non-approved concomitant medications from recruitment until the conclusion of the study.

General conditions

31. Any participant who, in the judgment of the Investigator, is likely to be noncompliant during the study, or is unable to cooperate because of a language problem or poor mental development.
32. Any participant in the exclusion period of a previous study according to applicable regulations.
33. Any participant who is the Principal Investigator or any sub-investigator, research assistant, pharmacist, study coordinator, or other staff thereof, directly involved in conducting the study.
34. Any participant without a good peripheral venous access.

Biological status

35. Positive result on any of the following tests: hepatitis B surface antigen (HBs Ag), anti-hepatitis B core antibodies (anti-HBc Ab), anti-hepatitis C virus (anti-HCV) antibodies, anti-human immunodeficiency virus 1 and 2 antibodies (anti-HIV1 and anti-HIV2 Ab).
36. Positive urine drug test. Any drug in the urine drug screen unless there is an explanation acceptable to the Investigator (e.g., the participant has stated in advance that they consumed a prescription or over-the-counter product which contained the detected drug) and/or the participant has a negative urine drug screen on retest by the pathology laboratory. Any participant testing positive for acetaminophen (paracetamol) at screening may still be eligible for study participation, at the Investigator's discretion.
37. Positive alcohol breath test.

Specific to the study

38. Cardiac/QT risk:
    - Family history of sudden death or of congenital prolongation of the QTc interval or known congenital prolongation of the QTc interval or any clinical condition known to prolong the QTc interval.
    - History of symptomatic cardiac arrhythmias or with clinically relevant bradycardia.
    - Electrolyte disturbances, particularly hypokalemia, hypocalcemia, or hypomagnesemia.
    - ECG abnormalities in the standard 12-lead ECG (at screening or at pre-inoculation on inoculation day) which in the opinion of the Investigator is clinically relevant or will interfere with the ECG analyses.
39. Known hypersensitivity to artesunate or any of its excipients, artemether or other artemisinin derivatives, piperaquine phosphate, proguanil/atovaquone, primaquine, or 4-aminoquinolines.
40. Known severe reaction to mosquito bites other than local itching and redness.
