# Supplementary material for: Safety and parasite clearance of artemisinin-resistant Plasmodium falciparum infection: A pilot and a randomised volunteer infection study in Australia
Source: PLoS Med. 2020 Aug 21;17(8):e1003203. doi: 10.1371/journal.pmed.1003203 (PMC7444516; doi:10.1371/journal.pmed.1003203)
Supplement: S3 Text — (PDF) [file pmed.1003203.s003.pdf]

S3 Text. Summary testing of the artemisinin-resistant ($K13^{R539T}$) *P. falciparum* master cell bank

Characterisation of the artemisinin-resistant ($K13^{R539T}$) master cell bank

The tests were performed to characterize the artemisinin-resistant ($K13^{R539T}$) *P. falciparum* master cell bank are summarised below.

Parasitaemia

The percentage parasitaemia (defined as the percent of parasitized red blood cells) and the percentage of ring stage parasites were determined by malaria thin film smears [1].

At the time of release of the artemisinin-resistant master cell bank, the percentage parasitaemia was 5%, and the percentage of ring stage parasites was 82%.

Parasite viability

The percentage of viable ring stage parasites was determined by flow cytometry using a two-colour flow cytometric method [2]. This method uses SYBR® Green I (SYBR Green, Invitrogen, Australia), a nucleic acid dye that indiscriminately stains DNA in both viable and non-viable or compromised parasites within erythrocytes, and MitoTracker® deep red FM (MTDR, Invitrogen, Australia), a membrane permeable dye that selectively stains only live parasites with intact membrane potential. Briefly, 25 µL of a suspension containing artemisinin-resistant parasitised red blood cells (RBCs) at 2.4% haematocrit were added to polypropylene FACS tubes in triplicate. Uninfected RBCs were used as controls. RBCs were washed in 2 mL of Dulbecco's Phosphate Buffered Saline (DPBS) by centrifugation at 525×*g* for 5 min at room temperature. Supernatant was aspirated gently to ensure RBC pellet was not disturbed. RBC pellets were then gently resuspended in 1 mL FACS buffer (DPBS/2% Foetal Calf Serum [FCS]) and centrifuged at 525×*g* for 5 min at room temperature. Supernatant was gently aspirated and discarded. Then, 100 µL of viability stain was added to each tube to give a final concentration of 0.2×SYBR Green and 0.3 µM MTDR, and samples were incubated at 37°C for 40 min, protected from light. Following incubation, RBCs were washed in 2 mL DPBS by centrifugation at 525×*g* for 5 min at room temperature. Supernatant was aspirated and RBC pellets were resuspended in 250 µL FACS buffer. Samples were transferred to a polystyrene FACS tube and immediately acquired on an LSR Fortessa 5 Flow Cytometer Analyser (BD Biosciences), equipped with 488 nm blue and 640 nm red laser. The percentage of SYBR Green positive cells was determined using green fluorescence (B530/30 bandpass filter) plots. The percentage of MTDR positive cells was determined using red fluorescence (R670/30 bandpass filter) plots. Viable rings stage parasites were gated on a green vs red fluorescence plot based on their low intensity of SYBR Green and MTDR positivity. Data was analysed using FlowJo Software (Treestar, Version 10, USA).

The percentage of viable ring stage parasites of the artemisinin-resistant master cell bank at the time of release was 54%.

In vitro antimalarial drug resistance testing of the artemisinin-resistant ($K13^{R539T}$) master cell bank

In vitro antimalarial drug resistance testing was performed on the artemisinin-resistant master cell bank using the [^{3}H]-hypoxanthine uptake inhibition assay, as previously described [3,4]. *P. falciparum* strains 3D7 (artemisinin and chloroquine sensitive) and W2 (chloroquine resistant) were used as controls. The assay was repeated twice and reproducible results were obtained. The mean IC_{50} results for each antimalarial drug are presented in Table A. In this assay, the artemisinin-resistant master cell bank was resistant to chloroquine, lumefantrine, mefloquine, and quinine, whereas it was sensitive to piperaquine, amodiaquine, atovaquone, and pyronaridine.

Ring-stage survival assay testing of the artemisinin-resistant ($K13^{R539T}$) master cell bank

The ring-stage survival assay was performed as previously described [5]. The assay was performed twice.

The ring-stage survival of the artemisinin-resistant master cell bank was 28.6% (SD 5.44). Parasites with >1% survival in this assay are considered artemisinin-resistant [6].

References

1. Moll K, Kaneko A, Scherf A, Wahlgren M, editors. Methods in malaria research. 6th ed. Glasgow, UK: EVIMalaR; 2013.
2. Amaratunga C, Neal AT, Fairhurst RM. Flow cytometry-based analysis of artemisinin-resistant Plasmodium falciparum in the ring-stage survival assay. Antimicrob Agents Chemother. 2014;58(8):4938-40.
3. Pawliw R, Farrow R, Sekuloski S, Jennings H, Healer J, Phuong T, et al. A bioreactor system for the manufacture of a genetically modified Plasmodium falciparum blood stage malaria cell bank for use in a clinical trial. Malar J. 2018;17(1):283.
4. WWARN. Estimation of Plasmodium falciparum drug susceptibility by the ^{3}H-hypoxanthine uptake inhibition assay. 2012.
5. Witkowski B, Amaratunga C, Khim N, Sreng S, Chim P, Kim S, et al. Novel phenotypic assays for the detection of artemisinin-resistant Plasmodium falciparum malaria in Cambodia: in-vitro and ex-vivo drug-response studies. Lancet Infect Dis. 2013;13(12):1043-9.
6. Fairhurst RM, Dondorp AM. Artemisinin-resistant Plasmodium falciparum malaria. Microbiol Spectr. 2016;4(3).
