# Supplementary material for: Safety and parasite clearance of artemisinin-resistant Plasmodium falciparum infection: A pilot and a randomised volunteer infection study in Australia
Source: PLoS Med. 2020 Aug 21;17(8):e1003203. doi: 10.1371/journal.pmed.1003203 (PMC7444516; doi:10.1371/journal.pmed.1003203)
Supplement: S4 Text — AS, artesunate; DHA, dihydroartemisinin. (PDF) [file pmed.1003203.s004.pdf]

S4 Text. Determination of artesunate and dihydroartemisinin concentrations in plasma samples

Sample timepoints

Plasma concentrations of artesunate and dihydroartemisinin (DHA) were measured in blood samples collected pre-artesunate administration and at 0.25, 0.5, 1, 1.5, 2, 2.5, 3, 4, 6, 8, 10, and 12 hours post-artesunate.

Plasma sample preparation

A total of 300 µL of methanol containing 200 µg/L internal standards (artesunate-d4 and dihydroartemisinin-d3) were added to 100 µL of plasma sample in a 2 mL deep 96-well plate. A cap mat was placed on top of the plate that was then vortexed for 2 min and centrifuged for 10 min at 3500 rpm. The plate was then transferred to the sample manager and 5 µL of the supernatant injected into the column.

Liquid chromatography and mass spectrometry

The concentration of artesunate and DHA in plasma samples was analysed in an ultra performance liquid chromatography system (Acquity) with a BEH C18 chromatography column of 1.7 µm particle size, 2.1 mm x 50 mm (Acquity). MassLynx V4.1 software was used for analysis. Samples (5 µL) were injected into the column and chromatographically resolved with 2 mM ammonium acetate and 0.1% formic acid (mobile phase A) and methanol with 2 mM ammonium acetate and 0.1% formic acid (mobile phase B) at a flow rate of 0.4 mL/min and a column temperature of 45°C. The initial conditions were 20% mobile phase A / 80% mobile phase B. A linear gradient was applied to reach 12% mobile phase A / 88% mobile phase B at 1.0 min. The column was then washed with 100% mobile phase B from 1.01 to 1.75 min before returning to the initial conditions. The run time was 2 min/sample.

A Micromass Quattro Premier XE mass spectrometer was used (Waters, Milford, USA). Ionization was achieved with positive mode electron spray and the mass transitions (m/z) were monitored in multiple-reaction-monitoring acquisition mode. The transitions of the parent / daughter ions were: artesunate (402.2>267.3 m/z, 402.2>163.0 m/z), artesunate-d4 (406.3>267.2 m/z), DHA (267.3>145.0 m/z, 367.3>163.1 m/z) and DHA-d3 (270.3>148.0 m/z). The dwell time was 0.02 s, cone voltage 10 V and the collision energy 15 eV. The capillary charge was 0.8 kV, the source temperature 120°C, the desolvation temperature 425°C, the desolvation gas flow 900 L/h with cone gas flow at 25 L/h and the collision cell gas flow (argon) 0.30 mL/min.

The sensitivity of the assay was 1 to 1000 µg/L for both artesunate and DHA. The coefficient of variation across 3 different concentration levels was <8.5% for the intra-assay and <11% for the inter-assay. Extracted samples were stable for at least 24 hours when stored at 10°C. Both artesunate and DHA did not exhibit any degradation across 3 freeze thaw cycles, and autosampler carryover was assessed as <0.01%.
