# Supplementary material for: Safety and parasite clearance of artemisinin-resistant Plasmodium falciparum infection: A pilot and a randomised volunteer infection study in Australia
Source: PLoS Med. 2020 Aug 21;17(8):e1003203. doi: 10.1371/journal.pmed.1003203 (PMC7444516; doi:10.1371/journal.pmed.1003203)
Supplement: S5 Text — (PDF) [file pmed.1003203.s005.pdf]

**S5 Text. Parasite growth dynamics**

The parasite growth dynamics were estimated by the sine-wave model [1].

$$\log_{10}(Y) = a + m \times time + c \times sin\left(\left(2 \times \frac{\pi}{period}\right) \times time + k\right),$$

where $Y$ = parasites per mL measured by qPCR, $a$ = intercept, $m$ = parasite growth rate, $c$ = amplitude of the sine-wave, $period$ = parasite lifecycle duration in days, $time$ = days from inoculation, and $k$ = phase shift in sine-wave.

The model was fit to each of the 2 participants in the pilot study using non-linear regression and fit to the comparative study using a non-linear mixed effects model with a random effect at the cohort level for $a$ and $k$, and the participant nested within cohort level for $a$.

Summary of the parameters estimated for the two participants in the pilot study was estimated by using the inverse variance method to calculate the weighted mean of the parameter and corresponding 95% confidence interval. The weight was based on the standard error of the corresponding parameter, $w_i = \frac{1}{(SE_i)^2}$, and the standard error of the weighted mean was derived by $\sqrt{\frac{1}{\sum_{i=1}^{n} w_i}}$.

**References**

1. Simpson JA, Aarons L, Collins WE, Jeffery GM, White NJ. Population dynamics of untreated Plasmodium falciparum malaria within the adult human host during the expansion phase of the infection. Parasitology. 2002;124(Pt 3):247-63.
