# Supplementary material for: Safety and parasite clearance of artemisinin-resistant Plasmodium falciparum infection: A pilot and a randomised volunteer infection study in Australia
Source: PLoS Med. 2020 Aug 21;17(8):e1003203. doi: 10.1371/journal.pmed.1003203 (PMC7444516; doi:10.1371/journal.pmed.1003203)
Supplement: S1 Fig — (PDF) [file pmed.1003203.s006.pdf]

S1 Fig. Individual parasite growth and clearance profiles in the pilot study

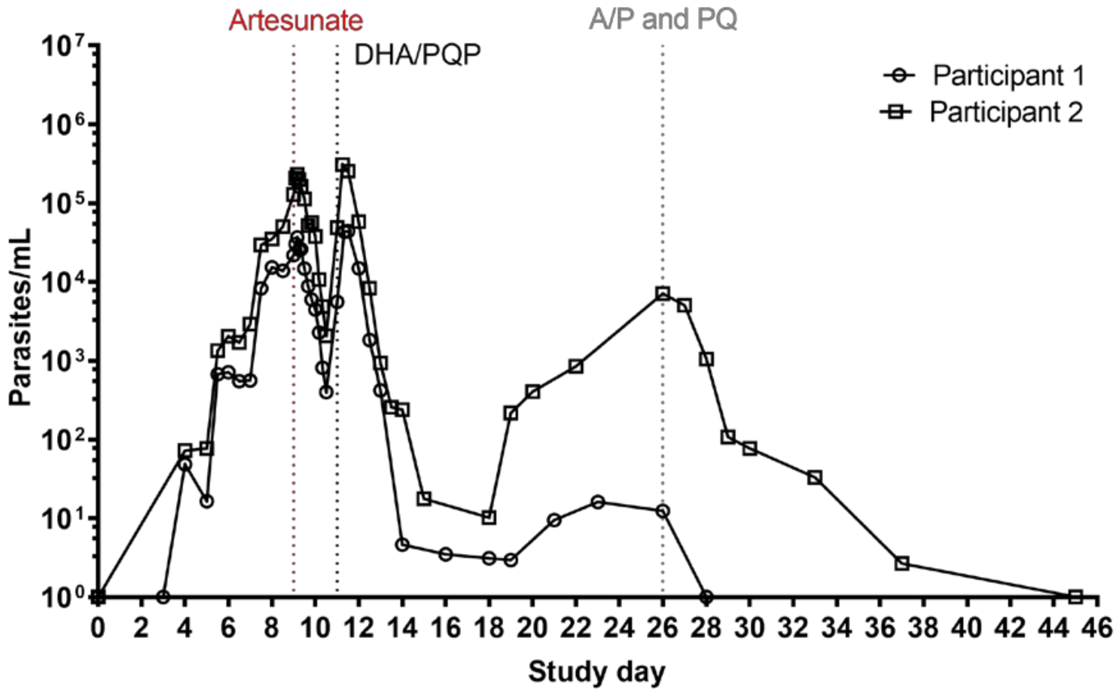

Parasitaemia during the pilot study as measured by 18S qPCR. Participants were inoculated with artemisinin-resistant parasites on Day 0 and administered artesunate on Day 9 (red dashed line), dihydroartemisinin/piperaquine phosphate (DHA/PQP) on Day 11 (black dashed line), and atovaquone/proguanil (A/P) and primaquine (PQ) on Day 26 (grey dashed line).
