# Supplementary material for: Safety and parasite clearance of artemisinin-resistant Plasmodium falciparum infection: A pilot and a randomised volunteer infection study in Australia
Source: PLoS Med. 2020 Aug 21;17(8):e1003203. doi: 10.1371/journal.pmed.1003203 (PMC7444516; doi:10.1371/journal.pmed.1003203)
Supplement: S2 Fig — (PDF) [file pmed.1003203.s007.pdf]

S2 Fig. Parasite lifecycle duration of artemisinin-resistant parasites in the pilot study

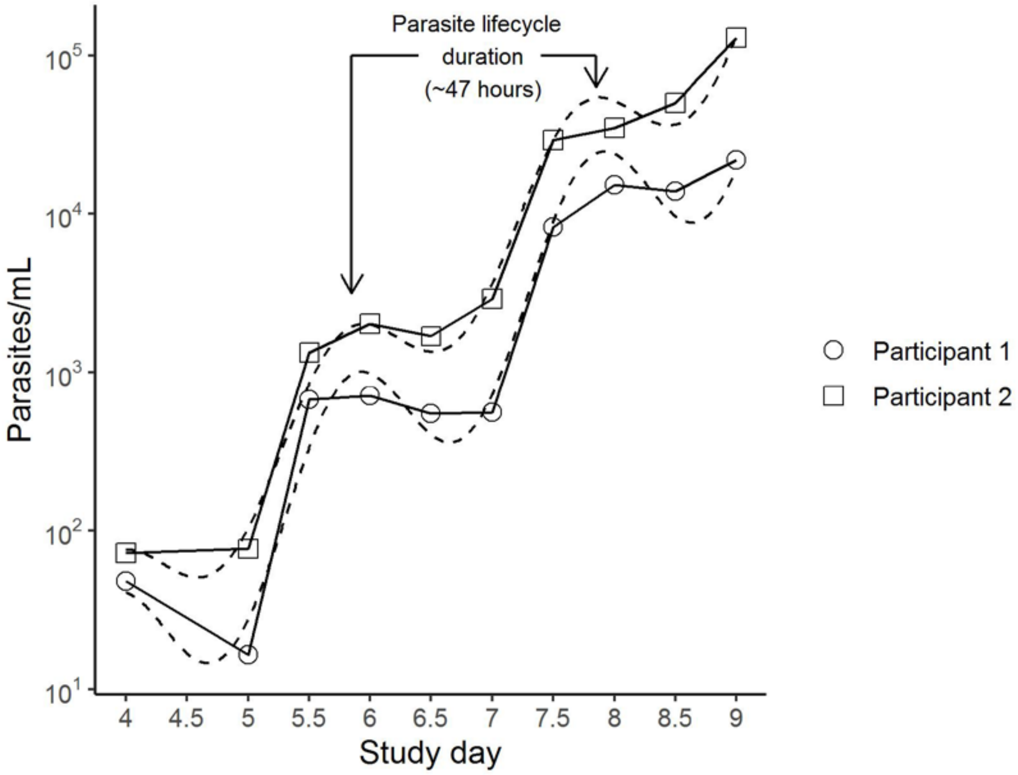

A sine-wave growth model was applied to the pre-artesunate 18S qPCR data for each participant (black solid line) in the pilot study to estimate the parasite lifecycle duration of the artemisinin-resistant parasites (black dashed line). The weighted mean parasite lifecycle duration of artemisinin-resistant parasites prior to artesunate administration was 46.7 hours (95% confidence interval: 43.8–49.6 hours). The 2 participants in the pilot study were inoculated 3 weeks apart with different artemisinin-resistant parasite inocula. Parasite growth was similar in the 2 participants.
