# Supplementary material for: Safety and parasite clearance of artemisinin-resistant Plasmodium falciparum infection: A pilot and a randomised volunteer infection study in Australia
Source: PLoS Med. 2020 Aug 21;17(8):e1003203. doi: 10.1371/journal.pmed.1003203 (PMC7444516; doi:10.1371/journal.pmed.1003203)
Supplement: S3 Fig — (PDF) [file pmed.1003203.s008.pdf]

S3 Fig. Sine-wave growth model for artemisinin-resistant and artemisinin-sensitive parasites in the comparative study

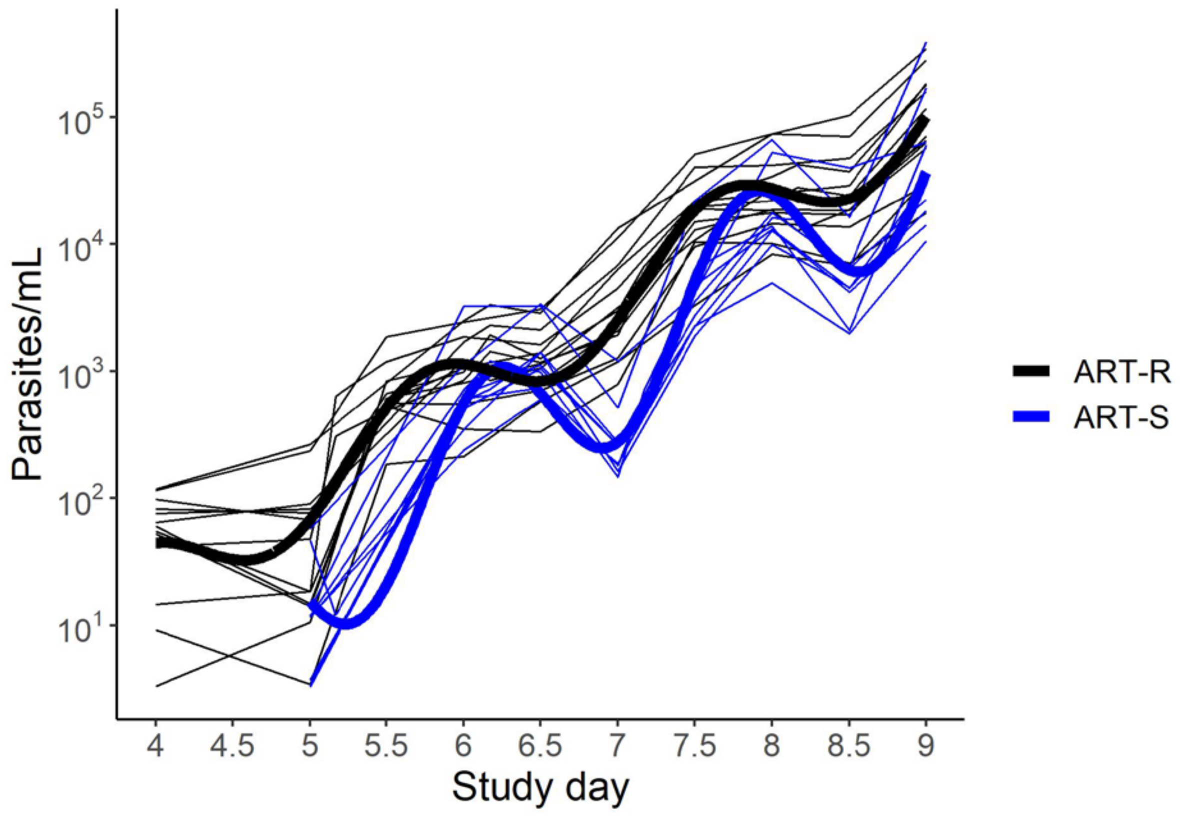

Participants were inoculated on Day 0 with artemisinin-resistant (ART-R) parasites or on Day 1 with artemisinin-sensitive (ART-S) parasites. The artemisinin-resistant and artemisinin-sensitive parasites were in a similar stage of development at the time of artesunate administration on Day 9. Thin lines represent individual participant data; thick lines represent the estimated sine-wave growth model for artemisinin-resistant (black lines) and artemisinin-sensitive (blue lines) parasites from a sine-wave mixed effects model, which included a random intercept for each participant, and random effects for the intercept and phase shift parameters at the cohort level.
