# Supplementary material for: Safety and parasite clearance of artemisinin-resistant Plasmodium falciparum infection: A pilot and a randomised volunteer infection study in Australia
Source: PLoS Med. 2020 Aug 21;17(8):e1003203. doi: 10.1371/journal.pmed.1003203 (PMC7444516; doi:10.1371/journal.pmed.1003203)
Supplement: S4 Fig — AS, artesunate; DHA, dihydroartemisinin. (PDF) [file pmed.1003203.s009.pdf]

S4 Fig. Plasma concentrations of artesunate and DHA in the pilot study

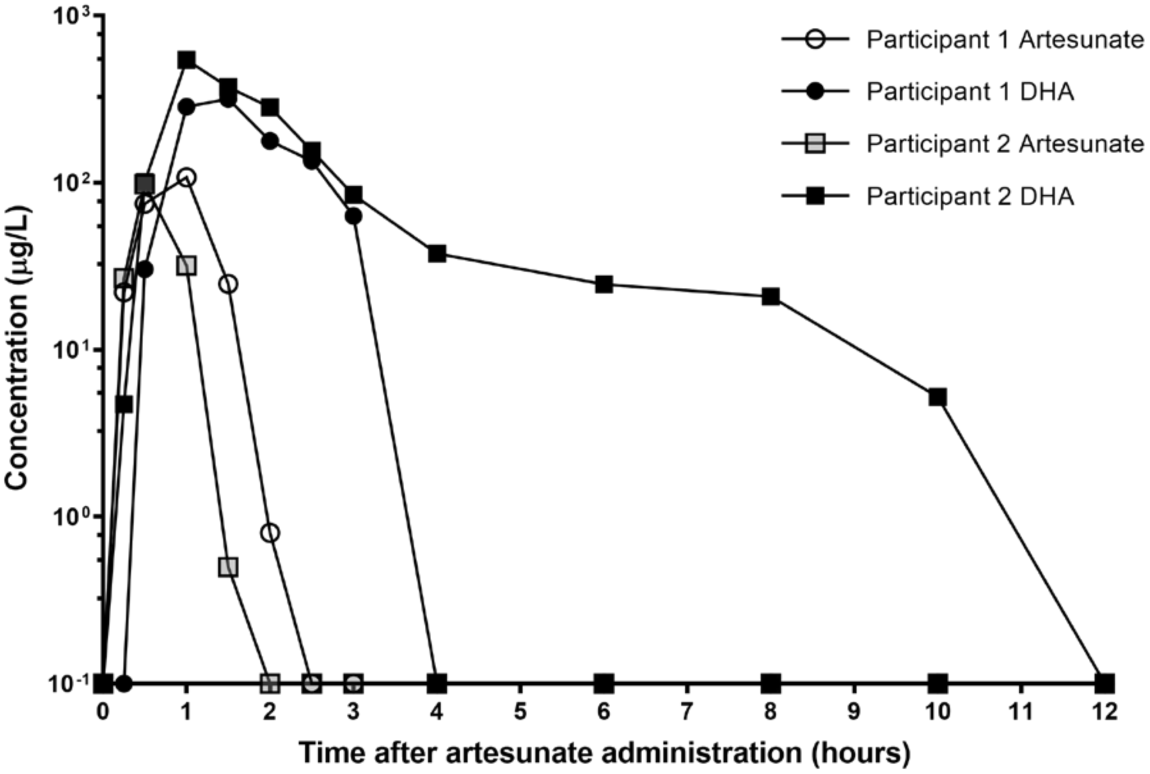

Plasma concentrations of artesunate and dihydroartemisinin (DHA) for each participant in the pilot study. Plasma concentrations of 0 were replaced with 0.1 µg/L for purposes of plotting on the logarithmic scale. The lower limit of quantification for artesunate and DHA was 1 µg/L.
