# Supplementary material for: Safety and parasite clearance of artemisinin-resistant Plasmodium falciparum infection: A pilot and a randomised volunteer infection study in Australia
Source: PLoS Med. 2020 Aug 21;17(8):e1003203. doi: 10.1371/journal.pmed.1003203 (PMC7444516; doi:10.1371/journal.pmed.1003203)
Supplement: S5 Fig — AS, artesunate; DHA, dihydroartemisinin. (PDF) [file pmed.1003203.s010.pdf]

S5 Fig. Geometric mean dose-normalised plasma concentration of artesunate and DHA in the comparative study

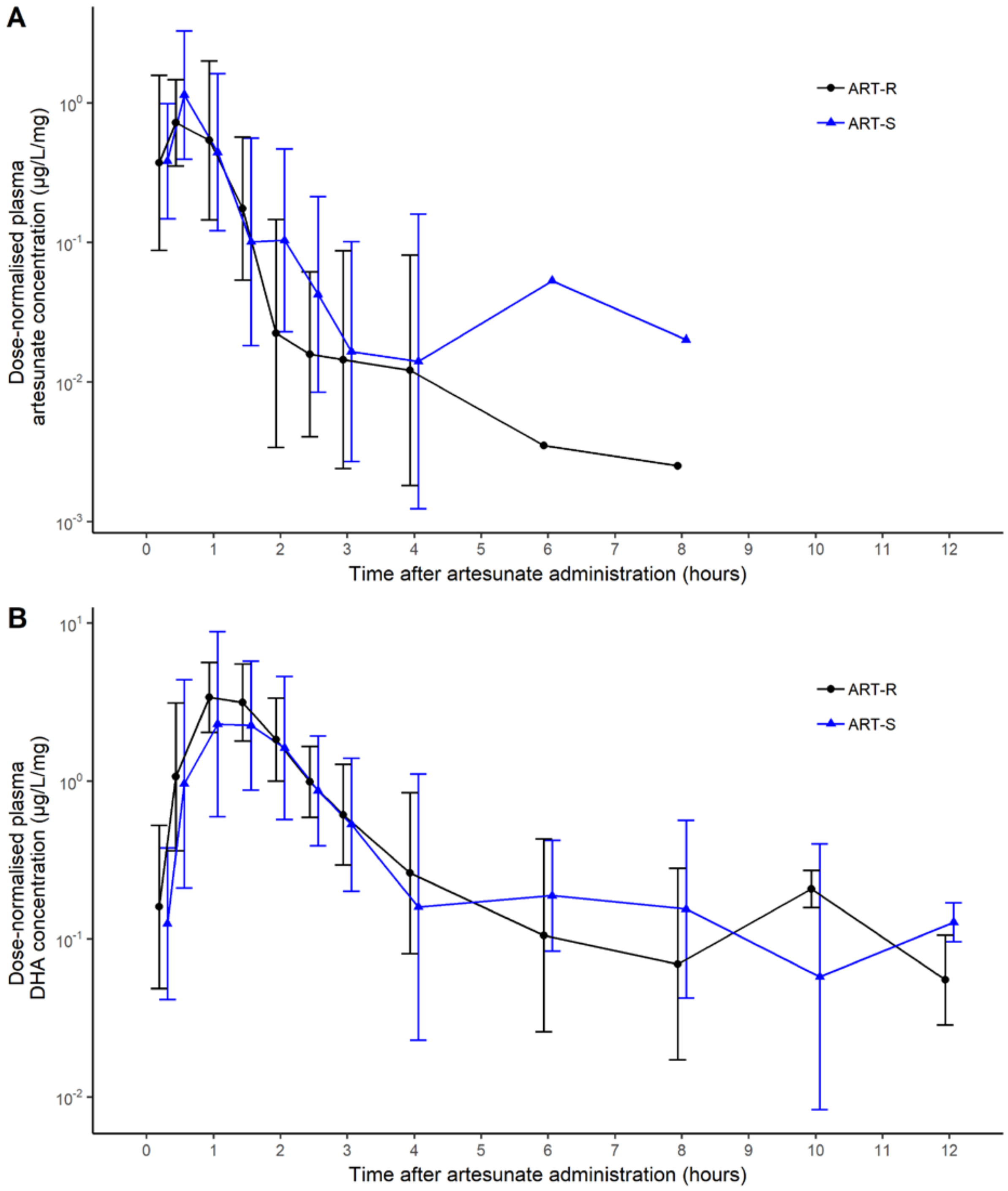

Dose-normalised plasma concentration of artesunate (Panel A) and dihydroartemisinin (DHA) (Panel B). Error bars present geometric standard deviation.
