# Supplementary material for: Safety and parasite clearance of artemisinin-resistant Plasmodium falciparum infection: A pilot and a randomised volunteer infection study in Australia
Source: PLoS Med. 2020 Aug 21;17(8):e1003203. doi: 10.1371/journal.pmed.1003203 (PMC7444516; doi:10.1371/journal.pmed.1003203)
Supplement: S1 Table — (PDF) [file pmed.1003203.s011.pdf]

S1 Table. In vitro antimalarial drug resistance testing of the artemisinin-resistant ($K13^{R539T}$) *P. falciparum* master cell bank

Antimalarial drug	Mean IC_{50} [nM] (95% CI)					
	Test 1			Test 2		
	3D7 (artemisinin and chloroquine sensitive)	W2 (chloroquine resistant)	Artemisinin-resistant MCB	3D7 (artemisinin and chloroquine sensitive)	W2 (chloroquine resistant)	Artemisinin-resistant MCB
**Dihydroartemisinin**	2.79 (2.01–3.88)	2.86 (1.86–4.40)	4.57 (2.93–7.11)	1.99 (1.54–2.57)	1.42 (1.05–1.90)	4.90 (3.26–7.38)
**Piperaquine**	15.11 (8.72–26.16)	53.39 (34.19–83.36)	17.79 (12.39–25.55)	12.62 (7.60–20.97)	28.49 (19.45–41.74)	13.24 (8.94–19.61)
**Lumefantrine**	130.6 (92.53–184.5)	17.27 (11.94–24.98)	177.7 (142.8–221.3)	167.9 (124.3–226.7)	23.1 (15.70–33.99)	291.4 (210.2–404.1)
**Desethylamodiaquine**	8.72 (4.81–15.78)	55.47 (34.48–89.23)	24.6 (16.54–36.60)	8.71 (4.72–16.06)	38.93 (22.06–68.70)	22.05 (13.92–34.93)
**Chloroquine**	13.59 (7.91–23.32)	224.4 (139.7–360.5)	137.2 (86.39–217.9)	6.20 (3.51–10.92)	166.0 (104.3–264.2)	99.03 (65.78–149.1)
**Atovaquone**	1.51 (1.34–1.70)	2.22 (1.93–2.56)	0.37 (0.33–0.41)	1.74 (1.52–1.99)	3.68 (3.14–4.32)	0.79 (0.74–0.85)
**Mefloquine HCl**	32.64 (21.99–48.45)	8.21 (5.43–12.40)	74.14 (60.87–90.30)	44.24 (29.68–65.95)	7.19 (4.90–10.56)	95.63 (77.86–117.4)
**Quinine HCl**	47.55 (36.45–62.03)	244.2 (165.4–360.5)	454.3 (326.9–631.3)	95.48 (70.47–129.4)	316.4 (219.0–457.1)	666.1 (461.7–961.1)
**Pyronaridine**	20.05 (8.58–46.86)	26.69 (10.75–66.27)	9.10 (6.11–13.53)	6.35 (3.24–12.45)	5.29 (3.04–9.23)	5.18 (3.56–7.53)

P. falciparum strains 3D7 (artemisinin and chloroquine sensitive) and W2 (chloroquine resistant) were used as controls. CI: confidence interval; IC_{50}: half maximal inhibitory concentration; MCB: master cell bank.
