# Supplementary material for: Safety and parasite clearance of artemisinin-resistant Plasmodium falciparum infection: A pilot and a randomised volunteer infection study in Australia
Source: PLoS Med. 2020 Aug 21;17(8):e1003203. doi: 10.1371/journal.pmed.1003203 (PMC7444516; doi:10.1371/journal.pmed.1003203)
Supplement: S2 Table — (PDF) [file pmed.1003203.s012.pdf]

S2 Table. Schedule of events for the pilot study

**Procedures**	**Screening**	**Safety visit (if required)**	**Malaria inoculation**	**Malaria monitoring**		**Drug administration**			**Safety monitoring**	**DHA/PQP (if required)**	**A/P**	**Follow -up visit**	**Follow-up phone call**	**EOS visit**
**Day**	**-D28 to -D1**	**-D3 to -D1**	**D0**	**D1 to D3**	**Daily from D4 until qPCR +ve and then AM & PM until admission**	**Admission approx. D8**	**Confinement at clinical unit**	**Exit from unit**	**Post-confinement**	**Post-confinement**	**D26±3**	**D28±3**	**D56±7**	**D90±14**
Informed consent & eligibility	X		X											
Medical history	X		X									X		X
Physical examination	X		X[a]		X[b]	X[a]	X[b]	X[a]	X[b]			X		X[a]
ECG	X		X			X				X		X		
Vital signs	X		X		X	X	X	X	X	X	X	X		X
Hematology & biochemistry	X	X				X		X	X	X	X	X		
Serology	X											X		
Red cell alloantibody	X											X		X
G6PD testing	X													
Urinalysis	X	X				X			X			X		
Drug & alcohol screen	X		X			X								
Phone call				X							X		X	
Blood stage parasite inoculation			X											
Unit confinement						X	X							
Drug administration							X			X	X			
Adverse events			X	X	X	X	X	X	X	X	X	X	X	X
Malaria qPCR			X		X	X	X	X	X	X		X		

Gametocyte qRT-PCR (if required)						X	X	X	X			X		
Artesunate and dihydroartemisinin concentration						X	X							
Safety serum storage			X									X		X[c]

[a] Abbreviated physical examination.

[b] Abbreviated physical examination if clinically indicated.

[c] Two serum samples to be taken at Day 90±14 (one serum sample at other time-points).

A/P: atovaquone/proguanil; DHA/PQP: dihydroartemisinin/piperaquine; ECG: electrocardiogram; EOS: End of Study; G6PD: glucose-6-phosphate dehydrogenase; qPCR: quantitative polymerase chain reaction; qRT-PCR: reverse transcription qPCR.
