# Supplementary material for: Safety and parasite clearance of artemisinin-resistant Plasmodium falciparum infection: A pilot and a randomised volunteer infection study in Australia
Source: PLoS Med. 2020 Aug 21;17(8):e1003203. doi: 10.1371/journal.pmed.1003203 (PMC7444516; doi:10.1371/journal.pmed.1003203)
Supplement: S3 Table — (PDF) [file pmed.1003203.s013.pdf]

S3 Table. Schedule of events for the comparative study

Procedures	Screening	Safety visit[a]	Malaria inoculation	Post-inoculation phone call or text message	Malaria monitoring[b]	Artesunate and clinical unit confinement[c]	Outpatient monitoring[c]	EOS visit
	**D-28 to D-1**	**D-3 to D-1**	**Artemisinin-resistant group: D0 Artemisinin-sensitive group: D1**	**Artemisinin-resistant group: D1 to D3 Artemisinin-sensitive group: D2 to D4**	**Artemisinin-resistant group: D4 to D8 Artemisinin-sensitive group: D5 to D8**	**Approx. D9 to D12**	**D13 to D27**	**D28±3**
Informed consent & BDI	X							
Medical history, eligibility & prior medications	X		X					
Drug & alcohol screen	X		X			X[k]		
Full physical examination	X							X
Abbreviated (Cohort 1) or symptom-directed physical examination (Cohorts 2 and 3)[d]			X		X	X	X	
Vital sign assessment	X		X		X	X	X	X
ECG	X		X			X[k]	X[m]	X
Urinalysis	X	X				X[k]	X	X
Hematology & biochemistry	X	X				X[l]	X	X
G6PD testing	X							
Red cell alloantibody	X							X
Serology	X							X
Pregnancy test[e]	X		X			X[k]		X
Safety serum storage			X					X
Adverse events & concomitant medications			X	X	X	X	X	X
Malaria clinical score			X		X	X	X[n]	
Malaria 18S qPCR blood sampling			X		X	X	X	X
Parasite lifecycle stage qRT-PCR blood sampling[f]						X	X	X
Artesunate and dihydroartemisinin concentration						X		
Malaria transmission assays[g]							X	
Artesunate administration						X		
Piperaquine phosphate administration[h]						X	X	
A/P administration[i]							X	
Primaquine administration[j]							X	

[a] An additional safety visit will occur between Day -3 and Day -1 to collect samples for hematology, biochemistry and urinalysis, unless screening laboratory assessments were conducted within this period.

[b] Daily visits until qPCR positive, and then twice daily visits until artesunate administration.

[c] Approximate days based on threshold for artesunate administration, which is expected to occur on Day 9 for both the artemisinin-resistant group and artemisinin-sensitive group.

[d] Abbreviated physical examinations (Cohort 1) or symptom-directed physical examinations (Cohorts 2 and 3) should be performed prior to inoculation, upon admission to the clinical unit for artesunate administration, and upon exit of the clinical unit. For Cohort 3, the symptom-directed physical examination at exit of the clinical unit will be performed at the Investigator’s discretion. At all other times, abbreviated physical examinations (Cohort 1) or symptom-directed physical examinations (Cohorts 2 and 3) will only be performed when signs or symptoms of malaria are identified and it is clinically indicated.

[e] Serum β-hCG pregnancy test (all female participants) and follicle stimulating hormone test (post-menopausal females) at screening. Urine β-hCG pregnancy test for all female participants at other specified time-points.

[f] If required, at the Investigator’s discretion.

[g] May be performed in selected cohorts at the Investigator’s discretion.

[h] Piperaquine phosphate will be administered if required. Artemisinin-resistant infected participants in Cohorts 2 and 3 will be administered piperaquine phosphate on Day 11 or earlier at the Investigator’s discretion.

[i] A/P administration will occur on Day 26±3 (Cohort 1), Day 25±3 (Cohorts 2 and 3), or earlier at the Investigator’s discretion.

[j] Primaquine administration if required (Cohort 1) or at Day 23±3 or earlier at the Investigator’s discretion (Cohorts 2 and 3).

[k] At time of admission to clinical unit. Artemisinin-resistant infected participants in Cohorts 2 and 3 administered piperaquine phosphate on Day 11 AM will have ECG testing performed prior to piperaquine administration.

[l] At time of admission and exit from clinical unit. Artemisinin-resistant infected participants in Cohorts 2 and 3 administered piperaquine phosphate on Day 11 will have blood samples collected for haematology and biochemistry prior to piperaquine administration.

[m] Only if piperaquine phosphate administration is required, ECG testing will occur prior to, and approximately 3 days after, piperaquine administration.

[n] Only if vital signs are abnormal, or at the Investigator’s discretion.

A/P: atovaquone/proguanil; BDI: Beck Depression Inventory; ECG: electrocardiogram; EOS: End of Study; G6PD: glucose-6-phosphate dehydrogenase; qPCR: quantitative polymerase chain reaction, qRT-PCR: reverse transcription qPCR.
