# Supplementary material for: Safety and parasite clearance of artemisinin-resistant Plasmodium falciparum infection: A pilot and a randomised volunteer infection study in Australia
Source: PLoS Med. 2020 Aug 21;17(8):e1003203. doi: 10.1371/journal.pmed.1003203 (PMC7444516; doi:10.1371/journal.pmed.1003203)
Supplement: S4 Table — AS, artesunate; qPCR, quantitative PCR. (PDF) [file pmed.1003203.s014.pdf]

S4 Table. Malaria 18S qPCR timepoints from pre-artesunate to 84 hours post-artesunate administration

Pilot study malaria 18S qPCR timepoints (h)*	Comparative study malaria 18S qPCR timepoints (h)*		
	Cohort 1	Cohort 2	Cohort 3
0	0	0	0
2	2	2	-
4	4	4	4
6	6	6	-
8	8	8	8
12	12	12	12
16	16	16	16
20	20	20	20
24	24	24	24
28	28	28	28
32	32	32	32
36	36	36	36
-	-	-	40
-	-	-	44
48	48	48	48
-	-	52†	52
-	-	56†	56
60	60	60	60
-	-	-	64
-	-	-	68
72	72	72	72
84	84	84	84

*Timepoints are provided as hours post artesunate administration; the sample at timepoint 0 h was collected before artesunate administration. †artemisinin-resistant infected participants only.
