# Supplementary material for: Safety and parasite clearance of artemisinin-resistant Plasmodium falciparum infection: A pilot and a randomised volunteer infection study in Australia
Source: PLoS Med. 2020 Aug 21;17(8):e1003203. doi: 10.1371/journal.pmed.1003203 (PMC7444516; doi:10.1371/journal.pmed.1003203)
Supplement: S5 Table — PQ, primaquine. (PDF) [file pmed.1003203.s015.pdf]

S5 Table. Gametocytaemia prior to primaquine administration

	Participant number	*P. falciparum* strain	Day of qRT-PCR	Day of primaquine administration	Female gametocytes/mL	Male gametocytes/mL
**Pilot study**	Participant 1	$K13^{R539T}$	D26	D26	200	NT
	Participant 2	$K13^{R539T}$	D26	D26	3656	NT
**Comparative study Cohort 1**	ART-R_1	$K13^{R539T}$	D27	D28	7295	8860
	ART-R_2	$K13^{R539T}$	D27	D28	4581	5373
	ART-R_3	$K13^{R539T}$	D27	D28	1172	624
	ART-S_1	3D7	D30	D35	36	7
	ART-S_2	3D7	D30	D31	8002	1387
	ART-S_3	3D7	D30	D35	449	12
**Comparative study Cohort 2**	ART-R_4	$K13^{R539T}$	D23	D23	456	7
	ART-R_5	$K13^{R539T}$	D23	D23	2253	1079
	ART-R_6	$K13^{R539T}$	D23	D23	15741	3307
	ART-R_7	$K13^{R539T}$	D23	D23	137	37
	ART-R_8	$K13^{R539T}$	D23	D23	5098	518
	ART-R_9	$K13^{R539T}$	D23	D23	7987	1029
	ART-R_10	$K13^{R539T}$	D23	D23	237	33
	ART-S_4	3D7	D23	D23	59	ND
	ART-S_5	3D7	D23	D23	337	ND
	ART-S_6	3D7	D23	D23	363	62
**Comparative study Cohort 3**	ART-R_11	$K13^{R539T}$	D24	D24	611	144
	ART-R_12	$K13^{R539T}$	D24	D24	6767	1636
	ART-R_13	$K13^{R539T}$	D24	D24	3001	1144
	ART-S_7	3D7	D24	D24	4439	757
	ART-S_8	3D7	D24	D24	930	46
	ART-S_9	3D7	D24	D24	883	132

Gametocytaemia was measured by reverse transcription quantitative PCR (qRT-PCR) for female-specific *pfs25* mRNA and male-specific *pfMGET* mRNA. ART-R: artemisinin-resistant; ART-S: artemisinin-sensitive; ND: not detected; NT: not tested.
