# Supplementary material for: Safety and parasite clearance of artemisinin-resistant Plasmodium falciparum infection: A pilot and a randomised volunteer infection study in Australia
Source: PLoS Med. 2020 Aug 21;17(8):e1003203. doi: 10.1371/journal.pmed.1003203 (PMC7444516; doi:10.1371/journal.pmed.1003203)
Supplement: S6 Table — (PDF) [file pmed.1003203.s016.pdf]

S6 Table. Antimalarial administration days

	Participant number	*P. falciparum* strain	Artesunate	DHA/PQP (pilot study) or PQP (comparative study)	A/P	Primaquine
**Pilot study**	Participant 1	K13^{R539T}	D9	D11	D26	D26
	Participant 2	K13^{R539T}	D9	D11	D26	D26
**Comparative study Cohort 1**	ART-R_1	K13^{R539T}	D9	D11	D20	D28
	ART-R_2	K13^{R539T}	D9	D11	D20	D28
	ART-R_3	K13^{R539T}	D9	D11	D16	D28
	ART-S_1	3D7	D9	D17	D27	D35
	ART-S_2	3D7	D9	D20	D27	D31
	ART-S _3	3D7	D9	D17	D27	D35
**Comparative study Cohort 2**	ART-R_4	K13^{R539T}	D9	D11	D28	D23
	ART-R_5	K13^{R539T}	D9	D11	D21	D23
	ART-R_6	K13^{R539T}	D9	D11	D19	D23
	ART-R_7	K13^{R539T}	D9	D11	D28	D23
	ART-R_8	K13^{R539T}	D9	D11	D28	D23
	ART-R_9	3D7	D9	D11	D28	D23
	ART-R_10	K13^{R539T}	D9	D11	D28	D23
	ART-S_4	3D7	D9	D19	D28	D23
	ART-S _5	3D7	D9	D17	D28	D23
	ART-S_6	3D7	D9	D17	D28	D23
**Comparative study Cohort 3**	ART-R_11	K13^{R539T}	D9	D11	D21	D24
	ART-R_12	K13^{R539T}	D9	D11	D21	D24
	ART-R_13	K13^{R539T}	D9	D11	D21	D24
	ART-S_7	3D7	D9	D15	D21	D24
	ART-S_8	3D7	D9	D15	D21	D24
	ART-S_9	3D7	D9	D15	D21	D24

ART-R: artemisinin-resistant; ART-S: artemisinin-sensitive; A/P: atovaquone/proguanil; D: Study Day; PQP: piperaquine phosphate.
