# Supplementary material for: Safety and parasite clearance of artemisinin-resistant Plasmodium falciparum infection: A pilot and a randomised volunteer infection study in Australia
Source: PLoS Med. 2020 Aug 21;17(8):e1003203. doi: 10.1371/journal.pmed.1003203 (PMC7444516; doi:10.1371/journal.pmed.1003203)
Supplement: S7 Table — AS, artesunate. (PDF) [file pmed.1003203.s017.pdf]

S7 Table. Individual parasite clearance slope, $\log_{10}PRR_{48}$, and parasite clearance half-life after artesunate administration

	Participant number	*P. falciparum* strain	Parasite clearance slope (95% CI)	$Log_{10}PRR_{48}$ (95% CI)	Parasite clearance half-life [hours] (95% CI)
**Pilot study**	Participant 1	$K13^{R539T}$	-0.055 ([-0.062]–[-0.048])	2.65 (2.32–2.98)	5.5 (4.8–6.2)
	Participant 2	$K13^{R539T}$	-0.058 ([-0.068]–[-0.048])	2.78 (2.32–3.25)	5.2 (4.5–6.2)
**Comparative study Cohort 1**	ART-R_1	$K13^{R539T}$	-0.054 ([-0.062]–[-0.046])	2.58 (2.20–2.96)	5.60 (4.88–6.57)
	ART-R_2	$K13^{R539T}$	-0.041 ([-0.048]–[-0.033])	1.95 (1.60–2.29)	7.43 (6.31–9.02)
	ART-R_3	$K13^{R539T}$	-0.053 ([-0.061]–[-0.045])	2.54 (2.15–2.92)	5.69 (4.95–6.71)
	ART-S_1	3D7	-0.211 ([-0.254]–[-0.169])	10.14 (8.11–12.18)	1.42 (1.19–1.78)
	ART-S_2	3D7	-0.088 ([-0.098]–[-0.078])	4.22 (3.76–4.68)	3.42 (3.08–3.84)
	ART-S _3	3D7	-0.113 ([-0.134]–[-0.092])	5.41 (4.40–6.41)	2.67 (2.25–3.28)
**Comparative study Cohort 2**	ART-R_4	$K13^{R539T}$	-0.045 ([-0.050]–[-0.039])	2.14 (1.89–2.40)	6.75 (6.03–7.65)
	ART-R_5	$K13^{R539T}$	-0.055 ([-0.068]–[-0.042])	2.64 (2.01–3.28)	5.46 (4.41–7.18)
	ART-R_6	$K13^{R539T}$	-0.041 ([-0.044]–[-0.037])	1.95 (1.78–2.12)	7.41 (6.81–8.12)
	ART-R_7	$K13^{R539T}$	-0.050 ([-0.060]–[-0.041])	2.42 (1.96–2.88)	5.97 (5.02–7.36)
	ART-R_8	$K13^{R539T}$	-0.045 ([-0.051]–[-0.038])	2.14 (1.81–2.47)	6.76 (5.85–7.99)
	ART-R_9	$K13^{R539T}$	-0.052 ([-0.058]–[-0.045])	2.48 (2.16–2.79)	5.84 (5.18–6.69)
	ART-R_10	$K13^{R539T}$	-0.051 ([-0.058]–[-0.044])	2.43 (2.09–2.77)	5.94 (5.21–6.91)
	ART-S_4	3D7	-0.119 ([-0.138]–[-0.099])	5.70 (4.75–6.64)	2.54 (2.18–3.04)
	ART-S_5	3D7	-0.136 ([-0.153]–[-0.120])	6.54 (5.75–7.34)	2.21 (1.97–2.51)
	ART-S_6	3D7	-0.099 ([-0.115]–[-0.083])	4.76 (4.01–5.51)	3.04 (2.62–3.61)
**Comparative study Cohort 3**	ART-R _11	$K13^{R539T}$	-0.051 ([-0.055]–[-0.047])	2.46 (2.25–2.66)	5.88 (5.43–6.42)
	ART-R_12	$K13^{R539T}$	-0.041 ([-0.046]–[-0.035])	1.95 (1.68–2.23)	7.40 (6.49–8.60)
	ART-R _13	$K13^{R539T}$	-0.056 ([-0.068]–[-0.045])	2.70 (2.14–3.26)	5.35 (4.43–6.76)
	ART-S_7	3D7	-0.094 ([-0.105]–[-0.083])	4.52 (3.99–5.06)	3.19 (2.86–3.62)
	ART-S_8	3D7	-0.103 ([-0.112]–[-0.093])	4.92 (4.48–5.36)	2.94 (2.70–3.23)
	ART-S_9	3D7	-0.060 ([-0.071]–[-0.049])	2.88 (2.38–3.39)	5.01 (4.26–6.08)

ART-R: artemisinin-resistant; ART-S: artemisinin-sensitive; CI: confidence interval; $\log_{10}PRR_{48}$: parasite reduction ratio per 48 hours in the logarithmic base 10 scale.
