# Supplementary material for: Safety and parasite clearance of artemisinin-resistant Plasmodium falciparum infection: A pilot and a randomised volunteer infection study in Australia
Source: PLoS Med. 2020 Aug 21;17(8):e1003203. doi: 10.1371/journal.pmed.1003203 (PMC7444516; doi:10.1371/journal.pmed.1003203)
Supplement: S8 Table — (PDF) [file pmed.1003203.s018.pdf]

S8 Table. Adverse events by System Organ Class, Preferred Term and *P. falciparum* strain

System Organ Class<br>Preferred Term	Pilot study	Comparative study	
	**Artemisinin-resistant (N=2) n (%) M**	**Artemisinin-resistant (N=13) n (%) M**	**Artemisinin-sensitive (N=9) n (%) M**
**General disorders and administration site conditions**	2 (100%) 8	11 (84.6%) 35	9 (100%) 32
Pyrexia	1 (50.0%) 2	10 (76.9%) 15	7 (77.8%) 13
Malaise	1 (50.0%) 1	5 (38.5%) 5	3 (33.3%) 6
Chills	2 (100%) 5	4 (30.8%) 4	3 (33.3%) 3
Fatigue	0 (0.0%) 0	3 (23.1%) 4	4 (44.4%) 8
Vessel puncture site bruise	0 (0.0%) 0	3 (23.1%) 4	1 (11.1%) 2
Vessel puncture site hematoma	0 (0.0%) 0	1 (7.7%) 2	0 (0.0%) 0
Feeling hot	0 (0.0%) 0	1 (7.7%) 1	0 (0.0%) 0
**Nervous system disorders**	2 (100%) 10	12 (92.3%) 28	8 (88.9%) 25
Headache	2 (100%) 5	12 (92.3%) 25	8 (88.9%) 22
Lethargy	1 (50.0%) 2	1 (7.7%) 1	2 (22.2%) 2
Dizziness	1 (50.0%) 3	1 (7.7%) 1	1 (11.1%) 1
Paraesthesia	0 (0.0%) 0	1 (7.7%) 1	0 (0.0%) 0
**Investigations**	2 (100%) 9	11 (84.6%) 24	6 (66.7%) 21
Neutrophil count decreased	2 (100%) 3	7 (53.8%) 8	2 (22.2%) 5
Lymphocyte count decreased	2 (100%) 2	6 (46.2%) 6	1 (11.1%) 1
White blood cell count decreased	1 (50.0%) 1	6 (46.2%) 6	1 (11.1%) 1
Alanine aminotransferase increased	1 (50.0%) 2	2 (15.4%) 3	4 (44.4%) 7
Aspartate aminotransferase increased	1 (50.0%) 1	0 (0.0%) 0	2 (22.2%) 4
Eosinophil count increased	0 (0.0%) 0	1 (7.7%) 1	1 (11.1%) 1
Blood phosphorus decreased	0 (0.0%) 0	0 (0.0%) 0	1 (11.1%) 1
Urobilinogen urine	0 (0.0%) 0	0 (0.0%) 0	1 (11.1%) 1
**Musculoskeletal and connective tissue disorders**	2 (100%) 3	9 (69.2%) 21	8 (88.9%) 18
Myalgia	2 (100%) 2	6 (46.2%) 11	7 (77.8%) 13
Arthralgia	1 (50.0%) 1	4 (30.8%) 7	2 (22.2%) 2
Back pain	0 (0.0%) 0	1 (7.7%) 1	2 (22.2%) 2
Musculoskeletal pain	0 (0.0%) 0	1 (7.7%) 1	1 (11.1%) 1
Neck pain	0 (0.0%) 0	1 (7.7%) 1	0 (0.0%) 0
**Gastrointestinal disorders**	1 (50.0%) 4	6 (46.2%) 13	6 (66.7%) 12
Nausea	1 (50.0%) 2	5 (38.5%) 9	6 (66.7%) 8
Abdominal discomfort	1 (50.0%) 1	1 (7.7%) 1	2 (22.2%) 2
Abdominal tenderness	0 (0.0%) 0	1 (7.7%) 1	1 (11.1%) 2
Diarrhoea	0 (0.0%) 0	1 (7.7%) 1	0 (0.0%) 0
Vomiting	1 (50.0%) 1	1 (7.7%) 1	0 (0.0%) 0
**Cardiac disorders**	0 (0.0%) 0	5 (38.5%) 6	3 (33.3%) 5
Tachycardia	0 (0.0%) 0	4 (30.8%) 5	3 (33.3%) 4
Ventricular extrasystoles	0 (0.0%) 0	1 (7.7%) 1	1 (11.1%) 1
**Injury, poisoning and procedural complications**	0 (0.0%) 0	6 (46.2%) 7	1 (11.1%) 2
Arthropod bite	0 (0.0%) 0	6 (46.2%) 6	1 (11.1%) 2
Thermal burn	0 (0.0%) 0	1 (7.7%) 1	0 (0.0%) 0
**Skin and subcutaneous tissue disorders**	0 (0.0%) 0	2 (15.4%) 4	4 (44.4%) 4
Hyperhidrosis	0 (0.0%) 0	2 (15.4%) 3	4 (44.4%) 4
Dermatitis contact	0 (0.0%) 0	1 (7.7%) 1	0 (0.0%) 0
**Infections and infestations**	0 (0.0%) 0	4 (30.8%) 5	1 (11.1%) 1
Upper respiratory tract infection	0 (0.0%) 0	4 (30.8%) 5	1 (11.1%) 1
**Metabolism and nutrition disorders**	0 (0.0%) 0	3 (23.1%) 3	2 (22.2%) 2
Decreased appetite	0 (0.0%) 0	2 (15.4%) 2	2 (22.2%) 2
Hypophosphatemia	0 (0.0%) 0	1 (7.7%) 1	0 (0.0%) 0
**Respiratory, thoracic and mediastinal disorders**	1 (50.0%) 2	2 (15.4%) 2	2 (22.2%) 2
Oropharyngeal pain	1 (50.0%) 1	2 (15.4%) 2	1 (11.1%) 1

**System Organ Class**<br>Preferred Term	**Pilot study**	**Comparative study**	
	**Artemisinin-resistant (N=2) n (%) M**	**Artemisinin-resistant (N=13) n (%) M**	**Artemisinin-sensitive (N=9) n (%) M**
Nasal congestion	0 (0.0%) 0	0 (0.0%) 0	1 (11.1%) 1
Rhinorrhea	1 (50.0%) 1	0 (0.0%) 0	0 (0.0%) 0
**Psychiatric disorders**	0 (0.0%) 0	1 (7.7%) 1	0 (0.0%) 0
Insomnia	0 (0.0%) 0	1 (7.7%) 1	0 (0.0%) 0
**Reproductive system and breast disorders**	0 (0.0%) 0	0 (0.0%) 0	1 (11.1%) 2
Dysmenorrhea	0 (0.0%) 0	0 (0.0%) 0	1 (11.1%) 2
**Vascular disorders**	0 (0.0%) 0	1 (7.7%) 1	0 (0.0%) 0
Thrombophlebitis superficial	0 (0.0%) 0	1 (7.7%) 1	0 (0.0%) 0
**Blood and lymphatic system disorders**	0 (0.0%) 0	1 (7.7%) 1	0 (0.0%) 0
Splenomegaly	0 (0.0%) 0	1 (7.7%) 1	0 (0.0%) 0

N: total number of participants in each cohort; n: number of participants presenting the adverse event (if a participant has multiple occurrences of an adverse event, the participant is counted only once for n in a given System Organ Class and Preferred Term); M: number of occurrences of adverse events. Adverse events were coded to System Organ Class and Preferred Term using MedDRA Version 20.1.
