# Supplementary material for: Safety and parasite clearance of artemisinin-resistant Plasmodium falciparum infection: A pilot and a randomised volunteer infection study in Australia
Source: PLoS Med. 2020 Aug 21;17(8):e1003203. doi: 10.1371/journal.pmed.1003203 (PMC7444516; doi:10.1371/journal.pmed.1003203)
Supplement: S9 Table — (PDF) [file pmed.1003203.s019.pdf]

S9 Table. Summary of adverse events related to antimalarial drugs

	**Participant number**	***P. falciparum* strain**	**Adverse event**	**Severity**	**Related to antimalarial drug**
**Pilot study**	Participant 2	$K13^{R539T}$	Nausea	Moderate	A/P and primaquine
			Vomiting	Moderate	A/P and primaquine
			Dizziness	Moderate	A/P and primaquine
			Nausea	Mild	A/P
**Comparative study**	ART-S_3	3D7	Decreased appetite	Mild	A/P
			Eosinophil count increased	Moderate	PQP and A/P
	ART-R_4	$K13^{R539T}$	Vomiting	Moderate	A/P
			Nausea	Mild	A/P
	ART-R_6	$K13^{R539T}$	Eosinophil count increased	Mild	A/P and primaquine
	ART-R _7	$K13^{R539T}$	Decreased appetite	Mild	A/P
			Insomnia	Mild	A/P
	ART-R_9	$K13^{R539T}$	Nausea	Mild	A/P
			Diarrhoea	Mild	A/P

ART-R: artemisinin-resistant; ART-S: artemisinin-sensitive; A/P: atovaquone/proguanil; PQP: piperaquine phosphate.
