# Supplementary material for: Safety and parasite clearance of artemisinin-resistant Plasmodium falciparum infection: A pilot and a randomised volunteer infection study in Australia
Source: PLoS Med. 2020 Aug 21;17(8):e1003203. doi: 10.1371/journal.pmed.1003203 (PMC7444516; doi:10.1371/journal.pmed.1003203)
Supplement: S10 Table — (PDF) [file pmed.1003203.s020.pdf]

S10 Table. Troponin I results for participants in the comparative study with ventricular extrasystoles

**Participant number**	***P. falciparum* strain**	**Day of sample**	**Troponin I (ng/L)**
ART-S_5	3D7	D1 Pre-inoculation	3
		D8 pm	<2
		D9 Pre-artesunate administration	30[*]
		D9 + 2 h	<2
		D9 + 4 h	<2
		D9 + 6 h	<2
		D9 + 8 h	4
		D10	<2
		D11	3
		D28	<2
		D31	<2
ART-R_12	$K13^{R539T}$	D0 Pre-inoculation	3
		D9 Pre-artesunate administration	<2
		D9 + 8 h	<2
		D10	<2
		D11	<2
		D28	<2
		D44	<2

[*]Abnormal value; normal range is <26 ng/L. h: hours. ART-R: artemisinin-resistant; ART-S: artemisinin-sensitive.
