# Supplementary material for: Safety and parasite clearance of artemisinin-resistant Plasmodium falciparum infection: A pilot and a randomised volunteer infection study in Australia
Source: PLoS Med. 2020 Aug 21;17(8):e1003203. doi: 10.1371/journal.pmed.1003203 (PMC7444516; doi:10.1371/journal.pmed.1003203)
Supplement: S11 Table — QTcF, QT interval corrected using Fridericia’s formula. (PDF) [file pmed.1003203.s021.pdf]

S11 Table. Summary of QTcF prolongations ≥30 msec in the comparative study

**Participant number**	***P. falciparum* strain**	**Study day**	**Baseline**	**QTcF prolongation (msec)**
ART-R_3	$K13^{R539T}$	D19	Pre-PQP (D11)	30
ART-R_4	$K13^{R539T}$	D14	Pre-inoculum (D0)	30
ART-R_12	$K13^{R539T}$	D9 (pre-artesunate)	Pre-inoculum (D0)	37*
		D9 (post-artesunate)	Pre-inoculum (D0)	54*
		D10	Pre-inoculum (D0)	56
		D11 (pre-PQP)	Pre-inoculum (D0)	48
		D12	Pre-inoculum (D0)	55*
		D12	Pre-PQP (D11)	44
		D28	Pre-inoculum (D0)	34
ART-R_13	$K13^{R539T}$	D14	Pre-PQP (D11)	35
ART-S_9	3D7	D18	Pre-inoculum (D1)	31

*Multiple electrocardiograms were conducted at these timepoints; the longest prolongation is shown in the table. Participant artemisinin-resistant_12 also had a ventricular extrasystoles adverse event. msec: millisecond; PQP: piperaquine phosphate. ART-R: artemisinin-resistant; ART-S: artemisinin-sensitive.
