# Supplementary material for: Safety and parasite clearance of artemisinin-resistant Plasmodium falciparum infection: A pilot and a randomised volunteer infection study in Australia
Source: PLoS Med. 2020 Aug 21;17(8):e1003203. doi: 10.1371/journal.pmed.1003203 (PMC7444516; doi:10.1371/journal.pmed.1003203)
Supplement: S12 Table — AS, artesunate; DHA, dihydroartemisinin. (PDF) [file pmed.1003203.s022.pdf]

S12 Table. Pharmacokinetic parameters of artesunate and DHA in the pilot study

	**Participant 1**	**Participant 2**
**Artesunate**		
C_{max} (μg/L)	107.5	97.8
$AUC_{0\text{-last}}$ (h·μg/L)	92.0	52.2
$AUC_{0\text{-}\infty}$ (h·μg/L)	NA	NA
t_{max} (h)	1.0	0.5
$t_{1/2}$ (h)	NA	NA
CL/F (L/h)	NA	NA
V_d/F (L)	NA	NA
**DHA**		
C_{max} (μg/L)	316.2	543.1
$AUC_{0\text{-last}}$ (h·μg/L)	477.3	914.6
$AUC_{0\text{-}\infty}$ (h·μg/L)	538.3	926.0
t_{max} (h)	1.5	1.0
$t_{1/2}$ (h)	0.7	1.5
CL/F (L/h)	180.0	104.7
V_d/F (L)	174.2	231.3

Artesunate was rapidly hydrolysed to DHA with artesunate concentration below the lower limit of quantification after 2 h post-artesunate administration. Therefore, the terminal slope of the artesunate concentration-time profile could not be estimated in the pilot study, due to limited samples after C_{max} with concentrations above the lower limit of quantification. Artesunate dose was based on weight at screening; both participants received 150 mg artesunate. $AUC_{0\text{-last}}$: area under the concentration-time curve from time 0 to the last measurable concentration; $AUC_{0\text{-}\infty}$: area under the concentration-time curve from time 0 to infinite time; CL/F: apparent clearance; C_{max}: maximum concentration; t_{max}: time of C_{max}; $t_{1/2}$: elimination half-life; V_d/F: apparent volume of distribution where F is bioavailability; DHA: dihydroartemisinin; h: hours; NA: not available.
