# Supplementary material for: Safety and parasite clearance of artemisinin-resistant Plasmodium falciparum infection: A pilot and a randomised volunteer infection study in Australia
Source: PLoS Med. 2020 Aug 21;17(8):e1003203. doi: 10.1371/journal.pmed.1003203 (PMC7444516; doi:10.1371/journal.pmed.1003203)
Supplement: S13 Table — AS, artesunate; DHA, dihydroartemisinin. (PDF) [file pmed.1003203.s023.pdf]

S13 Table. Pharmacokinetic parameters of artesunate and DHA in the comparative study

	**Artemisinin-resistant (n=13)**	**Artemisinin-sensitive (n=9)**	**p value**
**Artesunate**			
Dose-normalised C_{max} (µg/L/mg)	1.5 (75.6)	1.7 (71.5)	0.60
Dose-normalised $AUC_{0-\infty}$ (µg·h/L/mg)	1.1 (38.0)	1.3 (29.5)	0.28
t_{max} (h)	1.0 (0.3–1.5)	0.5 (0.5–2.0)	0.62
$t_{1/2}$ (h)	0.5 (96.7)	0.5 (96.5)	0.96
CL/F (L/h)	954.2 (38.0)	752.8 (29.5)	0.28
V_d/F (L)	727.4 (79.6)	576.5 (92.9)	0.46
**DHA**			
Dose-normalised C_{max} (µg/L/mg)	4.8 (52.0)	4.3 (104.3)	0.66
Dose-normalised $AUC_{0-\infty}$ (µg·h/L/mg)	7.5 (33.2)	6.3 (89.9)	0.85
t_{max} (h)	1.0 (52.0)	1.1 (51.8)	0.95
$t_{1/2}$ (h)	1.2 (67.8)	1.4 (102.4)	0.70
CL/F (L/h)	133.2 (33.2)	157.9 (89.9)	0.85
V_d/F (L)	221.1 (53.6)	310.3 (119.4)	0.74

Data are geometric means (coefficient of variation) except median (range) for t_{max}. Artesunate was rapidly hydrolysed to DHA with artesunate concentration below the lower limit of quantification after a median of 4 hours (range 1.5–10 hours). Therefore, the terminal slope of the artesunate concentration-time profile could not be estimated in 7 participants in the comparative study, due to limited samples after C_{max} with concentrations above the lower limit of quantification. Artesunate dose was based on weight at screening. Artemisinin-sensitive infected participants received 100 mg artesunate (n=2) or 150 mg artesunate (n=7). Artemisinin-resistant infected participants received 100 mg artesunate (n=6), 150 mg artesunate (n=6), or 200 mg artesunate (n=1). C_{max} and $AUC_{0-\infty}$ were normalised to the artesunate dose administered. $AUC_{0-\infty}$: area under the concentration-time curve from time 0 to infinite time; CL/F: apparent clearance; C_{max}: maximum concentration; t_{max} = time of C_{max}; $t_{1/2}$= elimination half-life; Vd/F: apparent volume of distribution where F is bioavailability; DHA: dihydroartemisinin; h: hours.
