# Supplementary material for: Safety and parasite clearance of artemisinin-resistant Plasmodium falciparum infection: A pilot and a randomised volunteer infection study in Australia
Source: PLoS Med. 2020 Aug 21;17(8):e1003203. doi: 10.1371/journal.pmed.1003203 (PMC7444516; doi:10.1371/journal.pmed.1003203)
Supplement: S1 Appendix — (PDF) [file pmed.1003203.s025.pdf]

TABLE OF CONTENTS

Appendix 16.1 Study Information

16.1.1 Protocol and protocol amendments

16.1.2 Sample case report form

16.1.3 List of Human Research Ethics Committees

16.1.4 List and description of investigators and other important participants in the study

16.1.5 Signature of Principal Investigator and Sponsor's Signatory

16.1.6 List of investigational product batch numbers

16.1.7 Randomisation scheme and codes

16.1.8 Audit certificates

16.1.9 Documentation of statistical methods

16.1.10 Documentation of inter-laboratory standardisation methods and laboratory QA procedures

16.1.11 Publications based on study

16.1.12 Important publications referenced in the report

16.1.13 Reports

Appendix 16.2 Subject Data Listings

16.2.1 Study enrolment and completion/discontinuation

16.2.2 Protocol deviations

16.2.3 Subjects excluded from the efficacy analysis

16.2.4 Demographic and baseline data

16.2.4.1 Demography

16.2.4.2 Medical history

16.2.4.5 Red cell allo-antibody

16.2.4.6 Serology and special tests

16.2.4.7 Urine drug screen and alcohol breath test

16.2.4.8 G6PD

16.2.4.10 Body weight, height and BMI

16.2.4.11 Concomitant medications

16.2.5 Compliance and drug concentration data

16.2.5.1 Malaria challenge agent administration

16.2.5.2 Study drug administration

16.2.5.3 Rescue medication administration

16.2.5.4 Artesunate and dihydroartemisinin (DHA) plasma concentrations

16.2.6 Parasitaemia and parasite lifecycle stage qPCR/qRT-PCR data

16.2.7 Adverse events

16.2.7.1 Adverse events

16.2.7.2 Serious adverse events

16.2.8 Clinical laboratory measurements

16.2.8.1 Individual abnormal haematology results

16.2.8.2 Individual abnormal biochemistry results

16.2.8.3 Individual abnormal urinalysis results (Dipstick)

16.2.8.4 Individual abnormal microscopy urinalysis results (laboratory)

16.2.9 Other safety data

16.2.9.1 Abnormal vital signs (Blood pressure and heart rate)

16.2.9.2 Abnormal vital signs (Body temperature and respiratory rate)

16.2.9.3 Abnormal ECG interpretation

16.2.9.4 Abnormal physical examination

16.2.9.5 Clinical Score

16.2.10 Additional Comments

Appendix 16.3 Case Report Forms Submitted

Appendix 16.1 Study Information

16.1.1 Protocol and protocol amendments

AN EXPERIMENTAL STUDY TO CHARACTERISE THE *IN VIVO* SAFETY AND INFECTIVITY OF A *PLASMODIUM FALCIPARUM* Cam3.II^{R539T} (K13) ARTEMISININ-RESISTANT ISOLATE IN HEALTHY PARTICIPANTS

Protocol Identifying Number: QP16C14/P2286

Principal Investigator: Prof. James McCarthy

Sponsor: QIMR Berghofer Medical Research Institute

Funded by: Medicines for Malaria Venture

Version Number: 2.0

17 May 2017

CONFIDENTIALITY STATEMENT

This document contains information that is privileged or confidential. As such, it may not be disclosed unless specific prior permission is granted in writing by QIMR Berghofer or such disclosure is required by federal or other laws or regulations. Persons to whom any of this information is to be disclosed must first be informed that the information is confidential. These restrictions on disclosure will apply equally to all future information supplied, which is indicated as privileged or confidential.

TABLE OF CONTENTS

List of Abbreviations **6**

Investigator Signature Page **8**

Protocol Summary **10**

Schematic of Study Design **14**

1.0 KEY ROLES **15**

2.0 INTRODUCTION **18**

2.1 Background Information 18

2.2 Rationale 19

2.3 Potential Risks and Benefits 20

2.3.1 Known Potential Risks 20

2.3.2 Risk Management 22

2.3.3 Known Potential Benefits 22

3.0 OBJECTIVES AND PURPOSE **23**

4.0 STUDY DESIGN AND ENDPOINTS **24**

4.1 Study Design 24

4.2 Endpoints 25

5.0 STUDY ENROLLMENT AND WITHDRAWAL **26**

5.1 Participant Inclusion Criteria 26

5.2 Participant Exclusion Criteria 26

5.3 Strategies for Recruitment 29

5.3.1 Number of Participants 30

5.4 Participant Withdrawal or Termination 30

5.4.1 Reasons for Withdrawal or Termination 30

5.4.2 Handling of Participant Withdrawals or Termination 30

5.5 Premature Termination or Suspension of Study 30

5.5.1 Guidance for stopping rules 31

5.5.2 Obligations of the Sponsor 31

6.0 STUDY AGENTS **32**

6.1 *P. falciparum* K13 Blood Stage Challenge Inoculum 32

6.1.1 Preparation of the *P. falciparum* K13 Master Cell Bank 32

6.1.2 Preparation of the Inoculum and Dosing 32

6.1.3 Packaging, Labelling and Storage of the Inoculum 33
6.1.4 Administration of the Inoculum 33
6.2 Artesunate 33
6.2.1 Preparation of Artesunate 33
6.2.2 Dosing and Administration of Artesunate 33
6.3 Eurartesim® 34
6.4 Malarone® 34
6.5 Primacin™ (If Required) 34
6.6 Packaging, Labelling and Storage of Antimalarial Drugs 34
6.7 Product Accountability 34
7.0 STUDY PROCEDURES AND SCHEDULE 36
7.1 Study Schedule 36
7.1.1 Screening Visit (Day -28 to Day -1) 36
7.1.2 Day -3 to Day -1 safety visit 37
7.1.3 Administration of Malaria Challenge Inoculum (Day 0) 37
7.1.4 Days 1, 2, and 3 Post-Induced Infection 38
7.1.5 Day 4 AM until qPCR Positive for Malaria 38
7.1.6 Day when qPCR Positive until Treatment Day 38
7.1.7 Inpatient Observation and Antimalarial Treatment Phase (approximately Day 8) 39
7.1.8 Out-Patient Safety Monitoring Post-Artesunate Treatment 40
7.1.9 Eurartesim® Treatment 41
7.1.10 Malarone® Treatment 41
7.1.11 Follow-up Visit (Day 28±3) 42
7.1.12 Follow-up Phone Call (Day 56±7) 42
7.1.13 Day 90±14 or End of Study (Final Visit) 42
7.1.14 Early Termination Visit 42
7.2 Study and Laboratory Procedures/Evaluations 43
7.2.1 Medical History 43
7.2.2 Physical Examination 43
7.2.3 Vital Signs 43
7.2.4 Electrocardiogram (ECG) 44
7.2.5 Cannulation for Blood Sampling 44

7.2.6 Clinical Laboratory Evaluations 44
7.2.7 Drug Screens and Alcohol Breath Tests 45
7.2.8 Malaria monitoring by qPCR 45
7.2.9 Pharmacokinetic assays 46
7.2.10 General 46
7.2.11 Specimen Preparation, Handling, and Storage 46
7.2.12 Specimen Shipment 47
7.2.13 Meals and Fluid Restrictions 47
7.3 Concomitant Medications 47
8.0 Assessment of safety 48
8.1 Specification of Safety Parameters 48
8.1.1 Adverse Events (AEs) 48
8.1.2 Definition of Adverse Events 48
8.1.3 Definition of Serious Adverse Events 49
8.1.4 Adverse Events Associated with the Study Design or Protocol-Mandated Procedures 50
8.1.5 Definition of Unexpected Events 50
8.2 Classification of an Adverse Event 50
8.2.1 Severity of Event 50
8.2.2 Relationship to Study Agent 50
8.3 Treatment and Follow-up of Adverse Events 51
8.4 Reporting Procedures 52
8.4.1 Serious Adverse Event Reporting 52
8.5 Emergency Procedures 53
8.6 Safety Oversight 53
9.0 Statistical Considerations 54
9.1 General Approach 54
9.2 Sample Size Calculations 54
9.3 Demographic and Clinical Laboratory Data 54
9.4 Analysis of Safety Data 54
9.5 Data Management 54
10.0 Ethical/Protection of participants 55
10.1 Ethical Principles 55

10.2 Ethical Review ... 55
10.3 Informed Consent Process ... 55
10.3.1 Consent Procedures and Documentation ... 55
10.3.2 Participant Compensation ... 55
10.4 Participant and Data Confidentiality ... 56
10.5 Future Use of Stored Specimens ... 56
11.0 Study Administration ... 57
11.1 Liability/Indemnity/Insurance ... 57
11.2 Protocol Amendments ... 57
11.2.1 Non-substantial amendment ... 57
11.2.2 Substantial amendment ... 57
11.2.3 Urgent amendment ... 57
11.3 Clinical Data Recording ... 58
11.4 Record Retention ... 58
11.5 Monitoring ... 58
11.6 Reporting and Communication of Results ... 59
11.7 Study Audit ... 59
12.0 References ... 60
13.0 Appendices ... 64
Appendix 1: Schedule of Events ... 64
Appendix 2: Detailed Laboratory Procedures ... 66
Appendix 3: Total Blood Volume ... 69
Appendix 4: Symptoms and Signs of Malaria ... 70
Appendix 5: Production Information and Consumer Information ... 71
Appendix 6: Clinical Score for Malaria ... 72
Appendix 7: Dosing Table for Artesunate Tablets ... 73
Appendix 8: Version History ... 74

LIST OF ABBREVIATIONS

AE	Adverse Event
ALT	Alanine Aminotransferase
anti-HBc Ab	Anti-Hepatitis B Core Antibodies
anti-HCV	Anti-Hepatitis C Virus
anti-HIV1	Anti-Human Immunodeficiency Virus 1
anti-HIV2	Anti-Human Immunodeficiency Virus 2
AST	Aspartate Aminotransferase
AUC	Area Under the Curve
AUC_{inf}	AUC Curve to Infinite Time
Blood Service	Australian Red Cross Blood Service
CHMI	Controlled Human Malaria Infection
C_{max}	Maximum Plasma Concentration
CMI	Consumer Medicines Information
CMV	Cytomegalovirus
CNS	Clinical Network Services
CRF	Case Report Form
CSR	Clinical Study Report
CRU	Clinical Research Unit
CTCAE	Common Terminology Criteria for Adverse Events
EBV	Epstein-Barr Virus
EC	Ethics Committee
ECG	Electrocardiogram
EOS	End of Study
FBC	Full Blood Count
FDA	Food and Drug Administration
G6PD	Glucose-6-Phosphate Dehydrogenase
GCP	Good Clinical Practice
GMP	Good Manufacturing Practice
HAV	Hepatitis A Virus
HBV	Hepatitis B Virus
HBs Ag	Hepatitis B surface antigen
HCV	Hepatitis C Virus
HEV	Hepatitis E Virus
HDL	High Density Lipoprotein

HIV	Human Immunodeficiency Virus
HREC	Human Research Ethics Committee
IBSM	Induced Blood Stage Malaria
ICH	International Conference on Harmonization
IMM	Independent Medical Monitor
IP	Investigational Product
LFT	Liver Function Test
MCB	Master Cell Bank
MMV	Medicines for Malaria Venture
ND	Non-Detectable
NH&MRC	National Health and Medical Research Council, Australia
PCR	Polymerase Chain Reaction
PD	Pharmacodynamic
PI	Principal Investigator
PICF	Patient Information Sheet and Consent Form
PK	Pharmacokinetic
PRR	Parasite Reduction Ratio
QIMR-B	Queensland Institute of Medical Research Berghofer
qPCR	Quantitative Polymerase Chain Reaction
RBC	Red Blood Cell
Rh	Rhesus Antibody
SAE	Serious Adverse Event
SOP	Standard Operating Procedures
SRT	Safety Review Team
SUSAR	Suspected Unexpected Serious Adverse Reaction
TGA	Therapeutic Goods Administration
T_{max}	Time taken to reach C_{max}
ULN	Upper Limit of Normal
U.S.	United States
WHO	World Health Organization

INVESTIGATOR SIGNATURE PAGE

I have read the protocol and agree that it contains all necessary details for carrying out the study as described. I will conduct this protocol as outlined herein and will make a reasonable effort to complete the study within the time designated.

I agree to personally conduct or supervise the described study.

The study will be conducted in accordance with the following:

- World Medical Association Declaration of Helsinki – Ethical Principles for Medical Research Involving Human Participants
- NH&MRC National Statement on Ethical Conduct in Human Research (2007)
- Notes for Guidance on Good Clinical Practice – Annotated with the Australian Therapeutic Goods Administration (TGA) Comments (CPMP/ICH/135/95), as adopted by the TGA (July 2000)
- Current ethics approved Clinical Trial Protocol.

I agree to inform all participants that the study drug is being used for investigational purposes and I will ensure that the requirements related to obtaining informed consent are in accordance with the International Council of Harmonisation (ICH) Guidelines for Good Clinical Practice (GCP) section 4.8 and local requirements.

I agree to report adverse events that occur in the course of the study to the Sponsor in accordance with ICH Guidelines for GCP section 4.11 and local requirements.

I have read and understand the information in the Investigator's Brochure, including the potential risks and side effects of the study agents.

I agree to promptly report to the Ethics Committee (EC) all changes in the research activity and all unanticipated problems involving risk to participants. I will not make any changes to the conduct of the study without EC and Sponsor approval, except when necessary to eliminate apparent immediate harm to participants.

I agree to maintain adequate and accurate records and make those records available in accordance with ICH Guidelines for GCP section 4.11 and local requirements.

I agree to ensure that all associates, colleagues, and employees assisting in the conduct of the study are informed about their obligations in meeting the above commitments.

I understand that the study may be terminated or enrolment suspended at any time by the Sponsor, with or without cause, or by me if it becomes necessary to protect the best interest of the participants.

_______________________________ Date: ______________________________

Prof. James S McCarthy MBBS, Principal Investigator

This clinical trial protocol has been reviewed and approved by the Sponsor.

_______________________________ Date: ______________________________

Prof. David Whiteman, Deputy Director, QIMR Berghofer Medical Research Institute

PROTOCOL SUMMARY

**Title:**	An experimental study to characterise the *in vivo* safety and infectivity of a *Plasmodium falciparum* Cam3.II^{R539T} (K13) artemisinin-resistant isolate in healthy participants.
**Study Design:**	This study will evaluate the *in vivo* safety and infectivity of an *in vitro* expanded *Plasmodium falciparum* Cam3.II^{R539T} (K13) artemisinin-resistant isolate (referred to as K13) in healthy participants using the induced blood stage malaria model. The study will be conducted in 2 participants. Consenting and eligible male participants will be inoculated on Day 0 with around 2,800 viable *P. falciparum* K13 parasite-infected erythrocytes. Inoculation of the second participant will occur at least 2 weeks after inoculation of the first participant, after the Safety Review Team reviews the safety data from the first participant. On an outpatient basis, participants will be monitored daily via phone call and then will attend the clinic daily from Day 4 until positive for presence of malaria parasites by quantitative PCR (qPCR). Once qPCR positive, they will be monitored twice daily until antimalarial treatment, for adverse events and the onset of malaria symptoms, signs or parasitological evidence of malaria. On the day of commencement of antimalarial treatment, as determined by qPCR results (≥5,000 parasites/mL) or clinical symptoms of malaria infection (clinical symptom score >6) or at the Investigator's discretion, participants will be admitted to the study unit and confined for safety monitoring and a single oral dose of approximately 2 mg/kg artesunate (approximately Day 8). Participants will be followed up as in-patients for 72 hours to ensure tolerance of therapy and adequate clinical response. Once clinically well, participants will be followed up on an out-patient basis for monitoring of safety and clearance of malaria parasites via qPCR. It is anticipated that artesunate treatment will clear parasitaemia, although at a slower rate than for drug sensitive isolates. If artesunate does not clear parasitaemia, participants will be administered piperaquine, which is known to be active against *P. falciparum* K13 (as a single oral dose of Eurartesim® piperaquine tetraphosphate/dihydroartemisinin tablets). This will occur if qPCR results indicate unsatisfactory parasite clearance (2 consecutive qPCR time-points showing a decrease in parasitaemia of less than 20% of baseline by 72 hours post-artesunate treatment), or if recrudescence of parasitaemia occurs after artesunate treatment (defined by parasite count of ≥5,000 asexual blood stage parasites/mL and a 2-fold increase within 48 hours, or a clinical symptom score >6), or at the Investigator's discretion. The registered antimalarial Malarone® (proguanil hydrochloride/atovaquone; 3 day course) will be administered to all participants on Day 26±3 as a rescue treatment. Participants will be treated with a single oral dose of Primacin™ at the time of Malarone® treatment if gametocytes are determined to be present based on reverse

	transcriptase qPCR (qRT-PCR) to ensure complete clearance of gametocytes. Follow-up for safety assessments will be performed on Day 28±3, Day 56±7 (phone call only), and Day 90±14 (End of Study).
**Objectives:**	Primary:<br>• To determine the safety of infection with the *P. falciparum* K13 artemisinin-resistant blood stage parasite in healthy participants.<br>• To characterise the infectivity and growth curve of the *P. falciparum* K13 blood stage parasite in healthy participants.<br>Secondary:<br>• To define the parasite clearance profile of the *P. falciparum* K13 blood stage parasite after administration of antimalarial drug.<br>Exploratory:<br>• To determine if gametocytes appear in the blood of participants infected with the *P. falciparum* K13 blood stage parasite.<br>• To characterise the pharmacokinetic profile of artesunate and its active metabolite, dihydroartemisinin, in *P. falciparum* K13 infected participants.
**Endpoints:**	Primary:<br>• The safety of the *P. falciparum* K13 isolate will be determined by monitoring of adverse events and serious adverse events including severity and causality. Safety parameters that will be monitored include physical examination, clinical biochemistry, haematology, and urinalysis.<br>• *P. falciparum* K13 isolate infectivity will be determined by presence of parasites in participants after inoculation as measured by qPCR. To visualise parasite growth curves, the number of parasites over time will be represented.<br>Secondary:<br>• To characterise the parasite clearance profile, the parasite reduction ratio (PRR) after artesunate treatment will be determined.<br>Exploratory:<br>• The presence of gametocytes in participants will be determined by qRT-PCR until approximately Day 28.<br>• The following pharmacokinetic parameters will be calculated for artesunate and dihydroartemisinin: AUC_{last}, AUC_{inf}, C_{max}, T_{max} and $t_{1/2}$.

**Population:**	2 healthy adult male participants, aged between 18-55 years old, who do not live alone from Day 0 until at least the end of the Malarone® treatment, and will be contactable and available for the duration of the trial and follow-up period.
**Phase:**	Phase I
**Number of Sites Enrolling Participants:**	1
**Description of Study Agents:**	**Investigational product:**<br><br>*P. falciparum* K13 blood stage challenge inoculum<br><br>A naturally occurring *P. falciparum* K13 artemisinin-resistant isolate was *in vitro* expanded to make a blood stage *P. falciparum* K13 Master Cell Bank. Each K13 challenge inoculum dose will be prepared aseptically from a single aliquot of the *P. falciparum* K13 Master Cell Bank. Each participant will be inoculated intravenously with a dose of around 2,800 viable *P. falciparum* K13-infected erythrocytes in 2 mL saline for injection.<br><br>**Antimalarial drugs:**<br><br>Artesunate<br><br>Artesunate is a semi-synthetic derivative of artemisinin. Artesunate 50 mg tablets for oral administration will be used. Participants will receive a single dose of artesunate tablets of approximately 2 mg/kg.<br><br>Eurartesim®<br><br>If artesunate does not clear the parasites, participants will be administered a single oral dose of Eurartesim® (3 tablets, each containing 320 mg piperaquine tetraphosphate and 40 mg dihydroartemisinin).<br><br>Malarone®<br><br>Malarone® will be administered to all participants on Day 26±3 as a rescue treatment. A treatment course consists of 4 tablets of Malarone® (proguanil hydrochloride 100 mg, atovaquone 250 mg) once daily, orally for 3 days.<br><br>Primacin™ (if required)<br><br>If gametocytes are determined to be present based on qRT-PCR at the time of Malarone® treatment, Primacin™ (6 tablets of primaquine phosphate equivalent to 45 mg primaquine) will be administered as a single oral dose.
**Study Duration:**	Approximately 5 months.

**Participant Duration:**	A screening period of up to 28 days plus the 90 day study.

SCHEMATIC OF STUDY DESIGN

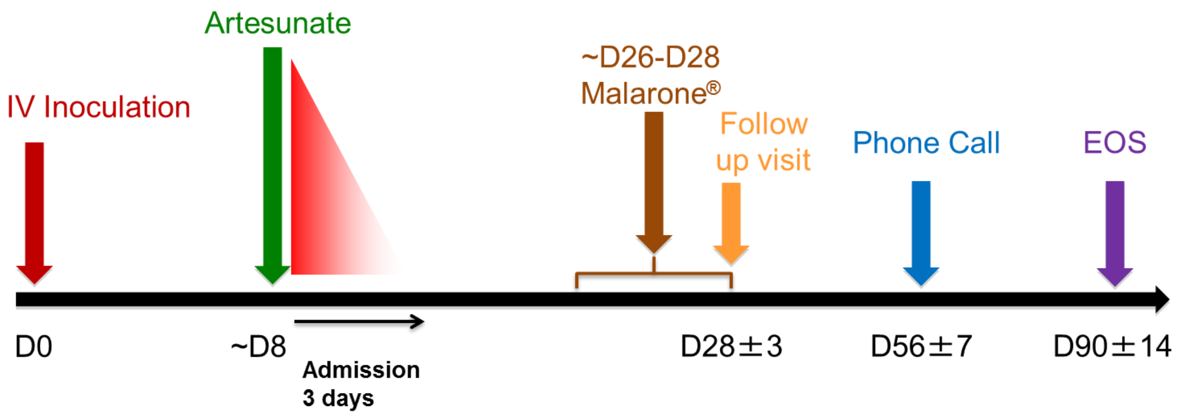

1.0 KEY ROLES

Principal Investigator:	Dr James McCarthy MBBS<br>Employed by and located at:<br>Q-Pharm Pty Ltd (visiting Medical Officer) and<br>QIMR Berghofer Medical Research Institute<br>Level 5, 300C Herston Road<br>Herston, QLD 4006<br>Australia<br>Tel: +61 (0)7 3845 3647 or +61 (0)7 3845 3636<br>Mobile: +61 4144204659<br>Email: j.mccarthy@uq.edu.au
Co-Investigators:	Dr Paul Griffin MBBS FRACP FRCPA<br>Q-Pharm Pty Ltd<br>Level 5, 300C Herston Road<br>Herston, QLD 4006<br>Australia<br>Tel: +61 (0)7 3845 3647<br>Fax: +61 (0)7 3845 3637<br>Email: paul.griffin@infectiousdiseasesqld.com.au<br><br>Dr Mark Armstrong<br>Level 5, 300C Herston Road<br>Herston, QLD 4006<br>Australia<br>Tel: +61 (0)7 3845 3636<br>Email: M.Armstrong@qpharm.com.au<br><br>Dr Anand Odedra<br>Level 5, 300C Herston Road<br>Herston, QLD 4006<br>Australia<br>Tel: +61 (0)7 3845 3636<br>Email: Anand.Odedra@qimrberghofer.edu.au
Independent Medical Monitor:	Professor Dennis Shanks<br>Australian Army Malaria Institute<br>Gallipoli Barracks<br>Enoggera, QLD 4051 Australia

	Tel: +61 (0)7 3332 4931<br>Email: Dennis.SHANKS@defence.gov.au
Statistician:	Prof Peter O'Rourke<br>Statistical Unit<br>QIMR Berghofer Medical Research Institute<br>Herston, QLD 4006<br>Australia<br>Tel: +61 (0)7 3845 3579<br>Email: peter.orourke@qimrberghofer.edu.au
Trial Sponsor and Local Australian Sponsor:	QIMR Berghofer Medical Research Institute<br>300 Herston Rd, Herston, QLD 4006<br>Tel: +61 (0)7 3362 0222
Authorised Sponsor Signatory	Prof. David Whiteman<br>Deputy Director<br>QIMR Berghofer Medical Research Institute<br>Tel: +61 (0)7 3362 0222
Sponsors Monitors	Clinical Network Services (CNS) Pty Ltd<br>Level 4, 88 Jephson St<br>Toowong QLD 4066, Australia<br>Tel: +61 (0)7 3719 6000
Institutional Ethics Committee	QIMR Berghofer Medical Research Institute Human Research Ethics Committee (QIMR Berghofer-HREC; EC00278)<br>Locked Bag 2000, Royal Brisbane and Women's Hospital, Brisbane, QLD 4029<br>Tel: +61 (0)7 3362 0117
Clinical Study Centre:	Q-Pharm Pty Limited<br>Level 5, 300C Herston Road and<br>Level 6, Block 8, Royal Brisbane and Women's Hospital<br>Herston QLD 4006<br>Tel: +61 (0)7 3845 3636
Clinical Laboratories:	Sullivan Nicolaides Pathology Central Laboratory (SNP)<br>24 Hurworth Street<br>Bowen Hills, QLD 4006<br>Australia<br>Tel: +61 (0)7 3377 8782
	Queensland Paediatric Infectious Diseases Laboratory

	(Q-PID), SASVRC<br>Level 8, Centre for Children's Health Research<br>62 Graham Street, South Brisbane, QLD 4101, Australia<br>Tel: +61 (0)7 3069 7464
	Brett McWhinney<br>Analytical Chemistry Unit<br>Department of Chemical Pathology<br>Pathology Queensland<br>Level 3, Block 7<br>Royal Brisbane and Women's Hospital<br>Brisbane QLD 4029

2.0 INTRODUCTION

2.1 Background Information

Malaria is one of the most important infectious diseases, which threatens half the world's population. In accordance with the latest estimates published by the World Health Organization (WHO) in 2015, there were around 3.2 billion people at risk of this parasitic disease with an estimated 214 million cases worldwide and 438,000 deaths [1]. Most of the malaria mortality was reported in sub-Saharan Africa and in children under 5 years of age [1]. Ill-fated efforts to eradicate malaria in the 1960s facilitated the emergence of resistance to both antimalarial chemotherapeutic agents and insecticides and precipitated concerns with regards to the ecological impact of vector control strategies. The WHO has declared that the response to malaria is a global development priority and has changed their recommendation from control to eradication programs.

The widespread resistance of *P. falciparum* to conventional monotherapies such as chloroquine, amodiaquine, sulfadoxine/pyrimethamine has led to an urgent need for new therapies. To combat concern for increasing levels of resistance, the WHO currently recommends artemisinin-based combination therapy as the first-line therapy in areas with high prevalence of resistance [1]. Even though the artemisinins are the most potent and rapidly acting antimalarial agents available to date [2], they have been associated with high recrudescence rates when used as monotherapy. Although the rate of recrudescence decreases when combination therapies are used, resistance to combination therapies has been reported across the Greater Mekong Subregion [3, 4]. Because of the concern for resistance, new drugs with new mechanisms of actions are needed. A robust pipeline of potential drug candidates is required, as well as efficient systems to determine the safety and clinical efficacy of these drugs.

Controlled human malaria infection (CHMI) is increasingly being used to evaluate antimalarial drug candidates [5-18]. Validation studies have shown a high correlation between natural and experimental infections, which further justifies the use of CHMI for testing new vaccines or drugs [13, 14, 18]. One type of CHMI is the induced blood stage malaria (IBSM) model, whereby participants are infected with blood stage malaria parasites. The IBSM model offers an attractive tool to test the efficacy of vaccines and drugs in non-immune participants in a rapid and cost effective manner. In the IBSM model, parasite growth after malaria challenge is monitored by quantitative PCR (qPCR) [19]. By following the parasitaemia decay in participants after drug or vaccine treatment, the antimalarial efficacy of a treatment can be determined.

For the IBSM model, blood stage parasite cell banks have been generated following collection of blood from either donors who have been exposed to laboratory developed *Plasmodium* strains, or donors who have acquired malaria naturally while visiting malaria endemic countries. So far, 3 *P. falciparum* and 2 *P. vivax* parasite cell banks have been used to challenge a total of 324 participants in experimental infections in 29 clinical trials, 25 of which were successfully undertaken at QIMR Berghofer/Q-Pharm [5-12, 15-17, 20-38]. A new parasite cell bank has recently been generated from a *P. falciparum* Cam3.II^{R539T} (K13) artemisinin-resistant malaria parasite isolated from a patient with natural malaria infection, and has been validated for use in the IBSM model.

Artemisinin resistance has been linked to mutations in the propeller domain of the *P. falciparum K13* gene [39]. The so-called artemisinin-resistant isolates have slower clearance, and therefore in severe malaria rely on the activity of the partner drug. A study in Cambodia, where artemisinin resistance is

a problem, showed that an artesunate dose of 2 mg per kg of body weight decreased *P. falciparum* parasites by 80% within 24 hours (Fig. 1)[40]. Therefore, in this proposed study with a *P. falciparum* K13 artemisinin-resistant isolate from Cambodia, approximately 2 mg/kg artesunate will be used to characterise the clearance profile of the isolate.

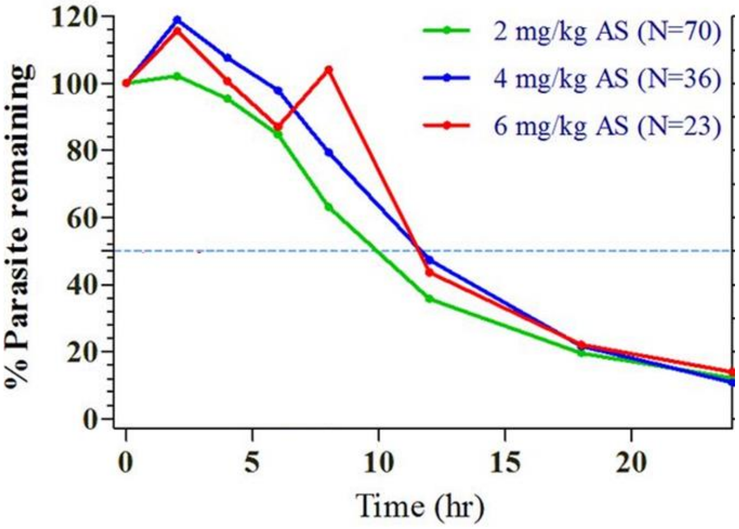

Figure 1: Parasite clearance as mean percentage of baseline values over 24 hours with different doses of artesunate. Modified from Saunders *et al.* 2012 [40].

2.2 Rationale

This study aims to evaluate the *in vivo* safety and infectivity of a *P. falciparum* Cam3.II^{R539T} (K13) artemisinin-resistant malaria parasite (referred to as K13) in healthy participants using the IBSM model. The *P. falciparum* K13 parasite was isolated from a patient in Cambodia [39] and was amenable to *in vitro* expansion. We expect that artesunate treatment will clear parasitaemia from participants inoculated with this artemisinin-resistant isolate, although at a slower rate compared to drug sensitive isolates. In addition, the *P. falciparum* K13 isolate retains sensitivity to piperaquine and atovaquone as determined by *in vitro* antimalarial drug susceptibility tests. Participants will be treated with artesunate to characterise the parasite clearance profile of *P. falciparum* K13 in response to artesunate. If artesunate does not clear parasitaemia from participants, or recrudescence occurs, participants will be treated with piperaquine (Eurartesim® tablets, which contain piperaquine tetraphosphate/dihydroartemisinin). The availability of a *P. falciparum* artemisinin-resistant isolate in the IBSM model will allow investigation of the efficacy of novel antimalarial drug candidates in clearance of artemisinin-resistant *P. falciparum*, which is a growing problem in South-East Asia and threatens to spread across the world.

2.3 Potential Risks and Benefits

2.3.1 Known Potential Risks

2.3.1.1 Induced Blood Stage Malaria (IBSM) Model Risks

Potential risks have been identified through review of previous clinical studies conducted to date using the IBSM model with other *P. falciparum* isolates, as well as review of the literature. A different isolate, *P. falciparum* 3D7, has been used to inoculate 296 volunteers in 20 IBSM studies [5-10, 15-17, 20, 22-30, 32-34, 36, 38, 41, 42]. Refer to the *P. falciparum* K13 Investigator's Brochure for more details [43].

Risk management of liver function derangements

Transient, asymptomatic liver function derangements reaching the threshold of serious adverse event (SAE) have been reported in several participants in IBSM studies, and may have been related to the challenge inoculum [44]. As a precaution all participants in this study will undergo regular safety monitoring to assess for asymptomatic liver function test abnormalities. Participants are required to reduce intake of possibly hepatotoxic substances during the course of the study including alcohol and paracetamol. No SAEs other than asymptomatic liver function derangements related to the challenge inoculum have been reported in any of the IBSM studies.

Risk management of blood borne infections

In this study, a cryopreserved inoculum containing *in vitro* expanded *P. falciparum* isolate K13 will be used. The *P. falciparum* K13 Master Cell Bank (Q-Gen ID: MBE-018) was produced using O Rh(D) Negative blood from the Australian Red Cross Blood Service (Blood Service). Overall, the risk of infection from a possible blood borne viruses from the blood transfused in this study would be expected to be very low for a number of reasons. Firstly, the Blood Service donor was screened and tested negative for presence of active blood borne infections. Secondly, white cells were removed from the donor blood as part of the Blood Service production process, which also lowers the risk of transfusion transmitted infections. Thirdly, the volume of blood used in the IBSM model for transmitting malaria is significantly lower than in a transfused unit, thus reducing the risk of infection. As part of the safety monitoring, all study participants will have serum stored for testing of blood-borne virus infections before and after the study (Appendix 2).

Risk management of reaction to the blood sample

The injection of parasites contains a small number of red blood cells (RBCs) from the Blood Service donor. The risk of developing RBC alloantibodies in this study is considered extremely low since the blood donor was blood group O Rh(D) Negative. People with this blood group are generally considered "universal donors", as recipients of their blood have minimal risk of developing RBC alloantibodies when given much larger volumes of blood than is used in the IBSM model. However, it is possible that participants could suffer a transfusion reaction after they receive the inoculum, or develop alloantibodies to the donor RBCs that may make blood transfusion more difficult in the future. Therefore, the presence of RBC alloantibodies will be monitored immediately after inoculation and at the end of the study in all participants as part of their safety monitoring.

Risk management of malaria infection

The number of blood stage parasites used to infect the participants in this study is much lower than that which reaches the blood after the bite of a single malaria-infected mosquito, where approximately 30,000 parasites are released into the blood when they break out of a single infected liver cell [45]. For this study, following administration of the malaria challenge inoculum, the growth of the parasites as well as any symptoms in the participants will be closely monitored. The threshold for commencement of antimalarial drug treatment with artesunate will be when participants reach ≥5,000 parasites/mL or have a malaria clinical symptom score >6 (Appendix 6, adapted from [46]), or at the Investigator's discretion. The treatment thresholds defined for this study are below the point at which clinically significant symptoms of malaria infection are likely to occur.

Risks specific to the K13 challenge inoculum

The K13 parasite used in this study is artemisinin-resistant, and therefore it is expected to show a slower clearance in response to artesunate treatment. However, if artesunate fails to clear parasitaemia from the blood of participants or there is recrudescence, they will be administered a drug known to be active against this isolate (piperaquine). Participants will also be treated with the registered antimalarial drug Malarone® to ensure parasite clearance. If participants comply with the curative antimalarial regimen as directed by the Investigator, there is no serious risk of clinical malaria.

2.3.1.2 Artesunate Risks

Possible adverse effects arising from the use of artesunate can be found in the WHO Public Assessment Report (2011) for artesunate 50 mg tablets [47]. These include mild electrocardiogram changes (QTc and PR increase), atrial extrasystoles, non-specific T-wave changes, gastrointestinal disturbances (nausea, vomiting, abdominal pain, diarrhoea), and dizziness. Participants will be counselled that certain adverse effects, for example dizziness, may impact on the performance of skilled tasks such as driving. Study participants will be confined for 72 hours after administration of artesunate to monitor for any adverse effects.

2.3.1.3 Piperaquine/dihydroartemisinin Risks

Piperaquine/dihydroartemisinin is well tolerated both in adults and in children [48], with the main adverse events reported to be gastrointestinal disturbance such as diarrhoea [49], although this varies considerably according to geographical region. Electrocardiographic effects of piperaquine/dihydroartemisinin have been specifically evaluated in two studies [50, 51]. Both demonstrated a prolongation of the corrected QT interval during treatment (between 11 and 14ms). Very few individual patients experienced a prolongation that could be regarded as clinically significant (>60ms); of note, the QTc prolongation induced by piperaquine/dihydroartemisinin has not been reported to be associated with clinically relevant cardiovascular events, suggesting a proarrhythmic effect. Therefore, although statistically significant, the QTc prolongation observed following piperaquine/dihydroartemisinin therapy is unlikely to be of clinical concern. European regulatory authorities have, however, advised that the drug Eurartesim® (piperaquine tetraphosphate/dihydroartemisinin) not be administered with food (to reduce peak concentrations),

and caution that prior and post electrocardiographic monitoring be undertaken, and avoidance on concomitant recent exposure to drugs at risk of QTc prolongation [52, 53].

The main risks identified in previous studies of piperaquine/dihydroartemisinin have been:

- Mild elevations in hepatic enzymes; transaminase elevations in malaria patients have typically been <2xULN, with no increases >5xULN, and with no severe liver function derangements (Hy's law cases) observed. The pattern of transaminase increases is not unusual with acute malaria, although there was a suggestion of a potentially dose-related effect.
- QTc prolongation (both QTcB and QTcF); although mostly in the range >30 msec but <60 msec, prolongations >60 msec have been observed with a single instance of QTcF that exceeded 500 msec. This risk is mitigated by administering the drug while the subject is fasting.

See the Eurartesim® product information for more details [52-54].

Piperaquine phosphate alone (not with dihydroartemisinin) has been used in previous IBSM studies and was well tolerated [8, 27, 28]. In these studies piperaquine treatment demonstrated a robust safety profile in doses up to 960 mg when used for the treatment of uncomplicated *P. falciparum* malaria infection.

2.3.1.4 Malarone® and Primacin™ Risks

Malarone® and Primacin™ risks are detailed in their respective approved manufacturer's prescribing information (Appendix 5).

2.3.2 Risk Management

In summary, the risk to participants in this study will be minimised as follows:

- Adherence to the inclusion/exclusion criteria will be maintained to ensure that only participants who are not at any perceived risk are enrolled in the study.
- Close clinical and laboratory monitoring to ensure the safety and well-being of the participants.
- Admission to the clinical trial unit for close medical supervision for 72 hours following initiation of artesunate treatment.
- The total volume of blood drawn from each participant will not exceed a standard unit of blood (approximately 450 mL) over any 30 day period.

The overall risk to the participants in the study is considered to be minimal and acceptable, and the potential of future improved treatment for malaria is considered to outweigh these potential risks.

2.3.3 Known Potential Benefits

There are no known direct benefits to the participants in this study. There may be a benefit to the participants from the results of the screening tests and procedures (blood tests, physical examination and electrocardiogram).

3.0 OBJECTIVES AND PURPOSE

Primary:

- To determine the safety of infection with the *P. falciparum* K13 artemisinin-resistant blood stage parasite in healthy participants.
- To characterise the infectivity and growth curve of the *P. falciparum* K13 blood stage parasite in healthy participants.

Secondary:

- To define the parasite clearance profile of the *P. falciparum* K13 blood stage parasite after administration of antimalarial drug.

Exploratory:

- To determine if gametocytes appear in the blood of participants infected with the *P. falciparum* K13 blood stage parasite.
- To characterise the pharmacokinetic profile of artesunate and its active metabolite, dihydroartemisinin, in *P. falciparum* K13 infected participants.

4.0 STUDY DESIGN AND ENDPOINTS

4.1 Study Design

This is a phase I, single-centre, open-label study using the IBSM model to characterise the safety and infectivity of an *in vitro* expanded *P. falciparum* K13 isolate in healthy malaria-naïve participants. The study will be conducted in 2 participants. Malaria parasite inoculation of the second participant will occur at least 2 weeks following inoculation of the first participant. A review of data from the first participant will be conducted by the Safety Review Team (SRT) prior to inoculation of the second participant. Safety, tolerability and parasitaemia data for a minimum of 14 days will be required for the review.

Each consenting and eligible male participant will be inoculated on Day 0 with ~2,800 viable *P. falciparum* K13-infected human erythrocytes administered intravenously. On an outpatient basis, participants will be monitored daily via phone call and then will attend the clinic daily (AM) from Day 4 until qPCR positive for presence of malaria parasites. Once qPCR positive they will be monitored twice daily, morning (AM) and evening (PM), until artesunate antimalarial treatment, for adverse events and the unexpected early onset of symptoms, signs or parasitological evidence of malaria. Microscopic examination for evidence of parasitaemia may be conducted at the discretion of the Investigator. On the day designated for commencement of antimalarial treatment, as determined by qPCR results (parasitaemia ≥5,000 parasites/mL) or a malaria clinical symptom score >6 (Appendix 6, adapted from [46]) or at the Investigator's discretion, participants will be admitted to the study unit and confined for safety monitoring and a single oral dose of approximately 2 mg/kg artesunate (Appendix 7). Based on previous studies with another *P. falciparum* isolate (3D7) it is anticipated that treatment may occur on approximately Day 8.

Following artesunate treatment, participants will be followed up as in-patients for 72 hours to ensure treatment tolerance and adequate clinical response. Once clinically well, participants will be followed up on an out-patient basis for monitoring of safety and clearance of malaria parasites via qPCR. The plasma concentration-time profiles of artesunate and its active metabolite dihydroartemisinin will be assessed from blood samples collected pre-dose and then following artesunate administration. Wherever possible, this pharmacokinetic (PK) sampling will coincide with post-dose blood collection for qPCR monitoring of parasitaemia.

It is anticipated that artesunate treatment will result in slower clearance of infection than for the reference strain *P. falciparum* 3D7. However, if parasite clearance does not occur, participants will be administered piperaquine, which is known to be active against this isolate, as a single dose of Eurartesim® tablets (piperaquine tetraphosphate/dihydroartemisinin). This will occur if qPCR results indicate unsatisfactory parasite clearance (2 consecutive qPCR time-points showing a decrease in parasitaemia of less than 20% of baseline by 72 hours post-artesunate treatment), or if recrudescence of parasitaemia occurs after artesunate treatment (defined by parasite count of ≥5,000 asexual blood stage parasites/mL and a 2-fold increase within 48 hours, or if clinical symptom score is >6), or at the Investigator's discretion.

Participants may also be evaluated for the presence of sexual parasite stages (gametocytes) in the blood, as determined by qRT-PCR of *pfs25* gametocyte-specific transcript, and/or male gametocyte marker, and/or ring-stage marker as appropriate, from approximately the day of artesunate

treatment [42]. qRT-PCR and microscopic examination for confirmation of gametocytaemia may be conducted at the discretion of the Investigator.

The registered antimalarial Malarone® (proguanil hydrochloride/atovaquone; 3 day course) will be administered to all participants at Day 26±3 as a rescue treatment. Participants will be treated with a single oral dose of Primacin™ (6 tablets of primaquine phosphate equivalent to 45 mg primaquine) at the time of Malarone® treatment if gametocytes are determined to be present based on qRT-PCR, to ensure complete clearance of gametocytes.

Follow-up for safety assessments will be performed on Day 28±3, Day 56±7 (phone call only), and Day 90±14 (End of Study). The overall period of participation will therefore be around 13 weeks from the time malaria infection. Participants are required to be contactable and available up to 2 weeks following the End of Study (EOS) visit (See Schedule of Events Appendix 1).

Adverse events will be monitored via telephone monitoring, during confinement within the clinical research unit, and on out-patient review following malaria challenge inoculation and administration of antimalarial treatment. Blood samples for safety evaluation, malaria monitoring, and PK determination of artesunate and dihydroartemisinin, will be drawn at nominated times.

4.2 Endpoints

Primary:

- The safety of the *P. falciparum* K13 isolate will be determined by monitoring of adverse events and serious adverse events including severity and causality. Safety parameters that will be monitored include physical examination, clinical biochemistry, haematology, and urinalysis.
- *P. falciparum* K13 isolate infectivity will be determined by presence of parasites in participants after inoculation as measured by qPCR. To visualise parasite growth curves, the number of parasites over time will be represented.

Secondary:

- To characterise the parasite clearance profile, the parasite reduction ratio (PRR) after artesunate treatment will be determined.

Exploratory:

- The presence of gametocytes in participants will be determined by qRT-PCR until approximately Day 28.
- The following pharmacokinetic parameters will be calculated for artesunate and dihydroartemisinin: AUC_{last}, AUC_{inf}, C_{max}, T_{max} and $t_{1/2}$.

5.0 STUDY ENROLLMENT AND WITHDRAWAL

5.1 Participant Inclusion Criteria

Participants eligible for inclusion in this study must fulfil **all** of the following criteria:

Demography

I 01. Adult male participants between 18 and 55 years of age inclusive, who do not live alone (from Day 0 until at least the end of the Malarone® treatment) and will be contactable and available for the duration of the trial and follow-up period (maximum 15 weeks).

I 02. Body weight minimum 50 kg, body mass index between 18 and 32 kg/m^2, inclusive.

Health status

I 03. Certified as healthy by a comprehensive clinical assessment (detailed medical history and complete physical examination).

I 04. Vital signs after 5 minutes resting in supine position:

90 mmHg ≤ systolic blood pressure (SBP) ≤ 140 mmHg,

50 mmHg ≤ diastolic blood pressure (DBP) ≤ 90 mmHg,

40 bpm ≤ heart rate (HR) ≤ 100 bpm.

I 05. Normal standard 12-lead electrocardiogram (ECG) after 5 minutes resting in supine position, QTcF≤450 ms with absence of second or third degree atrioventricular block or abnormal T wave morphology at screening and at pre-inoculation on Day 0.

I 06. Laboratory parameters within the normal range, unless the Investigator considers an abnormality to be clinically irrelevant for healthy participants enrolled in this clinical investigation in accordance with approved clinically acceptable laboratory ranges documented prior to study start. More specifically, for serum creatinine, hepatic transaminase enzymes (aspartate aminotransferase, alanine aminotransferase), and total bilirubin (unless the participant has documented Gilbert syndrome) should not exceed the approved acceptable ranges and haemoglobin must be equal or higher than the lower limit of the normal range.

Regulations

I 07. Having given written informed consent prior to undertaking any study-related procedure.

5.2 Participant Exclusion Criteria

Participants fulfilling **any** of the following criteria will not be eligible for inclusion in this study:

Medical history and clinical status

E 01. Any history of malaria or participation to a previous malaria challenge study.

E 02. Must not have travelled to or lived (>2 weeks) in a malaria-endemic region during the past 12 months or planned travel to a malaria-endemic region during the course of the study (for endemic regions see http://www.map.ox.ac.uk/browse-resources/).

E 03. Has evidence of increased cardiovascular disease risk (defined as >10%, 5 year risk for those greater than 35 years of age, as determined by the Australian Absolute Cardiovascular Disease

Risk Calculator (http://www.cvdcheck.org.au/). Risk factors include sex, age, systolic blood pressure (mm/Hg), smoking status, total and HDL cholesterol (mmol/L), and reported diabetes status.

E 04. History of splenectomy.

E 05. Presence or history of drug hypersensitivity, or allergic disease diagnosed by an allergist/immunologist and/or treated by a physician for allergy or history of a severe allergic reaction, anaphylaxis or convulsions following any vaccination or infusion.

E 06. Presence of current or suspected serious chronic diseases such as cardiac or autoimmune disease (HIV or other immuno-deficiencies), insulin-dependent and non-insulin dependent diabetes (excluding glucose intolerance if E03 is met), progressive neurological disease, severe malnutrition, acute or progressive hepatic disease, acute or progressive renal disease, porphyria, psoriasis, rheumatoid arthritis, asthma, epilepsy or obsessive compulsive disorder.

E 07. History of malignancy of any organ system (other than localised basal cell carcinoma of the skin or *in situ* cervical cancer), treated or untreated, within 5 years of screening, regardless of whether there is evidence of local recurrence or metastases.

E 08. Participants with history of schizophrenia, bi-polar disease, or other severe (disabling) chronic psychiatric diagnosis including depression or receiving psychiatric drugs or who has been hospitalised within the past 5 years prior to enrolment for psychiatric illness, history of suicide attempt or confinement for danger to self or others.

E 09. Frequent headaches and/or migraines, recurrent nausea, and/or vomiting (more than twice a month).

E 10. Presence of acute infectious disease or fever (e.g. sub-lingual temperature ≥38.5°C) within the 5 days prior to inoculation with malaria parasites.

E 11. Evidence of acute illness within the 4 weeks prior to screening that the Investigator deems may compromise participant safety.

E 12. Significant inter-current disease of any type, in particular liver, renal, cardiac, pulmonary, neurologic, rheumatologic, or autoimmune disease by history, physical examination, and/or laboratory studies including urinalysis.

E 13. Participant has a clinically significant disease or any condition or disease that might affect drug absorption, distribution or excretion (e.g. gastrectomy, diarrhoea).

E 14. Participation in any investigational product study within the 12 weeks preceding the study.

E 15. Blood donation of any volume within 1 month before inclusion, or participation in any research study involving blood sampling (more than 450 mL/unit of blood), or blood donation to Australian Red Cross Blood Service (Blood Service) or other blood bank during the 8 weeks prior to the treatment drug dose in the study.

E 16. Participant unwilling to defer blood donations to the Blood Service for at least 6 months.

E 17. Medical requirement for intravenous immunoglobulin or blood transfusions.

E 18. Participant who has ever received a blood transfusion.

E 19. Symptomatic postural hypotension at screening, irrespective of the decrease in blood pressure, or asymptomatic postural hypotension defined as a decrease in systolic blood pressure ≥20 mmHg within 2-3 minutes when changing from supine to standing position.

E 20. History or presence of alcohol abuse (alcohol consumption more than 40 g per day) or drug habituation, or any prior intravenous usage of an illicit substance.

E 21. Tobacco use of more than 5 cigarettes or equivalent per day and unable to stop smoking for the duration of the study.

E 22. Ingestion of any poppy seeds within the 24 hours prior to the screening blood test (participants will be advised by phone not to consume any poppy seeds in this time period).

E 23. Excessive consumption of beverages containing xanthine bases, including Red Bull, chocolate etc., more than 400 mg caffeine per day (equivalent to more than 4 cups per day).

Interfering substance

E 24. Any vaccination within the last 28 days.

E 25. Any corticosteroids, anti-inflammatory drugs, immunomodulators or anticoagulants. Any participant currently receiving or having previously received immunosuppressive therapy, including systemic steroids such as:

a. adrenocorticotrophic hormone or inhaled steroids in dosages which are associated with hypothalamic-pituitary-adrenal axis suppression (1 mg/kg/day)
b. prednisone or its equivalent
c. chronic use of inhaled high potency corticosteroids (budesonide 800 µg per day or fluticasone 750 µg).

E 26. Any recent (<6 weeks) or current systemic therapy with an antibiotic or drug with potential antimalarial activity (i.e. chloroquine, piperaquine, benzodiazepine, flunarizine, fluoxetine, tetracycline, azithromycin, clindamycin, doxycycline etc.).

General conditions

E 27. Any participant who, in the judgment of the Investigator, is likely to be noncompliant during the study, or is unable to cooperate because of a language problem or poor mental development.

E 28. Any participant in the exclusion period of a previous study according to applicable regulations.

E 29. Any participant who cannot be contacted in case of emergency for the duration of the trial and up to 2 weeks following EOS visit.

E 30. Any participant who is the Investigator or any sub-investigator, research assistant, pharmacist, study coordinator, or other staff thereof, directly involved in conducting the study.

E 31. Any participant without a good peripheral venous access.

E 32. Male participant with a female partner who is pregnant or lactating from the time of administration of study medication.

Biological status

E 33. Positive result on any of the following tests: hepatitis B surface (HBs Ag) antigen, anti-hepatitis B core antibodies (anti-HBc Ab), anti-hepatitis C virus (anti-HCV) antibodies, anti-human immunodeficiency virus 1 and 2 antibodies (anti-HIV1 and anti-HIV2 Ab).

E 34. Positive urine drug test. Any drug listed in Appendix 2 in the urine drug screen unless there is an explanation acceptable to the medical investigator (e.g., the participant has stated in advance that they consumed a prescription or over-the-counter product which contained the detected drug) and/or the participant has a negative urine drug screen on retest by the

pathology laboratory. Any participant testing positive for acetaminophen (paracetamol) at screening may still be eligible for study participation, at the Investigator's discretion.

E 35. Positive alcohol breath test.

Specific to the study

E 36. Cardiac/QT risk:

- Family history of sudden death or of congenital prolongation of the QTc interval or known congenital prolongation of the QTc interval or any clinical condition known to prolong the QTc interval.
- History of symptomatic cardiac arrhythmias or with clinically relevant bradycardia. Electrolyte disturbances, particularly hypokalaemia, hypocalcaemia, or hypomagnesaemia.
- Electrocardiogram (ECG) abnormalities in the standard 12-lead ECG (at screening or pre-inoculation on Day 0) which in the opinion of the Investigator is clinically relevant or will interfere with the ECG analyses.

E 37. Known hypersensitivity to artesunate or any of its excipients, artemether or other artemisinin derivatives, piperaquine, proguanil/atovaquone, primaquine, or 4-aminoquinolines.

E 38. Unwillingness to abstain from consumption of grapefruit or Seville oranges from inoculation (Day 0) until end of Malarone® treatment.

E 39. Unwillingness to abstain from consumption of quinine containing foods/beverages such as tonic water, lemon bitter, from inoculation (Day 0) until end of Malarone® treatment.

E 40. Use of prescription drugs or non-prescription drugs or herbal supplements (such as St John's Wort), within 14 days or 5 half-lives (whichever is longer) prior to the malaria parasite inoculation. As an exception, ibuprofen (preferred) may be used at doses of up to 1.2 g/day, or paracetamol at doses of up to 4 g/day after discussion with the Investigator. Limited use of other non-prescription medications or dietary supplements not believed to affect participant safety or the overall results of the study, may be permitted on a case-by-case basis following approval by the Sponsor in consultation with the Investigator. Participants are requested to refrain from taking non-approved concomitant medications from recruitment until the conclusion of the study.

Participants who are excluded from participation on study days for any of the above reasons may be eligible to participate on a postponed schedule if the Investigator considers this appropriate.

5.3 Strategies for Recruitment

No restrictions will apply for ethnic or racial categories. The expected population may include all Australian racial categories. Participants will be recruited from the QIMR Berghofer Human Research Ethics Committee (QIMR Berghofer HREC) approved database of healthy participants maintained by Q-Pharm, or by a general or study specific advertisement via print, radio or poster media to students of Queensland universities or to the general community, as approved by the QIMR Berghofer HREC.

Participants who attend the clinic for a recruiting medical interview will be allocated a screening number. After providing written informed consent at the screening visit, participants will undergo eligibility screening including medical history, physical examination including an ECG, laboratory

investigations including haematology testing, liver and renal function tests, HIV and Hepatitis B and C screening, urinalysis, and RBC antibody testing.

5.3.1 Number of Participants

A total of 2 participants are planned to be enrolled. This would be sufficient to characterise the safety and infectivity of *P. falciparum* K13 isolate in healthy participants. It is estimated that up to 4-6 participants may need to be screened to complete enrolment.

At least 1 eligible participant and 1 reserve will attend the clinic on Day 0. If the nominated participant ceases to be eligible (e.g., because of a protocol violation) or fails to appear or is unable to proceed, the reserve participant will be enrolled.

Participants who are dosed but fail to complete the study for any reason will not be replaced as agreed by the Investigator and the Sponsor.

5.4 Participant Withdrawal or Termination

5.4.1 Reasons for Withdrawal or Termination

Participants are free to withdraw from the study at any time. A participant may be considered withdrawn if he states an intention to withdraw, fails to return for scheduled protocol visits for any reason, or becomes lost to follow-up. Participants may also be withdrawn by the Investigator. Possible reasons for withdrawal by the Investigator include the occurrence of a serious adverse event (SAE), failure by the participant to comply with the requirements of the protocol, or for any other reason at the Investigator's discretion. The reason for withdrawal should be clearly recorded in the participant's CRU (Q-Pharm) Clinic File and CRF.

If premature withdrawal occurs for any reason, the Investigator must make every effort to determine the primary reason for a participant's premature withdrawal from the study and record this information on the Study Completion CRF.

5.4.2 Handling of Participant Withdrawals or Termination

If the participant is withdrawn from the study procedures or follow-up for any reason, with the participant's permission, medical care will be provided for any SAEs that occurred during participation in the study until the symptoms of any SAEs are resolved and the participant's condition becomes stable. If earlier withdrawal from further study procedures occurs, the participant will be asked to complete the antimalarial treatment. The participants will also be asked to complete the early termination evaluation as described in Section 7.1.14.

5.5 Premature Termination or Suspension of Study

The Sponsor, Principal Investigator, HREC and Regulatory Authorities independently reserve the right to discontinue the study at any time for safety or other reasons. This will be done in consultation with the Sponsor where practical. In the occurrence of premature trial termination or suspension, the above mentioned parties will be notified in writing by the terminator/suspender stating the reasons for early termination or suspension (with the exception of the Sponsor's responsibility for notifying the Regulatory Authorities). After such a decision, the Sponsor and the Investigator will

ensure that adequate consideration is given to the protection of the participants' interest. The Investigator must review all participants as soon as practical and complete all required records.

5.5.1 Guidance for stopping rules

In addition to the classic assessment of SAEs and the occurrence/severity of other AEs by the Sponsor and the Investigator, after exploring potential confounding factors, the following criteria should be considered as guidance for the decision to stop inoculation of further participants:

- The participant experiences an SAE that is related to the inoculum.
- There is insufficient response to both artesunate 2 mg/kg dose and Eurartesim® dose.
- The Investigator and Sponsor may decide to stop inoculation based on other safety signals not described in the above criteria.

5.5.2 Obligations of the Sponsor

During the course of the study, the Sponsor will report in an expedited manner:

- All SAEs that are both unexpected and at least reasonably related to the challenge inoculum or the treatment (SUSAR), to the TGA and HREC as appropriate and to the Investigator.
- All SAEs that are expected and at least reasonably related to the challenge inoculum or the treatment to the TGA and HREC, according to local regulations.

The Sponsor will report all safety observations made during the conduct of the trial in the CSR.

6.0 STUDY AGENTS

6.1 *P. falciparum* K13 Blood Stage Challenge Inoculum

6.1.1 Preparation of the *P. falciparum* K13 Master Cell Bank

The *P. falciparum* Cam3.II^{R539T}/RF967 (K13) parasite was isolated from the blood of a patient in Pursat, Cambodia who presented with clinical malaria [39]. The parasite has a R539T mutation in the propeller domain of the *K13* gene which mediates artemisinin resistance. The isolate was collected by Dr Rick Fairhurst and his team as part of an ethically approved clinical study, and shipped to the Fairhurst Laboratory, National Institute of Allergy and Infectious Disease, National Institutes of Health, Rockville, Maryland, United States (U.S.).

The isolate was then sent to Professor David Fidock at Columbia University Medical Center, New York, New York (U.S.). The K13 parasites were cultured and expanded *in vitro* for approximately 4 months in the Fidock laboratory to produce a K13 parasite seed bank. Aliquots of the K13 parasite seed bank were sent to Professor Leann Tilley at The University of Melbourne, Melbourne, Australia. The seed bank was *in vitro* expanded, with new aliquots of the K13 parasite sent on dry ice from The University of Melbourne to Q-Gen (QIMR-B), Brisbane, Australia. The K13 seed bank samples were tested for mycoplasma and found to be negative by Eurofins ams Laboratories (Australia) before being used for further expansion and production of the K13 Master Cell Bank (MCB) at Q-Gen.

The K13 MCB (Q-Gen ID: MBE-018) was then produced at Q-Gen, a TGA licensed facility for production of cellular products for use in early clinical studies. Briefly, the process involved thawing the contents of one vial of the K13 seed bank and growing and expanding the parasites in culture, using RPMI 1640 media supplemented with 10% heat treated pooled human serum and human erythrocytes (2-4% haematocrit) and hypoxanthine thymidine supplement (0.1 mM sodium hypoxanthine and 0.016 mM thymidine). The operations in this process were performed aseptically in a cleanroom suitable for production of sterile biologicals in accordance with Good Manufacturing Practice. The K13 MCB was cryopreserved using Glycerolyte 57 in a 1:2.2 ratio, aliquoted into 1 mL cryovials, and stored in vapour phase liquid nitrogen under controlled conditions. A total of 192 vials of K13 MCB were produced. The K13 MCB was tested for blood borne infections with all results negative/not detected. The K13 MCB was then reviewed for compliance and released for use. Percentage parasitaemia and parasite life cycle stage of the K13 MCB was assessed by thin blood film (microscopy) prior to harvest, pooling and cryopreservation. At the time of harvest, the parasites were >82% ring-stage at >5.3% parasitaemia. As determined by *in vitro* antimalarial drug susceptibility tests, the K13 MCB is resistant to chloroquine, dihydroartemisinin, lumefantrine, mefloquine and quinine. The K13 MCB is sensitive to piperaquine (a component of Eurartesim®), atovaquone (a component of the antimalarial drug Malarone®), amodiaquine, and pyronaridine. Refer to the *P. falciparum* K13 Investigator's Brochure for more details [43].

6.1.2 Preparation of the Inoculum and Dosing

The inoculum for each participant will be prepared aseptically from a separate frozen aliquot of the *P. falciparum* K13 MCB. The inoculum will be prepared aseptically at Q-Gen (QIMR Berghofer) by nominated QIMR Berghofer staff members under the guidance of the Investigator. The infected erythrocytes will be thawed and washed, resuspended in saline, diluted in a final volume of 2 mL of clinical grade saline and dispensed into syringes. The dose of the inoculum will contain an estimated

~2,800 viable *P. falciparum* K13 parasite-infected erythrocytes. The actual number of parasites inoculated will take into account the loss of viability resulting from cryopreservation, storage and thawing. Following inoculation of each participant, the parasite count of the inoculum will be quantified by qPCR using previously described techniques [19].

6.1.3 Packaging, Labelling and Storage of the Inoculum

The time between preparation of the inoculum and administration to each participant will be a maximum of 2 hours, during which time the syringes containing the inoculum will be kept at the required temperature. The syringes will be double contained following the preparation and labelled in accordance with GCP guidelines. Any remainder of the unused cells will be discarded as per approved procedures.

6.1.4 Administration of the Inoculum

The inoculum, a volume of 2 mL containing an estimated ~2,800 viable *P. falciparum* K13-infected erythrocytes, will be administrated intravenously to each participant on the morning of Day 0. Participants will undergo intravenous cannulation with an appropriate gauge cannula. Placement and patency will be checked by flushing the vein with 5-10 mL of clinical grade saline. The inoculum will be injected, and the cannula again flushed with 5-10 mL of clinical grade saline. The cannula will then be removed, and haemostasis ensured by use of an appropriate dressing. Participants may have food until at least half an hour prior to inoculation.

6.2 Artesunate

6.2.1 Preparation of Artesunate

Artesunate is a semi-synthetic derivative of artemisinin. Artesunate 50 mg tablets for oral use will be acquired from Guilin Pharmaceutical Co., Ltd. The manufacture of artesunate is undertaken in a WHO Pre-Qualified GMP facility. Import was facilitated by Medicines for Malaria Venture.

6.2.2 Dosing and Administration of Artesunate

Artesunate tablets will be administrated orally after a ≥8 hour fast. Participants will receive a single dose of artesunate tablets of approximately 2 mg/kg (see Appendix 7 dosing table). This dose has been previously shown to be effective in reducing parasitaemia in patients with uncomplicated *P. falciparum* malaria (Angus 2002, Saunders 2012). Artesunate tablets should be swallowed with water and should not be taken with a high-fat meal. The dose will be given at Q-Pharm in the presence of clinical staff.

Note: If the participant vomits or cannot tolerate oral drugs then artesunate will be administered intravenously at the recommended dose regimen. This drug is the recommended parenteral treatment for malaria in Australia. Currently, it is a Special Access Scheme drug, and has been sourced from Guilin Pharmaceutical (Shanghai). Import was facilitated by Medicines for Malaria Venture. The manufacture of IV artesunate is undertaken in a WHO Pre-Qualified GMP facility (http://www.mmv.org/access/access-portfolio/artesun-injectable-artesunate).

6.3 Eurartesim®

If artesunate does not clear the parasites or if participants develop symptoms, they will be administered a single oral dose of Eurartesim® consisting of 3 tablets (see Section 7.1.9). Each Eurartesim® tablet contains 320 mg piperaquine tetraphosphate and 40 mg dihydroartemisinin. The total dose will be 960 mg piperaquine tetraphosphate and 120 mg dihydroartemisinin. It is recommended that Eurartesim® is administered after a ≥3 hour fast. Participants will be requested to fast for a further 3 hours after Eurartesim® treatment.

6.4 Malarone®

The dose administered will be as recommended by the manufacturer for treatment of malaria. A treatment course of Malarone® consists of 4 tablets of Malarone® (proguanil hydrochloride 100 mg, atovaquone 250 mg) once daily, orally for 3 days. Each dose should be taken with food or drinks rich in fat (e.g., milk). Participants will be reminded of the potential side effects of Malarone® and given the consumer information sheet for Malarone®. Participants may be administered Malarone® on site for initial dosing followed by monitoring, either in the clinic, or by telephone for 3 days to ensure adherence to Malarone® therapy.

6.5 Primacin™ (If Required)

Participants will be treated with Primacin™ at the time of Malarone® treatment, if gametocytes are determined to be present based on qRT-PCR, to ensure complete clearance of gametocytes. If needed, participants will take 6 Primacin™ tablets, each containing 13.2 mg primaquine phosphate equivalent to 7.5 mg primaquine base (the total primaquine dose will be 45 mg). Primacin™ for oral use will be taken as a single dose with food.

6.6 Packaging, Labelling and Storage of Antimalarial Drugs

Artesunate, Eurartesim®, Malarone® and Primacin™ will be acquired by Q-Pharm, labelled according to identity, brand or source, and batch number. The supplies will be held in appropriate locked storage conditions at Q-Pharm until required. The contents of the label for drug to be administered to the participants will be in accordance with all applicable regulatory requirements.

6.7 Product Accountability

The Q-Pharm pharmacist will document receipt conditions and time restrictions of use for the inoculum. The storage, handling and the disposal of the inoculum will be in accordance with approved procedures.

The antimalarial drugs will be inventoried prior to the beginning of study enrolment on study accountability logs in regards to condition upon receipt, including lot numbers and expiry dates. The Investigator or qualified study person designated by the Investigator will ensure that the received products are the specified formulation. The site pharmacist or a nominee designated by the Investigator is responsible for maintaining an accurate inventory and accountability record of all product supplies for this study. The antimalarial medications will be dispensed and accounted for in accordance with Q-Pharm standard procedures. All used medications will be fully documented.

Study products and study accountability logs will be available to the Sponsor or Sponsor's representative as part of the study monitoring procedures.

All dosages prescribed and dispensed to the participants and all dose changes during the study must be recorded on the Case Report Forms (CRF).

All drug supplies are to be used only in accordance with this protocol, and not for any other purpose.

Used and unused drug containers must be destroyed at the site once drug accountability is final and has been checked by the Sponsor or its delegate, and written permission for destruction has been obtained from the Sponsor.

7.0 STUDY PROCEDURES AND SCHEDULE

7.1 Study Schedule

The Schedule of Events (Appendix 1) summarises the procedures to be conducted as per this protocol during screening, confinement and post-confinement. Section 7.2 and Appendix 2 provide detailed information on the procedures.

An experienced nurse will be in attendance at the CRU when participants are on-site and the Investigator or Co-Investigators will be available within approximately 30 minutes call back if required.

In the schedule of events, evaluation may be in the morning, between 6:00 AM to 11:00 AM, and in the afternoon, between 6:00 PM to 11:00 PM, therefore separated by approximately 12 hours.

7.1.1 Screening Visit (Day -28 to Day -1)

A screening visit will be scheduled after an initial telephone interview conducted by clinical trial staff has occurred to review background information. For the screening visit, potential participants will be told to come to the clinical unit after an overnight fast of ≥8 hours. During this initial screening visit, the potential participant will read the Participant Information Sheet and be encouraged to ask questions. Participants willing to be considered for inclusion may sign the screening consent form during the screening visit, or return to the clinical unit after further consideration. The participant will be given a copy of the Participant Information Sheet and signed consent form for their records. The signed and dated originals will be held on file by the CRU. Participation consent must be obtained from all eligible participants prior to enrolment.

After providing written consent to participate, the participant will be examined by the medical investigator and physical examinations, vital signs and ECG testing will be done together with collection of blood and urine samples for safety assessment. The participants will be fully informed of the nature of the study at this time, and advised of the requirement to repeat some screening tests during the Day -3 to Day -1 safety visit (if required) and/or on the day of malaria challenge inoculum administration to determine their continuing eligibility. Participants must confirm that they will not be living alone from Day 0 until the end of the Malarone® treatment.

The pre-study screening will be conducted within 4 weeks prior to the Day 0 malaria challenge day and will include:

1. Provide the Participation Information Sheet and Informed Consent form and give the participant sufficient time to review the contents.
2. Explain the study via the Participation Information Sheet and gain Informed Consent from the participant.
3. Ensure the participant has signed the Participation Information Sheet and Informed Consent and received a signed copy.
4. A screening number will be assigned to each participant.
5. Elicit a complete medical history and use of concomitant medications.
6. Elicit a social history including alcohol and tobacco use.
7. Perform alcohol breath test.
8. Undertake a complete physical examination.

9. Assessment of the cardiovascular disease risk (defined as >10%, 5 year risk when greater than 35 years of age) as determined by the Australian Absolute Cardiovascular Disease Risk Calculator (http://www.cvdcheck.org.au/). Risk factors include sex, age, systolic blood pressure (mm/Hg), smoking status, total and HDL cholesterol (mmol/L), and reported diabetes status.
10. Record vital signs.
11. Obtain a 12-lead ECG.
12. Collect urine for urinalysis and urine drug screen.
13. Collect blood samples for haematology, biochemistry, RBC alloantibodies, glucose-6-phosphate dehydrogenase (G6PD) testing, and serology (viral hepatitis B and C and HIV).
14. Verify participant meets inclusion/exclusion criteria.

Participants who complete all screening procedures and satisfy all entry criteria will be considered eligible to participate in this study. To be eligible for study entry, laboratory values at screening must not be outside the range of the normal values at a level deemed to be clinically significant. For eligibility parameters a repeat may be requested to exclude laboratory error.

If screening laboratory results are abnormal, e.g. HIV testing, the volunteer will be referred for appropriate counselling. If any clinically significant abnormalities are detected during screening, the volunteer will be referred for follow-up tests to a general practitioner or medical specialist as appropriate.

7.1.2 Day -3 to Day -1 safety visit

Participants (including reserve participants) will report to the CRU between Day -3 to Day -1 for the following baseline assessments, unless screening laboratory assessments were conducted within this period, in which case repeat sampling will not be required.

1. Collect blood samples for haematology and biochemistry analysis.
2. Collect urine for urinalysis.

The timing of these assessments is to ensure that results are available for review by the Investigator prior to inoculation on Day 0. Participants with clinically significant laboratory findings at this stage will not be eligible for malaria parasite inoculation.

7.1.3 Administration of Malaria Challenge Inoculum (Day 0)

Each participant (and one reserve participant) will report to the CRU on the morning of Day 0. The Investigator will review the participants' screening results prior to their enrolment into the study. The Investigator will emphasise the requirement to return for malaria drug treatment after the malaria inoculation. Participants will be reviewed by the Investigator to confirm their continued eligibility for the study, including confirmation that they will not be living alone from Day 0 until the end of the Malarone® treatment by checking housemates contact details recorded at screening visit.

On admission to the study centre, participants will be required to undertake further screening procedures to determine whether they remain eligible to be enrolled. A reserve participant may be asked to replace a participant who does not continue to meet eligibility. These reserves will be compensated for the study visit even if not inoculated, as described in the Participant Information and Consent Form.

The procedures that will be undertaken prior to inoculation include:

1. Verify that all applicable eligibility criteria have been met.
2. Elicit information regarding any new medical conditions or illnesses since screening.
3. Perform alcohol breath test.
4. Conduct abbreviated physical examination.
5. Record vital signs.
6. Obtain a 12-lead ECG.
7. Collect urine for drug screen.
8. Cannulate participants with an indwelling intravenous cannula for the malaria inoculum, and record which arm is utilised.
9. Collect blood samples for malaria qPCR and safety serum storage.

Administration of the malaria inoculum:

1. Administer the malaria inoculum of ~2,800 viable *P. falciparum* K13-infected human erythrocytes intravenously in the morning (approximately 9:00 AM).
2. Observe for a minimum of 60 minutes after administration of the inoculum to evaluate for immediate adverse reactions. Vital signs will be repeated prior to leaving the clinic (approximately 60 minutes after inoculation).
3. Educate participants on signs and symptoms of malaria (Appendix 4).
4. Emphasise to participants the importance of returning on the nominated day (approximately Day 8), or as advised by the clinical staff, for malaria treatment.
5. Provide participants with diary cards and thermometers to record any temperature readings in the event of symptoms of fever.
6. Record adverse events and concomitant medications.

7.1.4 Days 1, 2, and 3 Post-Induced Infection

During this period, participants are expected to be asymptomatic. A daily phone call will be made to the participants by clinic staff to monitor participant well-being and to solicit any adverse events.

7.1.5 Day 4 AM until qPCR Positive for Malaria

Follow-up from Day 4 until qPCR becomes positive will be undertaken through daily visits (approximately 8:00 AM) to the clinical site. The following procedures will occur during these visits:

1. Perform abbreviated physical examination when signs or symptoms of malaria are identified and it is clinically indicated (Appendix 4).
2. Record vital signs.
3. Collect blood sample for malaria qPCR.
4. Record malaria clinical symptom score which will be used to guide when to treat with artesunate (Appendix 6).
5. Record adverse events and use of concomitant medications.

7.1.6 Day when qPCR Positive until Treatment Day

Follow-up from the day that qPCR becomes positive until treatment day will be undertaken through twice daily (AM & PM) visits separated by approximately 12 hours to the clinical site (i.e. 06:00 – 11:00 and 18:00 – 23:00). The following procedures will occur during these visits:

1. Perform abbreviated physical examination when signs or symptoms of malaria are identified and it is clinically indicated (Appendix 4).
2. Record vital signs.
3. Collect blood sample for malaria qPCR.
4. Record clinical symptom score (Appendix 6).
5. Record adverse events and use of concomitant medications.

7.1.7 Inpatient Observation and Antimalarial Treatment Phase (approximately Day 8)

When a participant reaches a parasitaemia level of ≥5,000 parasites/mL or their clinical symptom score is >6 or at the Investigator's discretion, the participant will be admitted to the CRU at the Q-Pharm clinical trials facility for 72 hours for artesunate treatment and monitoring of clinical features of malaria. Participants will adhere to restrictions regarding activity, food, and water intake during this period.

7.1.7.1 Admission

The following procedures will occur at admission to Q-Pharm:

1. Perform abbreviated physical examination (Appendix 4).
2. Perform alcohol breath test.
3. Record vital signs.
4. Obtain a 12-lead ECG.
5. Collect urine for urinalysis and urine drug screen.
6. Cannulate participants with an indwelling intravenous cannula, and record which arm is utilised.
7. Collect blood samples for haematology, biochemistry, malaria qPCR, and PK analysis (measurement of artesunate and dihydroartemisinin levels).
   NOTE: As these tests results may not be available before the drug administration, the results of this time-point will be used for proper interpretation of study results.
8. Collect blood samples for gametocyte qRT-PCR (if required). These samples will be used for detection of the gametocyte-specific mRNA transcript *pfs25*, and/or male gametocyte marker, and/or ring-stage marker as appropriate.
9. Record clinical symptom score (Appendix 6).
10. Record adverse events and use of concomitant medications.

7.1.7.2 Treatment and Observation

The following procedures will occur during treatment and observation:

1. Administer approximately 2 mg/kg artesunate under direct observation (see Appendix 7 dosing table). In the rare event that a participant requires hospitalisation, this will be done at the Infectious Diseases Unit, Royal Brisbane and Women's Hospital.
2. Follow up participants as in-patients for 72 hours to ensure tolerance of the therapy and adequate clinical response.
3. Perform abbreviated physical examination when signs or symptoms of malaria are identified and it is clinically indicated (Appendix 4).
4. Assess vital signs 3 times a day whilst confined.

5. Collect blood samples for malaria qPCR following treatment at approximately 2, 4, 6, 8, 12, 16, 20, 24, 28, 32, 36, 48, 60 and 72 hours (exit from unit).
6. Collect blood samples for gametocyte qRT-PCR (if required).
7. Collect blood samples for PK assay of artesunate and dihydroartemisinin following artesunate treatment at approximately 0.25, 0.5, 1, 1.5, 2, 2.5, 3, 4, 6, 8, 10, and 12 hours.
8. Record clinical symptom score (Appendix 6).
9. Record adverse events and use of concomitant medications.

7.1.7.3 <u>Prior to Exit from the CRU</u>

Participants will be allowed to leave the unit 72 hours after initiation of artesunate treatment at the Investigator's discretion if they are asymptomatic and have a normal examination and no clinically significant laboratory abnormalities.

The following procedures will occur prior to discharge from the CRU:

1. Perform abbreviated physical examination.
2. Record vital signs.
3. Collect blood samples for haematology, biochemistry, malaria qPCR (~72 hour time-point), and gametocyte qRT-PCR (if required).
4. Record adverse events and use of concomitant medications.

Participants will be asked to return approximately 12 hours after exit from the CRU for further malaria qPCR sampling and clinical assessment.

7.1.8 Out-Patient Safety Monitoring Post-Artesunate Treatment

Daily follow-up at either AM (approximately 08:00) or AM and PM (if necessary, around 12 hours apart) will be undertaken on an out-patient basis through visits post-confinement for clinical evaluation and blood sampling. These visits will occur until the malaria qPCR results are negative, or low and stable as detailed below.

The following procedures will take place during these visits:

1. Collect blood samples for malaria qPCR at AM or AM/PM post-confinement. Collect a blood sample for malaria qPCR at approximately 84 hours after artesunate treatment. Participants may then return morning and evening until parasite counts are <~500 parasites/mL, which is when sampling may revert to daily visits at the Investigator's discretion. When parasite counts are <~200 parasites/mL, sampling may revert to alternate day visits at the Investigator's discretion. Once the qPCR results are negative, or low and stable, participants may be reviewed 3 times per week until Malarone® treatment, and at Day 28±3.
2. If qPCR results suggest presence of gametocytaemia in participants' blood, additional blood would be collected simultaneously with qPCR blood samples. This sample will be used for detection of the gametocyte-specific mRNA transcript *pfs25*, and/or male gametocyte marker, and/or ring-stage marker as appropriate by qRT-PCR.
3. Collect blood samples for haematology and biochemistry, and urine samples for urinalysis on Day 14±2 and Day 18±2, or at the Investigator's discretion.
4. Perform abbreviated physical examination when signs or symptoms of malaria are identified and it is clinically indicated (Appendix 4).

5. Record vital signs.
6. Record clinical symptom score (Appendix 6).
7. Record adverse events and use of concomitant medications.

7.1.9 Eurartesim® Treatment

If artesunate fails to clear parasitaemia, participants will be administered piperaquine which is known to be active against this isolate (as a single dose of Eurartesim® tablets, which contain piperaquine tetraphosphate/dihydroartemisinin). Eurartesim® will be administered if the Investigator deems it clinically necessary after artesunate treatment. For example, Eurartesim® may be given as indicated by the following criteria:

- If qPCR results indicate unsatisfactory clearance of the parasitaemia (2 consecutive qPCR time-points showing a decrease in parasitaemia of less than 20% of baseline by 72 hours post-artesunate treatment). The decision to institute Eurartesim® treatment will be made in consultation with the Local Independent Medical Monitor (IMM), who is an external malaria expert, to advise on the safety on continuing observation without Eurartesim® treatment versus administration of Eurartesim®,

or

- If recrudescence of parasitaemia occurs after artesunate treatment (defined by parasite count of ≥5,000 asexual blood stage parasites/mL and a 2-fold increase within 48 hours, or if clinical symptom score is >6).

or

- At the Investigator's discretion.

It is recommended that Eurartesim® is administered at the CRU after a ≥3 hour fast. Participants will be requested to fast for a further 3 hours after Eurartesim® treatment.

The following procedures will be performed prior to Eurartesim® treatment and approximately 3 days after treatment (or at the next scheduled visit).

1. Record vital signs.
2. Obtain a 12-lead ECG.
3. Collect blood samples for malaria qPCR, haematology, and biochemistry.
4. Record adverse events and use of concomitant medications.

7.1.10 Malarone® Treatment

Participants will begin a course of standard Malarone® treatment on Day 26±3. Participants may take the doses at the CRU or at home, as determined by the Investigator. Participants will receive a phone call from clinic staff to check on symptoms and ensure compliance/completion with treatment following the doses taken at home.

The following procedures will be performed prior to Malarone® treatment and after completion of Malarone® treatment (or at the next scheduled visit).

1. Record vital signs.
2. Collect blood samples for haematology and biochemistry.

3. Record adverse events and use of concomitant medications.

If gametocytes are determined to be present based on qRT-PCR at the time of Malarone® treatment, Primacin™ (6 tablets of primaquine phosphate equivalent to 45 mg primaquine) will be administered as a single oral dose.

7.1.11 Follow-up Visit (Day 28±3)

The following procedures will occur at the follow-up visit on Day 28±3:

1. Elicit information regarding any new medical conditions or illnesses.
2. Complete physical examination.
3. Record vital signs.
4. Obtain a 12-lead ECG.
5. Collect urine for urinalysis.
6. Collect blood samples for haematology, biochemistry, malaria qPCR, gametocyte qRT-PCR (if required), serology, RBC alloantibodies, and safety serum storage.
7. Record adverse events and use of concomitant medications.

7.1.12 Follow-up Phone Call (Day 56±7)

A phone call will be made on Day 56±7 to the participants by clinic staff to monitor participant well-being and to solicit any adverse events.

7.1.13 Day 90±14 or End of Study (Final Visit)

The following procedures will occur at the EOS visit:

1. Elicit information regarding any new medical conditions or illnesses.
2. Perform abbreviated physical examination.
3. Record vital signs.
4. Collect blood samples for RBC alloantibodies and safety serum storage (2 serum samples).
5. Record adverse events and use of concomitant medications.

7.1.14 Early Termination Visit

If voluntary withdrawal occurs at any stage of the study, the participant will be asked to complete an EOS evaluation. **In addition, participants are informed on the essential requirement to complete the antimalarial drug treatment for their safety, via the Participant Information Sheet.**

Participation in an EOS evaluation by each participant is voluntary. Procedures during early termination visit will include:

1. Perform medical history.
2. Perform complete physical examination.
3. Record vital signs.
4. Obtain a 12-lead ECG.
5. Collect urine sample for urinalysis.
6. Obtain blood for haematology, biochemistry, malaria qPCR, RBC alloantibodies and safety serum storage.

In a case of occurrence of SAEs, regardless of whether or not it is judged to be inoculum-or antimalarial drug-related, the participant will receive appropriate care under clinical supervision until all the symptoms of the SAEs have diminished or resolved and the participant's condition improved.

For ongoing AEs care will be provided for a period of time as specified in the clinical site work instruction protocols. However, if the nature of the ongoing AE is determined by the Principal Investigator as not being inoculum- or antimalarial drug-associated, the participant will be advised to visit his/her own general practitioner for further clinical care that he might require.

For participants who are lost to follow-up (i.e., those participants whose status is unclear because they fail to appear for study visits without stating an intention to withdraw), the Investigator should show 'due diligence' by documenting in the source documents steps taken to contact the participant, e.g., dates of telephone calls, registered letters, etc.

7.2 Study and Laboratory Procedures/Evaluations

Some safety and laboratory evaluation days may vary ±2 days based on qPCR counts and clinic visits at the discretion of the Investigator. Unscheduled qPCR and safety bloods may be required for safety monitoring.

7.2.1 Medical History

Medical history will be conducted at screening as described in Appendix 2. Information regarding any new medical conditions or illnesses will be elicited at Day 0 prior to malaria parasite inoculum, Day 28±3, and Day 90±14/EOS or early termination visit.

7.2.2 Physical Examination

Physical examination will be conducted as described in Appendix 2. Complete physical examination will be performed at screening and Day 28±3 or early termination visit.

Abbreviated physical examination will be performed at Day 0 prior to inoculum, on CRU admission prior to artesunate treatment, prior to exit of CRU confinement, and Day 90±14/EOS.

Abbreviated physical examination will be performed when signs or symptoms of malaria are identified if clinically indicated at the following time-points (as specified in Section 7.1): out-patient visits from Day 4 until CRU admission, during CRU confinement, and during out-patient safety monitoring post-artesunate treatment.

7.2.3 Vital Signs

Vital signs (temperature [sublingual], heart rate, respiratory rate and blood pressure) will be measured at screening after the participant has rested in the supine position for at least 5 minutes and in the standing position within 2-3 minutes when changing from the supine to standing position. At all other time-points, vital signs will be measured after the participant has rested in the seated position for at least 5 minutes.

Vital signs will be recorded at screening, Day 0 prior to inoculum, Day 0 prior to discharge (approximately 1 hour post-inoculum), out-patient visits from Day 4 until CRU admission, on CRU

admission prior to artesunate treatment, 3 times per day during confinement, prior to exit of confinement, at all out-patient safety monitoring visits post-artesunate treatment, Day 28±3, and Day 90±14/EOS or early termination visit.

7.2.4 Electrocardiogram (ECG)

A single 12-lead ECG will be recorded at the following time-points after resting supine for at least 5 minutes: screening, Day 0 prior to inoculum, on CRU admission prior to artesunate treatment, prior to Eurartesim® treatment, at the next scheduled visit after Eurartesim® treatment, and Day 28±3 or early termination visit.

7.2.5 Cannulation for Blood Sampling

Participants will be cannulated with an appropriate gauge intravenous cannula on Day 0 pre-inoculation and during the artesunate confinement period. Pre-dose blood samples will be collected before inoculation or artesunate treatment as outlined in this protocol. Q-Pharm's standard work instructions will apply to the allowed time windows. Blood will be collected into tubes containing the appropriate anti-coagulant. Samples will be processed according to the laboratory requirements.

7.2.6 Clinical Laboratory Evaluations

Any significant deviations from results obtained during screening will be followed until resolution or investigated fully, or until the participant is referred to a general practitioner. The Investigator will document the clinical significance of all results falling outside of the normal reference ranges. All abnormal laboratory tests results judged as being clinically significant will be recorded as adverse events.

7.2.6.1 Haematology and Biochemistry

Haematology and biochemistry evaluations will be conducted as described in Appendix 2. Blood will be collected at screening (baseline sample), Day -3 to Day -1 safety visit (if required), on CRU admission prior to artesunate treatment, prior to exit of confinement at ~72 hours post-artesunate treatment, Day 14±2, Day 18±2, prior to Eurartesim® treatment (if required), 3 days post-Eurartesim® treatment (or next scheduled visit), prior to Malarone® treatment, after completion of Malarone® treatment (or next scheduled visit), and Day 28±3 or early termination visit.

7.2.6.2 Urinalysis

Urinalysis will be conducted as described in Appendix 2. Urine will be collected at screening (baseline sample), Day -3 to Day -1 safety visit (if required), on CRU admission prior to artesunate treatment, Day 14±2, Day 18±2, and Day 28±3 or early termination visit. Urine will be tested by dipstick. If there are any abnormalities considered clinically significant in blood, leucocytes or protein, the urine will be sent for microscopy per the CRU (Q-Pharm) standard procedure.

7.2.6.3 RBC Alloantibodies

Blood for RBC alloantibodies testing will be collected at screening, Day 28±3, and Day 90±14 or early termination visit.

7.2.6.4 G6PD Testing

Blood for G6PD testing will be collected at screening only. G6PD deficiency is not an exclusion criterion but will be determined at screening to ensure the safety of Primacin™.

7.2.6.5 Serology

Serology will be conducted as described in Appendix 2. Blood will be collected at screening and Day 28±3.

7.2.6.6 Safety Serum Storage

Blood for serum storage as safety retention samples will be collected at Day 0 prior to inoculum (1 sample), Day 28±3 (1 sample), and Day 90±14 (2 samples) or early termination visit (2 samples).

7.2.7 Drug Screens and Alcohol Breath Tests

Urine drug screens will be conducted as described in Appendix 2. Urine will be collected at screening, Day 0 prior to inoculum, and on CRU admission prior to artesunate treatment.

Alcohol breath testing will be performed at the same time-points as urine drug screens. If the results of the tests are positive, participants may be allowed to continue, or may be delayed or withdrawn according to site-specific instructions.

All participants will be questioned about concomitant medications and use of recreational drugs. The urine screen may be repeated if the potential participant denies usage of any of these agents and the test result is believed to be a false positive.

Participants testing positive for paracetamol at screening and Day 0 may still be eligible for study participation, at the Investigator's discretion. Participants requiring paracetamol on a daily basis would not be eligible for study participation, as the use of any over-the-counter medication during the study is prohibited and potential participants should not discontinue their usual medications in order to participate in the study.

7.2.8 Malaria monitoring by qPCR

Blood will be collected for malaria 18S qPCR at the following time-points:

- Day 0 prior to inoculum (baseline sample).
- Daily from Day 4 (morning) until qPCR positive.
- When qPCR positive, twice daily (morning and evening) until dosing with artesunate.
- During admission/confinement: prior to artesunate treatment, and at approximately 2, 4, 6, 8, 12, 16, 20, 24, 28, 32, 36, 48, 60 and 72 hours (exit from unit) after treatment.
- During out-patient safety monitoring post-artesunate treatment. A blood sample will be collected for malaria qPCR at approximately 84 hours after artesunate treatment. Participants may then return morning and evening until parasite counts are <~500 parasites/mL which is when sampling may revert to daily visits at the Investigator's discretion. When parasite counts are <~200 parasites/mL sampling may revert to alternate

day visits at the Investigator's discretion. Once the qPCR results are negative, or low and stable, the participants may be reviewed 3 times per week until Malarone® treatment.

- Prior to Eurartesim® treatment (if required) and 3 days post-Eurartesim® treatment (or next scheduled visit).
- Follow-up visit at Day 28±3.

Additional blood (up to approximately 2.5 mL per time-point) may be collected for gametocyte *pfs25* qRT-PCR, and/or male gametocyte marker, and/or ring-stage marker as appropriate, at times indicated by 18S qPCR at the Investigator's discretion from the day of artesunate treatment and at Day 28±3. At up to four time-points, additional blood may be collected in two different sample tube types (up to approximately 4.5 mL total blood) for comparison of RNA extraction efficiency to improve the samples for qRT-PCR.

Microscopic examination for evidence of parasitaemia or gametocytaemia may be conducted at the Investigator's discretion. Thick films may be prepared from blood collected at time-points coinciding with qPCR sampling. Unscheduled qPCR testing may be required based on qPCR counts.

7.2.9 Pharmacokinetic assays

The participant's plasma concentrations of artesunate and dihydroartemisinin will be determined using liquid chromatography-tandem mass spectrometry (LC-MS/MS) as previously described [55] with modification (i.e. substituting a protein precipitation extraction for solid phase extraction). Blood will be collected for PK assays prior to artesunate treatment (baseline sample) and following treatment at approximately 0.25, 0.5, 1, 1.5, 2, 2.5, 3, 4, 6, 8, 10, and 12 hours.

7.2.10 General

If the observation time and blood sampling time coincide, for precision of timing, blood collection will take precedence over other procedures scheduled at the same time. With regard to time windows allowance for study procedures, Q-Pharm's standard work instructions will apply.

Participants may be quietly ambulant within the unit.

The Investigator, Co-Investigator(s), infectious disease clinician, or experienced nurse will monitor the participants during the confinement period in the morning and evening and at the out-patients visit on Day 0, study days following discharge and the follow-up/EOS visit.

Participants will be under observation and AEs (if any) will be recorded and dealt with appropriately.

At the post-confinement visits, participants will again be given the opportunity to mention any problems, and will be asked non-leading questions regarding their general well-being and medication intake.

7.2.11 Specimen Preparation, Handling, and Storage

Biological samples will be retained for the time required for assessment for analysis, and may then be discarded. Safety serum samples are stored indefinitely with the permission of the participants for any retrospective safety assessments.

7.2.12 Specimen Shipment

Samples collected will be shipped to nominated local or international laboratories for assessment. The site staff will be responsible for shipment of samples to analytical laboratories for testing. Samples must be packed securely together with completed shipment forms in shipping containers together with sufficient dry ice as per Shipper procedures.

7.2.13 Meals and Fluid Restrictions

- Participants must abstain from all food and drink (except water) for ≥8 hours prior to screening safety laboratory assessments.
- On Day 0, malaria inoculum day, participants may have food until at least half an hour prior to inoculation.
- On the admission of the participants into the CRU for artesunate treatment or as advised by the clinical staff, participants should come fasting (≥8 hours). If dosing is to occur in the evening, participants will be required to fast for ≥4 hours prior to admission. Participants should not eat food until 1 hour after artesunate treatment. Standard meals will be supplied whilst in the clinic unit during confinement. The clinic staff will ensure that participants maintain their fluid intake throughout the period of confinement. Participants may drink water and non-alcoholic, non-xanthine containing beverages as desired.
- It is recommended that Eurartesim® is administered after a ≥3 hour fast. Participants will be requested to fast for a further 3 hours after Eurartesim® treatment.
- Participants should not consume grapefruit or Seville oranges from Day 0 until the end of the Malarone® treatment.
- Participants should not consume quinine containing foods/beverages such as tonic water, lemon bitter, from Day 0 until the end of the Malarone® treatment.
- Participants should not eat any poppy seeds in the 24 hours before the following time-points: screening, malaria inoculum day (Day 0), and day of admission for artesunate treatment.
- Participants should not eat or drink any food or beverages that contain alcohol (e.g. beer, wine, and mixed drinks) from 24 hours before the malaria inoculum (Day -1) until the end of the Malarone® treatment.

7.3 Concomitant Medications

On inoculation day, participants will be questioned in relation to relevant aspects of compliance with the study protocol, including drug intake since their screening clinic visit. Details of all other drugs taken (prescription and over-the-counter, systemic and topical administration) will be recorded at this time and appropriate action taken. The Investigator may permit the use of ibuprofen (preferred) up to 1.2 g/day or paracetamol up to 4 g/day, for treatment of headache or other pain if required. Any medication taken during the study for treatment of a medical condition or adverse event is to be recorded in the concomitant medication pages in the CRF.

8.0 ASSESSMENT OF SAFETY

8.1 Specification of Safety Parameters

8.1.1 Adverse Events (AEs)

It is the responsibility of the Principal Investigator to ensure that AEs, which occur in the context of the study, are reported and documented. Expected AEs from the malaria parasite inoculum are listed in Appendix 4. Expected AEs from the antimalarial drugs used are listed in the Eurartesim® product information, artesunate WHO Public Assessment Report 2011, Malarone® Consumer Medicine Information, and Primacin™ Consumer Medicine Information (see Appendix 5). All observed events will be recorded and reported as described in this protocol.

In addition to determining whether an AE fulfils criteria for a serious adverse event or not, the severity of AEs experienced by study participants will be graded according to a set of criteria developed for guidance of commonly reported symptoms, signs and abnormal laboratory findings in malaria challenge studies. These were adapted from the Common Terminology Criteria for Adverse Events v4.0 published May 28, 2009 (CTCAE v4.0). This guidance provides a common language to describe levels of severity, to analyse and interpret data, to scale the aggregate AE score, and to articulate the clinical significance of all AEs.

Adverse events will be captured during observation and examination of each participant through the course of the study and recorded using study source documents.

8.1.2 Definition of Adverse Events

An AE is any adverse change, i.e., any unfavourable and unintended sign, including an abnormal laboratory finding, symptom or disease that occurs in a participant during the course of the study, whether or not considered by the Investigator as related to study treatment. For guidance for assigning severity of the malaria, the purpose-designed Clinical Score for Malaria AE scale will be used (see Appendix 6).

Adverse events include:

- Exacerbation of a pre-existing disease.
- Increase in frequency or intensity of a pre-existing episodic disease or medical condition.
- Disease or medical condition detected or diagnosed during the course of the study even though it may have been present prior to the start of the study.
- Continuous persistent disease or symptoms present at study start that worsen following the start of the study.
- Abnormal assessments, e.g., change on physical examination, ECG findings, if they represent a clinically significant finding that was not present at study start or worsened during the course of the study.
- Laboratory test abnormalities if they represent a clinically significant finding, symptomatic or not, which was not present at study start or worsened during the course of the study or led to dose reduction, interruption or permanent discontinuation of study treatment.

Overdose, misuse, and abuse of the study treatment should be reported as an AE and, in addition, study treatment errors must be documented in the study drug log of the CRF.

All AEs occurring after study enrolment and up to EOS must be recorded on specific AE pages of the CRF.

All malaria-specific AEs will be tabulated according to a purpose-designed table, and results graded according to the score sheet designed for this purpose.

8.1.3 Definition of Serious Adverse Events

A SAE is defined by the International Conference on Harmonisation (ICH) guidelines as any AE fulfilling at least one of the following criteria:

- Fatal.
- Life-threatening, referring to an event in which the participant was at risk of death at the time of the event. It does not refer to an event that hypothetically might have caused death had it been more severe.
- Requiring inpatient hospitalisation, or prolongation of existing hospitalisation.
- Resulting in persistent or significant disability or incapacity.
- Congenital anomaly or birth defect.
- Medically significant: refers to important medical events that may not immediately result in death, be life-threatening, or require hospitalisation but may be considered to be SAEs when, based upon appropriate medical judgment, they may jeopardise the participant, and may require medical or surgical intervention to prevent one of the outcomes listed in the definitions above.
- Constitutes a possible Hy's Law case defined as a participant with any value of alanine (ALT) or aspartate (AST) aminotransferase greater than 3 × ULN (upper limit of normal) together with an increase in total bilirubin to a value greater than 2 × ULN and not associated to an alkaline phosphatase (ALP) value greater than 2 × ULN (FDA Guidance on Drug Induced Liver Injury: Premarketing Clinical Evaluation [2009]).

The following reasons for hospitalisation are exempted from being reported:

- Hospitalisation for cosmetic elective surgery, or social and/or convenience reasons.
- Hospitalisation for pre-planned (i.e., planned prior to signing informed consent) surgery or standard monitoring of a pre-existing disease or medical condition that did not worsen, e.g., hospitalisation for coronary angiography in a participant with stable angina pectoris.

However, complications that occur during hospitalisation are AEs or SAEs (for example if a complication prolongs hospitalisation).

A **Suspected Unexpected Serious Adverse Reactions (SUSAR)** is any SAE where a causal relationship with the investigational product (K13 challenge inoculum) is at least a reasonable possibility, but the event is not listed in the Investigator Brochure(s) and/or Summary of Product Characteristics.

8.1.4 Adverse Events Associated with the Study Design or Protocol-Mandated Procedures

An AE is defined as related to study design or protocol-mandated procedures if it appears to have a reasonable possibility of a causal relationship to either the study design or to protocol-mandated procedures. Appendix 4 contains a list of events that are considered to be symptoms and signs of malaria. Their occurrence will be monitored, carefully considered, and discussed between the Investigator, drug safety physician, and the IMM, especially with regards to the need for administration of the rescue treatment in the circumstances of suboptimal response.

8.1.5 Definition of Unexpected Events

An adverse event is regarded as an **unexpected event** if its nature or severity is not consistent with the applicable reference safety information (Investigator's Brochure for the malaria parasite inoculum, or approved manufacturer's prescribing information for marketed drugs). Events that add significant information on the specificity, severity or frequency of previously described reactions, are also regarded as unexpected.

8.2 Classification of an Adverse Event

8.2.1 Severity of Event

The medical assessment of AE severity of the study will be recorded in accordance with the Common Terminology Criteria for Adverse Events v4.0 published May 28, 2009 (CTCAE v4.0).

The severity of adverse events will be graded as follows:

Grade 1: Mild; asymptomatic or mild symptoms; clinical or diagnostic observations only; intervention not indicated.

Grade 2: Moderate; minimal, local or non-invasive intervention indicated; limiting age-appropriate instrumental activities of daily living.

Grade 3: Severe or medically significant but not immediately life-threatening; hospitalisation or prolongation of hospitalisation indicated; disabling; limiting self-care activities of daily living.

Grade 4: Life-threatening consequences; urgent intervention indicated.

Grade 5: Death related to AE.

A mild, moderate, or severe AE may or may not be serious (see Section 8.1.3). These terms are used to describe the intensity of a specific event. Medical judgment should be used on a case-by-case basis.

Seriousness, rather than severity assessment, determines the regulatory reporting obligations.

8.2.2 Relationship to Study Agent

The Investigator or Co-Investigators will decide if AEs are related to the malaria parasite inoculum or the treatment drug (artesunate) or other antimalarial drugs administered. The assessment of causality will be made using the following definitions:

Unrelated

This category is applicable to those AEs which are judged to be clearly and incontrovertibly due to extraneous causes (disease, environment, etc.) and do not meet the criteria for the relationship listed under unlikely, possible or probable.

Unlikely

In general, this category is applicable to an AE which meets the following criteria (must have the first two):

1. It does **not** follow a reasonable temporal sequence from administration of any of the study agents.
2. It may readily have been produced by the participant's clinical state, environment or toxic factors, or other modes of therapy administered to the participant.
3. It does not follow a known pattern of response to the study agents.
4. It does not reappear or worsen when any of the study agents are re-administered.

Possible

This category applies to those AEs in which the connection with any of the study agents appears unlikely but cannot be ruled out with certainty. An adverse event may be considered possible if or when (must have the first two):

1. It follows a reasonable temporal sequence from administration of any of the study agents.
2. It may have been produced by the participant's clinical state, environment or toxic factors, or other modes of therapy administered to the participant.
3. It follows a known pattern of response to any of the study agents.

Probable

This category applies to those adverse events which are considered, with a high degree of certainty, to be related to the study agents. An adverse event may be considered probable if (must have the first three):

1. It follows a reasonable temporal sequence from administration of any of the study agents.
2. It cannot be reasonably explained by the known characteristics of the participant's clinical state, environment or toxic factors, or other modes of therapy administered to the participant.
3. It disappears or decreases on cessation or reduction in dose.
4. It follows a known pattern of response to any of the study agents.
5. It reappears on re-administration.

8.3 Treatment and Follow-up of Adverse Events

All AEs will be documented in the participant's CRF, and will be categorised according to their causality and severity and whether they are defined as a serious or non-serious adverse event.

Information that needs to be collected for each AE includes:

- Description of AE.
- Dates of onset and resolution of the event.
- Seriousness of the AE (SAE or not).
- Severity of AE (grade 1-5).

- Action taken in response to the event (including treatment required).
- The outcome of the event.
- The relationship of the event to any study procedure or treatment (causality assessment).

Changes in the severity of an AE will be documented to allow assessment of the duration of the event at each level of severity. AE characterised as intermittent require documentation of onset and duration at each episode. All AEs will be followed until they are either resolved or adequately explained.

8.4 Reporting Procedures

8.4.1 Serious Adverse Event Reporting

Review and reporting of SAEs will be in accordance with the Sponsor's and Q-Pharm's SAE reporting procedures. All SAE Reports will be sent to QIMR Berghofer Regulatory Affairs, QIMR Berghofer-HREC, and the IMM.

Within 24 hours of the Investigator or Co-investigators becoming aware of an SAE they should:

- Notify the Sponsor Representative and the Independent Medical Monitor of the SAE occurrence.
- Complete the initial SAE report by completing the details on the ICH or QIMR Berghofer SAE Report form.
- Fax and/or email the completed initial SAE report to the:

QIMR Berghofer Regulatory Office

clinical.trials@qimrberghofer.edu.au

- The SAE report should be submitted to the QIMR Berghofer HREC:

Within 14 days of onset of the SAE:

- The Principal Investigator will complete the follow-up SAE report by filling in SAE follow-up form.
- The original signed copy should be mailed to:

QIMR Berghofer Regulatory Office

Other supporting documents of the event may be requested by the Sponsor Representative or the IMM and will be provided by the Investigator or a delegate as soon as possible.

Summary reports for the occurrence and the follow-up of all SAEs observed will be sent to the IMM for review. Once the reports have been reviewed by the IMM, copies from the reports will be faxed back to the clinical site to be filed with the source documents and the medical records.

The Investigator will report all SAEs and unanticipated problems involving risks to participants to the Sponsor by email as soon as possible, but in no event later than one (1) business day of learning of such an incident or event.

8.5 Emergency Procedures

Emergency procedures are in place at the Q-Pharm clinics for dealing with any unforeseen clinical emergencies that may arise. The Investigator and/or an experienced nurse will be present at all times when participants are at the CRU.

8.6 Safety Oversight

Clinical Safety Oversight will be undertaken by the Principal Investigator and an IMM who will serve as an independent expert to advise on clinical safety specifically in the situation where expert external advice is required regarding the need for administration of alternative/rescue antimalarial treatment in the circumstance of suboptimal response.

Inoculation of the second participant will occur at least 2 weeks following inoculation of the first participant. A review of data from the first participant will be conducted by the SRT prior to inoculation of the second participant. Safety, tolerability and parasitaemia data for a minimum of 14 days will be required for the review.

The role of the SRT is defined in the study specific SRT Charter. The SRT will be composed of the Principal Investigator, IMM, and a physician with expertise in clinical trials or infectious diseases. The SRT will review the clinical and laboratory safety data package as well as the recorded AEs and SAEs. The SRT makes recommendations to the Sponsor. These recommendations are approved by the SRT Chair who signs a letter of recommendation that is sent to the Principal Investigator and the Sponsor.

Additionally, the SRT will meet to assess any events that trigger the stopping rules or as needed to provide a recommendation and findings to QIMRB-HREC and the Principal Investigator, in accordance to the approved SRT Charter.

Whether at a scheduled or unscheduled meeting, the SRT will consider safety signals to determine whether or not they can recommend that the study continue.

9.0 STATISTICAL CONSIDERATIONS

9.1 General Approach

This is a pilot study and no formal statistical analysis is planned. All measured variables and derived values will be listed, including data from all participants who meet the eligibility criteria and are enrolled in the study.

9.2 Sample Size Calculations

This study is a pilot safety and infectivity study. Therefore, statistical considerations regarding sample size do not apply. Pilot safety and infectivity studies of 5 parasite banks have been successfully undertaken at QIMR Berghofer, 3 *P. falciparum* [5-10, 15-17, 20, 22-30, 32-34, 36, 38, 41, 42, 56] and 2 *P. vivax* banks [11, 12, 30, 57]. In these studies the study size to gain pilot safety and infectivity data was 2 participants, thus informing determination of cohort size for the proposed study.

9.3 Demographic and Clinical Laboratory Data

Demographic data will be summarised by descriptive statistics and will include total number of observations (n), mean, standard deviation (SD) and range for continuous variables and number and percentages with characteristics for dichotomous variables.

Clinical laboratory data (haematology, biochemistry, and urinalysis) which is outside of the normal range will be listed in tables. Isolated laboratory abnormalities will be reported as AEs if they are considered to be clinically significant by the Investigator. Vital signs which are outside of the normal range and clinically significant will also be listed in tables. All AEs will be listed by participant and will include details of the treatment received prior to onset, onset time, duration, severity and relationship to the study agent.

9.4 Analysis of Safety Data

Separate assessments of systemic and local reactions will be performed. The overall number and percentage of participants with at least one AE (and SAE) will be tabulated over the entire study period. Any clinically important deviations from normal that occur in routine laboratory test results and/or vital signs as determined by the Investigator will be reported as AEs. Should the need arise for early termination of the study, the investigative team will discuss with the SRT the reason for termination.

9.5 Data Management

Clinical and laboratory data will be managed according to the standard procedures of Q-Pharm, supplemented if required by any specific requirements of the Sponsor.

10.0 ETHICAL/PROTECTION OF PARTICIPANTS

10.1 Ethical Principles

The study will be conducted in accordance with the protocol approved by QIMR Berghofer HREC, the principles of the Declaration of Helsinki (Recommendations guiding Medical Doctors in Biomedical Research Involving Human Participants), and with the NH&MRC National Statement on Ethical Conduct in Human Research (2007) [58]. The conduct of the study will be in accordance with the Notes for Guidance on Good Clinical Practice (GCP) (CPMP/ICH/135/95), as adopted by the Australian Therapeutic Goods Administration (2000) [59]. The Principal Investigator will take care to minimise any discomfort experienced by participants during these studies. The only invasive procedures will be the intravenous inoculation of the malaria parasite inoculum and the blood collection by cannulation/venepuncture. The maximum amount of blood to be collected from an individual in the study would be approximately 298 mL for participants (i.e. a volume which will not be more than the equivalent to a standard blood-bank donation and it will be taken over at least a 13 week interval). The total volume of blood drawn from each participant will not exceed 450 mL in any given 30-day period. This volume includes allowance for unscheduled safety laboratory assessments that may be required at the discretion of the Principal Investigator or the Sponsor to ensure participant safety.

10.2 Ethical Review

The protocol, consent forms and participant information sheets will be reviewed by the QIMR Berghofer HREC.

No study activities will be initiated prior to the approval of that Committee. All amendments and addenda to the protocol will similarly be submitted to the QIMR Berghofer HREC.

10.3 Informed Consent Process

10.3.1 Consent Procedures and Documentation

Participants will be fully informed of the nature of the study, the properties and side effects of the investigational products, and all relevant aspects of study procedures in the 'Participant Information Sheet'. Participants will receive a copy of the 'Participant Information Sheet', the product insert for artesunate, and the Consumer Medicines Information for Malarone® (Appendix 5). The nature of the study, the study agents, and their side effects will also be discussed with the participants by the Investigator during recruitment. Participants may ask questions of the Investigator or the clinic staff at any time. Participants will also receive any of the Consumer Medicine Information for any other registered antimalarial agents in the event that these would be required.

The 'Informed Consent Form' will be signed and dated by the participants in the presence of an Investigator. Participants will also be given a copy of their signed 'Informed Consent Form'.

10.3.2 Participant Compensation

Participants who complete the study up to Day 90±14/EOS will be paid $3100 compensation for their participation. Participants who withdraw or are withdrawn from the study will be compensated on a fractional basis for their involvement unless they are withdrawn as a consequence of their

misconduct. Reserve participants who do not participate in the study will be paid $150 compensation for the inconvenience associated with their attendance for screening and for their attendance on the inoculation day, in case they are required to participate.

10.4 Participant and Data Confidentiality

Participants will be informed that their data are held on file by Q-Pharm, and that these data may be viewed by staff of Q-Pharm (including, where necessary, staff of Q-Pharm other than the named investigators). Participants will be informed that samples collected for the purposes described in the protocol will be sent to Sponsor's nominated national or international laboratory for assessment. Samples will be stored for the time required to complete the assessment and then destroyed. Safety serum samples are collected and stored indefinitely at Q-Pharm/QIMR Berghofer.

Upon request, the investigator(s)/institution(s) will permit direct access to source data/documents for trial-related monitoring, audits, IRB/IEC review, and regulatory inspection(s) by the Sponsor (or their appropriately qualified delegate) and Regulatory Authorities. Direct access includes examination, analysis, verification and reproduction of records and reports that are important to the evaluation of the trial.

They will similarly be informed that a report of the study will be submitted to the Sponsor and may also be submitted to government agencies and perhaps for publication, but that they will only be identified in such reports by their study identification number, initials and perhaps their gender and age. The investigators undertake to hold all personal information in confidence.

10.5 Future Use of Stored Specimens

Consent must be obtained from the participants in this study to maintain their specimens for further use. All samples will be stored at QIMRB in accordance with the laboratory SOPs. The investigators will ensure that the confidentiality will be maintained continuously in all further studies that involve use of these specimens. The vials containing the specimens of the consented participants will be letter coded and the identifying information will not be released to any unauthorised third party. No genetic testing will be performed on these specimens.

There are no benefits to participants in the collection, storage and subsequent research use of their specimens. Reports about future research done with participant samples will NOT be kept in their health records, but participant's samples may be kept with the study records or in other secure areas. Participants can decide if they want their samples to be used for future research or have their samples destroyed at the EOS. A participant's decision can be changed at any time prior to the EOS by notifying the study doctors or nurses in writing. However, if a participant consents to future use and some of their blood has already been used for research purposes, the information from that research may still be used.

11.0 STUDY ADMINISTRATION

11.1 Liability/Indemnity/Insurance

The study Sponsor will ensure sufficient insurance is available to enable it to indemnify and hold the investigator(s) and relevant staff as well as any hospital, institution, ethics committee or the like, harmless from any claims for damages for unexpected injuries, including death, that may be caused by the participant's participation in the study but only to the extent that the claim is not caused by the fault or negligence of the participants or investigator(s). The Sponsor adheres to the guidelines of Medicines Australia for injury resulting from participation in a company sponsored trial, including the provision of 'No-fault clinical trial insurance'.

11.2 Protocol Amendments

Changes to the final study protocol can only be made with the prior consent of the Principal Investigator, the Sponsor and the Ethics Committee. All such changes must be attached to, or incorporated into, the final protocol, and communicated to all relevant members of Q-Pharm staff and, if appropriate, to trial participants. All deviations from this study protocol will be included in the trial master file and included in the final study report. An assessment of the significance of each protocol deviation will be given in the study report. All deviations/amendments will be reported to Sponsor.

11.2.1 Non-substantial amendment

Administrative or logistical minor changes require a non-substantial amendment. Such changes include but are not limited to changes in study staff or contact details (e.g., Sponsor instead of CRO monitors) or minor changes in the packaging or labelling of study drug. An amendment deemed to be non-substantial must have no ethical implications.

The implementation of a non-substantial amendment may be done without notification to the HREC. It does not require their approval or to be signed by the Investigator. The HREC will be notified for these non-substantial changes in the next submission round, with the annual study report or study close out report which ever comes sooner that will be submitted to HREC.

11.2.2 Substantial amendment

Significant changes require a substantial amendment. Significant changes include but are not limited to: new data affecting the safety of participants, change of the objectives/endpoints of the study, eligibility criteria, dose regimen, study assessments/procedures, treatment or study duration, with or without the need to modify the Participant Information Sheet and Informed Consent.

Substantial amendments are to be approved by the HREC. The implementation of a substantial amendment can only occur after formal approval by the HREC and must be signed by the Investigator.

11.2.3 Urgent amendment

An urgent amendment might become necessary to preserve the safety of the participants included in the study. The requirements for approval should in no way prevent any immediate action being

taken by the investigators or the Sponsor in the best interests of the participants. Therefore, if deemed necessary, an investigator can implement an immediate change to the protocol for safety reasons. This means that, exceptionally, the implementation of urgent amendments will occur before submission to and approval by the HREC.

In such cases, the Investigator must notify the Sponsor within 24 hours. A related substantial amendment will be written within 10 working days and submitted to the HREC, together with a description of the steps that have already been taken in regard to implementation of this amendment.

11.3 Clinical Data Recording

Each participant will have a Clinical File (source data) and a Case Report Form (CRF, for protocol specific data) into which relevant data will be recorded.

All recording will be done only in black ink.

Corrections will only be made by drawing a single line through the incorrect entry, writing the correction in the nearest practicable space and initialling and dating the correction. A log of names, signatures and initials of all staff entering data into a participant's Clinic File and CRF will be kept. Any corrections made after the review and signature of the Principal Investigator will be noted with the initials of the person making the change and countersigned by the Principal Investigator. Correction fluids are not allowed.

All deviations from this study protocol will be included in the Trial Master File and included in the final study report. An assessment of the significance of each protocol deviation will be given in the study report.

All CRFs will be reviewed internally by the CRU at the completion of each study visit for any omissions or apparent errors so that these can be corrected without delay.

11.4 Record Retention

All source data, clinical records and laboratory data relating to the study will be retained in the archive of the CRU (Q-Pharm) for a minimum of 15 years after the completion of the study. All data will be available for retrospective review or audit by arrangement with the Chief Executive Officer of the CRU (Q-Pharm). Written agreement from the Sponsor must precede destruction of the same.

11.5 Monitoring

It will be the Sponsor's responsibility to ensure that the study is monitored in accordance with the requirements of GCP. The conduct of the study will be reviewed internally by the CRU (Q-Pharm) in accordance with the CRU's (Q-Pharm) standard procedures and work instructions and GCP guidelines. The trial will be monitored according to the Sponsor's SOPs [60] and all protocol deviations that impact the safety of the participant or integrity of the data shall be reported to the Sponsor and QIMR Berghofer HREC.

11.6 Reporting and Communication of Results

The QIMR Berghofer team will provide a clinical study report at the conclusion of the study with all tables and listings as appendices if required.

Publication and reporting of results and outcomes of this trial will be accurate and honest, undertaken with integrity and transparency and in accordance with the relevant clauses outlined in the QIMR Berghofer Policy on Criteria for Authorship [61]. QIMR Berghofer and the Principal Investigator have a responsibility to ensure that results of scientific interest arising from the clinical trials are appropriately published and disseminated. Publication of results will be subjected to fair peer-review. Authorship will be given to all persons providing significant input into the conception, design, and execution or reporting of the research according to QIMR Berghofer Policy on the Criteria of Authorship. No person who is an author, consistent with this definition, will be excluded as an author without his/her permission in writing. Authorship will be discussed between researchers prior to study commencement (or as soon as possible thereafter) and reviewed whenever there are changes in participation. Acknowledgment will be given to collaborating institutions and hospitals and other individuals and organisations providing finance or facilities. All conflicts arising through disputes about authorship will be reviewed by the QIMR Berghofer Director.

In any press releases, publications or presentations, MMV's financial contribution to the Project and its participation in the collaboration shall be expressly acknowledged. QIMR Berghofer agrees that MMV will be entitled to access all the de-identified Clinical Trial data upon completion of the Clinical Trial. Data will not be released publicly until the manuscript is accepted for publication. In the case of no publication, information will only be released to the public and media in accordance with QIMR's Corporate Media Strategy Policy [62]. However, the Investigator undertakes not to make any publication or release pertaining to the study and/or results of the study without the Sponsor's prior written consent, being understood that the Sponsor will not unreasonably withhold its approval. The Sponsor has the right at any time to publish the results of the study. Patient confidentiality will be maintained by referring to individual participants by their identifying code used in the trial.

The Investigator shall not use the name(s) of the Sponsor and/or of its employees in advertising or promotional material or publication without the prior written consent of the Sponsor. The Sponsor shall not use the name(s) of the Investigator and/or the collaborators in advertising or promotional material or publication without having received his/her and/or their prior written consent(s).

QIMR Berghofer will ensure that the key design elements of this protocol are posted in a publicly accessible database such as ANZCTR or Clinicaltrials.gov. In addition, upon study completion and finalisation of the study report the results of this trial will be either submitted for publication in an open access journal and/or posted in a publicly accessible database of clinical trial results, such as ANZCTR or ClinicalTrials.gov.

11.7 Study Audit

Audits may be carried out by Sponsor quality assurance, local authorities or authorities to whom information on this study has been submitted. All documents pertinent to this study must be made available for such inspection after adequate notice of intention to audit.

12.0 REFERENCES

1. World Health Organization (WHO). World Malaria Report 2015. http://www.who.int/malaria/publications/world-malaria-report-2015/report/en/, accessed 11 October 2016: World Health Organization, **2015**.

2. Ashley EA, White NJ. Artemisinin-based combinations. Curr Opin Infect Dis **2005**; 18:531-6.

3. Dondorp AM, Nosten F, Yi P, et al. Artemisinin resistance in *Plasmodium falciparum* malaria. The New England journal of medicine **2009**; 361:455-67.

4. Woodrow CJ, White NJ. The clinical impact of artemisinin resistance in Southeast Asia and the potential for future spread. FEMS Microbiol Rev **2016**.

5. ANZCTR Trial ID: ACTRN12611001203943. An experimental study to characterize molecular signatures during early Plasmodium falciparum blood stage infection in healthy male volunteers., **2011**.

6. ANZCTR Trial ID: ACTRN12612000323820. An experimental study to characterize the effectiveness of Lariam(Registered Trademark) (Mefloquine) against early Plasmodium falciparum blood stage infection in healthy volunteers **2012**.

7. ANZCTR Trial ID: ACTRN12612000814875. An experimental study to characterize the effectiveness of OZ439 against early Plasmodium falciparum blood stage infection in healthy volunteers, **2012**.

8. ANZCTR Trial ID: ACTRN12613000565741. An experimental study to characterize the effectiveness of Piperaquine against early Plasmodium falciparum blood stage infection in healthy volunteers. **2013**.

9. ANZCTR Trial ID: ACTRN12613000698774. An experimental study to characterize the effectiveness of griseofulvin against early plasmodium falciparum blood stage infection in healthy volunteers. **2013**.

10. ANZCTR Trial ID: ACTRN12613001040752. An experimental study to characterize the effectiveness of ferroquine against early Plasmodium falciparum blood stage infection in healthy volunteers. **2013**.

11. ANZCTR Trial ID: ACTRN12614000930684. Blood stage challenge pilot study to assess the safety and the infectivity of Plasmodium vivax isolate HMPBS02-Pv in healthy volunteers. **2014**.

12. Griffin P, Pasay C, Elliott S, et al. Safety and Reproducibility of a Clinical Trial System Using Induced Blood Stage Plasmodium vivax Infection and Its Potential as a Model to Evaluate Malaria Transmission. PLoS neglected tropical diseases **2016**; 10:e0005139.

13. Kamau E, Alemayehu S, Feghali KC, et al. Measurement of parasitological data by quantitative real-time PCR from controlled human malaria infection trials at the Walter Reed Army Institute of Research. Malaria Journal **2014**; 13:288-.

14. McCall MB, Netea MG, Hermsen CC, et al. Plasmodium falciparum infection causes proinflammatory priming of human TLR responses. Journal of immunology (Baltimore, Md : 1950) **2007**; 179:162-71.

15. McCarthy JS, Sekuloski S, Griffin PM, et al. A pilot randomised trial of induced blood-stage Plasmodium falciparum infections in healthy volunteers for testing efficacy of new antimalarial drugs. PLoS One **2011**; 6:e21914.

16. Pombo DJ, Lawrence G, Hirunpetcharat C, et al. Immunity to malaria after administration of ultra-low doses of red cells infected with Plasmodium falciparum. Lancet **2002**; 360:610-7.

17. Sanderson F, Andrews L, Douglas AD, Hunt-Cooke A, Bejon P, Hill AV. Blood-stage challenge for malaria vaccine efficacy trials: a pilot study with discussion of safety and potential value. The American journal of tropical medicine and hygiene **2008**; 78:878-83.

18. Sauerwein RW, Roestenberg M, Moorthy VS. Experimental human challenge infections can accelerate clinical malaria vaccine development. Nature reviews Immunology **2011**; 11:57-64.

19. Rockett RJ, Tozer SJ, Peatey C, et al. A real-time, quantitative PCR method using hydrolysis probes for the monitoring of Plasmodium falciparum load in experimentally infected human volunteers. Malaria Journal **2011**; 10:48-.

20. ANZCTR Trial ID: ACTRN12613000533796, . A Phase I/Ib Study to Investigate the Safety, Tolerability and Pharmacokinetic Profile of DSM265 in Healthy Subjects and to Assess the Antimalarial Activity of DSM265 in Healthy Subjects with an Induced Blood Stage Plasmodium falciparum Infection. **2013**.

21. ANZCTR Trial ID: ACTRN12613001008718. A Pilot Study To Assess Mosquito Transmissibility of Plasmodium vivax In Participants Inoculated intravenously with the parasite Isolate Hmpbs-Pv. **2013**.

22. ANZCTR Trial ID: ACTRN12614000781640, . A proof-of-concept study to assess the effect of ACT-451840 against early Plasmodium falciparum blood stage infection in healthy subjects. **2014**.

23. Bijker EM, Bastiaens GJ, Teirlinck AC, et al. Protection against malaria after immunization by chloroquine prophylaxis and sporozoites is mediated by preerythrocytic immunity. Proceedings of the National Academy of Sciences of the United States of America **2013**; 110:7862-7.

24. Cheng Q, Lawrence G, Reed C, et al. Measurement of *Plasmodium falciparum* growth rates *in vivo*: a test of malaria vaccines. American Journal of Tropical Medicine and Hygiene **1997**; 57:495–500.

25. ClinicalTrials.gov ID: NCT02281344. A Proof-of-concept Study to Assess the Effect of MMV390048 Against Early Plasmodium Falciparum Blood Stage Infection in Healthy Participants, **2014**.

26. ClinicalTrials.gov ID: NCT02389348. A Proof-of-concept Study to Assess the Effect of a Range of Doses of Combined Therapy With OZ439 and DSM265 Against Early Plasmodium Falciparum Blood Stage Infection in Healthy Participants, **2015**.

27. ClinicalTrials.gov ID: NCT02431637. Blood Stage Challenge Study to Asses Mosquito Transmissibility in Participants Inoculated With Plasmodium Falciparum. **2015**.

28. ClinicalTrials.gov ID: NCT02431650. A Proof-Of-Concept Study to Assess the Effectiveness of OZ439 as a Gametocytocidal and Transmission Blocking Agent in Experimental P. Falciparum Infection, **2015**.

29. ClinicalTrials.gov ID: NCT02543086. A Phase1 Interventional Sequential Single Site Study to Characterize the Effectiveness of Oral KAE609 in Reducing Asexual &Sexual Blood-stage P. Falciparum Following Inoculation in Healthy Volunteers & Subsequent Infectivity to Mosquitoes, **2015**.

30. ClinicalTrials.gov ID: NCT02573857. A Phase Ib Study to Characterise the Antimalarial and Transmission Blocking Activity of a Single Dose of DSM265 or OZ439 in Healthy Subjects With Induced Blood Stage Plasmodium Falciparum or Plasmodium Vivax Infection, **2015**.

31. Duncan CJA, Sheehy SH, Ewer KJ, et al. Impact on Malaria Parasite Multiplication Rates in Infected Volunteers of the Protein-in-Adjuvant Vaccine AMA1-C1/Alhydrogel+CPG 7909. PLoS One **2011**; 6.

32. Krause A, Dingemanse J, Mathis A, Marquart L, Mohrle JJ, McCarthy JS. Pharmacokinetic/pharmacodynamic modelling of the antimalarial effect of Actelion-451840 in an

induced blood stage malaria study in healthy subjects. British journal of clinical pharmacology **2016**; 82:412-21.

33. Lawrence G, Cheng QQ, Reed C, et al. Effect of vaccination with 3 recombinant asexual-stage malaria antigens on initial growth rates of *Plasmodium falciparum* in non-immune volunteers. Vaccine **2000**; 18:1925-31.

34. McCarthy JS, Baker M, O'Rourke P, et al. Efficacy of OZ439 (artefenomel) against early Plasmodium falciparum blood-stage malaria infection in healthy volunteers. The Journal of antimicrobial chemotherapy **2016**; 71:2620-7.

35. McCarthy JS, Griffin PM, Sekuloski S, et al. Experimentally induced blood-stage Plasmodium vivax infection in healthy volunteers. The Journal of infectious diseases **2013**; 208:1688-94.

36. McCarthy JS, Ruckle T, Djeriou E, et al. A Phase II pilot trial to evaluate safety and efficacy of ferroquine against early Plasmodium falciparum in an induced blood-stage malaria infection study. Malar J **2016**; 15:469.

37. NCT02123290 CgI. A Proof-of-Concept, Open Label Study to Assess the Efficacy, Safety, Tolerability and Pharmacokinetics of Single Doses of DSM265 in Adult Patients With Acute, Uncomplicated Plasmodium Falciparum or Vivax Malaria Mono-Infection Over a 28-Day-Extended Observation Period. **2014**.

38. Payne RO, Milne KH, Elias SC, et al. Demonstration of the Blood-Stage Plasmodium falciparum Controlled Human Malaria Infection Model to Assess Efficacy of the P. falciparum Apical Membrane Antigen 1 Vaccine, FMP2.1/AS01. The Journal of infectious diseases **2016**.

39. Straimer J, Gnädig NF, Witkowski B, et al. K13-propeller mutations confer artemisinin resistance in *Plasmodium falciparum* clinical isolates. Science **2015**; 347:428-31.

40. Saunders D, Khemawoot P, Vanachayangkul P, et al. Pharmacokinetics and pharmacodynamics of oral artesunate monotherapy in patients with uncomplicated *Plasmodium falciparum* malaria in western Cambodia. Antimicrob Agents Chemother **2012**; 56:5484-93.

41. Duncan CJ, Sheehy SH, Ewer KJ, et al. Impact on malaria parasite multiplication rates in infected volunteers of the protein-in-adjuvant vaccine AMA1-C1/Alhydrogel+CPG 7909. PLoS One **2011**; 6:e22271.

42. Pasay CJ, Rockett R, Sekuloski S, et al. Piperaquine Monotherapy of Drug-Susceptible Plasmodium falciparum Infection Results in Rapid Clearance of Parasitemia but Is Followed by the Appearance of Gametocytemia. The Journal of infectious diseases **2016**; 214:105-13.

43. Investigator's Brochure: *In vitro* expanded blood stage *Plasmodium falciparum* Cam3.II^{R539T} (K13) artemisinin-resistant challenge inoculum. 1.1 ed: QIMR Berghofer Medical Research Institute, **2017**.

44. Chalon S, Akakpo S, Duparc S, Shapiro T, Griffin P, McCarthy J. Poster presentation: Moderate and severe LFT elevations in controlled human *P. falciparum* malaria infection model: recent experience, literature review and mechanistic hypotheses. American Society of Tropical Medicine and Hygiene: 65th Annual Meeting, **16 November 2016**.

45. Warrell DA, Gilles HM. Essential malariology. 4 ed. London: Arnold., **2002**.

46. Karunaweera ND, Carter R, Grau GE, Mendis KN. Demonstration of anti-disease immunity to Plasmodium vivax malaria in Sri Lanka using a quantitative method to assess clinical disease. The American journal of tropical medicine and hygiene **1998**; 58:204-10.

47. WHO Public Assessment Report: Artesunate 50 mg tablets. http://apps.who.int/prequal/whopar/whoparproducts/WHOPAR_MA044.htm, **2011**.

48. Myint HY, Ashley EA, Day NP, Nosten F, White NJ. Efficacy and safety of dihydroartemisinin-piperaquine. Transactions of the Royal Society of Tropical Medicine and Hygiene **2007**; 101:858-66.

49. Ratcliff A, Siswantoro H, Kenangalem E, et al. Two fixed-dose artemisinin combinations for drug-resistant falciparum and vivax malaria in Papua, Indonesia: an open-label randomised comparison. Lancet **2007**; 369:757-65.

50. Karunajeewa H, Lim C, Hung TY, et al. Safety evaluation of fixed combination piperaquine plus dihydroartemisinin (Artekin) in Cambodian children and adults with malaria. British journal of clinical pharmacology **2004**; 57:93-9.

51. Mytton OT, Ashley EA, Peto L, et al. Electrocardiographic safety evaluation of dihydroartemisinin piperaquine in the treatment of uncomplicated falciparum malaria. The American journal of tropical medicine and hygiene **2007**; 77:447-50.

52. European Medicines Agency. Eurartesim Assessment Report, **2011**.

53. European Medicines Agency. Eurartesim Product Information, **Updated 2016**.

54. Investigator's Brochure: Piperaquine phosphate: Medicines for Malaria Venture, **2012**.

55. Hanpithakpong W, Kamanikom B, Dondorp AM, et al. A liquid chromatographic-tandem mass spectrometric method for determination of artesunate and its metabolite dihydroartemisinin in human plasma. Journal of chromatography B, Analytical technologies in the biomedical and life sciences **2008**; 876:61-8.

56. ANZCTR Trial ID: ACTRN12612000824864. An experimental study to characterize the in vivo infectivity of the *Plasmodium falciparum* isolate HMP02Pf in healthy human volunteers, **2012**.

57. ANZCTR Trial ID: ACTRN12616000174482. A phase IB experimental study to assess the *in vivo* safety and response to chloroquine of *Plasmodium vivax* isolate HMPBS02-Pv in healthy participants with induced blood stage malaria infection, **2016**.

58. National Health and Medical Research Council. National statement on ethical conduct in human research 2007 2007 ed, **2007**:1 - 95.

59. Australian Therapeutic Goods Administration. Notes for Guidance on Good Clinical Practice (GCP) (CPMP/ICH/135/95). www.tga.gov.au/sites/default/files/ich13595an.pdf, **2000**.

60. QIMR Berghofer Medical Research Institute. Clinical Trial SOPs https://oldintranet.qimr.edu.au/intranet/corporate/ethics/clinicaltrials-sops.html, **Accessed 2017**.

61. QIMR Berghofer Medical Research Institute, . Policy on the Criteria for Authorship. https://oldintranet.qimr.edu.au/intranet/corporate/policy/2.11_AuthorshipCriteria.pdf, **2008, reviewed 2013**.

62. QIMR Berghofer Medical Research Institute. Media Relations Policy. https://oldintranet.qimr.edu.au/intranet/corporate/policy/1.4_Media%20Relations%20Policy.pdf, **2010**.

13.0 APPENDICES

Appendix 1: Schedule of Events

Approximate days based on threshold for commencement of treatment. Table indicative only. Refer to Section 7.1-7.2, and Appendix 2.

Procedures	Screen	Safety visit (if required)	Challenge Inoculation	Malaria Monitoring		Drug Treatment			Safety monitoring	Eurartesim® treatment (if required)	Malarone® treatment	Follow-up visit	Follow-up phone call	Final visit/EOS
**Day**	**-D28 to -D1**	**-D3 to -D1**	**D0**	**D1 to D3**	**Daily from D4 until qPCR +ve and then AM&PM until admission**	**Admission approx. D8**	**Confinement at clinical unit**	**Exit of unit**	**Post-confinement**	**Post-confinement**	**D26±3**	**D28±3**	**D56±7**	**D90±14**
Informed consent & eligibility	X		X											
Medical history	X		X									X		X
Physical examination	X		X[a]		X[b]	X[a]	X[b]	X[a]	X[b]			X		X[a]
ECG	X		X			X				X		X		
Vital signs	X		X		X	X	X	X	X	X	X	X		X
Haematology & biochemistry	X	X				X		X	X	X	X	X		
Serology	X											X		
RBC alloantibody	X											X		X
G6PD testing	X													
Urinalysis	X	X				X			X			X		
Drug & alcohol screen	X		X			X								
Phone call				X							X		X	
Blood stage challenge			X											
Unit confinement						X	X							
**Drug treatment**							X			X	X			
Adverse events			X	X	X	X	X	X	X	X	X	X	X	X
Malaria qPCR			X		X	X	X	X	X	X		X		

Gametocyte qRT-PCR (if required)						X	X	X	X			X		
PK (artesunate and dihydroartemisinin concentration)						X	X							
Safety serum storage			X									X		X[d]

[a] Abbreviated physical examination.

[b] Abbreviated physical examination if clinically indicated.

[d] 2 serum samples to be taken at Day 90±14 (1 serum sample at other time-points).

Appendix 2: Detailed Laboratory Procedures

Haematology

FBC w/ differential
White blood cell count (WBC)
WBC differential (diff)
A manual blood smear should be reviewed if there are immature/abnormal cells detected on the automated differential or if an automated differential was not able to be performed.
• neutrophils (NEUT)
• lymphocytes (LYM)
• monocytes (MON)
• eosinophils (EOS)
• basophils (BAS)
Red blood cell count (RBC)
Haemoglobin (HGB)
Haematocrit (HCT)
Platelet count (PLAT)
Reticulocyte count (RETI) ***(Day -3 to -1 safety visit and Day 28±3 only)***
RBC alloantibodies ***(Only at screening, Day 28±3, Day 90±14 or early termination visit).*** Blood Group and Rh(D) tests at screening only.
Glucose-6-phosphate dehydrogenase (G6PD) ***(Screening only)***

Biochemistry

Sodium (SODIUM)	Alkaline phosphatase (ALP)
Potassium (K)	Alanine aminotransferase (ALT, SGPT)
Chloride (CL)	Aspartate aminotransferase (AST, SGOT)
Bicarbonate (BICARB)	Calcium (CA)
Glucose (GLUC)	Phosphate (PHOS)
Blood urea	Lactate dehydrogenase (LDH)
Creatinine (CREAT)	Magnesium ***(Screening only)***
Uric acid	Cholesterol ***(Screening only)***
Albumin (ALB)	Triglycerides ***(Screening only)***
Globulin	HDL ***(Screening only)***
Total protein	
Total bilirubin (BILI)	
Direct (conjugated) bilirubin (BILDIR)	

Urinalysis - Clinical unit dipstick. Send to Pathology if abnormal and clinically significant.

Glucose (GLUC)
Bilirubin (BILI)
Ketone (KETONES)
Specific gravity (SPGRAV)
Blood

pH
Protein (PROT)
Urobilinogen (UROBIL)
Nitrite
Leukocytes (WBC)
Microscopy (performed only when urinalysis - leucocytes/ RBC/ protein results are abnormal)

Serology

HIV total Ab
Hepatitis B (HBsAg, anti-HBc (IgG + IgM if IgG is positive))
Hepatitis C (anti-HCV)
Hepatitis A (anti-HAV) (IgM) - performed off stored sample for testing, at Investigator's discretion
Hepatitis E (anti-HEV) (IgM) - performed off stored sample for testing, at Investigator's discretion
EBV - performed off stored sample for testing, at Investigator's discretion
CMV - performed off stored sample for testing, at Investigator's discretion

Urine Drug Screen and Alcohol Breath Test

**Urine Testing:**	
Amphetamines	Opiates
Methamphetamines	Phencyclidine
Barbiturates	Tetrahydrocannabinol (cannabis)
Benzodiazepines	Tricyclic antidepressants
Cocaine	Acetaminophen (paracetamol)*
Methadone	
**Alcohol breath test**	

Medical History

Past Medical/Surgical History Includes:
History of all known allergies
Current medications, including over-the-counter and herbal preparations
History of substance abuse and recreational drug use
History of depression, anxiety, mental illness, emotional problems, use of psychiatric medications
Surgical procedures and results

Physical Examination

Complete Physical Examination Includes:
Weight **(Screening only)**
Height **(Screening only)**
Vital signs (body temperature [sublingual], resting pulse, respiratory rate, resting blood pressure)
Review of systems excluding genitourinary examination and including the following:
• Head, neck (including thyroid), ears, eyes, nose and throat
• Heart/circulation
• Chest
• Lungs
• Abdomen
• Skin

• Neurological exam
• Extremities
• Back
• Dentition
Abbreviated Physical Examination Includes:
Vital signs (as above)
Systems/organs to examine:
• General appearance
• Skin
• Chest
• Lungs
• Heart/circulation
• Abdomen
• Brief neurological exam
• Other areas in relation to reported adverse events

Appendix 3: Total Blood Volume

Procedure	Sample	Volume per sample (mL)	No. samples per participant	Total volume per participant (mL)
Laboratory Safety Assessment	Haematology (including G6PD)	2	8	16
		4	2	8
	Biochemistry	5	10	50
	Serology	3.5	2	7
	Safety Serum storage	5	4	20
	Red Blood Cell alloantibody	4	3	12
Bioanalysis	PK analysis	2	13	26
Cannulation	Discard	2	23	46
Malaria Monitoring	Malaria qPCR (18S)	2	35	70
	Gametocyte qRT-PCR	Up to 2.5	10	25
	Gametocyte qRT-PCR	Up to 4.5	Up to 4	18
			**Study Total (mL)**	298

The actual times of blood sampling may change. Additional blood samples may be taken for unscheduled safety and qPCR assessments as required by the Investigator, provided the total volume taken during the study does not exceed 450 mL during any period of 30 consecutive days.

Appendix 4: Symptoms and Signs of Malaria

Following challenge via the intravenous malaria challenge inoculation and during the post-challenge period, the following signs and symptoms of malaria will be monitored:

Signs of Malaria

- Fever (oral temperature of ≥38°C)
- Chills/Shivering/Rigors
- Tachycardia
- Hypotension

Symptoms of Malaria

- Headache
- Myalgia (muscle ache)
- Arthralgia (joint ache)
- Fatigue/lethargy
- Malaise (general discomfort/uneasiness)
- Sweating/hot spells
- Anorexia
- Nausea
- Vomiting
- Abdominal discomfort

Appendix 5: Production Information and Consumer Information

Malarone® TGA May 2013

- Product Information
- Consumer Medicine Information

Primacin™ TGA 28 October 2014

- Product Information
- Consumer Medicine Information

Artesunate

- WHO Public Assessment Report (2011)

Eurartesim®

- Product Information (updated 2016)

Appendix 6: Clinical Score for Malaria

Visit	Date	Symptoms	Clinical Score			
			**Absent**	**Mild (1)**	**Moderate (2)**	**Severe (3)**
		Headache				
		Myalgia (muscle ache)				
		Arthralgia (joint ache)				
		Fatigue/lethargy				
		Malaise (general discomfort/uneasiness)				
		Chills/Shivering/Rigors				
		Sweating/hot spells				
		Anorexia				
		Nausea				
		Vomiting				
		Abdominal discomfort				
		Fever				
		Tachycardia				
		Hypotension				
		Total Score	0			

Maximum therefore 3 x 14 = 42

Threshold treatment proposed to be >6

AE Grading of Malaria Signs and Symptoms

Provided as a separate document

Appendix 7: Dosing Table for Artesunate Tablets

Weight range inclusive (kg) – based on weight at screening	Artesunate dose (mg)	Number of artesunate 50 mg tablets
50-59	100	2
60-69	100	2
70-79	150	3
80-89	150	3
90-99	200	4
100	200	4

Appendix 8: Version History

Version	Date	Author(s)/Reviewer(s)	Significant Revisions
1.0	10/01/2017	R. Watts	Initial version
1.1	23/02/2017	R. Watts	Clarified Primacin™ dose wording. Added Anand Odedra as a Co-investigator. Clarified that it is only grapefruit and Seville oranges that participants should not eat (and not other citrus). Changed piperaquine treatment to Eurartesim® (piperaquine /dihydroartemisinin) treatment since piperaquine is not available.
1.2	12/04/2017	J. Wilson	All women excluded from participation in study, following Australian Red Cross Blood Service HREC request to exclude women of child-bearing age. All references to female participants, pregnancy testing and FSH testing removed.
2.0	17/05/2017	R. Watts	Added additional malaria qPCR time-points based on updated statistics data that suggests these time-points are needed to determine the parasite clearance curve. Added malaria qPCR prior to Eurartesim® treatment and at the next scheduled visit after Eurartesim® treatment. Updated gametocyte qRT-PCR blood volume to up to 2.5 mL per sample at time-points from the day of artesunate treatment. Additionally, at up to four time-points, additional blood may be collected in two different sample tube types (up to approximately 4.5 mL total blood) for comparison of RNA extraction efficiency to improve the samples for qRT-PCR. Added that participants may be treated with antimalarial drugs at the Investigator's discretion (for safety reasons). Added that qPCR for male gametocyte marker may also be performed to measure gametocyte levels. Moved reticulocyte count to Day -3 to -1 safety visit instead of Day 0 since no safety blood sample is taken on Day 0. Clarified that blood group and Rh(D)

Version	Date	Author(s)/Reviewer(s)	Significant Revisions
			tests are only done at screening. Clarified the correct wording for urea, uric acid and direct (conjugated) bilirubin tests.

Summary of Changes to QP16C14 K13 Protocol (Version 1.2 to 2.0) – 17 May 2017

Protocol Section	Change
Section 7.1.7.2, 7.2.8, Appendix 3	• Added additional malaria qPCR time-points based on updated statistics data that suggests these time-points are needed to determine the parasite clearance curve. Updated total blood volume table to reflect the qPCR time-point changes.
Section 7.1.9, 7.2.8	• Added malaria qPCR prior to Eurartesim® treatment and at the next scheduled visit after Eurartesim® treatment – this was already captured in the total blood volume as part of out-patient monitoring.
Appendix 3	• Updated gametocyte qRT-PCR blood volume to up to 2.5 ml per sample since a different tube type may be used to improve RNA extraction.
Protocol summary, Section 2.3.1.1, 4.1, 7.1.7, 7.1.9	• Added that participants may be treated with antimalarial drugs at the Investigator's discretion (for safety reasons).
Section 4.1, 7.1.8, 7.2.8	• Added that qPCR for male gametocyte marker may also be performed to measure gametocyte levels.
Appendix 2	• Moved reticulocyte count to Day -3 to -1 safety visit instead of Day 0 since no safety blood sample is taken on Day 0.
Appendix 2	• Clarified that blood group and Rh(D) tests are only done at screening.
Appendix 2	• Clarified the correct wording for urea, uric acid and direct (conjugated) bilirubin tests.

Summary of Changes to QP16C14 K13 PICF (Version 1.2 to 2.0) – 17 May 2017

PICF Section	Change
Section 10	• Updated total blood volume from approximately 255 ml to approximately 298 ml.
Section 7	• Added malaria PCR prior to Eurartesim® treatment and at the next scheduled visit after Eurartesim® treatment

16.1.2 Sample case report form

Protocol No.: QP16C14 (P2286)<br><br>Adverse Events	Randomisation No.:<br>R	Participant's Initials:

Adverse Events

Were any adverse events experienced by the participant during the study (i.e. from inoculum until study completion)?

N/A ☐ Yes ☐ No ☐

***If YES**, record the details below*:

N/A is to be ticked if this is an additional Adverse Events CRF page.

**AE No.**			
**Adverse Event**<br>*Where possible give the diagnosis, not the symptom - use precise medical terminology.*<br>*If the severity of the adverse event changes, record this as a new adverse event.*			
**Last Treatment Administered**<br>*1 - Inoculum, 2 - Artesunate,*<br>*3 - Eurartesim®,*<br>*4 - Malarone®, 5 - Primacin™,*<br>*6 - Other (specify).*			
**Date of Onset**<br>*dd/mmm/yyyy*			
**Time of Onset**<br>*24 hour clock*			
**Severity**<br>*1 - Grade 1/Mild, 2 - Grade 2/Moderate,*<br>*3 - Grade 3/Severe,*<br>*4 - Grade 4/Life-threatening,*<br>*5 - Grade 5/Death.*			
**Action Taken**<br>*1 - None, 2 - Medications taken (record details on the Concomitant Medications CRF page),*<br>*3 - Other (specify).*			
**Date of Resolution**<br>*dd/mmm/yyyy*<br>*O - Ongoing.*			
**Time of Resolution**<br>*24 hour clock*<br>*O - Ongoing.*			
**Relationship to Inoculum**<br>*1 - Unrelated, 2 - Unlikely, 3 - Possible,*<br>*4 - Probable.*			
**Relationship to Artesunate**<br>*1 - Unrelated, 2 - Unlikely, 3 - Possible,*<br>*4 - Probable, 5 - N/A.*			
**Other Treatment/Study Procedure Directly Related to AE?**<br>*1 - Eurartesim®, 2 - Malarone®,*<br>*3 - Primacin™,*<br>*4 - Other (specify), 5 - Multiple treatments/study procedures (specify each treatment/study procedure) , 6 - N/A.*			
**Relationship to Other Treatment/Study Procedure**<br>*1 - Unrelated, 2 - Unlikely, 3 - Possible,*<br>*4 - Probable, 5 - Multiple treatments/study procedures (record the other treatment/procedure directly related to AE with their respective relationship e.g. Eurartesim® - 3 and Malarone® - 3),*<br>*5 - N/A.*			
**Seriousness**<br>*Does the AE meet the definition of serious?*<br>*1 - Yes, 2 - No.*<br>*(**If YES**, ensure that the SAE is reported to the sponsor within 24 hours).*			

Protocol No.: QP16C14 (P2286)<br><br>Adverse Events	Randomisation No.:<br>R ☐ ☐ ☐	Participant's Initials:<br>☐ ☐ ☐

Adverse Events N/A ☐

AE No.			
**Adverse Event**<br>*Where possible give the diagnosis, not the symptom - use precise medical terminology.*<br>*If the severity of the adverse event changes, record this as a new adverse event.*			
**Last Treatment Administered**<br>*1 - Inoculum, 2 - Artesunate, 3 - Eurartesim®, 4 - Malarone®, 5 - Primacin™, 6 - Other (specify).*			
**Date of Onset**<br>*dd/mmm/yyyy*			
**Time of Onset**<br>*24 hour clock*			
**Severity**<br>*1 - Grade 1/Mild, 2 - Grade 2/Moderate, 3 - Grade 3/Severe, 4 - Grade 4/Life-threatening, 5 - Grade 5/Death.*			
**Action Taken**<br>*1 - None, 2 - Medications taken (record details on the Concomitant Medications CRF page), 3 - Other (specify).*			
**Date of Resolution**<br>*dd/mmm/yyyy*<br>*O - Ongoing.*			
**Time of Resolution**<br>*24 hour clock*<br>*O - Ongoing.*			
**Relationship to Inoculum**<br>*1 - Unrelated, 2 - Unlikely, 3 - Possible, 4 - Probable.*			
**Relationship to Artesunate**<br>*1 - Unrelated, 2 - Unlikely, 3 - Possible, 4 - Probable, 5 - N/A.*			
**Other Treatment/Study Procedure Directly Related to AE?**<br>*1 - Eurartesim®, 2 - Malarone®, 3 - Primacin™, 4 - Other (specify), 5 - Multiple treatments/study procedures (specify each treatment/study procedure) , 6 - N/A.*			
**Relationship to Other Treatment/Study Procedure**<br>*1 - Unrelated, 2 - Unlikely, 3 - Possible, 4 - Probable, 5 - Multiple treatments/study procedures (record the other treatment/procedure directly related to AE with their respective relationship e.g. Eurartesim® - 3 and Malarone® - 3), 5 - N/A.*			
**Seriousness**<br>*Does the AE meet the definition of serious?*<br>*1 - Yes, 2 - No.*<br>***(If YES**, ensure that the SAE is reported to the sponsor within 24 hours).*			

Protocol No.: QP16C14 (P2286)<br><br>Comments	Randomisation No.:<br>R ☐☐☐	Participant's Initials:<br>☐☐☐

Comments

Any additional comments or clarifications regarding the data contained within the participant's Case Report Form may be entered. Any comments recorded on the source notes regarding clinically relevant data and study conduct should also be transferred to the comments section.

Are there any relevant comments? Yes ☐ No ☐
If YES, *record the details below.*

**Reference** *(I.e. visit and assessment, where possible)*	**Comments**

Protocol No.: QP16C14 (P2286)<br>Comments	Randomisation No.: R	Participant's Initials:

Comments

N/A ☐

Reference (I.e. visit and assessment, where possible)	Comments

**Protocol No.: QP16C14 (P2286)**<br>**Treatment Period: Confinement - Admission**	**Randomisation No.:**<br>R	**Participant's Initials:**

Date of Visit: d d m m m y y y y

Enter the visit day below:

Day __________

Pre-Dose Alcohol Breath Test

Date of breath test: d d m m m y y y y Time: __ __ : __ __ *24 hour clock*

Was the test result positive? Yes ☐ No ☐

Pre-Dose Urine Drug Screen

Date of urine collection: d d m m m y y y y Time: __ __ : __ __ *24 hour clock*

**Test**	**Result**<br>*1 - Negative,*<br>*2- Positive.*
Acetaminophen (Paracetamol)	
Amphetamines	
Methamphetamines	
Barbiturates	
Benzodiazepines	
Cocaine	
Methadone	
Opiates	
Phencyclidine	
Tetrahydrocannabinols (Cannabis)	
Tricyclic antidepressants	

Protocol No.: QP16C14 (P2286)<br><br>Treatment Period:<br>Confinement - Admission	Randomisation No.:<br>R ☐☐☐	Participant's Initials:<br>☐☐☐

Urinalysis

Date of urine collection: ☐☐ ☐☐☐ ☐☐☐☐ (d d m m m y y y y) Time: ☐☐ : ☐☐ (24 hour clock)

**Test**	**Result**	**Clinical Assessment**<br>*1 - Normal,*<br>*2 - Abnormal NCS,*<br>*3 - Abnormal CS.*
Glucose *(mmol/L)*		
Bilirubin		
Ketone *(mmol/L)*		
Specify gravity		
Blood *(Ery/µL)*		
pH		
Protein *(g/L)*		
Urobilinogen *(mmol/L)*		
Nitrite		
Leukocytes *(Leu/µL)*		

Was a microscopy performed?
***If YES,** record the details in the Microscopy table.*

Yes ☐ No ☐

Microscopy N/A ☐

See date and time of collection in the Urinalysis table.

Were any results abnormal?
***If YES**, record the details below.*

Yes ☐ No ☐

**Abnormal Test**	**Clinically Significant?**<br>*1 - Yes,*<br>*2 - No.*

**Protocol No.: QP16C14 (P2286)**<br><br>**Treatment Period: Confinement - Admission**	**Randomisation No.:** R	**Participant's Initials:**

Pre-Dose Vital Signs
(Seated)

Date of vital signs: d d m m m y y y y   Time: __ __ : __ __ *24 hour clock*

Vital Sign	Result	Clinical Assessment<br>*1 - Normal,*<br>*2 - Abnormal NCS,*<br>*3 - Abnormal CS.*
Systolic blood pressure *(mmHg)*		
Diastolic blood pressure *(mmHg)*		
Heart rate *(bpm)*		
Respiratory rate *(breaths/min)*		
Oral temperature *(°C)*		

Pre-Dose 12-Lead ECG
(Supine)

Date of ECG: d d m m m y y y y   Time: __ __ : __ __ *24 hour clock*

**ECG Parameters**							**Clinical Assessment**<br>*1 - Normal,*<br>*2 - Abnormal NCS,*<br>*3 - Abnormal CS.*	**If Abnormal, Specify Abnormality**	
**Ventricular Rate** *(bpm)*	**Intervals**								
	**PR** *(ms)*	**RR** *(ms)*	**QRS** *(ms)*	**QT** *(ms)*	**QTcB** *(ms)*	**QTcF** *(ms)*			
									N/A ☐

**Protocol No.: QP16C14 (P2286)**<br><br>**Treatment Period: Confinement - Admission**	**Randomisation No.:**<br>**R** ☐ ☐ ☐	**Participant's Initials:**<br>☐ ☐ ☐

Abbreviated Physical Examination

Select time of day: AM ☐ PM ☐

**Body System**	**Clinical Assessment**<br>*1 - Normal,*<br>*2 - Abnormal NCS,*<br>*3 - Abnormal CS.*	**If Abnormality Present, Specify Abnormal Conditions**	
Heart/Circulation			N/A ☐
Chest			N/A ☐
Lungs			N/A ☐
Abdomen			N/A ☐
Skin			N/A ☐
Neurological exam<br>*(Brief examination)*			N/A ☐
General appearance			N/A ☐
Other *(specify)*:<br>________________			N/A ☐
Other *(specify)*:<br>________________			N/A ☐
Other *(specify)*:<br>________________			N/A ☐

IV Cannulation

Indicate which arm was cannulated for dosing:

☐ Left arm
☐ Right arm

Protocol No.: QP16C14 (P2286)<br><br>Treatment Period:<br>Confinement - Admission	Randomisation No.:<br>R ☐ ☐ ☐	Participant's Initials:<br>☐ ☐ ☐

Biochemistry

Date of blood collection: ☐☐ ☐☐☐ ☐☐☐☐ (d d m m m y y y y) Time: ☐☐ : ☐☐ (24 hour clock)

Were any results abnormal?
***If YES**, record the details below.*

Yes ☐ No ☐

Abnormal Test	**Clinically Significant?**<br>*1 - Yes,*<br>*2 - No.*

Haematology

Date of blood collection: ☐☐ ☐☐☐ ☐☐☐☐ (d d m m m y y y y) Time: ☐☐ : ☐☐ (24 hour clock)

Were any results abnormal?
***If YES**, record the details below.*

Yes ☐ No ☐

Abnormal Test	**Clinically Significant?**<br>*1 - Yes,*<br>*2 - No.*

Protocol No.: QP16C14 (P2286)<br><br>Treatment Period:<br>Confinement - Admission	Randomisation No.:<br>R	Participant's Initials:

Administration of Artesunate

Record the date and time of artesunate administration:

Date of dosing: d d m m m y y y y   Time: __ __ : __ __ *24 hour clock*

How many 50 mg artesunate tablets were administered to the participant? ________ tablets

What was the total dose administered to the participant? ___________ mg

Was artesunate administered as per the protocol? Yes ☐ No ☐
***If NO**, record the details in the Comments CRF page.*

Midday Vital Signs
(Seated)

Date of vital signs: d d m m m y y y y   Time: __ __ : __ __ *24 hour clock*

Vital Sign	Result	Clinical Assessment<br>*1 - Normal,<br>2 - Abnormal NCS,<br>3 - Abnormal CS.*
Systolic blood pressure *(mmHg)*		
Diastolic blood pressure *(mmHg)*		
Heart rate *(bpm)*		
Respiratory rate *(breaths/min)*		
Oral temperature *(°C)*		

Protocol No.: QP16C14 (P2286)<br><br>Treatment Period:<br>Confinement - Admission	Randomisation No.:<br>R _ _ _	Participant's Initials:<br>_ _ _

Evening Vital Signs
(Seated)

Date of vital signs: _ _ _ _ _ _ _ _ _ (*d d m m m y y y y*) Time: _ _ : _ _ (*24 hour clock*)

Vital Sign	Result	Clinical Assessment<br>*1 - Normal,*<br>*2 - Abnormal NCS,*<br>*3 - Abnormal CS.*
Systolic blood pressure *(mmHg)*		
Diastolic blood pressure *(mmHg)*		
Heart rate *(bpm)*		
Respiratory rate *(breaths/min)*		
Oral temperature *(°C)*		

**Protocol No.: QP16C14 (P2286)**<br><br>**Treatment Period: Confinement - Admission + 1 Day**	**Randomisation No.:**<br>R _ _ _	**Participant's Initials:**<br>_ _ _

Date of Visit: _ _ / _ _ _ / _ _ _ _
d d m m m y y y y

Enter the visit day below:

Day __________

Morning Vital Signs
(Seated)

Date of vital signs: _ _ / _ _ _ / _ _ _ _ Time: _ _ : _ _
d d m m m y y y y *24 hour clock*

**Vital Sign**	**Result**	**Clinical Assessment**<br>*1 - Normal,*<br>*2 - Abnormal NCS,*<br>*3 - Abnormal CS.*
Systolic blood pressure *(mmHg)*		
Diastolic blood pressure *(mmHg)*		
Heart rate *(bpm)*		
Respiratory rate *(breaths/min)*		
Oral temperature *(°C)*		

Midday Vital Signs
(Seated)

Date of vital signs: _ _ / _ _ _ / _ _ _ _ Time: _ _ : _ _
d d m m m y y y y *24 hour clock*

**Vital Sign**	**Result**	**Clinical Assessment**<br>*1 - Normal,*<br>*2 - Abnormal NCS,*<br>*3 - Abnormal CS.*
Systolic blood pressure *(mmHg)*		
Diastolic blood pressure *(mmHg)*		
Heart rate *(bpm)*		
Respiratory rate *(breaths/min)*		
Oral temperature *(°C)*		

Protocol No.: QP16C14 (P2286)<br>Treatment Period: Confinement - Admission + 1 Day	Randomisation No.: R _ _ _	Participant's Initials: _ _ _

Evening Vital Signs
(Seated)

Date of vital signs: d d m m m y y y y   Time: __ : __ *24 hour clock*

Vital Sign	Result	Clinical Assessment<br>*1 - Normal,*<br>*2 - Abnormal NCS,*<br>*3 - Abnormal CS.*
Systolic blood pressure *(mmHg)*		
Diastolic blood pressure *(mmHg)*		
Heart rate *(bpm)*		
Respiratory rate *(breaths/min)*		
Oral temperature *(°C)*		

Protocol No.: QP16C14 (P2286)<br><br>Treatment Period:<br>Confinement - Admission + 2 Days	Randomisation No.:<br>R				Participant's Initials:		

Date of Visit: | d | d | m | m | m | y | y | y | y |

Enter the visit day below:

Day __________

Morning Vital Signs
(Seated)

Date of vital signs: d d m m m y y y y Time: __ __ : __ __ *24 hour clock*

**Vital Sign**	**Result**	**Clinical Assessment**<br>*1 - Normal,*<br>*2 - Abnormal NCS,*<br>*3 - Abnormal CS.*
Systolic blood pressure *(mmHg)*		
Diastolic blood pressure *(mmHg)*		
Heart rate *(bpm)*		
Respiratory rate *(breaths/min)*		
Oral temperature *(°C)*		

Midday Vital Signs
(Seated)

Date of vital signs: d d m m m y y y y Time: __ __ : __ __ *24 hour clock*

**Vital Sign**	**Result**	**Clinical Assessment**<br>*1 - Normal,*<br>*2 - Abnormal NCS,*<br>*3 - Abnormal CS.*
Systolic blood pressure *(mmHg)*		
Diastolic blood pressure *(mmHg)*		
Heart rate *(bpm)*		
Respiratory rate *(breaths/min)*		
Oral temperature *(°C)*		

Protocol No.: QP16C14 (P2286)<br><br>Treatment Period:<br>Confinement - Admission + 2 Days	Randomisation No.:<br>R	Participant's Initials:

Evening Vital Signs
(Seated)

Date of vital signs: d d m m m y y y y Time: __ __ : __ __ *24 hour clock*

Vital Sign	Result	Clinical Assessment<br>*1 - Normal,*<br>*2 - Abnormal NCS,*<br>*3 - Abnormal CS.*
Systolic blood pressure *(mmHg)*		
Diastolic blood pressure *(mmHg)*		
Heart rate *(bpm)*		
Respiratory rate *(breaths/min)*		
Oral temperature *(°C)*		

**Protocol No.: QP16C14 (P2286)**<br><br>**Treatment Period:**<br>**Confinement - Admission + 3 Days**	**Randomisation No.:**<br>R	**Participant's Initials:**

Date of Visit: d d m m m y y y y

Enter the visit day below:

Day __________

Biochemistry

Date of blood collection: d d m m m y y y y   Time: __ __ : __ __ *24 hour clock*

Were any results abnormal?
***If YES**, record the details below.*

Yes ☐ No ☐

**Abnormal Test**	**Clinically Significant?** *1 - Yes, 2 - No.*

Protocol No.: QP16C14 (P2286)<br><br>Treatment Period:<br>Confinement - Admission + 3 Days	Randomisation No.:<br>R _ _ _	Participant's Initials:<br>_ _ _

Haematology

Date of blood collection: _ _ / _ _ _ / _ _ _ _ (d d m m m y y y y) Time: _ _ : _ _ (24 hour clock)

Were any results abnormal? Yes ☐ No ☐
If YES, record the details below.

Abnormal Test	Clinically Significant? *1 - Yes, 2 - No.*

Morning Vital Signs
(Seated)

Date of vital signs: _ _ / _ _ _ / _ _ _ _ (d d m m m y y y y) Time: _ _ : _ _ (24 hour clock)

Vital Sign	Result	Clinical Assessment *1 - Normal, 2 - Abnormal NCS, 3 - Abnormal CS.*
Systolic blood pressure *(mmHg)*		
Diastolic blood pressure *(mmHg)*		
Heart rate *(bpm)*		
Respiratory rate *(breaths/min)*		
Oral temperature *(°C)*		

Protocol No.: QP16C14 (P2286)<br><br>Treatment Period:<br>Confinement - Admission + 3 Days	Randomisation No.:<br>R □ □ □	Participant's Initials:<br>□ □ □

Abbreviated Physical Examination

Select time of day: AM ☐ PM ☐

Body System	Clinical Assessment<br>*1 - Normal,*<br>*2 - Abnormal NCS,*<br>*3 - Abnormal CS.*	If Abnormality Present, Specify Abnormal Conditions	
Heart/Circulation			N/A ☐
Chest			N/A ☐
Lungs			N/A ☐
Abdomen			N/A ☐
Skin			N/A ☐
Neurological exam<br>*(Brief examination)*			N/A ☐
General appearance			N/A ☐
Other *(specify)*:<br>________________			N/A ☐
Other *(specify)*:<br>________________			N/A ☐
Other *(specify)*:<br>________________			N/A ☐

Protocol No.: QP16C14 (P2286)<br><br>Treatment Period: Malaria Monitoring	Randomisation No.: R ☐☐☐	Participant's Initials: ☐☐☐

Date of Visit: ☐☐ ☐☐☐ ☐☐☐☐
d d m m m y y y y

Enter the visit day below:

☐ Day __________ AM ☐ Day __________ PM

Vital Signs
(Seated)

Date of vital signs: ☐☐ ☐☐☐ ☐☐☐☐ Time: ☐☐ : ☐☐
d d m m m y y y y *24 hour clock*

**Vital Sign**	**Result**	**Clinical Assessment**<br>*1 - Normal,*<br>*2 - Abnormal NCS,*<br>*3 - Abnormal CS.*
Systolic blood pressure *(mmHg)*		
Diastolic blood pressure *(mmHg)*		
Heart rate *(bpm)*		
Respiratory rate *(breaths/min)*		
Oral temperature *(°C)*		

Protocol No.: QP16C14 (P2286)<br><br>Treatment Period:<br>Malaria Monitoring	Randomisation No.:<br>R ☐ ☐ ☐	Participant's Initials:<br>☐ ☐ ☐

Abbreviated Physical Examination
(If clinically indicated)

N/A ☐

Select time of day: AM ☐ PM ☐

Body System	Clinical Assessment<br>*1 - Normal,*<br>*2 - Abnormal NCS,*<br>*3 - Abnormal CS.*	If Abnormality Present, Specify Abnormal Conditions	
Heart/Circulation			N/A ☐
Chest			N/A ☐
Lungs			N/A ☐
Abdomen			N/A ☐
Skin			N/A ☐
Neurological exam<br>*(Brief examination)*			N/A ☐
General appearance			N/A ☐
Other *(specify):*<br>________________			N/A ☐
Other *(specify):*<br>________________			N/A ☐
Other *(specify):*<br>________________			N/A ☐

Protocol No.: QP16C14 (P2286)<br><br>Treatment Period:<br>Safety Monitoring/End of Study/Early Termination	Randomisation No.:<br>R _ _ _	Participant's Initials:<br>_ _ _

Date of Visit: _ _ / _ _ _ / _ _ _ _
d d m m m y y y y

Enter the type of visit below:

☐ Safety Monitoring ☐ Early Termination ☐ End of Study

Enter the visit day below:

☐ Day __________ AM ☐ Day __________ PM

Urinalysis

(Day 14, Day 18, Day 28 and Early Termination only)

Date of urine collection: _ _ / _ _ _ / _ _ _ _ Time: _ _ : _ _
d d m m m y y y y *24 hour clock*

Test	Result	Clinical Assessment<br>*1 - Normal,*<br>*2 - Abnormal NCS,*<br>*3 - Abnormal CS.*
Glucose *(mmol/L)*		
Bilirubin		
Ketone *(mmol/L)*		
Specify gravity		
Blood *(Ery/µL)*		
pH		
Protein *(g/L)*		
Urobilinogen *(mmol/L)*		
Nitrite		
Leukocytes *(Leu/µL)*		

Was a microscopy performed? Yes ☐ No ☐
***If YES,** record the details in the Microscopy table.*

Protocol No.: QP16C14 (P2286)<br><br>Treatment Period: Safety Monitoring/End of Study/Early Termination	Randomisation No.: R _ _ _	Participant's Initials: _ _ _

Microscopy N/A ☐

See date and time of collection in the Urinalysis table.

Were any results abnormal? Yes ☐ No ☐
***If YES**, record the details below.*

Abnormal Test	Clinically Significant? *1 - Yes, 2 - No.*

Vital Signs
(Seated)

Date of vital signs: _ _ / _ _ _ / _ _ _ _ (d d m m m y y y y) Time: _ _ : _ _ (24 hour clock)

Vital Sign	Result	Clinical Assessment *1 - Normal, 2 - Abnormal NCS, 3 - Abnormal CS.*
Systolic blood pressure *(mmHg)*		
Diastolic blood pressure *(mmHg)*		
Heart rate *(bpm)*		
Respiratory rate *(breaths/min)*		
Oral temperature *(°C)*		

**Protocol No.: QP16C14 (P2286)**<br><br>**Treatment Period: Safety Monitoring/End of Study/Early Termination**	**Randomisation No.:**<br>R ☐☐☐	**Participant's Initials:**<br>☐☐☐

12-Lead ECG

(Supine)
(Pre-Eurartesim® dosing, approximately 3 days Post-Eurartesim® dosing, Day 28 and Early Termination only)

N/A ☐

Date of ECG: d d m m m y y y y   Time: __ : __ *24 hour clock*

ECG Parameters							Clinical Assessment *1 - Normal, 2 - Abnormal NCS, 3 - Abnormal CS.*	If Abnormal, Specify Abnormality	
Ventricular Rate *(bpm)*	Intervals								
	PR *(ms)*	RR *(ms)*	QRS *(ms)*	QT *(ms)*	QTcB *(ms)*	QTcF *(ms)*			
									N/A ☐

Biochemistry

(Pre-Eurartesim®, approximately 3 days Post-Eurartesim® dosing, Day 14, Day 18, Pre-Malarone®, after completion of Malarone®, Day 28 and Early Termination only)

N/A ☐

Date of blood collection: d d m m m y y y y   Time: __ : __ *24 hour clock*

Were any results abnormal?
If YES, record the details below.

Yes ☐ No ☐

Abnormal Test	Clinically Significant? *1 - Yes, 2 - No.*

Protocol No.: QP16C14 (P2286)<br><br>Treatment Period:<br>Safety Monitoring/End of Study/Early Termination	Randomisation No.:<br>R _ _ _	Participant's Initials:<br>_ _ _

Haematology

(Pre-Eurartesim®, approximately 3 days Post-Eurartesim® dosing, Day 14, Day 18, Pre-Malarone®, after completion of Malarone®, Day 28 and Early Termination only) N/A ☐

Date of blood collection: d d m m m y y y y Time: _ _ : _ _ *24 hour clock*

Were any results abnormal?
If YES, record the details below. Yes ☐ No ☐

Abnormal Test	Clinically Significant? *1 - Yes, 2 - No.*

Serology

(Day 28 only) N/A ☐

Date of blood collection: d d m m m y y y y Time: _ _ : _ _ *24 hour clock*

Were any results positive?
If YES, record the details below. Yes ☐ No ☐

Abnormal Test	Clinically Significant? *1 - Yes, 2 - No.*

**Protocol No.: QP16C14 (P2286)**<br><br>**Treatment Period: Safety Monitoring/End of Study/Early Termination**	**Randomisation No.:**<br>R ☐ ☐ ☐	**Participant's Initials:**<br>☐ ☐ ☐

Safety Serum Storage

(Day 28, End of Study/Day 90 and Early Termination only)

N/A ☐

Date of blood collection: ☐☐ ☐☐☐ ☐☐☐☐ Time: ☐☐ : ☐☐

d d m m m y y y y — *24 hour clock*

Red Cell Alloantibody

(Day 28, End of Study/Day 90 and Early Termination only)

N/A ☐

Date of blood collection: ☐☐ ☐☐☐ ☐☐☐☐ Time: ☐☐ : ☐☐

d d m m m y y y y — *24 hour clock*

Were any results abnormal

***If YES**, record the details below.*

Yes ☐ No ☐

**Abnormal Test**	**Clinically Significant?** *1 - Yes, 2 - No.*

Protocol No.: QP16C14 (P2286)<br><br>Treatment Period:<br>Safety Monitoring/End of Study/Early Termination	Randomisation No.:<br>R _ _ _	Participant's Initials:<br>_ _ _

Physical Examination

An abbreviated physical exam is required at all safety monitoring visits, if clinically indicated, excluding Day 28. An abbreviated physical exam is required on End of Study/Day 90 only.
A complete physical examination is required on Day 28 and Early Termination only

N/A ☐

Select time of day: AM ☐ PM ☐

Body System	N/A	Clinical Assessment<br>*1 - Normal,*<br>*2 - Abnormal NCS,*<br>*3 - Abnormal CS.*	If Abnormality Present, Specify Abnormal Conditions	
HEENT<br>*(Including neck/thyroid)*	☐			N/A ☐
Heart/Circulation				N/A ☐
Chest				N/A ☐
Lungs				N/A ☐
Abdomen				N/A ☐
Skin				N/A ☐
Neurological exam<br>*(An abbreviated physical will have a brief examination performed)*				N/A ☐
General appearance				N/A ☐
Extremities	☐			N/A ☐
Back	☐			N/A ☐
Dentition	☐			N/A ☐
Other *(specify):* ________	☐			N/A ☐
Other *(specify):* ________	☐			N/A ☐
Other *(specify):* ________	☐			N/A ☐

AN EXPERIMENTAL STUDY TO CHARACTERISE THE *IN VIVO* SAFETY AND INFECTIVITY OF A *PLASMODIUM FALCIPARUM* Cam3.II^{R539T} (K13) ARTEMISININ-RESISTANT ISOLATE IN HEALTHY PARTICIPANTS

Protocol Number: QP16C14 (P2286)

CASE REPORT FORM
TREATMENT

PARTICIPANT INITIALS: | | | |

RANDOMISATION NUMBER: | R | | | |

**Study Centre:**	Q-Pharm Pty Limited<br>Level 6, Block 8<br>Royal Brisbane and Women's Hospital<br>Herston QLD 4006<br>Australia
	Level 5, CBCRC<br>300C Herston Road<br>Herston QLD 4006<br>Australia
**Principal Investigator:**	Dr. James McCarthy
**Sponsor:**	QIMR Berghofer Medical Research Institute

Protocol No.: QP16C14 (P2286)<br><br>Treatment Period: Safety Visit	Randomisation No.:<br>R	Participant's Initials:

Date of Visit: d d m m m y y y y

Urinalysis

Date of urine collection: d d m m m y y y y   Time: __ __ : __ __ *24 hour clock*

**Test**	**Result**	**Clinical Assessment**<br>*1 - Normal,*<br>*2 - Abnormal NCS,*<br>*3 - Abnormal CS.*
Glucose *(mmol/L)*		
Bilirubin		
Ketone *(mmol/L)*		
Specify gravity		
Blood *(Ery/µL)*		
pH		
Protein *(g/L)*		
Urobilinogen *(mmol/L)*		
Nitrite		
Leukocytes *(Leu/µL)*		

Was a microscopy performed?
If YES, *record the details in the Microscopy table.*

Yes ☐   No ☐

Protocol No.: QP16C14 (P2286)<br><br>Treatment Period: Safety Visit	Randomisation No.:<br>R ☐ ☐ ☐	Participant's Initials:<br>☐ ☐ ☐

Microscopy N/A ☐

See date and time of collection in the Urinalysis table.

Were any results abnormal? Yes ☐ No ☐
***If YES**, record the details below.*

Abnormal Test	Clinically Significant?<br>*1 - Yes,*<br>*2 - No.*

Biochemistry

Date of blood collection: ☐☐ ☐☐☐ ☐☐☐☐ (d d m m m y y y y) Time: ☐☐ : ☐☐ (*24 hour clock*)

Were any results abnormal? Yes ☐ No ☐
***If YES**, record the details below.*

Abnormal Test	Clinically Significant?<br>*1 - Yes,*<br>*2 - No.*

Protocol No.: QP16C14 (P2286)<br>Treatment Period: Safety Visit	Randomisation No.:<br>R _ _ _	Participant's Initials:<br>_ _ _

Haematology

Date of blood collection: __ __ / __ __ __ / __ __ __ __ (d d m m m y y y y) Time: __ __ : __ __ (24 hour clock)

Were any results abnormal? Yes ☐ No ☐
If YES, record the details below.

Abnormal Test	Clinically Significant? *1 - Yes, 2 - No.*

Protocol No.: QP16C14 (P2286)<br><br>Treatment Period: Day 0	Randomisation No.: R	Participant's Initials:

Date of Visit: d d m m m y y y y

Pre-Inoculum Alcohol Breath Test

Date of breath test: d d m m m y y y y   Time: __ __ : __ __ *24 hour clock*

Was the test result positive?   Yes ☐   No ☐

Pre-Inoculum Urine Drug Screen

Date of urine collection: d d m m m y y y y   Time: __ __ : __ __ *24 hour clock*

**Test**	**Result**<br>*1 - Negative,*<br>*2- Positive.*
Acetaminophen (Paracetamol)	
Amphetamines	
Methamphetamines	
Barbiturates	
Benzodiazepines	
Cocaine	
Methadone	
Opiates	
Phencyclidine	
Tetrahydrocannabinols (Cannabis)	
Tricyclic antidepressants	

**Protocol No.: QP16C14 (P2286)**<br><br>**Treatment Period: Day 0**	**Randomisation No.:**<br>**R** ☐☐☐	**Participant's Initials:**<br>☐☐☐

Pre-Inoculum Vital Signs
(Seated)

Date of vital signs: ☐☐☐☐☐☐☐☐☐ Time: ☐☐ : ☐☐

d d m m m y y y y *24 hour clock*

**Vital Sign**	**Result**	**Clinical Assessment**<br>*1 - Normal,*<br>*2 - Abnormal NCS,*<br>*3 - Abnormal CS.*
Systolic blood pressure *(mmHg)*		
Diastolic blood pressure *(mmHg)*		
Heart rate *(bpm)*		
Respiratory rate *(breaths/min)*		
Oral temperature *(°C)*		

Pre-Inoculum 12-Lead ECG
(Supine)

Date of ECG: ☐☐☐☐☐☐☐☐☐ Time: ☐☐ : ☐☐

d d m m m y y y y *24 hour clock*

**ECG Parameters**							**Clinical Assessment**<br>*1 - Normal,*<br>*2 - Abnormal NCS,*<br>*3 - Abnormal CS.*	**If Abnormal, Specify Abnormality**	
**Ventricular Rate** *(bpm)*	**Intervals**								
	**PR** *(ms)*	**RR** *(ms)*	**QRS** *(ms)*	**QT** *(ms)*	**QTcB** *(ms)*	**QTcF** *(ms)*			
									N/A ☐

Protocol No.: QP16C14 (P2286)<br>Treatment Period: Day 0	Randomisation No.: R ☐☐☐	Participant's Initials: ☐☐☐

Abbreviated Physical Examination

Select time of day: AM ☐ PM ☐

Body System	Clinical Assessment<br>*1 - Normal,*<br>*2 - Abnormal NCS,*<br>*3 - Abnormal CS.*	If Abnormality Present, Specify Abnormal Conditions	
Heart/Circulation			N/A ☐
Chest			N/A ☐
Lungs			N/A ☐
Abdomen			N/A ☐
Skin			N/A ☐
Neurological exam<br>*(Brief examination)*			N/A ☐
General appearance			N/A ☐
Other *(specify):* ____________			N/A ☐
Other *(specify):* ____________			N/A ☐
Other *(specify):* ____________			N/A ☐

Protocol No.: QP16C14 (P2286)<br><br>Treatment Period: Day 0	Randomisation No.:<br>R _ _ _	Participant's Initials:<br>_ _ _

Current Medical Conditions/Events

Does the participant have any new or continuing medical history/current medical conditions/events that have changed since the screening visit? Yes ☐ No ☐

***If YES,** record the details below:*

**Details of Medical/Surgical History or Current Medical Conditions/Events**<br>*(Where possible give the diagnosis, not the symptom - use precise medical terminology)*	**Date of Diagnosis/ Procedure**<br>*dd/mmm/yyyy*	**Date of Resolution**<br>*dd/mmm/yyyy*<br>*O - Ongoing.*

Inclusion Criteria and Exclusion Criteria

Did the participant meet all the inclusion and exclusion criteria? Yes ☐ No ☐

***If NO,** list the inclusion and/or exclusion criteria number/s the participant did not meet below:*

____

____

____

____

____

**Protocol No.: QP16C14 (P2286)**<br><br>**Treatment Period: Day 0**	**Randomisation No.:**<br>**R** _ _ _	**Participant's Initials:**<br>_ _ _

IV Cannulation

Indicate which arm has been cannulated:

☐ Left arm
☐ Right arm

Pre-Inoculum Safety Serum Storage

Date of blood collection: d d m m m y y y y Time: _ _ : _ _ *24 hour clock*

Administration of Malaria Inoculum

Record the date and time of malaria inoculum administration:

Date of Inoculation: d d m m m y y y y Time: _ _ : _ _ *24 hour clock*

Was the malaria inoculum administered as per the protocol? Yes ☐ No ☐
***If NO**, record the details in the Comments CRF page.*

Discharge Vital Signs
(Seated)

Date of vital signs: d d m m m y y y y Time: _ _ : _ _ *24 hour clock*

**Vital Sign**	**Result**	**Clinical Assessment**<br>*1 - Normal,*<br>*2 - Abnormal NCS,*<br>*3 - Abnormal CS.*
Systolic blood pressure *(mmHg)*		
Diastolic blood pressure *(mmHg)*		
Heart rate *(bpm)*		
Respiratory rate *(breaths/min)*		
Oral temperature *(°C)*		

**Protocol No.: QP16C14 (P2286)**<br><br>**Study Completion**	**Randomisation No.:**<br>R ☐☐☐	**Participant's Initials:**<br>☐☐☐

Study Completion

Did the participant complete the study?
Complete the date the participant completed/discontinued the study (i.e. last day of contact).
If NO, *record the primary reason that the participant was discontinued from the study.*

Yes ☐ No ☐

Date the participant completed/discontinued the study: d d m m m y y y y

Tick only one box below and specify details in the comments section:

- ☐ N/A
- ☐ Adverse event
- ☐ Inclusion/Exclusion criteria not met
- ☐ Sponsor's discretion
- ☐ Investigator's decision
- ☐ Lost to follow-up
- ☐ Consent withdrawn
- ☐ Other

Comments:

____

____

____

____

Investigator Declaration

I certify that this is a complete and accurate record of this participant's data and that the study was conducted according to the protocol and principles of Good Clinical Practice.

Investigator's Signature: ______________________ Date: __________

**Protocol No.: QP16C14 (P2286)**<br><br>**Clinical Score**	**Randomisation No.:**<br>R __ __ __	**Participant's Initials:**<br>__ __ __

Clinical Score

Visit *e.g. Day 4 AM.*	Date *dd/mmm/yyyy*	Time *24 hour clock*	Symptom														
			Headache	Myalgia *(Muscle ache)*	Arthralgia *(Joint ache)*	Fatigue/ Lethargy	Malaise *(General discomfort/ uneasiness)*	Chills/ Shivering/ Rigors	Sweating/ Hot Spells	Anorexia	Nausea	Vomiting	Abdominal Discomfort	Fever	Tachycardia	Hypotension	Total Score

Protocol No.: QP16C14 (P2286)<br><br>Clinical Score	Randomisation No.:<br>R ☐ ☐ ☐	Participant's Initials:<br>☐ ☐ ☐

Clinical Score N/A ☐

Visit *e.g. Day 4 AM.*	Date *dd/mmm/yyyy*	Time *24 hour clock*	Symptom														
			Headache	Myalgia *(Muscle ache)*	Arthralgia *(Joint ache)*	Fatigue/ Lethargy	Malaise *(General discomfort/ uneasiness)*	Chills/ Shivering/ Rigors	Sweating/ Hot Spells	Anorexia	Nausea	Vomiting	Abdominal Discomfort	Fever	Tachycardia	Hypotension	Total Score

Protocol No.: QP16C14 (P2286) Concomitant Medications	Randomisation No.: R	Participant's Initials:

Concomitant Medications

Were any concomitant medications taken by the participant during the study? N/A ☐ Yes ☐ No ☐

N/A is to be ticked if this is an additional Concomitant Medications CRF page.

***If YES**, record the details below*:

**Con Med No.**	**Drug Name** *Trade name preferred*	**Unit Strength** *e.g. grams, mg, µg.*	**Units/ Form** *e.g. 1 tablet, 2 capsules.*	**Frequency** *e.g. stat, bd.*	**Route** *e.g. oral, topical, IM.*	**Date Drug Started** *dd/mmm/yyyy*	**Time Drug Started** *24 hour clock*	**Date Drug Stopped** *dd/mmm/yyyy O - Ongoing.*	**Time Drug Stopped** *24 hour clock O - Ongoing.*	**Condition Treated/ Indication for Use**	**Was the Drug Taken for an AE?** *1 - Yes 2 - No.*

Note: If medication is given to treat an adverse event, ensure the adverse event is recorded on the Adverse Event CRF page(s).

Protocol No.: QP16C14 (P2286)<br>Concomitant Medications	Randomisation No.: R [ ] [ ] [ ]	Participant's Initials: [ ] [ ] [ ]

Concomitant Medications

N/A ☐

Con Med No.	Drug Name<br>*Trade name preferred*	Unit Strength<br>*e.g. grams, mg, µg.*	Units/ Form<br>*e.g. 1 tablet, 2 capsules.*	Frequency<br>*e.g. stat, bd.*	Route<br>*e.g. oral, topical, IM.*	Date Drug Started<br>*dd/mmm/yyyy*	Time Drug Started<br>*24 hour clock*	Date Drug Stopped<br>*dd/mmm/yyyy O - Ongoing.*	Time Drug Stopped<br>*24 hour clock O - Ongoing.*	Condition Treated/ Indication for Use	Was the Drug Taken for an AE?<br>*1 - Yes 2 - No.*

Note: If medication is given to treat an adverse event, ensure the adverse event is recorded on the Adverse Event CRF page(s).

Protocol No.: QP16C14 (P2286)<br><br>PCR Timesheet	Randomisation No.:<br>R	Participant's Initials:

PCR Timesheet

Record each malaria PCR sample collected below.

**Visit**<br>*e.g. Day 4 AM.*	**Date**<br>*dd/mmm/yyyy*	**Time**<br>*24 hour clock*	**Was a Malaria PCR Sample Collected?**<br>*1 - Yes,*<br>*2 - No.*	**Was a Gametocyte PCR Sample Collected?**<br>*1 - Yes,*<br>*2 - No.*

Protocol No.: QP16C14 (P2286)<br><br>PCR Timesheet	Randomisation No.:<br>R	Participant's Initials:

PCR Timesheet N/A ☐

Visit<br>*e.g. Day 4 AM.*	Date<br>*dd/mmm/yyyy*	Time<br>*24 hour clock*	Was a Malaria PCR Sample Collected?<br>*1 - Yes,*<br>*2 - No.*	Was a Gametocyte PCR Sample Collected?<br>*1 - Yes,*<br>*2 - No.*

**Protocol No.: QP16C14 (P2286)**<br><br>**PK Timesheet**	**Randomisation No.:**<br>**R** __ __ __	**Participant's Initials:**<br>__ __ __

PK Timesheet

Record each PK sample collected below.

Timepoint	Date<br>*dd/mmm/yyyy*	Time<br>*24 hour clock*
T = Pre-Dose<br>*(Prior to Artesunate treatment)*		
T = 15 minutes		
T = 30 minutes		
T = 1 hour		
T = 1 hour 30 minutes		
T = 2 hours		
T = 2 hours 30 minutes		
T = 3 hours		
T = 4 hours		
T = 6 hours		
T = 8 hours		
T = 10 hours		
T = 12 hours		

Protocol No.: QP16C14 (P2286) Rescue Medication Administration	Randomisation No.:	Participant's Initials:
	R | | |	| |

Rescue Medication Administration

**Rescue Medication Administered** *1 -Eurartesim®, 2 - Malarone, 3 - Primacin™.*	**Units/ Form** *e.g. 1 tablet, 2 capsules.*	**Route** *e.g. oral, topical, IM.*	**Date Drug Taken** *dd/mmm/yyyy*	**Time Drug Taken** *24 hour clock*	**Was Drug Administered As Per the Protocol?** *1 - Yes, 2 - No.*

Protocol No.: QP16C14 (P2286)<br><br>Rescue Medication Administration	Randomisation No.: R [ ] [ ] [ ]	Participant's Initials: [ ] [ ] [ ]

Rescue Medication Administration N/A ☐

**Rescue Medication Administered**<br>*1 -Riamet®,*<br>*2 - Artersunate,*<br>*3 - Primacint®.*	**Units/ Form**<br>*e.g.*<br>*1 tablet, 2 capsules.*	**Route**<br>*e.g. oral, topical, IM.*	**Date Drug Taken**<br>*dd/mmm/yyyy*	**Time Drug Taken**<br>*24 hour clock*	**Was Drug Administered As Per the Protocol?**<br>*1 - Yes,*<br>*2 - No.*

AN EXPERIMENTAL STUDY TO CHARACTERISE THE *IN VIVO* SAFETY AND INFECTIVITY OF A *PLASMODIUM FALCIPARUM* Cam3.II^{R539T} (K13) ARTEMISININ-RESISTANT ISOLATE IN HEALTHY PARTICIPANTS

Protocol Number: QP16C14 (P2286)

CASE REPORT FORM
SCREENING

PARTICIPANT INITIALS: | | | |

RANDOMISATION NUMBER: | R | | | |

Study Centre: Q-Pharm Pty Limited
Level 6, Block 8
Royal Brisbane and Women's Hospital
Herston QLD 4006
Australia

Level 5, CBCRC
300C Herston Road
Herston QLD 4006
Australia

Principal Investigator: Dr. James McCarthy

Sponsor: QIMR Berghofer Medical Research Institute

Protocol No.: QP16C14 (P2286)<br><br>Screening Period	Randomisation No.: R ☐☐☐	Participant's Initials: ☐☐☐

Date of Visit: ☐☐ ☐☐☐ ☐☐☐☐ (d d m m m y y y y)

Informed Consent Procedure

Date of Consent: ☐☐ ☐☐☐ ☐☐☐☐ (d d m m m y y y y)   Time of Consent: ☐☐ : ☐☐ (24 hour clock)

Date of Version: ☐☐ ☐☐☐ ☐☐☐☐ (d d m m m y y y y)   Version No: ☐ . ☐

Demographics

Date of Birth: ☐☐ ☐☐☐ ☐☐☐☐ (d d m m m y y y y)   Age: ☐☐

Gender: Male ☐

Race:
- ☐ American Indian or Alaska Native
- ☐ Asian
- ☐ Black or African American
- ☐ Native Hawaiian or other Pacific Islander
- ☐ White
- ☐ Other *(specify)*: ____________________________

Height and Weight

Weight: ☐☐☐ . ☐ kg   Height: ☐ . ☐☐ m   BMI: ☐☐ . ☐ kg/m^2

Smoking History

Is the participant, or has the participant ever been, a smoker?

- ☐ Never
- ☐ Ex-smoker   *Date participant ceased smoking:* ☐☐ ☐☐☐ ☐☐☐☐ (d d m m m y y y y)
- ☐ Current
  *Record the type of products and number of products/day:*______________________________________

**Protocol No.: QP16C14 (P2286)**<br><br>**Screening Period**	**Randomisation No.:**<br>R	**Participant's Initials:**

Medical/Surgical History or Current Conditions/Events

Does the participant have any medical/surgical history and/or current medical conditions/events? Yes ☐ No ☐

If YES, *record the details below:*

**Details of Medical/Surgical History or Current Medical Conditions/Events**<br>*(Where possible give the diagnosis, not the symptom - use precise medical terminology)*	**Date of Diagnosis/ Procedure**<br>*dd/mmm/yyyy*	**Date of Resolution**<br>*dd/mmm/yyyy*<br>*O - Ongoing.*

Protocol No.: QP16C14 (P2286) Screening Period	Randomisation No.: R ☐ ☐ ☐	Participant's Initials: ☐ ☐ ☐

Physical Examination

Select time of day: AM ☐ PM ☐

Body System	Clinical Assessment *1 - Normal, 2 - Abnormal NCS, 3 - Abnormal CS.*	If Abnormality Present, Specify Abnormal Conditions	
HEENT *(Including neck/thyroid)*			N/A ☐
Heart/Circulation			N/A ☐
Chest			N/A ☐
Lungs			N/A ☐
Abdomen			N/A ☐
Skin			N/A ☐
Neurological exam			N/A ☐
Extremities			N/A ☐
Back			N/A ☐
Dentition			N/A ☐
Other *(specify):* ____________			N/A ☐
Other *(specify):* ____________			N/A ☐
Other *(specify):* ____________			N/A ☐

Protocol No.: QP16C14 (P2286)<br>Screening Period	Randomisation No.: R	Participant's Initials:

Alcohol Breath Test

Date of breath test: d d m m m y y y y   Time: __ : __ (24 hour clock)

Was the test result positive?   Yes ☐   No ☐

Urine Drug Screen

Date of urine collection: d d m m m y y y y   Time: __ : __ (24 hour clock)

Test	Result<br>*1 - Negative,*<br>*2- Positive.*
Acetaminophen (Paracetamol)	
Amphetamines	
Methamphetamines	
Barbiturates	
Benzodiazepines	
Cocaine	
Methadone	
Opiates	
Phencyclidine	
Tetrahydrocannabinols (Cannabis)	
Tricyclic antidepressants	

Protocol No.: QP16C14 (P2286) Screening Period	Randomisation No.: R	Participant's Initials:

Urinalysis

Date of urine collection: d d m m m y y y y   Time: __ : __ (24 hour clock)

Test	Result	Clinical Assessment *1 - Normal, 2 - Abnormal NCS, 3 - Abnormal CS.*
Glucose *(mmol/L)*		
Bilirubin		
Ketone *(mmol/L)*		
Specify gravity		
Blood *(Ery/µL)*		
pH		
Protein *(g/L)*		
Urobilinogen *(mmol/L)*		
Nitrite		
Leukocytes *(Leu/µL)*		

Was a microscopy performed?
***If YES,** record the details in the Microscopy table.*

Yes ☐ No ☐

Microscopy N/A ☐

See date and time of collection in the Urinalysis table.

Were any results abnormal?
***If YES**, record the details below.*

Yes ☐ No ☐

Abnormal Test	Clinically Significant? *1 - Yes, 2 - No.*

Protocol No.: QP16C14 (P2286)<br><br>Screening Period	Randomisation No.: R				Participant's Initials:		

Vital Signs
(Supine)

Date of vital signs: | | | | | | | | | (d d m m m y y y y) Time: | | : | | (24 hour clock)

Vital Sign	Result	Clinical Assessment<br>*1 - Normal,*<br>*2 - Abnormal NCS,*<br>*3 - Abnormal CS.*
Systolic blood pressure *(mmHg)*		
Diastolic blood pressure *(mmHg)*		
Heart rate *(bpm)*		
Respiratory rate *(breaths/min)*		
Oral temperature *(°C)*		

Vital Signs
(Standing)

Date of vital signs: | | | | | | | | | (d d m m m y y y y) Time: | | : | | (24 hour clock)

Vital Sign	Result	Clinical Assessment<br>*1 - Normal,*<br>*2 - Abnormal NCS,*<br>*3 - Abnormal CS.*
Systolic blood pressure *(mmHg)*		
Diastolic blood pressure *(mmHg)*		
Heart rate *(bpm)*		

**Protocol No.: QP16C14 (P2286)** **Screening Period**	**Randomisation No.:** R _ _ _	**Participant's Initials:** _ _ _

12-Lead ECG
(Supine)

Date of ECG: d d m m m y y y y   Time: _ _ : _ _ *24 hour clock*

ECG Parameters							**Clinical Assessment** *1 - Normal, 2 - Abnormal NCS, 3 - Abnormal CS.*	**If Abnormal, Specify Abnormality**	
**Ventricular Rate** *(bpm)*	**Intervals**								
	**PR** *(ms)*	**RR** *(ms)*	**QRS** *(ms)*	**QT** *(ms)*	**QTcB** *(ms)*	**QTcF** *(ms)*			
									N/A ☐

Biochemistry
(Including magnesium, cholesterol, triglycerides and HDL)

Date of blood collection: d d m m m y y y y   Time: _ _ : _ _ *24 hour clock*

Were any results abnormal?   Yes ☐ No ☐
***If YES**, record the details below.*

**Abnormal Test**	**Clinically Significant?** *1 - Yes, 2 - No.*

**Protocol No.: QP16C14 (P2286)**<br><br>**Screening Period**	**Randomisation No.:**<br>**R**	**Participant's Initials:**

Haematology

Date of blood collection: d d m m m y y y y Time: : 24 hour clock

Were any results abnormal?
***If YES**, record the details below.*

Yes ☐ No ☐

**Abnormal Test**	**Clinically Significant?** *1 - Yes, 2 - No.*

Serology
(HIV, Hepatitis B & Hepatitis C)

Date of blood collection: d d m m m y y y y Time: : 24 hour clock

Were any results positive?
***If YES**, record the details below.*

Yes ☐ No ☐

**Abnormal Test**	**Clinically Significant?** *1 - Yes, 2 - No.*

**Protocol No.: QP16C14 (P2286)**<br>**Screening Period**	**Randomisation No.:**<br>R	**Participant's Initials:**

Red Cell Alloantibody
(Including blood group and Rh(D))

Date of blood collection: | | | | | | | | | | (d d m m m y y y y) Time: | | | : | | | (24 hour clock)

Were any results abnormal? Yes ☐ No ☐
***If YES**, record the details below.*

**Abnormal Test**	**Clinically Significant?**<br>*1 - Yes,*<br>*2 - No.*

G6PD

Date of blood collection: | | | | | | | | | | (d d m m m y y y y) Time: | | | : | | | (24 hour clock)

Were any results abnormal? Yes ☐ No ☐
***If YES**, record the details below.*

**Abnormal Test**	**Clinically Significant?**<br>*1 - Yes,*<br>*2 - No.*

Inclusion Criteria and Exclusion Criteria

Did the participant meet all the inclusion and exclusion criteria? Yes ☐ No ☐
***If NO,** list the inclusion and/or exclusion criteria number/s the participant did not meet below:*

STUDY SPECIFIC INSTRUCTION
QP16C14 (P2286)

Review (*):	PI Approval:	Implementation Date: 12 Dec 2017
Date: 12/12/2017	Date: 12/12/17	

*QA review advisable, especially for novel activities, otherwise a staff member with relevant experience.

Prepared by:

Signature: ______________________ 12 Dec 2017
Data Management Supervisor Date

TITLE: Completion of a Case Report Form (CRF)

PURPOSE: To standardise completion of CRFs

PERSONNEL: All study staff allocated to enter into the CRF and signed off on reading this document as training.

VERSION DETAILS: Version 1.2 dated 29NOV2017

NOTE: Trained study staff will enter source data from the participant notes into the CRF. The CRF is designed to record all of the protocol required information to be reported to the sponsor on each participant taking part in the study that has been allocated a randomisation number.

STEPS:

- Always use black ball point pen and ensure all entries are complete and legible.
- Do not leave blank spaces unless instructed to do so in the CRF. If data is missing, use one of the following abbreviations:
  NA - Not applicable
  ND - Not done
  UN or UNK or UNKN - Unknown
- If boxes are provided for data entry fields, enter a single digit in each cell using a zero to preceded the value when necessary e.g.
  weight

0	7	1	.	5	kg

- Complete all dates in the format of day, month, year, i.e. DD/MMM/YYYY.
- Complete all times in 24 hour format i.e. HH:MM.

Implementation / revision of this instruction shall be recorded using TP C407
Original document shall be archived in the Trial Master File

This document is ONLY for use in the nominated trial.

- Ensure all header information (i.e. participant initials and randomisation number) is completed consistently throughout the CRF. Missing initials should be recorded with a dash '-' e.g. M-G.

- Do not use participant identifiers anywhere in the CRF such as, name, address etc. in order to maintain the confidentiality of the participant.

- If it is necessary to change or correct an entry on the CRF, draw a single line through the incorrect entry and enter the correct information beside or near the original data. The original entry must remain visible. Initial and date each correction.

- Do not use whiteout, erase or overwrite original entries.

- If changes are made to any CRF page after the Principal Investigator (PI) has signed off on the completion of the CRF, then the PI must review the change and countersign the CRF.

- If changes are to be made post original CRF pages sent to the sponsor, a data clarification form (DCF) will be created to track the required change and must be reviewed and signed off by the PI or designee.

- Do not write in the margins of the forms, unless making corrections to incorrect data.

- Comments should be printed succinctly in either the comments fields provided or on the Comments CRF page and limited to those that are necessary to clarify data requested on the CRF.

- Unscheduled assessment CRF pages should be used for any unplanned assessments. Reasons for the unscheduled assessment are to be written on the Comments CRF page. If the unscheduled assessments occur during a planned visit the unscheduled assessment CRF page/s should be inserted beside the relevant visit page and the page number will be X.1 of Y, X.2 of Y, X.3 of Y, etc., where X will be the same page number the unscheduled assessment CRF page/s is placed next to and Y is the total number of pages within that visit document. E.g. if an unscheduled biochemistry was performed at a malaria monitoring visit the Unscheduled Biochemistry CRF page will be placed with the Treatment: Malaria Monitoring CRF document which has a total of 2 pages. The Unscheduled Biochemistry CRF page will then be numbered page 2.1 of 2. If an unscheduled haematology was also performed then the Unscheduled Haematology CRF page would be placed with the Unscheduled Biochemistry CRF page and numbered page 2.2 of 2.

- This CRF is compiled of separate documents to match the flow of the participant's visit e.g. Screening CRF, Treatment Period: Safety Visit to D0 CRF, Telephone Contact CRF, Treatment: Malaria Monitoring CRF etc.. Each document should only be placed in the participant's CRF folder when the visit occurs, however each participant should have at a minimum each of the following documents:
  - Screening CRF
  - Treatment Period: Safety Visit to Day 0 CRF
  - Treatment: Safety Monitoring/Early Term/EOS CRF
  - Rescue Medication Administration CRF
  - PCR Timesheet CRF
  - Concomitant Medications CRF
  - Adverse Events CRF
  - Comments CRF
  - Study Completion CRF

- The PI will review the CRFs for completeness and accuracy and provide his or her signature and date to the CRF as evidence thereof. The PI may delegate this to another Sub-Investigator.

Protocol No.: QP16C14 (P2286)<br><br>Treatment Period:<br>Telephone Contact	Randomisation No.:<br>R ☐☐☐	Participant's Initials:<br>☐☐☐

Enter the visit day below:

Day __________

Telephone Contact

Was telephone contact with the participant attempted? Yes ☐ No ☐

If telephone contact was attempted, was contact made? N/A ☐ Yes ☐ No ☐

***If YES**, record the date and time below:*

Date of contact: ☐☐ ☐☐☐ ☐☐☐☐ (*d d m m m y y y y*) Time: ☐☐ : ☐☐ (*24 hour clock*)

Enter the visit day below:

☐ Day __________ ☐ N/A

Telephone Contact

Was telephone contact with the participant attempted? Yes ☐ No ☐

If telephone contact was attempted, was contact made? N/A ☐ Yes ☐ No ☐

***If YES**, record the date and time below:*

Date of contact: ☐☐ ☐☐☐ ☐☐☐☐ (*d d m m m y y y y*) Time: ☐☐ : ☐☐ (*24 hour clock*)

Enter the visit day below:

☐ Day __________ ☐ N/A

Telephone Contact

Was telephone contact with the participant attempted? Yes ☐ No ☐

If telephone contact was attempted, was contact made? N/A ☐ Yes ☐ No ☐

***If YES**, record the date and time below:*

Date of contact: ☐☐ ☐☐☐ ☐☐☐☐ (*d d m m m y y y y*) Time: ☐☐ : ☐☐ (*24 hour clock*)

**Protocol No.: QP16C14 (P2286)**<br><br>**Unscheduled Biochemistry**	**Randomisation No.:**<br>R ☐ ☐ ☐	**Participant's Initials:**<br>☐ ☐ ☐

Biochemistry

Date of blood collection: ☐☐ ☐☐☐ ☐☐☐☐ (*d d m m m y y y y*) Time: ☐☐ : ☐☐ (*24 hour clock*)

Were any results abnormal? Yes ☐ No ☐

***If YES**, record the details below.*

**Abnormal Test**	**Clinically Significant?** *1 - Yes, 2 - No.*

**Protocol No.: QP16C14 (P2286)**<br><br>**Unscheduled ECG**	**Randomisation No.:**<br>**R** ☐☐☐	**Participant's Initials:**<br>☐☐☐

12-Lead ECG
(Supine)

Date of ECG: ☐☐ ☐☐☐ ☐☐☐☐ (d d m m m y y y y)   Time: ☐☐ : ☐☐ (*24 hour clock*)

**ECG Parameters**							**Clinical Assessment** *1 - Normal, 2 - Abnormal NCS, 3 - Abnormal CS.*	**If Abnormal, Specify Abnormality**	
**Ventricular Rate** *(bpm)*	**Intervals**								
	**PR** *(ms)*	**RR** *(ms)*	**QRS** *(ms)*	**QT** *(ms)*	**QTcB** *(ms)*	**QTcF** *(ms)*			
									N/A ☐

**Protocol No.: QP16C14 (P2286)**<br><br>**Unscheduled Haematology**	**Randomisation No.:**<br>**R**				**Participant's Initials:**		

Haematology

Date of blood collection: | | | | | | | | | (d d m m m y y y y)   Time: | | : | | (24 hour clock)

Were any results abnormal?
***If YES**, record the details below.*

Yes ☐   No ☐

**Abnormal Test**	**Clinically Significant?** *1 - Yes, 2 - No.*

**Protocol No.: QP16C14 (P2286)**<br><br>**Unscheduled Physical Exam**	**Randomisation No.:**<br>R ☐ ☐ ☐	**Participant's Initials:**<br>☐ ☐ ☐

Abbreviated Physical Examination N/A ☐

Select time of day: AM ☐ PM ☐

Body System	Clinical Assessment<br>*1 - Normal,*<br>*2 - Abnormal NCS,*<br>*3 - Abnormal CS.*	If Abnormality Present, Specify Abnormal Conditions	
Heart/Circulation			N/A ☐
Chest			N/A ☐
Lungs			N/A ☐
Abdomen			N/A ☐
Skin			N/A ☐
Neurological exam<br>*(Brief examination)*			N/A ☐
General appearance			N/A ☐
Other *(specify):*<br>____________________			N/A ☐
Other *(specify):*<br>____________________			N/A ☐
Other *(specify):*<br>____________________			N/A ☐

Protocol No.: QP16C14 (P2286)<br><br>Unscheduled Physical Exam	Randomisation No.:<br>R __ __ __	Participant's Initials:<br>__ __ __

Physical Examination N/A ☐

Select time of day: AM ☐ PM ☐

Body System	Clinical Assessment<br>*1 - Normal,*<br>*2 - Abnormal NCS,*<br>*3 - Abnormal CS.*	If Abnormality Present, Specify Abnormal Conditions	
HEENT<br>*(Including neck/thyroid)*			N/A ☐
Heart/Circulation			N/A ☐
Chest			N/A ☐
Lungs			N/A ☐
Abdomen			N/A ☐
Skin			N/A ☐
Neurological exam			N/A ☐
Extremities			N/A ☐
Back			N/A ☐
Dentition			N/A ☐
Other *(specify):*<br>________________			N/A ☐
Other *(specify):*<br>________________			N/A ☐
Other *(specify):*<br>________________			N/A ☐

Protocol No.: QP16C14 (P2286)<br><br>Unscheduled Urinalysis and Microscopy	Randomisation No.:<br>R	Participant's Initials:

Urinalysis

Date of urine collection: d d m m m y y y y   Time: __ __ : __ __ 24 hour clock

Test	Result	Clinical Assessment<br>*1 - Normal,*<br>*2 - Abnormal NCS,*<br>*3 - Abnormal CS.*
Glucose *(mmol/L)*		
Bilirubin		
Ketone *(mmol/L)*		
Specify gravity		
Blood *(Ery/µL)*		
pH		
Protein *(g/L)*		
Urobilinogen *(mmol/L)*		
Nitrite		
Leukocytes *(Leu/µL)*		

Was a microscopy performed?
If YES, *record the details in the Microscopy table.*
Yes ☐ No ☐

Microscopy N/A ☐

See date and time of collection in the Urinalysis table.

Were any results abnormal?
If YES*, record the details below.*
Yes ☐ No ☐

Abnormal Test	Clinically Significant?<br>*1 - Yes,*<br>*2 - No.*

Protocol No.: QP16C14 (P2286)<br><br>Unscheduled Vital Signs	Randomisation No.:<br>R ☐ ☐ ☐	Participant's Initials:<br>☐ ☐ ☐

Vital Signs

Date of vital signs: ☐☐ ☐☐☐ ☐☐☐☐ (d d m m m y y y y)   Time: ☐☐ : ☐☐ (24 hour clock)

Record the position the participant was in when the vital signs were taken:

☐ Supine ☐ Standing ☐ Seated ☐ Other position *(specify)*: __________

Vital Sign	Result	Clinical Assessment<br>*1 - Normal,*<br>*2 - Abnormal NCS,*<br>*3 - Abnormal CS.*
Systolic blood pressure *(mmHg)*		
Diastolic blood pressure *(mmHg)*		
Heart rate *(bpm)*		
Respiratory rate *(breaths/min)*		
Oral temperature *(°C)*		

Vital Signs N/A ☐

Date of vital signs: ☐☐ ☐☐☐ ☐☐☐☐ (d d m m m y y y y)   Time: ☐☐ : ☐☐ (24 hour clock)

Record the position the participant was in when the vital signs were taken:

☐ Supine ☐ Standing ☐ Seated ☐ Other position *(specify)*: __________

Vital Sign	Result	Clinical Assessment<br>*1 - Normal,*<br>*2 - Abnormal NCS,*<br>*3 - Abnormal CS.*
Systolic blood pressure *(mmHg)*		
Diastolic blood pressure *(mmHg)*		
Heart rate *(bpm)*		
Respiratory rate *(breaths/min)*		
Oral temperature *(°C)*		

Protocol No.: QP16C14 (P2286)<br><br>Unscheduled Vital Signs	Randomisation No.:<br>R ☐ ☐ ☐	Participant's Initials:<br>☐ ☐ ☐

Vital Signs N/A ☐

Date of vital signs: ☐☐☐☐☐☐☐☐☐ (d d m m m y y y y) Time: ☐☐ : ☐☐ (24 hour clock)

Record the position the participant was in when the vital signs were taken:

☐ Supine ☐ Standing ☐ Seated ☐ Other position *(specify)*: __________

Vital Sign	Result	Clinical Assessment<br>*1 - Normal,*<br>*2 - Abnormal NCS,*<br>*3 - Abnormal CS.*
Systolic blood pressure *(mmHg)*		
Diastolic blood pressure *(mmHg)*		
Heart rate *(bpm)*		
Respiratory rate *(breaths/min)*		
Oral temperature *(°C)*		

Vital Signs N/A ☐

Date of vital signs: ☐☐☐☐☐☐☐☐☐ (d d m m m y y y y) Time: ☐☐ : ☐☐ (24 hour clock)

Record the position the participant was in when the vital signs were taken:

☐ Supine ☐ Standing ☐ Seated ☐ Other position *(specify)*: __________

Vital Sign	Result	Clinical Assessment<br>*1 - Normal,*<br>*2 - Abnormal NCS,*<br>*3 - Abnormal CS.*
Systolic blood pressure *(mmHg)*		
Diastolic blood pressure *(mmHg)*		
Heart rate *(bpm)*		
Respiratory rate *(breaths/min)*		
Oral temperature *(°C)*		

16.1.3 List of Human Research Ethics Committees

QIMR Berghofer Medical Research Institute Human Research Ethics Committee

Phone: (07) 3362 0117

Fax: (07) 3362 0109

email: hrec.secretariat@qimrberghofer.edu.au

Australian Red Cross Blood Service Human Research Ethics Committee

Phone: (02) 9234 2368

email: ethics@redcrossblood.org.au

16.1.4 List and description of investigators and other important participants in the study

Principal Investigator:	Dr James McCarthy MBBS<br>Employed by and located at:<br>Q-Pharm Pty Ltd (visiting Medical Officer) and<br>QIMR Berghofer Medical Research Institute<br>Level 5, 300C Herston Road<br>Herston, QLD 4006<br>Australia<br>Tel: +61 (0)7 3845 3647 or +61 (0)7 3845 3636<br>Mobile: +61 4144204659<br>Email: j.mccarthy@uq.edu.au
Co-Investigators:	Dr Paul Griffin MBBS FRACP FRCPA<br>Q-Pharm Pty Ltd<br>Level 5, 300C Herston Road<br>Herston, QLD 4006<br>Australia<br>Tel: +61 (0)7 3845 3647<br>Fax: +61 (0)7 3845 3637<br>Email: paul.griffin@infectiousdiseasesqld.com.au<br><br>Dr Mark Armstrong<br>Level 5, 300C Herston Road<br>Herston, QLD 4006<br>Australia<br>Tel: +61 (0)7 3845 3636<br>Email: M.Armstrong@qpharm.com.au<br><br>Dr Anand Odedra<br>Level 5, 300C Herston Road<br>Herston, QLD 4006<br>Australia<br>Tel: +61 (0)7 3845 3636<br>Email: Anand.Odedra@qimrberghofer.edu.au
Independent Medical Monitor:	Professor Dennis Shanks<br>Australian Army Malaria Institute<br>Gallipoli Barracks<br>Enoggera, QLD 4051 Australia<br>Tel: +61 (0)7 3332 4931<br>Email: Dennis.SHANKS@defence.gov.au

Statistician:	Prof Peter O'Rourke<br>Statistical Unit<br>QIMR Berghofer Medical Research Institute<br>Herston, QLD 4006<br>Australia<br>Tel: +61 (0)7 3845 3579<br>Email: peter.orourke@qimrberghofer.edu.au
Trial Sponsor and Local Australian Sponsor:	QIMR Berghofer Medical Research Institute<br>300 Herston Rd, Herston, QLD 4006<br>Tel: +61 (0)7 3362 0222
Authorised Sponsor Signatory	Prof. David Whiteman<br>Deputy Director<br>QIMR Berghofer Medical Research Institute<br>Tel: +61 (0)7 3362 0279
Project Manager	Dr. Rebecca Watts<br>QIMR Berghofer Medical Research Institute<br>300 Herston Rd, Herston, QLD 4006<br>Tel: +61 (0)7 3845 3686
Data Manager	Ria Woo<br>QIMR Berghofer Medical Research Institute<br>300 Herston Rd, Herston, QLD 4006<br>Tel: +61 (0)7 3362 0350
Clinical Study Report Writer	Dr. Adam Potter<br>QIMR Berghofer Medical Research Institute<br>300 Herston Rd, Herston, QLD 4006<br>Tel: +61 (0)7 3362 0419
Sponsors Monitors	Clinical Network Services (CNS) Pty Ltd<br>Level 4, 88 Jephson St<br>Toowong QLD 4066, Australia<br>Tel: +61 (0)7 3719 6000
Institutional Ethics Committee	QIMR Berghofer Medical Research Institute Human Research Ethics Committee (QIMR Berghofer-HREC; EC00278)<br>Locked Bag 2000, Royal Brisbane and Women's Hospital, Brisbane, QLD 4029<br>Tel: +61 (0)7 3362 0117
Clinical Study Centre:	Q-Pharm Pty Limited<br>Level 5, 300C Herston Road and

	Level 6, Block 8, Royal Brisbane and Women's Hospital<br>Herston QLD 4006<br>Tel: +61 (0)7 3845 3636
Site for preparation of malaria challenge agent	Q-Gen Cell Therapeutics<br>300 Herston Rd, Herston, QLD 4006<br>Tel: +61 (0)7 3845 3851
Clinical Safety Laboratory	Sullivan Nicolaides Pathology Central Laboratory (SNP)<br>24 Hurworth Street<br>Bowen Hills, QLD 4006<br>Australia<br>Tel: +61 (0)7 3377 8782
Laboratory for parasitaemia measurements	Queensland Paediatric Infectious Diseases Laboratory<br>(Q-PID), SASVRC<br>Level 8, Centre for Children's Health Research<br>62 Graham Street, South Brisbane, QLD 4101, Australia<br>Tel: +61 (0)7 3069 7464
Laboratory for drug concentration measurements	Brett McWhinney<br>Analytical Chemistry Unit<br>Department of Chemical Pathology<br>Pathology Queensland<br>Level 3, Block 7<br>Royal Brisbane and Women's Hospital<br>Brisbane QLD 4029
Location of Trial Master File	QIMR Berghofer Medical Research Institute<br>Regulatory Affairs<br>300 Herston Rd, Herston, QLD 4006<br>Tel: +61 (0)7 3362 0433

16.1.5 Signature of Principal Investigator and Sponsor's Signatory

I have read the QP16C14 Clinical Study Report (final version, dated 12 December 2018) and confirm that to the best of my knowledge it accurately describes the conduct and results of the study.

PRINCIPAL INVESTIGATOR

Prof. James McCarthy

300 Herston Road, Herston 4006
QLD, Australia

12/12/18
Date

Signature

SPONSOR

Dr. Rebecca Watts

Project Manager

QIMR Berghofer Medical Research Institute

12/12/2018
Date

Signature

16.1.6 List of investigational product batch numbers

P. falciparum K13 malaria challenge agent

MCI-045, expiry 24 May 2017 11:11 am (Subject R001)

MCI-046, expiry 14 June 2017 11:23 am (Subject R002)

16.1.7 Randomisation scheme and codes

No randomisation was performed in this study.

16.1.8 Audit certificates

QP16C14: K13 Pilot Clinical Study Report Appendices

This study had not been audited at the time of completed of the Clinical Study Report.

16.1.9 Documentation of statistical methods

Statistical methods are described in the Study Protocol, no formal Statistical Analysis Plan was generated for this study.

16.1.10 Documentation of inter-laboratory standardisation methods and laboratory QA procedures

Method development and validation for Artesunate and Dihydro Artemisinin (DHA)

**Study title**	Development and validation of a method for the determination of Artesunate and Dihydro Artemisinin (DHA), in human plasma samples by LC-MS/MS
**Study no**	JIRA # PROJ-279
**Study facility**	Analytical Chemistry Unit (HPLC, LC MS/MS) RBWH Central Laboratory Pathology QLD
**Sponsor**	Prof James McCarthy, Queensland Institute of Medical Research (QIMR)
**Staff involved**	Dr Kobus Ungerer**,** Director of Chemical Pathology<br>Brett McWhinney, Supervising Scientist, HPLC section<br>Leanne Foyn, Scientist, Analytical Chemistry Unit
**Starting date**	February 2017
**Completion date**	April 2017

Table of Contents

1. Study synopsis 3

1.1 Schedule 4

2. Introduction 4

3. Reagents and materials 4

3.1 Reference items 4

3.2 Internal standard 5

3.3 Blank plasma 5

3.4 Chemicals and equipment 5

4. Analytical method 6

4.1 Preparation of calibration and quality control sample 6

4.2 Preparation of the internal standard 7

4.3 Storage conditions 7

4.4 Extraction method 7

4.5 HPLC-MS/MS conditions 7

4.6 Protein precipitation solution evaluation 9

4.7 References 9

5. Chromatograms 10

6. Certificates of Analysis 14

6.1 Artesunate certificate 14

6.2 Dihydro Artemisinin certificate 15

6.3 Artesunate-d4 certificate 16

6.4 Dihydro Artemisinin-d3 certificate 17

7. Data 18

7.1Precision 18

7.2 Internal standard, Artesunate and DHA post-extract stability 20

7.3 QC short term and freeze-thaw cycle stability 22

7.4 Carry-over study 22

7.5 Extraction comparison and recovery 23

7.6 Linearity 23

8. Recommendation 25

Sign off page 25

1. Study synopsis

Sponsor	Dept of Chemical Pathology, Pathology Queensland, Level 3, Block 7, RBWH
Reference item(s)	Artesunate and Dihydro Artemisinin
Internal standard(s)	Artesunate-d4 and Dihydro Artemisinin-d3
SBQ study no	
Study title	Method development and validation for Artesunate and Dihydro Artemisinin (DHA)
Specimens	spiked samples
Species	human
Biological matrix	plasma
Sensitivity of the assay	1 to 1000 µg/L for Artesunate and Dihydro Artemisinin for 5 µL injection
Concentration of the ISTD	200 µg/L of both Artesunate-d4 and Dihydro Artemisinin-d3
Intra-run Precision (CV)	5.2% to 7.5% (n=20) for Artesunate over 3 concentration levels<br>5.2% to 8.5% (n=20) for DHA over 3 concentration levels
Inter-run Precision (CV)	7.3% to 10.9% (n=7) for Artesunate over 3 concentration levels<br>3.8% to 9.3% (n=7) for DHA over 3 concentration levels
Accuracy	98.4% to 104.8% (n=5) for Artesunate<br>98.2% to 105.1% (n=5) for DHA
Post-preparative stability	stable for at least 24 hours at 10±3°C
Freeze-and-thaw stability	stable after at least three freeze and thaw cycles
Carry-over	<0.01 % for Artesunate after the highest calibration sample<br>0.01% for DHA after the highest calibration sample
Recovery of the analyte	
Overall-recovery of the ISTD	
Specificity	no observed interference

1.1 Schedule

Experimental starting date	February 2017
Experimental completion date	April 2017

2. Introduction

The purpose of this study was the validation of a method for the quantification of Artesunate and Dihydroartemisinin (DHA) in human plasma samples by LC-MS/MS. The heated electrospray interface (ESI) was used as ion source in positive ion mode. Artesunate, DHA and the internal standards Artesunate-d4 and DHA-d3 were measured in selected reaction monitoring mode. The plasma samples were precipitated with three volume equivalents of acetonitrile containing the internal standard artesunate-d4 and DHA-d3. After protein precipitation, the samples were vortexed then centrifuged, and the 96 well plate was transferred to the Autosampler. An aliquot of 5 µL of the sample was injected onto the UPLC system.

The following calibration range was applied:

1.00 to 1000 µg/L of Artesunate and DHA in human plasma

As part of this validation, the following parameters were investigated:

- Linearity and regression model
- Accuracy and precision
- Specificity of the method for the analyte and the internal standard
- Specificity of the method for the analyte and the internal standard in different protein precipitation solutions
- Stability of the analytes in extracted samples
- Overall-recovery of the internal standard
- Carry-over of the method

3. REAGENTS AND MATERIALS

3.1 Reference items

**Name**	**Artesunate**
Batch	2-ARP-152-1
Supplier	Toronto Research Chemical Inc. (2 Brisbane Road, Toronto, ON, Canada)
Storage	at -20°C Freezer.
Retest	May, 2018
Assay purity	98%
Molecular formula, base	$C_{19}H_{28}O_8$
Molecular weight, base	384.42
CoA date	24-05-2013

**Name**	**Dihydroartemisinin**
Batch	4-EOD-163-1
Supplier	Toronto Research Chemical Inc. (2 Brisbane Road, Toronto, ON, Canada)
Storage	at -20°C Freezer
Retest	October, 2021
Assay purity	95%
Molecular formula, base	$C_{15}H_{24}O_5$
Molecular weight, base	284.35
CoA date	05-10-2016

3.2 Internal standard

**Name**	**Artesunate-d4**
Batch	6-DHL-62-4
Supplier	Toronto Research Chemical Inc. (2 Brisbane Road, Toronto, ON, Canada)
Storage	at -20°C
Retest	February, 2022
Purity	97%
Molecular formula, base	$C_{19}H_{24}D_4O_8$
Molecular weight, base	388.45
CoA	03-02-2017

**Name**	**d3-Dihydroartemisinin**
Batch	25-GHZ-173-1
Supplier	Toronto Research Chemical Inc. (2 Brisbane Road, Toronto, ON, Canada)
Storage	at -20°C, Under Inert Atmosphere
Retest	October, 2017
Purity	98%
Molecular formula, base	$C_{15}H_{21}D_3O_5$
Molecular weight, base	287.37
CoA	10-10-2014

The characterization of the reference items was performed if not otherwise stated according to the quality standards mentioned in the certificate of analysis.

3.3 Blank plasma

The following source was used for the preparation of spiked samples:

Human plasma

3.4 Chemicals and equipment

All chemicals and equipment used during the analysis of the study samples were controlled or validated according to internal SOPs, unless otherwise stated.

Chemicals

Methanol	LiChromSolv	Merck, Kilsyth Victoria, Australia
Acetonitrile	LiChromSolv	Merck, Kilsyth Victoria, Australia
Water 18 mega ohm		SG Water Nashua, NH, USA
Formic acid	Sigma Aldrich	Sydney NSW, Australia

Apparatus

Micro balance	MT-5	Mettler-Toledo GmbH, Germany
Pipette	Research and Reference	Eppendorf AG, Hamburg, Germany
Centrifuge	16R Hereaus	Thermo Fisher Scientific,Australia
Vortexer	TallBoy Multi-tube	Henry Troemner, USA

Instrumentation

UPLC Pump	Acquity UPLC binary pumps Waters Corporation, Milford, MA USA
Autosampler	Acquity UPLC Autosampler Waters Corporation, Milford, MA USA
Mass spectrometer	Premier XE Waters Corporation, Milford, Massachusetts, USA

Software

Chromatography	MassLynx Version 4.1	Waters Corporation, Milford, MA USA

4. ANALYTICAL METHOD

4.1 Preparation of calibration samples and quality control samples

For the preparation of CAL samples, Artesunate and DHA were dissolved in methanol to a concentration of 0.500 g/L each for the combined solution. For the preparation of QC samples, a second solution (prepared by a different analyst) with a concentration of 0.500 g/L was used. Working solutions were prepared from a mixture by serial dilutions in methanol to concentrations 20 times higher than the corresponding concentrations in matrix. The concentrations were calculated under consideration of purity where applicable. The concentrations of the CAL samples were the following:

Working Standard Range (in blank plasma):
Artesunate and DHA (µg/L)

Std 1	0
Std 2	2
Std 3	5
Std 4	20
Std 5	100
Std 6	500
Std 7	700
Std 8	1000

Quality Control (in blank plasma):

Artesunate and DHA µg/L

QC L	15
QC M	80
QC H	800

4.2 Preparation of the internal standard

For the preparation of the internal standard solution, Artesunate-d4 and DHA-d3 were dissolved in methanol to a concentration of 0.01 g/L and 0.005 g/L respectively. Further dilutions to a final concentration of 200 µg/L for both Artesunate-d4 and DHA-d3 were combined with acetonitrile.

4.3 Storage conditions

CAL and QC samples as well as stock solutions and their dilutions were stored at -25±5°C.

4.4 Extraction Method:

1. Pipette 100 µL of STD, QC or sample into 2 mL deep 96 well plate
2. Add 300 µL of IS, 200 µg/L Artesunate-d4 and DHA-d3 in Acetonitrile
3. Place mat cap on top of plate and vortex 2 min
4. Centrifuge 10 min at 3500 rpm
5. Place into Sample Organizer
6. Inject 5 µL

4.5 HPLC-MS/MS conditions

The quantification of Artesunate and DHA was performed by UPLC column separation with reversed-phase chromatography followed by detection with triple-stage quadrupole MS/MS in the selected reaction monitoring mode.

Liquid chromatography

Mobile Phase A:	2mM Ammonium Acetate in water + 0.1% Formic Acid
Mobile Phase B:	2mM Ammonium Acetate in Methanol + 0.1% Formic Acid
Column:	Acquity UPLC BEH C_{18} 1.7µm 2.1x50mm column Part # 186002350
Precolumn:	Acquity BEH C18 1.7µm VanGuard Pre-column 2.1 x 5 mm Part # 186003975
Column Temp:	45° C

Instrument Conditions:

MRM Function

COMPOUND	MRM FUNCTION	DWELL (Sec)	CONE (V)	COLLISION ENERGY (eV)
**Artesunate Quantifier**	402.2>267.3	0.02	10	15
**Artesunate Qualifier**	402.2>163.0	0.02	10	20
**DHA Quantifier**	267.3>145.0	0.02	15	15
**DHA Qualifier**	367.3>163.1	0.02	15	10

**Artensunate-d4**	406.3>267.2	0.02	10	15
**DHA-d3**	270.3>148	0.02	15	15

MS Conditions

Source ES+

Capillary (kV)	0.80
Cone (V)	15
Extractor (V)	5
RF Lens (V)	0.2
Source Temperature (°C)	120
Desolvation Temperature (°C)	425
Desolvation Gas Flow (L/Hr)	900
Cone Gas Flow (L/Hr)	20

Analyser

LM 1 Resolution	14.0
HM 1 Resolution	14.0
Ion Energy 1	0.2
Entrance	0
Collision	10
Exit	0.0
LM 2 Resolution	14.0
HM 2 Resolution	13.0
Ion Energy 2	0.6
Multiplier	800
Collision Cell Gas flow (mL/min)	0.30

UPLC Conditions

Sample Manager

General

Sample Loop Option	Partial loop with needle overfill
Wash Solvents	Weak 1000µL<br>Strong 500µL
Loop Offline	Enable
Temperature	Column 45.0 ± 2.0°C<br>Sample 10.0 ± 2.0°C
Run Time	2.0 minutes
Needle depth	6.0 mm

Gradient Table

TIME (mins)	FLOW RATE (mL/min)	%A	%B	CURVE
Initial	0.400	20	80	Initial
1.00	0.400	12	88	6
1.01	0.400	2	98	1
1.75	0.400	20	80	11

4.6 Protein precipitation solution evaluation

A 96 well plate was used to assess protein precipitation solutions using 100% Methanol, 100% Acetonitrile and 0.1% Formic acid in Acetonitrile.

Each protein precipitation solution was used to extract a set of standards for the assay; the internal standard peak's area was then used to evaluate which protein precipitation solution was most effective in yielding the greatest recovery.

The following procedure was followed.

1. 100 µL of sample was added into the wells
2. 300 µL of IS was then added in the wells
3. The plate was capped and vortexed at maximum speed for 2 minutes
4. The plate was then centrifuged at 3500rpm for 10 minutes after which it was placed in the Autosampler.

Observations

After observing the internal standard area recovery of the three solutions, it was decided that 100% Acetonitrile had the best recovery and was therefore selected for the progression of the assay development.

4.7 References

1. Development and validation of a liquid chromatography and ion spray tandem mass spectrometry method for the quantification of artesunate, artemether and their major metabolites dihydroartemisinin and dihydroartemisinin-glucuronide in sheep plasma. Duthaler U, Keiser J, Huwyler J.

   J. Mass. Spectrom. 2011, 46, 172-181. DOI 10.1002. Epub: 2017 Feb 23

2. A simplified liquid chromatography-mass spectrometry assay for artesunate and dihydroartemisinin, its metabolite, in human plasma. Teja-Isavadharm P, Siriyanonda D, Siripokasupkul R, Apinan R, Chanarat N, Lim A, Wannaying S, Saunders D, Fukuda M, Miller R, Weina P, Melendez V.

   Molecules, 2010, 15, 8747-8768. DOI 10.3390. Epub 2017 Jan 23

5. Chromatograms

Below are the chromatograms for both Artesunate and DHA for the following sample types; Blank plasma with acetonitrile, blank plasma with internal standard, the low level of QC material and the highest standard for both assays.

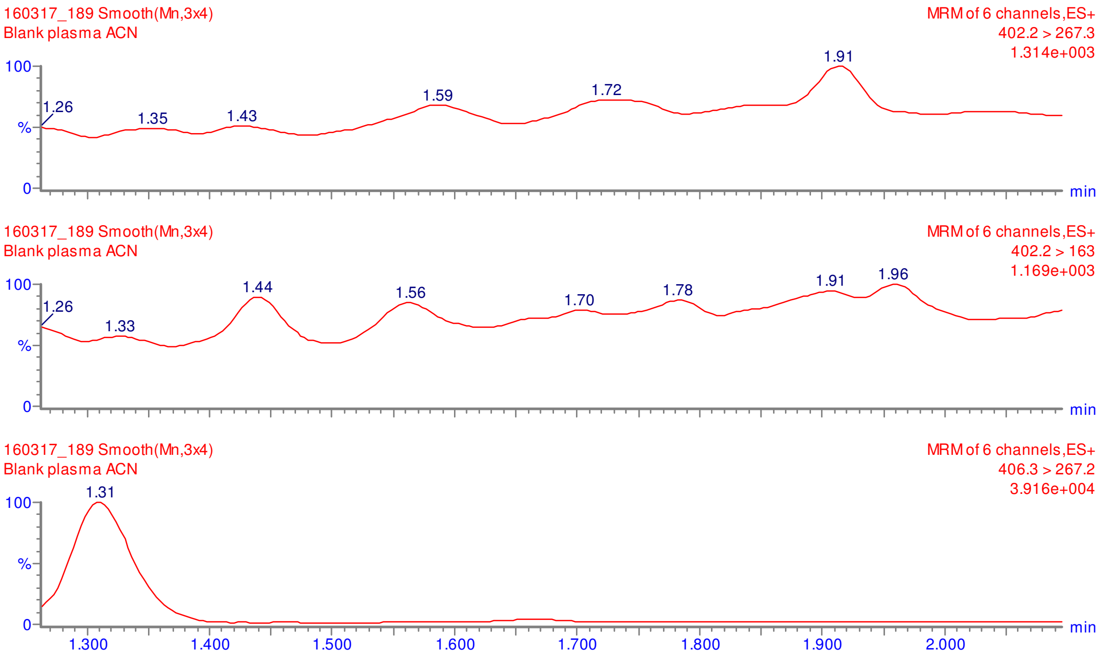

Figure 1: showing the chromatogram, primary and secondary channels as well as the internal standard peak, for Artesunate in a blank plasma sample with only 100% ACN added.

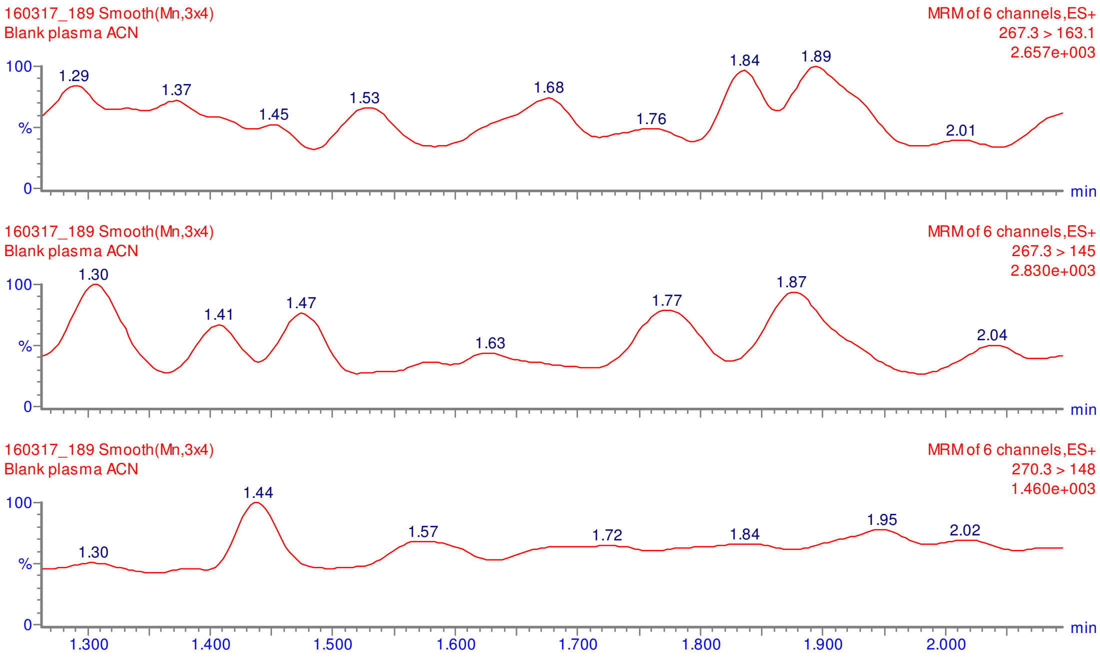

Figure 2: showing the chromatogram, primary and secondary channels as well as the internal standard peak, for DHA in a blank plasma sample with only 100% ACN added.

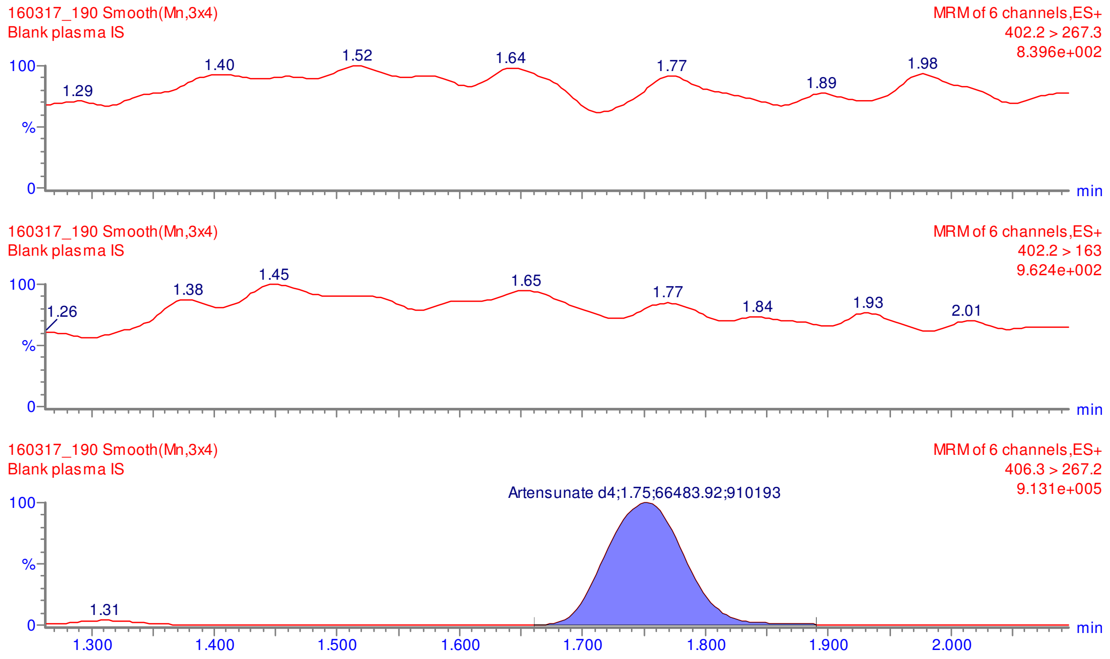

Figure 3: showing the chromatogram, primary and secondary channels as well as the internal standard peak, for Artesunate in a blank plasma sample with internal standard added.

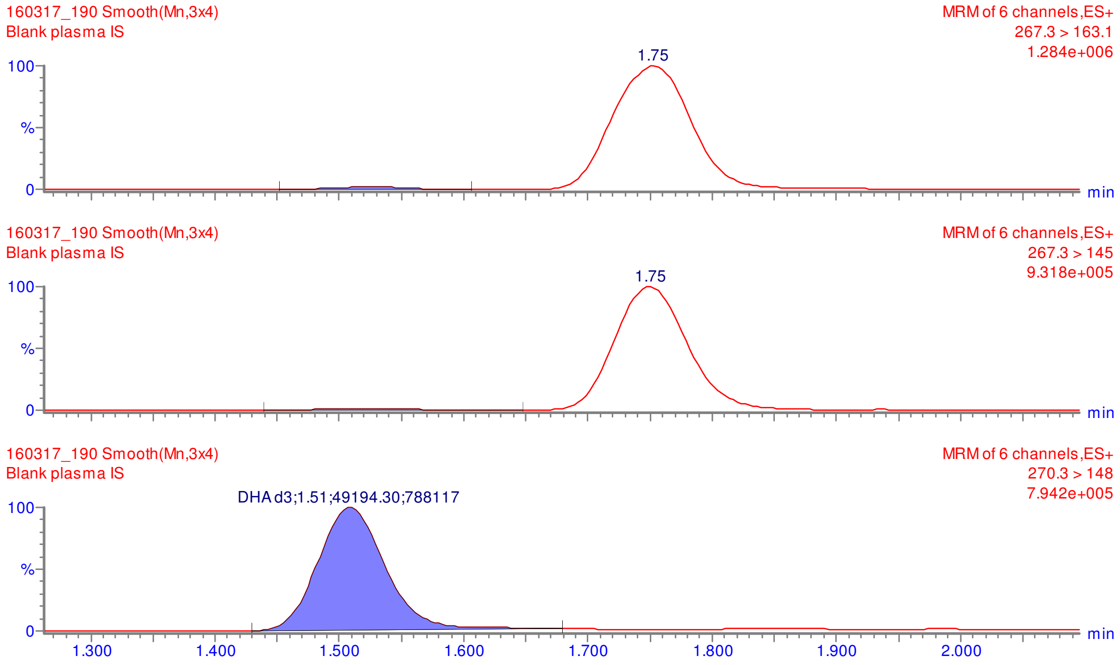

Figure 4: showing the chromatogram, primary and secondary channels as well as the internal standard peak, for DHA in a blank plasma sample with internal standard added.

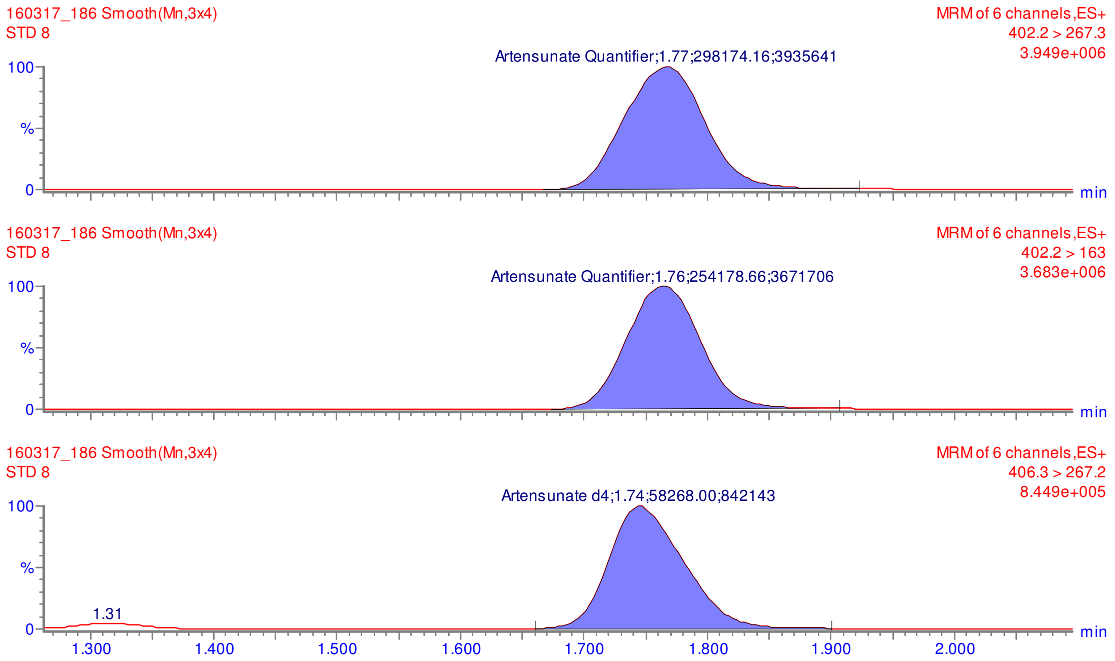

Figure 5: showing the chromatogram, primary and secondary channels as well as the internal standard peak, for Artesunate in an extracted Standard 8 sample with internal standard added.

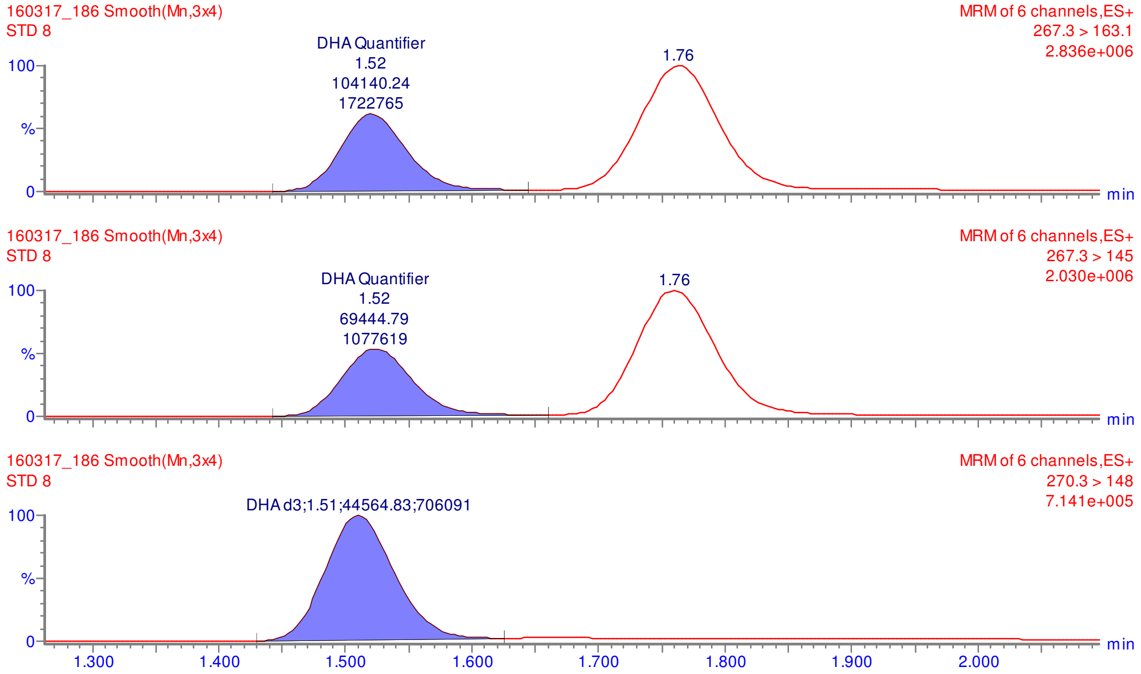

Figure 6: showing the chromatogram, primary and secondary channels as well as the internal standard peak, for DHA in an extracted Standard 8 sample with internal standard added.

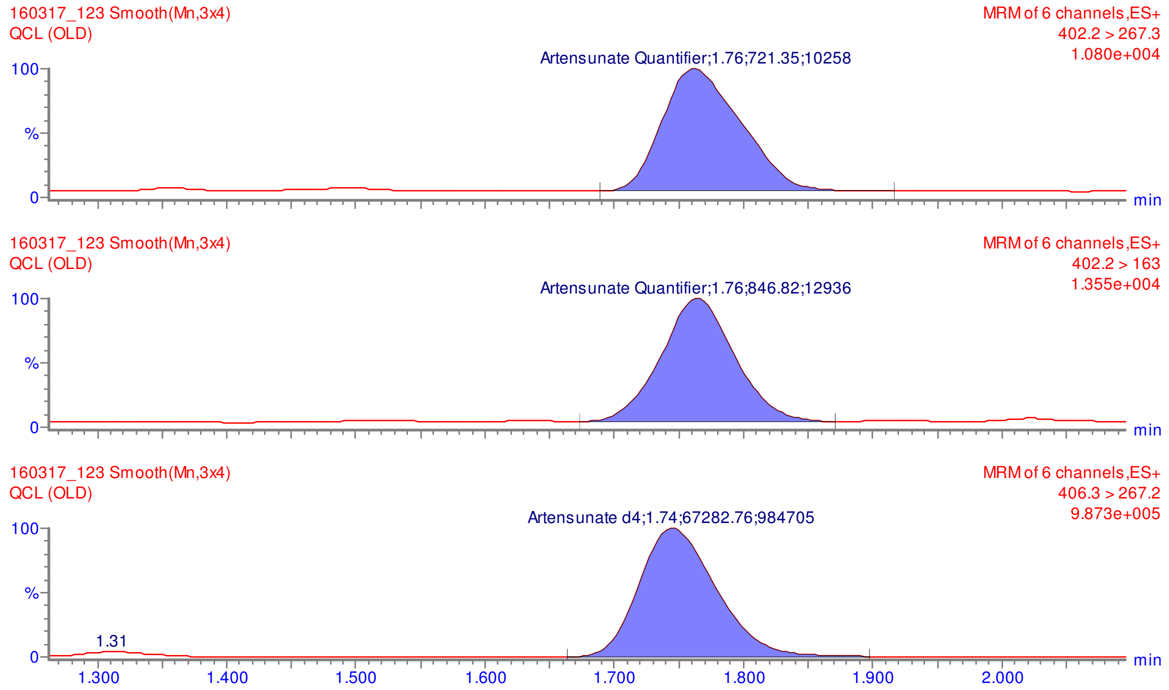

Figure 7: showing the chromatogram, primary and secondary channels as well as the internal standard peak, for Artesunate in an extracted QC 1 sample with internal standard added.

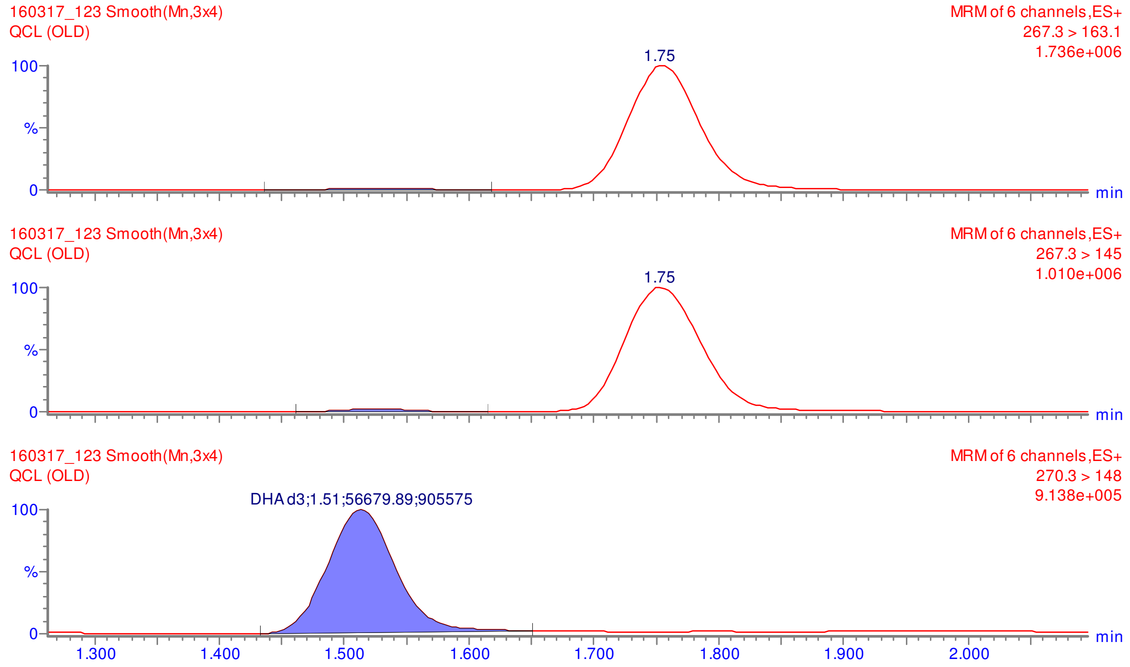

Figure 8: showing the chromatogram, primary and secondary channels as well as the internal standard peak, for DHA in an extracted QC 1 sample with internal standard added.

6. Certificates of Analysis

6.1 Artesunate certificate

Toronto Research Chemicals
products for innovative research

CERTIFICATE OF ANALYSIS

2 Brisbane Road, Toronto, ON. M3J 2J8 Canada Tel: (416) 665-9696 Fax: (416) 665-4439 E-mail: orders@trc-canada.com Website: www.trc-canada.com

1. Identification

CAS Number:
88495-63-0

Catalogue Number:
A777800

Product:
Artesunate

Synonyms:
Butanedioic Acid Mono(3R,5aS,6R,8aS,9R,10R,12R,12aR)-decahydro-3,6,9-trimethyl-3,12-epoxy-12H-pyrano[4,3-j]-1,2-benzodioxepin-10-yl] Ester; Artesunic Acid;

Structure:

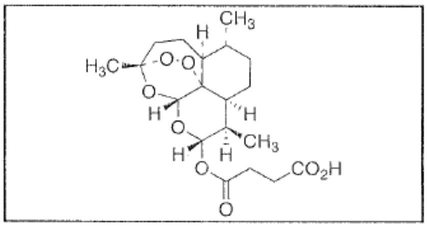

Molecular Formula:
$C_{19}H_{28}O_8$

Molecular Weight:
384.42

Source of Product:
N/A

2. Analytical Information

Lot Number:
2-ARP-152-1

Melting Point:
136 - 138°C

Boiling Point:
N/A

Atmosphere:
Air

Appearance of Product:
White Solid

Solubility
DMSO, Methanol

Method for Determining Identity:
^1H NMR (DMSO-d_6) and MS

Stability
Not Detemined

Purity:
98%

Long Term Storage Condition:
-20°C Freezer

Additional Information:
TLC Conditions: SiO_2: Dichloromethane : Methanol = 6 : 1; Visualized with AMCS; Single Spot, Rf = 0.7.
^1H NMR and MS conform to structure.

Philip Chan, Head of Quality Assurance

QC Test Date
May 24, 2013

Retest Date
May 22, 2018

PM Separations
Ph: +61 7 3390 1096
customerservice@pmsep.com.au
www.pmsep.com.au

6.2 Dihydro Artemisinin certificate

Toronto Research Chemicals
products for innovative research

CERTIFICATE OF ANALYSIS

2 Brisbane Road, Toronto, ON. M3J 2J8 Canada Tel: (416) 665-9696 Fax: (416) 665-4439 E-mail: orders@trc-canada.com Website: www.trc-canada.com

1. Identification

CAS Number:
71939-50-9

Catalogue Number:
D448360

Product:
Dihydro Artemisinin

Synonyms:
(3R,5aS,6R,8aS,9R,10S,12R,12aR)-Decahydro-3,6,9-trimethyl- 3,12-epoxy-12H-pyrano[4,3-j]-1,2-benzodioxepin-10-ol;β-Dihydroartemisinin; Alaxin; Cotecxin; Cotexin; DHQHS 2; Dihydroartemisinine; Dihydroqinghaosu; USP Artemether Related Compound A

Structure:

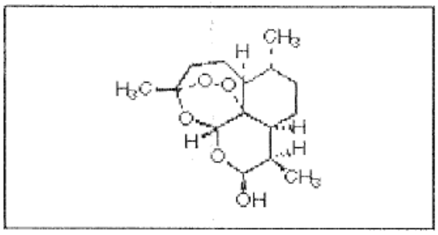

Molecular Formula:
$C_{15}H_{24}O_5$

Molecular Weight:
284.35

Source of Product:
N/A

2. Analytical Information

Lot Number:
4-EOD-163-1

Melting Point:
143 - 145°C

Boiling Point:
N/A

Atmosphere:
Air

Appearance of Product:
Off-White Solid

Solubility
Chloroform (Slightly), Methanol (Slightly)

Method for Determining Identity:
^1H NMR ($CDCl_3$) and MS

Stability
Not Determined

Purity:
95%

Long Term Storage Condition:
-20°C Freezer

Additional Information:
TLC Conditions: SiO_2; Dichloromethane : Methanol = 9 : 1; Visualized with $KMnO_4$; Single Spot, Rf = 0.40.
^1H NMR and MS conform to structure.

Philip Chan, Head of Quality Assurance

QC Test Date
October 5, 2016

Retest Date
October 3, 2021

PM Separations
Ph. +61 7 3390 1096
customerservice@pmsep.com.au
www.pmsep.com.au

6.3 Artesunate-d4 certificate

Toronto Research Chemicals
products for innovative research

CERTIFICATE OF ANALYSIS

2 Brisbane Road, Toronto, ON. M3J 2J8 Canada Tel: (416) 665-9696 Fax: (416) 665-4439 E-mail: orders@trc-canada.com Website: www.trc-canada.com

1. Identification

CAS Number:
1316753-15-7

Catalogue Number:
A777803

PM Separations
Ph: +61 7 3390 1096
customerservice@pmsep.com.au
www.pmsep.com.au

Product:
Artesunate-d4

Synonyms:
Butanedioic Acid Mono(3R,5aS,6R,8aS,9R,10R,12R,12aR)-decahydro-3,6,9-trimethyl-3,12-epoxy-12H-pyrano[4,3-j] -1,2-benzodioxepin-10-yl] Ester-d4; Artesunic Acid-d4;

Structure:

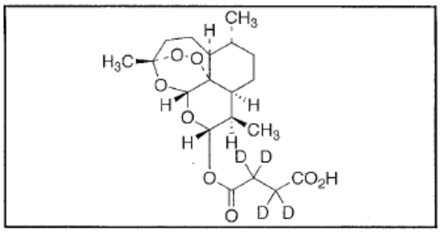

Molecular Formula:
$C_{19}H_{24}D_4O_8$

Molecular Weight:
388.45

Source of Product:
Synthetic

2. Analytical Information

Lot Number:
6-DHL-62-4

Melting Point:
130 - 132°C

Boiling Point:
N/A

Atmosphere:
Air

Appearance of Product:
White Solid

Solubility
Chloroform (Slightly), Methanol (Slightly)

Method for Determining Identity:
^1H NMR ($CDCl_3$) and MS

Stability
Not Determined

Purity:
Chemical Purity: 97%
Isotopic Purity: 97.9%

Long Term Storage Condition:
-20°C Freezer

Additional Information:
TLC Conditions: SiO_2; Dichloromethane : Methanol = 9 : 1; Visualized with UV and AMCS; Single Spot, Rf = 0.60.
^1H NMR and MS conform to structure.
Elemental Analysis: (Found) %C: 57.96, %H: 7.13; (Calculated) %C: 58.75, %H: 7.27
Normalized Intensity: d_0 = 0.27%, d_1 = 0.45%, d_2 = 1.08%, d_3 = 3.96%, d_4 = 94.24%

Philip Chan, Head of Quality Assurance

QC Test Date
February 3, 2017

Retest Date
February 1, 2022

6.4 Dihydro Artemisinin-d3 certificate

Toronto Research Chemicals
products for innovative research

CERTIFICATE OF ANALYSIS

2 Brisbane Road, Toronto, ON. M3J 2J8 Canada Tel: (416) 665-9696 Fax: (416) 665-4439 E-mail: orders@trc-canada.com Website: www.trc-canada.com

1. Identification

CAS Number:
176774-98-4

Catalogue Number:
D448362

Product:
Dihydro Artemisinin-d3

Synonyms:
(3R,5aS,6R,8aS,9R,10S,12R,12aR)-Decahydro-3,6,9-trimethyl- 3,12-epoxy-12H-pyrano[4,3-j]-1,2-benzodioxepin-10-ol-d3; β-Dihydroartemisinin-d3; Alaxin-d3; Cotecxin-d3; Cotexin-d3; DHQHS 2-d3; Dihydroartemisinine-d3; Dihydroqinghaosu-d3;

Structure:

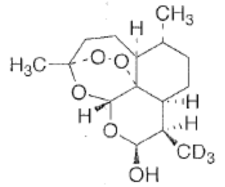

Molecular Formula:
$C_{15}H_{21}D_3O_5$

Molecular Weight:
287.37

Source of Product:
Synthetic

2. Analytical Information

Lot Number:
25-GHZ-173-1

Melting Point:
139.5 - 142.0°C

Boiling Point:
N/A

Atmosphere:
Inert Gas

Appearance of Product:
White Solid

Solubility
Chloroform

Method for Determining Identity:
^{1}H NMR ($CDCl_3$) and MS

Stability
Not Determined

Purity:
Chemical Purity: 98%
Isotopic Purity: 98.0%

Long Term Storage Condition:
-20°C Freezer, Under Inert Atmosphere

Additional Information:
TLC Conditions: SiO_2; Dichloromethane : Methanol = 9 : 1; Visualized with AMCS and $KMnO_4$; Single Spot, Rf = 0.60.
^{1}H NMR and MS conform to structure.
Elemental Analysis: (Found) %C: 62.47, %H: 8.95; (Calculated) %C: 62.69, %H: 8.42
Normalized Intensity: d_0 = 0.04%, d_1 = 0.00%, d_2 = 5.80%, d_3 = 94.16%
Specific Rotation: +145.1° (c = 0.20, Chloroform)

Philip Chan, Head of Quality Assurance

QC Test Date
October 10, 2014

Retest Date
October 8, 2017

PM Separations
Ph: +61 7 3390 1096
customerservice@pmsep.com.au
www.pmsep.com

7. DATA

7.1 Precision

Intra-run precision was determined by extracting 20 sets of QC 1, QC 2 and QC 3 samples and subsequently calculating the mean and coefficient of variation for both Artesunate and DHA. Inter-run precision was established by averaging the concentrations acquired for QC 1, QC 2 and QC 3 over multiple extractions on various days.

**Artesunate Primary channel (nmol/L)**				
**Intra Run Precision**		Date : 09/03/2017 – 30/03/2017		
**QC ID**	**n**	**Mean**	**SD**	**%CV**
QC 1	20	14.25	1.07	7.5
QC 2	20	76.00	5.16	6.8
QC 3	20	779.98	43.66	5.6

**Artesunate Secondary channel (nmol/L)**				
**Intra Run Precision**		Date : 09/03/2017 – 30/03/2017		
**QC ID**	**n**	**Mean**	**SD**	**%CV**
QC 1	20	13.59	1.16	8.5
QC 2	20	72.40	5.18	7.2
QC 3	20	732.11	40.28	5.5

**Inter Run Precision** (Artesunate)		Date : 09/03/2017 - 30/03/2017		
**QC ID**	**n**	**Mean**	**SD**	**%CV**
QC 1	7	15.41	1.68	10.9
QC 2	7	67.94	7.31	10.8
QC 3	7	811.80	58.97	7.3

DHA Primary channel (nmol/L)				
Intra Run		Date : 09/03/2017 – 30/03/2017		
**QC ID**	**n**	**Mean**	**SD**	**%CV**
QC 1	20	14.78	1.25	8.5
QC 2	20	84.57	6.13	7.2
QC 3	20	853.39	50.70	5.9

DHA Secondary channel (nmol/L)				
Intra Run		Date : 09/03/2017 – 30/03/2017		
**QC ID**	**n**	**Mean**	**SD**	**%CV**
QC 1	20	14.72	1.15	7.8
QC 2	20	83.56	6.32	7.6
QC 3	20	813.58	42.05	5.2

Inter Run Precision (DHA)		Date : 09/03/2017- 30/03/2017		
**QC ID**	**n**	**Mean**	**SD**	**%CV**
QC 1	7	16.70	1.55	9.3
QC 2	7	87.70	7.32	8.3
QC 3	7	815.00	31.02	3.8

7.2 Internal standard, Artesunate and DHA Post-Extract Stability

Post extraction stability data was obtained by extracting 10 sets of QC 1, QC 2 and QC 3, injecting the samples, and then allowing the plate to sit in the sample manager at 10°C. Following 12 hours, the samples were re-injected. The deviation percentage was then calculated from both sets of data for Artesunate and DHA.

Artesunate Extract Stability over 12hr (nmol/L)			
**Sample ID**	**Injection 1**	**Injection 2 (12 hr post)**	**% Deviation**
Sample 1	11.9	14.9	-25.8
Sample 2	16.7	13.6	18.8
Sample 3	15.8	14.3	9.5
Sample 4	14.4	9.8	32.2
Sample 5	14.6	14.8	-1
Sample 6	13.8	14.6	-5.4
Sample 7	12.9	16.4	-27.5
Sample 8	11.2	11	2
Sample 9	12	12.2	-1.5
Sample 10	12	11.9	0.3
Sample 11	56.1	62.3	-11
Sample 12	74.6	63.6	14.8
Sample 13	62.9	68.8	-9.5
Sample 14	67.9	65.3	3.9
Sample 15	60.9	64.1	-5.3
Sample 16	69.7	55	21.2
Sample 17	58.3	69.4	-19
Sample 18	78	75.1	3.7
Sample 19	65.9	61.5	6.8
Sample 20	57.7	64.7	-12
Sample 21	813.8	729.4	10.4
Sample 22	822.9	701.2	14.8
Sample 23	612.1	725.6	-18.5
Sample 24	755.5	772.9	-2.3
Sample 25	670	650.4	2.9
Sample 26	803.6	716.7	10.8
Sample 27	920.9	678.3	26.3
Sample 28	781	710.6	9
Sample 29	782.2	741.6	5.2
Sample 30	657.6	804.5	-22.3
**Average**			**1**

DHA Extract Stability over 12hr (nmol/L) Primary channel			
**Sample ID**	**Injection 1**	**Injection 2 (12 hr post)**	**% Deviation**
Sample 1	14.6	19.8	-35.4
Sample 2	13.2	19.4	-47.5
Sample 3	13.2	23.5	-78.0
Sample 4	13.2	22.7	-71.8
Sample 5	18.9	25.5	-35.0
Sample 6	19.4	21.7	-12.2
Sample 7	15.9	21.7	-36.4
Sample 8	19.2	17.8	7.4
Sample 9	16.4	13.0	20.4
Sample 10	17.1	26.8	-57.0
Sample 11	93.7	88.4	5.6
Sample 12	100.4	116.6	-16.2
Sample 13	100.4	93.3	7.0
Sample 14	87.5	91.0	-4.0
Sample 15	102.3	88.1	13.8
Sample 16	70.0	79.5	-13.6
Sample 17	101.0	98.9	2.1
Sample 18	100.6	80.5	20.0
Sample 19	105.9	83.7	20.9
Sample 20	91.5	101.2	-10.6
Sample 21	858.3	911.5	-6.2
Sample 22	947.5	1023.8	-8.1
Sample 23	949.9	1021.4	-7.5
Sample 24	874.5	967.8	-10.7
Sample 25	803.0	1025.0	-27.6
Sample 26	849.3	911.7	-7.3
Sample 27	893.4	919.4	-2.9
Sample 28	957.9	915.2	4.5
Sample 29	844.0	909.7	-7.8
Sample 30	957.0	1013.2	-5.9
**Average**			**-13.3**

7.3 Short term and Freeze-Thaw stability

The stability of QC1, QC 2 and QC 3, for both Artesunate and DHA, was established by thawing and freezing 3 sets of the samples in cycles. Therefore set 1 was thawed once then extracted, set 2 was thawed twice then extracted and set 3 was thawed three times then extracted. The deviation from the original results was then calculated.

**ART QC Stability over 3 freeze-thaw cycles (nmol/L)**					
**Sample ID**	**Thaw 1**	**Thaw 2**	**% Deviation (Thaw 1 to Thaw 2)**	**Thaw 3**	**% Deviation (Thaw 1 to Thaw 3)**
QC 1	15.66	14.3	-8.7	16.08	2.7
QC 2	74.75	79.89	6.9	78.94	5.6
QC 3	824.47	819.81	-1.0	803.13	-3.0
**Average**			**-0.9**		**1.8**
**DHA QC Stability over 3 freeze-thaw cycles (nmol/L)**					
**Sample ID**	**Thaw 1**	**Thaw 2**	**% Deviation (Thaw 1 to Thaw 2)**	**Thaw 3**	**% Deviation (Thaw 1 to Thaw 3)**
QC 1	15.77	15.33	-2.8	14.67	-7.0
QC 2	80.55	75.64	-6.1	86.29	7.1
QC 3	820.89	836.85	1.9	821.53	0.1
**Average**			**-2.6**		**0.0**

The above tables demonstrate that the sets of QC for this assay are stable after three cycles of freezing and thawing, which meets acceptable criteria.

7.4 Carry-Over Study

The carry-over within the system was assessed by injecting water (n=5) after a linear standard (1000 µg/L). The area of the Artesunate and DHA peaks in the water samples were expressed as a percentage of the peaks in the concentrated sample. Carry-over for each analyte was <0.1%

**Artesunate Carryover assessment (Peak Area)**			
**Sample ID**	**ART Std 8 (1000 ug/L) area**	**Blank Area**	**% Recovery**
Blank 1	371302	1	0.00
Blank 2	293225	2	0.00
Blank 3	320797	1	0.00
Blank 4	283752	4	0.00
Blank 5	298174	17	0.01
**Average**	**313450**	**5**	**0.00**

DHA Carryover assessment (Peak Area)			
**Sample ID**	**DHA Std 8 (1000 ug/L) area**	**Blank Area**	**% Recovery**
Blank 1	98459	1	0.00
Blank 2	94419	4	0.00
Blank 3	96193	2	0.00
Blank 4	94895	7	0.01
Blank 5	104140	38	0.04
**Average**	**97621**	**10**	**0.01**

7.5 Extraction comparison and Recovery

Each of three protein precipitation solutions was used to extract a set of standards; the internal standard peak's area was then used to evaluate which protein precipitation solution was most effective in yielding the greatest recovery. The table below demonstrates that the protein precipitation solution made with 100% acetonitrile yields the best recovery and was therefore chosen for this assay.

	IS Area 100% MeOH		IS Area 100% ACN		IS Area 0.1% Formic in ACN	
**Transition Sample**	**406.3>267.2 Artesunate**	**270.3>148.0 DHA**	**406.3>267.2 Artesunate**	**270.3>148.0 DHA**	**406.3>267.2 Artesunate**	**270.3>148.0 DHA**
STD 1	12456	11092	22699	17423	19820	17114
STD 2	14998	11483	20962	17022	16925	16207
STD 3	14300	11620	22501	16457	18825	16556
STD 4	13490	11726	21525	16566	16944	17131
STD 5	14229	11552	21865	16407	17095	16615
STD 6	13839	11983	17180	15133	15379	13875
STD 7	12052	10835	16983	14935	16359	14209
STD 8	12762	11216	15701	14655	175034	13624

7.6 Linearity

The linearity of the assay was established by running the standards (n=5), with a range of 1µg/L to 1000 µg/L, over 3 weeks. The results can be found in the table below.

Linearity								
Run #	Artesunate Std Concentration (µg/L)							
	**1**	**2**	**5**	**20**	**100**	**500**	**700**	**1000**
1	1.1	1.8	5.0	19.8	104.0	525.6	621.5	1049.2
2	1.0	2.0	4.9	21.2	91.5	518.7	787.3	901.4
3	0.7	2.0	7.0	20.3	89.1	559.7	696.2	953.1
4	0.8	2.1	5.5	19.5	96.6	550.7	713.7	938.9
5	1.2	2.1	5.4	17.5	86.5	466.1	733.9	1015.5
**Mean**	0.9	2.0	5.5	19.7	93.5	524.2	710.5	971.6
**SD**	0.2	0.1	0.8	1.4	7.0	36.7	60.4	59.7
**% CV**	4.6	14.9	6.6	14.5	13.4	14.3	11.8	16.3
**Accuracy** (%)	94.2	99.7	110.8	98.3	93.5	104.8	101.5	97.2

Linearity								
Run #	DHA Std Concentration (µg/L)							
	**1**	**2**	**5**	**20**	**100**	**500**	**700**	**1000**
1	1.1	3.9	5.0	18.6	94.2	508.2	698.9	1093.3
2	0.9	2.0	5.9	19.5	92.7	544.7	697.7	964.8
3	1.1	2.0	4.9	21.0	92.2	501.5	700.3	1005.1
4	0.9	1.9	5.1	21.9	102.0	499.4	697.7	999.1
5	1.0	2.2	5.2	16.9	99.9	494.5	715.6	992.7
**Mean**	1.0	2.4	5.2	19.6	96.2	509.6	702.1	1011.0
**SD**	0.1	0.8	0.4	2.0	4.4	20.2	7.7	48.5
**% CV**	11.0	2.8	13.7	10.0	21.6	25.3	91.6	20.8
**Accuracy** (%)	100.2	119.1	104.0	97.8	96.2	101.9	100.3	101.1

8.0 RECOMMENDATION

It is recommended that this assay is implemented for clinical trial use.

Brett McWhinney

Supervising Scientist, Analytical Chemistry Unit, Chemical Pathology, Pathology QLD

Signature: ____Date:______________

Dr Kobus Ungerer

Director of Chemical Pathology, Pathology Queensland

Signature: ____Date:______________

Ms Sandra Klingberg

Principal Chief Scientist, Chemical Pathology, Pathology QLD

Signature: ____Date:______________

Dr Kobus Ungerer

Director of Chemical Pathology, Pathology Queensland

Signature: ____Date:______________

16.1.11 Publications based on study

QP16C14: K13 Pilot Clinical Study Report Appendices

Not applicable.

16.1.12 Important publications referenced in the report

Author Manuscript
HHS Public Access
Author manuscript
Science. Author manuscript; available in PMC 2015 June 23.

Published in final edited form as:
Science. 2015 January 23; 347(6220): 428–431. doi:10.1126/science.1260867.

K13-propeller mutations confer artemisinin resistance in *Plasmodium falciparum* clinical isolates

Judith Straimer[1], **Nina F. Gnädig**[1], **Benoit Witkowski**[2,*], **Chanaki Amaratunga**[3,*], **Valentine Duru**[2,*], **Arba Pramundita Ramadani**[4,5,*,†], **Mélanie Dacheux**[1], **Nimol Khim**[2], **Lei Zhang**[6], **Stephen Lam**[6], **Philip D. Gregory**[6], **Fyodor D. Urnov**[6], **Odile Mercereau-Puijalon**[7], **Françoise Benoit-Vical**[4,5,‡], **Rick M. Fairhurst**[3,‡], **Didier Ménard**[2,‡], and **David A. Fidock**[1,8,§]

[1]Department of Microbiology and Immunology, Columbia University College of Physicians and Surgeons, New York, NY, USA

[2]Malaria Molecular Epidemiology Unit, Institut Pasteur du Cambodge, Phnom Penh, Cambodia

[3]Laboratory of Malaria and Vector Research, National Institute of Allergy and Infectious Diseases, National Institutes of Health, Bethesda, MD, USA

[4]Centre National de la Recherche Scientifique (CNRS), Laboratoire de Chimie de Coordination UPR8241, Toulouse, France

[5]Université de Toulouse, UPS, Institut National Polytechnique de Toulouse, Toulouse, France

[6]Sangamo BioSciences, Richmond, CA, USA

[7]Institut Pasteur, Parasite Molecular Immunology Unit, Paris, France

[8]Division of Infectious Diseases, Department of Medicine, Columbia University College of Physicians and Surgeons, New York, NY, USA

Abstract

The emergence of artemisinin resistance in Southeast Asia imperils efforts to reduce the global malaria burden. We genetically modified the *Plasmodium falciparum K13* locus using zinc-finger nucleases and measured ring-stage survival rates after drug exposure in vitro; these rates correlate with parasite clearance half-lives in artemisinin-treated patients. With isolates from Cambodia, where resistance first emerged, survival rates decreased from 13 to 49% to 0.3 to 2.4% after the removal of K13 mutations. Conversely, survival rates in wild-type parasites increased from ≤0.6% to 2 to 29% after the insertion of K13 mutations. These mutations conferred elevated resistance to

[§]To whom correspondence should be addressed. df2260@columbia.edu.
[*]These authors contributed equally to this work.
[†]Present address: Department of Pharmacology and Therapy, Faculty of Medicine, Gadjah Mada University, Yogyakarta, Indonesia.
[‡]These authors contributed equally to this work.

All other authors declare no competing financial interests.

SUPPLEMENTARY MATERIALS
www.sciencemag.org/content/347/6220/428/suppl/DC1
Materials and Methods
Figs. S1 to S4
Tables S1 to S5
Results of two-sample *t* tests with unequal variances
References (33–36)

Author Manuscript Author Manuscript Author Manuscript Author Manuscript

recent Cambodian isolates compared with that of reference lines, suggesting a contemporary contribution of additional genetic factors. Our data provide a conclusive rationale for worldwide K13-propeller sequencing to identify and eliminate artemisinin-resistant parasites.

The worldwide use of artemisinin (ART)–based combination therapies (ACTs) for the treatment of *Plasmodium falciparum* malaria is the foundation of renewed efforts to eradicate this leading cause of childhood mortality (1, 2). The pharmacodynamic properties of clinically used ART derivatives [artesunate, artemether, and dihydroartemisinin (DHA)] can reduce the biomass of drug-sensitive parasites by four orders of magnitude every 48 hours (3), corresponding to a single cycle of asexual blood-stage *P. falciparum* development. The short half-life (typically <1 hour) of ART derivatives in plasma necessitates the use of longer-lasting partner drugs that can eliminate residual parasites once the ART component has dropped to subtherapeutic concentrations (4). The use of ACTs in expanded malaria control and elimination programs has yielded notable successes in recent years, contributing to an estimated 30% reduction in global mortality rates in the past decade (5).

These impressive gains, however, are now threatened by the emergence of ART resistance, first detected in western Cambodia and now observed in Thailand, Vietnam, and Myanmar (6, 7). The severity of this situation is underscored by the fact that resistance to piperaquine, an ACT partner drug, is emerging in western Cambodia (8, 9). No alternative, fully effective first-line therapy is currently available to replace ACTs, should ART fail globally. Clinically, ART resistance is defined as a long parasite clearance half-life (the time it takes for the peripheral blood parasite density to decrease by 50%) after treatment with ART monotherapy or an ACT (6, 10, 11). This metric correlates with the percentage of early “ring-stage” parasites (0 to 3 hours after invasion of human erythrocytes) that survive a pharmacologically relevant exposure to DHA (the active metabolite of all ARTs), as measured in the in vitro Ring-stage Survival Assay ($RSA_{0–3h}$) (12).

Recently, mutations in the propeller domain of the *K13* gene were identified as candidate molecular markers of ART resistance (13). This gene resides on chromosome 13 of the *P. falciparum* genome, near regions earlier associated with slow parasite clearance rates (14–16). K13 belongs to the kelch superfamily of proteins, whose propeller domain harbors multiple protein-protein interaction sites and mediates diverse cellular functions, including ubiquitin-regulated protein degradation and oxidative stress responses (17). The K13 M476I mutation was first observed in Tanzanian F32 parasites that were exposed in vitro to escalating concentrations of ART over 5 years, yielding the F32-ART line (13, 18). [Single-letter abbreviations for the amino acid residues are as follows: A, Ala; C, Cys; D, Asp; E, Glu; F, Phe; G, Gly; H, His; I, Ile; K, Lys; L, Leu; M, Met; N, Asn; P, Pro; Q, Gln; R, Arg; S, Ser; T, Thr; V, Val; W, Trp; and Y, Tyr. In the mutants, other amino acids were substituted at certain locations; for example, M476I indicates that methionine at position 476 was replaced by isoleucine.] Subsequent genomic analysis of Cambodian isolates identified four prevalent K13-propeller mutations (Y493H, R539T, I543T, and C580Y) that were associated with elevated $RSA_{0–3h}$ survival rates in vitro and long parasite clearance half-lives (>5 hours) in patients (13, 19). Determining whether K13-propeller mutations confer

Author Manuscript

ART resistance in clinical isolates and assessing the contributions of individual polymorphisms in distinct genetic backgrounds is essential to defining the underlying molecular mechanisms.

We developed zinc-finger nucleases (ZFNs) (20) to enable targeted genetic engineering of *K13* in newly culture-adapted Cambodian isolates and older established reference lines of *P. falciparum* (tables S1 and S2). ZFNs were introduced into cultured intra-erythrocytic parasites via electroporation with plasmids containing *K13* donor templates. ZFNs triggered double-stranded breaks in the *K13* genomic target locus of this haploid organism, leading to DNA resection and repair events that captured mutations delivered by pZFN^{K13}-hdhfr plasmids (fig. S1). Donor plasmids contained additional synonymous mutations that preclude ZFN binding while preserving the K13-translated amino acid sequence across that same stretch of DNA base pairs. These silent ZFN binding-site mutations protected the donor sequence and prevented the edited recombinant locus from being recleaved by the nucleases. Plasmids contained either the wild-type *K13* allele or one of several mutations (present in the six-blade K13-propeller domain) found in ART-resistant Cambodian isolates or F32-ART. This strategy successfully introduced or removed mutations in a set of *P. falciparum* clinical isolates from Cambodia, the epicenter of emerging ART resistance, as well as reference laboratory lines from distinct geographic origins (Fig. 1 and table S3). Of note, $RSA_{0–3h}$ assays comparing parental and edited control parasites showed no difference if only the binding-site mutations were introduced into the K13-propeller domain, indicating that these synonymous mutations were phenotypically silent (Fig. 1 and fig. S2). Independent assays with the same parasite lines tested by our different groups yielded consistent survival rates between laboratories (fig. S3).

Using donor plasmids containing a wild-type *K13*-propeller sequence and silent binding-site mutations, we generated a series of clones in which individual K13 mutations were removed from ART-resistant Cambodian isolates. One of these isolates (Cam3.II) showed slow clearance after ART monotherapy (in vivo half-life 6.0 hours) (table S2). Parental Cam3.I^{R539T} and Cam3.II^{R539T} isolates harboring the R539T mutation showed 40 to 49% $RSA_{0–3h}$ survival, whereas edited Cam3.I^{rev} and Cam3.II^{rev} clones carrying the reverted wild-type allele showed only 0.3 to 0.7% survival (Fig. 2, A and B, and table S4). These highly significant differences in the survival rates of ring-stage parasites exposed to elevated DHA concentrations confirm the importance of R539T in mediating in vitro ART resistance in Cambodian isolates. Significant reductions in $RSA_{0–3h}$ survival rates were also observed upon removal of I543T (43% in Cam5^{I543T} versus 0.3% in Cam5^{rev}) (Fig. 2C) and C580Y (13% in Cam2^{C580Y} versus 2.4% in Cam2^{rev}) (Fig. 2D).

We also assessed the impact of introducing K13 mutations into a fast-clearing Cambodian isolate (CamWT; in vivo half-life 3.7 hours) (table S2), the Cam3.II^{rev} clone, and three reference lines (V1/S, F32-TEM, and FCB). CamWT and Cam3.II^{rev} parasites harboring wild-type *K13* alleles showed 0.6 to 0.7% $RSA_{0–3h}$ survival, whereas the corresponding C580Y-edited clones yielded 9 and 24% survival, respectively (Fig. 2, E and F). Introducing R539T into V1/S caused a similar increase in $RSA_{0–3h}$ survival (0.3 to 21%) (Fig. 2G and table S4). Editing F32-TEM to express M476I caused a moderate increase in $RSA_{0–3h}$ survival (<0.2% in F32-TEM to 1.7% in F32-TEM^{M476I}) (Fig. 2H). We also observed

Author Manuscript Author Manuscript Author Manuscript Author Manuscript

modest in vitro resistance in FCB parasites edited to express C580Y, with $RSA_{0–3h}$ survival increasing from 0.3% in the parental line to 1.9% in FCB^{C580Y} parasites (Fig. 2I). This result differs from a recent study of the use of Cas9 in *P. falciparum*, which reported a greater increase in $RSA_{0–3h}$ survival (11 to 15%) in two clones engineered to express K13 C580Y (21). That report used the drug-sensitive NF54 strain—which was isolated decades before ART use and the emergence of resistance (22)—and did not examine additional mutations or assess the impact of removing K13 mutations from ART-resistant clinical isolates.

In contrast to the substantial changes we observed in the $RSA_{0–3h}$, standard in vitro dose-response measurements by use of parental and *K13*-edited V1/S and Cam3.II parasites revealed no effect of R539T or C580Y on DHA or artesunate median inhibitory concentration (IC_{50}) values (fig. S4). This finding is consistent with earlier studies that showed no correlation between IC_{50} values and clinical ART resistance (6, 10, 12).

We subsequently investigated whether individual mutations confer different levels of ART resistance in the $RSA_{0–3h}$. In the Dd2 reference line, the introduction of M476I, R539T, or I543T mutations conferred considerably higher degrees of resistance than those of Y493H and C580Y (10 to 30% versus 2 to 4% survival, respectively) (Fig. 2J and table S4). These data corroborate the recent observation of higher levels of in vitro resistance in Cambodian isolates containing the R539T mutation as compared with Y493H or C580Y (23).

The relatively modest increase in survival of C580Y-expressing Dd2 parasites compared with R539T- and I543T-expressing clinical isolates and edited clones was quite unexpected, given that C580Y has rapidly become the predominant mutant allele in western Cambodia (7, 13). We thus explored the impact of C580Y in different genetic backgrounds. Introducing C580Y conferred greater levels of resistance in three Cambodian isolates as compared with Dd2 and FCB parasites (Fig. 2K), suggesting a role for additional parasite factors in augmenting K13-mediated resistance in these contemporary field isolates. The disparity between relatively low in vitro resistance conferred by C580Y and its widespread dissemination in Cambodia might be explained by a lower fitness cost or increased transmission potential of C580Y-expressing parasites, or by the parasite genetic background.

Cambodian parasites are specifically characterized by sympatric subpopulations that show only limited genetic admixture and that generally harbor distinct K13 mutations (16). These findings suggest that K13 mutations might have arisen preferentially on backgrounds with favorable genetic factors. In this context, recent comprehensive analyses of K13 mutations across multiple sites in Southeast Asia have documented a series of additional mutations associated with slow clearance rates in Cambodia, Thailand, Myanmar, Laos, and Vietnam (7, 24). K13 mutations have also been observed in African isolates (7, 25, 26), although none of these correspond to the most prevalent mutations in Cambodia, and ART or ACT treatments in African sites continue to show a high level of efficacy (7). A recent deep-sequencing study of the K13-propeller domain in more than 1110 *P. falciparum* infections collected from 14 sites across sub-Saharan Africa identified a large reservoir of naturally occurring K13-propeller variation, whose impact on artemisinin susceptibility is unknown and requires further investigation. These polymorphisms include one rare mutation

Author Manuscript Author Manuscript Author Manuscript Author Manuscript

previously observed in Cambodia (P553L) and several others (including A578S) close to known resistance-causing mutations in the propeller domain (26). Our gene-editing system can now be used to comprehensively dissect K13 polymorphisms across malaria-endemic regions and identify those that confer ring-stage ART resistance.

Mode-of-action studies have shown that ARTs are active against all asexual blood stages of parasite development. In the more mature trophozoite stages, ARTs are activated after hemoglobin degradation and liberation of reactive heme whose iron moiety can cleave the endoperoxide linkage of these sesquiterpene lactone drugs (27). Activation generates free radicals that are thought to trigger oxidative stress and damage cellular macromolecules, including parasite membrane components, proteins, and neutral lipids (28, 29). Recent evidence suggests that hemoglobin degradation begins early after merozoite invasion, potentially providing a source of ART activator in ring-stage parasites (30). Our RSA_{0-3h} data support earlier evidence that reduced ring-stage susceptibility accounts for the clinical phenotype of slow parasite clearance after ART treatment (12, 31). K13 mutations might achieve this by protecting parasites from the lethal effects of ART-induced oxidative damage, potentially via a cellular pathway similar to antioxidant transcriptional responses regulated by the mammalian ortholog Keap1 (32). Our set of K13-modified isogenic parasites with different levels of ART resistance on distinct genetic backgrounds now enables a search for K13-interacting partners and delivers tools to interrogate the underlying mechanism.

Our data demonstrate a central, causal role for K13-propeller mutations in conferring ART resistance in vitro and provide a molecular explanation for slow parasite clearance rates in patients (6, 7, 10). By exposing greater parasite biomasses to ACTs in vivo, K13-propeller mutations may promote the evolution of partner drug resistance (8, 9) and higher-grade ART resistance. Our study thus offers a conclusive rationale for a global K13 sequencing effort to track the spread of ART resistance and mitigate its impact on malaria treatment and control programs, particularly in hyperendemic regions in Africa.

Supplementary Material

Refer to Web version on PubMed Central for supplementary material.

Acknowledgments

D.A.F. gratefully acknowledges funding from the NIH (R01 AI109023). This study was supported in part by the Intramural Research Program of the National Institute of Allergy and Infectious Diseases, NIH, the French "Agence Nationale de la Recherche" (ANR-13-BSV3-0018-01 and the Laboratoire d'Excellence IBEID), and the Institut Pasteur, Division International (ACIP A-10-2010). Parental and transgenic parasite lines have been deposited and are being made available through BEI Resources (www.mr4.org) with the following accession numbers: MRA-1240, Cam3.IR539T (also known as IPC 5202); MRA-1252, Cam3.Irev; MRA-1241, Cam5I543T (also known as IPC 4912); MRA-1253, Cam5rev; MRA-1236, Cam2C580Y (also known as IPC 3445); MRA-1254, Cam2rev; MRA-1250, CamWT; MRA-1251, CamWTC580Y; MRA-150, Dd2; MRA-1255, Dd2R539T. Parasite lines generated for this study will also be provided upon request from D.A.F. Requests for ZFNs should be directed to F.D.U. (FUrnov@sangamo.com); a materials transfer agreement is required. We extend our gratitude to F. Ariey (Institut Pasteur, Paris) for his important contribution to initiating this study, I. McKeague and O. Lieberman (Columbia University Medical Center) for their statistical and scientific input, and E. Rebar and the Production Group at Sangamo BioSciences for ZFN assembly and validation. L.Z., S.L., P.D.G., and F.D.U. declare that they are full-time employees of Sangamo, which designed, validated, and provided the ZFNs used in this study. B.W., O.M.-P., F.B.-V., and D.M., are co-inventors on the pending patents US61/904651 and US62/062439, and N.K. is a

Author Manuscript Author Manuscript Author Manuscript Author Manuscript

co-inventor on the pending patent US62/062439. Both patents are filed by Institut Pasteur. These patents cover the use of K13 mutations as a molecular marker of *P. falciparum* ART resistance. Sangamo holds patents on engineered DNA-binding proteins and the use thereof in targeted genome engineering and gene-specific regulation.

REFERENCES AND NOTES

1. Feachem R, Sabot O. Lancet. 2008; 371:1633–1635. [PubMed: 18374409]
2. White NJ, et al. Lancet. 2014; 383:723–735. [PubMed: 23953767]
3. White NJ. Parassitologia. 1999; 41:301–308. [PubMed: 10697872]
4. Eastman RT, Fidock DA. Nat Rev Microbiol. 2009; 7:864–874. [PubMed: 19881520]
5. World Health Organization. World Malaria Report. WHO Press; Geneva, Switzerland: 2013. available at www.who.int/malaria/publications/world_malaria_report_2013/en
6. Dondorp AM, et al. N Engl J Med. 2009; 361:455–467. [PubMed: 19641202]
7. Ashley EA, et al. N Engl J Med. 2014; 371:411–423. [PubMed: 25075834]
8. Saunders DL, et al. N Engl J Med. 2014; 371:484–485. [PubMed: 25075853]
9. Leang R, et al. Antimicrob Agents Chemother. 2013; 57:818–826. [PubMed: 23208711]
10. Amaratunga C, et al. Lancet Infect Dis. 2012; 12:851–858. [PubMed: 22940027]
11. Flegg JA, et al. Malar J. 2013; 12:411. [PubMed: 24225303]
12. Witkowski B, et al. Lancet Infect Dis. 2013; 13:1043–1049. [PubMed: 24035558]
13. Ariey F, et al. Nature. 2014; 505:50–55. [PubMed: 24352242]
14. Cheeseman IH, et al. Science. 2012; 336:79–82. [PubMed: 22491853]
15. Takala-Harrison S, et al. Proc Natl Acad Sci USA. 2013; 110:240–245. [PubMed: 23248304]
16. Miotto O, et al. Nat Genet. 2013; 45:648–655. [PubMed: 23624527]
17. Adams J, Kelso R, Cooley L. Trends Cell Biol. 2000; 10:17–24. [PubMed: 10603472]
18. Witkowski B, et al. Antimicrob Agents Chemother. 2010; 54:1872–1877. [PubMed: 20160056]
19. Amaratunga C, Witkowski B, Khim N, Menard D, Fairhurst RM. Lancet Infect Dis. 2014; 14:449–450. [PubMed: 24849722]
20. Straimer J, et al. Nat Methods. 2012; 9:993–998. [PubMed: 22922501]
21. Ghorbal M, et al. Nat Biotechnol. 2014; 32:819–821. [PubMed: 24880488]
22. Ponnudurai T, Meuwissen JH, Leeuwenberg AD, Verhave JP, Lensen AH. Trans R Soc Trop Med Hyg. 1982; 76:242–250. [PubMed: 7048650]
23. Amaratunga C, et al. Antimicrob Agents Chemother. 2014; 58:4935–4937. [PubMed: 24867977]
24. Takala-Harrison S, et al. J Infect Dis. 201410.1093/infdis/jiu491
25. Conrad MD, et al. PLOS One. 2014; 9:e105690. [PubMed: 25144768]
26. Taylor SM, et al. J Infect Dis. 201410.1093/infdis/jiu467
27. Klonis N, Creek DJ, Tilley L. Curr Opin Microbiol. 2013; 16:722–727. [PubMed: 23932203]
28. Hartwig CL, et al. Biochem Pharmacol. 2009; 77:322–336. [PubMed: 19022224]
29. Antoine T, et al. J Antimicrob Chemother. 2014; 69:1005–1016. [PubMed: 24335485]
30. Klonis N, et al. Proc Natl Acad Sci USA. 2011; 108:11405–11410. [PubMed: 21709259]
31. Saralamba S, et al. Proc Natl Acad Sci USA. 2011; 108:397–402. [PubMed: 21173254]
32. Keum YS, Choi BY. Molecules. 2014; 19:10074–10089. [PubMed: 25014534]

Author Manuscript

Author Manuscript

Author Manuscript

Author Manuscript

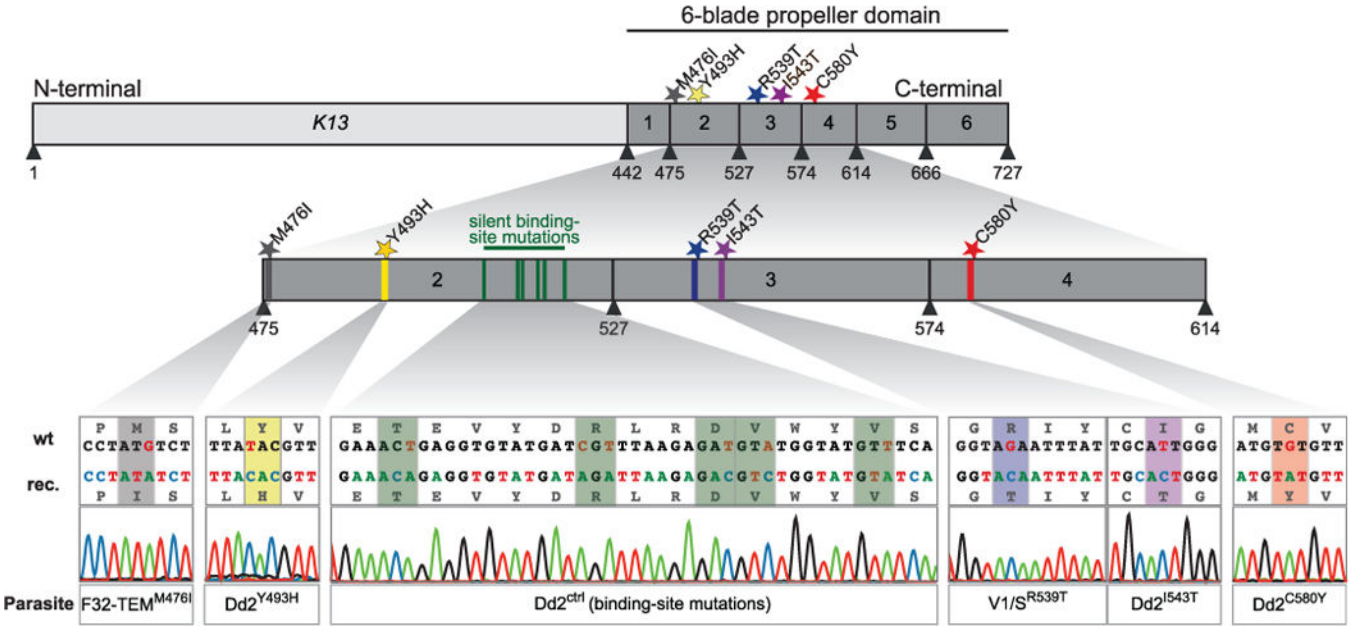

Fig. 1. Genetic modification of the K13-propeller domain

Location of K13-propeller mutations and sequencing results showing the insertion of individual mutations into recombinant parasites used in the $RSA_{0–3h}$. $Dd2^{ctrl}$ parasites contain only synonymous, phenotypically silent binding-site mutations and showed 0.7% survival rates, which is equivalent to those of parental Dd2 parasites (fig. S2).

Author Manuscript Author Manuscript Author Manuscript Author Manuscript

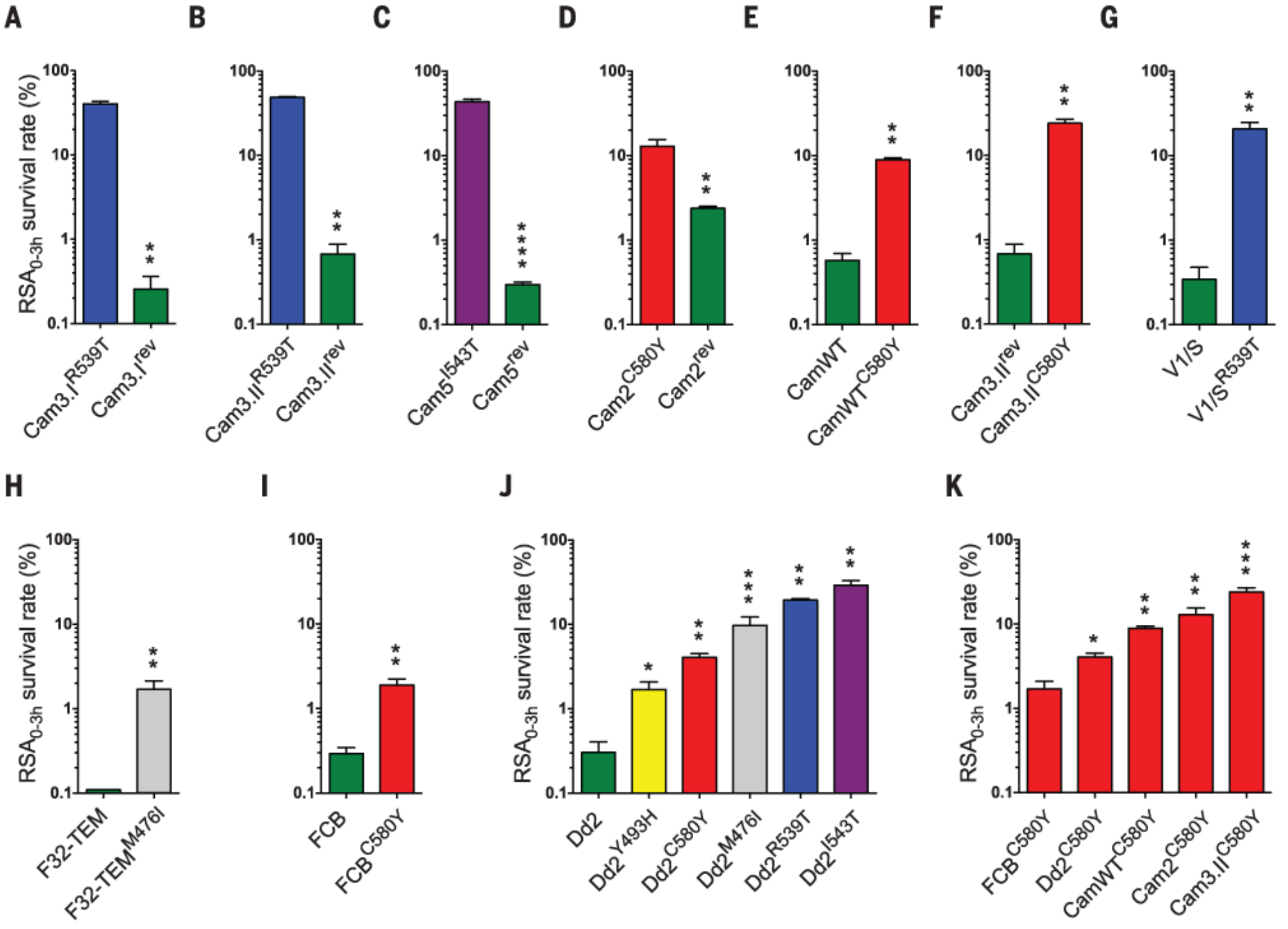

Fig. 2. K13-propeller mutations confer artemisinin resistance in clinical isolates and reference lines in vitro, as defined in the $RSA_{0–3h}$

Results show the percentage of early ring-stage parasites (0 to 3 hours after invasion of human erythrocytes) that survived a 6-hour pulse of 700 nM DHA (a pharmacologically relevant concentration of the active metabolite of ARTs), as measured by microscopy 66 hours later. Data show mean ± SEM percent survival compared with control dimethyl sulfoxide–treated parasites processed in parallel. (**A** to **D**) $RSA_{0–3h}$ survival for Cambodian isolates harboring native K13 mutations (shown in superscript) and ZFN-edited isogenic clones carrying wild-type *K13* alleles (superscript "rev"). (**E** to **I**) $RSA_{0–3h}$ survival for Cambodian isolates and reference lines harboring wild-type *K13* alleles and ZFN-edited isogenic clones carrying individual K13 mutations (shown in superscripts). (**J**) Impact of different K13 mutations on $RSA_{0–3h}$ survival in the Dd2 reference line, showing that I543Tand R539Tconfer the highest levels of resistance. (**K**) Introduction of C580Y into multiple Cambodian clinical isolates and reference lines, showing that this mutation confers varying degrees of in vitro resistance depending on the parasite genetic background. The geographic origins and known drug-resistance genotypes of these isolates and lines are provided in table S2. Results were obtained from 3 or 4 independent assays performed in duplicate (values provided in table S4; F32-TEM showed <0.2% $RSA_{0–3h}$ survival). Two-sample t tests with unequal variances (performed with the STATA package) were used to assess for statistically significant differences between *K13*-edited clones and their

Author Manuscript Author Manuscript Author Manuscript Author Manuscript

comparator lines—the parental isolates listed on the left in (A) to (J) and the FCB^{C580Y} clone in (K) (*$P < 0.05$; **$P < 0.01$; ***$P < 0.001$; ****$P < 0.0001$). Statistical outputs (including calculations of the SE of the difference between the means of samples being compared and the *P* values) are listed in the supplementary materials.

16.1.13 Reports

PRR Calculation - QP16C14 Cohort 1 and 2 - K13 Pilot Study

PRR analyses using the daily PCR data (tested in triplicates)

Louise Marquart and Lachlan Webb

14 June 2018

Dataset: Daily data for QP16C14 - P2286

Description:

The parasite reduction ratio (PRR_{48}) provides an estimate of the efficacy of an anti-malarial treatment and is the ratio of the parasite density over a 48 hour period. The PRR_{48} is estimated using the slope of the optimal fit of the log-linear relationship of the parasitemia decay. The optimal fit can be derived using summarised replicate parasitemia data, which have been cleaned by dealing with potential outliers, values below the limit of detection (LOD) and non-detectable values (ND). The optimal fit of the log-linear parasitemia by time relationship is determined by using left and right censoring to systematically remove the potential lag phase and tail phase of the parasitemia decay.

The PRR_{48} was calculated for each subject in QP16C14 using the daily PCR data; and if the model fit was adequate for the subject (defined as overall model p-value<0.001), the slope and corresponding standard error (SE) from the log-linear regression was used to calculate the overall study specific PRR_{48}. The details of the statistical methods used and the corresponding results are presented below.

Summary:

The PRR_{48} (95% CI) and corresponding parasite clearance half-life (95%CI) based on the 2 subjects who received the K13 strain in the pilot study is presented below:

Study	n	PRR_{48} (95% CI)	Parasite clearance $t_{1/2}$ (95% CI)
QP16C14	2	497 (266 - 926)	5.36 (4.87 - 5.96)

Statistical Methods:
Statistical analysis consists of two parts, initially calculating the decay rate (slope coefficient from the log-linear decay regression) for each individual, and then calculating the weighted average slope estimate and corresponding SE using inverse-variance method - which would then be used to estimate the study specific PRR_{48} and 95% CI. The details for initial data cleaning and preparation and both statistical analysis steps are briefly described below. For a more detailed description the reader is referred to: Marquart et al. (2015) Evaluating the pharmacodynamic effect of antimalarial drugs in clinical trials by Quantitative PCR, Antimicrobial Agents and Chemotherapy, 7(59), 4249-4259.

Dealing with parasitemia values below the limit of detection (LOD) and not detectable (ND) values
Once potential outliers have been identified and dealt with, the replicate parasitemia data were next cleaned to take into account parasitemia values where a value fell below the LOD or was not-detected (ND). For any replicate parasitemia values below LOD, the value was substituted with LOD/2. The LOD was assumed to be 64 parasites/mL of blood, based on Rockett et al. (2011). For any values that were ND the value was substituted with 1. If all replicates within a time-point were ND, the first time-point with all ND was included in model fitting, and all subsequent time-points were set to missing.

Dealing with qPCR replicates
The retrospective PCR data parasitemia values (tested in triplicate per time-point) were log10 transformed, and the mean of the log10 transformed parasitemia was used as a summary parasitemia value per time-point per subject. All regression analyses were performed using the mean log10 parasitemia.

Regression modelling to determine optimal fit
A regression modelling method, to incorporate both right and left censoring to remove potential lag and tail phases of the parasitemia decay curve in a systematic way, was used to determine the optimal number of data-points required to calculate the slope (β_1) of the log-linear parasitemia decay. A minimum number of 4 time-points was required for regression modelling.

The log-linear decay is modelled by the following:

$$\log_{10} Parasitemia = \beta_0 + \beta_1 Time$$

where time is the number of hours since administration of anti-malarial treatment, and β_0 and β_1 are the intercept and slope estimates of the log-linear regression, respectively.

The optimal log-linear regression model for a subject was deemed an appropriate fit if the overall model p-value < 0.001. Only optimal models with appropriate fit was used to calculate study specific PRR.

Estimating subject specific parasite reduction ratio (PRR)

The slope and corresponding standard error estimate of the optimal linear regression model was used to calculate the subject PRR_{48} estimate and corresponding 95% confidence interval (95% CI). The PRR_{48} and 95% CI was calculated for each subject using the following formula:

$$PRR_{48} = 10^{-48\times\beta_1} \text{ and}$$

$$95\% \text{ CI: } 10^{-48(\beta_1 \pm 1.96\times SE(\beta_1))}$$

Estimating study specific PRR

Of the subjects with appropriate overall fit (p<0.001), the average PRR_{48} and corresponding 95% CI for the study was estimated by using the inverse variance method to calculate the weighted average linear regression slope ($\overline{\beta}_1$) and corresponding SE. The weighted average slope for the n subjects in the subject is given by:

$$\overline{\beta}_1 = \frac{\sum_{i=1}^{n}(w_i \times \beta_{1,i})}{\sum_{i=1}^{n} w_i}, i = 1, \ldots, n,$$

where the weight $w_i = \frac{1}{SE(\beta_{1,i})^2}$. The standard error $\hat{\beta}_1$ is estimated as, $SE(\overline{\beta}_1) = \sqrt{\frac{1}{\sum_{i=1}^{n} w_i}}$.

Therefore, the study specific PRR_{48} and corresponding 95 % confidence interval is estimated as:

$$PRR_{48} = 10^{-48\times\overline{\beta}_1}$$

$$95\ \% \text{ CI: } 10^{-48\times(\overline{\beta}_1 \pm 1.96\times SE(\overline{\beta}_1))}$$

Parasite clearance half-life

The half-life is a transformation of the gradient (per time, equivalent to PRR) into a time period. The relationship between PRR and parasite reduction half-life ($t_{1/2}$) is a simple transformation of the PRR:

$$t_{1/2} = \log_{10}(2) \times \left(\frac{48 \text{ hours}}{\log_{10}(PRR_{48})}\right) = \frac{\log_{10}(2)}{-\beta_1},$$

where the PRR_{48} is the parasitemia ratio estimated over a 48 hour interval that is subsequently transformed into a per hour gradient.

Optimal Regression Results:

The last time-point included in the regression analysis (based on the last time-point with all replicate values set to ND) for the 2 subjects in QP16C14 is given in Table 1.

Table 1: Last time-point included in the regression analysis for each subject.

Subject	Last time-point (hrs)
R001 (S004)	60
R002 (S003)	60

The subject specific PRR_{48} and corresponding 95% confidence interval are presented in Table 2 for the two subjects in QP16C14. The results show the regression fit (R-squared (R^2)) and parameters (slope ($\hat{\beta}_1$)) and corresponding SE (SE($\hat{\beta}_1$)) for the subject based on the optimal regression. The corresponding plot of log-10 parasitemia over time is presented in Figure 1.

Table 2: Optimal regression fit for the subjects in QP16C14 Cohort 1 and 2.

Subject	Iter	R^2	p-value	$\hat{\beta}_1$	SE($\hat{\beta}_1$)	PRR (95% CI)	Half-life (95% CI)
QP16C14-R001	4	96.4%	8.13E-8	-0.055	0.004	447 (207-964)	5.45 (4.84-6.24)
QP16C14-R002	4	93.9%	8.94E-7	-0.058	0.005	609 (210-1767)	5.19 (4.45-6.22)

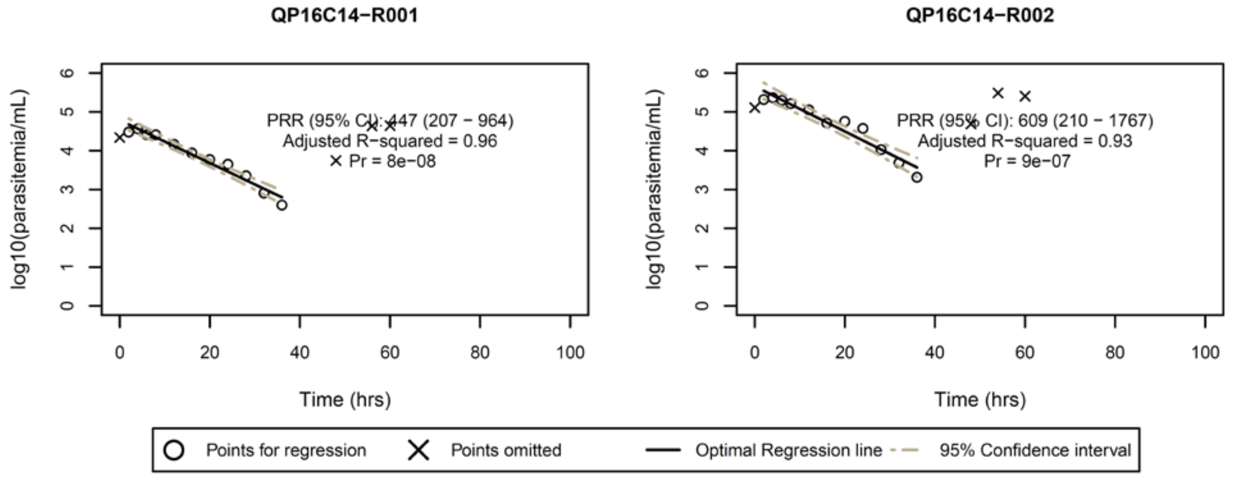

Figure 1: Optimal regression fit for the subjects in QP16C14 Cohort 1 and 2.

Study specific PRR:

A study specific PRR_{48} was calculated from the corresponding subjects that had significant regression models at the p=0.001 significance level.

All two of the subjects had significant regression models at the p=0.001 significance level, and contributed towards the study specific PRR_{48} in QP16C14. The overall slope estimate and corresponding standard error used in the calculation of the study specific PRR is:

$$\overline{\beta}_1 = -0.056$$

$$SE(\overline{\beta}_1) = 0.003$$

The PRR_{48} and 95% confidence interval for QP16C14 is:

$$PRR_{QP16C14} = 496.8 \text{ (95 \% CI: } 266.4 - 926.3)$$

The corresponding half-life and 95% confidence interval for QP16C14 is:

$$t_{1/2\ (QP16C14)} = 5.36 \text{ (95 \% CI: } 4.87 - 5.96)$$

PK Noncompartmental Analysis: QP16C14 - *P.falciparum* K13 treated with Artesunate

Azrin Abd Rahman

15 November 2018

Data Description:

Data was provided for artesunate and, its active metabolite, dihydroartemisin (DHA) plasma concentrations over a period of 12 hours following administration of 150 mg artesunate given orally for one patient for each cohort (total of 2 patients). The lower limit of quantification (LLOQ) of artesunate and DHA was 1 µg/L. The original data is located at:

L:\Lab_JamesM\2_STUD\1_CLIN_TRIALS\QP16C14_K13 Pilot_P2286\7_DATA\2_PK

Cohort 1 file names = “QP16C14_PathQLD PK sampling corhort 1 150617 complete.xlxs”

Cohort 2 file names = “QP16C14_PathQLD PK sampling corhort 2 280617.xlxs”

Methods:

Data preparation and analysis were performed in R (version 3.4.2) and the R package IQRtools (version 0.9.1, IntiQuan GmbH, Basel, Switzerland). Plasma artesunate and DHA concentration-time data were analysed by noncompartmental methods. Maximum drug plasma concentration (C_{max}) and time of C_{max} occurred (t_{max}) were taken from observed concentration-time profiles. The terminal slope (k_{el}) was determined by non-linear regression of the terminal portion of the concentration-time profile. The elimination half-life ($t_{½}$) was calculated as 0.693/k_{el}. The area under the concentration-time curve from time 0 to the last measurable concentration ($AUC0\text{-}_{last}$) was determined using the trapezoidal method. The AUC from time 0 to infinite time ($AUC0\text{-}_{inf}$) was calculated as:

$$\mathrm{AUC}_{0-\mathrm{inf}} = \mathrm{AUC}_{0-\mathrm{last}} + \mathrm{AUC}_{\mathrm{last-inf}}$$

where $AUC_{last\text{-}inf}$ is the AUC from the last measurable concentration to infinite time and can be calculated as C_{last}/k_{el}. The apparent clearance (CL/F) was determined as Dose/$AUC_{0\text{-}inf}$ and apparent volume of distribution (Vd/F) calculated as Dose/(k_{el} x $AUC_{0\text{-}inf}$) where the artesunate dose was assumed to be totally and exclusively converted to DHA. The DHA dose was assumed to be equal to the artesunate dose on a molar basis, calculated as:

$$\text{DHA dose (in mg)} = \text{Artesunate dose (in mg)} \times \frac{284.35}{384.42}$$

where 284.35 and 384.42 are the molecular weight of DHA and artesunate, respectively. The values for $t_{½}$, CL/F, Vd/F and $AUC_{0\text{-}inf}$ were reported if the following criteria are met:

- A minimum of 3 measurable concentration-time points during the log-linear portion of the terminal elimination phase, excluding C_{max}.
- R^2 >0.80 for the regression of the log-concentration time data during the terminal elimination phase.
- Extrapolated portion of $AUC_{0\text{-}inf}$ <20% of total $AUC_{0\text{-}inf}$.

Concentrations below the LLOQ were assigned to zero.

Noncompartmental Analysis Results

Artesunate was rapidly hydrolysed to DHA with artesunate concentration below the LLOQ after two hours post-dose (Figure 1 and 2). Therefore, the terminal slope of the artesunate concentration-time profile could not be estimated as there were the limited samples after C_{max} with concentrations above the LLOQ. The descriptive summary of artesunate and DHA PK parameters for two subjects in QP16C14 is given in Table 1.

Table 1: Descriptive summary of artesunate and dihysroartemisinin pharmacokinetic parameters.

PK parameter	Mean (SD)	Median (Min – Max)	Geometric mean (CV%)
Artesunate			
C_{max} (µg/L)	102.66 (6.84)	102.66 (97.83 – 107.50)	102.55 (6.67)
$AUC_{0\text{-last}}$ (h·µg/L)	72.08 (28.19)	72.08 (52.15 – 92.01)	69.27 (41.82)
t_{max} (h)	0.75 (0.35)	0.75 (0.5 – 1.0)	0.71 (52.11)
$t_{½}$ (h)	NA	NA	NA
CL/F (L/h)	NA	NA	NA
Vd/F (L)	NA	NA	NA
Dihydroartemisinin			
C_{max} (µg/L)	429.62 (160.41)	429.62 (36.2 – 543.05)	414.38 (39.68)
$AUC_{0\text{-last}}$ (h·µg/L)	695.96 (309.25)	695.96 (477.29 – 914.64)	660.72 (48.53)
t_{max} (h)	1.25 (0.35)	1.25 (1.0 – 1.5)	1.22 (29.27)
$t_{½}$ (h)	1.10 (0.61)	1.10 (0.67 – 1.53)	1.01 (63.77)
CL/F (L/h)	142.33 (53.29)	142.33 (104.65 – 180.02)	137.25 (29.81)
Vd/F (L)	202.71 (40.38)	202.71 (174.15 – 231.26)	200.69 (20.26)

PK = pharmacokinetic, C_{max} = maximum concentration, $AUC_{0\text{-last}}$ = area under the concentration-time curve from time 0 to the last measurable concentration, $AUC_{0\text{-inf}}$ = area under the concentration-time curve from time 0 to infinite time, t_{max} = time of C_{max}, $t_{½}$ = elimination half-life, CL/F = apparent clearance, Vd/F = apparent volume of distribution where F is bioavailability, NA = not available.

The subject specific artesunate and DHA PK parameters are presented in Table 2 for all subjects in QP16C14. The regression line fit of artesunate and DHA for each subject are shown in Figure 3 and 4, respectively.

Table 2: Individual artesunate and dihydroartemisinin pharmacokinetic parameters.

PK parameters	Artesunate		Dihydroartemisinin	
	R001	R002	R001	R002
C_{max} (µg/L)	107.50	97.83	316.20	543.05
$AUC_{0\text{-last}}$ (h·µg/L)	92.01	52.15	477.29	914.64
$AUC_{0\text{-inf}}$ (h·µg/L)	NA	NA	538.33	926.04
t_{max} (h)	1.0	0.5	1.5	1.0
$t_{½}$ (h)	NA	NA	0.67	1.53
CL/F (L/h)	NA	NA	180.02	104.65
Vd/F (L)	NA	NA	174.15	231.26

PK = pharmacokinetic, C_{max} = maximum concentration, $AUC_{0\text{-last}}$ = area under the concentration-time curve from time 0 to the last measurable concentration, $AUC_{0\text{-inf}}$ = area under the concentration-time curve from time 0 to infinite time, t_{max} = time of C_{max}, $t_{½}$ = elimination half-life, CL/F = apparent clearance, Vd/F = apparent volume of distribution where F is bioavailability, NA = not available.

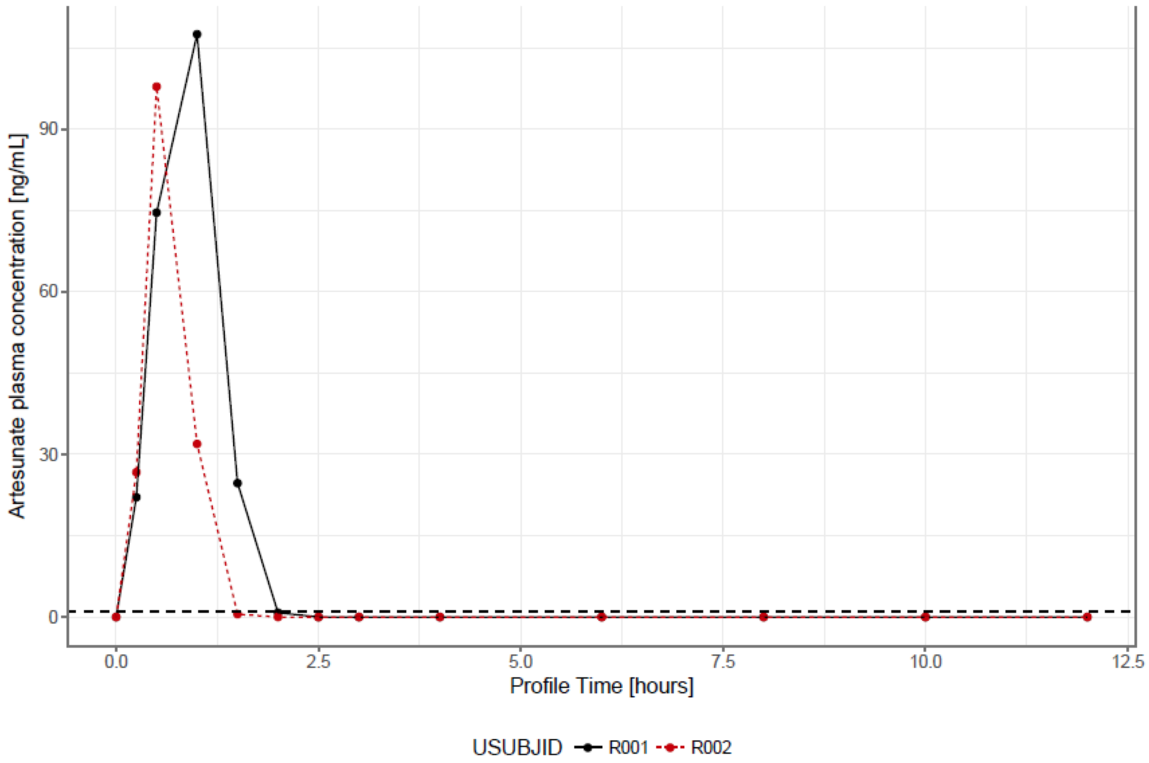

Figure 1a: Artesunate concentrations over time for two subjects in QP16C14 on a linear scale.

The black horizontal dashed line represents the lower limit of quantification (1 µg/L).

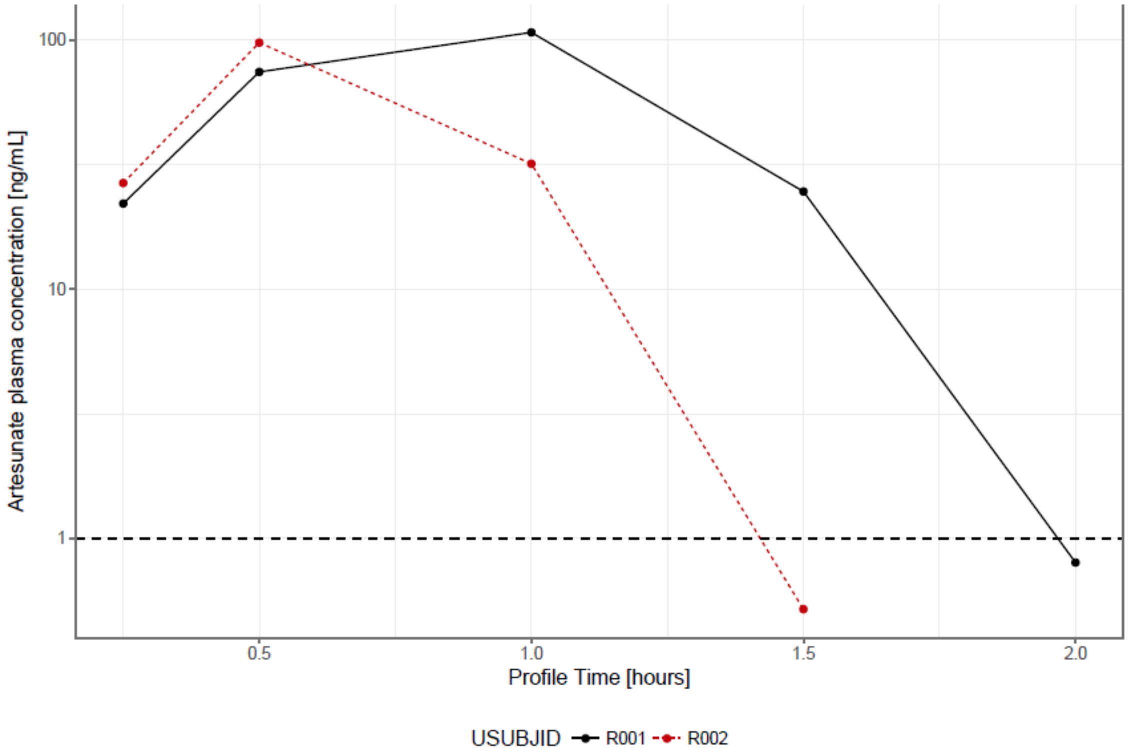

Figure 1b: Artesunate concentrations over time for two subjects in QP16C14 on a log scale.

The black horizontal dashed line represents the lower limit of quantification (1 µg/L).

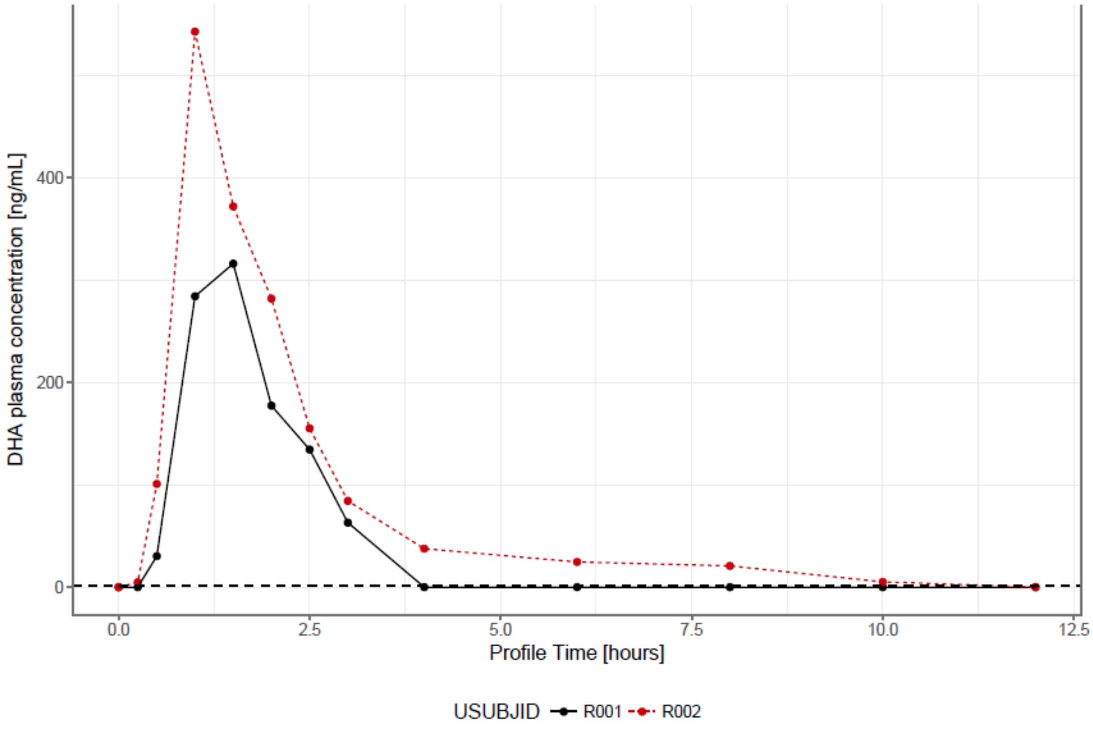

Figure 2a: Dihydroartemisinin concentrations over time for two subjects in QP16C14 on a linear scale.

The black horizontal dashed line represents the lower limit of quantification (1 µg/L).

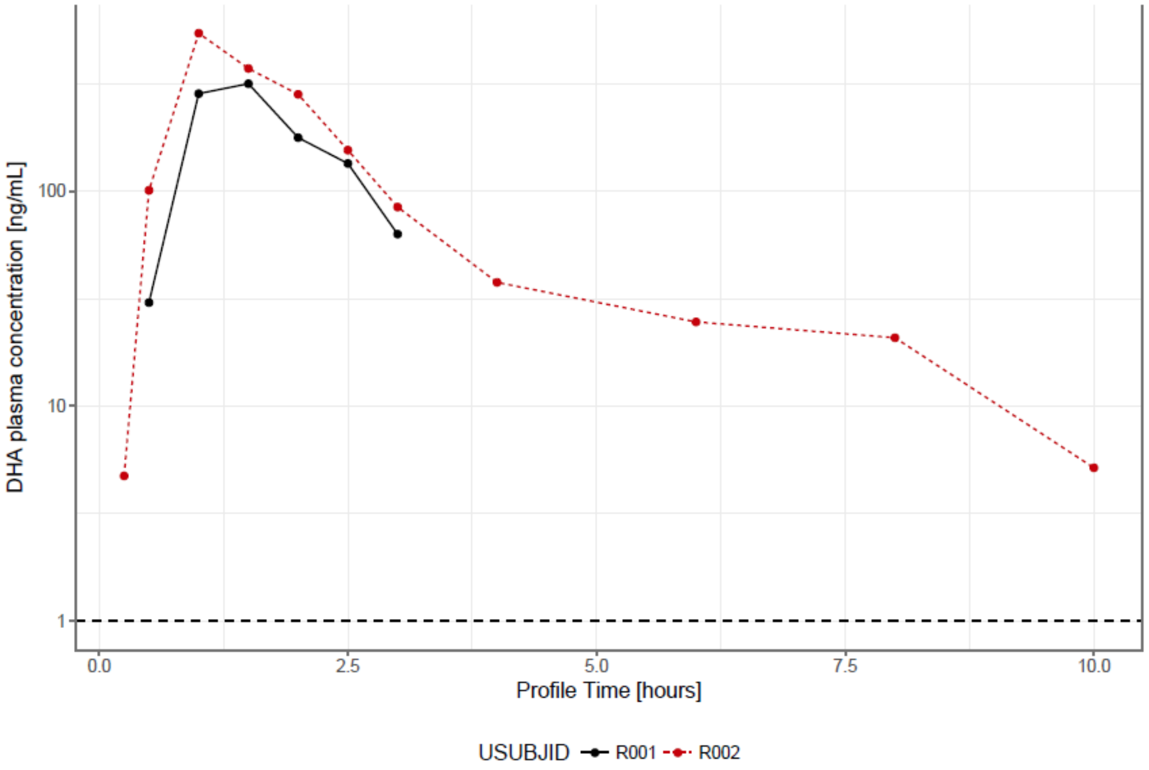

Figure 2a: Dihydroartemisinin concentrations over time for two subjects in QP16C14 on a log scale.

The black horizontal dashed line represents the lower limit of quantification (1 µg/L).

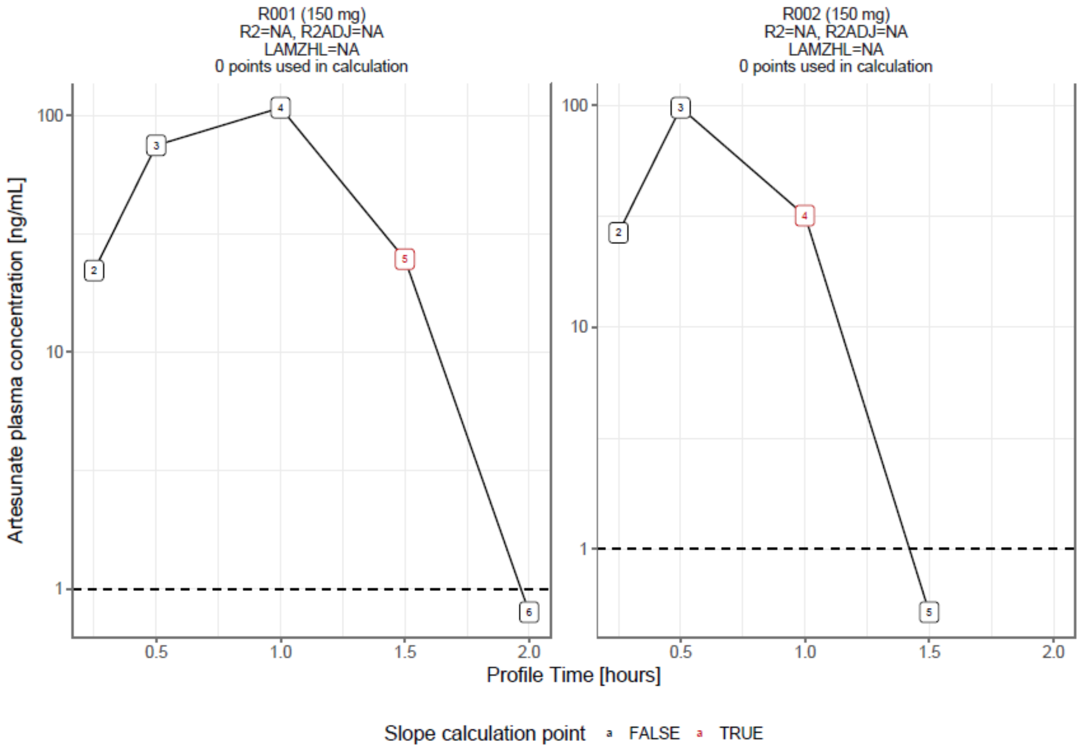

Figure 3: Regression line fit of artesunate for each subject in QP16C14.

Black horizontal dashed line represents the lower limit of quantification.

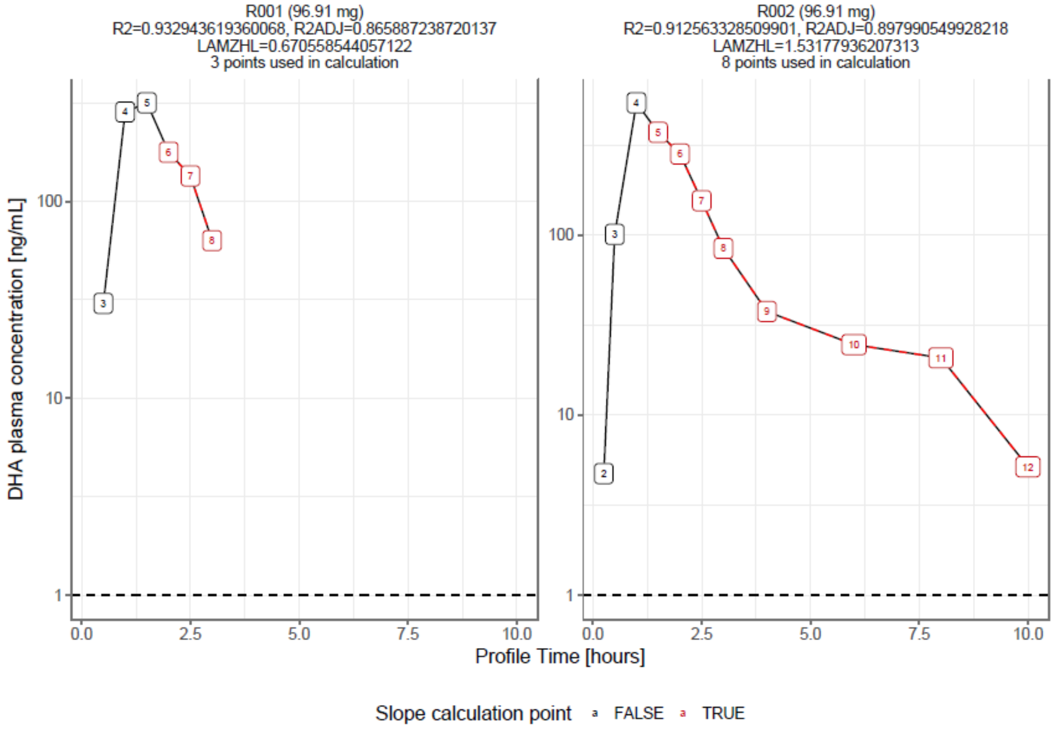

Figure 4: Regression line fit of dihydroartemisinin for each subject in QP16C14.

Black horizontal dashed line represents the lower limit of quantification.

Clinical Tropical Medicine, QIMR Berghofer
QIMR Berghofer Project: P2286

QIMR Berghofer
Medical Research Institute

Version: 1.0
Status: Final

Date Prepared: 14-Mar-17

**Document Type**	Safety Review Team Charter			
**Version #**	1.0			
**Protocol Title**	An experimental study to characterise the *in vivo* safety and infectivity of a *Plasmodium falciparum* Cam3.II^{R539T} (K13) artemisinin-resistant isolate in healthy participants.			
**Protocol No:**	QP16C14			
**Sponsor Project No.**	P2286			
**Author/Reviewer**	**Name**	Rebecca Watts	**Signature**	[illegible]
	**Position**	Project Manager	**Date**	15/03/2017
**Approval**	**Name**	James McCarthy	**Signature**	[illegible]
	**Position**	Principal Investigator - Clinical Site	**Date**	15/3/17
**Approval QIMR Berghofer Sponsor**	**Name**	Sashika Naidoo	**Signature**	[illegible]
	**Position**	Regulatory Affairs Manager	**Date**	21/03/2017
**Effective Date**	21/3/17		**Signature**	—
**Revision Date**	Two years after effective date			

1.0 PURPOSE

1.1 The purpose of this Safety Review Team (SRT) Charter is to outline the SRT procedures implemented for a project undertaken under the sponsorship of QIMR Berghofer.

1.2 Considering that this study will be the first time the *Plasmodium falciparum* Cam3.II^{R539T} (K13) artemisinin-resistant isolate will be given to healthy participants, a decision was made to establish a SRT. The role of the SRT will be to provide safety oversight of the proposed study. The role and the membership of the SRT are described in this procedure below.

2.0 DEFINITIONS

2.1 AE – adverse event
2.2 HREC – Human Research Ethics Committee
2.3 MMV – Medicines for Malaria Venture
2.4 PI – principal investigator
2.5 SAE – serious adverse event
2.6 SRT – Safety Review Team

3.0 ROLES AND RESPONSIBILITIES

3.1 Clinical safety oversight of this project will be undertaken by the Principal Investigator (PI) in conjunction with a SRT. The members of SRT will be composed of medical experts whose primary responsibility is to monitor the safety of study participants and to advise on clinical safety specifically in the situation where expert external advice is required regarding the need for administration of rescue antimalarial treatment in the circumstance of suboptimal response.

3.2 It is the responsibility of the PI to ensure that that the SRT is appraised of all new safety information relevant to the study product and the study. This

includes providing the Investigator's Brochure (IB) and study protocol in advance, as well as promptly providing all IB revisions, all other new safety information, all protocol revisions and other documents related to the study as required.

3.3 The PI will also consult with the SRT for any safety event that needs further evaluation.

3.4 The SRT, together with the PI, will be involved in the review of all adverse reactions.

3.5 The SRT is appointed by the PI in consultation and approval by the Sponsor of the study and the funding contributor Medicines for Malaria Venture (MMV).

3.6 The safety oversight will include but is not limited to:

3.6.1 Review serious adverse events (SAEs) that may be associated with the parasite inoculum or antimalarial drugs, and adverse events (AEs) of special interest.

3.6.2 Investigation of those events considered serious and unexpected.

3.6.3 Review of all clinical and laboratory data, clinical records and other study-related records for evaluation of study related AEs. These include but are not limited to:

3.6.3.1 List of the AEs and SAEs recorded for each participant.

3.6.3.2 List of all available clinical (electrocardiogram, vital signs, clinical scores) and laboratory (haematology, biochemistry, urinalysis) safety data including any recorded out of range results for the time points indicated in the study protocol for SRT review.

3.6.3.3 Available PK and PCR data for each participant.

3.6.3.4 List of concomitant medication taken by each participant.

3.6.3.5 Participant demography and withdrawals if applicable.

3.6.4 Review of participant outcomes.

3.6.5 Evaluation for adherence to the protocol.

3.6.6 Further investigation into specific events.

3.6.7 Advise Sponsor and funding contributor as to whether the study should continue or be modified or terminated.

3.7 All discussions involving the SRT will be treated with strict confidence and correspondences will be limited to authorised study personnel. Information will be shared with personnel authorised by the Sponsor, PI and MMV.

3.8 The decision to institute early curative treatment will be made in consultation with the SRT, to advise on the safety of continuing observation without rescue versus administration of curative treatment.

4.0 MEMBERSHIP

4.1 The members taking part in the safety oversight of this project include:

4.1.1 Prof. James McCarthy MBBS, MD, FRACP - Principal Investigator - voting member.

4.1.2 Prof. Dennis Shanks MD – SRT Chair, Independent Medial Monitor, and an expert in infectious diseases including malaria - voting member.

4.1.3 Dr Stephan Chalon MD – MMV Medical Director - voting member.

4.1.4 Dr Paul Griffin MBBS, FRACP, FRCPA - Co-investigator, non-voting member.

4.1.5 Dr Mark Armstrong MBChB - Co-investigator, non-voting member.

4.1.6 Dr Anand Odedra MBBS - Co-investigator, non-voting member.

4.2 Conflict of Interest

4.2.1 The PI is jointly the Visiting Medical Officer to the Q-Pharm clinical site and a Laboratory Head for QIMR Berghofer.

4.2.2 Dr Stephan Chalon is a funding contributor representative.

5.0 COMMUNICATIONS/MEETINGS

5.1 SRT meeting sessions will be open during which occurrences of AEs or general conduct and progress of the study will be discussed. These sessions will be open to study investigators, representatives and project managers.

5.2 The communications for the purpose of safety oversight will be in person or in electronic form (phone teleconference or by email). The study personnel or the PI will contact the SRT members as required.

5.3 The SRT meeting is requested by the Chair of the SRT or the PI, however any member of the SRT or the Sponsor may request a meeting.

5.4 The PI, or their delegate, is responsible for ensuring the distribution of the agenda and the materials for review to SRT members and other meeting participants.

5.5 The PI , or their delegate, is responsible after the meeting for ensuring the distribution of the meeting minutes in confidence to all SRT members and to any other persons from QIMR Berghofer, Q-Pharm or MMV as deemed appropriate.

5.6 The SRT will conduct a review of the safety, tolerability and parasitaemia data for a minimum of 14 days from the first study participant. This will occur prior to inoculation of the second study participant. The SRT members will review the available data and information as listed in Section 3.6.3.

5.7 Ad hoc meetings may also be organised if a significant safety concern has been raised or as required according to follow up of AEs. Additionally, the SRT will meet to assess any events that trigger the stopping rules.

5.8 Voting

5.8.1 After discussion of SRT members' opinions and rationale, each voting SRT member provides individual recommendation on whether to proceed to the next participant, postpone, or terminate the study.

5.8.2 The final SRT decision will be documented in a SRT decision form (see references) and signed by the SRT Chair and the PI and it will be filed in the Trial Master File. This information will also be submitted for notification to the funding contributor MMV and QIMR Berghofer Regulatory Affairs.

6.0 STUDY REPORTS FOR SRT REVIEW

6.1 It is the responsibility of the PI to ensure that the SRT members have received all new safety information relevant to the study. This will include trial data, dosage and treatment schedules, and AEs.

7.0 OTHER REPORTS OF STUDY PROGRESS

7.1 Safety and enrolment data should be forwarded periodically to the SRT members.

8.0 REPORTS FROM THE SRT

8.1 The SRT members will review the data and report back to the Sponsor and the HREC with recommendations for participant withdrawal, dosage adjustment, protocol amendment or study termination.

8.2 The PI or the clinical site will report any protocol deviations and violations, that have been identified and have a significant impact on safety, to SRT members and HREC.

8.3 Depending on the recommendations from the SRT, the Sponsor will liaise with the PI to determine a course of action.

8.4 The PI will respond to all SRT recommendations.

8.5 The PI or delegated clinical site nominee will submit to the HREC the final SRT recommendation, any protocol amendments, deviations or protocol violation reports as required.

8.6 The PI is also responsible for informing study participants of new safety data or changes to the study procedure if applicable.

9.0 REFERENCES

9.1 CTM QIMR QF 43A Safety Review Team Decision – Continue v3.0
9.2 CTM QIMR QF 43B Safety Review Team Decision – Postpone v3.0
9.3 CTM QIMR QF 43C Safety Review Team Decision – Terminate v3.0

Safety Review Team Meeting Minutes

**Project Number:**	**QP16C14/P2286 K13**
**Project Title:**	An experimental study to characterise the *in vivo* safety and infectivity of a *Plasmodium falciparum* Cam3.II^{R539T} (K13) artemisinin-resistant isolate in healthy participants
**Date of Meeting:**	Friday, 9 June 2017 (Brisbane time)
**Time and location:**	Correspondence by email

Attendees

Investigator/Co-Investigators	Independent Medical Monitor	MMV	QIMRB	Q-Pharm
Prof. James McCarthy<br>(Voting member)<br>Paul Griffin<br>Anand Odedra<br>Mark Armstrong	Prof. Dennis Shanks<br>(SRT Chair, Voting member)	Stephan Chalon<br>(Voting member)<br>Jörg Möhrle	Rebecca Watts<br>Silvana Sekuloski<br>Laura Cascales	Paul Morgan

Meeting objective: To review safety data from the first study participant, and to decide whether to progress to the second participant.

Documents distributed prior to the meeting:

- PowerPoint slides - QP16C14 K13 SRT 070617.pdf - sent 8 June 2017. Prepared by AO

Safety review:

- Reviewed safety, tolerability and parasitaemia data for 14 days from the first study participant
- No serious adverse events
- Adverse events, CTCAE Grade 1-3 – see PowerPoint slide 11
- Will continue to monitor liver function test changes including bilirubin levels. No specific clinical concerns other than the abnormal liver function tests
- JMc sees no safety signal that would concern him with moving on to the second participant. Advises that we will continue to watch the safety bloods of the first participant and we will monitor for further recrudescence of the parasite
- DS and SC agree with JMc's conclusion and have no concerns
- No protocol amendments were recommended

SRT decisions:

DS: Continue the study; proceed to the second participant

SC: Continue the study; proceed to the second participant

JMc: Continue the study; proceed to the second participant

SRT decision was captured in email record “QP16C14 K13 SRT meeting decision 9Jun2017”. SRT Decision form will be signed by SRT Chair (DS) and Investigator (JMc) once they return from overseas.

**From:**	Rebecca Watts
**To:**	Rebecca Watts
**Subject:**	RE: QP16C14 K13 SRT meeting and second participant[SEC=UNCLASSIFIED]
**Date:**	Monday, 12 June 2017 3:27:06 PM

From: James McCarthy [mailto:j.mccarthy@uq.edu.au]
Sent: Friday, 9 June 2017 9:02 PM
To: Rebecca Watts
Cc: shanks, dennis PROF; Stephan Chalon; Nicole Williams
Subject: Re: QP16C14 K13 SRT meeting and second participant[SEC=UNCLASSIFIED]

Hi Rebecca
Likewise for me, it may take a while for me to get you a wet ink-signed version. Can you take this email as my agreement for now.

thanks

From: Dennis Shanks <dennis.shanks@defence.gov.au>
Date: Friday, 9 June 2017 at 06:42
To: Rebecca Watts <Rebecca.Watts@qimrberghofer.edu.au>
Cc: "James.McCarthy@qimrberghofer.edu.au" <James.McCarthy@qimrberghofer.edu.au>
Subject: Re: QP16C14 K13 SRT meeting and second participant[SEC=UNCLASSIFIED]
Resent-From: <James.McCarthy@qimrberghofer.edu.au>
Resent-Date: Friday, 9 June 2017 at 06:43

Rebecca

Please proceed but it will take some time to get you a signature as I am on the way to Brazil right now

Dennis

Sent from my iPhone

On 9 Jun 2017, at 1:06 am, Rebecca Watts <Rebecca.Watts@qimrberghofer.edu.au> wrote:

> Hi All,
>
> **James and Dennis** – could you please sign the SRT decision form which will then be sent to QIMR-B Regulatory Affairs, and Q-Pharm for submission to HREC.
>
> I have also prepared brief minutes based on the slides and emails. Please let me know if you have any comments, then I will add the slides to the end, and finalise them for our records.
>
> I have stated in the minutes that no protocol amendments were recommended, as I believe we are proceeding with the second participant without making any changes to the study design. Please correct me if I am wrong.
>
> Best regards,

Rebecca

From: shanks, dennis PROF [mailto:dennis.shanks@defence.gov.au]
Sent: Friday, 9 June 2017 12:45 AM
To: Stephan Chalon
Cc: James McCarthy; Anand Odedra; p.morgan@qpharm.com.au; Rebecca Watts; Silvana Sekuloski; Paul Griffin; m.armstrong@qpharm.com.au; Laura Cascales; Joerg Moehrle
Subject: Re: QP16C14 K13 SRT meeting and second participant[SEC=UNCLASSIFIED]

James

I see no reason to be overly concerned.

Let me know if you need formal statement to proceed to second volunteer

Dennis

Sent from my iPhone

On 8 Jun 2017, at 5:11 am, Stephan Chalon <chalons@mmv.org> wrote:

> James / Anand,
>
> I agree. I have carefully reviewed the slides and don´t have concerns. As previously stated we would just need to ensure that bilirubin is not impacted.
>
> Kind Regards,
>
> Stephan
>
> **From:** James McCarthy [mailto:j.mccarthy@uq.edu.au]
> **Sent:** Thursday, 08 June 2017 12:43 PM
> **To:** Anand Odedra <Anand.Odedra@qimrberghofer.edu.au>; Stephan Chalon <chalons@mmv.org>; Dennis Shanks <dennis.shanks@defence.gov.au>
> **Cc:** p.morgan@qpharm.com.au; Rebecca Watts <Rebecca.Watts@qimrberghofer.edu.au>; Silvana Sekuloski <Silvana.Sekuloski@qimrberghofer.edu.au>; Paul Griffin <P.Griffin@qpharm.com.au>; m.armstrong@qpharm.com.au; Laura Cascales <Laura.Cascales@qimrberghofer.edu.au>; Joerg Moehrle <moehrlej@mmv.org>
> **Subject:** Re: QP16C14 K13 SRT meeting and second participant
>
> Dear Anand
> Thanks for preparing the slides. I see no safety signal that would concern me with moving on to the next subject. Obviously we will continue to watch the safety bloods and ensure that there are no further recrudescences. Dennis/Stephan, what do you think?

From: Anand Odedra <Anand.Odedra@qimrberghofer.edu.au>
Date: Thursday, 8 June 2017 at 04:40
To: Rebecca Watts <Rebecca.Watts@qimrberghofer.edu.au>, me <j.mccarthy@uq.edu.au>, Joerg Moehrle <moehrlej@mmv.org>, Stephan Chalon <chalons@mmv.org>, Dennis Shanks <dennis.shanks@defence.gov.au>
Cc: "p.morgan@qpharm.com.au" <p.morgan@qpharm.com.au>, Silvana Sekuloski <Silvana.Sekuloski@qimrberghofer.edu.au>, Paul Griffin <P.Griffin@qpharm.com.au>, "m.armstrong@qpharm.com.au" <m.armstrong@qpharm.com.au>, Laura Cascales <Laura.Cascales@qimrberghofer.edu.au>
Subject: RE: QP16C14 K13 SRT meeting and second participant

Dear all

please see attached SRT meeting slides.

As always I am happy to answer any questions.

Thanks

Anand

QP16C14 K13

AN EXPERIMENTAL STUDY TO CHARACTERISE THE *IN VIVO* SAFETY AND INFECTIVITY OF A *PLASMODIUM FALCIPARUM* Cam3.II^{R539T} (K13) ARTEMISININ-RESISTANT ISOLATE IN HEALTHY PARTICIPANTS

Safety Review Team meeting 8th June 2017

- **Principal Investigator : James McCarthy**
- **Members of SRT- James McCarthy, Stephan Chalon, Dennis Shanks**
- **Sub-investigator: Anand Odedra, Mark Armstrong, Paul Griffin**
- **Sponsor: Medicines for Malaria Venture**
- **Clinical Study Centre: Q-Pharm**
- **Slides by Anand Odedra**

Q-Pharm

QIMR Berghofer Medical Research Institute

MMV Medicines for Malaria Venture

Study design

- Study duration approximately 3 months
- Day 0 Inoculated *P. falc K13 R539T* ~2,800 parasites
- Day 1-3 daily phone call
- From Day 4 PCR daily until positive
- Once Positive AM/PM PCR until confinement/treatment
- Expected admission day 8
- 72 hour Confinement
- AM/PM PCR post Confinement until:

-PCR approx=<500 can reduce to Once daily PCR

-PCR approx=<200 can reduce to alternate day PCR

- Then 10, 28 and 84 days post treatment
- Additional PCR at investigator’s discretion

Q-Pharm

QIMR Berghofer Medical Research Institute

MMV Medicines for Malaria Venture

Treatment thresholds

Artesunate

- Parasitaemia ≥5,000 parasites/mL or clinical symptom score >6 or investigator discretion
- Admission to Q-pharm, for 72 hours

Eurartesim

- If clinically indicated
- 2 consecutive qPCR time-points show a decreased in parasitaemia of less then 20% of baseline 72 hours post artesunate Rx
- Recrudescence of parasitaemia occurs (count ≥5,000 parasites/mL and a 2-fold increase within 48 hours, or if clinical symptom score >6)
- Investigator discretion

Malarone® at day 26±3, primaquine if gametocytes detected

Q-Pharm
QIMR Berghofer Medical Research Institute
MMV Medicines for Malaria Venture

Analysis Schedule: Blood sampling – PCR, PK, safety bloods

Malaria qPCR-

Daily from day 4 post inoculation, and twice daily from detection to admission

Pre dose baseline (inpatient)

Post dose (inpatient) 4, 8, 12, 16, 24, 30, 36, 48, 60, and 72 hours

(outpatient) BD qPCR until <500 then daily, alt day if <200 (investigator discretion)

Pharmacokinetics (artesunate and dihydroartemisin)-

Post dose (inpatient) 0.25, 0.5, 1, 1.5, 2, 2.5, 3, 4, 6, 8, 10, and 12 hours

Haematology and Biochemistry-

Screening (baseline), Day -3 to -1 safety visit if required, on admission, prior to exit from confinement (72 hours post artesunate Rx), Day 14±2, Day 18±2, prior to piperaquine Rx (if required), 3 days post piperaquine Rx (or next visit), prior to Malarone® Rx, after Malarone®, Day28±3 or early termination visit

Q-Pharm

QIMR Berghofer Medical Research Institute

MMV Medicines for Malaria Venture

Pf IBSM Challenge - Design

Population	Artesunate	Rescue Medication
Inoculum *Pf* K13<br><br>2 cohorts<br>n = 1 per cohort	**2mg/kg dose (50mg oral tablets)**	Eurartesim<br>Malarone<br>Primaquine<br><br>In case of failed clearance, recrudescence<br>Routine Malarone at ~D26<br>In case of gametocytemia

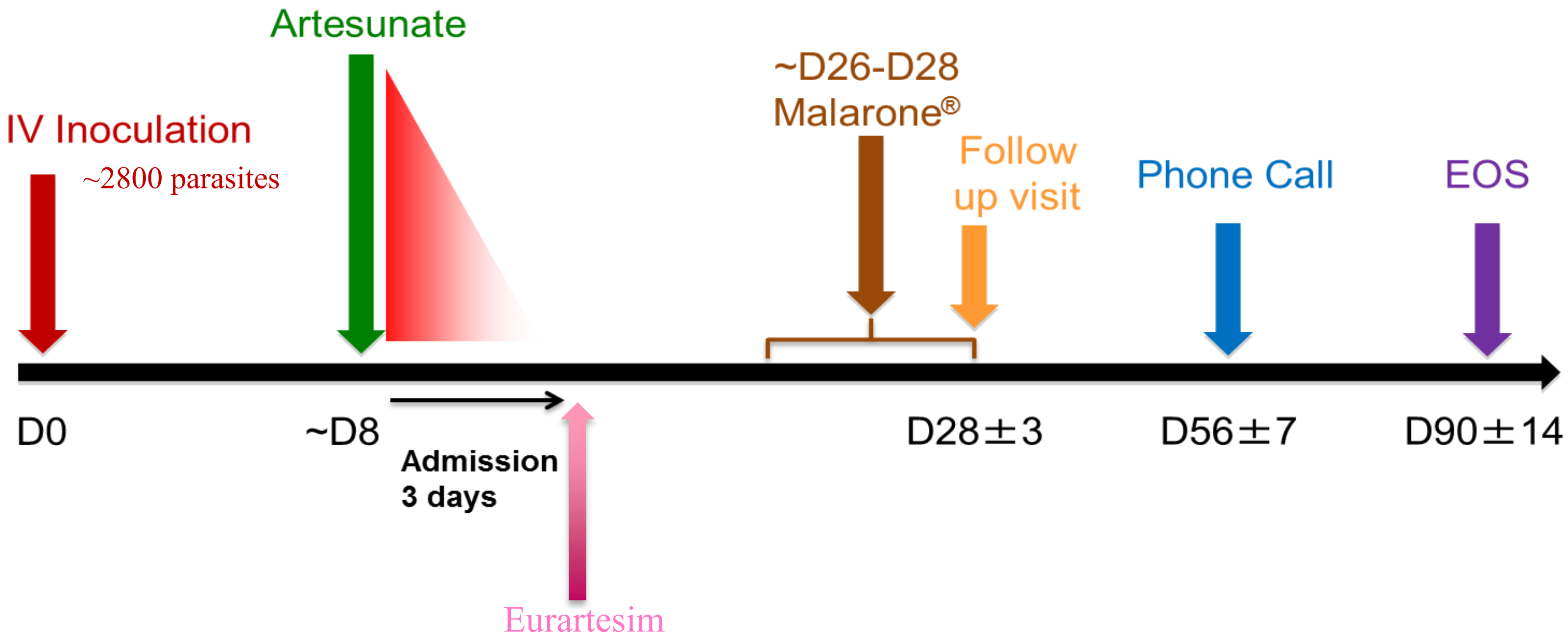

Q-Pharm
QIMR Berghofer Medical Research Institute
MMV Medicines for Malaria Venture

Schedule of events

Inoculum K13

Artesunate

Eurartesim (if required)

Malarone® (and Primaquine if required)

Day	-28 to -3	0	4	5	6	7	~8	9	10	11	12	13	14	X	18	X	26±3	28±3	D56±7	D90±14
Outpatient	X	X	X	X	X	X			X	X	X	X	X		X		X	X		X
Confinement																				
PK							XXXXXXXX X													
qPCR		X	X	X	X	X	XXXXXXXX X		X	X	X	X						X		
Gametocyte qRT-PCR										X	X							X		
Haematology and Biochemistry	X	X					X (admission and exit)		X	X			X		X					
Comments																			Phone call	

screening

- 4 Subjects passed screening
- 1 subject inoculated
- 23M
- No significant PMHX
- Normal examination/Urinalysis/bloods
- ECG-sinus bradycardia (physiological)

Q-Pharm
QIMR Berghofer Medical Research Institute
MMV Medicines for Malaria Venture

PCR Data K13 Artesunate

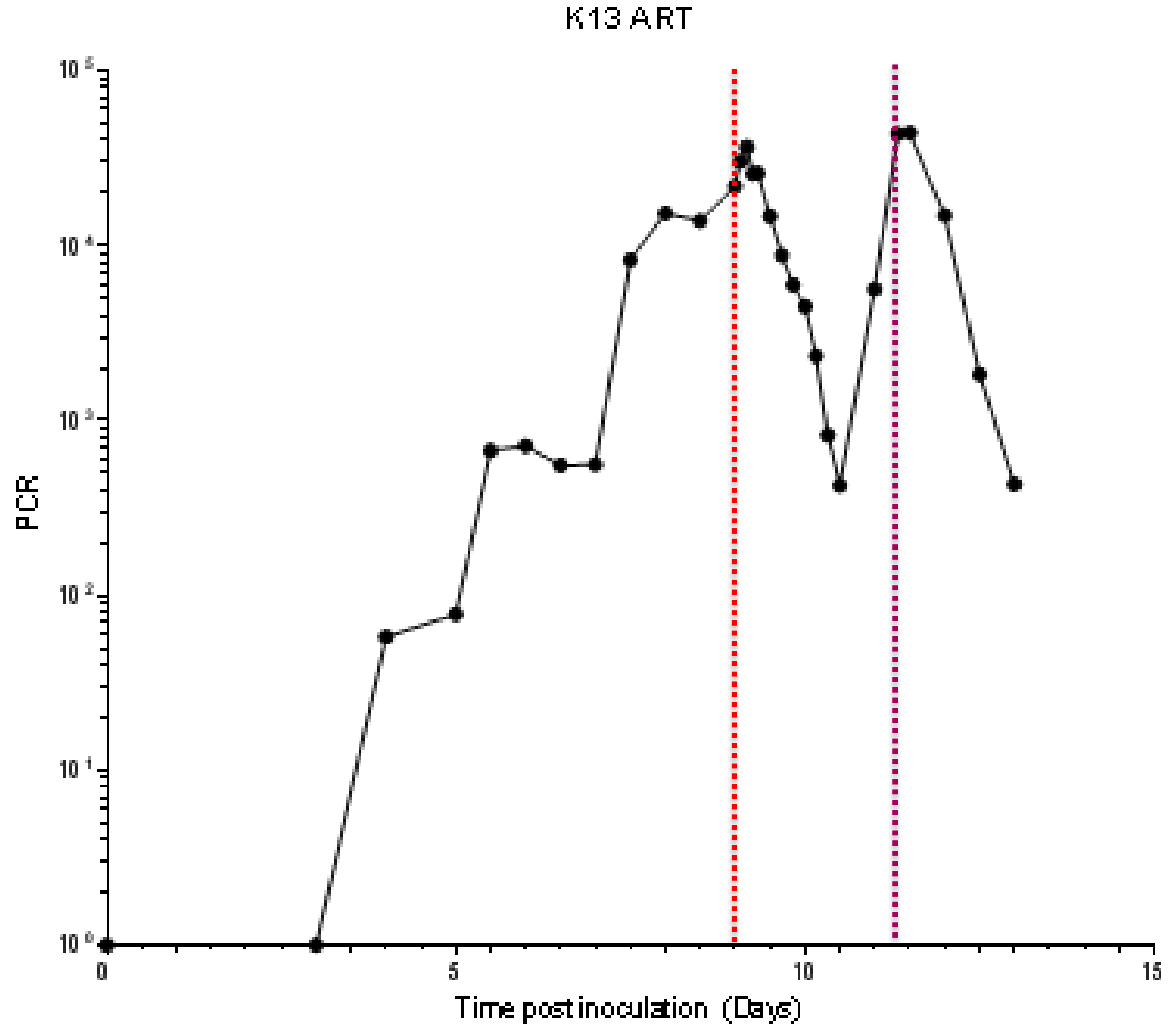

Q-Pharm
QIMR Berghofer Medical Research Institute
MMV Medicines for Malaria Venture

PCR Data K13 Artesunate vs 3D7 Artemether/lumefantrine

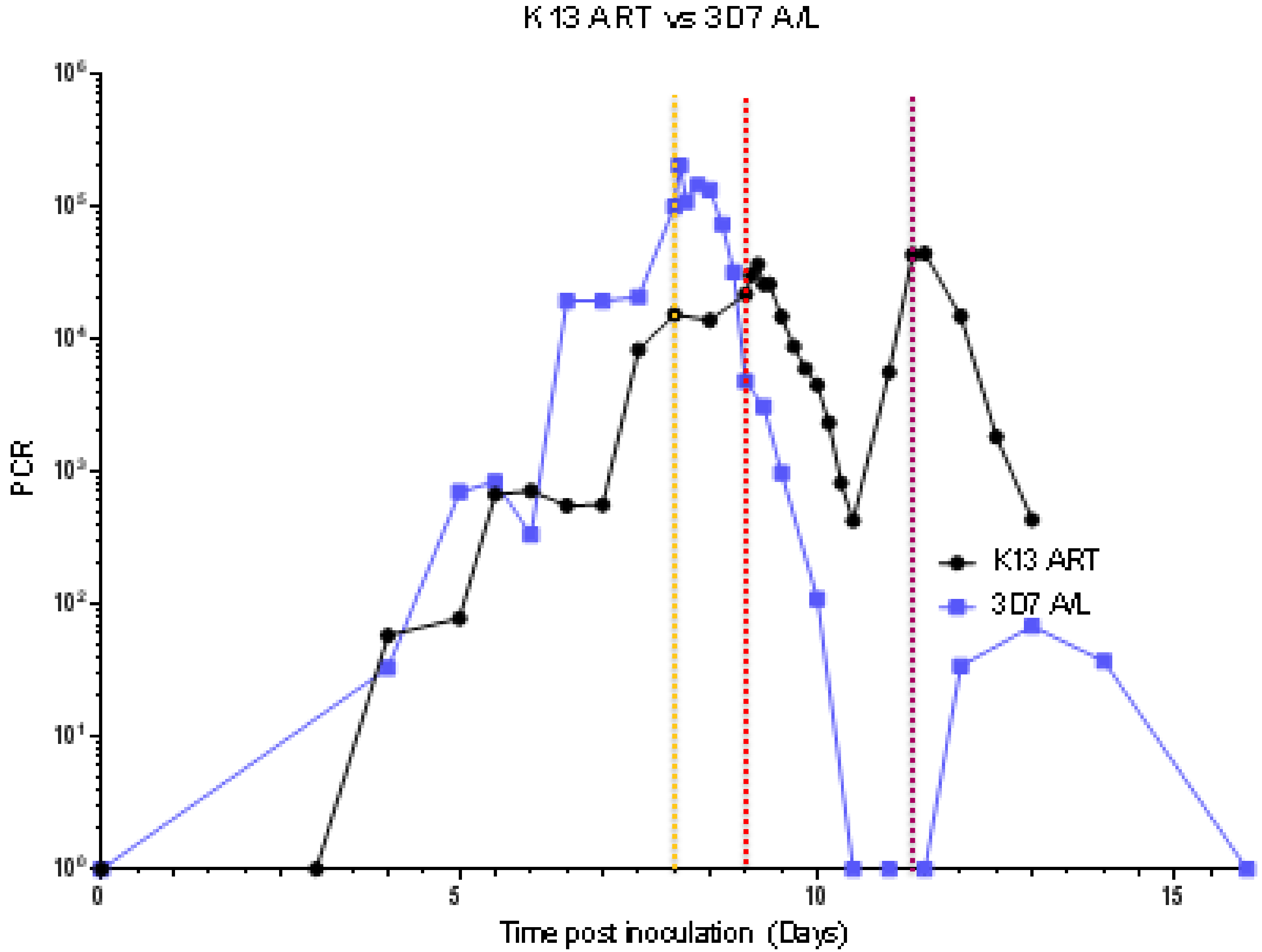

Outcome

- Inoculated 24/May/17
- Inoculum PCR 27,522
- PCR positive day 4 (day 3 PCR negative)
- First PCR >5,000 Day 7PM result became available Day 8 PM
- Treated with Artesunate day 9 based on PCR.
- Parasitemia

-pre Artesunate PCR: 21,871

- 4hrs post artesunate PCR: 36,668

-Nadir Post artesunate PCR: 426

-Pre Eurartesim: 43,469 (Rx initiated at first evidence of recrudescence PCR >5,000)

Q-Pharm

QIMR Berghofer Medical Research Institute

MMV Medicines for Malaria Venture

AEs/Investigations

- Sore throat/Headache/ myalgia/arthralgia/Fatigue/malaise/chills/runny nose (all grade 1)
- Neutropaenia (1.23) CTCAE grade 2 (LLN=1.5)
- Leukopaenia (3.0) grade 1
- Lymphopaenia (0.89) grade 1
- ALT 227 CTCAE Grade 3 (>5xULN)
- AST 159 CTCAE Grade 2 (>3xULN)
- Platelets 139
- Normal HB so far
- Normal observations throughout/Highest temp 37.4
- Normal examination throughout
- Physiological bradycardia (Climber/yoga) otherwise normal ECGs
- No SAEs

Q-Pharm
QIMR Berghofer Medical Research Institute
MMV Medicines for Malaria Venture

ALT

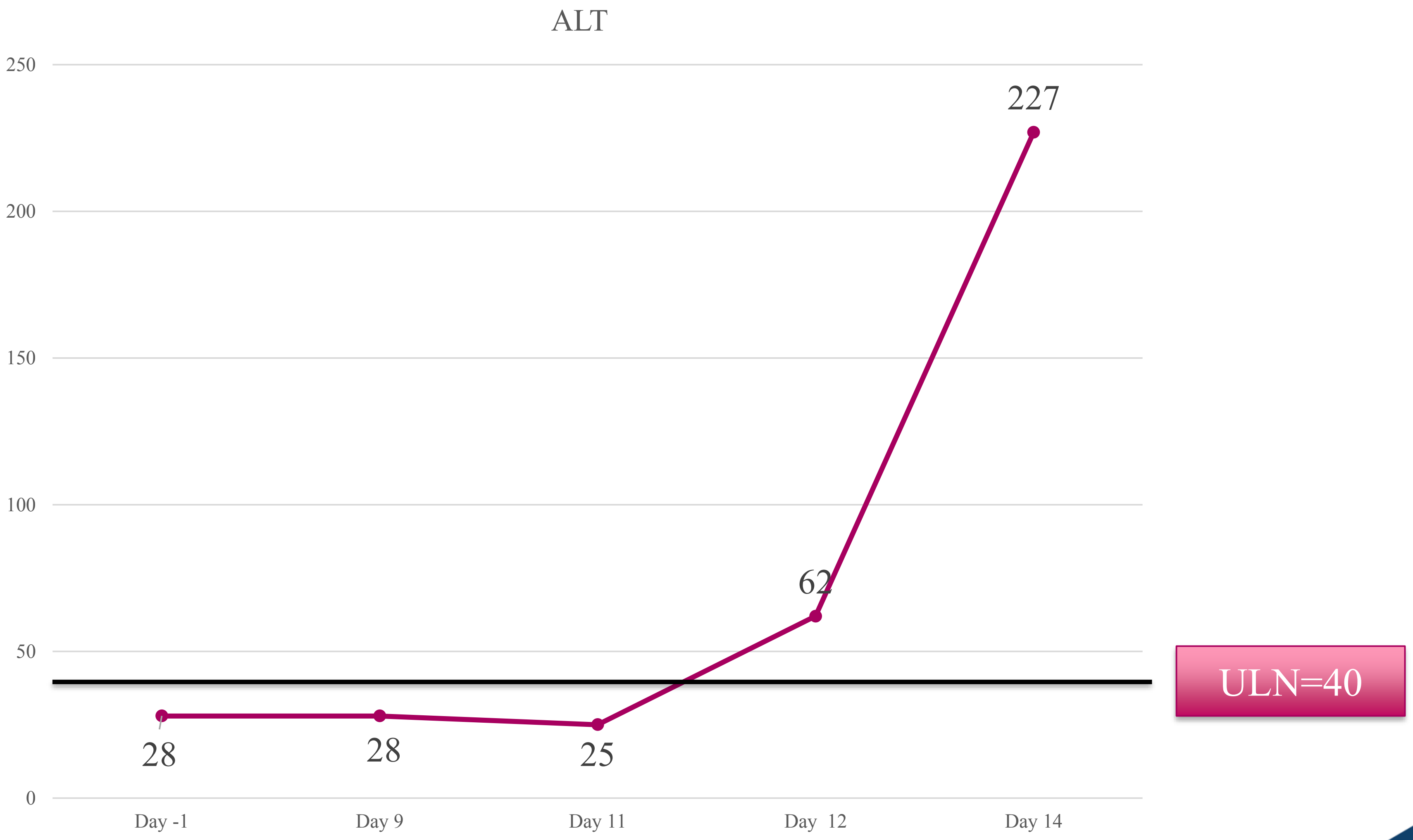
ALT
250
200
150
100
50
0
227
62
28
28
25
ULN=40
Day -1
Day 9
Day 11
Day 12
Day 14

AST

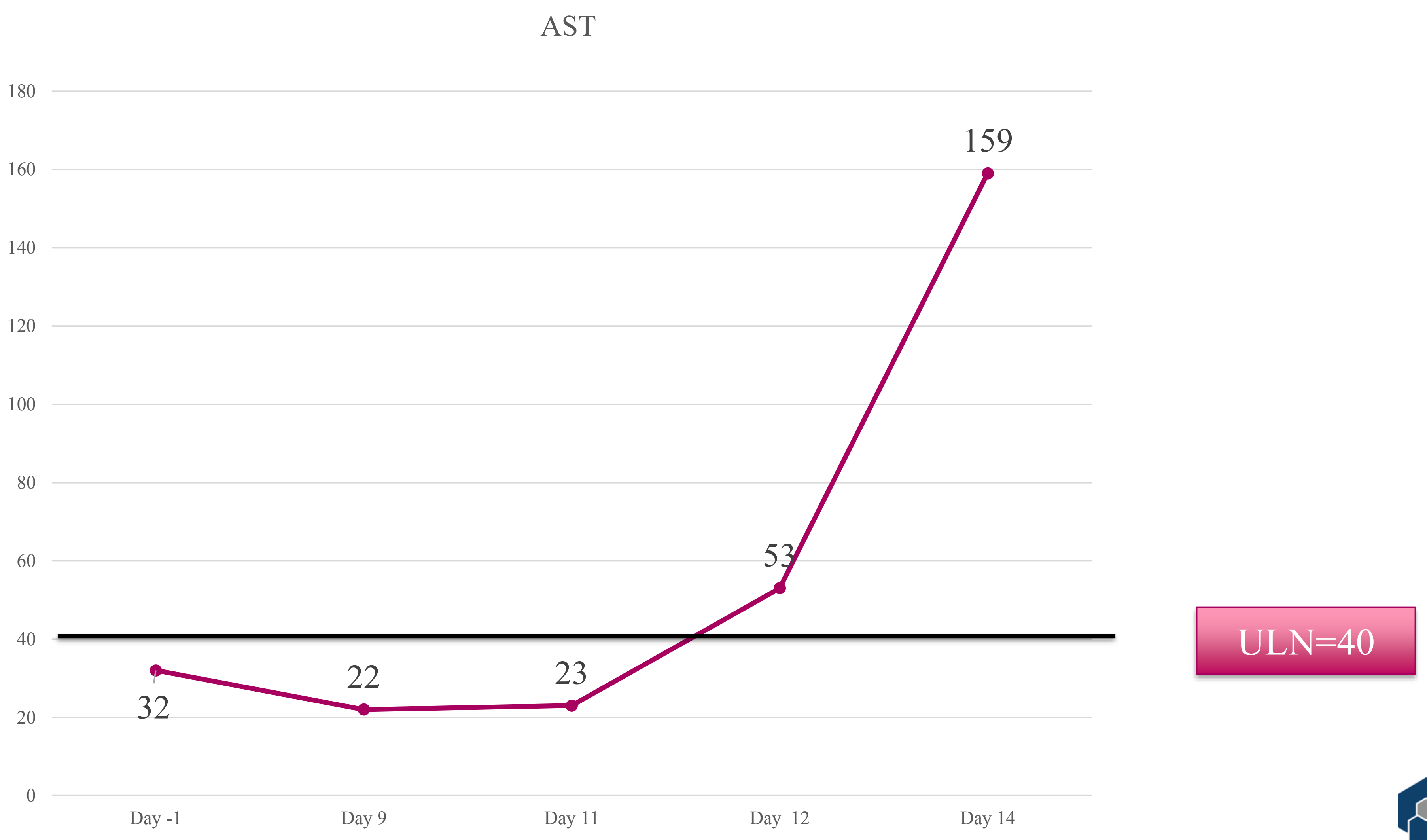

AST
180
160
140
120
100
80
60
40
20
0
159
53
32
22
23
Day -1
Day 9
Day 11
Day 12
Day 14
ULN=40

Bilirubin

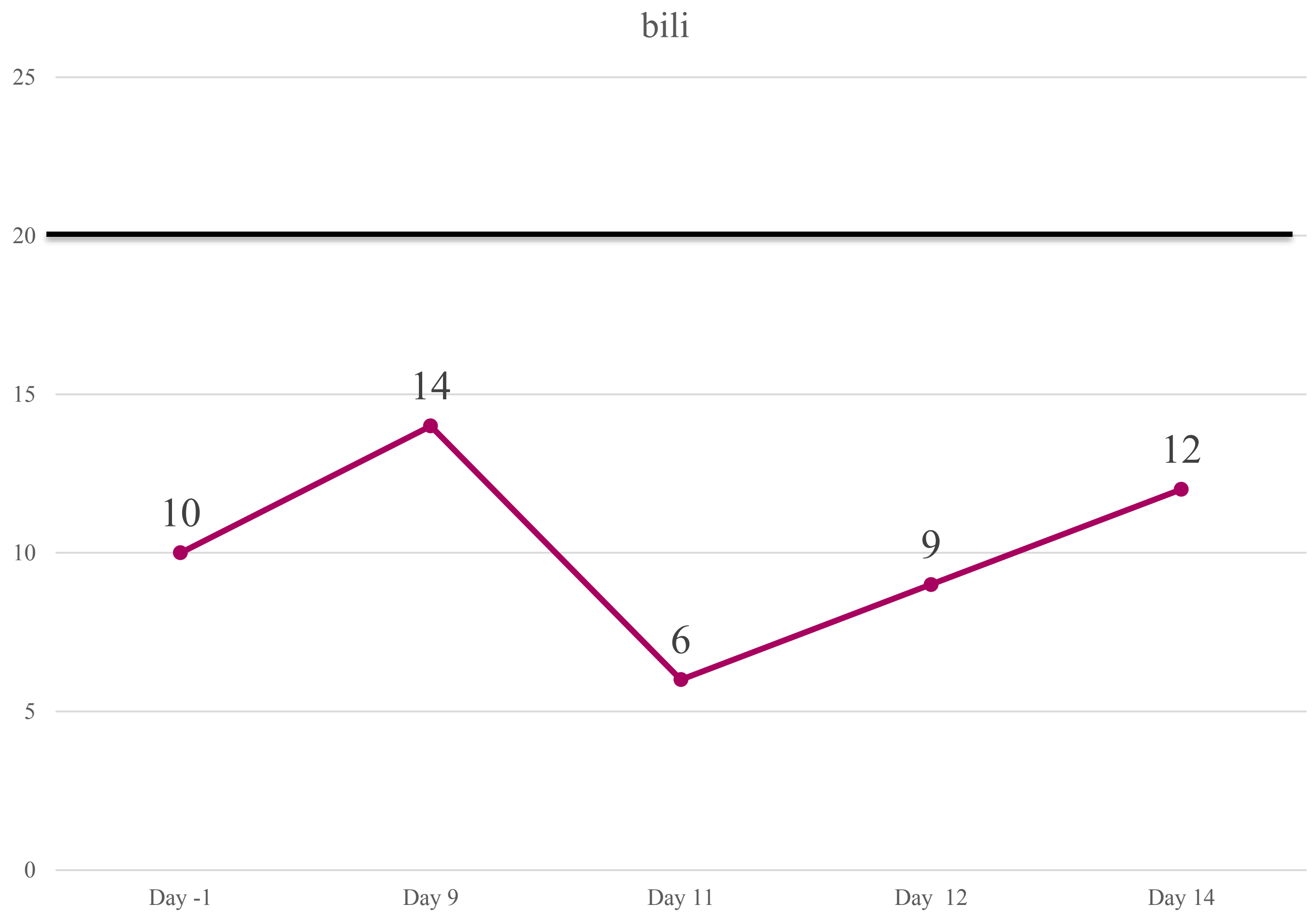

ULN=20

Clinical scores

- D0 to D8 CS=0
- D9 AM CS=1 (headache)
- D9 PM CS=0
- D10 AM/PM CS=0
- D11 AM/PM CS=0
- D12 AM CS=0
- D12 PM CS=6 (headache/myalgia/arthralgia/fatigue/malaise/chills)
- D13 AM CS=5 (headache/myalgia/arthralgia/fatigue/malaise)
- D14 AM CS=0

- Subject described all symptoms as mild and was happy with progress of trial

Medication use

- Artesunate
- Eurartesim
- Paracetamol 1g stat only
- Ibuprofen 400mg stat only

Conclusion

- Continue to monitor Transaminases closely for resolution and feedback to SRT. Repeat bloods D16 (tomorrow)
- Apart from transaminitis no specific clinical concerns
- Continue to monitor as planned especially PCR for dormancy and gametocytes-
- Continue with current schedule for cohort 2
- Highlight any future concerns

Q-Pharm
QIMR Berghofer Medical Research Institute
MMV Medicines for Malaria Venture

Appendix 16.2 Subject Data Listings

16.2.1 Study enrolment and completion/discontinuation

Listing 16.2.1.1
Study Enrolment and Completion/Discontinuation

Subject Number	Date of Consent	Time of Consent	Did Subject Complete Study?	Date of Inoculum Dose	Date of Artesunate Dose	Study Day of Artesunate Dose	Date of Piperaquine-DHA (Eurartesim) Dose	Study Day of Piperaquine-DHA (Eurartesim) Dose	Date of Final Study Participation	Study Day of Final Study Participation	Reason for Study Discontinuation
R001	09-May-2017	10:51	Yes	24-May-2017	02-Jun-2017	Day 9	04-Jun-2017	Day 11	11-Aug-2017	Day 79	
R002	27-Apr-2017	11:30	Yes	14-Jun-2017	23-Jun-2017	Day 9	25-Jun-2017	Day 11	12-Sep-2017	Day 90	

Notes:
Date of Inoculum dose is when Study Day = 0

16.2.2 Protocol deviations

Listing 16.2.2.1
Protocol Deviations

Subject Number	PD No.	Visit	Date of Deviation	Study Day	Day Relative to Artesunate	Protocol Deviation Category	Description of Protocol Deviation	Action Taken
There were no protocol deviations for this study								

Notes:
Protocol Deviation text presented as verbatim.
PD no. = Protocol deviation number.
Study Day is the number of days relative to day of administration of the inoculum, where Study Day = 0 for inoculum dosing day.
Day relative to Artesunate is the number of days relative to day of administration of Artesunate, where the day of first dose = 1.

16.2.3 Subjects excluded from the efficacy analysis

QP16C14: K13 Pilot Clinical Study Report Appendices

Not applicable.

16.2.4 Demographic and baseline data

16.2.4.1 Demography

Listing 16.2.4.1
Demographics

Subject Number	Date of Birth	Age (years)	Sex	Race
R001	15-Jul-1993	23	Male	White
R002	21-Oct-1988	28	Male	Asian

Notes:
Age is that at Screening visit.

16.2.4.2 Medical history

16.2.4.2
Medical History

Subject Number	Medical History Number	Medical Condition	Date of Diagnosis	Date of Resolution
R001	1	Fractured right wrist	UN-UN-2005	UN-UN-2005
	2	Surgery for fractured wrist	UN-UN-2005	UN-UN-2005
	3	Hayfever	UN-UN-2011	Ongoing
	4	Lactose intolerant	UN-UN-2013	Ongoing

Notes:
Medical Condition is presented as verbatim.
UN = Unknown; UNKK = Unknown.

16.2.4.5 Red cell allo-antibody

Listing 16.2.4.5
Red Cell Allo-Antibody

Subject Number	Visit	Date of Measurement	Time of Measurement	Study Day	Day Relative to Artesunate	Result
R001	Screening	09-May-2017	11:52	-15	-24	Negative
	Safety monitoring	21-Jun-2017	8:38	Day 28	20	Negative
	End of Study	11-Aug-2017	8:18	Day 79	71	Negative
R002	Screening	07-Jun-2017	9:58	-7	-16	Negative
	Safety monitoring	12-Jul-2017	9:30	Day 28	20	Negative
	End of Study	12-Sep-2017	9:43	Day 90	82	Negative

Notes:
Study Day is the number of days relative to day of administration of the inoculum, where Study Day = 0 for inoculum dosing day.
Day relative to Artesunate is the number of days relative to day of administration of Artesunate, where the day of first dose = 1.

16.2.4.6 Serology and special tests

Listing 16.2.4.6
Serology and Special Tests

Subject Number	Visit	Date of Measurement	Time of Measurement	Study Day	Day Relative to Artesunate	Parameter	Result
R001	Screening	09-May-2017	11:52	-15	-24	HIV Antibody	Negative
R001	Screening	09-May-2017	11:52	-15	-24	Hepatitis B Virus Core Antibody	Negative
R001	Screening	09-May-2017	11:52	-15	-24	Hepatitis B Virus Surface Antigen	Negative
R001	Screening	09-May-2017	11:52	-15	-24	Hepatitis C Virus IgG Antibody	Negative
R001	Safety monitoring	21-Jun-2017	8:38	Day 28	20	HIV Antibody	Weak positive (NCS)
R001	Safety monitoring	21-Jun-2017	8:38	Day 28	20	Hepatitis B Virus Core Antibody	Negative
R001	Safety monitoring	21-Jun-2017	8:38	Day 28	20	Hepatitis B Virus Surface Antigen	Negative
R001	Safety monitoring	21-Jun-2017	8:38	Day 28	20	Hepatitis C Virus IgG Antibody	Negative
R001	Unscheduled visit	29-Jun-2017	8:37	Day 36	28	HIV Antibody	Weak positive (NCS)
R001	End of Study	11-Aug-2017	8:18	Day 79	71	HIV Antibody	Negative
R002	Screening	07-Jun-2017	9:58	-7	-16	HIV Antibody	Negative
R002	Screening	07-Jun-2017	9:58	-7	-16	Hepatitis B Virus Core Antibody	Negative
R002	Screening	07-Jun-2017	9:58	-7	-16	Hepatitis B Virus Surface Antigen	Negative
R002	Screening	07-Jun-2017	9:58	-7	-16	Hepatitis C Virus IgG Antibody	Negative
R002	Safety monitoring	12-Jul-2017	9:30	Day 28	20	HIV Antibody	Negative
R002	Safety monitoring	12-Jul-2017	9:30	Day 28	20	Hepatitis B Virus Core Antibody	Negative
R002	Safety monitoring	12-Jul-2017	9:30	Day 28	20	Hepatitis B Virus Surface Antigen	Negative
R002	Safety monitoring	12-Jul-2017	9:30	Day 28	20	Hepatitis C Virus IgG Antibody	Negative

Notes:
Study Day is the number of days relative to day of administration of the inoculum, where Study Day = 0 for inoculum dosing day.
Day relative to Artesunate is the number of days relative to day of administration of Artesunate, where the day of first dose = 1.

16.2.4.7 Urine drug screen and alcohol breath test

Listing 16.2.4.7
Positive Urine Drug and Alcohol Breath Test

Subject Number	Visit	Date of Measurement	Time of Measurement	Study Day	Day Relative to Artesunate	Test	Category of Test
No positive urine drug or alcohol breath test reported in this study							

Notes:
Study Day is the number of days relative to day of administration of the inoculum, where Study Day = 0 for inoculum dosing day.
Day relative to Artesunate is the number of days relative to day of administration of Artesunate, where the day of first dose = 1.
Urine drug and alcohol breath test were performed for all participants as per the protocol. Only positive results are recorded in this listing.

16.2.4.8 G6PD

Listing 16.2.4.8
G6PD

Subject Number	Visit	Date of Measurement	Time of Measurement	Study Day	Day Relative to Artesunate	Parameter	Unit	Normal Range	Result
R001	Screening	09-May-2017	11:52	-15	-24	Glucose-6-Phosphate Dehydrogenase	U/L		2353
R001	Screening	09-May-2017	11:52	-15	-24	Glucose-6-Phosphate Dehydrogenase/HB	U/g Hb	[7 - 20.5]	16.3
R002	Screening	07-Jun-2017	9:58	-7	-16	Glucose-6-Phosphate Dehydrogenase	U/L		1776.4
R002	Screening	07-Jun-2017	9:58	-7	-16	Glucose-6-Phosphate Dehydrogenase/HB	U/g Hb	[7 - 20.5]	13.8

Notes:
Study Day is the number of days relative to day of administration of the inoculum, where Study Day = 0 for inoculum dosing day.
Day relative to Artesunate is the number of days relative to day of administration of Artesunate, where the day of first dose = 1.

16.2.4.10 Body weight, height and BMI

Listing 16.2.4.10
Body Weight, Height and BMI

Subject Number	Visit	Date of Test	Study Day	Day Relative to Artesunate	Test (unit)	Result
R001	Screening	09-May-2017	-15	-24	Weight (kg)	77.7
R001	Screening	09-May-2017	-15	-24	Height (cm)	185.0
R001	Screening	09-May-2017	-15	-24	Body Mass Index (kg/m^2)	22.7
R002	Screening	07-Jun-2017	-7	-16	Weight (kg)	71.3
R002	Screening	07-Jun-2017	-7	-16	Height (cm)	176.0
R002	Screening	07-Jun-2017	-7	-16	Body Mass Index (kg/m^2)	23.0

Notes:
Study Day is the number of days relative to day of administration of the inoculum, where Study Day = 0 for inoculum dosing day.
Day relative to Artesunate is the number of days relative to day of administration of Artesunate, where the day of first dose = 1.
BMI = Body Mass Index.

16.2.4.11 Concomitant medications

Listing 16.2.4.11
Concomitant Medications

Subject Number	CM. No.	Medication Name Verbatim	Preferred Name	ATC Class	Start Date	Start Time	Start Study Day	End Date	End Time	End Study Day	Dose	Unit	Route	Freq.	Indication
R001	1	Paracetamol	Paracetamol	Analgesics	05-Jun-2017	21:30	Day 12	05-Jun-2017	21:30	Day 12	500	mg	Oral	Stat	Headache/ arthralgia/ myalgia
R001	2	Ibuprofen	Ibuprofen	Antiinflammatory and antirheumatic products	06-Jun-2017	12:00	Day 13	06-Jun-2017	12:00	Day 13	200	mg	Oral	Stat	Headache
R001	3	Paracetamol	Paracetamol	Analgesics	15-Jun-2017	18:00	Day 22	15-Jun-2017	18:00	Day 22	500	mg	Oral	Stat	Headache
R002	1	Ibuprofen	Ibuprofen	Antiinflammatory and antirheumatic products	24-Jun-2017	13:33	Day 10	24-Jun-2017	13:33	Day 10	200	mg	Oral	Stat	Chills/ fever
R002	2	Ibuprofen	Ibuprofen	Antiinflammatory and antirheumatic products	24-Jun-2017	21:25	Day 10	24-Jun-2017	21:25	Day 10	200	mg	Oral	Stat	Chills
R002	3	Ibuprofen	Ibuprofen	Antiinflammatory and antirheumatic products	25-Jun-2017	19:09	Day 11	25-Jun-2017	19:09	Day 11	200	mg	Oral	Stat	Headache/ fever
R002	4	Ibuprofen	Ibuprofen	Antiinflammatory and antirheumatic products	26-Jun-2017	9:51	Day 12	26-Jun-2017	9:51	Day 12	200	mg	Oral	Stat	Headache
R002	5	Ibuprofen	Ibuprofen	Antiinflammatory and antirheumatic products	26-Jun-2017	20:00	Day 12	26-Jun-2017	20:00	Day 12	200	mg	Oral	Stat	Headache/ chills

Notes:
Study Day is the number of days relative to day of administration of the inoculum, where Study Day = 0 for inoculum dosing day.
Medications with a start date prior to administration of inoculum were also classified as prior medications.
CM. No. = Concomitant medication number; UN = Unknown; UNKK = Unknown; Once = Once per period; QD = Once a day; BID = Twice a day; QID = Four times a day; STAT = Immediately; Freq. = Frequency.

16.2.5 Compliance and drug concentration data

16.2.5.1 Malaria challenge agent administration

Listing 16.2.5.1
Malaria Inoculum Administration

Subject Number	Inoculum Name	Dose	Unit	Visit	Date Administered	Time Administered	Study Day	Day Relative to Artesunate	Was inoculum administered as per the protocol?
R001	*P. falciparum* K13	~ 2800	Viable parasites/2 mL	Day 0	24-May-2017	10:19	Day 0	-9	Yes
R002	*P. falciparum* K13	~ 2800	Viable parasites/2 mL	Day 0	14-Jun-2017	10:19	Day 0	-9	Yes

Notes:
P. falciparum K13 = *Plasmodium falciparum* K13.
Study Day is the number of days relative to day of administration of the inoculum, where Study Day = 0 for inoculum dosing day.
Day relative to Artesunate is the number of days relative to day of administration of Artesunate where the day of first dose = 1.

16.2.5.2 Study drug administration

Listing 16.2.5.2
Study Drug Administration

Subject Number	Treatment	Dose	Unit	Lot Number	Date Administered	Time Administered	Study Day	Day Relative to Artesunate	Was Drug Administered as Per the Protocol?
R001	Artesunate	150	mg	AS160501P	02-Jun-2017	8:21	Day 9	1	Yes
R001	Eurartesim	960 piperaquine tetraphosphate/ 120 dihydroartemisinin	mg	160068	04-Jun-2017	16:20	Day 11	3	Yes
R002	Artesunate	150	mg	AS160501P	23-Jun-2017	9:19	Day 9	1	Yes
R002	Eurartesim	960 piperaquine tetraphosphate/ 120 dihydroartemisinin	mg	160068	25-Jun-2017	16:02	Day 11	3	Yes

Notes:
Study Day is the number of days relative to day of administration of the inoculum, where Study Day = 0 for inoculum dosing day.
Day relative to Artesunate is the number of days relative to day of administration of Artesunate where the day of first dose = 1.

16.2.5.3 Rescue medication administration

Listing 16.2.5.3
Rescue Medication Administration

Subject Number	Treatment	Dose	Unit	Route of Administration	Date of Administration	Time of Administration	Study Day	Day Relative to Artesunate	Was Drug Administered as Per the Protocol?
R001	Malarone	4	Tablet	Oral	19-Jun-2017	8:09	Day 26	18	Yes
R001	Primacin	6	Tablet	Oral	19-Jun-2017	8:13	Day 26	18	Yes
R001	Malarone	4	Tablet	Oral	20-Jun-2017	9:20	Day 27	19	Yes
R001	Malarone	4	Tablet	Oral	21-Jun-2017	8:25	Day 28	20	Yes
R002	Malarone	4	Tablet	Oral	10-Jul-2017	8:57	Day 26	18	Yes
R002	Primacin	6	Tablet	Oral	10-Jul-2017	9:07	Day 26	18	Yes
R002	Malarone	4	Tablet	Oral	11-Jul-2017	9:02	Day 27	19	Yes
R002	Malarone	4	Tablet	Oral	12-Jul-2017	9:19	Day 28	20	Yes

Notes:
Study Day is the number of days relative to day of administration of the inoculum, where Study Day = 0 for inoculum dosing day.
Day relative to Artesunate is the number of days relative to day of administration of Artesunate where the day of first dose = 1.
UN = Unknown.

16.2.5.4 Artesunate and dihydroartemisinin (DHA) plasma concentrations

Analytical Chemistry Unit

Dept of Chemical Pathology, Pathology Queensland

Level 3, Block 7, Royal Brisbane & Women's Hospital

Herston Road, HERSTON Qld 4029, Australia

Health Services Support Agency | Queensland Health

Artesunate and DHA Concentrations – Time Data

K13 Pilot Participant No: R001

Sampling Interval	Actual sampling time	Date	Auslab No	Artesunate ug/L	DHA ug/L
PRE-DOSE	8:13:00	02/06/2017	908707267	0	0
15 MIN	8:37:00	02/06/2017	908707256	22.1	0
30 MIN	8:51:00	02/06/2017	908707240	74.6	30.3
1 HOUR	9:21:00	02/06/2017	908707234	107.5	284.3
1.5 HOUR	9:51:00	02/06/2017	908707223	24.7	316.2
2 HOUR	10:21:00	02/06/2017	908707212	0.8	177.4
2.5 HOUR	10:51:00	02/06/2017	908707201	0	134.5
3 HOUR	11:21:00	02/06/2017	908707196	0	63.1
4 HOUR	12:23:00	02/06/2017	908707185	0	0
6 HOUR	14:22:00	02/06/2017	908707179	0	0
8 HOUR	16:21:00	02/06/2017	908707168	0	0
10 HOUR	18:21:00	02/06/2017	908707157	0	0
12 HOUR	20:21:00	02/06/2017	908707141	0	0

Analysed by: Brett McWhinney

Checked by: Leanne Foyn

Date: 19/06/2017

Assay date: 15/06/2017

Sample set Name: Art DHA 150617 pilot

Analytical Chemistry Unit

Dept of Chemical Pathology, Pathology Queensland

Level 3, Block 7, Royal Brisbane & Women's Hospital

Herston Road, HERSTON Qld 4029, Australia

Health Services Support Agency | Queensland Health

Artesunate and DHA Concentrations – Time Data

K13 Pilot Participant No: R002

Sampling Interval	Actual sampling time	Date	Auslab No	Artesunate ug/L	DHA ug/L
PRE-DOSE	9:09:00	23/06/2017	798014755	0	0
15 MIN	9:34:00	23/06/2017	798014744	26.7	4.7
30 MIN	9:49:00	23/06/2017	798017139	97.8	100.9
1 HOUR	10:19:00	23/06/2017	798017044	31.9	543.1
1.5 HOUR	10:49:00	23/06/2017	798016994	0.5	372.1
2 HOUR	11:20:00	23/06/2017	798016949	0.0	282.1
2.5 HOUR	11:49:00	23/06/2017	798017055	0.0	155.2
3 HOUR	12:19:00	23/06/2017	798017000	0.0	84.3
4 HOUR	13:19:00	23/06/2017	798016953	0.0	37.6
6 HOUR	15:19:00	23/06/2017	798017066	0.0	24.6
8 HOUR	17:19:00	23/06/2017	798017011	0.0	20.8
10 HOUR	19:19:00	23/06/2017	798016962	0.0	5.2
12 HOUR	21:19:00	23/06/2017	798017077	0.0	0.0

Analysed by: Brett McWhinney **Checked by: Leanne Foyn**

Date: 30/06/2017

Assay date: 28/06/2017 **Sample set Name: Art DHA 280617 pilot 2**

16.2.6 Parasitaemia and parasite lifecycle stage qPCR/qRT-PCR data

Queensland Paediatric Infectious Diseases Laboratory

Quantitative Malaria Nucleic Acid Amplification Results

Level 8, Centre for Children's Health Research
62 Graham Street, South Brisbane Q4101

Phone: (07) 3069 7464

***Q-Pharm** Identifier*		***QP16C14 K13 COHORT 1/S004***		
Randomisation number:		R001		
Initials:		[redacted]		
***Patient Date of Birth**:*		[redacted]		
Subject results		Daily results	Pfs25 Daily results	Psf25 Retrospective results
time points	***collection time***	***Parasites /500ul of packed RBC***	***Copies/ml***	***Copies/ml***
**Day 0**	24/05/17 0938	ND	NT	ND
**Day 3**	27/05/17 0803	ND	NT	NT
**Day 4**	28/05/17 0828	58	NT	NT
**Day 5AM**	29/05/17 0759	78	NT	NT
**Day 5PM**	29/05/17 1846	674	NT	NT
**Day 6AM**	30/05/17 0744	715	NT	NT
**Day 6PM**	30/05/17 1842	554	NT	NT
**Day 7AM**	31/05/17 0747	561	NT	NT
**Day 7PM**	31/05/17 1849	8,292	NT	NT
**Day 8AM**	01/06/17 0812	15,301	NT	NT
**Day 8PM**	01/06/17 1930	13,847	NT	NT
**Day 9 (Adm+0hrs)**	02/06/17 0813	21,871	NT	NT
**Day 9 (Adm+2hrs)**	02/06/17 1021	30,304	NT	NT
**Day 9 (Adm+4hrs)**	02/06/17 1223	36,668	NT	NT
**Day 9 (Adm+6hrs)**	02/06/17 1422	25,846	NT	NT
**Day 9 (Adm+8hrs)**	02/06/17 1622	25,938	NT	NT
**Day 9 (Adm+12hrs)**	02/06/17 2022	14,737	NT	NT
**Day 9 (Adm+16hrs)**	03/06/17 0021	8,805	NT	NT
**Day 9 (Adm+20hrs)**	03/6/17 0421	5,973	NT	NT
**Day 10 (Adm+24hrs)**	03/06/17 0821	4,494	NT	NT
**Day 10 (Adm+28hrs)**	03/06/17 1223	2,340	NT	NT

**Day 10 (Adm+32hrs)**	03/06/17 1621	825	NT	NT
**Day 10 (Adm+36hrs)**	03/06/17 2021	426	NT	NT
**Day 11 (Adm+48hrs)**	04/06/17 0829	5,636	NT	NT
**Day 11 (Adm+56hrs)/Predose**	04/06/17 1606	43,469	NT	NT
**Day 11 (Adm+60hrs)**	04/06/17 2021	44,190	NT	NT
**Day 12 (Adm+72hrs)**	05/06/17 0823	14,896	NT	93
**Day 12 (Adm+84)**	05/06/17 1954	1,835	NT	NT
**Day 13 (Adm+96)**	06/06/17 0810	433	NT	NT
**Day 14**	07/06/17 0754	100	NT	ND
**Day 16**	09/06/17 0744	43	62700	5323
**Day 18**	11/06/17 0744	30	NT	3577
**Day 19**	12/06/17 0750	26	NT	17966
**Day 21**	14/06/17 0756	30	NT	5506
**Day 23**	16/06/17 0741	71	NT	57197
**Day 26**	19/06/17 0757	46	NT	55968
**Day 28**	21/06/17 0838	ND	NT	37458
**Day 79**	11/08/17 0818	ND	NT	NT

NOTES:

- ***Parasite quantification is indicated by fluorescence of the specific oligoprobe hybridized with expected PCR product (real-time PCR). Parasite concentration is calculated using a standard curve and simple linear regression and expressed as parasite number per /500ul of packed red blood cells. Daily results are performed in real time and are tested in duplicate and the average of these results is given above as a final concentration. Retrospective results are carried out on each subject at the end of the study; this enables all time points to be included on one PCR run where possible. Pre-admission specimens are retrospectively tested in duplicate and post admission time points are retrospectively tested in triplicate. The method is based on that described in Rockett et al Malar J. 2011 Feb 28; 10:48.***
- ***ND = Microbial template not detected by PCR; INHIB = PCR reaction inhibited; NT = Testing not performed; NC = specimens not collected***

This assay is a research procedure. It has not been validated for use as a diagnostic tool.

Scientist performing assay:

Printed: 06/12/2018

Signature:

Comments:

The information contained in this report is intended for the named recipients only. It may contain privileged and confidential information. If you are not the intended recipient you must not copy, distribute or take any action in reliance on this report, or disclose any details of this report to any other person, firm or corporation. If you have received this report in error, please notify us immediately.

Dr Julia Clark (Director) julia.clark@health.qld.gov.au
Queensland Paediatric Infectious Diseases Laboratory
L8, CCHR, 62 Graham St, South Brisbane 4101
Ph: (07) 3068 1558

Queensland Paediatric Infectious Diseases Laboratory

Quantitative Malaria Nucleic Acid Amplification Results

Level 8, Centre for Children's Health Research
62 Graham Street, South Brisbane Q4101

Phone: (07) 3069 7464

***Q-Pharm** Identifier*	***QP16C14 K13 COHORT 2/S003***			
Randomisation number:	R002			
Initials:	[redacted]			
***Patient Date of Birth**:*	[redacted]			

Subject results		Daily results	SBP-1 (RSA) Daily results	Pfs25 Daily results	Psf25 Retrospective results
time points	***collection time***	***Parasites /500ul of packed RBC***	***Copies/ml***	***Copies/ml***	***Copies/ml***
**Day 0**	14/06/17 1006	ND	NT	NT	ND
**Day 4AM**	18/06/17 0745	82	NT	NT	NT
**Day 5AM**	19/06/17 0837	95	NT	NT	NT
**Day 5PM**	19/06/17 1931	1,346	NT	NT	NT
**Day 6AM**	20/06/17 0818	2,053	NT	NT	NT
**Day 6PM**	20/06/17 1918	1,701	NT	NT	NT
**Day7AM**	21/06/17 0740	2,919	NT	NT	NT
**Day 7PM**	21/06/17 2243	29,395	NT	NT	NT
**Day 8AM**	22/06/17 0821	34,996	NT	NT	NT
**Day 8PM**	22/06/17 1903	50,225	NT	NT	NT
**Day 9 (Adm+0hrs)**	23/06/17 0909	128,833	NT	NT	NT
**Day 9 (Adm+2hrs)**	23/06/17 1120	208,524	NT	NT	NT
**Day 9 (Adm+4hrs)**	23/06/17 1319	232,065	NT	NT	NT
**Day 9 (Adm+6hrs)**	23/06/17 1519	198,903	NT	NT	NT
**Day 9 (Adm+8hrs)**	23/06/17 1719	163,354	NT	NT	NT
**Day 9 (Adm+12hrs)**	23/06/17 2119	113,320	NT	NT	NT
**Day 9 (Adm+16hrs)**	24/06/17 0119	51,799	NT	NT	NT
**Day 9 (Adm+20hrs)**	24/06/17 0519	57,387	NT	NT	NT
**Day 10 (Adm+24hrs)**	24/06/17 0919	37,750	NT	NT	NT
**Day 10 (Adm+28hrs)**	24/06/17 1319	10,740	NT	NT	NT
**Day 10 (Adm+32hrs)**	24/06/17 1719	4,902	NT	NT	NT

**Day 10 (Adm+36hrs)**	24/06/17 2119	2,110	NT	NT	NT
**Day 11 (Adm+48hrs)**	25/06/17 0919	48,958	NT	NT	NT
**Day 11 (Adm+54hrs)**	25/06/17 1544	307,831	NT	NT	NT
**Day 11 (Adm+60hrs)**	25/06/17 2121	254,262	NT	NT	NT
**Day 12 (Adm+72hrs)**	26/06/17 0923	58,261	NT	NT	NT
**Day 12 (Adm+84hrs)**	26/06/17 2023	8,349	NT	NT	80
**Day 13 (Adm+96hrs)**	27/06/17 0846	944	NT	NT	NT
**Day 13PM**	27/06/17 2030	267	NT	NT	NT
**Day 14AM**	28/06/17 0911	241	NT	NT	86
**Day 15**	29/06/17 0813	75	NT	NT	NT
**Day 18**	02/07/17 0846	33	NT	NT	49,820
**Day 19**	03/07/17 0816	243	NT	NT	NT
**Day 20**	04/07/17 0815	416	NT	NT	529,921
**Day 22**	06/07/17 0816	851	NT	3,206,867	704,487
**Day 26**	10/7/17 0852	7,100	14,900	2,194,100	1,035,949
**Day 27**	11/7/17 1746	5,043	3,800	7,654,000	NT
**Day 28**	12/7/17 0930	1,068	ND	8,290,300	578,676
**Day 29**	13/07/17 0938	144	NT	NT	NT
**Day 30**	14/07/17 0847	105	NT	NT	123,697
**Day 33**	17/07/17 0842	34	NT	NT	NT
**Day 37**	21/07/17 0910	19	NT	NT	NT
**Day 45**	29/07/17 1005	ND	NT	NT	NT
**Day 90**	12/09/17 0943	ND	NT	NT	NT

NOTES:

- ***Parasite quantification is indicated by fluorescence of the specific oligoprobe hybridized with expected PCR product (real-time PCR). Parasite concentration is calculated using a standard curve and simple linear regression and expressed as parasite number per /500ul of packed red blood cells. Daily results are performed in real time and are tested in duplicate and the average of these results is given above as a final concentration. Retrospective results are carried out on each subject at the end of the study; this enables all time points to be included on one PCR run where possible. Pre-admission specimens are retrospectively tested in duplicate and post admission time points are retrospectively tested in triplicate. The method is based on that described in Rockett et al Malar J. 2011 Feb 28; 10:48.***
- ***ND = Microbial template not detected by PCR; INHIB = PCR reaction inhibited; NT = Testing not performed; NC = specimens not collected***

This assay is a research procedure. It has not been validated for use as a diagnostic tool.

Scientist performing assay:

Printed: 06/12/2018

Signature:

Comments:

The information contained in this report is intended for the named recipients only. It may contain privileged and confidential information. If you are not the intended recipient you must not copy, distribute or take any action in reliance on this report, or disclose any details of this report to any other person, firm or corporation. If you have received this report in error, please notify us immediately

Dr Julia Clark (Director) julia.clark@health.qld.gov.au
Queensland Paediatric Infectious Diseases Laboratory
L8, CCHR, 62 Graham St, South Brisbane 4101
Ph: (07) 3068 1558

16.2.7 Adverse events

16.2.7.1 Adverse events

Listing 16.2.7.1
Adverse Events

Subject No.	AE No.	Adverse Event Verbatim	System Organ Class	Preferred Term	Onset Date	Onset Time	Onset Day	End Date	End Time	End Day	SAE/ Dis.	Severity	Action Taken/ Outcome	Treatment Directly Related to AE	Time of Onset Rel. to Inoc./ Artesunate	Dur. (hr)
R001	1	Sore throat	Respiratory, thoracic and mediastinal disorders	Oropharyngeal pain	29-May-2017	6:00	Day 5	30-May-2017	6:00	Day 6	No/ No	Mild	None/ Recovered	N/A	4.8 d post/ 4.1 d pre	24.0
R001	2	Intermittent Headache	Nervous system disorders	Headache	03-Jun-2017	17:30	Day 10	04-Jun-2017	8:40	Day 11	No/ No	Mild	None/ Recovered	Inoculum	10.3 d post/ 1.4 d post	15.2
R001	3	Headache	Nervous system disorders	Headache	05-Jun-2017	11:30	Day 12	06-Jun-2017	13:00	Day 13	No/ No	Mild	Meds. Taken/ Recovered	Inoculum	12.0 d post/ 3.1 d post	25.5
R001	4	Myalgia	Musculoskeletal and connective tissue disorders	Myalgia	05-Jun-2017	11:30	Day 12	06-Jun-2017	13:00	Day 13	No/ No	Mild	Meds. Taken/ Recovered	Inoculum	12.0 d post/ 3.1 d post	25.5
R001	5	Arthralgia	Musculoskeletal and connective tissue disorders	Arthralgia	05-Jun-2017	11:30	Day 12	06-Jun-2017	13:00	Day 13	No/ No	Mild	Meds. Taken/ Recovered	Inoculum	12.0 d post/ 3.1 d post	25.5
R001	6	Lethargy	General disorders and administration site conditions	Lethargy	05-Jun-2017	11:30	Day 12	06-Jun-2017	13:00	Day 13	No/ No	Mild	None/ Recovered	Inoculum	12.0 d post/ 3.1 d post	25.5
R001	7	Malaise	General disorders and administration site conditions	Malaise	05-Jun-2017	11:30	Day 12	06-Jun-2017	13:00	Day 13	No/ No	Mild	None/ Recovered	Inoculum	12.0 d post/ 3.1 d post	25.5
R001	8	Chills	General disorders and administration site conditions	Chills	05-Jun-2017	11:30	Day 12	06-Jun-2017	6:30	Day 13	No/ No	Mild	None/ Recovered	Inoculum	12.0 d post/ 3.1 d post	19.0

R001	9	Rhinorrhea	Respiratory, thoracic and mediastinal disorders	Rhinorrhea	05-Jun-2017	14:00	Day 12	06-Jun-2017	6:30	Day 13	No/ No	Mild	None/ Recovered	N/A	12.2 d post/ 3.2 d post	16.5
R001	10	Low neurophils count	Investigations	Neutrophil count decreased	04-Jun-2017	16:06	Day 11	05-Jun-2017	8:23	Day 12	No/ No	Moderate	None/ Recovered	Inoculum	11.2 d post/ 2.3 d post	16.3
R001	11	Headache	Nervous system disorders	Headache	15-Jun-2017	18:00	Day 22	15-Jun-2017	19:00	Day 22	No/ No	Mild	Meds. Taken/ Recovered	N/A	22.3 d post/ 13.4 d post	1.0
R001	12	Low neurtrophil count	Investigations	Neutrophil count decreased	07-Jun-2017	7:54	Day 14	11-Jun-2017	7:44	Day 18	No/ No	Moderate	None/ Recovered	Inoculum	13.9 d post/ 5.0 d post	95.8
R001	13	Elevated AST	Investigations	Aspartate aminotransferase increased	07-Jun-2017	7:54	Day 14	11-Jun-2017	7:44	Day 18	No/ No	Moderate	None/ Recovered	Inoculum	13.9 d post/ 5.0 d post	95.8
R001	14	Elevated ALT	Investigations	Alanine aminotransferase increased	07-Jun-2017	7:54	Day 14	11-Jun-2017	7:44	Day 18	No/ No	Severe	None/ Recovering	Inoculum	13.9 d post/ 5.0 d post	95.8
R001	15	Elevated ALT	Investigations	Alanine aminotransferase increased	11-Jun-2017	7:44	Day 18	19-Jun-2017	7:57	Day 26	No/ No	Moderate	None/ Recovered	Inoculum	17.9 d post/ 9.0 d post	192.2
R001	16	Lethargy	General disorders and administration site conditions	Lethargy	02-Jun-2017	20:04	Day 9	03-Jun-2017	8:28	Day 10	No/ No	Mild	None/ Recovered	Inoculum	9.4 d post/ 0.5 d post	12.4
R001	17	Decreased lymphocyte count	Investigations	Lymphocyte count decreased	05-Jun-2017	8:23	Day 12	07-Jun-2017	7:54	Day 14	No/ No	Moderate	None/ Recovered	Inoculum	11.9 d post/ 3.0 d post	47.5
R002	1	Abdominal discomfort	Gastrointestinal disorders	Abdominal discomfort	20-Jun-2017	6:00	Day 6	21-Jun-2017	6:30	Day 7	No/ No	Mild	None/ Recovered	Inoculum	5.8 d post / 3.1 d pre	24.5
R002	2	Myalgia	Musculoskeletal and connective tissue disorders	Myalgia	22-Jun-2017	14:30	Day 8	22-Jun-2017	14:40	Day 8	No/ No	Mild	None/ Recovered	Inoculum	8.2 d post / 0.8 d pre	0.2
R002	3	Dizziness	Nervous system disorders	Dizziness	23-Jun-2017	15:30	Day 9	23-Jun-2017	15:50	Day 9	No/ No	Mild	None/ Recovered	Inoculum	9.2 d post / 0.3 d post	0.3

R002	4	Dizziness	Nervous system disorders	Dizziness	24-Jun-2017	8:00	Day 10	24-Jun-2017	8:30	Day 10	No/ No	Mild	None/ Recovered	Inoculum	9.9 d post / 0.9 d post	0.5
R002	5	Chills	General disorders and administration site conditions	Chills	24-Jun-2017	12:34	Day 10	24-Jun-2017	14:00	Day 10	No/ No	Mild	Meds. Taken/ Recovered	Inoculum	10.1 d post/ 1.1 d post	1.4
R002	6	Intermittent Fever	General disorders and administration site conditions	Pyrexia	24-Jun-2017	13:15	Day 10	25-Jun-2017	5:02	Day 11	No/ No	Mild	Meds. Taken/ Recovered	Inoculum	10.1 d post/ 1.2 d post	15.8
R002	7	Chills	General disorders and administration site conditions	Chills	24-Jun-2017	20:30	Day 10	24-Jun-2017	20:45	Day 10	No/ No	Mild	Meds. Taken/ Recovered	Inoculum	10.4 d post/ 1.5 d post	0.3
R002	8	Headache	Nervous system disorders	Headache	25-Jun-2017	17:00	Day 11	25-Jun-2017	20:00	Day 11	No/ No	Mild	Meds. Taken/ Recovered	Inoculum	11.3 d post/ 2.3 d post	3.0
R002	9	Chills	General disorders and administration site conditions	Chills	25-Jun-2017	17:00	Day 11	25-Jun-2017	19:40	Day 11	No/ No	Mild	None/ Recovered	Inoculum	11.3 d post/ 2.3 d post	2.7
R002	10	Fever	General disorders and administration site conditions	Pyrexia	25-Jun-2017	18:55	Day 11	25-Jun-2017	20:09	Day 11	No/ No	Mild	Meds. Taken/ Recovered	Inoculum	11.4 d post/ 2.4 d post	1.2
R002	11	Lymphocyte count decreased	Investigations	Lymphocyte count decreased	25-Jun-2017	15:44	Day 11	28-Jun-2017	9:11	Day 14	No/ No	Moderate	None/ Recovered	Inoculum	11.2 d post/ 2.3 d post	65.5
R002	12	White cell count decreased	Investigations	White blood cell count decreased	25-Jun-2017	15:44	Day 11	28-Jun-2017	9:11	Day 14	No/ No	Moderate	None/ Recovered	Inoculum	11.2 d post/ 2.3 d post	65.5
R002	13	Headache	Nervous system disorders	Headache	25-Jun-2017	19:06	Day 11	26-Jun-2017	21:00	Day 12	No/ No	Mild	Meds. Taken/ Recovered	Inoculum	11.4 d post/ 2.4 d post	25.9
R002	14	Chills	General disorders and administration site conditions	Chills	26-Jun-2017	20:00	Day 12	26-Jun-2017	20:28	Day 12	No/ No	Moderate	Meds. Taken/ Recovered	Inoculum	12.4 d post/ 3.4 d post	0.5

R002	15	Neutrophil count decreased	Investigations	Neutrophil count decreased	28-Jun-2017	9:11	Day 14	02-Jul-2017	8:46	Day 18	No/ No	Moderate	None/ Recovered	Inoculum	14.0 d post/ 5.0 d post	95.6
R002	16	Nausea	Gastrointestinal disorders	Nausea	10-Jul-2017	10:10	Day 26	10-Jul-2017	10:40	Day 26	No/ No	Moderate	None/ Recovered	Malarone/ Primacin	26.0 d post/ 17.0 post	0.5
R002	17	Vomiting	Gastrointestinal disorders	Vomiting	10-Jul-2017	10:10	Day 26	10-Jul-2017	10:15	Day 26	No/ No	Moderate	None/ Recovered	Malarone/ Primacin	26.0 d post/ 17.0 post	0.1
R002	18	Dizziness	Nervous system disorders	Dizziness	10-Jul-2017	10:10	Day 26	10-Jul-2017	12:00	Day 26	No/ No	Moderate	None/ Recovered	Malarone/ Primacin	26.0 d post/ 17.0 post	1.8
R002	19	Nausea	Gastrointestinal disorders	Nausea	11-Jul-2017	9:30	Day 27	11-Jul-2017	9:50	Day 27	No/ No	Mild	None/ Recovered	Malarone	27.0 d post/ 18.0 d post	0.3

Notes:
(d) = Day from Artesunate dose
(study day) = number of days relative to day of administration of the inoculum, where Study Day = 0 for inoculum dosing day.
AE = Adverse Event; SAE = Serious adverse event; Dis. = Discontinuation

16.2.7.2 Serious adverse events

Listing 16.2.7.2
Serious Adverse Events

Subject No.	AE No.	Adverse Event Verbatim	System Organ Class	Preferred Term	Onset Date	Onset Time	Onset Day relative to Artesunate dose	Onset Study Day	End Date	End Time	End Day relative to Artesunate dose	End Study Day	Did AE result in Death?	Is AE life threatening?	Did AE result in initial or prolonged hospitalisation ?	Did AE Result in Persistent or Significant Disability or Incapacity ?	Is AE associated with congenital anomaly or birth defect?	Other Medically Important Event
No serious adverse events were reported in this study																		

Notes:
A treatment-emergent adverse event is defined as an AE that occurred or worsened following first administration of study drug.
(d) = Day from Artesunate dose
(study day) = number of days relative to day of administration of the inoculum, where Study Day = 0 for inoculum dosing day.
AE = Adverse Event; TEAE = A treatment-emergent adverse event

16.2.8 Clinical laboratory measurements

SUBJECT	DATE	TIME	TEST	RESULT	UNIT	MIN	MAX
R001	9-May-17	11:52:00	Bilirubin	6	umol/L	4	20
R001	9-May-17	11:52:00	Alk Phos	49	U/L	35	110
R001	9-May-17	11:52:00	AST	19	U/L	10	40
R001	9-May-17	11:52:00	ALT	19	U/L	5	40
R001	9-May-17	11:52:00	LDH	144	U/L	120	250
R001	9-May-17	11:52:00	Cholesterol	2.7	mmol/L	3.9	5.5
R001	9-May-17	11:52:00	Chylomicrons	NIL			
R001	9-May-17	11:52:00	HDL	1.08	mmol/L	0.9	1.5
R001	9-May-17	11:52:00	LDL	1.5	mmol/L	0	4
R001	9-May-17	11:52:00	Triglyceride	0.3	mmol/L	0.6	2
R001	9-May-17	11:52:00	Sodium	140	mmol/L	135	145
R001	9-May-17	11:52:00	Potassium	4.3	mmol/L	3.5	5.5
R001	9-May-17	11:52:00	Chloride	103	mmol/L	95	110
R001	9-May-17	11:52:00	Bicarbonate	30	mmol/L	20	32
R001	9-May-17	11:52:00	Creatinine	80	umol/L	60	110
R001	9-May-17	11:52:00	eGFR	>90			>59
R001	9-May-17	11:52:00	Urea	3.4	mmol/L	3	7.5
R001	9-May-17	11:52:00	Uric Acid	0.3	mmol/L	0.2	0.5
R001	9-May-17	11:52:00	Fasting Glucose	5	mmol/L	3.6	6
R001	9-May-17	11:52:00	Random Glucose		mmol/L		
R001	9-May-17	11:52:00	Total Protein	68	g/L	66	83
R001	9-May-17	11:52:00	Albumin	40	g/L	35	48
R001	9-May-17	11:52:00	Globulin	28	g/L	23	43
R001	9-May-17	11:52:00	Phosphate	1.4	mmol/L	0.8	1.5
R001	9-May-17	11:52:00	Calcium (Corrected)	2.28	mmol/L	2.1	2.6
R001	9-May-17	11:52:00	C Bilirubin	3	umol/L	0	7
R001	9-May-17	11:52:00	Tot Chol/HDL	2.5		0	4.5
R001	9-May-17	11:52:00	Magnesium	0.8	mmol/L	0.7	1.1
R001	9-May-17	11:52:00	Icteric	13		0	200
R001	9-May-17	11:52:00	Haemolysis Index	3		0	40
R001	9-May-17	11:52:00	Lip	0		0	10
R001	9-May-17	11:52:00	G6PD	2353	U/L		
R001	9-May-17	11:52:00	Haemoglobin	144.1	g/L		
R001	9-May-17	11:52:00	G6PD/Hb	16.3	U/g Hb	7	20.5
R001	9-May-17	11:52:00	HepB surface antigen	N			
R001	9-May-17	11:52:00	Hepatitis B Core Ab	N			
R001	9-May-17	11:52:00	HepC-IgG antibody	N			
R001	9-May-17	11:52:00	HIV Ag/Ab	N			
R001	9-May-17	11:52:00	Haemoglobin	154	g/L	135	175
R001	9-May-17	11:52:00	Haematocrit	0.43		0.4	0.54
R001	9-May-17	11:52:00	Red cell count	5	10^12/L	4.5	6.5
R001	9-May-17	11:52:00	MCV	86	fL	80	100
R001	9-May-17	11:52:00	White cell count	4.6	10^9/L	3.5	10
R001	9-May-17	11:52:00	Neutrophils	2.41	10^9/L	1.5	6.5
R001	9-May-17	11:52:00	Lymphocytes	1.59	10^9/L	1	4
R001	9-May-17	11:52:00	Monocytes	0.35	10^9/L	0	0.9
R001	9-May-17	11:52:00	Eosinophils	0.17	10^9/L	0	0.6
R001	9-May-17	11:52:00	Basophils	0.03	10^9/L	0	0.15
R001	9-May-17	11:52:00	Platelets	243	10^9/L	150	400

R001	9-May-17	11:52:00	Blood Group	APOS			
R001	9-May-17	11:52:00	Antibody Screen	N			
R001	23-May-17	8:47:00	Bilirubin	10	umol/L	4	20
R001	23-May-17	8:47:00	Alk Phos	59	U/L	35	110
R001	23-May-17	8:47:00	AST	32	U/L	10	40
R001	23-May-17	8:47:00	ALT	28	U/L	5	40
R001	23-May-17	8:47:00	LDH	219	U/L	120	250
R001	23-May-17	8:47:00	Sodium	141	mmol/L	135	145
R001	23-May-17	8:47:00	Potassium	4.5	mmol/L	3.5	5.5
R001	23-May-17	8:47:00	Chloride	106	mmol/L	95	110
R001	23-May-17	8:47:00	Bicarbonate	30	mmol/L	20	32
R001	23-May-17	8:47:00	Creatinine	75	umol/L	60	110
R001	23-May-17	8:47:00	eGFR	>90			>59
R001	23-May-17	8:47:00	Urea	3.4	mmol/L	3	7.5
R001	23-May-17	8:47:00	Uric Acid	0.297	mmol/L	0.2	0.5
R001	23-May-17	8:47:00	Fasting Glucose	4.6	mmol/L	3.6	6
R001	23-May-17	8:47:00	Total Protein	69	g/L	66	83
R001	23-May-17	8:47:00	Albumin	43	g/L	35	48
R001	23-May-17	8:47:00	Globulin	26	g/L	23	43
R001	23-May-17	8:47:00	Phosphate	1.11	mmol/L	0.8	1.5
R001	23-May-17	8:47:00	Calcium (Corrected)	2.29	mmol/L	2.1	2.6
R001	23-May-17	8:47:00	C Bilirubin	5	umol/L	0	7
R001	23-May-17	8:47:00	Icteric	16		0	200
R001	23-May-17	8:47:00	Haemolysis Index	8		0	40
R001	23-May-17	8:47:00	Lip	0		0	10
R001	23-May-17	8:47:00	Haemoglobin	155	g/L	135	175
R001	23-May-17	8:47:00	Haematocrit	0.44		0.4	0.54
R001	23-May-17	8:47:00	Red cell count	5	10^12/L	4.5	6.5
R001	23-May-17	8:47:00	MCV	87	fL	80	100
R001	23-May-17	8:47:00	White cell count	5.7	10^9/L	3.5	10
R001	23-May-17	8:47:00	Neutrophils	3.22	10^9/L	1.5	6.5
R001	23-May-17	8:47:00	Lymphocytes	1.61	10^9/L	1	4
R001	23-May-17	8:47:00	Monocytes	0.64	10^9/L	0	0.9
R001	23-May-17	8:47:00	Eosinophils	0.19	10^9/L	0	0.6
R001	23-May-17	8:47:00	Basophils	0.04	10^9/L	0	0.15
R001	23-May-17	8:47:00	Platelets	229	10^9/L	150	400
R001	23-May-17	8:47:00	Reticulocytes	49	10^9/L	25	120
R001	29-May-17	8:02:00	Human Metapneumovirus RNA	N			
R001	29-May-17	8:02:00	Rhinovirus RNA	N			
R001	29-May-17	8:02:00	Influenza A RNA	N			
R001	29-May-17	8:02:00	Influenza B RNA	N			
R001	29-May-17	8:02:00	Respiratory Syncytial Virus RNA	N			
R001	29-May-17	8:02:00	Parainfluenzae Type 1 RNA	N			
R001	29-May-17	8:02:00	Parainfluenzae Type 2 RNA	N			
R001	29-May-17	8:02:00	Parainfluenzae Type 3 RNA	N			
R001	29-May-17	8:02:00	Adenovirus DNA	N			
R001	29-May-17	8:02:00	Parainfluenzae Type 4 RNA	N			
R001	2-Jun-17	8:13:00	Bilirubin	14	umol/L	4	20
R001	2-Jun-17	8:13:00	Alk Phos	60	U/L	35	110
R001	2-Jun-17	8:13:00	AST	22	U/L	10	40

R001	2-Jun-17	8:13:00	ALT	28	U/L	5	40
R001	2-Jun-17	8:13:00	LDH	164	U/L	120	250
R001	2-Jun-17	8:13:00	Sodium	141	mmol/L	135	145
R001	2-Jun-17	8:13:00	Potassium	4.2	mmol/L	3.5	5.5
R001	2-Jun-17	8:13:00	Chloride	106	mmol/L	95	110
R001	2-Jun-17	8:13:00	Bicarbonate	27	mmol/L	20	32
R001	2-Jun-17	8:13:00	Creatinine	73	umol/L	60	110
R001	2-Jun-17	8:13:00	eGFR	>90			>59
R001	2-Jun-17	8:13:00	Urea	4.8	mmol/L	3	7.5
R001	2-Jun-17	8:13:00	Uric Acid	0.286	mmol/L	0.2	0.5
R001	2-Jun-17	8:13:00	Fasting Glucose	4.4	mmol/L	3.6	6
R001	2-Jun-17	8:13:00	Total Protein	70	g/L	66	83
R001	2-Jun-17	8:13:00	Albumin	42	g/L	35	48
R001	2-Jun-17	8:13:00	Globulin	28	g/L	23	43
R001	2-Jun-17	8:13:00	Phosphate	1.54	mmol/L	0.8	1.5
R001	2-Jun-17	8:13:00	Calcium (Corrected)	2.26	mmol/L	2.1	2.6
R001	2-Jun-17	8:13:00	C Bilirubin	7	umol/L	0	7
R001	2-Jun-17	8:13:00	Icteric	22		0	200
R001	2-Jun-17	8:13:00	Haemolysis Index	11		0	40
R001	2-Jun-17	8:13:00	Lip	0		0	10
R001	2-Jun-17	8:13:00	Haemoglobin	159	g/L	135	175
R001	2-Jun-17	8:13:00	Haematocrit	0.44		0.4	0.54
R001	2-Jun-17	8:13:00	Red cell count	5.2	10^12/L	4.5	6.5
R001	2-Jun-17	8:13:00	MCV	85	fL	80	100
R001	2-Jun-17	8:13:00	White cell count	3.4	10^9/L	3.5	10
R001	2-Jun-17	8:13:00	Neutrophils	1.52	10^9/L	1.5	6.5
R001	2-Jun-17	8:13:00	Lymphocytes	1.25	10^9/L	1	4
R001	2-Jun-17	8:13:00	Monocytes	0.43	10^9/L	0	0.9
R001	2-Jun-17	8:13:00	Eosinophils	0.13	10^9/L	0	0.6
R001	2-Jun-17	8:13:00	Basophils	0.04	10^9/L	0	0.15
R001	2-Jun-17	8:13:00	Platelets	217	10^9/L	150	400
R001	4-Jun-17	16:06:00	Bilirubin	6	umol/L	4	20
R001	4-Jun-17	16:06:00	Alk Phos	62	U/L	35	110
R001	4-Jun-17	16:06:00	AST	23	U/L	10	40
R001	4-Jun-17	16:06:00	ALT	25	U/L	5	40
R001	4-Jun-17	16:06:00	LDH	161	U/L	120	250
R001	4-Jun-17	16:06:00	Sodium	142	mmol/L	135	145
R001	4-Jun-17	16:06:00	Potassium	4	mmol/L	3.5	5.5
R001	4-Jun-17	16:06:00	Chloride	109	mmol/L	95	110
R001	4-Jun-17	16:06:00	Bicarbonate	27	mmol/L	20	32
R001	4-Jun-17	16:06:00	Creatinine	83	umol/L	60	110
R001	4-Jun-17	16:06:00	eGFR	>90			>59
R001	4-Jun-17	16:06:00	Urea	3.3	mmol/L	3	7.5
R001	4-Jun-17	16:06:00	Uric Acid	0.272	mmol/L	0.2	0.5
R001	4-Jun-17	16:06:00	Fasting Glucose	3.7	mmol/L	3.6	6
R001	4-Jun-17	16:06:00	Total Protein	69	g/L	66	83
R001	4-Jun-17	16:06:00	Albumin	40	g/L	35	48
R001	4-Jun-17	16:06:00	Globulin	29	g/L	23	43
R001	4-Jun-17	16:06:00	Phosphate	1.16	mmol/L	0.8	1.5
R001	4-Jun-17	16:06:00	Calcium (Corrected)	2.26	mmol/L	2.1	2.6

R001	4-Jun-17	16:06:00	C Bilirubin	3	umol/L	0	7
R001	4-Jun-17	16:06:00	Icteric	12		0	200
R001	4-Jun-17	16:06:00	Haemolysis Index	1		0	40
R001	4-Jun-17	16:06:00	Lip	0		0	10
R001	4-Jun-17	16:06:00	Haemoglobin	155	g/L	135	175
R001	4-Jun-17	16:06:00	Haematocrit	0.44		0.4	0.54
R001	4-Jun-17	16:06:00	Red cell count	5.1	10^12/L	4.5	6.5
R001	4-Jun-17	16:06:00	MCV	85	fL	80	100
R001	4-Jun-17	16:06:00	White cell count	3	10^9/L	3.5	10
R001	4-Jun-17	16:06:00	Neutrophils	1.42	10^9/L	1.5	6.5
R001	4-Jun-17	16:06:00	Lymphocytes	0.89	10^9/L	1	4
R001	4-Jun-17	16:06:00	Monocytes	0.56	10^9/L	0	0.9
R001	4-Jun-17	16:06:00	Eosinophils	0.06	10^9/L	0	0.6
R001	4-Jun-17	16:06:00	Basophils	0.02	10^9/L	0	0.15
R001	4-Jun-17	16:06:00	Platelets	173	10^9/L	150	400
R001	5-Jun-17	8:23:00	Bilirubin	9	umol/L	4	20
R001	5-Jun-17	8:23:00	Alk Phos	70	U/L	35	110
R001	5-Jun-17	8:23:00	AST	53	U/L	10	40
R001	5-Jun-17	8:23:00	ALT	62	U/L	5	40
R001	5-Jun-17	8:23:00	LDH	232	U/L	120	250
R001	5-Jun-17	8:23:00	Sodium	140	mmol/L	135	145
R001	5-Jun-17	8:23:00	Potassium	4.6	mmol/L	3.5	5.5
R001	5-Jun-17	8:23:00	Chloride	106	mmol/L	95	110
R001	5-Jun-17	8:23:00	Bicarbonate	30	mmol/L	20	32
R001	5-Jun-17	8:23:00	Creatinine	76	umol/L	60	110
R001	5-Jun-17	8:23:00	eGFR	>90			>59
R001	5-Jun-17	8:23:00	Urea	2.4	mmol/L	3	7.5
R001	5-Jun-17	8:23:00	Uric Acid	0.285	mmol/L	0.2	0.5
R001	5-Jun-17	8:23:00	Fasting Glucose	5.2	mmol/L	3.6	6
R001	5-Jun-17	8:23:00	Total Protein	67	g/L	66	83
R001	5-Jun-17	8:23:00	Albumin	39	g/L	35	48
R001	5-Jun-17	8:23:00	Globulin	28	g/L	23	43
R001	5-Jun-17	8:23:00	Phosphate	1.04	mmol/L	0.8	1.5
R001	5-Jun-17	8:23:00	Calcium (Corrected)	2.25	mmol/L	2.1	2.6
R001	5-Jun-17	8:23:00	C Bilirubin	4	umol/L	0	7
R001	5-Jun-17	8:23:00	Icteric	12		0	200
R001	5-Jun-17	8:23:00	Haemolysis Index	13		0	40
R001	5-Jun-17	8:23:00	Lip	0		0	10
R001	5-Jun-17	8:23:00	Haemoglobin	163	g/L	135	175
R001	5-Jun-17	8:23:00	Haematocrit	0.45		0.4	0.54
R001	5-Jun-17	8:23:00	Red cell count	5.4	10^12/L	4.5	6.5
R001	5-Jun-17	8:23:00	MCV	85	fL	80	100
R001	5-Jun-17	8:23:00	White cell count	3	10^9/L	3.5	10
R001	5-Jun-17	8:23:00	Neutrophils	1.79	10^9/L	1.5	6.5
R001	5-Jun-17	8:23:00	Lymphocytes	0.67	10^9/L	1	4
R001	5-Jun-17	8:23:00	Monocytes	0.4	10^9/L	0	0.9
R001	5-Jun-17	8:23:00	Eosinophils	0.06	10^9/L	0	0.6
R001	5-Jun-17	8:23:00	Basophils	0.03	10^9/L	0	0.15
R001	5-Jun-17	8:23:00	Platelets	153	10^9/L	150	400
R001	7-Jun-17	7:54:00	Bilirubin	12	umol/L	4	20

R001	7-Jun-17	7:54:00	Alk Phos	80	U/L	35	110
R001	7-Jun-17	7:54:00	AST	159	U/L	10	40
R001	7-Jun-17	7:54:00	ALT	227	U/L	5	40
R001	7-Jun-17	7:54:00	LDH	399	U/L	120	250
R001	7-Jun-17	7:54:00	Sodium	141	mmol/L	135	145
R001	7-Jun-17	7:54:00	Potassium	4.3	mmol/L	3.5	5.5
R001	7-Jun-17	7:54:00	Chloride	104	mmol/L	95	110
R001	7-Jun-17	7:54:00	Bicarbonate	29	mmol/L	20	32
R001	7-Jun-17	7:54:00	Creatinine	79	umol/L	60	110
R001	7-Jun-17	7:54:00	eGFR	>90			>59
R001	7-Jun-17	7:54:00	Urea	3.3	mmol/L	3	7.5
R001	7-Jun-17	7:54:00	Uric Acid	0.295	mmol/L	0.2	0.5
R001	7-Jun-17	7:54:00	Fasting Glucose	5.5	mmol/L	3.6	6
R001	7-Jun-17	7:54:00	Total Protein	69	g/L	66	83
R001	7-Jun-17	7:54:00	Albumin	39	g/L	35	48
R001	7-Jun-17	7:54:00	Globulin	30	g/L	23	43
R001	7-Jun-17	7:54:00	Phosphate	1.08	mmol/L	0.8	1.5
R001	7-Jun-17	7:54:00	Calcium (Corrected)	2.3	mmol/L	2.1	2.6
R001	7-Jun-17	7:54:00	C Bilirubin	5	umol/L	0	7
R001	7-Jun-17	7:54:00	Icteric	16		0	200
R001	7-Jun-17	7:54:00	Haemolysis Index	10		0	40
R001	7-Jun-17	7:54:00	Lip	0		0	10
R001	7-Jun-17	7:54:00	Haemoglobin	159	g/L	135	175
R001	7-Jun-17	7:54:00	Haematocrit	0.43		0.4	0.54
R001	7-Jun-17	7:54:00	Red cell count	5.1	10^12/L	4.5	6.5
R001	7-Jun-17	7:54:00	MCV	85	fL	80	100
R001	7-Jun-17	7:54:00	White cell count	3.1	10^9/L	3.5	10
R001	7-Jun-17	7:54:00	Neutrophils	1.23	10^9/L	1.5	6.5
R001	7-Jun-17	7:54:00	Lymphocytes	1.1	10^9/L	1	4
R001	7-Jun-17	7:54:00	Monocytes	0.58	10^9/L	0	0.9
R001	7-Jun-17	7:54:00	Eosinophils	0.14	10^9/L	0	0.6
R001	7-Jun-17	7:54:00	Basophils	0.02	10^9/L	0	0.15
R001	7-Jun-17	7:54:00	Platelets	139	10^9/L	150	400
R001	9-Jun-17	7:44:00	Bilirubin	10	umol/L	4	20
R001	9-Jun-17	7:44:00	Alk Phos	76	U/L	35	110
R001	9-Jun-17	7:44:00	AST	120	U/L	10	40
R001	9-Jun-17	7:44:00	AST/ALT Ratio	0.5		0.5	100
R001	9-Jun-17	7:44:00	ALT	250	U/L	5	40
R001	9-Jun-17	7:44:00	Gamma GT	15	U/L	5	50
R001	9-Jun-17	7:44:00	Total Protein	70	g/L	66	83
R001	9-Jun-17	7:44:00	Albumin	40	g/L	35	48
R001	9-Jun-17	7:44:00	Globulin	30	g/L	23	43
R001	9-Jun-17	7:44:00	Icteric	14		0	200
R001	9-Jun-17	7:44:00	Haemolysis Index	16		0	40
R001	9-Jun-17	7:44:00	Lip	0		0	10
R001	11-Jun-17	7:44:00	Bilirubin	7	umol/L	4	20
R001	11-Jun-17	7:44:00	Alk Phos	74	U/L	35	110
R001	11-Jun-17	7:44:00	AST	65	U/L	10	40
R001	11-Jun-17	7:44:00	ALT	166	U/L	5	40
R001	11-Jun-17	7:44:00	LDH	265	U/L	120	250

R001	11-Jun-17	7:44:00	Sodium	139	mmol/L	135	145
R001	11-Jun-17	7:44:00	Potassium	4.8	mmol/L	3.5	5.5
R001	11-Jun-17	7:44:00	Chloride	105	mmol/L	95	110
R001	11-Jun-17	7:44:00	Bicarbonate	26	mmol/L	20	32
R001	11-Jun-17	7:44:00	Creatinine	82	umol/L	60	110
R001	11-Jun-17	7:44:00	eGFR	>90			>59
R001	11-Jun-17	7:44:00	Urea	5.2	mmol/L	3	7.5
R001	11-Jun-17	7:44:00	Uric Acid	0.295	mmol/L	0.2	0.5
R001	11-Jun-17	7:44:00	Fasting Glucose	5.3	mmol/L	3.6	6
R001	11-Jun-17	7:44:00	Total Protein	73	g/L	66	83
R001	11-Jun-17	7:44:00	Albumin	42	g/L	35	48
R001	11-Jun-17	7:44:00	Globulin	31	g/L	23	43
R001	11-Jun-17	7:44:00	Phosphate	1.37	mmol/L	0.8	1.5
R001	11-Jun-17	7:44:00	Calcium (Corrected)	2.35	mmol/L	2.1	2.6
R001	11-Jun-17	7:44:00	C Bilirubin	4	umol/L	0	7
R001	11-Jun-17	7:44:00	Icteric	13		0	200
R001	11-Jun-17	7:44:00	Haemolysis Index	5		0	40
R001	11-Jun-17	7:44:00	Lip	0		0	10
R001	11-Jun-17	7:44:00	Haemoglobin	156	g/L	135	175
R001	11-Jun-17	7:44:00	Haematocrit	0.44		0.4	0.54
R001	11-Jun-17	7:44:00	Red cell count	5.2	10^12/L	4.5	6.5
R001	11-Jun-17	7:44:00	MCV	85	fL	80	100
R001	11-Jun-17	7:44:00	White cell count	4	10^9/L	3.5	10
R001	11-Jun-17	7:44:00	Neutrophils	1.84	10^9/L	1.5	6.5
R001	11-Jun-17	7:44:00	Lymphocytes	1.63	10^9/L	1	4
R001	11-Jun-17	7:44:00	Monocytes	0.4	10^9/L	0	0.9
R001	11-Jun-17	7:44:00	Eosinophils	0.13	10^9/L	0	0.6
R001	11-Jun-17	7:44:00	Basophils	0.03	10^9/L	0	0.15
R001	11-Jun-17	7:44:00	Platelets	196	10^9/L	150	400
R001	19-Jun-17	7:57:00	Bilirubin	11	umol/L	4	20
R001	19-Jun-17	7:57:00	Alk Phos	67	U/L	35	110
R001	19-Jun-17	7:57:00	AST	23	U/L	10	40
R001	19-Jun-17	7:57:00	ALT	50	U/L	5	40
R001	19-Jun-17	7:57:00	LDH	189	U/L	120	250
R001	19-Jun-17	7:57:00	Sodium	139	mmol/L	135	145
R001	19-Jun-17	7:57:00	Potassium	4.5	mmol/L	3.5	5.5
R001	19-Jun-17	7:57:00	Chloride	104	mmol/L	95	110
R001	19-Jun-17	7:57:00	Bicarbonate	29	mmol/L	20	32
R001	19-Jun-17	7:57:00	Creatinine	91	umol/L	60	110
R001	19-Jun-17	7:57:00	eGFR	>90			>59
R001	19-Jun-17	7:57:00	Urea	6.5	mmol/L	3	7.5
R001	19-Jun-17	7:57:00	Uric Acid	0.276	mmol/L	0.2	0.5
R001	19-Jun-17	7:57:00	Fasting Glucose	5.2	mmol/L	3.6	6
R001	19-Jun-17	7:57:00	Total Protein	70	g/L	66	83
R001	19-Jun-17	7:57:00	Albumin	40	g/L	35	48
R001	19-Jun-17	7:57:00	Globulin	30	g/L	23	43
R001	19-Jun-17	7:57:00	Phosphate	1.33	mmol/L	0.8	1.5
R001	19-Jun-17	7:57:00	Calcium (Corrected)	2.22	mmol/L	2.1	2.6
R001	19-Jun-17	7:57:00	C Bilirubin	6	umol/L	0	7
R001	19-Jun-17	7:57:00	Icteric	18		0	200

R001	19-Jun-17	7:57:00	Haemolysis Index	6		0	40
R001	19-Jun-17	7:57:00	Lip	0		0	10
R001	19-Jun-17	7:57:00	Haemoglobin	155	g/L	135	175
R001	19-Jun-17	7:57:00	Haematocrit	0.43		0.4	0.54
R001	19-Jun-17	7:57:00	Red cell count	5	10^12/L	4.5	6.5
R001	19-Jun-17	7:57:00	MCV	86	fL	80	100
R001	19-Jun-17	7:57:00	White cell count	3.9	10^9/L	3.5	10
R001	19-Jun-17	7:57:00	Neutrophils	1.67	10^9/L	1.5	6.5
R001	19-Jun-17	7:57:00	Lymphocytes	1.43	10^9/L	1	4
R001	19-Jun-17	7:57:00	Monocytes	0.53	10^9/L	0	0.9
R001	19-Jun-17	7:57:00	Eosinophils	0.19	10^9/L	0	0.6
R001	19-Jun-17	7:57:00	Basophils	0.04	10^9/L	0	0.15
R001	19-Jun-17	7:57:00	Platelets	248	10^9/L	150	400
R001	21-Jun-17	8:38:00	Bilirubin	16	umol/L	4	20
R001	21-Jun-17	8:38:00	Alk Phos	62	U/L	35	110
R001	21-Jun-17	8:38:00	AST	28	U/L	10	40
R001	21-Jun-17	8:38:00	ALT	38	U/L	5	40
R001	21-Jun-17	8:38:00	LDH	186	U/L	120	250
R001	21-Jun-17	8:38:00	Sodium	140	mmol/L	135	145
R001	21-Jun-17	8:38:00	Potassium	4.6	mmol/L	3.5	5.5
R001	21-Jun-17	8:38:00	Chloride	107	mmol/L	95	110
R001	21-Jun-17	8:38:00	Bicarbonate	30	mmol/L	20	32
R001	21-Jun-17	8:38:00	Creatinine	85	umol/L	60	110
R001	21-Jun-17	8:38:00	eGFR	>90			>59
R001	21-Jun-17	8:38:00	Urea	3.9	mmol/L	3	7.5
R001	21-Jun-17	8:38:00	Uric Acid	0.351	mmol/L	0.2	0.5
R001	21-Jun-17	8:38:00	Fasting Glucose	4.8	mmol/L	3.6	6
R001	21-Jun-17	8:38:00	Total Protein	65	g/L	66	83
R001	21-Jun-17	8:38:00	Albumin	38	g/L	35	48
R001	21-Jun-17	8:38:00	Globulin	27	g/L	23	43
R001	21-Jun-17	8:38:00	Phosphate	1.08	mmol/L	0.8	1.5
R001	21-Jun-17	8:38:00	Calcium (Corrected)	2.24	mmol/L	2.1	2.6
R001	21-Jun-17	8:38:00	C Bilirubin	8	umol/L	0	7
R001	21-Jun-17	8:38:00	Icteric	24		0	200
R001	21-Jun-17	8:38:00	Haemolysis Index	6		0	40
R001	21-Jun-17	8:38:00	Lip	0		0	10
R001	21-Jun-17	8:38:00	HepB surface antigen	N			
R001	21-Jun-17	8:38:00	Hepatitis B Core Ab	N			
R001	21-Jun-17	8:38:00	HepC-IgG antibody	N			
R001	21-Jun-17	8:38:00	HIV 1 Western Blot	:SC			
R001	21-Jun-17	8:38:00	HIV p24 Antigen	NR			
R001	21-Jun-17	8:38:00	HIV Ag/Ab	WP			
R001	21-Jun-17	8:38:00	Haemoglobin	142	g/L	135	175
R001	21-Jun-17	8:38:00	Haematocrit	0.41		0.4	0.54
R001	21-Jun-17	8:38:00	Red cell count	4.7	10^12/L	4.5	6.5
R001	21-Jun-17	8:38:00	MCV	88	fL	80	100
R001	21-Jun-17	8:38:00	White cell count	3.4	10^9/L	3.5	10
R001	21-Jun-17	8:38:00	Neutrophils	1.52	10^9/L	1.5	6.5
R001	21-Jun-17	8:38:00	Lymphocytes	1.18	10^9/L	1	4
R001	21-Jun-17	8:38:00	Monocytes	0.51	10^9/L	0	0.9

R001	21-Jun-17	8:38:00	Eosinophils	0.15	10^9/L	0	0.6
R001	21-Jun-17	8:38:00	Basophils	0.04	10^9/L	0	0.15
R001	21-Jun-17	8:38:00	Platelets	242	10^9/L	150	400
R001	21-Jun-17	8:38:00	Reticulocytes	45	10^9/L	25	120
R001	21-Jun-17	8:38:00	Blood Group	APOS			
R001	21-Jun-17	8:38:00	Antibody Screen	N			
R001	29-Jun-17	8:37:00	HIV 1 Western Blot	:SC			
R001	29-Jun-17	8:37:00	HIV p24 Antigen	NR			
R001	29-Jun-17	8:37:00	HIV Ag/Ab	WP			
R001	11-Aug-17	8:18:00	HIV Ag/Ab	N			
R001	11-Aug-17	8:18:00	Blood Group	APOS			
R001	11-Aug-17	8:18:00	Antibody Screen	N			
R002	27-Apr-17	12:19:00	Bilirubin	17	umol/L	4	20
R002	27-Apr-17	12:19:00	Alk Phos	68	U/L	35	110
R002	27-Apr-17	12:19:00	AST	29	U/L	10	40
R002	27-Apr-17	12:19:00	ALT	30	U/L	5	40
R002	27-Apr-17	12:19:00	LDH	231	U/L	120	250
R002	27-Apr-17	12:19:00	Cholesterol	6	mmol/L	3.9	5.5
R002	27-Apr-17	12:19:00	Chylomicrons	NIL			
R002	27-Apr-17	12:19:00	HDL	1.43	mmol/L	0.9	1.5
R002	27-Apr-17	12:19:00	LDL	3.9	mmol/L	0	4
R002	27-Apr-17	12:19:00	Triglyceride	1.4	mmol/L	0.6	2
R002	27-Apr-17	12:19:00	Sodium	141	mmol/L	135	145
R002	27-Apr-17	12:19:00	Potassium	3.9	mmol/L	3.5	5.5
R002	27-Apr-17	12:19:00	Chloride	105	mmol/L	95	110
R002	27-Apr-17	12:19:00	Bicarbonate	29	mmol/L	20	32
R002	27-Apr-17	12:19:00	Creatinine	73	umol/L	60	110
R002	27-Apr-17	12:19:00	eGFR	>90			>59
R002	27-Apr-17	12:19:00	Urea	4.4	mmol/L	3	7.5
R002	27-Apr-17	12:19:00	Uric Acid	0.39	mmol/L	0.2	0.5
R002	27-Apr-17	12:19:00	Fasting Glucose	5.2	mmol/L	3.6	6
R002	27-Apr-17	12:19:00	Total Protein	71	g/L	66	83
R002	27-Apr-17	12:19:00	Albumin	43	g/L	35	48
R002	27-Apr-17	12:19:00	Globulin	28	g/L	23	43
R002	27-Apr-17	12:19:00	Phosphate	1.2	mmol/L	0.8	1.5
R002	27-Apr-17	12:19:00	Calcium (Corrected)	2.33	mmol/L	2.1	2.6
R002	27-Apr-17	12:19:00	C Bilirubin	5	umol/L	0	7
R002	27-Apr-17	12:19:00	Tot Chol/HDL	4.2		0	4.5
R002	27-Apr-17	12:19:00	Magnesium	0.8	mmol/L	0.7	1.1
R002	27-Apr-17	12:19:00	Icteric	26		0	200
R002	27-Apr-17	12:19:00	Haemolysis Index	6		0	40
R002	27-Apr-17	12:19:00	Lip	0		0	10
R002	27-Apr-17	12:19:00	G6PD	1833.9	U/L		
R002	27-Apr-17	12:19:00	Haemoglobin	128.4	g/L		
R002	27-Apr-17	12:19:00	G6PD/Hb	14.3	U/g Hb	7	20.5
R002	27-Apr-17	12:19:00	HepB surface antigen	N			
R002	27-Apr-17	12:19:00	Hepatitis B Core Ab	N			
R002	27-Apr-17	12:19:00	HepC-IgG antibody	N			
R002	27-Apr-17	12:19:00	HIV Ag/Ab	N			
R002	27-Apr-17	12:19:00	Haemoglobin	141	g/L	135	175

R002	27-Apr-17	12:19:00	Haematocrit	0.42		0.4	0.54
R002	27-Apr-17	12:19:00	Red cell count	4.8	10^12/L	4.5	6.5
R002	27-Apr-17	12:19:00	MCV	88	fL	80	100
R002	27-Apr-17	12:19:00	White cell count	4.7	10^9/L	3.5	10
R002	27-Apr-17	12:19:00	Neutrophils	2.71	10^9/L	1.5	6.5
R002	27-Apr-17	12:19:00	Lymphocytes	1.53	10^9/L	1	4
R002	27-Apr-17	12:19:00	Monocytes	0.31	10^9/L	0	0.9
R002	27-Apr-17	12:19:00	Eosinophils	0.13	10^9/L	0	0.6
R002	27-Apr-17	12:19:00	Basophils	0.02	10^9/L	0	0.15
R002	27-Apr-17	12:19:00	Platelets	184	10^9/L	150	400
R002	27-Apr-17	12:19:00	Blood Group	BPOS			
R002	27-Apr-17	12:19:00	Antibody Screen	N			
R002	23-May-17	8:02:00	Bilirubin	14	umol/L	4	20
R002	23-May-17	8:02:00	Alk Phos	65	U/L	35	110
R002	23-May-17	8:02:00	AST	22	U/L	10	40
R002	23-May-17	8:02:00	ALT	27	U/L	5	40
R002	23-May-17	8:02:00	LDH	216	U/L	120	250
R002	23-May-17	8:02:00	Sodium	141	mmol/L	135	145
R002	23-May-17	8:02:00	Potassium	4.2	mmol/L	3.5	5.5
R002	23-May-17	8:02:00	Chloride	107	mmol/L	95	110
R002	23-May-17	8:02:00	Bicarbonate	28	mmol/L	20	32
R002	23-May-17	8:02:00	Creatinine	78	umol/L	60	110
R002	23-May-17	8:02:00	eGFR	>90			>59
R002	23-May-17	8:02:00	Urea	4.4	mmol/L	3	7.5
R002	23-May-17	8:02:00	Uric Acid	0.348	mmol/L	0.2	0.5
R002	23-May-17	8:02:00	Fasting Glucose	5.3	mmol/L	3.6	6
R002	23-May-17	8:02:00	Total Protein	67	g/L	66	83
R002	23-May-17	8:02:00	Albumin	40	g/L	35	48
R002	23-May-17	8:02:00	Globulin	27	g/L	23	43
R002	23-May-17	8:02:00	Phosphate	1.15	mmol/L	0.8	1.5
R002	23-May-17	8:02:00	Calcium (Corrected)	2.27	mmol/L	2.1	2.6
R002	23-May-17	8:02:00	C Bilirubin	5	umol/L	0	7
R002	23-May-17	8:02:00	Icteric	23		0	200
R002	23-May-17	8:02:00	Haemolysis Index	7		0	40
R002	23-May-17	8:02:00	Lip	0		0	10
R002	23-May-17	8:02:00	Haemoglobin	140	g/L	135	175
R002	23-May-17	8:02:00	Haematocrit	0.41		0.4	0.54
R002	23-May-17	8:02:00	Red cell count	4.7	10^12/L	4.5	6.5
R002	23-May-17	8:02:00	MCV	87	fL	80	100
R002	23-May-17	8:02:00	White cell count	4.4	10^9/L	3.5	10
R002	23-May-17	8:02:00	Neutrophils	1.83	10^9/L	1.5	6.5
R002	23-May-17	8:02:00	Lymphocytes	1.93	10^9/L	1	4
R002	23-May-17	8:02:00	Monocytes	0.41	10^9/L	0	0.9
R002	23-May-17	8:02:00	Eosinophils	0.21	10^9/L	0	0.6
R002	23-May-17	8:02:00	Basophils	0.03	10^9/L	0	0.15
R002	23-May-17	8:02:00	Platelets	157	10^9/L	150	400
R002	23-May-17	8:02:00	Reticulocytes	55	10^9/L	25	120
R002	7-Jun-17	9:58:00	Bilirubin	17	umol/L	4	20
R002	7-Jun-17	9:58:00	Alk Phos	55	U/L	35	110
R002	7-Jun-17	9:58:00	AST	19	U/L	10	40

R002	7-Jun-17	9:58:00	ALT	19	U/L	5	40
R002	7-Jun-17	9:58:00	LDH	193	U/L	120	250
R002	7-Jun-17	9:58:00	Cholesterol	5.5	mmol/L	3.9	5.5
R002	7-Jun-17	9:58:00	Chylomicrons	NIL			
R002	7-Jun-17	9:58:00	HDL	1.36	mmol/L	0.9	1.5
R002	7-Jun-17	9:58:00	LDL	3.6	mmol/L	0	4
R002	7-Jun-17	9:58:00	Triglyceride	1.1	mmol/L	0.6	2
R002	7-Jun-17	9:58:00	Sodium	140	mmol/L	135	145
R002	7-Jun-17	9:58:00	Potassium	4.1	mmol/L	3.5	5.5
R002	7-Jun-17	9:58:00	Chloride	105	mmol/L	95	110
R002	7-Jun-17	9:58:00	Bicarbonate	30	mmol/L	20	32
R002	7-Jun-17	9:58:00	Creatinine	71	umol/L	60	110
R002	7-Jun-17	9:58:00	eGFR	>90			>59
R002	7-Jun-17	9:58:00	Urea	5	mmol/L	3	7.5
R002	7-Jun-17	9:58:00	Uric Acid	0.395	mmol/L	0.2	0.5
R002	7-Jun-17	9:58:00	Fasting Glucose	5.1	mmol/L	3.6	6
R002	7-Jun-17	9:58:00	Total Protein	67	g/L	66	83
R002	7-Jun-17	9:58:00	Albumin	39	g/L	35	48
R002	7-Jun-17	9:58:00	Globulin	28	g/L	23	43
R002	7-Jun-17	9:58:00	Phosphate	1	mmol/L	0.8	1.5
R002	7-Jun-17	9:58:00	Calcium (Corrected)	2.31	mmol/L	2.1	2.6
R002	7-Jun-17	9:58:00	C Bilirubin	5	umol/L	0	7
R002	7-Jun-17	9:58:00	Tot Chol/HDL	4		0	4.5
R002	7-Jun-17	9:58:00	Magnesium	0.84	mmol/L	0.7	1.1
R002	7-Jun-17	9:58:00	Icteric	26		0	200
R002	7-Jun-17	9:58:00	Haemolysis Index	7		0	40
R002	7-Jun-17	9:58:00	Lip	0		0	10
R002	7-Jun-17	9:58:00	G6PD	1776.4	U/L		
R002	7-Jun-17	9:58:00	Haemoglobin	129	g/L		
R002	7-Jun-17	9:58:00	G6PD/Hb	13.8	U/g Hb	7	20.5
R002	7-Jun-17	9:58:00	HepB surface antigen	N			
R002	7-Jun-17	9:58:00	Hepatitis B Core Ab	N			
R002	7-Jun-17	9:58:00	HepC-IgG antibody	N			
R002	7-Jun-17	9:58:00	HIV Ag/Ab	N			
R002	7-Jun-17	9:58:00	Haemoglobin	139	g/L	135	175
R002	7-Jun-17	9:58:00	Haematocrit	0.41		0.4	0.54
R002	7-Jun-17	9:58:00	Red cell count	4.7	10^12/L	4.5	6.5
R002	7-Jun-17	9:58:00	MCV	87	fL	80	100
R002	7-Jun-17	9:58:00	White cell count	4.1	10^9/L	3.5	10
R002	7-Jun-17	9:58:00	Neutrophils	2.08	10^9/L	1.5	6.5
R002	7-Jun-17	9:58:00	Lymphocytes	1.58	10^9/L	1	4
R002	7-Jun-17	9:58:00	Monocytes	0.24	10^9/L	0	0.9
R002	7-Jun-17	9:58:00	Eosinophils	0.21	10^9/L	0	0.6
R002	7-Jun-17	9:58:00	Basophils	0.02	10^9/L	0	0.15
R002	7-Jun-17	9:58:00	Platelets	174	10^9/L	150	400
R002	7-Jun-17	9:58:00	Blood Group	BPOS			
R002	7-Jun-17	9:58:00	Antibody Screen	N			
R002	13-Jun-17	9:15:00	Bilirubin	8	umol/L	4	20
R002	13-Jun-17	9:15:00	Alk Phos	77	U/L	35	110
R002	13-Jun-17	9:15:00	AST	22	U/L	10	40

R002	13-Jun-17	9:15:00	ALT	25	U/L	5	40
R002	13-Jun-17	9:15:00	LDH	217	U/L	120	250
R002	13-Jun-17	9:15:00	Sodium	140	mmol/L	135	145
R002	13-Jun-17	9:15:00	Potassium	4.3	mmol/L	3.5	5.5
R002	13-Jun-17	9:15:00	Chloride	105	mmol/L	95	110
R002	13-Jun-17	9:15:00	Bicarbonate	27	mmol/L	20	32
R002	13-Jun-17	9:15:00	Creatinine	86	umol/L	60	110
R002	13-Jun-17	9:15:00	eGFR	>90			>59
R002	13-Jun-17	9:15:00	Urea	9.6	mmol/L	3	7.5
R002	13-Jun-17	9:15:00	Uric Acid	0.387	mmol/L	0.2	0.5
R002	13-Jun-17	9:15:00	Fasting Glucose	5.5	mmol/L	3.6	6
R002	13-Jun-17	9:15:00	Total Protein	81	g/L	66	83
R002	13-Jun-17	9:15:00	Albumin	45	g/L	35	48
R002	13-Jun-17	9:15:00	Globulin	36	g/L	23	43
R002	13-Jun-17	9:15:00	Phosphate	1.21	mmol/L	0.8	1.5
R002	13-Jun-17	9:15:00	Calcium (Corrected)	2.31	mmol/L	2.1	2.6
R002	13-Jun-17	9:15:00	C Bilirubin	3	umol/L	0	7
R002	13-Jun-17	9:15:00	Icteric	14		0	200
R002	13-Jun-17	9:15:00	Haemolysis Index	11		0	40
R002	13-Jun-17	9:15:00	Lip	0		0	10
R002	13-Jun-17	9:15:00	Haemoglobin	153	g/L	135	175
R002	13-Jun-17	9:15:00	Haematocrit	0.45		0.4	0.54
R002	13-Jun-17	9:15:00	Red cell count	5.2	10^12/L	4.5	6.5
R002	13-Jun-17	9:15:00	MCV	87	fL	80	100
R002	13-Jun-17	9:15:00	White cell count	5.1	10^9/L	3.5	10
R002	13-Jun-17	9:15:00	Neutrophils	2.35	10^9/L	1.5	6.5
R002	13-Jun-17	9:15:00	Lymphocytes	2.12	10^9/L	1	4
R002	13-Jun-17	9:15:00	Monocytes	0.36	10^9/L	0	0.9
R002	13-Jun-17	9:15:00	Eosinophils	0.19	10^9/L	0	0.6
R002	13-Jun-17	9:15:00	Basophils	0.03	10^9/L	0	0.15
R002	13-Jun-17	9:15:00	Platelets	181	10^9/L	150	400
R002	13-Jun-17	9:15:00	Reticulocytes	65	10^9/L	25	120
R002	23-Jun-17	9:09:00	Potassium (Corrected)	4.2	mmol/L	3.5	5.5
R002	23-Jun-17	9:09:00	Bilirubin	12	umol/L	4	20
R002	23-Jun-17	9:09:00	Alk Phos	53	U/L	35	110
R002	23-Jun-17	9:09:00	AST	:SPU	U/L		
R002	23-Jun-17	9:09:00	ALT	18	U/L	5	40
R002	23-Jun-17	9:09:00	LDH	:SPU	U/L		
R002	23-Jun-17	9:09:00	Sodium	138	mmol/L	135	145
R002	23-Jun-17	9:09:00	Potassium	5	mmol/L	3.5	5.5
R002	23-Jun-17	9:09:00	Chloride	107	mmol/L	95	110
R002	23-Jun-17	9:09:00	Bicarbonate	23	mmol/L	20	32
R002	23-Jun-17	9:09:00	Creatinine	83	umol/L	60	110
R002	23-Jun-17	9:09:00	eGFR	>90			>59
R002	23-Jun-17	9:09:00	Urea	7.3	mmol/L	3	7.5
R002	23-Jun-17	9:09:00	Uric Acid	0.412	mmol/L	0.2	0.5
R002	23-Jun-17	9:09:00	Fasting Glucose	5.1	mmol/L	3.6	6
R002	23-Jun-17	9:09:00	Total Protein	:SPU	g/L		
R002	23-Jun-17	9:09:00	Albumin	36	g/L	35	48
R002	23-Jun-17	9:09:00	Globulin	:SPU	g/L		

R002	23-Jun-17	9:09:00	Phosphate	:SPU	mmol/L		
R002	23-Jun-17	9:09:00	Calcium (Corrected)	2.19	mmol/L	2.1	2.6
R002	23-Jun-17	9:09:00	C Bilirubin	3	umol/L	0	7
R002	23-Jun-17	9:09:00	Icteric	2		0	200
R002	23-Jun-17	9:09:00	Haemolysis Index	223		0	40
R002	23-Jun-17	9:09:00	Lip	0		0	10
R002	23-Jun-17	9:09:00	Haemoglobin	137	g/L	135	175
R002	23-Jun-17	9:09:00	Haematocrit	0.4		0.4	0.54
R002	23-Jun-17	9:09:00	Red cell count	4.6	10^12/L	4.5	6.5
R002	23-Jun-17	9:09:00	MCV	87	fL	80	100
R002	23-Jun-17	9:09:00	White cell count	3.8	10^9/L	3.5	10
R002	23-Jun-17	9:09:00	Neutrophils	1.9	10^9/L	1.5	6.5
R002	23-Jun-17	9:09:00	Lymphocytes	1.4	10^9/L	1	4
R002	23-Jun-17	9:09:00	Monocytes	0.33	10^9/L	0	0.9
R002	23-Jun-17	9:09:00	Eosinophils	0.16	10^9/L	0	0.6
R002	23-Jun-17	9:09:00	Basophils	0.02	10^9/L	0	0.15
R002	23-Jun-17	9:09:00	Platelets	162	10^9/L	150	400
R002	24-Jun-17	11:37:00	Bilirubin	9	umol/L	4	20
R002	24-Jun-17	11:37:00	Alk Phos	65	U/L	35	110
R002	24-Jun-17	11:37:00	AST	19	U/L	10	40
R002	24-Jun-17	11:37:00	ALT	18	U/L	5	40
R002	24-Jun-17	11:37:00	LDH	201	U/L	120	250
R002	24-Jun-17	11:37:00	Sodium	139	mmol/L	135	145
R002	24-Jun-17	11:37:00	Potassium	3.9	mmol/L	3.5	5.5
R002	24-Jun-17	11:37:00	Chloride	103	mmol/L	95	110
R002	24-Jun-17	11:37:00	Bicarbonate	29	mmol/L	20	32
R002	24-Jun-17	11:37:00	Creatinine	82	umol/L	60	110
R002	24-Jun-17	11:37:00	eGFR	>90			>59
R002	24-Jun-17	11:37:00	Urea	3.5	mmol/L	3	7.5
R002	24-Jun-17	11:37:00	Uric Acid	0.323	mmol/L	0.2	0.5
R002	24-Jun-17	11:37:00	Random Glucose	5.2	mmol/L	3.6	7.7
R002	24-Jun-17	11:37:00	Total Protein	69	g/L	66	83
R002	24-Jun-17	11:37:00	Albumin	40	g/L	35	48
R002	24-Jun-17	11:37:00	Globulin	29	g/L	23	43
R002	24-Jun-17	11:37:00	Phosphate	1.12	mmol/L	0.8	1.5
R002	24-Jun-17	11:37:00	Calcium (Corrected)	2.36	mmol/L	2.1	2.6
R002	24-Jun-17	11:37:00	C Bilirubin	3	umol/L	0	7
R002	24-Jun-17	11:37:00	Icteric	13		0	200
R002	24-Jun-17	11:37:00	Haemolysis Index	9		0	40
R002	24-Jun-17	11:37:00	Lip	0		0	10
R002	25-Jun-17	15:44:00	Bilirubin	5	umol/L	4	20
R002	25-Jun-17	15:44:00	Alk Phos	72	U/L	35	110
R002	25-Jun-17	15:44:00	AST	22	U/L	10	40
R002	25-Jun-17	15:44:00	ALT	19	U/L	5	40
R002	25-Jun-17	15:44:00	LDH	214	U/L	120	250
R002	25-Jun-17	15:44:00	Sodium	138	mmol/L	135	145
R002	25-Jun-17	15:44:00	Potassium	4.3	mmol/L	3.5	5.5
R002	25-Jun-17	15:44:00	Chloride	105	mmol/L	95	110
R002	25-Jun-17	15:44:00	Bicarbonate	26	mmol/L	20	32
R002	25-Jun-17	15:44:00	Creatinine	83	umol/L	60	110

R002	25-Jun-17	15:44:00	eGFR	>90			>59
R002	25-Jun-17	15:44:00	Urea	4.4	mmol/L	3	7.5
R002	25-Jun-17	15:44:00	Uric Acid	0.263	mmol/L	0.2	0.5
R002	25-Jun-17	15:44:00	Fasting Glucose	5.3	mmol/L	3.6	6
R002	25-Jun-17	15:44:00	Total Protein	71	g/L	66	83
R002	25-Jun-17	15:44:00	Albumin	42	g/L	35	48
R002	25-Jun-17	15:44:00	Globulin	29	g/L	23	43
R002	25-Jun-17	15:44:00	Phosphate	1.14	mmol/L	0.8	1.5
R002	25-Jun-17	15:44:00	Calcium (Corrected)	2.23	mmol/L	2.1	2.6
R002	25-Jun-17	15:44:00	C Bilirubin	3	umol/L	0	7
R002	25-Jun-17	15:44:00	Icteric	12		0	200
R002	25-Jun-17	15:44:00	Haemolysis Index	7		0	40
R002	25-Jun-17	15:44:00	Lip	0		0	10
R002	25-Jun-17	15:44:00	Haemoglobin	154	g/L	135	175
R002	25-Jun-17	15:44:00	Haematocrit	0.44		0.4	0.54
R002	25-Jun-17	15:44:00	Red cell count	5.2	10^12/L	4.5	6.5
R002	25-Jun-17	15:44:00	MCV	86	fL	80	100
R002	25-Jun-17	15:44:00	White cell count	2.9	10^9/L	3.5	10
R002	25-Jun-17	15:44:00	Neutrophils	1.81	10^9/L	1.5	6.5
R002	25-Jun-17	15:44:00	Lymphocytes	0.61	10^9/L	1	4
R002	25-Jun-17	15:44:00	Monocytes	0.41	10^9/L	0	0.9
R002	25-Jun-17	15:44:00	Eosinophils	0.07	10^9/L	0	0.6
R002	25-Jun-17	15:44:00	Basophils	0.01	10^9/L	0	0.15
R002	25-Jun-17	15:44:00	Platelets	150	10^9/L	150	400
R002	26-Jun-17	9:23:00	Potassium (Corrected)	4.4	mmol/L	3.5	5.5
R002	26-Jun-17	9:23:00	Bilirubin	7	umol/L	4	20
R002	26-Jun-17	9:23:00	Alk Phos	63	U/L	35	110
R002	26-Jun-17	9:23:00	AST	:SPU	U/L		
R002	26-Jun-17	9:23:00	ALT	21	U/L	5	40
R002	26-Jun-17	9:23:00	LDH	398	U/L	120	250
R002	26-Jun-17	9:23:00	Sodium	133	mmol/L	135	145
R002	26-Jun-17	9:23:00	Potassium	4.9	mmol/L	3.5	5.5
R002	26-Jun-17	9:23:00	Chloride	101	mmol/L	95	110
R002	26-Jun-17	9:23:00	Bicarbonate	24	mmol/L	20	32
R002	26-Jun-17	9:23:00	Creatinine	80	umol/L	60	110
R002	26-Jun-17	9:23:00	eGFR	>90			>59
R002	26-Jun-17	9:23:00	Urea	3.7	mmol/L	3	7.5
R002	26-Jun-17	9:23:00	Uric Acid	0.301	mmol/L	0.2	0.5
R002	26-Jun-17	9:23:00	Random Glucose	7.2	mmol/L	3.6	7.7
R002	26-Jun-17	9:23:00	Total Protein	:SPU	g/L		
R002	26-Jun-17	9:23:00	Albumin	39	g/L	35	48
R002	26-Jun-17	9:23:00	Globulin	:SPU	g/L		
R002	26-Jun-17	9:23:00	Phosphate	:SPU	mmol/L		
R002	26-Jun-17	9:23:00	Calcium (Corrected)	2.32	mmol/L	2.1	2.6
R002	26-Jun-17	9:23:00	C Bilirubin	2	umol/L	0	7
R002	26-Jun-17	9:23:00	Icteric	5		0	200
R002	26-Jun-17	9:23:00	Haemolysis Index	152		0	40
R002	26-Jun-17	9:23:00	Lip	0		0	10
R002	26-Jun-17	9:23:00	Haemoglobin	153	g/L	135	175
R002	26-Jun-17	9:23:00	Haematocrit	0.44		0.4	0.54

R002	26-Jun-17	9:23:00	Red cell count	5.2	10^12/L	4.5	6.5
R002	26-Jun-17	9:23:00	MCV	85	fL	80	100
R002	26-Jun-17	9:23:00	White cell count	2.5	10^9/L	3.5	10
R002	26-Jun-17	9:23:00	Neutrophils	1.58	10^9/L	1.5	6.5
R002	26-Jun-17	9:23:00	Lymphocytes	0.62	10^9/L	1	4
R002	26-Jun-17	9:23:00	Monocytes	0.27	10^9/L	0	0.9
R002	26-Jun-17	9:23:00	Eosinophils	0.02	10^9/L	0	0.6
R002	26-Jun-17	9:23:00	Basophils	0.01	10^9/L	0	0.15
R002	26-Jun-17	9:23:00	Platelets	136	10^9/L	150	400
R002	28-Jun-17	9:11:00	Bilirubin	7	umol/L	4	20
R002	28-Jun-17	9:11:00	Alk Phos	76	U/L	35	110
R002	28-Jun-17	9:11:00	AST	39	U/L	10	40
R002	28-Jun-17	9:11:00	ALT	37	U/L	5	40
R002	28-Jun-17	9:11:00	LDH	344	U/L	120	250
R002	28-Jun-17	9:11:00	Sodium	137	mmol/L	135	145
R002	28-Jun-17	9:11:00	Potassium	4.4	mmol/L	3.5	5.5
R002	28-Jun-17	9:11:00	Chloride	104	mmol/L	95	110
R002	28-Jun-17	9:11:00	Bicarbonate	27	mmol/L	20	32
R002	28-Jun-17	9:11:00	Creatinine	71	umol/L	60	110
R002	28-Jun-17	9:11:00	eGFR	>90			>59
R002	28-Jun-17	9:11:00	Urea	7.5	mmol/L	3	7.5
R002	28-Jun-17	9:11:00	Uric Acid	0.356	mmol/L	0.2	0.5
R002	28-Jun-17	9:11:00	Fasting Glucose	5.3	mmol/L	3.6	6
R002	28-Jun-17	9:11:00	Total Protein	70	g/L	66	83
R002	28-Jun-17	9:11:00	Albumin	38	g/L	35	48
R002	28-Jun-17	9:11:00	Globulin	32	g/L	23	43
R002	28-Jun-17	9:11:00	Phosphate	0.98	mmol/L	0.8	1.5
R002	28-Jun-17	9:11:00	Calcium (Corrected)	2.29	mmol/L	2.1	2.6
R002	28-Jun-17	9:11:00	C Bilirubin	2	umol/L	0	7
R002	28-Jun-17	9:11:00	Icteric	12		0	200
R002	28-Jun-17	9:11:00	Haemolysis Index	17		0	40
R002	28-Jun-17	9:11:00	Lip	1		0	10
R002	28-Jun-17	9:11:00	Haemoglobin	137	g/L	135	175
R002	28-Jun-17	9:11:00	Haematocrit	0.38		0.4	0.54
R002	28-Jun-17	9:11:00	Red cell count	4.5	10^12/L	4.5	6.5
R002	28-Jun-17	9:11:00	MCV	85	fL	80	100
R002	28-Jun-17	9:11:00	White cell count	3.2	10^9/L	3.5	10
R002	28-Jun-17	9:11:00	Neutrophils	1.15	10^9/L	1.5	6.5
R002	28-Jun-17	9:11:00	Lymphocytes	1.47	10^9/L	1	4
R002	28-Jun-17	9:11:00	Monocytes	0.38	10^9/L	0	0.9
R002	28-Jun-17	9:11:00	Eosinophils	0.18	10^9/L	0	0.6
R002	28-Jun-17	9:11:00	Basophils	0.03	10^9/L	0	0.15
R002	28-Jun-17	9:11:00	Platelets	122	10^9/L	150	400
R002	29-Jun-17	8:13:00	Bilirubin	9	umol/L	4	20
R002	29-Jun-17	8:13:00	Alk Phos	60	U/L	35	110
R002	29-Jun-17	8:13:00	AST	41	U/L	10	40
R002	29-Jun-17	8:13:00	ALT	49	U/L	5	40
R002	29-Jun-17	8:13:00	LDH	346	U/L	120	250
R002	29-Jun-17	8:13:00	Sodium	139	mmol/L	135	145
R002	29-Jun-17	8:13:00	Potassium	4.2	mmol/L	3.5	5.5

R002	29-Jun-17	8:13:00	Chloride	104	mmol/L	95	110
R002	29-Jun-17	8:13:00	Bicarbonate	29	mmol/L	20	32
R002	29-Jun-17	8:13:00	Creatinine	77	umol/L	60	110
R002	29-Jun-17	8:13:00	eGFR	>90			>59
R002	29-Jun-17	8:13:00	Urea	6.1	mmol/L	3	7.5
R002	29-Jun-17	8:13:00	Uric Acid	0.385	mmol/L	0.2	0.5
R002	29-Jun-17	8:13:00	Fasting Glucose	7.5	mmol/L	3.6	6
R002	29-Jun-17	8:13:00	Total Protein	68	g/L	66	83
R002	29-Jun-17	8:13:00	Albumin	39	g/L	35	48
R002	29-Jun-17	8:13:00	Globulin	29	g/L	23	43
R002	29-Jun-17	8:13:00	Phosphate	1.14	mmol/L	0.8	1.5
R002	29-Jun-17	8:13:00	Calcium (Corrected)	2.27	mmol/L	2.1	2.6
R002	29-Jun-17	8:13:00	C Bilirubin	3	umol/L	0	7
R002	29-Jun-17	8:13:00	Icteric	14		0	200
R002	29-Jun-17	8:13:00	Haemolysis Index	3		0	40
R002	29-Jun-17	8:13:00	Lip	0		0	10
R002	29-Jun-17	8:13:00	Haemoglobin	135	g/L	135	175
R002	29-Jun-17	8:13:00	Haematocrit	0.38		0.4	0.54
R002	29-Jun-17	8:13:00	Red cell count	4.5	10^12/L	4.5	6.5
R002	29-Jun-17	8:13:00	MCV	83	fL	80	100
R002	29-Jun-17	8:13:00	White cell count	3.1	10^9/L	3.5	10
R002	29-Jun-17	8:13:00	Neutrophils	1.36	10^9/L	1.5	6.5
R002	29-Jun-17	8:13:00	Lymphocytes	1.15	10^9/L	1	4
R002	29-Jun-17	8:13:00	Monocytes	0.09	10^9/L	0	0.9
R002	29-Jun-17	8:13:00	Eosinophils	0.22	10^9/L	0	0.6
R002	29-Jun-17	8:13:00	Atypical lymphocytes	0.28	10^9/L	0	0.01
R002	29-Jun-17	8:13:00	Platelets	144	10^9/L	150	400
R002	2-Jul-17	8:46:00	Bilirubin	10	umol/L	4	20
R002	2-Jul-17	8:46:00	Alk Phos	60	U/L	35	110
R002	2-Jul-17	8:46:00	AST	44	U/L	10	40
R002	2-Jul-17	8:46:00	ALT	75	U/L	5	40
R002	2-Jul-17	8:46:00	LDH	286	U/L	120	250
R002	2-Jul-17	8:46:00	Sodium	141	mmol/L	135	145
R002	2-Jul-17	8:46:00	Potassium	4.5	mmol/L	3.5	5.5
R002	2-Jul-17	8:46:00	Chloride	107	mmol/L	95	110
R002	2-Jul-17	8:46:00	Bicarbonate	26	mmol/L	20	32
R002	2-Jul-17	8:46:00	Creatinine	85	umol/L	60	110
R002	2-Jul-17	8:46:00	eGFR	>90			>59
R002	2-Jul-17	8:46:00	Urea	6	mmol/L	3	7.5
R002	2-Jul-17	8:46:00	Uric Acid	0.482	mmol/L	0.2	0.5
R002	2-Jul-17	8:46:00	Fasting Glucose	5.5	mmol/L	3.6	6
R002	2-Jul-17	8:46:00	Total Protein	73	g/L	66	83
R002	2-Jul-17	8:46:00	Albumin	42	g/L	35	48
R002	2-Jul-17	8:46:00	Globulin	31	g/L	23	43
R002	2-Jul-17	8:46:00	Phosphate	1.27	mmol/L	0.8	1.5
R002	2-Jul-17	8:46:00	Calcium (Corrected)	2.26	mmol/L	2.1	2.6
R002	2-Jul-17	8:46:00	C Bilirubin	4	umol/L	0	7
R002	2-Jul-17	8:46:00	Icteric	16		0	200
R002	2-Jul-17	8:46:00	Haemolysis Index	2		0	40
R002	2-Jul-17	8:46:00	Lip	0		0	10

R002	2-Jul-17	8:46:00	Haemoglobin	142	g/L	135	175
R002	2-Jul-17	8:46:00	Haematocrit	0.41		0.4	0.54
R002	2-Jul-17	8:46:00	Red cell count	4.9	10^12/L	4.5	6.5
R002	2-Jul-17	8:46:00	MCV	84	fL	80	100
R002	2-Jul-17	8:46:00	White cell count	4.9	10^9/L	3.5	10
R002	2-Jul-17	8:46:00	Neutrophils	2.33	10^9/L	1.5	6.5
R002	2-Jul-17	8:46:00	Lymphocytes	2.1	10^9/L	1	4
R002	2-Jul-17	8:46:00	Monocytes	0.31	10^9/L	0	0.9
R002	2-Jul-17	8:46:00	Eosinophils	0.17	10^9/L	0	0.6
R002	2-Jul-17	8:46:00	Basophils	0.03	10^9/L	0	0.15
R002	2-Jul-17	8:46:00	Platelets	200	10^9/L	150	400
R002	4-Jul-17	8:15:00	Bilirubin	9	umol/L	4	20
R002	4-Jul-17	8:15:00	Alk Phos	172	U/L	35	110
R002	4-Jul-17	8:15:00	AST	30	U/L	10	40
R002	4-Jul-17	8:15:00	ALT	69	U/L	5	40
R002	4-Jul-17	8:15:00	LDH	264	U/L	120	250
R002	4-Jul-17	8:15:00	Sodium	138	mmol/L	135	145
R002	4-Jul-17	8:15:00	Potassium	4.3	mmol/L	3.5	5.5
R002	4-Jul-17	8:15:00	Chloride	104	mmol/L	95	110
R002	4-Jul-17	8:15:00	Bicarbonate	29	mmol/L	20	32
R002	4-Jul-17	8:15:00	Creatinine	75	umol/L	60	110
R002	4-Jul-17	8:15:00	eGFR	>90			>59
R002	4-Jul-17	8:15:00	Urea	7	mmol/L	3	7.5
R002	4-Jul-17	8:15:00	Uric Acid	0.409	mmol/L	0.2	0.5
R002	4-Jul-17	8:15:00	Fasting Glucose	5.6	mmol/L	3.6	6
R002	4-Jul-17	8:15:00	Total Protein	70	g/L	66	83
R002	4-Jul-17	8:15:00	Albumin	40	g/L	35	48
R002	4-Jul-17	8:15:00	Globulin	30	g/L	23	43
R002	4-Jul-17	8:15:00	Phosphate	1.21	mmol/L	0.8	1.5
R002	4-Jul-17	8:15:00	Calcium (Corrected)	2.33	mmol/L	2.1	2.6
R002	4-Jul-17	8:15:00	C Bilirubin	3	umol/L	0	7
R002	4-Jul-17	8:15:00	Icteric	14		0	200
R002	4-Jul-17	8:15:00	Haemolysis Index	4		0	40
R002	4-Jul-17	8:15:00	Lip	0		0	10
R002	6-Jul-17	8:16:00	Bilirubin	11	umol/L	4	20
R002	6-Jul-17	8:16:00	Alk Phos	66	U/L	35	110
R002	6-Jul-17	8:16:00	AST	34	U/L	10	40
R002	6-Jul-17	8:16:00	AST/ALT Ratio	0.5		0.5	100
R002	6-Jul-17	8:16:00	ALT	70	U/L	5	40
R002	6-Jul-17	8:16:00	Gamma GT	20	U/L	5	50
R002	6-Jul-17	8:16:00	Total Protein	72	g/L	66	83
R002	6-Jul-17	8:16:00	Albumin	41	g/L	35	48
R002	6-Jul-17	8:16:00	Globulin	31	g/L	23	43
R002	6-Jul-17	8:16:00	Icteric	15		0	200
R002	6-Jul-17	8:16:00	Haemolysis Index	3		0	40
R002	6-Jul-17	8:16:00	Lip	0		0	10
R002	10-Jul-17	8:52:00	Bilirubin	16	umol/L	4	20
R002	10-Jul-17	8:52:00	Alk Phos	59	U/L	35	110
R002	10-Jul-17	8:52:00	AST	23	U/L	10	40
R002	10-Jul-17	8:52:00	ALT	44	U/L	5	40

R002	10-Jul-17	8:52:00	LDH	240	U/L	120	250
R002	10-Jul-17	8:52:00	Sodium	139	mmol/L	135	145
R002	10-Jul-17	8:52:00	Potassium	3.9	mmol/L	3.5	5.5
R002	10-Jul-17	8:52:00	Chloride	104	mmol/L	95	110
R002	10-Jul-17	8:52:00	Bicarbonate	28	mmol/L	20	32
R002	10-Jul-17	8:52:00	Creatinine	82	umol/L	60	110
R002	10-Jul-17	8:52:00	eGFR	>90			>59
R002	10-Jul-17	8:52:00	Urea	5.2	mmol/L	3	7.5
R002	10-Jul-17	8:52:00	Uric Acid	0.401	mmol/L	0.2	0.5
R002	10-Jul-17	8:52:00	Random Glucose	6.9	mmol/L	3.6	7.7
R002	10-Jul-17	8:52:00	Total Protein	70	g/L	66	83
R002	10-Jul-17	8:52:00	Albumin	40	g/L	35	48
R002	10-Jul-17	8:52:00	Globulin	30	g/L	23	43
R002	10-Jul-17	8:52:00	Phosphate	1.14	mmol/L	0.8	1.5
R002	10-Jul-17	8:52:00	Calcium (Corrected)	2.33	mmol/L	2.1	2.6
R002	10-Jul-17	8:52:00	C Bilirubin	5	umol/L	0	7
R002	10-Jul-17	8:52:00	Icteric	20		0	200
R002	10-Jul-17	8:52:00	Haemolysis Index	4		0	40
R002	10-Jul-17	8:52:00	Lip	0		0	10
R002	10-Jul-17	8:52:00	Haemoglobin	137	g/L	135	175
R002	10-Jul-17	8:52:00	Haematocrit	0.4		0.4	0.54
R002	10-Jul-17	8:52:00	Red cell count	4.7	10^12/L	4.5	6.5
R002	10-Jul-17	8:52:00	MCV	85	fL	80	100
R002	10-Jul-17	8:52:00	White cell count	5.2	10^9/L	3.5	10
R002	10-Jul-17	8:52:00	Neutrophils	2.79	10^9/L	1.5	6.5
R002	10-Jul-17	8:52:00	Lymphocytes	1.31	10^9/L	1	4
R002	10-Jul-17	8:52:00	Monocytes	0.6	10^9/L	0	0.9
R002	10-Jul-17	8:52:00	Eosinophils	0.44	10^9/L	0	0.6
R002	10-Jul-17	8:52:00	Basophils	0.02	10^9/L	0	0.15
R002	10-Jul-17	8:52:00	Platelets	202	10^9/L	150	400
R002	12-Jul-17	9:30:00	Bilirubin	14	umol/L	4	20
R002	12-Jul-17	9:30:00	Alk Phos	65	U/L	35	110
R002	12-Jul-17	9:30:00	AST	20	U/L	10	40
R002	12-Jul-17	9:30:00	ALT	39	U/L	5	40
R002	12-Jul-17	9:30:00	LDH	232	U/L	120	250
R002	12-Jul-17	9:30:00	Sodium	139	mmol/L	135	145
R002	12-Jul-17	9:30:00	Potassium	4.4	mmol/L	3.5	5.5
R002	12-Jul-17	9:30:00	Chloride	103	mmol/L	95	110
R002	12-Jul-17	9:30:00	Bicarbonate	29	mmol/L	20	32
R002	12-Jul-17	9:30:00	Creatinine	86	umol/L	60	110
R002	12-Jul-17	9:30:00	eGFR	>90			>59
R002	12-Jul-17	9:30:00	Urea	6.2	mmol/L	3	7.5
R002	12-Jul-17	9:30:00	Uric Acid	0.434	mmol/L	0.2	0.5
R002	12-Jul-17	9:30:00	Fasting Glucose	5.5	mmol/L	3.6	6
R002	12-Jul-17	9:30:00	Total Protein	74	g/L	66	83
R002	12-Jul-17	9:30:00	Albumin	41	g/L	35	48
R002	12-Jul-17	9:30:00	Globulin	33	g/L	23	43
R002	12-Jul-17	9:30:00	Phosphate	1.17	mmol/L	0.8	1.5
R002	12-Jul-17	9:30:00	Calcium (Corrected)	2.36	mmol/L	2.1	2.6
R002	12-Jul-17	9:30:00	C Bilirubin	5	umol/L	0	7

R002	12-Jul-17	9:30:00	Icteric	18		0	200
R002	12-Jul-17	9:30:00	Haemolysis Index	3		0	40
R002	12-Jul-17	9:30:00	Lip	0		0	10
R002	12-Jul-17	9:30:00	HepB surface antigen	N			
R002	12-Jul-17	9:30:00	Hepatitis B Core Ab	N			
R002	12-Jul-17	9:30:00	HepC-IgG antibody	N			
R002	12-Jul-17	9:30:00	HIV Ag/Ab	N			
R002	12-Jul-17	9:30:00	Haemoglobin	141	g/L	135	175
R002	12-Jul-17	9:30:00	Haematocrit	0.4		0.4	0.54
R002	12-Jul-17	9:30:00	Red cell count	4.7	10^12/L	4.5	6.5
R002	12-Jul-17	9:30:00	MCV	84	fL	80	100
R002	12-Jul-17	9:30:00	White cell count	4	10^9/L	3.5	10
R002	12-Jul-17	9:30:00	Neutrophils	1.66	10^9/L	1.5	6.5
R002	12-Jul-17	9:30:00	Lymphocytes	1.52	10^9/L	1	4
R002	12-Jul-17	9:30:00	Monocytes	0.39	10^9/L	0	0.9
R002	12-Jul-17	9:30:00	Eosinophils	0.38	10^9/L	0	0.6
R002	12-Jul-17	9:30:00	Basophils	0.02	10^9/L	0	0.15
R002	12-Jul-17	9:30:00	Platelets	200	10^9/L	150	400
R002	12-Jul-17	9:30:00	Reticulocytes	65	10^9/L	25	120
R002	12-Jul-17	9:30:00	Blood Group	BPOS			
R002	12-Jul-17	9:30:00	Antibody Screen	N			
R002	12-Sep-17	9:43:00	Blood Group	BPOS			
R002	12-Sep-17	9:43:00	Antibody Screen	N			

16.2.8.1 Individual abnormal haematology results

Listing 16.2.8.1
Individual Abnormal Haematology Results

Subject Number	Visit	Test	Unit	Normal Range	Date of Measurement	Time of Measurement	Study Day	Day Relative to Artesunate	Result	Range Indicator	Clinical Assessment
R001	Pre-artesunate	White cell count	10^9/L	[3.5 - 10]	02-Jun-2017	8:13	Day 9	1	3.4	L	NCS
R001	Unscheduled Haematology	White cell count	10^9/L	[3.5 - 10]	04-Jun-2017	16:06	Day 11	3	3	L	NCS
R001	Unscheduled Haematology	Neutrophils	10^9/L	[1.5 - 6.5]	04-Jun-2017	16:06	Day 11	3	1.42	L	CS
R001	Unscheduled Haematology	Lymphocytes	10^9/L	[1 - 4]	04-Jun-2017	16:06	Day 11	3	0.89	L	NCS
R001	Admission 3	White cell count	10^9/L	[3.5 - 10]	05-Jun-2017	8:23	Day 12	4	3	L	NCS
R001	Admission 3	Lymphocytes	10^9/L	[1 - 4]	05-Jun-2017	8:23	Day 12	4	0.67	L	CS
R001	Safety monitoring	White cell count	10^9/L	[3.5 - 10]	07-Jun-2017	7:54	Day 14	6	3.1	L	NCS
R001	Safety monitoring	Platelets	10^9/L	[150 - 400]	07-Jun-2017	7:54	Day 14	6	139	L	NCS
R001	Safety monitoring	Neutrophils	10^9/L	[1.5 - 6.5]	07-Jun-2017	7:54	Day 14	6	1.23	L	CS
R001	Safety monitoring	White cell count	10^9/L	[3.5 - 10]	21-Jun-2017	8:38	Day 28	20	3.4	L	NCS
R002	Unscheduled Haematology	White cell count	10^9/L	[3.5 - 10]	25-Jun-2017	15:44	Day 11	3	2.9	L	CS
R002	Unscheduled Haematology	Lymphocytes	10^9/L	[1 - 4]	25-Jun-2017	15:44	Day 11	3	0.61	L	CS
R002	Admission 3	White cell count	10^9/L	[3.5 - 10]	26-Jun-2017	9:23	Day 12	4	2.5	L	CS
R002	Admission 3	Lymphocytes	10^9/L	[1 - 4]	26-Jun-2017	9:23	Day 12	4	0.62	L	CS
R002	Admission 3	Platelets	10^9/L	[150 - 400]	26-Jun-2017	9:23	Day 12	4	136	L	NCS
R002	Safety monitoring	Haematocrit		[0.4 - 0.54]	28-Jun-2017	9:11	Day 14	6	0.38	L	NCS
R002	Safety monitoring	White cell count	10^9/L	[3.5 - 10]	28-Jun-2017	9:11	Day 14	6	3.2	L	NCS
R002	Safety monitoring	Neutrophils	10^9/L	[1.5 - 6.5]	28-Jun-2017	9:11	Day 14	6	1.15	L	CS
R002	Safety monitoring	Platelets	10^9/L	[150 - 400]	28-Jun-2017	9:11	Day 14	6	122	L	NCS
R002	Safety monitoring	Haematocrit		[0.4 - 0.54]	29-Jun-2017	8:13	Day 15	7	0.38	L	NCS
R002	Safety monitoring	White cell count	10^9/L	[3.5 - 10]	29-Jun-2017	8:13	Day 15	7	3.1	L	NCS
R002	Safety monitoring	Neutrophils	10^9/L	[1.5 - 6.5]	29-Jun-2017	8:13	Day 15	7	1.36	L	CS
R002	Safety monitoring	Atypical lymphocytes	10^9/L	[0 - 0.01]	29-Jun-2017	8:13	Day 15	7	0.28	H	NCS
R002	Safety monitoring	Platelets	10^9/L	[150 - 400]	29-Jun-2017	8:13	Day 15	7	144	L	NCS

Notes:
Study Day is the number of days relative to day of administration of the inoculum, where Study Day = 0 for inoculum dosing day.
Day relative to Artesunate is the number of days relative to day of administration of Artesunate where the day of first dose = 1.
H = High; L = Low; CS = Clinically Significant; NCS = Not Clinically Significant.
Clinical laboratory evaluation for Haematology were performed for all participants as per the protocol. Only abnormal results are recorded in this listing.

16.2.8.2 Individual abnormal biochemistry results

Listing 16.2.8.2
Individual Abnormal Biochemistry Results

Subject Number	Visit	Test	Unit	Normal Range	Date of Measurement	Time of Measurement	Study Day	Day Relative to Artesunate	Result	Range Indicator	Clinical Assessment
R001	Screening	Cholesterol	mmol/L	[3.9 - 5.5]	09-May-2017	11:52	-15	-24	2.7	L	NCS
R001	Screening	Triglyceride	mmol/L	[0.6 - 2.0]	09-May-2017	11:52	-15	-24	0.3	L	NCS
R001	Pre-artesunate	Phosphate	mmol/L	[0.8 - 1.5]	02-Jun-2017	8:13	Day 9	1	1.54	H	NCS
R001	Admission 3	Urea	mmol/L	[3 - 7.5]	05-Jun-2017	8:23	Day 12	4	2.4	L	NCS
R001	Admission 3	AST	U/L	[10 - 40]	05-Jun-2017	8:23	Day 12	4	53	H	NCS
R001	Admission 3	ALT	U/L	[5 - 40]	05-Jun-2017	8:23	Day 12	4	62	H	NCS
R001	Safety Monitoring	AST	U/L	[10 - 40]	07-Jun-2017	7:54	Day 14	6	159	H	CS
R001	Safety Monitoring	ALT	U/L	[5 - 40]	07-Jun-2017	7:54	Day 14	6	227	H	CS
R001	Safety Monitoring	LDH	U/L	[120 - 250]	07-Jun-2017	7:54	Day 14	6	399	H	NCS
R001	Safety Monitoring	AST	U/L	[10 - 40]	09-Jun-2017	7:44	Day 16	8	120	H	CS
R001	Safety Monitoring	ALT	U/L	[5 - 40]	09-Jun-2017	7:44	Day 16	8	250	H	CS
R001	Safety Monitoring	AST	U/L	[10- 40]	11-Jun-2017	7:44	Day 18	10	65	H	NCS
R001	Safety Monitoring	ALT	U/L	[5 - 40]	11-Jun-2017	7:44	Day 18	10	166	H	CS
R001	Safety Monitoring	LDH	U/L	[120 - 250]	11-Jun-2017	7:44	Day 18	10	265	H	NCS
R001	Safety Monitoring	ALT	U/L	[5 - 40]	19-Jun-2017	7:57	Day 26	18	50	H	NCS
R001	Safety Monitoring	Total Protein	g/L	[66 - 83]	21-Jun-2017	8:38	Day 28	20	65	L	NCS
R001	Safety Monitoring	C Bilirubin	umol/L	[0 - 7]	21-Jun-2017	8:38	Day 28	20	8	H	NCS
R002	Safety Visit	Urea	mmol/L	[3 - 7.5]	13-Jun-2017	9:15	-1	-10	9.6	H	NCS
R002	Pre-artesunate	Haemolysis index		[0 - 40]	23-Jun-2017	9:09	Day 9	1	223	H	NCS
R002	Admission 3	Sodium	mmol/L	[135 - 145]	26-Jun-2017	9:23	Day 12	4	133	L	NCS
R002	Admission 3	LDH	U/L	[120 - 250]	26-Jun-2017	9:23	Day 12	4	398	H	NCS
R002	Admission 3	Haemolysis index		[0 - 40]	26-Jun-2017	9:23	Day 12	4	152	H	NCS
R002	Safety Monitoring	LDH	U/L	[120 - 250]	28-Jun-2017	9:11	Day 14	6	344	H	NCS
R002	Safety Monitoring	Fasting glucose	mmol/L	[3.6 - 6]	29-Jun-2017	8:13	Day 15	7	7.5	H	NCS
R002	Safety Monitoring	AST	U/L	[10 - 40]	29-Jun-2017	8:13	Day 15	7	41	H	NCS
R002	Safety Monitoring	ALT	U/L	[5 - 40]	29-Jun-2017	8:13	Day 15	7	49	H	NCS
R002	Safety Monitoring	LDH	U/L	[120 - 250]	29-Jun-2017	8:13	Day 15	7	346	H	NCS
R002	Safety Monitoring	AST	U/L	[10 - 40]	02-Jul-2017	8:46	Day 18	10	44	H	NCS

R002	Safety Monitoring	ALT	U/L	[5 - 40]	02-Jul-2017	8:46	Day 18	10	75	H	NCS
R002	Safety Monitoring	LDH	U/L	[120 - 250]	02-Jul-2017	8:46	Day 18	10	286	H	NCS
R002	Safety Monitoring	Alkaline Phosphatase	U/L	[35 - 110]	04-Jul-2017	8:15	Day 20	12	172	H	NCS
R002	Safety Monitoring	ALT	U/L	[5 - 40]	04-Jul-2017	8:15	Day 20	12	69	H	NCS
R002	Safety Monitoring	LDH	U/L	[120 -250]	04-Jul-2017	8:15	Day 20	12	264	H	NCS
R002	Safety Monitoring	ALT	U/L	[5 - 40]	06-Jul-2017	8:16	Day 22	14	70	H	NCS
R002	Safety Monitoring	ALT	U/L	[5 - 40]	10-Jul-2017	8:52	Day 26	18	44	H	NCS

Notes:
Study Day is the number of days relative to day of administration of the inoculum, where Study Day = 0 for inoculum dosing day.
Day relative to Artesunate is the number of days relative to day of administration of Artesunate where the day of first dose = 1.
H = High; L = Low.
CS = Clinically Significant; NCS = Not Clinically Significant. ALT = Alanine transaminase; AST = Aspartate aminotransferase; LDH = Lactate dehydrogenase
Clinical laboratory evaluation for Biochemistry were performed for all participants as per the protocol. Only abnormal results are recorded in this listing.

16.2.8.3 Individual abnormal urinalysis results (Dipstick)

Listing 16.2.8.3
Individual Abnormal Urinalysis Results (Dipstick)

Subject Number	Visit	Test	Unit	Normal Range	Date of Measurement	Time of Measurement	Study Day	Day Relative to Artesunate	Result	Range Indicator	Clinical Assessment
R002	Safety monitoring	Ketone	mmol/L		28-Jun-2017	8:10	Day 14	6	Trace		NCS

Notes:
Study Day is the number of days relative to day of administration of the inoculum, where Study Day = 0 for inoculum dosing day.
Day relative to Artesunate is the number of days relative to day of administration of Artesunate where the day of first dose = 1.
H = High; L = Low; CS = Clinically Significant; NCS = Not Clinically Significant.
Clinical laboratory evaluation for Urinalysis (Dipstick) were performed for all participants as per the protocol. Only abnormal results are recorded in this listing.

16.2.8.4 Individual abnormal microscopy urinalysis results (laboratory)

Listing 16.2.8.4
Individual Abnormal Microscopy and Urinalysis Results (Laboratory)

Subject Number	Visit	Test	Unit	Normal Range	Date of Measurement	Time of Measurement	Study Day	Day Relative to Artesunate	Result	Range Indicator	Clinical Assessment
No abnormal results to report											

Notes:
Study Day is the number of days relative to day of administration of the inoculum, where Study Day = 0 for inoculum dosing day.
Day relative to Artesunate is the number of days relative to day of administration of Artesunate where the day of first dose = 1.
NPI = No Pathogens Isolated; NG = No Growth.
CS = Clinically Significant; NCS = Not Clinically Significant.
Clinical laboratory evaluation for Microscopy and Urinalysis (Laboratory) were performed for all participants as per the protocol. Only abnormal results are recorded in this listing.

16.2.9 Other safety data

16.2.9.1 Abnormal vital signs (Blood pressure and heart rate)

Listing 16.2.9.1
Abnormal Vital Signs (Blood Pressure and Heart Rate)

Subject Number	Parameter (e.g. SBP, DBP or HR)	Unit	Visit	Position	Timepoint	Study Day	Day Relative to Artesunate	Date of Measurement	Time of Measurement	Normal Range	Result	Clinical Assessment
R001	HR	bpm	Screening	Supine	AM	-15	-24	09-May-2017	11:44	50-100	43	NCS
R001	HR	bpm	Unscheduled vital signs	Supine	AM	-15	-24	09-May-2017	11:46	50-100	45	NCS
R001	HR	bpm	Unscheduled vital signs	NR	AM	Day 3	-6	27-May-2017	7:55	50-100	47	NCS
R001	HR	bpm	Unscheduled vital signs	NR	AM	Day 3	-6	27-May-2017	7:56	50-100	49	NCS

Notes:
Study Day is the number of days relative to day of administration of the inoculum, where Study Day = 0 for inoculum dosing day.
Day relative to Artesunate is the number of days relative to day of administration of Artesunate where the day of first dose = 1.
NR = Not Recorded; ND = Not Done
SBP = Systolic Blood Pressure; DBP = Diastolic Blood Pressure; HR = Heart Rate; bpm = Beats per minute
CS = Clinically Significant; NCS = Not Clinically Significant.
Clinical laboratory evaluation for Vital Signs (Blood Pressure and Heart Rate) were performed for all participants as per the protocol. Only abnormal results are recorded in this listing.

16.2.9.2 Abnormal vital signs (Body temperature and respiratory rate)

Listing 16.2.9.2
Abnormal Vital Signs (Temperature and Respiratory Rate)

Subject Number	Parameter (e.g. TEMP or RR)	Unit	Visit	Position	Timepoint	Study Day	Day Relative to Artesunate	Date of Measurement	Time of Measurement	Normal Range	Result	Clinical Assessment
R001	TEMP	°C	Safety monitoring	Sitting	AM	Day 16	8	09-Jun-2017	7:40	35.0-37.5	34.8	NCS
R001	TEMP	°C	Safety monitoring	Sitting	NR	Day 16	8	09-Jun-2017	NR	35.0-37.5	34.8	NCS
R002	TEMP	°C	Unscheduled vital signs	Semi supine	PM	Day 10	2	24-Jun-2017	13:15	35.0-37.5	38.5 (tympanic)	CS
R002	TEMP	°C	Unscheduled vital signs	Semi supine	PM	Day 10	2	24-Jun-2017	13:47	35.0-37.5	38.4 (tympanic)	CS
R002	TEMP	°C	Unscheduled vital signs	Semi supine	PM	Day 10	2	24-Jun-2017	14:38	35.0-37.5	37.9 (oral)	NCS
R002	TEMP	°C	Unscheduled vital signs	Semi supine	PM	Day 10	2	24-Jun-2017	14:39	35.0-37.5	38.1 (tympanic)	CS
R002	TEMP	°C	Unscheduled vital signs	Semi supine	PM	Day 10	2	24-Jun-2017	23:13	35.0-37.5	38.2 (tympanic)	CS
R002	TEMP	°C	Unscheduled vital signs	Sitting	PM	Day 11	3	25-Jun-2017	15:17	35.0-37.5	37.9 (tympanic)	NCS
R002	TEMP	°C	Admission 2	Sitting	PM	Day 11	3	25-Jun-2017	19:01	35.0-37.5	37.8 (oral)	NCS
R002	TEMP	°C	Admission 2	Sitting	PM	Day 11	3	25-Jun-2017	19:01	35.0-37.5	38.5 (tympanic)	CS
R002	TEMP	°C	Unscheduled vital signs	Sitting	PM	Day 11	3	25-Jun-2017	19:02	35.0-37.5	37.9	NCS
R002	TEMP	°C	Admission 3	Sitting	AM	Day 12	4	26-Jun-2017	9:31	35.0-37.5	37.9	NCS
R002	TEMP	°C	Unscheduled vital signs	Sitting	AM	Day 12	4	26-Jun-2017	9:32	35.0-37.5	37.9	NCS
R002	TEMP	°C	Safety monitoring	Sitting	AM	Day 18	10	02-Jul-2017	8:33	35.0-37.5	34.4	NCS
R002	TEMP	°C	Safety monitoring	Sitting	AM	Day 37	29	21-Jul-2017	8:58	35.0-37.5	34.9	NCS

Notes:
Study Day is the number of days relative to day of administration of the inoculum, where Study Day = 0 for inoculum dosing day.
Day relative to Artesunate is the number of days relative to day of administration of Artesunate where the day of first dose = 1.
NR = Not Recorded
RR = Respiratory Rate; TEMP = Temperature.
CS = Clinically Significant; NCS = Not Clinically Significant.
Clinical laboratory evaluation for Vital Signs (Temperature and Respiratory Rate) were performed for all participants as per the protocol. Only abnormal results are recorded in this listing.

16.2.9.3 Abnormal ECG interpretation

Listing 16.2.9.3
Abnormal ECG Interpretation

Subject Number	Visit	Timepoint	Rep No.	Date of ECG	Time of ECG	Study Day	Day Relative to Artesunate	Clinical Assessment	Description of Abnormality
R001	Screening		1	09-May-2017	11:37	-15	-24	NCS	Sinus Bradycardia
R001	Pre-inoculum		1	24-May-2017	8:51	Day 0	-9	NCS	Sinus Arrhythmia
R001	Pre-artesunate		1	02-Jun-2017	7:33	Day 9	1	NCS	Sinus Bradycardia
R001	Unscheduled ECG		1	04-Jun-2017	15:51	Day 11	3	NCS	Sinus Bradycardia
R002	Screening		1	07-Jun-2017	9:46	-7	-16	NCS	Sinus Arrhythmia

Notes:
Study Day is the number of days relative to day of administration of the inoculum, where Study Day = 0 for inoculum dosing day.
Day relative to Artesunate is the number of days relative to day of administration of Artesunate where the day of first dose = 1.
Rep No. = Replicate Number
CS = Clinically Significant; NCS = Not Clinically Significant.
Clinical laboratory evaluation for ECG interpretation were performed for all participants as per the protocol. Only abnormal results are recorded in this listing.

16.2.9.4 Abnormal physical examination

Listing 16.2.9.4
Abnormal Physical Examination

Subject Number	Visit	Date of Exam	Timepoint	Study Day	Day Relative to Artesunate	Anatomical Site	Description of Abnormality	Clinical Assessment
R001	Admission	24-May-2017	AM	Day 0	-9	Other: extremities	Rope burn from climbing left forearm healing no sign of infection	NCS
R001	Malaria monitoring	29-May-2017	AM	Day 5	-4	Other: extremities	Rope burn on left forearm healing well	NCS

Notes:
Study Day is the number of days relative to day of administration of the inoculum, where Study Day = 0 for inoculum dosing day.
Day relative to Artesunate is the number of days relative to day of administration of Artesunate where the day of first dose = 1.
AE = Adverse Event
CS = Clinically Significant; NCS = Not Clinically Significant.
Clinical laboratory evaluation for Physical Examination were performed for all participants as per the protocol. Only abnormal results are recorded in this listing.

16.2.9.5 Clinical score

Listing 16.2.9.5
Clinical Score

Subject Number	Study Day	Day Relative to Artesunate	Timepoint	Questionnaire	Date of Measurement	Time of Measurement	Result
R001	3	-6	Day 3 AM	Overall score	27-May-2017	7:57	0
				Headache	27-May-2017	7:57	0
				Myalgia	27-May-2017	7:57	0
				Arthralgia	27-May-2017	7:57	0
				Fatigue/Lethargy	27-May-2017	7:57	0
				Malaise	27-May-2017	7:57	0
				Chills/Shiving/Rigors	27-May-2017	7:57	0
				Sweating/Hot spells	27-May-2017	7:57	0
				Anorexia	27-May-2017	7:57	0
				Nausea	27-May-2017	7:57	0
				Vomiting	27-May-2017	7:57	0
				Abdominal discomfort	27-May-2017	7:57	0
				Fever	27-May-2017	7:57	0
				Tachycardia	27-May-2017	7:57	0
				Hypotension	27-May-2017	7:57	0
R001	4	-5	Day 4 AM	Overall score	28-May-2017	8:23	0
				Headache	28-May-2017	8:23	0
				Myalgia	28-May-2017	8:23	0
				Arthralgia	28-May-2017	8:23	0
				Fatigue/Lethargy	28-May-2017	8:23	0
				Malaise	28-May-2017	8:23	0
				Chills/Shiving/Rigors	28-May-2017	8:23	0
				Sweating/Hot spells	28-May-2017	8:23	0
				Anorexia	28-May-2017	8:23	0
				Nausea	28-May-2017	8:23	0
				Vomiting	28-May-2017	8:23	0
				Abdominal discomfort	28-May-2017	8:23	0
				Fever	28-May-2017	8:23	0
				Tachycardia	28-May-2017	8:23	0

				Hypotension	28-May-2017	8:23	0
R001	5	-4	Day 5 AM	Overall score	29-May-2017	7:50	0
				Headache	29-May-2017	7:50	0
				Myalgia	29-May-2017	7:50	0
				Arthralgia	29-May-2017	7:50	0
				Fatigue/Lethargy	29-May-2017	7:50	0
				Malaise	29-May-2017	7:50	0
				Chills/Shiving/Rigors	29-May-2017	7:50	0
				Sweating/Hot spells	29-May-2017	7:50	0
				Anorexia	29-May-2017	7:50	0
				Nausea	29-May-2017	7:50	0
				Vomiting	29-May-2017	7:50	0
				Abdominal discomfort	29-May-2017	7:50	0
				Fever	29-May-2017	7:50	0
				Tachycardia	29-May-2017	7:50	0
				Hypotension	29-May-2017	7:50	0
R001	5	-4	Day 5 PM	Overall score	29-May-2017	18:43	0
				Headache	29-May-2017	18:43	0
				Myalgia	29-May-2017	18:43	0
				Arthralgia	29-May-2017	18:43	0
				Fatigue/Lethargy	29-May-2017	18:43	0
				Malaise	29-May-2017	18:43	0
				Chills/Shiving/Rigors	29-May-2017	18:43	0
				Sweating/Hot spells	29-May-2017	18:43	0
				Anorexia	29-May-2017	18:43	0
				Nausea	29-May-2017	18:43	0
				Vomiting	29-May-2017	18:43	0
				Abdominal discomfort	29-May-2017	18:43	0
				Fever	29-May-2017	18:43	0
				Tachycardia	29-May-2017	18:43	0
				Hypotension	29-May-2017	18:43	0
R001	6	-3	Day 6 AM	Overall score	30-May-2017	7:38	0
				Headache	30-May-2017	7:38	0

				Myalgia	30-May-2017	7:38	0
				Arthralgia	30-May-2017	7:38	0
				Fatigue/Lethargy	30-May-2017	7:38	0
				Malaise	30-May-2017	7:38	0
				Chills/Shiving/Rigors	30-May-2017	7:38	0
				Sweating/Hot spells	30-May-2017	7:38	0
				Anorexia	30-May-2017	7:38	0
				Nausea	30-May-2017	7:38	0
				Vomiting	30-May-2017	7:38	0
				Abdominal discomfort	30-May-2017	7:38	0
				Fever	30-May-2017	7:38	0
				Tachycardia	30-May-2017	7:38	0
				Hypotension	30-May-2017	7:38	0
R001	6	-3	Day 6 PM	Overall score	30-May-2017	18:38	0
				Headache	30-May-2017	18:38	0
				Myalgia	30-May-2017	18:38	0
				Arthralgia	30-May-2017	18:38	0
				Fatigue/Lethargy	30-May-2017	18:38	0
				Malaise	30-May-2017	18:38	0
				Chills/Shiving/Rigors	30-May-2017	18:38	0
				Sweating/Hot spells	30-May-2017	18:38	0
				Anorexia	30-May-2017	18:38	0
				Nausea	30-May-2017	18:38	0
				Vomiting	30-May-2017	18:38	0
				Abdominal discomfort	30-May-2017	18:38	0
				Fever	30-May-2017	18:38	0
				Tachycardia	30-May-2017	18:38	0
				Hypotension	30-May-2017	18:38	0
R001	7	-2	Day 7 AM	Overall score	31-May-2017	7:40	0
				Headache	31-May-2017	7:40	0
				Myalgia	31-May-2017	7:40	0
				Arthralgia	31-May-2017	7:40	0
				Fatigue/Lethargy	31-May-2017	7:40	0

				Malaise	31-May-2017	7:40	0
				Chills/Shiving/Rigors	31-May-2017	7:40	0
				Sweating/Hot spells	31-May-2017	7:40	0
				Anorexia	31-May-2017	7:40	0
				Nausea	31-May-2017	7:40	0
				Vomiting	31-May-2017	7:40	0
				Abdominal discomfort	31-May-2017	7:40	0
				Fever	31-May-2017	7:40	0
				Tachycardia	31-May-2017	7:40	0
				Hypotension	31-May-2017	7:40	0
R001	7	-2	Day 7 PM	Overall score	31-May-2017	18:47	0
				Headache	31-May-2017	18:47	0
				Myalgia	31-May-2017	18:47	0
				Arthralgia	31-May-2017	18:47	0
				Fatigue/Lethargy	31-May-2017	18:47	0
				Malaise	31-May-2017	18:47	0
				Chills/Shiving/Rigors	31-May-2017	18:47	0
				Sweating/Hot spells	31-May-2017	18:47	0
				Anorexia	31-May-2017	18:47	0
				Nausea	31-May-2017	18:47	0
				Vomiting	31-May-2017	18:47	0
				Abdominal discomfort	31-May-2017	18:47	0
				Fever	31-May-2017	18:47	0
				Tachycardia	31-May-2017	18:47	0
				Hypotension	31-May-2017	18:47	0
R001	8	-1	Day 8 AM	Overall score	01-Jun-2017	8:05	0
				Headache	01-Jun-2017	8:05	0
				Myalgia	01-Jun-2017	8:05	0
				Arthralgia	01-Jun-2017	8:05	0
				Fatigue/Lethargy	01-Jun-2017	8:05	0
				Malaise	01-Jun-2017	8:05	0
				Chills/Shiving/Rigors	01-Jun-2017	8:05	0
				Sweating/Hot spells	01-Jun-2017	8:05	0

				Anorexia	01-Jun-2017	8:05	0
				Nausea	01-Jun-2017	8:05	0
				Vomiting	01-Jun-2017	8:05	0
				Abdominal discomfort	01-Jun-2017	8:05	0
				Fever	01-Jun-2017	8:05	0
				Tachycardia	01-Jun-2017	8:05	0
				Hypotension	01-Jun-2017	8:05	0
R001	8	-1	Day 8 PM	Overall score	01-Jun-2017	19:30	0
				Headache	01-Jun-2017	19:30	0
				Myalgia	01-Jun-2017	19:30	0
				Arthralgia	01-Jun-2017	19:30	0
				Fatigue/Lethargy	01-Jun-2017	19:30	0
				Malaise	01-Jun-2017	19:30	0
				Chills/Shiving/Rigors	01-Jun-2017	19:30	0
				Sweating/Hot spells	01-Jun-2017	19:30	0
				Anorexia	01-Jun-2017	19:30	0
				Nausea	01-Jun-2017	19:30	0
				Vomiting	01-Jun-2017	19:30	0
				Abdominal discomfort	01-Jun-2017	19:30	0
				Fever	01-Jun-2017	19:30	0
				Tachycardia	01-Jun-2017	19:30	0
				Hypotension	01-Jun-2017	19:30	0
R001	9	1	Day 9 AM	Overall score	02-Jun-2017	7:35	0
				Headache	02-Jun-2017	7:35	0
				Myalgia	02-Jun-2017	7:35	0
				Arthralgia	02-Jun-2017	7:35	0
				Fatigue/Lethargy	02-Jun-2017	7:35	0
				Malaise	02-Jun-2017	7:35	0
				Chills/Shiving/Rigors	02-Jun-2017	7:35	0
				Sweating/Hot spells	02-Jun-2017	7:35	0
				Anorexia	02-Jun-2017	7:35	0
				Nausea	02-Jun-2017	7:35	0
				Vomiting	02-Jun-2017	7:35	0

				Abdominal discomfort	02-Jun-2017	7:35	0
				Fever	02-Jun-2017	7:35	0
				Tachycardia	02-Jun-2017	7:35	0
				Hypotension	02-Jun-2017	7:35	0
R001	9	1	Day 9 Mid	Overall score	02-Jun-2017	12:34	0
				Headache	02-Jun-2017	12:34	0
				Myalgia	02-Jun-2017	12:34	0
				Arthralgia	02-Jun-2017	12:34	0
				Fatigue/Lethargy	02-Jun-2017	12:34	0
				Malaise	02-Jun-2017	12:34	0
				Chills/Shiving/Rigors	02-Jun-2017	12:34	0
				Sweating/Hot spells	02-Jun-2017	12:34	0
				Anorexia	02-Jun-2017	12:34	0
				Nausea	02-Jun-2017	12:34	0
				Vomiting	02-Jun-2017	12:34	0
				Abdominal discomfort	02-Jun-2017	12:34	0
				Fever	02-Jun-2017	12:34	0
				Tachycardia	02-Jun-2017	12:34	0
				Hypotension	02-Jun-2017	12:34	0
R001	9	1	Day 9 PM	Overall score	02-Jun-2017	20:04	1
				Headache	02-Jun-2017	20:04	0
				Myalgia	02-Jun-2017	20:04	0
				Arthralgia	02-Jun-2017	20:04	0
				Fatigue/Lethargy	02-Jun-2017	20:04	1
				Malaise	02-Jun-2017	20:04	0
				Chills/Shiving/Rigors	02-Jun-2017	20:04	0
				Sweating/Hot spells	02-Jun-2017	20:04	0
				Anorexia	02-Jun-2017	20:04	0
				Nausea	02-Jun-2017	20:04	0
				Vomiting	02-Jun-2017	20:04	0
				Abdominal discomfort	02-Jun-2017	20:04	0
				Fever	02-Jun-2017	20:04	0
				Tachycardia	02-Jun-2017	20:04	0

				Hypotension	02-Jun-2017	20:04	0
R001	10	2	Day 10 AM	Overall score	03-Jun-2017	8:28	0
				Headache	03-Jun-2017	8:28	0
				Myalgia	03-Jun-2017	8:28	0
				Arthralgia	03-Jun-2017	8:28	0
				Fatigue/Lethargy	03-Jun-2017	8:28	0
				Malaise	03-Jun-2017	8:28	0
				Chills/Shiving/Rigors	03-Jun-2017	8:28	0
				Sweating/Hot spells	03-Jun-2017	8:28	0
				Anorexia	03-Jun-2017	8:28	0
				Nausea	03-Jun-2017	8:28	0
				Vomiting	03-Jun-2017	8:28	0
				Abdominal discomfort	03-Jun-2017	8:28	0
				Fever	03-Jun-2017	8:28	0
				Tachycardia	03-Jun-2017	8:28	0
				Hypotension	03-Jun-2017	8:28	0
R001	10	2	Day 10 Mid	Overall score	03-Jun-2017	12:31	0
				Headache	03-Jun-2017	12:31	0
				Myalgia	03-Jun-2017	12:31	0
				Arthralgia	03-Jun-2017	12:31	0
				Fatigue/Lethargy	03-Jun-2017	12:31	0
				Malaise	03-Jun-2017	12:31	0
				Chills/Shiving/Rigors	03-Jun-2017	12:31	0
				Sweating/Hot spells	03-Jun-2017	12:31	0
				Anorexia	03-Jun-2017	12:31	0
				Nausea	03-Jun-2017	12:31	0
				Vomiting	03-Jun-2017	12:31	0
				Abdominal discomfort	03-Jun-2017	12:31	0
				Fever	03-Jun-2017	12:31	0
				Tachycardia	03-Jun-2017	12:31	0
				Hypotension	03-Jun-2017	12:31	0
R001	10	2	Day 10 PM	Overall score	03-Jun-2017	20:23	1
				Headache	03-Jun-2017	20:23	1

				Myalgia	03-Jun-2017	20:23	0
				Arthralgia	03-Jun-2017	20:23	0
				Fatigue/Lethargy	03-Jun-2017	20:23	0
				Malaise	03-Jun-2017	20:23	0
				Chills/Shiving/Rigors	03-Jun-2017	20:23	0
				Sweating/Hot spells	03-Jun-2017	20:23	0
				Anorexia	03-Jun-2017	20:23	0
				Nausea	03-Jun-2017	20:23	0
				Vomiting	03-Jun-2017	20:23	0
				Abdominal discomfort	03-Jun-2017	20:23	0
				Fever	03-Jun-2017	20:23	0
				Tachycardia	03-Jun-2017	20:23	0
				Hypotension	03-Jun-2017	20:23	0
R001	11	3	Day 11 AM	Overall score	04-Jun-2017	8:40	0
				Headache	04-Jun-2017	8:40	0
				Myalgia	04-Jun-2017	8:40	0
				Arthralgia	04-Jun-2017	8:40	0
				Fatigue/Lethargy	04-Jun-2017	8:40	0
				Malaise	04-Jun-2017	8:40	0
				Chills/Shiving/Rigors	04-Jun-2017	8:40	0
				Sweating/Hot spells	04-Jun-2017	8:40	0
				Anorexia	04-Jun-2017	8:40	0
				Nausea	04-Jun-2017	8:40	0
				Vomiting	04-Jun-2017	8:40	0
				Abdominal discomfort	04-Jun-2017	8:40	0
				Fever	04-Jun-2017	8:40	0
				Tachycardia	04-Jun-2017	8:40	0
				Hypotension	04-Jun-2017	8:40	0
R001	11	3	Day 11 Mid	Overall score	04-Jun-2017	12:23	0
				Headache	04-Jun-2017	12:23	0
				Myalgia	04-Jun-2017	12:23	0
				Arthralgia	04-Jun-2017	12:23	0
				Fatigue/Lethargy	04-Jun-2017	12:23	0

				Malaise	04-Jun-2017	12:23	0
				Chills/Shiving/Rigors	04-Jun-2017	12:23	0
				Sweating/Hot spells	04-Jun-2017	12:23	0
				Anorexia	04-Jun-2017	12:23	0
				Nausea	04-Jun-2017	12:23	0
				Vomiting	04-Jun-2017	12:23	0
				Abdominal discomfort	04-Jun-2017	12:23	0
				Fever	04-Jun-2017	12:23	0
				Tachycardia	04-Jun-2017	12:23	0
				Hypotension	04-Jun-2017	12:23	0
R001	11	3	Day 11 PM	Overall score	04-Jun-2017	20:25	0
				Headache	04-Jun-2017	20:25	0
				Myalgia	04-Jun-2017	20:25	0
				Arthralgia	04-Jun-2017	20:25	0
				Fatigue/Lethargy	04-Jun-2017	20:25	0
				Malaise	04-Jun-2017	20:25	0
				Chills/Shiving/Rigors	04-Jun-2017	20:25	0
				Sweating/Hot spells	04-Jun-2017	20:25	0
				Anorexia	04-Jun-2017	20:25	0
				Nausea	04-Jun-2017	20:25	0
				Vomiting	04-Jun-2017	20:25	0
				Abdominal discomfort	04-Jun-2017	20:25	0
				Fever	04-Jun-2017	20:25	0
				Tachycardia	04-Jun-2017	20:25	0
				Hypotension	04-Jun-2017	20:25	0
R001	12	4	Day 12 AM	Overall score	05-Jun-2017	8:15	0
				Headache	05-Jun-2017	8:15	0
				Myalgia	05-Jun-2017	8:15	0
				Arthralgia	05-Jun-2017	8:15	0
				Fatigue/Lethargy	05-Jun-2017	8:15	0
				Malaise	05-Jun-2017	8:15	0
				Chills/Shiving/Rigors	05-Jun-2017	8:15	0
				Sweating/Hot spells	05-Jun-2017	8:15	0

				Anorexia	05-Jun-2017	8:15	0
				Nausea	05-Jun-2017	8:15	0
				Vomiting	05-Jun-2017	8:15	0
				Abdominal discomfort	05-Jun-2017	8:15	0
				Fever	05-Jun-2017	8:15	0
				Tachycardia	05-Jun-2017	8:15	0
				Hypotension	05-Jun-2017	8:15	0
R001	12	4	Day 12 PM	Overall score	05-Jun-2017	19:01	6
				Headache	05-Jun-2017	19:01	1
				Myalgia	05-Jun-2017	19:01	1
				Arthralgia	05-Jun-2017	19:01	1
				Fatigue/Lethargy	05-Jun-2017	19:01	1
				Malaise	05-Jun-2017	19:01	1
				Chills/Shiving/Rigors	05-Jun-2017	19:01	1
				Sweating/Hot spells	05-Jun-2017	19:01	0
				Anorexia	05-Jun-2017	19:01	0
				Nausea	05-Jun-2017	19:01	0
				Vomiting	05-Jun-2017	19:01	0
				Abdominal discomfort	05-Jun-2017	19:01	0
				Fever	05-Jun-2017	19:01	0
				Tachycardia	05-Jun-2017	19:01	0
				Hypotension	05-Jun-2017	19:01	0
R001	13	5	Day 13 AM	Overall score	06-Jun-2017	7:51	5
				Headache	06-Jun-2017	7:51	1
				Myalgia	06-Jun-2017	7:51	1
				Arthralgia	06-Jun-2017	7:51	1
				Fatigue/Lethargy	06-Jun-2017	7:51	1
				Malaise	06-Jun-2017	7:51	1
				Chills/Shiving/Rigors	06-Jun-2017	7:51	0
				Sweating/Hot spells	06-Jun-2017	7:51	0
				Anorexia	06-Jun-2017	7:51	0
				Nausea	06-Jun-2017	7:51	0
				Vomiting	06-Jun-2017	7:51	0

				Abdominal discomfort	06-Jun-2017	7:51	0
				Fever	06-Jun-2017	7:51	0
				Tachycardia	06-Jun-2017	7:51	0
				Hypotension	06-Jun-2017	7:51	0
R001	14	6	Day 14 AM	Overall score	07-Jun-2017	7:46	0
				Headache	07-Jun-2017	7:46	0
				Myalgia	07-Jun-2017	7:46	0
				Arthralgia	07-Jun-2017	7:46	0
				Fatigue/Lethargy	07-Jun-2017	7:46	0
				Malaise	07-Jun-2017	7:46	0
				Chills/Shiving/Rigors	07-Jun-2017	7:46	0
				Sweating/Hot spells	07-Jun-2017	7:46	0
				Anorexia	07-Jun-2017	7:46	0
				Nausea	07-Jun-2017	7:46	0
				Vomiting	07-Jun-2017	7:46	0
				Abdominal discomfort	07-Jun-2017	7:46	0
				Fever	07-Jun-2017	7:46	0
				Tachycardia	07-Jun-2017	7:46	0
				Hypotension	07-Jun-2017	7:46	0
R001	16	8	Day 16 AM	Overall score	09-Jun-2017	7:47	0
				Headache	09-Jun-2017	7:47	0
				Myalgia	09-Jun-2017	7:47	0
				Arthralgia	09-Jun-2017	7:47	0
				Fatigue/Lethargy	09-Jun-2017	7:47	0
				Malaise	09-Jun-2017	7:47	0
				Chills/Shiving/Rigors	09-Jun-2017	7:47	0
				Sweating/Hot spells	09-Jun-2017	7:47	0
				Anorexia	09-Jun-2017	7:47	0
				Nausea	09-Jun-2017	7:47	0
				Vomiting	09-Jun-2017	7:47	0
				Abdominal discomfort	09-Jun-2017	7:47	0
				Fever	09-Jun-2017	7:47	0
				Tachycardia	09-Jun-2017	7:47	0

				Hypotension	09-Jun-2017	7:47	0
R001	18	10	Day 18 AM	Overall score	11-Jun-2017	7:37	0
				Headache	11-Jun-2017	7:37	0
				Myalgia	11-Jun-2017	7:37	0
				Arthralgia	11-Jun-2017	7:37	0
				Fatigue/Lethargy	11-Jun-2017	7:37	0
				Malaise	11-Jun-2017	7:37	0
				Chills/Shiving/Rigors	11-Jun-2017	7:37	0
				Sweating/Hot spells	11-Jun-2017	7:37	0
				Anorexia	11-Jun-2017	7:37	0
				Nausea	11-Jun-2017	7:37	0
				Vomiting	11-Jun-2017	7:37	0
				Abdominal discomfort	11-Jun-2017	7:37	0
				Fever	11-Jun-2017	7:37	0
				Tachycardia	11-Jun-2017	7:37	0
				Hypotension	11-Jun-2017	7:37	0
R001	19	11	Day 19 AM	Overall score	12-Jun-2017	7:40	0
				Headache	12-Jun-2017	7:40	0
				Myalgia	12-Jun-2017	7:40	0
				Arthralgia	12-Jun-2017	7:40	0
				Fatigue/Lethargy	12-Jun-2017	7:40	0
				Malaise	12-Jun-2017	7:40	0
				Chills/Shiving/Rigors	12-Jun-2017	7:40	0
				Sweating/Hot spells	12-Jun-2017	7:40	0
				Anorexia	12-Jun-2017	7:40	0
				Nausea	12-Jun-2017	7:40	0
				Vomiting	12-Jun-2017	7:40	0
				Abdominal discomfort	12-Jun-2017	7:40	0
				Fever	12-Jun-2017	7:40	0
				Tachycardia	12-Jun-2017	7:40	0
				Hypotension	12-Jun-2017	7:40	0
R001	21	13	Day 21 AM	Overall score	14-Jun-2017	7:51	0
				Headache	14-Jun-2017	7:51	0

				Myalgia	14-Jun-2017	7:51	0
				Arthralgia	14-Jun-2017	7:51	0
				Fatigue/Lethargy	14-Jun-2017	7:51	0
				Malaise	14-Jun-2017	7:51	0
				Chills/Shiving/Rigors	14-Jun-2017	7:51	0
				Sweating/Hot spells	14-Jun-2017	7:51	0
				Anorexia	14-Jun-2017	7:51	0
				Nausea	14-Jun-2017	7:51	0
				Vomiting	14-Jun-2017	7:51	0
				Abdominal discomfort	14-Jun-2017	7:51	0
				Fever	14-Jun-2017	7:51	0
				Tachycardia	14-Jun-2017	7:51	0
				Hypotension	14-Jun-2017	7:51	0
R001	23	15	Day 23 AM	Overall score	16-Jun-2017	7:39	0
				Headache	16-Jun-2017	7:39	0
				Myalgia	16-Jun-2017	7:39	0
				Arthralgia	16-Jun-2017	7:39	0
				Fatigue/Lethargy	16-Jun-2017	7:39	0
				Malaise	16-Jun-2017	7:39	0
				Chills/Shiving/Rigors	16-Jun-2017	7:39	0
				Sweating/Hot spells	16-Jun-2017	7:39	0
				Anorexia	16-Jun-2017	7:39	0
				Nausea	16-Jun-2017	7:39	0
				Vomiting	16-Jun-2017	7:39	0
				Abdominal discomfort	16-Jun-2017	7:39	0
				Fever	16-Jun-2017	7:39	0
				Tachycardia	16-Jun-2017	7:39	0
				Hypotension	16-Jun-2017	7:39	0
R001	26	18	Day 26 AM	Overall score	19-Jun-2017	7:46	0
				Headache	19-Jun-2017	7:46	0
				Myalgia	19-Jun-2017	7:46	0
				Arthralgia	19-Jun-2017	7:46	0
				Fatigue/Lethargy	19-Jun-2017	7:46	0

				Malaise	19-Jun-2017	7:46	0
				Chills/Shiving/Rigors	19-Jun-2017	7:46	0
				Sweating/Hot spells	19-Jun-2017	7:46	0
				Anorexia	19-Jun-2017	7:46	0
				Nausea	19-Jun-2017	7:46	0
				Vomiting	19-Jun-2017	7:46	0
				Abdominal discomfort	19-Jun-2017	7:46	0
				Fever	19-Jun-2017	7:46	0
				Tachycardia	19-Jun-2017	7:46	0
				Hypotension	19-Jun-2017	7:46	0
R001	28	20	Day 28 AM	Overall score	21-Jun-2017	8:32	0
				Headache	21-Jun-2017	8:32	0
				Myalgia	21-Jun-2017	8:32	0
				Arthralgia	21-Jun-2017	8:32	0
				Fatigue/Lethargy	21-Jun-2017	8:32	0
				Malaise	21-Jun-2017	8:32	0
				Chills/Shiving/Rigors	21-Jun-2017	8:32	0
				Sweating/Hot spells	21-Jun-2017	8:32	0
				Anorexia	21-Jun-2017	8:32	0
				Nausea	21-Jun-2017	8:32	0
				Vomiting	21-Jun-2017	8:32	0
				Abdominal discomfort	21-Jun-2017	8:32	0
				Fever	21-Jun-2017	8:32	0
				Tachycardia	21-Jun-2017	8:32	0
				Hypotension	21-Jun-2017	8:32	0
R002	4	-5	Day 4 AM	Overall score	18-Jun-2017	7:14	0
				Headache	18-Jun-2017	7:14	0
				Myalgia	18-Jun-2017	7:14	0
				Arthralgia	18-Jun-2017	7:14	0
				Fatigue/Lethargy	18-Jun-2017	7:14	0
				Malaise	18-Jun-2017	7:14	0
				Chills/Shiving/Rigors	18-Jun-2017	7:14	0

				Sweating/Hot spells	18-Jun-2017	7:14	0
				Anorexia	18-Jun-2017	7:14	0
				Nausea	18-Jun-2017	7:14	0
				Vomiting	18-Jun-2017	7:14	0
				Abdominal discomfort	18-Jun-2017	7:14	0
				Fever	18-Jun-2017	7:14	0
				Tachycardia	18-Jun-2017	7:14	0
				Hypotension	18-Jun-2017	7:14	0
R002	5	-4	Day 5 AM	Overall score	19-Jun-2017	8:34	0
				Headache	19-Jun-2017	8:34	0
				Myalgia	19-Jun-2017	8:34	0
				Arthralgia	19-Jun-2017	8:34	0
				Fatigue/Lethargy	19-Jun-2017	8:34	0
				Malaise	19-Jun-2017	8:34	0
				Chills/Shiving/Rigors	19-Jun-2017	8:34	0
				Sweating/Hot spells	19-Jun-2017	8:34	0
				Anorexia	19-Jun-2017	8:34	0
				Nausea	19-Jun-2017	8:34	0
				Vomiting	19-Jun-2017	8:34	0
				Abdominal discomfort	19-Jun-2017	8:34	0
				Fever	19-Jun-2017	8:34	0
				Tachycardia	19-Jun-2017	8:34	0
				Hypotension	19-Jun-2017	8:34	0
R002	5	-4	Day 5 PM	Overall score	19-Jun-2017	19:30	0
				Headache	19-Jun-2017	19:30	0
				Myalgia	19-Jun-2017	19:30	0
				Arthralgia	19-Jun-2017	19:30	0
				Fatigue/Lethargy	19-Jun-2017	19:30	0
				Malaise	19-Jun-2017	19:30	0
				Chills/Shiving/Rigors	19-Jun-2017	19:30	0
				Sweating/Hot spells	19-Jun-2017	19:30	0
				Anorexia	19-Jun-2017	19:30	0
				Nausea	19-Jun-2017	19:30	0

				Vomiting	19-Jun-2017	19:30	0
				Abdominal discomfort	19-Jun-2017	19:30	0
				Fever	19-Jun-2017	19:30	0
				Tachycardia	19-Jun-2017	19:30	0
				Hypotension	19-Jun-2017	19:30	0
R002	6	-3	Day 6 AM	Overall score	20-Jun-2017	8:15	0
				Headache	20-Jun-2017	8:15	0
				Myalgia	20-Jun-2017	8:15	0
				Arthralgia	20-Jun-2017	8:15	0
				Fatigue/Lethargy	20-Jun-2017	8:15	0
				Malaise	20-Jun-2017	8:15	0
				Chills/Shiving/Rigors	20-Jun-2017	8:15	0
				Sweating/Hot spells	20-Jun-2017	8:15	0
				Anorexia	20-Jun-2017	8:15	0
				Nausea	20-Jun-2017	8:15	0
				Vomiting	20-Jun-2017	8:15	0
				Abdominal discomfort	20-Jun-2017	8:15	0
				Fever	20-Jun-2017	8:15	0
				Tachycardia	20-Jun-2017	8:15	0
				Hypotension	20-Jun-2017	8:15	0
R002	6	-3	Day 6 PM	Overall score	20-Jun-2017	19:09	0
				Headache	20-Jun-2017	19:09	0
				Myalgia	20-Jun-2017	19:09	0
				Arthralgia	20-Jun-2017	19:09	0
				Fatigue/Lethargy	20-Jun-2017	19:09	0
				Malaise	20-Jun-2017	19:09	0
				Chills/Shiving/Rigors	20-Jun-2017	19:09	0
				Sweating/Hot spells	20-Jun-2017	19:09	0
				Anorexia	20-Jun-2017	19:09	0
				Nausea	20-Jun-2017	19:09	0
				Vomiting	20-Jun-2017	19:09	0
				Abdominal discomfort	20-Jun-2017	19:09	0
				Fever	20-Jun-2017	19:09	0

				Tachycardia	20-Jun-2017	19:09	0
				Hypotension	20-Jun-2017	19:09	0
R002	7	-2	Day 7 AM	Overall score	21-Jun-2017	7:27	0
				Headache	21-Jun-2017	7:27	0
				Myalgia	21-Jun-2017	7:27	0
				Arthralgia	21-Jun-2017	7:27	0
				Fatigue/Lethargy	21-Jun-2017	7:27	0
				Malaise	21-Jun-2017	7:27	0
				Chills/Shiving/Rigors	21-Jun-2017	7:27	0
				Sweating/Hot spells	21-Jun-2017	7:27	0
				Anorexia	21-Jun-2017	7:27	0
				Nausea	21-Jun-2017	7:27	0
				Vomiting	21-Jun-2017	7:27	0
				Abdominal discomfort	21-Jun-2017	7:27	0
				Fever	21-Jun-2017	7:27	0
				Tachycardia	21-Jun-2017	7:27	0
				Hypotension	21-Jun-2017	7:27	0
R002	7	-2	Day 7 PM	Overall score	21-Jun-2017	22:39	0
				Headache	21-Jun-2017	22:39	0
				Myalgia	21-Jun-2017	22:39	0
				Arthralgia	21-Jun-2017	22:39	0
				Fatigue/Lethargy	21-Jun-2017	22:39	0
				Malaise	21-Jun-2017	22:39	0
				Chills/Shiving/Rigors	21-Jun-2017	22:39	0
				Sweating/Hot spells	21-Jun-2017	22:39	0
				Anorexia	21-Jun-2017	22:39	0
				Nausea	21-Jun-2017	22:39	0
				Vomiting	21-Jun-2017	22:39	0
				Abdominal discomfort	21-Jun-2017	22:39	0
				Fever	21-Jun-2017	22:39	0
				Tachycardia	21-Jun-2017	22:39	0
				Hypotension	21-Jun-2017	22:39	0
R002	8	-1	Day 8 AM	Overall score	22-Jun-2017	8:15	0

				Headache	22-Jun-2017	8:15	0
				Myalgia	22-Jun-2017	8:15	0
				Arthralgia	22-Jun-2017	8:15	0
				Fatigue/Lethargy	22-Jun-2017	8:15	0
				Malaise	22-Jun-2017	8:15	0
				Chills/Shiving/Rigors	22-Jun-2017	8:15	0
				Sweating/Hot spells	22-Jun-2017	8:15	0
				Anorexia	22-Jun-2017	8:15	0
				Nausea	22-Jun-2017	8:15	0
				Vomiting	22-Jun-2017	8:15	0
				Abdominal discomfort	22-Jun-2017	8:15	0
				Fever	22-Jun-2017	8:15	0
				Tachycardia	22-Jun-2017	8:15	0
				Hypotension	22-Jun-2017	8:15	0
R002	8	-1	Day 8 PM	Overall score	22-Jun-2017	19:00	0
				Headache	22-Jun-2017	19:00	0
				Myalgia	22-Jun-2017	19:00	0
				Arthralgia	22-Jun-2017	19:00	0
				Fatigue/Lethargy	22-Jun-2017	19:00	0
				Malaise	22-Jun-2017	19:00	0
				Chills/Shiving/Rigors	22-Jun-2017	19:00	0
				Sweating/Hot spells	22-Jun-2017	19:00	0
				Anorexia	22-Jun-2017	19:00	0
				Nausea	22-Jun-2017	19:00	0
				Vomiting	22-Jun-2017	19:00	0
				Abdominal discomfort	22-Jun-2017	19:00	0
				Fever	22-Jun-2017	19:00	0
				Tachycardia	22-Jun-2017	19:00	0
				Hypotension	22-Jun-2017	19:00	0
R002	9	1	Day 9 AM	Overall score	23-Jun-2017	8:18	0
				Headache	23-Jun-2017	8:18	0
				Myalgia	23-Jun-2017	8:18	0
				Arthralgia	23-Jun-2017	8:18	0

				Fatigue/Lethargy	23-Jun-2017	8:18	0
				Malaise	23-Jun-2017	8:18	0
				Chills/Shiving/Rigors	23-Jun-2017	8:18	0
				Sweating/Hot spells	23-Jun-2017	8:18	0
				Anorexia	23-Jun-2017	8:18	0
				Nausea	23-Jun-2017	8:18	0
				Vomiting	23-Jun-2017	8:18	0
				Abdominal discomfort	23-Jun-2017	8:18	0
				Fever	23-Jun-2017	8:18	0
				Tachycardia	23-Jun-2017	8:18	0
				Hypotension	23-Jun-2017	8:18	0
R002	9	1	Day 9 Mid	Overall score	23-Jun-2017	12:27	0
				Headache	23-Jun-2017	12:27	0
				Myalgia	23-Jun-2017	12:27	0
				Arthralgia	23-Jun-2017	12:27	0
				Fatigue/Lethargy	23-Jun-2017	12:27	0
				Malaise	23-Jun-2017	12:27	0
				Chills/Shiving/Rigors	23-Jun-2017	12:27	0
				Sweating/Hot spells	23-Jun-2017	12:27	0
				Anorexia	23-Jun-2017	12:27	0
				Nausea	23-Jun-2017	12:27	0
				Vomiting	23-Jun-2017	12:27	0
				Abdominal discomfort	23-Jun-2017	12:27	0
				Fever	23-Jun-2017	12:27	0
				Tachycardia	23-Jun-2017	12:27	0
				Hypotension	23-Jun-2017	12:27	0
R002	9	1	Day 9 PM	Overall score	23-Jun-2017	19:20	0
				Headache	23-Jun-2017	19:20	0
				Myalgia	23-Jun-2017	19:20	0
				Arthralgia	23-Jun-2017	19:20	0
				Fatigue/Lethargy	23-Jun-2017	19:20	0
				Malaise	23-Jun-2017	19:20	0
				Chills/Shiving/Rigors	23-Jun-2017	19:20	0

				Sweating/Hot spells	23-Jun-2017	19:20	0
				Anorexia	23-Jun-2017	19:20	0
				Nausea	23-Jun-2017	19:20	0
				Vomiting	23-Jun-2017	19:20	0
				Abdominal discomfort	23-Jun-2017	19:20	0
				Fever	23-Jun-2017	19:20	0
				Tachycardia	23-Jun-2017	19:20	0
				Hypotension	23-Jun-2017	19:20	0
R002	10	2	Day 10 AM	Overall score	24-Jun-2017	9:27	0
				Headache	24-Jun-2017	9:27	0
				Myalgia	24-Jun-2017	9:27	0
				Arthralgia	24-Jun-2017	9:27	0
				Fatigue/Lethargy	24-Jun-2017	9:27	0
				Malaise	24-Jun-2017	9:27	0
				Chills/Shiving/Rigors	24-Jun-2017	9:27	0
				Sweating/Hot spells	24-Jun-2017	9:27	0
				Anorexia	24-Jun-2017	9:27	0
				Nausea	24-Jun-2017	9:27	0
				Vomiting	24-Jun-2017	9:27	0
				Abdominal discomfort	24-Jun-2017	9:27	0
				Fever	24-Jun-2017	9:27	0
				Tachycardia	24-Jun-2017	9:27	0
				Hypotension	24-Jun-2017	9:27	0
R002	10	2	Day 10 Mid	Overall score	24-Jun-2017	12:23	0
				Headache	24-Jun-2017	12:23	0
				Myalgia	24-Jun-2017	12:23	0
				Arthralgia	24-Jun-2017	12:23	0
				Fatigue/Lethargy	24-Jun-2017	12:23	0
				Malaise	24-Jun-2017	12:23	0
				Chills/Shiving/Rigors	24-Jun-2017	12:23	0
				Sweating/Hot spells	24-Jun-2017	12:23	0
				Anorexia	24-Jun-2017	12:23	0
				Nausea	24-Jun-2017	12:23	0

				Vomiting	24-Jun-2017	12:23	0
				Abdominal discomfort	24-Jun-2017	12:23	0
				Fever	24-Jun-2017	12:23	0
				Tachycardia	24-Jun-2017	12:23	0
				Hypotension	24-Jun-2017	12:23	0
R002	10	2	Day 10 PM	Overall score	24-Jun-2017	21:26	1
				Headache	24-Jun-2017	21:26	0
				Myalgia	24-Jun-2017	21:26	0
				Arthralgia	24-Jun-2017	21:26	0
				Fatigue/Lethargy	24-Jun-2017	21:26	0
				Malaise	24-Jun-2017	21:26	0
				Chills/Shiving/Rigors	24-Jun-2017	21:26	0
				Sweating/Hot spells	24-Jun-2017	21:26	0
				Anorexia	24-Jun-2017	21:26	0
				Nausea	24-Jun-2017	21:26	0
				Vomiting	24-Jun-2017	21:26	0
				Abdominal discomfort	24-Jun-2017	21:26	0
				Fever	24-Jun-2017	21:26	1
				Tachycardia	24-Jun-2017	21:26	0
				Hypotension	24-Jun-2017	21:26	0
R002	11	3	Day 11 AM	Overall score	25-Jun-2017	9:35	0
				Headache	25-Jun-2017	9:35	0
				Myalgia	25-Jun-2017	9:35	0
				Arthralgia	25-Jun-2017	9:35	0
				Fatigue/Lethargy	25-Jun-2017	9:35	0
				Malaise	25-Jun-2017	9:35	0
				Chills/Shiving/Rigors	25-Jun-2017	9:35	0
				Sweating/Hot spells	25-Jun-2017	9:35	0
				Anorexia	25-Jun-2017	9:35	0
				Nausea	25-Jun-2017	9:35	0
				Vomiting	25-Jun-2017	9:35	0
				Abdominal discomfort	25-Jun-2017	9:35	0
				Fever	25-Jun-2017	9:35	0

				Tachycardia	25-Jun-2017	9:35	0
				Hypotension	25-Jun-2017	9:35	0
R002	11	3	Day 11 Mid	Overall score	25-Jun-2017	12:45	0
				Headache	25-Jun-2017	12:45	0
				Myalgia	25-Jun-2017	12:45	0
				Arthralgia	25-Jun-2017	12:45	0
				Fatigue/Lethargy	25-Jun-2017	12:45	0
				Malaise	25-Jun-2017	12:45	0
				Chills/Shiving/Rigors	25-Jun-2017	12:45	0
				Sweating/Hot spells	25-Jun-2017	12:45	0
				Anorexia	25-Jun-2017	12:45	0
				Nausea	25-Jun-2017	12:45	0
				Vomiting	25-Jun-2017	12:45	0
				Abdominal discomfort	25-Jun-2017	12:45	0
				Fever	25-Jun-2017	12:45	0
				Tachycardia	25-Jun-2017	12:45	0
				Hypotension	25-Jun-2017	12:45	0
R002	11	3	Day 11 PM	Overall score	25-Jun-2017	19:06	3
				Headache	25-Jun-2017	19:06	1
				Myalgia	25-Jun-2017	19:06	0
				Arthralgia	25-Jun-2017	19:06	0
				Fatigue/Lethargy	25-Jun-2017	19:06	0
				Malaise	25-Jun-2017	19:06	0
				Chills/Shiving/Rigors	25-Jun-2017	19:06	1
				Sweating/Hot spells	25-Jun-2017	19:06	0
				Anorexia	25-Jun-2017	19:06	0
				Nausea	25-Jun-2017	19:06	0
				Vomiting	25-Jun-2017	19:06	0
				Abdominal discomfort	25-Jun-2017	19:06	0
				Fever	25-Jun-2017	19:06	1
				Tachycardia	25-Jun-2017	19:06	0
				Hypotension	25-Jun-2017	19:06	0
R002	12	4	Day 12 AM	Overall score	26-Jun-2017	9:40	1

				Headache	26-Jun-2017	9:40	1
				Myalgia	26-Jun-2017	9:40	0
				Arthralgia	26-Jun-2017	9:40	0
				Fatigue/Lethargy	26-Jun-2017	9:40	0
				Malaise	26-Jun-2017	9:40	0
				Chills/Shiving/Rigors	26-Jun-2017	9:40	0
				Sweating/Hot spells	26-Jun-2017	9:40	0
				Anorexia	26-Jun-2017	9:40	0
				Nausea	26-Jun-2017	9:40	0
				Vomiting	26-Jun-2017	9:40	0
				Abdominal discomfort	26-Jun-2017	9:40	0
				Fever	26-Jun-2017	9:40	0
				Tachycardia	26-Jun-2017	9:40	0
				Hypotension	26-Jun-2017	9:40	0
R002	12	4	Day 12 PM	Overall score	26-Jun-2017	20:29	1
				Headache	26-Jun-2017	20:29	1
				Myalgia	26-Jun-2017	20:29	0
				Arthralgia	26-Jun-2017	20:29	0
				Fatigue/Lethargy	26-Jun-2017	20:29	0
				Malaise	26-Jun-2017	20:29	0
				Chills/Shiving/Rigors	26-Jun-2017	20:29	0
				Sweating/Hot spells	26-Jun-2017	20:29	0
				Anorexia	26-Jun-2017	20:29	0
				Nausea	26-Jun-2017	20:29	0
				Vomiting	26-Jun-2017	20:29	0
				Abdominal discomfort	26-Jun-2017	20:29	0
				Fever	26-Jun-2017	20:29	0
				Tachycardia	26-Jun-2017	20:29	0
				Hypotension	26-Jun-2017	20:29	0
R002	13	5	Day 13 AM	Overall score	27-Jun-2017	8:32	0
				Headache	27-Jun-2017	8:32	0
				Myalgia	27-Jun-2017	8:32	0
				Arthralgia	27-Jun-2017	8:32	0

				Fatigue/Lethargy	27-Jun-2017	8:32	0
				Malaise	27-Jun-2017	8:32	0
				Chills/Shiving/Rigors	27-Jun-2017	8:32	0
				Sweating/Hot spells	27-Jun-2017	8:32	0
				Anorexia	27-Jun-2017	8:32	0
				Nausea	27-Jun-2017	8:32	0
				Vomiting	27-Jun-2017	8:32	0
				Abdominal discomfort	27-Jun-2017	8:32	0
				Fever	27-Jun-2017	8:32	0
				Tachycardia	27-Jun-2017	8:32	0
				Hypotension	27-Jun-2017	8:32	0
R002	13	5	Day 13 PM	Overall score	27-Jun-2017	20:34	0
				Headache	27-Jun-2017	20:34	0
				Myalgia	27-Jun-2017	20:34	0
				Arthralgia	27-Jun-2017	20:34	0
				Fatigue/Lethargy	27-Jun-2017	20:34	0
				Malaise	27-Jun-2017	20:34	0
				Chills/Shiving/Rigors	27-Jun-2017	20:34	0
				Sweating/Hot spells	27-Jun-2017	20:34	0
				Anorexia	27-Jun-2017	20:34	0
				Nausea	27-Jun-2017	20:34	0
				Vomiting	27-Jun-2017	20:34	0
				Abdominal discomfort	27-Jun-2017	20:34	0
				Fever	27-Jun-2017	20:34	0
				Tachycardia	27-Jun-2017	20:34	0
				Hypotension	27-Jun-2017	20:34	0
R002	14	6	Day 14 AM	Overall score	28-Jun-2017	8:55	0
				Headache	28-Jun-2017	8:55	0
				Myalgia	28-Jun-2017	8:55	0
				Arthralgia	28-Jun-2017	8:55	0
				Fatigue/Lethargy	28-Jun-2017	8:55	0
				Malaise	28-Jun-2017	8:55	0
				Chills/Shiving/Rigors	28-Jun-2017	8:55	0

				Sweating/Hot spells	28-Jun-2017	8:55	0
				Anorexia	28-Jun-2017	8:55	0
				Nausea	28-Jun-2017	8:55	0
				Vomiting	28-Jun-2017	8:55	0
				Abdominal discomfort	28-Jun-2017	8:55	0
				Fever	28-Jun-2017	8:55	0
				Tachycardia	28-Jun-2017	8:55	0
				Hypotension	28-Jun-2017	8:55	0
R002	15	7	Day 15 AM	Overall score	29-Jun-2017	8:04	0
				Headache	29-Jun-2017	8:04	0
				Myalgia	29-Jun-2017	8:04	0
				Arthralgia	29-Jun-2017	8:04	0
				Fatigue/Lethargy	29-Jun-2017	8:04	0
				Malaise	29-Jun-2017	8:04	0
				Chills/Shiving/Rigors	29-Jun-2017	8:04	0
				Sweating/Hot spells	29-Jun-2017	8:04	0
				Anorexia	29-Jun-2017	8:04	0
				Nausea	29-Jun-2017	8:04	0
				Vomiting	29-Jun-2017	8:04	0
				Abdominal discomfort	29-Jun-2017	8:04	0
				Fever	29-Jun-2017	8:04	0
				Tachycardia	29-Jun-2017	8:04	0
				Hypotension	29-Jun-2017	8:04	0
R002	18	10	Day 18 AM	Overall score	02-Jul-2017	8:34	0
				Headache	02-Jul-2017	8:34	0
				Myalgia	02-Jul-2017	8:34	0
				Arthralgia	02-Jul-2017	8:34	0
				Fatigue/Lethargy	02-Jul-2017	8:34	0
				Malaise	02-Jul-2017	8:34	0
				Chills/Shiving/Rigors	02-Jul-2017	8:34	0
				Sweating/Hot spells	02-Jul-2017	8:34	0
				Anorexia	02-Jul-2017	8:34	0
				Nausea	02-Jul-2017	8:34	0

				Vomiting	02-Jul-2017	8:34	0
				Abdominal discomfort	02-Jul-2017	8:34	0
				Fever	02-Jul-2017	8:34	0
				Tachycardia	02-Jul-2017	8:34	0
				Hypotension	02-Jul-2017	8:34	0
R002	19	11	Day 19 AM	Overall score	03-Jul-2017	8:09	0
				Headache	03-Jul-2017	8:09	0
				Myalgia	03-Jul-2017	8:09	0
				Arthralgia	03-Jul-2017	8:09	0
				Fatigue/Lethargy	03-Jul-2017	8:09	0
				Malaise	03-Jul-2017	8:09	0
				Chills/Shiving/Rigors	03-Jul-2017	8:09	0
				Sweating/Hot spells	03-Jul-2017	8:09	0
				Anorexia	03-Jul-2017	8:09	0
				Nausea	03-Jul-2017	8:09	0
				Vomiting	03-Jul-2017	8:09	0
				Abdominal discomfort	03-Jul-2017	8:09	0
				Fever	03-Jul-2017	8:09	0
				Tachycardia	03-Jul-2017	8:09	0
				Hypotension	03-Jul-2017	8:09	0
R002	20	12	Day 20 AM	Overall score	04-Jul-2017	8:04	0
				Headache	04-Jul-2017	8:04	0
				Myalgia	04-Jul-2017	8:04	0
				Arthralgia	04-Jul-2017	8:04	0
				Fatigue/Lethargy	04-Jul-2017	8:04	0
				Malaise	04-Jul-2017	8:04	0
				Chills/Shiving/Rigors	04-Jul-2017	8:04	0
				Sweating/Hot spells	04-Jul-2017	8:04	0
				Anorexia	04-Jul-2017	8:04	0
				Nausea	04-Jul-2017	8:04	0
				Vomiting	04-Jul-2017	8:04	0
				Abdominal discomfort	04-Jul-2017	8:04	0
				Fever	04-Jul-2017	8:04	0

				Tachycardia	04-Jul-2017	8:04	0
				Hypotension	04-Jul-2017	8:04	0
R002	22	14	Day 22 AM	Overall score	06-Jul-2017	8:15	0
				Headache	06-Jul-2017	8:15	0
				Myalgia	06-Jul-2017	8:15	0
				Arthralgia	06-Jul-2017	8:15	0
				Fatigue/Lethargy	06-Jul-2017	8:15	0
				Malaise	06-Jul-2017	8:15	0
				Chills/Shiving/Rigors	06-Jul-2017	8:15	0
				Sweating/Hot spells	06-Jul-2017	8:15	0
				Anorexia	06-Jul-2017	8:15	0
				Nausea	06-Jul-2017	8:15	0
				Vomiting	06-Jul-2017	8:15	0
				Abdominal discomfort	06-Jul-2017	8:15	0
				Fever	06-Jul-2017	8:15	0
				Tachycardia	06-Jul-2017	8:15	0
				Hypotension	06-Jul-2017	8:15	0
R002	26	18	Day 26 AM	Overall score	10-Jul-2017	8:53	0
				Headache	10-Jul-2017	8:53	0
				Myalgia	10-Jul-2017	8:53	0
				Arthralgia	10-Jul-2017	8:53	0
				Fatigue/Lethargy	10-Jul-2017	8:53	0
				Malaise	10-Jul-2017	8:53	0
				Chills/Shiving/Rigors	10-Jul-2017	8:53	0
				Sweating/Hot spells	10-Jul-2017	8:53	0
				Anorexia	10-Jul-2017	8:53	0
				Nausea	10-Jul-2017	8:53	0
				Vomiting	10-Jul-2017	8:53	0
				Abdominal discomfort	10-Jul-2017	8:53	0
				Fever	10-Jul-2017	8:53	0
				Tachycardia	10-Jul-2017	8:53	0
				Hypotension	10-Jul-2017	8:53	0
R002	27	19	Day 27 PM	Overall score	11-Jul-2017	17:42	0

				Headache	11-Jul-2017	17:42	0
				Myalgia	11-Jul-2017	17:42	0
				Arthralgia	11-Jul-2017	17:42	0
				Fatigue/Lethargy	11-Jul-2017	17:42	0
				Malaise	11-Jul-2017	17:42	0
				Chills/Shiving/Rigors	11-Jul-2017	17:42	0
				Sweating/Hot spells	11-Jul-2017	17:42	0
				Anorexia	11-Jul-2017	17:42	0
				Nausea	11-Jul-2017	17:42	0
				Vomiting	11-Jul-2017	17:42	0
				Abdominal discomfort	11-Jul-2017	17:42	0
				Fever	11-Jul-2017	17:42	0
				Tachycardia	11-Jul-2017	17:42	0
				Hypotension	11-Jul-2017	17:42	0
R002	28	20	Day 28 AM	Overall score	12-Jul-2017	9:10	0
				Headache	12-Jul-2017	9:10	0
				Myalgia	12-Jul-2017	9:10	0
				Arthralgia	12-Jul-2017	9:10	0
				Fatigue/Lethargy	12-Jul-2017	9:10	0
				Malaise	12-Jul-2017	9:10	0
				Chills/Shiving/Rigors	12-Jul-2017	9:10	0
				Sweating/Hot spells	12-Jul-2017	9:10	0
				Anorexia	12-Jul-2017	9:10	0
				Nausea	12-Jul-2017	9:10	0
				Vomiting	12-Jul-2017	9:10	0
				Abdominal discomfort	12-Jul-2017	9:10	0
				Fever	12-Jul-2017	9:10	0
				Tachycardia	12-Jul-2017	9:10	0
				Hypotension	12-Jul-2017	9:10	0
R002	29	21	Day 29 AM	Overall score	13-Jul-2017	9:35	0
				Headache	13-Jul-2017	9:35	0
				Myalgia	13-Jul-2017	9:35	0
				Arthralgia	13-Jul-2017	9:35	0

				Fatigue/Lethargy	13-Jul-2017	9:35	0
				Malaise	13-Jul-2017	9:35	0
				Chills/Shiving/Rigors	13-Jul-2017	9:35	0
				Sweating/Hot spells	13-Jul-2017	9:35	0
				Anorexia	13-Jul-2017	9:35	0
				Nausea	13-Jul-2017	9:35	0
				Vomiting	13-Jul-2017	9:35	0
				Abdominal discomfort	13-Jul-2017	9:35	0
				Fever	13-Jul-2017	9:35	0
				Tachycardia	13-Jul-2017	9:35	0
				Hypotension	13-Jul-2017	9:35	0
R002	30	22	Day 30 AM	Overall score	14-Jul-2017	8:40	0
				Headache	14-Jul-2017	8:40	0
				Myalgia	14-Jul-2017	8:40	0
				Arthralgia	14-Jul-2017	8:40	0
				Fatigue/Lethargy	14-Jul-2017	8:40	0
				Malaise	14-Jul-2017	8:40	0
				Chills/Shiving/Rigors	14-Jul-2017	8:40	0
				Sweating/Hot spells	14-Jul-2017	8:40	0
				Anorexia	14-Jul-2017	8:40	0
				Nausea	14-Jul-2017	8:40	0
				Vomiting	14-Jul-2017	8:40	0
				Abdominal discomfort	14-Jul-2017	8:40	0
				Fever	14-Jul-2017	8:40	0
				Tachycardia	14-Jul-2017	8:40	0
				Hypotension	14-Jul-2017	8:40	0
R002	33	25	Day 33 AM	Overall score	17-Jul-2017	8:40	0
				Headache	17-Jul-2017	8:40	0
				Myalgia	17-Jul-2017	8:40	0
				Arthralgia	17-Jul-2017	8:40	0
				Fatigue/Lethargy	17-Jul-2017	8:40	0
				Malaise	17-Jul-2017	8:40	0
				Chills/Shiving/Rigors	17-Jul-2017	8:40	0

				Sweating/Hot spells	17-Jul-2017	8:40	0
				Anorexia	17-Jul-2017	8:40	0
				Nausea	17-Jul-2017	8:40	0
				Vomiting	17-Jul-2017	8:40	0
				Abdominal discomfort	17-Jul-2017	8:40	0
				Fever	17-Jul-2017	8:40	0
				Tachycardia	17-Jul-2017	8:40	0
				Hypotension	17-Jul-2017	8:40	0
R002	37	29	Day 37 AM	Overall score	21-Jul-2017	9:03	0
				Headache	21-Jul-2017	9:03	0
				Myalgia	21-Jul-2017	9:03	0
				Arthralgia	21-Jul-2017	9:03	0
				Fatigue/Lethargy	21-Jul-2017	9:03	0
				Malaise	21-Jul-2017	9:03	0
				Chills/Shiving/Rigors	21-Jul-2017	9:03	0
				Sweating/Hot spells	21-Jul-2017	9:03	0
				Anorexia	21-Jul-2017	9:03	0
				Nausea	21-Jul-2017	9:03	0
				Vomiting	21-Jul-2017	9:03	0
				Abdominal discomfort	21-Jul-2017	9:03	0
				Fever	21-Jul-2017	9:03	0
				Tachycardia	21-Jul-2017	9:03	0
				Hypotension	21-Jul-2017	9:03	0
R002	45	37	Day 45 AM	Overall score	29-Jul-2017	9:56	0
				Headache	29-Jul-2017	9:56	0
				Myalgia	29-Jul-2017	9:56	0
				Arthralgia	29-Jul-2017	9:56	0
				Fatigue/Lethargy	29-Jul-2017	9:56	0
				Malaise	29-Jul-2017	9:56	0
				Chills/Shiving/Rigors	29-Jul-2017	9:56	0
				Sweating/Hot spells	29-Jul-2017	9:56	0
				Anorexia	29-Jul-2017	9:56	0
				Nausea	29-Jul-2017	9:56	0

				Vomiting	29-Jul-2017	9:56	0
				Abdominal discomfort	29-Jul-2017	9:56	0
				Fever	29-Jul-2017	9:56	0
				Tachycardia	29-Jul-2017	9:56	0
				Hypotension	29-Jul-2017	9:56	0
R002	90	82	Day 90	Overall score	12-Sep-2017	9:37	0
				Headache	12-Sep-2017	9:37	0
				Myalgia	12-Sep-2017	9:37	0
				Arthralgia	12-Sep-2017	9:37	0
				Fatigue/Lethargy	12-Sep-2017	9:37	0
				Malaise	12-Sep-2017	9:37	0
				Chills/Shiving/Rigors	12-Sep-2017	9:37	0
				Sweating/Hot spells	12-Sep-2017	9:37	0
				Anorexia	12-Sep-2017	9:37	0
				Nausea	12-Sep-2017	9:37	0
				Vomiting	12-Sep-2017	9:37	0
				Abdominal discomfort	12-Sep-2017	9:37	0
				Fever	12-Sep-2017	9:37	0
				Tachycardia	12-Sep-2017	9:37	0
				Hypotension	12-Sep-2017	9:37	0

Notes:
Study Day is the number of days relative to day of administration of the inoculum, where Study Day = 0 for inoculum dosing day.
Day relative to Artesunate is the number of days relative to day of administration of Artesunate where the day of first dose = 1.
Result: 0 = Absent; 1= Mild; 2 = Moderate; 3 = Severe.

16.2.10 Additional comments

Listing 16.2.10
Additional Comments

Subject Number	Comment Number	Comment Reference	Comment
R001	1	Screening consent	Consent v2.0 signed 24 May 2017 at 07:41
	2	Screening vitals signs	Repeat vitals taken for out of range heart rate
	3	Day 0 Pre-inoculum vitals	Repeat vitals taken due to being out of window
	4	Day 0 Pre-inoculum ECG	Repeat ECG taken due to being out of window
	5	Day 3 telephone contact	Participant was brought in for Day 3 PCR sample due to PI request
	6	Day 3 27 May 2017	Unscheduled vitals taken, repeat HR taken due to being out of range
	7	Vital signs (unscheduled)	Performed to monitor fever
	8	Day 16 AM vital signs	Repeat vitals taken for out of range temperature
	9	Day 3 vital signs (unscheduled)	Position for unscheduled vitals was not recorded
	10	Day 16 AM vital signs	Time was not recorded for a repeat, out of range temperature
	11	Unscheduled HIV bloods 29Jun2017	Repeat HIV serology performed at 08:37 on 29 Jun 2017
	12	Serology 21 Jun2017	HIV result was reported as weak positive for HIV Ag/Ab HIV1 Western Blot performed. Comments on pathology report "Unable to interpret HIV Western Blot due to high background staining. In this situation HIV1 proviral DNA testing may help clarify the screening result". HIV p24 Antigen also performed/tested result = non reactive. Investigator requested to repeat serology and proviral DNA to be sent.

	13	Unscheduled serology 29Jun2017	HIV Ag/Ab result - weak positive. p24 Antigen result - non reactive. HIV1 Western blot tested/performed. Comments on pathogology report "Unable to interpret HIV Western Blot due to high background staining. In this situation HIV1 proviral DNA testing may help clarify the screening result". Investigator commented likely false positive HIV Ag/Ab given negative p24 and proviral DNA sent to St Vincent's laboratory in Sydney returned negative. Further HIV serology tests conducted, laboratory findings for HIV screening assay Architect HIV Ab/Ag - reactive (NCS), Liason HIV Ab/Ag - non reactive, Western blot - indeterminate (NCS), HIV-1 p24 antigen not tested, HIV-1 proviral DNA - not detected. HIV antibody status - non reactive. Comment on pathology report "The screening test repeat showed some reactivity which commonly occurs in uninfected individuals due to the presence of non-specific antibodies. In a comprehensive study by HIV state reference laboratories, this indeterminate HIV-1 Western blot reactivity is not associated with seroconversion to HIV-1 positive. Because of the negative results in HIV supplemental assays including the HIV proviral DNA PCR test, this patient is considered unlikely to have HIV since the sample was collected".
	14	21Jun2017 serology	On 29Jun2018 the Investigator documented in source notes "HIV Ag/Ab weak positive. HIV.1 Western blot - unable to interpret high background noise. HIV p24 Antigen negative, unlikely to represent HIV infection, likely false positive due to recent malaria infection.
R002	1	Screening consent	Consent v2.0 signed 24 May 2017 at 09:10
	2	Day 11 vitals signs	Repeat vitals taken for out of range temperature
	3	Day 12 vitals signs	Repeat vitals taken for out of range temperature
	4	Day 18 vitals signs	Repeat vitals taken for out of range temperature
	5	Day 37 vitals signs	Repeat vitals taken for out of range temperature
	6	Unscheduled vitals signs	From 24 Jun 2017 to 25 Jun 2017 unscheduled vitals signs taken to monitor fevers overnight

Notes:

Appendix 16.3 Case Report Forms Submitted

Not applicable.
