# Supplementary material for: Safety and parasite clearance of artemisinin-resistant Plasmodium falciparum infection: A pilot and a randomised volunteer infection study in Australia
Source: PLoS Med. 2020 Aug 21;17(8):e1003203. doi: 10.1371/journal.pmed.1003203 (PMC7444516; doi:10.1371/journal.pmed.1003203)
Supplement: S2 Appendix — (PDF) [file pmed.1003203.s026.pdf]

TABLE OF CONTENTS

Appendix 16.1 Study Information

16.1.1 Protocol and protocol amendments

16.1.2 Sample case report form

16.1.3 List of Human Research Ethics Committees

16.1.4 List and description of investigators and other important participants in the study

16.1.5 Signature of Principal Investigator

16.1.6 List of investigational product(s) batch numbers

16.1.7 Randomisation scheme and codes

16.1.8 Audit certificates

16.1.9 Documentation of statistical methods

16.1.10 Documentation of inter-laboratory standardisation methods and laboratory QA procedures

16.1.11 Publications based on study

16.1.12 Important publications referenced in the report

16.1.13 Reports

Appendix 16.2 Subject Data Listings

16.2.1 Study enrolment and completion/discontinuation

16.2.1.1 Study enrolment and completion/discontinuation

16.2.1.2 Visit Dates

16.2.2 Protocol deviations

16.2.2.1 Protocol Deviations

16.2.3 Subjects excluded from the efficacy analysis

16.2.3.1 Analysis Population Assignment

16.2.4 Demographic and baseline data

16.2.4.1 Demography

16.2.4.2 Inclusion and Exclusion Criteria

16.2.4.3 Medical History

16.2.4.4 Pregnancy Test

16.2.4.5 Follicle Stimulating Hormone (FSH) Test

16.2.4.6 RBC Alloantibody

16.2.4.7 Serology

16.2.4.8 Urine Drug Screen

16.2.4.9 Alcohol Breath Test

16.2.4.10 G6PD

16.2.4.11 Body Weight, Height and BMI

16.2.4.12a Prior Medications

16.2.4.12b Concomitant Medications

16.2.5 Compliance and drug concentration data

16.2.5.1 Malaria Inoculum Administration

16.2.5.2 Artesunate Administration

16.2.5.3 Piperaquine Phosphate Administration

16.2.5.4 Rescue Medications (Malarone® and Primacin™) Administration

16.2.5.5 Plasma Pharmacokinetic Sample Collection Time and Concentration For Artesunate

16.2.5.6 Plasma pharmacokinetic sample collection time and concentration Ffor dihydroartemisinin

16.2.6 Parasitaemia and parasite lifecycle stage qPCR/qRT-PCR data

16.2.6.1 Direct Feeding Assay

16.2.6.2 Membrane Feeding Assay

16.2.6.3 Malaria 18S qPCR

16.2.6.4 qRT-PCR *pfs25* Daily

16.2.6.5 qRT-PCR *pfs25* Retrospective

16.2.7 Adverse events

16.2.7.1 Adverse Events

16.2.7.1a Adverse Events Related to Inoculum Only

16.2.7.1b Treatment Emergent Adverse Events Related to Artesunate Only

16.2.7.1c Treatment Emergent Adverse Events Related to Piperaquine Only

16.2.7.2 Serious Adverse Events

16.2.7.3 Adverse Events Leading to Discontinuation or Withdrawal

16.2.7.4 Deaths

16.2.8 Clinical laboratory measurements

16.2.8.1a Individual Haematology Results

16.2.8.1b Individual Abnormal Haematology Results

16.2.8.2a Individual Biochemistry Results

16.2.8.2b Individual Abnormal Biochemistry Results

16.2.8.3a Individual Urinalysis Results (Dipstick)

16.2.8.3b Individual Abnormal Urinalysis Results (Dipstick)

16.2.8.4a Individual Microscopy and Urinalysis Results (Laboratory)

16.2.8.4b Individual Abnormal Microscopy and Urinalysis Results (Laboratory)

Appendix 16.3 Case Report Forms

Appendix 16.4 Other Safety Data in Subject Data Listings

16.4.1 Vital Signs (Blood Pressure and Heart Rate)

16.4.2 Vital Signs (Body Temperature and Respiratory Rate)

16.4.3 Individual Abnormal Vital Signs

16.4.4a ECG Findings

16.4.4b Abnormal ECG Interpretation

16.4.5 Physical Examination

16.4.6 Individual Abnormal Physical Examination

16.4.7 Malaria Clinical Score

16.4.8 General Comments

Appendix 16.1 Study Information

16.1.1 Protocol and protocol amendments

QIMR Berghofer
Medical Research Institute

A comparative study of the parasitological response to single dose artesunate therapy among subjects experimentally infected with either the artemisinin-resistant *Plasmodium falciparum* isolate K13 or the artemisinin-sensitive isolate 3D7

Protocol Identifying Number: QP17C11/P2339

Principal Investigator: Prof. James McCarthy

Sponsor: QIMR Berghofer Medical Research Institute

Funded by: Medicines for Malaria Venture

Draft or Version Number: v3.0

23 July 2018

CONFIDENTIALITY STATEMENT

This document contains information that is privileged or confidential. As such, it may not be disclosed unless specific prior permission is granted in writing by QIMR Berghofer or such disclosure is required by federal or other laws or regulations. Persons to whom any of this information is to be disclosed must first be informed that the information is confidential. These restrictions on disclosure will apply equally to all future information supplied, which is indicated as privileged or confidential.

Table of Contents

LIST OF ABBREVIATIONS 1
STATEMENT OF COMPLIANCE 3
PROTOCOL SUMMARY 5
SCHEMATIC OF STUDY DESIGN 12
1 KEY ROLES 13
2 INTRODUCTION: BACKGROUND INFORMATION AND SCIENTIFIC RATIONALE 16
2.1 Background Information 16
2.2 Rationale 17
2.3 Potential Risks and Benefits 19
2.3.1 Known Potential Risks 19
2.3.2 Known Potential Benefits 24
3 OBJECTIVES AND PURPOSE 24
4 STUDY DESIGN AND ENDPOINTS 25
4.1 Description of the Study Design 25
4.2 Study Endpoints 29
4.2.1 Primary Endpoint 29
4.2.2 Secondary Endpoints 29
4.2.3 Exploratory Endpoints 29
5 STUDY ENROLLMENT AND WITHDRAWAL 30
5.1 Subject Inclusion Criteria 30
5.2 Subject Exclusion Criteria 31
5.3 Strategies for Recruitment and Retention 36
5.4 Subject Withdrawal or termination 36
5.4.1 Reasons for Withdrawal or Termination 36
5.4.2 Handling of Subject Withdrawals or termination 37
5.5 Premature Termination or Suspension of Study 37
6 STUDY AGENT 38
6.1 Study Agent(s) and Control Description 38
6.1.1 Acquisition 38
6.1.2 Formulation, Appearance, Packaging, and Labeling 39
6.1.3 Product Storage and Stability 40
6.1.4 Preparation 40
6.1.5 Dosing and Administration 41
6.1.6 Route of Administration 44
6.1.7 Starting Dose and Dose Escalation Schedule 44
6.1.8 Dose Adjustments/Modifications/Delays 44
6.1.9 Duration of Therapy 44
6.1.10 Tracking of Dose 44
6.2 Study agent Accountability Procedures 45

7 STUDY PROCEDURES AND SCHEDULE 45
7.1 Study Procedures/Evaluations 45
7.1.1 Study specific procedures 45
7.1.2 Standard of care study procedures 48
7.2 Laboratory Procedures/Evaluations 49
7.2.1 Clinical Laboratory Evaluations 49
7.2.2 Other Assays or Procedures 51
7.2.3 Specimen Preparation, Handling, and Storage 55
7.2.4 Specimen Shipment 55
7.3 Study Schedule 55
7.3.1 Screening 55
7.3.2 Enrollment/Baseline 57
7.3.3 Follow-up 59
7.3.4 Final Study Visit 64
7.3.5 Early Termination Visit 65
7.3.6 Unscheduled Visit 65
7.3.7 Schedule of Events Table 65
7.4 Justification for Sensitive Procedures 68
7.5 Concomitant Medications, Treatments, and Procedures 68
7.5.1 Precautionary Medications, Treatments, and Procedures 68
7.6 Prohibited Medications, Treatments, and Procedures 68
7.7 Prophylactic Medications, Treatments, and Procedures 69
7.8 Rescue Medications, Treatments, and Procedures 69
7.9 Subject Access to Study Agent At Study Closure 69
8 ASSESSMENT OF SAFETY 69
8.1 Specification of Safety Parameters 69
8.1.1 Definition of Adverse Events (AE) 70
8.1.2 Definition of Serious Adverse Events (SAE) 71
8.1.3 Definition of Unanticipated Problems (UP) 72
8.2 Classification of an Adverse Event 72
8.2.1 Severity of Event 72
8.2.2 Relationship to Study Agent 73
8.2.3 Expectedness 74
8.3 Time Period and Frequency for Event Assessment and Follow-Up 75
8.4 Reporting Procedures 75
8.4.1 Adverse Event Reporting 75
8.4.2 Serious Adverse Event Reporting 76
8.4.3 Unanticipated Problem Reporting 77
8.4.4 Events of Special Interest 77
8.4.5 Reporting of Pregnancy 77

8.5 Study Halting Rules ... 78
8.6 Safety Oversight ... 78
9 CLINICAL MONITORING ... 79
10 STATISTICAL CONSIDERATIONS ... 79
10.1 Statistical and Analytical Plans ... 79
10.2 Statistical Hypotheses ... 79
10.3 Analysis Datasets ... 80
10.4 Description of Statistical Methods ... 80
10.4.1 General Approach ... 80
10.4.2 Analysis of the Primary Efficacy Endpoint(s) ... 80
10.4.3 Analysis of the Secondary Endpoint(s) ... 80
10.4.4 Safety Analyses ... 80
10.4.5 Adherence and Retention Analyses ... 81
10.4.6 Baseline Descriptive Statistics ... 81
10.4.7 Planned Interim Analyses ... 81
10.4.8 Additional Sub-Group Analyses ... 82
10.4.9 Multiple Comparison/Multiplicity ... 82
10.4.10 Tabulation of Individual Response Data ... 82
10.4.11 Exploratory Analyses ... 82
10.5 Sample Size ... 82
10.6 Measures to Minimize Bias ... 83
10.6.1 Enrollment/ Randomization/ Masking Procedures ... 83
10.6.2 Evaluation of Success of Blinding ... 83
10.6.3 Breaking the Study Blind/Subject Code ... 84
11 SOURCE DOCUMENTS AND ACCESS TO SOURCE DATA/DOCUMENTS ... 84
12 QUALITY ASSURANCE AND QUALITY CONTROL ... 84
13 ETHICS/PROTECTION OF HUMAN SUBJECTS ... 85
13.1 Ethical Standard ... 85
13.2 Ethical Review ... 85
13.3 Informed Consent Process ... 87
13.3.1 Consent/assent and Other Informational Documents Provided to Subjects ... 87
13.3.2 Consent Procedures and Documentation ... 87
13.4 Subject and data Confidentiality ... 87
13.4.1 Research Use of Stored Human Samples, Specimens or Data ... 88
13.5 Future Use of Stored Specimens ... 88
14 DATA HANDLING AND RECORD KEEPING ... 89
14.1 Data Collection and Management Responsibilities ... 89
14.2 Study Records Retention ... 89
14.3 Protocol Deviations ... 90
14.4 Publication and Data Sharing Policy ... 90

15 STUDY ADMINISTRATION 91
15.1 Study Leadership 91
15.2 Liability/Indemnity/Insurance 91
16 CONFLICT OF INTEREST POLICY 92
17 LITERATURE REFERENCES 92
APPENDIX 96

LIST OF ABBREVIATIONS

AE	Adverse Event
ALT	Alanine Aminotransferase
anti-HBc Ab	Anti-Hepatitis B Core Antibodies
anti-HCV	Anti-Hepatitis C Virus
anti-HIV1	Anti-Human Immunodeficiency Virus 1
anti-HIV2	Anti-Human Immunodeficiency Virus 2
AST	Aspartate Aminotransferase
AUC	Area Under the Curve
AUC_{inf}	AUC Curve to Infinite Time
AUC_{last}	AUC Curve to Last Quantifiable Concentration
Blood Service	Australian Red Cross Blood Service
C_{max}	Maximum Plasma Concentration
CNS	Clinical Network Services
CSR	Clinical Study Report
CTCAE	Common Terminology Criteria for Adverse Events
DFA	Direct Feeding Assay
eCRF	Electronic Case Report Form
ECG	Electrocardiogram
EOS	End of Study
FDA	Food and Drug Administration
G6PD	Glucose-6-Phosphate Dehydrogenase
GCP	Good Clinical Practice
GMP	Good Manufacturing Practice
HAV	Hepatitis A Virus
HBs Ag	Hepatitis B surface antigen
HCV	Hepatitis C Virus
HEV	Hepatitis E Virus
HDL	High Density Lipoprotein
HIV	Human Immunodeficiency Virus
HREC	Human Research Ethics Committee
IBSM	Induced Blood Stage Malaria
ICH	International Conference on Harmonization
IMM	Independent Medical Monitor
IMP	Investigational Medicinal Product
IV	Intravenous
LFT	Liver Function Test
MCB	Master Cell Bank
MedDRA	Medical Dictionary for Regulatory Activities
MFA	Membrane Feeding Assay
MMV	Medicines for Malaria Venture
MRI	Magnetic Resonance Imaging
NHMRC	National Health and Medical Research Council

PD	Pharmacodynamic
PET	Positron Emission Tomography
PK	Pharmacokinetic
QIMR Berghofer	Queensland Institute of Medical Research Berghofer
qPCR	Quantitative Polymerase Chain Reaction
qRT-PCR	Quantitative Reverse Transcription Polymerase Chain Reaction
QTc	Corrected QT
RBC	Red Blood Cell
Rh	Rhesus Antibody
SAE	Serious Adverse Event
SAP	Statistical Analysis Plan
SOP	Standard Operating Procedures
SRT	Safety Review Team
SUSAR	Suspected Unexpected Serious Adverse Reaction
$t_{1/2}$	Terminal Half-Life
TEAE	Treatment-Emergent Adverse Event
TGA	Therapeutic Goods Administration
T_{max}	Time taken to reach C_{max}
ULN	Upper Limit of Normal
WHO	World Health Organization
WOCBP	Women of Childbearing Potential

STATEMENT OF COMPLIANCE

Investigator declaration

I have read the protocol and agree that it contains all necessary details for carrying out the study as described. I will conduct this protocol as outlined herein and will make a reasonable effort to complete the study within the time designated.

I agree to personally conduct or supervise the described study.

The study will be conducted in accordance with the following:

- World Medical Association Declaration of Helsinki – Ethical Principles for Medical Research Involving Human Subjects (Fortaleza, Brazil 2013).
- NHMRC National Statement on Ethical Conduct in Human Research (2007, updated May 2015).
- Note for Guidance on Good Clinical Practice – Annotated with TGA Comments (CPMP/ICH/135/95), as adopted by the Australian Therapeutic Goods Administration (July 2000).
- Current ethics approved Clinical Trial Protocol.

I agree to inform all subjects that the study drugs are being used for investigational purposes and I will ensure that the requirements related to obtaining informed consent are in accordance with International Council of Harmonisation (ICH) Guidelines for Good Clinical Practice (GCP) section 4.8 and local requirements.

I agree to report adverse events that occur in the course of the study to the Sponsor in accordance with ICH Guidelines for GCP section 4.11 and local requirements.

I have read and understand the information in the Investigator's Brochures, including the potential risks and side effects of the study drugs.

I agree to promptly report to the Human Research Ethics Committee (HREC) all changes in the research activity and all unanticipated problems involving risk to subjects. I will not make any changes to the conduct of the study without HREC and Sponsor approval, except when necessary to eliminate apparent immediate harm to subjects.

I agree to maintain adequate and accurate records and make those records available in accordance with ICH Guidelines for GCP section 4.11 and local requirements.

I agree to ensure that all associates, colleagues, and employees assisting in the conduct of the study are informed about their obligations in meeting the above commitments.

I understand that the study may be terminated or enrolment suspended at any time by the Sponsor, with or without cause, or by me if it becomes necessary to protect the best interest of the subjects.

Professor James McCarthy, Principal Investigator

Date: 24/7/18

Signatories

The undersigned parties agree that the protocol was written in accordance with the World Medical Association Declaration of Helsinki – Ethical Principles for Medical Research Involving Human Subjects (Fortaleza, Brazil 2013), the NHMRC National Statement on Ethical Conduct in Human Research (2007, updated May 2015), and the Note for Guidance on Good Clinical Practice – Annotated with TGA Comments (CPMP/ICH/135/95), as adopted by the Australian Therapeutic Goods Administration (July 2000).

**Name**	Signature	Date
Protocol Writer:<br>**Dr Rebecca Watts PhD**<br>Clinical Trials Project Manager<br>QIMR Berghofer Medical Research Institute	[signature]	23/07/18

This clinical trial protocol has been reviewed and approved by the Sponsor.

**Name**	Signature	Date
Sponsor or Sponsor Representative:<br>**Prof. David Whiteman MBBS, PhD, FAFPHM**<br>Deputy Director<br>QIMR Berghofer Medical Research Institute	[signature]	24/7/18

PROTOCOL SUMMARY

**Title:**	A comparative study of the parasitological response to single dose artesunate therapy among subjects experimentally infected with either the artemisinin-resistant *Plasmodium falciparum* isolate K13 or the artemisinin-sensitive isolate 3D7
**Précis:**	This study will compare the response of the *Plasmodium falciparum* isolates Cam3.II^{R539T} (referred to as K13, artemisinin-resistant) and 3D7 (artemisinin-sensitive) to a single dose of the antimalarial drug artesunate in healthy subjects using the induced blood stage malaria model. The study will be conducted in up to 27 subjects, in up to 3 cohorts. Up to 18 subjects will receive the K13 challenge agent and up to 9 subjects will receive the 3D7 challenge agent, to achieve a ratio of 2:1 over the whole study. Cohort 1 planned to recruit 9 subjects, randomised to receive either the K13 or 3D7 challenge agent, in a 2:1 ratio. However, only 6 subjects received the challenge agents in Cohort 1 (3 K13 and 3 3D7). Up to 21 subjects distributed over Cohorts 2 and 3 will receive either the K13 or 3D7 challenge agents, to reach up to 27 subjects in total.<br><br>A review of the safety, tolerability and parasitaemia data from the preceding cohort will be conducted by the Safety Review Team prior to malaria parasite inoculation of the next cohort. Data from at least 3 weeks post-inoculation will be required for each review.<br><br>Subjects randomised to receive the K13 challenge agent will be inoculated on Day 0 with approximately 2 800 viable *P. falciparum* K13 parasite-infected red blood cells (RBCs). Subjects randomised to receive the 3D7 challenge agent will be inoculated on Day 1 with approximately 2 800 viable *P. falciparum* 3D7 parasite-infected RBCs. On an outpatient basis, subjects will be monitored daily via phone and then will attend the clinical unit daily from 4 days post-inoculation – Day 4 for K13 subjects and Day 5 for 3D7 subjects – until positive for presence of malaria parasites by quantitative polymerase chain reaction (qPCR) targeting the 18S rRNA gene (referred to as malaria 18S qPCR). Once qPCR positive, subjects will be monitored twice daily until artesunate treatment for parasitaemia, symptoms or signs of malaria, and adverse events. The threshold for commencement of artesunate treatment will be when

	all subjects reach a parasitaemia of ≥5 000 parasites/mL. If the parasitaemia of any subject is ≥5 000 parasites/mL, and is accompanied by a malaria clinical score >6 before all subjects have reached the treatment threshold, then treatment of that subject will begin within a 24 hour period. Subjects may also receive artesunate treatment at any time based on parasitaemia and/or clinical symptoms at the Investigator's discretion. Subjects will be admitted to the clinical unit (approximately Day 9), administered a single oral dose of approximately 2 mg/kg artesunate, and confined for 72 hours to monitor for safety and tolerability of therapy and to ensure adequate clinical and parasitological response. The plasma concentration-time profiles of artesunate and its active metabolite dihydroartemisinin will also be assessed.<br><br>Once clinically well, subjects will be followed up on an out-patient basis for monitoring of safety and parasite clearance. Subjects may also be evaluated for the presence of sexual parasite stages (gametocytes) and other parasite lifecycle stages in the blood, by parasite lifecycle stage reverse transcription qPCR (qRT-PCR) targeting genes such as the female gametocyte-specific transcript *pfs25*, the male gametocyte-specific transcript *pfMGET*, and/or the ring-stage transcript *pfSBP-1*. Infectivity of parasites to *Anopheles* mosquitoes may be investigated by direct skin feeding and/or membrane feeding assays, at the Investigator's discretion using parasite lifecycle stage qRT-PCR to determine probability of infectivity.<br><br>It is anticipated that artesunate treatment will reduce K13 parasitaemia, although at a slower rate than for the 3D7 artemisinin-sensitive isolate. If artesunate does not clear parasitaemia, subjects will be administered a drug known to be active against both K13 and 3D7 isolates (piperaquine phosphate as a single dose of 960 mg).<br><br><u>Cohort 1:</u><br><br>For Cohort 1, piperaquine phosphate treatment will occur on an individual subject basis in the event of unsatisfactory parasite clearance, defined as 2 consecutive malaria 18S qPCR time-points showing a decrease in parasitaemia of less than 20% of baseline (i.e., the parasitaemia before drug treatment) by 72 hours post-artesunate treatment. Subjects will also be treated with piperaquine phosphate if recrudescence occurs after artesunate treatment,

	defined as ≥5 000 blood stage parasites/mL and a 2-fold increase within 48 hours, or a malaria clinical score >6. Piperaquine phosphate treatment can also be undertaken at the Investigator's discretion.<br><br>All Cohort 1 subjects will receive compulsory rescue treatment with Malarone® (atovaquone/proguanil hydrochloride; 3 day course) on Day 26±3 or earlier at the Investigator's discretion. Subjects may also be treated with a single oral dose of 45 mg primaquine (Primacin™) at the time of Malarone® treatment, if gametocytaemia is suspected from parasite lifecycle stage qRT-PCR or by the presence of stable low level parasitaemia, or at the Investigator's discretion, to ensure complete clearance of gametocytes.<br><br><u>Cohorts 2 and 3:</u><br><br>For Cohorts 2 and 3, piperaquine phosphate treatment will occur for subjects inoculated with the 3D7 challenge agent as outlined above for Cohort 1. However, K13 subjects in Cohorts 2 and 3 will be treated with piperaquine phosphate on Day 11 AM or earlier at the Investigator's discretion. This is to avoid the high parasitaemias seen in K13 subjects in Cohort 1 on Day 11 PM prior to piperaquine phosphate treatment.<br><br>All Cohort 2 and 3 subjects will be treated with a single oral dose of 45 mg primaquine (Primacin™) on Day 23±3 or earlier at the Investigator's discretion. This is to ensure complete clearance of gametocytes before Day 28±3 (End of Study). All subjects will receive compulsory rescue treatment with Malarone® (atovaquone/proguanil hydrochloride; 3 day course) on Day 25±3 or earlier at the Investigator's discretion.<br><br><u>All cohorts:</u><br><br>Follow-up for safety assessments will be performed on Day 28±3 (End of Study). The Safety Review Team will review the safety and tolerability data from the study after completion of the last cohort.<br><br>Subjects who are enrolled in this study may also enrol in optional exploratory malaria research studies, which are outlined in a separate protocol and informed Consent Form. These optional exploratory studies include research into parasite dormancy, parasite growth rates, *var* gene expression, parasite biomarkers (BIOME),

	immunology, serology, and metabolomics.<br><br>In Cohort 3, up to two subjects may also be recruited for a parallel, optional, observational exploratory study "Advanced medical imaging in subpatent malaria: a second pilot study" which is outlined in a separate protocol and informed Consent Form.
**Objectives:**	Primary:<br><br>• To compare the parasite clearance profiles of the *P. falciparum* K13 and 3D7 isolates after administration of artesunate.<br><br>• To determine the safety and tolerability associated with infection of healthy subjects with the *P. falciparum* K13 and 3D7 isolates.<br><br>Exploratory:<br><br>• To characterise the pharmacokinetic profile of artesunate and its active metabolite, dihydroartemisinin, in *P. falciparum* K13 and 3D7 infected subjects.<br><br>• To characterise the pharmacokinetic/pharmacodynamic relationship between artesunate and dihydroartemisinin pharmacokinetics and parasite clearance in *P. falciparum* K13 and 3D7 infected subjects.<br><br>• To determine if gametocytes appear in the blood of subjects infected with *P. falciparum* K13 or 3D7.<br><br>• To determine if K13 and 3D7 gametocytes, if present, are infective to mosquitoes (may be performed in selected cohorts at the Investigator's discretion).<br><br>• To investigate and identify any possible associations between subjects genetic polymorphisms and liver function (or phenotype) following infection with *P. falciparum* K13 or 3D7 (optional and requires consent via genetic testing Consent Form).<br><br>• To characterise the antimalarial treatment effects on *P. falciparum* K13 and 3D7 parasites using phenotypic and transcriptomic approaches (Cohorts 2 and 3).<br><br>• To investigate the *in vivo* presence of dormant parasites (Cohorts

	2 and 3).
**Endpoints:**	Primary:<br>• The parasite clearance profiles of *P. falciparum* K13 and 3D7 after administration of artesunate will be characterised by the parasite reduction ratio and parasite clearance $t_{1/2}$. A t-test will be used to compare the slope of the clearance curves for K13 and 3D7 isolates.<br>• The safety and tolerability of *P. falciparum* K13 and 3D7 infection will be evaluated from adverse event and serious adverse event recording (including severity and causality), physical examinations, vital signs, clinical biochemistry, haematology, and urinalysis.<br><br>Exploratory:<br>• The following pharmacokinetic parameters will be estimated for artesunate and dihydroartemisinin: AUC_{last}, AUC_{inf}, C_{max}, T_{max} and $t_{1/2}$.<br>• A pharmacokinetic/pharmacodynamic model describing the relationship between artesunate and dihydroartemisinin plasma concentration and the effect on parasitaemia measured by qPCR will be developed.<br>• The presence of gametocytes in subjects will be determined by parasite lifecycle stage qRT-PCR until approximately Day 28±3.<br>• Infectivity of gametocytes (if present) to mosquitoes will be reported as prevalence of infection (percentage of oocyst positive mosquitoes) (may be performed in selected cohorts at the Investigator's discretion).<br>• The absence or presence of a genetic variation that may have a link to changes in liver function (or phenotype) following infection with *P. falciparum* (optional and requires consent via genetic testing Consent Form).<br>• The following assays will be performed to characterise the antimalarial treatment effects on *P. falciparum* K13 and 3D7 parasites using phenotypic and transcriptomic approaches (Cohorts 2 and 3). 1. Assess re-growth of parasites *ex vivo* by qPCR, flow cytometry and microscopy. 2. Perform *in vitro* drug sensitivity testing determining the 50% inhibitory concentrations (IC50s) and the percentage surviving parasites. 3. Perform

	molecular methods including whole genome sequencing; identification of molecular markers such as *gEXP-5*, *pfSBP-1*, *pfMGET*, *pfs25* etc; whole RNA sequence analysis to determine family-genes with differential expression between pre- and post-antimalarial treatment.<br>• The *in vivo* presence of dormant parasites will be investigated and characterised by determining the transcriptional molecular signature of dormant parasites and performing parasite lifecycle stage qRT-PCR on K13 and 3D7 parasites pre- and post-artesunate treatment (Cohorts 2 and 3).
**Population:**	Up to 27 subjects will be inoculated with K13 or 3D7 challenge agents in this study, in up to 3 cohorts. Subjects will be malaria naïve healthy adults (male and non-pregnant, non-lactating female), aged between 18-55 years old, who do not live alone from malaria inoculation day until Day 28±3 (End of Study), and will be contactable and available for the duration of the trial, and contactable up to 2 weeks following the End of Study visit.
**Phase:**	Phase 1/1b
**Number of sites enrolling subjects:**	Q-Pharm Pty Ltd<br>Levels 3 and 5, QIMR Berghofer CBCRC, 300C Herston Rd, Herston QLD 4006, Australia
**Description of study agents:**	**Malaria challenge agents:**<br><br>*P. falciparum* K13 blood stage challenge agent<br><br>A naturally occurring *P. falciparum* K13 artemisinin-resistant isolate was expanded *in vitro* to produce a blood stage *P. falciparum* K13 Master Cell Bank (MCB). Each K13 inoculum dose will be prepared aseptically from an aliquot of the *P. falciparum* K13 MCB. Each subject randomised to K13 will be inoculated intravenously with a dose of approximately 2 800 viable *P. falciparum* K13-infected RBCs in 2 mL saline for injection.<br><br>*P. falciparum* 3D7 blood stage challenge agent<br><br>The *P. falciparum* 3D7 MCB was produced from blood collected from a donor with clinical manifestation of malaria. Each 3D7 inoculum dose will be prepared aseptically from an aliquot of the *P.*

	falciparum 3D7 MCB. Each subject randomised to 3D7 will be inoculated intravenously with a dose of approximately 2 800 viable *P. falciparum* 3D7-infected RBCs in 2 mL saline for injection.<br><br>**Investigational medicinal products:**<br><br><u>Artesunate</u><br><br>Artesunate is a semi-synthetic derivative of artemisinin. Artesunate 50 mg tablets for oral administration will be used. Subjects will receive a single dose of artesunate tablets of approximately 2 mg/kg.<br><br><u>Piperaquine phosphate</u><br><br>If artesunate does not clear the parasites, subjects will be administered 960 mg piperaquine phosphate as a single oral dose. K13 subjects in Cohorts 2 and 3 will be treated with piperaquine phosphate on Day 11 AM or earlier at the Investigator's discretion.<br><br>**Antimalarial rescue medications:**<br><br><u>Malarone®</u><br><br>Malarone® will be administered to all subjects on Day 26±3 (Cohort 1), or Day 25±3 (Cohorts 2 and 3), or earlier at the Investigator's discretion. A treatment course consists of 4 tablets of Malarone® (atovaquone 250 mg, proguanil hydrochloride 100 mg) once daily, administered orally for 3 days.<br><br><u>Primacin™ (if required)</u><br><br>Subjects may receive a single oral dose of Primacin™ equivalent to 45 mg primaquine (6 tablets, each containing primaquine phosphate 13.2 mg, equivalent to 7.5 mg of primaquine) at the time of Malarone® treatment (Cohort 1) or on Day 23±3 or earlier at the Investigator's discretion (Cohorts 2 and 3), to ensure complete clearance of gametocytes.
**Study duration:**	Approximately 6 months.
**Subject duration:**	A screening period of up to 28 days plus the 28 day study (up to approximately 2 months).

SCHEMATIC OF STUDY DESIGN

Cohort 1:

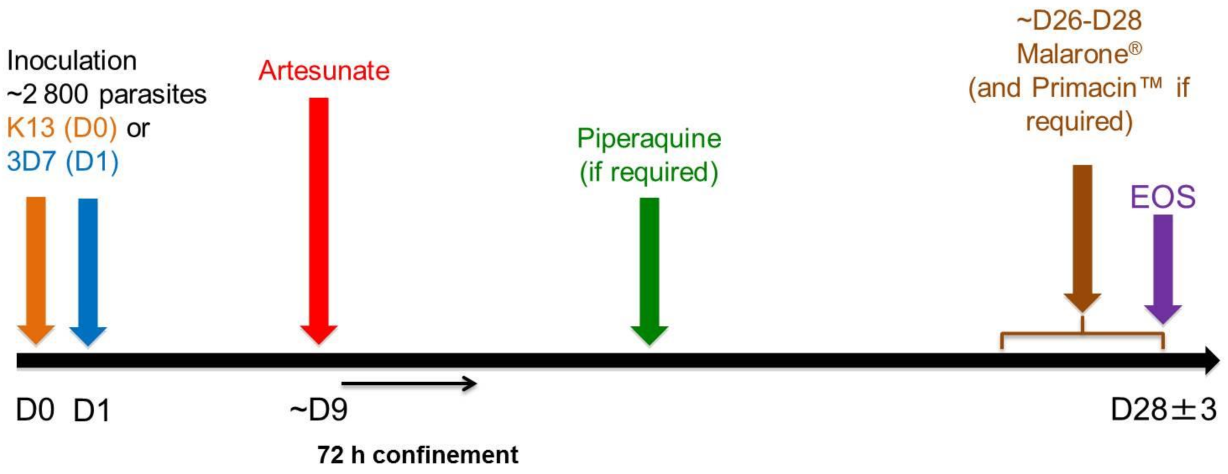

Cohorts 2 and 3:

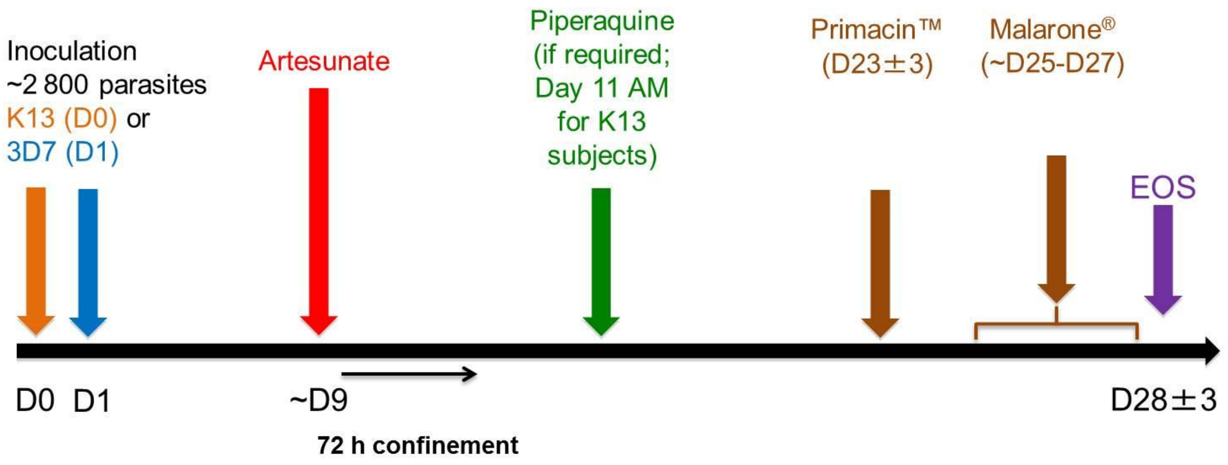

1 KEY ROLES

Principal Investigator	Prof. James McCarthy MBBS<br>Q-Pharm Pty Ltd (visiting Medical Officer) and<br>QIMR Berghofer Medical Research Institute (Clinical Tropical Medicine Group Leader)<br>Level 5, 300C Herston Rd<br>Herston QLD 4006, Australia<br>Tel: +61(0)7 3845 3647 or +61 (0)7 3845 3636<br>Mobile: +61 414424659<br>Email: j.mccarthy@uq.edu.au
Co-Investigator/s	Dr Paul Griffin MBBS FRACP FRCPA<br>Q-Pharm Pty Ltd<br>Level 5, 300C Herston Road<br>Herston QLD 4006, Australia<br>Tel: +61 (0)7 3845 3647<br>Fax: +61 (0)7 3845 3637<br>Email: paul.griffin@infectiousdiseasesqld.com.au<br><br>Dr Anand Odedra MBBS<br>Q-Pharm Pty Ltd (visiting Medical Officer) and<br>QIMR Berghofer Medical Research Institute<br>Level 5, 300C Herston Road<br>Herston QLD 4006, Australia<br>Tel: +61 (0)7 3845 3636<br>Email: Anand.Odedra@qimrberghofer.edu.au<br><br>Dr Bridget Barber MBBS, DTM&H, MPH, FRACP, PhD<br>Q-Pharm Pty Ltd (visiting Medical Officer) and<br>QIMR Berghofer Medical Research Institute<br>Level 5, 300C Herston Road<br>Herston QLD 4006, Australia<br>Tel: +61 424 737 153<br>Email: Bridget.Barber@qimrberghofer.edu.au
Trial Sponsor	QIMR Berghofer Medical Research Institute (QIMR Berghofer)<br>300 Herston Road<br>Herston QLD 4006, Australia<br>Tel: +61 (0)7 3362 0222
Sponsor Signatory	Prof. David Whiteman MBBS, PhD, FAFPHM

	Deputy Director<br>QIMR Berghofer Medical Research Institute<br>300 Herston Road<br>Herston QLD 4006, Australia<br>Tel: +61 (0)7 3362 0222<br>Email: David.Whiteman@qimrberghofer.edu.au
Funded by	Medicines for Malaria Venture (MMV)<br>ICC – Block G, 3rd floor<br>20, route de Pré-Bois<br>PO Box 1826<br>1215 Geneva 15<br>Switzerland<br>Tel: +41 22 555 0300<br>Fax: +41 22 555 0369
Independent Medical Monitor	Prof. Dennis Shanks<br>Australian Army Malaria Institute<br>Gallipoli Barracks<br>Enoggera QLD 4051, Australia<br>Tel: +61 (0)7 3332 4931<br>Email: Dennis.SHANKS@defence.gov.au
Institutional Ethics Committee	QIMR Berghofer Medical Research Institute Human Research Ethics Committee (QIMR Berghofer HREC; EC00278)<br>Locked Bag 2000, Royal Brisbane and Women's Hospital<br>Brisbane QLD 4029, Australia<br>Tel: +61 (0)7 3362 0117
Statistician	Dr Louise Marquart, PhD<br>QIMR Berghofer Medical Research Institute<br>300 Herston Road<br>Herston QLD 4006, Australia<br>Tel: +61 (0)7 3845 3555<br>Email: Louise.Marquart@qimrberghofer.edu.au
Sponsor's Monitors	Clinical Network Services (CNS) Pty Ltd<br>Level 4, 88 Jephson St<br>Toowong QLD 4066, Australia<br>Tel: +61 (0)7 3719 6000
Data Management	Clinical Network Services (CNS) Pty Ltd<br>Level 4, 88 Jephson St<br>Toowong QLD 4066, Australia<br>Tel: +61 (0)7 3719 6000

Clinical Study Centre	Q-Pharm Pty Ltd<br>Levels 3 and 5, QIMR Berghofer CBCRC<br>300C Herston Rd<br>Herston QLD 4006, Australia<br>Tel: +61 (0)7 3845 3636
Clinical Laboratories	Clinical laboratory measurements<br>Ms Marie-Claire Keogh<br>Sullivan Nicolaides Pathology Central Laboratory (SNP)<br>24 Hurworth Street<br>Bowen Hills QLD 4006, Australia<br>Tel: +61 (0)7 3377 8782<br>Email: marie-clarie_keogh@snp.com.au<br><br>Parasite quantification in blood samples<br>Ms Jane Gaydon<br>Queensland Paediatric Infectious Diseases Laboratory (Q-PID)<br>SASVRC, Level 8, Centre for Children's Health Research<br>62 Graham Street<br>South Brisbane QLD 4101, Australia<br>Tel: +61 (0)7 3069 7464<br>Email: j.gaydon@uq.edu.au<br><br>Investigational drug quantification in blood samples<br>Brett McWhinney<br>Analytical Chemistry Unit, Department of Chemical Pathology<br>Pathology Queensland<br>Level 3, Block 7, Royal Brisbane and Women's Hospital<br>Brisbane QLD 4029, Australia<br>Email: Brett.McWhinney@health.qld.gov.au
Protocol Writer	Dr Rebecca Watts PhD<br>Clinical Trials Project Manager<br>QIMR Berghofer Medical Research Institute<br>300 Herston Road<br>Herston QLD 4006, Australia<br>Tel: +61 (0)7 3845 3686<br>Email: Rebecca.Watts@qimrberghofer.edu.au

2 INTRODUCTION: BACKGROUND INFORMATION AND SCIENTIFIC RATIONALE

2.1 BACKGROUND INFORMATION

Malaria is one of the most important infectious diseases, which threatens half the world's population. In accordance with the latest estimates published by the World Health Organization (WHO), there were an estimated 212 million new cases of malaria worldwide and 429,000 deaths in 2015 [1]. The majority of these deaths were due to infection with *Plasmodium falciparum*.

Artemisinins are the most potent and rapidly acting antimalarial drugs available to date [2]. The WHO currently recommends artemisinin-based combination therapy as the first-line therapy in areas with high prevalence of resistance to other antimalarial drugs [1]. However, resistance to artemisinin-based combination therapies has been reported across the Greater Mekong Subregion in South-East Asia [3, 4]. Artemisinin resistance has been linked to mutations in the propeller domain of the *P. falciparum K13* gene [5]. Artemisinin-resistant isolates are not completely resistant to artemisinins, but instead have a slow parasite clearance phenotype compared to artemisinin-sensitive isolates. Therefore, parasite clearance in artemisinin-resistant severe malaria relies on the activity of the partner drug of the artemisinin combination therapy. Because of the concern for malaria drug resistance, new drugs with new mechanisms of actions are needed as well as efficient systems to determine the safety and clinical activity of these drugs.

The induced blood stage malaria (IBSM) model, whereby subjects are challenged with blood stage malaria parasites, is an attractive tool to test the activity of drugs and vaccines in non-immune subjects in a rapid and cost effective manner. By following the parasitaemia decay in subjects after drug or vaccine treatment by quantitative polymerase chain reaction (qPCR) [6], the antimalarial activity of a treatment can be determined.

The challenge agents used in the IBSM model are produced from blood stage parasite Master Cell Banks (MCBs). The MCBs have been generated following collection of blood from either donors who have been exposed to laboratory developed *Plasmodium* strains, or donors who have acquired malaria naturally while visiting malaria endemic countries.

The *P. falciparum* 3D7 challenge agent has been used in experimental studies in a total of 312 subjects in 25 IBSM clinical trials, 18 of which were successfully undertaken at QIMR Berghofer/Q-Pharm [7-32]. These trials have demonstrated the safety of the 3D7 challenge agent and the reliability of the approach in terms of infectivity and parasite growth rate. Adverse events (AEs) in these studies were consistent with the symptoms of early malaria infection including headache, fever, fatigue, malaise, myalgia, arthralgia and gastrointestinal upset. The only serious adverse events (SAEs) likely to be related to the 3D7 challenge agent were asymptomatic liver function derangements that resolved by the end of the studies (Section 2.3.1) [33]. *P. falciparum*

3D7 is sensitive to artemisinins and standard antimalarial drugs, such as chloroquine, Riamet®, and Malarone®.

A new parasite MCB has recently been generated from a *P. falciparum* Cam3.II^{R539T} (K13) artemisinin-resistant malaria parasite (referred to as K13), which was isolated from a patient in Cambodia with natural malaria infection. The K13 MCB is resistant to dihydroartemisinin, chloroquine, lumefantrine, mefloquine, and quinine, as determined by *in vitro* antimalarial drug susceptibility tests [34]. Conversely, the K13 MCB is sensitive to 4 antimalarial drugs, including piperaquine phosphate and atovaquone (a component of Malarone®), which can be used in the IBSM model to rescue subjects challenged with K13. An IBSM pilot study (QP16C14/P2286) is currently ongoing in which 2 subjects were challenged with ~2 800 viable *P. falciparum* K13 parasite-infected red blood cells (RBCs) and treated with approximately 2 mg/kg artesunate (a semi-synthetic derivative of artemisinin) on Day 9 [35]. The pilot study results to date show that the *P. falciparum* K13 challenge agent is safe, well tolerated, and infective (refer to the *P. falciparum* K13 Investigator's Brochure for further details [34]). The availability of a *P. falciparum* artemisinin-resistant challenge agent in the IBSM model will allow investigation of the activity of novel antimalarial drug candidates in clearance of artemisinin-resistant *P. falciparum*, which is a growing problem in South-East Asia and threatens to spread across the world.

2.2 RATIONALE

This study aims to characterise and compare the parasitological response of the *P. falciparum* K13 (artemisinin-resistant) and 3D7 (artemisinin-sensitive) isolates to the artemisinin antimalarial drug artesunate in healthy subjects inoculated in the IBSM model. We hypothesise that artesunate will clear parasitaemia from subjects challenged with K13 at a 30% slower rate compared to subjects challenged with 3D7.

Once the *P. falciparum* K13 isolate is properly characterised in the IBSM model with respect to resistance to an artemisinin drug, it can then be used in future studies to investigate the activity of novel, investigational antimalarial drugs in clearance of artemisinin-resistant *P. falciparum*. This study will also continue to assess the safety and tolerability of the K13 isolate in healthy subjects.

The 3D7 isolate was selected as the comparator because it is a artemisinin-sensitive *P. falciparum* isolate, is safe and well tolerated, and has been extensively characterised in 312 subjects in the IBSM model [33]. Future studies may compare the clearance profile of new antimalarial drugs in clinical development following inoculation with K13 and 3D7 isolates. This comparison would provide insight into the antimalarial drug's activity against both artemisinin-resistant and artemisinin-sensitive parasites.

Subjects randomised to receive the K13 challenge agent will be inoculated on Day 0 while subjects randomised to receive the 3D7 challenge agent will be inoculated on Day 1. These inoculation days were chosen based on the isolate growth rates, to give a similar parasitaemia and parasite lifecycle stage at the day of treatment. Preliminary data from 2 subjects in the K13 pilot study and 127 subjects inoculated with 3D7 in previous studies [35, 36], were used to graph the parasite growth rate from inoculation to antimalarial drug treatment. This indicated that the K13 isolate at 9 days post-inoculation and the 3D7 isolate at 8 days post-inoculation were synchronised and close to the parasitaemia threshold for treatment. Therefore, K13 subjects will be inoculated approximately 24 hours earlier than 3D7 subjects to reach the artesunate treatment threshold on the same day.

Artesunate was chosen as the artemisinin drug since it was available as a single agent drug and previous studies have studied the parasite clearance of artemisinin-resistant *P. falciparum* in response to artesunate monotherapy [3, 37]. A study in Cambodia, where artemisinin resistance is a problem, showed that an artesunate dose of 2 mg/kg of body weight decreased *P. falciparum* parasites by 80% within 24 hours (Fig. 1) [37]. The currently ongoing K13 pilot study (QP16C14/P2286) with 2 subjects found that approximately 2 mg/kg artesunate on Day 9 initially decreased parasitaemia, with recrudescence observed on Day 11 (Fig. 2). Therefore, this artesunate dose should allow the parasite clearance profile for both K13 and 3D7 to be properly characterised and compared.

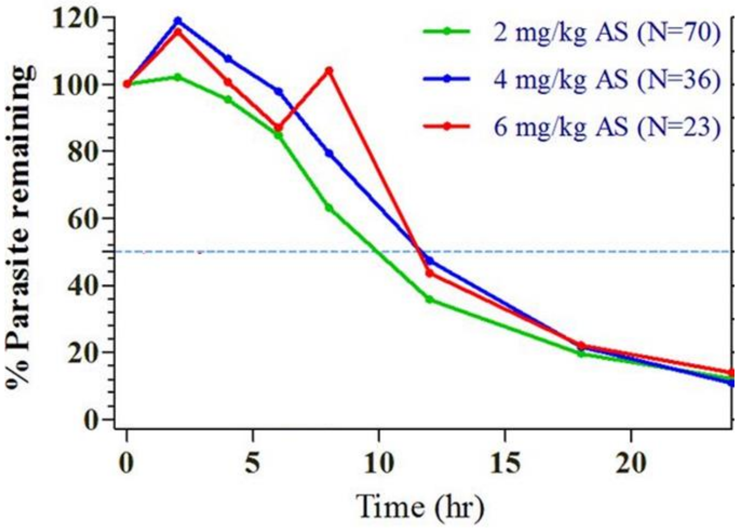

Figure 1: Parasite clearance as mean percentage of baseline values over 24 hours with different doses of artesunate. Modified from Saunders *et al.* 2012 [37].

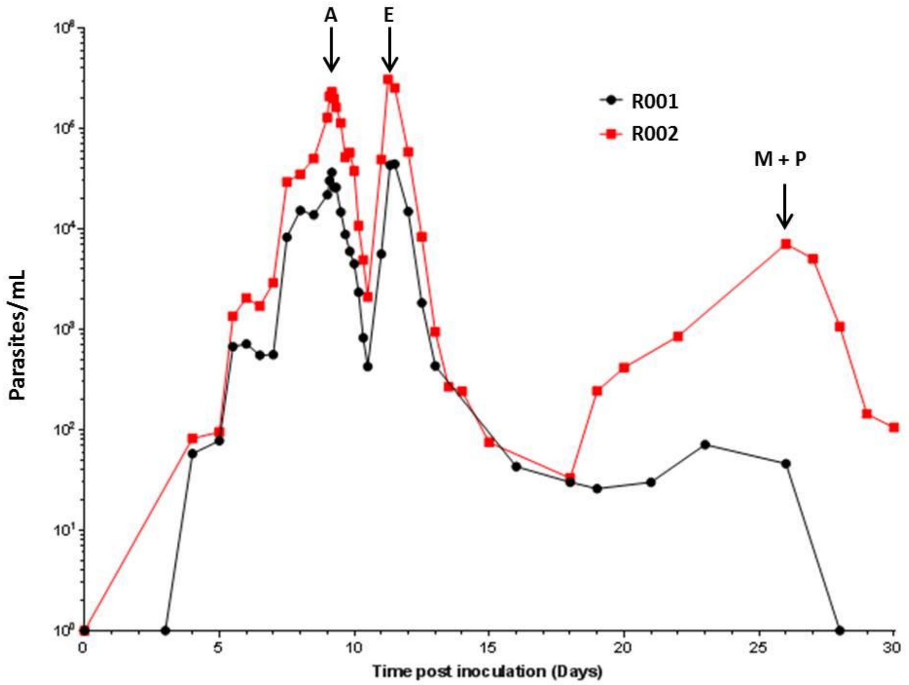

Figure 2: Parasitaemia in 2 healthy subjects following inoculation with *P. falciparum* K13. Vertical arrows represent the time of dosing of artesunate (A), Eurartesim® (E; piperaquine tetraphosphate/dihydroartemisinin), first dose of Malarone® (M), and Primacin™ (P).

2.3 POTENTIAL RISKS AND BENEFITS

2.3.1 KNOWN POTENTIAL RISKS

Potential risks have been identified through review of previous clinical studies conducted to date using the IBSM model with *P. falciparum* isolates, as well as review of the literature. *P. falciparum* 3D7 has been used to challenge 312 healthy subjects in 25 IBSM Phase 1 clinical trials, 18 of which were successfully undertaken at QIMR Berghofer/Q-Pharm [7-32]. *P. falciparum* K13 has been used to inoculate 2 healthy subjects in an IBSM pilot study which is currently ongoing [35]. Refer to the *P. falciparum* 3D7 and K13 Investigator's Brochures for further details [33, 34].

Risk management of blood borne infections

In this study, *P. falciparum* 3D7 or K13 challenge agents will be used. These contain a very small amount of donor blood. However, the risk of infection from a possible blood borne virus from the blood transfused in this study is expected to be very low for a number of reasons. Firstly, the donors were screened and tested negative for presence of active blood borne infections. Secondly, white blood cells were removed from the donor blood by the Australian

Red Cross Blood Service (Blood Service), which lowers the risk of transfusion transmitted infections. Thirdly, the volume of blood used in the IBSM model for transmitting malaria is many thousands times smaller than in a transfused unit, thus reducing the risk of infection. As part of the safety monitoring, all subjects will have serum stored for testing of blood borne virus infections before and after the study (Section 7.2.1). To date, no blood borne infections have been reported in any of the 312 subjects who have received the *P. falciparum* 3D7 inoculum in IBSM studies.

Risk management of reaction to the blood sample

The risk of developing RBC alloantibodies in this study is considered extremely low since the donor blood used to produce the inocula was blood group O Rh(D) Negative. People with this blood group are generally considered “universal donors”, as recipients of their blood have minimal risk of developing RBC alloantibodies when given much larger volumes of blood than is used in the IBSM model. However, it is possible that subjects could suffer a transfusion reaction after they receive the inoculum, or develop alloantibodies to the donor RBCs that may make blood transfusion more difficult in the future. To date, one subject has developed an antibody response to a minor Rh antigen (anti-E antibody) following inoculation with *P. falciparum* 3D7 [33]. However, there was no laboratory evidence to indicate that the specific Rh phenotype of the donor RBCs in the inoculum provoked production of this allo anti-E antibody. Subjects will be monitored for signs and symptoms in the period immediately after administration of the inoculum to further assess the risk of the inoculum in causing transfusion reaction. Subjects will also be tested for RBC alloantibodies at screening and at the end of the study as part of their safety monitoring (Section 7.2.1).

Women of childbearing potential (WOCBP) have a small additional risk of developing RBC alloantibodies that could cause problems during pregnancy. WOCBP have participated in several IBSM trials with *P. falciparum* isolate 3D7 with no known issues to date. Specific strict contraception requirements will be requested for this population during the study (Section 5.1). Including WOCBP in the trial enhances the generalisability of the study results.

Risk management of malaria infection

The number of blood stage parasites used to infect the subjects in this study (~2 800) is much lower than the parasitaemia reached after the bite of a single malaria-infected mosquito, where approximately 30 000 parasites are released into the blood when they break out of a single infected liver cell [38]. In this study, parasite growth as well as malaria symptoms will be closely monitored in subjects following administration of the challenge agent. The threshold for commencement of antimalarial drug treatment defined for this study (Section 6.1.5) has been selected since it is below the point at which advanced clinical symptoms of malaria are likely to occur, as observed in the previous 25 clinical trials performed with the 3D7 challenge agent.

The K13 parasite used in this study is artemisinin-resistant, and therefore it is expected to show a slower clearance in response to artesunate treatment. If artesunate fails to clear parasitaemia from the blood of K13 or 3D7 subjects or there is recrudescence, they will be administered a drug known to be active against these isolates (piperaquine phosphate). All subjects will also be treated with the registered antimalarial drug Malarone® to ensure parasite clearance. If subjects comply with the curative antimalarial regimen as directed by the Investigator, there is no serious risk of clinical malaria.

Risk management of liver function derangements

Transient, asymptomatic liver function test (LFT) derangements including rare cases of alanine aminotransferase/aspartate aminotransferase elevations >10 x ULN (upper limit of normal) have been reported in several subjects in IBSM studies [33, 39]. However, no changes in bilirubin were reported except for in one subject with unappreciated pre-existing liver disease [33, 39]. The LFT derangements did not require treatment, and resolved by the end of the studies. A few cases of the LFT elevations were considered as SAEs by two Pharma sponsors due to internal processes for SAE notifications. Following an independent review involving drug-induced liver injury experts, it was found that these LFT elevations are most likely a direct consequence of the malaria infection rather than reflecting a direct drug-induced liver injury caused by an investigational antimalarial drug. As a precaution all subjects in this study will undergo regular safety monitoring to assess for asymptomatic LFT abnormalities. Subjects are required to reduce intake of possibly hepatotoxic substances during the course of the study including alcohol and paracetamol.

Risk management of cardiac adverse events

To our knowledge, 3 cardiac SAEs have been reported in healthy subjects in the Netherlands participating in malaria challenge studies using sporozoites i.e. direct feeds by infected mosquitoes rather than IBSM infection. Refer to the *P. falciparum* 3D7 and K13 Investigator's Brochures for further details [33, 34]. No cardiac SAEs caused by the inoculum have been reported in IBSM studies. As a precaution, people at significant risk of cardiovascular disease will be excluded from participating in IBSM studies, and regular safety monitoring including physical examination and electrocardiogram (ECG) recordings will take place for all subjects.

Artesunate risks

Possible adverse events arising from the use of artesunate can be found in the WHO Public Assessment Report (2011) for artesunate 50 mg tablets [40]. These include mild electrocardiogram changes (corrected QT interval and PR interval increase), atrial extrasystoles, non-specific T-wave changes, gastrointestinal disturbances (nausea, vomiting, abdominal pain, diarrhoea), and dizziness. Subjects will be counselled that certain adverse events, for example dizziness, may impact on the performance of skilled tasks such as driving. Delayed drops in haemoglobin have been observed in patients administered artesunate for treatment of clinical

malaria; these drops have not been clinically significant. Subjects will be confined for 72 hours after administration of artesunate to monitor for any adverse events.

Piperaquine phosphate risks

Piperaquine phosphate, as a combination antimalarial with dihydroartemisinin, is well tolerated in both adults and children, with the main adverse events reported to be gastrointestinal disturbances such as diarrhoea [41, 42]. Studies with dihydroartemisinin/piperaquine demonstrated corrected QT (QTc) interval prolongation during treatment [43, 44]. Very few individual patients experienced a prolongation that could be regarded as clinically significant (>60 msec); of note, the QTc prolongation induced by piperaquine phosphate has not been reported to be associated with clinically relevant cardiovascular events, suggesting a proarrhythmic effect. Therefore, although statistically significant, the QTc prolongation observed following piperaquine phosphate therapy is unlikely to be of clinical concern. European regulatory authorities have advised that Eurartesim® (dihydroartemisinin/piperaquine) not be administered with food to reduce piperaquine peak concentrations, and caution that prior and post-electrocardiographic monitoring be undertaken, and avoidance of concomitant recent exposure to drugs at risk of QTc prolongation [16, 44, 45]. Therefore, during this study, subjects will receive piperaquine phosphate under fasting conditions and will have ECGs recorded before and after treatment.

Piperaquine phosphate was clinically well tolerated in previous IBSM studies using this drug [26, 27, 32]. In these studies piperaquine phosphate treatment demonstrated a robust safety profile in doses up to 960 mg when used for the treatment of uncomplicated *P. falciparum* malaria infection.

See the piperaquine phosphate Investigator’s Brochure for more details [46].

Rescue treatment risks: Malarone® and Primacin™

Malarone® and Primacin™ risks are detailed in their respective approved manufacturer’s prescribing information [47-50] (Appendix 1). Primacin™ may cause severe haemolytic anaemia in subjects with glucose-6-phosphate dehydrogenase (G6PD) deficiency. Subjects will be tested for G6PD deficiency at screening to ensure the safety of Primacin™. The G6PD status will determine how the subject is treated with Primacin™.

Risk management of direct mosquito feeding

Subjects will be continuously monitored during the direct feeding of mosquitoes. If a subject develops severe skin sensitivity or experiences discomfort during the mosquito feed process, they will be given the option to withdraw from this process. Prevention and management of possible skin reactions/irritation induced by mosquito bites will also be provided as outlined below.

a. Pre-existing hypersensitivity to mosquito bites

Subjects with known severe reaction to mosquito bites other than local itching and redness will be excluded as per exclusion criterion number 40. As hypersensitivity is generally due to sensitisation to species-specific salivary proteins and this species is not endemic in this region the background rate of hypersensitivity is expected to be low.

b. Treatment

In the event that a subject experiences symptoms attributable to a reaction to mosquito bites they will be offered a moderately potent topical glucocorticoid cream, 0.1 mometasone, to be applied twice daily for up to 10 days. If this is not sufficient or the subject is intolerant to this class of treatment, a non-sedating, non-impairing H1 antihistamine cetirizine which has been shown to relieve itching in the early phase allergic reaction and reduce late phase reactions including swelling, redness and induration will be provided.

Risks of genetic testing (optional genetic research)

Subjects may optionally consent to provide a blood sample for genetic research (see Section 7.2.2 for details). Genetic testing may raise important issues because subjects may learn from their result that they have or are at risk of inherited diseases or disorders. Therefore, results from genetic research might affect subjects and their families emotionally. However, subjects can choose not to be informed of their results. Subjects can request to speak to a genetic counsellor, which will be organised with no cost to the subject.

Statutory or contractual duties may require subjects to disclose results of genetic tests or analysis to third parties (e.g., insurance companies), particularly where results provide information about their future health. If a subject chooses to obtain their genetic test results they may then be obliged to disclose this on any future application for insurance should it be requested. However, if a subject chooses not to receive their test results, then future requests for insurance may not be affected by participating in this research. There is also a low risk of breach of confidentiality which may then impact insurability. Only authorised people will have access to the subjects' results.

To our knowledge, the genetic tests we are intending to do in this study have no implications on the health or insurance of the subjects.

General risk management

The risk to subjects in this study will also be minimised as follows:

- Adherence to the inclusion/exclusion criteria to ensure that only subjects who are not at any perceived risk are enrolled in the study.

- Close clinical and laboratory monitoring to ensure the safety and well-being of the subjects.
- Subjects will be prescribed curative therapy for malaria (Malarone® with the addition of Primacin™ if required) for final clearance during or at the end of the study.
- The total volume of blood drawn from each subject enrolled into the study (including optional exploratory research sampling) will not exceed a standard unit of blood (approximately 450 mL) over any 30 day period.
- In the rare event that a subject requires hospitalisation at the request of the Principal Investigator or their representative, this will be done at the Infectious Diseases Unit, Royal Brisbane and Women's Hospital. Emergency procedures are in place at the Q-Pharm clinics for dealing with any unforeseen clinical emergencies which may arise.

With these safety provisions, the overall risk to the subjects in the study is considered to be minimal and acceptable, and the potential of future improved treatment for malaria is considered to outweigh these potential risks.

2.3.2 KNOWN POTENTIAL BENEFITS

There are no known direct benefits to the subjects in this study. There may be a benefit to the subjects from the results of the screening tests and procedures (blood tests, physical examination and ECGs).

Subjects may optionally consent to provide an additional blood sample during the study which will be used for genetic research (Section 7.2.2). There may be a benefit from receiving the genetic results e.g. information about risk of an inherited condition.

3 OBJECTIVES AND PURPOSE

Primary:

- To compare the parasite clearance profiles of the *P. falciparum* K13 and 3D7 isolates after administration of artesunate.

- To determine the safety and tolerability associated with infection of healthy subjects with the *P. falciparum* K13 and 3D7 isolates.

Exploratory:

- To characterise the pharmacokinetic profile of artesunate and its active metabolite, dihydroartemisinin, in *P. falciparum* K13 and 3D7 infected subjects.

- To characterise the pharmacokinetic/pharmacodynamic relationship between artesunate and dihydroartemisinin pharmacokinetics and parasite clearance in *P. falciparum* K13 and 3D7 infected subjects.
- To determine if gametocytes appear in the blood of subjects infected with *P. falciparum* K13 or 3D7.
- To determine if K13 and 3D7 gametocytes, if present, are infective to mosquitoes (may be performed in selected cohorts at the Investigator's discretion).
- To investigate and identify any possible associations between subjects genetic polymorphisms and liver function (or phenotype) following infection with *P. falciparum* K13 or 3D7 (optional and requires consent via genetic testing Consent Form).
- To characterise the antimalarial treatment effects on *P. falciparum* K13 and 3D7 parasites using phenotypic and transcriptomic approaches (Cohorts 2 and 3).
- To investigate the *in vivo* presence of dormant parasites (Cohorts 2 and 3).

4 STUDY DESIGN AND ENDPOINTS

4.1 DESCRIPTION OF THE STUDY DESIGN

This Phase 1/1b, single-centre study will compare the parasitological response of the *P. falciparum* isolates K13 (artemisinin-resistant) and 3D7 (artemisinin-sensitive) following a single dose of artesunate in healthy subjects using the IBSM model. The study will be conducted in up to 27 subjects, in up to 3 cohorts. Up to 18 subjects will receive the K13 challenge agent and up to 9 subjects will receive the 3D7 challenge agent, to achieve a ratio of 2:1 over the whole study (see Section 10.5 for sample size information and 10.6.1). Cohort 1 planned to recruit 9 subjects, randomised to receive either the K13 or 3D7 challenge agent, in a 2:1 ratio. However, only 6 subjects received the challenge agents in Cohort 1 (3 K13 and 3 3D7). Up to 21 subjects distributed over Cohorts 2 and 3 will receive either the K13 or 3D7 challenge agents, to reach up to 27 subjects in total.

A review of the safety, tolerability, and parasitaemia data from the preceding cohort will be conducted by the Safety Review Team (SRT) prior to inoculation of the next cohort. Data from at least 3 weeks post-inoculation will be required for each review (Section 10.4.7). The SRT meeting conducted after Cohort 2 inoculation will have analysis available for the parasitaemia data from both Cohort 1 and 2 to determine whether a third cohort is needed to complete the primary objective of comparing artesunate parasite clearance profiles.

Subjects will be consenting and eligible healthy adults (male and non-pregnant, non-lactating female), aged between 18-55 years old. Subjects randomised to receive the K13 challenge agent will be inoculated on Day 0 with approximately 2 800 viable *P. falciparum* K13 parasite-infected RBCs administered intravenously. Subjects randomised to receive the 3D7 challenge agent will be inoculated on Day 1 with approximately 2 800 viable *P. falciparum* 3D7 parasite-infected RBCs administered intravenously.

NOTE: when "inoculation day" is referred to in this protocol it will mean Day 0 for K13 subjects and Day 1 for 3D7 subjects. These inoculation days were chosen based on the growth rates of the isolates, to give a similar parasitaemia and parasite lifecycle stage at the day of artesunate treatment (Section 2.2).

On an outpatient basis, subjects will be monitored daily via phone and then will attend the clinical unit daily from 4 days post-inoculation – Day 4 for K13 subjects and Day 5 for 3D7 subjects – for blood sampling to measure parasitaemia via qPCR targeting the *P. falciparum* 18S rRNA gene (referred to as malaria 18S qPCR). Once parasitaemia is detected by qPCR, subjects will attend the clinical unit twice daily, morning (AM) and evening (PM), for blood sampling to measure parasitaemia, to monitor symptoms and signs of malaria, and to record adverse events. Microscopic examination (thin and thick films) for evidence of parasitaemia may be conducted at the Investigator's discretion.

The threshold for commencement of artesunate treatment will occur when all subjects reach a parasitaemia of ≥5 000 parasites/mL. If the parasitaemia of any subject is ≥5 000 parasites/mL, and is accompanied by a malaria clinical score >6 (Section 7.1.1, adapted from [51]) before all subjects have reached the treatment threshold, then treatment of that subject will begin within a 24 hour period. Subjects may also receive artesunate treatment at any time based on parasitaemia and/or clinical symptoms at the Investigator's discretion. On the day designated for artesunate treatment, subjects will be admitted to the clinical unit (approximately Day 9), administered a single oral dose of approximately 2 mg/kg artesunate (Appendix 2), and confined for 72 hours to monitor for safety and tolerability of therapy and to ensure adequate clinical and parasitological response. Blood samples will be collected pre-dose and following artesunate treatment to measure plasma concentrations of artesunate and its active metabolite dihydroartemisinin (Section 7.2.2). Wherever possible, this PK sampling will coincide with post-dose blood collection for monitoring of parasitaemia.

After discharge from the clinical unit, subjects will be followed up on an out-patient basis for monitoring of safety and parasite clearance. Subjects may also be evaluated for the presence of sexual parasite stages (gametocytes) and other parasite lifecycle stages in the blood at the Investigator's discretion, by parasite lifecycle stage reverse transcription qPCR (qRT-PCR), targeting genes such as the female gametocyte-specific transcript *pfs25*, the male gametocyte-specific transcript *pfMGET*, and/or the ring-stage transcript *pfSBP-1* as appropriate (Section 7.2.2). Infectivity of parasites to *Anopheles* mosquitoes may be investigated by malaria

transmission assays, at the Investigator's discretion using parasite lifecycle stage qRT-PCR to determine probability of infectivity. For Cohorts 1 and 2, direct feeding assays (DFAs) at one time-point per subject, and membrane feeding assays (MFAs) at one time-point per subject, may be performed from approximately 10 days post-artesunate treatment. For Cohort 3, DFAs and MFAs may be performed at up to two time-points each per subject. The second DFA will be optional for the subjects as outlined in the informed Consent Form. The timing of the Cohort 3 DFAs and MFAs for each subject will be at the Investigator's discretion. Microscopic examination for confirmation of gametocytaemia may be conducted at the Investigator's discretion.

It is anticipated that artesunate treatment will reduce K13 parasitaemia, although at a slower rate than for the 3D7 artemisinin-sensitive isolate. If artesunate does not clear parasitaemia, subjects will be administered a drug known to be active against both K13 and 3D7 isolates (piperaquine phosphate as a single dose of 960 mg).

Cohort 1:

For Cohort 1, piperaquine phosphate treatment will occur on an individual subject basis in the event of unsatisfactory parasite clearance, defined as 2 consecutive malaria 18S qPCR time-points showing a decrease in parasitaemia of less than 20% of baseline (i.e., the parasitaemia before drug treatment) by 72 hours post-artesunate treatment. Piperaquine phosphate treatment will also occur if recrudescence of parasitaemia occurs after artesunate treatment, defined as ≥5 000 blood stage parasites/mL and a 2-fold increase within 48 hours, or a malaria clinical score >6. Piperaquine phosphate treatment can also be undertaken at the Investigator's discretion.

All Cohort 1 subjects will receive compulsory rescue treatment with Malarone® (atovaquone/proguanil hydrochloride; 3 day course) on Day 26±3 or earlier at the Investigator's discretion. Subjects may also be treated with a single oral dose of Primacin™ (equivalent to 45 mg primaquine) at the time of Malarone® treatment if gametocytaemia is suspected from parasite lifecycle stage qRT-PCR or by the presence of stable low level parasitaemia, or at the Investigator's discretion, to ensure complete clearance of gametocytes.

Cohorts 2 and 3:

For Cohorts 2 and 3, piperaquine phosphate treatment will occur for subjects inoculated with the 3D7 challenge agent as outlined above for Cohort 1. However, K13 subjects in Cohorts 2 and 3 will be treated with piperaquine phosphate on Day 11 AM or earlier at the Investigator's discretion. This is to avoid the high parasitaemias seen in K13 subjects in Cohort 1 on Day 11 PM prior to piperaquine phosphate treatment.

All Cohort 2 and 3 subjects will be treated with a single oral dose of 45 mg primaquine (Primacin™) on Day 23±3 or earlier at the Investigator's discretion. This is to ensure complete clearance of gametocytes before Day 28±3 (End of Study). All subjects will receive compulsory

rescue treatment with Malarone® (atovaquone/proguanil hydrochloride; 3 day course) on Day 25±3 or earlier at the Investigator's discretion.

Cohorts 2 and 3 may also include additional blood samples for drug response research and dormancy detection research at the Investigator's discretion (Sections 7.2.2 and 7.3).

<u>All Cohorts:</u>

Follow-up for safety assessments will be performed on Day 28±3 (End of Study). The overall duration of participation will therefore be ~4 weeks from the time of inoculation. Subjects are required to be contactable up to 2 weeks following the End of Study (EOS) visit. The SRT will review the safety and tolerability data from the study after completion of the last cohort.

Subjects may optionally consent to provide an additional blood sample (approximately 4 mL) during the study which will be used for genetic research to investigate any associations between genetic polymorphisms and abnormal liver function (or phenotype) after *P. falciparum* K13 or 3D7 inoculation. The purpose of this research is to screen for liver genetic polymorphisms which are associated with possible liver changes that might take place during the study. Examples of genetic variations that may be screened for include:

- Variations in the *UGT1A1* gene which cause Gilbert syndrome. Gilbert syndrome results in higher than normal blood levels of bilirubin (a breakdown product of RBCs), and normally has no serious health consequences.
- Variations in the promoter region of haem oxidase-1 gene (*HMOX-1*).
- Variations in the promoter region of tumor necrosis factor alpha gene (*TNFA*).
- Variations in currently unknown genes or regulatory regions which may be discovered in the future that could have an impact on liver function.

It is important for this study that we know if any changes in liver function tests in subjects after *P. falciparum* infection are due to inherited genetic variations that affect how the liver responds to malaria or how the liver handles antimalarial drugs. Subjects will be asked to sign an additional informed Consent Form if they agree to participate in the genetic research. Subjects can choose whether or not they are informed of their test results. If a subject decides to see their test results, the Principal Investigator, or clinical unit staff authorised by the Principal Investigator, will discuss the results with the subject. During this discussion subjects can also request to speak to a genetic counsellor. If requested, a genetic counsellor will be organised with no cost to the subject.

Subjects who are enrolled in this study may also enrol in optional, exploratory malaria research studies, outlined in a separate protocol and informed Consent Form, that require separate consent for subjects agreeing to participate in any of these (Section 7.2.2).

In Cohort 3, up to two subjects may also be recruited for a parallel, optional, observational exploratory study “Advanced medical imaging in subpatent malaria: a second pilot study” which is outlined in a separate protocol and informed Consent Form (Section 7.2.2).

4.2 STUDY ENDPOINTS

4.2.1 PRIMARY ENDPOINT

- The parasite clearance profiles of *P. falciparum* K13 and 3D7 after administration of artesunate will be characterised by the parasite reduction ratio and parasite clearance $t_{1/2}$. A t-test will be used to compare the slope of the clearance curves for K13 and 3D7 isolates.
- The safety and tolerability of *P. falciparum* K13 and 3D7 infection will be evaluated from adverse event and serious adverse event recording (including severity and causality), physical examinations, vital signs, clinical biochemistry, haematology, and urinalysis.

4.2.2 SECONDARY ENDPOINTS

Not applicable.

4.2.3 EXPLORATORY ENDPOINTS

<u>Exploratory:</u>

- The following pharmacokinetic parameters will be estimated for artesunate and dihydroartemisinin: AUC_{last}, AUC_{inf}, C_{max}, T_{max} and $t_{1/2}$.
- A pharmacokinetic/pharmacodynamic model describing the relationship between artesunate and dihydroartemisinin plasma concentration and the effect on parasitaemia measured by qPCR will be developed.
- The presence of gametocytes in subjects will be determined by parasite lifecycle stage qRT-PCR until approximately Day 28±3.
- Infectivity of gametocytes (if present) to mosquitoes will be reported as prevalence of infection (percentage of oocyst positive mosquitoes) (may be performed in selected cohorts at the Investigator’s discretion).
- The absence or presence of a genetic variation that may have a link to changes in liver function (or phenotype) following infection with *P. falciparum* (optional and requires consent via genetic testing Consent Form).

- The following assays will be performed to characterise the antimalarial treatment effects on *P. falciparum* K13 and 3D7 parasites using phenotypic and transcriptomic approaches (Cohorts 2 and 3). 1. Assess re-growth of parasites *ex vivo* by qPCR, flow cytometry and microscopy. 2. Perform *in vitro* drug sensitivity testing determining the 50% inhibitory concentrations (IC50s) and the percentage surviving parasites. 3. Perform molecular methods including whole genome sequencing; identification of molecular markers such as *gEXP-5*, *pfSBP-1*, *pfMGET*, *pfs25* etc; whole RNA sequence analysis to determine family-genes with differential expression between pre- and post-antimalarial treatment.
- The *in vivo* presence of dormant parasites will be investigated and characterised by determining the transcriptional molecular signature of dormant parasites and performing parasite lifecycle stage qRT-PCR on K13 and 3D7 parasites pre- and post-artesunate treatment (Cohorts 2 and 3).

Results of these exploratory endpoints will not be presented in the Clinical Study Report (CSR) but will be presented in separate reports or publications.

5 STUDY ENROLLMENT AND WITHDRAWAL

5.1 SUBJECT INCLUSION CRITERIA

Subjects eligible for inclusion in this study must fulfil **all** of the following criteria:

Demography

1. Adult (male and non-pregnant, non-lactating female) subjects between 18 and 55 years of age inclusive, who do not live alone (from inoculation day until at least the end of the Malarone® treatment) and will be contactable and available for the duration of the trial and contactable up to 2 weeks following the End of Study visit (approximately 6 weeks).

2. Body weight minimum 50 kg, body mass index between 18 and 32 kg/m2, inclusive.

Health status

3. Certified as healthy by a comprehensive clinical assessment (detailed medical history and complete physical examination).

4. Vital signs after 5 minutes resting in supine position:

   90 mmHg ≤ systolic blood pressure (SBP) ≤ 140 mmHg,

   50 mmHg ≤ diastolic blood pressure (DBP) ≤ 90 mmHg,

   40 bpm ≤ heart rate (HR) ≤ 100 bpm.

5. Normal standard 12-lead ECG after 5 minutes resting in supine position, QTcF≤450 msec with absence of second or third degree atrioventricular block or abnormal T wave morphology at screening and at pre-inoculation on inoculation day.

6. Laboratory parameters within the normal range, unless the Investigator considers an abnormality to be clinically irrelevant for healthy subjects enrolled in this clinical investigation in accordance with approved clinically acceptable laboratory ranges documented prior to study start. More specifically, serum creatinine, hepatic transaminase enzymes (aspartate aminotransferase, alanine aminotransferase), and total bilirubin (unless the subject has documented Gilbert syndrome) should not exceed the approved acceptable ranges and haemoglobin must be equal or higher than the lower limit of the normal range.

7. Heterosexual women of childbearing potential should be surgically sterile or using an insertable, injectable, transdermal or combination oral contraceptive approved by the TGA combined with a barrier contraceptive for the duration of the study, and have negative results on a serum pregnancy test done before inoculation. Abstinent, heterosexual female subjects must agree to start a double method if they start a sexual relationship during the study. Adequate contraception does not apply to subjects of childbearing potential with same sex partners (abstinence from penile-vaginal intercourse), when this is their preferred and usual lifestyle. Female subjects with same sex partners must not be planning *in vitro* fertilisation within the required contraception period.

   Women of non-childbearing potential who will not require contraception during the study are defined as: post-menopausal (spontaneous amenorrhoea for ≥ 12 months, or spontaneous amenorrhoea for 6-12 months and follicle-stimulating hormone (FSH) ≥ 40 IU/mL; either should be together with the absence of oral contraceptive use for > 12 months).

Regulations

8. Having given written informed consent prior to undertaking any study-related procedure.

5.2 SUBJECT EXCLUSION CRITERIA

Subjects fulfilling **any** of the following criteria will not be eligible for inclusion in this study:

Medical history and clinical status

1. Any history of malaria or participation in a previous malaria challenge study.

2. Must not have travelled to or lived (>2 weeks) in a malaria-endemic region during the past 12 months or planned travel to a malaria-endemic region during the course of the study (for

endemic regions see https://map.ox.ac.uk/country-profiles/#!/). Bali is not considered a malaria-endemic region.

3. Participation in any investigational product study within the 12 weeks preceding the study (i.e. there should be 12 weeks between the last dose of a previous investigational product and malaria parasite inoculation, at the Investigator's discretion).

4. Has evidence of increased cardiovascular disease risk (defined as >10%, 5 year risk for those greater than 35 years of age, as determined by the Australian Absolute Cardiovascular Disease Risk Calculator (http://www.cvdcheck.org.au/)). Risk factors include sex, age, systolic blood pressure (mm/Hg), smoking status, total and HDL cholesterol (mmol/L), and reported diabetes status.

5. Symptomatic postural hypotension at screening, irrespective of the decrease in blood pressure, or asymptomatic postural hypotension defined as a decrease in systolic blood pressure ≥20 mmHg within 2-3 minutes when changing from supine to standing position.

6. History of splenectomy.

7. Presence or history of drug hypersensitivity, or allergic disease diagnosed by an allergist/immunologist and/or treated by a physician for allergy or history of a severe allergic reaction, anaphylaxis or convulsions following any vaccination or infusion.

8. Presence of current or suspected serious chronic diseases such as cardiac or autoimmune disease (HIV or other immuno-deficiencies), insulin-dependent and non-insulin dependent diabetes (excluding glucose intolerance if exclusion criterion 4 is met), progressive neurological disease, severe malnutrition, acute or progressive hepatic disease, acute or progressive renal disease, porphyria, psoriasis, rheumatoid arthritis, asthma, epilepsy, or obsessive compulsive disorder.

9. History of malignancy of any organ system (other than localised basal cell carcinoma of the skin or *in situ* cervical cancer), treated or untreated, within 5 years of screening, regardless of whether there is evidence of local recurrence or metastases.

10. Subjects with history of schizophrenia, bi-polar disease, or other severe (disabling) chronic psychiatric diagnosis including depression or receiving psychiatric drugs or who has been hospitalised within the past 5 years prior to enrolment for psychiatric illness, history of suicide attempt, or confinement for danger to self or others.

11. History of serious psychiatric condition that may affect participation in the study or preclude compliance with the protocol, including but not limited to past or present psychoses, disorders requiring lithium, a history of attempted or planned suicide, more than one previous episode of major depression, any previous single episode of major depression

lasting for or requiring treatment for more than 6 months, or any episode of major depression during the 5 years preceding screening.

The Beck Depression Inventory will be used as an objective tool for the assessment of depression at screening. In addition to the conditions listed above, subjects with a score of 20 or more on the Beck Depression Inventory and/or a response of 1, 2 or 3 for item 9 of this inventory (related to suicidal ideation) will not be eligible for participation. These subjects will be referred to a general practitioner or medical specialist as appropriate. Subjects with a Beck score of 17 to 19 may be enrolled at the discretion of the Investigator if they do not have a history of the psychiatric conditions mentioned in this criterion and their mental state is not considered to pose additional risk to the health of the subject or to the execution of the study and interpretation of the data gathered.

12. Frequent headaches and/or migraines, recurrent nausea, and/or vomiting (more than twice a month).

13. Presence of acute infectious disease or fever (e.g. sublingual temperature ≥38.5°C) within the 5 days prior to inoculation with malaria parasites.

14. Evidence of acute illness within the 4 weeks prior to screening that the Investigator deems may compromise subject safety.

15. Significant inter-current disease of any type, in particular liver, renal, cardiac, pulmonary, neurologic, rheumatologic, or autoimmune disease by history, physical examination, and/or laboratory studies including urinalysis.

16. Subject has a clinically significant disease or any condition or disease that might affect drug absorption, distribution or excretion (e.g. gastrectomy, diarrhoea).

17. Blood donation of any volume within 1 month before inclusion, or participation in any research study involving blood sampling (more than 450 mL/unit of blood), or blood donation to Australian Red Cross Blood Service (Blood Service) or other blood bank during the 8 weeks prior to the treatment drug dose in the study.

18. Subject unwilling to defer blood donations to the Blood Service for at least 6 months.

19. Medical requirement for intravenous immunoglobulin or blood transfusions.

20. Subject who has ever received a blood transfusion.

21. History or presence of alcohol abuse (alcohol consumption more than 40 g per day) or drug habituation, or any prior intravenous usage of an illicit substance.

22. Tobacco use of more than 5 cigarettes or equivalent per day, and unable to stop smoking for the duration of the clinical unit confinement.

Interfering substances

23. Any vaccination within the last 28 days.

24. Any corticosteroids, anti-inflammatory drugs, immunomodulators or anticoagulants. Any subject currently receiving or having previously received immunosuppressive therapy (including systemic steroids, adrenocorticotrophic hormone or inhaled steroids) at a dose or duration associated with hypothalamic-pituitary-adrenal axis suppression (e.g. 1 mg/kg/day prednisone, chronic use of inhaled high potency corticosteroids such as budesonide 800 µg/day or fluticasone 750 µg, or equivalent).

25. Any recent (<6 weeks) or current systemic therapy with an antibiotic or drug with potential antimalarial activity (e.g. chloroquine, piperaquine phosphate, benzodiazepine, flunarizine, fluoxetine, tetracycline, azithromycin, clindamycin, doxycycline etc.).

26. Ingestion of any poppy seeds within the 24 hours prior to the screening blood test (subjects will be advised by phone not to consume any poppy seeds in this time period).

27. Excessive consumption of beverages or food containing xanthine bases including Red Bull, chocolate, coffee etc. (more than 400 mg caffeine per day, equivalent to more than 4 cups of coffee per day).

28. Unwillingness to abstain from consumption of grapefruit or Seville oranges from inoculation day until end of Malarone® treatment.

29. Unwillingness to abstain from consumption of quinine containing foods/beverages such as tonic water and lemon bitter, from inoculation day until end of Malarone® treatment.

30. Use of prescription drugs or non-prescription drugs or herbal supplements (such as St John's Wort), within 14 days or 5 half-lives (whichever is longer) prior to the malaria challenge agent inoculation. As an exception, ibuprofen (preferred) may be used at doses of up to 1.2 g/day, or paracetamol at doses of up to 4 g/day after discussion with the Investigator. Limited use of other non-prescription medications or dietary supplements, not believed to affect subject safety or the overall results of the study, may be permitted on a case-by-case basis following approval by the Sponsor in consultation with the Investigator. Subjects are requested to refrain from taking non-approved concomitant medications from recruitment until the conclusion of the study. Cohort 3 subjects enrolled in the parallel, optional, observational exploratory study "Advanced medical imaging in subpatent malaria: a second pilot study" will be allowed administration of the radiotracer [^{18}F] fluoro-2-deoxy-2-D-glucose.

General conditions

31. Any subject who, in the judgment of the Investigator, is likely to be noncompliant during the study, or is unable to cooperate because of a language problem or poor mental development.

32. Any subject in the exclusion period of a previous study according to applicable regulations.

33. Any subject who is the Principal Investigator or any sub-investigator, research assistant, pharmacist, study coordinator, or other staff thereof, directly involved in conducting the study.

34. Any subject without a good peripheral venous access.

Biological status

35. Positive result on any of the following tests: hepatitis B surface antigen (HBs Ag), anti-hepatitis B core antibodies (anti-HBc Ab), anti-hepatitis C virus (anti-HCV) antibodies, anti-human immunodeficiency virus 1 and 2 antibodies (anti-HIV1 and anti-HIV2 Ab).

36. Positive urine drug test. Any drug listed in Section 7.2.1 in the urine drug screen unless there is an explanation acceptable to the Investigator (e.g., the subject has stated in advance that they consumed a prescription or over-the-counter product which contained the detected drug) and/or the subject has a negative urine drug screen on retest by the pathology laboratory. Any subject testing positive for acetaminophen (paracetamol) at screening may still be eligible for study participation, at the Investigator's discretion.

37. Positive alcohol breath test.

Specific to the study

38. Cardiac/QT risk:

- Family history of sudden death or of congenital prolongation of the QTc interval or known congenital prolongation of the QTc interval or any clinical condition known to prolong the QTc interval.
- History of symptomatic cardiac arrhythmias or with clinically relevant bradycardia.
- Electrolyte disturbances, particularly hypokalaemia, hypocalcaemia, or hypomagnesaemia.
- ECG abnormalities in the standard 12-lead ECG (at screening or at pre-inoculation on inoculation day) which in the opinion of the Investigator is clinically relevant or will interfere with the ECG analyses.

39. Known hypersensitivity to artesunate or any of its excipients, artemether or other artemisinin derivatives, piperaquine phosphate, proguanil/atovaquone, primaquine, or 4-aminoquinolines.

40. Known severe reaction to mosquito bites other than local itching and redness.

Subjects who are excluded from participation on study days for any of the above reasons may be eligible to participate on a postponed schedule, if the Investigator considers this appropriate.

5.3 STRATEGIES FOR RECRUITMENT AND RETENTION

Up to 27 subjects are planned to be inoculated with K13 or 3D7 challenge agents in the study. It is estimated that approximately 45 subjects may need to be screened to complete enrolment. Subjects who do not receive either the K13 or 3D7 challenge agent may be replaced, in order to reach a study total of up to 27 subjects.

Subjects will be recruited from the QIMR Berghofer Human Research Ethics Committee (QIMR Berghofer HREC) approved database of healthy subjects maintained by Q-Pharm, or by a general or study specific advertisement via print, radio or poster media to students of Queensland universities or to the general community, as approved by the QIMR Berghofer HREC. No restrictions will apply for ethnic or racial categories; the expected population may include all Australian racial categories.

Subjects who complete the study up to Day 28±3/EOS will be paid $2,695 compensation for their participation. Subjects who withdraw or are withdrawn from the study will be compensated on a fractional basis for their involvement unless they are withdrawn as a consequence of their misconduct. Reserve subjects who do not participate in the study will be paid $150 compensation (per inoculation day) for the inconvenience associated with their attendance for screening and for their attendance on the inoculation day, in case they are required to participate. Subjects in Cohorts 2 and 3 who are screened but are ineligible for the study will be paid up to $75 compensation for the inconvenience associated with their attendance for screening (unless there is evidence that the subject knowingly omitted information about their eligibility before screening e.g. positive urine drug screen or positive alcohol breath test etc.). Subjects in Cohort 3 will be paid an additional $50 if they consent to the optional, second DFA.

5.4 SUBJECT WITHDRAWAL OR TERMINATION

5.4.1 REASONS FOR WITHDRAWAL OR TERMINATION

Subjects are free to withdraw from the study at any time. A subject may be considered withdrawn if he/she states an intention to withdraw, fails to return for scheduled protocol visits for any reason, or becomes lost to follow-up. Subjects may also be withdrawn by the Investigator. Possible reasons for withdrawal by the Investigator include the occurrence of a SAE, failure by the subject to comply with the requirements of the protocol, or for any other reason at the Investigator's discretion.

5.4.2 HANDLING OF SUBJECT WITHDRAWALS OR TERMINATION

The Investigator will make every effort to determine the primary reason for a subject's withdrawal from the study and record this information in the electronic case report form (eCRF). For subjects who are lost to follow-up, the Investigator will demonstrate due diligence by documenting all steps taken to contact the subject (e.g. dates of phone calls, registered letter, home visit, etc.) in the source documents. If earlier withdrawal from further study procedures occurs, the subject will be asked to complete the antimalarial rescue treatment. The subjects will also be asked to complete the early termination evaluation as described in Section 7.3.5.

If the subject is withdrawn from the study procedures or follow-up for any reason, with the subject's permission, medical care will be provided for any SAEs that occurred during participation in the study until the symptoms of any SAEs are resolved and the subject's condition becomes stable. Follow-up for AEs is decribed in Section 8.3.

Subjects who are inoculated but fail to complete the study for any reason will not be replaced as agreed by the Investigator and the Sponsor.

5.5 PREMATURE TERMINATION OR SUSPENSION OF STUDY

The Sponsor, Principal Investigator, HREC and Regulatory Authorities independently reserve the right to discontinue the study at any time for safety or other reasons. This will be done in consultation with the Sponsor where practical. In the event of premature trial termination or suspension, the above-mentioned parties will be notified in writing by the terminator/suspender stating the reasons for early termination or suspension (with the exception of the Sponsor's responsibility for notifying the Regulatory Authorities). After such a decision, the Sponsor and the Principal Investigator will ensure that adequate consideration is given to the protection of the subjects' interests and safety. The Investigator must review all subjects as soon as practical and complete all required records.

In addition to the classic assessment of SAEs and the occurrence/severity of other AEs by the Sponsor and the Investigator, after exploring potential confounding factors, the following criteria should be considered as guidance for the decision to stop inoculation of further subjects:

- A subject experiences an SAE that is related to the inoculum.
- There is insufficient response to both artesunate and piperaquine phosphate treatments.
- The Investigator and Sponsor may decide to stop inoculation based on other safety signals not described in the above criteria.

6 STUDY AGENT

6.1 STUDY AGENT(S) AND CONTROL DESCRIPTION

6.1.1 ACQUISITION

P. falciparum K13 and 3D7 challenge agents

The *P. falciparum* Cam3.II^{R539T}/RF967 (K13) parasite was isolated from the blood of a patient in Pursat, Cambodia who presented with clinical malaria [5]. The parasite has a R539T mutation in the propeller domain of the *K13* gene which mediates artemisinin resistance. *In vitro* expansion was used to produce a *P. falciparum* K13 MCB (Q-Gen ID: MBE-018) at Q-Gen, a TGA licensed facility for production of cellular products for use in early clinical studies. The K13 MCB was cryopreserved, aliquoted into cryovials and stored in liquid nitrogen under controlled conditions. Refer to the *P. falciparum* K13 Investigator's Brochure for more details [34]. A K13 MCB cryovial will be retrieved from storage, thawed and used to aseptically prepare the K13 inoculum at Q-Gen.

The *P. falciparum* 3D7 MCB was produced from a person with O Rh(D) Negative blood who was infected with the parasite by mosquito bite. The 3D7 MCB was cryopreserved, aliquoted into cryovials and stored in liquid nitrogen under controlled conditions. Refer to the *P. falciparum* 3D7 Investigator's Brochure for more details [33]. A 3D7 MCB cryovial will be retrieved from storage, thawed, and used to aseptically prepare the 3D7 inoculum at Q-Gen.

Artesunate

Artesunate 50 mg tablets for oral use will be acquired from Guilin Pharmaceutical Co., Ltd. (Guangxi, China). The manufacture of artesunate is undertaken in a WHO Pre-Qualified GMP facility. Import will be facilitated by Medicines for Malaria Venture (MMV). Artesunate will be shipped either directly to Q-Pharm, or via Pharmaceutical Packaging Professionals Pty Ltd (Victoria, Australia).

If a subject vomits or cannot tolerate oral drugs then artesunate will be administered intravenously as described in Section 6.1.5. This drug is the recommended parenteral treatment for malaria in Australia. Currently, it is a Special Access Scheme drug, and has been sourced from Guilin Pharmaceutical (Shanghai) Co., Ltd. Import was facilitated by MMV. The

manufacture of intravenous (IV) artesunate is undertaken in a WHO Pre-Qualified GMP facility (*http://www.mmv.org/access/access-portfolio/artesun-injectable-artesunate*).

Piperaquine phosphate

Piperaquine phosphate will be acquired from PCI Pharma Services and shipped either directly to Q-Pharm, or via Pharmaceutical Packaging Professionals Pty Ltd. Import will be facilitated by MMV.

Rescue medications

Malarone® distributed by GlaxoSmithKline Australia Pty Ltd, and Primacin™ distributed by Boucher & Muir Pty Ltd will be acquired by Q-Pharm.

6.1.2 FORMULATION, APPEARANCE, PACKAGING, AND LABELING

P. falciparum K13 and 3D7 challenge agents

Each K13 and 3D7 inoculum dose will contain parasitised and unparasitised RBCs, resuspended in 0.9% Sodium Chloride Intravenous Infusion, in a total volume of 2 mL in syringes. The syringes will be double contained following preparation and labelled in accordance with GCP guidelines and the Access to Unapproved Therapeutic Goods - Clinical Trials in Australia (TGA, 2004) .

Artesunate

Artesunate is formulated as white, round, 50 mg tablets with a score line and imprinted with "AS 50" on both sides [40]. Artesunate will be labelled according to identity, brand or source, and batch number. The contents of the label for drug to be administered to the subjects will be in accordance with all applicable regulatory requirements.

Piperaquine phosphate

Piperaquine phosphate is formulated as tablets (80, 160, or 320 mg per tablet). Piperaquine phosphate will be labelled according to identity, brand or source, and batch number. The contents of the label for drug to be administered to the subjects will be in accordance with all applicable regulatory requirements.

Rescue medications

Malarone® tablets are round, pink and film-coated, and are engraved with "GX CM3" [47, 50]. Malarone® tablets are supplied in blister packs of 12 or 24 tablets. Primacin™ tablets are round, flat, orange uncoated tablets [48, 49]. Primacin™ tablets are available in bottles of 28 or 56 tablets. Malarone® and Primacin™ will be labelled according to identity, brand or source, and

batch number. The contents of the label for drug to be administered to the subjects will be in accordance with all applicable regulatory requirements.

6.1.3 PRODUCT STORAGE AND STABILITY

P. falciparum K13 and 3D7 challenge agents

Each challenge agent is prepared at Q-Gen on inoculation day (Day 0 for K13 and Day 1 for 3D7). The time between preparation of the K13 or 3D7 inoculum and administration to each subject will be a maximum of 2 hours, during which time the syringes will be kept at the required temperature as recorded on the label.

Artesunate

Artesunate is to be stored protected from light and below 30°C [40].

Piperaquine phosphate

Piperaquine phosphate is to be stored as advised by the manufacturer.

Rescue medications

Malarone® is to be stored below 30°C [47, 50]. Primacin™ is to be stored below 25°C [48, 49].

All drugs will be held in appropriate locked storage conditions at the clinical unit until required.

6.1.4 PREPARATION

P. falciparum K13 and 3D7 challenge agents

The inocula will be prepared aseptically at Q-Gen (QIMR Berghofer), from a frozen cryovial of the *P. falciparum* K13 or 3D7 MCB, by nominated QIMR Berghofer staff members under the guidance of the Investigator. The infected RBCs will be thawed, washed, resuspended in saline, diluted in a final volume of 2 mL of clinical grade saline, and dispensed into syringes [33, 34]. Any remaining unused infected RBCs will be discarded as per approved standard operating procedures.

Artesunate

Artesunate is available as tablets and no preparation is required.

Piperaquine phosphate

Piperaquine phosphate is available as tablets and no preparation is required.

Rescue medications

Malarone® and Primacin™ are available as tablets and no preparation is required.

6.1.5 DOSING AND ADMINISTRATION

P. falciparum K13 and 3D7 challenge agents

An inoculum dose, containing an estimated ~2 800 viable *P. falciparum* K13 or 3D7 parasite-infected RBCs in a volume of 2 mL, will be administrated intravenously to each subject on either Day 0 (K13 subjects) or Day 1 (3D7 subjects). The actual number of parasites inoculated will take into account the loss of viability resulting from cryopreservation, storage and thawing. On inoculation day, subjects may have food until at least half an hour prior to inoculation. Subjects will undergo intravenous cannulation with an appropriate gauge cannula. Placement and patency will be checked by flushing the vein with 5-10 mL of clinical grade saline. The inoculum will be injected, and the cannula again flushed with 5-10 mL of clinical grade saline. The cannula will then be removed, and haemostasis ensured by use of an appropriate dressing. All subjects in a cohort receiving the same inoculum (i.e. K13 or 3D7) will be inoculated intravenously within 60 minutes of each other. See Section 6.1.3 for stability information. Following inoculation of the subjects, the parasite count of the inocula will be quantified by malaria 18S qPCR.

Artesunate

The threshold for commencement of artesunate treatment will be when all subjects reach a parasitaemia of ≥5 000 parasites/mL as determined by malaria 18S qPCR. If the parasitaemia of any subject is ≥5 000 parasites/mL, and is accompanied by a malaria clinical score >6 (Section 7.1.1, adapted from [51]) before all subjects have reached the treatment threshold, then treatment of that subject will begin within a 24 hour period. Subjects may also receive artesunate treatment at any time based on parasitaemia and/or clinical symptoms at the Investigator’s discretion.

Artesunate tablets should be administrated after a ≥8 hour fast, once the subjects have been admitted into the clinical unit or as advised by clinical unit staff. If dosing is to occur in the evening, subjects will be required to fast for ≥4 hours prior to admission. Subjects will receive a single oral dose of artesunate tablets of approximately 2 mg/kg (see Appendix 2 dosing table), in the presence of clinical unit staff. Artesunate tablets should be swallowed with water and should not be taken with a high-fat meal. Subjects should not eat food until 1 hour after artesunate treatment. This dose has been previously shown to reduce parasitaemia in patients with uncomplicated *P. falciparum* malaria [3, 37], and should allow the parasite clearance profile for both K13 and 3D7 to be properly characterised and compared.

Note: If a subject vomits or cannot tolerate oral drugs then artesunate will be administered intravenously. If the subject is vomiting after treatment with the single dose of oral artesunate and it is deemed likely by the Investigator that the subject has absorbed a sufficient amount of artesunate, IV artesunate may not be given immediately. IV artesunate may be initiated in the future by the Investigator for safety reasons if vomiting continues and the subject cannot tolerate oral drugs. This would be done at the recommended dose regime of 2.4 mg/kg at approximately 12, 24, 48 hours post oral artesunate or until able to take oral drugs.

If the subject is vomiting after treatment with the single dose of oral artesunate and it is deemed likely by the Investigator that the subject has not absorbed a sufficient amount of artesunate, IV artesunate may be initiated by the Investigator as a single dose of 2.4 mg/kg IV as a replacement for single dose oral artesunate.

If the subject is vomiting before treatment with the single dose of oral artesunate, IV artesunate may be initiated by the Investigator on clinical grounds as a single dose of 2.4 mg/kg IV as a replacement for single dose oral artesunate.

Piperaquine phosphate

Subjects will receive treatment with piperaquine phosphate (960 mg) if required as a single dose orally (Section 7.3.3).

Cohort 1:

For Cohort 1, piperaquine phosphate treatment will occur on an individual subject basis in the event of unsatisfactory parasite clearance after artesunate treatment, defined as 2 consecutive time-points showing a decrease in parasitaemia of less than 20% of baseline (i.e., the parasitaemia before drug treatment) by 72 hours post-artesunate treatment. Piperaquine phosphate treatment will also occur if there is recrudescence of parasitaemia after artesunate treatment, defined as ≥5 000 blood stage parasites/mL and a 2-fold increase within 48 hours, or a malaria clinical score >6. Piperaquine phosphate treatment can also be undertaken at the Investigator's discretion.

The decision to administer piperaquine to subjects will be made by the Investigator in consultation with the local Independent Medical Monitor (IMM), who is an external malaria expert, to advise on the safety of continuing subject(s) observation without piperaquine phosphate treatment versus administration of piperaquine phosphate.

Cohorts 2 and 3:

For Cohorts 2 and 3, piperaquine phosphate treatment will occur for subjects inoculated with the 3D7 challenge agent as outlined above for Cohort 1. However, K13 subjects in Cohorts 2 and 3 will be treated with piperaquine phosphate on Day 11 AM or earlier at the Investigator's

discretion. This is to avoid the high parasitaemias seen in K13 subjects in Cohort 1 on Day 11 PM prior to piperaquine phosphate treatment.

All cohorts:

Piperaquine phosphate 80, 160 or 320 mg tablets may be used. It is recommended that piperaquine phosphate is administered at the clinical unit after a ≥4 hour fast. Subjects will be requested to fast for a further 4 hours after piperaquine phosphate treatment.

<u>Rescue medications</u>

Cohort 1:

All Cohort 1 subjects will receive compulsory rescue treatment with Malarone® on Day 26±3 or earlier at the Investigator's discretion. Subjects may also be treated with Primacin™ at the time of Malarone® treatment, if gametocytaemia is suspected from parasite lifecycle stage qRT-PCR or by the presence of stable low level parasitaemia, or at the Investigator's discretion, to ensure complete clearance of gametocytes.

Cohorts 2 and 3:

All Cohort 2 and 3 subjects will be treated with Primacin™ on Day 23±3 or earlier at the Investigator's discretion. This is to ensure complete clearance of gametocytes before Day 28±3 (End of Study). All subjects will receive compulsory rescue treatment with Malarone® (atovaquone/proguanil hydrochloride; 3 day course) on Day 25±3 or earlier at the Investigator's discretion.

All cohorts:

Malarone® will be administered as recommended by the manufacturer for treatment of malaria [47, 50]. A treatment course of Malarone® consists of 4 tablets of Malarone® (atovaquone 250 mg, proguanil hydrochloride 100 mg) once daily, orally for 3 days. Each dose should be taken with food or drinks rich in fat (e.g., milk). Subjects will be reminded of the potential side effects of Malarone® and given the Consumer Medicine Information for Malarone® [50].

If needed, subjects will take 6 Primacin™ tablets, each containing 13.2 mg primaquine phosphate equivalent to 7.5 mg primaquine (the total primaquine dose will be 45 mg). Primacin™ for oral use will be taken as a single dose with food. Subjects will be reminded of the potential side effects of Primacin™ and given the Consumer Medicine Information for Primacin™ [48].

IV artesunate may be used as a rescue medication if a subject vomits or cannot tolerate oral drugs. See artesunate section above.

6.1.6 ROUTE OF ADMINISTRATION

P. falciparum K13 and 3D7 challenge agents

Intravenous administration.

Artesunate

Oral administration.

If a subject vomits or cannot tolerate oral drugs then artesunate will be administered intravenously as described in Section 6.1.5.

Piperaquine phosphate

Oral administration.

Rescue medications

Oral administration.

6.1.7 STARTING DOSE AND DOSE ESCALATION SCHEDULE

See Section 6.1.5 for dose information. No dose escalation will be performed.

6.1.8 DOSE ADJUSTMENTS/MODIFICATIONS/DELAYS

Not applicable.

6.1.9 DURATION OF THERAPY

See Section 6.1.5 for dose information.

6.1.10 TRACKING OF DOSE

The *P. falciparum* challenge agents, artesunate, piperaquine phosphate, and Primacin™ will be administered at the clinical unit in the presence of clinical unit staff. Subjects may be

administered Malarone® on site for initial dosing followed by monitoring, either in the clinic, or by phone for 3 days to ensure adherence to Malarone® therapy.

6.2 STUDY AGENT ACCOUNTABILITY PROCEDURES

The Q-Pharm pharmacist or delegate, as nominated by the Principal Investigator, is responsible for maintaining accurate study agent accountability records throughout the study. Study agents include the challenge agents, artesunate, piperaquine, and the rescue medications. Dispensing, accountability and documentation will be in accordance with Q-Pharm standard procedures. All products will be inventoried upon receipt by the Q-Pharm pharmacist. The condition of the products at the time of receipt by the pharmacist will be documented, as will the time restrictions of use for the syringes containing the challenge agents. The lot numbers and expiry dates of the inoculum and antimalarial drugs will be documented. The Q-Pharm pharmacist or delegate will ensure that the received products are the specified formulation.

The storage, handling and the disposal of the challenge agents will be in accordance with approved procedures. All dosages prescribed and dispensed to the subjects and all dose changes during the study must be recorded in the eCRFs. All drug supplies are to be used only in accordance with this protocol, and not for any other purpose. All used medications will be fully documented. Used and unused drug containers must be destroyed at the site once drug accountability is final and has been checked by the Sponsor or its delegate, and written permission for destruction has been obtained from the Sponsor.

Study products and study accountability logs will be available to the Sponsor or their representative as part of the study monitoring procedures. Upon completion of the study, copies of all study drug management records will be provided to the Sponsor. Original records will be maintained at the clinical site with the rest of the study records.

7 STUDY PROCEDURES AND SCHEDULE

7.1 STUDY PROCEDURES/EVALUATIONS

7.1.1 STUDY SPECIFIC PROCEDURES

Medical history

Medical history will be elicited at screening as described below. Information regarding any new medical conditions or illnesses will be elicited prior to inoculation on inoculation day, and Day 28±3/EOS or early termination visit.

Past medical/surgical history includes:
History of all known allergies
Current medications, including over-the-counter and herbal preparations
History of substance abuse and recreational drug use
History of depression, anxiety, mental illness, emotional problems, use of psychiatric medications and previous psychotherapy
Surgical procedures and results

Physical examination

Complete physical examination includes:
Weight **(Screening only)**
Height **(Screening only)**
Vital signs (temperature, heart rate, respiratory rate, and blood pressure)
Review of systems excluding genitourinary examination and including the following:
Head, neck (including thyroid), ears, eyes, nose and throat
Heart/circulation
Chest
Lungs
Abdomen
Skin
Neurological exam
Extremities
Back
Dentition

Abbreviated physical examination (Cohort 1) includes:
Vital signs (as above)
Systems/organs to examine:
General appearance
Skin
Chest
Lungs
Heart/circulation
Abdomen
Brief neurological exam
Other areas in relation to reported adverse events

Symptom-Directed Physical Examination (Cohorts 2 and 3): physical examinations will be symptom-directed at specified time-points (i.e. body systems will be reviewed only if clinically indicated at the discretion of the Investigator).

Beck Depression Inventory

All subjects will be required to complete the Beck Depression Inventory at screening. This is a validated questionnaire used as an objective tool for the assessment of depression.

Vital signs

Vital signs (temperature, heart rate, respiratory rate and blood pressure) will be measured at screening after the subject has rested in the supine position for at least 5 minutes and in the standing position within 2-3 minutes when changing from the supine to standing position (blood pressure and heart rate only). At all other time-points, vital signs will be measured after the subject has rested in the seated position for at least 5 minutes. Tympanic temperature will be taken at the clinical unit, and sublingual temperature will be taken by subjects at home for practical reasons.

Electrocardiograms

A single 12-lead ECG will be recorded after resting supine for at least 5 minutes.

Blood sampling

Subjects will be cannulated with an appropriate gauge intravenous cannula prior to inoculation on inoculation day, and during the artesunate confinement period. It will be recorded which arm is utilised. At other times blood samples will be taken by venepuncture.

Blood will be collected for clinical laboratory evaluations including haematology, clinical biochemistry, serology and pregnancy testing (see Section 7.2.1). Blood samples will also be collected to monitor parasitaemia and to quantify plasma drug concentrations for PK analysis (see Section 7.2.2). The estimated blood volume required for these tests is listed in Appendix 3. The total volume of blood drawn from each subject will not exceed 450 mL in any given 30-day period. This volume includes allowance for unscheduled safety laboratory assessments that may be required at the discretion of the Principal Investigator or the Sponsor to ensure subject safety.

Urine sample collection

Urine will be collected for urinalysis and drug screens (see Section 7.2.1).

Adverse event recording

Adverse events will be recorded as described in Section 8.

Malaria clinical score

The below scoring system will be used to generate an overall clinical score for malaria signs/symptoms, which is used to guide antimalarial treatment (adapted from [51]). The

maximum total score is 42. Individual scores for each symptom as well as the total score will be recorded.

**Visit**	**Date**	**Symptoms**	**Malaria clinical score**			
			**Absent (0)**	**Mild (1)**	**Moderate (2)**	**Severe (3)**
		Headache				
		Myalgia (muscle ache)				
		Arthralgia (joint ache)				
		Fatigue/lethargy				
		Malaise (general discomfort/uneasiness)				
		Chills/shivering/rigors				
		Sweating/hot spells				
		Anorexia				
		Nausea				
		Vomiting				
		Abdominal discomfort				
		Fever				
		Tachycardia				
		Hypotension				
		**Total score**				

7.1.2 STANDARD OF CARE STUDY PROCEDURES

Not applicable.

7.2 LABORATORY PROCEDURES/EVALUATIONS

7.2.1 CLINICAL LABORATORY EVALUATIONS

Any significant deviations from results obtained during screening will be followed until resolution or investigated fully, or until the subject is referred to a general practitioner. The Investigator will document the clinical significance of all results falling outside of the normal reference ranges. All abnormal laboratory tests results judged as being clinically significant will be recorded as adverse events.

The estimated blood volume required for these tests is listed in Appendix 3.

Haematology

**Full blood count (FBC) with differential**
White blood cell count (WBC)
WBC differential (diff)
A manual blood smear should be reviewed if there are immature/abnormal cells detected on the automated differential or if an automated differential was not able to be performed.
Neutrophils (NEUT)
Lymphocytes (LYM)
Monocytes (MON)
Eosinophils (EOS)
Basophils (BAS)
Red blood cell count (RBC)
Haemoglobin (HGB)
Haematocrit (HCT)
Platelet count (PLAT)
Reticulocyte count (RETI) **(Day -3 to -1 safety visit or at screening if between Day -3 to -1, and Day 28±3 or early termination visit only)**
Blood Group and Rh(D) tests **(Screening only)**

RBC alloantibodies testing

Blood for RBC alloantibodies testing will be collected at screening and Day 28±3 or early termination visit.

G6PD testing

Blood for G6PD screening will be collected at screening only. G6PD deficiency is not an exclusion criterion but will be determined at screening to ensure the safety of Primacin™.

Biochemistry

Sodium (SODIUM)	Alkaline phosphatase (ALP)
Potassium (K)	Alanine aminotransferase (ALT, SGPT)
Chloride (CL)	Aspartate aminotransferase (AST, SGOT)
Bicarbonate (BICARB)	Calcium corrected
Glucose (GLUC)	Phosphate (PHOS)
Urea	Lactate dehydrogenase (LDH)
Creatinine (CREAT)	Magnesium ***(Screening only)***
Urate	Cholesterol ***(Screening only)***
Albumin (ALB)	Triglycerides ***(Screening only)***
Globulin	HDL ***(Screening only)***
Total protein	Estimated glomerular filtration rate (eGFR)
Total bilirubin (BILI)	C-reactive protein (medium sensitivity) (Cohorts 2 and 3)
Direct (conjugated) bilirubin (BILDIR)	

Urinalysis

Urine will be tested by dipstick at the clinical unit. If there are any abnormalities considered clinically significant in blood, leucocytes or protein, the urine will be sent for formal laboratory urinalysis per the clinical unit standard procedure.

Glucose (GLUC)
Bilirubin (BILI)
Ketone (KETONES)
Specific gravity (SPGRAV)
Blood
pH
Protein (PROT)
Urobilinogen (UROBIL)
Nitrite
Leukocytes (WBC)
Formal laboratory urinalysis (if required)

Urine drug screens and alcohol breath tests

If the results of the urine drug screens or alcohol breath tests are positive, subjects may be allowed to continue, or may be delayed or withdrawn according to site-specific instructions.

All subjects will be questioned about concomitant medications and use of recreational drugs. The urine drug screen may be repeated if the potential subject denies usage of any of these agents and the test result is believed to be a false positive.

Subjects testing positive for paracetamol at screening and/or inoculation day may still be eligible for study participation, at the Investigator's discretion. Subjects requiring paracetamol on a daily basis would not be eligible to enrol in the study, as the use of any over-the-counter medication during the study is restricted and potential subjects should not discontinue their usual medications in order to participate in the study.

**Urine drug screens:**	
Amphetamines	Opiates
Methamphetamines	Phencyclidine
Barbiturates	Tetrahydrocannabinol (cannabis)
Benzodiazepines	Tricyclic antidepressants
Cocaine	Acetaminophen (paracetamol)
Methadone	
**Alcohol breath test**	

Serology

HIV 1/2 (anti-HIV1 and anti-HIV2 Ab)
Hepatitis B (HBs Ag, anti-HBc (IgG + IgM if IgG is positive))
Hepatitis C (anti-HCV)
Hepatitis A (anti-HAV) (IgM) - performed off stored sample for testing, at Investigator's discretion
Hepatitis E (anti-HEV) (IgM) - performed off stored sample for testing, at Investigator's discretion
Epstein-Barr virus - performed off stored sample for testing, at Investigator's discretion
Cytomegalovirus - performed off stored sample for testing, at Investigator's discretion

Pregnancy testing

At screening, a serum β-hCG pregnancy test will be conducted on all female subjects, and follicle stimulating hormone levels will be tested in post-menopausal women of non-childbearing potential. After enrolment in the study, urine β-hCG pregnancy tests will be conducted on all female subjects. If a subject is found to have a positive urine pregnancy test at any stage during the study, a confirmatory serum β-hCG will be performed. If confirmed positive, the subject will be discontinued from the study.

Safety serum storage

Blood for serum storage as safety retention samples will be collected at times outlined in Section 7.3.

7.2.2 OTHER ASSAYS OR PROCEDURES

The estimated blood volume required for these tests is listed in Appendix 3.

Malaria monitoring

Blood will be collected to monitor parasitaemia using qPCR targeting the 18S rRNA gene [6]. These samples will be up to or approximately 2 mL for Cohorts 1 and 2. These samples may be approximately 2 mL or 3 mL depending on the time-point for Cohort 3.

Additional blood (up to approximately 2.5 mL per time-point) may be collected for parasite lifecycle stage qRT-PCR at the Investigator's discretion. This is to evaluate for the presence of sexual parasite stages (gametocytes) and other parasite lifecycle stages in the blood. This blood may also be used for research into various aspects of parasite biology e.g. gametocytes, parasite lifecycle stages, recrudescence, commitment etc. The qRT-PCR may target genes including but not limited to: the female gametocyte-specific transcript *pfs25*, the male gametocyte-specific transcript *pfMGET*, and the ring-stage transcript *pfSBP-1* as appropriate. The samples may be taken at time-points indicated by malaria 18S qPCR, or at the Investigator's discretion. Parasite lifecycle stage qRT-PCR may also be performed using malaria 18S qPCR samples at the Investigator's discretion.

Microscopic examination for evidence of parasitaemia or gametocytaemia may be conducted at the Investigator's discretion. Thin and thick films may be prepared from blood collected at time-points coinciding with MFA.

Drug concentration measurements (PK samples)

Plasma concentrations of artesunate and dihydroartemisinin will be determined using liquid chromatography-tandem mass spectrometry (LC-MS/MS) as previously described [52] with modification (i.e. substituting a protein precipitation extraction for solid phase extraction).

Malaria transmission assays

Transmission ability of gametocytes to laboratory reared female *Anopheles* mosquitoes may be determined using DFAs and MFAs. This may be done for selected cohorts at the Investigator's discretion. Mosquitoes will be maintained in a controlled environment in the PC3 QIMR Berghofer insectary.

- DFA: subjects will be escorted to the insectary and asked to allow mosquitoes to feed on them. Up to approximately 60 *Anopheles* mosquitoes/cup at one time-point (Cohorts 1 and 2) or at up to 2 time-points (Cohort 3) will be allowed to bite on the volar surface of the forearms, thighs or calves of subjects to directly feed for approximately 15±5 minutes to enable mosquitoes to fully engorge. An experienced nurse will be in attendance at the clinical unit and the insectary throughout the period of direct feeding and the Investigator will be available within 30 minutes call-back if required.
- MFA: Up to approximately 36 mL blood may be collected at one time-point (Cohorts 1 and 2) or up to approximately 36 mL blood per time-point may be collected at up to two time-

points (Cohort 3) and transported at ~37°C. Mosquitoes will be distributed into containers with gauze lids and starved prior to feeding assays. Mosquitoes will be allowed to feed for approximately 30 minutes on the blood through bovine caecum or parafilm membranes on water jacketed glass feeders attached to a 37°C water bath.

For both assays, after feeding, the number of non-engorged mosquitoes will be recorded. Following the feeds, the mosquitoes will be maintained in a controlled environment and will be provided with a sugar solution supplemented with 0.05% para-amino benzoic acid to promote the sporogonic cycle. Seven to 10 days after blood feeding, mosquitoes will be dissected and evaluated for the presence of oocysts in midgut preparations using malaria 18S qPCR, microscopy (oocysts will be stained with 0.1% mercurochrome), immunofluorescence assay, or CS ELISA.

A subset of fed mosquitoes may be kept for up to 24 days in order to investigate for development of salivary gland sporozoites. The prevalence and/or intensity of oocyst infection will be determined in the blood fed mosquitoes. The number of mosquitoes dying prior to dissection will be recorded.

Exploratory objective – drug response research (Cohorts 2 and 3)

Blood samples may be collected for drug response research during Cohorts 2 and 3 of the study at the Investigator's discretion. The blood samples will be used to characterise the antimalarial treatment effects on *P. falciparum* K13 and 3D7 parasites using phenotypic and transcriptomic approaches. These approaches may include the following: 1. Assess re-growth of parasites *ex vivo* by qPCR, flow cytometry and microscopy. 2. Perform *in vitro* drug sensitivity testing determining the 50% inhibitory concentrations (IC50s) and the percentage surviving parasites. 3. Perform molecular methods including whole genome sequencing; identification of molecular markers such as *gEXP-5*, *pfSBP-1*, *pfMGET*, *pfs25* etc; whole RNA sequence analysis to determine family-genes with differential expression between pre- and post-antimalarial treatment. Microscopic examination (thin and thick films) may also be performed using these samples.

The blood samples will be collected at up to approximately 13 time-points as outlined in Section 7.3 and Table 1. The blood samples may be between approximately 2 mL to 10 mL depending on the time-point, as outlined in Appendix 3. The samples may be shipped to nominated local or international laboratories for assessment.

Exploratory objective – dormancy detection research (Cohorts 2 and 3)

Bloods samples may be collected for dormancy detection research during Cohorts 2 and 3 of the study at the Investigator's discretion. The blood samples will be used to investigate and characterise the *in vivo* presence of dormant parasites by determining the transcriptional molecular signature of dormant parasites and performing parasite lifecycle stage qRT-PCR on

K13 and 3D7 parasites pre- and post-artesunate treatment. The blood samples (up to approximately 30 mL per time-point) will be collected at 2 time-points as outlined in Section 7.3 and Table 1. The post-artesunate treatment sample will be taken at 72 hours post-artesunate for Cohort 2 and 48 hours post-artesunate for Cohort 3. The samples may be shipped to nominated local or international laboratories for assessment.

Optional exploratory objective - genetic research

Blood (approximately 4 mL) may be collected for optional genetic research at one of the clinical visits during the study at the Investigator's discretion and will not require an additional clinical visit (see Section 4.1). The sample will be stored indefinitely at QIMR Berghofer, Q-Pharm, or a nominated testing laboratory. The sample may be shipped to nominated local or international laboratories for assessment. The sample will have all identifiers (e.g. name and personal details) removed and replaced by a code so that it is re-identifiable.

Optional, exploratory malaria research studies

Subjects who are enrolled in this study may also enrol in optional, exploratory malaria research studies, outlined in a separate protocol and Participant Information Sheet and informed Consent Form that require separate consent for subjects agreeing to participate in any of these. These optional, exploratory studies include research into parasite dormancy, parasite growth rates, *var* gene expression, parasite biomarkers (BIOME), immunology, serology, and metabolomics. The exploratory studies will involve collection of additional blood samples during this study (up to approximately 201 mL). Refusal to participate in the optional exploratory studies will not jeopardise a subject's participation in this study.

Optional, exploratory medical imaging study

In Cohort 3, up to two subjects may also be recruited for a parallel, optional, observational exploratory study "Advanced medical imaging in subpatent malaria: a second pilot study" which is outlined in a separate protocol and informed Consent Form. The purpose of the exploratory study is to apply functional nuclear medicine imaging techniques to determine parasite biodistribution and biomass in subpatent malaria. These subjects will receive a whole body positron emission tomography/magnetic resonance imaging (PET/MRI) scan and dedicated brain MRI within approximately one week prior to inoculation and repeat imaging one to two days prior to confinement with peak parasitaemia post inoculation (estimated Day 7 to 9). Imaging will take place at the Herston Imaging Research Facility located in the Herston Health Precinct. Preparation for imaging involves fasting for 6 hours prior, abstaining from excessive physical exertion and following a low carbohydrate diet 24 hours prior (recorded on a provided diet and activity sheet). Scans will be performed on the Biograph mMR PET/MRI system after the intravenous infusion of the standard radiotracer [^{18}F] fluoro-2-deoxy-2-D-glucose and a MAGNETOM Prisma 3T MRI system. Imaging is not expected to impact on the clinical course of the main study. If applicable, specific AEs reported during these evaluations will be related to

the study procedures. Subjects will be compensated for their time separate to the main study reimbursement. Further details are located in the exploratory informed Consent Form, which will be provided to the subjects.

7.2.3 SPECIMEN PREPARATION, HANDLING, AND STORAGE

Q-Pharm's standard work instructions will apply to the allowed time windows for study procedures and sample collection. If the observation time and blood sampling time coincide, for precision of timing, blood collection will take precedence over other procedures scheduled at the same time.

Blood will be collected into tubes containing the appropriate anti-coagulant. Samples will be processed according to the laboratory requirements.

Biological samples will be retained for the time required for assessment for analysis, and may then be discarded. Safety serum samples will be stored indefinitely with the permission of the subjects for any retrospective safety assessments.

7.2.4 SPECIMEN SHIPMENT

Samples collected will be shipped to nominated local or international laboratories for assessment (Section 1). The clinical unit staff will be responsible for shipment of samples to analytical laboratories for testing. Samples must be packed securely together with completed shipment forms in shipping containers together with sufficient dry ice as per Shipper procedures.

7.3 STUDY SCHEDULE

7.3.1 SCREENING

A screening visit will be scheduled after an initial phone interview conducted by clinical unit staff has occurred, to review background information. For the screening visit, potential subjects will be asked to come to the clinical unit after an overnight fast of ≥8 hours. During this initial screening visit, the potential subject will read the Participant Information Sheet and be encouraged to ask questions.

Subjects will be fully informed of the nature of the study at this time, and advised of the requirement to repeat some screening tests during the Day -3 to Day -1 safety visit (if required) and/or on inoculation day to determine their continuing eligibility. Subjects must confirm that they will not be living alone from inoculation day until the end of the Malarone® treatment.

Individuals willing to be considered for inclusion may sign the Consent Form during the screening visit, or return to the clinical unit after further consideration. The subject will be given a copy of the Participant Information Sheet and signed Consent Form for their records. The signed and dated originals will be held on file by the clinical unit. Participation consent must be obtained from all subjects prior to screening tests.

The consenting process will take place with one of the Q-Pharm medical staff or Investigators and will include:

1. Provide the Participation Information Sheet and informed Consent Form and give the subject sufficient time to review the contents.
2. Explain the study via the Participation Information Sheet and gain informed consent from the subject.
3. Ensure the subject has signed the informed Consent Form and received a signed copy.

Once the potential subject has provided written consent to participate, the pre-study screening may be initiated. The screening will be conducted within 4 weeks prior to the inoculation day and will include:

1. A screening number will be assigned to each subject.
2. Elicit a complete medical history and use of medications.
3. Elicit a social history including alcohol and tobacco use.
4. Perform alcohol breath test.
5. Perform complete physical examination.
6. Ask subject to complete the Beck Depression Inventory.
7. Assess the cardiovascular disease risk as per exclusion criterion 4.
8. Record vital signs.
9. Obtain a 12-lead ECG.
10. Collect urine for urinalysis and drug screen.
11. Collect blood samples for haematology, biochemistry, RBC alloantibodies, G6PD testing, and serology (viral hepatitis B and C and HIV).
12. Perform serum β-hCG pregnancy test for all female subjects and follicle stimulating hormone test for post-menopausal females.
13. Verify subject meets inclusion/exclusion criteria.

Subjects who complete all screening procedures and satisfy all entry criteria will be considered eligible to participate in this study. To be eligible for study entry, laboratory values at screening must not be outside the range of the normal values at a level deemed to be clinically significant. For eligibility parameters a repeat may be requested to exclude laboratory error. Re-screening will not be allowed unless the Investigator considers the cause of the initial pre-screening failure to be of an acute and completely reversible nature.

If screening laboratory results are abnormal, e.g. HIV testing, the subject will be referred for appropriate counselling. If any clinically significant abnormalities are detected during screening, the subject will be referred for follow-up tests to a general practitioner or medical specialist as appropriate.

Subjects will be further invited to participate in optional, exploratory malaria research studies that may also be undertaken in conjunction with this study (Section 7.2.2). A separate Participant Information Sheet and informed Consent Form will be provided to subjects interested in taking part in the exploratory studies.

7.3.2 ENROLLMENT/BASELINE

Eligible, consenting subjects will be randomised to receive either the K13 challenge agent or the 3D7 challenge agent, in accordance with a randomisation schedule generated by a statistician using a validated system (see Section 10.6.1 for further details). This will occur prior to Day 0.

Day -3 to Day -1 safety visit

Subjects (including reserve subjects) will report to the clinical unit between Day -3 to Day -1 for the following baseline assessments, unless screening laboratory assessments were conducted within this period, in which case repeat sampling will not be required.

This visit will include:

1. Collect blood samples for haematology and biochemistry.
2. Collect urine for urinalysis.

The timing of these assessments is to ensure that results are available for review by the Investigator prior to inoculation on inoculation day. Subjects with clinically significant laboratory findings at this stage will not be eligible for inoculation.

Malaria inoculation day (Day 0 for K13 subjects and Day 1 for 3D7 subjects)

Subjects, including approximately 2 to 3 reserve subjects per inoculation day, will report to the clinical unit on the morning of inoculation day. This will be Day 0 for subjects randomised to receive the K13 challenge agent, and Day 1 for subjects randomised to receive the 3D7 challenge agent. The Investigator will review the subjects’ screening results prior to their enrolment into the study. The Investigator will emphasise the requirement to return for antimalarial drug treatment after the inoculation. Subjects will be reviewed by the Investigator to confirm their continued eligibility for the study, including confirmation that they will not be living alone from inoculation day until the end of the study by checking housemates contact details recorded at screening visit.

On admission to the clinical unit, subjects will be required to undertake further screening procedures to determine whether they remain eligible to be enrolled. A reserve subject may be asked to replace a subject who does not continue to meet eligibility. These reserves will be compensated for the study visit even if not inoculated, as described in the Participant Information Sheet and Consent Form.

The procedures that will be undertaken prior to inoculation include:

1. Verify that all applicable eligibility criteria have been met.
2. Elicit information regarding any new medical conditions, illnesses, or medications since screening.
3. Perform alcohol breath test.
4. Perform abbreviated physical examination (Cohort 1) or symptom-directed physical examination (Cohorts 2 and 3).
5. Record vital signs.
6. Obtain a 12-lead ECG.
7. Perform urine β-hCG pregnancy test for all female subjects.
8. Collect urine for drug screen.
9. Cannulate subjects with an indwelling intravenous cannula for the malaria parasite inoculation.
10. Collect blood samples for malaria 18S qPCR (parasitaemia baseline sample) and safety serum storage.

Administration of the malaria inoculum:

1. Administer the malaria inoculum of ~2 800 viable *P. falciparum* K13 or 3D7 infected human RBCs intravenously in the morning (approximately 9:00 AM).
2. Observe for a minimum of 60 minutes after inoculation to evaluate for immediate adverse reactions.
3. Educate subjects on signs and symptoms of malaria (Appendix 4).
4. Emphasise to subjects the importance of returning on the nominated day (approximately Day 9), or as advised by the clinical unit staff, for antimalarial treatment.
5. Provide subjects with diary cards and thermometers to record any temperature readings during the study in the event of symptoms of fever. Subjects will also record symptoms and concomitant medications on the diary cards during the study.
6. Record adverse events and concomitant medications.
7. Record vital signs prior to leaving the clinical unit (approximately 60 minutes after inoculation).
8. Record malaria clinical score prior to leaving the clinical unit (malaria clinical score baseline sample; see Section 7.1.1).

7.3.3 FOLLOW-UP

Malaria monitoring via phone (Days 1-3 for K13 subjects; Days 2-4 for 3D7 subjects)

During this period, subjects are expected to be asymptomatic. A daily phone call or text message will be made to the subjects by clinical unit staff to monitor subject well-being and to solicit any adverse events.

Daily visits for malaria monitoring (Day 4 AM for K13 subjects and Day 5 AM for 3D7 subjects, until qPCR positive for malaria)

Follow-up from Day 4 (K13 subjects) or Day 5 (3D7 subjects) until qPCR becomes positive will be undertaken through daily visits (approximately 8:00 AM) to the clinical site.

The following procedures will occur during these visits:

1. Perform abbreviated physical examination (Cohort 1) or symptom-directed physical examination (Cohorts 2 and 3) when signs or symptoms of malaria are identified and it is clinically indicated (Appendix 4).
2. Record vital signs.
3. Collect blood sample for malaria 18S qPCR.
4. Record malaria clinical score.
5. Record adverse events and use of concomitant medications.

Day when qPCR Positive until treatment day

Follow-up from the day that malaria 18S qPCR becomes positive until treatment day will be undertaken through twice daily (AM & PM) visits to the clinical site separated by approximately 12 hours (i.e. 06:00 – 11:00 and 18:00 – 23:00). The following procedures will occur during these visits:

1. Perform abbreviated physical examination (Cohort 1) or symptom-directed physical examination (Cohorts 2 and 3) when signs or symptoms of malaria are identified and it is clinically indicated.
2. Record vital signs.
3. Collect blood sample for malaria 18S qPCR.
4. Record malaria clinical score.
5. Record adverse events and use of concomitant medications.

In-patient observation and antimalarial treatment phase (from approximately Day 9)

Subjects will be admitted to the clinical unit for 72 hours, when they meet the treatment threshold (Section 6.1.5) or at the Investigator’s discretion, for artesunate treatment and

monitoring of clinical features of malaria. All K13 and 3D7 subjects will be treated on the same day (unless any subject required earlier treatment at the Investigator's discretion).

Admission

The following procedures will occur at admission to the clinical unit:

1. Perform abbreviated physical examination (Cohort 1) or symptom-directed physical examination (Cohorts 2 and 3).
2. Perform alcohol breath test.
3. Record vital signs.
4. Obtain a 12-lead ECG.
5. Collect urine for urinalysis and drug screen.
6. Perform urine β-hCG pregnancy test for all female subjects.
7. Cannulate subjects with an indwelling intravenous cannula.
8. Collect blood samples for haematology, biochemistry, malaria 18S qPCR (parasite clearance baseline sample), parasite lifecycle stage qRT-PCR at the Investigator's discretion (Section 7.2.2), PK analysis (measurement of artesunate and dihydroartemisinin levels; PK baseline sample), drug response research (Cohorts 2 and 3 at the Investigator's discretion; baseline sample), and dormancy detection research (Cohorts 2 and 3 at the Investigator's discretion; baseline sample).
   NOTE: As these test results may not be available before the drug administration, the results of this time-point will be used for proper interpretation of study results.
9. Record malaria clinical score.
10. Record adverse events and use of concomitant medications.

Treatment and observation

The following procedures will occur during treatment and observation:

1. Administer approximately 2 mg/kg artesunate under direct observation (see Appendix 2 dosing table).
2. Follow up subjects as in-patients for 72 hours to ensure tolerance of the therapy and adequate clinical response.
3. Perform abbreviated physical examination (Cohort 1) or symptom-directed physical examination (Cohorts 2 and 3) when signs or symptoms of malaria are identified and it is clinically indicated.
4. Record vital signs 3 times a day whilst confined.
5. Record malaria clinical score 3 times a day whilst confined.
6. Collect blood samples for malaria 18S qPCR at the approximate time-points post artesunate treatment specified below:

Malaria 18S qPCR time-points post artesunate treatment by cohort

Cohort 1	Cohort 2	Cohort 3
2 h	2 h	-
4 h	4 h	4 h
6 h	6 h	-
8 h	8 h	8 h
12 h	12 h	12 h
16 h	16 h	16 h
20 h	20 h	20 h
24 h	24 h	24 h
28 h	28 h	28 h
32 h	32 h	32 h
36 h	36 h	36 h
-	-	40 h
-	-	44 h
48 h	48 h	48 h
-	52 h (K13 subjects only)	52 h
-	56 h (K13 subjects only)	56 h
60 h	60 h	60 h
-	-	64 h
-	-	68 h
72 h	72 h	72 h

h = hours post artesunate treatment

7. Collect blood samples for PK analysis of artesunate and dihydroartemisinin following artesunate treatment at approximately 0.25, 0.5, 1, 1.5, 2, 2.5, 3, 4, 6, 8, 10, and 12 hours post-artesunate treatment.
8. Collect blood samples for drug response research following artesunate treatment at approximately 2, 4, 8, 12, 16, 20, 24, 48 and 72 hours post-artesunate treatment (Cohort 2 at the Investigator's discretion) and at up to 2 time-points post-artesunate treatment (Cohort 3 at the Investigator's discretion).
9. Collect blood samples for dormancy detection research (~48 hour post-artesunate treatment time-point; Cohort 3 only at the Investigator's discretion).
10. Collect blood samples for parasite lifecycle stage qRT-PCR (if required).
11. Record adverse events and use of concomitant medications.

<u>NOTE:</u> K13 subjects in Cohorts 2 and 3 will be treated with piperaquine phosphate on Day 11 AM (during the in-patient treatment and observation period) or earlier at the Investigator's discretion. See piperaquine phosphate treatment section below for details of procedures to be conducted prior to piperaquine phosphate treatment and approximately 3 days after treatment (or at the next scheduled visit).

Prior to exit from the clinical unit

Subjects will be allowed to leave the clinical unit 72 hours after initiation of artesunate treatment at the Investigator's discretion if they are asymptomatic and have a normal examination and no clinically significant laboratory abnormalities (Cohorts 1 and 2). For Cohort 3, subjects will be allowed to leave the clinical unit 72 hours after initiation of artesunate treatment at the Investigator's discretion. Before exit from confinement, Q-Pharm nursing staff must confirm with the Principal Investigator or Co-Investigator if any subject requires symptom-directed physical examination prior to discharge.

The following procedures will occur prior to discharge from the clinical unit:

1. Perform abbreviated physical examination (Cohort 1) or symptom-directed physical examination (Cohort 2), or symptom-directed physical examination at the Investigator's discretion.
2. Record vital signs.
3. Collect blood samples for haematology, biochemistry, malaria 18S qPCR (~72 hour post-artesunate treatment time-point), parasite lifecycle stage qRT-PCR (if required), drug response research (~72 hour post-artesunate treatment time-point; Cohort 2 at the Investigator's discretion), and dormancy detection research (~72 hour post-artesunate treatment time-point; Cohort 2 only at the Investigator's discretion).
4. Record adverse events and use of concomitant medications.

Out-patient monitoring post-artesunate treatment

Follow-up at either AM (approximately 08:00) or AM and PM (if necessary, approximately 12 hours apart) will be undertaken on an out-patient basis through visits post-confinement for clinical evaluation and blood sampling.

The following procedures will take place during these visits:

1. Collect a blood sample for malaria 18S qPCR at approximately 84 hours post-artesunate treatment. Subjects may then return morning and evening until parasite counts are <~500 parasites/mL, which is when sampling may revert to daily visits (AM) at the Investigator's discretion. When parasite counts are <~200 parasites/mL, sampling may revert to alternate day visits at the Investigator's discretion. Once the malaria 18S qPCR results are negative, or low and stable, subjects may be reviewed 3 times per week until Malarone® treatment. For Cohort 3 3D7 subjects, twice daily malaria 18S qPCR samples may be taken from approximately Day 13 to approximately Day 18 at the Investigator's discretion to better monitor recrudescence.
2. Collect blood samples for parasite lifecycle stage qRT-PCR (if required).
3. Collect blood samples for haematology and biochemistry, and urine for urinalysis on Day 14±2 and Day 18±2, or at the Investigator's discretion.

4. Perform abbreviated physical examination (Cohort 1) or symptom-directed physical examination (Cohorts 2 and 3) when signs or symptoms of malaria are identified and it is clinically indicated.
5. Record vital signs.
6. Record malaria clinical score if vital signs are abnormal or at the Investigator's discretion.
7. Record adverse events and use of concomitant medications.

Piperaqune phosphate treatment

Piperaquine phosphate will be administered if required, according to the criteria in Section 6.1.5. For Cohorts 2 and 3, K13 subjects will be treated with piperaquine phosphate on Day 11 AM (during the in-patient treatment and observation period) or earlier at the Investigator's discretion.

The following procedures will be performed prior to piperaquine phosphate treatment and approximately 3 days after treatment (or at the next scheduled visit).

1. Record vital signs.
2. Obtain a 12-lead ECG.
3. Collect blood samples for haematology, biochemistry, malaria 18S qPCR, and parasite lifecycle stage qRT-PCR (if required).
4. Record malaria clinical score if vital signs are abnormal or at the Investigator's discretion.
5. Record adverse events and use of concomitant medications.
6. Collect blood samples for drug response research (prior to piperaquine treatment only; Cohort 2 at the Investigator's discretion).

Study days for malaria transmission assays

Malaria transmission assays may be performed at the Investigator's discretion. MFA and DFA are anticipated to commence from approximately 10 days post-artesunate treatment (Cohorts 1 and 2) or at the Investigator's discretion (Cohort 3) (see Section 7.2.2 for more details). MFA and DFA may be undertaken at one time-point each prior to Malarone® treatment (Cohorts 1 and 2) or at up to two time-points each with timing at the Investigator's discretion (Cohort 3). The second DFA for Cohort 3 will be optional for the subjects as outlined in the informed Consent Form. As a guide, MFA and DFA may occur at the Investigator's discretion using parasite lifecycle stage qRT-PCR to determine probability of infectivity.

At these transmission assay time-points, vital signs will be recorded and an abbreviated physical examination (Cohort 1) or symptom-directed physical examination (Cohorts 2 and 3) will be performed if symptoms or signs of malaria are identified and it is clinically indicated. Blood may also be collected for malaria 18S qPCR and parasite lifecycle stage qRT-PCR. Thin and thick films may also be performed at the Investigator's discretion.

Rescue treatment with Malarone®

Subjects will begin a standard course of Malarone® treatment on Day 26±3 (Cohort 1), or Day Day 25±3 (Cohorts 2 and 3), or earlier at the Investigator's discretion. Subjects may take the doses at the clinical unit or at home, as determined by the Investigator. Subjects will receive a phone call or text message from clinical unit staff to check on symptoms and ensure compliance and completion of treatment following the doses taken at home.

The following procedures will be performed prior to Malarone® treatment and after completion of Malarone® treatment (or at the next scheduled visit).

1. Record vital signs.
2. Collect blood samples for haematology, biochemistry, malaria 18S qPCR, and parasite lifecycle stage qRT-PCR (if required).
3. Collect blood samples for drug response research prior to Malarone® treatment and approximately 1 to 3 days after the start of Malarone® treatment (Cohort 3 at the Investigator's discretion).
4. Record malaria clinical score if vital signs are abnormal or at the Investigator's discretion.
5. Record adverse events and use of concomitant medications.

Rescue treatment with Primacin™

Primacin™ may also be administered at the time of Malarone® treatment (Cohort 1) or on Day 23±3 or earlier at the Investigator's discretion (Cohorts 2 and 3). See Section 6.1.5.

1. Collect blood samples for drug response research on Day 23±3 prior to Primacin™ treatment and approximately 1 day after Primacin™ treatment or at the next scheduled visit (Cohort 2 at the Investigator's discretion).

7.3.4 FINAL STUDY VISIT

The following procedures will occur at the EOS visit (Day 28±3):

1. Elicit information regarding any new medical conditions or illnesses.
2. Perform complete physical examination.
3. Record vital signs.
4. Obtain a 12-lead ECG.
5. Collect urine for urinalysis.
6. Perform urine β-hCG pregnancy test for all female subjects.
7. Collect blood samples for haematology, biochemistry, malaria 18S qPCR, parasite lifecycle stage qRT-PCR (if required), serology, RBC alloantibodies, and safety serum storage.
8. Record adverse events and use of concomitant medications.

See Section 8.3 for follow-up procedures for ongoing AEs/SAEs.

7.3.5 EARLY TERMINATION VISIT

If withdrawal occurs at any stage of the study, the subject will be asked to complete an EOS evaluation. **In addition, subjects are informed on the essential requirement to complete the antimalarial drug treatment for their safety, via the Participant Information Sheet.**

Participation in an early termination evaluation by each subject is voluntary. Procedures during the early termination visit are the same as for the Day 28±3/EOS visit (Section 7.3.4).

7.3.6 UNSCHEDULED VISIT

Unscheduled visits for malaria 18S qPCR or safety monitoring may be required at the Investigator’s discretion based on parasitaemia, clinical symptoms or laboratory results. Subjects will be contacted by phone to arrange these visits. Where possible, visits will be arranged at a time that is both convenient for the subject and meets any clinical urgency as determined by the Investigator. Unscheduled visits will be documented in the source documents and eCRF.

7.3.7 SCHEDULE OF EVENTS TABLE

The Schedule of Events Table summarises the procedures to be conducted as per this protocol during screening, confinement and post-confinement. Section 7.1 and 7.2 provide detailed information on the procedures.

An experienced nurse will be in attendance at the clinical unit when subjects are on-site and the Investigator will be available within approximately 30 minutes call back if required.

In the schedule of events, evaluation may be in the morning, between 6:00 AM to 11:00 AM, and in the afternoon, between 6:00 PM to 11:00 PM, therefore separated by approximately 12 hours.

Some safety and laboratory evaluation days may vary ±2 days based on qPCR counts and clinical unit visits at the Investigator’s discretion.

Table 1: Schedule of Events

Procedure	Screening	Safety visit[a]	Malaria inoculation day	Post-inoculation phone call or text message	Malaria monitoring[b]	Artesunate treatment and clinical unit confinement[c]	Out-patient monitoring[c]	EOS visit
	D-28 to D-1	D-3 to D-1	K13 group: D0<br>3D7 group: D1	K13 group: D1 to D3<br>3D7 group: D2 to D4	K13 group: D4 to D8<br>3D7 group: D5 to D8	Approx. D9 to D12	D13 to D27	D28±3
Eligibility and safety assessments								
Informed consent & BDI	X							
Medical history, eligibility & prior medications	X		X					
Drug & alcohol screen	X		X			X[k]		
Full physical examination	X							X
Abbreviated (Cohort 1) or symptom-directed physical examination (Cohorts 2 and 3)[d]			X		X	X	X	
Vital sign assessment	X		X		X	X	X	X
ECG	X		X			X[k]	X[m]	X
Urinalysis	X	X				X[k]	X	X
Haematology & biochemistry	X	X				X[l]	X	X
G6PD testing	X							
RBC alloantibody	X							X
Serology	X							X
Pregnancy test[e]	X		X			X[k]		X
Safety serum storage			X					X
Adverse events & concomitant medications			X	X	X	X	X	X
Malaria clinical score			X		X	X	X[n]	
PK, PD, transmission, and research assessments								
Malaria 18S qPCR blood sampling			X		X	X	X	X
Parasite lifecycle stage qRT-PCR blood sampling[f]						X	X	X
Artesunate drug concentration blood sampling						X		
Malaria transmission assays (MFA and DFA)[g]							X	
Drug response research (Cohorts 2 and 3)						X	X	
Dormancy detection research (Cohorts 2 and 3)						X		

Drug treatment								
Artesunate treatment						X		
Piperaquine phosphate treatment [h]						X	X	
Malarone® treatment [i]							X	
Primacin™ treatment [j]							X	

BDI: Beck Depression Inventory, EOS: End of Study, ECG: electrocardiogram, G6PD: glucose-6-phosphate dehydrogenase, RBC: red blood cell, PK: pharmacokinetic, PD: pharmacodynamic, qPCR: quantitative polymerase chain reaction, qRT-PCR: reverse transcription qPCR, MFA: membrane feeding assay, DFA: direct feeding assay.

[a] An additional safety visit will occur between Day -3 and Day -1 to collect samples for haematology, biochemistry and urinalysis, unless screening laboratory assessments were conducted within this period.

[b] Daily visits until qPCR positive, and then twice daily visits until artesunate treatment.

[c] Approximate days based on threshold for artesunate treatment, which is expected to occur on Day 9 for both the K13 group and 3D7 group. Table indicative only. Refer to Section 7.3.3.

[d] Abbreviated physical examinations (Cohort 1) or symptom-directed physical examinations (Cohorts 2 and 3) should be performed prior to inoculation, upon admission to the clinical unit for artesunate treatment, and upon exit of the clinical unit. For Cohort 3, the symptom-directed physical examination at exit of the clinical unit will be performed at the Investigator’s discretion. At all other times, abbreviated physical examinations (Cohort 1) or symptom-directed physical examinations (Cohorts 2 and 3) will only be performed when signs or symptoms of malaria are identified and it is clinically indicated.

[e] Serum β-hCG pregnancy test (all female subjects) and follicle stimulating hormone test (post-menopausal females) at screening. Urine β-hCG pregnancy test for all female subjects at other specified time-points.

[f] If required, at the Investigator’s discretion.

[g] May be performed in selected cohorts at the Investigator’s discretion.

[h] Piperaquine phosphate will be administered if required, according to the criteria in Section 6.1.5. K13 subjects in Cohorts 2 and 3 will be treated with piperaquine phosphate on Day 11 AM or earlier at the Investigator’s discretion.

[i] Malarone® treatment will occur on Day 26±3 (Cohort 1), Day 25±3 (Cohorts 2 and 3), or earlier at the Investigator’s discretion.

[j] Primacin™ treatment if required (Cohort 1) or at Day 23±3 or earlier at the Investigator’s discretion (Cohorts 2 and 3). Refer to Section 6.1.5.

[k] At time of admission to clinical unit. K13 subjects in Cohorts 2 and 3 treated with piperaquine phosphate on Day 11 AM will have ECG testing performed prior to piperaquine treatment.

[l] At time of admission and exit from clinical unit. K13 subjects in Cohorts 2 and 3 treated with piperaquine phosphate on Day 11 AM will have blood samples collected for haematology and biochemistry prior to piperaquine treatment.

[m] Only if piperaquine phosphate treatment is required, ECG testing will occur prior to, and approximately 3 days after, piperaquine administration.

[n] Only if vital signs are abnormal, or at the Investigator’s discretion.

7.4 JUSTIFICATION FOR SENSITIVE PROCEDURES

Not applicable.

7.5 CONCOMITANT MEDICATIONS, TREATMENTS, AND PROCEDURES

Concomitant medications, treatments and procedures are those occurring from malaria inoculation until the end of the study (last visit). Those occurring prior to inoculation are classified as prior medications, treatments and procedures. Medications taken within 28 days before the malaria inoculation will be recorded as prior medication. Prior and concomitant medications, treatments and procedures permitted in this study are outlined in the inclusion/exclusion criteria (Section 5.1 and 5.2).

On inoculation day, subjects will be questioned in relation to relevant aspects of compliance with the study protocol, including drug intake since their screening visit. Details of all other drugs taken (prescription and over-the-counter, systemic and topical administration) will be recorded at this time and appropriate action taken. The Investigator may permit the use of ibuprofen (preferred) up to 1.2 g/day or paracetamol up to 4 g/day, for treatment of headache or other pain if required. Any medication taken from inoculation day to the end of the study (last visit), for treatment of a medical condition or adverse event is to be recorded in the concomitant medication pages in the eCRF. The exact dose and timing of each dose should be recorded.

7.5.1 PRECAUTIONARY MEDICATIONS, TREATMENTS, AND PROCEDURES

Not applicable.

7.6 PROHIBITED MEDICATIONS, TREATMENTS, AND PROCEDURES

Subjects will be informed and reminded of the following restrictions during recruitment, the informed consent process, and during screening and other assessments:

- Subjects should not consume grapefruit or Seville oranges from inoculation day until the end of the Malarone® treatment.
- Subjects should not consume quinine containing foods/beverages such as tonic water, lemon bitter, from inoculation day until the end of the Malarone® treatment.
- Subjects should not eat any poppy seeds in the 24 hours before the following time-points: screening, inoculation day, and day of admission for artesunate treatment.

- Subjects should not eat or drink any food or beverages that contain alcohol (e.g. beer, wine, and mixed drinks) during confinement at the clinical unit and also 24 hours prior to each alcohol breath test. Subjects should not drink more than 2 standard drinks per day from 24 hours before inoculation until the end of the Malarone® treatment.
- Subjects should not consume beverages that contain xanthine bases (e.g. Red Bull, coffee) during confinement at the clinical unit. Subjects should not consume more than 400 mg caffeine per day, equivalent to more than 4 cups of coffee, from inoculation until the end of the Malarone® treatment.
- Subjects should not use tobacco during confinement at the clinical unit. Subjects should not smoke more than 5 cigarettes or equivalent per day from inoculation until the end of the Malarone® treatment.

7.7 PROPHYLACTIC MEDICATIONS, TREATMENTS, AND PROCEDURES

Not applicable.

7.8 RESCUE MEDICATIONS, TREATMENTS, AND PROCEDURES

The rescue medications used in this study are Malarone® and Primacin™ (if required). See details in Section 6. If a subject vomits or cannot tolerate oral drugs then artesunate will be administered intravenously as described in Section 6.1.5.

7.9 SUBJECT ACCESS TO STUDY AGENT AT STUDY CLOSURE

Not applicable.

8 ASSESSMENT OF SAFETY

8.1 SPECIFICATION OF SAFETY PARAMETERS

Safety parameters that are primary endpoints include AEs and SAEs, physical examination, vital signs, clinical biochemistry, haematology, and urinalysis. Other safety parameters monitored during this study include ECGs, serology, RBC alloantibody testing, and malaria clinical score. See Sections 7.1 and 7.2 for details of these procedures. All safety parameters will be recorded in the eCRF.

8.1.1 DEFINITION OF ADVERSE EVENTS (AE)

An AE is any adverse change, i.e., any unfavourable and unintended sign (including an abnormal laboratory finding), symptom or disease that occurs in a subject during the course of the study, whether or not considered by the Investigator as related to study treatment.

A treatment-emergent adverse event (TEAE) is an event that emerges following treatment with the investigational medicinal products (IMPs; artesunate and piperaquine phosphate if administered), having been absent pre-treatment, or worsens relative to the pre-treatment state.

AEs include:

- A new symptom, sign or medical condition.
- A disease or medical condition detected or diagnosed during the course of the study even though it may have been present prior to the start of the study.
- An exacerbation of a pre-existing medical condition or disease.
- An increase in frequency or intensity of a pre-existing episodic disease or medical condition.
- Continuous persistent disease or symptoms present at study start that worsen following the start of the study.
- An abnormal assessment (e.g. change on physical examination, ECG findings) if it represents a clinically significant finding that was not present at study start or worsened during the course of the study.
- An abnormal laboratory test result if it represents a clinically significant finding, symptomatic or not, which was not present at study start or worsened during the course of the study or led to dose reduction, interruption or permanent discontinuation of study treatment.
- Mosquito bite reactions (if DFA are performed) regardless of clinical significance.

Borderline abnormal laboratory findings and other objective assessments should NOT be routinely captured and reported as AEs, as they will be collected and analysed separately. However, abnormal laboratory findings or other objective measurements that meet the following criteria should be captured and reported in the AE section of the eCRF:

- The result meets the criteria for reporting as an SAE
- The test result is associated with accompanying symptoms, and/or
- It requires additional diagnostic testing or medical/surgical intervention, and/or
- It leads to a change in trial dosing, or discontinuation from the trial, significant additional concomitant drug treatment, or other therapy, and/or
- It is considered by the Investigator or Sponsor to be clinically significant or represent a clinically significant change from baseline.

Merely repeating an abnormal test, in the absence of any of the above conditions, does not constitute an AE. Any abnormal test result that is determined to be an error does not require reporting as an AE.

If a clinical diagnosis is associated with an abnormal laboratory finding, the relevant AE should be recorded as the diagnosis rather than the incidental laboratory finding (e.g. “hepatitis” should be recorded rather than “elevated transaminases”).

Surgical procedures themselves are not AEs; they are therapeutic measures for conditions which may, or may not, be AEs.

8.1.2 DEFINITION OF SERIOUS ADVERSE EVENTS (SAE)

A serious adverse event (SAE) is defined as an AE which fulfils at least one of the following criteria:

- Results in death.
- Is life-threatening
  - The term "life-threatening" in the definition of "serious" refers to an event in which the subject was at immediate risk of death at the time of the event; it does not refer to an event which hypothetically might have caused death if it was more severe.
- Requires inpatient hospitalisation or prolongs existing hospitalisation, unless this is for:
  - Elective or pre-planned treatment or standard monitoring for a pre-existing condition that is unrelated to the study and has not worsened since the start of the study.
  - Cosmetic surgery, or for social reasons, or respite care in the absence of any deterioration in the subject’s general condition.
- Results in persistent or significant disability/incapacity.
- Is a congenital abnormality/birth defect.
- Is considered medically important
  - Medical and scientific judgement should be exercised in deciding whether other AEs are to be considered serious, such as important medical events that may not be immediately life-threatening but may jeopardise the subject or may require intervention to prevent one of the other outcomes listed in the definition above. Examples of such events are: intensive treatment in an emergency room or at home for allergic bronchospasm; blood dyscrasias; convulsions that do not result in hospitalisation; development of drug dependency or drug abuse.
- Constitutes a possible Hy’s Law case

- Hy's Law case is defined as a subject with any value of alanine or aspartate aminotransferase greater than 3 × ULN together with an increase in total bilirubin to a value greater than 2 × ULN and not associated to an alkaline phosphatase value greater than 2 × ULN (FDA Guidance on Drug Induced Liver Injury: Premarketing Clinical Evaluation [2009]).

A **Suspected Unexpected Serious Adverse Reaction (SUSAR)** is any SAE where a causal relationship with the malaria challenge agents (K13 or 3D7) or the IMPs (artesunate or piperaquine phosphate) is at least a reasonable possibility, but the event is not listed in the Investigator Brochure(s) and/or Summary of Product Characteristics.

8.1.3 DEFINITION OF UNANTICIPATED PROBLEMS (UP)

Not applicable.

8.2 CLASSIFICATION OF AN ADVERSE EVENT

8.2.1 SEVERITY OF EVENT

In addition to determining whether an AE fulfils the criteria for a SAE or not, the severity of AEs experienced by study subjects will be graded according to the Common Terminology Criteria for Adverse Events v4.03 published 14 June 2010 (CTCAE v4.03). This guidance provides a common language to describe levels of severity, to analyse and interpret data, to scale the aggregate AE score, and to articulate the clinical significance of all AEs.

The severity of AEs will be graded as follows:

Grade 1: Mild; asymptomatic or mild symptoms; clinical or diagnostic observations only; intervention not indicated.

Grade 2: Moderate; minimal, local or non-invasive intervention indicated; limiting age-appropriate instrumental activities of daily living.

Grade 3: Severe or medically significant but not immediately life-threatening; hospitalisation or prolongation of hospitalisation indicated; disabling; limiting self-care activities of daily living.

Grade 4: Life-threatening consequences; urgent intervention indicated.

Grade 5: Death related to AE.

A mild, moderate, or severe AE may or may not be serious (see Section 8.1.2). These terms are used to describe the intensity of a specific event. Medical judgment should be used on a case-by-case basis.

Seriousness, rather than severity assessment, determines the regulatory reporting obligations.

For guidance for assigning severity of the malaria, the purpose-designed malaria clinical score will be used (Section 7.1.1).

8.2.2 RELATIONSHIP TO STUDY AGENT

The Investigator will decide if AEs are related to any of the study agents or procedures. Where possible, a distinction should be made between events considered related to the malaria challenge agents or the IMPs and those related to protocol-mandated procedures.

The assessment of causality will be made using the following definitions:

Unrelated

This category is applicable to those AEs which are judged to be clearly and incontrovertibly due to extraneous causes (disease, environment, etc.) and do not meet the criteria for the relationship listed under unlikely, possible or probable.

Unlikely

In general, this category is applicable to an AE which meets the following criteria (must have the first two):

1. It does **not** follow a reasonable temporal sequence from administration of any of the study agents.
2. It may readily have been produced by the subject's clinical state, environment or toxic factors, or other modes of therapy administered to the subject.
3. It does not follow a known pattern of response to the study agents.
4. It does not reappear or worsen when any of the study agents are re-administered.

Possible

This category applies to those AEs in which the connection with any of the study agents appears unlikely but cannot be ruled out with certainty. An AE may be considered possible if or when (must have the first two):

1. It follows a reasonable temporal sequence from administration of any of the study agents.

2. It may have been produced by the subject's clinical state, environment or toxic factors, or other modes of therapy administered to the subject.
3. It follows a known pattern of response to any of the study agents.

Probable

This category applies to those AEs which are considered, with a high degree of certainty, to be related to the study agents. An AE may be considered probable if (must have the first three):

1. It follows a reasonable temporal sequence from administration of any of the study agents.
2. It cannot be reasonably explained by the known characteristics of the subject's clinical state, environment or toxic factors, or other modes of therapy administered to the subject.
3. It disappears or decreases on cessation or reduction in dose.
4. It follows a known pattern of response to any of the study agents.
5. It reappears on re-administration.

Cohort 3 - Optional, exploratory medical imaging study

For subjects in Cohort 3 enrolled in the optional exploratory study "Advanced medical imaging in subpatent malaria: a second pilot study" all AEs deemed related to [^{18}F] fluoro-2-deoxy-2-D-glucose radiotracer administration or other imaging procedures will be documented in an exploratory study AE log including diagnosis, severity, toxicity, date of onset and resolution, and any action taken. These AEs will also be recorded in the main study subject clinic file as per main study processes. AEs possibly related to this exploratory study will be reported by the Investigator as "PET study related". Further details regarding AEs are located in the exploratory study protocol.

8.2.3 EXPECTEDNESS

An AE is regarded as an unexpected event if its nature or severity is not consistent with the applicable reference safety information (Investigator's Brochures or approved manufacturer's prescribing information for marketed drugs). Events that add significant information on the specificity, severity or frequency of previously described reactions, are also regarded as unexpected.

Expected AEs from the malaria infection are listed in Appendix 4 and the Investigator's Brochures for K13 and 3D7 challenge agents [33, 34]. Expected AEs from the antimalarial drugs used are listed in the piperaquine Investigator's Brochure [46], artesunate WHO Public

Assessment Report 2011, Malarone® Consumer Medicine Information, and Primacin™ Consumer Medicine Information (see Appendix 1).

8.3 TIME PERIOD AND FREQUENCY FOR EVENT ASSESSMENT AND FOLLOW-UP

All AEs must be documented and followed up by the Investigator until:

- The event is resolved, or
- No further medically relevant information in relation to the event can be expected, and
- The Investigator considers it justifiable to terminate the follow-up.

Events that are unresolved at the time of the subject's last follow-up visit should continue to be followed up by the Investigator for as long as medically indicated. The Sponsor retains the right to request additional information for any subject with ongoing AE(s)/SAE(s) at the end of the study, if judged necessary.

All AEs should be treated appropriately. The Investigator will decide upon the appropriate action to be taken in response to an AE, which may include one or more of the following:

- No action taken (i.e. further observation only).
- Dosing with the IMP is withheld and the subject withdrawn from the study.
- Administration of a concomitant medication.
- Hospitalisation or prolongation of current hospitalisation (event to be reported as an SAE).
- Other.

In a case of occurrence of SAEs, regardless of whether or not it is judged to be challenge agent- or antimalarial drug-related, the subject will receive appropriate care under clinical supervision until all the symptoms of the SAEs have diminished or resolved and the subject's condition improved.

For ongoing AEs, care will be provided for a period of time as specified in the clinical site work instruction protocols. However, if the nature of the ongoing AE is determined by the Investigator as not being inoculum- or antimalarial drug-associated, the subject will be advised to visit his/her own general practitioner for further clinical care that he might require.

8.4 REPORTING PROCEDURES

8.4.1 ADVERSE EVENT REPORTING

It is the Investigator's responsibility to document and report all AEs occurring in the study whether spontaneously reported by the subject, observed by the Investigator (either directly or by

laboratory or other assessments), or elicited by general questioning. The period of observation for collection of AEs extends from the time of inoculation up to the end of the study; these AEs must be recorded on specific AE pages of the eCRF. Events reported prior to this will be recorded as medical history, unless the symptoms worsen during the study.

The following information should be recorded for all AEs:

- Description of the AE.
- Dates and times of onset and resolution of the event.
- Duration of the event in hours.
- The time of onset relative to the administration of the challenge agent and/or the IMP.
- Seriousness of the AE (SAE or not).
- Severity of the event.
- Action taken in response to the event (including treatment required).
- Outcome of the event.
- Relationship of the event to the study agents or procedures (causality assessment), including challenge agent, artesunate, piperaquine, rescue medication, mosquito feeding, or any other treatment or procedure conducted during the study.

Changes in the severity of an AE will be documented to allow assessment of the duration of the event at each level of severity. AEs changing severity will be documented as separate AEs; those worsening in severity will be considered unresolved and those reducing in severity will be considered resolving. AEs characterised as intermittent require documentation of onset and duration at each episode.

All malaria-specific AEs will be tabulated and results graded according to a purpose-designed malaria clinical score table (Section 7.1.1).

8.4.2 SERIOUS ADVERSE EVENT REPORTING

The Investigator will take immediate appropriate action in response to SAEs to ensure subject safety and in an attempt to identify the causes of the event. Review and reporting of SAEs will be in accordance with the Sponsor's and Q-Pharm's SAE reporting procedures. The Investigator will notify the Sponsor representative (QIMR Berghofer Regulatory Affairs), the QIMR Berghofer HREC, and the IMM, of the occurrence of any SAE within 24 hours of becoming aware of the event. The notification should be in writing by email or fax, and documented on a standard SAE report form.

<u>Sponsor representative:</u>
QIMR Berghofer Regulatory Affairs

E-mail: clinical.trials@qimrberghofer.edu.au

<u>QIMR Berghofer HREC contact:</u>
QIMR Berghofer HREC Secretary
E-mail: HREC.Secretariat@qimrberghofer.edu.au
Phone: +61 7 3362 0117

<u>Independent medical monitor:</u>
Prof. Dennis Shanks
E-mail: Dennis.SHANKS@defence.gov.au
Phone: +61 (0)7 3332 4931

The Investigator will complete a follow-up SAE report within 14 days of the SAE, unless no further information is available in which case the follow-up report will be provided as soon as new information becomes available. The follow-up SAE report will be sent to QIMR Berghofer Regulatory Affairs, the QIMR Berghofer HREC, and the IMM. Other supporting documents may be requested by these parties and will be provided by the Investigator or a delegate as soon as possible.

Any SAE that meets the criteria of a SUSAR (Section 8.1.2) will be reported to the TGA by QIMR Berghofer Regulatory Affairs in accordance with the Sponsor's reporting procedures.

8.4.3 UNANTICIPATED PROBLEM REPORTING

Not applicable.

8.4.4 EVENTS OF SPECIAL INTEREST

Not applicable.

8.4.5 REPORTING OF PREGNANCY

Female subjects should be instructed to notify the Investigator if they become pregnant within 4 weeks of antimalarial drug administration. The occurrence of pregnancy in any of the female subjects at any stage during the study will be recorded in the subject's eCRF.

Whenever possible, a pregnancy should be followed to term, any premature termination reported, and the status of the mother and child should be reported to QIMR Berghofer after delivery, and

the progress of the child should be followed and reported to QIMR Berghofer for at least 6 months after delivery. The pregnancy outcome will be recorded in the eCRF.

8.5 STUDY HALTING RULES

See Section 5.5.

8.6 SAFETY OVERSIGHT

Safety oversight will be undertaken by the Principal Investigator and the IMM who will serve as an independent expert to advise on clinical safety specifically in the situation where expert external advice is required regarding the need for administration of alternative/rescue antimalarial treatment in the circumstance of suboptimal response.

The SRT will be responsible for decisions related to the safety of subjects and the continuation of the study. The role and composition of the SRT is outlined in the study specific SRT Charter. The SRT will be composed of the Principal Investigator, IMM, and a physician with expertise in clinical trials or infectious diseases. The SRT will review the clinical and laboratory safety data as well as the recorded AEs and SAEs. The SRT makes recommendations to the Sponsor. These recommendations are approved by the SRT Chair who signs a letter of recommendation that is sent to the Principal Investigator and the Sponsor.

A review of the safety, tolerability, and parasitaemia data from the preceding cohort will be conducted by the SRT prior to inoculation of the next cohort. Data from at least 3 weeks post-inoculation will be required for each review. The SRT meeting conducted after Cohort 2 inoculation will have analysis available for the parasitaemia data from both Cohort 1 and 2 to determine whether a third cohort is needed to complete the primary objective of comparing artesunate parasite clearance profiles (Section 10.4.7.2).

Additionally, the SRT may meet to assess any events that trigger the stopping rules or as needed to provide a recommendation and findings to QIMR Berghofer HREC and the Principal Investigator, in accordance with the approved SRT Charter.

Whether at a scheduled or unscheduled meeting, the SRT will consider safety signals to determine whether or not they can recommend that the study continue.

The SRT will also review the safety and tolerability data from the study after completion of the last cohort.

9 CLINICAL MONITORING

It will be the Sponsor's responsibility to ensure that the study is monitored in accordance with the requirements of GCP. The conduct of the study will be reviewed internally by the clinical unit (Q-Pharm) in accordance with their standard procedures and work instructions, and GCP guidelines. The study will be monitored according to the Sponsor's SOPs [53] and all protocol deviations will be reported to the Sponsor (see Section 14.3 for more detail). Protocol deviations that impact subject safety or data integrity will also be reported to the QIMR Berghofer HREC.

During the study, appointed study monitor(s) (on behalf of the Sponsor) will visit the site to check completeness of subject records, accuracy of eCRF entries, adherence to the protocol and to GCP, progress of enrolment, and to ensure that study agents were stored, dispensed, and accounted for according to specifications. Key study personnel are required to be available to assist the study monitor during these visits.

The Investigator will be required to give the monitor access to all relevant source documents to confirm their consistency with the eCRF entries. The Sponsor will require full verification for the presence of informed consent, adherence to the inclusion/exclusion criteria, documentation of SAEs, and the recording of data that is used for all primary and safety variables. Additional checks of the consistency of the source data with the eCRFs will be performed according to the study-specific monitoring plan. No information in source documents about the identity of the subjects will be disclosed.

10 STATISTICAL CONSIDERATIONS

10.1 STATISTICAL AND ANALYTICAL PLANS

The following sections describe the statistical analysis as it is foreseen when the study is being planned. A detailed Statistical Analysis Plan (SAP) will be finalised and approved prior to database lock and will provide details of all analyses to be performed as well as the format of listings and tables to be provided for completion of the Clinical Study Report (CSR). Any deviations from the SAP will be described and justified in the final CSR.

10.2 STATISTICAL HYPOTHESES

For the parasite clearance primary endpoint, the null hypothesis is that the clearance rate of the K13 and 3D7 isolates are the same in response to artesunate treatment. The alternative hypothesis is that artesunate will clear parasitaemia from subjects challenged with K13 at a 30% slower rate compared to subjects challenged with 3D7.

For the safety primary endpoint, we hypothesise that inoculating with ~2 800 viable *P. falciparum* K13 or 3D7 parasite-infected RBCs and treating with artesunate at the treatment threshold is safe, results in no SAEs, and does not cause severe malaria symptoms.

10.3 ANALYSIS DATASETS

The safety analysis dataset will include all subjects who receive the malaria inocula. This population will be used to analyse all safety data as well as demographic and baseline data. Safety data will be presented by challenge agent group (K13 or 3D7).

The populations used for analysis of PK, PD and transmission data will be defined in the SAP.

10.4 DESCRIPTION OF STATISTICAL METHODS

10.4.1 GENERAL APPROACH

The study will use parallel group comparison.

Continuous data will be summarised using descriptive statistics (mean and standard deviation, or median and interquartile range). Categorical data will be presented using N and % (using the number of subjects without missing data in the calculation).

10.4.2 ANALYSIS OF THE PRIMARY EFFICACY ENDPOINT(S)

The parasite clearance profiles of *P. falciparum* K13 and 3D7 after administration of artesunate will be characterised by the parasite reduction ratio and parasite clearance $t_{1/2}$. A t-test will be used to compare the slope of the clearance curves for K13 and 3D7 isolates.

10.4.3 ANALYSIS OF THE SECONDARY ENDPOINT(S)

Not applicable.

10.4.4 SAFETY ANALYSES

The overall number and percentage of subjects with at least one AE (and SAE) will be tabulated over the entire study period. All AE data will be summarised by challenge agent and treatment

received, MedDRA system organ class and preferred term, and maximum severity. Vital signs, routine safety laboratory data and ECG parameters will be summarised descriptively by challenge agent and treatment received, and by time-point. Both absolute values and change from baseline (inoculation, artesunate treatment and piperaquine treatment) will be presented.

10.4.5 ADHERENCE AND RETENTION ANALYSES

Not applicable.

10.4.6 BASELINE DESCRIPTIVE STATISTICS

Demographic data will be summarised by descriptive statistics and will include total number of observations (n), mean, standard deviation (SD) and range for continuous variables and number and percentages with characteristics for dichotomous variables.

The subject disposition will be summarised. Study completion, study withdrawals, exclusions and violations will be summarised and the reasons for withdrawal, exclusions and violations will be listed.

Descriptive statistics will be provided by challenge agent received. Medical history, current medical conditions, results of laboratory screening tests, drug tests and any other relevant baseline information will be listed by challenge agent received and subject. Prior medications taken at any stage from 4 weeks prior to inoculation will be listed by challenge agent received. Concomitant medication taken during the study period will be listed by challenge agent received.

10.4.7 PLANNED INTERIM ANALYSES

10.4.7.1 SAFETY REVIEW

A review of the safety and tolerability data from the preceding cohort will be conducted by the SRT prior to inoculation of the next cohort. Data from at least 3 weeks post-inoculation will be required for each review. See Section 5.5 for study stopping rules.

10.4.7.2 EFFICACY REVIEW

A review of the parasitaemia data from the preceding cohort will be conducted by the SRT prior to inoculation of the next cohort. Data from at least 3 weeks post-inoculation will be required for each review. If a statistically significant difference in parasite clearance of K13 compared to 3D7

has already been demonstrated in the cohort being reviewed, the subsequent cohort may not be conducted. The SRT will consult with the study statistician to reach this decision.

The SRT meeting conducted after Cohort 2 inoculation will also have analysis available for the parasitaemia data from both Cohort 1 and 2 to determine whether a third cohort is needed to complete the primary objective of comparing artesunate parasite clearance profiles (Section 10.5).

10.4.8 ADDITIONAL SUB-GROUP ANALYSES

Not applicable.

10.4.9 MULTIPLE COMPARISON/MULTIPLICITY

Not applicable.

10.4.10 TABULATION OF INDIVIDUAL RESPONSE DATA

All individual subject data will be listed by measure and time-point.

10.4.11 EXPLORATORY ANALYSES

No formal statistical analyses are planned for exploratory endpoints.

If transmission assays have been performed, the prevalence and/or intensity of oocyst infection will be determined in the blood fed mosquitoes.

10.5 SAMPLE SIZE

The sample size required to complete the primary objective of comparing the artesunate parasite clearance profiles of K13 and 3D7 isolates was estimated. Background information used to estimate the required sample size for this study came from previous IBSM studies with the 3D7 isolate and fast acting antimalarial drugs, as well as artesunate clearance data from artemisinin-resistant isolates [3]. nQuery Adviser was used to calculate the sample size required. Satterthwaite t-test of equal means but unequal variances was used with a 2-sided $\alpha=0.05$, an

estimated 30% mean difference in decay rate between the K13 and 3D7 isolates, and a power of 80%. The estimated sample size using a 2:1 ratio of K13 to 3D7 was 18 to 9 subjects.

If the mean difference in parasite clearance between the K13 and 3D7 isolates is greater than 30%, fewer subjects may be required. Therefore, a review of the parasitaemia data from the preceding cohort will be conducted by the SRT prior to inoculation of the next cohort (Section 10.4.7.2).

Refer to Section 5.3 for recruitment strategies.

10.6 MEASURES TO MINIMIZE BIAS

10.6.1 ENROLLMENT/ RANDOMIZATION/ MASKING PROCEDURES

The study will be conducted in up to 27 subjects, in up to 3 cohorts. Up to 18 subjects will receive the K13 challenge agent and up to 9 subjects will receive the 3D7 challenge agent, to achieve a ratio of 2:1 over the whole study.

The randomisation for Cohort 1 was to be performed in the rate of 2:1 for K13 to 3D7 challenge agents, for a cohort size of 9. However, only 6 subjects received the challenge agents in Cohort 1 (3 K13 and 3 3D7). Up to 21 subjects distributed over Cohorts 2 and 3 will receive either the K13 or 3D7 challenge agent, to reach up to 27 subjects in total.

Subjects will be screened according to Section 7.3.1, and a list of eligible and suitable subjects will be compiled. These subjects will be randomised into 3 groups prior to Day 0: group 1 (K13 subjects), group 2 (3D7 subjects), and group 3 will be the remaining subjects in a sequenced list (the reserves). Group 1 subjects will be inoculated on Day 0 with the K13 challenge agent, and group 2 subjects will be inoculated on Day 1 with the 3D7 challenge agent. If any of the group 1 or group 2 subjects do not continue to meet eligibility on the designated inoculation day, a reserve subject from group 3 will replace them in order of the sequenced list, i.e. reserve 1 will be the first replacement, followed by reserve 2, etc.

The randomisation schedule will be generated by a statistician using a validated system. A copy of the randomisation schedule will be sent to the clinical unit pharmacist and clinical unit project manager.

Due to feasibility, this study will not be blinded.

10.6.2 EVALUATION OF SUCCESS OF BLINDING

Not applicable.

10.6.3 BREAKING THE STUDY BLIND/SUBJECT CODE

Not applicable.

11 SOURCE DOCUMENTS AND ACCESS TO SOURCE DATA/DOCUMENTS

The Investigator will maintain source documents for each subject in the study. Information entered into eCRFs will be traceable to these source documents in the subject's file. The Investigator must certify that the data entered into the eCRFs are complete and accurate. After database lock, the Investigator will retain copies of the subject data for archiving at the investigational site.

Upon request, the Investigator(s)/institution(s) will permit direct access to source data/documents for trial-related monitoring, audits, Ethics Committee review, and regulatory inspection(s) by the Sponsor (or their appropriately qualified delegate) and Regulatory Authorities. Direct access includes examination, analysis, verification and reproduction of records and reports that are important to the evaluation of the trial.

12 QUALITY ASSURANCE AND QUALITY CONTROL

Data management, including the development and management of a secure database, will be performed in accordance with regulatory requirements. Clinical Network Services will review the data entered into the eCRFs by clinical unit staff for completeness and accuracy. A formal querying process will be followed whereby the data management team will request the clinical site staff to clarify any apparent erroneous entries or inconsistencies and will request additional information from the clinical site as required.

Medical history/current medical conditions and adverse events will be coded using the Medical dictionary for regulatory activities (MedDRA) terminology (version 19.0 or later).

After all data have been captured and reviewed, all queries have been resolved with the site and any protocol non-compliances that were identified during the data management processes have been confirmed by the site, the database will be declared to be complete and accurate, it will be locked and made available for data analysis. Any changes to the database after that time may only be made by the data manager, in consultation with the Sponsor and in accordance with documented database unlock and relock procedures.

Clinical monitoring will be conducted as described in Section 9.

Audits may be carried out by Sponsor quality assurance representatives, local authorities or authorities to whom information on this study has been submitted. All documents pertinent to this study must be made available for such inspections after adequate notice of intention to audit.

13 ETHICS/PROTECTION OF HUMAN SUBJECTS

13.1 ETHICAL STANDARD

The study will be conducted in accordance with the protocol approved by the QIMR Berghofer HREC, the principles of the Declaration of Helsinki (Recommendations guiding Medical Doctors in Biomedical Research Involving Human Participants, Fortaleza, Brazil 2013), the NHMRC National Statement on Ethical Conduct in Human Research (2007, updated May 2015) [54] and the Note for Guidance on Good Clinical Practice – Annotated with TGA Comments (CPMP/ICH/135/95), as adopted by the Australian Therapeutic Goods Administration (July 2000) [55].

The Investigator will minimise any discomfort experienced by subjects during the study. The only invasive procedures will be the intravenous inoculation of the malaria inoculum and the blood collection by cannulation/venepuncture. The maximum amount of blood to be collected from an individual in the study would be up to approximately 467 mL for subjects involved in both the main study and the additional optional exploratory studies (i.e. a volume which will not be more than the equivalent to a standard blood bank donation and it will be taken over at least a 4-8 week interval). For subjects who do not choose to participate in the optional exploratory studies, the total volume of blood will be up to approximately 301.5 mL (Cohort 1) or up to approximately 421 mL (Cohort 2) if MFA is performed (Appendix 3). For Cohort 3 subjects who do not choose to participate in the optional exploratory studies, the total volume of blood for the whole study will be up to approximately 460 mL (Appendix 3). The total volume of blood drawn from each subject will not exceed 450 mL in any given 30 day period. This volume includes allowance for unscheduled safety and qPCR assessments that may be required at the discretion of the Principal Investigator or the Sponsor to ensure subject safety. If the total blood volume is estimated to exceed 450 mL in a 30 day period due to the timing of unscheduled safety or qPCR assessments, the blood volume for the optional exploratory studies will be decreased.

13.2 ETHICAL REVIEW

The protocol, participant information sheets and informed consent forms will be reviewed by the QIMR Berghofer HREC, and no study activities will be initiated prior to approval from the HREC. All amendments and addenda to the protocol and consent forms will similarly be submitted to the QIMR Berghofer HREC for approval prior to their implementation.

Changes to the final study protocol can only be made with the prior consent of the Principal Investigator, the Sponsor and the HREC. All such changes must be attached to, or incorporated into, the final protocol, and communicated to all relevant members of Q-Pharm staff and, if appropriate, to study subjects. All deviations from this study protocol will be included in the trial master file and included in the CSR. An assessment of the significance of each protocol deviation will be given in the CSR. All deviations/amendments will be reported to Sponsor. The different types of amendments are discussed below.

<u>Non-substantial amendment</u>

Administrative or logistical minor changes require a non-substantial amendment. Such changes include but are not limited to changes in study staff or contact details (e.g., Sponsor instead of CRO monitors) or minor changes in the packaging or labelling of study drug. An amendment deemed to be non-substantial must have no ethical implications.

The implementation of a non-substantial amendment may be done without notification to the HREC. It does not require their approval or to be signed by the Investigator. The HREC will be notified for these non-substantial changes in the next submission round, with the annual study report or study close out report which ever comes sooner that will be submitted to HREC.

<u>Substantial amendment</u>

Significant changes require a substantial amendment. Significant changes include but are not limited to: new data affecting the safety of subjects, change of the objectives/endpoints of the study, eligibility criteria, dose regimen, study assessments/procedures, treatment or study duration, with or without the need to modify the Participant Information Sheet and Informed Consent.

Substantial amendments are to be approved by the HREC. The implementation of a substantial amendment can only occur after formal approval by the HREC and must be signed by the Investigator.

<u>Urgent amendment</u>

An urgent amendment might become necessary to preserve the safety of the subjects included in the study. The requirements for approval should in no way prevent any immediate action being taken by the Investigator or the Sponsor in the best interests of the subjects. Therefore, if deemed necessary, an Investigator can implement an immediate change to the protocol for safety reasons. This means that, exceptionally, the implementation of urgent amendments will occur before submission to and approval by the HREC.

In such cases, the Investigator must notify the Sponsor within 24 hours. A related substantial amendment will be written within 10 working days and submitted to the HREC, together with a

description of the steps that have already been taken in regard to implementation of this amendment.

HREC approval of future research

In the event that the Principal Investigator or the Sponsor want to perform testing on the samples that is not described in the protocol, additional HREC approval will be sought. This may be done if a subject consents to blood storage for use in future research (Section 13.3.1)

13.3 INFORMED CONSENT PROCESS

13.3.1 CONSENT/ASSENT AND OTHER INFORMATIONAL DOCUMENTS PROVIDED TO SUBJECTS

The Participant Information Sheet and informed Consent Form describes in detail the study agents, study procedures, and risks. Subjects will also receive an Informed Consent for Blood Storage and an option to grant permission to be contacted about future studies involvement.

Subjects will also receive the product insert for artesunate, and the Consumer Medicine Information for Malarone® and Primacin™ (if required; Appendix 1). Subjects may also receive the Consumer Medicine Information for any other registered antimalarial agents in the event that these are required.

Subjects may also receive separate Participant Information Sheets and informed Consent Forms for genetic testing and/or optional exploratory research studies if required.

13.3.2 CONSENT PROCEDURES AND DOCUMENTATION

During the initial screening visit/recruitment, potential subjects will read the Participant Information Sheet. The Investigator or clinical unit staff will explain the study via the Participant Information Sheet and the potential subjects will be encouraged to ask questions. Individuals willing to be considered for inclusion in the study will sign and date the informed Consent Form in the presence of an Investigator. Subjects will be given a copy of their signed informed Consent Form. Once the subject has consented to the study, the trial-specific screening activities may commence. See Section 7.3.1 for further details.

13.4 SUBJECT AND DATA CONFIDENTIALITY

Subjects will be informed that their data will be held on file by Q-Pharm and that these data may be viewed by staff of Q-Pharm (including, where necessary, staff of Q-Pharm other than the named Investigators).

Upon request, the Investigator(s)/institution(s) will permit direct access to data and documents for trial-related monitoring, audits, Ethics Committee review, and regulatory inspection(s) by the Sponsor (or their appropriately qualified delegates) and Regulatory Authorities (see Section 11).

Subjects will also be informed that a report of the study will be submitted to the Sponsor and may also be submitted to government agencies and perhaps for publication, but that they will only be identified in such reports by their study identification number, and their gender and age. The Investigator undertakes to hold all personal information in confidence.

Subjects will be informed that samples collected for the purposes described in the protocol will be sent to Sponsor's nominated national or international laboratory for assessment.

If a subject consents for the optional genetic research, only authorised people will have access to their results. The sample will have all identifiers (e.g. name and personal details) removed and replaced by a code so that it is re-identifiable.

13.4.1 RESEARCH USE OF STORED HUMAN SAMPLES, SPECIMENS OR DATA

Samples and data collected during this study will be used to achieve the study objectives. Genetic testing may be performed if subjects are asked to participate in the optional genetic research and sign the informed Consent Form for that testing.

Samples and data will be stored according to Q-Pharm and QIMR Berghofer SOPs, and access will be limited to authorised personnel. Biological samples will be retained for the time required to complete analysis, and may then be discarded.

13.5 FUTURE USE OF STORED SPECIMENS

As part of the study, safety serum samples will be stored indefinitely at Q-Pharm/QIMR Berghofer for retrospective safety assessments that may later be indicated. Subjects consent to this storage and the use of the sample for safety assessments, when they sign the informed Consent Form for the study.

For all other samples, consent must be obtained from the subjects to store and use their samples for future research. This is done via the Informed Consent for Blood Storage that subjects receive during recruitment/screening. Subjects can decide if they want their samples to be used for future research or have their samples destroyed at the end of the study. A subject's decision can be changed at any time prior to the end of the study by notifying the study doctors or nurses in writing. However, if a subject consents to future use and some of their blood has already been used for research purposes, the information from that research may still be used.

Any future research using the stored samples that is beyond the current study will be reviewed by the QIMR Berghofer HREC (Section 13.2). All samples will be stored at QIMR Berghofer in accordance with the laboratory SOPs. The Investigator will ensure that confidentiality will be maintained continuously in all future research that involves use of these samples. The vials containing the samples of the consented subjects will be coded and the identifying information will not be released to any unauthorised third party. The subjects can also choose (via the Informed Consent for Blood Storage Form) for the samples to be re-labelled with only the study number, malaria strain and visit. No genetic testing will be performed on the stored samples without obtaining consent from the subjects. The stored samples will not be sold or used directly for production of any commercial product. There are no benefits to subjects in the collection, storage and subsequent research use of their samples. Reports about future research done with subject samples will NOT be kept in their health records, but a subject's samples may be kept with the study records or in other secure areas.

14 DATA HANDLING AND RECORD KEEPING

14.1 DATA COLLECTION AND MANAGEMENT RESPONSIBILITIES

Each subject will have a Clinic File (source data) and eCRF (for protocol specific data) into which relevant data will be recorded. All recording will be done only in black ink. Corrections will only be made by drawing a single line through the incorrect entry, writing the correction in the nearest practicable space, and initialing and dating the correction. Correction fluids are not allowed.

A log of names, signatures and initials of all staff authorised to enter data into a subject's Clinic File and eCRF will be kept. Upon completion of each study visit, all eCRFs will be reviewed internally by the clinical unit for omissions or apparent errors so that these can be corrected without delay. Any corrections made after the review and signature of the Principal Investigator will be noted in the audit trail and will required reauthorisation (electronic sign off) by the Principal Investigator.

14.2 STUDY RECORDS RETENTION

All source data, clinical records and laboratory data relating to the study will be retained in the archive of the clinical unit (Q-Pharm) for a minimum of 15 years after the completion of the study. Data will be available for retrospective review or audit by arrangement with the Chief Executive Officer of the clinical unit. Written agreement from the Sponsor must precede destruction of the same.

14.3 PROTOCOL DEVIATIONS

A protocol deviation is any change, divergence or departure from the study design or procedures defined in the protocol.

All protocol deviations will be documented in the trial master file and included in the CSR. An assessment of the significance of each protocol deviation will be discussed in the CSR.

All protocol deviations will be reported to the Sponsor. Protocol deviations that are not approved by the Sponsor and QIMR Berghofer HREC prior to implementation should be reported to the Sponsor by the clinical unit. Protocol deviations should be recorded on a protocol deviation log by the clinical unit staff. Protocol deviation logs should be submitted to the Sponsor and QIMR Berghofer HREC via inclusion with the annual report.

Protocol deviations that impact subject safety or data integrity will be reported by the clinical site to both the Sponsor and the QIMR Berghofer HREC as early as possible, but within 7 days. A protocol deviation report form will be used for this purpose.

14.4 PUBLICATION AND DATA SHARING POLICY

The data management, statistical and medical writing team appointed by the Sponsor will collaborate to provide a detailed CSR upon conclusion of the study. This will include appendices of all tables and listings generated during the analyses of data. The tables and listings will be provided by Clinical Network Services. The Sponsor undertakes to ensure that all safety observations made during the conduct of the trial are documented in this report.

Publication and reporting of results and outcomes of this trial will be accurate and honest, undertaken with integrity and transparency and in accordance with the relevant clauses outlined in the QIMR Berghofer Policy on Criteria for Authorship [56]. QIMR Berghofer and the Principal Investigator have a responsibility to ensure that results of scientific interest arising from the clinical trials are appropriately published and disseminated. Publication of results will be subjected to fair peer-review. Authorship will be given to all persons providing significant input into the conception, design, and execution or reporting of the research according to the QIMR Berghofer Policy on Criteria of Authorship. No person who is an author, consistent with this definition, will be excluded as an author without his/her permission in writing. Authorship will be discussed between researchers prior to study commencement (or as soon as possible thereafter) and reviewed whenever there are changes in participation. Acknowledgment will be given to collaborating institutions and hospitals and other individuals and organisations providing finance or facilities. All conflicts arising through disputes about authorship will be reviewed by the QIMR Berghofer Director.

In any press releases, publications or presentations, MMV's financial contribution to the study and its participation in the collaboration shall be expressly acknowledged. QIMR Berghofer agrees that MMV will be entitled to access all the de-identified clinical trial data upon completion of the trial. Data will not be released publicly until the manuscript is accepted for publication. In the case of no publication, information will only be released to the public and media in accordance with the QIMR Berghofer Corporate Media Strategy Policy [57]. However, the Investigator undertakes not to make any publication or release pertaining to the study and/or results of the study without the Sponsor's prior written consent, being understood that the Sponsor will not unreasonably withhold its approval. The Sponsor has the right to publish the results of the study at any time.

The Investigator shall not use the name(s) of the Sponsor and/or of its employees in advertising or promotional material or publication without the prior written consent of the Sponsor. The Sponsor shall not use the name(s) of the Investigator and/or the collaborators in advertising or promotional material or publication without having received his/her and/or their prior written consent(s).

QIMR Berghofer will ensure that the key design elements of this protocol are posted in a publicly accessible database such as Australian New Zealand Clinical Trials Registry (ANZCTR) or Clinicaltrials.gov. In addition, upon study completion and finalisation of the study report the results of this trial will be either submitted for publication in an open access journal and/or posted in a publicly accessible database of clinical trial results.

Results from the exploratory analyses will be reported in a separate document to the main study clinical study report.

15 STUDY ADMINISTRATION

15.1 STUDY LEADERSHIP

See Section 1 for key roles.

15.2 LIABILITY/INDEMNITY/INSURANCE

The study Sponsor will ensure sufficient insurance is available to enable it to indemnify and hold the Investigator(s) and relevant staff as well as any hospital, institution, Ethics Committee or the like, harmless from any claims for damages for unexpected injuries, including death, that may be caused by the subject's participation in the study but only to the extent that the claim is not caused by the fault or negligence of the subjects or Investigator(s). The Sponsor adheres to the

guidelines of Medicines Australia for injury resulting from participation in a company sponsored trial, including the provision of 'No-fault clinical trial insurance'.

16 CONFLICT OF INTEREST POLICY

No conflicts of interest are applicable in this study.

17 LITERATURE REFERENCES

1. World Health Organization (WHO). World Malaria Report 2016. *http://www.who.int/malaria/publications/world-malaria-report-2016/report/en/*, accessed 23 July 2017, **2016**.

2. Ashley EA, White NJ. Artemisinin-based combinations. Curr Opin Infect Dis **2005**; 18:531-6.

3. Dondorp AM, Nosten F, Yi P, et al. Artemisinin resistance in *Plasmodium falciparum* malaria. The New England journal of medicine **2009**; 361:455-67.

4. Woodrow CJ, White NJ. The clinical impact of artemisinin resistance in Southeast Asia and the potential for future spread. FEMS Microbiol Rev **2016**.

5. Straimer J, Gnädig NF, Witkowski B, et al. K13-propeller mutations confer artemisinin resistance in *Plasmodium falciparum* clinical isolates. Science **2015**; 347:428-31.

6. Rockett RJ, Tozer SJ, Peatey C, et al. A real-time, quantitative PCR method using hydrolysis probes for the monitoring of Plasmodium falciparum load in experimentally infected human volunteers. Malaria Journal **2011**; 10:48-.

7. ANZCTR Trial ID: ACTRN12611001203943. An experimental study to characterize molecular signatures during early Plasmodium falciparum blood stage infection in healthy male volunteers., **2011**.

8. ANZCTR Trial ID: ACTRN12612000323820. An experimental study to characterize the effectiveness of Lariam(Registered Trademark) (Mefloquine) against early Plasmodium falciparum blood stage infection in healthy volunteers **2012**.

9. ANZCTR Trial ID: ACTRN12612000814875. An experimental study to characterize the effectiveness of OZ439 against early Plasmodium falciparum blood stage infection in healthy volunteers, **2012**.

10. ANZCTR Trial ID: ACTRN12613000533796, . A Phase I/Ib Study to Investigate the Safety, Tolerability and Pharmacokinetic Profile of DSM265 in Healthy Subjects and to Assess the Antimalarial Activity of DSM265 in Healthy Subjects with an Induced Blood Stage Plasmodium falciparum Infection. **2013**.

11. ANZCTR Trial ID: ACTRN12613000565741. An experimental study to characterize the effectiveness of Piperaquine against early Plasmodium falciparum blood stage infection in healthy volunteers. **2013**.

12. ANZCTR Trial ID: ACTRN12613000698774. An experimental study to characterize the effectiveness of griseofulvin against early plasmodium falciparum blood stage infection in healthy volunteers. **2013**.

13. ANZCTR Trial ID: ACTRN12613001040752. An experimental study to characterize the effectiveness of ferroquine against early Plasmodium falciparum blood stage infection in healthy volunteers. **2013**.

14. ANZCTR Trial ID: ACTRN12614000781640, . A proof-of-concept study to assess the effect of ACT-451840 against early Plasmodium falciparum blood stage infection in healthy subjects. **2014**.

15. Bijker EM, Bastiaens GJ, Teirlinck AC, et al. Protection against malaria after immunization by chloroquine prophylaxis and sporozoites is mediated by preerythrocytic immunity. Proceedings of the National Academy of Sciences of the United States of America **2013**; 110:7862-7.

16. Cheng Q, Lawrence G, Reed C, et al. Measurement of *Plasmodium falciparum* growth rates *in vivo*: a test of malaria vaccines. American Journal of Tropical Medicine and Hygiene **1997**; 57:495–500.

17. ClinicalTrials.gov ID: NCT02281344. A Proof-of-concept Study to Assess the Effect of MMV390048 Against Early Plasmodium Falciparum Blood Stage Infection in Healthy Participants, **2014**.

18. ClinicalTrials.gov ID: NCT02389348. A Proof-of-concept Study to Assess the Effect of a Range of Doses of Combined Therapy With OZ439 and DSM265 Against Early Plasmodium Falciparum Blood Stage Infection in Healthy Participants, **2015**.

19. ClinicalTrials.gov ID: NCT02543086. A Phase1 Interventional Sequential Single Site Study to Characterize the Effectiveness of Oral KAE609 in Reducing Asexual &Sexual Blood-stage P. Falciparum Following Inoculation in Healthy Volunteers & Subsequent Infectivity to Mosquitoes, **2015**.

20. Duncan CJA, Sheehy SH, Ewer KJ, et al. Impact on Malaria Parasite Multiplication Rates in Infected Volunteers of the Protein-in-Adjuvant Vaccine AMA1-C1/Alhydrogel+CPG 7909. PLoS One **2011**; 6.

21. Lawrence G, Cheng QQ, Reed C, et al. Effect of vaccination with 3 recombinant asexual-stage malaria antigens on initial growth rates of *Plasmodium falciparum* in non-immune volunteers. Vaccine **2000**; 18:1925-31.

22. McCarthy JS, Sekuloski S, Griffin PM, et al. A pilot randomised trial of induced blood-stage Plasmodium falciparum infections in healthy volunteers for testing efficacy of new antimalarial drugs. PLoS One **2011**; 6:e21914.

23. Payne RO, Milne KH, Elias SC, et al. Demonstration of the Blood-Stage Plasmodium falciparum Controlled Human Malaria Infection Model to Assess Efficacy of the P. falciparum Apical Membrane Antigen 1 Vaccine, FMP2.1/AS01. The Journal of infectious diseases **2016**.

24. Pombo DJ, Lawrence G, Hirunpetcharat C, et al. Immunity to malaria after administration of ultra-low doses of red cells infected with Plasmodium falciparum. Lancet **2002**; 360:610-7.

25. Sanderson F, Andrews L, Douglas AD, Hunt-Cooke A, Bejon P, Hill AV. Blood-stage challenge for malaria vaccine efficacy trials: a pilot study with discussion of safety and potential value. The American journal of tropical medicine and hygiene **2008**; 78:878-83.

26. ClinicalTrials.gov ID: NCT02431637. Blood Stage Challenge Study to Asses Mosquito Transmissibility in Participants Inoculated With Plasmodium Falciparum. **2015**.

27. ClinicalTrials.gov ID: NCT02431650. A Proof-Of-Concept Study to Assess the Effectiveness of OZ439 as a Gametocytocidal and Transmission Blocking Agent in Experimental P. Falciparum Infection, **2015**.

28. ClinicalTrials.gov ID: NCT02573857. A Phase Ib Study to Characterise the Antimalarial and Transmission Blocking Activity of a Single Dose of DSM265 or OZ439 in Healthy Subjects With Induced Blood Stage Plasmodium Falciparum or Plasmodium Vivax Infection, **2015**.

29. Krause A, Dingemanse J, Mathis A, Marquart L, Mohrle JJ, McCarthy JS. Pharmacokinetic/pharmacodynamic modelling of the antimalarial effect of Actelion-451840 in an induced blood stage malaria study in healthy subjects. British journal of clinical pharmacology **2016**; 82:412-21.

30. McCarthy JS, Baker M, O'Rourke P, et al. Efficacy of OZ439 (artefenomel) against early Plasmodium falciparum blood-stage malaria infection in healthy volunteers. The Journal of antimicrobial chemotherapy **2016**; 71:2620-7.

31. McCarthy JS, Ruckle T, Djeriou E, et al. A Phase II pilot trial to evaluate safety and efficacy of ferroquine against early Plasmodium falciparum in an induced blood-stage malaria infection study. Malar J **2016**; 15:469.

32. Pasay CJ, Rockett R, Sekuloski S, et al. Piperaquine Monotherapy of Drug-Susceptible Plasmodium falciparum Infection Results in Rapid Clearance of Parasitemia but Is Followed by the Appearance of Gametocytemia. The Journal of infectious diseases **2016**; 214:105-13.

33. Investigator's Brochure: Blood stage *Plasmodium falciparum* challenge inoculum *P. falciparum* 3D7 (BSPC) v8.0 ed: QIMR Berghofer Medical Research Institute, **2017**.

34. Investigator's Brochure: *In vitro* expanded blood stage *Plasmodium falciparum* Cam3.II^{R539T} (K13) artemisinin-resistant challenge inoculum. 2.0 ed: QIMR Berghofer Medical Research Institute, **2017**.

35. ANZCTR Trial ID: ACTRN12617000244303. An experimental study to characterise the *in vivo* safety and infectivity of a *Plasmodium falciparum* Cam3.II^{R539T} (K13) artemisinin-resistant isolate in healthy participants, **2017**.

36. Wockner L, Hoffman I, Marquart L, Khoury D, O'Rourke P, McCarthy JS. Estimation of *Plasmodium falciparum* parasite multiplication rate in the induced blood stage malaria model and comparison with other controlled human malaria infection systems. In preparation **2017**.

37. Saunders D, Khemawoot P, Vanachayangkul P, et al. Pharmacokinetics and pharmacodynamics of oral artesunate monotherapy in patients with uncomplicated *Plasmodium falciparum* malaria in western Cambodia. Antimicrob Agents Chemother **2012**; 56:5484-93.

38. Warrell DA, Gilles HM. Essential malariology. 4 ed. London: Arnold., **2002**.

39. Chalon S, Akakpo S, Duparc S, Shapiro T, Griffin P, McCarthy J. Poster presentation: Moderate and severe LFT elevations in controlled human *P. falciparum* malaria infection model: recent experience, literature review and mechanistic hypotheses. American Society of Tropical Medicine and Hygiene: 65th Annual Meeting, **16 November 2016**.

40. WHO Public Assessment Report: Artesunate 50 mg tablets (MA044). *https://extranet.who.int/prequal/WHOPAR/ma044*, **2011**.

41. Myint HY, Ashley EA, Day NP, Nosten F, White NJ. Efficacy and safety of dihydroartemisinin-piperaquine. Transactions of the Royal Society of Tropical Medicine and Hygiene **2007**; 101:858-66.

42. Ratcliff A, Siswantoro H, Kenangalem E, et al. Two fixed-dose artemisinin combinations for drug-resistant falciparum and vivax malaria in Papua, Indonesia: an open-label randomised comparison. Lancet **2007**; 369:757-65.

43. Karunajeewa H, Lim C, Hung TY, et al. Safety evaluation of fixed combination piperaquine plus dihydroartemisinin (Artekin) in Cambodian children and adults with malaria. British journal of clinical pharmacology **2004**; 57:93-9.

44. Mytton OT, Ashley EA, Peto L, et al. Electrocardiographic safety evaluation of dihydroartemisinin piperaquine in the treatment of uncomplicated falciparum malaria. The American journal of tropical medicine and hygiene **2007**; 77:447-50.

45. European Medicines Agency. Eurartesim: European Public Assessment Report - Product Information, **Updated 2016**.

46. Investigator's Brochure: Piperaquine phosphate: Medicines for Malaria Venture, **2012**.

47. MIMS Abbreviated Prescribing Information: Malarone® Tablets (250/100), **2013**.

48. Boucher & Muir Pty Ltd. Consumer Medicine Information: Primacin®, **2017**.

49. Boucher & Muir Pty Ltd. Product Information: Primacin® Tablets. **2017**.

50. GlaxoSmithKline. Consumer Medicine Information: Malarone® Tablets (250/100). *https://au.gsk.com/en-au/products/our-prescription-medicines-and-vaccines/medicines/malarone/*, **2015**.

51. Karunaweera ND, Carter R, Grau GE, Mendis KN. Demonstration of anti-disease immunity to Plasmodium vivax malaria in Sri Lanka using a quantitative method to assess clinical disease. The American journal of tropical medicine and hygiene **1998**; 58:204-10.

52. Hanpithakpong W, Kamanikom B, Dondorp AM, et al. A liquid chromatographic-tandem mass spectrometric method for determination of artesunate and its metabolite dihydroartemisinin in human plasma. Journal of chromatography B, Analytical technologies in the biomedical and life sciences **2008**; 876:61-8.

53. QIMR Berghofer Medical Research Institute. Clinical Trial SOPs *https://oldintranet.qimr.edu.au/intranet/corporate/ethics/clinicaltrials-sops.html*, **Accessed 2017**.

54. National Health and Medical Research Council. National statement on ethical conduct in human research 2007 2007 ed, **2007**:1 - 95.

55. Australian Therapeutic Goods Administration. Note for Guidance on Good Clinical Practice (GCP) (CPMP/ICH/135/95). *www.tga.gov.au/sites/default/files/ich13595an.pdf*, **2000**.

56. QIMR Berghofer Medical Research Institute. Policy on the Criteria for Authorship. *https://oldintranet.qimr.edu.au/intranet/corporate/policy/2.11_AuthorshipCriteria.pdf*, **2008, reviewed 2013**.

57. QIMR Berghofer Medical Research Institute. Media Relations Policy. *https://oldintranet.qimr.edu.au/intranet/corporate/policy/1.4_Media%20Relations%20Policy.pdf*, **2010**.

APPENDIX

Appendix 1: Product information and consumer medicine information

Malarone®

- Product Information (TGA, updated August 2016)
- Consumer Medicine Information (TGA, updated December 2015)

Primacin™

- Product Information (TGA, updated February 2017)
- Consumer Medicine Information (TGA, updated February 2017)

Artesunate

- WHO Public Assessment Report (2011)

Appendix 2: Dosing table for artesunate tablets

Weight range inclusive (kg) – based on weight at screening	Artesunate dose (mg)	Number of artesunate 50 mg tablets
50-59	100	2
60-69	100	2
70-79	150	3
80-89	150	3
90-99	200	4
100	200	4

Appendix 3: Total blood volume

These tables are indicative and may vary based on qPCR levels and safety follow-up samples. Additional blood samples may be taken for unscheduled safety and qPCR assessments as required by the Investigator, provided the total volume taken during the study does not exceed 450 mL during any period of 30 consecutive days.

Cohort 1:

**Procedure**	**Sample**	**Volume per sample (mL)**	**No. samples per subject**	**Total volume per subject (mL)**
Laboratory Safety Assessment	Haematology (including G6PD)	2	8	16
		4	2	8
	Biochemistry	5	10	50
	Serology	3.5	2	7
	Safety serum storage	5	2	10
	RBC alloantibody	4	2	8
Bioanalysis	PK analysis	2	13	26
Cannulation	Discard	2	23	46
Malaria Monitoring	Malaria 18S qPCR	2	36	72
	Parasite lifecycle stage qRT-PCR	Up to 2.5	9	22.5
Malaria Transmission	MFA	Up to 36	Up to 1	36
**Main study total (mL)**				**301.5**
**Optional genetic research**				**4**
**Main study + optional genetic research**				**305.5**

Cohort 2:

<table>
<tr><th>Procedure</th><th>Sample</th><th>Volume per sample (mL)</th><th>No. samples per subject</th><th>Total volume per subject (mL)</th></tr>
<tr><td rowspan="6">Laboratory Safety Assessment</td><td rowspan="2">Haematology (including G6PD and RBC alloantibody)</td><td>2</td><td>7</td><td>14</td></tr>
<tr><td>4</td><td>3</td><td>12</td></tr>
<tr><td>Biochemistry</td><td>5</td><td>10</td><td>50</td></tr>
<tr><td>Serology</td><td>3.5</td><td>2</td><td>7</td></tr>
<tr><td>Safety serum storage</td><td>5</td><td>2</td><td>10</td></tr>
<tr><td colspan="4"></td></tr>
<tr><td>Bioanalysis</td><td>PK analysis</td><td>2</td><td>13</td><td>26</td></tr>
<tr><td>Cannulation</td><td>Discard</td><td>2</td><td>25</td><td>50</td></tr>
<tr><td rowspan="2">Malaria Monitoring</td><td>Malaria 18S qPCR</td><td>2</td><td>38</td><td>76</td></tr>
<tr><td>Parasite lifecycle stage qRT-PCR</td><td>2</td><td>9</td><td>18</td></tr>
<tr><td>Malaria Transmission</td><td>MFA</td><td>Up to 36</td><td>Up to 1</td><td>36</td></tr>
<tr><td rowspan="4">Exploratory Research</td><td rowspan="3">Drug response research</td><td>10</td><td>Up to 3</td><td>30</td></tr>
<tr><td>8</td><td>Up to 2</td><td>16</td></tr>
<tr><td>2</td><td>Up to 8</td><td>16</td></tr>
<tr><td>Dormancy detection research</td><td>30</td><td>2</td><td>60</td></tr>
<tr><td colspan="4">Main study total (mL)</td><td>421</td></tr>
<tr><td colspan="4">Optional genetic research</td><td>4</td></tr>
<tr><td colspan="4">Main study + optional genetic research</td><td>425</td></tr>
</table>

Cohort 3:

**Procedure**	**Sample**	**Volume per sample (mL)**	**No. samples per subject**	**Total volume per subject (mL)**
Laboratory Safety Assessment	Haematology	2	10	20
	G6PD	2	1	2
	RBC alloantibody	2	2	4
	Biochemistry	5	10	50
	Serology	3.5	2	7
	Safety serum storage	5	2	10
Bioanalysis	PK analysis	2	13	26
Cannulation	Discard	2	29	58
Malaria Monitoring	Malaria 18S qPCR and parasite lifecycle stage qRT-PCR	2 mL or 3 mL or 2 x 2 mL depending on the time-point	49	Up to 123
Malaria Transmission	MFA	Up to 36	Up to 2	Up to 72
Exploratory Research	Drug response research	8	2	16
		4	2	8
		2	2	4
	Dormancy detection research	30	2	60
**Main study total (mL)**				**460**
**Optional genetic research**				**4**
**Main study + optional genetic research**				**464**

Appendix 4: Symptoms and signs of malaria

Following challenge via the intravenous malaria parasite inoculation and during the post-challenge period, the following signs and symptoms of malaria will be monitored:

Signs of malaria

- Fever (oral temperature of ≥38°C)
- Chills/shivering/rigors
- Tachycardia
- Hypotension

Symptoms of malaria

- Headache
- Myalgia (muscle ache)
- Arthralgia (joint ache)
- Fatigue/lethargy
- Malaise (general discomfort/uneasiness)
- Sweating/hot spells
- Anorexia
- Nausea
- Vomiting
- Abdominal discomfort

Appendix 5: Version History

Version	Date	Author(s)/Reviewer(s)	Significant Revisions
1.0	25 Jul 2017	R. Watts	Initial version
1.1	11 Aug 2017	R. Watts	Clarified when and how IV artesunate can be given. Added exclusion criterion 11 and Beck Depression Inventory to assess depression at screening.
2.0	25 Jan 2018	R. Watts	Modified the wording regarding cohort size so there are no restrictions on cohort size in order to achieve up to 27 subjects over the whole study. Modified timing of piperaquine, Malarone® and Primacin™ treatments. Added exploratory objectives for drug response research and dormancy detection research. See summary of changes document for more details.
3.0	23 July 2018	R. Watts	Updated exploratory objective time-points. Updated malaria 18S qPCR time-points and sample volumes. Increased the number of membrane feeding assays and direct feeding assays for Cohort 3 to up to 2 time-points each per subject. Added a parallel, optional, observational exploratory study "Advanced medical imaging in subpatent malaria: a second pilot study" which is outlined in a separate protocol and informed Consent Form. See summary of changes document for more details.

16.1.2 Sample case report form

IBM Clinical Development Blank CRFs

Study Name	QP17C11 (PROD)
Generated	15-NOV-17 05:49:19 GMT

Table of Contents

Initial revision of study........ 1

Schedule........ 1

Screening........ 9

- Visit Date........ 9
- Informed Consent........ 10
- Inclusion / Exclusion Summary........ 11
- Inclusion / Exclusion Criteria........ 12
- Demographics........ 13
- Physical Exam........ 15
- Physical Exam (extra)........ 18
- Height and Weight........ 19
- ECG........ 20
- Vital Signs........ 21
- Lab Sample Collection Summary........ 23
- Lab Sample Collection........ 24
- Lab Test Results........ 25
- Lab Test Assessment........ 26
- Serology........ 30
- FSH........ 31

Pregnancy Test.......... 32
Urinalysis.......... 33
Urine Microscopy Assessment.......... 37
Urine Drug Screen.......... 38
Alcohol Breath Test.......... 40
Safety Visit.......... 41
Visit Date.......... 41
Lab Sample Collection Summary.......... 42
Lab Sample Collection.......... 43
Lab Test Results.......... 44
Lab Test Assessment.......... 45
Urinalysis.......... 49
Urine Microscopy Assessment.......... 53
Inoculum.......... 54
Visit Date.......... 54
Inclusion / Exclusion Summary.......... 55
Inclusion / Exclusion Criteria.......... 56
Urine Drug Screen.......... 57
Alcohol Breath Test.......... 59
Randomisation.......... 60

Abb. Physical Exam........ 61
Physical Exam (extra)........ 64
ECG........ 65
Vitals Signs........ 66
Pregnancy Test........ 68
Inoculum Administration........ 69
Safety Serum Storage........ 70
PCR Collection........ 71
Telephone Contact 1........ 72
Phone Call........ 72
Telephone Contact 2........ 73
Phone Call........ 73
Telephone Contact 3........ 74
Phone Call........ 74
Malaria Monitoring........ 75
Visit Date........ 75
Abb. Physical Exam........ 76
Physical Exam (extra)........ 79
Vital Signs........ 80
PCR Collection........ 82

Admission.. 83
Visit Date.. 83
Alcohol Breath Test.. 84
Urine Drug Screen.. 85
Abb. Physical Exam.. 87
Physical Exam (extra).. 90
ECG.. 91
Vital Signs.. 92
Lab Sample Collection Summary.. 94
Lab Sample Collection.. 95
Lab Test Result.. 96
Lab Test Assessment.. 97
Urinalysis.. 101
Urine Microscopy Results.. 105
Pregnancy Test.. 106
IP administration.. 107
IP administration (IV).. 108
PK Collection.. 109
Admission 1.. 111
Visit Date.. 111

Abb. Physical Exam........ 112
Physical Exam (extra)........ 115
Vital Signs........ 116
Admission 2........ 118
Visit Date........ 118
Abb. Physical Exam........ 119
Physical Exam (extra)........ 122
Vital Signs........ 123
Admission 3........ 125
Visit Date........ 125
Abb. Physical Exam........ 126
Physical Exam (extra)........ 129
Vital Signs........ 130
Lab Sample Collection Summary........ 132
Lab Sample Collection........ 133
Lab Test Result........ 134
Lab Test Assessment........ 135
Safety Monitoring........ 139
Visit Date........ 139
Abb. Physical Exam........ 140

Physical Exam (extra).......... 143

Vital Signs.......... 144

ECG.......... 146

Lab Sample Collection Summary.......... 147

Lab Sample Collection.......... 148

Lab Test Result.......... 149

Lab Test Assessment.......... 150

Urinalysis.......... 154

Urine Microscopy Results.......... 158

PCR Collection.......... 159

Feeding Summary.......... 160

MFA/DFA.......... 161

End of Study.......... 162

Visit Date.......... 162

Physical Exam.......... 163

Physical Exam (extra).......... 166

ECG.......... 167

Vital Signs.......... 168

Lab Sample Collection Summary.......... 170

Lab Sample Collection.......... 171

Lab Test Result.. 172
Lab Test Assessment.. 173
Pregnancy Test.. 177
Urinalysis.. 178
Urine Microscopy Assessment.. 182
Serology.. 183
Safety Serum Storage.. 184
PCR Collection.. 185
Phone Call.. 186
Phone Call.. 186
Common Forms.. 187
AE Summary.. 187
Adverse Events.. 188
Concomitant Medications Summary.. 199
Concomitant Medication.. 200
Medical History Summary.. 211
Medical History.. 212
Comments Summary.. 213
Comments.. 214
Clinical Score.. 215

Protocol Deviations Summary.......... 220

Protocol Deviations.......... 221

Rescue Medication.......... 222

PCR Collection.......... 223

Completion Page.......... 226

Death.......... 227

Unscheduled.......... 228

Visit Date.......... 228

Unscheduled Visit.......... 229

Temperature.......... 230

Vitals Signs.......... 231

Vitals Signs (Supine to Standing).......... 233

Physical Exam.......... 235

Abb. Physical Exam.......... 238

Physical Exam (extra).......... 241

Pregnancy Test.......... 242

Lab Sample Collection.......... 243

Lab Test Result.......... 244

Lab Test Assessment.......... 245

Alcohol Breath Test.......... 249

- Urine Drug Screen........ 250
- Urinalysis........ 252
- Urine Microscopy Assessment........ 256
- PCR Collection........ 257
- Feeding........ 258
- PK........ 259
- ECG........ 260
- Piperaquine Dosing........ 261
- Genetic Testing........ 262

Reconsent........ 263
- Reconsent........ 263

Early Termination Visit........ 264
- Visit Date........ 264
- Physical Exam........ 265
- Physical Exam (extra)........ 268
- ECG (single)........ 269
- Vital Signs (single)........ 270
- Lab Sample Collection Summary........ 272
- Lab Sample Collection........ 273
- Lab Test Result........ 274

Lab Test Assessment.......... 275
Serology.......... 279
Pregnancy Test.......... 280
Urinalysis.......... 281
Urine Microscopy Assessment.......... 285
Safety Serum Storage.......... 286
PCR Collection.......... 287

Initial revision of study

Schedule

Table

Unique Identifier	Visit	Visit - Page	Visit ID, Page ID	Notes
visit-10	Screening		10	Added: On Add Subject
		Visit Date	10,180	
		Informed Consent	10,10	
		Inclusion / Exclusion Summary	10,140	
		Inclusion / Exclusion Criteria	10,150	Repeats, Maximum= Unlimited<br>Added by visit schedule rule(s) #103
		Demographics	10,260	
		Physical Exam	10,40	
		Physical Exam (extra)	10,50	Repeats, Maximum= Unlimited<br>Added by visit schedule rule(s) #94
		Height and Weight	10,240	
		ECG	10,60	
		Vital Signs	10,70	
		Lab Sample Collection Summary	10,80	
		Lab Sample Collection	10,90	Repeats, Maximum= Unlimited<br>Added by visit schedule rule(s) #95
		Lab Test Results	10,160	Added by visit schedule rule(s) #96, #97
		Lab Test Assessment	10,170	Repeats, Maximum= Unlimited<br>Added by visit schedule rule(s) #104
		Serology	10,230	
		FSH	10,250	Added by visit schedule rule(s) #205
		Pregnancy Test	10,100	Added by visit schedule rule(s) #201
		Urinalysis	10,120	
		Urine Microscopy Assessment	10,200	Repeats, Maximum= Unlimited<br>Added by visit schedule rule(s) #189
		Urine Drug Screen	10,130	

Unique Identifier	Visit	Visit - Page	Visit ID, Page ID	Notes
		Alcohol Breath Test	10,210	
visit-180	Safety Visit		180	Added: On Add Subject
		Visit Date	180,80	
		Lab Sample Collection Summary	180,10	
		Lab Sample Collection	180,20	Repeats, Maximum= Unlimited<br>Added by visit schedule rule(s) #181
		Lab Test Results	180,30	Added by visit schedule rule(s) #182
		Lab Test Assessment	180,40	Repeats, Maximum= Unlimited<br>Added by visit schedule rule(s) #183
		Urinalysis	180,50	
		Urine Microscopy Assessment	180,70	Repeats, Maximum= Unlimited<br>Added by visit schedule rule(s) #190
visit-20	Inoculum		20	Added: On Add Subject
		Visit Date	20,130	
		Inclusion / Exclusion Summary	20,10	
		Inclusion / Exclusion Criteria	20,20	Repeats, Maximum= Unlimited<br>Added by visit schedule rule(s) #106
		Urine Drug Screen	20,100	
		Alcohol Breath Test	20,160	
		Randomisation	20,170	
		Abb. Physical Exam	20,30	
		Physical Exam (extra)	20,40	Repeats, Maximum= Unlimited<br>Added by visit schedule rule(s) #107
		ECG	20,50	
		Vitals Signs	20,60	Repeats, Maximum=2
		Pregnancy Test	20,90	Added by visit schedule rule(s) #202
		Inoculum Administration	20,110	
		Safety Serum Storage	20,120	
		PCR Collection	20,180	
visit-30	Telephone Contact 1		30	Added: On Add Subject
		Phone Call	30,10	
visit-40	Telephone Contact 2		40	Added: On Add Subject

Unique Identifier	Visit	Visit - Page	Visit ID, Page ID	Notes
		Phone Call	40,10	
visit-50	Telephone Contact 3		50	Added: On Add Subject
		Phone Call	50,10	
visit-60	Malaria Monitoring		60	Added: Add Subject and By User<br>Repeats, Maximum=Unlimited
		Visit Date	60,70	
		Abb. Physical Exam	60,10	
		Physical Exam (extra)	60,20	Repeats, Maximum= Unlimited<br>Added by visit schedule rule(s) #114
		Vital Signs	60,30	
		PCR Collection	60,110	
visit-70	Admission		70	Added: On Add Subject
		Visit Date	70,120	
		Alcohol Breath Test	70,160	
		Urine Drug Screen	70,80	
		Abb. Physical Exam	70,10	
		Physical Exam (extra)	70,20	Repeats, Maximum= Unlimited<br>Added by visit schedule rule(s) #119
		ECG	70,30	
		Vital Signs	70,40	Repeats, Maximum=3
		Lab Sample Collection Summary	70,50	
		Lab Sample Collection	70,60	Repeats, Maximum= Unlimited<br>Added by visit schedule rule(s) #126
		Lab Test Result	70,130	Added by visit schedule rule(s) #127
		Lab Test Assessment	70,140	Repeats, Maximum= Unlimited<br>Added by visit schedule rule(s) #129
		Urinalysis	70,170	
		Urine Microscopy Results	70,180	Repeats, Maximum= Unlimited<br>Added by visit schedule rule(s) #191
		Pregnancy Test	70,70	Added by visit schedule rule(s) #203
		IP administration	70,90	
		IP administration (IV)	70,200	Repeats, Maximum= Unlimited<br>Added by visit schedule rule(s) #197

Unique Identifier	Visit	Visit - Page	Visit ID, Page ID	Notes
		PK Collection	70,190	
visit-80	Admission 1		80	Added: On Add Subject
		Visit Date	80,70	
		Abb. Physical Exam	80,10	
		Physical Exam (extra)	80,20	Repeats, Maximum= Unlimited<br>Added by visit schedule rule(s) #131
		Vital Signs	80,30	Repeats, Maximum=3
visit-90	Admission 2		90	Added: On Add Subject
		Visit Date	90,60	
		Abb. Physical Exam	90,10	
		Physical Exam (extra)	90,20	Repeats, Maximum= Unlimited<br>Added by visit schedule rule(s) #138
		Vital Signs	90,30	Repeats, Maximum=3
visit-100	Admission 3		100	Added: On Add Subject
		Visit Date	100,70	
		Abb. Physical Exam	100,10	
		Physical Exam (extra)	100,20	Repeats, Maximum= Unlimited<br>Added by visit schedule rule(s) #145, #153
		Vital Signs	100,30	
		Lab Sample Collection Summary	100,50	
		Lab Sample Collection	100,60	Repeats, Maximum= Unlimited<br>Added by visit schedule rule(s) #146
		Lab Test Result	100,80	Added by visit schedule rule(s) #147
		Lab Test Assessment	100,90	Repeats, Maximum= Unlimited<br>Added by visit schedule rule(s) #148
visit-110	Safety Monitoring		110	Added: Add Subject and By User<br>Repeats, Maximum=Unlimited
		Visit Date	110,60	
		Abb. Physical Exam	110,10	
		Physical Exam (extra)	110,20	Repeats, Maximum= Unlimited<br>Added by visit schedule rule(s) #149
		Vital Signs	110,30	
		ECG	110,120	

Unique Identifier	Visit	Visit - Page	Visit ID, Page ID	Notes
		Lab Sample Collection Summary	110,40	
		Lab Sample Collection	110,50	Repeats, Maximum= Unlimited<br>Added by visit schedule rule(s) #151
		Lab Test Result	110,70	Added by visit schedule rule(s) #150
		Lab Test Assessment	110,80	Repeats, Maximum= Unlimited<br>Added by visit schedule rule(s) #152
		Urinalysis	110,130	
		Urine Microscopy Results	110,140	Repeats, Maximum= Unlimited<br>Added by visit schedule rule(s) #192
		PCR Collection	110,90	
		Feeding Summary	110,100	
		MFA/DFA	110,110	Repeats, Maximum= Unlimited<br>Added by visit schedule rule(s) #193
visit-120	End of Study		120	Added: On Add Subject
		Visit Date	120,100	
		Physical Exam	120,10	
		Physical Exam (extra)	120,20	Repeats, Maximum= Unlimited<br>Added by visit schedule rule(s) #154
		ECG	120,30	
		Vital Signs	120,40	
		Lab Sample Collection Summary	120,50	
		Lab Sample Collection	120,60	Repeats, Maximum= Unlimited<br>Added by visit schedule rule(s) #155
		Lab Test Result	120,110	Added by visit schedule rule(s) #156
		Lab Test Assessment	120,120	Repeats, Maximum= Unlimited<br>Added by visit schedule rule(s) #158
		Pregnancy Test	120,70	Added by visit schedule rule(s) #204
		Urinalysis	120,80	
		Urine Microscopy Assessment	120,140	Repeats, Maximum= Unlimited<br>Added by visit schedule rule(s) #194
		Serology	120,160	
		Safety Serum Storage	120,90	
		PCR Collection	120,150	

Unique Identifier	Visit	Visit - Page	Visit ID, Page ID	Notes
visit-140	Phone Call		140	Added: By User After Add Subject<br>Repeats, Maximum=Unlimited
		Phone Call	140,10	
visit-150	Common Forms		150	Added: On Add Subject
		AE Summary	150,10	
		Adverse Events	150,20	Repeats, Maximum= Unlimited<br>Added by visit schedule rule(s) #177
		Concomitant Medications Summary	150,30	
		Concomitant Medication	150,40	Repeats, Maximum= Unlimited<br>Added by visit schedule rule(s) #178
		Medical History Summary	150,140	
		Medical History	150,150	Repeats, Maximum= Unlimited<br>Added by visit schedule rule(s) #188
		Comments Summary	150,50	
		Comments	150,120	Repeats, Maximum= Unlimited<br>Added by visit schedule rule(s) #179
		Clinical Score	150,60	Repeats, Maximum= Unlimited
		Protocol Deviations Summary	150,70	
		Protocol Deviations	150,80	Repeats, Maximum= Unlimited<br>Added by visit schedule rule(s) #180
		Rescue Medication	150,160	Repeats, Maximum= Unlimited
		PCR Collection	150,100	
		Completion Page	150,110	
		Death	150,130	Added by visit schedule rule(s) #187
visit-130	Unscheduled		130	Added: By User After Add Subject<br>Repeats, Maximum=Unlimited
		Visit Date	130,170	
		Unscheduled Visit	130,10	
		Temperature	130,260	Repeats, Maximum= Unlimited<br>Added by visit schedule rule(s) #199
		Vitals Signs	130,150	Repeats, Maximum= Unlimited<br>Added by visit schedule rule(s) #160
		Vitals Signs (Supine to Standing)	130,30	Added by visit schedule rule(s) #161

Unique Identifier	Visit	Visit - Page	Visit ID, Page ID	Notes
		Physical Exam	130,40	Added by visit schedule rule(s) #162
		Abb. Physical Exam	130,240	Added by visit schedule rule(s) #
		Physical Exam (extra)	130,50	Repeats, Maximum= Unlimited<br>Added by visit schedule rule(s) #173
		Pregnancy Test	130,60	Added by visit schedule rule(s) #163
		Lab Sample Collection	130,70	Repeats, Maximum= Unlimited<br>Added by visit schedule rule(s) #164
		Lab Test Result	130,180	Added by visit schedule rule(s) #170
		Lab Test Assessment	130,190	Repeats, Maximum= Unlimited<br>Added by visit schedule rule(s) #175
		Alcohol Breath Test	130,250	Added by visit schedule rule(s) #
		Urine Drug Screen	130,80	Added by visit schedule rule(s) #165
		Urinalysis	130,110	Added by visit schedule rule(s) #166
		Urine Microscopy Assessment	130,220	Repeats, Maximum= Unlimited<br>Added by visit schedule rule(s) #198
		PCR Collection	130,130	Added by visit schedule rule(s) #168
		Feeding	130,200	Repeats, Maximum= Unlimited<br>Added by visit schedule rule(s) #171
		PK	130,140	Added by visit schedule rule(s) #167
		ECG	130,160	Repeats, Maximum= Unlimited<br>Added by visit schedule rule(s) #169
		Piperaquine Dosing	130,230	Added by visit schedule rule(s) #172
		Genetic Testing	130,270	Added by visit schedule rule(s) #200
visit-160	Reconsent		160	Added: By User After Add Subject<br>Repeats, Maximum=Unlimited
		Reconsent	160,10	
visit-190	Early Termination Visit		190	Added: By User After Add Subject
		Visit Date	190,100	
		Physical Exam	190,10	
		Physical Exam (extra)	190,20	Repeats, Maximum= Unlimited<br>Added by visit schedule rule(s) #209
		ECG (single)	190,30	
		Vital Signs (single)	190,40	

Unique Identifier	Visit	Visit - Page	Visit ID, Page ID	Notes
		Lab Sample Collection Summary	190,50	
		Lab Sample Collection	190,60	Repeats, Maximum= Unlimited<br>Added by visit schedule rule(s) #206
		Lab Test Result	190,110	Added by visit schedule rule(s) #207
		Lab Test Assessment	190,120	Repeats, Maximum= Unlimited<br>Added by visit schedule rule(s) #208
		Serology	190,160	
		Pregnancy Test	190,70	Added by visit schedule rule(s) #196
		Urinalysis	190,80	
		Urine Microscopy Assessment	190,140	Repeats, Maximum= Unlimited<br>Added by visit schedule rule(s) #195
		Safety Serum Storage	190,90	
		PCR Collection	190,150	

Screening

Visit Date

[Revision: Initial revision of study]

(Visit ID = 10 / Visit Display Name = Screening / Visit Abbrev = SCR / PageID = 180 / Page Display Name = Visit Date / Description =)

Visit Not Done?	☐	
* Visit Date		(DD-MMM-YYYY)

Informed Consent

[Revision: Initial revision of study]

(Visit ID = 10 / Visit Display Name = Screening / Visit Abbrev = SCR / PageID = 10 / Page Display Name = Informed Consent / Description = Enrollment)

* Date of Informed Consent		(DD-MMM-YYYY)
* Time of Informed Consent		(HH24:MI)
* Informed Consent Version		
* Screening Number		

Inclusion / Exclusion Summary

[Revision: Initial revision of study]

(Visit ID = 10 / Visit Display Name = Screening / Visit Abbrev = SCR / PageID = 140 / Page Display Name = Inclusion / Exclusion Summary / Description = Inclusion / Exclusion Header)

* Did the subject meet all eligibility criteria?	○ No<br>○ Yes

Inclusion / Exclusion Criteria

[Revision: Initial revision of study]

(Visit ID = 10 / Visit Display Name = Screening / Visit Abbrev = SCR / PageID = 150 (*) / Page Display Name = Inclusion / Exclusion Criteria / Description = Inclusion and Exclusion Criteria)

* What was the category of the criterion?	○ EXCLUSION<br>○ INCLUSION	
* What was the criterion not met		(format xx)

Demographics

[Revision: Initial revision of study]

(Visit ID = 10 / Visit Display Name = Screening / Visit Abbrev = SCR / PageID = 260 / Page Display Name = Demographics / Description = Demographics)

* What is the subject's date of birth?	☐	(DD-MMM-YYYY)
* Age	(auto calculated)	(format xxx)
* What is the sex of the subject?	○ F - Female<br>○ M - Male<br>○ U - Unknown<br>○ UN - Undifferentiated	
* Is the participant of childbearing potential?	○ Yes<br>○ No	
* Are they post menopausal	○ Yes<br>○ No	
* If no, reason	☐	
* What is the ethnicity of the subject?	○ HISPANIC OR LATINO<br>○ NOT HISPANIC OR LATINO<br>○ NOT REPORTED<br>○ UNKNOWN	
**Race (check all that apply)**		
American Indian or Alaskan Native	☐	
Asian	☐	
Black or African American	☐	
Native Hawaiian or Pacific Islander	☐	
White	☐	
Other	☐	
* Other, specify	☐	

Physical Exam

[Revision: Initial revision of study]

(Visit ID = 10 / Visit Display Name = Screening / Visit Abbrev = SCR / PageID = 40 / Page Display Name = Physical Exam / Description = Physical Exam)

* Was Physical Exam Done	○ Yes<br>○ No	
* Date of Physical Exam	[ ]	(DD-MMM-YYYY)
* Time of Physical Exam	[ ]	(HH24:MI)
AM/PM	○ AM<br>○ PM	

Body System	Assessment	Description	Clinical Significance
Head/Neck/Ears/Eyes/Nose/Throat	○ Normal<br>○ Abnormal<br>○ Not Done		○ Not Clinically Significant<br>○ Clinically Significant
Heart/circulation	○ Normal<br>○ Abnormal<br>○ Not Done		○ Not Clinically Significant<br>○ Clinically Significant
Chest	○ Normal<br>○ Abnormal<br>○ Not Done		○ Not Clinically Significant<br>○ Clinically Significant
Lungs	○ Normal<br>○ Abnormal<br>○ Not Done		○ Not Clinically Significant<br>○ Clinically Significant
Abdomen	○ Normal<br>○ Abnormal<br>○ Not Done		○ Not Clinically Significant<br>○ Clinically Significant
Skin	○ Normal<br>○ Abnormal<br>○ Not Done		○ Not Clinically Significant<br>○ Clinically Significant
Neurological	○ Normal<br>○ Abnormal<br>○ Not Done		○ Not Clinically Significant<br>○ Clinically Significant

Extremities	○ Normal<br>○ Abnormal<br>○ Not Done		○ Not Clinically Significant<br>○ Clinically Significant
Back	○ Normal<br>○ Abnormal<br>○ Not Done		○ Not Clinically Significant<br>○ Clinically Significant
Dentition	○ Normal<br>○ Abnormal<br>○ Not Done		○ Not Clinically Significant<br>○ Clinically Significant

Other Body Systems

* Were any other abnormalities noted?	○ No<br>○ Yes

Physical Exam (extra)

[Revision: Initial revision of study]

(Visit ID = 10 / Visit Display Name = Screening / Visit Abbrev = SCR / PageID = 50 (*) / Page Display Name = Physical Exam (extra) / Description = a page to capture other or extra body systems)

* Body System	○ Head/Ears/Eyes/Nose/Throat<br>○ Heart<br>○ Chest<br>○ Lungs<br>○ Abdomen<br>○ Skin<br>○ Neurological exam<br>○ Extremities<br>○ Back<br>○ Dentition<br>○ General Appearance<br>○ Other
* Other Body System	
* Assessment	○ Normal<br>○ Abnormal<br>○ Not Done
* Description of abnormality	
* Clinical Significance	○ Not Clinically Significant<br>○ Clinically Significant

Height and Weight

[Revision: Initial revision of study]

(Visit ID = 10 / Visit Display Name = Screening / Visit Abbrev = SCR / PageID = 240 / Page Display Name = Height and Weight / Description =)

* Was height and weight assessed?	○ Yes<br>○ No	
* Date performed		(DD-MMM-YYYY)
* Height (cm)		(format xxx)
* Weight (kg)		(format xxx.x)
* BMI	(auto calculated)	(format xx.x)

ECG

[Revision: Initial revision of study]

(Visit ID = 10 / Visit Display Name = Screening / Visit Abbrev = SCR / PageID = 60 / Page Display Name = ECG / Description = ECG Single)

* Was ECG performed	○ Yes<br>○ No	
* Date of ECG		(DD-MMM-YYYY)
* Time of ECG		(HH24:MI)
* Heart Rate (bpm)		(format xxx)
* PR Interval (msecs)		(format xxx.x)
* RR Interval (msecs)		(format xxxx.x)
* QRS Interval (msecs)		(format xxx.x)
* QT Interval (msecs)		(format xxx.xx)
* QTcF Interval (msecs)		(format xxxxx)
* ECG Interpretation	○ Normal<br>○ Abnormal NCS<br>○ Abnormal CS	
* If Abnormal, describe the abnormality		

Vital Signs

[Revision: Initial revision of study]

(Visit ID = 10 / Visit Display Name = Screening / Visit Abbrev = SCR / PageID = 70 / Page Display Name = Vital Signs / Description = Vital Signs at screening)

* Were Vital Signs assessed?	○ Yes<br>○ No	
* Date performed		(DD-MMM-YYYY)
* Temperature Method	○ Tympanic<br>○ Sublingual	
* Temperature (C)		(format xx.x)
* Assessment	○ Normal<br>○ Abnormal NCS<br>○ Abnormal CS	
* Respiratory Rate (breaths per minute)		(format xxx)
* Assessment	○ Normal<br>○ Abnormal NCS<br>○ Abnormal CS	
* Time of Supine Vital Sign Measurements		(HH24:MI)
* Supine Systolic Blood Pressure (mmHg)		(format xxx)
* Assessment	○ Normal<br>○ Abnormal NCS<br>○ Abnormal CS	
* Supine Diastolic Blood Pressure (mmHg)		(format xxx)
* Assessment	○ Normal<br>○ Abnormal NCS<br>○ Abnormal CS	

* Supine Heart Rate (bpm)		(format xxx)
* Assessment	○ Normal<br>○ Abnormal NCS<br>○ Abnormal CS	
* Time of Standing Vital Sign Measurements		(HH24:MI)
* Standing Systolic Blood Pressure (mmHg)		(format xxx)
* Assessment	○ Normal<br>○ Abnormal NCS<br>○ Abnormal CS	
* Standing Diastolic Blood Pressure (mmHg)		(format xxx)
* Assessment	○ Normal<br>○ Abnormal NCS<br>○ Abnormal CS	
* Standing Heart Rate (bpm)		(format xxx)
* Assessment	○ Normal<br>○ Abnormal NCS<br>○ Abnormal CS	

Lab Sample Collection Summary

[Revision: Initial revision of study]

(Visit ID = 10 / Visit Display Name = Screening / Visit Abbrev = SCR / PageID = 80 / Page Display Name = Lab Sample Collection Summary / Description = Lab collection header)

* Were any Labs collected ?	○ No<br>○ Yes	
* Sample date		(DD-MMM-YYYY)
* Sample time		(HH24:MI)

Lab Sample Collection

[Revision: Initial revision of study]

(Visit ID = 10 / Visit Display Name = Screening / Visit Abbrev = SCR / PageID = 90 (*) / Page Display Name = Lab Sample Collection / Description = Labs collected)

* Panel test	○ Biochemistry<br>○ Haematology<br>○ CMV<br>○ EBV<br>○ G6PD<br>○ LFT<br>○ Red Cell Alloantibodies<br>○ Serology<br>○ Duffy<br>○ Rh Status<br>○ Immunology<br>○ Thyroid Tests<br>○ Coagulation<br>○ Endocrinology<br>○ Blood Bank<br>○ Microbiology<br>○ Other
* If Other, specify	

Lab Test Results

[Revision: Initial revision of study]

(Visit ID = 10 / Visit Display Name = Screening / Visit Abbrev = SCR / PageID = 160 / Page Display Name = Lab Test Results / Description = Were any lab tests flagged as being out of range)

* Were any of the lab tests flagged as being out of range ?	○ No<br>○ Yes

Lab Test Assessment

[Revision: Initial revision of study]

(Visit ID = 10 / Visit Display Name = Screening / Visit Abbrev = SCR / PageID = 170 (*) / Page Display Name = Lab Test Assessment / Description = Assessment of all out of range labs)

* Choose the category of the tests that were out of range	○ Biochemistry<br>○ Haematology<br>○ CMV<br>○ EBV<br>○ G6PD<br>○ LFT<br>○ Red Cell Alloantibodies<br>○ Serology<br>○ Duffy<br>○ Rh Status<br>○ Immunology<br>○ Thyroid Tests<br>○ Coagulation<br>○ Endocrinology<br>○ Blood Bank<br>○ Microbiology<br>○ Other
* If Other, specify	

* List all tests that were out of range	○ White Blood Cells count<br>○ Neutrophils<br>○ Lymphocytes<br>○ Monocytes<br>○ Eosinophils<br>○ Basophils<br>○ Red blood cell count<br>○ Haemoglobin<br>○ Haematocrit<br>○ Platelet Count<br>○ Reticulocyte Count<br>○ Red Cell Allo-Antibodies<br>○ Duffy Blood Group<br>○ Sodium<br>○ Potassium<br>○ Chloride<br>○ Bicarbonate<br>○ Glucose<br>○ Urea<br>○ Creatinine<br>○ Urate<br>○ Albumin<br>○ Globulin<br>○ Total Protein<br>○ Direct Bilirubin<br>○ Alkaline Phosphatase<br>○ Alanine aminotransferase<br>○ Aspartate aminotransferase

- ○ Calcium Corrected
- ○ Phosphate
- ○ Lactate dehyrogenase
- ○ Magnesium
- ○ Cholesterol
- ○ Triglycerides
- ○ HDL
- ○ LDL
- ○ Cholesterol/HDL
- ○ Total Bilirubin
- ○ eGFR
- ○ Pregnancy Test
- ○ FSH
- ○ HIV
- ○ HBsAg
- ○ anti-HBc
- ○ anti-HCV
- ○ anti-HAV
- ○ anti-HEV
- ○ EBV NA IgG
- ○ EBV VCA IgM
- ○ CMV IgG
- ○ CMV IgM
- ○ Other

* If Other, Specify	
* Clinical Significance	○ Abnormal NCS<br>○ Abnormal CS

If a lab test has been assessed as being Clinically Significant please add as an Adverse Event,

Serology

[Revision: Initial revision of study]

(Visit ID = 10 / Visit Display Name = Screening / Visit Abbrev = SCR / PageID = 230 / Page Display Name = Serology / Description = Hep and HIV)

* Were serology tests done?	○ Yes<br>○ No	
* Date of blood collection		(DD-MMM-YYYY)
* Time of blood collection		(HH24:MI)
* HIV Ag/Ab	○ Negative<br>○ Positive	
* HBsAg	○ Negative<br>○ Positive	
* HCV	○ Negative<br>○ Positive	

FSH

[Revision: Initial revision of study]

(Visit ID = 10 / Visit Display Name = Screening / Visit Abbrev = SCR / PageID = 250 / Page Display Name = FSH / Description = Follicle stimulating hormone test)

* Was FSH test done?	○ Yes<br>○ No	
* Date of test		(DD-MMM-YYYY)
* Time of test		(HH24:MI)

Pregnancy Test

[Revision: Initial revision of study]

(Visit ID = 10 / Visit Display Name = Screening / Visit Abbrev = SCR / PageID = 100 / Page Display Name = Pregnancy Test / Description = Pregnancy test)

* Was Pregnancy Test performed?	○ Yes<br>○ No	
* Serum or Urine	○ Serum<br>○ Urine	
* Date of pregnancy test	[ ]	(DD-MMM-YYYY)
* Time of pregnancy test	[ ]	(HH24:MI)
* Result	○ Negative<br>○ Positive	

Urinalysis

[Revision: Initial revision of study]

(Visit ID = 10 / Visit Display Name = Screening / Visit Abbrev = SCR / PageID = 120 / Page Display Name = Urinalysis / Description = Urinanalysis)

* Was Urinalysis performed?	○ Yes<br>○ No	
* Date of Urinalysis		(DD-MMM-YYYY)
* Time of Urinalysis		(HH24:MI)

Test	Result	Assessment
Glucose (mmol/L)	◯ Negative<br>◯ 5.5<br>◯ 14<br>◯ 28<br>◯ >=55	◯ Normal<br>◯ Abnormal NCS<br>◯ Abnormal CS
Bilirubin	◯ Negative<br>◯ Small<br>◯ Moderate<br>◯ Large	◯ Normal<br>◯ Abnormal NCS<br>◯ Abnormal CS
Ketone (mmol/L)	◯ Negative<br>◯ Trace<br>◯ 1.5<br>◯ 3.9<br>◯ 7.8<br>◯ >=15.6	◯ Normal<br>◯ Abnormal NCS<br>◯ Abnormal CS
Specific Gravity	◯ <=1.005<br>◯ 1.010<br>◯ 1.015<br>◯ 1.020<br>◯ 1.025<br>◯ >=1.030	◯ Normal<br>◯ Abnormal NCS<br>◯ Abnormal CS
Blood (Ery/uL)	◯ Negative<br>◯ Trace-lysed<br>◯ Trace-intact<br>◯ Ca 25<br>◯ Ca 80<br>◯ Ca 200	◯ Normal<br>◯ Abnormal NCS<br>◯ Abnormal CS

pH	○ 5.0<br>○ 5.5<br>○ 6.0<br>○ 6.5<br>○ 7.0<br>○ 7.5<br>○ 8.0<br>○ 8.5<br>○ >=9.0	○ Normal<br>○ Abnormal NCS<br>○ Abnormal CS
Protein (g/L)	○ Negative<br>○ Trace<br>○ 0.15<br>○ 0.3<br>○ 1.0<br>○ 3.0<br>○ >=3.0	○ Normal<br>○ Abnormal NCS<br>○ Abnormal CS
Urobilinogen (mmol/L)	○ 3.2<br>○ 16<br>○ 33<br>○ 66<br>○ >+131	○ Normal<br>○ Abnormal NCS<br>○ Abnormal CS
Nitrite	○ Negative<br>○ Positive	○ Normal<br>○ Abnormal NCS<br>○ Abnormal CS

Leukocytes (Leu/uL)	○ Negative<br>○ Ca 15<br>○ Ca 70<br>○ Ca 125<br>○ Ca 500	○ Normal<br>○ Abnormal NCS<br>○ Abnormal CS
* Was the sample sent for microscopy	○ No<br>○ Yes	
* Were any microscopy parameters out of range?	○ No<br>○ Yes	

Urine Microscopy Assessment

[Revision: Initial revision of study]

(Visit ID = 10 / Visit Display Name = Screening / Visit Abbrev = SCR / PageID = 200 (*) / Page Display Name = Urine Microscopy Assessment / Description = Microscopy Assessment)

* List all tests that were out of range	○ Leucocytes<br>○ Erythrocytes<br>○ Protein<br>○ glucose<br>○ Epithelial Cells<br>○ pH<br>○ Casts<br>○ Blood<br>○ Ketones<br>○ Leucocyte Esterase<br>○ Specific gravity<br>○ Bilirubin<br>○ Urobilinogen<br>○ Nitrites<br>○ Bacteria<br>○ Other
* If Other, Specify	
* Clinical Significance	○ Abnormal NCS<br>○ Abnormal CS

Urine Drug Screen

[Revision: Initial revision of study]

(Visit ID = 10 / Visit Display Name = Screening / Visit Abbrev = SCR / PageID = 130 / Page Display Name = Urine Drug Screen / Description = Drug Screen)

* Was Urine Drug Screen Performed?	○ Yes<br>○ No	
* Date of Drug Screen		(DD-MMM-YYYY)
* Time of Drug Screen		(HH24:MI)

Drug	Result
Amphetamines	○ Negative<br>○ Positive
Methamphetamines	○ Negative<br>○ Positive
Barbiturates	○ Negative<br>○ Positive
Benzodiazepines	○ Negative<br>○ Positive
Cocaine	○ Negative<br>○ Positive
Methadone	○ Negative<br>○ Positive
Opiates	○ Negative<br>○ Positive
Phencyclidine	○ Negative<br>○ Positive
Cannabis	○ Negative<br>○ Positive
Tricyclic antidepressants	○ Negative<br>○ Positive
Acetaminophen (paracetamol)	○ Negative<br>○ Positive

Alcohol Breath Test

[Revision: Initial revision of study]

(Visit ID = 10 / Visit Display Name = Screening / Visit Abbrev = SCR / PageID = 210 / Page Display Name = Alcohol Breath Test / Description = Alcohol Breath Test)

* Was Alcohol Breath Test done	○ Yes<br>○ No	
* Date of Breath Test		(DD-MMM-YYYY)
* Time of Breath Test		(HH24:MI)
* Result	○ Negative<br>○ Positive	

Safety Visit

Visit Date

[Revision: Initial revision of study]

(Visit ID = 180 / Visit Display Name = Safety Visit / Visit Abbrev = SV / PageID = 80 / Page Display Name = Visit Date / Description =)

Visit Not Done?	☐	
* Visit Date		(DD-MMM-YYYY)

Lab Sample Collection Summary

[Revision: Initial revision of study]

(Visit ID = 180 / Visit Display Name = Safety Visit / Visit Abbrev = SV / PageID = 10 / Page Display Name = Lab Sample Collection Summary / Description = Lab collection header)

* Were any Labs collected ?	○ No<br>○ Yes	
* Sample date		(DD-MMM-YYYY)
* Sample time		(HH24:MI)

Lab Sample Collection

[Revision: Initial revision of study]

(Visit ID = 180 / Visit Display Name = Safety Visit / Visit Abbrev = SV / PageID = 20 (*) / Page Display Name = Lab Sample Collection / Description = Labs collected)

* Panel test	○ Biochemistry<br>○ Haematology<br>○ CMV<br>○ EBV<br>○ G6PD<br>○ LFT<br>○ Red Cell Alloantibodies<br>○ Serology<br>○ Duffy<br>○ Rh Status<br>○ Immunology<br>○ Thyroid Tests<br>○ Coagulation<br>○ Endocrinology<br>○ Blood Bank<br>○ Microbiology<br>○ Other
* If Other, specify	

Lab Test Results

[Revision: Initial revision of study]

(Visit ID = 180 / Visit Display Name = Safety Visit / Visit Abbrev = SV / PageID = 30 / Page Display Name = Lab Test Results / Description = Were any lab tests flagged as being out of range)

* Were any of the lab tests flagged as being out of range ?	○ No<br>○ Yes

Lab Test Assessment

[Revision: Initial revision of study]

(Visit ID = 180 / Visit Display Name = Safety Visit / Visit Abbrev = SV / PageID = 40 (*) / Page Display Name = Lab Test Assessment / Description = Assessment of all out of range labs)

* Choose the category of the tests that were out of range	○ Biochemistry<br>○ Haematology<br>○ CMV<br>○ EBV<br>○ G6PD<br>○ LFT<br>○ Red Cell Alloantibodies<br>○ Serology<br>○ Duffy<br>○ Rh Status<br>○ Immunology<br>○ Thyroid Tests<br>○ Coagulation<br>○ Endocrinology<br>○ Blood Bank<br>○ Microbiology<br>○ Other
* If Other, specify	

* List all tests that were out of range

- ○ White Blood Cells count
- ○ Neutrophils
- ○ Lymphocytes
- ○ Monocytes
- ○ Eosinophils
- ○ Basophils
- ○ Red blood cell count
- ○ Haemoglobin
- ○ Haematocrit
- ○ Platelet Count
- ○ Reticulocyte Count
- ○ Red Cell Allo-Antibodies
- ○ Duffy Blood Group
- ○ Sodium
- ○ Potassium
- ○ Chloride
- ○ Bicarbonate
- ○ Glucose
- ○ Urea
- ○ Creatinine
- ○ Urate
- ○ Albumin
- ○ Globulin
- ○ Total Protein
- ○ Direct Bilirubin
- ○ Alkaline Phosphatase
- ○ Alanine aminotransferase
- ○ Aspartate aminotransferase

	○ Calcium Corrected<br>○ Phosphate<br>○ Lactate dehyrogenase<br>○ Magnesium<br>○ Cholesterol<br>○ Triglycerides<br>○ HDL<br>○ LDL<br>○ Cholesterol/HDL<br>○ Total Bilirubin<br>○ eGFR<br>○ Pregnancy Test<br>○ FSH<br>○ HIV<br>○ HBsAg<br>○ anti-HBc<br>○ anti-HCV<br>○ anti-HAV<br>○ anti-HEV<br>○ EBV NA IgG<br>○ EBV VCA IgM<br>○ CMV IgG<br>○ CMV IgM<br>○ Other
* If Other, Specify	
* Clinical Significance	○ Abnormal NCS<br>○ Abnormal CS

If a lab test has been assessed as being Clinically Significant please add as an Adverse Event,

Urinalysis

[Revision: Initial revision of study]

(Visit ID = 180 / Visit Display Name = Safety Visit / Visit Abbrev = SV / PageID = 50 / Page Display Name = Urinalysis / Description = Urinanalysis)

* Was Urinalysis performed?	○ Yes<br>○ No	
* Date of Urinalysis	[ ]	(DD-MMM-YYYY)
* Time of Urinalysis	[ ]	(HH24:MI)

Test	Result	Assessment
Glucose (mmol/L)	○ Negative<br>○ 5.5<br>○ 14<br>○ 28<br>○ >=55	○ Normal<br>○ Abnormal NCS<br>○ Abnormal CS
Bilirubin	○ Negative<br>○ Small<br>○ Moderate<br>○ Large	○ Normal<br>○ Abnormal NCS<br>○ Abnormal CS
Ketone (mmol/L)	○ Negative<br>○ Trace<br>○ 1.5<br>○ 3.9<br>○ 7.8<br>○ >=15.6	○ Normal<br>○ Abnormal NCS<br>○ Abnormal CS
Specific Gravity	○ <=1.005<br>○ 1.010<br>○ 1.015<br>○ 1.020<br>○ 1.025<br>○ >=1.030	○ Normal<br>○ Abnormal NCS<br>○ Abnormal CS
Blood (Ery/uL)	○ Negative<br>○ Trace-lysed<br>○ Trace-intact<br>○ Ca 25<br>○ Ca 80<br>○ Ca 200	○ Normal<br>○ Abnormal NCS<br>○ Abnormal CS

pH	○ 5.0<br>○ 5.5<br>○ 6.0<br>○ 6.5<br>○ 7.0<br>○ 7.5<br>○ 8.0<br>○ 8.5<br>○ >=9.0	○ Normal<br>○ Abnormal NCS<br>○ Abnormal CS
Protein (g/L)	○ Negative<br>○ Trace<br>○ 0.15<br>○ 0.3<br>○ 1.0<br>○ 3.0<br>○ >=3.0	○ Normal<br>○ Abnormal NCS<br>○ Abnormal CS
Urobilinogen (mmol/L)	○ 3.2<br>○ 16<br>○ 33<br>○ 66<br>○ >+131	○ Normal<br>○ Abnormal NCS<br>○ Abnormal CS
Nitrite	○ Negative<br>○ Positive	○ Normal<br>○ Abnormal NCS<br>○ Abnormal CS

Leukocytes (Leu/uL)	○ Negative<br>○ Ca 15<br>○ Ca 70<br>○ Ca 125<br>○ Ca 500	○ Normal<br>○ Abnormal NCS<br>○ Abnormal CS
* Was the sample sent for microscopy	○ No<br>○ Yes	
* Were any microscopy parameters out of range?	○ No<br>○ Yes	

Urine Microscopy Assessment

[Revision: Initial revision of study]

(Visit ID = 180 / Visit Display Name = Safety Visit / Visit Abbrev = SV / PageID = 70 (*) / Page Display Name = Urine Microscopy Assessment / Description = Microscopy Assessment)

* List all tests that were out of range	○ Leucocytes<br>○ Erythrocytes<br>○ Protein<br>○ glucose<br>○ Epithelial Cells<br>○ pH<br>○ Casts<br>○ Blood<br>○ Ketones<br>○ Leucocyte Esterase<br>○ Specific gravity<br>○ Bilirubin<br>○ Urobilinogen<br>○ Nitrites<br>○ Bacteria<br>○ Other
* If Other, Specify	
* Clinical Significance	○ Abnormal NCS<br>○ Abnormal CS

Inoculum

Visit Date

[Revision: Initial revision of study]

(Visit ID = 20 / Visit Display Name = Inoculum / Visit Abbrev = INOC / PageID = 130 / Page Display Name = Visit Date / Description =)

Visit Not Done?	☐	
* Visit Date		(DD-MMM-YYYY)

Inclusion / Exclusion Summary

[Revision: Initial revision of study]

(Visit ID = 20 / Visit Display Name = Inoculum / Visit Abbrev = INOC / PageID = 10 / Page Display Name = Inclusion / Exclusion Summary / Description = Inclusion / Exclusion Header)

* Did the subject meet all eligibility criteria?	○ No<br>○ Yes

Inclusion / Exclusion Criteria

[Revision: Initial revision of study]

(Visit ID = 20 / Visit Display Name = Inoculum / Visit Abbrev = INOC / PageID = 20 (*) / Page Display Name = Inclusion / Exclusion Criteria / Description = Inclusion and Exclusion Criteria)

* What was the category of the criterion?	○ EXCLUSION<br>○ INCLUSION	
* What was the criterion not met		(format xx)

Urine Drug Screen

[Revision: Initial revision of study]

(Visit ID = 20 / Visit Display Name = Inoculum / Visit Abbrev = INOC / PageID = 100 / Page Display Name = Urine Drug Screen / Description = Drug Screen)

* Was Urine Drug Screen Performed?	○ Yes<br>○ No	
* Date of Drug Screen		(DD-MMM-YYYY)
* Time of Drug Screen		(HH24:MI)

Drug	Result
Amphetamines	○ Negative<br>○ Positive
Methamphetamines	○ Negative<br>○ Positive
Barbiturates	○ Negative<br>○ Positive
Benzodiazepines	○ Negative<br>○ Positive
Cocaine	○ Negative<br>○ Positive
Methadone	○ Negative<br>○ Positive
Opiates	○ Negative<br>○ Positive
Phencyclidine	○ Negative<br>○ Positive
Cannabis	○ Negative<br>○ Positive
Tricyclic antidepressants	○ Negative<br>○ Positive
Acetaminophen (paracetamol)	○ Negative<br>○ Positive

Alcohol Breath Test

[Revision: Initial revision of study]

(Visit ID = 20 / Visit Display Name = Inoculum / Visit Abbrev = INOC / PageID = 160 / Page Display Name = Alcohol Breath Test / Description = Alcohol Breath Test)

* Was Alcohol Breath Test done	○ Yes<br>○ No	
* Date of Breath Test		(DD-MMM-YYYY)
* Time of Breath Test		(HH24:MI)
* Result	○ Negative<br>○ Positive	

Randomisation

[Revision: Initial revision of study]

(Visit ID = 20 / Visit Display Name = Inoculum / Visit Abbrev = INOC / PageID = 170 / Page Display Name = Randomisation / Description =)

* Date of Randomisation	______	(DD-MMM-YYYY)
* Cohort	○ K13 - Cohort 1<br>○ 3D7 - Cohort 1<br>○ K13 - Cohort 2<br>○ 3D7 - Cohort 2<br>○ K13 - Cohort 3<br>○ 3D7 - Cohort 3	
* Enrolment Number	______	

Abb. Physical Exam

[Revision: Initial revision of study]

(Visit ID = 20 / Visit Display Name = Inoculum / Visit Abbrev = INOC / PageID = 30 / Page Display Name = Abb. Physical Exam / Description =)

* Was Abbreviated Physical Exam Done	○ Yes<br>○ No	
* Date of Physical Exam		(DD-MMM-YYYY)
* Time of Physical Exam		(HH24:MI)
AM/PM	○ AM<br>○ PM	

Body System	Assessment	Description	Clinical Significance
General Appearance	○ Normal<br>○ Abnormal<br>○ Not Done		○ Not Clinically Significant<br>○ Clinically Significant
Heart/circulation	○ Normal<br>○ Abnormal<br>○ Not Done		○ Not Clinically Significant<br>○ Clinically Significant
Chest	○ Normal<br>○ Abnormal<br>○ Not Done		○ Not Clinically Significant<br>○ Clinically Significant
Lungs	○ Normal<br>○ Abnormal<br>○ Not Done		○ Not Clinically Significant<br>○ Clinically Significant
Abdomen	○ Normal<br>○ Abnormal<br>○ Not Done		○ Not Clinically Significant<br>○ Clinically Significant
Skin	○ Normal<br>○ Abnormal<br>○ Not Done		○ Not Clinically Significant<br>○ Clinically Significant
Brief Neurological	○ Normal<br>○ Abnormal<br>○ Not Done		○ Not Clinically Significant<br>○ Clinically Significant

Other Body Systems

* Were any other abnormalities noted?

○ No
○ Yes

Physical Exam (extra)

[Revision: Initial revision of study]

(Visit ID = 20 / Visit Display Name = Inoculum / Visit Abbrev = INOC / PageID = 40 (*) / Page Display Name = Physical Exam (extra) / Description = a page to capture other or extra body systems)

* Body System	○ Head/Ears/Eyes/Nose/Throat<br>○ Heart<br>○ Chest<br>○ Lungs<br>○ Abdomen<br>○ Skin<br>○ Neurological exam<br>○ Extremities<br>○ Back<br>○ Dentition<br>○ General Appearance<br>○ Other
* Other Body System	
* Assessment	○ Normal<br>○ Abnormal<br>○ Not Done
* Description of abnormality	
* Clinical Significance	○ Not Clinically Significant<br>○ Clinically Significant

ECG

[Revision: Initial revision of study]

(Visit ID = 20 / Visit Display Name = Inoculum / Visit Abbrev = INOC / PageID = 50 / Page Display Name = ECG / Description = ECG Single)

* Was ECG performed	○ Yes<br>○ No	
* Date of ECG		(DD-MMM-YYYY)
* Time of ECG		(HH24:MI)
* Heart Rate (bpm)		(format xxx)
* PR Interval (msecs)		(format xxx.x)
* RR Interval (msecs)		(format xxxx.x)
* QRS Interval (msecs)		(format xxx.x)
* QT Interval (msecs)		(format xxx.xx)
* QTcF Interval (msecs)		(format xxxxx)
* ECG Interpretation	○ Normal<br>○ Abnormal NCS<br>○ Abnormal CS	
* If Abnormal, describe the abnormality		

Vitals Signs

[Revision: Initial revision of study]

(Visit ID = 20 / Visit Display Name = Inoculum / Visit Abbrev = INOC / PageID = 60 (*) / Page Display Name = Vitals Signs / Description = Vital Signs Inoculum)

* Time point	○ Pre-inoculum<br>○ 60 minutes post inoculum	
* Were Vital Signs assessed?	○ Yes<br>○ No	
* Date performed		(DD-MMM-YYYY)
* Time performed		(HH24:MI)
* Temperature Method	○ Tympanic<br>○ Sublingual	
* Temperature (C)		(format xx.x)
* Assessment	○ Normal<br>○ Abnormal NCS<br>○ Abnormal CS	
* Respiratory Rate (breaths per minute)		(format xxx)
* Assessment	○ Normal<br>○ Abnormal NCS<br>○ Abnormal CS	
* Systolic Blood Pressure (mmHg)		(format xxx)
* Assessment	○ Normal<br>○ Abnormal NCS<br>○ Abnormal CS	
* Diastolic Blood Pressure (mmHg)		(format xxx)

* Assessment	○ Normal<br>○ Abnormal NCS<br>○ Abnormal CS	
* Heart Rate (bpm)		(format xxx)
* Assessment	○ Normal<br>○ Abnormal NCS<br>○ Abnormal CS	

Pregnancy Test

[Revision: Initial revision of study]

(Visit ID = 20 / Visit Display Name = Inoculum / Visit Abbrev = INOC / PageID = 90 / Page Display Name = Pregnancy Test / Description = Pregnancy test)

* Was Pregnancy Test performed?	○ Yes<br>○ No	
* Serum or Urine	○ Serum<br>○ Urine	
* Date of pregnancy test		(DD-MMM-YYYY)
* Time of pregnancy test		(HH24:MI)
* Result	○ Negative<br>○ Positive	

Inoculum Administration

[Revision: Initial revision of study]

(Visit ID = 20 / Visit Display Name = Inoculum / Visit Abbrev = INOC / PageID = 110 / Page Display Name = Inoculum Administration / Description = Inoculum Details)

* Inoculum Type	○ P. falciparum isolate K13<br>○ P. falciparum isolate 3D7	
* Date of Preparation		(DD-MMM-YYYY)
* Time of Preparation		(HH24:MI)
* Date of Administration		(DD-MMM-YYYY)
* Time of Administration		(HH24:MI)
* Dose		
* Was the Malaria inoculum administered as per protocol?	○ No<br>○ Yes	

Safety Serum Storage

[Revision: Initial revision of study]

(Visit ID = 20 / Visit Display Name = Inoculum / Visit Abbrev = INOC / PageID = 120 / Page Display Name = Safety Serum Storage / Description = Safety Serum)

* Was the Safety Serum collected	○ No<br>○ Yes	
* Date of Serum Sample Collection		(DD-MMM-YYYY)
* Time of Serum Sample Collection		(HH24:MI)

PCR Collection

[Revision: Initial revision of study]

(Visit ID = 20 / Visit Display Name = Inoculum / Visit Abbrev = INOC / PageID = 180 / Page Display Name = PCR Collection / Description =)

Not Done	☐	
* Date of PCR Collection		(DD-MMM-YYYY)
* Time of PCR Collection		(HH24:MI)

Telephone Contact 1

Phone Call

[Revision: Initial revision of study]

(Visit ID = 30 / Visit Display Name = Telephone Contact 1 / Visit Abbrev = T1 / PageID = 10 / Page Display Name = Phone Call / Description = follow up phone call)

* Was contact with the participant attempted?	○ No<br>○ Yes	
* If yes, Was contact made	○ No<br>○ Yes	
* Date of contact	[ ]	(DD-MMM-YYYY)
* Time of Contact	[ ]	(HH24:MI)

Telephone Contact 2

Phone Call

[Revision: Initial revision of study]

(Visit ID = 40 / Visit Display Name = Telephone Contact 2 / Visit Abbrev = T2 / PageID = 10 / Page Display Name = Phone Call / Description = follow up phone call)

* Was contact with the participant attempted?	○ No<br>○ Yes	
* If yes, Was contact made	○ No<br>○ Yes	
* Date of contact	[ ]	(DD-MMM-YYYY)
* Time of Contact	[ ]	(HH24:MI)

Telephone Contact 3

Phone Call

[Revision: Initial revision of study]

(Visit ID = 50 / Visit Display Name = Telephone Contact 3 / Visit Abbrev = T3 / PageID = 10 / Page Display Name = Phone Call / Description = follow up phone call)

* Was contact with the participant attempted?	○ No<br>○ Yes	
* If yes, Was contact made	○ No<br>○ Yes	
* Date of contact	[ ]	(DD-MMM-YYYY)
* Time of Contact	[ ]	(HH24:MI)

Malaria Monitoring

Visit Date

[Revision: Initial revision of study]

(Visit ID = 60 (*) / Visit Display Name = Malaria Monitoring / Visit Abbrev = MM / PageID = 70 / Page Display Name = Visit Date / Description = Visit Date)

Visit Not Done?	☐	
* Visit Date		(DD-MMM-YYYY)
* AM or PM	◯ AM<br>◯ PM	

Abb. Physical Exam

[Revision: Initial revision of study]

(Visit ID = 60 (*) / Visit Display Name = Malaria Monitoring / Visit Abbrev = MM / PageID = 10 / Page Display Name = Abb. Physical Exam / Description =)

* Was Abbreviated Physical Exam Done	○ Yes<br>○ No	
* Date of Physical Exam		(DD-MMM-YYYY)
* Time of Physical Exam		(HH24:MI)
AM/PM	○ AM<br>○ PM	

Body System	Assessment	Description	Clinical Significance
General Appearance	○ Normal<br>○ Abnormal<br>○ Not Done		○ Not Clinically Significant<br>○ Clinically Significant
Heart/circulation	○ Normal<br>○ Abnormal<br>○ Not Done		○ Not Clinically Significant<br>○ Clinically Significant
Chest	○ Normal<br>○ Abnormal<br>○ Not Done		○ Not Clinically Significant<br>○ Clinically Significant
Lungs	○ Normal<br>○ Abnormal<br>○ Not Done		○ Not Clinically Significant<br>○ Clinically Significant
Abdomen	○ Normal<br>○ Abnormal<br>○ Not Done		○ Not Clinically Significant<br>○ Clinically Significant
Skin	○ Normal<br>○ Abnormal<br>○ Not Done		○ Not Clinically Significant<br>○ Clinically Significant
Brief Neurological	○ Normal<br>○ Abnormal<br>○ Not Done		○ Not Clinically Significant<br>○ Clinically Significant

Other Body Systems

* Were any other abnormalities noted?
○ No
○ Yes

Physical Exam (extra)

[Revision: Initial revision of study]

(Visit ID = 60 (*) / Visit Display Name = Malaria Monitoring / Visit Abbrev = MM / PageID = 20 (*) / Page Display Name = Physical Exam (extra) / Description = a page to capture other or extra body systems)

* Body System	○ Head/Ears/Eyes/Nose/Throat<br>○ Heart<br>○ Chest<br>○ Lungs<br>○ Abdomen<br>○ Skin<br>○ Neurological exam<br>○ Extremities<br>○ Back<br>○ Dentition<br>○ General Appearance<br>○ Other
* Other Body System	
* Assessment	○ Normal<br>○ Abnormal<br>○ Not Done
* Description of abnormality	
* Clinical Significance	○ Not Clinically Significant<br>○ Clinically Significant

Vital Signs

[Revision: Initial revision of study]

(Visit ID = 60 (*) / Visit Display Name = Malaria Monitoring / Visit Abbrev = MM / PageID = 30 / Page Display Name = Vital Signs / Description = Vital Signs single assessment)

* Were Vital Signs assessed?	○ Yes<br>○ No	
* Date performed	[ ]	(DD-MMM-YYYY)
* At what time were the measurements performed?	[ ]	(HH24:MI)
* Systolic Blood Pressure (mmHg)	[ ]	(format xxx)
* Assessment	○ Normal<br>○ Abnormal NCS<br>○ Abnormal CS	
* Diastolic Blood Pressure (mmHg)	[ ]	(format xxx)
* Assessment	○ Normal<br>○ Abnormal NCS<br>○ Abnormal CS	
* Heart Rate (bpm)	[ ]	(format xxx)
* Assessment	○ Normal<br>○ Abnormal NCS<br>○ Abnormal CS	
* Respiratory Rate (breaths per minute)	[ ]	(format xxx)
* Assessment	○ Normal<br>○ Abnormal NCS<br>○ Abnormal CS	

* Temperature Method	○ Tympanic<br>○ Sublingual	
* Temperature (C)	[ ]	(format xx.x)
* Assessment	○ Normal<br>○ Abnormal NCS<br>○ Abnormal CS	

PCR Collection

[Revision: Initial revision of study]

(Visit ID = 60 (*) / Visit Display Name = Malaria Monitoring / Visit Abbrev = MM / PageID = 110 / Page Display Name = PCR Collection / Description =)

Not Done	☐	
* Date of PCR Collection		(DD-MMM-YYYY)
* Time of PCR Collection		(HH24:MI)

Admission

Visit Date

[Revision: Initial revision of study]

(Visit ID = 70 / Visit Display Name = Admission / Visit Abbrev = ADM / PageID = 120 / Page Display Name = Visit Date / Description =)

Visit Not Done?	☐	
* Visit Date		(DD-MMM-YYYY)

Alcohol Breath Test

[Revision: Initial revision of study]

(Visit ID = 70 / Visit Display Name = Admission / Visit Abbrev = ADM / PageID = 160 / Page Display Name = Alcohol Breath Test / Description = Alcohol Breath Test)

* Was Alcohol Breath Test done	○ Yes<br>○ No	
* Date of Breath Test		(DD-MMM-YYYY)
* Time of Breath Test		(HH24:MI)
* Result	○ Negative<br>○ Positive	

Urine Drug Screen

[Revision: Initial revision of study]

(Visit ID = 70 / Visit Display Name = Admission / Visit Abbrev = ADM / PageID = 80 / Page Display Name = Urine Drug Screen / Description = Drug Screen)

* Was Urine Drug Screen Performed?	○ Yes<br>○ No	
* Date of Drug Screen		(DD-MMM-YYYY)
* Time of Drug Screen		(HH24:MI)

Drug	Result
Amphetamines	○ Negative<br>○ Positive
Methamphetamines	○ Negative<br>○ Positive
Barbiturates	○ Negative<br>○ Positive
Benzodiazepines	○ Negative<br>○ Positive
Cocaine	○ Negative<br>○ Positive
Methadone	○ Negative<br>○ Positive
Opiates	○ Negative<br>○ Positive
Phencyclidine	○ Negative<br>○ Positive
Cannabis	○ Negative<br>○ Positive
Tricyclic antidepressants	○ Negative<br>○ Positive
Acetaminophen (paracetamol)	○ Negative<br>○ Positive

Abb. Physical Exam

[Revision: Initial revision of study]

(Visit ID = 70 / Visit Display Name = Admission / Visit Abbrev = ADM / PageID = 10 / Page Display Name = Abb. Physical Exam / Description =)

* Was Abbreviated Physical Exam Done	○ Yes<br>○ No	
* Date of Physical Exam		(DD-MMM-YYYY)
* Time of Physical Exam		(HH24:MI)
AM/PM	○ AM<br>○ PM	

Body System	Assessment	Description	Clinical Significance
General Appearance	○ Normal<br>○ Abnormal<br>○ Not Done		○ Not Clinically Significant<br>○ Clinically Significant
Heart/circulation	○ Normal<br>○ Abnormal<br>○ Not Done		○ Not Clinically Significant<br>○ Clinically Significant
Chest	○ Normal<br>○ Abnormal<br>○ Not Done		○ Not Clinically Significant<br>○ Clinically Significant
Lungs	○ Normal<br>○ Abnormal<br>○ Not Done		○ Not Clinically Significant<br>○ Clinically Significant
Abdomen	○ Normal<br>○ Abnormal<br>○ Not Done		○ Not Clinically Significant<br>○ Clinically Significant
Skin	○ Normal<br>○ Abnormal<br>○ Not Done		○ Not Clinically Significant<br>○ Clinically Significant
Brief Neurological	○ Normal<br>○ Abnormal<br>○ Not Done		○ Not Clinically Significant<br>○ Clinically Significant

Other Body Systems

* Were any other abnormalities noted? ○ No ○ Yes

Physical Exam (extra)

[Revision: Initial revision of study]

(Visit ID = 70 / Visit Display Name = Admission / Visit Abbrev = ADM / PageID = 20 (*) / Page Display Name = Physical Exam (extra) / Description = a page to capture other or extra body systems)

* Body System	○ Head/Ears/Eyes/Nose/Throat<br>○ Heart<br>○ Chest<br>○ Lungs<br>○ Abdomen<br>○ Skin<br>○ Neurological exam<br>○ Extremities<br>○ Back<br>○ Dentition<br>○ General Appearance<br>○ Other
* Other Body System	
* Assessment	○ Normal<br>○ Abnormal<br>○ Not Done
* Description of abnormality	
* Clinical Significance	○ Not Clinically Significant<br>○ Clinically Significant

ECG

[Revision: Initial revision of study]

(Visit ID = 70 / Visit Display Name = Admission / Visit Abbrev = ADM / PageID = 30 / Page Display Name = ECG / Description = ECG Single)

* Was ECG performed	○ Yes<br>○ No	
* Date of ECG		(DD-MMM-YYYY)
* Time of ECG		(HH24:MI)
* Heart Rate (bpm)		(format xxx)
* PR Interval (msecs)		(format xxx.x)
* RR Interval (msecs)		(format xxxx.x)
* QRS Interval (msecs)		(format xxx.x)
* QT Interval (msecs)		(format xxx.xx)
* QTcF Interval (msecs)		(format xxxxx)
* ECG Interpretation	○ Normal<br>○ Abnormal NCS<br>○ Abnormal CS	
* If Abnormal, describe the abnormality		

Vital Signs

[Revision: Initial revision of study]

(Visit ID = 70 / Visit Display Name = Admission / Visit Abbrev = ADM / PageID = 40 (*) / Page Display Name = Vital Signs / Description = VS at admission (3 times a day))

* Time point	○ Morning<br>○ Midday<br>○ Evening	
* Were Vital Signs assessed?	○ Yes<br>○ No	
* Date performed		(DD-MMM-YYYY)
* Time performed		(HH24:MI)
* Temperature Method	○ Tympanic<br>○ Sublingual	
* Temperature (C)		(format xx.x)
* Assessment	○ Normal<br>○ Abnormal NCS<br>○ Abnormal CS	
* Respiratory Rate (breaths per minute)		(format xxx)
* Assessment	○ Normal<br>○ Abnormal NCS<br>○ Abnormal CS	
* Systolic Blood Pressure (mmHg)		(format xxx)
* Assessment	○ Normal<br>○ Abnormal NCS<br>○ Abnormal CS	

* Diastolic Blood Pressure (mmHg)		(format xxx)
* Assessment	○ Normal<br>○ Abnormal NCS<br>○ Abnormal CS	
* Heart Rate (bpm)		(format xxx)
* Assessment	○ Normal<br>○ Abnormal NCS<br>○ Abnormal CS	

Lab Sample Collection Summary

[Revision: Initial revision of study]

(Visit ID = 70 / Visit Display Name = Admission / Visit Abbrev = ADM / PageID = 50 / Page Display Name = Lab Sample Collection Summary / Description = Lab collection header)

* Were any Labs collected ?	○ No<br>○ Yes	
* Sample date		(DD-MMM-YYYY)
* Sample time		(HH24:MI)

Lab Sample Collection

[Revision: Initial revision of study]

(Visit ID = 70 / Visit Display Name = Admission / Visit Abbrev = ADM / PageID = 60 (*) / Page Display Name = Lab Sample Collection / Description = Labs collected)

* Panel test	○ Biochemistry<br>○ Haematology<br>○ CMV<br>○ EBV<br>○ G6PD<br>○ LFT<br>○ Red Cell Alloantibodies<br>○ Serology<br>○ Duffy<br>○ Rh Status<br>○ Immunology<br>○ Thyroid Tests<br>○ Coagulation<br>○ Endocrinology<br>○ Blood Bank<br>○ Microbiology<br>○ Other
* If Other, specify	

Lab Test Result

[Revision: Initial revision of study]

(Visit ID = 70 / Visit Display Name = Admission / Visit Abbrev = ADM / PageID = 130 / Page Display Name = Lab Test Result / Description = Were any lab tests flagged as being out of range)

* Were any of the lab tests flagged as being out of range ?	○	No
	○	Yes

Lab Test Assessment

[Revision: Initial revision of study]

(Visit ID = 70 / Visit Display Name = Admission / Visit Abbrev = ADM / PageID = 140 (*) / Page Display Name = Lab Test Assessment / Description = Assessment of all out of range labs)

* Choose the category of the tests that were out of range	○ Biochemistry<br>○ Haematology<br>○ CMV<br>○ EBV<br>○ G6PD<br>○ LFT<br>○ Red Cell Alloantibodies<br>○ Serology<br>○ Duffy<br>○ Rh Status<br>○ Immunology<br>○ Thyroid Tests<br>○ Coagulation<br>○ Endocrinology<br>○ Blood Bank<br>○ Microbiology<br>○ Other
* If Other, specify	

* List all tests that were out of range	○ White Blood Cells count<br>○ Neutrophils<br>○ Lymphocytes<br>○ Monocytes<br>○ Eosinophils<br>○ Basophils<br>○ Red blood cell count<br>○ Haemoglobin<br>○ Haematocrit<br>○ Platelet Count<br>○ Reticulocyte Count<br>○ Red Cell Allo-Antibodies<br>○ Duffy Blood Group<br>○ Sodium<br>○ Potassium<br>○ Chloride<br>○ Bicarbonate<br>○ Glucose<br>○ Urea<br>○ Creatinine<br>○ Urate<br>○ Albumin<br>○ Globulin<br>○ Total Protein<br>○ Direct Bilirubin<br>○ Alkaline Phosphatase<br>○ Alanine aminotransferase<br>○ Aspartate aminotransferase

- ○ Calcium Corrected
- ○ Phosphate
- ○ Lactate dehyrogenase
- ○ Magnesium
- ○ Cholesterol
- ○ Triglycerides
- ○ HDL
- ○ LDL
- ○ Cholesterol/HDL
- ○ Total Bilirubin
- ○ eGFR
- ○ Pregnancy Test
- ○ FSH
- ○ HIV
- ○ HBsAg
- ○ anti-HBc
- ○ anti-HCV
- ○ anti-HAV
- ○ anti-HEV
- ○ EBV NA IgG
- ○ EBV VCA IgM
- ○ CMV IgG
- ○ CMV IgM
- ○ Other

* If Other, Specify	
* Clinical Significance	○ Abnormal NCS<br>○ Abnormal CS

If a lab test has been assessed as being Clinically Significant please add as an Adverse Event,

Urinalysis

[Revision: Initial revision of study]

(Visit ID = 70 / Visit Display Name = Admission / Visit Abbrev = ADM / PageID = 170 / Page Display Name = Urinalysis / Description = Urinanalysis)

* Was Urinalysis performed?	○ Yes<br>○ No	
* Date of Urinalysis		(DD-MMM-YYYY)
* Time of Urinalysis		(HH24:MI)

Test	Result	Assessment
Glucose (mmol/L)	○ Negative<br>○ 5.5<br>○ 14<br>○ 28<br>○ >=55	○ Normal<br>○ Abnormal NCS<br>○ Abnormal CS
Bilirubin	○ Negative<br>○ Small<br>○ Moderate<br>○ Large	○ Normal<br>○ Abnormal NCS<br>○ Abnormal CS
Ketone (mmol/L)	○ Negative<br>○ Trace<br>○ 1.5<br>○ 3.9<br>○ 7.8<br>○ >=15.6	○ Normal<br>○ Abnormal NCS<br>○ Abnormal CS
Specific Gravity	○ <=1.005<br>○ 1.010<br>○ 1.015<br>○ 1.020<br>○ 1.025<br>○ >=1.030	○ Normal<br>○ Abnormal NCS<br>○ Abnormal CS
Blood (Ery/uL)	○ Negative<br>○ Trace-lysed<br>○ Trace-intact<br>○ Ca 25<br>○ Ca 80<br>○ Ca 200	○ Normal<br>○ Abnormal NCS<br>○ Abnormal CS

pH	○ 5.0<br>○ 5.5<br>○ 6.0<br>○ 6.5<br>○ 7.0<br>○ 7.5<br>○ 8.0<br>○ 8.5<br>○ >=9.0	○ Normal<br>○ Abnormal NCS<br>○ Abnormal CS
Protein (g/L)	○ Negative<br>○ Trace<br>○ 0.15<br>○ 0.3<br>○ 1.0<br>○ 3.0<br>○ >=3.0	○ Normal<br>○ Abnormal NCS<br>○ Abnormal CS
Urobilinogen (mmol/L)	○ 3.2<br>○ 16<br>○ 33<br>○ 66<br>○ >+131	○ Normal<br>○ Abnormal NCS<br>○ Abnormal CS
Nitrite	○ Negative<br>○ Positive	○ Normal<br>○ Abnormal NCS<br>○ Abnormal CS

Leukocytes (Leu/uL)	○ Negative<br>○ Ca 15<br>○ Ca 70<br>○ Ca 125<br>○ Ca 500	○ Normal<br>○ Abnormal NCS<br>○ Abnormal CS
* Was the sample sent for microscopy	○ No<br>○ Yes	
* Were any microscopy parameters out of range?	○ No<br>○ Yes	

Urine Microscopy Results

[Revision: Initial revision of study]

(Visit ID = 70 / Visit Display Name = Admission / Visit Abbrev = ADM / PageID = 180 (*) / Page Display Name = Urine Microscopy Results / Description = Microscopy Assessment)

* List all tests that were out of range	○ Leucocytes<br>○ Erythrocytes<br>○ Protein<br>○ glucose<br>○ Epithelial Cells<br>○ pH<br>○ Casts<br>○ Blood<br>○ Ketones<br>○ Leucocyte Esterase<br>○ Specific gravity<br>○ Bilirubin<br>○ Urobilinogen<br>○ Nitrites<br>○ Bacteria<br>○ Other
* If Other, Specify	
* Clinical Significance	○ Abnormal NCS<br>○ Abnormal CS

Pregnancy Test

[Revision: Initial revision of study]

(Visit ID = 70 / Visit Display Name = Admission / Visit Abbrev = ADM / PageID = 70 / Page Display Name = Pregnancy Test / Description = Pregnancy test)

* Was Pregnancy Test performed?	○ Yes<br>○ No	
* Serum or Urine	○ Serum<br>○ Urine	
* Date of pregnancy test		(DD-MMM-YYYY)
* Time of pregnancy test		(HH24:MI)
* Result	○ Negative<br>○ Positive	

IP administration

[Revision: Initial revision of study]

(Visit ID = 70 / Visit Display Name = Admission / Visit Abbrev = ADM / PageID = 90 / Page Display Name = IP administration / Description = IP admin)

* Drug	○ Artesunate	
* Date of administration		(DD-MMM-YYYY)
* Time of administration		(HH24:MI)
* Lot Number		
* Total Dose (mg)		
* Route of administration	○ Oral	
* Was IP given as per protocol	○ Yes<br>○ No	
* Was IV administration of Artesunate required?	○ Yes<br>○ No	

IP administration (IV)

[Revision: Initial revision of study]

(Visit ID = 70 / Visit Display Name = Admission / Visit Abbrev = ADM / PageID = 200 (*) / Page Display Name = IP administration (IV) / Description =)

* Drug	○ Artesunate	
* Date of administration	[ ]	(DD-MMM-YYYY)
* Time of administration	[ ]	(HH24:MI)
* Lot Number	[ ]	
* Total Dose (mg)	[ ]	
* Route of administration	○ IV	
* Was IP given as per protocol	○ Yes<br>○ No	

PK Collection

[Revision: Initial revision of study]

(Visit ID = 70 / Visit Display Name = Admission / Visit Abbrev = ADM / PageID = 190 / Page Display Name = PK Collection / Description = PK sample times)

PK Timepoint	Not Done	Date	Time
Pre-dose	☐	(DD-MMM-YYYY)	(HH24:MI)
0.25 hour	☐	(DD-MMM-YYYY)	(HH24:MI)
0.5 hour	☐	(DD-MMM-YYYY)	(HH24:MI)
1 hour	☐	(DD-MMM-YYYY)	(HH24:MI)
1.5 hour	☐	(DD-MMM-YYYY)	(HH24:MI)
2 hour	☐	(DD-MMM-YYYY)	(HH24:MI)
2.5 hour	☐	(DD-MMM-YYYY)	(HH24:MI)
3 hour	☐	(DD-MMM-YYYY)	(HH24:MI)
4 hour	☐	(DD-MMM-YYYY)	(HH24:MI)
6 hour	☐	(DD-MMM-YYYY)	(HH24:MI)
8 hour	☐	(DD-MMM-YYYY)	(HH24:MI)
10 hour	☐	(DD-MMM-YYYY)	(HH24:MI)
12 hour	☐	(DD-MMM-YYYY)	(HH24:MI)

Admission 1

Visit Date

[Revision: Initial revision of study]

(Visit ID = 80 / Visit Display Name = Admission 1 / Visit Abbrev = ADM1 / PageID = 70 / Page Display Name = Visit Date / Description =)

Visit Not Done?	☐	
* Visit Date		(DD-MMM-YYYY)

Abb. Physical Exam

[Revision: Initial revision of study]

(Visit ID = 80 / Visit Display Name = Admission 1 / Visit Abbrev = ADM1 / PageID = 10 / Page Display Name = Abb. Physical Exam / Description =)

* Was Abbreviated Physical Exam Done	○ Yes<br>○ No	
* Date of Physical Exam		(DD-MMM-YYYY)
* Time of Physical Exam		(HH24:MI)
AM/PM	○ AM<br>○ PM	

Body System	Assessment	Description	Clinical Significance
General Appearance	○ Normal<br>○ Abnormal<br>○ Not Done		○ Not Clinically Significant<br>○ Clinically Significant
Heart/circulation	○ Normal<br>○ Abnormal<br>○ Not Done		○ Not Clinically Significant<br>○ Clinically Significant
Chest	○ Normal<br>○ Abnormal<br>○ Not Done		○ Not Clinically Significant<br>○ Clinically Significant
Lungs	○ Normal<br>○ Abnormal<br>○ Not Done		○ Not Clinically Significant<br>○ Clinically Significant
Abdomen	○ Normal<br>○ Abnormal<br>○ Not Done		○ Not Clinically Significant<br>○ Clinically Significant
Skin	○ Normal<br>○ Abnormal<br>○ Not Done		○ Not Clinically Significant<br>○ Clinically Significant
Brief Neurological	○ Normal<br>○ Abnormal<br>○ Not Done		○ Not Clinically Significant<br>○ Clinically Significant

Other Body Systems

* Were any other abnormalities noted?

○ No

○ Yes

Physical Exam (extra)

[Revision: Initial revision of study]

(Visit ID = 80 / Visit Display Name = Admission 1 / Visit Abbrev = ADM1 / PageID = 20 (*) / Page Display Name = Physical Exam (extra) / Description = a page to capture other or extra body systems)

* Body System	○ Head/Ears/Eyes/Nose/Throat<br>○ Heart<br>○ Chest<br>○ Lungs<br>○ Abdomen<br>○ Skin<br>○ Neurological exam<br>○ Extremities<br>○ Back<br>○ Dentition<br>○ General Appearance<br>○ Other
* Other Body System	
* Assessment	○ Normal<br>○ Abnormal<br>○ Not Done
* Description of abnormality	
* Clinical Significance	○ Not Clinically Significant<br>○ Clinically Significant

Vital Signs

[Revision: Initial revision of study]

(Visit ID = 80 / Visit Display Name = Admission 1 / Visit Abbrev = ADM1 / PageID = 30 (*) / Page Display Name = Vital Signs / Description = VS at admission (3 times a day))

* Time point	○ Morning<br>○ Midday<br>○ Evening	
* Were Vital Signs assessed?	○ Yes<br>○ No	
* Date performed		(DD-MMM-YYYY)
* Time performed		(HH24:MI)
* Temperature Method	○ Tympanic<br>○ Sublingual	
* Temperature (C)		(format xx.x)
* Assessment	○ Normal<br>○ Abnormal NCS<br>○ Abnormal CS	
* Respiratory Rate (breaths per minute)		(format xxx)
* Assessment	○ Normal<br>○ Abnormal NCS<br>○ Abnormal CS	
* Systolic Blood Pressure (mmHg)		(format xxx)
* Assessment	○ Normal<br>○ Abnormal NCS<br>○ Abnormal CS	

* Diastolic Blood Pressure (mmHg)		(format xxx)
* Assessment	○ Normal<br>○ Abnormal NCS<br>○ Abnormal CS	
* Heart Rate (bpm)		(format xxx)
* Assessment	○ Normal<br>○ Abnormal NCS<br>○ Abnormal CS	

Admission 2

Visit Date

[Revision: Initial revision of study]

(Visit ID = 90 / Visit Display Name = Admission 2 / Visit Abbrev = ADM2 / PageID = 60 / Page Display Name = Visit Date / Description =)

Visit Not Done?	☐	
* Visit Date		(DD-MMM-YYYY)

Abb. Physical Exam

[Revision: Initial revision of study]

(Visit ID = 90 / Visit Display Name = Admission 2 / Visit Abbrev = ADM2 / PageID = 10 / Page Display Name = Abb. Physical Exam / Description =)

* Was Abbreviated Physical Exam Done	○ Yes<br>○ No	
* Date of Physical Exam		(DD-MMM-YYYY)
* Time of Physical Exam		(HH24:MI)
AM/PM	○ AM<br>○ PM	

Body System	Assessment	Description	Clinical Significance
General Appearance	○ Normal<br>○ Abnormal<br>○ Not Done		○ Not Clinically Significant<br>○ Clinically Significant
Heart/circulation	○ Normal<br>○ Abnormal<br>○ Not Done		○ Not Clinically Significant<br>○ Clinically Significant
Chest	○ Normal<br>○ Abnormal<br>○ Not Done		○ Not Clinically Significant<br>○ Clinically Significant
Lungs	○ Normal<br>○ Abnormal<br>○ Not Done		○ Not Clinically Significant<br>○ Clinically Significant
Abdomen	○ Normal<br>○ Abnormal<br>○ Not Done		○ Not Clinically Significant<br>○ Clinically Significant
Skin	○ Normal<br>○ Abnormal<br>○ Not Done		○ Not Clinically Significant<br>○ Clinically Significant
Brief Neurological	○ Normal<br>○ Abnormal<br>○ Not Done		○ Not Clinically Significant<br>○ Clinically Significant

Other Body Systems

* Were any other abnormalities noted?
○ No
○ Yes

Physical Exam (extra)

[Revision: Initial revision of study]

(Visit ID = 90 / Visit Display Name = Admission 2 / Visit Abbrev = ADM2 / PageID = 20 (*) / Page Display Name = Physical Exam (extra) / Description = a page to capture other or extra body systems)

* Body System	○ Head/Ears/Eyes/Nose/Throat<br>○ Heart<br>○ Chest<br>○ Lungs<br>○ Abdomen<br>○ Skin<br>○ Neurological exam<br>○ Extremities<br>○ Back<br>○ Dentition<br>○ General Appearance<br>○ Other
* Other Body System	
* Assessment	○ Normal<br>○ Abnormal<br>○ Not Done
* Description of abnormality	
* Clinical Significance	○ Not Clinically Significant<br>○ Clinically Significant

Vital Signs

[Revision: Initial revision of study]

(Visit ID = 90 / Visit Display Name = Admission 2 / Visit Abbrev = ADM2 / PageID = 30 (*) / Page Display Name = Vital Signs / Description = VS at admission (3 times a day))

* Time point	○ Morning<br>○ Midday<br>○ Evening	
* Were Vital Signs assessed?	○ Yes<br>○ No	
* Date performed		(DD-MMM-YYYY)
* Time performed		(HH24:MI)
* Temperature Method	○ Tympanic<br>○ Sublingual	
* Temperature (C)		(format xx.x)
* Assessment	○ Normal<br>○ Abnormal NCS<br>○ Abnormal CS	
* Respiratory Rate (breaths per minute)		(format xxx)
* Assessment	○ Normal<br>○ Abnormal NCS<br>○ Abnormal CS	
* Systolic Blood Pressure (mmHg)		(format xxx)
* Assessment	○ Normal<br>○ Abnormal NCS<br>○ Abnormal CS	

* Diastolic Blood Pressure (mmHg)		(format xxx)
* Assessment	○ Normal<br>○ Abnormal NCS<br>○ Abnormal CS	
* Heart Rate (bpm)		(format xxx)
* Assessment	○ Normal<br>○ Abnormal NCS<br>○ Abnormal CS	

Admission 3

Visit Date

[Revision: Initial revision of study]

(Visit ID = 100 / Visit Display Name = Admission 3 / Visit Abbrev = ADM3 / PageID = 70 / Page Display Name = Visit Date / Description =)

Visit Not Done?	☐	
* Visit Date		(DD-MMM-YYYY)

Abb. Physical Exam

[Revision: Initial revision of study]

(Visit ID = 100 / Visit Display Name = Admission 3 / Visit Abbrev = ADM3 / PageID = 10 / Page Display Name = Abb. Physical Exam / Description =)

* Was Abbreviated Physical Exam Done	○ Yes<br>○ No	
* Date of Physical Exam		(DD-MMM-YYYY)
* Time of Physical Exam		(HH24:MI)
AM/PM	○ AM<br>○ PM	

Body System	Assessment	Description	Clinical Significance
General Appearance	○ Normal<br>○ Abnormal<br>○ Not Done		○ Not Clinically Significant<br>○ Clinically Significant
Heart/circulation	○ Normal<br>○ Abnormal<br>○ Not Done		○ Not Clinically Significant<br>○ Clinically Significant
Chest	○ Normal<br>○ Abnormal<br>○ Not Done		○ Not Clinically Significant<br>○ Clinically Significant
Lungs	○ Normal<br>○ Abnormal<br>○ Not Done		○ Not Clinically Significant<br>○ Clinically Significant
Abdomen	○ Normal<br>○ Abnormal<br>○ Not Done		○ Not Clinically Significant<br>○ Clinically Significant
Skin	○ Normal<br>○ Abnormal<br>○ Not Done		○ Not Clinically Significant<br>○ Clinically Significant
Brief Neurological	○ Normal<br>○ Abnormal<br>○ Not Done		○ Not Clinically Significant<br>○ Clinically Significant

Other Body Systems

* Were any other abnormalities noted? ○ No ○ Yes

Physical Exam (extra)

[Revision: Initial revision of study]

(Visit ID = 100 / Visit Display Name = Admission 3 / Visit Abbrev = ADM3 / PageID = 20 (*) / Page Display Name = Physical Exam (extra) / Description = a page to capture other or extra body systems)

* Body System	○ Head/Ears/Eyes/Nose/Throat<br>○ Heart<br>○ Chest<br>○ Lungs<br>○ Abdomen<br>○ Skin<br>○ Neurological exam<br>○ Extremities<br>○ Back<br>○ Dentition<br>○ General Appearance<br>○ Other
* Other Body System	
* Assessment	○ Normal<br>○ Abnormal<br>○ Not Done
* Description of abnormality	
* Clinical Significance	○ Not Clinically Significant<br>○ Clinically Significant

Vital Signs

[Revision: Initial revision of study]

(Visit ID = 100 / Visit Display Name = Admission 3 / Visit Abbrev = ADM3 / PageID = 30 / Page Display Name = Vital Signs / Description = Vital Signs single assessment)

* Were Vital Signs assessed?	○ Yes<br>○ No	
* Date performed		(DD-MMM-YYYY)
* At what time were the measurements performed?		(HH24:MI)
* Systolic Blood Pressure (mmHg)		(format xxx)
* Assessment	○ Normal<br>○ Abnormal NCS<br>○ Abnormal CS	
* Diastolic Blood Pressure (mmHg)		(format xxx)
* Assessment	○ Normal<br>○ Abnormal NCS<br>○ Abnormal CS	
* Heart Rate (bpm)		(format xxx)
* Assessment	○ Normal<br>○ Abnormal NCS<br>○ Abnormal CS	
* Respiratory Rate (breaths per minute)		(format xxx)
* Assessment	○ Normal<br>○ Abnormal NCS<br>○ Abnormal CS	

* Temperature Method	○ Tympanic<br>○ Sublingual	
* Temperature (C)	[ ]	(format xx.x)
* Assessment	○ Normal<br>○ Abnormal NCS<br>○ Abnormal CS	

Lab Sample Collection Summary

[Revision: Initial revision of study]

(Visit ID = 100 / Visit Display Name = Admission 3 / Visit Abbrev = ADM3 / PageID = 50 / Page Display Name = Lab Sample Collection Summary / Description = Lab collection header)

* Were any Labs collected ?	○ No<br>○ Yes	
* Sample date		(DD-MMM-YYYY)
* Sample time		(HH24:MI)

Lab Sample Collection

[Revision: Initial revision of study]

(Visit ID = 100 / Visit Display Name = Admission 3 / Visit Abbrev = ADM3 / PageID = 60 (*) / Page Display Name = Lab Sample Collection / Description = Labs collected)

* Panel test	○ Biochemistry<br>○ Haematology<br>○ CMV<br>○ EBV<br>○ G6PD<br>○ LFT<br>○ Red Cell Alloantibodies<br>○ Serology<br>○ Duffy<br>○ Rh Status<br>○ Immunology<br>○ Thyroid Tests<br>○ Coagulation<br>○ Endocrinology<br>○ Blood Bank<br>○ Microbiology<br>○ Other
* If Other, specify	

Lab Test Result

[Revision: Initial revision of study]

(Visit ID = 100 / Visit Display Name = Admission 3 / Visit Abbrev = ADM3 / PageID = 80 / Page Display Name = Lab Test Result / Description = Were any lab tests flagged as being out of range)

* Were any of the lab tests flagged as being out of range ?	○ No<br>○ Yes

Lab Test Assessment

[Revision: Initial revision of study]

(Visit ID = 100 / Visit Display Name = Admission 3 / Visit Abbrev = ADM3 / PageID = 90 (*) / Page Display Name = Lab Test Assessment / Description = Assessment of all out of range labs)

* Choose the category of the tests that were out of range	○ Biochemistry<br>○ Haematology<br>○ CMV<br>○ EBV<br>○ G6PD<br>○ LFT<br>○ Red Cell Alloantibodies<br>○ Serology<br>○ Duffy<br>○ Rh Status<br>○ Immunology<br>○ Thyroid Tests<br>○ Coagulation<br>○ Endocrinology<br>○ Blood Bank<br>○ Microbiology<br>○ Other
* If Other, specify	

* List all tests that were out of range

- ○ White Blood Cells count
- ○ Neutrophils
- ○ Lymphocytes
- ○ Monocytes
- ○ Eosinophils
- ○ Basophils
- ○ Red blood cell count
- ○ Haemoglobin
- ○ Haematocrit
- ○ Platelet Count
- ○ Reticulocyte Count
- ○ Red Cell Allo-Antibodies
- ○ Duffy Blood Group
- ○ Sodium
- ○ Potassium
- ○ Chloride
- ○ Bicarbonate
- ○ Glucose
- ○ Urea
- ○ Creatinine
- ○ Urate
- ○ Albumin
- ○ Globulin
- ○ Total Protein
- ○ Direct Bilirubin
- ○ Alkaline Phosphatase
- ○ Alanine aminotransferase
- ○ Aspartate aminotransferase

	○ Calcium Corrected<br>○ Phosphate<br>○ Lactate dehyrogenase<br>○ Magnesium<br>○ Cholesterol<br>○ Triglycerides<br>○ HDL<br>○ LDL<br>○ Cholesterol/HDL<br>○ Total Bilirubin<br>○ eGFR<br>○ Pregnancy Test<br>○ FSH<br>○ HIV<br>○ HBsAg<br>○ anti-HBc<br>○ anti-HCV<br>○ anti-HAV<br>○ anti-HEV<br>○ EBV NA IgG<br>○ EBV VCA IgM<br>○ CMV IgG<br>○ CMV IgM<br>○ Other
* If Other, Specify	
* Clinical Significance	○ Abnormal NCS<br>○ Abnormal CS

If a lab test has been assessed as being Clinically Significant please add as an Adverse Event,

Safety Monitoring

Visit Date

[Revision: Initial revision of study]

(Visit ID = 110 (*) / Visit Display Name = Safety Monitoring / Visit Abbrev = SMV / PageID = 60 / Page Display Name = Visit Date / Description = Visit Date)

Visit Not Done?	☐	
* Visit Date		(DD-MMM-YYYY)
* AM or PM	○ AM<br>○ PM	

Abb. Physical Exam

[Revision: Initial revision of study]

(Visit ID = 110 (*) / Visit Display Name = Safety Monitoring / Visit Abbrev = SMV / PageID = 10 / Page Display Name = Abb. Physical Exam / Description =)

* Was Abbreviated Physical Exam Done	○ Yes<br>○ No	
* Date of Physical Exam	[ ]	(DD-MMM-YYYY)
* Time of Physical Exam	[ ]	(HH24:MI)
AM/PM	○ AM<br>○ PM	

Body System	Assessment	Description	Clinical Significance
General Appearance	○ Normal<br>○ Abnormal<br>○ Not Done		○ Not Clinically Significant<br>○ Clinically Significant
Heart/circulation	○ Normal<br>○ Abnormal<br>○ Not Done		○ Not Clinically Significant<br>○ Clinically Significant
Chest	○ Normal<br>○ Abnormal<br>○ Not Done		○ Not Clinically Significant<br>○ Clinically Significant
Lungs	○ Normal<br>○ Abnormal<br>○ Not Done		○ Not Clinically Significant<br>○ Clinically Significant
Abdomen	○ Normal<br>○ Abnormal<br>○ Not Done		○ Not Clinically Significant<br>○ Clinically Significant
Skin	○ Normal<br>○ Abnormal<br>○ Not Done		○ Not Clinically Significant<br>○ Clinically Significant
Brief Neurological	○ Normal<br>○ Abnormal<br>○ Not Done		○ Not Clinically Significant<br>○ Clinically Significant

Other Body Systems

* Were any other abnormalities noted?
○ No
○ Yes

Physical Exam (extra)

[Revision: Initial revision of study]

(Visit ID = 110 (*) / Visit Display Name = Safety Monitoring / Visit Abbrev = SMV / PageID = 20 (*) / Page Display Name = Physical Exam (extra) / Description = a page to capture other or extra body systems)

* Body System	○ Head/Ears/Eyes/Nose/Throat<br>○ Heart<br>○ Chest<br>○ Lungs<br>○ Abdomen<br>○ Skin<br>○ Neurological exam<br>○ Extremities<br>○ Back<br>○ Dentition<br>○ General Appearance<br>○ Other
* Other Body System	
* Assessment	○ Normal<br>○ Abnormal<br>○ Not Done
* Description of abnormality	
* Clinical Significance	○ Not Clinically Significant<br>○ Clinically Significant

Vital Signs

[Revision: Initial revision of study]

(Visit ID = 110 (*) / Visit Display Name = Safety Monitoring / Visit Abbrev = SMV / PageID = 30 / Page Display Name = Vital Signs / Description = Vital Signs single assessment)

* Were Vital Signs assessed?	○ Yes<br>○ No	
* Date performed	[ ]	(DD-MMM-YYYY)
* At what time were the measurements performed?	[ ]	(HH24:MI)
* Systolic Blood Pressure (mmHg)	[ ]	(format xxx)
* Assessment	○ Normal<br>○ Abnormal NCS<br>○ Abnormal CS	
* Diastolic Blood Pressure (mmHg)	[ ]	(format xxx)
* Assessment	○ Normal<br>○ Abnormal NCS<br>○ Abnormal CS	
* Heart Rate (bpm)	[ ]	(format xxx)
* Assessment	○ Normal<br>○ Abnormal NCS<br>○ Abnormal CS	
* Respiratory Rate (breaths per minute)	[ ]	(format xxx)
* Assessment	○ Normal<br>○ Abnormal NCS<br>○ Abnormal CS	

* Temperature Method	○ Tympanic<br>○ Sublingual	
* Temperature (C)	[ ]	(format xx.x)
* Assessment	○ Normal<br>○ Abnormal NCS<br>○ Abnormal CS	

ECG

[Revision: Initial revision of study]

(Visit ID = 110 (*) / Visit Display Name = Safety Monitoring / Visit Abbrev = SMV / PageID = 120 / Page Display Name = ECG / Description = ECG Single)

* Was ECG performed	○ Yes<br>○ No	
* Date of ECG		(DD-MMM-YYYY)
* Time of ECG		(HH24:MI)
* Heart Rate (bpm)		(format xxx)
* PR Interval (msecs)		(format xxx.x)
* RR Interval (msecs)		(format xxxx.x)
* QRS Interval (msecs)		(format xxx.x)
* QT Interval (msecs)		(format xxx.xx)
* QTcF Interval (msecs)		(format xxxxx)
* ECG Interpretation	○ Normal<br>○ Abnormal NCS<br>○ Abnormal CS	
* If Abnormal, describe the abnormality		

Lab Sample Collection Summary

[Revision: Initial revision of study]

(Visit ID = 110 (*) / Visit Display Name = Safety Monitoring / Visit Abbrev = SMV / PageID = 40 / Page Display Name = Lab Sample Collection Summary / Description = Lab collection header)

* Were any Labs collected ?	○ No<br>○ Yes	
* Sample date		(DD-MMM-YYYY)
* Sample time		(HH24:MI)

Lab Sample Collection

[Revision: Initial revision of study]

(Visit ID = 110 (*) / Visit Display Name = Safety Monitoring / Visit Abbrev = SMV / PageID = 50 (*) / Page Display Name = Lab Sample Collection / Description = Labs collected)

* Panel test	○ Biochemistry<br>○ Haematology<br>○ CMV<br>○ EBV<br>○ G6PD<br>○ LFT<br>○ Red Cell Alloantibodies<br>○ Serology<br>○ Duffy<br>○ Rh Status<br>○ Immunology<br>○ Thyroid Tests<br>○ Coagulation<br>○ Endocrinology<br>○ Blood Bank<br>○ Microbiology<br>○ Other
* If Other, specify	

Lab Test Result

[Revision: Initial revision of study]

(Visit ID = 110 (*) / Visit Display Name = Safety Monitoring / Visit Abbrev = SMV / PageID = 70 / Page Display Name = Lab Test Result / Description = Were any lab tests flagged as being out of range)

* Were any of the lab tests flagged as being out of range ?	○ No<br>○ Yes

Lab Test Assessment

[Revision: Initial revision of study]

(Visit ID = 110 (*) / Visit Display Name = Safety Monitoring / Visit Abbrev = SMV / PageID = 80 (*) / Page Display Name = Lab Test Assessment / Description = Assessment of all out of range labs)

* Choose the category of the tests that were out of range	○ Biochemistry<br>○ Haematology<br>○ CMV<br>○ EBV<br>○ G6PD<br>○ LFT<br>○ Red Cell Alloantibodies<br>○ Serology<br>○ Duffy<br>○ Rh Status<br>○ Immunology<br>○ Thyroid Tests<br>○ Coagulation<br>○ Endocrinology<br>○ Blood Bank<br>○ Microbiology<br>○ Other
* If Other, specify	

* List all tests that were out of range	○ White Blood Cells count<br>○ Neutrophils<br>○ Lymphocytes<br>○ Monocytes<br>○ Eosinophils<br>○ Basophils<br>○ Red blood cell count<br>○ Haemoglobin<br>○ Haematocrit<br>○ Platelet Count<br>○ Reticulocyte Count<br>○ Red Cell Allo-Antibodies<br>○ Duffy Blood Group<br>○ Sodium<br>○ Potassium<br>○ Chloride<br>○ Bicarbonate<br>○ Glucose<br>○ Urea<br>○ Creatinine<br>○ Urate<br>○ Albumin<br>○ Globulin<br>○ Total Protein<br>○ Direct Bilirubin<br>○ Alkaline Phosphatase<br>○ Alanine aminotransferase<br>○ Aspartate aminotransferase

- ○ Calcium Corrected
- ○ Phosphate
- ○ Lactate dehyrogenase
- ○ Magnesium
- ○ Cholesterol
- ○ Triglycerides
- ○ HDL
- ○ LDL
- ○ Cholesterol/HDL
- ○ Total Bilirubin
- ○ eGFR
- ○ Pregnancy Test
- ○ FSH
- ○ HIV
- ○ HBsAg
- ○ anti-HBc
- ○ anti-HCV
- ○ anti-HAV
- ○ anti-HEV
- ○ EBV NA IgG
- ○ EBV VCA IgM
- ○ CMV IgG
- ○ CMV IgM
- ○ Other

* If Other, Specify	
* Clinical Significance	○ Abnormal NCS<br>○ Abnormal CS

If a lab test has been assessed as being Clinically Significant please add as an Adverse Event,

Urinalysis

[Revision: Initial revision of study]

(Visit ID = 110 (*) / Visit Display Name = Safety Monitoring / Visit Abbrev = SMV / PageID = 130 / Page Display Name = Urinalysis / Description = Urinanalysis)

* Was Urinalysis performed?	○ Yes<br>○ No	
* Date of Urinalysis		(DD-MMM-YYYY)
* Time of Urinalysis		(HH24:MI)

Test	Result	Assessment
Glucose (mmol/L)	○ Negative<br>○ 5.5<br>○ 14<br>○ 28<br>○ >=55	○ Normal<br>○ Abnormal NCS<br>○ Abnormal CS
Bilirubin	○ Negative<br>○ Small<br>○ Moderate<br>○ Large	○ Normal<br>○ Abnormal NCS<br>○ Abnormal CS
Ketone (mmol/L)	○ Negative<br>○ Trace<br>○ 1.5<br>○ 3.9<br>○ 7.8<br>○ >=15.6	○ Normal<br>○ Abnormal NCS<br>○ Abnormal CS
Specific Gravity	○ <=1.005<br>○ 1.010<br>○ 1.015<br>○ 1.020<br>○ 1.025<br>○ >=1.030	○ Normal<br>○ Abnormal NCS<br>○ Abnormal CS
Blood (Ery/uL)	○ Negative<br>○ Trace-lysed<br>○ Trace-intact<br>○ Ca 25<br>○ Ca 80<br>○ Ca 200	○ Normal<br>○ Abnormal NCS<br>○ Abnormal CS

pH	○ 5.0<br>○ 5.5<br>○ 6.0<br>○ 6.5<br>○ 7.0<br>○ 7.5<br>○ 8.0<br>○ 8.5<br>○ >=9.0	○ Normal<br>○ Abnormal NCS<br>○ Abnormal CS
Protein (g/L)	○ Negative<br>○ Trace<br>○ 0.15<br>○ 0.3<br>○ 1.0<br>○ 3.0<br>○ >=3.0	○ Normal<br>○ Abnormal NCS<br>○ Abnormal CS
Urobilinogen (mmol/L)	○ 3.2<br>○ 16<br>○ 33<br>○ 66<br>○ >+131	○ Normal<br>○ Abnormal NCS<br>○ Abnormal CS
Nitrite	○ Negative<br>○ Positive	○ Normal<br>○ Abnormal NCS<br>○ Abnormal CS

Leukocytes (Leu/uL)	○ Negative<br>○ Ca 15<br>○ Ca 70<br>○ Ca 125<br>○ Ca 500	○ Normal<br>○ Abnormal NCS<br>○ Abnormal CS
* Was the sample sent for microscopy	○ No<br>○ Yes	
* Were any microscopy parameters out of range?	○ No<br>○ Yes	

Urine Microscopy Results

[Revision: Initial revision of study]

(Visit ID = 110 (*) / Visit Display Name = Safety Monitoring / Visit Abbrev = SMV / PageID = 140 (*) / Page Display Name = Urine Microscopy Results / Description = Microscopy Assessment)

* List all tests that were out of range	○ Leucocytes<br>○ Erythrocytes<br>○ Protein<br>○ glucose<br>○ Epithelial Cells<br>○ pH<br>○ Casts<br>○ Blood<br>○ Ketones<br>○ Leucocyte Esterase<br>○ Specific gravity<br>○ Bilirubin<br>○ Urobilinogen<br>○ Nitrites<br>○ Bacteria<br>○ Other
* If Other, Specify	
* Clinical Significance	○ Abnormal NCS<br>○ Abnormal CS

PCR Collection

[Revision: Initial revision of study]

(Visit ID = 110 (*) / Visit Display Name = Safety Monitoring / Visit Abbrev = SMV / PageID = 90 / Page Display Name = PCR Collection / Description =)

Not Done	☐	
* Date of PCR Collection		(DD-MMM-YYYY)
* Time of PCR Collection		(HH24:MI)

Feeding Summary

[Revision: Initial revision of study]

(Visit ID = 110 (*) / Visit Display Name = Safety Monitoring / Visit Abbrev = SMV / PageID = 100 / Page Display Name = Feeding Summary / Description = Was Feeding performed)

* Was Feeding done at this visit?	○ No<br>○ Yes

MFA/DFA

[Revision: Initial revision of study]

(Visit ID = 110 (*) / Visit Display Name = Safety Monitoring / Visit Abbrev = SMV / PageID = 110 (*) / Page Display Name = MFA/DFA / Description = Feeding (direct / indirect))

* Type of Feeding	○ DFA<br>○ MFA	
* Date of collection		(DD-MMM-YYYY)
* Site of feeding	○ Right Forearm<br>○ Left Forearm<br>○ Right Thigh<br>○ Left Thigh<br>○ Right Calf<br>○ Left Calf<br>○ Other	
* If Other, Specify		
* Start Time of collection		(HH24:MI)
* End time of collection		(HH24:MI)

End of Study

Visit Date

[Revision: Initial revision of study]

(Visit ID = 120 / Visit Display Name = End of Study / Visit Abbrev = EOS / PageID = 100 / Page Display Name = Visit Date / Description =)

Visit Not Done?	☐	
* Visit Date		(DD-MMM-YYYY)

Physical Exam

[Revision: Initial revision of study]

(Visit ID = 120 / Visit Display Name = End of Study / Visit Abbrev = EOS / PageID = 10 / Page Display Name = Physical Exam / Description = Physical Exam)

* Was Physical Exam Done	○ Yes<br>○ No	
* Date of Physical Exam		(DD-MMM-YYYY)
* Time of Physical Exam		(HH24:MI)
AM/PM	○ AM<br>○ PM	

Body System	Assessment	Description	Clinical Significance
Head/Neck/Ears/Eyes/Nose/Throat	○ Normal ○ Abnormal ○ Not Done		○ Not Clinically Significant ○ Clinically Significant
Heart/circulation	○ Normal ○ Abnormal ○ Not Done		○ Not Clinically Significant ○ Clinically Significant
Chest	○ Normal ○ Abnormal ○ Not Done		○ Not Clinically Significant ○ Clinically Significant
Lungs	○ Normal ○ Abnormal ○ Not Done		○ Not Clinically Significant ○ Clinically Significant
Abdomen	○ Normal ○ Abnormal ○ Not Done		○ Not Clinically Significant ○ Clinically Significant
Skin	○ Normal ○ Abnormal ○ Not Done		○ Not Clinically Significant ○ Clinically Significant
Neurological	○ Normal ○ Abnormal ○ Not Done		○ Not Clinically Significant ○ Clinically Significant

Extremities	○ Normal<br>○ Abnormal<br>○ Not Done		○ Not Clinically Significant<br>○ Clinically Significant
Back	○ Normal<br>○ Abnormal<br>○ Not Done		○ Not Clinically Significant<br>○ Clinically Significant
Dentition	○ Normal<br>○ Abnormal<br>○ Not Done		○ Not Clinically Significant<br>○ Clinically Significant

Other Body Systems

* Were any other abnormalities noted?	○ No<br>○ Yes

Physical Exam (extra)

[Revision: Initial revision of study]

(Visit ID = 120 / Visit Display Name = End of Study / Visit Abbrev = EOS / PageID = 20 (*) / Page Display Name = Physical Exam (extra) / Description = a page to capture other or extra body systems)

* Body System	○ Head/Ears/Eyes/Nose/Throat<br>○ Heart<br>○ Chest<br>○ Lungs<br>○ Abdomen<br>○ Skin<br>○ Neurological exam<br>○ Extremities<br>○ Back<br>○ Dentition<br>○ General Appearance<br>○ Other
* Other Body System	
* Assessment	○ Normal<br>○ Abnormal<br>○ Not Done
* Description of abnormality	
* Clinical Significance	○ Not Clinically Significant<br>○ Clinically Significant

ECG

[Revision: Initial revision of study]

(Visit ID = 120 / Visit Display Name = End of Study / Visit Abbrev = EOS / PageID = 30 / Page Display Name = ECG / Description = ECG Single)

* Was ECG performed	○ Yes<br>○ No	
* Date of ECG		(DD-MMM-YYYY)
* Time of ECG		(HH24:MI)
* Heart Rate (bpm)		(format xxx)
* PR Interval (msecs)		(format xxx.x)
* RR Interval (msecs)		(format xxxx.x)
* QRS Interval (msecs)		(format xxx.x)
* QT Interval (msecs)		(format xxx.xx)
* QTcF Interval (msecs)		(format xxxxx)
* ECG Interpretation	○ Normal<br>○ Abnormal NCS<br>○ Abnormal CS	
* If Abnormal, describe the abnormality		

Vital Signs

[Revision: Initial revision of study]

(Visit ID = 120 / Visit Display Name = End of Study / Visit Abbrev = EOS / PageID = 40 / Page Display Name = Vital Signs / Description = Vital Signs single assessment)

* Were Vital Signs assessed?	○ Yes<br>○ No	
* Date performed		(DD-MMM-YYYY)
* At what time were the measurements performed?		(HH24:MI)
* Systolic Blood Pressure (mmHg)		(format xxx)
* Assessment	○ Normal<br>○ Abnormal NCS<br>○ Abnormal CS	
* Diastolic Blood Pressure (mmHg)		(format xxx)
* Assessment	○ Normal<br>○ Abnormal NCS<br>○ Abnormal CS	
* Heart Rate (bpm)		(format xxx)
* Assessment	○ Normal<br>○ Abnormal NCS<br>○ Abnormal CS	
* Respiratory Rate (breaths per minute)		(format xxx)
* Assessment	○ Normal<br>○ Abnormal NCS<br>○ Abnormal CS	

* Temperature Method	○ Tympanic<br>○ Sublingual	
* Temperature (C)		(format xx.x)
* Assessment	○ Normal<br>○ Abnormal NCS<br>○ Abnormal CS	

Lab Sample Collection Summary

[Revision: Initial revision of study]

(Visit ID = 120 / Visit Display Name = End of Study / Visit Abbrev = EOS / PageID = 50 / Page Display Name = Lab Sample Collection Summary / Description = Lab collection header)

* Were any Labs collected ?	○ No<br>○ Yes	
* Sample date		(DD-MMM-YYYY)
* Sample time		(HH24:MI)

Lab Sample Collection

[Revision: Initial revision of study]

(Visit ID = 120 / Visit Display Name = End of Study / Visit Abbrev = EOS / PageID = 60 (*) / Page Display Name = Lab Sample Collection / Description = Labs collected)

* Panel test	○ Biochemistry<br>○ Haematology<br>○ CMV<br>○ EBV<br>○ G6PD<br>○ LFT<br>○ Red Cell Alloantibodies<br>○ Serology<br>○ Duffy<br>○ Rh Status<br>○ Immunology<br>○ Thyroid Tests<br>○ Coagulation<br>○ Endocrinology<br>○ Blood Bank<br>○ Microbiology<br>○ Other
* If Other, specify	

Lab Test Result

[Revision: Initial revision of study]

(Visit ID = 120 / Visit Display Name = End of Study / Visit Abbrev = EOS / PageID = 110 / Page Display Name = Lab Test Result / Description = Were any lab tests flagged as being out of range)

* Were any of the lab tests flagged as being out of range ?	○ No<br>○ Yes

Lab Test Assessment

[Revision: Initial revision of study]

(Visit ID = 120 / Visit Display Name = End of Study / Visit Abbrev = EOS / PageID = 120 (*) / Page Display Name = Lab Test Assessment / Description = Assessment of all out of range labs)

* Choose the category of the tests that were out of range	○ Biochemistry<br>○ Haematology<br>○ CMV<br>○ EBV<br>○ G6PD<br>○ LFT<br>○ Red Cell Alloantibodies<br>○ Serology<br>○ Duffy<br>○ Rh Status<br>○ Immunology<br>○ Thyroid Tests<br>○ Coagulation<br>○ Endocrinology<br>○ Blood Bank<br>○ Microbiology<br>○ Other
* If Other, specify	

* List all tests that were out of range	○ White Blood Cells count<br>○ Neutrophils<br>○ Lymphocytes<br>○ Monocytes<br>○ Eosinophils<br>○ Basophils<br>○ Red blood cell count<br>○ Haemoglobin<br>○ Haematocrit<br>○ Platelet Count<br>○ Reticulocyte Count<br>○ Red Cell Allo-Antibodies<br>○ Duffy Blood Group<br>○ Sodium<br>○ Potassium<br>○ Chloride<br>○ Bicarbonate<br>○ Glucose<br>○ Urea<br>○ Creatinine<br>○ Urate<br>○ Albumin<br>○ Globulin<br>○ Total Protein<br>○ Direct Bilirubin<br>○ Alkaline Phosphatase<br>○ Alanine aminotransferase<br>○ Aspartate aminotransferase

	○ Calcium Corrected<br>○ Phosphate<br>○ Lactate dehyrogenase<br>○ Magnesium<br>○ Cholesterol<br>○ Triglycerides<br>○ HDL<br>○ LDL<br>○ Cholesterol/HDL<br>○ Total Bilirubin<br>○ eGFR<br>○ Pregnancy Test<br>○ FSH<br>○ HIV<br>○ HBsAg<br>○ anti-HBc<br>○ anti-HCV<br>○ anti-HAV<br>○ anti-HEV<br>○ EBV NA IgG<br>○ EBV VCA IgM<br>○ CMV IgG<br>○ CMV IgM<br>○ Other
* If Other, Specify	
* Clinical Significance	○ Abnormal NCS<br>○ Abnormal CS

If a lab test has been assessed as being Clinically Significant please add as an Adverse Event,

Pregnancy Test

[Revision: Initial revision of study]

(Visit ID = 120 / Visit Display Name = End of Study / Visit Abbrev = EOS / PageID = 70 / Page Display Name = Pregnancy Test / Description = Pregnancy test)

* Was Pregnancy Test performed?	○ Yes<br>○ No	
* Serum or Urine	○ Serum<br>○ Urine	
* Date of pregnancy test		(DD-MMM-YYYY)
* Time of pregnancy test		(HH24:MI)
* Result	○ Negative<br>○ Positive	

Urinalysis

[Revision: Initial revision of study]

(Visit ID = 120 / Visit Display Name = End of Study / Visit Abbrev = EOS / PageID = 80 / Page Display Name = Urinalysis / Description = Urinanalysis)

* Was Urinalysis performed?	○ Yes<br>○ No	
* Date of Urinalysis		(DD-MMM-YYYY)
* Time of Urinalysis		(HH24:MI)

Test	Result	Assessment
Glucose (mmol/L)	○ Negative<br>○ 5.5<br>○ 14<br>○ 28<br>○ >=55	○ Normal<br>○ Abnormal NCS<br>○ Abnormal CS
Bilirubin	○ Negative<br>○ Small<br>○ Moderate<br>○ Large	○ Normal<br>○ Abnormal NCS<br>○ Abnormal CS
Ketone (mmol/L)	○ Negative<br>○ Trace<br>○ 1.5<br>○ 3.9<br>○ 7.8<br>○ >=15.6	○ Normal<br>○ Abnormal NCS<br>○ Abnormal CS
Specific Gravity	○ <=1.005<br>○ 1.010<br>○ 1.015<br>○ 1.020<br>○ 1.025<br>○ >=1.030	○ Normal<br>○ Abnormal NCS<br>○ Abnormal CS
Blood (Ery/uL)	○ Negative<br>○ Trace-lysed<br>○ Trace-intact<br>○ Ca 25<br>○ Ca 80<br>○ Ca 200	○ Normal<br>○ Abnormal NCS<br>○ Abnormal CS

pH	○ 5.0<br>○ 5.5<br>○ 6.0<br>○ 6.5<br>○ 7.0<br>○ 7.5<br>○ 8.0<br>○ 8.5<br>○ >=9.0	○ Normal<br>○ Abnormal NCS<br>○ Abnormal CS
Protein (g/L)	○ Negative<br>○ Trace<br>○ 0.15<br>○ 0.3<br>○ 1.0<br>○ 3.0<br>○ >=3.0	○ Normal<br>○ Abnormal NCS<br>○ Abnormal CS
Urobilinogen (mmol/L)	○ 3.2<br>○ 16<br>○ 33<br>○ 66<br>○ >+131	○ Normal<br>○ Abnormal NCS<br>○ Abnormal CS
Nitrite	○ Negative<br>○ Positive	○ Normal<br>○ Abnormal NCS<br>○ Abnormal CS

Leukocytes (Leu/uL)	○ Negative<br>○ Ca 15<br>○ Ca 70<br>○ Ca 125<br>○ Ca 500	○ Normal<br>○ Abnormal NCS<br>○ Abnormal CS
* Was the sample sent for microscopy	○ No<br>○ Yes	
* Were any microscopy parameters out of range?	○ No<br>○ Yes	

Urine Microscopy Assessment

[Revision: Initial revision of study]

(Visit ID = 120 / Visit Display Name = End of Study / Visit Abbrev = EOS / PageID = 140 (*) / Page Display Name = Urine Microscopy Assessment / Description = Microscopy Assessment)

* List all tests that were out of range	○ Leucocytes<br>○ Erythrocytes<br>○ Protein<br>○ glucose<br>○ Epithelial Cells<br>○ pH<br>○ Casts<br>○ Blood<br>○ Ketones<br>○ Leucocyte Esterase<br>○ Specific gravity<br>○ Bilirubin<br>○ Urobilinogen<br>○ Nitrites<br>○ Bacteria<br>○ Other
* If Other, Specify	
* Clinical Significance	○ Abnormal NCS<br>○ Abnormal CS

Serology

[Revision: Initial revision of study]

(Visit ID = 120 / Visit Display Name = End of Study / Visit Abbrev = EOS / PageID = 160 / Page Display Name = Serology / Description = Hep and HIV)

* Were serology tests done?	○ Yes<br>○ No	
* Date of blood collection		(DD-MMM-YYYY)
* Time of blood collection		(HH24:MI)
* HIV Ag/Ab	○ Negative<br>○ Positive	
* HBsAg	○ Negative<br>○ Positive	
* HCV	○ Negative<br>○ Positive	

Safety Serum Storage

[Revision: Initial revision of study]

(Visit ID = 120 / Visit Display Name = End of Study / Visit Abbrev = EOS / PageID = 90 / Page Display Name = Safety Serum Storage / Description = Safety Serum)

* Was the Safety Serum collected	○ No<br>○ Yes	
* Date of Serum Sample Collection		(DD-MMM-YYYY)
* Time of Serum Sample Collection		(HH24:MI)

PCR Collection

[Revision: Initial revision of study]

(Visit ID = 120 / Visit Display Name = End of Study / Visit Abbrev = EOS / PageID = 150 / Page Display Name = PCR Collection / Description =)

Not Done	☐	
* Date of PCR Collection		(DD-MMM-YYYY)
* Time of PCR Collection		(HH24:MI)

Phone Call

Phone Call

[Revision: Initial revision of study]

(Visit ID = 140 (*) / Visit Display Name = Phone Call / Visit Abbrev = Phone / PageID = 10 / Page Display Name = Phone Call / Description = follow up phone call)

* Was contact with the participant attempted?	○ No<br>○ Yes	
* If yes, Was contact made	○ No<br>○ Yes	
* Date of contact		(DD-MMM-YYYY)
* Time of Contact		(HH24:MI)

Common Forms

AE Summary

[Revision: Initial revision of study]

(Visit ID = 150 / Visit Display Name = Common Forms / Visit Abbrev = COMM / PageID = 10 / Page Display Name = AE Summary / Description = Adverse Event Header Question (OPTIONAL))

* Were any adverse events experienced?	○	No
	○	Yes

Adverse Events

[Revision: Initial revision of study]

(Visit ID = 150 / Visit Display Name = Common Forms / Visit Abbrev = COMM / PageID = 20 (*) / Page Display Name = Adverse Events / Description = Adverse Events)

* What is the adverse event term?		
* Start Date		(UNK-UNK-UNK)

* Start Time (hr)

- ○ 00
- ○ 01
- ○ 02
- ○ 03
- ○ 04
- ○ 05
- ○ 06
- ○ 07
- ○ 08
- ○ 09
- ○ 10
- ○ 11
- ○ 12
- ○ 13
- ○ 14
- ○ 15
- ○ 16
- ○ 17
- ○ 18
- ○ 19
- ○ 20
- ○ 21
- ○ 22
- ○ 23
- ○ UNK

* Start Time (min)	○	00
	○	01
	○	02
	○	03
	○	04
	○	05
	○	06
	○	07
	○	08
	○	09
	○	10
	○	11
	○	12
	○	13
	○	14
	○	15
	○	16
	○	17
	○	18
	○	19
	○	20
	○	21
	○	22
	○	23
	○	24
	○	25
	○	26
	○	27

- ○ 28
- ○ 29
- ○ 30
- ○ 31
- ○ 32
- ○ 33
- ○ 34
- ○ 35
- ○ 36
- ○ 37
- ○ 38
- ○ 39
- ○ 40
- ○ 41
- ○ 42
- ○ 43
- ○ 44
- ○ 45
- ○ 46
- ○ 47
- ○ 48
- ○ 49
- ○ 50
- ○ 51
- ○ 52
- ○ 53
- ○ 54
- ○ 55

	○ 56<br>○ 57<br>○ 58<br>○ 59<br>○ UNK
Is the adverse event ongoing?	☐
* End Date	(UNK-UNK-UNK)

* End Time (hr)

- ○ 00
- ○ 01
- ○ 02
- ○ 03
- ○ 04
- ○ 05
- ○ 06
- ○ 07
- ○ 08
- ○ 09
- ○ 10
- ○ 11
- ○ 12
- ○ 13
- ○ 14
- ○ 15
- ○ 16
- ○ 17
- ○ 18
- ○ 19
- ○ 20
- ○ 21
- ○ 22
- ○ 23
- ○ UNK

* End Time (min)	○	00
	○	01
	○	02
	○	03
	○	04
	○	05
	○	06
	○	07
	○	08
	○	09
	○	10
	○	11
	○	12
	○	13
	○	14
	○	15
	○	16
	○	17
	○	18
	○	19
	○	20
	○	21
	○	22
	○	23
	○	24
	○	25
	○	26
	○	27

- ○ 28
- ○ 29
- ○ 30
- ○ 31
- ○ 32
- ○ 33
- ○ 34
- ○ 35
- ○ 36
- ○ 37
- ○ 38
- ○ 39
- ○ 40
- ○ 41
- ○ 42
- ○ 43
- ○ 44
- ○ 45
- ○ 46
- ○ 47
- ○ 48
- ○ 49
- ○ 50
- ○ 51
- ○ 52
- ○ 53
- ○ 54
- ○ 55

	○	56
	○	57
	○	58
	○	59
	○	UNK
* What was the severity of the adverse event?	○	Grade 1: Mild
	○	Grade 2: Moderate
	○	Grade 3: Severe
	○	Grade 4: Life-threatening
	○	Grade 5: Death
* Is the adverse event serious?	○	No
	○	Yes
Is the adverse event associated with a congenital anomaly or birth defect?	☐	
Did the adverse event result in persistent or significant disability or incapacity?	☐	
Did the adverse event result in death?	☐	
Did the adverse event result in initial or prolonged hospitalization for the patient?	☐	
Is the adverse event life threatening?	☐	
Does the adverse event involve cancer?	☐	
Did the adverse event occur with an overdose?	☐	
Constitutes a possible Hy's Law case	☐	
Is the adverse event associated with other serious or important medical events?	☐	
* Relationship to study treatment (Artesunate)	○	Unrelated
	○	Unlikely
	○	Possible
	○	Probable

* Relationship to Inoculum?	○ Unrelated<br>○ Unlikely<br>○ Possible<br>○ Probable
Relationship to Piperaquine?	○ Unrelated<br>○ Unlikely<br>○ Possible<br>○ Probable
Relationship to Malarone?	○ Unrelated<br>○ Unlikely<br>○ Possible<br>○ Probable
Relationship to Primacin?	○ Unrelated<br>○ Unlikely<br>○ Possible<br>○ Probable
Relationship to Mosquito Feeding?	○ Unrelated<br>○ Unlikely<br>○ Possible<br>○ Probable
If unrelated to all of the above, what else is the AE related to?	

* What action was taken with study treatment (Artesunate)?	○ DOSE INCREASED<br>○ DOSE NOT CHANGED<br>○ DOSE REDUCED<br>○ DRUG INTERRUPTED<br>○ DRUG WITHDRAWN<br>○ NOT APPLICABLE<br>○ UNKNOWN
What other action was taken in response to this adverse event?	○ None<br>○ Medication<br>○ Other<br>○ Medication and Other
If Other, Specify	
List the medications	
* What was the outcome of this adverse event?	○ FATAL<br>○ NOT RECOVERED/NOT RESOLVED<br>○ RECOVERED/RESOLVED<br>○ RECOVERED/RESOLVED WITH SEQUELAE<br>○ RECOVERING/RESOLVING<br>○ UNKNOWN
* Did the adverse event cause the subject to be discontinued from the study?	○ No<br>○ Yes

Concomitant Medications Summary

[Revision: Initial revision of study]

(Visit ID = 150 / Visit Display Name = Common Forms / Visit Abbrev = COMM / PageID = 30 / Page Display Name = Concomitant Medications Summary / Description = Concomitant Medication Header)

* Is the participant taking any medication prior or during the study	○ No<br>○ Yes

Concomitant Medication

[Revision: Initial revision of study]

(Visit ID = 150 / Visit Display Name = Common Forms / Visit Abbrev = COMM / PageID = 40 (*) / Page Display Name = Concomitant Medication / Description = Concomitant Medications)

Record all medications taken during the study and those taken up to 28 days prior to inoculation.

* What was the term for the medication/therapy taken?		Medication Name; Full Trade or Proprietary Name recommended
* For what indication was the medication/therapy taken?		
* What was the dose of the medication/therapy?		

* What was the unit of the medication/therapy?

- ○ mg
- ○ mL
- ○ mol
- ○ V
- ○ m/sec
- ○ kg/m3
- ○ ug
- ○ g
- ○ CAPSULE
- ○ INHALATION
- ○ LOZENGE
- ○ umol
- ○ mmol
- ○ PATCH
- ○ SCOOPFUL
- ○ SPRAY
- ○ STRIP
- ○ SUPPOSITORY
- ○ Tbsp
- ○ TABLET
- ○ tsp
- ○ WAFER
- ○ mol/L
- ○ IU
- ○ mmol/L
- ○ PUFF
- ○ Other

If Other, specify

* What was the frequency of the medication/therapy?	○ UNKNOWN<br>○ CONTINUOUS<br>○ BID - Twice per day<br>○ PRN - As needed<br>○ QH - Every hour<br>○ TID - 3 times per day<br>○ QID - 4 times per day<br>○ ONCE - Once<br>○ INTERMITTENT<br>○ QD - Daily<br>○ Other
If Other, specify	
* What was the route of administration of the medication/therapy?	○ INTRADERMAL<br>○ INTRAMUSCULAR<br>○ INTRAVENOUS<br>○ NASAL<br>○ ORAL<br>○ RESPIRATORY (INHALATION)<br>○ SUBCUTANEOUS<br>○ SUBLINGUAL<br>○ TOPICAL<br>○ Other
If Other, specify	
* Start Date	(UNK-UNK-UNK)

* Start Time (hr)

- ○ 00
- ○ 01
- ○ 02
- ○ 03
- ○ 04
- ○ 05
- ○ 06
- ○ 07
- ○ 08
- ○ 09
- ○ 10
- ○ 11
- ○ 12
- ○ 13
- ○ 14
- ○ 15
- ○ 16
- ○ 17
- ○ 18
- ○ 19
- ○ 20
- ○ 21
- ○ 22
- ○ 23
- ○ UNK

* Start Time (min)	○	00
	○	01
	○	02
	○	03
	○	04
	○	05
	○	06
	○	07
	○	08
	○	09
	○	10
	○	11
	○	12
	○	13
	○	14
	○	15
	○	16
	○	17
	○	18
	○	19
	○	20
	○	21
	○	22
	○	23
	○	24
	○	25
	○	26
	○	27

- ○ 28
- ○ 29
- ○ 30
- ○ 31
- ○ 32
- ○ 33
- ○ 34
- ○ 35
- ○ 36
- ○ 37
- ○ 38
- ○ 39
- ○ 40
- ○ 41
- ○ 42
- ○ 43
- ○ 44
- ○ 45
- ○ 46
- ○ 47
- ○ 48
- ○ 49
- ○ 50
- ○ 51
- ○ 52
- ○ 53
- ○ 54
- ○ 55

	○ 56<br>○ 57<br>○ 58<br>○ 59<br>○ UNK
Is the medication/therapy still ongoing?	☐
* End Date	(UNK-UNK-UNK)

* End Time (hr)	○	00
	○	01
	○	02
	○	03
	○	04
	○	05
	○	06
	○	07
	○	08
	○	09
	○	10
	○	11
	○	12
	○	13
	○	14
	○	15
	○	16
	○	17
	○	18
	○	19
	○	20
	○	21
	○	22
	○	23
	○	UNK

* End Time (min)	○ 00<br>○ 01<br>○ 02<br>○ 03<br>○ 04<br>○ 05<br>○ 06<br>○ 07<br>○ 08<br>○ 09<br>○ 10<br>○ 11<br>○ 12<br>○ 13<br>○ 14<br>○ 15<br>○ 16<br>○ 17<br>○ 18<br>○ 19<br>○ 20<br>○ 21<br>○ 22<br>○ 23<br>○ 24<br>○ 25<br>○ 26<br>○ 27

- ○ 28
- ○ 29
- ○ 30
- ○ 31
- ○ 32
- ○ 33
- ○ 34
- ○ 35
- ○ 36
- ○ 37
- ○ 38
- ○ 39
- ○ 40
- ○ 41
- ○ 42
- ○ 43
- ○ 44
- ○ 45
- ○ 46
- ○ 47
- ○ 48
- ○ 49
- ○ 50
- ○ 51
- ○ 52
- ○ 53
- ○ 54
- ○ 55

- ○ 56
- ○ 57
- ○ 58
- ○ 59
- ○ UNK

Medical History Summary

[Revision: Initial revision of study]

(Visit ID = 150 / Visit Display Name = Common Forms / Visit Abbrev = COMM / PageID = 140 / Page Display Name = Medical History Summary / Description = Medical History Header)

* Has the subject experienced any past and/or concomitant diseases or past surgeries?	○ No<br>○ Yes

Medical History

[Revision: Initial revision of study]

(Visit ID = 150 / Visit Display Name = Common Forms / Visit Abbrev = COMM / PageID = 150 (*) / Page Display Name = Medical History / Description = Medical History)

* What is the verbatim term for the medical history condition/event?		
* What was the date the medical history event or condition started?		(UNK-UNK-UNK)
Is the medical history disease/condition or event still ongoing?	☐	
* What was the date the medical history event or condition ended?		(UNK-UNK-UNK)
* Please tick if any medications are given for the ongoing event.	☐	

Comments Summary

[Revision: Initial revision of study]

(Visit ID = 150 / Visit Display Name = Common Forms / Visit Abbrev = COMM / PageID = 50 / Page Display Name = Comments Summary / Description = Comments Summary)

* Any comments?	○	No
	○	Yes

Comments

[Revision: Initial revision of study]

(Visit ID = 150 / Visit Display Name = Common Forms / Visit Abbrev = COMM / PageID = 120 (*) / Page Display Name = Comments / Description = Comments)

* What Visit is the Comment associated with?	
* What Form is the Comment associated with?	
* Give a detailed description of the Comment	

Clinical Score

[Revision: Initial revision of study]

(Visit ID = 150 / Visit Display Name = Common Forms / Visit Abbrev = COMM / PageID = 60 (*) / Page Display Name = Clinical Score / Description = Clinical score with individual symptoms split out)

* Time Point

- ○ Day 0
- ○ Day 1
- ○ Day 2
- ○ Day 3
- ○ Day 4
- ○ Day 5
- ○ Day 6
- ○ Day 7
- ○ Day 8
- ○ Day 9
- ○ Day 10
- ○ Day 11
- ○ Day 12
- ○ Day 13
- ○ Day 14
- ○ Day 15
- ○ Day 16
- ○ Day 17
- ○ Day 18
- ○ Day 19
- ○ Day 20
- ○ Day 21
- ○ Day 22
- ○ Day 23
- ○ Day 24
- ○ Day 25
- ○ Day 26
- ○ Day 27

	○ Day 28<br>○ Day 29<br>○ Day 30<br>○ Day 31<br>○ Day 32<br>○ Day 33<br>○ Day 34<br>○ Day 35
* Date of Clinical Score	[ ] (DD-MMM-YYYY)
* Time of Clinical Score	[ ] (HH24:MI)
* Headache	○ Absent (0)<br>○ Mild (1)<br>○ Moderate (2)<br>○ Severe (3)
* Myalgia (Muscle Ache)	○ Absent (0)<br>○ Mild (1)<br>○ Moderate (2)<br>○ Severe (3)
* Arthralgia (Joint Ache)	○ Absent (0)<br>○ Mild (1)<br>○ Moderate (2)<br>○ Severe (3)
* Fatigue/Lethargy	○ Absent (0)<br>○ Mild (1)<br>○ Moderate (2)<br>○ Severe (3)

* Malaise/General Discomfort/Uneasiness	○	Absent (0)
	○	Mild (1)
	○	Moderate (2)
	○	Severe (3)
* Chills/Shivering/Rigors	○	Absent (0)
	○	Mild (1)
	○	Moderate (2)
	○	Severe (3)
* Sweating/Hot Spells	○	Absent (0)
	○	Mild (1)
	○	Moderate (2)
	○	Severe (3)
* Anorexia	○	Absent (0)
	○	Mild (1)
	○	Moderate (2)
	○	Severe (3)
* Nausea	○	Absent (0)
	○	Mild (1)
	○	Moderate (2)
	○	Severe (3)
* Vomiting	○	Absent (0)
	○	Mild (1)
	○	Moderate (2)
	○	Severe (3)
* Abdominal Discomfort	○	Absent (0)
	○	Mild (1)
	○	Moderate (2)
	○	Severe (3)

* Fever	○	Absent (0)
	○	Mild (1)
	○	Moderate (2)
	○	Severe (3)
* Tachycardia	○	Absent (0)
	○	Mild (1)
	○	Moderate (2)
	○	Severe (3)
* Hypotension	○	Absent (0)
	○	Mild (1)
	○	Moderate (2)
	○	Severe (3)
* Score		(format xxx)

Protocol Deviations Summary

[Revision: Initial revision of study]

(Visit ID = 150 / Visit Display Name = Common Forms / Visit Abbrev = COMM / PageID = 70 / Page Display Name = Protocol Deviations Summary / Description = Protocol Deviation Header)

* Were there any protocol deviations?	○ No<br>○ Yes

Protocol Deviations

[Revision: Initial revision of study]

(Visit ID = 150 / Visit Display Name = Common Forms / Visit Abbrev = COMM / PageID = 80 (*) / Page Display Name = Protocol Deviations / Description = Protocol Deviations)

* What was the protocol deviation type?	○ Protocol Deviation<br>○ Protocol Violation	
* What was the protocol deviation category	○ Eligibility<br>○ Study Schedule Compliance<br>○ Study Drug Compliance<br>○ Other	
* If Other, specify		
* What was the protocol deviation?		
* Date of deviation?		(UNK-UNK-UNK)

Rescue Medication

[Revision: Initial revision of study]

(Visit ID = 150 / Visit Display Name = Common Forms / Visit Abbrev = COMM / PageID = 160 (*) / Page Display Name = Rescue Medication / Description =)

* Treatment Administered	○ Primacin<br>○ Malarone<br>○ IV Artesunate
* Dose Amount	
* Dose Unit	○ Tablet<br>○ Capsule<br>○ mL<br>○ ug<br>○ mg
* Route of Administration	○ Oral<br>○ IV
* Date of Administration	(DD-MMM-YYYY)
* Time of Administration	(HH24:MI)
* Was drug administered as per protocol?	○ Yes<br>○ No

PCR Collection

[Revision: Initial revision of study]

(Visit ID = 150 / Visit Display Name = Common Forms / Visit Abbrev = COMM / PageID = 100 / Page Display Name = PCR Collection / Description = PCR sampling timepoints)

PCR Timepoint	Not Done	Date		Time	
Pre-dose	☐		(DD-MMM-YYYY)		(HH24:MI)
2 hour	☐		(DD-MMM-YYYY)		(HH24:MI)
4 hour	☐		(DD-MMM-YYYY)		(HH24:MI)
6 hour	☐		(DD-MMM-YYYY)		(HH24:MI)
8 hour	☐		(DD-MMM-YYYY)		(HH24:MI)
12 hour	☐		(DD-MMM-YYYY)		(HH24:MI)
16 hour	☐		(DD-MMM-YYYY)		(HH24:MI)
20 hour	☐		(DD-MMM-YYYY)		(HH24:MI)
24 hour	☐		(DD-MMM-YYYY)		(HH24:MI)
28 hour	☐		(DD-MMM-YYYY)		(HH24:MI)
32 hour	☐		(DD-MMM-YYYY)		(HH24:MI)
36 hour	☐		(DD-MMM-YYYY)		(HH24:MI)
48 hour	☐		(DD-MMM-YYYY)		(HH24:MI)
60 hour	☐		(DD-MMM-YYYY)		(HH24:MI)
72 hour	☐		(DD-MMM-YYYY)		(HH24:MI)

Completion Page

[Revision: Initial revision of study]

(Visit ID = 150 / Visit Display Name = Common Forms / Visit Abbrev = COMM / PageID = 110 / Page Display Name = Completion Page / Description = Completion Page)

* Date of final participation		(DD-MMM-YYYY)
* Status	○ Completed<br>○ Adverse event<br>○ Lost to follow up<br>○ Withdrawal by Subject<br>○ Study Terminated by Sponsor<br>○ Physician Decision<br>○ Protocol Violation<br>○ Death<br>○ Screen Failure<br>○ Other	
* If Other, specify		
* If Consent Withdrawn, specify reason (if given)		
* If Consent withdrawn, give date of withdrawal		(UNK-UNK-UNK)

Death

[Revision: Initial revision of study]

(Visit ID = 150 / Visit Display Name = Common Forms / Visit Abbrev = COMM / PageID = 130 / Page Display Name = Death / Description = Death Form)

* Date of Death		(DD-MMM-YYYY)
Primary cause of death (if known)		
* Was an autopsy performed?	○ Yes<br>○ No<br>○ Unknown	

Unscheduled

Visit Date

[Revision: Initial revision of study]

(Visit ID = 130 (*) / Visit Display Name = Unscheduled / Visit Abbrev = UNS / PageID = 170 / Page Display Name = Visit Date / Description =)

Visit Not Done?	☐	
* Visit Date		(DD-MMM-YYYY)

Unscheduled Visit

[Revision: Initial revision of study]

(Visit ID = 130 (*) / Visit Display Name = Unscheduled / Visit Abbrev = UNS / PageID = 10 / Page Display Name = Unscheduled Visit / Description = Unscheduled Visit/Assessment)

Please tick ALL unscheduled assessments that were done at this visit. No need to add a new visit for each one. The CRFs will generate when you tick the check box.

* Reason for Unscheduled Visit / Assessment	
Temperature	☐
Vital Signs (BP, RR, HR, Temp)	☐
Vitals Signs (supine to standing)	☐
ECG (single)	☐
Physical Exam (standard)	☐
Pregnancy	☐
Labs	☐
Drug Test	☐
Feeding	☐
Urinalysis	☐
PK sampling	☐
PCR Collection	☐
Piperaquine Dosing	☐
Genetic Testing	☐

Temperature

[Revision: Initial revision of study]

(Visit ID = 130 (*) / Visit Display Name = Unscheduled / Visit Abbrev = UNS / PageID = 260 (*) / Page Display Name = Temperature / Description =)

* Date		(DD-MMM-YYYY)
* Time		(HH24:MI)
* Temperature Method	○ Tympanic<br>○ Sublingual	
* Temperature (C)		(format xx.x)
* Assessment	○ Normal<br>○ Abnormal NCS<br>○ Abnormal CS	

Vitals Signs

[Revision: Initial revision of study]

(Visit ID = 130 (*) / Visit Display Name = Unscheduled / Visit Abbrev = UNS / PageID = 150 (*) / Page Display Name = Vitals Signs / Description = Vital Signs single assessment)

* Were Vital Signs assessed?	○ Yes<br>○ No	
* Date performed		(DD-MMM-YYYY)
* At what time were the measurements performed?		(HH24:MI)
* Systolic Blood Pressure (mmHg)		(format xxx)
* Assessment	○ Normal<br>○ Abnormal NCS<br>○ Abnormal CS	
* Diastolic Blood Pressure (mmHg)		(format xxx)
* Assessment	○ Normal<br>○ Abnormal NCS<br>○ Abnormal CS	
* Heart Rate (bpm)		(format xxx)
* Assessment	○ Normal<br>○ Abnormal NCS<br>○ Abnormal CS	
* Respiratory Rate (breaths per minute)		(format xxx)
* Assessment	○ Normal<br>○ Abnormal NCS<br>○ Abnormal CS	

* Temperature Method	○ Tympanic<br>○ Sublingual	
* Temperature (C)	[ ]	(format xx.x)
* Assessment	○ Normal<br>○ Abnormal NCS<br>○ Abnormal CS	

Vitals Signs (Supine to Standing)

[Revision: Initial revision of study]

(Visit ID = 130 (*) / Visit Display Name = Unscheduled / Visit Abbrev = UNS / PageID = 30 / Page Display Name = Vitals Signs (Supine to Standing) / Description = Vital Signs at screening)

* Were Vital Signs assessed?	○ Yes<br>○ No	
* Date performed		(DD-MMM-YYYY)
* Temperature Method	○ Tympanic<br>○ Sublingual	
* Temperature (C)		(format xx.x)
* Assessment	○ Normal<br>○ Abnormal NCS<br>○ Abnormal CS	
* Respiratory Rate (breaths per minute)		(format xxx)
* Assessment	○ Normal<br>○ Abnormal NCS<br>○ Abnormal CS	
* Time of Supine Vital Sign Measurements		(HH24:MI)
* Supine Systolic Blood Pressure (mmHg)		(format xxx)
* Assessment	○ Normal<br>○ Abnormal NCS<br>○ Abnormal CS	
* Supine Diastolic Blood Pressure (mmHg)		(format xxx)

* Assessment	○ Normal<br>○ Abnormal NCS<br>○ Abnormal CS	
* Supine Heart Rate (bpm)		(format xxx)
* Assessment	○ Normal<br>○ Abnormal NCS<br>○ Abnormal CS	
* Time of Standing Vital Sign Measurements		(HH24:MI)
* Standing Systolic Blood Pressure (mmHg)		(format xxx)
* Assessment	○ Normal<br>○ Abnormal NCS<br>○ Abnormal CS	
* Standing Diastolic Blood Pressure (mmHg)		(format xxx)
* Assessment	○ Normal<br>○ Abnormal NCS<br>○ Abnormal CS	
* Standing Heart Rate (bpm)		(format xxx)
* Assessment	○ Normal<br>○ Abnormal NCS<br>○ Abnormal CS	

Physical Exam

[Revision: Initial revision of study]

(Visit ID = 130 (*) / Visit Display Name = Unscheduled / Visit Abbrev = UNS / PageID = 40 / Page Display Name = Physical Exam / Description = Physical Exam)

* Was Physical Exam Done	○ Yes<br>○ No	
* Date of Physical Exam	[ ]	(DD-MMM-YYYY)
* Time of Physical Exam	[ ]	(HH24:MI)
AM/PM	○ AM<br>○ PM	

Body System	Assessment	Description	Clinical Significance
Head/Neck/Ears/Eyes/Nose/Throat	○ Normal ○ Abnormal ○ Not Done		○ Not Clinically Significant ○ Clinically Significant
Heart/circulation	○ Normal ○ Abnormal ○ Not Done		○ Not Clinically Significant ○ Clinically Significant
Chest	○ Normal ○ Abnormal ○ Not Done		○ Not Clinically Significant ○ Clinically Significant
Lungs	○ Normal ○ Abnormal ○ Not Done		○ Not Clinically Significant ○ Clinically Significant
Abdomen	○ Normal ○ Abnormal ○ Not Done		○ Not Clinically Significant ○ Clinically Significant
Skin	○ Normal ○ Abnormal ○ Not Done		○ Not Clinically Significant ○ Clinically Significant
Neurological	○ Normal ○ Abnormal ○ Not Done		○ Not Clinically Significant ○ Clinically Significant

Extremities	○ Normal<br>○ Abnormal<br>○ Not Done		○ Not Clinically Significant<br>○ Clinically Significant
Back	○ Normal<br>○ Abnormal<br>○ Not Done		○ Not Clinically Significant<br>○ Clinically Significant
Dentition	○ Normal<br>○ Abnormal<br>○ Not Done		○ Not Clinically Significant<br>○ Clinically Significant

Other Body Systems

* Were any other abnormalities noted?	○ No<br>○ Yes

Abb. Physical Exam

[Revision: Initial revision of study]

(Visit ID = 130 (*) / Visit Display Name = Unscheduled / Visit Abbrev = UNS / PageID = 240 / Page Display Name = Abb. Physical Exam / Description =)

* Was Abbreviated Physical Exam Done	○ Yes<br>○ No	
* Date of Physical Exam		(DD-MMM-YYYY)
* Time of Physical Exam		(HH24:MI)
AM/PM	○ AM<br>○ PM	

Body System	Assessment	Description	Clinical Significance
General Appearance	○ Normal<br>○ Abnormal<br>○ Not Done		○ Not Clinically Significant<br>○ Clinically Significant
Heart/circulation	○ Normal<br>○ Abnormal<br>○ Not Done		○ Not Clinically Significant<br>○ Clinically Significant
Chest	○ Normal<br>○ Abnormal<br>○ Not Done		○ Not Clinically Significant<br>○ Clinically Significant
Lungs	○ Normal<br>○ Abnormal<br>○ Not Done		○ Not Clinically Significant<br>○ Clinically Significant
Abdomen	○ Normal<br>○ Abnormal<br>○ Not Done		○ Not Clinically Significant<br>○ Clinically Significant
Skin	○ Normal<br>○ Abnormal<br>○ Not Done		○ Not Clinically Significant<br>○ Clinically Significant
Brief Neurological	○ Normal<br>○ Abnormal<br>○ Not Done		○ Not Clinically Significant<br>○ Clinically Significant

Other Body Systems

* Were any other abnormalities noted? ○ No ○ Yes

Physical Exam (extra)

[Revision: Initial revision of study]

(Visit ID = 130 (*) / Visit Display Name = Unscheduled / Visit Abbrev = UNS / PageID = 50 (*) / Page Display Name = Physical Exam (extra) / Description = a page to capture other or extra body systems)

* Body System	○ Head/Ears/Eyes/Nose/Throat<br>○ Heart<br>○ Chest<br>○ Lungs<br>○ Abdomen<br>○ Skin<br>○ Neurological exam<br>○ Extremities<br>○ Back<br>○ Dentition<br>○ General Appearance<br>○ Other
* Other Body System	
* Assessment	○ Normal<br>○ Abnormal<br>○ Not Done
* Description of abnormality	
* Clinical Significance	○ Not Clinically Significant<br>○ Clinically Significant

Pregnancy Test

[Revision: Initial revision of study]

(Visit ID = 130 (*) / Visit Display Name = Unscheduled / Visit Abbrev = UNS / PageID = 60 / Page Display Name = Pregnancy Test / Description = Pregnancy test)

* Was Pregnancy Test performed?	○ Yes<br>○ No	
* Serum or Urine	○ Serum<br>○ Urine	
* Date of pregnancy test	[ ]	(DD-MMM-YYYY)
* Time of pregnancy test	[ ]	(HH24:MI)
* Result	○ Negative<br>○ Positive	

Lab Sample Collection

[Revision: Initial revision of study]

(Visit ID = 130 (*) / Visit Display Name = Unscheduled / Visit Abbrev = UNS / PageID = 70 (*) / Page Display Name = Lab Sample Collection / Description =)

* Sample date		(DD-MMM-YYYY)
* Sample time		(HH24:MI)
* Panel test	○ Biochemistry<br>○ Haematology<br>○ CMV<br>○ EBV<br>○ G6PD<br>○ LFT<br>○ Red Cell Alloantibodies<br>○ Serology<br>○ Duffy<br>○ Rh Status<br>○ Immunology<br>○ Thyroid Tests<br>○ Coagulation<br>○ Endocrinology<br>○ Blood Bank<br>○ Microbiology<br>○ Other	
* If Other, specify		

Lab Test Result

[Revision: Initial revision of study]

(Visit ID = 130 (*) / Visit Display Name = Unscheduled / Visit Abbrev = UNS / PageID = 180 / Page Display Name = Lab Test Result / Description = Were any lab tests flagged as being out of range)

* Were any of the lab tests flagged as being out of range ?	○ No<br>○ Yes

Lab Test Assessment

[Revision: Initial revision of study]

(Visit ID = 130 (*) / Visit Display Name = Unscheduled / Visit Abbrev = UNS / PageID = 190 (*) / Page Display Name = Lab Test Assessment / Description = Assessment of all out of range labs)

* Choose the category of the tests that were out of range	○ Biochemistry<br>○ Haematology<br>○ CMV<br>○ EBV<br>○ G6PD<br>○ LFT<br>○ Red Cell Alloantibodies<br>○ Serology<br>○ Duffy<br>○ Rh Status<br>○ Immunology<br>○ Thyroid Tests<br>○ Coagulation<br>○ Endocrinology<br>○ Blood Bank<br>○ Microbiology<br>○ Other
* If Other, specify	

* List all tests that were out of range	
	○ White Blood Cells count<br>○ Neutrophils<br>○ Lymphocytes<br>○ Monocytes<br>○ Eosinophils<br>○ Basophils<br>○ Red blood cell count<br>○ Haemoglobin<br>○ Haematocrit<br>○ Platelet Count<br>○ Reticulocyte Count<br>○ Red Cell Allo-Antibodies<br>○ Duffy Blood Group<br>○ Sodium<br>○ Potassium<br>○ Chloride<br>○ Bicarbonate<br>○ Glucose<br>○ Urea<br>○ Creatinine<br>○ Urate<br>○ Albumin<br>○ Globulin<br>○ Total Protein<br>○ Direct Bilirubin<br>○ Alkaline Phosphatase<br>○ Alanine aminotransferase<br>○ Aspartate aminotransferase

- ○ Calcium Corrected
- ○ Phosphate
- ○ Lactate dehyrogenase
- ○ Magnesium
- ○ Cholesterol
- ○ Triglycerides
- ○ HDL
- ○ LDL
- ○ Cholesterol/HDL
- ○ Total Bilirubin
- ○ eGFR
- ○ Pregnancy Test
- ○ FSH
- ○ HIV
- ○ HBsAg
- ○ anti-HBc
- ○ anti-HCV
- ○ anti-HAV
- ○ anti-HEV
- ○ EBV NA IgG
- ○ EBV VCA IgM
- ○ CMV IgG
- ○ CMV IgM
- ○ Other

* If Other, Specify	
* Clinical Significance	○ Abnormal NCS<br>○ Abnormal CS

If a lab test has been assessed as being Clinically Significant please add as an Adverse Event,

Alcohol Breath Test

[Revision: Initial revision of study]

(Visit ID = 130 (*) / Visit Display Name = Unscheduled / Visit Abbrev = UNS / PageID = 250 / Page Display Name = Alcohol Breath Test / Description = Alcohol Breath Test)

* Was Alcohol Breath Test done	○ Yes<br>○ No	
* Date of Breath Test		(DD-MMM-YYYY)
* Time of Breath Test		(HH24:MI)
* Result	○ Negative<br>○ Positive	

Urine Drug Screen

[Revision: Initial revision of study]

(Visit ID = 130 (*) / Visit Display Name = Unscheduled / Visit Abbrev = UNS / PageID = 80 / Page Display Name = Urine Drug Screen / Description = Drug Screen)

* Was Urine Drug Screen Performed?	○ Yes<br>○ No	
* Date of Drug Screen		(DD-MMM-YYYY)
* Time of Drug Screen		(HH24:MI)

Drug	Result
Amphetamines	○ Negative<br>○ Positive
Methamphetamines	○ Negative<br>○ Positive
Barbiturates	○ Negative<br>○ Positive
Benzodiazepines	○ Negative<br>○ Positive
Cocaine	○ Negative<br>○ Positive
Methadone	○ Negative<br>○ Positive
Opiates	○ Negative<br>○ Positive
Phencyclidine	○ Negative<br>○ Positive
Cannabis	○ Negative<br>○ Positive
Tricyclic antidepressants	○ Negative<br>○ Positive
Acetaminophen (paracetamol)	○ Negative<br>○ Positive

Urinalysis

[Revision: Initial revision of study]

(Visit ID = 130 (*) / Visit Display Name = Unscheduled / Visit Abbrev = UNS / PageID = 110 / Page Display Name = Urinalysis / Description = Urinanalysis)

* Was Urinalysis performed?	◯ Yes<br>◯ No	
* Date of Urinalysis		(DD-MMM-YYYY)
* Time of Urinalysis		(HH24:MI)

Test	Result	Assessment
Glucose (mmol/L)	○ Negative<br>○ 5.5<br>○ 14<br>○ 28<br>○ >=55	○ Normal<br>○ Abnormal NCS<br>○ Abnormal CS
Bilirubin	○ Negative<br>○ Small<br>○ Moderate<br>○ Large	○ Normal<br>○ Abnormal NCS<br>○ Abnormal CS
Ketone (mmol/L)	○ Negative<br>○ Trace<br>○ 1.5<br>○ 3.9<br>○ 7.8<br>○ >=15.6	○ Normal<br>○ Abnormal NCS<br>○ Abnormal CS
Specific Gravity	○ <=1.005<br>○ 1.010<br>○ 1.015<br>○ 1.020<br>○ 1.025<br>○ >=1.030	○ Normal<br>○ Abnormal NCS<br>○ Abnormal CS
Blood (Ery/uL)	○ Negative<br>○ Trace-lysed<br>○ Trace-intact<br>○ Ca 25<br>○ Ca 80<br>○ Ca 200	○ Normal<br>○ Abnormal NCS<br>○ Abnormal CS

pH	○ 5.0<br>○ 5.5<br>○ 6.0<br>○ 6.5<br>○ 7.0<br>○ 7.5<br>○ 8.0<br>○ 8.5<br>○ >=9.0	○ Normal<br>○ Abnormal NCS<br>○ Abnormal CS
Protein (g/L)	○ Negative<br>○ Trace<br>○ 0.15<br>○ 0.3<br>○ 1.0<br>○ 3.0<br>○ >=3.0	○ Normal<br>○ Abnormal NCS<br>○ Abnormal CS
Urobilinogen (mmol/L)	○ 3.2<br>○ 16<br>○ 33<br>○ 66<br>○ >+131	○ Normal<br>○ Abnormal NCS<br>○ Abnormal CS
Nitrite	○ Negative<br>○ Positive	○ Normal<br>○ Abnormal NCS<br>○ Abnormal CS

Leukocytes (Leu/uL)	○ Negative<br>○ Ca 15<br>○ Ca 70<br>○ Ca 125<br>○ Ca 500	○ Normal<br>○ Abnormal NCS<br>○ Abnormal CS
* Was the sample sent for microscopy	○ No<br>○ Yes	
* Were any microscopy parameters out of range?	○ No<br>○ Yes	

Urine Microscopy Assessment

[Revision: Initial revision of study]

(Visit ID = 130 (*) / Visit Display Name = Unscheduled / Visit Abbrev = UNS / PageID = 220 (*) / Page Display Name = Urine Microscopy Assessment / Description = Microscopy Assessment)

* List all tests that were out of range	○ Leucocytes<br>○ Erythrocytes<br>○ Protein<br>○ glucose<br>○ Epithelial Cells<br>○ pH<br>○ Casts<br>○ Blood<br>○ Ketones<br>○ Leucocyte Esterase<br>○ Specific gravity<br>○ Bilirubin<br>○ Urobilinogen<br>○ Nitrites<br>○ Bacteria<br>○ Other
* If Other, Specify	
* Clinical Significance	○ Abnormal NCS<br>○ Abnormal CS

PCR Collection

[Revision: Initial revision of study]

(Visit ID = 130 (*) / Visit Display Name = Unscheduled / Visit Abbrev = UNS / PageID = 130 / Page Display Name = PCR Collection / Description =)

Not Done	☐	
* Date of PCR Collection		(DD-MMM-YYYY)
* Time of PCR Collection		(HH24:MI)

Feeding

[Revision: Initial revision of study]

(Visit ID = 130 (*) / Visit Display Name = Unscheduled / Visit Abbrev = UNS / PageID = 200 (*) / Page Display Name = Feeding / Description = Feeding (direct / indirect))

* Type of Feeding	○ DFA<br>○ MFA	
* Date of collection	[ ]	(DD-MMM-YYYY)
* Site of feeding	○ Right Forearm<br>○ Left Forearm<br>○ Right Thigh<br>○ Left Thigh<br>○ Right Calf<br>○ Left Calf<br>○ Other	
* If Other, Specify	[ ]	
* Start Time of collection	[ ]	(HH24:MI)
* End time of collection	[ ]	(HH24:MI)

PK

[Revision: Initial revision of study]

(Visit ID = 130 (*) / Visit Display Name = Unscheduled / Visit Abbrev = UNS / PageID = 140 / Page Display Name = PK / Description =)

* Date of PK Collection		(DD-MMM-YYYY)
* Time of PK Collection		(HH24:MI)

ECG

[Revision: Initial revision of study]

(Visit ID = 130 (*) / Visit Display Name = Unscheduled / Visit Abbrev = UNS / PageID = 160 (*) / Page Display Name = ECG / Description = ECG Single)

* Was ECG performed	○ Yes<br>○ No	
* Date of ECG		(DD-MMM-YYYY)
* Time of ECG		(HH24:MI)
* Heart Rate (bpm)		(format xxx)
* PR Interval (msecs)		(format xxx.x)
* RR Interval (msecs)		(format xxxx.x)
* QRS Interval (msecs)		(format xxx.x)
* QT Interval (msecs)		(format xxx.xx)
* QTcF Interval (msecs)		(format xxxxx)
* ECG Interpretation	○ Normal<br>○ Abnormal NCS<br>○ Abnormal CS	
* If Abnormal, describe the abnormality		

Piperaquine Dosing

[Revision: Initial revision of study]

(Visit ID = 130 (*) / Visit Display Name = Unscheduled / Visit Abbrev = UNS / PageID = 230 / Page Display Name = Piperaquine Dosing / Description =)

* Date of administration		(DD-MMM-YYYY)
* Time of administration		(HH24:MI)
* Lot Number		
* Total Dose (mg)		
* Route of administration	○ Oral	

Genetic Testing

[Revision: Initial revision of study]

(Visit ID = 130 (*) / Visit Display Name = Unscheduled / Visit Abbrev = UNS / PageID = 270 / Page Display Name = Genetic Testing / Description =)

* Date of Informed Consent for Genetic Testing		(DD-MMM-YYYY)
* Time of Informed Consent for Genetic Testing		(HH24:MI)
* Informed Consent Version		
* Date of Sample Collection		(DD-MMM-YYYY)
* Time of Sample Collection		(HH24:MI)

Reconsent

Reconsent

[Revision: Initial revision of study]

(Visit ID = 160 (*) / Visit Display Name = Reconsent / Visit Abbrev = RECONSENT / PageID = 10 / Page Display Name = Reconsent / Description = reconsent form)

* Date of Informed Consent		(DD-MMM-YYYY)
* Time of Informed Consent		(HH24:MI)
* Informed Consent Version		

Early Termination Visit

Visit Date

[Revision: Initial revision of study]

(Visit ID = 190 / Visit Display Name = Early Termination Visit / Visit Abbrev = ETV / PageID = 100 / Page Display Name = Visit Date / Description =)

Visit Not Done?	☐	
* Visit Date		(DD-MMM-YYYY)

Physical Exam

[Revision: Initial revision of study]

(Visit ID = 190 / Visit Display Name = Early Termination Visit / Visit Abbrev = ETV / PageID = 10 / Page Display Name = Physical Exam / Description = Physical Exam)

* Was Physical Exam Done	○ Yes<br>○ No	
* Date of Physical Exam		(DD-MMM-YYYY)
* Time of Physical Exam		(HH24:MI)
AM/PM	○ AM<br>○ PM	

Body System	Assessment	Description	Clinical Significance
Head/Neck/Ears/Eyes/Nose/Throat	○ Normal ○ Abnormal ○ Not Done		○ Not Clinically Significant ○ Clinically Significant
Heart/circulation	○ Normal ○ Abnormal ○ Not Done		○ Not Clinically Significant ○ Clinically Significant
Chest	○ Normal ○ Abnormal ○ Not Done		○ Not Clinically Significant ○ Clinically Significant
Lungs	○ Normal ○ Abnormal ○ Not Done		○ Not Clinically Significant ○ Clinically Significant
Abdomen	○ Normal ○ Abnormal ○ Not Done		○ Not Clinically Significant ○ Clinically Significant
Skin	○ Normal ○ Abnormal ○ Not Done		○ Not Clinically Significant ○ Clinically Significant
Neurological	○ Normal ○ Abnormal ○ Not Done		○ Not Clinically Significant ○ Clinically Significant

Extremities	○ Normal<br>○ Abnormal<br>○ Not Done		○ Not Clinically Significant<br>○ Clinically Significant
Back	○ Normal<br>○ Abnormal<br>○ Not Done		○ Not Clinically Significant<br>○ Clinically Significant
Dentition	○ Normal<br>○ Abnormal<br>○ Not Done		○ Not Clinically Significant<br>○ Clinically Significant

Other Body Systems

* Were any other abnormalities noted?	○ No<br>○ Yes

Physical Exam (extra)

[Revision: Initial revision of study]

(Visit ID = 190 / Visit Display Name = Early Termination Visit / Visit Abbrev = ETV / PageID = 20 (*) / Page Display Name = Physical Exam (extra) / Description = a page to capture other or extra body systems)

* Body System	○ Head/Ears/Eyes/Nose/Throat<br>○ Heart<br>○ Chest<br>○ Lungs<br>○ Abdomen<br>○ Skin<br>○ Neurological exam<br>○ Extremities<br>○ Back<br>○ Dentition<br>○ General Appearance<br>○ Other
* Other Body System	
* Assessment	○ Normal<br>○ Abnormal<br>○ Not Done
* Description of abnormality	
* Clinical Significance	○ Not Clinically Significant<br>○ Clinically Significant

ECG (single)

[Revision: Initial revision of study]

(Visit ID = 190 / Visit Display Name = Early Termination Visit / Visit Abbrev = ETV / PageID = 30 / Page Display Name = ECG (single) / Description = ECG Single)

* Was ECG performed	○ Yes<br>○ No	
* Date of ECG		(DD-MMM-YYYY)
* Time of ECG		(HH24:MI)
* Heart Rate (bpm)		(format xxx)
* PR Interval (msecs)		(format xxx.x)
* RR Interval (msecs)		(format xxxx.x)
* QRS Interval (msecs)		(format xxx.x)
* QT Interval (msecs)		(format xxx.xx)
* QTcF Interval (msecs)		(format xxxxx)
* ECG Interpretation	○ Normal<br>○ Abnormal NCS<br>○ Abnormal CS	
* If Abnormal, describe the abnormality		

Vital Signs (single)

[Revision: Initial revision of study]

(Visit ID = 190 / Visit Display Name = Early Termination Visit / Visit Abbrev = ETV / PageID = 40 / Page Display Name = Vital Signs (single) / Description = Vital Signs single assessment)

* Were Vital Signs assessed?	○ Yes<br>○ No	
* Date performed	[ ]	(DD-MMM-YYYY)
* At what time were the measurements performed?	[ ]	(HH24:MI)
* Systolic Blood Pressure (mmHg)	[ ]	(format xxx)
* Assessment	○ Normal<br>○ Abnormal NCS<br>○ Abnormal CS	
* Diastolic Blood Pressure (mmHg)	[ ]	(format xxx)
* Assessment	○ Normal<br>○ Abnormal NCS<br>○ Abnormal CS	
* Heart Rate (bpm)	[ ]	(format xxx)
* Assessment	○ Normal<br>○ Abnormal NCS<br>○ Abnormal CS	
* Respiratory Rate (breaths per minute)	[ ]	(format xxx)
* Assessment	○ Normal<br>○ Abnormal NCS<br>○ Abnormal CS	

* Temperature Method	○ Tympanic<br>○ Sublingual	
* Temperature (C)	[ ]	(format xx.x)
* Assessment	○ Normal<br>○ Abnormal NCS<br>○ Abnormal CS	

Lab Sample Collection Summary

[Revision: Initial revision of study]

(Visit ID = 190 / Visit Display Name = Early Termination Visit / Visit Abbrev = ETV / PageID = 50 / Page Display Name = Lab Sample Collection Summary / Description = Lab collection header)

* Were any Labs collected ?	○ No<br>○ Yes	
* Sample date		(DD-MMM-YYYY)
* Sample time		(HH24:MI)

Lab Sample Collection

[Revision: Initial revision of study]

(Visit ID = 190 / Visit Display Name = Early Termination Visit / Visit Abbrev = ETV / PageID = 60 (*) / Page Display Name = Lab Sample Collection / Description = Labs collected)

* Panel test	○ Biochemistry<br>○ Haematology<br>○ CMV<br>○ EBV<br>○ G6PD<br>○ LFT<br>○ Red Cell Alloantibodies<br>○ Serology<br>○ Duffy<br>○ Rh Status<br>○ Immunology<br>○ Thyroid Tests<br>○ Coagulation<br>○ Endocrinology<br>○ Blood Bank<br>○ Microbiology<br>○ Other
* If Other, specify	

Lab Test Result

[Revision: Initial revision of study]

(Visit ID = 190 / Visit Display Name = Early Termination Visit / Visit Abbrev = ETV / PageID = 110 / Page Display Name = Lab Test Result / Description = Were any lab tests flagged as being out of range)

* Were any of the lab tests flagged as being out of range ?	○ No<br>○ Yes

Lab Test Assessment

[Revision: Initial revision of study]

(Visit ID = 190 / Visit Display Name = Early Termination Visit / Visit Abbrev = ETV / PageID = 120 (*) / Page Display Name = Lab Test Assessment / Description = Assessment of all out of range labs)

* Choose the category of the tests that were out of range	○ Biochemistry<br>○ Haematology<br>○ CMV<br>○ EBV<br>○ G6PD<br>○ LFT<br>○ Red Cell Alloantibodies<br>○ Serology<br>○ Duffy<br>○ Rh Status<br>○ Immunology<br>○ Thyroid Tests<br>○ Coagulation<br>○ Endocrinology<br>○ Blood Bank<br>○ Microbiology<br>○ Other
* If Other, specify	

* List all tests that were out of range

- ○ White Blood Cells count
- ○ Neutrophils
- ○ Lymphocytes
- ○ Monocytes
- ○ Eosinophils
- ○ Basophils
- ○ Red blood cell count
- ○ Haemoglobin
- ○ Haematocrit
- ○ Platelet Count
- ○ Reticulocyte Count
- ○ Red Cell Allo-Antibodies
- ○ Duffy Blood Group
- ○ Sodium
- ○ Potassium
- ○ Chloride
- ○ Bicarbonate
- ○ Glucose
- ○ Urea
- ○ Creatinine
- ○ Urate
- ○ Albumin
- ○ Globulin
- ○ Total Protein
- ○ Direct Bilirubin
- ○ Alkaline Phosphatase
- ○ Alanine aminotransferase
- ○ Aspartate aminotransferase

- ○ Calcium Corrected
- ○ Phosphate
- ○ Lactate dehyrogenase
- ○ Magnesium
- ○ Cholesterol
- ○ Triglycerides
- ○ HDL
- ○ LDL
- ○ Cholesterol/HDL
- ○ Total Bilirubin
- ○ eGFR
- ○ Pregnancy Test
- ○ FSH
- ○ HIV
- ○ HBsAg
- ○ anti-HBc
- ○ anti-HCV
- ○ anti-HAV
- ○ anti-HEV
- ○ EBV NA IgG
- ○ EBV VCA IgM
- ○ CMV IgG
- ○ CMV IgM
- ○ Other

* If Other, Specify	
* Clinical Significance	○ Abnormal NCS<br>○ Abnormal CS

If a lab test has been assessed as being Clinically Significant please add as an Adverse Event,

Serology

[Revision: Initial revision of study]

(Visit ID = 190 / Visit Display Name = Early Termination Visit / Visit Abbrev = ETV / PageID = 160 / Page Display Name = Serology / Description = Hep and HIV)

* Were serology tests done?	○ Yes<br>○ No	
* Date of blood collection	[ ]	(DD-MMM-YYYY)
* Time of blood collection	[ ]	(HH24:MI)
* HIV Ag/Ab	○ Negative<br>○ Positive	
* HBsAg	○ Negative<br>○ Positive	
* HCV	○ Negative<br>○ Positive	

Pregnancy Test

[Revision: Initial revision of study]

(Visit ID = 190 / Visit Display Name = Early Termination Visit / Visit Abbrev = ETV / PageID = 70 / Page Display Name = Pregnancy Test / Description = Pregnancy test)

* Was Pregnancy Test performed?	○ Yes<br>○ No	
* Serum or Urine	○ Serum<br>○ Urine	
* Date of pregnancy test		(DD-MMM-YYYY)
* Time of pregnancy test		(HH24:MI)
* Result	○ Negative<br>○ Positive	

Urinalysis

[Revision: Initial revision of study]

(Visit ID = 190 / Visit Display Name = Early Termination Visit / Visit Abbrev = ETV / PageID = 80 / Page Display Name = Urinalysis / Description = Urinanalysis)

* Was Urinalysis performed?	○ Yes<br>○ No	
* Date of Urinalysis		(DD-MMM-YYYY)
* Time of Urinalysis		(HH24:MI)

Test	Result	Assessment
Glucose (mmol/L)	○ Negative<br>○ 5.5<br>○ 14<br>○ 28<br>○ >=55	○ Normal<br>○ Abnormal NCS<br>○ Abnormal CS
Bilirubin	○ Negative<br>○ Small<br>○ Moderate<br>○ Large	○ Normal<br>○ Abnormal NCS<br>○ Abnormal CS
Ketone (mmol/L)	○ Negative<br>○ Trace<br>○ 1.5<br>○ 3.9<br>○ 7.8<br>○ >=15.6	○ Normal<br>○ Abnormal NCS<br>○ Abnormal CS
Specific Gravity	○ <=1.005<br>○ 1.010<br>○ 1.015<br>○ 1.020<br>○ 1.025<br>○ >=1.030	○ Normal<br>○ Abnormal NCS<br>○ Abnormal CS
Blood (Ery/uL)	○ Negative<br>○ Trace-lysed<br>○ Trace-intact<br>○ Ca 25<br>○ Ca 80<br>○ Ca 200	○ Normal<br>○ Abnormal NCS<br>○ Abnormal CS

pH	○ 5.0<br>○ 5.5<br>○ 6.0<br>○ 6.5<br>○ 7.0<br>○ 7.5<br>○ 8.0<br>○ 8.5<br>○ >=9.0	○ Normal<br>○ Abnormal NCS<br>○ Abnormal CS
Protein (g/L)	○ Negative<br>○ Trace<br>○ 0.15<br>○ 0.3<br>○ 1.0<br>○ 3.0<br>○ >=3.0	○ Normal<br>○ Abnormal NCS<br>○ Abnormal CS
Urobilinogen (mmol/L)	○ 3.2<br>○ 16<br>○ 33<br>○ 66<br>○ >+131	○ Normal<br>○ Abnormal NCS<br>○ Abnormal CS
Nitrite	○ Negative<br>○ Positive	○ Normal<br>○ Abnormal NCS<br>○ Abnormal CS

Leukocytes (Leu/uL)	○ Negative<br>○ Ca 15<br>○ Ca 70<br>○ Ca 125<br>○ Ca 500	○ Normal<br>○ Abnormal NCS<br>○ Abnormal CS
* Was the sample sent for microscopy	○ No<br>○ Yes	
* Were any microscopy parameters out of range?	○ No<br>○ Yes	

Urine Microscopy Assessment

[Revision: Initial revision of study]

(Visit ID = 190 / Visit Display Name = Early Termination Visit / Visit Abbrev = ETV / PageID = 140 (*) / Page Display Name = Urine Microscopy Assessment / Description = Microscopy Assessment)

* List all tests that were out of range	○ Leucocytes<br>○ Erythrocytes<br>○ Protein<br>○ glucose<br>○ Epithelial Cells<br>○ pH<br>○ Casts<br>○ Blood<br>○ Ketones<br>○ Leucocyte Esterase<br>○ Specific gravity<br>○ Bilirubin<br>○ Urobilinogen<br>○ Nitrites<br>○ Bacteria<br>○ Other
* If Other, Specify	
* Clinical Significance	○ Abnormal NCS<br>○ Abnormal CS

Safety Serum Storage

[Revision: Initial revision of study]

(Visit ID = 190 / Visit Display Name = Early Termination Visit / Visit Abbrev = ETV / PageID = 90 / Page Display Name = Safety Serum Storage / Description = Safety Serum)

* Was the Safety Serum collected	○ No<br>○ Yes	
* Date of Serum Sample Collection		(DD-MMM-YYYY)
* Time of Serum Sample Collection		(HH24:MI)

PCR Collection

[Revision: Initial revision of study]

(Visit ID = 190 / Visit Display Name = Early Termination Visit / Visit Abbrev = ETV / PageID = 150 / Page Display Name = PCR Collection / Description =)

Not Done	☐	
* Date of PCR Collection		(DD-MMM-YYYY)
* Time of PCR Collection		(HH24:MI)

16.1.3 List of Human Research Ethics Committees

QIMR Berghofer Medical Research Committee Human Research Ethics Committee

Chairperson: Dr Ian Wilkey

Phone: 61 7 3362 0117

Fax: 61 7 3362 0109

email: hrec.secretariat@qimrberghofer.edu.au

Australian Red Cross Blood Service Human Research Ethics Committee (now known as Australian Red Cross Lifeblood Human Research Ethics Committee)

Chairperson: Professor Lesley Barclay

Phone: 61 2 9234 2368

email: ethics@redcrossblood.org.au

16.1.4 List and description of investigators and other important participants in the study

16.1.4 List and description of investigators and other important participants in the study

**Sponsor**<br>Research Office, QIMR Berghofer Medical Research Institute<br>300C Herston Road, Herston Qld 4006, Australia	**Services**<br>Study management, statistics, pharmacometrics, medical writing, human-to-mosquito transmission studies<br>**Personnel**<br>Research Office representative: Alicia Rooney<br>Project manager: Dr Rebecca Watts<br>Statisticians: Dr Louise Marquart, Lachlan Webb<br>Pharmacometrician: Dr Azrin Rahman<br>Medical writer: Jacinda Wilson
**Funding organisation**<br>Medicines for Malaria Venture (MMV)<br>20, Route de Pré-Bois 1215 Geneva 15 Switzerland	**Personnel**<br>Head of Experimental Medicine and Clinical Pharmacology : Dr Stephan Chalon, MD<br>Project Director: Dr Jörg Möhrle
**Study Centre/Clinical Unit**<br>Q-Pharm Pty Limited<br>Level 5 Clive Berghofer Cancer Research Centre, 300C Herston Road, Herston Qld 4006, Australia	**Services**<br>Study conduct<br>**Personnel**<br>Project managers: Brenda Cerqueira, Paul Morgan
**Contract Research Organisation**<br>Clinical Network Services (CNS) Pty Ltd<br>Level 2, 381 MacArthur Avenue, Hamilton Qld 4007, Australia	**Services**<br>Data management, monitoring<br>**Personnel**<br>Project Manager: Leanne West

**Laboratories**	**Services**
Analytical Chemistry Unit, Department of Chemical Pathology, Pathology Queensland<br><br>Level 3, Block 7, Royal Brisbane and Women's Hospital, Brisbane Qld 4029, Australia	Measurement of artesunate and dihydroartemisinin<br><br>**Personnel**<br><br>Scientist: Brett McWhinney
Sullivan Nicolaides Pathology Central Laboratory<br><br>24 Hurworth Street, Bowen Hills Qld 4006, Australia	**Services**<br><br>Clinical safety laboratory<br><br>**Personnel**<br><br>Clinical Trials Manager: Marie-Claire Keogh
Queensland Paediatric Infectious Diseases (QPID) Laboratory<br><br>SASVRC, Level 8, Centre for Children's Health Research, Lady Cilento Children's Hospital Precinct (now Queensland Children's Hospital), 62 Graham Street, South Brisbane Qld 4101, Australia	**Services**<br><br>Analytical study centre for parasite quantification<br><br>**Personnel**<br><br>Scientists: Jane Gaydon, Clare Wang

16.1.5 Signature of Principal Investigator

SIGNATURE PAGE

I have read this report and confirm that to the best of my knowledge it accurately describes the conduct and results of the study.

PRINCIPAL INVESTIGATOR

Professor James McCarthy, MBBS MD FRACP

QIMR Berghofer Medical Research Institute

300C Herston Road

Herston Qld 4006 AUSTRALIA

	Date	Signature

SIGNATURE PAGE

I have read this report and confirm that to the best of my knowledge it accurately describes the conduct and results of the study.

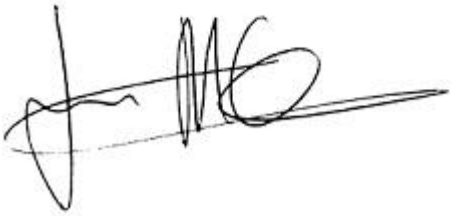

PRINCIPAL INVESTIGATOR

Professor James McCarthy, MBBS MD FRACP

QIMR Berghofer Medical Research Institute

300C Herston Road

Herston Qld 4006 AUSTRALIA

Date 27 March 2020

16.1.6 List of investigational product(s) batch numbers

P. falciparum 3D7 malaria challenge agent

MCI-061, expiry 15 November 2017 11:33 am (Cohort 1)

MCI-063, expiry 20 February 2018 11:09 am (Cohort 2)

MCI-073, expiry 05 September 2018 12:25 pm (Cohort 3)

P. falciparum K13 MBE-018 malaria challenge agent

MCI-060, expiry 14 November 2017 11:35 am (Cohort 1)

MCI-062, expiry 19 February 2018 10:43 am (Cohort 2)

MCI-072, expiry 04 September 2018 11:03 am (Cohort 3)

16.1.7 Randomisation scheme and codes

.

Randomisation Schedule

Protocol number: QP17C11
QIMR reference: P2339
Title: A comparative study of the parasitological response to single dose artesunate therapy among subjects experimentally infected with either the artemisinin-resistant *Plasmodium falciparum* isolate K13 or the artemisinin-sensitive isolate 3D7
Cohort: 1
Date of report: 30/11/2017
Coordinating Principal Investigator:

Professor James McCarthy — QIMR Berghofer Medical Research Institute, RBWH, University of Queensland

Created by: Mandy Way

For all enquiries contact:
Mandy Way
Biostatistician,
Statistics Unit,
QIMR Berghofer Medical Research Institute,
300 Herston Road, Herston QLD 4006
Mandy.Way@qimrberghofer.edu.au

Reviewed by: Professor Peter O'Rourke PhD (Senior Biostatistician)

Prepared for: Q-Pharm Pharmacy

File Names:
Word document: L:\Lab_Stats_Unit\2015_and_Onwards\Projects\HMMU\Project Management\Malaria Challange Studies_ Current Projects\QP17C11\ QP17C11_randomisation_cohort_1
STATA code: L:\Lab_Stats_Unit\2015_and_Onwards\Projects\HMMU\Project Management\Malaria Challange Studies_ Current Projects\QP17C11\Rebecca_Watts_randomisation_procedure.do

Study design:

This study uses the induced blood stage malaria model to compare the response of the *Plasmodium falciparum* isolates Cam3.II^{R539T} (referred to as K13, artemisinin-resistant) and 3D7 (artemisinin-sensitive) to a single dose of the antimalarial drug artesunate in healthy subjects. The study is to be conducted in up to 9 subjects per cohort, with up to 3 cohorts. Within each cohort, subjects are to be randomised in a 2:1 ratio to receive either the K13 or 3D7 challenge inoculum.

Methods:

Randomisation was performed in STATA 13, using the coding located at:
L:\Lab_Stats_Unit\2015_and_Onwards\Projects\HMMU\Project Management\Malaria Challange Studies_ Current Projects\QP17C11\ Rebecca_Watts_randomisation_procedure.do

If 9 or more subjects were available, 6 subjects were to be randomly assigned to K13, and 3 subjects randomly assigned to 3D7. Any remaining subjects were to be an ordered sequence of reserves. If only 8 subjects were available, 5 subjects were to be assigned to K13 and the remaining 3 subjects to

3D7. If 7 subjects were available, 5 subjects were to be assigned to K13 and 2 subjects were to be assigned to 3D7. If 6 subjects were available, 4 subjects were to be assigned to K13 and 2 subjects were to be assigned to 3D7. A random list of Sequence Numbers was generated.

```
STATA instructions
*selecting the number of rows you want, this number will vary
depending on the trial*
set obs 9 *if 8 participants seed  used was 47, if 7 participants
seed used was 59*
*setting a seed will enable reproducibility, change for different
trials, you don't want the same ordering*
set seed 33
*generating ids*
gen id=_n
*assigning random numbers to each id*
gen rand_num=uniform()
*ordering the random numbers*
egen ordering = rank(rand_num)
*producing a list*
list id ordering
```

Randomisation Cohort 1:

The randomisation sequence for 8 subjects was generated as shown in Table 1 (Sequence Number).

Table 1. Random Sequence Number for Cohort 1

Sequence Number
8
2
3
7
5
1
4
6

Eight subjects were eligible for the study at the time of randomisation (Day -1), and were listed in ascending order according to their screening number (S number). The screening numbers were matched with the Sequence Numbers as listed in Table 2. The Sequence Number became the R number for that subject e.g. S003 with Sequence Number 8 became R108. R101 to R105 were to receive the *Plasmodium falciparum* K13 inoculum and R106 to R108 were to receive the *P. falciparum* 3D7 inoculum. Post randomisation 2 subjects withdrew: R101 and R102. This resulted in 3 subjects receiving the K13 inoculum (R103, R104, R105) and 3 subjects receiving the 3D7 inoculum (R106, R107, R108).

Table 2. Allocation of R Numbers to Cohort 1

S Number	Sequence Number	R Number	Inoculum
S003	8	R108	3D7
S005	2	R102*	K13
S006	3	R103	K13
S007	7	R107	3D7
S011	5	R105	K13
S012	1	R101*	K13
S013	4	R104	K13
S014	6	R106	3D7

*Subjects withdrew post randomisation and before inoculation

Randomisation Schedule

Protocol number: QP17C11
QIMR reference: P2339
Title: A comparative study of the parasitological response to single dose artesunate therapy among subjects experimentally infected with either the artemisinin-resistant *Plasmodium falciparum* isolate K13 or the artemisinin-sensitive isolate 3D7
Cohort: 2
Date of report: 20/02/2018
Coordinating Principal Investigator:

Professor James McCarthy — QIMR Berghofer Medical Research Institute
RBWH, University of Queensland

Created by: Stacey Llewellyn

For all enquiries contact:
Stacey Llewellyn
Biostatistician,
Statistics Unit,
QIMR Berghofer Medical Research Institute,
300 Herston Road, Herston QLD 4006
Stacey.llewellyn@qimrberghofer.edu.au

Reviewed by: Professor Peter O'Rourke PhD (Senior Biostatistician)

Prepared for: Q-Pharm Pharmacy

File Names:
Word document: L:\Lab_Stats_Unit\2015_and_Onwards\Projects\HMMU\Project Management\Malaria Challange Studies_ Current Projects\QP17C11\ QP17C11_randomisation_cohort_2_SL20Feb18
STATA code: L:\Lab_Stats_Unit\2015_and_Onwards\Projects\HMMU\Project Management\Malaria Challange Studies_ Current Projects\QP17C11\Rebecca_Watts_randomisation_procedure_Cohort2.do

Study design:

This study uses the induced blood stage malaria model to compare the response of the *Plasmodium falciparum* isolates Cam3.II^{R539T} (referred to as K13, artemisinin-resistant) and 3D7 (artemisinin-sensitive) to a single dose of the antimalarial drug artesunate in healthy subjects. The study is to be conducted in up to 27 subjects, with up to 3 cohorts. Subjects are to be randomised to receive either the K13 or 3D7 challenge inoculum, to achieve a ratio of 2:1 ratio over the whole study.

Methods:

Randomisation was performed in STATA 13, using the coding located at:
L:\Lab_Stats_Unit\2015_and_Onwards\Projects\HMMU\Project Management\Malaria Challange Studies_ Current Projects\QP17C11\ Rebecca_Watts_randomisation_procedure_Cohort2.do

If 12 or more subjects were available, 9 subjects were to be randomly assigned to K13, and 3 subjects randomly assigned to 3D7. Any remaining subjects were to be an ordered sequence of

reserves. For less than 12 subjects the participants were assigned to K13 or 3D7 as per Table 1. A random list of Sequence Numbers was generated for the total number of participants.

Table 1. Cohort 2 Participant Allocation Breakdown

Total no. participants	K13 participants	3D7 participants
12	9	3
11	8	3
10	7	3
9	6	3
8	5	3
7	4	3
6	3	3

```
STATA instructions
*selecting the number of rows you want, this number will vary
depending on the trial*
set obs 10 *if 8 participants seed  used was 47, if 7 participants
seed used was 59*
*setting a seed will enable reproducibility, change for different
trials, you don't want the same ordering*
set seed 213
*generating ids*
gen id=_n
*assigning random numbers to each id*
gen rand_num=uniform()
*ordering the random numbers*
egen ordering = rank(rand_num)
*producing a list*
list id ordering
```

Randomisation Cohort 2:

The randomisation sequence for 10 subjects was generated as shown in Table 2 (Sequence Number).

Table 2. Random Sequence Number for Cohort 2

Sequence Number
5
8
4
6
9
1
10
3
2
7

Ten subjects were eligible for the study at the time of randomisation (Day -2), and were listed in ascending order according to their screening number (S number). The screening numbers were matched with the Sequence Numbers as listed in Table 3. The Sequence Number became the R number for that subject e.g. S016 with Sequence Number 5 became R205. The first seven R numbers (R201 to R207) were to receive the *P. falciparum* K13 inoculum and the last three R numbers (R208 to R210) were to receive the *P. falciparum* 3D7 inoculum.

Table 3. Allocation of R Numbers to Cohort 2

S Number	Initials	Sequence Number	R Number	Inoculum
S016	MJL	5	R205	K13
S018	BMM	8	R208	3D7
S020	CLR	4	R204	K13
S022	C-D	6	R206	K13
S025	DMS	9	R209	3D7
S026	S-F	1	R201	K13
S027	LCW	10	R210	3D7
S028	EAW	3	R203	K13
S029	BSW	2	R202	K13
S030	GEP	7	R207	K13

16.1.8 Audit certificates

16.1.9 Documentation of statistical methods

STATISTICAL ANALYSIS PLAN

DATE OF PLAN:

FINAL Version 1.0, 18 December, 2018

BASED ON:

Protocol Final Version 3.0, dated

23 July 2018

STUDY DRUG:

artesunate and piperaquine phosphate

PROTOCOL NUMBER:

QP17C11/P2339

STUDY TITLE:

A comparative study of the parasitological response to single dose artesunate therapy among subjects experimentally infected with either the artemisinin-resistant Plasmodium falciparum isolate K13 or the artemisinin-sensitive isolate 3D7

SPONSOR:

QIMR Berghofer Medical Research Institute

300C Herston Road, Herston,

QLD 4006,

Australia

This study is being conducted in compliance with good clinical practice, including the archiving of essential documents.

SIGNATURE PAGE

This document has been prepared and/or reviewed by:

Bilyana Dabin<br>Project Statistician<br>Clinical Network Services Pty Ltd	Signature	Date
Leanne West<br>Project Manager<br>Clinical Network Services Pty Ltd	Signature	Date

This document has been reviewed and accepted by:

Prof. James McCarthy<br>Principal Investigator<br>QIMR Berghofer Medical Research Institute	Signature	Date

SIGNATURE PAGE

This document has been prepared and/or reviewed by:

Bilyana Dabin
Project Statistician
Clinical Network Services Pty Ltd

Signature | Date

Leanne West
Project Manager
Clinical Network Services Pty Ltd

Signature | Date

This document has been reviewed and accepted by:

17/12/18

Prof. James McCarthy
Principal Investigator
QIMR Berghofer Medical Research Institute

Signature | Date

TECHNICAL SUMMARY REPORT (TSR)

**Name of Sponsor/Company:**<br>QIMR Berghofer Medical Research Institute	**Individual Study Table Referring to Part of the Dossier:** N/A<br>**Volume:** N/A	***(For National Authority Use Only)*****:**
**Name of Finished Product:**<br>artesunate<br>piperaquine	**Page:** N/A	
**Name of Active Ingredient**:<br>artesunate<br>piperaquine phosphate		
**Title Of Study:**<br>A comparative study of the parasitological response to single dose artesunate therapy among subjects experimentally infected with either the artemisinin-resistant *Plasmodium falciparum* isolate K13 or the artemisinin-sensitive isolate 3D7		
**Investigators**:<br>Professor James McCarthy<br>**Study Center(s):**<br>Q-Pharm Pty Ltd<br>Levels 3 and 5, 300C Herston Rd<br>Herston QLD 4006, Australia		
**Study period:**<br>Study participation from Screening to Follow-Up is approximately:<br>11 months	**Phase of development:** 1/1b	

Objectives:

Primary:

- To compare the parasite clearance profiles of the *P. falciparum* K13 and 3D7 isolates after administration of artesunate.
- To determine the safety and tolerability associated with infection of healthy subjects with the *P. falciparum* K13 and 3D7 isolates.

Exploratory:

- To characterise the pharmacokinetic profile of artesunate and its active metabolite, dihydroartemisinin, in *P. falciparum* K13 and 3D7 infected subjects.
- To characterise the pharmacokinetic/pharmacodynamic relationship between artesunate and dihydroartemisinin pharmacokinetics and parasite clearance in *P. falciparum* K13 and 3D7 infected subjects.
- To determine if gametocytes appear in the blood of subjects infected with *P. falciparum* K13 or 3D7.
- To determine if K13 and 3D7 gametocytes, if present, are infective to mosquitoes (may be performed in selected cohorts at the Investigator's discretion).
- To investigate and identify any possible associations between subjects genetic polymorphisms and liver function (or phenotype) following infection with *P. falciparum* K13 or 3D7 (optional and requires consent via genetic testing Consent Form).
- To characterise the antimalarial treatment effects on *P. falciparum* K13 and 3D7 parasites using phenotypic and transcriptomic approaches (Cohorts 2 and 3).
- To investigate the *in vivo* presence of dormant parasites (Cohorts 2 and 3).

Methodology:

This study compared the response of the *Plasmodium falciparum* isolates Cam3.II^{R539T} (referred to as K13, artemisinin-resistant) and 3D7 (artemisinin-sensitive) to a single dose of the antimalarial drug artesunate in healthy subjects using the induced blood stage malaria (IBSM) model. The study planned to include up to 27 subjects, in up to 3 cohorts. Up to 18 subjects were to receive the K13 challenge agent and up to 9 subjects were to receive the 3D7 challenge agent, to achieve a ratio of 2:1 over the whole study. However, over the whole study 22 subjects were inoculated with a challenge agent: Cohort 1 (K13, n=3; 3D7 n=3), Cohort 2 (K13 n=7; 3D7 n=3), and Cohort 3 (K13 n=3, 3D7 n=3).

A review of the safety, tolerability and parasitaemia data from the preceding cohort was conducted by the Safety Review Team prior to malaria parasite inoculation of the next cohort. Data from at least 3 weeks post-inoculation was required for each review.

Subjects randomised to receive the K13 challenge agent were inoculated on Day 0 with approximately 2 800 viable *P. falciparum* K13 parasite-infected red blood cells (RBCs). Subjects randomised to receive the 3D7 challenge agent were inoculated on Day 1 with approximately 2 800 viable *P. falciparum* 3D7 parasite-infected RBCs. On an outpatient basis, subjects were monitored daily via phone and then attended the clinical unit daily from 4 days postinoculation – Day 4 for K13 subjects and Day 5 for 3D7 subjects – until positive for presence of malaria parasites by quantitative polymerase chain reaction (qPCR) targeting the 18S rRNA gene (referred to as malaria 18S qPCR). Once qPCR positive, subjects were monitored twice daily until artesunate treatment for parasitaemia, symptoms or signs of malaria, and adverse events. The threshold for commencement of artesunate treatment was when all subjects reached a parasitaemia of ≥5 000 parasites/mL. If the parasitaemia of any subject was ≥5 000 parasites/mL, and was accompanied by a malaria clinical score >6 before all subjects reached the treatment threshold, then treatment of that subject began within a 24 hour period. Subjects could receive artesunate treatment at any time based on parasitaemia and/or clinical symptoms at the Investigator’s discretion. Subjects were admitted to the clinical unit (approximately Day 9), administered a single oral dose of approximately 2 mg/kg artesunate, and confined for 72 hours to monitor for safety and tolerability of therapy and to ensure adequate clinical and parasitological response. The plasma concentration-time profiles of artesunate and its active metabolite dihydroartemisinin were also assessed.

Once clinically well, subjects were followed up on an out-patient basis for monitoring of safety and parasite clearance. Subjects might also have been evaluated for the presence of sexual parasite stages (gametocytes) and other parasite lifecycle stages in the blood, by parasite lifecycle stage reverse transcription qPCR (qRT-PCR) targeting genes such as the female gametocyte-specific transcript *pfs25*, the male gametocyte-specific transcript *pfMGET*, and/or the ring-stage transcript *pfSBP-1*. Infectivity of parasites to *Anopheles* mosquitoes might have been investigated by direct skin feeding and/or membrane feeding assays, at the Investigator’s discretion using parasite lifecycle stage qRT-PCR to determine probability of infectivity.

It was anticipated that artesunate treatment would reduce K13 parasitaemia, although at a slower rate than for the 3D7 artemisinin-sensitive isolate. If artesunate did not clear parasitaemia, subjects were administered a drug known to be active against both K13 and 3D7 isolates (piperaquine phosphate as a single dose of 960 mg).

Cohort 1:

For Cohort 1, piperaquine phosphate treatment was to occur on an individual subject basis in the event of unsatisfactory parasite clearance, defined as 2 consecutive malaria 18S qPCR time-points showing a decrease in parasitaemia of less than 20% of baseline (i.e., the parasitaemia before drug treatment) by 72 hours post-artesunate treatment. Subjects were also treated with piperaquine phosphate if recrudescence occurred after artesunate treatment, defined as ≥5 000 blood stage parasites/mL and a 2-fold increase within 48 hours, or a malaria clinical score >6. piperaquine phosphate treatment could also be undertaken at the Investigator's discretion.

All Cohort 1 subjects received compulsory rescue treatment with Malarone® (atovaquone/proguanil hydrochloride; 3 day course) on Day 26±3 or earlier at the Investigator's discretion. Subjects might also have been treated with a single oral dose of 45 mg primaquine (Primacin™) at the time of Malarone® treatment, if gametocytaemia was suspected from parasite lifecycle stage qRT-PCR or by the presence of stable low level parasitaemia, or at the Investigator's discretion, to ensure complete clearance of gametocytes.

Cohorts 2 and 3:

For Cohorts 2 and 3, piperaquine phosphate treatment occurred for subjects inoculated with the 3D7 challenge agent as outlined above for Cohort 1. However, K13 subjects in Cohorts 2 and 3 were treated with piperaquine phosphate on Day 11 AM or earlier at the Investigator's discretion. This was to avoid the high parasitaemias seen in K13 subjects in Cohort 1 on Day 11 PM prior to piperaquine phosphate treatment.

All Cohort 2 and 3 subjects were treated with a single oral dose of 45 mg primaquine (Primacin™) on Day 23±3 or earlier at the Investigator's discretion. This was to ensure complete clearance of gametocytes before Day 28±3 (End of Study). All subjects received compulsory rescue treatment with Malarone® (atovaquone/proguanil hydrochloride; 3 day course) on Day 25±3 or earlier at the Investigator's discretion.

All cohorts:

Follow-up for safety assessments was performed on Day 28±3 (End of Study). The Safety Review Team reviewed the safety and tolerability data from the study after completion of the last cohort.

Number of Subjects (planned and analyzed):

Planned:

9 subjects per cohort, with up to 3 cohorts

Study Total number of Subjects = 27

Analyzed:

25 subjects (22 randomized and inoculated; 3 randomized but withdrew before inoculation)

Diagnosis and main criteria for inclusion:

Inclusion Criteria:

Demography

1. Adult (male and non-pregnant, non-lactating female) subjects between 18 and 55 years of age inclusive, who do not live alone (from inoculation day until at least the end of the Malarone® treatment) and will be contactable and available for the duration of the trial and contactable up to 2 weeks following the End of Study visit (approximately 6 weeks).

2. Body weight minimum 50 kg, body mass index between 18 and 32 kg/m2, inclusive.

Health status

3. Certified as healthy by a comprehensive clinical assessment (detailed medical history and complete physical examination).

4. Vital signs after 5 minutes resting in supine position:

   90 mmHg ≤ systolic blood pressure (SBP) ≤ 140 mmHg,

   50 mmHg ≤ diastolic blood pressure (DBP) ≤ 90 mmHg,

   40 bpm ≤ heart rate (HR) ≤ 100 bpm.

5. Normal standard 12-lead ECG after 5 minutes resting in supine position, QTcF≤450 msec with absence of second or third degree atrioventricular block or abnormal T wave morphology at screening and at pre-inoculation on inoculation day.

6. Laboratory parameters within the normal range, unless the Investigator considers an abnormality to be clinically irrelevant for healthy subjects enrolled in this clinical investigation in accordance with approved clinically acceptable laboratory ranges documented prior to study start. More specifically, serum creatinine, hepatic transaminase enzymes (aspartate aminotransferase, alanine aminotransferase), and total bilirubin (unless the subject has documented Gilbert syndrome) should not exceed the approved acceptable ranges and haemoglobin must be equal or higher than the lower limit of the normal range.

7. Heterosexual women of childbearing potential should be surgically sterile or using an insertable, injectable, transdermal or combination oral contraceptive approved by the TGA combined with a barrier contraceptive for the duration of the study, and have negative results on a serum pregnancy test done before inoculation. Abstinent, heterosexual female subjects must agree to start a double method if they start a sexual relationship during the study. Adequate contraception does not apply to subjects of childbearing potential with same sex partners (abstinence from penile-vaginal intercourse), when this is their preferred and usual lifestyle. Female subjects with same sex partners must not be planning in vitro fertilisation within the required contraception period.

Women of non-childbearing potential who will not require contraception during the study are defined as: post-menopausal (spontaneous amenorrhoea for ≥ 12 months, or spontaneous amenorrhoea for 6-12 months and follicle-stimulating hormone (FSH) ≥ 40 IU/mL; either should be together with the absence of oral contraceptive use for > 12 months).

Regulations

8. Having given written informed consent prior to undertaking any study-related procedure.

Exclusion Criteria:

Subjects fulfilling any of the following criteria will not be eligible for inclusion in this study:

Medical history and clinical status

1. Any history of malaria or participation in a previous malaria challenge study.
2. Must not have travelled to or lived (>2 weeks) in a malaria-endemic region during the past 12 months or planned travel to a malaria-endemic region during the course of the study (for endemic regions see https://map.ox.ac.uk/country-profiles/#!/). Bali is not considered a malaria-endemic region.
3. Participation in any investigational product study within the 12 weeks preceding the study (i.e. there should be 12 weeks between the last dose of a previous investigational product and malaria parasite inoculation, at the Investigator's discretion).
4. Has evidence of increased cardiovascular disease risk (defined as >10%, 5 year risk for those greater than 35 years of age, as determined by the Australian Absolute Cardiovascular Disease Risk Calculator (http://www.cvdcheck.org.au/)). Risk factors include sex, age, systolic blood pressure (mm/Hg), smoking status, total and HDL cholesterol (mmol/L), and reported diabetes status.
5. Symptomatic postural hypotension at screening, irrespective of the decrease in blood pressure, or asymptomatic postural hypotension defined as a decrease in systolic blood pressure ≥20 mmHg within 2-3 minutes when changing from supine to standing position.
6. History of splenectomy.
7. Presence or history of drug hypersensitivity, or allergic disease diagnosed by an allergist/immunologist and/or treated by a physician for allergy or history of a severe allergic reaction, anaphylaxis or convulsions following any vaccination or infusion.
8. Presence of current or suspected serious chronic diseases such as cardiac or autoimmune disease (HIV or other immuno-deficiencies), insulin-dependent and non-insulin dependent diabetes (excluding glucose intolerance if exclusion criterion 4 is met), progressive neurological disease, severe malnutrition, acute or progressive hepatic disease, acute or progressive renal disease, porphyria, psoriasis, rheumatoid arthritis, asthma, epilepsy, or obsessive compulsive disorder.
9. History of malignancy of any organ system (other than localised basal cell carcinoma of the skin or in situ cervical cancer), treated or untreated, within 5 years of screening, regardless of whether there is evidence of local recurrence or metastases.

10. Subjects with history of schizophrenia, bi-polar disease, or other severe (disabling) chronic psychiatric diagnosis including depression or receiving psychiatric drugs or who has been hospitalised within the past 5 years prior to enrolment for psychiatric illness, history of suicide attempt, or confinement for danger to self or others.
11. History of serious psychiatric condition that may affect participation in the study or preclude compliance with the protocol, including but not limited to past or present psychoses, disorders requiring lithium, a history of attempted or planned suicide, more than one previous episode of major depression, any previous single episode of major depression lasting for or requiring treatment for more than 6 months, or any episode of major depression during the 5 years preceding screening.

    The Beck Depression Inventory will be used as an objective tool for the assessment of depression at screening. In addition to the conditions listed above, subjects with a score of 20 or more on the Beck Depression Inventory and/or a response of 1, 2 or 3 for item 9 of this inventory (related to suicidal ideation) will not be eligible for participation. These subjects will be referred to a general practitioner or medical specialist as appropriate. Subjects with a Beck score of 17 to 19 may be enrolled at the discretion of the Investigator if they do not have a history of the psychiatric conditions mentioned in this criterion and their mental state is not considered to pose additional risk to the health of the subject or to the execution of the study and interpretation of the data gathered.
12. Frequent headaches and/or migraines, recurrent nausea, and/or vomiting (more than twice a month).
13. Presence of acute infectious disease or fever (e.g. sublingual temperature ≥38.5°C) within the 5 days prior to inoculation with malaria parasites.
14. Evidence of acute illness within the 4 weeks prior to screening that the Investigator deems may compromise subject safety.
15. Significant inter-current disease of any type, in particular liver, renal, cardiac, pulmonary, neurologic, rheumatologic, or autoimmune disease by history, physical examination, and/or laboratory studies including urinalysis.
16. Subject has a clinically significant disease or any condition or disease that might affect drug absorption, distribution or excretion (e.g. gastrectomy, diarrhoea).
17. Blood donation of any volume within 1 month before inclusion, or participation in any research study involving blood sampling (more than 450 mL/unit of blood), or blood donation to Australian Red Cross Blood Service (Blood Service) or other blood bank during the 8 weeks prior to the treatment drug dose in the study.
18. Subject unwilling to defer blood donations to the Blood Service for at least 6 months.
19. Medical requirement for intravenous immunoglobulin or blood transfusions.
20. Subject who has ever received a blood transfusion.
21. History or presence of alcohol abuse (alcohol consumption more than 40 g per day) or drug habituation, or any prior intravenous usage of an illicit substance.
22. Tobacco use of more than 5 cigarettes or equivalent per day, and unable to stop smoking for the duration of the clinical unit confinement.

Interfering substances

23. Any vaccination within the last 28 days.
24. Any corticosteroids, anti-inflammatory drugs, immunomodulators or anticoagulants. Any subject currently receiving or having previously received immunosuppressive therapy (including systemic steroids, adrenocorticotrophic hormone or inhaled steroids) at a dose or duration associated with hypothalamic-pituitary-adrenal axis suppression (e.g. 1 mg/kg/day prednisone, chronic use of inhaled high potency corticosteroids such as budesonide 800 µg/day or fluticasone 750 µg, or equivalent).
25. Any recent (<6 weeks) or current systemic therapy with an antibiotic or drug with potential antimalarial activity (e.g. chloroquine, piperaquine phosphate, benzodiazepine, flunarizine, fluoxetine, tetracycline, azithromycin, clindamycin, doxycycline etc.).
26. Ingestion of any poppy seeds within the 24 hours prior to the screening blood test (subjects will be advised by phone not to consume any poppy seeds in this time period).
27. Excessive consumption of beverages or food containing xanthine bases including Red Bull, chocolate, coffee etc. (more than 400 mg caffeine per day, equivalent to more than 4 cups of coffee per day).
28. Unwillingness to abstain from consumption of grapefruit or Seville oranges from inoculation day until end of Malarone® treatment.
29. Unwillingness to abstain from consumption of quinine containing foods/beverages such as tonic water and lemon bitter, from inoculation day until end of Malarone® treatment.
30. Use of prescription drugs or non-prescription drugs or herbal supplements (such as St John's Wort), within 14 days or 5 half-lives (whichever is longer) prior to the malaria challenge agent inoculation. As an exception, ibuprofen (preferred) may be used at doses of up to 1.2 g/day, or paracetamol at doses of up to 4 g/day after discussion with the Investigator. Limited use of other non-prescription medications or dietary supplements, not believed to affect subject safety or the overall results of the study, may be permitted on a case-by-case basis following approval by the Sponsor in consultation with the Investigator. Subjects are requested to refrain from taking non-approved concomitant medications from recruitment until the conclusion of the study.

General conditions

31. Any subject who, in the judgment of the Investigator, is likely to be noncompliant during the study, or is unable to cooperate because of a language problem or poor mental development.
32. Any subject in the exclusion period of a previous study according to applicable regulations.
33. Any subject who is the Principal Investigator or any sub-investigator, research assistant, pharmacist, study coordinator, or other staff thereof, directly involved in conducting the study.
34. Any subject without a good peripheral venous access.

Biological status

35. Positive result on any of the following tests: hepatitis B surface antigen (HBs Ag), anti-hepatitis B core antibodies (anti-HBc Ab), anti-hepatitis C virus (anti-HCV) antibodies, anti-human immunodeficiency virus 1 and 2 antibodies (anti-HIV1 and anti-HIV2 Ab).
36. Positive urine drug test. Any drug listed in Section 7.2.1 in the urine drug screen unless there is an explanation acceptable to the Investigator (e.g., the subject has stated in advance that they consumed a prescription or over-the-counter product which contained the detected drug) and/or the subject has a negative urine drug screen on retest by the pathology laboratory. Any subject testing positive for acetaminophen (paracetamol) at screening may still be eligible for study participation, at the Investigator's discretion.
37. Positive alcohol breath test.

Specific to the study

38. Cardiac/QT risk:

- Family history of sudden death or of congenital prolongation of the QTc interval or known congenital prolongation of the QTc interval or any clinical condition known to prolong the QTc interval.
- History of symptomatic cardiac arrhythmias or with clinically relevant bradycardia.
- Electrolyte disturbances, particularly hypokalaemia, hypocalcaemia, or hypomagnesaemia.
- ECG abnormalities in the standard 12-lead ECG (at screening or at pre-inoculation on inoculation day) which in the opinion of the Investigator is clinically relevant or will interfere with the ECG analyses.

39. Known hypersensitivity to artesunate or any of its excipients, artemether or other artemisinin derivatives, piperaquine phosphate, proguanil/atovaquone, primaquine, or 4-aminoquinolines.

40. Known severe reaction to mosquito bites other than local itching and redness.

Subjects who are excluded from participation on study days for any of the above reasons may be eligible to participate on a postponed schedule, if the Investigator considers this appropriate.

Test product, dose and mode of administration, batch number:

Test product names:

P. falciparum K13 blood stage challenge agent

P. falciparum 3D7 blood stage challenge agent

Doses: approximately 2 800 viable *P. falciparum*-infected RBCs

Mode of administration: intravenous

Duration of treatment:

Duration of treatment is as follows: single dose.

**Study drugs, dose and mode of administration, batch number:**<br>Study drugs names:<br>artesunate (Batch Numbers: PPP.18.983, AS160501P)<br>piperaquine phosphate (Batch number: 14536)<br>Doses:<br>Approximately 2 mg/kg artesunate<br>960 mg piperaquine phosphate<br>Mode of administration: oral
**Rescue treatment, dose and mode of administration, batch number:**<br>Reference therapy names:<br>Malarone®<br>Primacin™<br>Doses:<br>4 tablets of Malarone® (atovaquone 250 mg, proguanil hydrochloride 100 mg) once daily, administered orally for 3 days.<br>45 mg primaquine (6 tablets, each containing primaquine phosphate 13.2 mg, equivalent to 7.5 mg of primaquine).<br>Mode of administration: oral.
**Criteria for evaluation:**<br>*Pharmacokinetics:*<br>Blood samples for plasma PK analysis<br>*Pharmacodynamics/ Efficacy:*<br>Blood samples for malaria 18S qPCR<br>*Safety*:<br>Vital signs (heart rate, systolic and diastolic blood pressure, temperature and respiratory rate), physical examination, 12-lead electrocardiogram (ECG), laboratory assessments (biochemistry, haematology and urinalysis), urine pregnancy test (all women) and concomitant medications.

Statistical methods:

The sample size required to complete the primary objective of comparing the artesunate parasite clearance profiles of K13 and 3D7 isolates was estimated based on background information from previous IBSM studies with the 3D7 isolate and fast acting antimalarial drugs, as well as artesunate clearance data from artemisinin-resistant isolates.

Satterthwaite t-test of equal means but unequal variances was used with a 2-sided $\alpha=0.05$, an estimated 30% mean difference in decay rate between the K13 and 3D7 isolates, and a power of 80%. The estimated sample size using a 2:1 ratio of K13 to 3D7 was 18 to 9 subjects.

All randomised subjects who receive any amount of challenge agent (K13 or 3D7) were included in the safety analysis.

Descriptive statistics, including the numbers and percentages for categorical variables and the numbers, means, standard deviations, standard error of the means, medians, minimums and maximums for continuous variables will be provided by cohort and by challenge agents (K13 and 3D7).

Safety and Tolerability:

Continuous safety data will be summarised with descriptive statistics (arithmetic mean, standard deviation [SD], median, minimum and maximum) by cohort and by challenge agents (K13 and 3D7).

Categorical safety data will be summarised with frequency counts and percentages for each cohort and for each malaria challenge agent (K13 and 3D7). Within each study cohort, all active subjects will be pooled into a single active challenge agents (K13 and 3D7) for summary data presentations as applicable.

AEs will be coded using the Medical Dictionary for Regulatory Activities (MedDRA) Version 20.1. A by-subject AE data listing, including verbatim term, preferred term, system organ class, classification as TEAE/SAE, challenge agent, severity, action taken, outcome, time of onset and resolution, time of onset relative to the inoculum and artesunate, duration, and relationship to study agents, will be provided. All AEs will be summarised by system organ class and preferred term and will be provided by cohort and by challenge agent (K13 and 3D7). Additionally, the number of subjects experiencing treatment emergent AEs (TEAEs)will be defined as Adverse Events (AEs) which worsen in severity or commence on or after the time of first study drug administration (artesunate) – and number of individual TEAEs will be summarised by system organ class and preferred term and presented by challenge agents (K13 and 3D7) and by cohort. TEAEs will also be summarised by severity and by relationship to study drugs (artesunate and piperaquine phosphate). In addition, all SAEs, including deaths, will be listed separately and summarised.

Laboratory evaluations, vital signs assessments and ECG parameters will be summarised by challenge agents (K13 and 3D7), by cohort and protocol scheduled collection time point. A

summary of change-from-baseline relative to inoculum, artesunate and piperaquine treatment at each protocol scheduled time-point by challenge agents (K13 and 3D7) as well as by cohort will also be presented and where applicable shift tables will be prepared for laboratory data.

Medical history and prior/ concomitant medications will be listed by challenge agents (K13 and 3D7) and subject.

Malaria clinical score will be listed by challenge agents (K13 and 3D7) and subject.

Pharmacokinetic Analyses:

Concentration-Time Data

Plasma artesunate and Dihydroartemisinin concentration data will be listed for each subject.

Pharmacodynamic/ Efficacy Analyses:

Direct feeding assay, membrane feeding assay, malaria 18S qPCR, female/male gametocyte marker daily/ retrospective, ring stage marker daily/ retrospective. Will be listed for each participant.

Table of Contents

1. LIST OF ABBREVIATIONS....20
2. INTRODUCTION....23
3. STUDY OBJECTIVE(S) AND ENDPOINT(S)....25
3.1. Study Objective(s)....25
3.1.1. Primary Objectives....25
3.1.2. Secondary Objectives....25
3.1.3. Exploratory Objectives....25
3.2. Study Endpoint(s)....25
3.2.1. Primary Endpoints....25
3.2.2. Secondary Endpoints....26
3.2.3. Exploratory Endpoints....26
3.3. Statistical Hypotheses....26
3.4. Pharmacokinetic (PK) and PK/Pharmacodynamic (PD) Hypotheses....27
4. STUDY DESIGN....28
4.1. Definition of Study Agents....30
4.2. Sample Size Considerations....31
4.2.1. Sample Size Justifications....31
4.2.2. Sample Size Re-estimation....31
4.3. Randomisation....31
4.4. Clinical Assessments....31
5. PLANNED ANALYSES....33
5.1. Interim Analysis....33
5.2. Final Analysis....33
6. GENERAL CONSIDERATIONS FOR DATA ANALYSES AND HANDLING....34
6.1. General Summary Table and Individual Subject Data Listing Considerations....34
6.2. General Post Text Summary Table and Individual Subject Data Listing Format Considerations....34
6.3. Data Management....35
6.4. Data Presentation Conventions....35
6.5. Analysis Populations....36

6.5.1. Screen Failures 36
6.5.2. Safety Population 36
6.6. Baseline Definition 36
6.7. Derived and Transformed Data 37
6.7.1. Baseline Age 37
6.7.2. Reference Days 37
6.7.3. Change from Baseline 37
6.7.4. Observed Cases (OC) and Last Observation Carried Forward (LOCF), Missing Data and Outliers 37
6.7.5. Completers 37
6.7.6. Treatment Compliance 37
6.7.7. Other Derivations 38
7. TREATMENT COMPARISONS 39
7.1. Data Display Treatment and Other Sub-Group Descriptors 39
8. GENERAL CONSIDERATIONS FOR DATA ANALYSES 40
8.1. Data handling conventions 40
8.1.1. Premature Withdrawal and Missing Data 40
8.1.2. Handling of Dropouts 40
8.1.3. Additional/Unscheduled Assessments and Missing Data 40
8.1.4. Assessment Windows 41
8.2. Derived and Transformed Data 41
9. STUDY POPULATION 42
9.1. Disposition of Subjects 42
9.2. Protocol Deviations 42
9.3. Demographic and Baseline Characteristics 42
9.4. Treatment Compliance 42
9.4.1. Compliance to Study Drug 43
9.4.2. Measurement of Treatment Compliance 43
9.5. Prior and Concomitant Medications 43
10. SAFETY ANALYSES 44
10.1. Adverse Events 44
10.2. Deaths and Serious Adverse Events 46

10.3. Adverse Events Leading to Discontinuation of Investigational Product and/or Withdrawal from the Study and Other Significant Adverse Events 46
10.4. Pregnancies 46
10.5. Clinical Laboratory Evaluations 46
10.6. Vital Signs Evaluations...46
10.7. ECG Evaluations 46
10.8. Other Safety Measures...47
11. CLINICAL PHARMACOLOGY DATA ANALYSES 48
11.1. Pharmacokinetic Analyses..48
11.1.1. Concentration-Time Data 48
11.1.2. Plasma Pharmacokinetic Parameters 48
11.2. Pharmacodynamic/ Efficacy Analyses 48
11.3. Pharmacokinetic/Pharmacodynamic Analyses 48
12. ATTACHMENTS..49
12.1. Table of Contents for Data Display Specifications 49
12.1.1. Planned Data Listings 49
12.1.2. Planned Data Tables 52

LIST OF TABLES

Table 1: List of Abbreviations 20

1. LIST OF ABBREVIATIONS

Table 1: List of Abbreviations

**Abbreviation**	**Term**
AE	Adverse Event
ALT	Alanine Aminotransferase
AST	Aspartate Aminotransferase
AUC	Area Under the Curve
AUCinf	AUC Curve to Infinite Time
AUClast	AUC Curve to Last Quantifiable Concentration
Cmax	Maximum Plasma Concentration
CNS	Clinical Network Services
CSR	Clinical Study Report
DFA	Direct Feeding Assay
eCRF	Electronic Case Report Form
ECG	Electrocardiogram
EOS	End of Study
G6PD	Glucose-6-Phosphate Dehydrogenase
GCP	Good Clinical Practice
GMP	Good Manufacturing Practice
HAV	Hepatitis A Virus
HBs Ag	Hepatitis B surface antigen
HCV	Hepatitis C Virus
HEV	Hepatitis E Virus
HDL	High Density Lipoprotein
HIV	Human Immunodeficiency Virus
HREC	Human Research Ethics Committee
IBSM	Induced Blood Stage Malaria
ICH	International Conference on Harmonization
IMM	Independent Medical Monitor
IMP	Investigational Medicinal Product
IV	Intravenous

LFT	Liver Function Test
MCB	Master Cell Bank
MedDRA	Medical Dictionary for Regulatory Activities
MFA	Membrane Feeding Assay
MMV	Medicines for Malaria Venture
NHMRC	National Health and Medical Research Council
PD	Pharmacodynamic
PK	Pharmacokinetic
QIMR Berghofer	Queensland Institute of Medical Research Berghofer
qPCR	Quantitative Polymerase Chain Reaction
qRT-PCR	Quantitative Reverse Transcription Polymerase Chain Reaction
QTc	Corrected QT
RBC	Red Blood Cell
Rh	Rhesus Antibody
SAE	Serious Adverse Event
SAP	Statistical Analysis Plan
SOP	Standard Operating Procedures
SRT	Safety Review Team
SUSAR	Suspected Unexpected Serious Adverse Reaction
t1/2	Terminal Half-Life
TEAE	Treatment-Emergent Adverse Event
TGA	Therapeutic Goods Administration
Tmax	Time taken to reach Cmax
ULN	Upper Limit of Normal
WHO	World Health Organization

2. INTRODUCTION

Malaria is one of the most important infectious diseases, which threatens half the world's population. In accordance with the latest estimates published by the World Health Organization (WHO), there were an estimated 212 million new cases of malaria worldwide and 429,000 deaths in 2015. The majority of these deaths were due to infection with *Plasmodium falciparum*.

Artemisinins are the most potent and rapidly acting antimalarial drugs available to date. The WHO currently recommends artemisinin-based combination therapy as the first-line therapy in areas with high prevalence of resistance to other antimalarial drugs. However, resistance to artemisinin-based combination therapies has been reported across the Greater Mekong Subregion in South-East Asia. Artemisinin resistance has been linked to mutations in the propeller domain of the *P. falciparum K13* gene. Artemisinin-resistant isolates are not completely resistant to artemisinins, but instead have a slow parasite clearance phenotype compared to artemisinin-sensitive isolates. Therefore, parasite clearance in artemisinin-resistant severe malaria relies on the activity of the partner drug of the artemisinin combination therapy. Because of the concern for malaria drug resistance, new drugs with new mechanisms of actions are needed as well as efficient systems to determine the safety and clinical activity of these drugs.

The induced blood stage malaria (IBSM) model, whereby subjects are challenged with blood stage malaria parasites, is an attractive tool to test the activity of drugs and vaccines in non-immune subjects in a rapid and cost effective manner. By following the parasitaemia decay in subjects after drug or vaccine treatment by quantitative polymerase chain reaction (qPCR), the antimalarial activity of a treatment can be determined.

The challenge agents used in the IBSM model are produced from blood stage parasite Master Cell Banks (MCBs). The MCBs have been generated following collection of blood from either donors who have been exposed to laboratory developed *Plasmodium* strains, or donors who have acquired malaria naturally while visiting malaria endemic countries.

The *P. falciparum* 3D7 challenge agent has been used in experimental studies in a total of 312 subjects in 25 IBSM clinical trials, 18 of which were successfully undertaken at QIMR Berghofer/Q-Pharm. These trials have demonstrated the safety of the 3D7 challenge agent and the reliability of the approach in terms of infectivity and parasite growth rate. Adverse events (AEs) in these studies were consistent with the symptoms of early malaria infection including headache, fever, fatigue, malaise, myalgia, arthralgia and gastrointestinal upset. The only serious adverse events (SAEs) likely to be related to the 3D7 challenge agent were asymptomatic liver function derangements that resolved by the end of the studies. *P. falciparum* 3D7 is sensitive to artemisinins and standard antimalarial drugs, such as chloroquine, Riamet®, and Malarone®.

A new parasite MCB has recently been generated from a *P. falciparum* Cam3.II^{R539T} (K13) artemisinin-resistant malaria parasite (referred to as K13), which was isolated from a patient in Cambodia with natural malaria infection. The K13 MCB is resistant to dihydroartemisinin, chloroquine, lumefantrine, mefloquine, and quinine, as determined by *in vitro* antimalarial drug susceptibility tests. Conversely, the K13 MCB is sensitive to 4 antimalarial drugs, including piperaquine phosphate and atovaquone (a component of Malarone®), which can be used in the IBSM model to rescue subjects challenged with K13. An IBSM pilot study (QP16C14/P2286) was conducted in which 2 subjects were challenged with ~2 800 viable *P. falciparum* K13 parasite-infected red blood cells (RBCs) and treated with approximately 2 mg/kg artesunate (a semi-

synthetic derivative of artemisinin) on Day 9. The pilot study results showed that the *P. falciparum* K13 challenge agent is safe, well tolerated, and infective (refer to the *P. falciparum* K13 Investigator's Brochure for further details). The availability of a *P. falciparum* artemisinin-resistant challenge agent in the IBSM model will allow investigation of the activity of novel antimalarial drug candidates in clearance of artemisinin-resistant *P. falciparum*, which is a growing problem in South-East Asia and threatens to spread across the world.

This study aims to characterise and compare the parasitological response of the *P. falciparum* K13 (artemisinin-resistant) and 3D7 (artemisinin-sensitive) isolates to the artemisinin antimalarial drug artesunate in healthy subjects inoculated in the IBSM model. We hypothesise that artesunate will clear parasitaemia from subjects challenged with K13 at a 30% slower rate compared to subjects challenged with 3D7.

Once the *P. falciparum* K13 isolate is properly characterised in the IBSM model with respect to resistance to an artemisinin drug, it can then be used in future studies to investigate the activity of novel, investigational antimalarial drugs in clearance of artemisinin-resistant *P. falciparum*. This study will also continue to assess the safety and tolerability of the K13 isolate in healthy subjects.

3. STUDY OBJECTIVE(S) AND ENDPOINT(S)

3.1. Study Objective(s)

3.1.1. Primary Objectives

The primary objectives of the study are:

- To compare the parasite clearance profiles of the *P. falciparum* K13 and 3D7 isolates after administration of artesunate.
- To determine the safety and tolerability associated with infection of healthy subjects with the *P. falciparum* K13 and 3D7 isolates.

3.1.2. Secondary Objectives

Not applicable for this study.

3.1.3. Exploratory Objectives

The exploratory objectives of the study are:

- To characterise the pharmacokinetic profile of artesunate and its active metabolite, dihydroartemisinin, in *P. falciparum* K13 and 3D7 infected subjects.
- To characterise the pharmacokinetic/pharmacodynamic relationship between artesunate and dihydroartemisinin pharmacokinetics and parasite clearance in *P. falciparum* K13 and 3D7 infected subjects.
- To determine if gametocytes appear in the blood of subjects infected with *P. falciparum* K13 or 3D7.
- To determine if K13 and 3D7 gametocytes, if present, are infective to mosquitoes (may be performed in selected cohorts at the Investigator's discretion).
- To investigate and identify any possible associations between subjects genetic polymorphisms and liver function (or phenotype) following infection with *P. falciparum* K13 or 3D7 (optional and requires consent via genetic testing Consent Form).
- To characterise the antimalarial treatment effects on *P. falciparum* K13 and 3D7 parasites using phenotypic and transcriptomic approaches (Cohorts 2 and 3).
- To investigate the *in vivo* presence of dormant parasites (Cohorts 2 and 3).

3.2. Study Endpoint(s)

3.2.1. Primary Endpoints

The primary endpoints of the study are:

- The parasite clearance profiles of *P. falciparum* K13 and 3D7 after administration of artesunate will be characterised by the parasite reduction ratio and parasite clearance $t_{1/2}$. A t-test will be used to compare the slope of the clearance curves for K13 and 3D7 isolates.

- The safety and tolerability of *P. falciparum* K13 and 3D7 infection will be evaluated from adverse event and serious adverse event recording (including severity and causality), physical examinations, vital signs, clinical biochemistry, haematology, and urinalysis.

3.2.2. Secondary Endpoints

Not applicable for this study.

3.2.3. Exploratory Endpoints

The exploratory endpoints of the study are:

- The following pharmacokinetic parameters will be estimated for artesunate and dihydroartemisinin: AUClast, AUCinf, Cmax, Tmax and t1/2.
- A pharmacokinetic/pharmacodynamic model describing the relationship between artesunate and dihydroartemisinin plasma concentration and the effect on parasitaemia measured by qPCR will be developed.
- The presence of gametocytes in subjects will be determined by parasite lifecycle stage qRT-PCR until approximately Day 28±3.
- Infectivity of gametocytes (if present) to mosquitoes will be reported as prevalence of infection (percentage of oocyst positive mosquitoes) (may be performed in selected cohorts at the Investigator’s discretion).
- The absence or presence of a genetic variation that may have a link to changes in liver function (or phenotype) following infection with *P. falciparum* (optional and requires consent via genetic testing Consent Form).
- The following assays will be performed to characterise the antimalarial treatment effects on *P. falciparum* K13 and 3D7 parasites using phenotypic and transcriptomic approaches (Cohorts 2 and 3). 1. Assess re-growth of parasites *ex vivo* by qPCR, flow cytometry and microscopy. 2. Perform *in vitro* drug sensitivity testing determining the 50% inhibitory concentrations (IC50s) and the percentage surviving parasites. 3. Perform molecular methods including whole genome sequencing; identification of molecular markers such as *gEXP-5, pfSBP-1, pfMGET, pfs25* etc; whole RNA sequence analysis to determine family-genes with differential expression between pre- and post-antimalarial treatment.
- The *in vivo* presence of dormant parasites will be investigated and characterised by determining the transcriptional molecular signature of dormant parasites and performing parasite lifecycle stage qRT-PCR on K13 and 3D7 parasites pre- and post-artesunate treatment (Cohorts 2 and 3).

3.3. Statistical Hypotheses

For the parasite clearance primary endpoint, the null hypothesis is that the clearance rate of the K13 and 3D7 isolates are the same in response to artesunate treatment. The alternative hypothesis is that artesunate will clear parasitaemia from subjects challenged with K13 at a 30% slower rate compared to subjects challenged with 3D7.

3.4. Pharmacokinetic (PK) and PK/Pharmacodynamic (PD) Hypotheses

There are no formal PK or PK/ PD statistical hypotheses defined for this Phase I study.

Note: as this SAP does not discuss the analysis of PK/PD, efficacy or exploratory data, the PK/PD, efficacy and exploratory endpoints from the protocol have been listed above for completeness and will not be discussed further in the current SAP.

4. STUDY DESIGN

This Phase 1/1b, single-centre study compared the parasitological response of the *P. falciparum* isolates K13 (artemisinin-resistant) and 3D7 (artemisinin-sensitive) following a single dose of artesunate in healthy subjects using the IBSM model.

The study planned to include up to 27 subjects, in up to 3 cohorts. Up to 18 subjects were to receive the K13 challenge agent and up to 9 subjects were to receive the 3D7 challenge agent, to achieve a ratio of 2:1 over the whole study. However, over the whole study 22 subjects were inoculated with a challenge agent: Cohort 1 (K13, n=3; 3D7 n=3), Cohort 2 (K13 n=7; 3D7 n=3), and Cohort 3 (K13 n=3, 3D7 n=3).

A review of the safety, tolerability and parasitaemia data from the preceding cohort was conducted by the Safety Review Team prior to malaria parasite inoculation of the next cohort. Data from at least 3 weeks post-inoculation was required for each review.

Subjects randomised to receive the K13 challenge agent were inoculated on Day 0 with approximately 2 800 viable *P. falciparum* K13 parasite-infected red blood cells (RBCs). Subjects randomised to receive the 3D7 challenge agent were inoculated on Day 1 with approximately 2 800 viable *P. falciparum* 3D7 parasite-infected RBCs. On an outpatient basis, subjects were monitored daily via phone and then attended the clinical unit daily from 4 days postinoculation – Day 4 for K13 subjects and Day 5 for 3D7 subjects – until positive for presence of malaria parasites by quantitative polymerase chain reaction (qPCR) targeting the 18S rRNA gene (referred to as malaria 18S qPCR). Once qPCR positive, subjects were monitored twice daily until artesunate treatment for parasitaemia, symptoms or signs of malaria, and adverse events. The threshold for commencement of artesunate treatment was when all subjects reach a parasitaemia of ≥5 000 parasites/mL. If the parasitaemia of any subject was ≥5 000 parasites/mL, and was accompanied by a malaria clinical score >6 before all subjects reached the treatment threshold, then treatment of that subject began within a 24 hour period. Subjects could receive artesunate treatment at any time based on parasitaemia and/or clinical symptoms at the Investigator's discretion. Subjects were admitted to the clinical unit (approximately Day 9), administered a single oral dose of approximately 2 mg/kg artesunate, and confined for 72 hours to monitor for safety and tolerability of therapy and to ensure adequate clinical and parasitological response. The plasma concentration-time profiles of artesunate and its active metabolite dihydroartemisinin were also assessed.

Once clinically well, subjects were followed up on an out-patient basis for monitoring of safety and parasite clearance. Subjects 'might also have been evaluated for the presence of sexual parasite stages (gametocytes) and other parasite lifecycle stages in the blood, by parasite lifecycle stage reverse transcription qPCR (qRT-PCR) targeting genes such as the female gametocyte-specific transcript *pfs25*, the male gametocyte-specific transcript *pfMGET*, and/or the ring-stage transcript *pfSBP-1*. Infectivity of parasites to *Anopheles* mosquitoes might have been investigated by direct skin feeding and/or membrane feeding assays, at the Investigator's discretion using parasite lifecycle stage qRT-PCR to determine probability of infectivity.

It was anticipated that artesunate treatment would reduce K13 parasitaemia, although at a slower rate than for the 3D7 artemisinin-sensitive isolate. If artesunate did not clear parasitaemia, subjects

were administered a drug known to be active against both K13 and 3D7 isolates (piperaquine phosphate as a single dose of 960 mg).

Cohort 1:

For Cohort 1, piperaquine phosphate treatment was to occur on an individual subject basis in the event of unsatisfactory parasite clearance, defined as 2 consecutive malaria 18S qPCR time-points showing a decrease in parasitaemia of less than 20% of baseline (i.e., the parasitaemia before drug treatment) by 72 hours post-artesunate treatment. Subjects were also treated with piperaquine phosphate if recrudescence occurred after artesunate treatment, defined as ≥5 000 blood stage parasites/mL and a 2-fold increase within 48 hours, or a malaria clinical score >6. piperaquine phosphate treatment could also be undertaken at the Investigator's discretion.

All Cohort 1 subjects received compulsory rescue treatment with Malarone® (atovaquone/proguanil hydrochloride; 3 day course) on Day 26±3 or earlier at the Investigator's discretion. Subjects might also have been treated with a single oral dose of 45 mg primaquine (Primacin™) at the time of Malarone® treatment, if gametocytaemia was suspected from parasite lifecycle stage qRT-PCR or by the presence of stable low level parasitaemia, or at the Investigator's discretion, to ensure complete clearance of gametocytes.

Cohorts 2 and 3:

For Cohorts 2 and 3, piperaquine phosphate treatment occurred for subjects inoculated with the 3D7 challenge agent as outlined above for Cohort 1. However, K13 subjects in Cohorts 2 and 3 were treated with piperaquine phosphate on Day 11 AM or earlier at the Investigator's discretion. This was to avoid the high parasitaemias seen in K13 subjects in Cohort 1 on Day 11 PM prior to piperaquine phosphate treatment.

All Cohort 2 and 3 subjects were treated with a single oral dose of 45 mg primaquine (Primacin™) on Day 23±3 or earlier at the Investigator's discretion. This was to ensure complete clearance of gametocytes before Day 28±3 (End of Study). All subjects received compulsory rescue treatment with Malarone® (atovaquone/proguanil hydrochloride; 3 day course) on Day 25±3 or earlier at the Investigator's discretion.

All cohorts:

Follow-up for safety assessments was performed on Day 28±3 (End of Study). The Safety Review Team reviewed the safety and tolerability data from the study after completion of the last cohort.

NOTE: when "inoculation day" is referred to, it will mean Day 0 for K13 subjects and Day 1 for 3D7 subjects.

4.1. Definition of Study Agents

The following study agents will be used for this study:

Malaria challenge agents:

P. falciparum K13 blood stage challenge agent

A naturally occurring *P. falciparum* K13 artemisinin-resistant isolate was expanded *in vitro* to produce a blood stage *P. falciparum* K13 Master Cell Bank (MCB). Each K13 inoculum dose will be prepared aseptically from an aliquot of the *P. falciparum* K13 MCB. Each subject randomised to K13 will be inoculated intravenously with a dose of approximately 2 800 viable *P. falciparum* K13-infected RBCs in 2 mL saline for injection.

P. falciparum 3D7 blood stage challenge agent

The *P. falciparum* 3D7 MCB was produced from blood collected from a donor with clinical manifestation of malaria. Each 3D7 inoculum dose will be prepared aseptically from an aliquot of the *P. falciparum* 3D7 MCB. Each subject randomised to 3D7 will be inoculated intravenously with a dose of approximately 2 800 viable *P. falciparum* 3D7-infected RBCs in 2 mL saline for injection.

Investigational medicinal products:

artesunate

artesunate is a semi-synthetic derivative of artemisinin. artesunate 50 mg tablets for oral administration will be used. Subjects will receive a single dose of artesunate tablets of approximately 2 mg/kg.

piperaquine phosphate

If artesunate does not clear the parasites, subjects will be administered 960 mg piperaquine phosphate as a single oral dose. K13 subjects in Cohorts 2 and 3 will be treated with piperaquine phosphate on Day 11 AM or earlier at the Investigator’s discretion.

Antimalarial rescue medications:

Malarone®

Malarone® will be administered to all subjects on Day 26±3 (Cohort 1), or Day 25±3 (Cohorts 2 and 3), or earlier at the Investigator’s discretion. A treatment course consists of 4 tablets of Malarone® (atovaquone 250 mg, proguanil hydrochloride 100 mg) once daily, administered orally for 3 days.

Primacin™ (if required)

Subjects may receive a single oral dose of Primacin™ equivalent to 45 mg primaquine (6 tablets, each containing primaquine phosphate 13.2 mg, equivalent to 7.5 mg of primaquine) at the time of Malarone® treatment (Cohort 1) or on Day 23±3 or earlier at the Investigator’s discretion (Cohorts 2 and 3), to ensure complete clearance of gametocytes.

4.2. Sample Size Considerations

4.2.1. Sample Size Justifications

The sample size required to complete the primary objective of comparing the artesunate parasite clearance profiles of K13 and 3D7 isolates was estimated based on background information from previous IBSM studies with the 3D7 isolate and fast acting antimalarial drugs, as well as artesunate clearance data from artemisinin-resistant isolates.

Satterthwaite t-test of equal means but unequal variances was used with a 2-sided α=0.05, an estimated 30% mean difference in decay rate between the K13 and 3D7 isolates, and a power of 80%. The estimated sample size using a 2:1 ratio of K13 to 3D7 was 18 to 9 subjects.

4.2.2. Sample Size Re-estimation

If the mean difference in parasite clearance between the K13 and 3D7 isolates is greater than 30%, fewer subjects may be required. Therefore, a review of the parasitaemia data from the preceding cohort will be conducted by the SRT prior to inoculation of the next cohort.

4.3. Randomisation

The randomisation for Cohort 1 was to be performed in the rate of 2:1 for K13 to 3D7 challenge agents, for a cohort size of 9. However, only 6 subjects received the challenge agents in Cohort 1 (3 K13 and 3 3D7). Up to 21 subjects distributed over Cohorts 2 and 3 were planned to receive either the K13 or 3D7 challenge agent, to reach up to 27 subjects in total. However, 10 subjects received the challenge agents in Cohort 2 (10 K13 and 3 3D7) and 6 subjects received the challenge agents in Cohort 3 (3 K13 and 3 3D7).

Eligible subjects were randomised into 3 groups prior to Day 0: group 1 (K13 subjects), group 2 (3D7 subjects), and group 3 (the remaining subjects in a sequenced list as the reserves). Group 1 subjects were inoculated on Day 0 with the K13 challenge agent, and group 2 subjects were inoculated on Day 1 with the 3D7 challenge agent.

If any of the group 1 or group 2 subjects do not continue to meet eligibility on the designated inoculation day, a reserve subject from group 3 will replace them in order of the sequenced list, i.e. reserve 1 will be the first replacement, followed by reserve 2, etc.

The randomisation schedule was generated by a statistician; using a validated system. A copy of the randomisation schedule was sent to the clinical unit pharmacist and clinical unit project manager.

Due to feasibility, this study was not blinded.

4.4. Clinical Assessments

Screening

Demography (date of birth, age, gender, race and ethnicity, height, weight, body mass index (BMI)), medical history, prior medications, RBC alloantibodies, G6PD testing, serology (HIV 1/2 and Hepatitis B and C), urine drug screen and alcohol breath test, serum pregnancy test (all women) and FSH test (post-menopausal women only).

Safety

Vital signs (heart rate, systolic and diastolic blood pressure, temperature and respiratory rate), physical examination, 12-lead electrocardiogram (ECG), laboratory assessments (biochemistry, haematology and urinalysis), urine pregnancy test and concomitant medications.

Pharmacokinetics

Blood samples for plasma PK analysis.

Pharmacodynamics/ Efficacy

Blood samples for malaria 18S qPCR.

5. PLANNED ANALYSES

5.1. Interim Analysis

No formal interim analyses was conducted.

5.2. Final Analysis

The analysis of the data will be conducted once all subjects have completed the study and the clinical database has been locked.

6. GENERAL CONSIDERATIONS FOR DATA ANALYSES AND HANDLING

6.1. General Summary Table and Individual Subject Data Listing Considerations

Summary tables and listings will be prepared according to ICH Guideline E3 (as appropriate for a Phase I study).

In general, summary tables will be organised with respect to individual cohorts in each inoculum group (K13 or 3D7) and presented as:

- Cohort 1 K13
- Cohort 2 K13
- Cohort 3 K13
- Total K13
- Cohort 1 3D7
- Cohort 2 3D7
- Cohort 3 3D7
- Total 3D7

Where applicable, a Total column, encompassing information from all 6 cohorts from both challenge agents will be included.

Adverse event preferred terms and body/organ systems and medical history conditions will be coded using the Medical Dictionary for Regulatory Activities (MedDRA®) dictionary Version 20.1.

Listings will also be sorted by randomized challenge agents (K13 and 3D7) and randomisation number. Listings will also include visit date/time as well as days relative to inoculation (Day 0 for K13 and Day 1 for 3D7) and days relative to administration of artesunate.

In general, missing data will not be imputed unless otherwise specified. Any imputed or derived data will be flagged in the individual subject data listings. Imputed data will not be incorporated into any raw or primary datasets. These data will be retained in derived analysis datasets.

6.2. General Post Text Summary Table and Individual Subject Data Listing Format Considerations

The tables, figures and listings will be numbered using a decimal system to reflect main levels of unique tables and listings and sub-levels of replicate tables and listings with two digits per level (e.g., Table XX.YY.ZZ. …).

1. The first level number will be consistent with the corresponding Clinical Study Report (CSR) appendix in which the tables or listings will appear. For example, the post text tables will appear in Appendix 14 (and will be numbered 14.XX.YY) and the individual subject data listings will appear in Appendix 16 (and will be numbered 16.XX.YY). The subject disposition table will be first in the first section of the report and will be

numbered Table 14.1.1. The supportive subject data listing will be Listing 16.2.1. Any subset table will have the number Table 14.1.2, etc.

2. Table numbering will follow ICH E3 for Phase I CSRs. Subject disposition, baseline and demography tables should appear as the second level number (Table 14.1 series). Safety tables will follow next (Table 14.3 series). Similar conventions will be applied to the subject data listings.
3. Each table and listing title will be complete, accurate and concise. The last line of the title will provide the analysis group being summarized (e.g., Safety Population).

6.3. Data Management

All data will be recorded by the site in individual source documents. An eCRF will be created by the data management group for recording of the required data in the study database. All eCRF information is to be filled in by site staff. If an item is not available or is not applicable, this fact should be indicated. Blank spaces should not be present unless otherwise directed.

The study monitor will perform source data verification of data entered into the eCRF and raise queries for correction by the site. The data entered into the eCRF will be subject to data validation checks for consistency and completeness by the data management group. Data queries will then be generated and sent to the investigational site for response before the database is locked and released for statistical analysis.

Data storage, data transfer and data cleaning will be conducted according to the relevant CNS Standard Operating Procedures (SOPs).

Derived datasets are created using SAS® software. Data analyses and summary tables will be generated using the currently supported version at the time of data analysis.

6.4. Data Presentation Conventions

Continuous safety variables (e.g., clinical laboratory values, vital signs and ECGs) will be reported to the same precision as the source data. Derived variables will be reported using the same precision as the value(s) from which they were derived. For the reporting of descriptive statistics, the mean and median will be reported to 1 decimal place more than the source data; the minimum and the maximum values will be presented to the same precision as the source data and standard deviations (SD) and standard error of the mean (SEM) will be reported to 2 decimal places more than the source data. The coefficient of variation (CV%), as a percentage (i.e., x100%), will always be reported to 1 decimal place.

For categorical/discrete variables, the frequency count and the percentage (of available data) for each class of the variable will be presented and will be displayed in the form XX (XX.X%) where the percentage is in the parentheses.

Date variables will be formatted as DDMMMYYYY for presentation. Time will be formatted in military time as HH:MM for presentation.

Extra measurements (such as unscheduled or repeat assessments) will not be included in summary tables but will be included in subject listings. They will be used in summary tables which are not 'time specific', for example, summaries of maximum post dose values.

Wherever possible, data will be decimal aligned.

The table, figures and listing shells and table of contents as part of this Statistical Analysis Plan (SAP) provide the expected layout and titles of the tables, listings and figures. Any changes to format, layout, titles, numbering, or any other minor deviation will not necessitate a revision to the SAP nor will it be considered a deviation from planned analyses. Only true differences in the analysis methods or data handling will necessitate such documentation. The appropriate listings supporting the tables will be included and are not specified in the individual sections throughout the document.

Minor modifications may be necessary to the planned design of tables, listings and figures to accommodate data collected during the actual study conduct. Any major deviations from the final approved SAP (e.g., change in the population used, change from statistical method/assumption listed, transformation of data type [e.g., continuous data transformed to categorical], exclusion of planned analysis, etc.) or additional unplanned analyses will be documented (with justification) in the CSR.

6.5. Analysis Populations

Subject inclusion into each population will be determined after database lock for the final analysis.

6.5.1. Screen Failures

Data from screen failures will not be recorded in the eCRF. Reasons for screen failures will be collected in study screening logs for inclusion in the CSR.

6.5.2. Safety Population

The Safety population will comprise all randomised subjects who were inoculated and will be based on challenge agents (K13 and 3D7) received, if this differs from what the subject was randomised to. The Safety population will be used for the summaries of all safety data. In addition, the safety population will be used for the summaries of all disposition, demographics and baseline and prior/concomitant medications. All listings will also be produced using the Safety population.

6.6. Baseline Definition

A separate baseline will be defined for each treatment period and will be defined as the last available, valid, non-missing assessment prior to first study agent administration for each period (inoculum, artesunate and piperaquine phosphate). Baseline relative to piperaquine phosphate will be used only for AEs, QT parameters of ECT summary tables and listings and ALT and AST summaries and listings.

In general, Unknown, Not Done, Not Applicable and other classifications of missing data will not be considered when calculating baseline observations. However, valid categorical observations will be considered for baseline calculations. In addition, non-missing results from

unscheduled assessments prior to first study drug administration may also be considered in the calculation of baseline observations.

6.7. Derived and Transformed Data

6.7.1. Baseline Age

Age, in completed years, at screening will be defined as:

Age (years) = integer value ((Date Signed Informed Consent – Date of Birth + 1) / 365.25)

6.7.2. Reference Days

There will be two reference days calculated:

1. Day relative to inoculation
2. Day relative to artesunate

The calculation of these reference days will use the first administration of inoculation (for day relative to inoculation) and first day of artesunate administration (for day relative to artesunate).

If the date of interest occurs on or after the first reference agent administration date, then reference day will be calculated as (date of interest – date of first study agent administration) + 1. If the date of interest occurs prior to the first study agent administration date, then reference day will be calculated as (date of interest – date of first study agent administration).

Data listings will present both reference days (day relative to inoculation, as well as day relative to artesunate) in addition to assessment dates. With regards to Day relative to artesunate, the first day of dosing of artesunate will be identified as Day 0. With regards to Day relative to inoculation, the first day of dosing of inoculation will be identified as Day 0 for K13 subjects and Day 1 for 3D7 subjects.

6.7.3. Change from Baseline

Change from baseline will be calculated as (post-baseline result – baseline result). This calculation will be applied to all three baselines (relative to administration of the three study agents inoculum, artesunate and piperaquine phosphate).

6.7.4. Observed Cases (OC) and Last Observation Carried Forward (LOCF), Missing Data and Outliers

Not applicable for this study. Missing data will not be imputed.

6.7.5. Completers

Not applicable for this study.

6.7.6. Treatment Compliance

Not applicable for this study.

6.7.7. Other Derivations

Body Mass Index (kg/m^2, to one decimal place) = Body Weight (kg) / (Height x Height (m)).

An AE is any adverse change, i.e., any unfavourable and unintended sign (including an abnormal laboratory finding), symptom or disease that occurs in a subject during the course of the study, whether or not considered by the Investigator as related to study treatment.

A treatment-emergent adverse event (TEAE) is an event that emerges or worsens following treatment with the investigational medicinal products (artesunate and piperaquine phosphate, if administered), having been absent pre-treatment, or worsens relative to the pre-treatment state.

AEs without an onset date or time will be defined as treatment emergent except if an incomplete date (e.g., month and year) clearly indicates that the event started prior to first study drug administration or if the AE stop date indicates that the event started and/or stopped prior to first study drug administration.

Adverse event time since study agent (inoculum, artesunate or piperaquine phosphate) administration (in hours, to one decimal place) = (Onset Date + Onset Time) – (Study Agent Administration Date + Study Agent Administration Time).

Duration of AE (in hours, to two decimal place) = (Resolution Date + Resolution Time) - (Onset Date + Onset Time).

Actual PK sample time post-dose (in hours, to two decimal places) = (Sample Date + Sample Time) – (Study Drug Administration Date + Dose Time).

7. TREATMENT COMPARISONS

7.1. Data Display Treatment and Other Sub-Group Descriptors

The following labels for the cohorts of each challenge agent (K13 and 3D7) will be used on all tabulations, in the following order:

- Cohort 1 K13
- Cohort 2 K13
- Cohort 3 K13
- Total K13
- Cohort 1 3D7
- Cohort 2 3D7
- Cohort 3 3D7
- Total 3D7
- Total subjects (if applicable)

8. GENERAL CONSIDERATIONS FOR DATA ANALYSES

The listings, figures and summary tables for the safety will be the responsibility of the study Biostatistician at CNS.

Pharmacokinetic analysis of concentration-time data (i.e., PK parameter estimation) and PD efficacy will be not be the responsibility of CNS.

The currently supported version of SAS software will be used to perform all data analyses. The actual SAS version used will be presented in the Clinical Study Report.

All data in the database will be presented in the data listings. Unless otherwise stated, all listings will be sorted by cohort, subject number and assessment date/time. In the listings, subject number will be of the format Rcnn, etc.

Unless otherwise stated, continuous variables will be summarized using descriptive statistics including number of non-missing observations, mean, standard deviation, standard error of the mean, median, minimum and maximum values. The minimum and maximum values will be presented to the same number of decimal places as the source data, mean and median will be presented to one more decimal place than the source data, and standard deviation and standard error of the mean will be presented to two more decimal places than the source data.

Categorical variables will be summarized with frequency counts and percentages. The population size (N for sample size and n for available data) and the percentage (of available data) for each class of the variable will be presented. Percentages will be rounded to one decimal place, with the denominator being the number of subjects in the relevant population, unless otherwise stated.

Only data from nominal protocol scheduled visits will be included in the summary tables. Data from unscheduled visits will be used in summary tables which are not ‘time specific’, for example, summaries of maximum post dose values.

8.1. Data handling conventions

8.1.1. Premature Withdrawal and Missing Data

For subjects who are withdrawn from the study prior to study completion, all data compiled up to the point of discontinuation will be used for analysis. All withdrawals will be included in all analyses up to the time of withdrawal.

There will be no imputation for missing data, unless otherwise stated.

8.1.2. Handling of Dropouts

No imputation of missing data will be performed for early termination subjects. All data collected prior to drop out will be listed and included in data summaries.

8.1.3. Additional/Unscheduled Assessments and Missing Data

Generally, missing dates/times will not be imputed. Adverse events with missing or partial dates will be handled such that in the absence of contradictory information an AE is treatment emergent. So for a missing start date (where the stop date is after the date of first study drug (artesunate) administration the start date will be imputed as the first study agent administration

date. Similarly, for a missing stop date, the stop date will be imputed as the date of last visit. If a partial date is recorded, the following convention will be used to assign the AE:

- If a start date is missing the day information and the month/year is the same as the first study agent administration date, then use the first study agent administration date, else ‘01’ will be used for the day. If a start date is missing the month and the year is the same as the first study agent administration date, then use the first study agent administration date, else ‘January’ will be used for the start month.
- If a stop date is missing the day information and month/year is the same as the last study date, then use the last study date, else the last day of the given month will be used for the stop day. If a stop date is missing the month and the year is the same as the last study date, then use the last study date, else ‘December’ will be used for the stop month.

Extra measurements (such as unscheduled or repeat assessments) will not be included in summary tables but will be included in subject listings. They may be used if a scheduled measurement is missing and the timing of the extra measurement is suitable. They will be used in summary tables which are not ‘time specific’; for example, summaries of maximum post dose values.

The original data will always be presented in the listings.

8.1.4. Assessment Windows

No visit windows will be applied to assessments. All assessments will be included in the listings.

8.2. Derived and Transformed Data

The required endpoints and variables will be derived by the Statistical Programmers at CNS using the derivations specified in Section 6.7 of this SAP.

9. STUDY POPULATION

All disposition, baseline and demographic analyses will be conducted on the Safety population. All summaries will be by challenge agents (K13 and 3D7) as well as by cohort.

9.1. Disposition of Subjects

A disposition listing will present date/time of informed consent, date of randomisation, date/time of inoculation, date/time of artesunate treatment, date/time of piperaquine treatment, date of study completion or early termination, the primary reason for early discontinuation, if applicable and whether included in each population and reasons for exclusion, for each subject.

A listing of whether or not all inclusion and exclusion criteria were met and if not, which criteria were not met, by subject, will also be presented.

The following will be summarized, by challenge agent (K13 or 3D7) and by cohort:

- Number of subjects randomised
- Number of subjects inoculated
- Number of subjects completed the study
- Number of subjects withdrawn and reason for withdrawal
- Number of subjects in each analysis population

9.2. Protocol Deviations

Subject data will be examined for evidence of protocol deviations in order to assess how well the protocol was followed.

All protocol deviations will be detailed in listings.

9.3. Demographic and Baseline Characteristics

For the purposes of listing and tabulating demographic and baseline characteristics, ‘baseline’ in this context is referred to as data recorded at screening and prior to inoculation. All baseline and demographic data recorded at screening and prior to inoculation will be listed.

Subject demographic and baseline variables (age, gender, race, ethnicity, height, body weight and BMI) will be summarized, by challenge agent (K13 or 3D7) as well as by cohort.

Medical history, prior and concomitant medications, RBC alloantibodies, G6PD testing, urine/serum pregnancy test (all women), urine drug test, alcohol breath test, serology (HIV 1/2 (anti-HIV1 and anti-HIV2 Ab), Hepatitis B (HBs Ag, anti-HBc (IgG + IgM if IgG is positive), Hepatitis C (anti-HCV) and FSH (post-menopausal women only) will be listed only.

9.4. Treatment Compliance

All study agents administration data will be listed. In addition, protocol adherence at study agents administration will also be recorded and listed. Individual records of date and time of inoculum administration, IMP (artesunate and piperaquine phosphate) administration and rescue medication administration will be presented in individual data listings.

9.4.1. Compliance to Study Drug

Not applicable for this study.

9.4.2. Measurement of Treatment Compliance

Not applicable for this study.

9.5. Prior and Concomitant Medications

The start dates of medications will be used to assign medications into different categories.

- Prior medication: Any medication which started before the date of inoculation, regardless of when it ended. If the start date of the medication is not complete or missing, it will be treated as prior medication.
- Concomitant medication: Any medication which continued or started on or after the date of inoculation until the end of the study (last visit).

Medications will be classified as a prior medication and/or a concomitant medication. If a medication has a missing or partial missing start/end date or time and it cannot be determined whether it was taken before the date of inoculation, concomitantly, or after the last dose of study drug, it will be considered as both prior and concomitant.

Prior medications taken at any stage from 4 weeks prior to inoculation will be listed by challenge agent received and subject number. Concomitant medication taken during the study period will be listed by challenge agent received and subject number.

10. SAFETY ANALYSES

All safety analyses will be conducted on the Safety population.

Summaries of laboratory data, vital signs and ECG data will be presented by challenge agent (K13 or 3D7), by cohort as well as overall subjects. Summary tables will present absolute values as well as change from baseline. There will be three separate change from baseline measures presented:

1. Baseline relative to inoculum,
2. Baseline relative to artesunate and
3. Baseline relative to piperaquine phosphate (QT parameter of ECG, ALT and AST only).

All summaries of adverse events will be summarized by challenge agent (K13 or 3D7), by cohort, as well as overall subjects for inoculum, artesunate and piperaquine phosphate.

No inferential statistical testing will be performed for safety variables.

10.1. Adverse Events

Adverse events will be coded using MedDRA® Version 20.1. Adverse events will be grouped by system organ class (SOC) and preferred term (PT) and summarized, by challenge agent. The summary tables will present the frequency and percentage of total subjects, by system organ class and by preferred term. The event rate and number of follow-up weeks will also be presented.

AE summaries will be presented for inoculum, artesunate and piperaquine phosphate, separately. TEAEs summaries will be restricted to artesunate and piperaquine phosphate only. For the summaries of AEs, subjects who experience the same AE (in terms of the MedDRA preferred term) more than once will only be counted once for that event in the number of subjects and will be summarized according to the maximum severity recorded for that AE. Similarly, for relationship to study drug.

Note, the severity categories are Grade 1: Mild, Grade 2: Moderate, Grade 3: Severe or medically significant, Grade 4: Life-threatening and Grade 5: Death related to AE.

Any AEs with a missing or unknown severity will be considered as the highest severity (Severe) in the summary tables. Similarly, any AEs with a missing or unknown relationship to study agents (Inoculum, artesunate and piperaquine phosphate) will be considered related to study agent in the summary tables.

Incidence of adverse events as well as onset and end date/time, duration, severity, relationship to treatment, classification as TEAE, classification as SAE, AE verbatim/ system organ class/ preferred term, outcome and actions taken will be listed for each subject. In addition, listings of AEs leading to discontinuation of the study, SAEs and deaths, will be provided if applicable.

The following AE summaries will be provided:

- Overall summary of AEs/ TEAEs
  - Any AE/ TEAEs

  - Any severe AE/ TEAEs (or Grade 3, Grade 4, Grade 5)
  - Any AE/ TEAEs considered as related to study agent (evaluated by the investigator as possibly related or probably related)
  - Any serious AE/ TEAEs
  - Any serious and related AE/ TEAEs
  - Any AE/ TEAEs leading to study drug withdrawal
  - Any AE/ TEAEs leading to discontinuation from study
  - Any life threatening AE/ TEAEs
  - Any AE/ TEAEs leading to death
- Summary of AEs/ TEAEs
  - Number of AEs/ TEAEs
  - Number (%) of subjects reporting at least one:
    - AE/ TEAEs
    - Serious AE/ TEAEs
    - AE/ TEAEs leading to study drug withdrawal
    - AE/ TEAEs leading to discontinuation from study
  - Number (%) of subjects with AEs/ TEAEs by severity
  - Number (%) of subjects with AEs/ TEAEs by relationship to study agent (inoculum, artesunate and piperaquine phosphate)
- AE/ TEAEs by PT (sorted by decreasing frequency of total events overall)
- AEs/ TEAEs overall and by SOC and PT
- AEs/ TEAEs related to study agent, overall and by SOC and PT (an AE related to study agent is defined as any AE that reported as being possibly related or probably related to the study agent)
- AEs/ TEAEs by maximum severity, overall and by SOC and PT
- AEs/ TEAEs by maximum relationship to study agent, overall and by SOC and PT
- AEs/ TEAEs leading to study agent withdrawal, overall and by SOC and PT
- AEs/ TEAEs leading to discontinuation from study, overall and by SOC and PT
- Serious AEs/ TEAEs overall and by SOC and PT

In the summary tables, AEs/ TEAEs will be presented by decreasing frequency of total events overall within each SOC and then similarly by decreasing frequency of total events overall within each PT. SOCs or PTs with equal frequencies will be sorted alphabetically.

Note: study agent refers to inoculum, artesunate and piperaquine phosphate.

TEAEs defined as any AE with an onset after administration of IMP (artesunate and piperaquine phosphate). AEs occurred at the time of inoculation or after will be summarized as AEs.

10.2. Deaths and Serious Adverse Events

Incidence of SAEs as well as the duration, severity, relationship to treatment, outcome and actions taken will be listed for each subject and summarized by actual treatment with frequency counts by SOC and PT.

Deaths, if any, will be listed for subject, challenge agents (K13 and 3D7), date, primary cause and autopsy finding and summarized with frequency counts by SOC and PT.

10.3. Adverse Events Leading to Discontinuation of Investigational Product and/or Withdrawal from the Study and Other Significant Adverse Events

Incidence of AEs leading to study drug withdrawal or discontinuation from study, as well as the duration, severity, relationship to treatment, outcome and actions taken will be listed for each subject.

10.4. Pregnancies

All pregnancy test (serum and urine) data will be listed only.

10.5. Clinical Laboratory Evaluations

All individual clinical laboratory results and clinical assessment (haematology, biochemistry and urinalysis) will be presented in data listings. Normal ranges will also be included in the listings. Values outside the laboratory reference range will be flagged (low-L, high-H) and clinical significance will be assigned as Clinically Significant (CS) or Not Clinically Significant (NSC).

Actual values, actual change from baseline (inoculation, artesunate treatment and piperaquine treatment) and clinical assessment for clinical laboratory data (haematology, biochemistry and urinalysis) will be summarized at each protocol scheduled time point, by challenge agent (K13 or 3D7).

Any available microscopic urinalysis will be listed.

10.6. Vital Signs Evaluations

All individual vital signs results (systolic blood pressure, diastolic blood pressure, heart rate, respiratory rate, body temperature) will be presented in data listings.

Actual values and actual change from baseline (inoculation, artesunate treatment and piperaquine phosphate treatment) for vital signs parameters will be summarized at each protocol scheduled time point, by challenge agent (K13 or 3D7).

10.7. ECG Evaluations

All individual ECG parameter results (heart rate, PR interval, RR interval, QRS interval, QT interval and QTcF interval) and the clinical assessment of the ECG from all scheduled and unscheduled visits will be presented in data listings.

Actual values and change from baseline (inoculation, artesunate treatment and piperaquine phosphate treatment) for ECG parameter values will be summarized at each protocol scheduled time point, by challenge agent (K13 or 3D7) as well as by cohort.

The number of subjects with maximum post-dose QTc values ≤450 ms, >450 ms, >480 ms and >500 ms will be tabulated from the three baselines (inoculum, artesunate and piperaquine phosphate) by challenge agent (K13 or 3D7) as well as by cohort. In addition, the number of subjects with maximum post-dose increases from baseline < 30 ms, ≥30 ms and ≥ 60 ms will be tabulated by challenge agent (K13 or 3D7) as well as by cohort.

10.8. Other Safety Measures

All physical examination data will be listed.

Malaria clinical score data will be presented, in a listing, for all subjects included in the Safety population.

11. CLINICAL PHARMACOLOGY DATA ANALYSES

All PK listings will be based on the safety population.

11.1. Pharmacokinetic Analyses

11.1.1. Concentration-Time Data

Plasma artesunate and Dihydroartemisinin concentration data will be listed for each subject.

11.1.2. Plasma Pharmacokinetic Parameters

Not applicable for this study.

11.2. Pharmacodynamic/ Efficacy Analyses

Direct feeding assay, membrane feeding assay, malaria 18S qPCR, qRT-PCR *pfs25* daily/retrospective, qRT-PCR *pfMGET* daily/retrospective and qRT-PCR *pfSBP-1* daily/retrospective will be listed for each subject.

11.3. Pharmacokinetic/Pharmacodynamic Analyses

Not applicable for this study.

12. ATTACHMENTS

12.1. Table of Contents for Data Display Specifications

12.1.1. Planned Data Listings

The following listings are planned to be generated for the QP17C11-P2339 study (Note: Numbering is indicative only and may be updated):

Listing Number	Listing Title	Population
16.2.1 Subject Disposition		
Listing16.2.1.1	Study Enrolment and Completion/Discontinuation	Safety
Listing16.2.1.2	Visit Dates	Safety
16.2.2 Protocol Deviations		
Listing16.2.2.1	Protocol Deviations	Safety
16.2.3 Subject Excluded from Analyses		
Listing16.2.3.1	Analysis Population Assignment	Safety
16.2.4 Demographic and Other Baseline Data		
Listing16.2.4.1	Demography	Safety
Listing16.2.4.2	Inclusion and Exclusion Criteria	Safety
Listing16.2.4.3	Medical History	Safety
Listing16.2.4.4	Pregnancy Test	Safety
Listing16.2.4.5	Follicle Stimulating Hormone (FSH) Test	Safety
Listing16.2.4.6	RBC Alloantibody	Safety
Listing16.2.4.7	Serology	Safety
Listing16.2.4.8	Urine Drug Screen	Safety
Listing 16.2.4.9	Alcohol Breath Test	Safety
Listing16.2.4.10	G6PD	Safety
Listing16.2.4.11	Body Weight, Height and BMI	Safety
Listing16.2.4.12.a	Prior Medications	Safety
Listing16.2.4.12.b	Concomitant Medications	Safety
16.2.5 Compliance and Drug Concentration Data		
Listing16.2.5.1	Malaria Inoculum Administration	Safety
Listing16.2.5.2	Artesunate Administration	Safety

Listing16.2.5.3	Piperaquine phosphate Administration	Safety
Listing16.2.5.4	Rescue Medications (Malarone® and Primacin™) Administration	Safety
Listing16.2.5.5	Plasma Pharmacokinetic Sample Collection Times And Concentrations For Artesunate	Safety
Listing16.2.5.6	Plasma Pharmacokinetic Sample Collection Times And Concentrations For Dihydroartemisinin	Safety
16.2.6 Individual Efficacy Response Data		
Listing16.2.6.1	Direct Feeding Assay	Safety
Listing16.2.6.2	Membrane Feeding Assay	Safety
Listing16.2.6.3	Malaria 18S qPCR	Safety
Listing16.2.6.4	qRT-PCR *pfs25* Daily	Safety
Listing16.2.6.5	qRT-PCR *pfs25* Retrospective	Safety
Listing16.2.6.6	qRT-PCR *pfMGET* Daily	Safety
Listing16.2.6.7	qRT-PCR *pfMGET* Retrospective	Safety
Listing16.2.6.8	qRT-PCR *pfSBP-1* Daily	Safety
Listing16.2.6.9	qRT-PCR *pfSBP-1* Retrospective	Safety
16.2.7 Adverse Event Data		
Listing16.2.7.1	Adverse Events	Safety
Listing16.2.7.1a	Adverse Events Related to Inoculum Only	Safety
Listing16.2.7.1b	Treatment Emergent Adverse Events Related to Artesunate Only	Safety
Listing 16.2.7.1c	Treatment Emergent Adverse Events Related to Piperaquine Only	Safety
Listing16.2.7.2	Serious Adverse Events	Safety
Listing16.2.7.3	Adverse Events leading to Discontinuation or Withdrawal	Safety
Listing16.2.7.4	Deaths	Safety
16.2.8 Laboratory Data		
Listing16.2.8.1.a	Individual Haematology Results	Safety
Listing16.2.8.1.b	Individual Abnormal Haematology Results	Safety
Listing16.2.8.2.a	Individual Biochemistry Results	Safety
Listing16.2.8.2.b	Individual Abnormal Biochemistry Results	Safety
Listing16.2.8.3.a	Individual Urinalysis Results (Dipstick)	Safety
Listing16.2.8.3.b	Individual Abnormal Urinalysis Results (Dipstick)	Safety

Listing16.2.8.4.a	Individual Microscopy and Urinalysis Results (Laboratory)	Safety
Listing16.2.8.4.b	Individual Abnormal Microscopy and Urinalysis Results (Laboratory)	Safety
16.4 Other Safety Data		
Listing16.4.1	Vital Signs (Blood Pressure and Heart Rate)	Safety
Listing16.4.2	Vital Signs (Body Temperature and Respiratory Rate)	Safety
Listing16.4.3	Individual Abnormal Vital Signs	Safety
Listing16.4.4.a	ECG Findings	Safety
Listing16.4.4b	Abnormal ECG Interpretation	Safety
Listing16.4.5	Physical Examination	Safety
Listing16.4.5	Individual Abnormal Physical Examination	Safety
Listing16.4.7	Malaria Clinical Score	Safety
Listing16.4.8	General Comments	Safety

12.1.2. Planned Data Tables

The following tables are planned to be generated for the QP17C11-P2339 study (Note: Numbering is indicative only and may be updated):

Table Number	Table Title	Population
14.1 Demographic Data		
Table14.1.1	Study Participation and Disposition	All- Subjects
Table14.1.2	Demographics and Baseline Characteristics	All- Subjects
Table14.1.2a	Demographics and Baseline Characteristics	Safety
Table14.1.3	Study Drug Administration	Safety
14.3 Safety Data		
Table14.3.1.1a	Overall Summary of Adverse Events (Inoculum)	
Table14.3.1.1b	Overall Summary of Treatment-Emergent Adverse Events (Artesunate)	Safety
Table14.3.1.1c	Overall Summary of Treatment-Emergent Adverse Events (Piperaquine Phosphate)	Safety
Table14.3.1.2a	Adverse Events (Inoculum) by System Organ Class and Preferred Term and by challenge agents	Safety
Table14.3.1.2b	Treatment-Emergent Adverse Events (Artesunate) by System Organ Class and Preferred Term and by challenge agents	Safety
Table14.3.1.2c	Treatment-Emergent Adverse Events (Piperaquine Phosphate) by System Organ Class and Preferred Term and by challenge agents	Safety
Table14.3.1.3a	Adverse Events (Inoculum) by Maximum Severity and challenge agents	Safety
Table14.3.1.3b	Treatment-Emergent Adverse Events (Artesunate) by Maximum Severity and challenge agents	Safety
Table14.3.1.3c	Treatment-Emergent Adverse Events (Piperaquine Phosphate) by Maximum Severity and challenge agents	Safety

Table Number	Table Title	Population
Table14.3.1.4a	Related Adverse Events (Inoculum) by challenge agents	Safety
Table14.3.1.4b	Treatment-Related Treatment-Emergent Adverse Events (Artesunate) by challenge agents	Safety
Table14.3.1.4c	Treatment-Related Treatment-Emergent Adverse Events (Piperaquine Phosphate) by challenge agents	Safety
Table14.3.1.5a	Related Adverse Events from Inoculum by Maximum Severity and challenge agents	Safety
Table14.3.1.5b	Treatment-Related Treatment-Emergent Adverse Events from Artesunate by Maximum Severity and challenge agents	Safety
Table14.3.1.5c	Treatment-Related Treatment-Emergent Adverse Events from Piperaquine Phosphate by Maximum Severity and challenge agents	Safety
14.3.4 Clinical Laboratory Data		
Table14.3.4.1a	Haematology Results and Change from Baseline (Inoculum) by challenge agents and Scheduled Assessment Time-point	Safety
Table14.3.4.1b	Haematology Results and Change from Baseline (Artesunate) by challenge agents and Scheduled Assessment Time-point	Safety
Table14.3.4.2a	Biochemistry Results and Change from Baseline (Inoculum) by challenge agents and Scheduled Assessment Time-point	Safety
Table14.3.4.2b	Biochemistry Results and Change from Baseline (Artesunate) by challenge agents and Scheduled Assessment Time-point	Safety
Table14.3.4.2c	ALT and AST and Change from Baseline (piperaquine phosphate) by challenge agents and Scheduled Assessment Time-point	Safety
14.3.5 Vital Signs		
Table14.3.5.1a	Heart Rate and Change from Baseline (Inoculum) by challenge agents and Scheduled Assessment Time-point	Safety

Table Number	Table Title	Population
Table14.3.5.1b	Heart Rate and Change from Baseline (Artesunate) by challenge agents and Scheduled Assessment Time-point	Safety
Table14.3.5.2a	Systolic Blood Pressure and Change from Baseline (Inoculum) by challenge agents and Scheduled Assessment Time-point	Safety
Table14.3.5.2b	Systolic Blood Pressure and Change from Baseline (Artesunate) by challenge agents and Scheduled Assessment Time-point	Safety
Table14.3.5.3a	Diastolic Blood Pressure and Change from Baseline (Inoculum) by challenge agents and Scheduled Assessment Time-point	Safety
Table14.3.5.3b	Diastolic Blood Pressure and Change from Baseline (Artesunate) by challenge agents and Scheduled Assessment Time-point	Safety
Table14.3.5.4a	Respiratory Rate and Change from Baseline (Inoculum) by challenge agents and Scheduled Assessment Time-point	Safety
Table14.3.5.4b	Respiratory Rate and Change from Baseline (Artesunate) by challenge agents and Scheduled Assessment Time-point	Safety
Table14.3.5.5a	Temperature and Change from Baseline (Inoculum) by challenge agents and Scheduled Assessment Time-point	Safety
Table14.3.5.5b	Temperature and Change from Baseline (Artesunate) by challenge agents and Scheduled Assessment Time-point	Safety
14.3.6 ECG Findings		
Table14.3.6.1a	ECG parameter (Heart Rate) and Change from Baseline (Inoculum) by challenge agents and Scheduled Assessment Time-point	Safety
Table14.3.6.1.b	ECG parameter (Heart Rate) and Change from Baseline (Artesunate) by challenge agents and Scheduled Assessment Time-point	Safety
Table14.3.6.2.a	ECG parameter (PR Interval) and Change from Baseline (Inoculum) by challenge agents and Scheduled Assessment Time-point	Safety

Table Number	Table Title	Population
Table14.3.6.2.b	ECG parameter (PR Interval) and Change from Baseline (Artesunate) by challenge agents and Scheduled Assessment Time-point	Safety
Table14.3.6.3.a	ECG parameter (RR Interval) and Change from Baseline (Inoculum) by challenge agents and Scheduled Assessment Time-point	Safety
Table14.3.6.3.b	ECG parameter (RR Interval) and Change from Baseline (Artesunate) by challenge agents and Scheduled Assessment Time-point	Safety
Table14.3.6.4a	ECG parameter (QRS Interval) and Change from Baseline (Inoculum) by challenge agents and Scheduled Assessment Time-point	Safety
Table14.3.6.4b	ECG parameter (QRS Interval) and Change from Baseline (Artesunate) by challenge agents and Scheduled Assessment Time-point	Safety
Table14.3.6.5a	ECG parameter (QT Interval) and Change from Baseline (Inoculum) by challenge agents and Scheduled Assessment Time-point	Safety
Table14.3.6.5b	ECG parameter (QT Interval) and Change from Baseline (Artesunate) by challenge agents and Scheduled Assessment Time-point	Safety
Table14.3.6.5c	ECG parameter (QT Interval) and Change from Baseline (Piperaquine Phosphate) by challenge agents and Scheduled Assessment Time-point	Safety
Table14.3.6.6a	ECG parameter (QTcF Interval) and Change from Baseline (Inoculum) by challenge agents and Scheduled Assessment Time-point	Safety
Table14.3.6.6b	ECG parameter (QTcF Interval) and Change from Baseline (Artesunate) by challenge agents and Scheduled Assessment Time-point	Safety
Table14.3.6.6c	ECG parameter (QTcF Interval) and Change from Baseline (Piperaquine Phosphate) by challenge agents and Scheduled Assessment Time-point	Safety
Table14.3.6.7a	Maximum Post-dose ECG QTcF Interval Out of Range Values and Changes from Baseline (Inoculum)	Safety

Table Number	Table Title	Population
Table14.3.6.7b	Maximum Post-dose ECG QTcF Interval Out of Range Values and Changes from Baseline (Artesunate)	Safety
Table14.3.6.7c	Maximum Post-dose ECG QTcF Interval Out of Range Values and Changes from Baseline (Piperaquine Phosphate)	Safety

16.1.10 Documentation of inter-laboratory standardisation methods and laboratory QA procedures

Method development and validation for Artesunate and Dihydro Artemisinin (DHA)

**Study title**	Development and validation of a method for the determination of Artesunate and Dihydro Artemisinin (DHA), in human plasma samples by LC-MS/MS
**Study no**	JIRA # PROJ-279
**Study facility**	Analytical Chemistry Unit (HPLC, LC MS/MS) RBWH Central Laboratory Pathology QLD
**Sponsor**	Prof James McCarthy, Queensland Institute of Medical Research (QIMR)
**Staff involved**	Dr Kobus Ungerer, Director of Chemical Pathology<br>Brett McWhinney, Supervising Scientist, HPLC section<br>Leanne Foyn, Scientist, Analytical Chemistry Unit
**Starting date**	February 2017
**Completion date**	April 2017

Table of Contents

1. Study synopsis....................**3**

1.1 Schedule....................4

2. Introduction....................**4**

3. Reagents and materials....................**4**

3.1 Reference items....................4

3.2 Internal standard....................5

3.3 Blank plasma....................5

3.4 Chemicals and equipment....................5

4. Analytical method....................**6**

4.1 Preparation of calibration and quality control sample....................6

4.2 Preparation of the internal standard....................7

4.3 Storage conditions....................7

4.4 Extraction method....................7

4.5 HPLC-MS/MS conditions....................7

4.6 Protein precipitation solution evaluation.................... 9

4.7 References....................9

5. Chromatograms....................**10**

6. Certificates of Analysis....................**14**

6.1 Artesunate certificate.................... 14

6.2 Dihydro Artemisinin certificate.................... 15

6.3 Artesunate-d4 certificate.................... 16

6.4 Dihydro Artemisinin-d3 certificate.................... 17

7. Data....................**18**

7.1Precision....................18

7.2 Internal standard, Artesunate and DHA post-extract stability....................20

7.3 QC short term and freeze-thaw cycle stability....................22

7.4 Carry-over study....................22

7.5 Extraction comparison and recovery....................23

7.6 Linearity....................23

8. Recommendation....................**25**

Sign off page....................**25**

1. Study synopsis

Sponsor	Dept of Chemical Pathology, Pathology Queensland, Level 3, Block 7, RBWH
Reference item(s)	Artesunate and Dihydro Artemisinin
Internal standard(s)	Artesunate-d4 and Dihydro Artemisinin-d3
SBQ study no	
Study title	Method development and validation for Artesunate and Dihydro Artemisinin (DHA)
Specimens	spiked samples
Species	human
Biological matrix	plasma
Sensitivity of the assay	1 to 1000 µg/L for Artesunate and Dihydro Artemisinin for 5 µL injection
Concentration of the ISTD	200 µg/L of both Artesunate-d4 and Dihydro Artemisinin-d3
Intra-run Precision (CV)	5.2% to 7.5% (n=20) for Artesunate over 3 concentration levels<br>5.2% to 8.5% (n=20) for DHA over 3 concentration levels
Inter-run Precision (CV)	7.3% to 10.9% (n=7) for Artesunate over 3 concentration levels<br>3.8% to 9.3% (n=7) for DHA over 3 concentration levels
Accuracy	98.4% to 104.8% (n=5) for Artesunate<br>98.2% to 105.1% (n=5) for DHA
Post-preparative stability	stable for at least 24 hours at 10±3°C
Freeze-and-thaw stability	stable after at least three freeze and thaw cycles
Carry-over	<0.01 % for Artesunate after the highest calibration sample<br>0.01% for DHA after the highest calibration sample
Recovery of the analyte	
Overall-recovery of the ISTD	
Specificity	no observed interference

1.1 Schedule

Experimental starting date	February 2017
Experimental completion date	April 2017

2. Introduction

The purpose of this study was the validation of a method for the quantification of Artesunate and Dihydroartemisinin (DHA) in human plasma samples by LC-MS/MS. The heated electrospray interface (ESI) was used as ion source in positive ion mode. Artesunate, DHA and the internal standards Artesunate-d4 and DHA-d3 were measured in selected reaction monitoring mode. The plasma samples were precipitated with three volume equivalents of acetonitrile containing the internal standard artesunate-d4 and DHA-d3. After protein precipitation, the samples were vortexed then centrifuged, and the 96 well plate was transferred to the Autosampler. An aliquot of 5 µL of the sample was injected onto the UPLC system.

The following calibration range was applied:

1.00 to 1000 µg/L of Artesunate and DHA in human plasma

As part of this validation, the following parameters were investigated:

- Linearity and regression model
- Accuracy and precision
- Specificity of the method for the analyte and the internal standard
- Specificity of the method for the analyte and the internal standard in different protein precipitation solutions
- Stability of the analytes in extracted samples
- Overall-recovery of the internal standard
- Carry-over of the method

3. REAGENTS AND MATERIALS

3.1 Reference items

**Name**	**Artesunate**
Batch	2-ARP-152-1
Supplier	Toronto Research Chemical Inc. (2 Brisbane Road, Toronto, ON, Canada)
Storage	at -20°C Freezer.
Retest	May, 2018
Assay purity	98%
Molecular formula, base	$C_{19}H_{28}O_8$
Molecular weight, base	384.42
CoA date	24-05-2013

**Name**	**Dihydroartemisinin**
Batch	4-EOD-163-1
Supplier	Toronto Research Chemical Inc. (2 Brisbane Road, Toronto, ON, Canada)
Storage	at -20°C Freezer
Retest	October, 2021
Assay purity	95%
Molecular formula, base	$C_{15}H_{24}O_5$
Molecular weight, base	284.35
CoA date	05-10-2016

3.2 Internal standard

**Name**	**Artesunate-d4**
Batch	6-DHL-62-4
Supplier	Toronto Research Chemical Inc. (2 Brisbane Road, Toronto, ON, Canada)
Storage	at -20°C
Retest	February, 2022
Purity	97%
Molecular formula, base	$C_{19}H_{24}D_4O_8$
Molecular weight, base	388.45
CoA	03-02-2017

**Name**	**d3-Dihydroartemisinin**
Batch	25-GHZ-173-1
Supplier	Toronto Research Chemical Inc. (2 Brisbane Road, Toronto, ON, Canada)
Storage	at -20°C, Under Inert Atmosphere
Retest	October, 2017
Purity	98%
Molecular formula, base	$C_{15}H_{21}D_3O_5$
Molecular weight, base	287.37
CoA	10-10-2014

The characterization of the reference items was performed if not otherwise stated according to the quality standards mentioned in the certificate of analysis.

3.3 Blank plasma

The following source was used for the preparation of spiked samples:

Human plasma

3.4 Chemicals and equipment

All chemicals and equipment used during the analysis of the study samples were controlled or validated according to internal SOPs, unless otherwise stated.

Chemicals

Methanol	LiChromSolv	Merck, Kilsyth Victoria, Australia
Acetonitrile	LiChromSolv	Merck, Kilsyth Victoria, Australia
Water 18 mega ohm		SG Water Nashua, NH, USA
Formic acid	Sigma Aldrich	Sydney NSW, Australia

Apparatus

Micro balance	MT-5	Mettler-Toledo GmbH, Germany
Pipette	Research and Reference	Eppendorf AG, Hamburg, Germany
Centrifuge	16R Hereaus	Thermo Fisher Scientific,Australia
Vortexer	TallBoy Multi-tube	Henry Troemner, USA

Instrumentation

UPLC Pump	Acquity UPLC binary pumps Waters Corporation, Milford, MA USA
Autosampler	Acquity UPLC Autosampler Waters Corporation, Milford, MA USA
Mass spectrometer	Premier XE Waters Corporation, Milford, Massachusetts, USA

Software

Chromatography	MassLynx Version 4.1	Waters Corporation, Milford, MA USA

4. ANALYTICAL METHOD

4.1 Preparation of calibration samples and quality control samples

For the preparation of CAL samples, Artesunate and DHA were dissolved in methanol to a concentration of 0.500 g/L each for the combined solution. For the preparation of QC samples, a second solution (prepared by a different analyst) with a concentration of 0.500 g/L was used. Working solutions were prepared from a mixture by serial dilutions in methanol to concentrations 20 times higher than the corresponding concentrations in matrix. The concentrations were calculated under consideration of purity where applicable. The concentrations of the CAL samples were the following:

Working Standard Range (in blank plasma):
Artesunate and DHA (µg/L)

Std 1	0
Std 2	2
Std 3	5
Std 4	20
Std 5	100
Std 6	500
Std 7	700
Std 8	1000

Quality Control (in blank plasma):

Artesunate and DHA µg/L

QC L	15
QC M	80
QC H	800

4.2 Preparation of the internal standard

For the preparation of the internal standard solution, Artesunate-d4 and DHA-d3 were dissolved in methanol to a concentration of 0.01 g/L and 0.005 g/L respectively. Further dilutions to a final concentration of 200 µg/L for both Artesunate-d4 and DHA-d3 were combined with acetonitrile.

4.3 Storage conditions

CAL and QC samples as well as stock solutions and their dilutions were stored at -25±5°C.

4.4 Extraction Method:

1. Pipette 100 µL of STD, QC or sample into 2 mL deep 96 well plate
2. Add 300 µL of IS, 200 µg/L Artesunate-d4 and DHA-d3 in Acetonitrile
3. Place mat cap on top of plate and vortex 2 min
4. Centrifuge 10 min at 3500 rpm
5. Place into Sample Organizer
6. Inject 5 µL

4.5 HPLC-MS/MS conditions

The quantification of Artesunate and DHA was performed by UPLC column separation with reversed-phase chromatography followed by detection with triple-stage quadrupole MS/MS in the selected reaction monitoring mode.

Liquid chromatography

Mobile Phase A:	2mM Ammonium Acetate in water + 0.1% Formic Acid
Mobile Phase B:	2mM Ammonium Acetate in Methanol + 0.1% Formic Acid
Column:	Acquity UPLC BEH C_{18} 1.7µm 2.1x50mm column Part # 186002350
Precolumn:	Acquity BEH C18 1.7µm VanGuard Pre-column 2.1 x 5 mm Part # 186003975
Column Temp:	45° C

Instrument Conditions:

MRM Function

COMPOUND	MRM FUNCTION	DWELL (Sec)	CONE (V)	COLLISION ENERGY (eV)
**Artesunate Quantifier**	402.2>267.3	0.02	10	15
**Artesunate Qualifier**	402.2>163.0	0.02	10	20
**DHA Quantifier**	267.3>145.0	0.02	15	15
**DHA Qualifier**	367.3>163.1	0.02	15	10

**Artensunate-d4**	406.3>267.2	0.02	10	15
**DHA-d3**	270.3>148	0.02	15	15

MS Conditions

**Source ES+**	
Capillary (kV)	0.80
Cone (V)	15
Extractor (V)	5
RF Lens (V)	0.2
Source Temperature (°C)	120
Desolvation Temperature (°C)	425
Desolvation Gas Flow (L/Hr)	900
Cone Gas Flow (L/Hr)	20
**Analyser**	
LM 1 Resolution	14.0
HM 1 Resolution	14.0
Ion Energy 1	0.2
Entrance	0
Collision	10
Exit	0.0
LM 2 Resolution	14.0
HM 2 Resolution	13.0
Ion Energy 2	0.6
Multiplier	800
Collision Cell Gas flow (mL/min)	0.30

UPLC Conditions

Sample Manager

General	
Sample Loop Option	Partial loop with needle overfill
Wash Solvents	Weak 1000μL
	Strong 500μL
Loop Offline	Enable
Temperature	Column 45.0 ± 2.0°C
	Sample 10.0 ± 2.0°C
Run Time	2.0 minutes
Needle depth	6.0 mm

Gradient Table

TIME (mins)	FLOW RATE (mL/min)	%A	%B	CURVE
Initial	0.400	20	80	Initial
1.00	0.400	12	88	6
1.01	0.400	2	98	1
1.75	0.400	20	80	11

4.6 Protein precipitation solution evaluation

A 96 well plate was used to assess protein precipitation solutions using 100% Methanol, 100% Acetonitrile and 0.1% Formic acid in Acetonitrile.

Each protein precipitation solution was used to extract a set of standards for the assay; the internal standard peak's area was then used to evaluate which protein precipitation solution was most effective in yielding the greatest recovery.

The following procedure was followed.

1. 100 µL of sample was added into the wells
2. 300 µL of IS was then added in the wells
3. The plate was capped and vortexed at maximum speed for 2 minutes
4. The plate was then centrifuged at 3500rpm for 10 minutes after which it was placed in the Autosampler.

Observations

After observing the internal standard area recovery of the three solutions, it was decided that 100% Acetonitrile had the best recovery and was therefore selected for the progression of the assay development.

4.7 References

1. Development and validation of a liquid chromatography and ion spray tandem mass spectrometry method for the quantification of artesunate, artemether and their major metabolites dihydroartemisinin and dihydroartemisinin-glucuronide in sheep plasma. Duthaler U, Keiser J, Huwyler J.

   J. Mass. Spectrom. 2011, 46, 172-181. DOI 10.1002. Epub: 2017 Feb 23

2. A simplified liquid chromatography-mass spectrometry assay for artesunate and dihydroartemisinin, its metabolite, in human plasma. Teja-Isavadharm P, Siriyanonda D, Siripokasupkul R, Apinan R, Chanarat N, Lim A, Wannaying S, Saunders D, Fukuda M, Miller R, Weina P, Melendez V.

   Molecules, 2010, 15, 8747-8768. DOI 10.3390. Epub 2017 Jan 23

5. Chromatograms

Below are the chromatograms for both Artesunate and DHA for the following sample types; Blank plasma with acetonitrile, blank plasma with internal standard, the low level of QC material and the highest standard for both assays.

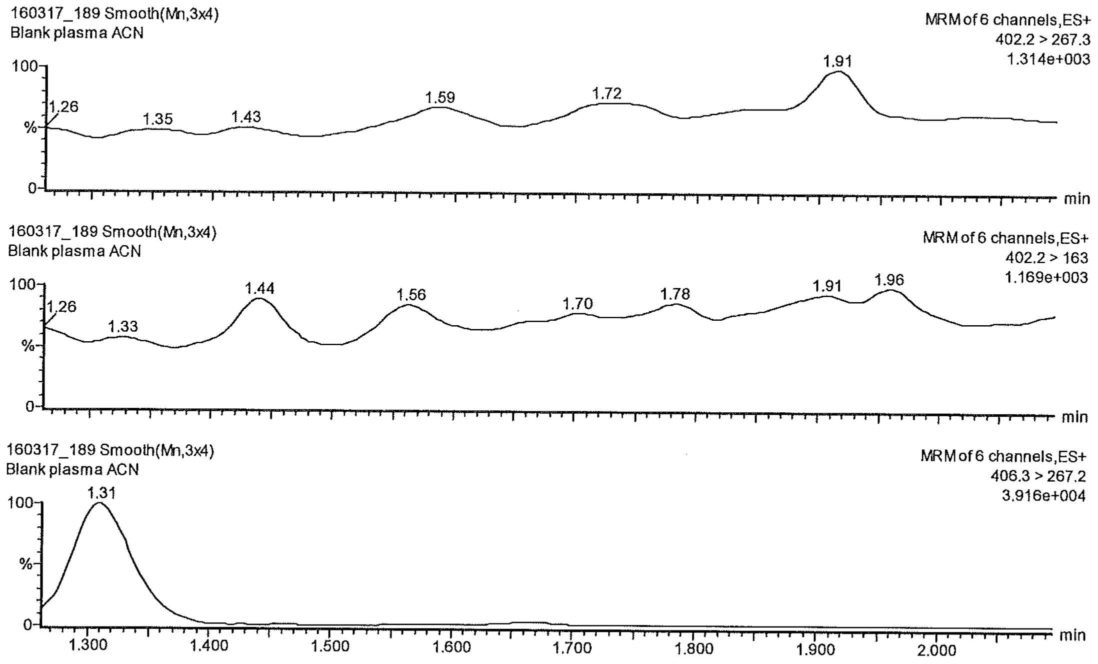

Figure 1: showing the chromatogram, primary and secondary channels as well as the internal standard peak, for Artesunate in a blank plasma sample with only 100% ACN added.

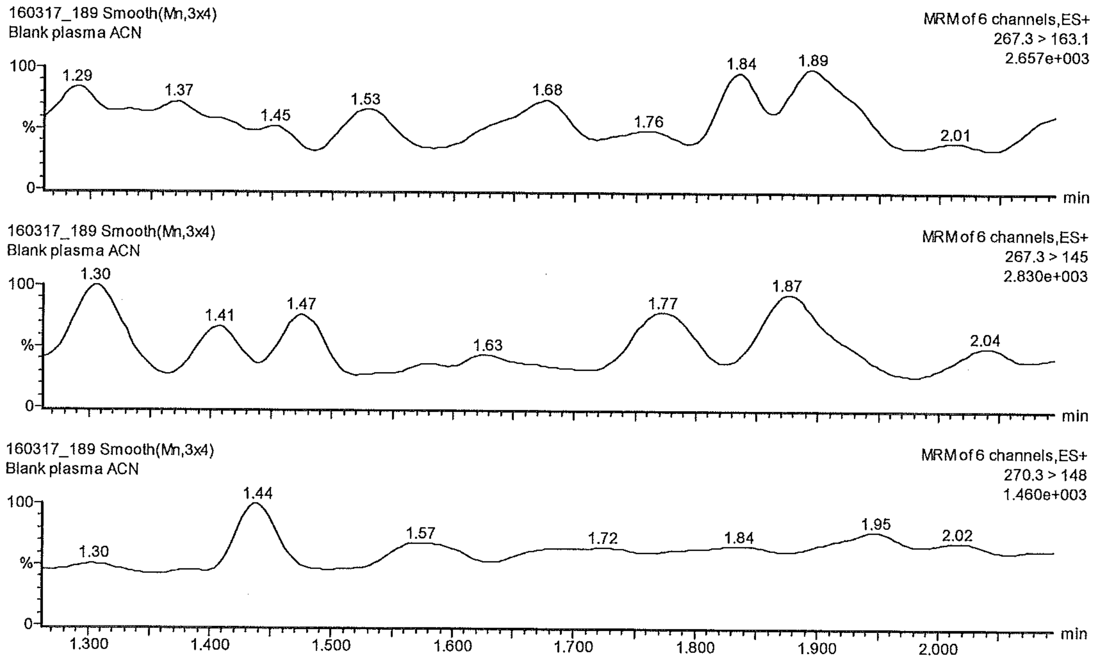

Figure 2: showing the chromatogram, primary and secondary channels as well as the internal standard peak, for DHA in a blank plasma sample with only 100% ACN added.

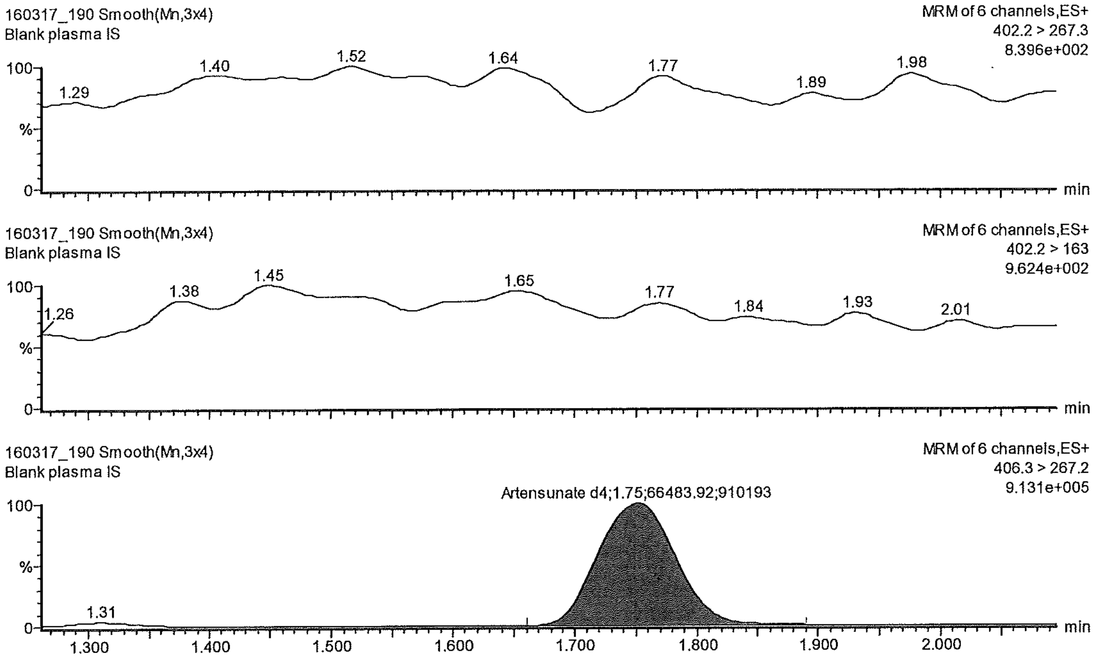

Figure 3: showing the chromatogram, primary and secondary channels as well as the internal standard peak, for Artesunate in a blank plasma sample with internal standard added.

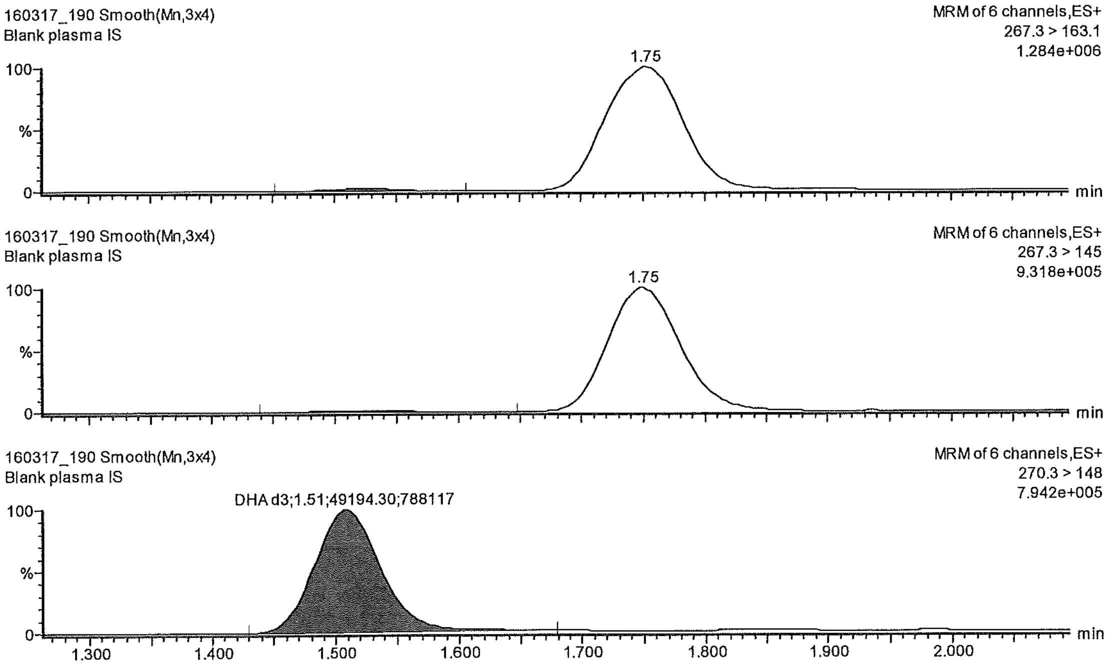

Figure 4: showing the chromatogram, primary and secondary channels as well as the internal standard peak, for DHA in a blank plasma sample with internal standard added.

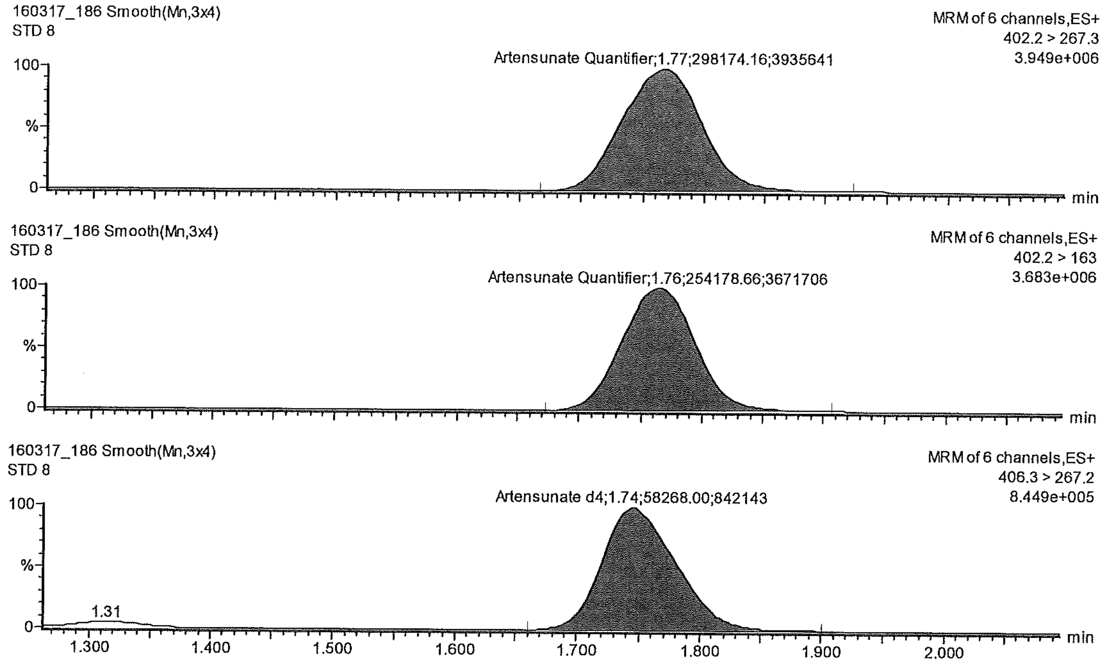

Figure 5: showing the chromatogram, primary and secondary channels as well as the internal standard peak, for Artesunate in an extracted Standard 8 sample with internal standard added.

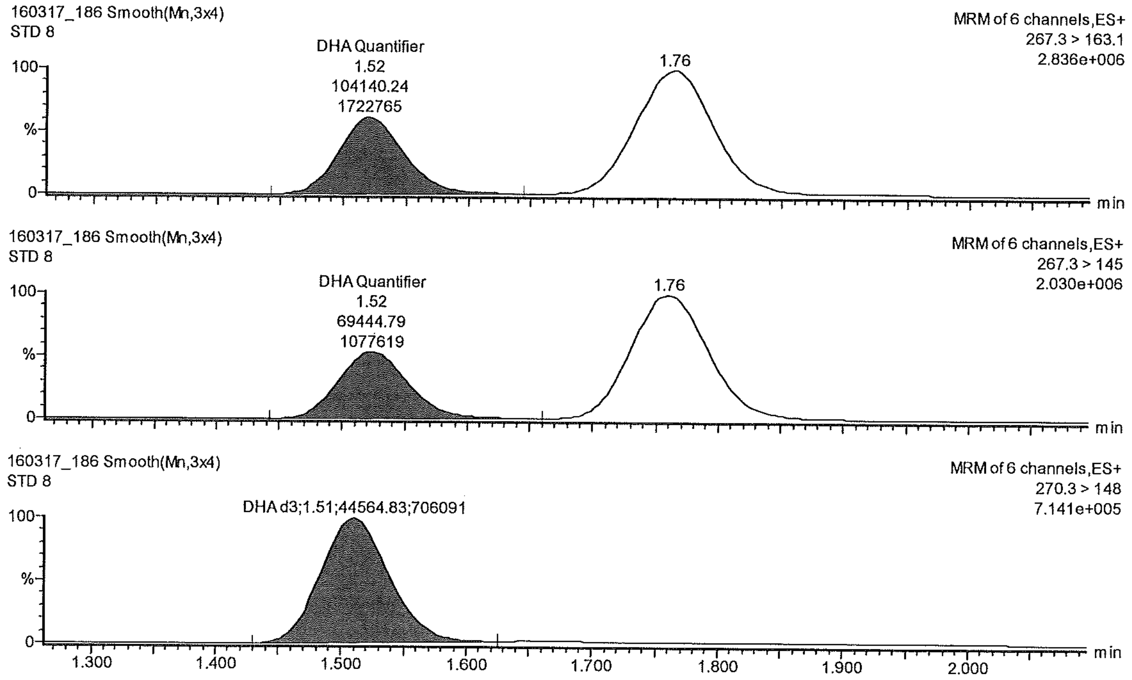

Figure 6: showing the chromatogram, primary and secondary channels as well as the internal standard peak, for DHA in an extracted Standard 8 sample with internal standard added.

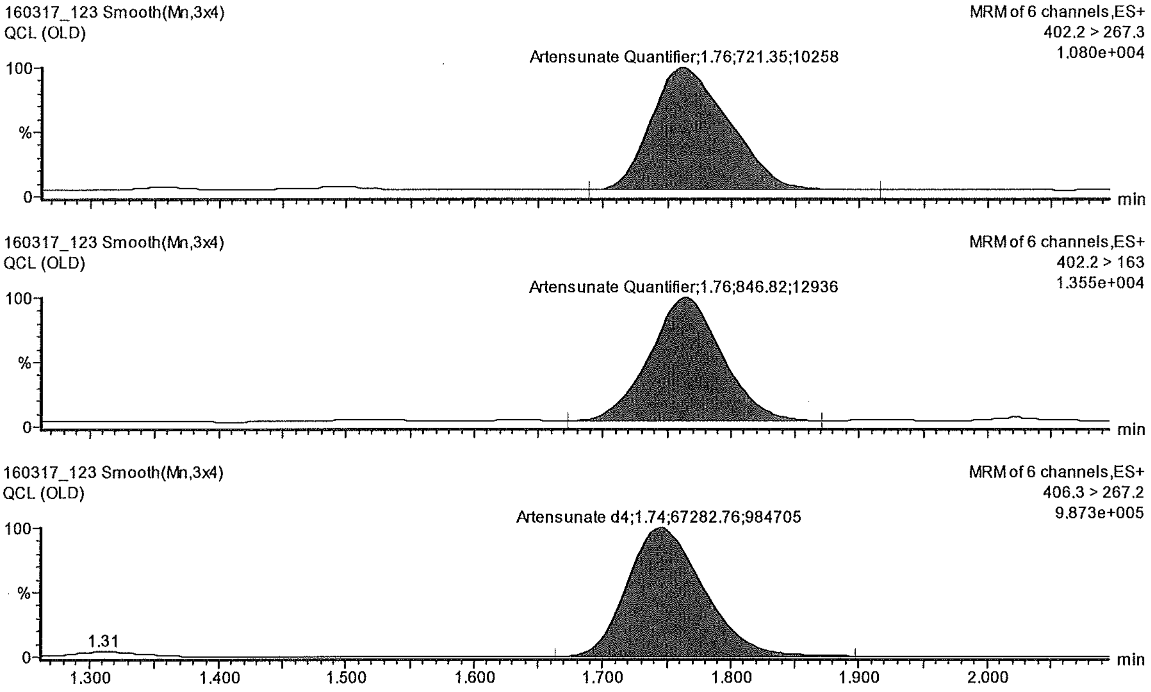

Figure 7: showing the chromatogram, primary and secondary channels as well as the internal standard peak, for Artesunate in an extracted QC 1 sample with internal standard added.

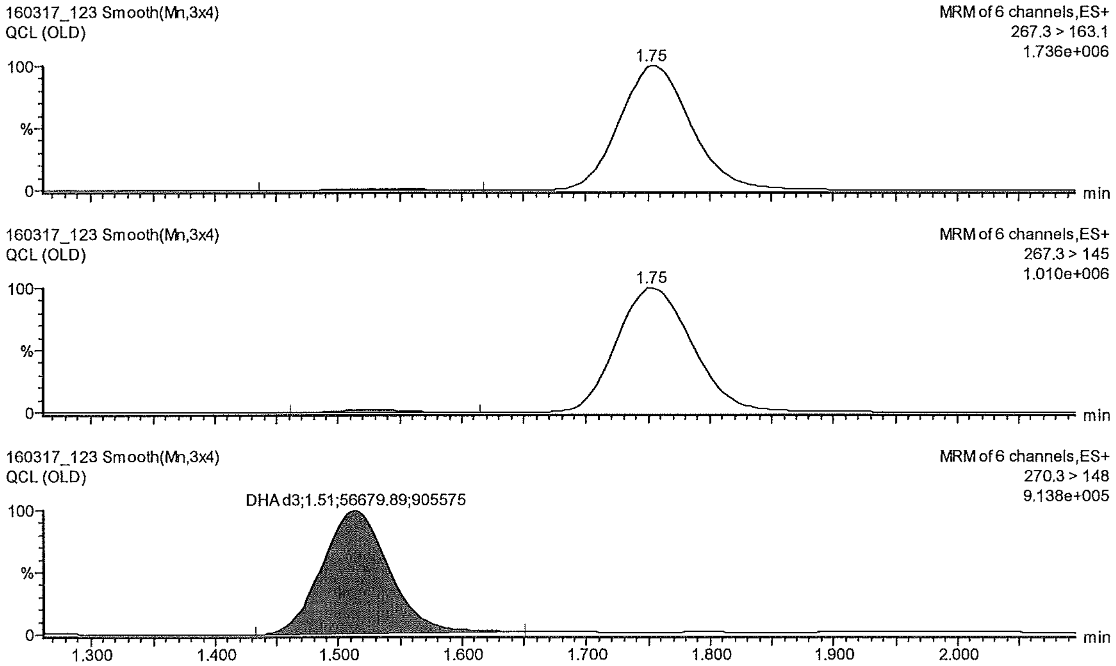

Figure 8: showing the chromatogram, primary and secondary channels as well as the internal standard peak, for DHA in an extracted QC 1 sample with internal standard added.

6. Certificates of Analysis

6.1 Artesunate certificate

Toronto Research Chemicals
products for innovative research

CERTIFICATE OF ANALYSIS

2 Brisbane Road, Toronto, ON. M3J 2J8 Canada Tel: (416) 665-9696 Fax: (416) 665-4439 E-mail: orders@trc-canada.com Website: www.trc-canada.com

1. Identification

CAS Number:
88495-63-0

Catalogue Number:
A777800

Product:
Artesunate

Synonyms:
Butanedioic Acid Mono(3R,5aS,6R,8aS,9R,10R,12R,12aR)-decahydro-3,6,9-trimethyl-3,12-epoxy-12H-pyrano[4,3-j]-1,2-benzodioxepin-10-yl] Ester; Artesunic Acid;

Structure:

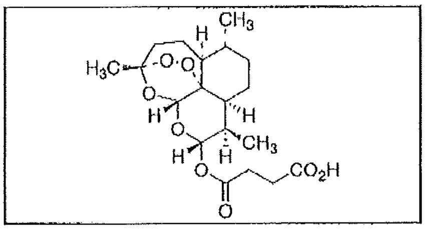

Molecular Formula:
$C_{19}H_{28}O_8$

Molecular Weight:
384.42

Source of Product:
N/A

2. Analytical Information

Lot Number:
2-ARP-152-1

Melting Point:
136 - 138°C

Boiling Point:
N/A

Atmosphere:
Air

Appearance of Product:
White Solid

Solubility
DMSO, Methanol

Method for Determining Identity:
^{1}H NMR (DMSO-d_6) and MS

Stability
Not Detemined

Purity:
98%

Long Term Storage Condition:
-20°C Freezer

Additional Information:
TLC Conditions: SiO_2: Dichloromethane : Methanol = 6 : 1; Visualized with AMCS; Single Spot, Rf = 0.7.
^{1}H NMR and MS conform to structure.

Philip Chan, Head of Quality Assurance

QC Test Date
May 24, 2013

Retest Date
May 22, 2018

PM Separations
Ph +61 7 3390 1096
customerservice@pmsep.com.au
www.pmsep.com.au

6.2 Dihydro Artemisinin certificate

Toronto Research Chemicals
products for innovative research

CERTIFICATE OF ANALYSIS

2 Brisbane Road, Toronto, ON. M3J 2J8 Canada Tel: (416) 665-9696 Fax: (416) 665-4439 E-mail: orders@trc-canada.com Website: www.trc-canada.com

1. Identification

CAS Number:
71939-50-9

Catalogue Number:
D448360

Product:
Dihydro Artemisinin

Synonyms:
(3R,5aS,6R,8aS,9R,10S,12R,12aR)-Decahydro-3,6,9-trimethyl- 3,12-epoxy-12H-pyrano[4,3-j]-1,2-benzodioxepin-10-ol;β-Dihydroartemisinin; Alaxin; Cotecxin; Cotexin; DHQHS 2; Dihydroartemisinine; Dihydroqinghaosu; USP Artemether Related Compound A

Structure:

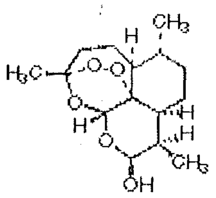

Molecular Formula:
$C_{15}H_{24}O_5$

Molecular Weight:
284.35

Source of Product:
N/A

2. Analytical Information

Lot Number:
4-EOD-163-1

Melting Point:
143 - 145°C

Boiling Point:
N/A

Atmosphere:
Air

Appearance of Product:
Off-White Solid

Solubility
Chloroform (Slightly), Methanol (Slightly)

Method for Determining Identity:
^1H NMR ($CDCl_3$) and MS

Stability
Not Determined

Purity:
95%

Long Term Storage Condition:
-20°C Freezer

Additional Information:
TLC Conditions: SiO_2; Dichloromethane : Methanol = 9 : 1; Visualized with $KMnO_4$; Single Spot, Rf = 0.40.
^1H NMR and MS conform to structure.

Philip Chan, Head of Quality Assurance

QC Test Date
October 5, 2016

Retest Date
October 3, 2021

PM Separations
Ph. +61 7 3390 1096
customerservice@pmsep.com.au
www.pmsep.com.au

6.3 Artesunate-d4 certificate

Toronto Research Chemicals
products for innovative research

CERTIFICATE OF ANALYSIS

2 Brisbane Road, Toronto, ON. M3J 2J8 Canada Tel: (416) 665-9696 Fax: (416) 665-4439 E-mail: orders@trc-canada.com Website: www.trc-canada.com

1. Identification

CAS Number:
1316753-15-7

Catalogue Number:
A777803

PM Separations
Ph: +61 7 3390 1096
customerservice@pmsep.com.au
www.pmsep.com.au

Product:
Artesunate-d4

Synonyms:
Butanedioic Acid Mono(3R,5aS,6R,8aS,9R,10R,12R,12aR)-decahydro-3,6,9-trimethyl-3,12-epoxy-12H-pyrano[4,3-j]-1,2-benzodioxepin-10-yl] Ester-d4; Artesunic Acid-d4;

Structure:

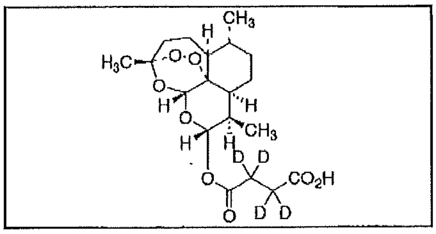

Molecular Formula:
$C_{19}H_{24}D_4O_8$

Molecular Weight:
388.45

Source of Product:
Synthetic

2. Analytical Information

Lot Number:
6-DHL-62-4

Melting Point:
130 - 132°C

Boiling Point:
N/A

Atmosphere:
Air

Appearance of Product:
White Solid

Solubility
Chloroform (Slightly), Methanol (Slightly)

Method for Determining Identity:
^{1}H NMR ($CDCl_3$) and MS

Stability
Not Determined

Purity:
Chemical Purity: 97%
Isotopic Purity: 97.9%

Long Term Storage Condition:
-20°C Freezer

Additional Information:
TLC Conditions: SiO_2; Dichloromethane : Methanol = 9 : 1; Visualized with UV and AMCS; Single Spot, Rf = 0.60.
^{1}H NMR and MS conform to structure.
Elemental Analysis: (Found) %C: 57.96, %H: 7.13; (Calculated) %C: 58.75, %H: 7.27
Normalized Intensity: d_0 = 0.27%, d_1 = 0.45%, d_2 = 1.08%, d_3 = 3.96%, d_4 = 94.24%

Philip Chan, Head of Quality Assurance

QC Test Date
February 3, 2017

Retest Date
February 1, 2022

6.4 Dihydro Artemisinin-d3 certificate

Toronto Research Chemicals
products for innovative research

CERTIFICATE OF ANALYSIS

2 Brisbane Road, Toronto, ON. M3J 2J8 Canada Tel: (416) 665-9696 Fax: (416) 665-4439 E-mail: orders@trc-canada.com Website: www.trc-canada.com

1. Identification

CAS Number:
176774-98-4

Catalogue Number:
D448362

Product:
Dihydro Artemisinin-d3

Synonyms:
(3R,5aS,6R,8aS,9R,10S,12R,12aR)-Decahydro-3,6,9-trimethyl- 3,12-epoxy-12H-pyrano[4,3-j]-1,2-benzodioxepin-10-ol-d3; β-Dihydroartemisinin-d3; Alaxin-d3; Cotecxin-d3; Cotexin-d3; DHQHS 2-d3; Dihydroartemisinine-d3; Dihydroqinghaosu-d3;

Structure:

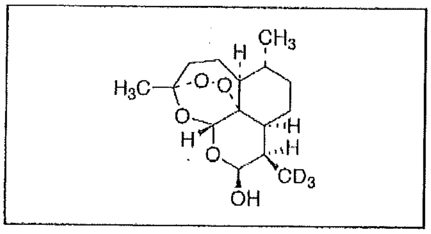

Molecular Formula:
$C_{15}H_{21}D_3O_5$

Molecular Weight:
287.37

Source of Product:
Synthetic

2. Analytical Information

Lot Number:
25-GHZ-173-1

Melting Point:
139.5 - 142.0°C

Boiling Point:
N/A

Atmosphere:
Inert Gas

Appearance of Product:
White Solid

Solubility
Chloroform

Method for Determining Identity:
^{1}H NMR ($CDCl_3$) and MS

Stability
Not Determined

Purity:
Chemical Purity: 98%
Isotopic Purity: 98.0%

Long Term Storage Condition:
-20°C Freezer, Under Inert Atmosphere

Additional Information:
TLC Conditions: SiO_2; Dichloromethane : Methanol = 9 : 1; Visualized with AMCS and $KMnO_4$; Single Spot, Rf = 0.60.
^{1}H NMR and MS conform to structure.
Elemental Analysis: (Found) %C: 62.47, %H: 8.95; (Calculated) %C: 62.69, %H: 8.42
Normalized Intensity: d_0 = 0.04%, d_1 = 0.00%, d_2 = 5.80%, d_3 = 94.16%
Specific Rotation: +145.1° (c = 0.20, Chloroform)

Philip Chan, Head of Quality Assurance

QC Test Date
October 10, 2014

Retest Date
October 8, 2017

PM Separations
Ph: +61 7 3390 1096
customerservice@pmsep.com.au
[illegible]

7. DATA

7.1 Precision

Intra-run precision was determined by extracting 20 sets of QC 1, QC 2 and QC 3 samples and subsequently calculating the mean and coefficient of variation for both Artesunate and DHA. Inter-run precision was established by averaging the concentrations acquired for QC 1, QC 2 and QC 3 over multiple extractions on various days.

**Artesunate Primary channel (nmol/L)**				
**Intra Run Precision**		Date : 09/03/2017 – 30/03/2017		
**QC ID**	**n**	**Mean**	**SD**	**%CV**
QC 1	20	14.25	1.07	7.5
QC 2	20	76.00	5.16	6.8
QC 3	20	779.98	43.66	5.6

**Artesunate Secondary channel (nmol/L)**				
**Intra Run Precision**		Date : 09/03/2017 – 30/03/2017		
**QC ID**	**n**	**Mean**	**SD**	**%CV**
QC 1	20	13.59	1.16	8.5
QC 2	20	72.40	5.18	7.2
QC 3	20	732.11	40.28	5.5

**Inter Run Precision** (Artesunate)		Date : 09/03/2017 - 30/03/2017		
**QC ID**	**n**	**Mean**	**SD**	**%CV**
QC 1	7	15.41	1.68	10.9
QC 2	7	67.94	7.31	10.8
QC 3	7	811.80	58.97	7.3

DHA Primary channel (nmol/L)				
**Intra Run**		Date : 09/03/2017 – 30/03/2017		
**QC ID**	**n**	**Mean**	**SD**	**%CV**
QC 1	20	14.78	1.25	8.5
QC 2	20	84.57	6.13	7.2
QC 3	20	853.39	50.70	5.9

DHA Secondary channel (nmol/L)				
**Intra Run**		Date : 09/03/2017 – 30/03/2017		
**QC ID**	**n**	**Mean**	**SD**	**%CV**
QC 1	20	14.72	1.15	7.8
QC 2	20	83.56	6.32	7.6
QC 3	20	813.58	42.05	5.2

**Inter Run Precision** (DHA)		Date : 09/03/2017- 30/03/2017		
**QC ID**	**n**	**Mean**	**SD**	**%CV**
QC 1	7	16.70	1.55	9.3
QC 2	7	87.70	7.32	8.3
QC 3	7	815.00	31.02	3.8

7.2 Internal standard, Artesunate and DHA Post-Extract Stability

Post extraction stability data was obtained by extracting 10 sets of QC 1, QC 2 and QC 3, injecting the samples, and then allowing the plate to sit in the sample manager at 10°C. Following 12 hours, the samples were re-injected. The deviation percentage was then calculated from both sets of data for Artesunate and DHA.

Artesunate Extract Stability over 12hr (nmol/L)			
**Sample ID**	**Injection 1**	**Injection 2 (12 hr post)**	**% Deviation**
Sample 1	11.9	14.9	-25.8
Sample 2	16.7	13.6	18.8
Sample 3	15.8	14.3	9.5
Sample 4	14.4	9.8	32.2
Sample 5	14.6	14.8	-1
Sample 6	13.8	14.6	-5.4
Sample 7	12.9	16.4	-27.5
Sample 8	11.2	11	2
Sample 9	12	12.2	-1.5
Sample 10	12	11.9	0.3
Sample 11	56.1	62.3	-11
Sample 12	74.6	63.6	14.8
Sample 13	62.9	68.8	-9.5
Sample 14	67.9	65.3	3.9
Sample 15	60.9	64.1	-5.3
Sample 16	69.7	55	21.2
Sample 17	58.3	69.4	-19
Sample 18	78	75.1	3.7
Sample 19	65.9	61.5	6.8
Sample 20	57.7	64.7	-12
Sample 21	813.8	729.4	10.4
Sample 22	822.9	701.2	14.8
Sample 23	612.1	725.6	-18.5
Sample 24	755.5	772.9	-2.3
Sample 25	670	650.4	2.9
Sample 26	803.6	716.7	10.8
Sample 27	920.9	678.3	26.3
Sample 28	781	710.6	9
Sample 29	782.2	741.6	5.2
Sample 30	657.6	804.5	-22.3
**Average**			**1**

DHA Extract Stability over 12hr (nmol/L) Primary channel			
**Sample ID**	**Injection 1**	**Injection 2 (12 hr post)**	**% Deviation**
Sample 1	14.6	19.8	-35.4
Sample 2	13.2	19.4	-47.5
Sample 3	13.2	23.5	-78.0
Sample 4	13.2	22.7	-71.8
Sample 5	18.9	25.5	-35.0
Sample 6	19.4	21.7	-12.2
Sample 7	15.9	21.7	-36.4
Sample 8	19.2	17.8	7.4
Sample 9	16.4	13.0	20.4
Sample 10	17.1	26.8	-57.0
Sample 11	93.7	88.4	5.6
Sample 12	100.4	116.6	-16.2
Sample 13	100.4	93.3	7.0
Sample 14	87.5	91.0	-4.0
Sample 15	102.3	88.1	13.8
Sample 16	70.0	79.5	-13.6
Sample 17	101.0	98.9	2.1
Sample 18	100.6	80.5	20.0
Sample 19	105.9	83.7	20.9
Sample 20	91.5	101.2	-10.6
Sample 21	858.3	911.5	-6.2
Sample 22	947.5	1023.8	-8.1
Sample 23	949.9	1021.4	-7.5
Sample 24	874.5	967.8	-10.7
Sample 25	803.0	1025.0	-27.6
Sample 26	849.3	911.7	-7.3
Sample 27	893.4	919.4	-2.9
Sample 28	957.9	915.2	4.5
Sample 29	844.0	909.7	-7.8
Sample 30	957.0	1013.2	-5.9
**Average**			**-13.3**

7.3 Short term and Freeze-Thaw stability

The stability of QC1, QC 2 and QC 3, for both Artesunate and DHA, was established by thawing and freezing 3 sets of the samples in cycles. Therefore set 1 was thawed once then extracted, set 2 was thawed twice then extracted and set 3 was thawed three times then extracted. The deviation from the original results was then calculated.

ART QC Stability over 3 freeze-thaw cycles (nmol/L)					
**Sample ID**	**Thaw 1**	**Thaw 2**	**% Deviation (Thaw 1 to Thaw 2)**	**Thaw 3**	**% Deviation (Thaw 1 to Thaw 3)**
QC 1	15.66	14.3	-8.7	16.08	2.7
QC 2	74.75	79.89	6.9	78.94	5.6
QC 3	824.47	819.81	-1.0	803.13	-3.0
**Average**			**-0.9**		**1.8**
**DHA QC Stability over 3 freeze-thaw cycles (nmol/L)**					
**Sample ID**	**Thaw 1**	**Thaw 2**	**% Deviation (Thaw 1 to Thaw 2)**	**Thaw 3**	**% Deviation (Thaw 1 to Thaw 3)**
QC 1	15.77	15.33	-2.8	14.67	-7.0
QC 2	80.55	75.64	-6.1	86.29	7.1
QC 3	820.89	836.85	1.9	821.53	0.1
**Average**			**-2.6**		**0.0**

The above tables demonstrate that the sets of QC for this assay are stable after three cycles of freezing and thawing, which meets acceptable criteria.

7.4 Carry-Over Study

The carry-over within the system was assessed by injecting water (n=5) after a linear standard (1000 µg/L). The area of the Artesunate and DHA peaks in the water samples were expressed as a percentage of the peaks in the concentrated sample. Carry-over for each analyte was <0.1%

Artesunate Carryover assessment (Peak Area)			
**Sample ID**	**ART Std 8 (1000 ug/L) area**	**Blank Area**	**% Recovery**
Blank 1	371302	1	0.00
Blank 2	293225	2	0.00
Blank 3	320797	1	0.00
Blank 4	283752	4	0.00
Blank 5	298174	17	0.01
**Average**	**313450**	**5**	**0.00**

DHA Carryover assessment (Peak Area)			
**Sample ID**	**DHA Std 8 (1000 ug/L) area**	**Blank Area**	**% Recovery**
Blank 1	98459	1	0.00
Blank 2	94419	4	0.00
Blank 3	96193	2	0.00
Blank 4	94895	7	0.01
Blank 5	104140	38	0.04
**Average**	**97621**	**10**	**0.01**

7.5 Extraction comparison and Recovery

Each of three protein precipitation solutions was used to extract a set of standards; the internal standard peak's area was then used to evaluate which protein precipitation solution was most effective in yielding the greatest recovery. The table below demonstrates that the protein precipitation solution made with 100% acetonitrile yields the best recovery and was therefore chosen for this assay.

Transition Sample	IS Area 100% MeOH		IS Area 100% ACN		IS Area 0.1% Formic in ACN	
	406.3>267.2 Artesunate	270.3>148.0 DHA	406.3>267.2 Artesunate	270.3>148.0 DHA	406.3>267.2 Artesunate	270.3>148.0 DHA
STD 1	12456	11092	22699	17423	19820	17114
STD 2	14998	11483	20962	17022	16925	16207
STD 3	14300	11620	22501	16457	18825	16556
STD 4	13490	11726	21525	16566	16944	17131
STD 5	14229	11552	21865	16407	17095	16615
STD 6	13839	11983	17180	15133	15379	13875
STD 7	12052	10835	16983	14935	16359	14209
STD 8	12762	11216	15701	14655	175034	13624

7.6 Linearity

The linearity of the assay was established by running the standards (n=5), with a range of 1µg/L to 1000 µg/L, over 3 weeks. The results can be found in the table below.

Linearity								
Run #	Artesunate Std Concentration (µg/L)							
	**1**	**2**	**5**	**20**	**100**	**500**	**700**	**1000**
1	1.1	1.8	5.0	19.8	104.0	525.6	621.5	1049.2
2	1.0	2.0	4.9	21.2	91.5	518.7	787.3	901.4
3	0.7	2.0	7.0	20.3	89.1	559.7	696.2	953.1
4	0.8	2.1	5.5	19.5	96.6	550.7	713.7	938.9
5	1.2	2.1	5.4	17.5	86.5	466.1	733.9	1015.5
**Mean**	0.9	2.0	5.5	19.7	93.5	524.2	710.5	971.6
**SD**	0.2	0.1	0.8	1.4	7.0	36.7	60.4	59.7
**% CV**	4.6	14.9	6.6	14.5	13.4	14.3	11.8	16.3
**Accuracy (%)**	94.2	99.7	110.8	98.3	93.5	104.8	101.5	97.2

Linearity								
Run #	DHA Std Concentration (µg/L)							
	**1**	**2**	**5**	**20**	**100**	**500**	**700**	**1000**
1	1.1	3.9	5.0	18.6	94.2	508.2	698.9	1093.3
2	0.9	2.0	5.9	19.5	92.7	544.7	697.7	964.8
3	1.1	2.0	4.9	21.0	92.2	501.5	700.3	1005.1
4	0.9	1.9	5.1	21.9	102.0	499.4	697.7	999.1
5	1.0	2.2	5.2	16.9	99.9	494.5	715.6	992.7
**Mean**	1.0	2.4	5.2	19.6	96.2	509.6	702.1	1011.0
**SD**	0.1	0.8	0.4	2.0	4.4	20.2	7.7	48.5
**% CV**	11.0	2.8	13.7	10.0	21.6	25.3	91.6	20.8
**Accuracy (%)**	100.2	119.1	104.0	97.8	96.2	101.9	100.3	101.1

8.0 RECOMMENDATION

It is recommended that this assay is implemented for clinical trial use.

Brett McWhinney

Supervising Scientist, Analytical Chemistry Unit, Chemical Pathology, Pathology QLD

Signature: [signature] **Date:** 01/08/2017

16.1.11 Publications based on study

16.1.12 Important publications referenced in the report

Not applicable

16.1.13 Reports

PK Noncompartmental Analysis: QP17C11 - *P.falciparum* K13 vs 3D7 strains treated with Artesunate

Azrin Abd Rahman

4 March 2019

Data Description:

Data was provided for artesunate and, its active metabolite, dihydroartemisinin (DHA) plasma concentrations over a period of 12 hours following administration single dose of 2 mg/kg artesunate given orally in healthy volunteers inoculated with either 3D7 (artemisinin-sensitive) or K13 (artemisinin-resistant) *P. falciparum* strains. The lower limit of quantification (LLOQ) of artesunate and DHA was 1 µg/L. The original data is located at:

L:\Lab_JamesM\2_STUD\1_CLIN_TRIALS\QP17C11_K13vs3D7_P2339\7_DATA\2_PK

Cohort 1 file names = "QP17C11_PathQLD PK sampling corhort 1 271117.xls"

Cohort 2 file names = "QP17C11_PathQLD PK sampling corhort 2 040119.xls"

Cohort 3 file names = "QP17C11_PathQLD PK sampling corhort 3 130918.xls"

Methods:

Data preparation and analysis were performed in R (version 3.4.2) and the R package IQRtools (version 0.9.1, IntiQuan GmbH, Basel, Switzerland). Plasma artesunate and DHA concentration-time data were analysed by noncompartmental methods. Maximum drug plasma concentration (C_{max}) and time of C_{max} occurred (t_{max}) were taken from observed concentration-time profiles. The terminal slope (k_{el}) was determined by non-linear regression of the terminal portion of the concentration-time profile. The elimination half-life ($t_{½}$) was calculated as 0.693/k_{el}. The area under the concentration-time curve from time 0 to the last measurable concentration ($AUC0\text{-}_{last}$) was determined using the trapezoidal method. The AUC from time 0 to infinite time ($AUC0\text{-}_{inf}$) was calculated as:

$$\mathrm{AUC}_{0-\mathrm{inf}} = \mathrm{AUC}_{0-\mathrm{last}} + \mathrm{AUC}_{\mathrm{last-inf}}$$

where $AUC_{last\text{-}inf}$ is the AUC from the last measurable concentration to infinite time and can be calculated as C_{last}/k_{el}. The apparent clearance (CL/F) was determined as Dose/$AUC_{0\text{-}inf}$ and apparent volume of distribution (Vd/F) calculated as Dose/(k_{el} x $AUC_{0\text{-}inf}$) where the artesunate dose was assumed to be totally and exclusively converted to DHA. The DHA dose was assumed to be equal to the artesunate dose on a molar basis, calculated as:

$$\text{DHA dose (in mg)} = \text{Artesunate dose (in mg)} \times \frac{284.35}{384.42}$$

where 284.35 and 384.42 are the molecular weight of DHA and artesunate, respectively. The values for $t_{½}$, CL/F, Vd/F and $AUC_{0\text{-}inf}$ were reported if the following criteria are met:

- A minimum of 3 measurable concentration-time points during the log-linear portion of the terminal elimination phase, excluding C_{max}.

- R^2 >0.80 for the regression of the log-concentration time data during the terminal elimination phase.
- Extrapolated portion of $AUC_{0\text{-}inf}$ <20% of total $AUC_{0\text{-}inf}$.

Concentrations below the LLOQ were assigned to zero. Mann-Whitney *U* test was used to compare PK parameters between subjects inoculated with 3D7 and K13 strains. Differences with $P < 0.05$ were regarded as statistically significant.

Noncompartmental Analysis Results

A total of 22 subjects were inoculated with K13 or 3D7 strains: Cohort 1 (K13, n=3; 3D7, n=3), Cohort 2 (K13, n=7; 3D7, n=3) and Cohort 3 (K13, n=3; 3D7, n=3). In subjects inoculated with 3D7 strain, two subjects received 100 mg while seven subjects were given 150 mg single dose artesunate. In those who were inoculated with K13 strain, six subjects each received 100 mg and 150 mg and only one subject was administered with 200 mg single dose artesunate.

Artesunate was rapidly hydrolysed to DHA with artesunate concentration below the LLOQ after median 4 (range: 1.5 – 10) hours post-dose. There was no difference in time to concentration below the LLOQ between doses or strains. Therefore, the terminal slope of the artesunate concentration-time profile could not be estimated in 7 subjects as there were limited samples after C_{max} with concentrations above the LLOQ. The regression line fit of artesunate and DHA for each subject are shown in Appendix 1 and 2, respectively.

Individual subject plasma concentrations of artesunate and DHA are presented in Figure 1 and 2, respectively. Dose-normalised maximum plasma artesunate concentrations ranged from 0.64 to 6.35 µg/L/mg across the 22 subjects and were reached 0.25 h (3 subjects), 0.5 h (8 subjects), 1 h (8 subjects), 1.5 h (2 subjects) and 2 h (1 subject) after dosing. Dose-normalised maximum plasma DHA concentrations ranged from 0.53 to 11.25 µg/L/mg across the 22 subjects and were reached 0.5 h (5 subjects), 1 h (8 subjects), 1.5 h (6 subjects) and 2 h (3 subjects) after dosing. PK parameters for plasma concentrations of artesunate and DHA are summarised by cohort and *P. falciparum* strains in Table 1.

The geometric mean dose-normalised artesunate and DHA concentration-time profiles of 3D7 and K13 groups were similar, with overlapping geometric standard deviations at all time-points (Figure 3 and 4, respectively). PK parameters for subjects inoculated with 3D7 were compared with those for subjects inoculated with K13, and presented in Table 2. There were no significant differences in PK parameters for artesunate and DHA between subjects inoculated with 3D7 and K13 strains.

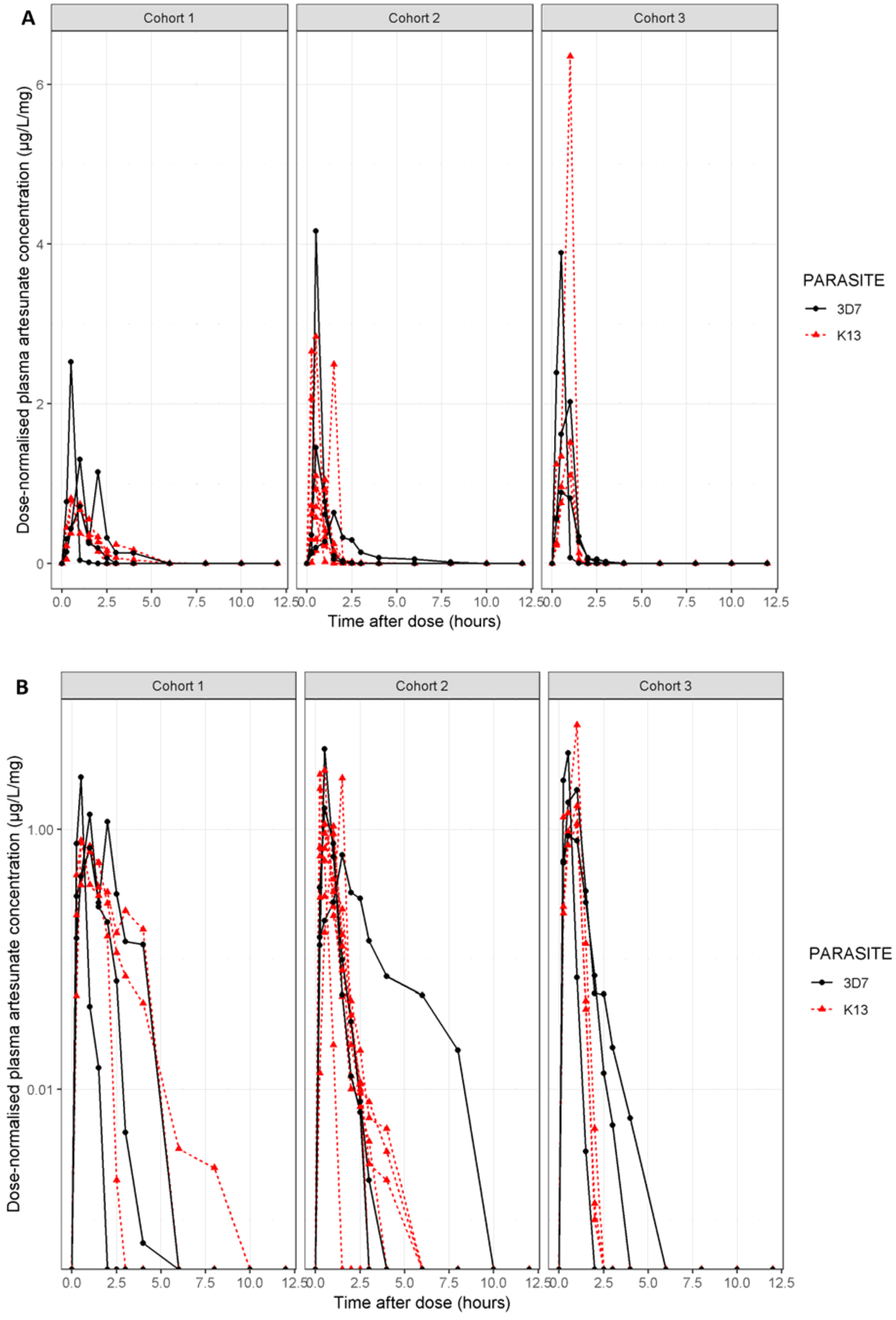

Figure 1: Individual subject dose-normalised plasma artesunate concentration-time profiles plotted on a (A) linear and (B) log-linear scale.

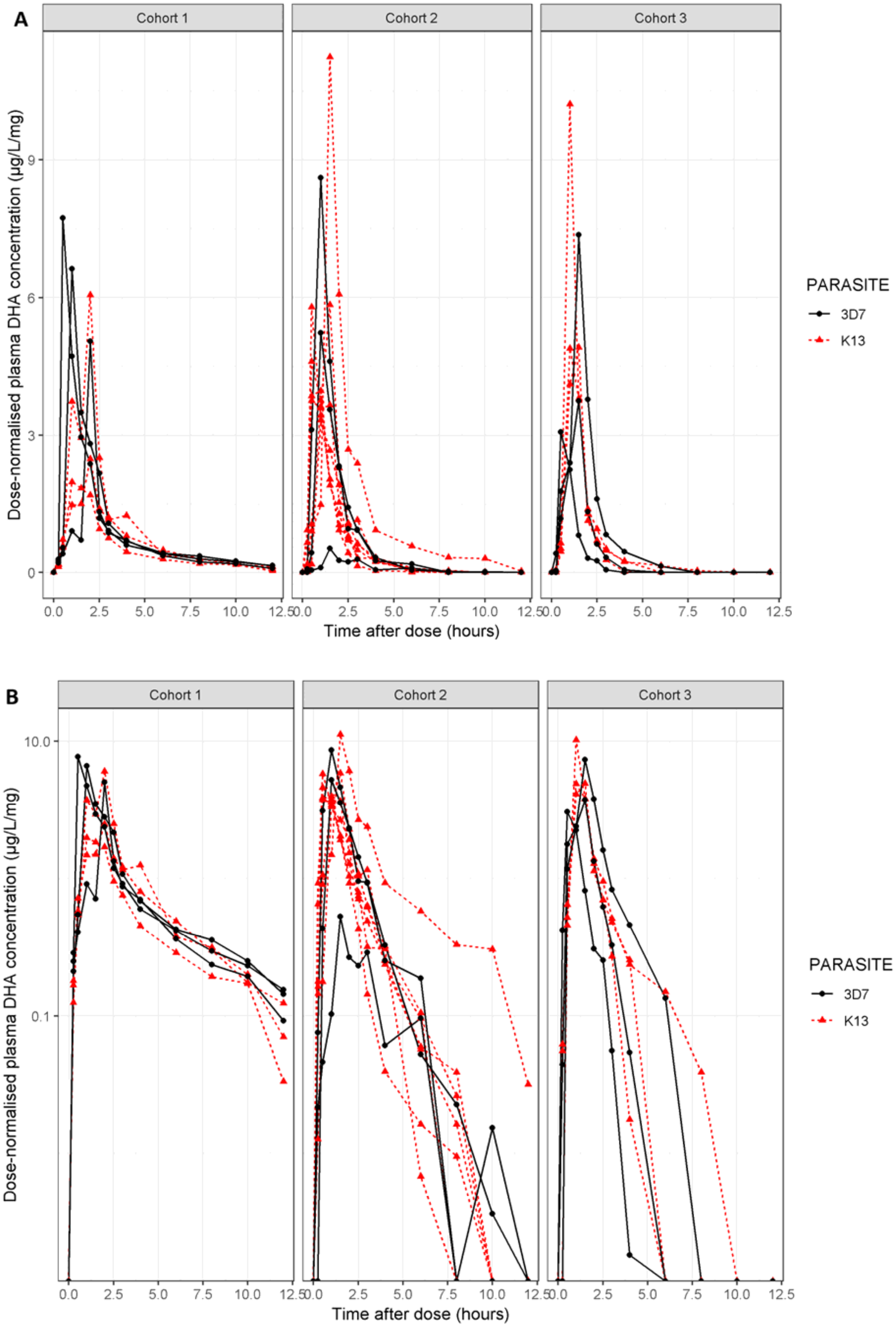

Figure 2: Individual subject dose-normalised plasma dihydroartemisinin concentration-time profiles plotted on a (A) linear and (B) log-linear scale.

Table 1: Descriptive statistics of pharmacokinetic parameters of artesunate and dihydroartemisinin by cohort and *P. falciparum* strains

PK parameters[a]	Cohort 1		Cohort 2		Cohort 3	
	3D7 (n = 3)	K13 (n = 3)	3D7 (n = 3)	K13 (n =7)	3D7 (n = 3)	K13 (n = 3)
**Artesunate**						
Dose-normalised C_{max} (µg/L/mg)	1.56 (44.3)	0.78 (4.8)	1.57 (119.5)	1.61 (61.9)	1.91 (85.2)	2.2 (117.8)
Dose-normalised $AUC_{0\text{-}inf}$ (µg·h/L/mg)	1.38 (42.4)	1.19 (51.6)	1.26 (27.5)	0.97 (32.3)	1.38 (43.9)	NA
t_{max} (h)[b]	1.0 (0.5 -2.0)	0.5 (0.5 – 1.0)	0.5 (0.5 – 1.5)	0.5 (0.25 – 1.5)	0.5 (0.5 – 1.0)	1.0 (1.0 – 1.0)
$t_{½}$ (h)	0.84 (73.9)	1.29 (63.1)	0.56 (148.0)	0.31 (16.2)	0.31 (44.1)	NA
CL/F (L/h)	726.22 (42.4)	843.1 (51.6)	791.67 (27.5)	1027.8 (32.3)	723.55 (43.9)	NA
Vd/F (L)	877.25 (25.8)	1572.89 (18.2)	642.54 (163.5)	457.96 (37.2)	322.04 (0.2)	NA
**Dihydroartemisinin**						
Dose-normalised C_{max} (µg/L/mg)	6.37 (21.8)	3.1 (64.6)	2.88 (285.6)	5.07 (42.9)	4.4 (48.3)	6.26 (44.4)
Dose-normalised $AUC_{0\text{-}inf}$ (µg·h/L/mg)	10.73 (29.2)	8.54 (36.6)	4.62 (163.8)	7.4 (37.6)	5.12 (64.5)	6.83 (25.8)
t_{max} (h)[b]	1.0 (0.5 – 2.0)	2.0 (1.0 – 2.0)	1.0 (1.0 – 1.5)	1.0 (0.5 – 1.5)	1.5 (0.5 – 1.5)	1.0 (1.0 – 1.5)
$t_{½}$ (h)	20.5 (81.2)	2.42 (16.5)	1.29 (48.5)	1.03 (35.2)	0.58 (63.1)	0.71 (97.5)
CL/F (L/h)	93.18 (29.2)	117.05 (36.6)	216.24 (163.8)	135.14 (37.6)	195.43 (64.5)	146.51 (25.8)
Vd/F (L)	448.92 (21.5)	409.12 (19.2)	403.66 (345.1)	200.45 (28.8)	164.93 (18.7)	150.17 (71.2)

[a] Results are presented in geometric mean (geometric coefficient of variation %) unless stated otherwise.

[b] These values are expressed as median (range).

PK = pharmacokinetic, C_{max} = maximum concentration, $AUC_{0\text{-}inf}$ = area under the concentration-time curve from time 0 to infinite time, t_{max} = time of C_{max}, $t_{½}$ = elimination half-life, CL/F = apparent clearance, Vd/F = apparent volume of distribution where F is bioavailability, NA = not available due to limited number of samples for estimation of elimination slope.

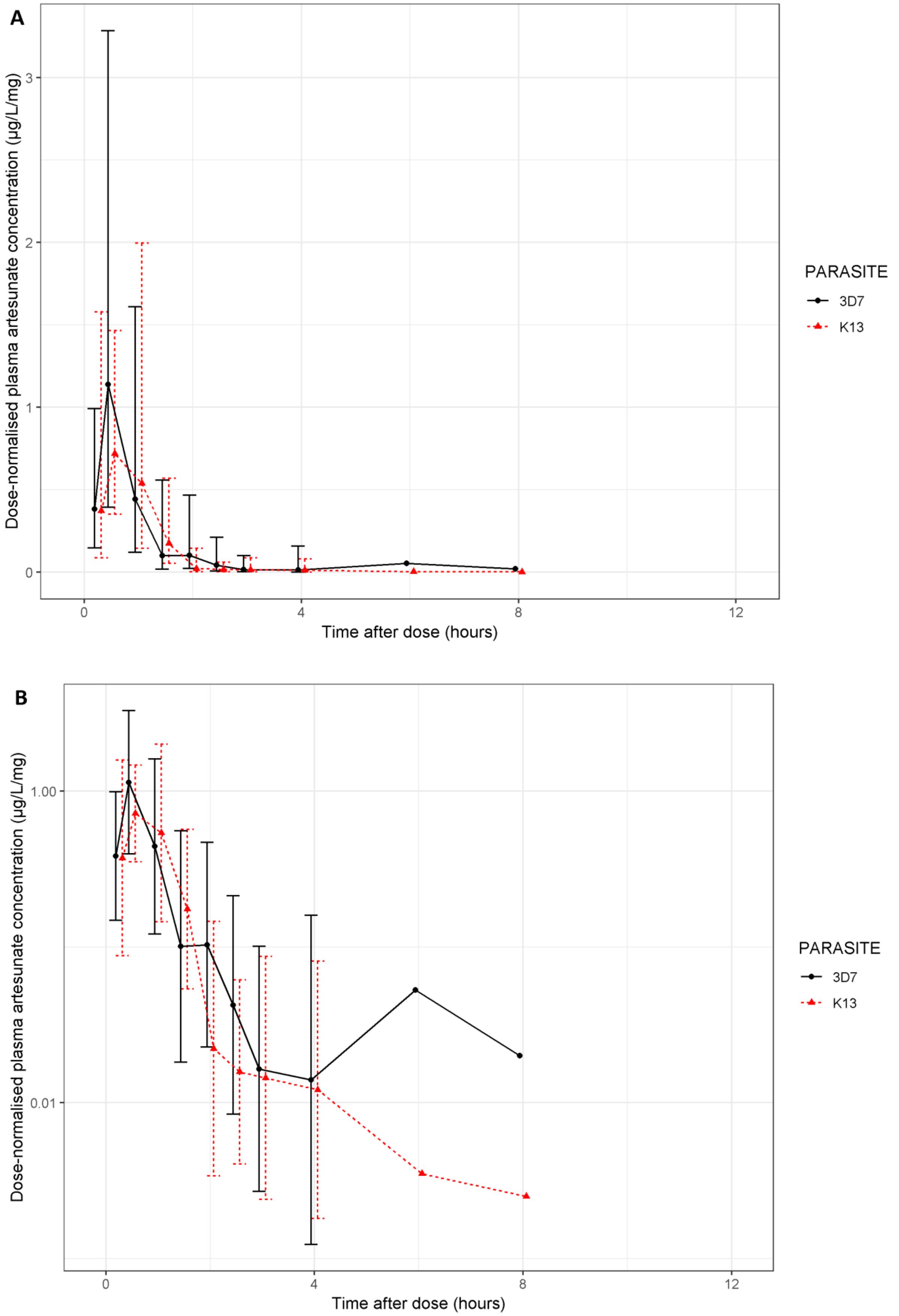

Figure 3: Geometric mean dose-normalised plasma artesunate concentration-time profiles plotted on a (A) linear and (B) log-linear scale. Error bars present ±1 geometric standard deviation.

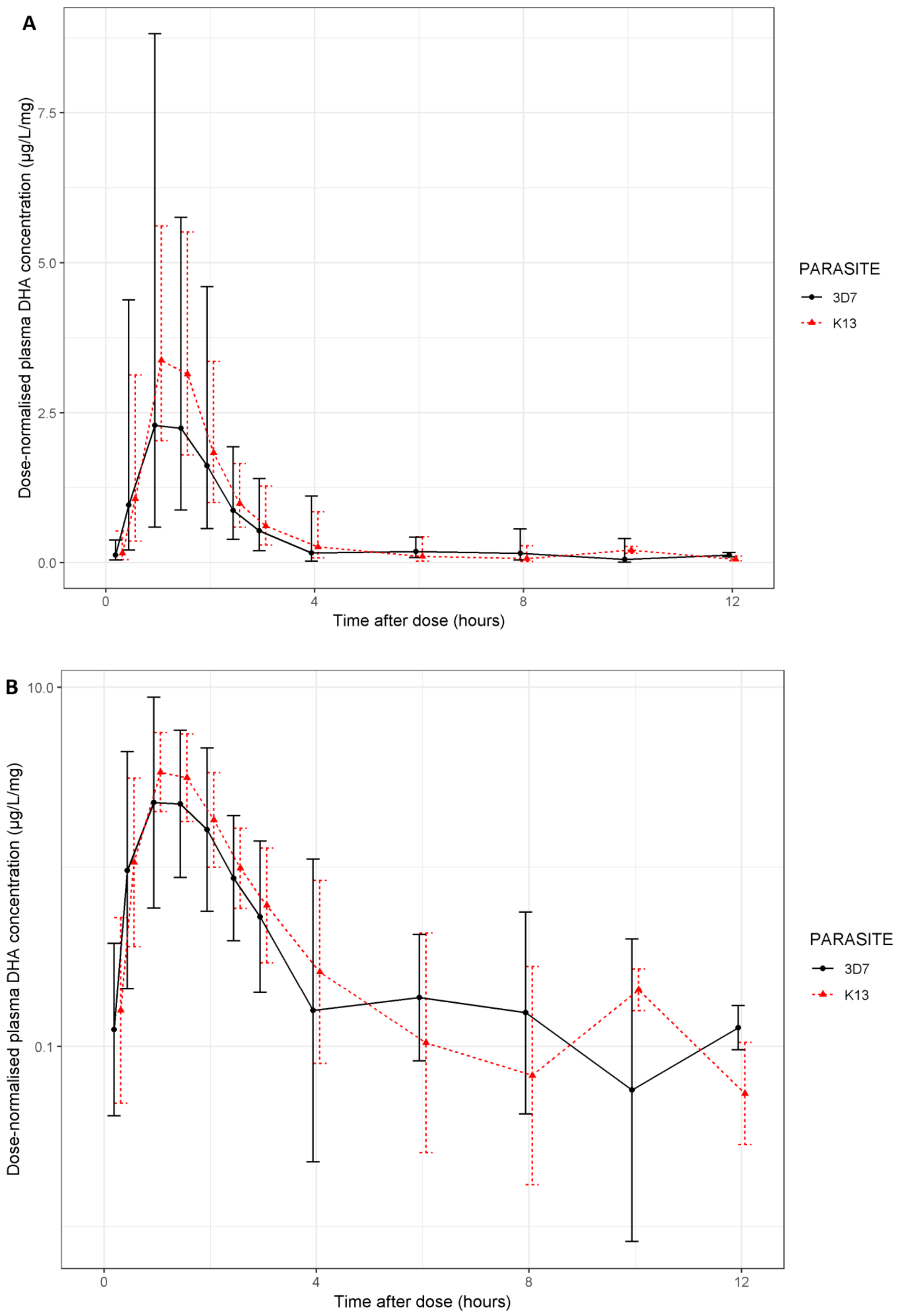

Figure 3: Geometric mean dose-normalised plasma dihydroartemisinin concentration-time profiles plotted on a (A) linear and (B) log-linear scale. Error bars present ±1 geometric standard deviation.

Table 2: Comparison of artesunate and dihydroartemisinin pharmacokinetic parameters by *P. falciparum* strains.

PK parameters	3D7 (n = 9)		K13 (n = 13)		*p*-value
	Geometric mean (CV%)	Median (Range)	Geometric mean (CV%)	Median (Range)	
**Artesunate**					
Dose-normalised C_{max} (µg/L/mg)	1.67 (71.5)	1.45 (0.64 – 4.16)	1.46 (75.6)	1.10 (0.73 – 6.35)	0.601
Dose-normalised $AUC_{0\text{-}inf}$ (µg·h/L/mg)	1.33 (29.5)	1.25 (0.97 – 1.86)	1.05 (38.0)	1.14 (0.27 – 1.82)	0.281
t_{max} (h)	0.77 (59.5)	0.5 (0.5 – 2.0)	0.64 (69.7)	1.0 (0.25 – 1.5)	0.623
$t_{½}$ (h)	0.53 (96.5)	0.42 (0.23 – 1.94)	0.53 (96.7)	0.35 (0.24 – 2.38)	0.955
CL/F (L/h)	752.79 (29.5)	798.93 (537.62 – 1032.3)	954.22 (38.0)	873.66 (550.36 – 1748.5)	0.281
Vd/F (L)	576.54 (92.9)	496.42 (238.83 – 2237.5)	727.41 (79.6)	650.03 (357.41 – 1892.9)	0.463
**Dihydroartemisinin**					
Dose-normalised C_{max} (µg/L/mg)	4.32 (104.3)	5.23 (0.53 – 8.61)	4.75 (52.0)	4.89 (1.98 – 11.25)	0.664
Dose-normalised $AUC_{0\text{-}inf}$ (µg·h/L/mg)	6.33 (89.9)	7.72 (1.25 – 13.0)	7.51 (33.2)	6.66 (5.56 – 16.34)	0.845
t_{max} (h)	1.06 (51.8)	1.0 (0.5 – 2.0)	1.04 (52.0)	1.0 (0.5 – 2.0)	0.945
$t_{½}$ (h)	1.36 (102.4)	1.14 (0.41 – 4.21)	1.15 (67.8)	1.21 (0.28 – 2.91)	0.695
CL/F (L/h)	157.91 (89.9)	129.5 (76.94 – 798.04)	133.19 (33.2)	150.07 (61.21 – 179.93)	0.845
Vd/F (L)	310.34 (119.4)	213.08 (123.89 – 2491.7)	221.09 (53.6)	237.05 (72.19 – 504.18)	0.744

PK = pharmacokinetic, CV = coefficient of variation, C_{max} = maximum concentration, $AUC_{0\text{-}inf}$ = area under the concentration-time curve from time 0 to infinite time, t_{max} = time of C_{max}, $t_{½}$ = elimination half-life, CL/F = apparent clearance, Vd/F = apparent volume of distribution where F is bioavailability.

Appendix 1: Regression line fit of plasma artesunate concentrations over time

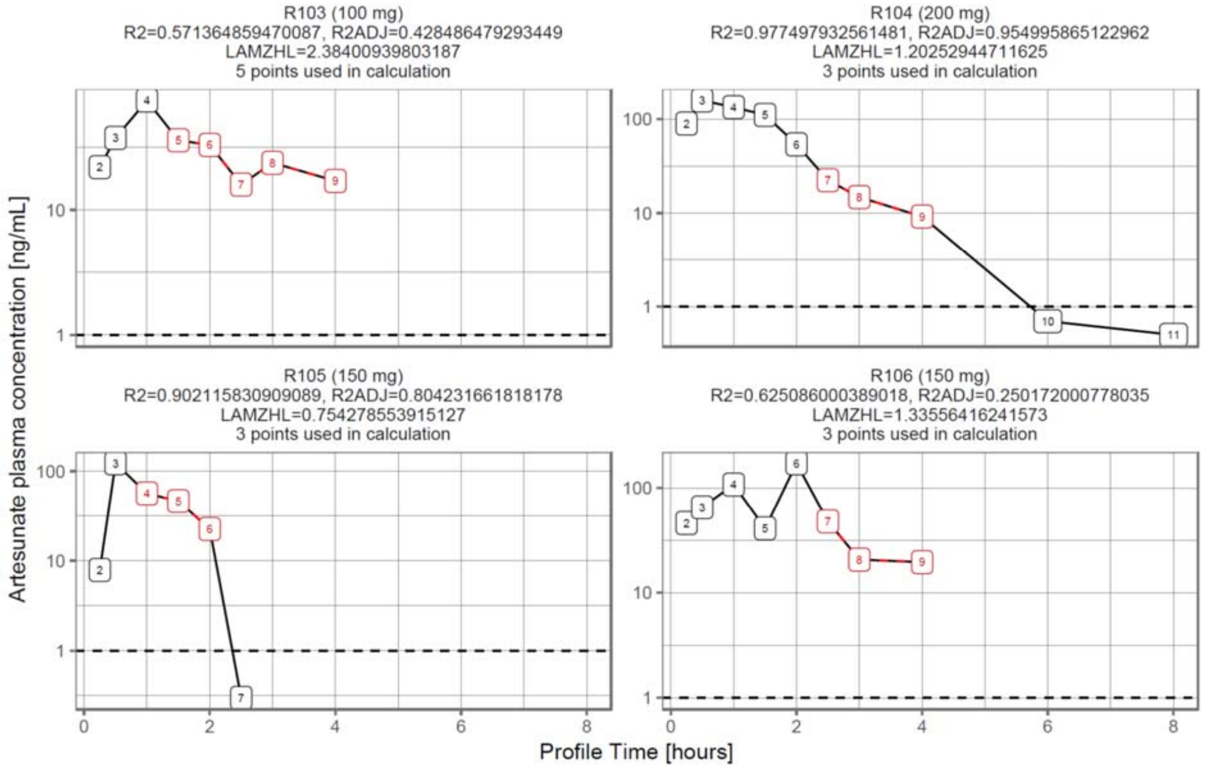

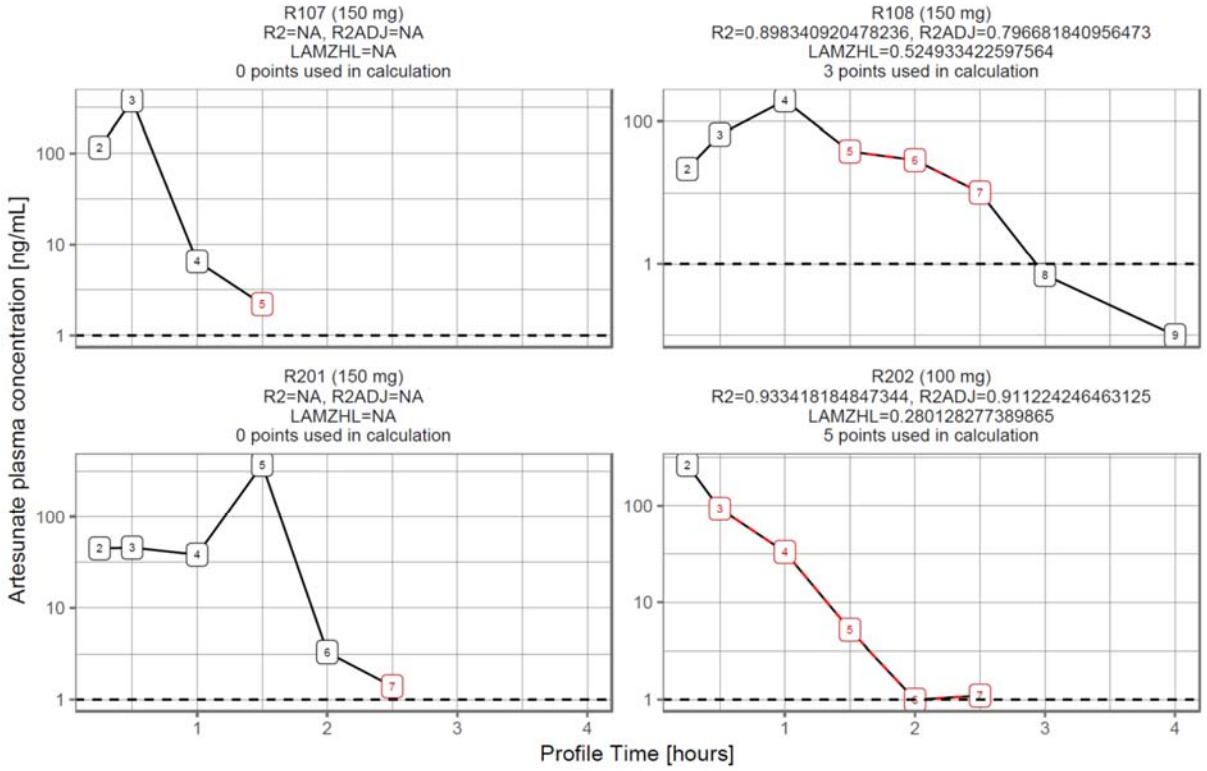

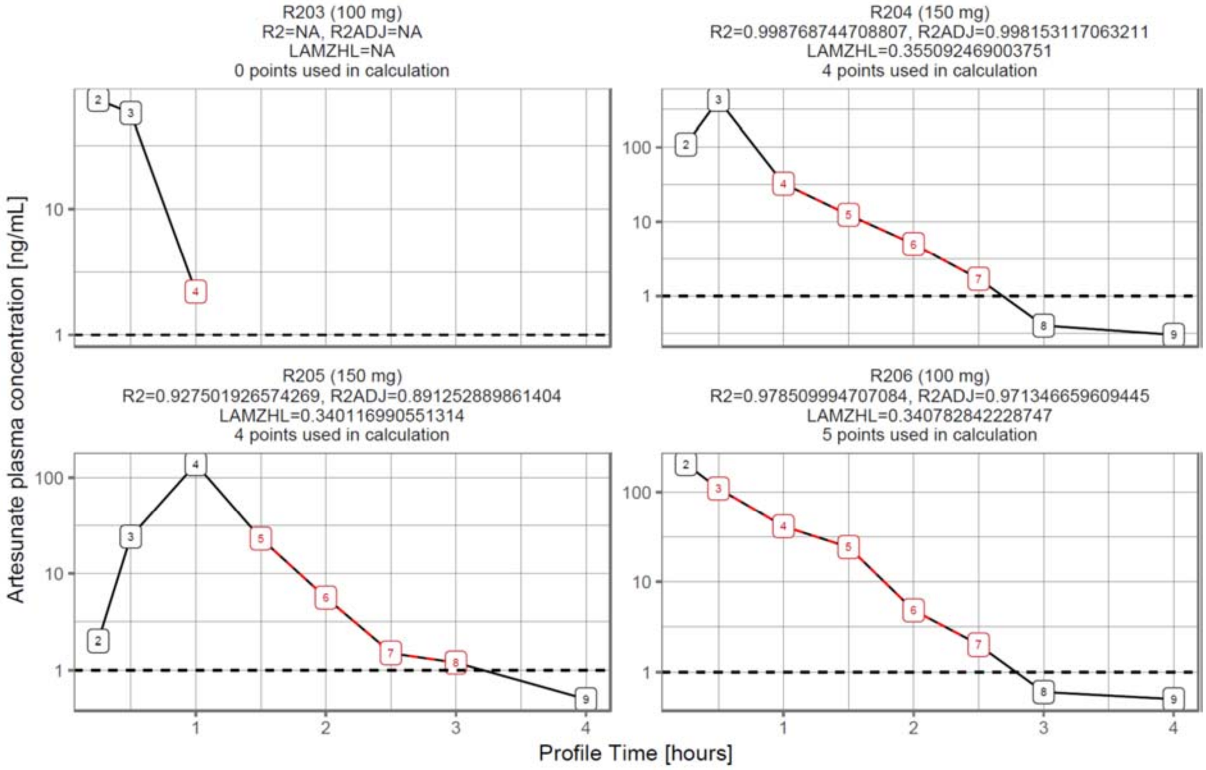
R203 (100 mg)
R2=NA, R2ADJ=NA
LAMZHL=NA
0 points used in calculation
R204 (150 mg)
R2=0.998768744708807, R2ADJ=0.998153117063211
LAMZHL=0.355092469003751
4 points used in calculation
R205 (150 mg)
R2=0.927501926574269, R2ADJ=0.891252889861404
LAMZHL=0.340116990551314
4 points used in calculation
R206 (100 mg)
R2=0.978509994707084, R2ADJ=0.971346659609445
LAMZHL=0.340782842228747
5 points used in calculation
Artesunate plasma concentration [ng/mL]
Profile Time [hours]

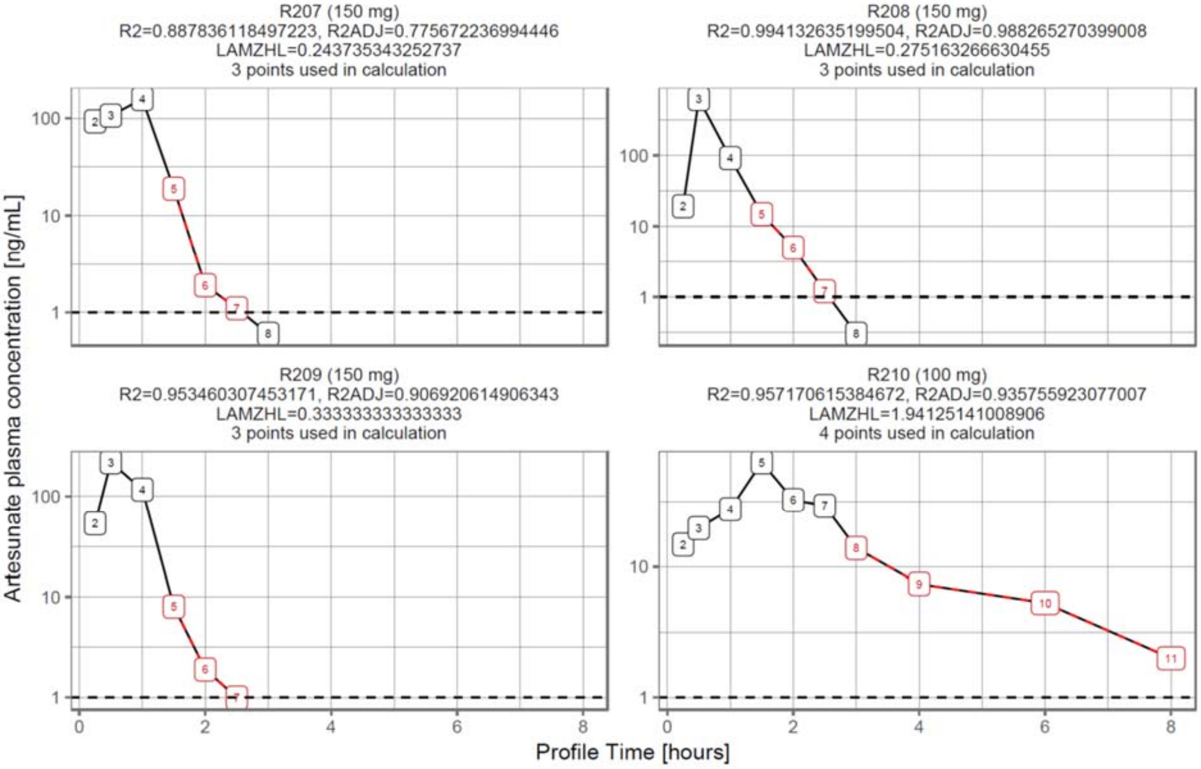
R207 (150 mg)
R2=0.887836118497223, R2ADJ=0.775672236994446
LAMZHL=0.243735343252737
3 points used in calculation
R208 (150 mg)
R2=0.994132635199504, R2ADJ=0.988265270399008
LAMZHL=0.275163266630455
3 points used in calculation
R209 (150 mg)
R2=0.953460307453171, R2ADJ=0.906920614906343
LAMZHL=0.333333333333333
3 points used in calculation
R210 (100 mg)
R2=0.957170615384672, R2ADJ=0.935755923077007
LAMZHL=1.94125141008906
4 points used in calculation
Artesunate plasma concentration [ng/mL]
Profile Time [hours]

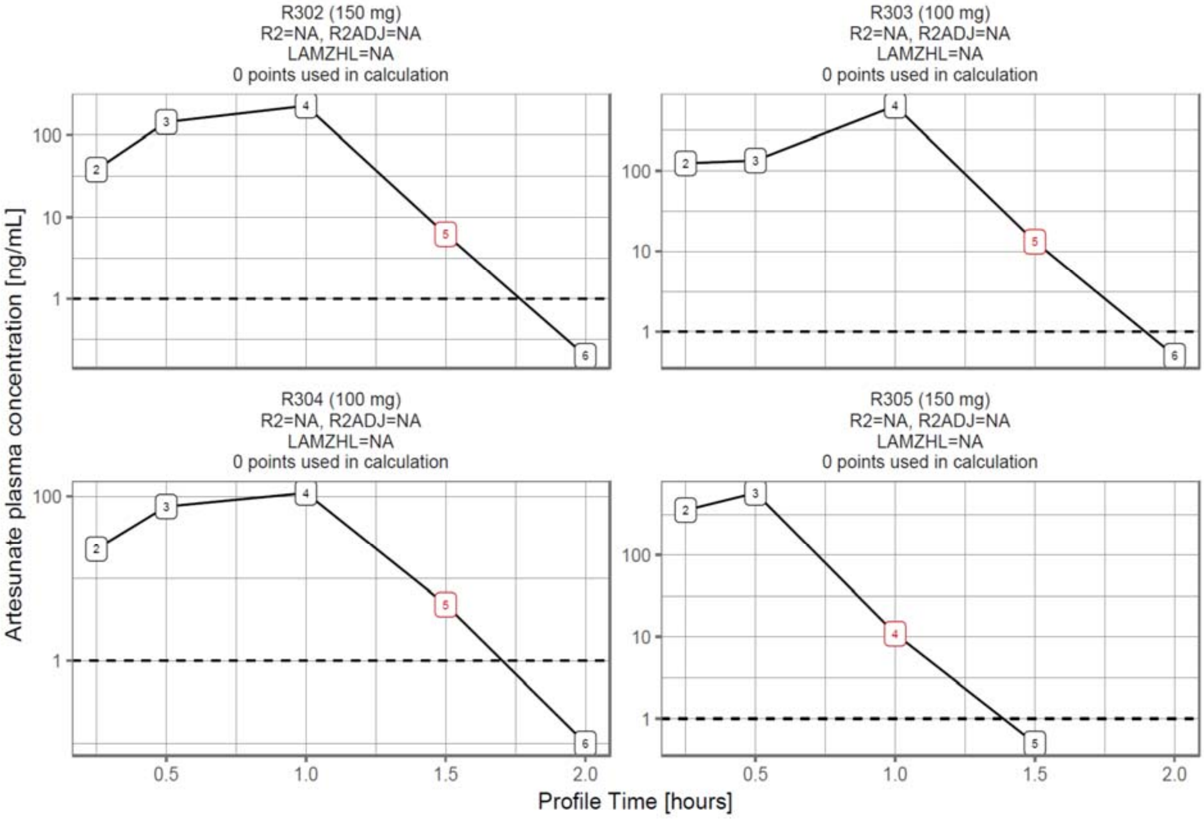
R302 (150 mg)
R2=NA, R2ADJ=NA
LAMZHL=NA
0 points used in calculation
R303 (100 mg)
R2=NA, R2ADJ=NA
LAMZHL=NA
0 points used in calculation
R304 (100 mg)
R2=NA, R2ADJ=NA
LAMZHL=NA
0 points used in calculation
R305 (150 mg)
R2=NA, R2ADJ=NA
LAMZHL=NA
0 points used in calculation
Artesunate plasma concentration [ng/mL]
Profile Time [hours]

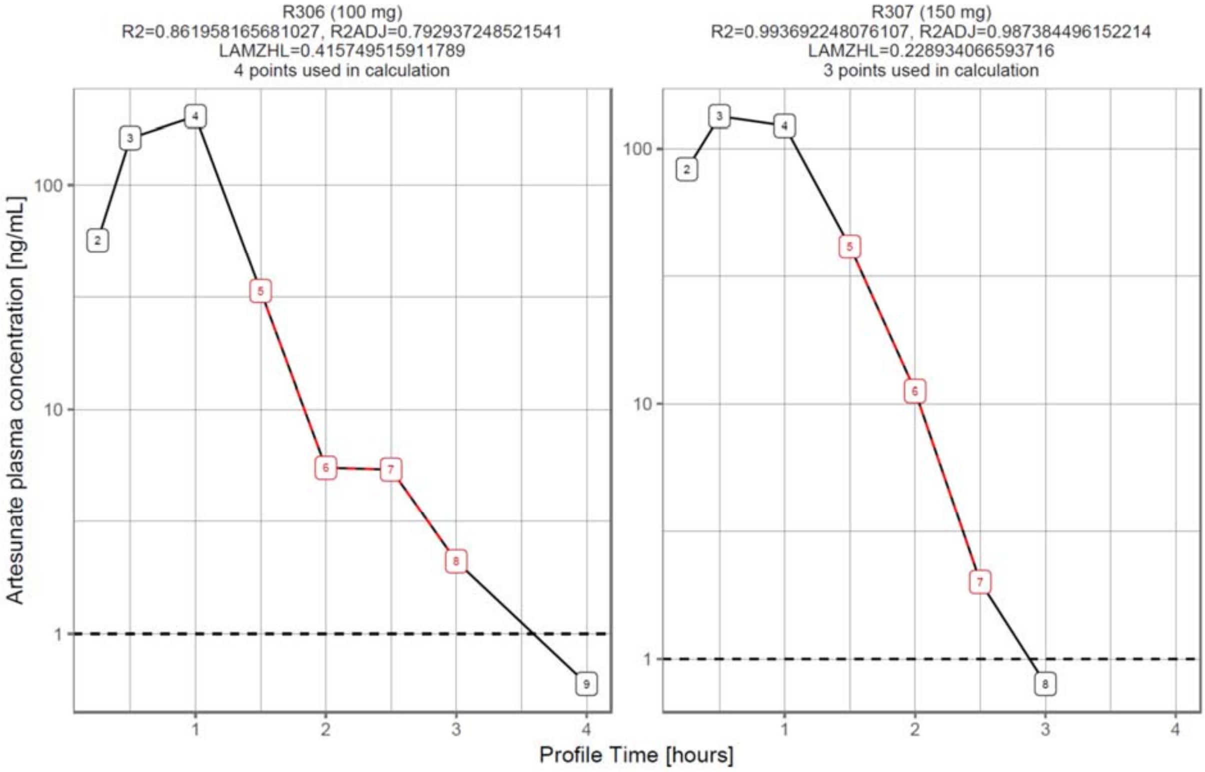
R306 (100 mg)
R2=0.861958165681027, R2ADJ=0.792937248521541
LAMZHL=0.415749515911789
4 points used in calculation
R307 (150 mg)
R2=0.993692248076107, R2ADJ=0.987384496152214
LAMZHL=0.228934066593716
3 points used in calculation
Artesunate plasma concentration [ng/mL]
Profile Time [hours]

Appendix 2: Regression line fit of plasma dihydroartemisinin concentrations over time.

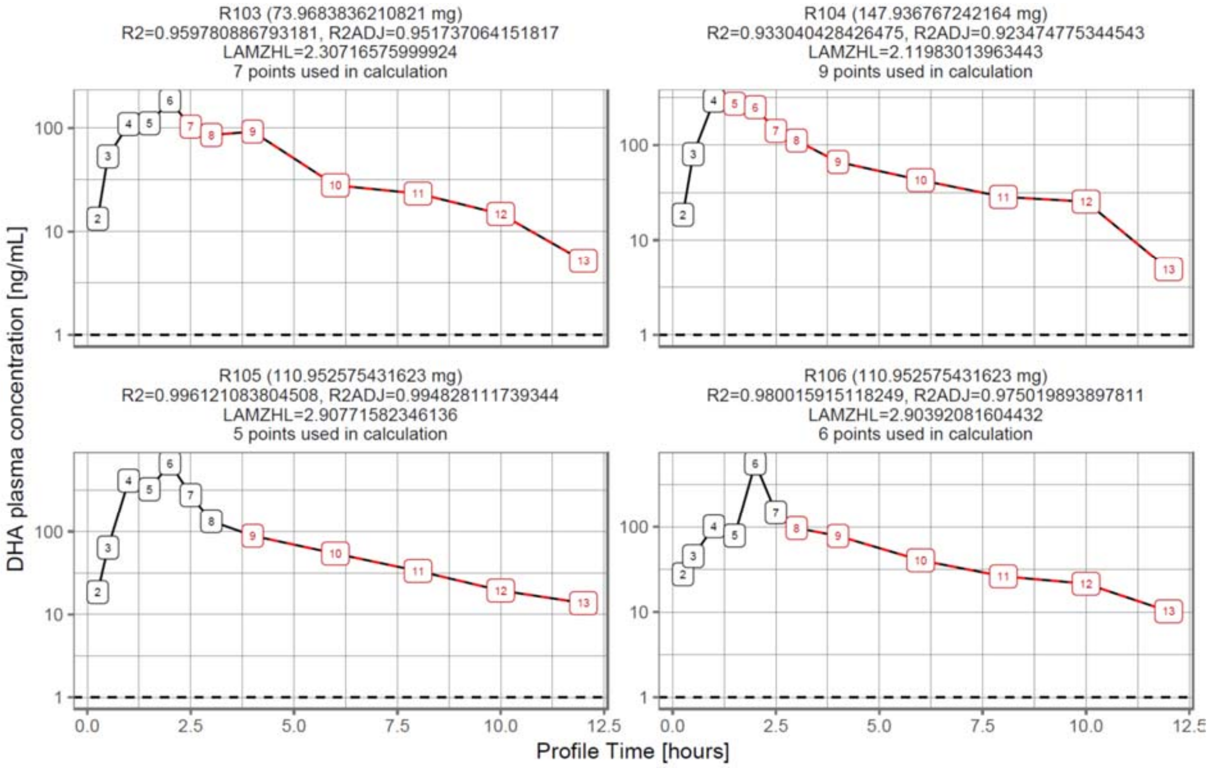

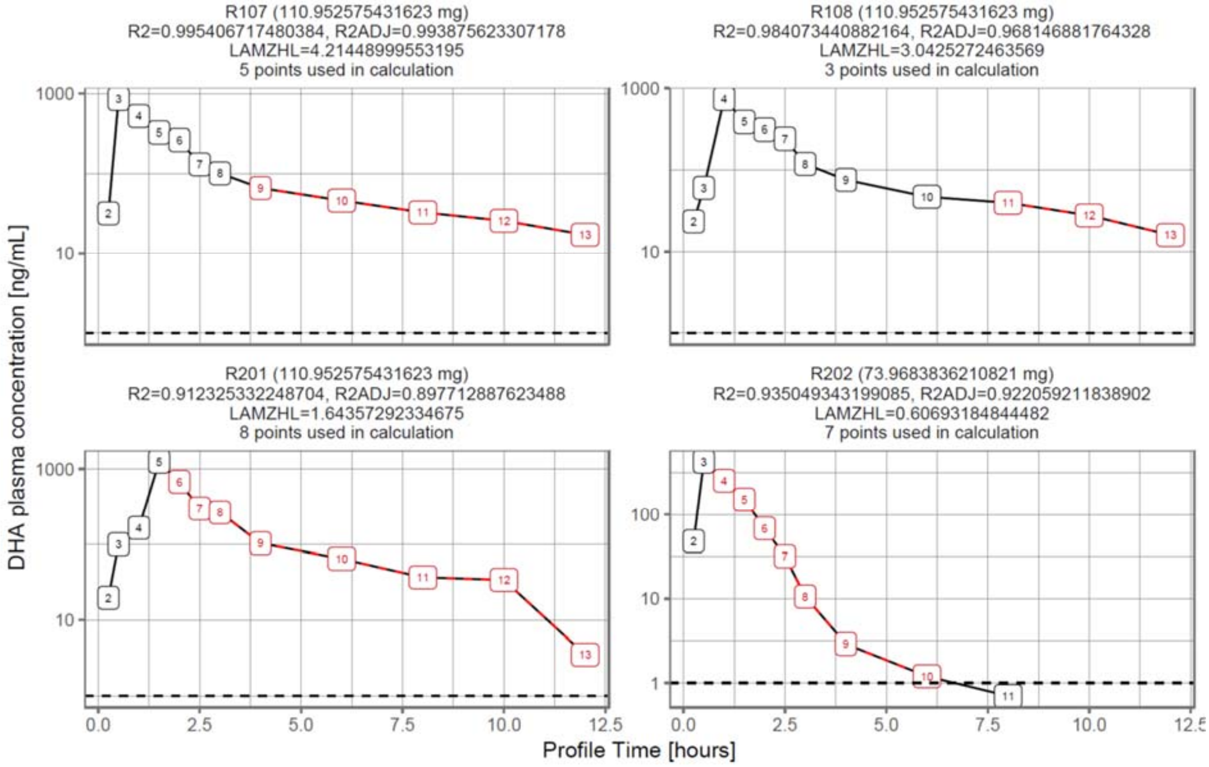

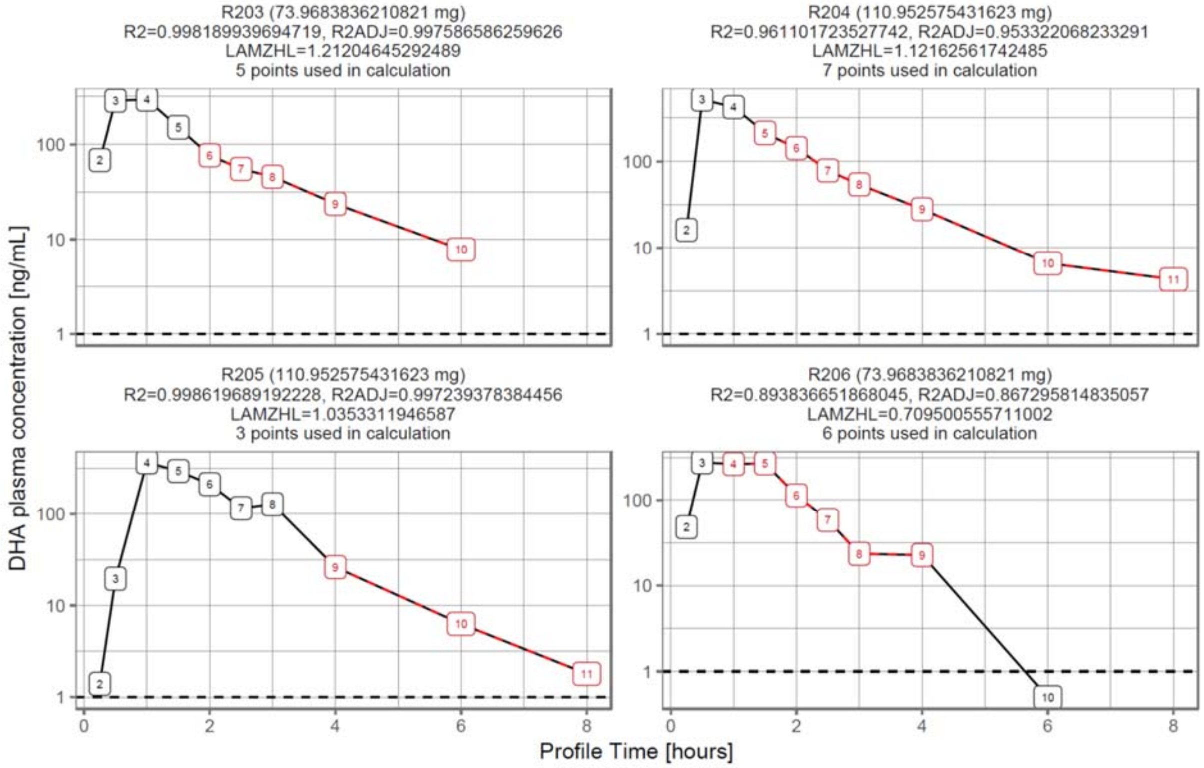
R203 (73.9683836210821 mg)
R2=0.998189939694719, R2ADJ=0.997586586259626
LAMZHL=1.21204645292489
5 points used in calculation
R204 (110.952575431623 mg)
R2=0.961101723527742, R2ADJ=0.953322068233291
LAMZHL=1.12162561742485
7 points used in calculation
R205 (110.952575431623 mg)
R2=0.998619689192228, R2ADJ=0.997239378384456
LAMZHL=1.0353311946587
3 points used in calculation
R206 (73.9683836210821 mg)
R2=0.893836651868045, R2ADJ=0.867295814835057
LAMZHL=0.709500555711002
6 points used in calculation
DHA plasma concentration [ng/mL]
Profile Time [hours]

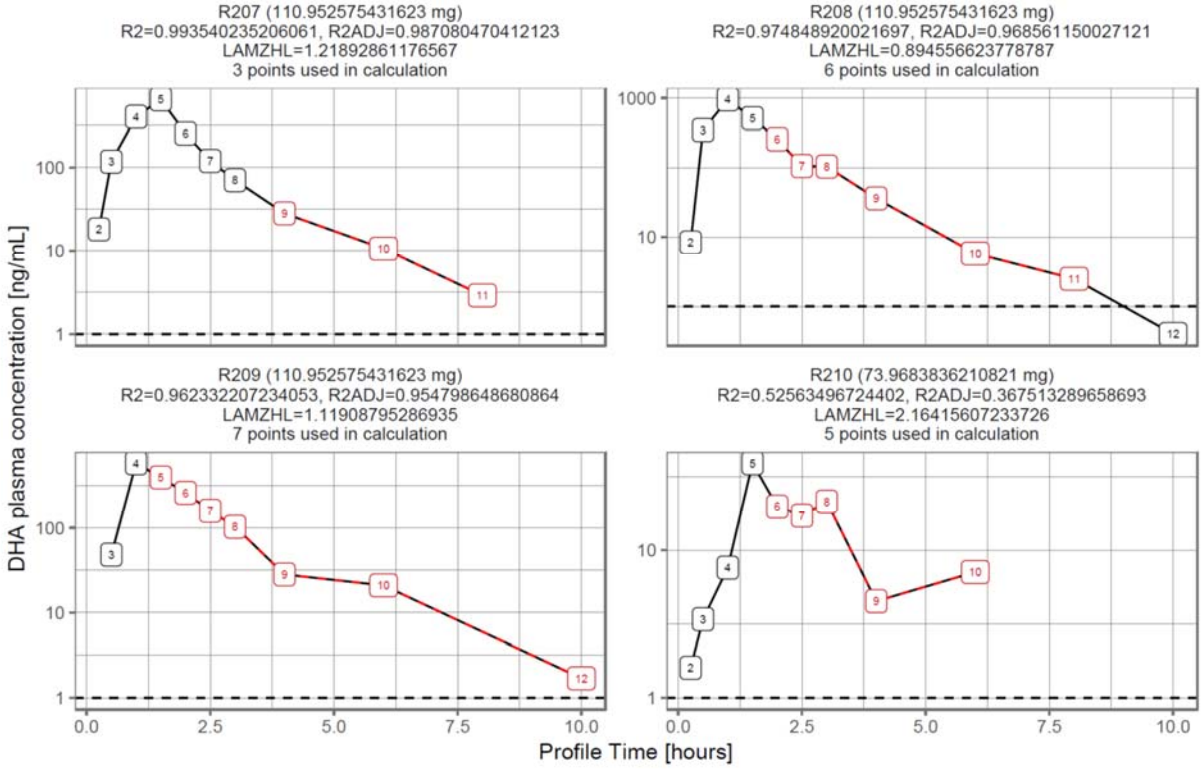
R207 (110.952575431623 mg)
R2=0.993540235206061, R2ADJ=0.987080470412123
LAMZHL=1.21892861176567
3 points used in calculation
R208 (110.952575431623 mg)
R2=0.974848920021697, R2ADJ=0.968561150027121
LAMZHL=0.894556623778787
6 points used in calculation
R209 (110.952575431623 mg)
R2=0.962332207234053, R2ADJ=0.954798648680864
LAMZHL=1.11908795286935
7 points used in calculation
R210 (73.9683836210821 mg)
R2=0.52563496724402, R2ADJ=0.367513289658693
LAMZHL=2.16415607233726
5 points used in calculation
DHA plasma concentration [ng/mL]
Profile Time [hours]

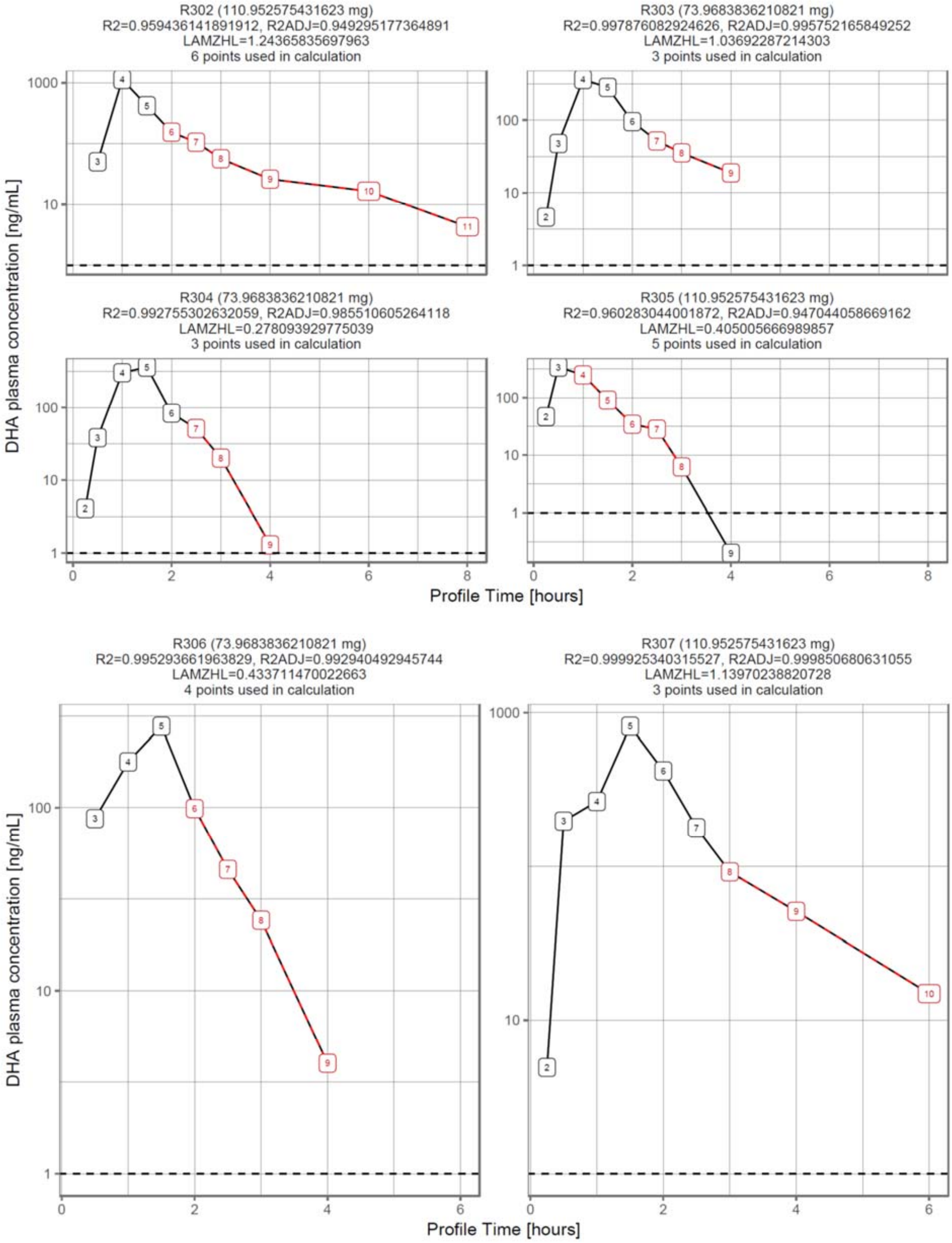
R302 (110.952575431623 mg)
R2=0.959436141891912, R2ADJ=0.949295177364891
LAMZHL=1.24365835697963
6 points used in calculation
R303 (73.9683836210821 mg)
R2=0.997876082924626, R2ADJ=0.995752165849252
LAMZHL=1.03692287214303
3 points used in calculation
R304 (73.9683836210821 mg)
R2=0.992755302632059, R2ADJ=0.985510605264118
LAMZHL=0.278093929775039
3 points used in calculation
R305 (110.952575431623 mg)
R2=0.960283044001872, R2ADJ=0.947044058669162
LAMZHL=0.405005666989857
5 points used in calculation
DHA plasma concentration [ng/mL]
Profile Time [hours]
R306 (73.9683836210821 mg)
R2=0.995293661963829, R2ADJ=0.992940492945744
LAMZHL=0.433711470022663
4 points used in calculation
R307 (110.952575431623 mg)
R2=0.999925340315527, R2ADJ=0.999850680631055
LAMZHL=1.13970238820728
3 points used in calculation
DHA plasma concentration [ng/mL]
Profile Time [hours]

PRR - Calculation - QP17C11 - All Cohorts Finalised

Lachlan Webb and Louise Marquart
5 February 2019

PRR_{48} analyses using the daily PCR data (tested in triplicates)

Dataset: Daily data for QP17C11 - P2339

Description:

The parasite reduction ratio (PRR_{48}) provides an estimate of the efficacy of an anti-malarial treatment and is the ratio of the parasite density over a 48 hour time-period. The PRR_{48} is estimated using the slope of the optimal fit of the log-linear relationship of the parasitemia decay.

The optimal fit can be derived using summarised replicate parasitemia data, which have been cleaned by dealing with potential outliers, values below the limit of detection (LOD) and non-detectable values (ND). The optimal fit of the log-linear parasitemia by time relationship is determined by using left and right censoring to systematically remove the potential lag phase and tail phase of the parasitemia decay.

The PRR_{48} was calculated for each subject in QP17C11 using the daily PCR data; and if the model fit was adequate for the subject (defined as overall model p-value<0.001), the slope and corresponding standard error (SE) from the log-linear regression was used to calculate the overall strain-specific PRR_{48} The details of the statistical methods used and the corresponding results are presented below.

Summary:

The PRR_{48} (95% CI) and corresponding parasite clearance half-life (95%CI) based on the 9 subjects in the three cohorts of QP17C11 who received the 3D7 strain, and the 13 subjects who received the K13 strain is presented below:

Study	Strain	n	PRR_{48} (95% CI)	Parasite clearance $t_{1/2}$ (95% CI)
QP17C11	3D7	9	38463 (23780 - 62212)	3.15 (3.01 - 3.30)
QP17C11	K13	13	169 (140 - 204)	6.49 (6.26 - 6.74)

Statistical Methods:

Statistical analysis consists of two parts, initially calculating the decay rate (slope coefficient from the log-linear decay regression) for each individual, and then calculating the weighted average slope estimate and corresponding SE using inverse-variance method - which would then be used to estimate the strain-specific PRR_{48} and 95% CI. The details for initial data cleaning and preparation and both statistical analysis steps are briefly described below. For a more detailed description the reader is referred to: Marquart et al. (2015) Evaluating the pharmacodynamic effect of antimalarial drugs in clinical trials by Quantitative PCR, Antimicrobial Agents and Chemotherapy, 7(59), 4249-4259.

Dealing with parasitemia values below the limit of detection (LOD), not detectable (ND) and clotted values

Once potential outliers have been identified and dealt with, the replicate parasitemia data were next cleaned to take into account parasitemia values where a value fell below the LOD or was not-detected (ND). For any replicate parasitemia values below LOD, the value was substituted with LOD/2. The LOD was assumed to be 64 parasites/mL of blood, based on Rockett et al. (2011). For any values that were ND the value was substituted with 1. If all replicates within a time-point were ND, the first time-point with all ND was included in model fitting, and all other time-points were set to missing.

Dealing with qPCR replicates

The daily tested PCR data parasitemia values (tested in triplicate per time-point) were log10 transformed, and the mean of the log10 transformed parasitemia was used as a summary parasitemia value per time-point per subject. All regression analyses were performed using the mean log10 parasitemia.

Regression modelling to determine optimal fit.

A regression modelling method, to incorporate both right and left censoring to remove potential lag and tail phases of the parasitemia decay curve in a systematic way, was used to determine the optimal number of data-points required to calculate the slope (β_1) of the log-linear parasitemia decay. A minimum number of 4 time-points was required for regression modelling.

The log-linear decay is modelled by the following:

$$\log_{10} Parasitemia = \beta_0 + \beta_1 Time$$

where time is the number of hours since administration of anti-malarial treatment, and β_0 and β_1 are the intercept and slope estimates of the log-linear regression, respectively.

The optimal log-linear regression model for a subject was deemed an appropriate fit if the overall model p-value < 0.001. Only optimal models with appropriate fit was used to calculate strain-specific PRR_{48}.

Estimating subject specific parasite reduction ratio (PRR_{48})

The slope and corresponding standard error estimate of the optimal linear regression model was used to calculate the subject PRR_{48} estimate and corresponding 95% confidence interval (95% CI). The PRR_{48} and 95% CI was calculated for each subject within each strain using the following formula:

$$PRR_{48} = 10^{-48\times\beta_1} \text{ and}$$

$$95\% \text{ CI: } 10^{-48(\beta_1 \pm 1.96\times SE(\beta_1))}$$

Estimating strain-specific PRR_{48}.

Of the subjects with appropriate overall fit (p<0.001), the average PRR_{48} and corresponding 95% CI for each strain was estimated by using the inverse variance method to calculate the weighted average linear regression slope ($\overline{\beta}_1$) and corresponding SE. The weighted average slope for n subjects in the strain is given by:

$$\overline{\beta}_1 = \frac{\sum_{i=1}^{n}(w_i \times \beta_{1,i})}{\sum_{i=1}^{n} w_i}, i = 1, \dots, n,$$

where the weight $w_i = \frac{1}{SE(\beta_{1,i})^2}$. The standard error $\hat{\beta}_1$ is estimated as, $SE(\overline{\beta}_1) = \sqrt{\frac{1}{\sum_{i=1}^{n} w_i}}$.

Therefore, the strain-specific PRR_{48} and corresponding 95 % confidence interval is estimated as:

$$PRR_{48} = 10^{-48\times\overline{\beta}_1}$$

$$95\ \% \text{ CI: } 10^{-48\times(\overline{\beta}_1 \pm 1.96\times SE(\overline{\beta}_1))}$$

Parasite clearance half-life.

The half-life is a transformation of the gradient (per time, equivalent to PRR_{48}) into a time period. The relationship between PRR_{48} and parasite reduction half-life ($t_{1/2}$) is a simple transformation of the PRR_{48}:

$$t_{1/2} = \log_{10}(2) \times \left(\frac{48 \text{ hours}}{\log_{10}(PRR_{48})}\right) = \frac{\log_{10}(2)}{-\beta_1},$$

where the PRR_{48} is the parasitemia ratio estimated over a 48 hour interval that is subsequently transformed into a per hour gradient.

Optimal Regression Results:

All 3 Cohorts:

The last time-point included in the regression analysis (based on the last time-point with all replicate values set to ND) for the 22 subjects in QP17C11 Cohort 1, 2 and 3 is given in Table 1.

Table 1: Last time-point included in the regression analysis for each subject

Subject	Strain	Last time-point (hrs)
R103	K13	60
R104	K13	60
R105	K13	60
R106	3D7	24
R107	3D7	72
R108	3D7	48
R201	K13	56
R202	K13	56
R203	K13	56
R204	K13	56
R205	K13	56
R206	K13	56
R207	K13	56
R208	3D7	60
R209	3D7	72
R210	3D7	60
R302	K13	52
R303	K13	48
R304	K13	44
R305	3D7	52
R306	3D7	52
R307	3D7	108

The subject specific PRR_{48} and corresponding 95% confidence interval are presented in Table 2 for the subjects in QP17C11 Cohort 1, 2 and 3. The results show the regression fit (R-squared (R^2)) and parameters (slope ($\hat{\beta}_1$)) and corresponding SE (SE($\hat{\beta}_1$)) for the subject based on the optimal regression.

The corresponding plot of log-10 parasitemia over time is presented in Figures 1, 2 and 3.

Table 2: Optimal regression fit for the subjects in QP17C11 Cohort 1, 2 and 3, participants in italics had non-significant regression fits and were not included in strain-specific PRR_{48} estimate.

Subject	Iter.	R^2	p-value	$\hat{\beta}_1$	$SE(\hat{\beta}_1)$	$PRR_{48}(95\%CI)$	Half-life (95% CI)
QP17C11-R103	5	95.7 %	9.8e-07	-0.054	0.004	380 (158-913)	5.60 (4.88-6.57)
QP17C11-R104	4	93.2 %	1.5e-06	-0.041	0.004	88 (40-195)	7.43 (6.31-9.02)
QP17C11-R105	5	95.5 %	1.2e-06	-0.053	0.004	345 (143-832)	5.69 (4.95-6.71)
QP17C11-R106	1	94.1 %	6.6e-05	-0.211	0.022	13850005272 (128179643-1496514117525)	1.42 (1.19-1.78)
QP17C11-R107	4	97.3 %	2.4e-08	-0.088	0.005	16676 (5751-48362)	3.42 (3.08-3.84)
QP17C11-R108	0	91.0 %	4.3e-07	-0.113	0.011	256105 (25381-2584233)	2.67 (2.25-3.28)
QP17C11-R201	6	98.2 %	1.5e-05	-0.045	0.003	139 (77-248)	6.75 (6.03-7.65)
QP17C11-R202	5	89.3 %	3.7e-05	-0.055	0.007	441 (103-1896)	5.46 (4.41-7.18)
QP17C11-R203	8	99.0 %	3.3e-06	-0.041	0.002	89 (60-132)	7.41 (6.81-8.12)
QP17C11-R204	4	93.1 %	6.5e-06	-0.050	0.005	263 (92-756)	5.97 (5.02-7.36)
QP17C11-R205	5	95.3 %	1.4e-06	-0.045	0.004	138 (64-295)	6.76 (5.85-7.99)
QP17C11-R206	5	96.7 %	3.1e-07	-0.052	0.003	299 (145-618)	5.84 (5.18-6.69)
QP17C11-R207	4	95.6 %	2.1e-07	-0.051	0.004	270 (123-591)	5.94 (5.21-6.91)
QP17C11-R208	2	93.3 %	3.5e-07	-0.119	0.010	495565 (55993-4385963)	2.54 (2.18-3.04)
QP17C11-R209	4	96.7 %	6.0e-08	-0.136	0.008	3485515 (559550-21711753)	2.21 (1.97-2.51)
QP17C11-R210	0	92.8 %	3.3e-08	-0.099	0.008	57142 (10121-322626)	3.04 (2.62-3.61)
QP17C11-R302	4	98.6 %	1.2e-08	-0.051	0.002	286 (179-459)	5.88 (5.43-6.42)
QP17C11-R303	2	95.6 %	2.1e-07	-0.041	0.003	90 (48-169)	7.40 (6.49-8.60)
QP17C11-R304	3	92.7 %	3.2e-05	-0.056	0.006	500 (137-1825)	5.35 (4.43-6.76)
QP17C11-R305	4	97.2 %	1.8e-07	-0.094	0.006	33428 (9758-114511)	3.19 (2.86-3.62)
QP17C11-R306	4	98.4 %	2.0e-08	-0.103	0.005	83211 (30202-229260)	2.94 (2.70-3.23)
QP17C11-R307	3	88.0 %	3.1e-09	-0.060	0.005	764 (238-2454)	5.01 (4.26-6.08)

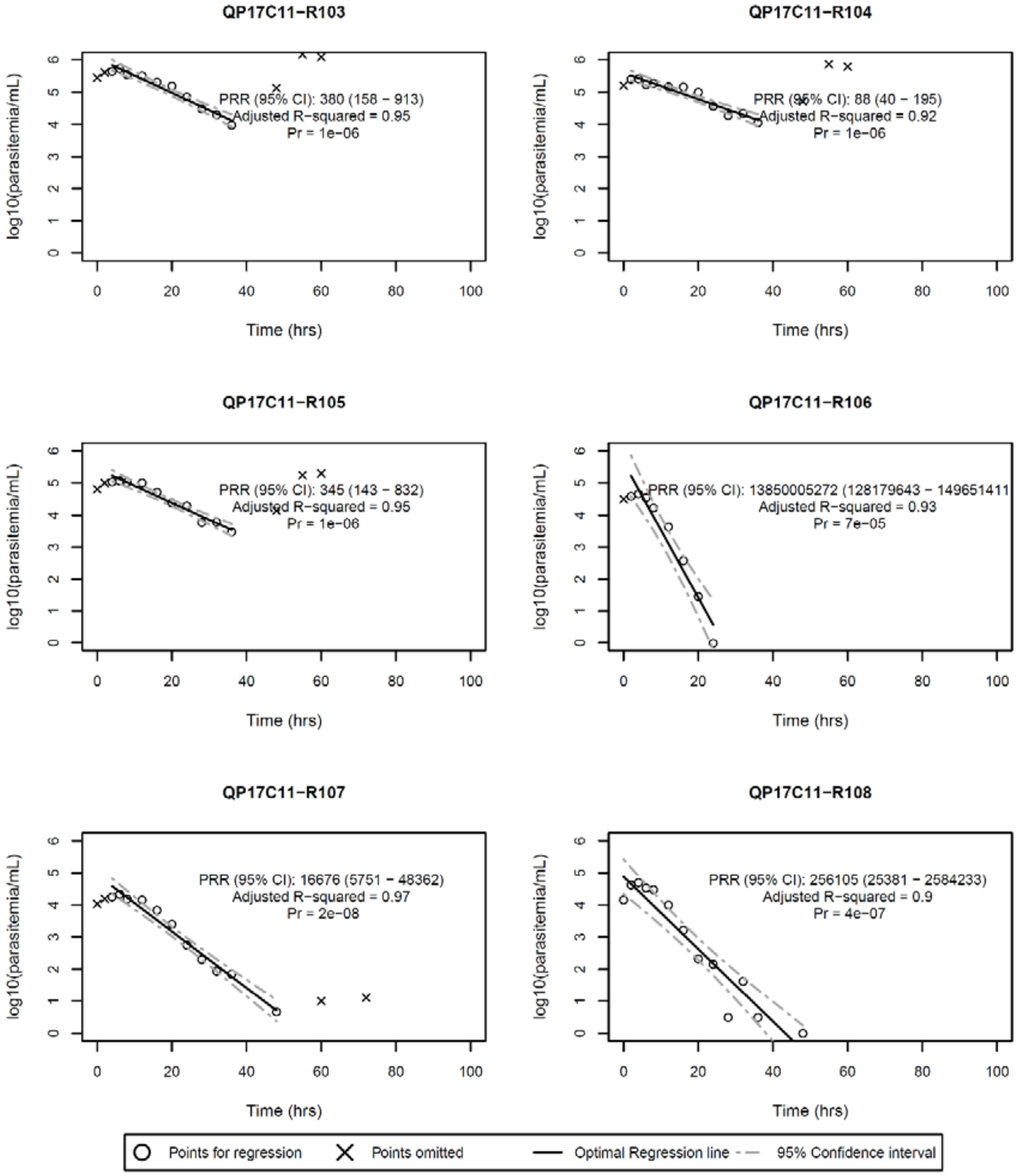

Figure 1: Optimal regression fit for the subjects in QP17C11 Cohort 1

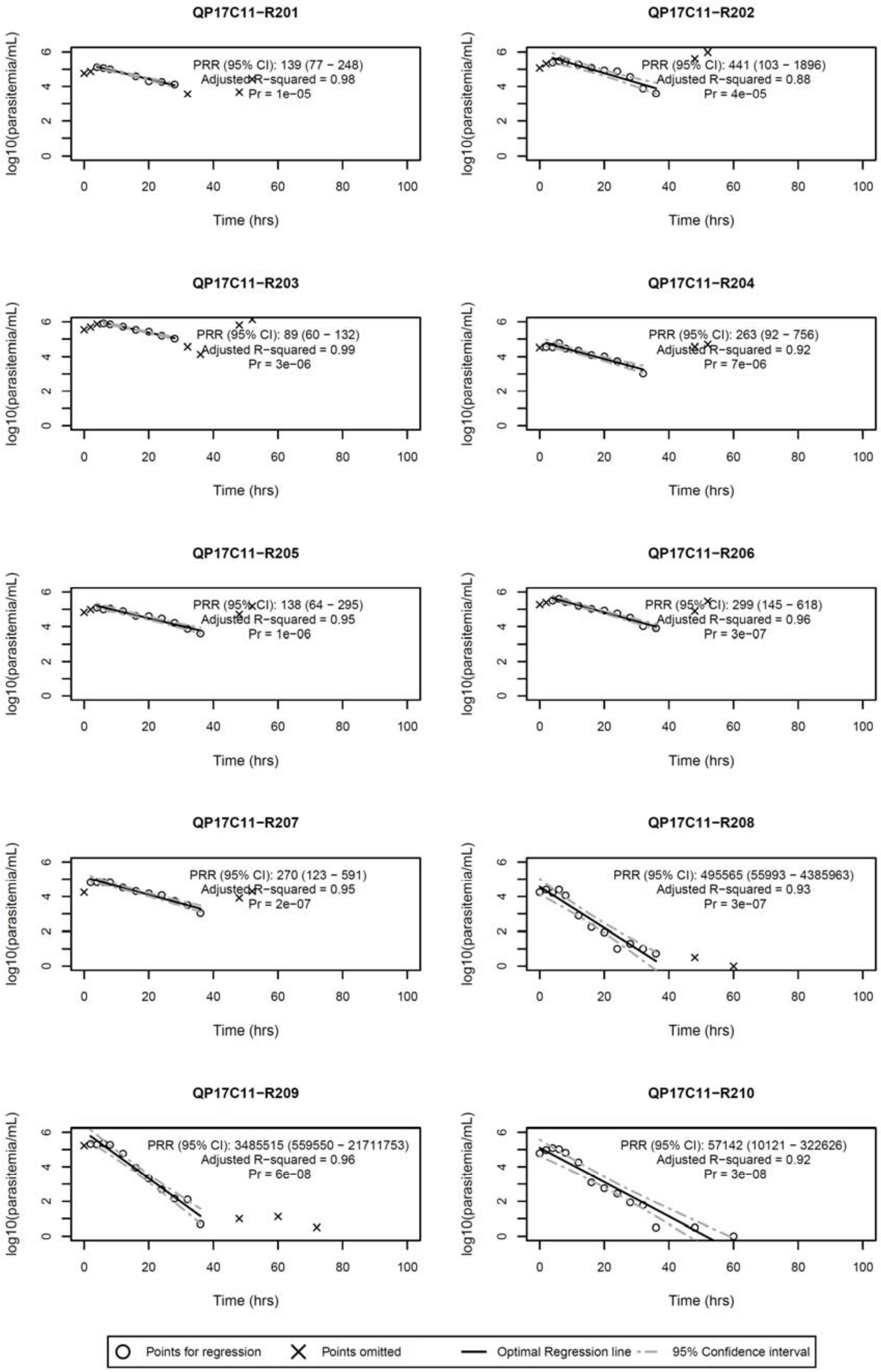

Figure 2: Optimal regression fit for the subjects in QP17C11 Cohort 2

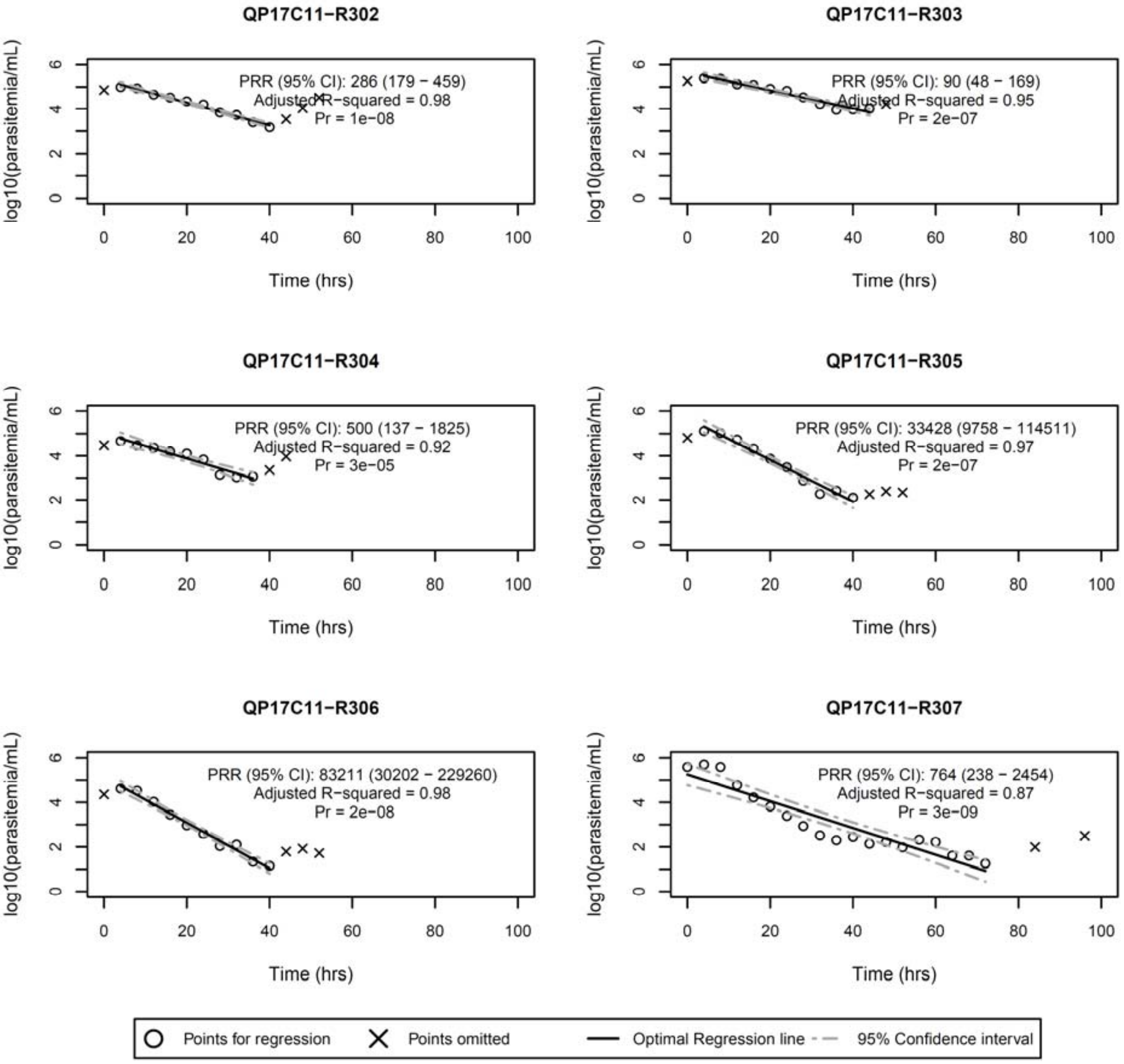

Figure 3: Optimal regression fit for the subjects in QP17C11 Cohort 3

Strain-specific PRR$_{48}$:

A strain-specific PRR$_{48}$ was calculated from the corresponding subjects that had significant regression models at the p=0.001 significance level.

3D7

All 9 of the subjects had significant regression models at the p=0.001 significance level, and contributed towards the strain-specific PRR$_{48}$ for 3D7 in QP17C11. The overall slope estimate and corresponding standard error used in the calculation of the strain-specific PRR$_{48}$ is:

$$\overline{\beta}_1 = -0.096$$

$$SE(\overline{\beta}_1) = 0.002$$

The PRR$_{48}$ and 95% confidence interval for QP17C11 is:

$$PRR_{QP17C11-3D7} = 38463 \text{ (95 \% CI: } 23780 - 62212)$$

The corresponding half-life and 95% confidence interval for QP17C11 is:

$$t_{1/2_{QP17C11-3D7}} = 3.15 \text{ (95 \% CI: } 3.01 - 3.3)$$

K13

All 13 of the subjects had significant regression models at the p=0.001 significance level, and contributed towards the strain-specific PRR$_{48}$ for K13 in QP17C11. The overall slope estimate and corresponding standard error used in the calculation of the strain-specific PRR$_{48}$ is:

$$\overline{\beta}_1 = -0.046$$

$$SE(\overline{\beta}_1) = 0.001$$

The PRR$_{48}$ and 95% confidence interval for QP17C11 is:

$$PRR_{QP17C11-K13} = 169 \text{ (95 \% CI: } 140 - 204)$$

The corresponding half-life and 95% confidence interval for QP17C11 is:

$$t_{1/2_{QP17C11-K13}} = 6.49 \text{ (95 \% CI: } 6.26 - 6.74)$$

Comparing the regression slopes of 3D7 and K13 strains in QP17C11 Cohorts 1, 2 and 3

In QP17C11 Cohorts 1, 2 and 3, 13 participants were inoculated with the K13 parasite strain and the remaining 9 participants were inoculated with the 3D7 strain. The following analysis was performed to determine whether there were differences between the optimal decay slopes for the two parasite strains using an omnibus test for between-group differences (Cooper, Hedges and Valentine, 2009). The test is used to assess whether there are differences in the weighted mean slope of the two strains: $\overline{\beta}_{1\ (K13)}$ and $\overline{\beta}_{1\ (3D7)}$, and is based on the following test statistic:

$$Q_B = \sum_{j=1}^{p} w_{j\cdot}\,(\overline{\beta}_{j\cdot} - \overline{\beta}_{\cdot\cdot})^2\ \chi^2_{p-1}$$

which follows a chi-squared distribution with P-1 degrees of freedom, where P is the number of strains to compare. The weight for the jth strain is denoted by $w_{j\cdot} = \sum_{i=1}^{n_i} w_{ij}$ for subject i in strain j. The $\overline{\beta}_{j\cdot}$ is the weighted average slope for strain j as derived on Page 3, and $\overline{\beta}_{\cdot\cdot}$ is the weighted grand mean given by:

$$\overline{\beta}_{\cdot\cdot} = \frac{\sum_{j=1}^{p} w_{j\cdot}\,\overline{\beta}_{j\cdot}}{\sum_{j=1}^{p} w_{j\cdot}}$$

The results for comparing K13 and 3D7 parasite strains in Cohorts 1, 2 and 3 of QP17C11 are presented below.

Table 3: Q_B test for between-strain differences of weighted slope (with corresponding PRR_{48} and half-life estimates).

Parasite strain	n	$\overline{\beta}_1$ (95% CI)	PRR_{48} (95% CI)	Half-life (95% CI)	Q_B Test Statistic, df	P-value
K13	13	-0.046 (-0.048, -0.045)	169 (140 - 204)	6.49 (6.26 - 6.74)	423.7, df = 1	<0.001
3D7	9	-0.096 (-0.1, -0.091)	38463 (23780 - 62212)	3.15 (3.01 - 3.3)		

Table 3:

Conclusion:

At the 5% significance level there is a significant difference in the weighted regression slopes (and hence, corresponding PRR_{48} estimates) of subjects inoculated with K13 or 3D7 in Cohorts 1, 2 and 3 of QP17C11 (p<0.001).

Cooper, H., Hedges, L.V. and Valentine, J.C. Chapter 15: Analyzing effect sizes: Fixed-Effects models in *The Handbook of Research Synthesis and Meta-Analysis, Second Edition, 2009 Russell Sage Foundation, New York.*

Appendix 16.2 Subject Data Listings

16.2.1 Study enrolment and completion/discontinuation

16.2.1.1 Study enrolment and completion/discontinuation

Listing 16.2.1.1
Study Enrolment and Completion/Discontinuation

Rando. Number	Challenge Agent	Date/Time of Informed Consent	Date of Randomisation	Did Subject Complete Study?	Date/Time of Inoculum Dose	Date/Time of Artesunate Dose	Date/Time of Piperaquine Dose	Date of Final Study Participation	Primary Reason for Study Discontinuation
R103	K13	01-NOV-2017/ 10:58	13-NOV-2017	Yes	14-NOV-2017/ 10:38	23-NOV-2017/ 10:50	25-NOV-2017/ 18:05	30-DEC-2017	
R104	K13	09-NOV-2017/ 09:05	13-NOV-2017	Yes	14-NOV-2017/ 10:42	23-NOV-2017/ 10:40	25-NOV-2017/ 18:02	30-DEC-2017	
R105	K13	08-NOV-2017/ 11:45	13-NOV-2017	Yes	14-NOV-2017/ 10:46	23-NOV-2017/ 10:30	25-NOV-2017/ 17:59	22-DEC-2017	
R106	3D7	09-NOV-2017/ 09:05	13-NOV-2017	Yes	15-NOV-2017/ 10:25	23-NOV-2017/ 10:20	01-DEC-2017/ 08:40	30-DEC-2017	
R107	3D7	03-NOV-2017/ 10:04	13-NOV-2017	Yes	15-NOV-2017/ 10:30	23-NOV-2017/ 10:10	04-DEC-2017	04-JAN-2018	
R108	3D7	26-OCT-2017/ 08:17	13-NOV-2017	Yes	15-NOV-2017/ 10:34	23-NOV-2017/ 10:01	01-DEC-2017/ 07:34	10-JAN-2018	
R201	K13	08-FEB-2018/ 10:36	19-FEB-2018	Yes	19-FEB-2018/ 10:23	28-FEB-2018/ 13:22	02-MAR-2018/ 13:45	22-MAR-2018	
R202	K13	13-FEB-2018/ 10:23	19-FEB-2018	Yes	19-FEB-2018/ 10:27	28-FEB-2018/ 13:03	02-MAR-2018/ 13:58	04-APR-2018	
R203	K13	13-FEB-2018/ 09:09	19-FEB-2018	Yes	19-FEB-2018/ 10:31	28-FEB-2018/ 11:40	02-MAR-2018/ 13:35	17-APR-2018	
R204	K13	05-FEB-2018/ 09:07	19-FEB-2018	Yes	19-FEB-2018/ 10:34	28-FEB-2018/ 13:15	02-MAR-2018/ 13:52	27-MAR-2018	
R205	K13	31-JAN-2018/ 08:50	19-FEB-2018	Yes	19-FEB-2018/ 10:37	28-FEB-2018/ 13:10	02-MAR-2018/ 13:48	26-MAR-2018	
R206	K13	06-FEB-2018/ 12:08	19-FEB-2018	Yes	19-FEB-2018/ 10:40	28-FEB-2018/ 13:26	02-MAR-2018/ 13:40	03-MAY-2018	
R207	K13	14-FEB-2018/ 10:35	19-FEB-2018	Yes	19-FEB-2018/ 10:42	28-FEB-2018/ 12:58	02-MAR-2018/ 13:58	24-APR-2018	
R208	3D7	01-FEB-2018/ 10:56	20-FEB-2018	Yes	20-FEB-2018/ 10:18	28-FEB-2018/ 11:35	10-MAR-2018/ 08:03	28-MAR-2018	
R209	3D7	08-FEB-2018/ 09:32	20-FEB-2018	Yes	20-FEB-2018/ 10:21	28-FEB-2018/ 15:45	08-MAR-2018/ 08:49	22-MAR-2018	

Notes: Rando. = Randomisation.

Listing 16.2.1.1
Study Enrolment and Completion/Discontinuation

**Rando. Number**	**Challenge Agent**	**Date/Time of Informed Consent**	**Date of Randomisation**	**Did Subject Complete Study?**	**Date/Time of Inoculum Dose**	**Date/Time of Artesunate Dose**	**Date/Time of Piperaquine Dose**	**Date of Final Study Participation**	**Primary Reason for Study Discontinuation**
R210	3D7	12-FEB-2018/ 08:22	20-FEB-2018	Yes	20-FEB-2018/ 10:25	28-FEB-2018/ 11:45	08-MAR-2018/ 07:51	22-MAR-2018	
R302	K13	16-AUG-2018/ 08:41	04-SEP-2018	Yes	04-SEP-2018/ 10:26	13-SEP-2018/ 09:05	15-SEP-2018/ 09:10	12-OCT-2018	
R303	K13	09-AUG-2018/ 09:23	04-SEP-2018	Yes	04-SEP-2018/ 10:31	13-SEP-2018/ 11:18	15-SEP-2018/ 11:25	22-OCT-2018	
R304	K13	13-AUG-2018/ 10:55	04-SEP-2018	Yes	04-SEP-2018/ 10:36	13-SEP-2018/ 09:22	15-SEP-2018/ 09:33	12-OCT-2018	
R305	3D7	13-AUG-2018/ 08:24	05-SEP-2018	Yes	05-SEP-2018/ 11:25	13-SEP-2018/ 09:35	19-SEP-2018	19-NOV-2018	
R306	3D7	28-AUG-2018/ 10:42	05-SEP-2018	Yes	05-SEP-2018/ 11:19	13-SEP-2018/ 09:45	19-SEP-2018	11-OCT-2018	
R307	3D7	07-AUG-2018/ 11:31	05-SEP-2018	Yes	05-SEP-2018/ 11:30	13-SEP-2018/ 09:55	19-SEP-2018	19-OCT-2018	

Notes: Rando. = Randomisation.

16.2.1.2 Visit Dates

Listing 16.2.1.2
Visit Dates

Randomisation Number	Challenge Agent	Visit	Date of Visit	Day Relative to Inoculum	Day Relative to IP
R103	K13	Screening	01-NOV-2017	-13	-22
		Unscheduled	09-NOV-2017	-5	-14
		Safety Visit	13-NOV-2017	-1	-10
		Inoculum	14-NOV-2017	0	-9
		Malaria Monitoring	18-NOV-2017	4	-5
		Malaria Monitoring	19-NOV-2017	5	-4
		Malaria Monitoring	20-NOV-2017	6	-3
		Malaria Monitoring	21-NOV-2017	7	-2
		Malaria Monitoring	22-NOV-2017	8	-1
		Admission	23-NOV-2017	9	1
		Admission 1	24-NOV-2017	10	2
		Admission 2	25-NOV-2017	11	3
		Admission 3	26-NOV-2017	12	4
		Safety Monitoring	26-NOV-2017	12	4
		Safety Monitoring	27-NOV-2017	13	5
		Safety Monitoring	28-NOV-2017	14	6
		Safety Monitoring	29-NOV-2017	15	7
		Safety Monitoring	30-NOV-2017	16	8
		Safety Monitoring	01-DEC-2017	17	9
		Safety Monitoring	03-DEC-2017	19	11
		Safety Monitoring	04-DEC-2017	20	12
		Safety Monitoring	05-DEC-2017	21	13
		Safety Monitoring	07-DEC-2017	23	15
		Safety Monitoring	11-DEC-2017	27	19

Notes: Day relative to inoculum is the number of days relative to day of administration of the inoculum, where the day of administration = 0.
The inoculum was administered on Study Day 0 for K13-infected subjects and Study Day 1 for 3D7-infected subjects.
Day relative to IP is the number of days relative to day of administration of Artesunate, where the day of dose = 1.

Listing 16.2.1.2
Visit Dates

Randomisation Number	Challenge Agent	Visit	Date of Visit	Day Relative to Inoculum	Day Relative to IP
R103	K13	Safety Monitoring	12-DEC-2017	28	20
		End of Study	14-DEC-2017	30	22
		Unscheduled	18-DEC-2017	34	26
		Unscheduled	22-DEC-2017	38	30
		Unscheduled	27-DEC-2017	43	35
R104	K13	Screening	09-NOV-2017	-5	-14
		Safety Visit	13-NOV-2017	-1	-10
		Inoculum	14-NOV-2017	0	-9
		Malaria Monitoring	18-NOV-2017	4	-5
		Malaria Monitoring	19-NOV-2017	5	-4
		Malaria Monitoring	20-NOV-2017	6	-3
		Malaria Monitoring	21-NOV-2017	7	-2
		Malaria Monitoring	22-NOV-2017	8	-1
		Admission	23-NOV-2017	9	1
		Admission 1	24-NOV-2017	10	2
		Admission 2	25-NOV-2017	11	3
		Admission 3	26-NOV-2017	12	4
		Safety Monitoring	26-NOV-2017	12	4
		Safety Monitoring	27-NOV-2017	13	5
		Safety Monitoring	28-NOV-2017	14	6
		Safety Monitoring	29-NOV-2017	15	7
		Safety Monitoring	30-NOV-2017	16	8
		Safety Monitoring	01-DEC-2017	17	9
		Safety Monitoring	03-DEC-2017	19	11

Notes: Day relative to inoculum is the number of days relative to day of administration of the inoculum, where the day of administration = 0.
The inoculum was administered on Study Day 0 for K13-infected subjects and Study Day 1 for 3D7-infected subjects.
Day relative to IP is the number of days relative to day of administration of Artesunate, where the day of dose = 1.

Listing 16.2.1.2
Visit Dates

Randomisation Number	Challenge Agent	Visit	Date of Visit	Day Relative to Inoculum	Day Relative to IP
R104	K13	Safety Monitoring	04-DEC-2017	20	12
		Safety Monitoring	05-DEC-2017	21	13
		Safety Monitoring	07-DEC-2017	23	15
		Safety Monitoring	08-DEC-2017	24	16
		Safety Monitoring	10-DEC-2017	26	18
		Safety Monitoring	11-DEC-2017	27	19
		Safety Monitoring	12-DEC-2017	28	20
		End of Study	14-DEC-2017	30	22
		Unscheduled	18-DEC-2017	34	26
		Unscheduled	21-DEC-2017	37	29
		Unscheduled	29-DEC-2017	45	37
R105	K13	Screening	08-NOV-2017	-6	-15
		Safety Visit	13-NOV-2017	-1	-10
		Inoculum	14-NOV-2017	0	-9
		Malaria Monitoring	18-NOV-2017	4	-5
		Malaria Monitoring	19-NOV-2017	5	-4
		Malaria Monitoring	20-NOV-2017	6	-3
		Malaria Monitoring	21-NOV-2017	7	-2
		Malaria Monitoring	22-NOV-2017	8	-1
		Admission	23-NOV-2017	9	1
		Admission 1	24-NOV-2017	10	2
		Admission 2	25-NOV-2017	11	3
		Admission 3	26-NOV-2017	12	4
		Safety Monitoring	26-NOV-2017	12	4

Notes: Day relative to inoculum is the number of days relative to day of administration of the inoculum, where the day of administration = 0.
The inoculum was administered on Study Day 0 for K13-infected subjects and Study Day 1 for 3D7-infected subjects.
Day relative to IP is the number of days relative to day of administration of Artesunate, where the day of dose = 1.

Listing 16.2.1.2
Visit Dates

Randomisation Number	Challenge Agent	Visit	Date of Visit	Day Relative to Inoculum	Day Relative to IP
R105	K13	Safety Monitoring	27-NOV-2017	13	5
		Safety Monitoring	28-NOV-2017	14	6
		Safety Monitoring	29-NOV-2017	15	7
		Safety Monitoring	30-NOV-2017	16	8
		Safety Monitoring	01-DEC-2017	17	9
		Safety Monitoring	03-DEC-2017	19	11
		Safety Monitoring	04-DEC-2017	20	12
		Safety Monitoring	05-DEC-2017	21	13
		Safety Monitoring	07-DEC-2017	23	15
		Safety Monitoring	11-DEC-2017	27	19
		Safety Monitoring	12-DEC-2017	28	20
		End of Study	13-DEC-2017	29	21
		Unscheduled	18-DEC-2017	34	26
		Unscheduled	21-DEC-2017	37	29
R106	3D7	Screening	09-NOV-2017	-6	-14
		Safety Visit	13-NOV-2017	-2	-10
		Inoculum	15-NOV-2017	0	-8
		Malaria Monitoring	19-NOV-2017	4	-4
		Malaria Monitoring	20-NOV-2017	5	-3
		Malaria Monitoring	21-NOV-2017	6	-2
		Malaria Monitoring	22-NOV-2017	7	-1
		Admission	23-NOV-2017	8	1
		Admission 1	24-NOV-2017	9	2
		Admission 2	25-NOV-2017	10	3

Notes: Day relative to inoculum is the number of days relative to day of administration of the inoculum, where the day of administration = 0.
The inoculum was administered on Study Day 0 for K13-infected subjects and Study Day 1 for 3D7-infected subjects.
Day relative to IP is the number of days relative to day of administration of Artesunate, where the day of dose = 1.

Listing 16.2.1.2
Visit Dates

Randomisation Number	Challenge Agent	Visit	Date of Visit	Day Relative to Inoculum	Day Relative to IP
R106	3D7	Admission 3	26-NOV-2017	11	4
		Safety Monitoring	26-NOV-2017	11	4
		Safety Monitoring	27-NOV-2017	12	5
		Safety Monitoring	28-NOV-2017	13	6
		Safety Monitoring	29-NOV-2017	14	7
		Safety Monitoring	30-NOV-2017	15	8
		Safety Monitoring	01-DEC-2017	16	9
		Safety Monitoring	03-DEC-2017	18	11
		Safety Monitoring	04-DEC-2017	19	12
		Safety Monitoring	05-DEC-2017	20	13
		Safety Monitoring	07-DEC-2017	22	15
		Safety Monitoring	11-DEC-2017	26	19
		End of Study	14-DEC-2017	29	22
		Unscheduled	18-DEC-2017	33	26
		Unscheduled	21-DEC-2017	36	29
		Unscheduled	29-DEC-2017	44	37
R107	3D7	Screening	03-NOV-2017	-12	-20
		Safety Visit	13-NOV-2017	-2	-10
		Inoculum	15-NOV-2017	0	-8
		Malaria Monitoring	19-NOV-2017	4	-4
		Malaria Monitoring	20-NOV-2017	5	-3
		Malaria Monitoring	21-NOV-2017	6	-2
		Malaria Monitoring	22-NOV-2017	7	-1
		Admission	23-NOV-2017	8	1

Notes: Day relative to inoculum is the number of days relative to day of administration of the inoculum, where the day of administration = 0.
The inoculum was administered on Study Day 0 for K13-infected subjects and Study Day 1 for 3D7-infected subjects.
Day relative to IP is the number of days relative to day of administration of Artesunate, where the day of dose = 1.

Listing 16.2.1.2
Visit Dates

Randomisation Number	Challenge Agent	Visit	Date of Visit	Day Relative to Inoculum	Day Relative to IP
R107	3D7	Admission 1	24-NOV-2017	9	2
		Admission 2	25-NOV-2017	10	3
		Admission 3	26-NOV-2017	11	4
		Safety Monitoring	26-NOV-2017	11	4
		Safety Monitoring	27-NOV-2017	12	5
		Safety Monitoring	28-NOV-2017	13	6
		Safety Monitoring	29-NOV-2017	14	7
		Safety Monitoring	30-NOV-2017	15	8
		Safety Monitoring	01-DEC-2017	16	9
		Safety Monitoring	03-DEC-2017	18	11
		Safety Monitoring	04-DEC-2017	19	12
		Safety Monitoring	05-DEC-2017	20	13
		Safety Monitoring	07-DEC-2017	22	15
		Safety Monitoring	11-DEC-2017	26	19
		End of Study	14-DEC-2017	29	22
		Unscheduled	15-DEC-2017	30	23
		Unscheduled	18-DEC-2017	33	26
		Unscheduled	21-DEC-2017	36	29
		Unscheduled	27-DEC-2017	42	35
		Unscheduled	03-JAN-2018	49	42
R108	3D7	Screening	26-OCT-2017	-20	-28
		Safety Visit	13-NOV-2017	-2	-10
		Inoculum	15-NOV-2017	0	-8
		Malaria Monitoring	19-NOV-2017	4	-4

Notes: Day relative to inoculum is the number of days relative to day of administration of the inoculum, where the day of administration = 0.
The inoculum was administered on Study Day 0 for K13-infected subjects and Study Day 1 for 3D7-infected subjects.
Day relative to IP is the number of days relative to day of administration of Artesunate, where the day of dose = 1.

Listing 16.2.1.2
Visit Dates

Randomisation Number	Challenge Agent	Visit	Date of Visit	Day Relative to Inoculum	Day Relative to IP
R108	3D7	Malaria Monitoring	20-NOV-2017	5	-3
		Malaria Monitoring	21-NOV-2017	6	-2
		Malaria Monitoring	22-NOV-2017	7	-1
		Admission	23-NOV-2017	8	1
		Admission 1	24-NOV-2017	9	2
		Admission 2	25-NOV-2017	10	3
		Admission 3	26-NOV-2017	11	4
		Safety Monitoring	26-NOV-2017	11	4
		Safety Monitoring	27-NOV-2017	12	5
		Safety Monitoring	28-NOV-2017	13	6
		Safety Monitoring	29-NOV-2017	14	7
		Safety Monitoring	30-NOV-2017	15	8
		Safety Monitoring	01-DEC-2017	16	9
		Unscheduled	02-DEC-2017	17	10
		Safety Monitoring	03-DEC-2017	18	11
		Safety Monitoring	04-DEC-2017	19	12
		Safety Monitoring	05-DEC-2017	20	13
		Safety Monitoring	07-DEC-2017	22	15
		Safety Monitoring	11-DEC-2017	26	19
		End of Study	14-DEC-2017	29	22
		Unscheduled	18-DEC-2017	33	26
		Unscheduled	21-DEC-2017	36	29
		Unscheduled	08-JAN-2018	54	47
R201	K13	Screening	08-FEB-2018	-11	-20

Notes: Day relative to inoculum is the number of days relative to day of administration of the inoculum, where the day of administration = 0.
The inoculum was administered on Study Day 0 for K13-infected subjects and Study Day 1 for 3D7-infected subjects.
Day relative to IP is the number of days relative to day of administration of Artesunate, where the day of dose = 1.

Printed: 24FEB2020 (Final)

Listing 16.2.1.2
Visit Dates

Randomisation Number	Challenge Agent	Visit	Date of Visit	Day Relative to Inoculum	Day Relative to IP
R201	K13	Unscheduled	13-FEB-2018	-6	-15
		Safety Visit	16-FEB-2018	-3	-12
		Inoculum	19-FEB-2018	0	-9
		Malaria Monitoring	23-FEB-2018	4	-5
		Malaria Monitoring	24-FEB-2018	5	-4
		Malaria Monitoring	25-FEB-2018	6	-3
		Malaria Monitoring	26-FEB-2018	7	-2
		Malaria Monitoring	27-FEB-2018	8	-1
		Admission	28-FEB-2018	9	1
		Admission 1	01-MAR-2018	10	2
		Admission 2	02-MAR-2018	11	3
		Admission 3	03-MAR-2018	12	4
		Safety Monitoring	03-MAR-2018	12	4
		Safety Monitoring	04-MAR-2018	13	5
		Safety Monitoring	05-MAR-2018	14	6
		Safety Monitoring	06-MAR-2018	15	7
		Safety Monitoring	07-MAR-2018	16	8
		Safety Monitoring	08-MAR-2018	17	9
		Safety Monitoring	09-MAR-2018	18	10
		Safety Monitoring	12-MAR-2018	21	13
		Safety Monitoring	14-MAR-2018	23	15
		Safety Monitoring	16-MAR-2018	25	17
		Safety Monitoring	19-MAR-2018	28	20
		End of Study	22-MAR-2018	31	23

Notes: Day relative to inoculum is the number of days relative to day of administration of the inoculum, where the day of administration = 0.
The inoculum was administered on Study Day 0 for K13-infected subjects and Study Day 1 for 3D7-infected subjects.
Day relative to IP is the number of days relative to day of administration of Artesunate, where the day of dose = 1.

Listing 16.2.1.2
Visit Dates

Randomisation Number	Challenge Agent	Visit	Date of Visit	Day Relative to Inoculum	Day Relative to IP
R202	K13	Screening	13-FEB-2018	-6	-15
		Safety Visit	16-FEB-2018	-3	-12
		Unscheduled	17-FEB-2018	-2	-11
		Inoculum	19-FEB-2018	0	-9
		Malaria Monitoring	23-FEB-2018	4	-5
		Malaria Monitoring	24-FEB-2018	5	-4
		Malaria Monitoring	25-FEB-2018	6	-3
		Malaria Monitoring	26-FEB-2018	7	-2
		Malaria Monitoring	27-FEB-2018	8	-1
		Admission	28-FEB-2018	9	1
		Admission 1	01-MAR-2018	10	2
		Admission 2	02-MAR-2018	11	3
		Admission 3	03-MAR-2018	12	4
		Safety Monitoring	03-MAR-2018	12	4
		Safety Monitoring	04-MAR-2018	13	5
		Safety Monitoring	05-MAR-2018	14	6
		Safety Monitoring	06-MAR-2018	15	7
		Safety Monitoring	07-MAR-2018	16	8
		Safety Monitoring	08-MAR-2018	17	9
		Safety Monitoring	09-MAR-2018	18	10
		Safety Monitoring	10-MAR-2018	19	11
		Safety Monitoring	11-MAR-2018	20	12
		Safety Monitoring	12-MAR-2018	21	13
		Safety Monitoring	14-MAR-2018	23	15

Notes: Day relative to inoculum is the number of days relative to day of administration of the inoculum, where the day of administration = 0.
The inoculum was administered on Study Day 0 for K13-infected subjects and Study Day 1 for 3D7-infected subjects.
Day relative to IP is the number of days relative to day of administration of Artesunate, where the day of dose = 1.

Listing 16.2.1.2
Visit Dates

Randomisation Number	Challenge Agent	Visit	Date of Visit	Day Relative to Inoculum	Day Relative to IP
R202	K13	Safety Monitoring	16-MAR-2018	25	17
		Safety Monitoring	19-MAR-2018	28	20
		End of Study	22-MAR-2018	31	23
		Unscheduled	27-MAR-2018	36	28
		Unscheduled	03-APR-2018	43	35
R203	K13	Screening	13-FEB-2018	-6	-15
		Safety Visit	16-FEB-2018	-3	-12
		Inoculum	19-FEB-2018	0	-9
		Malaria Monitoring	23-FEB-2018	4	-5
		Malaria Monitoring	24-FEB-2018	5	-4
		Malaria Monitoring	25-FEB-2018	6	-3
		Malaria Monitoring	26-FEB-2018	7	-2
		Malaria Monitoring	27-FEB-2018	8	-1
		Admission	28-FEB-2018	9	1
		Admission 1	01-MAR-2018	10	2
		Admission 2	02-MAR-2018	11	3
		Admission 3	03-MAR-2018	12	4
		Safety Monitoring	03-MAR-2018	12	4
		Safety Monitoring	04-MAR-2018	13	5
		Safety Monitoring	05-MAR-2018	14	6
		Safety Monitoring	06-MAR-2018	15	7
		Safety Monitoring	07-MAR-2018	16	8
		Safety Monitoring	08-MAR-2018	17	9
		Safety Monitoring	09-MAR-2018	18	10

Notes: Day relative to inoculum is the number of days relative to day of administration of the inoculum, where the day of administration = 0.
The inoculum was administered on Study Day 0 for K13-infected subjects and Study Day 1 for 3D7-infected subjects.
Day relative to IP is the number of days relative to day of administration of Artesunate, where the day of dose = 1.

Listing 16.2.1.2
Visit Dates

Randomisation Number	Challenge Agent	Visit	Date of Visit	Day Relative to Inoculum	Day Relative to IP
R203	K13	Safety Monitoring	10-MAR-2018	19	11
		Safety Monitoring	12-MAR-2018	21	13
		Safety Monitoring	13-MAR-2018	22	14
		Safety Monitoring	14-MAR-2018	23	15
		Safety Monitoring	16-MAR-2018	25	17
		Safety Monitoring	19-MAR-2018	28	20
		End of Study	22-MAR-2018	31	23
		Unscheduled	28-MAR-2018	37	29
		Unscheduled	02-APR-2018	42	34
		Unscheduled	14-APR-2018	54	46
R204	K13	Screening	05-FEB-2018	-14	-23
		Safety Visit	16-FEB-2018	-3	-12
		Inoculum	19-FEB-2018	0	-9
		Malaria Monitoring	20-FEB-2018	1	-8
		Malaria Monitoring	23-FEB-2018	4	-5
		Malaria Monitoring	24-FEB-2018	5	-4
		Malaria Monitoring	25-FEB-2018	6	-3
		Malaria Monitoring	26-FEB-2018	7	-2
		Malaria Monitoring	27-FEB-2018	8	-1
		Admission	28-FEB-2018	9	1
		Admission 1	01-MAR-2018	10	2
		Admission 2	02-MAR-2018	11	3
		Admission 3	03-MAR-2018	12	4
		Safety Monitoring	03-MAR-2018	12	4

Notes: Day relative to inoculum is the number of days relative to day of administration of the inoculum, where the day of administration = 0.
The inoculum was administered on Study Day 0 for K13-infected subjects and Study Day 1 for 3D7-infected subjects.
Day relative to IP is the number of days relative to day of administration of Artesunate, where the day of dose = 1.

Listing 16.2.1.2
Visit Dates

Randomisation Number	Challenge Agent	Visit	Date of Visit	Day Relative to Inoculum	Day Relative to IP
R204	K13	Safety Monitoring	04-MAR-2018	13	5
		Safety Monitoring	05-MAR-2018	14	6
		Safety Monitoring	06-MAR-2018	15	7
		Safety Monitoring	07-MAR-2018	16	8
		Safety Monitoring	08-MAR-2018	17	9
		Safety Monitoring	09-MAR-2018	18	10
		Safety Monitoring	12-MAR-2018	21	13
		Safety Monitoring	14-MAR-2018	23	15
		Safety Monitoring	16-MAR-2018	25	17
		Safety Monitoring	19-MAR-2018	28	20
		End of Study	22-MAR-2018	31	23
R205	K13	Screening	31-JAN-2018	-19	-28
		Unscheduled	06-FEB-2018	-13	-22
		Safety Visit	16-FEB-2018	-3	-12
		Unscheduled	17-FEB-2018	-2	-11
		Inoculum	19-FEB-2018	0	-9
		Malaria Monitoring	23-FEB-2018	4	-5
		Malaria Monitoring	24-FEB-2018	5	-4
		Malaria Monitoring	25-FEB-2018	6	-3
		Malaria Monitoring	26-FEB-2018	7	-2
		Malaria Monitoring	27-FEB-2018	8	-1
		Admission	28-FEB-2018	9	1
		Admission 1	01-MAR-2018	10	2
		Admission 2	02-MAR-2018	11	3

Notes: Day relative to inoculum is the number of days relative to day of administration of the inoculum, where the day of administration = 0.
The inoculum was administered on Study Day 0 for K13-infected subjects and Study Day 1 for 3D7-infected subjects.
Day relative to IP is the number of days relative to day of administration of Artesunate, where the day of dose = 1.

Listing 16.2.1.2
Visit Dates

Randomisation Number	Challenge Agent	Visit	Date of Visit	Day Relative to Inoculum	Day Relative to IP
R205	K13	Admission 3	03-MAR-2018	12	4
		Safety Monitoring	03-MAR-2018	12	4
		Safety Monitoring	04-MAR-2018	13	5
		Safety Monitoring	05-MAR-2018	14	6
		Safety Monitoring	06-MAR-2018	15	7
		Safety Monitoring	07-MAR-2018	16	8
		Safety Monitoring	08-MAR-2018	17	9
		Safety Monitoring	09-MAR-2018	18	10
		Safety Monitoring	12-MAR-2018	21	13
		Safety Monitoring	14-MAR-2018	23	15
		Safety Monitoring	16-MAR-2018	25	17
		Safety Monitoring	19-MAR-2018	28	20
		End of Study	22-MAR-2018	31	23
		Unscheduled	26-MAR-2018	35	27
R206	K13	Screening	06-FEB-2018	-13	-22
		Safety Visit	16-FEB-2018	-3	-12
		Inoculum	19-FEB-2018	0	-9
		Malaria Monitoring	23-FEB-2018	4	-5
		Malaria Monitoring	24-FEB-2018	5	-4
		Malaria Monitoring	25-FEB-2018	6	-3
		Malaria Monitoring	26-FEB-2018	7	-2
		Malaria Monitoring	27-FEB-2018	8	-1
		Admission	28-FEB-2018	9	1
		Admission 1	01-MAR-2018	10	2

Notes: Day relative to inoculum is the number of days relative to day of administration of the inoculum, where the day of administration = 0.
The inoculum was administered on Study Day 0 for K13-infected subjects and Study Day 1 for 3D7-infected subjects.
Day relative to IP is the number of days relative to day of administration of Artesunate, where the day of dose = 1.

Listing 16.2.1.2
Visit Dates

Randomisation Number	Challenge Agent	Visit	Date of Visit	Day Relative to Inoculum	Day Relative to IP
R206	K13	Admission 2	02-MAR-2018	11	3
		Admission 3	03-MAR-2018	12	4
		Safety Monitoring	03-MAR-2018	12	4
		Safety Monitoring	04-MAR-2018	13	5
		Safety Monitoring	05-MAR-2018	14	6
		Safety Monitoring	06-MAR-2018	15	7
		Safety Monitoring	07-MAR-2018	16	8
		Safety Monitoring	08-MAR-2018	17	9
		Safety Monitoring	09-MAR-2018	18	10
		Safety Monitoring	12-MAR-2018	21	13
		Safety Monitoring	14-MAR-2018	23	15
		Safety Monitoring	16-MAR-2018	25	17
		Safety Monitoring	19-MAR-2018	28	20
		End of Study	22-MAR-2018	31	23
		Unscheduled	28-MAR-2018	37	29
R207	K13	Screening	14-FEB-2018	-5	-14
		Safety Visit	16-FEB-2018	-3	-12
		Inoculum	19-FEB-2018	0	-9
		Malaria Monitoring	23-FEB-2018	4	-5
		Malaria Monitoring	24-FEB-2018	5	-4
		Malaria Monitoring	25-FEB-2018	6	-3
		Malaria Monitoring	26-FEB-2018	7	-2
		Malaria Monitoring	27-FEB-2018	8	-1
		Admission	28-FEB-2018	9	1

Notes: Day relative to inoculum is the number of days relative to day of administration of the inoculum, where the day of administration = 0.
The inoculum was administered on Study Day 0 for K13-infected subjects and Study Day 1 for 3D7-infected subjects.
Day relative to IP is the number of days relative to day of administration of Artesunate, where the day of dose = 1.

Listing 16.2.1.2
Visit Dates

Randomisation Number	Challenge Agent	Visit	Date of Visit	Day Relative to Inoculum	Day Relative to IP
R207	K13	Admission 1	01-MAR-2018	10	2
		Admission 2	02-MAR-2018	11	3
		Admission 3	03-MAR-2018	12	4
		Safety Monitoring	03-MAR-2018	12	4
		Safety Monitoring	04-MAR-2018	13	5
		Safety Monitoring	05-MAR-2018	14	6
		Safety Monitoring	06-MAR-2018	15	7
		Safety Monitoring	07-MAR-2018	16	8
		Safety Monitoring	08-MAR-2018	17	9
		Safety Monitoring	09-MAR-2018	18	10
		Safety Monitoring	12-MAR-2018	21	13
		Safety Monitoring	14-MAR-2018	23	15
		Safety Monitoring	16-MAR-2018	25	17
		Safety Monitoring	19-MAR-2018	28	20
		End of Study	22-MAR-2018	31	23
R208	3D7	Screening	01-FEB-2018	-19	-27
		Unscheduled	06-FEB-2018	-14	-22
		Safety Visit	16-FEB-2018	-4	-12
		Inoculum	20-FEB-2018	0	-8
		Malaria Monitoring	24-FEB-2018	4	-4
		Malaria Monitoring	25-FEB-2018	5	-3
		Malaria Monitoring	26-FEB-2018	6	-2
		Malaria Monitoring	27-FEB-2018	7	-1
		Admission	28-FEB-2018	8	1

Notes: Day relative to inoculum is the number of days relative to day of administration of the inoculum, where the day of administration = 0.
The inoculum was administered on Study Day 0 for K13-infected subjects and Study Day 1 for 3D7-infected subjects.
Day relative to IP is the number of days relative to day of administration of Artesunate, where the day of dose = 1.

Listing 16.2.1.2
Visit Dates

Randomisation Number	Challenge Agent	Visit	Date of Visit	Day Relative to Inoculum	Day Relative to IP
R208	3D7	Admission 1	01-MAR-2018	9	2
		Admission 2	02-MAR-2018	10	3
		Admission 3	03-MAR-2018	11	4
		Safety Monitoring	04-MAR-2018	12	5
		Safety Monitoring	05-MAR-2018	13	6
		Safety Monitoring	07-MAR-2018	15	8
		Safety Monitoring	08-MAR-2018	16	9
		Safety Monitoring	09-MAR-2018	17	10
		Safety Monitoring	10-MAR-2018	18	11
		Safety Monitoring	12-MAR-2018	20	13
		Safety Monitoring	13-MAR-2018	21	14
		Safety Monitoring	14-MAR-2018	22	15
		Safety Monitoring	16-MAR-2018	24	17
		Safety Monitoring	19-MAR-2018	27	20
		End of Study	22-MAR-2018	30	23
		Unscheduled	27-MAR-2018	35	28
R209	3D7	Screening	08-FEB-2018	-12	-20
		Safety Visit	16-FEB-2018	-4	-12
		Inoculum	20-FEB-2018	0	-8
		Malaria Monitoring	24-FEB-2018	4	-4
		Malaria Monitoring	25-FEB-2018	5	-3
		Malaria Monitoring	26-FEB-2018	6	-2
		Malaria Monitoring	27-FEB-2018	7	-1
		Admission	28-FEB-2018	8	1

Notes: Day relative to inoculum is the number of days relative to day of administration of the inoculum, where the day of administration = 0.
The inoculum was administered on Study Day 0 for K13-infected subjects and Study Day 1 for 3D7-infected subjects.
Day relative to IP is the number of days relative to day of administration of Artesunate, where the day of dose = 1.

Listing 16.2.1.2
Visit Dates

Randomisation Number	Challenge Agent	Visit	Date of Visit	Day Relative to Inoculum	Day Relative to IP
R209	3D7	Admission 1	01-MAR-2018	9	2
		Admission 2	02-MAR-2018	10	3
		Admission 3	03-MAR-2018	11	4
		Safety Monitoring	04-MAR-2018	12	5
		Safety Monitoring	05-MAR-2018	13	6
		Safety Monitoring	07-MAR-2018	15	8
		Safety Monitoring	08-MAR-2018	16	9
		Safety Monitoring	09-MAR-2018	17	10
		Safety Monitoring	12-MAR-2018	20	13
		Safety Monitoring	14-MAR-2018	22	15
		Safety Monitoring	16-MAR-2018	24	17
		Safety Monitoring	19-MAR-2018	27	20
		End of Study	22-MAR-2018	30	23
R210	3D7	Screening	12-FEB-2018	-8	-16
		Safety Visit	16-FEB-2018	-4	-12
		Unscheduled	17-FEB-2018	-3	-11
		Inoculum	20-FEB-2018	0	-8
		Malaria Monitoring	24-FEB-2018	4	-4
		Malaria Monitoring	25-FEB-2018	5	-3
		Malaria Monitoring	26-FEB-2018	6	-2
		Malaria Monitoring	27-FEB-2018	7	-1
		Admission	28-FEB-2018	8	1
		Admission 1	01-MAR-2018	9	2
		Admission 2	02-MAR-2018	10	3

Notes: Day relative to inoculum is the number of days relative to day of administration of the inoculum, where the day of administration = 0.
The inoculum was administered on Study Day 0 for K13-infected subjects and Study Day 1 for 3D7-infected subjects.
Day relative to IP is the number of days relative to day of administration of Artesunate, where the day of dose = 1.

Listing 16.2.1.2
Visit Dates

Randomisation Number	Challenge Agent	Visit	Date of Visit	Day Relative to Inoculum	Day Relative to IP
R210	3D7	Admission 3	03-MAR-2018	11	4
		Safety Monitoring	04-MAR-2018	12	5
		Safety Monitoring	05-MAR-2018	13	6
		Safety Monitoring	07-MAR-2018	15	8
		Safety Monitoring	08-MAR-2018	16	9
		Safety Monitoring	09-MAR-2018	17	10
		Safety Monitoring	12-MAR-2018	20	13
		Safety Monitoring	14-MAR-2018	22	15
		Safety Monitoring	16-MAR-2018	24	17
		Safety Monitoring	19-MAR-2018	27	20
		End of Study	22-MAR-2018	30	23
R302	K13	Screening	16-AUG-2018	-19	-28
		Safety Visit	03-SEP-2018	-1	-10
		Inoculum	04-SEP-2018	0	-9
		Malaria Monitoring	08-SEP-2018	4	-5
		Malaria Monitoring	09-SEP-2018	5	-4
		Malaria Monitoring	10-SEP-2018	6	-3
		Malaria Monitoring	11-SEP-2018	7	-2
		Malaria Monitoring	12-SEP-2018	8	-1
		Admission	13-SEP-2018	9	1
		Admission 1	14-SEP-2018	10	2
		Admission 2	15-SEP-2018	11	3
		Admission 3	16-SEP-2018	12	4
		Safety Monitoring	16-SEP-2018	12	4

Notes: Day relative to inoculum is the number of days relative to day of administration of the inoculum, where the day of administration = 0.
The inoculum was administered on Study Day 0 for K13-infected subjects and Study Day 1 for 3D7-infected subjects.
Day relative to IP is the number of days relative to day of administration of Artesunate, where the day of dose = 1.

Listing 16.2.1.2
Visit Dates

Randomisation Number	Challenge Agent	Visit	Date of Visit	Day Relative to Inoculum	Day Relative to IP
R302	K13	Safety Monitoring	17-SEP-2018	13	5
		Safety Monitoring	18-SEP-2018	14	6
		Safety Monitoring	19-SEP-2018	15	7
		Safety Monitoring	20-SEP-2018	16	8
		Safety Monitoring	22-SEP-2018	18	10
		Safety Monitoring	24-SEP-2018	20	12
		Safety Monitoring	25-SEP-2018	21	13
		Safety Monitoring	27-SEP-2018	23	15
		Safety Monitoring	28-SEP-2018	24	16
		End of Study	02-OCT-2018	28	20
		Unscheduled	05-OCT-2018	31	23
		Unscheduled	11-OCT-2018	37	29
R303	K13	Screening	09-AUG-2018	-26	-35
		Safety Visit	03-SEP-2018	-1	-10
		Inoculum	04-SEP-2018	0	-9
		Malaria Monitoring	08-SEP-2018	4	-5
		Malaria Monitoring	09-SEP-2018	5	-4
		Malaria Monitoring	10-SEP-2018	6	-3
		Malaria Monitoring	11-SEP-2018	7	-2
		Malaria Monitoring	12-SEP-2018	8	-1
		Admission	13-SEP-2018	9	1
		Admission 1	14-SEP-2018	10	2
		Admission 2	15-SEP-2018	11	3
		Admission 3	16-SEP-2018	12	4

Notes: Day relative to inoculum is the number of days relative to day of administration of the inoculum, where the day of administration = 0.
The inoculum was administered on Study Day 0 for K13-infected subjects and Study Day 1 for 3D7-infected subjects.
Day relative to IP is the number of days relative to day of administration of Artesunate, where the day of dose = 1.

Listing 16.2.1.2
Visit Dates

Randomisation Number	Challenge Agent	Visit	Date of Visit	Day Relative to Inoculum	Day Relative to IP
R303	K13	Safety Monitoring	16-SEP-2018	12	4
		Safety Monitoring	17-SEP-2018	13	5
		Safety Monitoring	18-SEP-2018	14	6
		Safety Monitoring	19-SEP-2018	15	7
		Safety Monitoring	20-SEP-2018	16	8
		Safety Monitoring	22-SEP-2018	18	10
		Safety Monitoring	24-SEP-2018	20	12
		Safety Monitoring	25-SEP-2018	21	13
		Safety Monitoring	27-SEP-2018	23	15
		Safety Monitoring	28-SEP-2018	24	16
		End of Study	02-OCT-2018	28	20
		Unscheduled	05-OCT-2018	31	23
		Unscheduled	11-OCT-2018	37	29
		Unscheduled	18-OCT-2018	44	36
R304	K13	Screening	13-AUG-2018	-22	-31
		Unscheduled	16-AUG-2018	-19	-28
		Safety Visit	03-SEP-2018	-1	-10
		Inoculum	04-SEP-2018	0	-9
		Malaria Monitoring	08-SEP-2018	4	-5
		Malaria Monitoring	09-SEP-2018	5	-4
		Malaria Monitoring	10-SEP-2018	6	-3
		Malaria Monitoring	11-SEP-2018	7	-2
		Malaria Monitoring	12-SEP-2018	8	-1
		Admission	13-SEP-2018	9	1

Notes: Day relative to inoculum is the number of days relative to day of administration of the inoculum, where the day of administration = 0.
The inoculum was administered on Study Day 0 for K13-infected subjects and Study Day 1 for 3D7-infected subjects.
Day relative to IP is the number of days relative to day of administration of Artesunate, where the day of dose = 1.

Listing 16.2.1.2
Visit Dates

Randomisation Number	Challenge Agent	Visit	Date of Visit	Day Relative to Inoculum	Day Relative to IP
R304	K13	Admission 1	14-SEP-2018	10	2
		Admission 2	15-SEP-2018	11	3
		Admission 3	16-SEP-2018	12	4
		Safety Monitoring	16-SEP-2018	12	4
		Safety Monitoring	17-SEP-2018	13	5
		Safety Monitoring	18-SEP-2018	14	6
		Safety Monitoring	19-SEP-2018	15	7
		Safety Monitoring	20-SEP-2018	16	8
		Safety Monitoring	22-SEP-2018	18	10
		Safety Monitoring	24-SEP-2018	20	12
		Safety Monitoring	25-SEP-2018	21	13
		Safety Monitoring	27-SEP-2018	23	15
		Safety Monitoring	28-SEP-2018	24	16
		End of Study	02-OCT-2018	28	20
		Unscheduled	05-OCT-2018	31	23
		Unscheduled	11-OCT-2018	37	29
R305	3D7	Screening	13-AUG-2018	-23	-31
		Unscheduled	20-AUG-2018	-16	-24
		Safety Visit	03-SEP-2018	-2	-10
		Inoculum	05-SEP-2018	0	-8
		Malaria Monitoring	09-SEP-2018	4	-4
		Malaria Monitoring	10-SEP-2018	5	-3
		Malaria Monitoring	11-SEP-2018	6	-2
		Malaria Monitoring	12-SEP-2018	7	-1

Notes: Day relative to inoculum is the number of days relative to day of administration of the inoculum, where the day of administration = 0.
The inoculum was administered on Study Day 0 for K13-infected subjects and Study Day 1 for 3D7-infected subjects.
Day relative to IP is the number of days relative to day of administration of Artesunate, where the day of dose = 1.

Listing 16.2.1.2
Visit Dates

Randomisation Number	Challenge Agent	Visit	Date of Visit	Day Relative to Inoculum	Day Relative to IP
R305	3D7	Admission	13-SEP-2018	8	1
		Admission 1	14-SEP-2018	9	2
		Admission 2	15-SEP-2018	10	3
		Admission 3	16-SEP-2018	11	4
		Safety Monitoring	16-SEP-2018	11	4
		Safety Monitoring	17-SEP-2018	12	5
		Safety Monitoring	18-SEP-2018	13	6
		Safety Monitoring	19-SEP-2018	14	7
		Safety Monitoring	20-SEP-2018	15	8
		Safety Monitoring	21-SEP-2018	16	9
		Safety Monitoring	22-SEP-2018	17	10
		Safety Monitoring	24-SEP-2018	19	12
		Safety Monitoring	25-SEP-2018	20	13
		Safety Monitoring	28-SEP-2018	23	16
		End of Study	02-OCT-2018	27	20
		Unscheduled	04-OCT-2018	29	22
		Unscheduled	10-OCT-2018	35	28
		Unscheduled	17-OCT-2018	42	35
R306	3D7	Screening	28-AUG-2018	-8	-16
		Safety Visit	03-SEP-2018	-2	-10
		Inoculum	05-SEP-2018	0	-8
		Malaria Monitoring	09-SEP-2018	4	-4
		Malaria Monitoring	10-SEP-2018	5	-3
		Malaria Monitoring	11-SEP-2018	6	-2

Notes: Day relative to inoculum is the number of days relative to day of administration of the inoculum, where the day of administration = 0.
The inoculum was administered on Study Day 0 for K13-infected subjects and Study Day 1 for 3D7-infected subjects.
Day relative to IP is the number of days relative to day of administration of Artesunate, where the day of dose = 1.

Listing 16.2.1.2
Visit Dates

Randomisation Number	Challenge Agent	Visit	Date of Visit	Day Relative to Inoculum	Day Relative to IP
R306	3D7	Malaria Monitoring	12-SEP-2018	7	-1
		Admission	13-SEP-2018	8	1
		Admission 1	14-SEP-2018	9	2
		Admission 2	15-SEP-2018	10	3
		Admission 3	16-SEP-2018	11	4
		Safety Monitoring	16-SEP-2018	11	4
		Safety Monitoring	17-SEP-2018	12	5
		Safety Monitoring	18-SEP-2018	13	6
		Safety Monitoring	19-SEP-2018	14	7
		Safety Monitoring	20-SEP-2018	15	8
		Safety Monitoring	21-SEP-2018	16	9
		Safety Monitoring	22-SEP-2018	17	10
		Safety Monitoring	24-SEP-2018	19	12
		Safety Monitoring	25-SEP-2018	20	13
		Safety Monitoring	28-SEP-2018	23	16
		End of Study	02-OCT-2018	27	20
		Unscheduled	08-OCT-2018	33	26
R307	3D7	Screening	07-AUG-2018	-29	-37
		Safety Visit	03-SEP-2018	-2	-10
		Inoculum	05-SEP-2018	0	-8
		Malaria Monitoring	09-SEP-2018	4	-4
		Malaria Monitoring	10-SEP-2018	5	-3
		Malaria Monitoring	11-SEP-2018	6	-2
		Malaria Monitoring	12-SEP-2018	7	-1

Notes: Day relative to inoculum is the number of days relative to day of administration of the inoculum, where the day of administration = 0.
The inoculum was administered on Study Day 0 for K13-infected subjects and Study Day 1 for 3D7-infected subjects.
Day relative to IP is the number of days relative to day of administration of Artesunate, where the day of dose = 1.

Listing 16.2.1.2
Visit Dates

Randomisation Number	Challenge Agent	Visit	Date of Visit	Day Relative to Inoculum	Day Relative to IP
R307	3D7	Admission	13-SEP-2018	8	1
		Admission 1	14-SEP-2018	9	2
		Admission 2	15-SEP-2018	10	3
		Admission 3	16-SEP-2018	11	4
		Safety Monitoring	16-SEP-2018	11	4
		Safety Monitoring	17-SEP-2018	12	5
		Safety Monitoring	18-SEP-2018	13	6
		Safety Monitoring	19-SEP-2018	14	7
		Safety Monitoring	20-SEP-2018	15	8
		Safety Monitoring	21-SEP-2018	16	9
		Safety Monitoring	22-SEP-2018	17	10
		Safety Monitoring	24-SEP-2018	19	12
		Safety Monitoring	25-SEP-2018	20	13
		Safety Monitoring	28-SEP-2018	23	16
		End of Study	02-OCT-2018	27	20
		Unscheduled	05-OCT-2018	30	23
		Unscheduled	11-OCT-2018	36	29
		Unscheduled	18-OCT-2018	43	36

Notes: Day relative to inoculum is the number of days relative to day of administration of the inoculum, where the day of administration = 0.
The inoculum was administered on Study Day 0 for K13-infected subjects and Study Day 1 for 3D7-infected subjects.
Day relative to IP is the number of days relative to day of administration of Artesunate, where the day of dose = 1.

Printed: 24FEB2020 (Final)

16.2.2 Protocol deviations

16.2.2.1 Protocol Deviations

Listing 16.2.2.1
Protocol Deviations

Rando Number	Challenge Agent	PD Number	Date of Deviation	Day Relative to Inoculum	Day Relative to IP	Protocol Deviation Category	Description of Protocol Deviation
R103	K13	1	13-NOV-2017	-1	-10	STUDY SCHEDULE COMPLIANCE	Day -1: A total of 9 participants were required for Cohort 1, at a ratio of 2:1. however, only 6 participants were eligible, thus they were randomised at a 1:1 ratio, with the intention of adding theextra K13 participants in cohort 2.
		2	13-NOV-2017	-1	-10	STUDY SCHEDULE COMPLIANCE	Day -1: additional CRP testing was collected at the PI's discretion in order to investigate the safety and tolerability of the inoculum-inflammation.
		3	19-NOV-2017	5	-4	STUDY SCHEDULE COMPLIANCE	An additional 2 ml PCR sample were collected on Day 5 AM and Day 6 AM in error.
		4	19-NOV-2017	5	-4	STUDY SCHEDULE COMPLIANCE	Day 5 PM - qPCR sample collection for post inoculum malaria monitoring visit was not performed.
		5	21-NOV-2017	7	-2	STUDY SCHEDULE COMPLIANCE	Day 7, afternoon (PM) samples for parasitemia PCR testing were not collect for the above participant as participant did not show for visit. Participant had been previously been advised by the clinic staff and by the sub-investigator.
		6	04-DEC-2017	20	12	STUDY SCHEDULE COMPLIANCE	An additional 4ml blood sample was collected for culturing and approved by the sponsor on Day 20 (admission +11 days)
R104	K13	1	13-NOV-2017	-1	-10	STUDY SCHEDULE COMPLIANCE	Day -1: Additional CRP testing was collected at the PI's discretion in order to investigate the safety and tolerability of the inoculum-inflammation.
		2	13-NOV-2017	-1	-10	STUDY SCHEDULE COMPLIANCE	Day-1: A total of 9 participants were required for cohort 1, at a ratio of 2:1. However only 6 participants were eligible, thus they were randomised at a 1:1 ratio, with the intention of adding the extra K13 participants in cohort 2.

Notes: Protocol Deviation text presented as verbatim.
Day relative to inoculum is the number of days relative to day of administration of the inoculum, where the day of administration = 0.
The inoculum was administered on Study Day 0 for K13-infected subjects and Study Day 1 for 3D7-infected subjects.
Day relative to IP is the number of days relative to day of administration of Artesunate, where the day of dose = 1.
Rando = Randomisation; PD = Protocol Deviation.

Listing 16.2.2.1
Protocol Deviations

Rando Number	Challenge Agent	PD Number	Date of Deviation	Day Relative to Inoculum	Day Relative to IP	Protocol Deviation Category	Description of Protocol Deviation
R104	K13	3	19-NOV-2017	5	-4	STUDY SCHEDULE COMPLIANCE	Additional 2ml PCR samples were collected on Day 5 AM and Day 6 AM in error
		4	19-NOV-2017	5	-4	STUDY SCHEDULE COMPLIANCE	Day 5PM qPCR sample collection was not collected for post malaria monitoring visit.
		5	04-DEC-2017	20	12	STUDY SCHEDULE COMPLIANCE	Day 20: An additional 4ml blood sample was collected for culturing and approved by the sponsor.
R105	K13	1	13-NOV-2017	-1	-10	STUDY SCHEDULE COMPLIANCE	A total of 9 participants were required for cohort 1, at a ratio of 2:1. However only 6 participants were eligible, thus they were randomised at a 1:1 ratio, with the intention of adding the extra K13participants in Cohort 2.
		2	13-NOV-2017	-1	-10	STUDY SCHEDULE COMPLIANCE	Additional CRP testing was collected at the PI's discretion in order to investigate the safety and tolerability of the inoculum- information
		3	19-NOV-2017	5	-4	STUDY SCHEDULE COMPLIANCE	Additional 2ml PCR samples were collected on Day 5 AM and Day 6 AM in error
		4	19-NOV-2017	5	-4	STUDY SCHEDULE COMPLIANCE	Day 5PM qPCR sample collection was not collected for post malaria monitoring visit.
		5	27-NOV-2017	13	5	STUDY SCHEDULE COMPLIANCE	Staff failed to record diastolic blood pressure result for Day 13 visit.
		6	01-DEC-2017	17	9	STUDY SCHEDULE COMPLIANCE	An extra 4ml sample was collected for culturing and approved by the Sponsor on Admission + 8 days- Day 17

Notes: Protocol Deviation text presented as verbatim.
Day relative to inoculum is the number of days relative to day of administration of the inoculum, where the day of administration = 0.
The inoculum was administered on Study Day 0 for K13-infected subjects and Study Day 1 for 3D7-infected subjects.
Day relative to IP is the number of days relative to day of administration of Artesunate, where the day of dose = 1.
Rando = Randomisation; PD = Protocol Deviation.

Listing 16.2.2.1
Protocol Deviations

Rando Number	Challenge Agent	PD Number	Date of Deviation	Day Relative to Inoculum	Day Relative to IP	Protocol Deviation Category	Description of Protocol Deviation
R106	3D7	1	09-NOV-2017	-6	-14	STUDY SCHEDULE COMPLIANCE	Clinic staff failed to report respiratory rate at screening. However the respiratory rate was checked pre-inoculum
		2	13-NOV-2017	-2	-10	STUDY SCHEDULE COMPLIANCE	Day -1 Additional CRP testing was collected at teh IP's discretion in order to investigate the safety and tolerability of the inoculum-inflammation.
		3	13-NOV-2017	-2	-10	STUDY SCHEDULE COMPLIANCE	Day -1- A total of 9 participants were required for cohort 1, at a ratio of 2:1. However, only 6 participants were eligible, thus they were randomised at a 1:1 ratio, with the intention of adding theextra K13 participants in Cohort 2.
		4	20-NOV-2017	5	-3	STUDY SCHEDULE COMPLIANCE	Day 6- Additional 2ml PCR samples were collected on the Day 6, morning AM in error.
R107	3D7	1	13-NOV-2017	-2	-10	STUDY SCHEDULE COMPLIANCE	Day -1: A total of 9 participants were required for cohort 1, at a ratio of 2:1. however, only 6 participants were eligible, thus they were randomised at a 1:1 ratio, with the intention of adding theextra K13 participants in cohort 2.
		2	13-NOV-2017	-2	-10	STUDY SCHEDULE COMPLIANCE	Day -1: Additional CRP testing was collected at the PI's discretion in order to investigate the safety and tolerability of the inoculum-inflammation.
		3	20-NOV-2017	5	-3	STUDY SCHEDULE COMPLIANCE	Day 6: Additional 2 ml PCR samples were collected on Day 6 AM in error.
		4	05-DEC-2017	20	13	STUDY SCHEDULE COMPLIANCE	Day 3 Post PPQ dosing: Staff failed to take bloods within the protocol specified window. safety bloods were subsequently taken at 7 days post-dosing. This constitutes a deviation from the protocol.

Notes: Protocol Deviation text presented as verbatim.
Day relative to inoculum is the number of days relative to day of administration of the inoculum, where the day of administration = 0.
The inoculum was administered on Study Day 0 for K13-infected subjects and Study Day 1 for 3D7-infected subjects.
Day relative to IP is the number of days relative to day of administration of Artesunate, where the day of dose = 1.
Rando = Randomisation; PD = Protocol Deviation.

Listing 16.2.2.1
Protocol Deviations

Rando Number	Challenge Agent	PD Number	Date of Deviation	Day Relative to Inoculum	Day Relative to IP	Protocol Deviation Category	Description of Protocol Deviation
R108	3D7	1	13-NOV-2017	-2	-10	STUDY SCHEDULE COMPLIANCE	A total of 9 participants were required for Cohort 1, at a ratio of 2:1. However, only 6 participants were eligible, thus they were randomised at a 1:1 ratio, with the intention of adding the extra K13 participants in Cohort 2.
		2	13-NOV-2017	-2	-10	STUDY SCHEDULE COMPLIANCE	Additonal CRP testing was collected at the PI's discretion in order to investigate the safety and tolerability of the inoculum-inflammation.
		3	20-NOV-2017	5	-3	STUDY SCHEDULE COMPLIANCE	Day 6 additional 2ml PCR samples were collected on the Day 6 AM in error
		4	23-NOV-2017	8	1	STUDY SCHEDULE COMPLIANCE	Day 9 - Ad per HREC approved protocol (V1.1 dated 11th Aug 2017) 6 hour PK sample due at 16:01 was not collected until 16:12 due to cannula failure thus requiring venepuncture. This constitutes a deviation from protocol.
		5	01-DEC-2017	16	9	STUDY SCHEDULE COMPLIANCE	Admission + 8 days An additional 4ml blood was taken for culturing in error. participant and subinvestigator were made aware at the time and sample was destroyed prior to processing.
		6	01-DEC-2017	16	9	STUDY SCHEDULE COMPLIANCE	Day 17 (admission +8 days). Participants due for dosing with piperaquine phosphate which required 4 hours fasting before and after dose. clinic staff provided participants with snack prior to dosing and participant had one bite before staff realised and removed food. SubInvestigator informed at time of incident and requested to continue with dosing.

Notes: Protocol Deviation text presented as verbatim.
Day relative to inoculum is the number of days relative to day of administration of the inoculum, where the day of administration = 0.
The inoculum was administered on Study Day 0 for K13-infected subjects and Study Day 1 for 3D7-infected subjects.
Day relative to IP is the number of days relative to day of administration of Artesunate, where the day of dose = 1.
Rando = Randomisation; PD = Protocol Deviation.

Listing 16.2.2.1
Protocol Deviations

Rando Number	Challenge Agent	PD Number	Date of Deviation	Day Relative to Inoculum	Day Relative to IP	Protocol Deviation Category	Description of Protocol Deviation
R201	K13	1	28-FEB-2018	9	1	STUDY SCHEDULE COMPLIANCE	As per HREC approved protocol (v2.0 dated 25 January 2018), vital signs including respiratory rate are to be recorded on Day 9 in the evening. At the Day 9 evening vital signs testing, staff failed torecord respiratory rate result. This constitutes a deviation from protocol.
		2	02-MAR-2018	11	3	STUDY SCHEDULE COMPLIANCE	Day 11 - As per HREC approved protocol (v2.0 25th Jan 2018), piperaquine dosing for K13 participants was to be conducted on Day 11 AM (scheduled for the 2nd March 2018). Artesunate dosing was delayedon day of admission due to issues regarding a participant’s safety which required the sub investigator to consult a Cardiology Specialist prior to giving drug to participant 101-R209. This delayed artesunate dosing of all participants until the afternoon. Therefore, Day 11 piperaquine dosing was delayed until the afternoon of 2nd Mar 2018 for participants 101-R201 to 101-R207 (instead of Day 11 AMas written in the protocol). This constitutes a deviation from the protocol

Notes: Protocol Deviation text presented as verbatim.
Day relative to inoculum is the number of days relative to day of administration of the inoculum, where the day of administration = 0.
The inoculum was administered on Study Day 0 for K13-infected subjects and Study Day 1 for 3D7-infected subjects.
Day relative to IP is the number of days relative to day of administration of Artesunate, where the day of dose = 1.
Rando = Randomisation; PD = Protocol Deviation.

Listing 16.2.2.1
Protocol Deviations

Rando Number	Challenge Agent	PD Number	Date of Deviation	Day Relative to Inoculum	Day Relative to IP	Protocol Deviation Category	Description of Protocol Deviation
R201	K13	3	02-MAR-2018	11	3	STUDY SCHEDULE COMPLIANCE	Day 11 - As per HREC approved protocol (v2.0 25th Jan 2018),18S qPCR samples were to be collected following treatment at time points 32 and 36 hours post artesunate treatment. Cannula failed at +32 hour malaria 18S qPCR time point. Nursing staff attempted venepuncture 3 times and were unsuccessful. Sub-Investigator contacted and attended clinic. +32 hour sample collected late after second venepuncture attempt by Sub-I at 22:40. Sub-Investigator advised staff that if they were unable to collect 36 hour sample after two venepuncture attempts not to proceed with any further attempts and for the sample to be classified as a missed sample. The participant 101-R201 36 hour malaria 18S qPCR sample due for collection at 01:22 2nd Mar 2018 was unable to be collected, as staff had two attempts at venepuncture. Therefore sample not collected. This constitutes a deviation from protocol.
R202	K13	1	02-MAR-2018	11	3	STUDY SCHEDULE COMPLIANCE	As per HREC approved protocol (v2.0 25th Jan 2018), piperaquine dosing for K13 participants was to be conducted on Day 11 AM (scheduled for the 2nd March 2018). Artesunate dosing was delayed on day ofadmission due to issues regarding a participant’s safety which required the sub investigator to consult a Cardiology Specialist prior to giving drug to participant 101-R209. This delayed artesunate dosing of all participants until the afternoon. Therefore, Day 11 piperaquine dosing was delayed until the afternoon of 2nd Mar 2018 for participants 101-R201 to 101-R207 (instead of Day 11 AM as written in the protocol). This constitutes a deviation from the protocol.

Notes: Protocol Deviation text presented as verbatim.
Day relative to inoculum is the number of days relative to day of administration of the inoculum, where the day of administration = 0.
The inoculum was administered on Study Day 0 for K13-infected subjects and Study Day 1 for 3D7-infected subjects.
Day relative to IP is the number of days relative to day of administration of Artesunate, where the day of dose = 1.
Rando = Randomisation; PD = Protocol Deviation.

Listing 16.2.2.1
Protocol Deviations

Rando Number	Challenge Agent	PD Number	Date of Deviation	Day Relative to Inoculum	Day Relative to IP	Protocol Deviation Category	Description of Protocol Deviation
R203	K13	1	02-MAR-2018	11	3	STUDY SCHEDULE COMPLIANCE	As per HREC approved protocol (v2.0 25th Jan 2018), piperaquine dosing for K13 participants was to be conducted on Day 11 AM (scheduled for the 2nd March 2018). Artesunate dosing was delayed on day ofadmission due to issues regarding a participant’s safety which required the sub investigator to consult a Cardiology Specialist prior to giving drug to participant 101-R209. This delayed artesunate dosing of all participants until the afternoon. Therefore, Day 11 piperaquine dosing was delayed until the afternoon of 2nd Mar 2018 for participants 101-R201 to 101-R207 (instead of Day 11 AM as written in the protocol). This constitutes a deviation from the protocol.
		2	10-MAR-2018	19	11	STUDY SCHEDULE COMPLIANCE	As per HREC approved protocol (v2.0 25th Jan 2018). Source documents state Drug Response sample is due Pre-Piperaquine and Pre-and Post-Primacin, at Investigators discretion. Participant 101-R203 had 2ml Drug Response sample collected on 10th March 2018; Day 19 (Protocol states Day 23±3 prior to Primacin treatment), taken a day earlier at PI’s discretion due to needing to treat the participantearlier with malarone. Primacin treatment occurred 14th March 2018; Day 23 therefore Drug Response sample collected out of window. This is a protocol deviation as it was collected earlier than the protocol allowed.

Notes: Protocol Deviation text presented as verbatim.
Day relative to inoculum is the number of days relative to day of administration of the inoculum, where the day of administration = 0.
The inoculum was administered on Study Day 0 for K13-infected subjects and Study Day 1 for 3D7-infected subjects.
Day relative to IP is the number of days relative to day of administration of Artesunate, where the day of dose = 1.
Rando = Randomisation; PD = Protocol Deviation.

Listing 16.2.2.1
Protocol Deviations

Rando Number	Challenge Agent	PD Number	Date of Deviation	Day Relative to Inoculum	Day Relative to IP	Protocol Deviation Category	Description of Protocol Deviation
R204	K13	1	02-MAR-2018	11	3	STUDY SCHEDULE COMPLIANCE	Day 11 - As per HREC approved protocol (v2.0 25th Jan 2018), piperaquine dosing for K13 participants was to be conducted on Day 11 AM (scheduled for the 2nd March 2018). Artesunate dosing was delayedon day of admission due to issues regarding a participant’s safety which required the sub investigator to consult a Cardiology Specialist prior to giving drug to participant 101-R209. This delayed artesunate dosing of all participants until the afternoon. Therefore, Day 11 piperaquine dosing was delayed until the afternoon of 2nd Mar 2018 for participants 101-R201 to 101-R207 (instead of Day 11 AMas written in the protocol). This constitutes a deviation from the protocol.
		2	03-MAR-2018	12	4	STUDY SCHEDULE COMPLIANCE	As per HREC approved protocol (V2.0 25 Jan 2018) on day: Adm+3, discharge, a symptom-directed physical examination needs to be performed for a participants if symptoms are present. On day: Adm + 3 days, discharge, a symptom-directed physical examination was not completed on participant 101-R204. The participant had a symptom which was also considered an adverse event (AE). This constitutes a deviation from the protocol. Prior to discharge (Friday 2/3/18), the Clinical Trial Coordinator discussed with the PI and Sub-Investigator the availability of doctors for the physical exam on discharge for Day Adm + 3 (3/3/18). The PI and Sub-Investigator were away at the time of discharge. Arrangements were made for the PI to be contacted via phone if required and the Medical Director to be on call.The participant was assessed by two senior registered nurses prior to discharge. The AE present was bruising/haematoma to left arm and right arm, which was mild, had not worsened and was previously present from 20th Feb 2018 until 21st Mar 2018 (AE’s resolved). At the time of discharge, this was deemed mild with no safety concerns present. The participant returned in the evening for a scheduled blood sample collection and was reassessed by the Clinical Trial Coordinator. No safety concerns were present.
		3	22-MAR-2018	31	23	STUDY SCHEDULE COMPLIANCE	Day 31 - As per HREC approved protocol (V2.0 dated 25 January 2018), PE was performed by Sub Investigator at EOS visit (Day 31) on the 22 March 2018 as per the protocol requirements. Sub Investigatorincorrectly documented the date as 28/3/18 instead of the correct date. The Sub Investigator confirmed the date of the PE and made the correction in the source documents.

Notes: Protocol Deviation text presented as verbatim.
Day relative to inoculum is the number of days relative to day of administration of the inoculum, where the day of administration = 0.
The inoculum was administered on Study Day 0 for K13-infected subjects and Study Day 1 for 3D7-infected subjects.
Day relative to IP is the number of days relative to day of administration of Artesunate, where the day of dose = 1.
Rando = Randomisation; PD = Protocol Deviation.

Listing 16.2.2.1
Protocol Deviations

Rando Number	Challenge Agent	PD Number	Date of Deviation	Day Relative to Inoculum	Day Relative to IP	Protocol Deviation Category	Description of Protocol Deviation
R205	K13	1	02-MAR-2018	11	3	STUDY SCHEDULE COMPLIANCE	Day 11 - As per HREC approved protocol (v2.0 25th Jan 2018), piperaquine dosing for K13 participants was to be conducted on Day 11 AM (scheduled for the 2nd March 2018). Artesunate dosing was delayedon day of admission due to issues regarding a participant's safety which required the sub investigator to consult a Cardiology Specialist prior to giving drug to participant 101-R209. This delayed artesunate dosing of all participants until the afternoon. Therefore, Day 11 piperaquine dosing was delayed until the afternoon of 2nd Mar 2018 for participants 101-R201 to 101-R207 (instead of Day 11 AMas written in the protocol). This constitutes a deviation from the protocol.

Notes: Protocol Deviation text presented as verbatim.
Day relative to inoculum is the number of days relative to day of administration of the inoculum, where the day of administration = 0.
The inoculum was administered on Study Day 0 for K13-infected subjects and Study Day 1 for 3D7-infected subjects.
Day relative to IP is the number of days relative to day of administration of Artesunate, where the day of dose = 1.
Rando = Randomisation; PD = Protocol Deviation.

Listing 16.2.2.1
Protocol Deviations

Rando Number	Challenge Agent	PD Number	Date of Deviation	Day Relative to Inoculum	Day Relative to IP	Protocol Deviation Category	Description of Protocol Deviation
R206	K13	1	02-MAR-2018	11	3	STUDY SCHEDULE COMPLIANCE	Day 11 AM As per HREC approved protocol (v2.0 25th Jan 2018), piperaquine dosing for K13 participants was to be conducted on Day 11 AM (scheduled for the 2nd March 2018). Artesunate dosing was delayedon day of admission due to issues regarding a participant’s safety which required the sub investigator to consult a Cardiology Specialist prior to giving drug to participant 101-R209. This delayed artesunate dosing of all participants until the afternoon. Therefore, Day 11 piperaquine dosing was delayed until the afternoon of 2nd Mar 2018 for participants 101-R201 to 101-R207 (instead of Day 11 AM as written in the protocol). This constitutes a deviation from the protocol.

Notes: Protocol Deviation text presented as verbatim.
Day relative to inoculum is the number of days relative to day of administration of the inoculum, where the day of administration = 0.
The inoculum was administered on Study Day 0 for K13-infected subjects and Study Day 1 for 3D7-infected subjects.
Day relative to IP is the number of days relative to day of administration of Artesunate, where the day of dose = 1.
Rando = Randomisation; PD = Protocol Deviation.

Printed: 24FEB2020 (Final)

Listing 16.2.2.1
Protocol Deviations

Rando Number	Challenge Agent	PD Number	Date of Deviation	Day Relative to Inoculum	Day Relative to IP	Protocol Deviation Category	Description of Protocol Deviation
R207	K13	1	02-MAR-2018	11	3	STUDY SCHEDULE COMPLIANCE	Day 11- As per HREC approved protocol (v2.0 25th Jan 2018), piperaquine dosing for K13 participants was to be conducted on Day 11 AM (scheduled for the 2nd March 2018). Artesunate dosing was delayed on day of admission due to issues regarding a participant's safety which required the sub investigator to consult a Cardiology Specialist prior to giving drug to participant 101-R209. This delayed artesunate dosing of all participants until the afternoon. Therefore, Day 11 piperaquine dosing was delayed until the afternoon of 2nd Mar 2018 for participants 101-R201 to 101-R207 (instead of Day 11 AMas written in the protocol). This constitutes a deviation from the protocol.
		2	03-MAR-2018	12	4	STUDY SCHEDULE COMPLIANCE	As per HREC approved protocol (v2.0 25th Jan 2018) on Day: Adm + 3, discharge, a symptom-directed physical examination needs to be performed for a participant if symptoms are present. On Day: Adm +3, discharge, a symptom-directed physical examination (PE) was not completed on Participant 101-R207. The Participant had a symptom which was considered an adverse event (AE). This constitutes a deviation from the protocol. Prior to discharge (Friday 2/3/18), the Clinical Trial Coordinator discussed with the PI and Sub Investigator the availability of doctors for the PE on discharge for Day Adm+ 3 (3/3/18). The PI and Sub-Investigator were away at the time of discharge. Arrangements were made for the PI to be contacted via phone if required and the Medical Director to be on call. The Participant was assessed by two senior registered nurses prior to discharge. The AE present was arthralgia to one finger joint, which was reported by the participant as very mild. At the time of discharge, there were no safety concerns present. The Participant returned to the clinic in the evening and was reassessed by the Clinical Trial Coordinator. The Participant reported that the AE had resolved. No safety concerns were present.
		3	04-MAR-2018	13	5	STUDY SCHEDULE COMPLIANCE	Day 13 - Staff failed to record position start time for vitals although ticked that participant had been resting in seated position for 5 minutes in source.

Notes: Protocol Deviation text presented as verbatim.
Day relative to inoculum is the number of days relative to day of administration of the inoculum, where the day of administration = 0.
The inoculum was administered on Study Day 0 for K13-infected subjects and Study Day 1 for 3D7-infected subjects.
Day relative to IP is the number of days relative to day of administration of Artesunate, where the day of dose = 1.
Rando = Randomisation; PD = Protocol Deviation.

Listing 16.2.2.1
Protocol Deviations

Rando Number	Challenge Agent	PD Number	Date of Deviation	Day Relative to Inoculum	Day Relative to IP	Protocol Deviation Category	Description of Protocol Deviation
R208	3D7	1	04-MAR-2018	12	5	STUDY SCHEDULE COMPLIANCE	As per HREC approved protocol (v2.0 25 Jan 2018), an Admission + 84 hr malaria 18s qPCR sample was to be collected from all participants (scheduled for the afternoon of the 3rd Mar 2018). This samplewas not collected for participant 101-R208. This was because the Admission + 72 hr qPCR time-point was delayed due to a delay in Artesunate dosing for all participants. The 72 hr time-point was collected in the early afternoon on 3rd Mar. Therefore, the 84 hr qPCR samples were due around 11 PM to 3 AM on the 3rd - 4th Mar 2018 (depending on the participant). It was decided by the Sub Investigatornot to ask the participants to visit the clinic at these late night times, so the samples were not collected. This constitutes a deviation from protocol. The next malaria 18s qPCR sample for these participants was collected between 0700-1000 hrs the day after discharge, 4th Mar 2018, and these samples were labelled as the Admission + 96 hr time-point. This deviation means that there is one less data point for the 3D7 participants. However, the parasitaemia levels were already low at Admission + 72 hr, and the participants were brought in to the clinic the next morning for the 96 hr time-point. Therefore, this protocol deviation did not affect the safety of the participants. A study statistician confirmed that the missed time-point did not affect calculation of the parasite reduction ratioand therefore did not have a significant impact on the study data.
		2	22-MAR-2018	30	23	STUDY SCHEDULE COMPLIANCE	Day 31 - As per HREC approved protocol (V2.0 dated 25 January 2018), PE was performed by Sub Investigator at EOS visit (Day 31) on the 22 March 2018 as per the protocol requirements. Sub Investigatorperformed the PE on the correct date (22/3/18), but forgot to complete the source document. The Sub Investigator confirmed the date of the PE and made the correction in the source documents.

Notes: Protocol Deviation text presented as verbatim.
Day relative to inoculum is the number of days relative to day of administration of the inoculum, where the day of administration = 0.
The inoculum was administered on Study Day 0 for K13-infected subjects and Study Day 1 for 3D7-infected subjects.
Day relative to IP is the number of days relative to day of administration of Artesunate, where the day of dose = 1.
Rando = Randomisation; PD = Protocol Deviation.

Listing 16.2.2.1
Protocol Deviations

Rando Number	Challenge Agent	PD Number	Date of Deviation	Day Relative to Inoculum	Day Relative to IP	Protocol Deviation Category	Description of Protocol Deviation
R209	3D7	1	03-MAR-2018	11	4	STUDY SCHEDULE COMPLIANCE	As per HREC approved protocol (v2.0 25 Jan 2018), an Admission + 84 hr malaria 18s qPCR sample was to be collected from all participants (scheduled for the afternoon of the 3rd Mar 2018). This samplewas not collected for participant 101-R209. This was because the Admission + 72 hr qPCR time-point was delayed due to a delay in Artesunate dosing for all participants. The 72 hr time-point was collected in the early afternoon on 3rd Mar. Therefore, the 84 hr qPCR samples were due around 11 PM to 3 AM on the 3rd - 4th Mar 2018 (depending on the participant). It was decided by the Sub Investigatornot to ask the participants to visit the clinic at these late night times, so the samples were not collected. This constitutes a deviation from protocol. The next malaria 18s qPCR sample for these participants was collected between 0700-1000 hrs the day after discharge, 4th Mar 2018, and these samples were labelled as the Admission + 96 hr time-point. This deviation means that there is one less data point for the 3D7 participants. However, the parasitaemia levels were already low at Admission + 72 hr, and the participants were brought in to the clinic the next morning for the 96 hr time-point. Therefore, this protocol deviation did not affect the safety of the participants. A study statistician confirmed that the missed time-point did not affect calculation of the parasite reduction ratioand therefore did not have a significant impact on the study data.

Notes: Protocol Deviation text presented as verbatim.
Day relative to inoculum is the number of days relative to day of administration of the inoculum, where the day of administration = 0.
The inoculum was administered on Study Day 0 for K13-infected subjects and Study Day 1 for 3D7-infected subjects.
Day relative to IP is the number of days relative to day of administration of Artesunate, where the day of dose = 1.
Rando = Randomisation; PD = Protocol Deviation.

Listing 16.2.2.1
Protocol Deviations

Rando Number	Challenge Agent	PD Number	Date of Deviation	Day Relative to Inoculum	Day Relative to IP	Protocol Deviation Category	Description of Protocol Deviation
		2	03-MAR-2018	11	4	STUDY SCHEDULE COMPLIANCE	As per HREC approved protocol (v2.0 25th Jan 2018) on Day: Adm + 3, discharge, a symptom-directed physical examination needs to be performed for a participant if symptoms are present. On Day: Adm + 3,discharge, a symptom-directed physical examination (PE) was not completed on Participant 101-R209. The Participant had symptoms which were also considered adverse events (AEs). This constitutes a deviation from the protocol. Prior to discharge (02/03/18), the clinical trial coordinator discussed with the PI and Sub-Investigator the availability of doctors for the PE on discharge for Day Adm + 3(03/03/18). The PI and Sub-Investigator were away at the time of discharge. Arrangements were made for the PI to be contacted via phone if required and the Medical Director to be on call. The participant was assessed by two senior registered nurses prior to discharge. The symptoms present were a mild intermittent headache and mild intermittent myalgia which were previously present, from the 1st Mar 2018 until resolved on 13th Mar 2018. At the time of discharge, both symptoms were mild and intermittent and not present at time of discharge. There were no safety concerns to the participant. Theparticipant was discharged in the afternoon at 1600hrs and returned to the clinic the next morning for the scheduled blood sample collection. The participant was reassessed by the Clinical Trial Coordinator the following morning. No safety concerns were present.

Notes: Protocol Deviation text presented as verbatim.
Day relative to inoculum is the number of days relative to day of administration of the inoculum, where the day of administration = 0.
The inoculum was administered on Study Day 0 for K13-infected subjects and Study Day 1 for 3D7-infected subjects.
Day relative to IP is the number of days relative to day of administration of Artesunate, where the day of dose = 1.
Rando = Randomisation; PD = Protocol Deviation.

Listing 16.2.2.1
Protocol Deviations

Rando Number	Challenge Agent	PD Number	Date of Deviation	Day Relative to Inoculum	Day Relative to IP	Protocol Deviation Category	Description of Protocol Deviation
R210	3D7	1	04-MAR-2018	12	5	STUDY SCHEDULE COMPLIANCE	As per HREC approved protocol (v2.0 25 Jan 2018), an Admission + 84 hr malaria 18s qPCR sample was to be collected from all participants (scheduled for the afternoon of the 3rd Mar 2018). This samplewas not collected for participant 101-R210. This was because the Admission + 72 hr qPCR time-point was delayed due to a delay in Artesunate dosing for all participants. The 72 hr time-point was collected in the early afternoon on 3rd Mar. Therefore, the 84 hr qPCR samples were due around 11 PM to 3 AM on the 3rd - 4th Mar 2018 (depending on the participant). It was decided by the Sub Investigatornot to ask the participants to visit the clinic at these late night times, so the samples were not collected. This constitutes a deviation from protocol. The next malaria 18s qPCR sample for these participants was collected between 0700-1000 hrs the day after discharge, 4th Mar 2018, and these samples were labelled as the Admission + 96 hr time-point. This deviation means that there is one less data point for the 3D7 participants. However, the parasitaemia levels were already low at Admission + 72 hr, and the participants were brought in to the clinic the next morning for the 96 hr time-point. Therefore, this protocol deviation did not affect the safety of the participants. A study statistician confirmed that the missed time-point did not affect calculation of the parasite reduction ratioand therefore did not have a significant impact on the study data.
R302	K13	1	25-SEP-2018	21	13	STUDY SCHEDULE COMPLIANCE	Subject had concomitant medication (Novasone cream) usage recorded as PRN in error. This constitutes a protocol deviation as the protocol asks for the exact dose and timing of each dose to be recorded
		2	26-SEP-2018	22	14	STUDY SCHEDULE COMPLIANCE	All subjects started a 3-day course of Malarone on 25 SEP 2018. As per HREC approved protocol (V3.0 dated 23 JUL 2018), subjects were due to be monitored either in the clinic or by phone for the 3 days to ensure adherence to the Malarone therapy. All subjects were dosed in the clinic on 25 SEP 2018, so staff at the clinic monitored the first dose administration. Subjects were sent home with theremaining two doses and instructed when and how to take them. No subjects were required to attend the clinic the next day (26 SEP 2018), and in error, no subjects were contacted by phone to confirmthey had taken the drug that day. This constitutes a deviation from the protocol.

Notes: Protocol Deviation text presented as verbatim.
Day relative to inoculum is the number of days relative to day of administration of the inoculum, where the day of administration = 0.
The inoculum was administered on Study Day 0 for K13-infected subjects and Study Day 1 for 3D7-infected subjects.
Day relative to IP is the number of days relative to day of administration of Artesunate, where the day of dose = 1.
Rando = Randomisation; PD = Protocol Deviation.

Listing 16.2.2.1
Protocol Deviations

Rando Number	Challenge Agent	PD Number	Date of Deviation	Day Relative to Inoculum	Day Relative to IP	Protocol Deviation Category	Description of Protocol Deviation
R303	K13	1	15-SEP-2018	11	3	STUDY SCHEDULE COMPLIANCE	Day 11 (in-patient) - Midday clinical score not performed.
		2	25-SEP-2018	21	13	STUDY SCHEDULE COMPLIANCE	Subject had concomitant medication (Novasone cream) usage recorded as PRN in error. This constitutes a protocol deviation as the protocol asks for the exact dose and timing of each dose to be recorded
		3	26-SEP-2018	22	14	STUDY SCHEDULE COMPLIANCE	All subjects started a 3-day course of Malarone on 25 SEP 2018. As per HREC approved protocol (V3.0 dated 23 JUL 2018), subjects were due to be monitored either in the clinic or by phone for the 3 days to ensure adherence to the Malarone therapy. All subjects were dosed in the clinic on 25 SEP 2018, so staff at the clinic monitored the first dose administration. Subjects were sent home with theremaining two doses and instructed when and how to take them. No subjects were required to attend the clinic the next day (26 SEP 2018), and in error, no subjects were contacted by phone to confirmthey had taken the drug that day. This constitutes a deviation from the protocol.
R304	K13	1	12-SEP-2018	8	-1	STUDY SCHEDULE COMPLIANCE	Day 8 PM - Subject was not seated for at least 5 minutes prior to having vital signs taken.
		2	26-SEP-2018	22	14	STUDY SCHEDULE COMPLIANCE	All subjects started a 3-day course of Malarone on 25 SEP 2018. As per HREC approved protocol (V3.0 dated 23 JUL 2018), subjects were due to be monitored either in the clinic or by phone for the 3 days to ensure adherence to the Malarone therapy. All subjects were dosed in the clinic on 25 SEP 2018, so staff at the clinic monitored the first dose administration. Subjects were sent home with theremaining two doses and instructed when and how to take them. No subjects were required to attend the clinic the next day (26 SEP 2018), and in error, no subjects were contacted by phone to confirmthey had taken the drug that day. This constitutes a deviation from the protocol.

Notes: Protocol Deviation text presented as verbatim.
Day relative to inoculum is the number of days relative to day of administration of the inoculum, where the day of administration = 0.
The inoculum was administered on Study Day 0 for K13-infected subjects and Study Day 1 for 3D7-infected subjects.
Day relative to IP is the number of days relative to day of administration of Artesunate, where the day of dose = 1.
Rando = Randomisation; PD = Protocol Deviation.

Listing 16.2.2.1
Protocol Deviations

Rando Number	Challenge Agent	PD Number	Date of Deviation	Day Relative to Inoculum	Day Relative to IP	Protocol Deviation Category	Description of Protocol Deviation
R305	3D7	1	19-SEP-2018	14	7	STUDY SCHEDULE COMPLIANCE	Day 15 PM - the time of dosing for Piperaquine was not recorded.
		2	25-SEP-2018	20	13	STUDY SCHEDULE COMPLIANCE	Subject had concomitant medication (Systane lubricant eye drops, and paracetamol) usage recorded as PRN in error. This constitutes a protocol deviation as the protocol asks for the exact dose and timing of each dose to be recorded.
		3	26-SEP-2018	21	14	STUDY SCHEDULE COMPLIANCE	All subjects started a 3-day course of Malarone on 25 SEP 2018. As per HREC approved protocol (V3.0 dated 23 JUL 2018), subjects were due to be monitored either in the clinic or by phone for the 3 days to ensure adherence to the Malarone therapy. All subjects were dosed in the clinic on 25 SEP 2018, so staff at the clinic monitored the first dose administration. Subjects were sent home with theremaining two doses and instructed when and how to take them. No subjects were required to attend the clinic the next day (26 SEP 2018), and in error, no subjects were contacted by phone to confirmthey had taken the drug that day. This constitutes a deviation from the protocol.
		4	27-SEP-2018	22	15	STUDY SCHEDULE COMPLIANCE	All subjects started a 3-day course of Malarone on 25 SEP 2018. As per HREC approved protocol (V3.0 dated 23 JUL 2018), subjects were due to be monitored either in the clinic or by phone for the 3 days to ensure adherence to the Malarone therapy. All subjects were dosed in the clinic on 25 SEP 2018, so staff at the clinic monitored the first dose administration. Subjects were sent home with theremaining two doses and instructed when and how to take them. Subject was not required to attend the clinic on 27 SEP 2018, and in error they were not contacted with specific regards to dosing compliance (they were contacted about their next visit to the clinic). This constitutes a deviation from the protocol.

Notes: Protocol Deviation text presented as verbatim.
Day relative to inoculum is the number of days relative to day of administration of the inoculum, where the day of administration = 0.
The inoculum was administered on Study Day 0 for K13-infected subjects and Study Day 1 for 3D7-infected subjects.
Day relative to IP is the number of days relative to day of administration of Artesunate, where the day of dose = 1.
Rando = Randomisation; PD = Protocol Deviation.

Listing 16.2.2.1
Protocol Deviations

Rando Number	Challenge Agent	PD Number	Date of Deviation	Day Relative to Inoculum	Day Relative to IP	Protocol Deviation Category	Description of Protocol Deviation
R306	3D7	1	12-SEP-2018	7	-1	STUDY SCHEDULE COMPLIANCE	Day 8 PM - subject was not seated for at least 5 minutes prior to having vital signs taken.
		2	16-SEP-2018	11	4	STUDY SCHEDULE COMPLIANCE	Day 12 PM - subject was not seated for at least 5 minutes prior to having vital signs taken.
		3	19-SEP-2018	14	7	STUDY SCHEDULE COMPLIANCE	Day 15 PM - the time of dosing for Piperaquine was not recorded.
		4	26-SEP-2018	21	14	STUDY SCHEDULE COMPLIANCE	All subjects started a 3-day course of Malarone on 25 SEP 2018. As per HREC approved protocol (V3.0 dated 23 JUL 2018), subjects were due to be monitored either in the clinic or by phone for the 3 days to ensure adherence to the Malarone therapy. All subjects were dosed in the clinic on 25 SEP 2018, so staff at the clinic monitored the first dose administration. Subjects were sent home with theremaining two doses and instructed when and how to take them. No subjects were required to attend the clinic the next day (26 SEP 2018), and in error, no subjects were contacted by phone to confirmthey had taken the drug that day. This constitutes a deviation from the protocol.

Notes: Protocol Deviation text presented as verbatim.
Day relative to inoculum is the number of days relative to day of administration of the inoculum, where the day of administration = 0.
The inoculum was administered on Study Day 0 for K13-infected subjects and Study Day 1 for 3D7-infected subjects.
Day relative to IP is the number of days relative to day of administration of Artesunate, where the day of dose = 1.
Rando = Randomisation; PD = Protocol Deviation.

Listing 16.2.2.1
Protocol Deviations

Rando Number	Challenge Agent	PD Number	Date of Deviation	Day Relative to Inoculum	Day Relative to IP	Protocol Deviation Category	Description of Protocol Deviation
R306	3D7	5	27-SEP-2018	22	15	STUDY SCHEDULE COMPLIANCE	All subjects started a 3-day course of Malarone on 25 SEP 2018. As per HREC approved protocol (V3.0 dated 23 JUL 2018), subjects were due to be monitored either in the clinic or by phone for the 3 days to ensure adherence to the Malarone therapy. All subjects were dosed in the clinic on 25 SEP 2018, so staff at the clinic monitored the first dose administration. Subjects were sent home with theremaining two doses and instructed when and how to take them. Subject was not required to attend the clinic on 27 SEP 2018, and in error they were not contacted with specific regards to dosing compliance (they were contacted about their next visit to the clinic). This constitutes a deviation from the protocol.

Notes: Protocol Deviation text presented as verbatim.
Day relative to inoculum is the number of days relative to day of administration of the inoculum, where the day of administration = 0.
The inoculum was administered on Study Day 0 for K13-infected subjects and Study Day 1 for 3D7-infected subjects.
Day relative to IP is the number of days relative to day of administration of Artesunate, where the day of dose = 1.
Rando = Randomisation; PD = Protocol Deviation.

Listing 16.2.2.1
Protocol Deviations

Rando Number	Challenge Agent	PD Number	Date of Deviation	Day Relative to Inoculum	Day Relative to IP	Protocol Deviation Category	Description of Protocol Deviation
R307	3D7	1	19-SEP-2018	14	7	STUDY SCHEDULE COMPLIANCE	Day 15 PM: The time of dosing for Piperaquine was not recorded.
		2	26-SEP-2018	21	14	STUDY SCHEDULE COMPLIANCE	All subjects started a 3-day course of Malarone on 25 SEP 2018. As per HREC approved protocol (V3.0 dated 23 JUL 2018), subjects were due to be monitored either in the clinic or by phone for the 3 days to ensure adherence to the Malarone therapy. All subjects were dosed in the clinic on 25 SEP 2018, so staff at the clinic monitored the first dose administration. Subjects were sent home with theremaining two doses and instructed when and how to take them. No subjects were required to attend the clinic the next day (26 SEP 2018), and in error, no subjects were contacted by phone to confirmthey had taken the drug that day. This constitutes a deviation from the protocol.
		3	27-SEP-2018	22	15	STUDY SCHEDULE COMPLIANCE	All subjects started a 3-day course of Malarone on 25 SEP 2018. As per HREC approved protocol (V3.0 dated 23 JUL 2018), subjects were due to be monitored either in the clinic or by phone for the 3 days to ensure adherence to the Malarone therapy. All subjects were dosed in the clinic on 25 SEP 2018, so staff at the clinic monitored the first dose administration. Subjects were sent home with theremaining two doses and instructed when and how to take them. Subject was not required to attend the clinic on 27 SEP 2018, and in error they were not contacted with specific regards to dosing compliance (they were contacted about their next visit to the clinic). This constitutes a deviation from the protocol.

Notes: Protocol Deviation text presented as verbatim.
Day relative to inoculum is the number of days relative to day of administration of the inoculum, where the day of administration = 0.
The inoculum was administered on Study Day 0 for K13-infected subjects and Study Day 1 for 3D7-infected subjects.
Day relative to IP is the number of days relative to day of administration of Artesunate, where the day of dose = 1.
Rando = Randomisation; PD = Protocol Deviation.

16.2.3 Subjects excluded from the efficacy analysis

16.2.3.1 Analysis Population Assignment

Listing 16.2.3.1
Analysis Population Assignment

Randomisation Number	Challenge Agent	Safety Population
R103	K13	Yes
R104	K13	Yes
R105	K13	Yes
R106	3D7	Yes
R107	3D7	Yes
R108	3D7	Yes
R201	K13	Yes
R202	K13	Yes
R203	K13	Yes
R204	K13	Yes
R205	K13	Yes
R206	K13	Yes
R207	K13	Yes
R208	3D7	Yes
R209	3D7	Yes
R210	3D7	Yes
R302	K13	Yes
R303	K13	Yes
R304	K13	Yes
R305	3D7	Yes
R306	3D7	Yes
R307	3D7	Yes

16.2.4 Demographic and baseline data

16.2.4.1 Demography

Listing 16.2.4.1
Demography

Randomisation Number	Challenge Agent	Date of Birth	Age (years)	Sex	Race	Ethnicity
R103	K13	05-JAN-1999	18	Male	White	Not Hispanic or Latino
R104	K13	14-AUG-1990	27	Male	Native Hawaiian or Other Pacific Islander	Not Hispanic or Latino
R105	K13	24-DEC-1998	18	Male	White	Not Hispanic or Latino
R106	3D7	25-JAN-1989	28	Female	White	Not Hispanic or Latino
R107	3D7	19-SEP-1991	26	Male	Asian	Not Hispanic or Latino
R108	3D7	21-MAY-1992	25	Male	White	Not Hispanic or Latino
R201	K13	23-AUG-1991	26	Female	White	Not Hispanic or Latino
R202	K13	14-NOV-1995	22	Male	White	Not Hispanic or Latino
R203	K13	30-APR-1995	22	Female	White	Not Hispanic or Latino
R204	K13	12-MAR-1984	33	Female	White	Not Hispanic or Latino
R205	K13	20-OCT-1992	25	Female	White	Not Hispanic or Latino
R206	K13	29-DEC-1977	40	Female	Other: Native South American	Hispanic or Latino
R207	K13	17-NOV-1996	21	Male	White	Not Hispanic or Latino
R208	3D7	23-AUG-1987	30	Female	White	Not Hispanic or Latino
R209	3D7	30-MAR-1989	28	Male	White	Not Hispanic or Latino
R210	3D7	14-SEP-1995	22	Female	White	Not Hispanic or Latino
R302	K13	29-JUN-1997	21	Male	White	Not Hispanic or Latino
R303	K13	11-MAR-1991	27	Female	White	Not Hispanic or Latino
R304	K13	21-AUG-1995	22	Male	White	Not Hispanic or Latino
R305	3D7	26-AUG-1985	32	Male	Asian	Not Hispanic or Latino
R306	3D7	18-APR-1983	35	Male	White	Not Hispanic or Latino
R307	3D7	04-DEC-1991	26	Female	White	Not Hispanic or Latino

Notes: Age is that at Screening visit.

16.2.4.2 Inclusion and Exclusion Criteria

Listing 16.2.4.2
Inclusion and Exclusion Criteria

Randomisation Number	Challenge Agent	Visit	Date of Assessment	Subject Meets All Inclusion/Exclusion Criteria?	If Not, Criterion Subject Did Not Meet
R103	K13	Screening	01-NOV-2017	Yes	
		Inoculum	14-NOV-2017	Yes	
R104	K13	Screening	09-NOV-2017	Yes	
		Inoculum	14-NOV-2017	Yes	
R105	K13	Screening	08-NOV-2017	Yes	
		Inoculum	14-NOV-2017	Yes	
R106	3D7	Screening	09-NOV-2017	Yes	
		Inoculum	15-NOV-2017	Yes	
R107	3D7	Screening	03-NOV-2017	Yes	
		Inoculum	15-NOV-2017	Yes	
R108	3D7	Screening	26-OCT-2017	Yes	
		Inoculum	15-NOV-2017	Yes	
R201	K13	Screening	08-FEB-2018	Yes	
		Inoculum	19-FEB-2018	Yes	
R202	K13	Screening	13-FEB-2018	Yes	
		Inoculum	19-FEB-2018	Yes	
R203	K13	Screening	13-FEB-2018	Yes	
		Inoculum	19-FEB-2018	Yes	
R204	K13	Screening	05-FEB-2018	Yes	
		Inoculum	19-FEB-2018	Yes	
R205	K13	Screening	31-JAN-2018	Yes	
		Inoculum	19-FEB-2018	Yes	
R206	K13	Screening	06-FEB-2018	Yes	
		Inoculum	19-FEB-2018	Yes	
R207	K13	Screening	14-FEB-2018	Yes	
		Inoculum	19-FEB-2018	Yes	

Listing 16.2.4.2
Inclusion and Exclusion Criteria

Randomisation Number	Challenge Agent	Visit	Date of Assessment	Subject Meets All Inclusion/Exclusion Criteria?	If Not, Criterion Subject Did Not Meet
R208	3D7	Screening	01-FEB-2018	Yes	
		Inoculum	20-FEB-2018	Yes	
R209	3D7	Screening	08-FEB-2018	Yes	
		Inoculum	20-FEB-2018	Yes	
R210	3D7	Screening	12-FEB-2018	Yes	
		Inoculum	20-FEB-2018	Yes	
R302	K13	Screening	16-AUG-2018	Yes	
		Inoculum	04-SEP-2018	Yes	
R303	K13	Screening	09-AUG-2018	Yes	
		Inoculum	04-SEP-2018	Yes	
R304	K13	Screening	13-AUG-2018	Yes	
		Inoculum	04-SEP-2018	Yes	
R305	3D7	Screening	13-AUG-2018	Yes	
		Inoculum	05-SEP-2018	Yes	
R306	3D7	Screening	28-AUG-2018	Yes	
		Inoculum	05-SEP-2018	Yes	
R307	3D7	Screening	07-AUG-2018	Yes	
		Inoculum	05-SEP-2018	Yes	

16.2.4.3 Medical History

Listing 16.2.4.3
Medical History

Randomisation Number	Challenge Agent	Medical History Number	System Organ Class/ Preferred Term/ Medical Condition Verbatim	Date of Diagnosis	Date of Resolution
R103	K13	1	Skin and subcutaneous tissue disorders/ Eczema/ eczema	UN-UNK-2000	UN-UNK-2007
R104	K13	1	Infections and infestations/ Otitis media/ Otitis media	16-OCT-2017	23-OCT-2017
R106	3D7	1	Immune system disorders/ Mite allergy/ Allergy to dust mites	UN-UNK-2001	Ongoing
		2	Immune system disorders/ Seasonal allergy/ Hayfever (chronic)	UN-UNK-2001	Ongoing
R107	3D7	1	Psychiatric disorders/ Depression/ Depression	UN-JUN-2011	UN-JUN-2012
		2	Injury, poisoning and procedural complications/ Lumbar vertebral fracture/ Lumbar (single) vertebral fracture	01-JUL-2017	UN-SEP-2017
		3	Injury, poisoning and procedural complications/ Foot fracture/ Right calcaneum fracture	01-JUL-2017	UN-SEP-2017
R108	3D7	1	Skin and subcutaneous tissue disorders/ Eczema/ Eczema	UN-UNK-1996	UN-UNK-2000

Notes: Medical history was coded to system organ class and preferred term using MedDRA Version 20.1.
UN = Unknown; UNK = Unknown; UNKK = Unknown.

Printed: 24FEB2020 (Final)

Listing 16.2.4.3
Medical History

Randomisation Number	Challenge Agent	Medical History Number	System Organ Class/ Preferred Term/ Medical Condition Verbatim	Date of Diagnosis	Date of Resolution
R108	3D7	2	Immune system disorders/ Seasonal allergy/ Hayfever	UN-UNK-1999	Ongoing
		3	Gastrointestinal disorders/ Gastrooesophageal reflux disease/ Gastro oesophageal reflux disease	UN-UNK-2016	Ongoing
R201	K13	1	General disorders and administration site conditions/ Temperature intolerance/ Air conditioning sensitivity	UN-UNK-2001	Ongoing
		2	Surgical and medical procedures/ Cholecystectomy/ Cholecystectomy	UN-UNK-2011	UN-UNK-2011
		3	Hepatobiliary disorders/ Cholelithiasis/ Gallstones	UN-UNK-2011	UN-UNK-2011
		4	Gastrointestinal disorders/ Obstructive pancreatitis/ Pancreatitis secondary gallstones	UN-UNK-2011	UN-UNK-2011
R202	K13	1	Immune system disorders/ Seasonal allergy/ Hayfever	UN-UNK-2008	Ongoing
R203	K13	1	Nervous system disorders/ Migraine/ Migraine	UN-UNK-2006	Ongoing

Notes: Medical history was coded to system organ class and preferred term using MedDRA Version 20.1.
UN = Unknown; UNK = Unknown; UNKK = Unknown.

Listing 16.2.4.3
Medical History

Randomisation Number	Challenge Agent	Medical History Number	System Organ Class/ Preferred Term/ Medical Condition Verbatim	Date of Diagnosis	Date of Resolution
R203	K13	2	Investigations/ Laparoscopy/ Laparoscopy	UN-NOV-2017	UN-NOV-2017
		3	Injury, poisoning and procedural complications/ Injury/ Stabbed by knife via accident	UN-NOV-2017	UN-NOV-2017
R204	K13	1	Immune system disorders/ Allergy to animal/ Cat allergy	UN-UNK-1997	Ongoing
		2	Nervous system disorders/ Restless legs syndrome/ Restless legs	UN-UNK-2002	Ongoing
		3	Psychiatric disorders/ Perinatal depression/ Post natal depression	UN-NOV-2003	UN-FEB-2004
		4	Gastrointestinal disorders/ Toothache/ Toothache	03-FEB-2018	03-FEB-2018
		5	Nervous system disorders/ Headache/ Headache	05-FEB-2018	05-FEB-2018
		6	Reproductive system and breast disorders/ Dysmenorrhoea/ Dysmenorrhoea	10-FEB-2018	10-FEB-2018
R205	K13	1	Immune system disorders/ Allergy to animal/ Cat allergy	UN-UNK-1997	Ongoing

Notes: Medical history was coded to system organ class and preferred term using MedDRA Version 20.1.
UN = Unknown; UNK = Unknown; UNKK = Unknown.

Listing 16.2.4.3
Medical History

Randomisation Number	Challenge Agent	Medical History Number	System Organ Class/ Preferred Term/ Medical Condition Verbatim	Date of Diagnosis	Date of Resolution
R205	K13	2	Immune system disorders/ Seasonal allergy/ Hayfever mild	UN-UNK-1997	Ongoing
		3	Injury, poisoning and procedural complications/ Wrist fracture/ Left wrist fracture	UN-UNK-2001	UN-UNK-2001
		4	Injury, poisoning and procedural complications/ Wrist fracture/ Right wrist fracture	UN-UNK-2003	UN-UNK-2003
		5	Skin and subcutaneous tissue disorders/ Acne/ Acne	UN-UNK-2007	UN-UNK-2009
R206	K13	1	Reproductive system and breast disorders/ Dysmenorrhoea/ Dysmenorrhoea	UN-UNK-2002	Ongoing
		2	Surgical and medical procedures/ Skin operation/ Skin reduction surgery following weight loss	UN-UNK-2004	UN-UNK-2004
		3	Surgical and medical procedures/ Caesarean section/ caesarean section	UN-UNK-2013	UN-UNK-2013
		4	Nervous system disorders/ Headache/ headache	02-FEB-2018	02-FEB-2018
		5	Nervous system disorders/ Headache/ headache	17-FEB-2018	17-FEB-2018

Notes: Medical history was coded to system organ class and preferred term using MedDRA Version 20.1.
UN = Unknown; UNK = Unknown; UNKK = Unknown.

Listing 16.2.4.3
Medical History

Randomisation Number	Challenge Agent	Medical History Number	System Organ Class/ Preferred Term/ Medical Condition Verbatim	Date of Diagnosis	Date of Resolution
R208	3D7	1	Immune system disorders/ Seasonal allergy/ Hayfever	UN-UNK-2000	Ongoing
R209	3D7	1	Immune system disorders/ Allergy to arthropod sting/ Bee sting allergy	UN-UNK-1999	Ongoing
		2	Immune system disorders/ Seasonal allergy/ Hayfever	UN-UNK-2001	Ongoing
		3	Respiratory, thoracic and mediastinal disorders/ Asthma/ Asthma	UN-UNK-2001	UN-UNK-2004
R210	3D7	1	Nervous system disorders/ Headache/ headache	17-FEB-2018	17-FEB-2018
R302	K13	1	Skin and subcutaneous tissue disorders/ Acne/ Acne	UN-UNK-2012	UN-UNK-2014
R303	K13	1	Vascular disorders/ Varicose vein/ Single varicose vein left leg	UN-DEC-2012	Ongoing

Notes: Medical history was coded to system organ class and preferred term using MedDRA Version 20.1.
UN = Unknown; UNK = Unknown; UNKK = Unknown.

Listing 16.2.4.3
Medical History

Randomisation Number	Challenge Agent	Medical History Number	System Organ Class/ Preferred Term/ Medical Condition Verbatim	Date of Diagnosis	Date of Resolution
R304	K13	1	Infections and infestations/ Pharyngitis/ Recurrent pharyngitis	UN-UNK-2002	UN-UNK-2003
		2	Surgical and medical procedures/ Tonsillectomy/ Tonsillectomy	UN-UNK-2003	UN-UNK-2003
		3	Skin and subcutaneous tissue disorders/ Acne/ Acne	UN-UNK-2009	UN-UNK-2010
		4	Infections and infestations/ Upper respiratory tract infection/ Upper respiratory tract infection	29-JUL-2018	29-AUG-2018
R305	3D7	1	Skin and subcutaneous tissue disorders/ Eczema/ Eczema	UN-UNK-1997	UN-UNK-2005
		2	Nervous system disorders/ Migraine/ Migraine	UN-UNK-1999	Ongoing
		3	Immune system disorders/ Seasonal allergy/ Hayfever	UN-UNK-2013	Ongoing
		4	Eye disorders/ Dry eye/ Dry eyes	UN-AUG-2017	Ongoing
R307	3D7	1	Injury, poisoning and procedural complications/ Ligament rupture/ Left anterior cruciate ligament rupture	UN-DEC-2015	UN-DEC-2015

Notes: Medical history was coded to system organ class and preferred term using MedDRA Version 20.1.
UN = Unknown; UNK = Unknown; UNKK = Unknown.

Printed: 24FEB2020 (Final)

Listing 16.2.4.3
Medical History

Randomisation Number	Challenge Agent	Medical History Number	System Organ Class/ Preferred Term/ Medical Condition Verbatim	Date of Diagnosis	Date of Resolution
R307	3D7	2	Metabolism and nutrition disorders/ Lactose intolerance/ Lactose intolerance mild	UN-UNK-2016	Ongoing
		3	Surgical and medical procedures/ Ligament operation/ Left anterior cruciate ligament repair	UN-SEP-2016	UN-SEP-2016
		4	Nervous system disorders/ Headache/ Headache	02-SEP-2018	02-SEP-2018

Notes: Medical history was coded to system organ class and preferred term using MedDRA Version 20.1.
UN = Unknown; UNK = Unknown; UNKK = Unknown.

16.2.4.4 Pregnancy Test

Listing 16.2.4.4
Pregnancy Test

Randomisation Number	Challenge Agent	Visit	Day Relative to Inoculum	Day Relative to IP	Sample Type	Test Performed?	Collection Date	Collection Time	Result
R106	3D7	Screening	-6	-14	Serum	Yes	09-NOV-2017	10:57	Negative
		Inoculum	0	-8	Urine	Yes	15-NOV-2017	07:55	Negative
		Admission	8	1	Urine	Yes	23-NOV-2017	08:59	Negative
		End of Study	29	22	Urine	Yes	14-DEC-2017	08:43	Negative
R201	K13	Screening	-11	-20	Serum	Yes	08-FEB-2018	12:01	Negative
		Inoculum	0	-9	Urine	Yes	19-FEB-2018	08:16	Negative
		Admission	9	1	Urine	Yes	28-FEB-2018	07:50	Negative
		End of Study	31	23	Urine	Yes	22-MAR-2018	08:06	Negative
R203	K13	Screening	-6	-15	Serum	Yes	13-FEB-2018	10:18	Negative
		Inoculum	0	-9	Urine	Yes	19-FEB-2018	08:18	Negative
		Admission	9	1	Urine	Yes	28-FEB-2018	07:18	Negative
		End of Study	31	23	Urine	Yes	22-MAR-2018	09:43	Negative
R204	K13	Screening	-14	-23	Serum	Yes	05-FEB-2018	10:46	Negative
		Inoculum	0	-9	Urine	Yes	19-FEB-2018	08:18	Negative
		Admission	9	1	Urine	Yes	28-FEB-2018	07:48	Negative
		End of Study	31	23	Urine	Yes	22-MAR-2018	08:45	Negative
R205	K13	Screening	-19	-28	Serum	Yes	31-JAN-2018	10:01	Negative
		Inoculum	0	-9	Urine	Yes	19-FEB-2018	08:19	Negative
		Admission	9	1	Urine	Yes	28-FEB-2018	07:20	Negative
		End of Study	31	23	Urine	Yes	22-MAR-2018	07:03	Negative

Notes: Day relative to inoculum is the number of days relative to day of administration of the inoculum, where the day of administration = 0.
The inoculum was administered on Study Day 0 for K13-infected subjects and Study Day 1 for 3D7-infected subjects.
Day relative to IP is the number of days relative to day of administration of Artesunate, where the day of dose = 1.

Listing 16.2.4.4
Pregnancy Test

Randomisation Number	Challenge Agent	Visit	Day Relative to Inoculum	Day Relative to IP	Sample Type	Test Performed?	Collection Date	Collection Time	Result
R206	K13	Screening	-13	-22	Serum	Yes	06-FEB-2018	13:32	Negative
		Inoculum	0	-9	Urine	Yes	19-FEB-2018	08:32	Negative
		Admission	9	1	Urine	Yes	28-FEB-2018	08:32	Negative
		End of Study	31	23	Urine	Yes	22-MAR-2018	08:56	Negative
R208	3D7	Screening	-19	-27	Serum	Yes	01-FEB-2018	12:12	Negative
		Inoculum	0	-8	Urine	Yes	20-FEB-2018	08:10	Negative
		Admission	8	1	Urine	Yes	28-FEB-2018	06:59	Negative
		End of Study	30	23	Urine	Yes	22-MAR-2018	07:00	Negative
R210	3D7	Screening	-8	-16	Serum	Yes	12-FEB-2018	09:38	Negative
		Inoculum	0	-8	Urine	Yes	20-FEB-2018	08:41	Negative
		Admission	8	1	Urine	Yes	28-FEB-2018	07:40	Negative
		End of Study	30	23	Urine	Yes	22-MAR-2018	07:02	Negative
R303	K13	Screening	-26	-35	Serum	Yes	09-AUG-2018	10:30	Negative
		Inoculum	0	-9	Urine	Yes	04-SEP-2018	07:33	Negative
		Admission	9	1	Urine	Yes	13-SEP-2018	07:23	Negative
		End of Study	28	20	Urine	Yes	02-OCT-2018	08:27	Negative
R307	3D7	Screening	-29	-37	Serum	Yes	07-AUG-2018	12:31	Negative
		Inoculum	0	-8	Urine	Yes	05-SEP-2018	08:28	Negative
		Admission	8	1	Urine	Yes	13-SEP-2018	07:39	Negative
		End of Study	27	20	Urine	Yes	02-OCT-2018	07:37	Negative

Notes: Day relative to inoculum is the number of days relative to day of administration of the inoculum, where the day of administration = 0.
The inoculum was administered on Study Day 0 for K13-infected subjects and Study Day 1 for 3D7-infected subjects.
Day relative to IP is the number of days relative to day of administration of Artesunate, where the day of dose = 1.

Printed: 24FEB2020 (Final)

16.2.4.5 Follicle Stimulating Hormone (FSH) Test

Listing 16.2.4.5
Follicle Stimulating Hormone (FSH) Test

No Follicle Stimulating Hormone data reported.

16.2.4.6 RBC Alloantibody

Listing 16.2.4.6
RBC Alloantibody

Randomisation Number	Challenge Agent	Visit	Date of Sample Collection	Time of Sample Collection	Day Relative to Inoculum	Day Relative to IP	Result
R103	K13	Screening	01-NOV-2017	12:16	-13	-22	Negative
		End of Study	14-DEC-2017	09:37	30	22	Negative
R104	K13	Screening	09-NOV-2017	10:32	-5	-14	Negative
		End of Study	14-DEC-2017	08:42	30	22	Negative
R105	K13	Screening	08-NOV-2017	12:46	-6	-15	Negative
		End of Study	13-DEC-2017	08:54	29	21	Negative
R106	3D7	Screening	09-NOV-2017	10:57	-6	-14	Negative
		End of Study	14-DEC-2017	08:53	29	22	Negative
R107	3D7	Screening	03-NOV-2017	11:25	-12	-20	Negative
		End of Study	14-DEC-2017	09:31	29	22	Negative
R108	3D7	Screening	26-OCT-2017	09:30	-20	-28	Negative
		End of Study	14-DEC-2017	08:37	29	22	Negative
R201	K13	Screening	08-FEB-2018	12:01	-11	-20	Negative
		End of Study	22-MAR-2018	08:31	31	23	Negative
R202	K13	Screening	13-FEB-2018	11:20	-6	-15	Negative
		End of Study	22-MAR-2018	10:00	31	23	Negative
R203	K13	Screening	13-FEB-2018	10:18	-6	-15	Negative
		End of Study	22-MAR-2018	10:14	31	23	Negative

Notes: Day relative to inoculum is the number of days relative to day of administration of the inoculum, where the day of administration = 0.
The inoculum was administered on Study Day 0 for K13-infected subjects and Study Day 1 for 3D7-infected subjects.
Day relative to IP is the number of days relative to day of administration of Artesunate, where the day of dose = 1.

Listing 16.2.4.6
RBC Alloantibody

Randomisation Number	Challenge Agent	Visit	Date of Sample Collection	Time of Sample Collection	Day Relative to Inoculum	Day Relative to IP	Result
R204	K13	Screening	05-FEB-2018	10:46	-14	-23	Negative
		End of Study	22-MAR-2018	08:43	31	23	Negative
R205	K13	Screening	31-JAN-2018	10:01	-19	-28	Negative
		End of Study	22-MAR-2018	07:30	31	23	Negative
R206	K13	Screening	06-FEB-2018	13:32	-13	-22	Negative
		End of Study	22-MAR-2018	09:19	31	23	Negative
R207	K13	Screening	14-FEB-2018	11:34	-5	-14	Negative
		End of Study	22-MAR-2018	09:32	31	23	Negative
R208	3D7	Screening	01-FEB-2018	12:12	-19	-27	Negative
		End of Study	22-MAR-2018	07:50	30	23	Negative
R209	3D7	Screening	08-FEB-2018	10:36	-12	-20	Negative
		End of Study	22-MAR-2018	08:16	30	23	Negative
R210	3D7	Screening	12-FEB-2018	09:38	-8	-16	Negative
		End of Study	22-MAR-2018	07:34	30	23	Negative
R302	K13	Screening	16-AUG-2018	09:36	-19	-28	Negative
		End of Study	02-OCT-2018	08:19	28	20	Negative
R303	K13	Screening	09-AUG-2018	10:30	-26	-35	Negative
		End of Study	02-OCT-2018	09:05	28	20	Negative

Notes: Day relative to inoculum is the number of days relative to day of administration of the inoculum, where the day of administration = 0.
The inoculum was administered on Study Day 0 for K13-infected subjects and Study Day 1 for 3D7-infected subjects.
Day relative to IP is the number of days relative to day of administration of Artesunate, where the day of dose = 1.

Listing 16.2.4.6
RBC Alloantibody

Randomisation Number	Challenge Agent	Visit	Date of Sample Collection	Time of Sample Collection	Day Relative to Inoculum	Day Relative to IP	Result
R304	K13	Screening	13-AUG-2018	11:57	-22	-31	Negative
		End of Study	02-OCT-2018	08:46	28	20	Negative
R305	3D7	Screening	13-AUG-2018	09:38	-23	-31	Negative
		End of Study	02-OCT-2018	07:50	27	20	Negative
R306	3D7	Screening	28-AUG-2018	11:39	-8	-16	Negative
		End of Study	02-OCT-2018	08:20	27	20	Negative
R307	3D7	Screening	07-AUG-2018	12:31	-29	-37	Negative
		End of Study	02-OCT-2018	07:21	27	20	Negative

Notes: Day relative to inoculum is the number of days relative to day of administration of the inoculum, where the day of administration = 0.
The inoculum was administered on Study Day 0 for K13-infected subjects and Study Day 1 for 3D7-infected subjects.
Day relative to IP is the number of days relative to day of administration of Artesunate, where the day of dose = 1.

16.2.4.7 Serology

Listing 16.2.4.7
Serology

Randomisation Number	Challenge Agent	Visit	Date of Sample Collection	Time of Sample Collection	Day Relative to Inoculum	Day Relative to IP	Parameter	Result
R103	K13	Screening	01-NOV-2017	12:16	-13	-22	HIV-1/2 Antibody	Negative
							Hepatitis B Virus Core Antibody	Negative
							Hepatitis B Virus Surface Antigen	Negative
							Hepatitis C Virus Antibody	Negative
		End Of Study	14-DEC-2017	09:37	30	22	HIV-1/2 Antibody	Negative
							Hepatitis B Virus Core Antibody	Negative
							Hepatitis B Virus Surface Antigen	Negative
							Hepatitis C Virus Antibody	Negative
R104	K13	Screening	09-NOV-2017	10:32	-5	-14	HIV-1/2 Antibody	Negative
							Hepatitis B Virus Core Antibody	Negative
							Hepatitis B Virus Surface Antigen	Negative
							Hepatitis C Virus Antibody	Negative
		End Of Study	14-DEC-2017	08:42	30	22	HIV-1/2 Antibody	Negative
							Hepatitis B Virus Core Antibody	Negative
							Hepatitis B Virus Surface Antigen	Negative
							Hepatitis C Virus Antibody	Positive
		Unscheduled	21-DEC-2017	07:35	37	29	Hepatitis C Virus Antibody	Positive
							Hepatitis C Virus RNA	Negative
R105	K13	Screening	08-NOV-2017	12:46	-6	-15	HIV-1/2 Antibody	Negative
							Hepatitis B Virus Core Antibody	Negative
							Hepatitis B Virus Surface Antigen	Negative
							Hepatitis C Virus Antibody	Negative

Notes: Day relative to inoculum is the number of days relative to day of administration of the inoculum, where the day of administration = 0.
The inoculum was administered on Study Day 0 for K13-infected subjects and Study Day 1 for 3D7-infected subjects.
Day relative to IP is the number of days relative to day of administration of Artesunate, where the day of dose = 1.

Listing 16.2.4.7
Serology

Randomisation Number	Challenge Agent	Visit	Date of Sample Collection	Time of Sample Collection	Day Relative to Inoculum	Day Relative to IP	Parameter	Result
R105	K13	End Of Study	13-DEC-2017	08:54	29	21	HIV-1/2 Antibody	Negative
							Hepatitis B Virus Core Antibody	Negative
							Hepatitis B Virus Surface Antigen	Negative
							Hepatitis C Virus Antibody	Negative
R106	3D7	Screening	09-NOV-2017	10:57	-6	-14	HIV-1/2 Antibody	Negative
							Hepatitis B Virus Core Antibody	Negative
							Hepatitis B Virus Surface Antigen	Negative
							Hepatitis C Virus Antibody	Negative
		End Of Study	14-DEC-2017	08:53	29	22	HIV-1/2 Antibody	Negative
							Hepatitis B Virus Core Antibody	Negative
							Hepatitis B Virus Surface Antigen	Negative
							Hepatitis C Virus Antibody	Negative
R107	3D7	Screening	03-NOV-2017	11:25	-12	-20	HIV-1/2 Antibody	Negative
							Hepatitis B Virus Core Antibody	Negative
							Hepatitis B Virus Surface Antigen	Negative
							Hepatitis C Virus Antibody	Negative
		End Of Study	14-DEC-2017	09:31	29	22	HIV-1/2 Antibody	Negative
							Hepatitis B Virus Core Antibody	Negative
							Hepatitis B Virus Surface Antigen	Negative
							Hepatitis C Virus Antibody	Negative

Notes: Day relative to inoculum is the number of days relative to day of administration of the inoculum, where the day of administration = 0.
The inoculum was administered on Study Day 0 for K13-infected subjects and Study Day 1 for 3D7-infected subjects.
Day relative to IP is the number of days relative to day of administration of Artesunate, where the day of dose = 1.

Listing 16.2.4.7
Serology

Randomisation Number	Challenge Agent	Visit	Date of Sample Collection	Time of Sample Collection	Day Relative to Inoculum	Day Relative to IP	Parameter	Result
R108	3D7	Screening	26-OCT-2017	09:30	-20	-28	HIV-1/2 Antibody	Negative
							Hepatitis B Virus Core Antibody	Negative
							Hepatitis B Virus Surface Antigen	Negative
							Hepatitis C Virus Antibody	Negative
		End Of Study	14-DEC-2017	08:37	29	22	HIV-1/2 Antibody	Negative
							Hepatitis B Virus Core Antibody	Negative
							Hepatitis B Virus Surface Antigen	Negative
							Hepatitis C Virus Antibody	Negative
R201	K13	Screening	08-FEB-2018	12:01	-11	-20	HIV-1/2 Antibody	Negative
							Hepatitis B Virus Core Antibody	Negative
							Hepatitis B Virus Surface Antigen	Negative
							Hepatitis C Virus Antibody	Negative
		End Of Study	22-MAR-2018	08:31	31	23	HIV-1/2 Antibody	Negative
							Hepatitis B Virus Core Antibody	Negative
							Hepatitis B Virus Surface Antigen	Negative
							Hepatitis C Virus Antibody	Negative
R202	K13	Screening	13-FEB-2018	11:20	-6	-15	HIV-1/2 Antibody	Negative
							Hepatitis B Virus Core Antibody	Negative
							Hepatitis B Virus Surface Antigen	Negative
							Hepatitis C Virus Antibody	Negative

Notes: Day relative to inoculum is the number of days relative to day of administration of the inoculum, where the day of administration = 0.
The inoculum was administered on Study Day 0 for K13-infected subjects and Study Day 1 for 3D7-infected subjects.
Day relative to IP is the number of days relative to day of administration of Artesunate, where the day of dose = 1.

Listing 16.2.4.7
Serology

Randomisation Number	Challenge Agent	Visit	Date of Sample Collection	Time of Sample Collection	Day Relative to Inoculum	Day Relative to IP	Parameter	Result
R202	K13	End Of Study	22-MAR-2018	10:00	31	23	HIV-1/2 Antibody	Negative
							Hepatitis B Virus Core Antibody	Negative
							Hepatitis B Virus Surface Antigen	Negative
							Hepatitis C Virus Antibody	Negative
R203	K13	Screening	13-FEB-2018	10:18	-6	-15	HIV-1/2 Antibody	Negative
							Hepatitis B Virus Core Antibody	Negative
							Hepatitis B Virus Surface Antigen	Negative
							Hepatitis C Virus Antibody	Negative
		End Of Study	22-MAR-2018	10:14	31	23	HIV-1/2 Antibody	Negative
							Hepatitis B Virus Core Antibody	Negative
							Hepatitis B Virus Surface Antigen	Negative
							Hepatitis C Virus Antibody	Negative
R204	K13	Screening	05-FEB-2018	10:46	-14	-23	HIV-1/2 Antibody	Negative
							Hepatitis B Virus Core Antibody	Negative
							Hepatitis B Virus Surface Antigen	Negative
							Hepatitis C Virus Antibody	Negative
		End Of Study	22-MAR-2018	08:43	31	23	HIV-1/2 Antibody	Negative
							Hepatitis B Virus Core Antibody	Negative
							Hepatitis B Virus Surface Antigen	Negative
							Hepatitis C Virus Antibody	Negative

Notes: Day relative to inoculum is the number of days relative to day of administration of the inoculum, where the day of administration = 0.
The inoculum was administered on Study Day 0 for K13-infected subjects and Study Day 1 for 3D7-infected subjects.
Day relative to IP is the number of days relative to day of administration of Artesunate, where the day of dose = 1.

Listing 16.2.4.7
Serology

Randomisation Number	Challenge Agent	Visit	Date of Sample Collection	Time of Sample Collection	Day Relative to Inoculum	Day Relative to IP	Parameter	Result
R205	K13	Screening	31-JAN-2018	10:01	-19	-28	HIV-1/2 Antibody	Negative
							Hepatitis B Virus Core Antibody	Negative
							Hepatitis B Virus Surface Antigen	Negative
							Hepatitis C Virus Antibody	Negative
		End Of Study	22-MAR-2018	07:30	31	23	HIV-1/2 Antibody	Negative
							Hepatitis B Virus Core Antibody	Negative
							Hepatitis B Virus Surface Antigen	Negative
							Hepatitis C Virus Antibody	Negative
R206	K13	Screening	06-FEB-2018	13:32	-13	-22	HIV-1/2 Antibody	Negative
							Hepatitis B Virus Core Antibody	Negative
							Hepatitis B Virus Surface Antigen	Negative
							Hepatitis C Virus Antibody	Negative
		End Of Study	22-MAR-2018	09:19	31	23	HIV-1/2 Antibody	Negative
							Hepatitis B Virus Core Antibody	Negative
							Hepatitis B Virus Surface Antigen	Negative
							Hepatitis C Virus Antibody	Negative
R207	K13	Screening	14-FEB-2018	11:34	-5	-14	HIV-1/2 Antibody	Negative
							Hepatitis B Virus Core Antibody	Negative
							Hepatitis B Virus Surface Antigen	Negative
							Hepatitis C Virus Antibody	Negative

Notes: Day relative to inoculum is the number of days relative to day of administration of the inoculum, where the day of administration = 0.
The inoculum was administered on Study Day 0 for K13-infected subjects and Study Day 1 for 3D7-infected subjects.
Day relative to IP is the number of days relative to day of administration of Artesunate, where the day of dose = 1.

Listing 16.2.4.7
Serology

Randomisation Number	Challenge Agent	Visit	Date of Sample Collection	Time of Sample Collection	Day Relative to Inoculum	Day Relative to IP	Parameter	Result
R207	K13	End Of Study	22-MAR-2018	09:32	31	23	HIV-1/2 Antibody	Negative
							Hepatitis B Virus Core Antibody	Negative
							Hepatitis B Virus Surface Antigen	Negative
							Hepatitis C Virus Antibody	Negative
R208	3D7	Screening	01-FEB-2018	12:12	-19	-27	HIV-1/2 Antibody	Negative
							Hepatitis B Virus Core Antibody	Negative
							Hepatitis B Virus Surface Antigen	Negative
							Hepatitis C Virus Antibody	Negative
		End Of Study	22-MAR-2018	07:50	30	23	HIV-1/2 Antibody	Negative
							Hepatitis B Virus Core Antibody	Negative
							Hepatitis B Virus Surface Antigen	Negative
							Hepatitis C Virus Antibody	Negative
R209	3D7	Screening	08-FEB-2018	10:36	-12	-20	HIV-1/2 Antibody	Negative
							Hepatitis B Virus Core Antibody	Negative
							Hepatitis B Virus Surface Antigen	Negative
							Hepatitis C Virus Antibody	Negative
		End Of Study	22-MAR-2018	08:16	30	23	HIV-1/2 Antibody	Negative
							Hepatitis B Virus Core Antibody	Negative
							Hepatitis B Virus Surface Antigen	Negative
							Hepatitis C Virus Antibody	Negative

Notes: Day relative to inoculum is the number of days relative to day of administration of the inoculum, where the day of administration = 0.
The inoculum was administered on Study Day 0 for K13-infected subjects and Study Day 1 for 3D7-infected subjects.
Day relative to IP is the number of days relative to day of administration of Artesunate, where the day of dose = 1.

Listing 16.2.4.7
Serology

Randomisation Number	Challenge Agent	Visit	Date of Sample Collection	Time of Sample Collection	Day Relative to Inoculum	Day Relative to IP	Parameter	Result
R210	3D7	Screening	12-FEB-2018	09:38	-8	-16	HIV-1/2 Antibody	Negative
							Hepatitis B Virus Core Antibody	Negative
							Hepatitis B Virus Surface Antigen	Negative
							Hepatitis C Virus Antibody	Negative
		End Of Study	22-MAR-2018	07:34	30	23	HIV-1/2 Antibody	Negative
							Hepatitis B Virus Core Antibody	Negative
							Hepatitis B Virus Surface Antigen	Negative
							Hepatitis C Virus Antibody	Negative
R302	K13	Screening	16-AUG-2018	09:36	-19	-28	HIV-1/2 Antibody	Negative
							Hepatitis B Virus Core Antibody	Negative
							Hepatitis B Virus Surface Antigen	Negative
							Hepatitis C Virus Antibody	Negative
		End Of Study	02-OCT-2018	08:19	28	20	HIV-1/2 Antibody	Negative
							Hepatitis B Virus Core Antibody	Negative
							Hepatitis B Virus Surface Antigen	Negative
							Hepatitis C Virus Antibody	Negative
R303	K13	Screening	09-AUG-2018	10:30	-26	-35	HIV-1/2 Antibody	Negative
							Hepatitis B Virus Core Antibody	Negative
							Hepatitis B Virus Surface Antigen	Negative
							Hepatitis C Virus Antibody	Negative

Notes: Day relative to inoculum is the number of days relative to day of administration of the inoculum, where the day of administration = 0.
The inoculum was administered on Study Day 0 for K13-infected subjects and Study Day 1 for 3D7-infected subjects.
Day relative to IP is the number of days relative to day of administration of Artesunate, where the day of dose = 1.

Listing 16.2.4.7
Serology

Randomisation Number	Challenge Agent	Visit	Date of Sample Collection	Time of Sample Collection	Day Relative to Inoculum	Day Relative to IP	Parameter	Result
R303	K13	End Of Study	02-OCT-2018	09:05	28	20	HIV-1/2 Antibody	Negative
							Hepatitis B Virus Core Antibody	Negative
							Hepatitis B Virus Surface Antigen	Negative
							Hepatitis C Virus Antibody	Negative
R304	K13	Screening	13-AUG-2018	11:57	-22	-31	HIV-1/2 Antibody	Negative
							Hepatitis B Virus Core Antibody	Negative
							Hepatitis B Virus Surface Antigen	Negative
							Hepatitis C Virus Antibody	Negative
		End Of Study	02-OCT-2018	08:46	28	20	HIV-1/2 Antibody	Negative
							Hepatitis B Virus Core Antibody	Negative
							Hepatitis B Virus Surface Antigen	Negative
							Hepatitis C Virus Antibody	Negative
R305	3D7	Screening	13-AUG-2018	09:38	-23	-31	HIV-1/2 Antibody	Negative
							Hepatitis B Virus Core Antibody	Negative
							Hepatitis B Virus Surface Antigen	Negative
							Hepatitis C Virus Antibody	Negative
		End Of Study	02-OCT-2018	07:50	27	20	HIV-1/2 Antibody	Negative
							Hepatitis B Virus Core Antibody	Negative
							Hepatitis B Virus Surface Antigen	Negative
							Hepatitis C Virus Antibody	Negative

Notes: Day relative to inoculum is the number of days relative to day of administration of the inoculum, where the day of administration = 0.
The inoculum was administered on Study Day 0 for K13-infected subjects and Study Day 1 for 3D7-infected subjects.
Day relative to IP is the number of days relative to day of administration of Artesunate, where the day of dose = 1.

Listing 16.2.4.7
Serology

Randomisation Number	Challenge Agent	Visit	Date of Sample Collection	Time of Sample Collection	Day Relative to Inoculum	Day Relative to IP	Parameter	Result
R306	3D7	Screening	28-AUG-2018	11:39	-8	-16	HIV-1/2 Antibody	Negative
							Hepatitis B Virus Core Antibody	Negative
							Hepatitis B Virus Surface Antigen	Negative
							Hepatitis C Virus Antibody	Negative
		End Of Study	02-OCT-2018	08:20	27	20	HIV-1/2 Antibody	Negative
							Hepatitis B Virus Core Antibody	Negative
							Hepatitis B Virus Surface Antigen	Negative
							Hepatitis C Virus Antibody	Negative
R307	3D7	Screening	07-AUG-2018	12:31	-29	-37	HIV-1/2 Antibody	Negative
							Hepatitis B Virus Core Antibody	Negative
							Hepatitis B Virus Surface Antigen	Negative
							Hepatitis C Virus Antibody	Negative
		End Of Study	02-OCT-2018	07:21	27	20	HIV-1/2 Antibody	Negative
							Hepatitis B Virus Core Antibody	Negative
							Hepatitis B Virus Surface Antigen	Negative
							Hepatitis C Virus Antibody	Negative

Notes: Day relative to inoculum is the number of days relative to day of administration of the inoculum, where the day of administration = 0.
The inoculum was administered on Study Day 0 for K13-infected subjects and Study Day 1 for 3D7-infected subjects.
Day relative to IP is the number of days relative to day of administration of Artesunate, where the day of dose = 1.

16.2.4.8 Urine Drug Screen

Listing 16.2.4.8
Urine Drug Screen

Randomisation Number	Challenge Agent	Visit	Date of Sample Collection	Time of Sample Collection	Day Relative to Inoculum	Day Relative to IP	Test	Result
R103	K13	Screening	01-NOV-2017	11:39	-13	-22	Acetaminophen	Negative
							Amphetamine	Negative
							Barbiturates	Negative
							Benzodiazepine	Negative
							Cannabinoids	Negative
							Cocaine	Negative
							Methadone	Negative
							Methamphetamine	Negative
							Opiate	Negative
							Phencyclidine	Negative
							Tricyclic Antidepressants	Negative
		Inoculum	14-NOV-2017	07:34	0	-9	Acetaminophen	Negative
							Amphetamine	Negative
							Barbiturates	Negative
							Benzodiazepine	Negative
							Cannabinoids	Negative
							Cocaine	Negative
							Methadone	Negative
							Methamphetamine	Negative
							Opiate	Negative
							Phencyclidine	Negative
							Tricyclic Antidepressants	Negative

Notes: Day relative to inoculum is the number of days relative to day of administration of the inoculum, where the day of administration = 0.
The inoculum was administered on Study Day 0 for K13-infected subjects and Study Day 1 for 3D7-infected subjects.
Day relative to IP is the number of days relative to day of administration of Artesunate, where the day of dose = 1.

Listing 16.2.4.8
Urine Drug Screen

Randomisation Number	Challenge Agent	Visit	Date of Sample Collection	Time of Sample Collection	Day Relative to Inoculum	Day Relative to IP	Test	Result
R103	K13	Admission	23-NOV-2017	08:10	9	1	Acetaminophen	Negative
							Amphetamine	Negative
							Barbiturates	Negative
							Benzodiazepine	Negative
							Cannabinoids	Negative
							Cocaine	Negative
							Methadone	Negative
							Methamphetamine	Negative
							Opiate	Negative
							Phencyclidine	Negative
							Tricyclic Antidepressants	Negative
R104	K13	Screening	09-NOV-2017	10:04	-5	-14	Acetaminophen	Negative
							Amphetamine	Negative
							Barbiturates	Negative
							Benzodiazepine	Negative
							Cannabinoids	Negative
							Cocaine	Negative
							Methadone	Negative
							Methamphetamine	Negative
							Opiate	Negative
							Phencyclidine	Negative
							Tricyclic Antidepressants	Negative

Notes: Day relative to inoculum is the number of days relative to day of administration of the inoculum, where the day of administration = 0.
The inoculum was administered on Study Day 0 for K13-infected subjects and Study Day 1 for 3D7-infected subjects.
Day relative to IP is the number of days relative to day of administration of Artesunate, where the day of dose = 1.

Listing 16.2.4.8
Urine Drug Screen

Randomisation Number	Challenge Agent	Visit	Date of Sample Collection	Time of Sample Collection	Day Relative to Inoculum	Day Relative to IP	Test	Result
R104	K13	Inoculum	14-NOV-2017	07:32	0	-9	Acetaminophen	Negative
							Amphetamine	Negative
							Barbiturates	Negative
							Benzodiazepine	Negative
							Cannabinoids	Negative
							Cocaine	Negative
							Methadone	Negative
							Methamphetamine	Negative
							Opiate	Negative
							Phencyclidine	Negative
							Tricyclic Antidepressants	Negative
		Admission	23-NOV-2017	08:16	9	1	Acetaminophen	Negative
							Amphetamine	Negative
							Barbiturates	Negative
							Benzodiazepine	Negative
							Cannabinoids	Negative
							Cocaine	Negative
							Methadone	Negative
							Methamphetamine	Negative
							Opiate	Negative
							Phencyclidine	Negative
							Tricyclic Antidepressants	Negative

Notes: Day relative to inoculum is the number of days relative to day of administration of the inoculum, where the day of administration = 0.
The inoculum was administered on Study Day 0 for K13-infected subjects and Study Day 1 for 3D7-infected subjects.
Day relative to IP is the number of days relative to day of administration of Artesunate, where the day of dose = 1.

Listing 16.2.4.8
Urine Drug Screen

Randomisation Number	Challenge Agent	Visit	Date of Sample Collection	Time of Sample Collection	Day Relative to Inoculum	Day Relative to IP	Test	Result
R105	K13	Screening	08-NOV-2017	12:19	-6	-15	Acetaminophen	Negative
							Amphetamine	Negative
							Barbiturates	Negative
							Benzodiazepine	Negative
							Cannabinoids	Negative
							Cocaine	Negative
							Methadone	Negative
							Methamphetamine	Negative
							Opiate	Negative
							Phencyclidine	Negative
							Tricyclic Antidepressants	Negative
		Inoculum	14-NOV-2017	07:38	0	-9	Acetaminophen	Negative
							Amphetamine	Negative
							Barbiturates	Negative
							Benzodiazepine	Negative
							Cannabinoids	Negative
							Cocaine	Negative
							Methadone	Negative
							Methamphetamine	Negative
							Opiate	Negative
							Phencyclidine	Negative
							Tricyclic Antidepressants	Negative

Notes: Day relative to inoculum is the number of days relative to day of administration of the inoculum, where the day of administration = 0.
The inoculum was administered on Study Day 0 for K13-infected subjects and Study Day 1 for 3D7-infected subjects.
Day relative to IP is the number of days relative to day of administration of Artesunate, where the day of dose = 1.

Listing 16.2.4.8
Urine Drug Screen

Randomisation Number	Challenge Agent	Visit	Date of Sample Collection	Time of Sample Collection	Day Relative to Inoculum	Day Relative to IP	Test	Result
R105	K13	Admission	23-NOV-2017	07:59	9	1	Acetaminophen	Negative
							Amphetamine	Negative
							Barbiturates	Negative
							Benzodiazepine	Negative
							Cannabinoids	Negative
							Cocaine	Negative
							Methadone	Negative
							Methamphetamine	Negative
							Opiate	Negative
							Phencyclidine	Negative
							Tricyclic Antidepressants	Negative
R106	3D7	Screening	09-NOV-2017	10:28	-6	-14	Acetaminophen	Negative
							Amphetamine	Negative
							Barbiturates	Negative
							Benzodiazepine	Negative
							Cannabinoids	Negative
							Cocaine	Negative
							Methadone	Negative
							Methamphetamine	Negative
							Opiate	Negative
							Phencyclidine	Negative
							Tricyclic Antidepressants	Negative

Notes: Day relative to inoculum is the number of days relative to day of administration of the inoculum, where the day of administration = 0.
The inoculum was administered on Study Day 0 for K13-infected subjects and Study Day 1 for 3D7-infected subjects.
Day relative to IP is the number of days relative to day of administration of Artesunate, where the day of dose = 1.

Listing 16.2.4.8
Urine Drug Screen

Randomisation Number	Challenge Agent	Visit	Date of Sample Collection	Time of Sample Collection	Day Relative to Inoculum	Day Relative to IP	Test	Result
R106	3D7	Inoculum	15-NOV-2017	07:39	0	-8	Acetaminophen	Negative
							Amphetamine	Negative
							Barbiturates	Negative
							Benzodiazepine	Negative
							Cannabinoids	Negative
							Cocaine	Negative
							Methadone	Negative
							Methamphetamine	Negative
							Opiate	Negative
							Phencyclidine	Negative
							Tricyclic Antidepressants	Negative
		Admission	23-NOV-2017	08:59	8	1	Acetaminophen	Negative
							Amphetamine	Negative
							Barbiturates	Negative
							Benzodiazepine	Negative
							Cannabinoids	Negative
							Cocaine	Negative
							Methadone	Negative
							Methamphetamine	Negative
							Opiate	Negative
							Phencyclidine	Negative
							Tricyclic Antidepressants	Negative

Notes: Day relative to inoculum is the number of days relative to day of administration of the inoculum, where the day of administration = 0.
The inoculum was administered on Study Day 0 for K13-infected subjects and Study Day 1 for 3D7-infected subjects.
Day relative to IP is the number of days relative to day of administration of Artesunate, where the day of dose = 1.

Listing 16.2.4.8
Urine Drug Screen

Randomisation Number	Challenge Agent	Visit	Date of Sample Collection	Time of Sample Collection	Day Relative to Inoculum	Day Relative to IP	Test	Result
R107	3D7	Screening	03-NOV-2017	10:56	-12	-20	Acetaminophen	Negative
							Amphetamine	Negative
							Barbiturates	Negative
							Benzodiazepine	Negative
							Cannabinoids	Negative
							Cocaine	Negative
							Methadone	Negative
							Methamphetamine	Negative
							Opiate	Negative
							Phencyclidine	Negative
							Tricyclic Antidepressants	Negative
		Inoculum	15-NOV-2017	07:45	0	-8	Acetaminophen	Negative
							Amphetamine	Negative
							Barbiturates	Negative
							Benzodiazepine	Negative
							Cannabinoids	Negative
							Cocaine	Negative
							Methadone	Negative
							Methamphetamine	Negative
							Opiate	Negative
							Phencyclidine	Negative
							Tricyclic Antidepressants	Negative

Notes: Day relative to inoculum is the number of days relative to day of administration of the inoculum, where the day of administration = 0.
The inoculum was administered on Study Day 0 for K13-infected subjects and Study Day 1 for 3D7-infected subjects.
Day relative to IP is the number of days relative to day of administration of Artesunate, where the day of dose = 1.

Listing 16.2.4.8
Urine Drug Screen

Randomisation Number	Challenge Agent	Visit	Date of Sample Collection	Time of Sample Collection	Day Relative to Inoculum	Day Relative to IP	Test	Result
R107	3D7	Admission	23-NOV-2017	08:21	8	1	Acetaminophen	Negative
							Amphetamine	Negative
							Barbiturates	Negative
							Benzodiazepine	Negative
							Cannabinoids	Negative
							Cocaine	Negative
							Methadone	Negative
							Methamphetamine	Negative
							Opiate	Negative
							Phencyclidine	Negative
							Tricyclic Antidepressants	Negative
R108	3D7	Screening	26-OCT-2017	09:05	-20	-28	Acetaminophen	Negative
							Amphetamine	Negative
							Barbiturates	Negative
							Benzodiazepine	Negative
							Cannabinoids	Negative
							Cocaine	Negative
							Methadone	Negative
							Methamphetamine	Negative
							Opiate	Negative
							Phencyclidine	Negative
							Tricyclic Antidepressants	Negative

Notes: Day relative to inoculum is the number of days relative to day of administration of the inoculum, where the day of administration = 0.
The inoculum was administered on Study Day 0 for K13-infected subjects and Study Day 1 for 3D7-infected subjects.
Day relative to IP is the number of days relative to day of administration of Artesunate, where the day of dose = 1.

Listing 16.2.4.8
Urine Drug Screen

Randomisation Number	Challenge Agent	Visit	Date of Sample Collection	Time of Sample Collection	Day Relative to Inoculum	Day Relative to IP	Test	Result
R108	3D7	Inoculum	15-NOV-2017	07:46	0	-8	Acetaminophen	Negative
							Amphetamine	Negative
							Barbiturates	Negative
							Benzodiazepine	Negative
							Cannabinoids	Negative
							Cocaine	Negative
							Methadone	Negative
							Methamphetamine	Negative
							Opiate	Negative
							Phencyclidine	Negative
							Tricyclic Antidepressants	Negative
		Admission	23-NOV-2017	08:03	8	1	Acetaminophen	Positive
							Amphetamine	Negative
							Barbiturates	Negative
							Benzodiazepine	Negative
							Cannabinoids	Negative
							Cocaine	Negative
							Methadone	Negative
							Methamphetamine	Negative
							Opiate	Negative
							Phencyclidine	Negative
							Tricyclic Antidepressants	Negative

Notes: Day relative to inoculum is the number of days relative to day of administration of the inoculum, where the day of administration = 0.
The inoculum was administered on Study Day 0 for K13-infected subjects and Study Day 1 for 3D7-infected subjects.
Day relative to IP is the number of days relative to day of administration of Artesunate, where the day of dose = 1.

Listing 16.2.4.8
Urine Drug Screen

Randomisation Number	Challenge Agent	Visit	Date of Sample Collection	Time of Sample Collection	Day Relative to Inoculum	Day Relative to IP	Test	Result
R201	K13	Screening	08-FEB-2018	11:35	-11	-20	Acetaminophen	Negative
							Amphetamine	Negative
							Barbiturates	Negative
							Benzodiazepine	Negative
							Cannabinoids	Negative
							Cocaine	Negative
							Methadone	Negative
							Methamphetamine	Negative
							Opiate	Negative
							Phencyclidine	Negative
							Tricyclic Antidepressants	Negative
		Inoculum	19-FEB-2018	08:16	0	-9	Acetaminophen	Negative
							Amphetamine	Negative
							Barbiturates	Negative
							Benzodiazepine	Negative
							Cannabinoids	Negative
							Cocaine	Negative
							Methadone	Negative
							Methamphetamine	Negative
							Opiate	Negative
							Phencyclidine	Negative
							Tricyclic Antidepressants	Negative

Notes: Day relative to inoculum is the number of days relative to day of administration of the inoculum, where the day of administration = 0.
The inoculum was administered on Study Day 0 for K13-infected subjects and Study Day 1 for 3D7-infected subjects.
Day relative to IP is the number of days relative to day of administration of Artesunate, where the day of dose = 1.

Listing 16.2.4.8
Urine Drug Screen

Randomisation Number	Challenge Agent	Visit	Date of Sample Collection	Time of Sample Collection	Day Relative to Inoculum	Day Relative to IP	Test	Result
R201	K13	Admission	28-FEB-2018	07:50	9	1	Acetaminophen	Negative
							Amphetamine	Negative
							Barbiturates	Negative
							Benzodiazepine	Negative
							Cannabinoids	Negative
							Cocaine	Negative
							Methadone	Negative
							Methamphetamine	Negative
							Opiate	Negative
							Phencyclidine	Negative
							Tricyclic Antidepressants	Negative
R202	K13	Screening	13-FEB-2018	10:57	-6	-15	Acetaminophen	Negative
							Amphetamine	Negative
							Barbiturates	Negative
							Benzodiazepine	Negative
							Cannabinoids	Negative
							Cocaine	Negative
							Methadone	Negative
							Methamphetamine	Negative
							Opiate	Negative
							Phencyclidine	Negative
							Tricyclic Antidepressants	Negative

Notes: Day relative to inoculum is the number of days relative to day of administration of the inoculum, where the day of administration = 0.
The inoculum was administered on Study Day 0 for K13-infected subjects and Study Day 1 for 3D7-infected subjects.
Day relative to IP is the number of days relative to day of administration of Artesunate, where the day of dose = 1.

Listing 16.2.4.8
Urine Drug Screen

Randomisation Number	Challenge Agent	Visit	Date of Sample Collection	Time of Sample Collection	Day Relative to Inoculum	Day Relative to IP	Test	Result
R202	K13	Inoculum	19-FEB-2018	08:17	0	-9	Acetaminophen	Negative
							Amphetamine	Negative
							Barbiturates	Negative
							Benzodiazepine	Negative
							Cannabinoids	Negative
							Cocaine	Negative
							Methadone	Negative
							Methamphetamine	Negative
							Opiate	Negative
							Phencyclidine	Negative
							Tricyclic Antidepressants	Negative
		Admission	28-FEB-2018	07:07	9	1	Acetaminophen	Negative
							Amphetamine	Negative
							Barbiturates	Negative
							Benzodiazepine	Negative
							Cannabinoids	Negative
							Cocaine	Negative
							Methadone	Negative
							Methamphetamine	Negative
							Opiate	Negative
							Phencyclidine	Negative
							Tricyclic Antidepressants	Negative

Notes: Day relative to inoculum is the number of days relative to day of administration of the inoculum, where the day of administration = 0.
The inoculum was administered on Study Day 0 for K13-infected subjects and Study Day 1 for 3D7-infected subjects.
Day relative to IP is the number of days relative to day of administration of Artesunate, where the day of dose = 1.

Listing 16.2.4.8
Urine Drug Screen

Randomisation Number	Challenge Agent	Visit	Date of Sample Collection	Time of Sample Collection	Day Relative to Inoculum	Day Relative to IP	Test	Result
R203	K13	Screening	13-FEB-2018	09:19	-6	-15	Acetaminophen	Negative
							Amphetamine	Negative
							Barbiturates	Negative
							Benzodiazepine	Negative
							Cannabinoids	Negative
							Cocaine	Negative
							Methadone	Negative
							Methamphetamine	Negative
							Opiate	Negative
							Phencyclidine	Negative
							Tricyclic Antidepressants	Negative
		Inoculum	19-FEB-2018	08:18	0	-9	Acetaminophen	Negative
							Amphetamine	Negative
							Barbiturates	Negative
							Benzodiazepine	Negative
							Cannabinoids	Negative
							Cocaine	Negative
							Methadone	Negative
							Methamphetamine	Negative
							Opiate	Negative
							Phencyclidine	Negative
							Tricyclic Antidepressants	Negative

Notes: Day relative to inoculum is the number of days relative to day of administration of the inoculum, where the day of administration = 0.
The inoculum was administered on Study Day 0 for K13-infected subjects and Study Day 1 for 3D7-infected subjects.
Day relative to IP is the number of days relative to day of administration of Artesunate, where the day of dose = 1.

Listing 16.2.4.8
Urine Drug Screen

Randomisation Number	Challenge Agent	Visit	Date of Sample Collection	Time of Sample Collection	Day Relative to Inoculum	Day Relative to IP	Test	Result
R203	K13	Admission	28-FEB-2018	07:18	9	1	Acetaminophen	Negative
							Amphetamine	Negative
							Barbiturates	Negative
							Benzodiazepine	Negative
							Cannabinoids	Negative
							Cocaine	Negative
							Methadone	Negative
							Methamphetamine	Negative
							Opiate	Negative
							Phencyclidine	Negative
							Tricyclic Antidepressants	Negative
R204	K13	Screening	05-FEB-2018	10:10	-14	-23	Acetaminophen	Positive
							Amphetamine	Negative
							Barbiturates	Negative
							Benzodiazepine	Negative
							Cannabinoids	Negative
							Cocaine	Negative
							Methadone	Negative
							Methamphetamine	Negative
							Opiate	Negative
							Phencyclidine	Negative
							Tricyclic Antidepressants	Negative

Notes: Day relative to inoculum is the number of days relative to day of administration of the inoculum, where the day of administration = 0.
The inoculum was administered on Study Day 0 for K13-infected subjects and Study Day 1 for 3D7-infected subjects.
Day relative to IP is the number of days relative to day of administration of Artesunate, where the day of dose = 1.

Listing 16.2.4.8
Urine Drug Screen

Randomisation Number	Challenge Agent	Visit	Date of Sample Collection	Time of Sample Collection	Day Relative to Inoculum	Day Relative to IP	Test	Result
R204	K13	Inoculum	19-FEB-2018	08:18	0	-9	Acetaminophen	Negative
							Amphetamine	Negative
							Barbiturates	Negative
							Benzodiazepine	Negative
							Cannabinoids	Negative
							Cocaine	Negative
							Methadone	Negative
							Methamphetamine	Negative
							Opiate	Negative
							Phencyclidine	Negative
							Tricyclic Antidepressants	Negative
		Admission	28-FEB-2018	07:48	9	1	Acetaminophen	Negative
							Amphetamine	Negative
							Barbiturates	Negative
							Benzodiazepine	Negative
							Cannabinoids	Negative
							Cocaine	Negative
							Methadone	Negative
							Methamphetamine	Negative
							Opiate	Negative
							Phencyclidine	Negative
							Tricyclic Antidepressants	Negative

Notes: Day relative to inoculum is the number of days relative to day of administration of the inoculum, where the day of administration = 0.
The inoculum was administered on Study Day 0 for K13-infected subjects and Study Day 1 for 3D7-infected subjects.
Day relative to IP is the number of days relative to day of administration of Artesunate, where the day of dose = 1.

Listing 16.2.4.8
Urine Drug Screen

Randomisation Number	Challenge Agent	Visit	Date of Sample Collection	Time of Sample Collection	Day Relative to Inoculum	Day Relative to IP	Test	Result
R205	K13	Screening	31-JAN-2018	09:35	-19	-28	Acetaminophen	Negative
							Amphetamine	Negative
							Barbiturates	Negative
							Benzodiazepine	Negative
							Cannabinoids	Negative
							Cocaine	Negative
							Methadone	Negative
							Methamphetamine	Negative
							Opiate	Negative
							Phencyclidine	Negative
							Tricyclic Antidepressants	Negative
		Inoculum	19-FEB-2018	08:19	0	-9	Acetaminophen	Negative
							Amphetamine	Negative
							Barbiturates	Negative
							Benzodiazepine	Negative
							Cannabinoids	Negative
							Cocaine	Negative
							Methadone	Negative
							Methamphetamine	Negative
							Opiate	Negative
							Phencyclidine	Negative
							Tricyclic Antidepressants	Negative

Notes: Day relative to inoculum is the number of days relative to day of administration of the inoculum, where the day of administration = 0.
The inoculum was administered on Study Day 0 for K13-infected subjects and Study Day 1 for 3D7-infected subjects.
Day relative to IP is the number of days relative to day of administration of Artesunate, where the day of dose = 1.

Listing 16.2.4.8
Urine Drug Screen

Randomisation Number	Challenge Agent	Visit	Date of Sample Collection	Time of Sample Collection	Day Relative to Inoculum	Day Relative to IP	Test	Result
R205	K13	Admission	28-FEB-2018	07:20	9	1	Acetaminophen	Negative
							Amphetamine	Negative
							Barbiturates	Negative
							Benzodiazepine	Negative
							Cannabinoids	Negative
							Cocaine	Negative
							Methadone	Negative
							Methamphetamine	Negative
							Opiate	Negative
							Phencyclidine	Negative
							Tricyclic Antidepressants	Negative
R206	K13	Screening	06-FEB-2018	13:06	-13	-22	Acetaminophen	Negative
							Amphetamine	Negative
							Barbiturates	Negative
							Benzodiazepine	Negative
							Cannabinoids	Negative
							Cocaine	Negative
							Methadone	Negative
							Methamphetamine	Negative
							Opiate	Negative
							Phencyclidine	Negative
							Tricyclic Antidepressants	Negative

Notes: Day relative to inoculum is the number of days relative to day of administration of the inoculum, where the day of administration = 0.
The inoculum was administered on Study Day 0 for K13-infected subjects and Study Day 1 for 3D7-infected subjects.
Day relative to IP is the number of days relative to day of administration of Artesunate, where the day of dose = 1.

Listing 16.2.4.8
Urine Drug Screen

Randomisation Number	Challenge Agent	Visit	Date of Sample Collection	Time of Sample Collection	Day Relative to Inoculum	Day Relative to IP	Test	Result
R206	K13	Inoculum	19-FEB-2018	08:32	0	-9	Acetaminophen	Negative
							Amphetamine	Negative
							Barbiturates	Negative
							Benzodiazepine	Negative
							Cannabinoids	Negative
							Cocaine	Negative
							Methadone	Negative
							Methamphetamine	Negative
							Opiate	Negative
							Phencyclidine	Negative
							Tricyclic Antidepressants	Negative
		Admission	28-FEB-2018	08:32	9	1	Acetaminophen	Negative
							Amphetamine	Negative
							Barbiturates	Negative
							Benzodiazepine	Negative
							Cannabinoids	Negative
							Cocaine	Negative
							Methadone	Negative
							Methamphetamine	Negative
							Opiate	Negative
							Phencyclidine	Negative
							Tricyclic Antidepressants	Negative

Notes: Day relative to inoculum is the number of days relative to day of administration of the inoculum, where the day of administration = 0.
The inoculum was administered on Study Day 0 for K13-infected subjects and Study Day 1 for 3D7-infected subjects.
Day relative to IP is the number of days relative to day of administration of Artesunate, where the day of dose = 1.

Listing 16.2.4.8
Urine Drug Screen

Randomisation Number	Challenge Agent	Visit	Date of Sample Collection	Time of Sample Collection	Day Relative to Inoculum	Day Relative to IP	Test	Result
R207	K13	Screening	14-FEB-2018	11:08	-5	-14	Acetaminophen	Negative
							Amphetamine	Negative
							Barbiturates	Negative
							Benzodiazepine	Negative
							Cannabinoids	Negative
							Cocaine	Negative
							Methadone	Negative
							Methamphetamine	Negative
							Opiate	Negative
							Phencyclidine	Negative
							Tricyclic Antidepressants	Negative
		Inoculum	19-FEB-2018	08:21	0	-9	Acetaminophen	Negative
							Amphetamine	Negative
							Barbiturates	Negative
							Benzodiazepine	Negative
							Cannabinoids	Negative
							Cocaine	Negative
							Methadone	Negative
							Methamphetamine	Negative
							Opiate	Negative
							Phencyclidine	Negative
							Tricyclic Antidepressants	Negative

Notes: Day relative to inoculum is the number of days relative to day of administration of the inoculum, where the day of administration = 0.
The inoculum was administered on Study Day 0 for K13-infected subjects and Study Day 1 for 3D7-infected subjects.
Day relative to IP is the number of days relative to day of administration of Artesunate, where the day of dose = 1.

Listing 16.2.4.8
Urine Drug Screen

Randomisation Number	Challenge Agent	Visit	Date of Sample Collection	Time of Sample Collection	Day Relative to Inoculum	Day Relative to IP	Test	Result
R207	K13	Admission	28-FEB-2018	07:08	9	1	Acetaminophen	Negative
							Amphetamine	Negative
							Barbiturates	Negative
							Benzodiazepine	Negative
							Cannabinoids	Negative
							Cocaine	Negative
							Methadone	Negative
							Methamphetamine	Negative
							Opiate	Negative
							Phencyclidine	Negative
							Tricyclic Antidepressants	Negative
R208	3D7	Screening	01-FEB-2018	11:49	-19	-27	Acetaminophen	Negative
							Amphetamine	Negative
							Barbiturates	Negative
							Benzodiazepine	Negative
							Cannabinoids	Negative
							Cocaine	Negative
							Methadone	Negative
							Methamphetamine	Negative
							Opiate	Negative
							Phencyclidine	Negative
							Tricyclic Antidepressants	Negative

Notes: Day relative to inoculum is the number of days relative to day of administration of the inoculum, where the day of administration = 0.
The inoculum was administered on Study Day 0 for K13-infected subjects and Study Day 1 for 3D7-infected subjects.
Day relative to IP is the number of days relative to day of administration of Artesunate, where the day of dose = 1.

Listing 16.2.4.8
Urine Drug Screen

Randomisation Number	Challenge Agent	Visit	Date of Sample Collection	Time of Sample Collection	Day Relative to Inoculum	Day Relative to IP	Test	Result
R208	3D7	Inoculum	20-FEB-2018	08:10	0	-8	Acetaminophen	Negative
							Amphetamine	Negative
							Barbiturates	Negative
							Benzodiazepine	Negative
							Cannabinoids	Negative
							Cocaine	Negative
							Methadone	Negative
							Methamphetamine	Negative
							Opiate	Negative
							Phencyclidine	Negative
							Tricyclic Antidepressants	Negative
		Admission	28-FEB-2018	06:59	8	1	Acetaminophen	Negative
							Amphetamine	Negative
							Barbiturates	Negative
							Benzodiazepine	Negative
							Cannabinoids	Negative
							Cocaine	Negative
							Methadone	Negative
							Methamphetamine	Negative
							Opiate	Negative
							Phencyclidine	Negative
							Tricyclic Antidepressants	Negative

Notes: Day relative to inoculum is the number of days relative to day of administration of the inoculum, where the day of administration = 0.
The inoculum was administered on Study Day 0 for K13-infected subjects and Study Day 1 for 3D7-infected subjects.
Day relative to IP is the number of days relative to day of administration of Artesunate, where the day of dose = 1.

Listing 16.2.4.8
Urine Drug Screen

Randomisation Number	Challenge Agent	Visit	Date of Sample Collection	Time of Sample Collection	Day Relative to Inoculum	Day Relative to IP	Test	Result
R209	3D7	Screening	08-FEB-2018	10:12	-12	-20	Acetaminophen	Negative
							Amphetamine	Negative
							Barbiturates	Negative
							Benzodiazepine	Negative
							Cannabinoids	Negative
							Cocaine	Negative
							Methadone	Negative
							Methamphetamine	Negative
							Opiate	Negative
							Phencyclidine	Negative
							Tricyclic Antidepressants	Negative
		Inoculum	20-FEB-2018	08:12	0	-8	Acetaminophen	Negative
							Amphetamine	Negative
							Barbiturates	Negative
							Benzodiazepine	Negative
							Cannabinoids	Negative
							Cocaine	Negative
							Methadone	Negative
							Methamphetamine	Negative
							Opiate	Negative
							Phencyclidine	Negative
							Tricyclic Antidepressants	Negative

Notes: Day relative to inoculum is the number of days relative to day of administration of the inoculum, where the day of administration = 0.
The inoculum was administered on Study Day 0 for K13-infected subjects and Study Day 1 for 3D7-infected subjects.
Day relative to IP is the number of days relative to day of administration of Artesunate, where the day of dose = 1.

Listing 16.2.4.8
Urine Drug Screen

Randomisation Number	Challenge Agent	Visit	Date of Sample Collection	Time of Sample Collection	Day Relative to Inoculum	Day Relative to IP	Test	Result
R209	3D7	Admission	28-FEB-2018	07:31	8	1	Acetaminophen	Negative
							Amphetamine	Negative
							Barbiturates	Negative
							Benzodiazepine	Negative
							Cannabinoids	Negative
							Cocaine	Negative
							Methadone	Negative
							Methamphetamine	Negative
							Opiate	Negative
							Phencyclidine	Negative
							Tricyclic Antidepressants	Negative
R210	3D7	Screening	12-FEB-2018	09:04	-8	-16	Acetaminophen	Negative
							Amphetamine	Negative
							Barbiturates	Negative
							Benzodiazepine	Negative
							Cannabinoids	Negative
							Cocaine	Negative
							Methadone	Negative
							Methamphetamine	Negative
							Opiate	Negative
							Phencyclidine	Negative
							Tricyclic Antidepressants	Negative

Notes: Day relative to inoculum is the number of days relative to day of administration of the inoculum, where the day of administration = 0.
The inoculum was administered on Study Day 0 for K13-infected subjects and Study Day 1 for 3D7-infected subjects.
Day relative to IP is the number of days relative to day of administration of Artesunate, where the day of dose = 1.

Listing 16.2.4.8
Urine Drug Screen

Randomisation Number	Challenge Agent	Visit	Date of Sample Collection	Time of Sample Collection	Day Relative to Inoculum	Day Relative to IP	Test	Result
R210	3D7	Inoculum	20-FEB-2018	08:41	0	-8	Acetaminophen	Negative
							Amphetamine	Negative
							Barbiturates	Negative
							Benzodiazepine	Negative
							Cannabinoids	Negative
							Cocaine	Negative
							Methadone	Negative
							Methamphetamine	Negative
							Opiate	Negative
							Phencyclidine	Negative
							Tricyclic Antidepressants	Negative
		Admission	28-FEB-2018	07:40	8	1	Acetaminophen	Negative
							Amphetamine	Negative
							Barbiturates	Negative
							Benzodiazepine	Negative
							Cannabinoids	Negative
							Cocaine	Negative
							Methadone	Negative
							Methamphetamine	Negative
							Opiate	Negative
							Phencyclidine	Negative
							Tricyclic Antidepressants	Negative

Notes: Day relative to inoculum is the number of days relative to day of administration of the inoculum, where the day of administration = 0.
The inoculum was administered on Study Day 0 for K13-infected subjects and Study Day 1 for 3D7-infected subjects.
Day relative to IP is the number of days relative to day of administration of Artesunate, where the day of dose = 1.

Listing 16.2.4.8
Urine Drug Screen

Randomisation Number	Challenge Agent	Visit	Date of Sample Collection	Time of Sample Collection	Day Relative to Inoculum	Day Relative to IP	Test	Result
R302	K13	Screening	16-AUG-2018	09:14	-19	-28	Acetaminophen	Negative
							Amphetamine	Negative
							Barbiturates	Negative
							Benzodiazepine	Negative
							Cannabinoids	Negative
							Cocaine	Negative
							Methadone	Negative
							Methamphetamine	Negative
							Opiate	Negative
							Phencyclidine	Negative
							Tricyclic Antidepressants	Negative
		Inoculum	04-SEP-2018	07:43	0	-9	Acetaminophen	Negative
							Amphetamine	Negative
							Barbiturates	Negative
							Benzodiazepine	Negative
							Cannabinoids	Negative
							Cocaine	Negative
							Methadone	Negative
							Methamphetamine	Negative
							Opiate	Negative
							Phencyclidine	Negative
							Tricyclic Antidepressants	Negative

Notes: Day relative to inoculum is the number of days relative to day of administration of the inoculum, where the day of administration = 0.
The inoculum was administered on Study Day 0 for K13-infected subjects and Study Day 1 for 3D7-infected subjects.
Day relative to IP is the number of days relative to day of administration of Artesunate, where the day of dose = 1.

Listing 16.2.4.8
Urine Drug Screen

Randomisation Number	Challenge Agent	Visit	Date of Sample Collection	Time of Sample Collection	Day Relative to Inoculum	Day Relative to IP	Test	Result
R302	K13	Admission	13-SEP-2018	07:31	9	1	Acetaminophen	Negative
							Amphetamine	Negative
							Barbiturates	Negative
							Benzodiazepine	Negative
							Cannabinoids	Negative
							Cocaine	Negative
							Methadone	Negative
							Methamphetamine	Negative
							Opiate	Negative
							Phencyclidine	Negative
							Tricyclic Antidepressants	Negative
R303	K13	Screening	09-AUG-2018	09:46	-26	-35	Acetaminophen	Negative
							Amphetamine	Negative
							Barbiturates	Negative
							Benzodiazepine	Negative
							Cannabinoids	Negative
							Cocaine	Negative
							Methadone	Negative
							Methamphetamine	Negative
							Opiate	Negative
							Phencyclidine	Negative
							Tricyclic Antidepressants	Negative

Notes: Day relative to inoculum is the number of days relative to day of administration of the inoculum, where the day of administration = 0.
The inoculum was administered on Study Day 0 for K13-infected subjects and Study Day 1 for 3D7-infected subjects.
Day relative to IP is the number of days relative to day of administration of Artesunate, where the day of dose = 1.

Listing 16.2.4.8
Urine Drug Screen

Randomisation Number	Challenge Agent	Visit	Date of Sample Collection	Time of Sample Collection	Day Relative to Inoculum	Day Relative to IP	Test	Result
R303	K13	Inoculum	04-SEP-2018	07:33	0	-9	Acetaminophen	Negative
							Amphetamine	Negative
							Barbiturates	Negative
							Benzodiazepine	Negative
							Cannabinoids	Negative
							Cocaine	Negative
							Methadone	Negative
							Methamphetamine	Negative
							Opiate	Negative
							Phencyclidine	Negative
							Tricyclic Antidepressants	Negative
		Admission	13-SEP-2018	07:23	9	1	Acetaminophen	Negative
							Amphetamine	Negative
							Barbiturates	Negative
							Benzodiazepine	Negative
							Cannabinoids	Negative
							Cocaine	Negative
							Methadone	Negative
							Methamphetamine	Negative
							Opiate	Negative
							Phencyclidine	Negative
							Tricyclic Antidepressants	Negative

Notes: Day relative to inoculum is the number of days relative to day of administration of the inoculum, where the day of administration = 0.
The inoculum was administered on Study Day 0 for K13-infected subjects and Study Day 1 for 3D7-infected subjects.
Day relative to IP is the number of days relative to day of administration of Artesunate, where the day of dose = 1.

Listing 16.2.4.8
Urine Drug Screen

Randomisation Number	Challenge Agent	Visit	Date of Sample Collection	Time of Sample Collection	Day Relative to Inoculum	Day Relative to IP	Test	Result
R304	K13	Screening	13-AUG-2018	11:29	-22	-31	Acetaminophen	Negative
							Amphetamine	Negative
							Barbiturates	Negative
							Benzodiazepine	Negative
							Cannabinoids	Negative
							Cocaine	Negative
							Methadone	Negative
							Methamphetamine	Negative
							Opiate	Negative
							Phencyclidine	Negative
							Tricyclic Antidepressants	Negative
		Inoculum	04-SEP-2018	07:45	0	-9	Acetaminophen	Negative
							Amphetamine	Negative
							Barbiturates	Negative
							Benzodiazepine	Negative
							Cannabinoids	Negative
							Cocaine	Negative
							Methadone	Negative
							Methamphetamine	Negative
							Opiate	Negative
							Phencyclidine	Negative
							Tricyclic Antidepressants	Negative

Notes: Day relative to inoculum is the number of days relative to day of administration of the inoculum, where the day of administration = 0.
The inoculum was administered on Study Day 0 for K13-infected subjects and Study Day 1 for 3D7-infected subjects.
Day relative to IP is the number of days relative to day of administration of Artesunate, where the day of dose = 1.

Listing 16.2.4.8
Urine Drug Screen

Randomisation Number	Challenge Agent	Visit	Date of Sample Collection	Time of Sample Collection	Day Relative to Inoculum	Day Relative to IP	Test	Result
R304	K13	Admission	13-SEP-2018	07:20	9	1	Acetaminophen	Negative
							Amphetamine	Negative
							Barbiturates	Negative
							Benzodiazepine	Negative
							Cannabinoids	Negative
							Cocaine	Negative
							Methadone	Negative
							Methamphetamine	Negative
							Opiate	Negative
							Phencyclidine	Negative
							Tricyclic Antidepressants	Negative
R305	3D7	Screening	13-AUG-2018	09:04	-23	-31	Acetaminophen	Negative
							Amphetamine	Negative
							Barbiturates	Negative
							Benzodiazepine	Negative
							Cannabinoids	Negative
							Cocaine	Negative
							Methadone	Negative
							Methamphetamine	Negative
							Opiate	Negative
							Phencyclidine	Negative
							Tricyclic Antidepressants	Negative

Notes: Day relative to inoculum is the number of days relative to day of administration of the inoculum, where the day of administration = 0.
The inoculum was administered on Study Day 0 for K13-infected subjects and Study Day 1 for 3D7-infected subjects.
Day relative to IP is the number of days relative to day of administration of Artesunate, where the day of dose = 1.

Listing 16.2.4.8
Urine Drug Screen

Randomisation Number	Challenge Agent	Visit	Date of Sample Collection	Time of Sample Collection	Day Relative to Inoculum	Day Relative to IP	Test	Result
R305	3D7	Inoculum	05-SEP-2018	07:32	0	-8	Acetaminophen	Negative
							Amphetamine	Negative
							Barbiturates	Negative
							Benzodiazepine	Negative
							Cannabinoids	Negative
							Cocaine	Negative
							Methadone	Negative
							Methamphetamine	Negative
							Opiate	Negative
							Phencyclidine	Negative
							Tricyclic Antidepressants	Negative
		Admission	13-SEP-2018	07:49	8	1	Acetaminophen	Negative
							Amphetamine	Negative
							Barbiturates	Negative
							Benzodiazepine	Negative
							Cannabinoids	Negative
							Cocaine	Negative
							Methadone	Negative
							Methamphetamine	Negative
							Opiate	Negative
							Phencyclidine	Negative
							Tricyclic Antidepressants	Negative

Notes: Day relative to inoculum is the number of days relative to day of administration of the inoculum, where the day of administration = 0.
The inoculum was administered on Study Day 0 for K13-infected subjects and Study Day 1 for 3D7-infected subjects.
Day relative to IP is the number of days relative to day of administration of Artesunate, where the day of dose = 1.

Listing 16.2.4.8
Urine Drug Screen

Randomisation Number	Challenge Agent	Visit	Date of Sample Collection	Time of Sample Collection	Day Relative to Inoculum	Day Relative to IP	Test	Result
R306	3D7	Screening	28-AUG-2018	11:12	-8	-16	Acetaminophen	Negative
							Amphetamine	Negative
							Barbiturates	Negative
							Benzodiazepine	Negative
							Cannabinoids	Negative
							Cocaine	Negative
							Methadone	Negative
							Methamphetamine	Negative
							Opiate	Negative
							Phencyclidine	Negative
							Tricyclic Antidepressants	Negative
		Inoculum	05-SEP-2018	07:32	0	-8	Acetaminophen	Negative
							Amphetamine	Negative
							Barbiturates	Negative
							Benzodiazepine	Negative
							Cannabinoids	Negative
							Cocaine	Negative
							Methadone	Negative
							Methamphetamine	Negative
							Opiate	Negative
							Phencyclidine	Negative
							Tricyclic Antidepressants	Negative

Notes: Day relative to inoculum is the number of days relative to day of administration of the inoculum, where the day of administration = 0.
The inoculum was administered on Study Day 0 for K13-infected subjects and Study Day 1 for 3D7-infected subjects.
Day relative to IP is the number of days relative to day of administration of Artesunate, where the day of dose = 1.

Listing 16.2.4.8
Urine Drug Screen

Randomisation Number	Challenge Agent	Visit	Date of Sample Collection	Time of Sample Collection	Day Relative to Inoculum	Day Relative to IP	Test	Result
R306	3D7	Admission	13-SEP-2018	07:25	8	1	Acetaminophen	Negative
							Amphetamine	Negative
							Barbiturates	Negative
							Benzodiazepine	Negative
							Cannabinoids	Negative
							Cocaine	Negative
							Methadone	Negative
							Methamphetamine	Negative
							Opiate	Negative
							Phencyclidine	Negative
							Tricyclic Antidepressants	Negative
R307	3D7	Screening	07-AUG-2018	12:03	-29	-37	Acetaminophen	Negative
							Amphetamine	Negative
							Barbiturates	Negative
							Benzodiazepine	Negative
							Cannabinoids	Negative
							Cocaine	Negative
							Methadone	Negative
							Methamphetamine	Negative
							Opiate	Negative
							Phencyclidine	Negative
							Tricyclic Antidepressants	Negative

Notes: Day relative to inoculum is the number of days relative to day of administration of the inoculum, where the day of administration = 0.
The inoculum was administered on Study Day 0 for K13-infected subjects and Study Day 1 for 3D7-infected subjects.
Day relative to IP is the number of days relative to day of administration of Artesunate, where the day of dose = 1.

Listing 16.2.4.8
Urine Drug Screen

Randomisation Number	Challenge Agent	Visit	Date of Sample Collection	Time of Sample Collection	Day Relative to Inoculum	Day Relative to IP	Test	Result
R307	3D7	Inoculum	05-SEP-2018	08:28	0	-8	Acetaminophen	Negative
							Amphetamine	Negative
							Barbiturates	Negative
							Benzodiazepine	Negative
							Cannabinoids	Negative
							Cocaine	Negative
							Methadone	Negative
							Methamphetamine	Negative
							Opiate	Negative
							Phencyclidine	Negative
							Tricyclic Antidepressants	Negative
		Admission	13-SEP-2018	07:39	8	1	Acetaminophen	Negative
							Amphetamine	Negative
							Barbiturates	Negative
							Benzodiazepine	Negative
							Cannabinoids	Negative
							Cocaine	Negative
							Methadone	Negative
							Methamphetamine	Negative
							Opiate	Negative
							Phencyclidine	Negative
							Tricyclic Antidepressants	Negative

Notes: Day relative to inoculum is the number of days relative to day of administration of the inoculum, where the day of administration = 0.
The inoculum was administered on Study Day 0 for K13-infected subjects and Study Day 1 for 3D7-infected subjects.
Day relative to IP is the number of days relative to day of administration of Artesunate, where the day of dose = 1.

16.2.4.9 Alcohol Breath Test

Listing 16.2.4.9
Alcohol Breath Test

Randomisation Number	Challenge Agent	Visit	Date of Sample Collection	Time of Sample Collection	Day Relative to Inoculum	Day Relative to IP	Test	Result
R103	K13	Screening	01-NOV-2017	11:45	-13	-22	Ethanol	Negative
		Inoculum	14-NOV-2017	07:21	0	-9	Ethanol	Negative
		Admission	23-NOV-2017	07:30	9	1	Ethanol	Negative
R104	K13	Screening	09-NOV-2017	10:02	-5	-14	Ethanol	Negative
		Inoculum	14-NOV-2017	07:07	0	-9	Ethanol	Negative
		Admission	23-NOV-2017	08:08	9	1	Ethanol	Negative
R105	K13	Screening	08-NOV-2017	12:29	-6	-15	Ethanol	Negative
		Inoculum	14-NOV-2017	07:04	0	-9	Ethanol	Negative
		Admission	23-NOV-2017	06:43	9	1	Ethanol	Negative
R106	3D7	Screening	09-NOV-2017	10:40	-6	-14	Ethanol	Negative
		Inoculum	15-NOV-2017	07:24	0	-8	Ethanol	Negative
		Admission	23-NOV-2017	08:54	8	1	Ethanol	Negative
R107	3D7	Screening	03-NOV-2017	10:53	-12	-20	Ethanol	Negative
		Inoculum	15-NOV-2017	07:31	0	-8	Ethanol	Negative
		Admission	23-NOV-2017	07:57	8	1	Ethanol	Negative
R108	3D7	Screening	26-OCT-2017	09:02	-20	-28	Ethanol	Negative
		Inoculum	15-NOV-2017	07:13	0	-8	Ethanol	Negative
		Admission	23-NOV-2017	07:35	8	1	Ethanol	Negative

Notes: Day relative to inoculum is the number of days relative to day of administration of the inoculum, where the day of administration = 0.
The inoculum was administered on Study Day 0 for K13-infected subjects and Study Day 1 for 3D7-infected subjects.
Day relative to IP is the number of days relative to day of administration of Artesunate, where the day of dose = 1.

Listing 16.2.4.9
Alcohol Breath Test

Randomisation Number	Challenge Agent	Visit	Date of Sample Collection	Time of Sample Collection	Day Relative to Inoculum	Day Relative to IP	Test	Result
R201	K13	Screening	08-FEB-2018	11:35	-11	-20	Ethanol	Negative
		Inoculum	19-FEB-2018	07:47	0	-9	Ethanol	Negative
		Admission	28-FEB-2018	07:19	9	1	Ethanol	Negative
R202	K13	Screening	13-FEB-2018	11:01	-6	-15	Ethanol	Negative
		Inoculum	19-FEB-2018	07:58	0	-9	Ethanol	Negative
		Admission	28-FEB-2018	07:08	9	1	Ethanol	Negative
R203	K13	Screening	13-FEB-2018	09:55	-6	-15	Ethanol	Negative
		Inoculum	19-FEB-2018	07:53	0	-9	Ethanol	Negative
		Admission	28-FEB-2018	07:09	9	1	Ethanol	Negative
R204	K13	Screening	05-FEB-2018	10:19	-14	-23	Ethanol	Negative
		Inoculum	19-FEB-2018	07:50	0	-9	Ethanol	Negative
		Admission	28-FEB-2018	07:22	9	1	Ethanol	Negative
R205	K13	Screening	31-JAN-2018	09:42	-19	-28	Ethanol	Negative
		Inoculum	19-FEB-2018	07:45	0	-9	Ethanol	Negative
		Admission	28-FEB-2018	07:15	9	1	Ethanol	Negative
R206	K13	Screening	06-FEB-2018	13:14	-13	-22	Ethanol	Negative
		Inoculum	19-FEB-2018	08:06	0	-9	Ethanol	Negative
		Admission	28-FEB-2018	08:28	9	1	Ethanol	Negative

Notes: Day relative to inoculum is the number of days relative to day of administration of the inoculum, where the day of administration = 0.
The inoculum was administered on Study Day 0 for K13-infected subjects and Study Day 1 for 3D7-infected subjects.
Day relative to IP is the number of days relative to day of administration of Artesunate, where the day of dose = 1.

Listing 16.2.4.9
Alcohol Breath Test

Randomisation Number	Challenge Agent	Visit	Date of Sample Collection	Time of Sample Collection	Day Relative to Inoculum	Day Relative to IP	Test	Result
R207	K13	Screening	14-FEB-2018	11:06	-5	-14	Ethanol	Negative
		Inoculum	19-FEB-2018	08:04	0	-9	Ethanol	Negative
		Admission	28-FEB-2018	07:05	9	1	Ethanol	Negative
R208	3D7	Screening	01-FEB-2018	11:45	-19	-27	Ethanol	Negative
		Inoculum	20-FEB-2018	07:38	0	-8	Ethanol	Negative
		Admission	28-FEB-2018	06:55	8	1	Ethanol	Negative
R209	3D7	Screening	08-FEB-2018	10:07	-12	-20	Ethanol	Negative
		Inoculum	20-FEB-2018	07:42	0	-8	Ethanol	Negative
		Admission	28-FEB-2018	07:26	8	1	Ethanol	Negative
R210	3D7	Screening	12-FEB-2018	09:10	-8	-16	Ethanol	Negative
		Inoculum	20-FEB-2018	08:33	0	-8	Ethanol	Negative
		Admission	28-FEB-2018	07:33	8	1	Ethanol	Negative
R302	K13	Screening	16-AUG-2018	09:07	-19	-28	Ethanol	Negative
		Inoculum	04-SEP-2018	07:15	0	-9	Ethanol	Negative
		Admission	13-SEP-2018	07:11	9	1	Ethanol	Negative
R303	K13	Screening	09-AUG-2018	09:55	-26	-35	Ethanol	Negative
		Inoculum	04-SEP-2018	07:18	0	-9	Ethanol	Negative
		Admission	13-SEP-2018	07:07	9	1	Ethanol	Negative

Notes: Day relative to inoculum is the number of days relative to day of administration of the inoculum, where the day of administration = 0.
The inoculum was administered on Study Day 0 for K13-infected subjects and Study Day 1 for 3D7-infected subjects.
Day relative to IP is the number of days relative to day of administration of Artesunate, where the day of dose = 1.

Listing 16.2.4.9
Alcohol Breath Test

Randomisation Number	Challenge Agent	Visit	Date of Sample Collection	Time of Sample Collection	Day Relative to Inoculum	Day Relative to IP	Test	Result
R304	K13	Screening	13-AUG-2018	11:37	-22	-31	Ethanol	Negative
		Inoculum	04-SEP-2018	07:29	0	-9	Ethanol	Negative
		Admission	13-SEP-2018	07:14	9	1	Ethanol	Negative
R305	3D7	Screening	13-AUG-2018	09:15	-23	-31	Ethanol	Negative
		Inoculum	05-SEP-2018	07:05	0	-8	Ethanol	Negative
		Admission	13-SEP-2018	07:36	8	1	Ethanol	Negative
R306	3D7	Screening	28-AUG-2018	11:19	-8	-16	Ethanol	Negative
		Inoculum	05-SEP-2018	06:59	0	-8	Ethanol	Negative
		Admission	13-SEP-2018	06:58	8	1	Ethanol	Negative
R307	3D7	Screening	07-AUG-2018	12:11	-29	-37	Ethanol	Negative
		Inoculum	05-SEP-2018	08:09	0	-8	Ethanol	Negative
		Admission	13-SEP-2018	06:56	8	1	Ethanol	Negative

Notes: Day relative to inoculum is the number of days relative to day of administration of the inoculum, where the day of administration = 0.
The inoculum was administered on Study Day 0 for K13-infected subjects and Study Day 1 for 3D7-infected subjects.
Day relative to IP is the number of days relative to day of administration of Artesunate, where the day of dose = 1.

16.2.4.10 G6PD

Listing 16.2.4.10
G6PD

Randomisation Number	Challenge Agent	Visit	Date of Sample Collection	Time of Sample Collection	Day Relative to Inoculum	Day Relative to IP	Parameter (unit) [Normal Range]	Result	Clinical Assessment
R103	K13	Screening	01-NOV-2017	12:16	-13	-22	Glucose-6-Phosphate Dehydrogenase (U/L)	2156.4	Normal
							Glucose-6-Phosphate Dehydrogenase/Hb (U/g Hb) [7 - 20.5]	15.8	
R104	K13	Screening	09-NOV-2017	10:32	-5	-14	Glucose-6-Phosphate Dehydrogenase (U/L)	2044.2	Normal
							Glucose-6-Phosphate Dehydrogenase/Hb (U/g Hb) [7 - 20.5]	16.1	
R105	K13	Screening	08-NOV-2017	12:46	-6	-15	Glucose-6-Phosphate Dehydrogenase (U/L)	2144.9	Normal
							Glucose-6-Phosphate Dehydrogenase/Hb (U/g Hb) [7 - 20.5]	15.4	
R106	3D7	Screening	09-NOV-2017	10:57	-6	-14	Glucose-6-Phosphate Dehydrogenase (U/L)	1828.1	Normal
							Glucose-6-Phosphate Dehydrogenase/Hb (U/g Hb) [7 - 20.5]	14.7	
R107	3D7	Screening	03-NOV-2017	11:25	-12	-20	Glucose-6-Phosphate Dehydrogenase (U/L)	2128.1	Normal
							Glucose-6-Phosphate Dehydrogenase/Hb (U/g Hb) [7 - 20.5]	15.2	

Notes: Day relative to inoculum is the number of days relative to day of administration of the inoculum, where the day of administration = 0.
The inoculum was administered on Study Day 0 for K13-infected subjects and Study Day 1 for 3D7-infected subjects.
Day relative to IP is the number of days relative to day of administration of Artesunate, where the day of dose = 1.
Hb = Hemoglobin.

Printed: 24FEB2020 (Final)

Listing 16.2.4.10
G6PD

Randomisation Number	Challenge Agent	Visit	Date of Sample Collection	Time of Sample Collection	Day Relative to Inoculum	Day Relative to IP	Parameter (unit) [Normal Range]	Result	Clinical Assessment
R108	3D7	Screening	26-OCT-2017	09:30	-20	-28	Glucose-6-Phosphate Dehydrogenase (U/L)	2531.8	Normal
							Glucose-6-Phosphate Dehydrogenase/Hb (U/g Hb) [7 - 20.5]	17.7	
R201	K13	Screening	08-FEB-2018	12:01	-11	-20	Glucose-6-Phosphate Dehydrogenase (U/L)	2412.4	Normal
							Glucose-6-Phosphate Dehydrogenase/Hb (U/g Hb) [7 - 20.5]	19.3	
R202	K13	Screening	13-FEB-2018	11:20	-6	-15	Glucose-6-Phosphate Dehydrogenase (U/L)	2033.9	Normal
							Glucose-6-Phosphate Dehydrogenase/Hb (U/g Hb) [7 - 20.5]	16.2	
R203	K13	Screening	13-FEB-2018	10:18	-6	-15	Glucose-6-Phosphate Dehydrogenase (U/L)	2054.2	Normal
							Glucose-6-Phosphate Dehydrogenase/Hb (U/g Hb) [7 - 20.5]	16.7	
R204	K13	Screening	05-FEB-2018	10:46	-14	-23	Glucose-6-Phosphate Dehydrogenase (U/L)	2292.5	Normal
							Glucose-6-Phosphate Dehydrogenase/Hb (U/g Hb) [7 - 20.5]	17.7	

Notes: Day relative to inoculum is the number of days relative to day of administration of the inoculum, where the day of administration = 0.
The inoculum was administered on Study Day 0 for K13-infected subjects and Study Day 1 for 3D7-infected subjects.
Day relative to IP is the number of days relative to day of administration of Artesunate, where the day of dose = 1.
Hb = Hemoglobin.

Printed: 24FEB2020 (Final)

Listing 16.2.4.10
G6PD

Randomisation Number	Challenge Agent	Visit	Date of Sample Collection	Time of Sample Collection	Day Relative to Inoculum	Day Relative to IP	Parameter (unit) [Normal Range]	Result	Clinical Assessment
R205	K13	Screening	31-JAN-2018	10:01	-19	-28	Glucose-6-Phosphate Dehydrogenase (U/L)	1727.4	Normal
							Glucose-6-Phosphate Dehydrogenase/Hb (U/g Hb) [7 - 20.5]	14.7	
R206	K13	Screening	06-FEB-2018	13:32	-13	-22	Glucose-6-Phosphate Dehydrogenase (U/L)	1709.7	Normal
							Glucose-6-Phosphate Dehydrogenase/Hb (U/g Hb) [7 - 20.5]	13.1	
R207	K13	Screening	14-FEB-2018	11:34	-5	-14	Glucose-6-Phosphate Dehydrogenase (U/L)	2189.5	Normal
							Glucose-6-Phosphate Dehydrogenase/Hb (U/g Hb) [7 - 20.5]	17	
R208	3D7	Screening	01-FEB-2018	12:12	-19	-27	Glucose-6-Phosphate Dehydrogenase (U/L)	2299.1	Normal
							Glucose-6-Phosphate Dehydrogenase/Hb (U/g Hb) [7 - 20.5]	16.8	
R209	3D7	Screening	08-FEB-2018	10:36	-12	-20	Glucose-6-Phosphate Dehydrogenase (U/L)	2489.8	Normal
							Glucose-6-Phosphate Dehydrogenase/Hb (U/g Hb) [7 - 20.5]	17.1	

Notes: Day relative to inoculum is the number of days relative to day of administration of the inoculum, where the day of administration = 0.
The inoculum was administered on Study Day 0 for K13-infected subjects and Study Day 1 for 3D7-infected subjects.
Day relative to IP is the number of days relative to day of administration of Artesunate, where the day of dose = 1.
Hb = Hemoglobin.

Listing 16.2.4.10
G6PD

Randomisation Number	Challenge Agent	Visit	Date of Sample Collection	Time of Sample Collection	Day Relative to Inoculum	Day Relative to IP	Parameter (unit) [Normal Range]	Result	Clinical Assessment
R210	3D7	Screening	12-FEB-2018	09:38	-8	-16	Glucose-6-Phosphate Dehydrogenase (U/L)	2068.8	Normal
							Glucose-6-Phosphate Dehydrogenase/Hb (U/g Hb) [7 - 20.5]	18.5	
R302	K13	Screening	16-AUG-2018	09:36	-19	-28	Glucose-6-Phosphate Dehydrogenase (U/L)	2404.7	Normal
							Glucose-6-Phosphate Dehydrogenase/Hb (U/g Hb) [7 - 20.5]	16.7	
R303	K13	Screening	09-AUG-2018	10:30	-26	-35	Glucose-6-Phosphate Dehydrogenase (U/L)	2283.9	Normal
							Glucose-6-Phosphate Dehydrogenase/Hb (U/g Hb) [7 - 20.5]	18.5	
R304	K13	Screening	13-AUG-2018	11:57	-22	-31	Glucose-6-Phosphate Dehydrogenase (U/L)	2556.2	Normal
							Glucose-6-Phosphate Dehydrogenase/Hb (U/g Hb) [7 - 20.5]	16.1	
R305	3D7	Screening	13-AUG-2018	09:38	-23	-31	Glucose-6-Phosphate Dehydrogenase (U/L)	1824.6	Normal
							Glucose-6-Phosphate Dehydrogenase/Hb (U/g Hb) [7 - 20.5]	12.7	

Notes: Day relative to inoculum is the number of days relative to day of administration of the inoculum, where the day of administration = 0.
The inoculum was administered on Study Day 0 for K13-infected subjects and Study Day 1 for 3D7-infected subjects.
Day relative to IP is the number of days relative to day of administration of Artesunate, where the day of dose = 1.
Hb = Hemoglobin.

Listing 16.2.4.10
G6PD

Randomisation Number	Challenge Agent	Visit	Date of Sample Collection	Time of Sample Collection	Day Relative to Inoculum	Day Relative to IP	Parameter (unit) [Normal Range]	Result	Clinical Assessment
R306	3D7	Screening	28-AUG-2018	11:39	-8	-16	Glucose-6-Phosphate Dehydrogenase (U/L)	2318.8	Normal
							Glucose-6-Phosphate Dehydrogenase/Hb (U/g Hb) [7 - 20.5]	15.4	
R307	3D7	Screening	07-AUG-2018	12:31	-29	-37	Glucose-6-Phosphate Dehydrogenase (U/L)	1742.1	Normal
							Glucose-6-Phosphate Dehydrogenase/Hb (U/g Hb) [7 - 20.5]	15.5	

Notes: Day relative to inoculum is the number of days relative to day of administration of the inoculum, where the day of administration = 0.
The inoculum was administered on Study Day 0 for K13-infected subjects and Study Day 1 for 3D7-infected subjects.
Day relative to IP is the number of days relative to day of administration of Artesunate, where the day of dose = 1.
Hb = Hemoglobin.

16.2.4.11 Body Weight, Height and BMI

Listing 16.2.4.11
Body Weight, Height and BMI

Randomisation Number	Challenge Agent	Visit	Date of Test	Day Relative to Inoculum	Day Relative to IP	Test (unit)	Result
R103	K13	Screening	01-NOV-2017	-13	-22	Weight (kg)	63.4
						Height (cm)	178
						Body Mass Index (kg/m²)	20
R104	K13	Screening	09-NOV-2017	-5	-14	Weight (kg)	103.8
						Height (cm)	187
						Body Mass Index (kg/m²)	29.7
R105	K13	Screening	08-NOV-2017	-6	-15	Weight (kg)	78.1
						Height (cm)	172
						Body Mass Index (kg/m²)	26.4
R106	3D7	Screening	09-NOV-2017	-6	-14	Weight (kg)	78.7
						Height (cm)	172
						Body Mass Index (kg/m²)	26.6
R107	3D7	Screening	03-NOV-2017	-12	-20	Weight (kg)	77.9
						Height (cm)	175
						Body Mass Index (kg/m²)	25.4
R108	3D7	Screening	26-OCT-2017	-20	-28	Weight (kg)	77.8
						Height (cm)	168
						Body Mass Index (kg/m²)	27.6

Notes: BMI = Body Mass Index.
Day relative to inoculum is the number of days relative to day of administration of the inoculum, where the day of administration = 0.
The inoculum was administered on Study Day 0 for K13-infected subjects and Study Day 1 for 3D7-infected subjects.
Day relative to IP is the number of days relative to day of administration of Artesunate, where the day of dose = 1.

Listing 16.2.4.11
Body Weight, Height and BMI

Randomisation Number	Challenge Agent	Visit	Date of Test	Day Relative to Inoculum	Day Relative to IP	Test (unit)	Result
R201	K13	Screening	08-FEB-2018	-11	-20	Weight (kg)	78.1
						Height (cm)	167
						Body Mass Index (kg/m²)	28
R202	K13	Screening	13-FEB-2018	-6	-15	Weight (kg)	66.4
						Height (cm)	176
						Body Mass Index (kg/m²)	21.4
R203	K13	Screening	13-FEB-2018	-6	-15	Weight (kg)	59.4
						Height (cm)	164
						Body Mass Index (kg/m²)	22.1
R204	K13	Screening	05-FEB-2018	-14	-23	Weight (kg)	81.3
						Height (cm)	176
						Body Mass Index (kg/m²)	26.2
R205	K13	Screening	31-JAN-2018	-19	-28	Weight (kg)	72.3
						Height (cm)	176
						Body Mass Index (kg/m²)	23.3
R206	K13	Screening	06-FEB-2018	-13	-22	Weight (kg)	67
						Height (cm)	161
						Body Mass Index (kg/m²)	25.8

Notes: BMI = Body Mass Index.
Day relative to inoculum is the number of days relative to day of administration of the inoculum, where the day of administration = 0.
The inoculum was administered on Study Day 0 for K13-infected subjects and Study Day 1 for 3D7-infected subjects.
Day relative to IP is the number of days relative to day of administration of Artesunate, where the day of dose = 1.

Listing 16.2.4.11
Body Weight, Height and BMI

Randomisation Number	Challenge Agent	Visit	Date of Test	Day Relative to Inoculum	Day Relative to IP	Test (unit)	Result
R207	K13	Screening	14-FEB-2018	-5	-14	Weight (kg)	73.3
						Height (cm)	173
						Body Mass Index (kg/m²)	24.5
R208	3D7	Screening	01-FEB-2018	-19	-27	Weight (kg)	85.5
						Height (cm)	165
						Body Mass Index (kg/m²)	31.4
R209	3D7	Screening	08-FEB-2018	-12	-20	Weight (kg)	85.4
						Height (cm)	180
						Body Mass Index (kg/m²)	26.4
R210	3D7	Screening	12-FEB-2018	-8	-16	Weight (kg)	54.2
						Height (cm)	162
						Body Mass Index (kg/m²)	20.7
R302	K13	Screening	16-AUG-2018	-19	-28	Weight (kg)	86.5
						Height (cm)	182
						Body Mass Index (kg/m²)	26.1
R303	K13	Screening	09-AUG-2018	-26	-35	Weight (kg)	69.7
						Height (cm)	166
						Body Mass Index (kg/m²)	25.3

Notes: BMI = Body Mass Index.
Day relative to inoculum is the number of days relative to day of administration of the inoculum, where the day of administration = 0.
The inoculum was administered on Study Day 0 for K13-infected subjects and Study Day 1 for 3D7-infected subjects.
Day relative to IP is the number of days relative to day of administration of Artesunate, where the day of dose = 1.

Listing 16.2.4.11
Body Weight, Height and BMI

Randomisation Number	Challenge Agent	Visit	Date of Test	Day Relative to Inoculum	Day Relative to IP	Test (unit)	Result
R304	K13	Screening	13-AUG-2018	-22	-31	Weight (kg)	64.7
						Height (cm)	171
						Body Mass Index (kg/m²)	22.1
R305	3D7	Screening	13-AUG-2018	-23	-31	Weight (kg)	82.4
						Height (cm)	173
						Body Mass Index (kg/m²)	27.5
R306	3D7	Screening	28-AUG-2018	-8	-16	Weight (kg)	63.4
						Height (cm)	180
						Body Mass Index (kg/m²)	19.6
R307	3D7	Screening	07-AUG-2018	-29	-37	Weight (kg)	76.4
						Height (cm)	169
						Body Mass Index (kg/m²)	26.7

Notes: BMI = Body Mass Index.
Day relative to inoculum is the number of days relative to day of administration of the inoculum, where the day of administration = 0.
The inoculum was administered on Study Day 0 for K13-infected subjects and Study Day 1 for 3D7-infected subjects.
Day relative to IP is the number of days relative to day of administration of Artesunate, where the day of dose = 1.

16.2.4.12a Prior Medications

Listing 16.2.4.12a
Prior Medications

Rando. Number	Challenge Agent	PM. No.	ATC Class/ Preferred Name/ Medication Name Verbatim	Start Date/ Start Time / End Date/ End Time	Start Day/ End Day Relative to Inoculum	Start Day/ End Day Relative to IP	Dose	Unit	Route	Frequency	Indication
R104	K13	1	GENERAL NUTRIENTS/ PROTEIN SUPPLEMENTS/ Protein Shake	09-OCT-2017/ UN:UN/ 09-NOV-2017/ UN:UN	-36/ -5	-45/ -14	30	g	ORAL	QD	Body building
		2	ANTIEMETICS AND ANTINAUSEANTS/ PROCHLORPERAZINE/ Prochlorperazine	16-OCT-2017/ 15:00/ 23-OCT-2017/ UN:UN	-29/ -22	-38/ -31	10	mg	ORAL	TID	Vertigo (otitis media)
R106	3D7	1	SEX HORMONES AND MODULATORS OF THE GENITAL SYSTEM/ EUGYNON/ Femme-Tab ED	UN-JAN-2015/ UN:UN/ Ongoing			1	TABLET	ORAL	QD	Contraception
R201	K13	1	SEX HORMONES AND MODULATORS OF THE GENITAL SYSTEM/ ETONOGESTREL/ Implanon	UN-JUL-2017/ UN:UN/ Ongoing			1	IMPLANT	INTRADERMAL	3 YEARLY	Contraception
		2	ANTIHISTAMINES FOR SYSTEMIC USE/ LORATADINE/ Claratyne	06-FEB-2018/ 09:00/ 06-FEB-2018/ 09:00	-13/ -13	-22/ -22	10	mg	ORAL	ONCE	Air conditioning sensitivity
R204	K13	1	SEX HORMONES AND MODULATORS OF THE GENITAL SYSTEM/ EUGYNON/ Loette	UN-JUN-2017/ UN:UN/ Ongoing			1	TABLET	ORAL	QD	Contraception

Notes: Medications were coded to preferred drug name and ATC class using WHO DD version 2015:03
Day relative to inoculum is number of days relative to day of administration of the inoculum.
Day relative to IP is the number of days relative to day of administration of Artesunate, where the day of dose = 1.
Prior medications with an end date after the administrations of inoculum are also classified as concomitant medication.
Rando. = Randomization; PM. No. = Prior medication number; IP = Artesunate; UN = Unknown; UNK = Unknown.
QD = Daily; TID = 3 times per day; PRN = As needed.

Listing 16.2.4.12a
Prior Medications

Rando. Number	Challenge Agent	PM. No.	ATC Class/ Preferred Name/ Medication Name Verbatim	Start Date/ Start Time / End Date/ End Time	Start Day/ End Day Relative to Inoculum	Start Day/ End Day Relative to IP	Dose	Unit	Route	Frequency	Indication
R204	K13	2	ANALGESICS/ PARACETAMOL/ Paracetamol	03-FEB-2018/ 17:00/ 03-FEB-2018/ 17:00	-16/ -16	-25/ -25	1000	mg	ORAL	ONCE	Toothache
		3	ANALGESICS/ PARACETAMOL/ Paracetamol	05-FEB-2018/ 05:15/ 05-FEB-2018/ 05:15	-14/ -14	-23/ -23	1000	mg	ORAL	ONCE	Headache
		4	ANTIINFLAMMATORY AND ANTIRHEUMATIC PRODUCTS/ NAPROXEN/ Naprogesic	10-FEB-2018/ 14:00/ 10-FEB-2018/ 14:00	-9/ -9	-18/ -18	550	mg	ORAL	ONCE	Dysmenorrhoea
R205	K13	1	SEX HORMONES AND MODULATORS OF THE GENITAL SYSTEM/ DIANE/ Estelle 35 ED	UN-UNK-2008/ UN:UN/ Ongoing			1	TABLET	ORAL	QD	Contraception

Notes: Medications were coded to preferred drug name and ATC class using WHO DD version 2015:03
Day relative to inoculum is number of days relative to day of administration of the inoculum.
Day relative to IP is the number of days relative to day of administration of Artesunate, where the day of dose = 1.
Prior medications with an end date after the administrations of inoculum are also classified as concomitant medication.
Rando. = Randomization; PM. No. = Prior medication number; IP = Artesunate; UN = Unknown; UNK = Unknown.
QD = Daily; TID = 3 times per day; PRN = As needed.

Listing 16.2.4.12a
Prior Medications

Rando. Number	Challenge Agent	PM. No.	ATC Class/ Preferred Name/ Medication Name Verbatim	Start Date/ Start Time / End Date/ End Time	Start Day/ End Day Relative to Inoculum	Start Day/ End Day Relative to IP	Dose	Unit	Route	Frequency	Indication
R206	K13	1	OTHER GYNECOLOGICALS/ LEVONORGESTREL/ mirena coil	UN-UNK-2013/ UN:UN/ Ongoing			1	IUD	INTRAUTERINE	EVERY THREE YEARS	contraception
		2	OTHER NERVOUS SYSTEM DRUGS/ NICOTINE/ nicotine patch - nicobate	25-JAN-2018/ UN:UN/ 26-JAN-2018/ UN:UN	-25/ -24	-34/ -33	211	mg/24 hours	TRANSDERMAL	ONCE	trying to quit smoking
		3	ANTIINFLAMMATORY AND ANTIRHEUMATIC PRODUCTS/ IBUPROFEN/ ibuprofen	02-FEB-2018/ 12:00/ 12-FEB-2018/ 12:00	-17/ -7	-26/ -16	200	mg	ORAL	ONCE	headache

Notes: Medications were coded to preferred drug name and ATC class using WHO DD version 2015:03
Day relative to inoculum is number of days relative to day of administration of the inoculum.
Day relative to IP is the number of days relative to day of administration of Artesunate, where the day of dose = 1.
Prior medications with an end date after the administrations of inoculum are also classified as concomitant medication.
Rando. = Randomization; PM. No. = Prior medication number; IP = Artesunate; UN = Unknown; UNK = Unknown.
QD = Daily; TID = 3 times per day; PRN = As needed.

Listing 16.2.4.12a
Prior Medications

Rando. Number	Challenge Agent	PM. No.	ATC Class/ Preferred Name/ Medication Name Verbatim	Start Date/ Start Time / End Date/ End Time	Start Day/ End Day Relative to Inoculum	Start Day/ End Day Relative to IP	Dose	Unit	Route	Frequency	Indication
R206	K13	4	ANTIINFLAMMATORY AND ANTIRHEUMATIC PRODUCTS/ IBUPROFEN/ ibuprofen	17-FEB-2018/ 08:00/ 17-FEB-2018/ 08:00	-2/ -2	-11/ -11	400	mg	ORAL	ONCE	headache
R210	3D7	1	SEX HORMONES AND MODULATORS OF THE GENITAL SYSTEM/ EUGYNON/ Levlen ED	UN-UNK-2016/ UN:UN/ Ongoing			1	TABLET	ORAL	QD	Contraception
		2	ANTIINFLAMMATORY AND ANTIRHEUMATIC PRODUCTS/ IBUPROFEN/ Ibuprofen	17-FEB-2018/ 16:00/ 17-FEB-2018/ 16:00	-3/ -3	-11/ -11	200	mg	ORAL	ONCE	headache
R303	K13	1	SEX HORMONES AND MODULATORS OF THE GENITAL SYSTEM/ DIANE/ Estelle 35 ED	UN-AUG-2017/ UN:UN/ Ongoing			1	TABLET	ORAL	QD	Contraception
		2	VITAMINS/ MULTIVITAMINS, PLAIN/ Multivitamin Swiss	UN-FEB-2018/ UN:UN/ 28-JUL-2018/ 08:00	/ -38	/ -47	1	TABLET	ORAL	QD	Multivitamin
R305	3D7	1	OPHTHALMOLOGICALS/ SYSTANE LUBRICANT/ Systane lubricant eye drops (0.4%)	UN-UNK-2017/ UN:UN/ Ongoing			1	DROP	TOPICAL	PRN	Dry eyes

Notes: Medications were coded to preferred drug name and ATC class using WHO DD version 2015:03
Day relative to inoculum is number of days relative to day of administration of the inoculum.
Day relative to IP is the number of days relative to day of administration of Artesunate, where the day of dose = 1.
Prior medications with an end date after the administrations of inoculum are also classified as concomitant medication.
Rando. = Randomization; PM. No. = Prior medication number; IP = Artesunate; UN = Unknown; UNK = Unknown.
QD = Daily; TID = 3 times per day; PRN = As needed.

Listing 16.2.4.12a
Prior Medications

Rando. Number	Challenge Agent	PM. No.	ATC Class/ Preferred Name/ Medication Name Verbatim	Start Date/ Start Time / End Date/ End Time	Start Day/ End Day Relative to Inoculum	Start Day/ End Day Relative to IP	Dose	Unit	Route	Frequency	Indication
R307	3D7	1	ANALGESICS/ PARACETAMOL/ Paracetamol	02-SEP-2018/ 10:00/ 02-SEP-2018/ 10:00	-3/ -3	-11/ -11	1000	mg	ORAL	ONCE	Headache

Notes: Medications were coded to preferred drug name and ATC class using WHO DD version 2015:03
Day relative to inoculum is number of days relative to day of administration of the inoculum.
Day relative to IP is the number of days relative to day of administration of Artesunate, where the day of dose = 1.
Prior medications with an end date after the administrations of inoculum are also classified as concomitant medication.
Rando. = Randomization; PM. No. = Prior medication number; IP = Artesunate; UN = Unknown; UNK = Unknown.
QD = Daily; TID = 3 times per day; PRN = As needed.

16.2.4.12b Concomitant Medications

Listing 16.2.4.12b
Concomitant Medications

Rando. Number	Challenge Agent	CM. No.	ATC Class/ Preferred Name/ Medication Name Verbatim	Start Date/ Start Time / End Date/ End Time	Start Day/ End Day Relative to Inoculum	Start Day/ End Day Relative to IP	Dose	Unit	Route	Frequency	Indication
R103	K13	1	ANTIINFLAMMATORY AND ANTIRHEUMATIC PRODUCTS/ IBUPROFEN/ Ibuprofen	24-NOV-2017/ 08:33/ 24-NOV-2017/ 08:33	10/ 10	2/ 2	400	mg	ORAL	ONCE	Headache
		2	ANTIINFLAMMATORY AND ANTIRHEUMATIC PRODUCTS/ IBUPROFEN/ Ibuprofen	24-NOV-2017/ 20:39/ 24-NOV-2017/ 20:39	10/ 10	2/ 2	400	mg	ORAL	ONCE	myalgia
		3	ANTIINFLAMMATORY AND ANTIRHEUMATIC PRODUCTS/ IBUPROFEN/ Ibuprofen	25-NOV-2017/ 20:00/ 25-NOV-2017/ 20:00	11/ 11	3/ 3	400	mg	ORAL	ONCE	Intermittent headache
		4	ANTIINFLAMMATORY AND ANTIRHEUMATIC PRODUCTS/ IBUPROFEN/ ibuprofen	26-NOV-2017/ 09:42/ 26-NOV-2017/ 09:42	12/ 12	4/ 4	400	mg	ORAL	ONCE	headache
		5	ANALGESICS/ PARACETAMOL/ Paracetamol	26-NOV-2017/ 11:12/ 26-NOV-2017/ 11:12	12/ 12	4/ 4	1000	mg	ORAL	ONCE	myalgia
		6	ANTIINFLAMMATORY AND ANTIRHEUMATIC PRODUCTS/ IBUPROFEN/ Ibuprofen	27-NOV-2017/ 11:07/ 27-NOV-2017/ 11:07	13/ 13	5/ 5	400	mg	ORAL	ONCE	headache

Notes: Medications were coded to preferred drug name and ATC class using WHO DD version 2015:03
Day relative to inoculum is number of days relative to day of administration of the inoculum.
Day relative to IP is the number of days relative to day of administration of Artesunate, where the day of dose = 1.
Concomitant medications are defined as those medications taken following the administration of inoculum or medication which has continued from prior medications. CM. No. = Concomitant medication number; IP = Artesunate; UN = Unknown; UNK = Unknown.
QD = Daily; BID = Twice per day; PRN = As needed.

Listing 16.2.4.12b
Concomitant Medications

Rando. Number	Challenge Agent	CM. No.	ATC Class/ Preferred Name/ Medication Name Verbatim	Start Date/ Start Time / End Date/ End Time	Start Day/ End Day Relative to Inoculum	Start Day/ End Day Relative to IP	Dose	Unit	Route	Frequency	Indication
R103	K13	7	ANTIINFLAMMATORY AND ANTIRHEUMATIC PRODUCTS/ IBUPROFEN/ Ibuprofen	28-NOV-2017/ 21:00/ 28-NOV-2017/ 21:00	14/ 14	6/ 6	400	mg	ORAL	ONCE	Headache
		8	ANTIINFLAMMATORY AND ANTIRHEUMATIC PRODUCTS/ IBUPROFEN/ Ibuprofen	01-DEC-2017/ 15:00/ 01-DEC-2017/ 15:00	17/ 17	9/ 9	400	mg	ORAL	ONCE	headache
R104	K13	3	ANTIINFLAMMATORY AND ANTIRHEUMATIC PRODUCTS/ IBUPROFEN/ Ibuprofen	22-NOV-2017/ 13:15/ 22-NOV-2017/ 13:15	8/ 8	-1/ -1	400	mg	ORAL	ONCE	Arthralgia
		4	ANTIINFLAMMATORY AND ANTIRHEUMATIC PRODUCTS/ IBUPROFEN/ Ibuprofen	23-NOV-2017/ 20:31/ 23-NOV-2017/ 20:31	9/ 9	1/ 1	400	mg	ORAL	ONCE	Headache, fever, arthralgia, hot spells
		5	ANTIINFLAMMATORY AND ANTIRHEUMATIC PRODUCTS/ IBUPROFEN/ Ibuprofen	24-NOV-2017/ 06:45/ 24-NOV-2017/ 06:45	10/ 10	2/ 2	400	mg	ORAL	ONCE	Arthralgia, fever, intermittent headache, myalgia
		6	ANTIINFLAMMATORY AND ANTIRHEUMATIC PRODUCTS/ IBUPROFEN/ Ibuprofen	24-NOV-2017/ 18:44/ 24-NOV-2017/ 18:44	10/ 10	2/ 2	400	mg	ORAL	ONCE	Myalgia, intermittent headache

Notes: Medications were coded to preferred drug name and ATC class using WHO DD version 2015:03
Day relative to inoculum is number of days relative to day of administration of the inoculum.
Day relative to IP is the number of days relative to day of administration of Artesunate, where the day of dose = 1.
Concomitant medications are defined as those medications taken following the administration of inoculum or medication which has continued from prior medications. CM. No. = Concomitant medication number; IP = Artesunate; UN = Unknown; UNK = Unknown.
QD = Daily; BID = Twice per day; PRN = As needed.

Listing 16.2.4.12b
Concomitant Medications

Rando. Number	Challenge Agent	CM. No.	ATC Class/ Preferred Name/ Medication Name Verbatim	Start Date/ Start Time / End Date/ End Time	Start Day/ End Day Relative to Inoculum	Start Day/ End Day Relative to IP	Dose	Unit	Route	Frequency	Indication
R104	K13	7	ANALGESICS/ PARACETAMOL/ Paracetamol	24-NOV-2017/ 20:30/ 24-NOV-2017/ 20:30	10/ 10	2/ 2	1000	mg	ORAL	ONCE	Myalgia, intermittent headache
		8	ANTIINFLAMMATORY AND ANTIRHEUMATIC PRODUCTS/ IBUPROFEN/ Ibuprofen	25-NOV-2017/ 08:39/ 25-NOV-2017/ 08:39	11/ 11	3/ 3	400	mg	ORAL	ONCE	Intermittent headache
		9	ANTIINFLAMMATORY AND ANTIRHEUMATIC PRODUCTS/ IBUPROFEN/ Ibuprofen	25-NOV-2017/ 18:35/ 25-NOV-2017/ 18:35	11/ 11	3/ 3	400	mg	ORAL	ONCE	Intermittent headache
		10	ANALGESICS/ PARACETAMOL/ Paracetamol	25-NOV-2017/ 19:32/ 25-NOV-2017/ 19:32	11/ 11	3/ 3	1000	mg	ORAL	ONCE	Intermittent headache
		11	ANTIINFLAMMATORY AND ANTIRHEUMATIC PRODUCTS/ IBUPROFEN/ Ibuprofen	26-NOV-2017/ 11:35/ 26-NOV-2017/ 11:35	12/ 12	4/ 4	400	mg	ORAL	ONCE	Intermittent arthralgia
		12	ANTIINFLAMMATORY AND ANTIRHEUMATIC PRODUCTS/ IBUPROFEN/ Ibuprofen	26-NOV-2017/ 20:30/ 26-NOV-2017/ 20:30	12/ 12	4/ 4	400	mg	ORAL	ONCE	Intermittent arthralgia

Notes: Medications were coded to preferred drug name and ATC class using WHO DD version 2015:03
Day relative to inoculum is number of days relative to day of administration of the inoculum.
Day relative to IP is the number of days relative to day of administration of Artesunate, where the day of dose = 1.
Concomitant medications are defined as those medications taken following the administration of inoculum or medication which has continued from prior medications. CM. No. = Concomitant medication number; IP = Artesunate; UN = Unknown; UNK = Unknown.
QD = Daily; BID = Twice per day; PRN = As needed.

Listing 16.2.4.12b
Concomitant Medications

Rando. Number	Challenge Agent	CM. No.	ATC Class/ Preferred Name/ Medication Name Verbatim	Start Date/ Start Time / End Date/ End Time	Start Day/ End Day Relative to Inoculum	Start Day/ End Day Relative to IP	Dose	Unit	Route	Frequency	Indication
R104	K13	13	ANTIINFLAMMATORY AND ANTIRHEUMATIC PRODUCTS/ IBUPROFEN/ Ibuprofen	27-NOV-2017/ 11:00/ 27-NOV-2017/ 11:00	13/ 13	5/ 5	400	mg	ORAL	ONCE	Intermittent arthralgia
R106	3D7	1	SEX HORMONES AND MODULATORS OF THE GENITAL SYSTEM/ EUGYNON/ Femme-Tab ED	UN-JAN-2015/ UN:UN/ Ongoing			1	TABLET	ORAL	QD	Contraception
		2	ANTIHISTAMINES FOR SYSTEMIC USE/ LORATADINE/ Loratadine	18-NOV-2017/ 12:30/ 18-NOV-2017/ 12:31	3/ 3	-5/ -5	10	mg	ORAL	ONCE	Hayfever (chronic)
		3	ANTIINFLAMMATORY AND ANTIRHEUMATIC PRODUCTS/ IBUPROFEN/ Ibuprofen	19-NOV-2017/ 21:30/ 19-NOV-2017/ 21:30	4/ 4	-4/ -4	400	mg	ORAL	ONCE	Headache
		4	ANTIINFLAMMATORY AND ANTIRHEUMATIC PRODUCTS/ IBUPROFEN/ Ibuprofen	24-NOV-2017/ 11:41/ 24-NOV-2017/ 11:41	9/ 9	2/ 2	400	mg	ORAL	ONCE	Intermittent headache, myalgia
		5	ANTIINFLAMMATORY AND ANTIRHEUMATIC PRODUCTS/ IBUPROFEN/ Ibuprofen	25-NOV-2017/ 19:50/ 25-NOV-2017/ 19:50	10/ 10	3/ 3	400	mg	ORAL	ONCE	Headache

Notes: Medications were coded to preferred drug name and ATC class using WHO DD version 2015:03
Day relative to inoculum is number of days relative to day of administration of the inoculum.
Day relative to IP is the number of days relative to day of administration of Artesunate, where the day of dose = 1.
Concomitant medications are defined as those medications taken following the administration of inoculum or medication which has continued from prior medications. CM. No. = Concomitant medication number; IP = Artesunate; UN = Unknown; UNK = Unknown.
QD = Daily; BID = Twice per day; PRN = As needed.

Listing 16.2.4.12b
Concomitant Medications

Rando. Number	Challenge Agent	CM. No.	ATC Class/ Preferred Name/ Medication Name Verbatim	Start Date/ Start Time / End Date/ End Time	Start Day/ End Day Relative to Inoculum	Start Day/ End Day Relative to IP	Dose	Unit	Route	Frequency	Indication
R106	3D7	6	ANTIINFLAMMATORY AND ANTIRHEUMATIC PRODUCTS/ IBUPROFEN/ Ibuprofen	26-NOV-2017/ 11:30/ 26-NOV-2017/ 11:30	11/ 11	4/ 4	400	mg	ORAL	ONCE	Headache
		7	ANTIINFLAMMATORY AND ANTIRHEUMATIC PRODUCTS/ IBUPROFEN/ Ibuprofen	27-NOV-2017/ 08:35/ 27-NOV-2017/ 08:35	12/ 12	5/ 5	400	mg	ORAL	ONCE	Back pain
		8	ANTIINFLAMMATORY AND ANTIRHEUMATIC PRODUCTS/ IBUPROFEN/ Ibuprofen	28-NOV-2017/ 22:00/ 28-NOV-2017/ 22:00	13/ 13	6/ 6	400	mg	ORAL	ONCE	Dysmenorrhea
		9	ANTIINFLAMMATORY AND ANTIRHEUMATIC PRODUCTS/ IBUPROFEN/ Ibuprofen	29-NOV-2017/ 16:00/ 29-NOV-2017/ 17:00	14/ 14	7/ 7	400	mg	ORAL	ONCE	dysmenorrhea
		10	ANTIINFLAMMATORY AND ANTIRHEUMATIC PRODUCTS/ IBUPROFEN/ Ibuprofen	30-NOV-2017/ 17:45/ 30-NOV-2017/ 17:45	15/ 15	8/ 8	400	mg	ORAL	ONCE	Intermittent headache
		11	ANTIINFLAMMATORY AND ANTIRHEUMATIC PRODUCTS/ IBUPROFEN/ Ibuprofen	01-DEC-2017/ 20:00/ 01-DEC-2017/ 20:00	16/ 16	9/ 9	400	mg	ORAL	ONCE	fever

Notes: Medications were coded to preferred drug name and ATC class using WHO DD version 2015:03
Day relative to inoculum is number of days relative to day of administration of the inoculum.
Day relative to IP is the number of days relative to day of administration of Artesunate, where the day of dose = 1.
Concomitant medications are defined as those medications taken following the administration of inoculum or medication which has continued from prior medications. CM. No. = Concomitant medication number; IP = Artesunate; UN = Unknown; UNK = Unknown.
QD = Daily; BID = Twice per day; PRN = As needed.

Listing 16.2.4.12b
Concomitant Medications

Rando. Number	Challenge Agent	CM. No.	ATC Class/ Preferred Name/ Medication Name Verbatim	Start Date/ Start Time / End Date/ End Time	Start Day/ End Day Relative to Inoculum	Start Day/ End Day Relative to IP	Dose	Unit	Route	Frequency	Indication
R106	3D7	12	ANTIINFLAMMATORY AND ANTIRHEUMATIC PRODUCTS/ IBUPROFEN/ Ibuprofen	03-DEC-2017/ 20:30/ 03-DEC-2017/ 20:30	18/ 18	11/ 11	400	mg	ORAL	ONCE	Intermittent headache
		13	ANTIHISTAMINES FOR SYSTEMIC USE/ LORATADINE/ Loratadine	13-DEC-2017/ 10:30/ 13-DEC-2017/ 10:30	28/ 28	21/ 21	10	mg	ORAL	ONCE	Hayfever (chronic)
R107	3D7	1	ANTIEMETICS AND ANTINAUSEANTS/ ONDANSETRON/ Ondansetron	21-NOV-2017/ 07:45/ 21-NOV-2017/ 07:45	6/ 6	-2/ -2	4	mg	ORAL	ONCE	Nausea
		2	ANTIEMETICS AND ANTINAUSEANTS/ ONDANSETRON/ Ondansetron	23-NOV-2017/ 09:38/ 23-NOV-2017/ 09:38	8/ 8	1/ 1	4	mg	ORAL	ONCE	Nausea
		3	ANTIEMETICS AND ANTINAUSEANTS/ ONDANSETRON/ Ondansetron	23-NOV-2017/ 12:55/ 23-NOV-2017/ 12:55	8/ 8	1/ 1	4	mg	ORAL	ONCE	Nausea
		4	ANTIEMETICS AND ANTINAUSEANTS/ ONDANSETRON/ Ondansetron	03-DEC-2017/ 21:22/ 03-DEC-2017/ 21:22	18/ 18	11/ 11	4	mg	ORAL	ONCE	Myalgia

Notes: Medications were coded to preferred drug name and ATC class using WHO DD version 2015:03
Day relative to inoculum is number of days relative to day of administration of the inoculum.
Day relative to IP is the number of days relative to day of administration of Artesunate, where the day of dose = 1.
Concomitant medications are defined as those medications taken following the administration of inoculum or medication which has continued from prior medications. CM. No. = Concomitant medication number; IP = Artesunate; UN = Unknown; UNK = Unknown.
QD = Daily; BID = Twice per day; PRN = As needed.

Listing 16.2.4.12b
Concomitant Medications

Rando. Number	Challenge Agent	CM. No.	ATC Class/ Preferred Name/ Medication Name Verbatim	Start Date/ Start Time / End Date/ End Time	Start Day/ End Day Relative to Inoculum	Start Day/ End Day Relative to IP	Dose	Unit	Route	Frequency	Indication
R107	3D7	5	ANTIINFLAMMATORY AND ANTIRHEUMATIC PRODUCTS/ IBUPROFEN/ ibuprofen	04-DEC-2017/ 21:50/ 04-DEC-2017/ 21:50	19/ 19	12/ 12	400	mg	ORAL	ONCE	headache
R108	3D7	1	ANALGESICS/ PARACETAMOL/ Paracetamol	22-NOV-2017/ 22:00/ 22-NOV-2017/ 22:00	7/ 7	-1/ -1	1000	mg	ORAL	ONCE	Headache
		2	ANTIINFLAMMATORY AND ANTIRHEUMATIC PRODUCTS/ IBUPROFEN/ Ibuprofen	23-NOV-2017/ 23:19/ 23-NOV-2017/ 23:19	8/ 8	1/ 1	400	mg	ORAL	ONCE	Intermittent headache
		3	ANTIINFLAMMATORY AND ANTIRHEUMATIC PRODUCTS/ IBUPROFEN/ Ibuprofen	24-NOV-2017/ 07:30/ 24-NOV-2017/ 07:30	9/ 9	2/ 2	400	mg	ORAL	ONCE	Intermittent abdominal discomfort, intermittent headache
		4	ANTIINFLAMMATORY AND ANTIRHEUMATIC PRODUCTS/ IBUPROFEN/ Ibuprofen	24-NOV-2017/ 16:45/ 24-NOV-2017/ 16:45	9/ 9	2/ 2	400	mg	ORAL	ONCE	Intermittent fever, intermittent headache
		5	ANTIINFLAMMATORY AND ANTIRHEUMATIC PRODUCTS/ IBUPROFEN/ Ibuprofen	25-NOV-2017/ 08:30/ 25-NOV-2017/ 08:30	10/ 10	3/ 3	400	mg	ORAL	ONCE	Intermittent headache

Notes: Medications were coded to preferred drug name and ATC class using WHO DD version 2015:03
Day relative to inoculum is number of days relative to day of administration of the inoculum.
Day relative to IP is the number of days relative to day of administration of Artesunate, where the day of dose = 1.
Concomitant medications are defined as those medications taken following the administration of inoculum or medication which has continued from prior medications. CM. No. = Concomitant medication number; IP = Artesunate; UN = Unknown; UNK = Unknown.
QD = Daily; BID = Twice per day; PRN = As needed.

Listing 16.2.4.12b
Concomitant Medications

Rando. Number	Challenge Agent	CM. No.	ATC Class/ Preferred Name/ Medication Name Verbatim	Start Date/ Start Time / End Date/ End Time	Start Day/ End Day Relative to Inoculum	Start Day/ End Day Relative to IP	Dose	Unit	Route	Frequency	Indication
R108	3D7	6	ANTIINFLAMMATORY AND ANTIRHEUMATIC PRODUCTS/ IBUPROFEN/ Ibuprofen	25-NOV-2017/ 18:31/ 25-NOV-2017/ 18:31	10/ 10	3/ 3	400	mg	ORAL	ONCE	Intermittent headache
		7	ANTIINFLAMMATORY AND ANTIRHEUMATIC PRODUCTS/ IBUPROFEN/ Ibuprofen	01-DEC-2017/ 11:30/ 01-DEC-2017/ 11:30	16/ 16	9/ 9	400	mg	ORAL	ONCE	Headache
		8	ANALGESICS/ PARACETAMOL/ Paracetamol	03-DEC-2017/ 20:00/ 03-DEC-2017/ 20:00	18/ 18	11/ 11	1000	mg	ORAL	ONCE	Right shoulder pain
R201	K13	1	SEX HORMONES AND MODULATORS OF THE GENITAL SYSTEM/ ETONOGESTREL/ Implanon	UN-JUL-2017/ UN:UN/ Ongoing			1	IMPLANT	INTRADERMAL	3 YEARLY	Contraception
		3	ANTIINFLAMMATORY AND ANTIRHEUMATIC PRODUCTS/ IBUPROFEN/ ibuprofen	01-MAR-2018/ 17:45/ 01-MAR-2018/ 17:45	10/ 10	2/ 2	400	mg	ORAL	ONCE	headache and irritated, itchy throat
		4	ANTIHISTAMINES FOR SYSTEMIC USE/ LORATADINE/ claratyne	10-MAR-2018/ 19:30/ 10-MAR-2018/ 19:30	19/ 19	11/ 11	10	mg	ORAL	ONCE	air conditioning sensitivity

Notes: Medications were coded to preferred drug name and ATC class using WHO DD version 2015:03
Day relative to inoculum is number of days relative to day of administration of the inoculum.
Day relative to IP is the number of days relative to day of administration of Artesunate, where the day of dose = 1.
Concomitant medications are defined as those medications taken following the administration of inoculum or medication which has continued from prior medications. CM. No. = Concomitant medication number; IP = Artesunate; UN = Unknown; UNK = Unknown.
QD = Daily; BID = Twice per day; PRN = As needed.

Listing 16.2.4.12b
Concomitant Medications

Rando. Number	Challenge Agent	CM. No.	ATC Class/ Preferred Name/ Medication Name Verbatim	Start Date/ Start Time / End Date/ End Time	Start Day/ End Day Relative to Inoculum	Start Day/ End Day Relative to IP	Dose	Unit	Route	Frequency	Indication
R201	K13	5	ANTIINFLAMMATORY AND ANTIRHEUMATIC PRODUCTS/ IBUPROFEN/ ibuprofen	12-MAR-2018/ 22:30/ 12-MAR-2018/ 22:30	21/ 21	13/ 13	400	mg	ORAL	ONCE	headache
		6	ANTIHISTAMINES FOR SYSTEMIC USE/ LORATADINE/ claratyne	14-MAR-2018/ 19:30/ 14-MAR-2018/ 19:30	23/ 23	15/ 15	10	mg	ORAL	ONCE	air conditioning sensitivity
		7	ANTIHISTAMINES FOR SYSTEMIC USE/ LORATADINE/ claratyne	17-MAR-2018/ 13:30/ 17-MAR-2018/ 13:30	26/ 26	18/ 18	10	mg	ORAL	ONCE	air conditioning sensitivity
R202	K13	1	ANTIINFLAMMATORY AND ANTIRHEUMATIC PRODUCTS/ IBUPROFEN/ ibuprofen	03-MAR-2018/ 09:59/ 03-MAR-2018/ 09:59	12/ 12	4/ 4	400	mg	ORAL	ONCE	fever
		2	ANTIINFLAMMATORY AND ANTIRHEUMATIC PRODUCTS/ IBUPROFEN/ ibuprofen	03-MAR-2018/ 22:30/ 03-MAR-2018/ 22:30	12/ 12	4/ 4	400	mg	ORAL	ONCE	Febrile

Notes: Medications were coded to preferred drug name and ATC class using WHO DD version 2015:03
Day relative to inoculum is number of days relative to day of administration of the inoculum.
Day relative to IP is the number of days relative to day of administration of Artesunate, where the day of dose = 1.
Concomitant medications are defined as those medications taken following the administration of inoculum or medication which has continued from prior medications. CM. No. = Concomitant medication number; IP = Artesunate; UN = Unknown; UNK = Unknown.
QD = Daily; BID = Twice per day; PRN = As needed.

Listing 16.2.4.12b
Concomitant Medications

Rando. Number	Challenge Agent	CM. No.	ATC Class/ Preferred Name/ Medication Name Verbatim	Start Date/ Start Time / End Date/ End Time	Start Day/ End Day Relative to Inoculum	Start Day/ End Day Relative to IP	Dose	Unit	Route	Frequency	Indication
R202	K13	3	CORTICOSTEROIDS, DERMATOLOGICAL PREPARATIONS/ MOMETASONE/ Mometasone	14-MAR-2018/ UN:UN/ 18-MAR-2018/ UN:UN	23/ 27	15/ 19	0.1	%	TOPICAL	BID	Mosquito bite reaction
R203	K13	1	ANTIINFLAMMATORY AND ANTIRHEUMATIC PRODUCTS/ IBUPROFEN/ ibuprofen	01-MAR-2018/ 08:43/ 01-MAR-2018/ 08:43	10/ 10	2/ 2	400	mg	ORAL	ONCE	intermittent headache
		2	ANTIEMETICS AND ANTINAUSEANTS/ ONDANSETRON/ ondansetron	01-MAR-2018/ 09:11/ 01-MAR-2018/ 09:11	10/ 10	2/ 2	8	mg	ORAL	ONCE	nausea
		3	ANALGESICS/ PARACETAMOL/ paracetamol	01-MAR-2018/ 09:11/ 01-MAR-2018/ 09:11	10/ 10	2/ 2	1000	mg	ORAL	ONCE	intermittent headache
		4	ANTIINFLAMMATORY AND ANTIRHEUMATIC PRODUCTS/ IBUPROFEN/ ibuprofen	02-MAR-2018/ 10:02/ 02-MAR-2018/ 10:02	11/ 11	3/ 3	400	mg	ORAL	ONCE	intermittent headache
		5	ANTIINFLAMMATORY AND ANTIRHEUMATIC PRODUCTS/ IBUPROFEN/ ibuprofen	03-MAR-2018/ 10:02/ 03-MAR-2018/ 10:02	12/ 12	4/ 4	400	mg	ORAL	ONCE	intermittent headache

Notes: Medications were coded to preferred drug name and ATC class using WHO DD version 2015:03
Day relative to inoculum is number of days relative to day of administration of the inoculum.
Day relative to IP is the number of days relative to day of administration of Artesunate, where the day of dose = 1.
Concomitant medications are defined as those medications taken following the administration of inoculum or medication which has continued from prior medications. CM. No. = Concomitant medication number; IP = Artesunate; UN = Unknown; UNK = Unknown.
QD = Daily; BID = Twice per day; PRN = As needed.

Listing 16.2.4.12b
Concomitant Medications

Rando. Number	Challenge Agent	CM. No.	ATC Class/ Preferred Name/ Medication Name Verbatim	Start Date/ Start Time / End Date/ End Time	Start Day/ End Day Relative to Inoculum	Start Day/ End Day Relative to IP	Dose	Unit	Route	Frequency	Indication
R203	K13	6	ANTIINFLAMMATORY AND ANTIRHEUMATIC PRODUCTS/ IBUPROFEN/ ibuprofen	03-MAR-2018/ 21:15/ 03-MAR-2018/ 21:15	12/ 12	4/ 4	400	mg	ORAL	ONCE	chills, fever, myalgia
		7	ANTIEMETICS AND ANTINAUSEANTS/ ONDANSETRON/ ondansetron	03-MAR-2018/ 21:15/ 03-MAR-2018/ 21:15	12/ 12	4/ 4	8	mg	ORAL	ONCE	intermittent nausea
		8	ANALGESICS/ PARACETAMOL/ paracetamol	03-MAR-2018/ 22:30/ 03-MAR-2018/ 22:30	12/ 12	4/ 4	1000	mg	ORAL	ONCE	Chills, fever, myalgia
		9	ANTIEMETICS AND ANTINAUSEANTS/ ONDANSETRON/ Ondansetron	04-MAR-2018/ 07:00/ 04-MAR-2018/ 07:00	13/ 13	5/ 5	8	mg	ORAL	ONCE	intermittent nausea
		10	ANTIINFLAMMATORY AND ANTIRHEUMATIC PRODUCTS/ IBUPROFEN/ ibuprofen	04-MAR-2018/ 10:30/ 04-MAR-2018/ 10:30	13/ 13	5/ 5	400	mg	ORAL	ONCE	malaise, intermittent myalgia and intermittent headache
		11	ANALGESICS/ PARACETAMOL/ paracetamol	04-MAR-2018/ 12:00/ 04-MAR-2018/ 12:00	13/ 13	5/ 5	1000	mg	ORAL	ONCE	malaise, intermittent myalgia and intermittent headache

Notes: Medications were coded to preferred drug name and ATC class using WHO DD version 2015:03
Day relative to inoculum is number of days relative to day of administration of the inoculum.
Day relative to IP is the number of days relative to day of administration of Artesunate, where the day of dose = 1.
Concomitant medications are defined as those medications taken following the administration of inoculum or medication which has continued from prior medications. CM. No. = Concomitant medication number; IP = Artesunate; UN = Unknown; UNK = Unknown.
QD = Daily; BID = Twice per day; PRN = As needed.

Listing 16.2.4.12b
Concomitant Medications

Rando. Number	Challenge Agent	CM. No.	ATC Class/ Preferred Name/ Medication Name Verbatim	Start Date/ Start Time / End Date/ End Time	Start Day/ End Day Relative to Inoculum	Start Day/ End Day Relative to IP	Dose	Unit	Route	Frequency	Indication
R203	K13	12	ANTIINFLAMMATORY AND ANTIRHEUMATIC PRODUCTS/ IBUPROFEN/ ibuprofen	06-MAR-2018/ 18:30/ 06-MAR-2018/ 18:30	15/ 15	7/ 7	400	mg	ORAL	ONCE	migraine
		13	ANTIINFLAMMATORY AND ANTIRHEUMATIC PRODUCTS/ IBUPROFEN/ ibuprofen	10-MAR-2018/ 09:00/ 10-MAR-2018/ 09:00	19/ 19	11/ 11	400	mg	ORAL	ONCE	as migraine prevention
		14	ANTIEMETICS AND ANTINAUSEANTS/ ONDANSETRON/ ondansetron	10-MAR-2018/ 09:00/ 10-MAR-2018/ 09:00	19/ 19	11/ 11	8	mg	ORAL	ONCE	as migraine prevention
		15	ANTIINFLAMMATORY AND ANTIRHEUMATIC PRODUCTS/ IBUPROFEN/ ibuprofen	10-MAR-2018/ 15:00/ 10-MAR-2018/ 15:00	19/ 19	11/ 11	400	mg	ORAL	ONCE	as migraine prevention
		16	ANTIEMETICS AND ANTINAUSEANTS/ ONDANSETRON/ ondansetron	10-MAR-2018/ 15:00/ 10-MAR-2018/ 15:00	19/ 19	11/ 11	8	mg	ORAL	ONCE	as migraine prevention of vomiting
		17	ANTIEMETICS AND ANTINAUSEANTS/ ONDANSETRON/ Ondansetron	10-MAR-2018/ 21:00/ 10-MAR-2018/ 21:00	19/ 19	11/ 11	8	mg	ORAL	ONCE	As migraine prevention of vomiting

Notes: Medications were coded to preferred drug name and ATC class using WHO DD version 2015:03
Day relative to inoculum is number of days relative to day of administration of the inoculum.
Day relative to IP is the number of days relative to day of administration of Artesunate, where the day of dose = 1.
Concomitant medications are defined as those medications taken following the administration of inoculum or medication which has continued from prior medications. CM. No. = Concomitant medication number; IP = Artesunate; UN = Unknown; UNK = Unknown.
QD = Daily; BID = Twice per day; PRN = As needed.

Listing 16.2.4.12b
Concomitant Medications

Rando. Number	Challenge Agent	CM. No.	ATC Class/ Preferred Name/ Medication Name Verbatim	Start Date/ Start Time / End Date/ End Time	Start Day/ End Day Relative to Inoculum	Start Day/ End Day Relative to IP	Dose	Unit	Route	Frequency	Indication
R203	K13	18	ANTIINFLAMMATORY AND ANTIRHEUMATIC PRODUCTS/ IBUPROFEN/ ibuprofen	10-MAR-2018/ 21:00/ 10-MAR-2018/ 21:00	19/ 19	11/ 11	400	mg	ORAL	ONCE	as migraine prevention
		19	ANTIINFLAMMATORY AND ANTIRHEUMATIC PRODUCTS/ IBUPROFEN/ ibuprofen	11-MAR-2018/ 08:50/ 11-MAR-2018/ 08:50	20/ 20	12/ 12	400	mg	ORAL	ONCE	as migraine prevention
R204	K13	1	SEX HORMONES AND MODULATORS OF THE GENITAL SYSTEM/ EUGYNON/ Loette	UN-JUN-2017/ UN:UN/ Ongoing			1	TABLET	ORAL	QD	Contraception
		5	ANTIINFLAMMATORY AND ANTIRHEUMATIC PRODUCTS/ IBUPROFEN/ ibuprofen	01-MAR-2018/ 12:20/ 01-MAR-2018/ 12:20	10/ 10	2/ 2	400	mg	ORAL	ONCE	intermittent headache
		6	ANTIINFLAMMATORY AND ANTIRHEUMATIC PRODUCTS/ IBUPROFEN/ ibuprofen	04-MAR-2018/ 14:00/ 04-MAR-2018/ 14:00	13/ 13	5/ 5	400	mg	ORAL	ONCE	intermittent headache
		7	ANTIINFLAMMATORY AND ANTIRHEUMATIC PRODUCTS/ IBUPROFEN/ ibuprofen	05-MAR-2018/ 03:00/ 05-MAR-2018/ 03:00	14/ 14	6/ 6	400	mg	ORAL	ONCE	intermittent headache

Notes: Medications were coded to preferred drug name and ATC class using WHO DD version 2015:03
Day relative to inoculum is number of days relative to day of administration of the inoculum.
Day relative to IP is the number of days relative to day of administration of Artesunate, where the day of dose = 1.
Concomitant medications are defined as those medications taken following the administration of inoculum or medication which has continued from prior medications. CM. No. = Concomitant medication number; IP = Artesunate; UN = Unknown; UNK = Unknown.
QD = Daily; BID = Twice per day; PRN = As needed.

Listing 16.2.4.12b
Concomitant Medications

Rando. Number	Challenge Agent	CM. No.	ATC Class/ Preferred Name/ Medication Name Verbatim	Start Date/ Start Time / End Date/ End Time	Start Day/ End Day Relative to Inoculum	Start Day/ End Day Relative to IP	Dose	Unit	Route	Frequency	Indication
R205	K13	1	SEX HORMONES AND MODULATORS OF THE GENITAL SYSTEM/ DIANE/ Estelle 35 ED	UN-UNK-2008/ UN:UN/ Ongoing			1	TABLET	ORAL	QD	Contraception
		2	ANTIINFLAMMATORY AND ANTIRHEUMATIC PRODUCTS/ IBUPROFEN/ ibuprofen	01-MAR-2018/ 13:52/ 01-MAR-2018/ 13:52	10/ 10	2/ 2	400	mg	ORAL	ONCE	lower back pain
		3	ANALGESICS/ PARACETAMOL/ paracetamol	01-MAR-2018/ 17:52/ 01-MAR-2018/ 17:52	10/ 10	2/ 2	1000	mg	ORAL	ONCE	Intermittent headache
		4	CORTICOSTEROIDS, DERMATOLOGICAL PREPARATIONS/ MOMETASONE/ Mometasone	14-MAR-2018/ UN:UN/ 14-MAR-2018/ UN:UN	23/ 23	15/ 15	0.1	%	TOPICAL	ONCE	Mosquito bite reaction
R206	K13	1	OTHER GYNECOLOGICALS/ LEVONORGESTREL/ mirena coil	UN-UNK-2013/ UN:UN/ Ongoing			1	IUD	INTRAUTERINE	EVERY THREE YEARS	contraception
		5	ANTIINFLAMMATORY AND ANTIRHEUMATIC PRODUCTS/ IBUPROFEN/ ibuprofen	24-FEB-2018/ 22:00/ 24-FEB-2018/ 22:00	5/ 5	-4/ -4	400	mg	ORAL	ONCE	dysmenorrhoea

Notes: Medications were coded to preferred drug name and ATC class using WHO DD version 2015:03
Day relative to inoculum is number of days relative to day of administration of the inoculum.
Day relative to IP is the number of days relative to day of administration of Artesunate, where the day of dose = 1.
Concomitant medications are defined as those medications taken following the administration of inoculum or medication which has continued from prior medications. CM. No. = Concomitant medication number; IP = Artesunate; UN = Unknown; UNK = Unknown.
QD = Daily; BID = Twice per day; PRN = As needed.

Listing 16.2.4.12b
Concomitant Medications

Rando. Number	Challenge Agent	CM. No.	ATC Class/ Preferred Name/ Medication Name Verbatim	Start Date/ Start Time / End Date/ End Time	Start Day/ End Day Relative to Inoculum	Start Day/ End Day Relative to IP	Dose	Unit	Route	Frequency	Indication
R206	K13	6	ANTIINFLAMMATORY AND ANTIRHEUMATIC PRODUCTS/ IBUPROFEN/ ibuprofen	26-FEB-2018/ 21:30/ 26-FEB-2018/ 21:30	7/ 7	-2/ -2	400	mg	ORAL	ONCE	Upper respiratory tract infection
		7	ANTIINFLAMMATORY AND ANTIRHEUMATIC PRODUCTS/ IBUPROFEN/ ibuprofen	27-FEB-2018/ 16:00/ 27-FEB-2018/ 16:00	8/ 8	-1/ -1	400	mg	ORAL	ONCE	Upper respiratory tract infection
		8	ANTIINFLAMMATORY AND ANTIRHEUMATIC PRODUCTS/ IBUPROFEN/ ibuprofen	27-FEB-2018/ 21:00/ 27-FEB-2018/ 21:00	8/ 8	-1/ -1	400	mg	ORAL	ONCE	Upper respiratory tract infection
		9	ANTIINFLAMMATORY AND ANTIRHEUMATIC PRODUCTS/ IBUPROFEN/ ibuprofen	28-FEB-2018/ 17:55/ 28-FEB-2018/ 17:55	9/ 9	1/ 1	400	mg	ORAL	ONCE	intermittent headache and intermittent myalgia
		10	ANTIINFLAMMATORY AND ANTIRHEUMATIC PRODUCTS/ IBUPROFEN/ ibuprofen	28-FEB-2018/ 23:24/ 28-FEB-2018/ 23:24	9/ 9	1/ 1	400	mg	ORAL	ONCE	intermittent headache and myalgia
		11	ANTIINFLAMMATORY AND ANTIRHEUMATIC PRODUCTS/ IBUPROFEN/ ibuprofen	01-MAR-2018/ 05:48/ 01-MAR-2018/ 05:48	10/ 10	2/ 2	400	mg	ORAL	ONCE	intermittent headache and intermittent myalgia

Notes: Medications were coded to preferred drug name and ATC class using WHO DD version 2015:03
Day relative to inoculum is number of days relative to day of administration of the inoculum.
Day relative to IP is the number of days relative to day of administration of Artesunate, where the day of dose = 1.
Concomitant medications are defined as those medications taken following the administration of inoculum or medication which has continued from prior medications. CM. No. = Concomitant medication number; IP = Artesunate; UN = Unknown; UNK = Unknown.
QD = Daily; BID = Twice per day; PRN = As needed.

Listing 16.2.4.12b
Concomitant Medications

Rando. Number	Challenge Agent	CM. No.	ATC Class/ Preferred Name/ Medication Name Verbatim	Start Date/ Start Time / End Date/ End Time	Start Day/ End Day Relative to Inoculum	Start Day/ End Day Relative to IP	Dose	Unit	Route	Frequency	Indication
R206	K13	12	ANALGESICS/ PARACETAMOL/ paracetamol	01-MAR-2018/ 09:35/ 01-MAR-2018/ 09:35	10/ 10	2/ 2	1000	mg	ORAL	ONCE	intermittent headache and intermittent myalgia
		13	ANTIINFLAMMATORY AND ANTIRHEUMATIC PRODUCTS/ IBUPROFEN/ ibuprofen	01-MAR-2018/ 13:37/ 01-MAR-2018/ 13:37	10/ 10	2/ 2	400	mg	ORAL	ONCE	intermittent headache and intermittent myalgia
		14	ANALGESICS/ PARACETAMOL/ paracetamol	01-MAR-2018/ 18:10/ 01-MAR-2018/ 18:10	10/ 10	2/ 2	1000	mg	ORAL	ONCE	intermittent headache and intermittent myalgia
		15	ANTIINFLAMMATORY AND ANTIRHEUMATIC PRODUCTS/ IBUPROFEN/ ibuprofen	02-MAR-2018/ 01:36/ 02-MAR-2018/ 01:36	11/ 11	3/ 3	400	mg	ORAL	ONCE	intermittent headache
		16	ANALGESICS/ PARACETAMOL/ paracetamol	02-MAR-2018/ 08:45/ 02-MAR-2018/ 08:45	11/ 11	3/ 3	1000	mg	ORAL	ONCE	Intermittent headache
		17	ANTIINFLAMMATORY AND ANTIRHEUMATIC PRODUCTS/ IBUPROFEN/ ibuprofen	02-MAR-2018/ 18:23/ 02-MAR-2018/ 18:23	11/ 11	3/ 3	400	mg	ORAL	ONCE	intermittent headache and myalgia
		18	ANTIINFLAMMATORY AND ANTIRHEUMATIC PRODUCTS/ IBUPROFEN/ ibuprofen	03-MAR-2018/ 05:30/ 03-MAR-2018/ 05:30	12/ 12	4/ 4	400	mg	ORAL	ONCE	intermittent myalgia

Notes: Medications were coded to preferred drug name and ATC class using WHO DD version 2015:03
Day relative to inoculum is number of days relative to day of administration of the inoculum.
Day relative to IP is the number of days relative to day of administration of Artesunate, where the day of dose = 1.
Concomitant medications are defined as those medications taken following the administration of inoculum or medication which has continued from prior medications. CM. No. = Concomitant medication number; IP = Artesunate; UN = Unknown; UNK = Unknown.
QD = Daily; BID = Twice per day; PRN = As needed.

Listing 16.2.4.12b
Concomitant Medications

Rando. Number	Challenge Agent	CM. No.	ATC Class/ Preferred Name/ Medication Name Verbatim	Start Date/ Start Time / End Date/ End Time	Start Day/ End Day Relative to Inoculum	Start Day/ End Day Relative to IP	Dose	Unit	Route	Frequency	Indication
R206	K13	19	ANTIINFLAMMATORY AND ANTIRHEUMATIC PRODUCTS/ IBUPROFEN/ ibuprofen	04-MAR-2018/ 23:00/ 04-MAR-2018/ 23:00	13/ 13	5/ 5	400	mg	ORAL	ONCE	intermittent headache
		20	ANTIINFLAMMATORY AND ANTIRHEUMATIC PRODUCTS/ IBUPROFEN/ ibuprofen	11-MAR-2018/ 20:00/ 11-MAR-2018/ 20:00	20/ 20	12/ 12	400	mg	ORAL	ONCE	intermittent myalgia
		21	CORTICOSTEROIDS, DERMATOLOGICAL PREPARATIONS/ MOMETASONE/ Mometasone	14-MAR-2018/ 20:00/ 02-APR-2018/ 20:00	23/ 42	15/ 34	0.1	%	TOPICAL	QD	Mosquito bite reaction - itchiness
R207	K13	1	ANTIINFLAMMATORY AND ANTIRHEUMATIC PRODUCTS/ IBUPROFEN/ ibuprofen	01-MAR-2018/ 08:25/ 01-MAR-2018/ 08:25	10/ 10	2/ 2	400	mg	ORAL	ONCE	intermittent headache
		2	ANTIINFLAMMATORY AND ANTIRHEUMATIC PRODUCTS/ IBUPROFEN/ ibuprofen	01-MAR-2018/ 18:03/ 01-MAR-2018/ 18:03	10/ 10	2/ 2	400	mg	ORAL	ONCE	intermittent headache
		3	ANTIINFLAMMATORY AND ANTIRHEUMATIC PRODUCTS/ IBUPROFEN/ ibuprofen	03-MAR-2018/ 15:00/ 03-MAR-2018/ 15:00	12/ 12	4/ 4	400	mg	ORAL	ONCE	intermittent headache

Notes: Medications were coded to preferred drug name and ATC class using WHO DD version 2015:03
Day relative to inoculum is number of days relative to day of administration of the inoculum.
Day relative to IP is the number of days relative to day of administration of Artesunate, where the day of dose = 1.
Concomitant medications are defined as those medications taken following the administration of inoculum or medication which has continued from prior medications. CM. No. = Concomitant medication number; IP = Artesunate; UN = Unknown; UNK = Unknown.
QD = Daily; BID = Twice per day; PRN = As needed.

Listing 16.2.4.12b
Concomitant Medications

Rando. Number	Challenge Agent	CM. No.	ATC Class/ Preferred Name/ Medication Name Verbatim	Start Date/ Start Time / End Date/ End Time	Start Day/ End Day Relative to Inoculum	Start Day/ End Day Relative to IP	Dose	Unit	Route	Frequency	Indication
R207	K13	4	CORTICOSTEROIDS, DERMATOLOGICAL PREPARATIONS/ MOMETASONE/ mometasone cream	15-MAR-2018/ 20:00/ 15-MAR-2018/ 20:00	24/ 24	16/ 16	0.1	%	TOPICAL	ONCE	mosquito bites - itchiness
R209	3D7	1	ANTIINFLAMMATORY AND ANTIRHEUMATIC PRODUCTS/ IBUPROFEN/ Ibuprofen	01-MAR-2018/ 00:03/ 01-MAR-2018/ 00:03	9/ 9	2/ 2	400	mg	ORAL	ONCE	Intermittent headache
		2	ANTIINFLAMMATORY AND ANTIRHEUMATIC PRODUCTS/ IBUPROFEN/ Ibuprofen	01-MAR-2018/ 08:43/ 01-MAR-2018/ 08:43	9/ 9	2/ 2	400	mg	ORAL	ONCE	Intermittent headache
		3	ANALGESICS/ PARACETAMOL/ Paracetamol	01-MAR-2018/ 12:12/ 01-MAR-2018/ 12:12	9/ 9	2/ 2	1000	mg	ORAL	ONCE	Intermittent headache
		4	ANTIINFLAMMATORY AND ANTIRHEUMATIC PRODUCTS/ IBUPROFEN/ Ibuprofen	02-MAR-2018/ 07:14/ 02-MAR-2018/ 07:14	10/ 10	3/ 3	400	mg	ORAL	ONCE	Intermittent headache, Intermittent myalgia
		5	ANTIINFLAMMATORY AND ANTIRHEUMATIC PRODUCTS/ IBUPROFEN/ Ibuprofen	02-MAR-2018/ 16:31/ 02-MAR-2018/ 16:31	10/ 10	3/ 3	400	mg	ORAL	ONCE	Intermittent headache, Intermittent myalgia

Notes: Medications were coded to preferred drug name and ATC class using WHO DD version 2015:03
Day relative to inoculum is number of days relative to day of administration of the inoculum.
Day relative to IP is the number of days relative to day of administration of Artesunate, where the day of dose = 1.
Concomitant medications are defined as those medications taken following the administration of inoculum or medication which has continued from prior medications. CM. No. = Concomitant medication number; IP = Artesunate; UN = Unknown; UNK = Unknown.
QD = Daily; BID = Twice per day; PRN = As needed.

Listing 16.2.4.12b
Concomitant Medications

Rando. Number	Challenge Agent	CM. No.	ATC Class/ Preferred Name/ Medication Name Verbatim	Start Date/ Start Time / End Date/ End Time	Start Day/ End Day Relative to Inoculum	Start Day/ End Day Relative to IP	Dose	Unit	Route	Frequency	Indication
R209	3D7	6	ANTIINFLAMMATORY AND ANTIRHEUMATIC PRODUCTS/ IBUPROFEN/ Ibuprofen	03-MAR-2018/ 03:55/ 03-MAR-2018/ 03:55	11/ 11	4/ 4	400	mg	ORAL	ONCE	Intermittent headache
		7	ANTIINFLAMMATORY AND ANTIRHEUMATIC PRODUCTS/ IBUPROFEN/ Ibuprofen	03-MAR-2018/ 19:00/ 03-MAR-2018/ 19:00	11/ 11	4/ 4	400	mg	ORAL	ONCE	Intermittent headache
		8	TOPICAL PRODUCTS FOR JOINT AND MUSCULAR PAIN/ DEEP HEAT SPORT/ Deep heat mentholatum regular relief	03-MAR-2018/ 22:00/ 03-MAR-2018/ 22:00	11/ 11	4/ 4		mg/g	TOPICAL	ONCE	Intermittent myalgia
		9	TOPICAL PRODUCTS FOR JOINT AND MUSCULAR PAIN/ DEEP HEAT SPORT/ deep heat mentholatum regular relief	04-MAR-2018/ 22:00/ 04-MAR-2018/ 22:00	12/ 12	5/ 5		mg/g	TOPICAL	ONCE	intermittent myalgia
		10	ANTIINFLAMMATORY AND ANTIRHEUMATIC PRODUCTS/ IBUPROFEN/ Ibuprofen	08-MAR-2018/ 21:00/ 08-MAR-2018/ 21:00	16/ 16	9/ 9	400	mg	ORAL	ONCE	Intermittent myalgia
		11	ANTIINFLAMMATORY AND ANTIRHEUMATIC PRODUCTS/ IBUPROFEN/ ibuprofen	09-MAR-2018/ 22:00/ 09-MAR-2018/ 22:00	17/ 17	10/ 10	400	mg	ORAL	ONCE	Intermittent myalgia

Notes: Medications were coded to preferred drug name and ATC class using WHO DD version 2015:03
Day relative to inoculum is number of days relative to day of administration of the inoculum.
Day relative to IP is the number of days relative to day of administration of Artesunate, where the day of dose = 1.
Concomitant medications are defined as those medications taken following the administration of inoculum or medication which has continued from prior medications. CM. No. = Concomitant medication number; IP = Artesunate; UN = Unknown; UNK = Unknown.
QD = Daily; BID = Twice per day; PRN = As needed.

Listing 16.2.4.12b
Concomitant Medications

Rando. Number	Challenge Agent	CM. No.	ATC Class/ Preferred Name/ Medication Name Verbatim	Start Date/ Start Time / End Date/ End Time	Start Day/ End Day Relative to Inoculum	Start Day/ End Day Relative to IP	Dose	Unit	Route	Frequency	Indication
R210	3D7	1	SEX HORMONES AND MODULATORS OF THE GENITAL SYSTEM/ EUGYNON/ Levlen ED	UN-UNK-2016/ UN:UN/ Ongoing			1	TABLET	ORAL	QD	Contraception
		3	ANTIINFLAMMATORY AND ANTIRHEUMATIC PRODUCTS/ IBUPROFEN/ ibuprofen	23-FEB-2018/ 14:00/ 23-FEB-2018/ 14:00	3/ 3	-5/ -5	400	mg	ORAL	ONCE	Myalgia
		4	ANTIINFLAMMATORY AND ANTIRHEUMATIC PRODUCTS/ IBUPROFEN/ ibuprofen	28-FEB-2018/ 23:45/ 28-FEB-2018/ 23:45	8/ 8	1/ 1	400	mg	ORAL	ONCE	Myalgia
		5	ANTIINFLAMMATORY AND ANTIRHEUMATIC PRODUCTS/ IBUPROFEN/ ibuprofen	01-MAR-2018/ 10:20/ 01-MAR-2018/ 10:20	9/ 9	2/ 2	400	mg	ORAL	ONCE	Intermittent headache, intermittent myalgia
		6	ANTIINFLAMMATORY AND ANTIRHEUMATIC PRODUCTS/ IBUPROFEN/ ibuprofen	01-MAR-2018/ 18:00/ 01-MAR-2018/ 18:00	9/ 9	2/ 2	400	mg	ORAL	ONCE	Intermittent myalgia
		7	ANTIINFLAMMATORY AND ANTIRHEUMATIC PRODUCTS/ IBUPROFEN/ ibuprofen	04-MAR-2018/ 11:30/ 04-MAR-2018/ 11:30	12/ 12	5/ 5	400	mg	ORAL	ONCE	nausea

Notes: Medications were coded to preferred drug name and ATC class using WHO DD version 2015:03
Day relative to inoculum is number of days relative to day of administration of the inoculum.
Day relative to IP is the number of days relative to day of administration of Artesunate, where the day of dose = 1.
Concomitant medications are defined as those medications taken following the administration of inoculum or medication which has continued from prior medications. CM. No. = Concomitant medication number; IP = Artesunate; UN = Unknown; UNK = Unknown.
QD = Daily; BID = Twice per day; PRN = As needed.

Listing 16.2.4.12b
Concomitant Medications

Rando. Number	Challenge Agent	CM. No.	ATC Class/ Preferred Name/ Medication Name Verbatim	Start Date/ Start Time / End Date/ End Time	Start Day/ End Day Relative to Inoculum	Start Day/ End Day Relative to IP	Dose	Unit	Route	Frequency	Indication
R210	3D7	8	ANTIINFLAMMATORY AND ANTIRHEUMATIC PRODUCTS/ IBUPROFEN/ ibuprofen	05-MAR-2018/ 11:30/ 05-MAR-2018/ 11:30	13/ 13	6/ 6	400	mg	ORAL	ONCE	intermittent headache and sore throat
		9	ANTIINFLAMMATORY AND ANTIRHEUMATIC PRODUCTS/ IBUPROFEN/ ibuprofen	06-MAR-2018/ 17:00/ 06-MAR-2018/ 17:00	14/ 14	7/ 7	400	mg	ORAL	ONCE	intermittent headache and sore throat
		10	ANTIINFLAMMATORY AND ANTIRHEUMATIC PRODUCTS/ IBUPROFEN/ ibuprofen	07-MAR-2018/ 21:00/ 07-MAR-2018/ 21:00	15/ 15	8/ 8	400	mg	ORAL	ONCE	intermittent headache
		11	ANTIINFLAMMATORY AND ANTIRHEUMATIC PRODUCTS/ IBUPROFEN/ ibuprofen	08-MAR-2018/ 13:00/ 08-MAR-2018/ 13:00	16/ 16	9/ 9	400	mg	ORAL	ONCE	Intermittent sweating, myalgia, intermittent headache
		12	ANTIINFLAMMATORY AND ANTIRHEUMATIC PRODUCTS/ IBUPROFEN/ ibuprofen	08-MAR-2018/ 22:00/ 08-MAR-2018/ 22:00	16/ 16	9/ 9	400	mg	ORAL	ONCE	Intermittent sweating, myalgia, intermittent headache
		13	ANTIINFLAMMATORY AND ANTIRHEUMATIC PRODUCTS/ IBUPROFEN/ ibuprofen	09-MAR-2018/ 14:00/ 09-MAR-2018/ 14:00	17/ 17	10/ 10	400	mg	ORAL	ONCE	Intermittent sweating, myalgia

Notes: Medications were coded to preferred drug name and ATC class using WHO DD version 2015:03
Day relative to inoculum is number of days relative to day of administration of the inoculum.
Day relative to IP is the number of days relative to day of administration of Artesunate, where the day of dose = 1.
Concomitant medications are defined as those medications taken following the administration of inoculum or medication which has continued from prior medications. CM. No. = Concomitant medication number; IP = Artesunate; UN = Unknown; UNK = Unknown.
QD = Daily; BID = Twice per day; PRN = As needed.

Listing 16.2.4.12b
Concomitant Medications

Rando. Number	Challenge Agent	CM. No.	ATC Class/ Preferred Name/ Medication Name Verbatim	Start Date/ Start Time / End Date/ End Time	Start Day/ End Day Relative to Inoculum	Start Day/ End Day Relative to IP	Dose	Unit	Route	Frequency	Indication
R302	K13	1	ANTIINFLAMMATORY AND ANTIRHEUMATIC PRODUCTS/ IBUPROFEN/ Ibuprofen	14-SEP-2018/ 11:30/ 14-SEP-2018/ 11:30	10/ 10	2/ 2	400	mg	ORAL	ONCE	Intermittent headache, intermittent myalgia
		2	ANTIINFLAMMATORY AND ANTIRHEUMATIC PRODUCTS/ IBUPROFEN/ Ibuprofen	15-SEP-2018/ 22:15/ 15-SEP-2018/ 22:15	11/ 11	3/ 3	400	mg	ORAL	ONCE	Intermittent headache
		3	CORTICOSTEROIDS, DERMATOLOGICAL PREPARATIONS/ MOMETASONE/ Mometasone cream	25-SEP-2018/ 10:00/ 29-SEP-2018/ 12:00	21/ 25	13/ 17	0.1	%	TOPICAL	PRN	Mosquito bites
R303	K13	1	SEX HORMONES AND MODULATORS OF THE GENITAL SYSTEM/ DIANE/ Estelle 35 ED	UN-AUG-2017/ UN:UN/ Ongoing			1	TABLET	ORAL	QD	Contraception
		3	ANTIINFLAMMATORY AND ANTIRHEUMATIC PRODUCTS/ IBUPROFEN/ Ibuprofen	13-SEP-2018/ 18:28/ 13-SEP-2018/ 18:28	9/ 9	1/ 1	400	mg	ORAL	ONCE	fever
		4	ANTIINFLAMMATORY AND ANTIRHEUMATIC PRODUCTS/ IBUPROFEN/ Ibuprofen	14-SEP-2018/ 03:29/ 14-SEP-2018/ 03:29	10/ 10	2/ 2	400	mg	ORAL	ONCE	Intermittent fever, intermittent headache

Notes: Medications were coded to preferred drug name and ATC class using WHO DD version 2015:03
Day relative to inoculum is number of days relative to day of administration of the inoculum.
Day relative to IP is the number of days relative to day of administration of Artesunate, where the day of dose = 1.
Concomitant medications are defined as those medications taken following the administration of inoculum or medication which has continued from prior medications. CM. No. = Concomitant medication number; IP = Artesunate; UN = Unknown; UNK = Unknown.
QD = Daily; BID = Twice per day; PRN = As needed.

Listing 16.2.4.12b
Concomitant Medications

Rando. Number	Challenge Agent	CM. No.	ATC Class/ Preferred Name/ Medication Name Verbatim	Start Date/ Start Time / End Date/ End Time	Start Day/ End Day Relative to Inoculum	Start Day/ End Day Relative to IP	Dose	Unit	Route	Frequency	Indication
R303	K13	5	ANTIINFLAMMATORY AND ANTIRHEUMATIC PRODUCTS/ IBUPROFEN/ Ibuprofen	14-SEP-2018/ 10:00/ 14-SEP-2018/ 10:00	10/ 10	2/ 2	400	mg	ORAL	ONCE	Intermittent headache, Intermittent rigors
		6	ANTIINFLAMMATORY AND ANTIRHEUMATIC PRODUCTS/ IBUPROFEN/ Ibuprofen	14-SEP-2018/ 17:30/ 14-SEP-2018/ 17:30	10/ 10	2/ 2	400	mg	ORAL	ONCE	Intermittent headache and fever
		7	ANALGESICS/ PARACETAMOL/ Paracetamol	14-SEP-2018/ 22:56/ 14-SEP-2018/ 22:56	10/ 10	2/ 2	1000	mg	ORAL	ONCE	Intermittent Headache
		8	ANTIEMETICS AND ANTINAUSEANTS/ ONDANSETRON/ Ondansetron	15-SEP-2018/ 03:35/ 15-SEP-2018/ 03:35	11/ 11	3/ 3	8	mg	SUBLINGUAL	ONCE	Intermittent Nausea
		9	ANTIINFLAMMATORY AND ANTIRHEUMATIC PRODUCTS/ IBUPROFEN/ Ibuprofen	17-SEP-2018/ 20:15/ 17-SEP-2018/ 20:15	13/ 13	5/ 5	400	mg	ORAL	ONCE	headache
		10	CORTICOSTEROIDS, DERMATOLOGICAL PREPARATIONS/ MOMETASONE/ Mometasone cream	25-SEP-2018/ 14:00/ 30-SEP-2018/ 14:00	21/ 26	13/ 18	0.1	%	TOPICAL	PRN	Mosquito bites reaction itching

Notes: Medications were coded to preferred drug name and ATC class using WHO DD version 2015:03
Day relative to inoculum is number of days relative to day of administration of the inoculum.
Day relative to IP is the number of days relative to day of administration of Artesunate, where the day of dose = 1.
Concomitant medications are defined as those medications taken following the administration of inoculum or medication which has continued from prior medications. CM. No. = Concomitant medication number; IP = Artesunate; UN = Unknown; UNK = Unknown.
QD = Daily; BID = Twice per day; PRN = As needed.

Listing 16.2.4.12b
Concomitant Medications

Rando. Number	Challenge Agent	CM. No.	ATC Class/ Preferred Name/ Medication Name Verbatim	Start Date/ Start Time / End Date/ End Time	Start Day/ End Day Relative to Inoculum	Start Day/ End Day Relative to IP	Dose	Unit	Route	Frequency	Indication
R304	K13	1	ANTIINFLAMMATORY AND ANTIRHEUMATIC PRODUCTS/ IBUPROFEN/ Ibuprofen	13-SEP-2018/ 19:34/ 13-SEP-2018/ 19:34	9/ 9	1/ 1	400	mg	ORAL	ONCE	Intermittent headache
		2	ANTIINFLAMMATORY AND ANTIRHEUMATIC PRODUCTS/ IBUPROFEN/ Ibuprofen	14-SEP-2018/ 07:50/ 14-SEP-2018/ 07:50	10/ 10	2/ 2	400	mg	ORAL	ONCE	Intermittent Headache
		3	ANTIINFLAMMATORY AND ANTIRHEUMATIC PRODUCTS/ IBUPROFEN/ Ibuprofen	14-SEP-2018/ 16:35/ 14-SEP-2018/ 16:35	10/ 10	2/ 2	400	mg	ORAL	ONCE	Intermittent Headache
		4	ANTIINFLAMMATORY AND ANTIRHEUMATIC PRODUCTS/ IBUPROFEN/ Ibuprofen	15-SEP-2018/ 21:19/ 15-SEP-2018/ 21:19	11/ 11	3/ 3	400	mg	ORAL	ONCE	Intermittent headache
		5	ANTIINFLAMMATORY AND ANTIRHEUMATIC PRODUCTS/ IBUPROFEN/ ibuprofen	17-SEP-2018/ 08:20/ 17-SEP-2018/ 08:20	13/ 13	5/ 5	400	mg	ORAL	ONCE	Intermittent headache and myalgia
		6	ANTIINFLAMMATORY AND ANTIRHEUMATIC PRODUCTS/ IBUPROFEN/ Ibuprofen	19-SEP-2018/ 14:00/ 19-SEP-2018/ 14:00	15/ 15	7/ 7	400	mg	ORAL	ONCE	Intermittent headache

Notes: Medications were coded to preferred drug name and ATC class using WHO DD version 2015:03
Day relative to inoculum is number of days relative to day of administration of the inoculum.
Day relative to IP is the number of days relative to day of administration of Artesunate, where the day of dose = 1.
Concomitant medications are defined as those medications taken following the administration of inoculum or medication which has continued from prior medications. CM. No. = Concomitant medication number; IP = Artesunate; UN = Unknown; UNK = Unknown.
QD = Daily; BID = Twice per day; PRN = As needed.

Listing 16.2.4.12b
Concomitant Medications

Rando. Number	Challenge Agent	CM. No.	ATC Class/ Preferred Name/ Medication Name Verbatim	Start Date/ Start Time / End Date/ End Time	Start Day/ End Day Relative to Inoculum	Start Day/ End Day Relative to IP	Dose	Unit	Route	Frequency	Indication
R305	3D7	1	OPHTHALMOLOGICALS/ SYSTANE LUBRICANT/ Systane lubricant eye drops (0.4%)	UN-UNK-2017/ UN:UN/ Ongoing			1	DROP	TOPICAL	PRN	Dry eyes
		2	ANTIINFLAMMATORY AND ANTIRHEUMATIC PRODUCTS/ IBUPROFEN/ Ibuprofen	10-SEP-2018/ 13:52/ 10-SEP-2018/ 13:52	5/ 5	-3/ -3	400	mg	ORAL	ONCE	Headache
		3	ANTIINFLAMMATORY AND ANTIRHEUMATIC PRODUCTS/ IBUPROFEN/ Ibuprofen	13-SEP-2018/ 12:21/ 13-SEP-2018/ 12:21	8/ 8	1/ 1	400	mg	ORAL	ONCE	Intermittent Headache
		4	ANTIINFLAMMATORY AND ANTIRHEUMATIC PRODUCTS/ IBUPROFEN/ Ibuprofen	14-SEP-2018/ 08:16/ 14-SEP-2018/ 08:16	9/ 9	2/ 2	400	mg	ORAL	ONCE	Intermittent headache
		5	ANTIINFLAMMATORY AND ANTIRHEUMATIC PRODUCTS/ IBUPROFEN/ Ibuprofen	14-SEP-2018/ 16:18/ 14-SEP-2018/ 16:18	9/ 9	2/ 2	400	mg	ORAL	ONCE	Myalgia
		6	ANTIINFLAMMATORY AND ANTIRHEUMATIC PRODUCTS/ IBUPROFEN/ Ibuprofen	15-SEP-2018/ 08:58/ 15-SEP-2018/ 08:58	10/ 10	3/ 3	400	mg	ORAL	ONCE	Myalgia

Notes: Medications were coded to preferred drug name and ATC class using WHO DD version 2015:03
Day relative to inoculum is number of days relative to day of administration of the inoculum.
Day relative to IP is the number of days relative to day of administration of Artesunate, where the day of dose = 1.
Concomitant medications are defined as those medications taken following the administration of inoculum or medication which has continued from prior medications. CM. No. = Concomitant medication number; IP = Artesunate; UN = Unknown; UNK = Unknown.
QD = Daily; BID = Twice per day; PRN = As needed.

Listing 16.2.4.12b
Concomitant Medications

Rando. Number	Challenge Agent	CM. No.	ATC Class/ Preferred Name/ Medication Name Verbatim	Start Date/ Start Time / End Date/ End Time	Start Day/ End Day Relative to Inoculum	Start Day/ End Day Relative to IP	Dose	Unit	Route	Frequency	Indication
R305	3D7	7	ANTIINFLAMMATORY AND ANTIRHEUMATIC PRODUCTS/ IBUPROFEN/ Ibuprofen	16-SEP-2018/ 12:00/ 16-SEP-2018/ 12:00	11/ 11	4/ 4	400	mg	ORAL	ONCE	Malaise
		8	ANTIINFLAMMATORY AND ANTIRHEUMATIC PRODUCTS/ IBUPROFEN/ Ibuprofen	17-SEP-2018/ 06:44/ 17-SEP-2018/ 06:44	12/ 12	5/ 5	400	mg	ORAL	ONCE	Headache
		9	ANTIEMETICS AND ANTINAUSEANTS/ ONDANSETRON/ ondansetron	20-SEP-2018/ 07:10/ 20-SEP-2018/ 07:10	15/ 15	8/ 8	8	mg	ORAL	ONCE	Nausea
		10	ANALGESICS/ PARACETAMOL/ Paracetamol	20-SEP-2018/ 07:15/ 20-SEP-2018/ 07:15	15/ 15	8/ 8	1000	mg	ORAL	ONCE	Malaise and arthralgia
		11	ANTIINFLAMMATORY AND ANTIRHEUMATIC PRODUCTS/ IBUPROFEN/ Ibuprofen	23-SEP-2018/ 02:00/ 23-SEP-2018/ 02:00	18/ 18	11/ 11	400	mg	ORAL	ONCE	Migraine
		12	CORTICOSTEROIDS, DERMATOLOGICAL PREPARATIONS/ MOMETASONE/ Mometasone cream	25-SEP-2018/ 10:00/ 25-SEP-2018/ 10:00	20/ 20	13/ 13	0.1	%	TOPICAL	ONCE	Mosquito bites

Notes: Medications were coded to preferred drug name and ATC class using WHO DD version 2015:03
Day relative to inoculum is number of days relative to day of administration of the inoculum.
Day relative to IP is the number of days relative to day of administration of Artesunate, where the day of dose = 1.
Concomitant medications are defined as those medications taken following the administration of inoculum or medication which has continued from prior medications. CM. No. = Concomitant medication number; IP = Artesunate; UN = Unknown; UNK = Unknown.
QD = Daily; BID = Twice per day; PRN = As needed.

Listing 16.2.4.12b
Concomitant Medications

Rando. Number	Challenge Agent	CM. No.	ATC Class/ Preferred Name/ Medication Name Verbatim	Start Date/ Start Time / End Date/ End Time	Start Day/ End Day Relative to Inoculum	Start Day/ End Day Relative to IP	Dose	Unit	Route	Frequency	Indication
R305	3D7	13	ANTIINFLAMMATORY AND ANTIRHEUMATIC PRODUCTS/ IBUPROFEN/ Ibuprofen	27-SEP-2018/ 10:45/ 27-SEP-2018/ 10:45	22/ 22	15/ 15	400	mg	ORAL	ONCE	Intermittent migraine
		14	ANTIINFLAMMATORY AND ANTIRHEUMATIC PRODUCTS/ IBUPROFEN/ Ibuprofen	28-SEP-2018/ 20:00/ 28-SEP-2018/ 20:00	23/ 23	16/ 16	400	mg	ORAL	ONCE	Migraine
		15	ANTIINFLAMMATORY AND ANTIRHEUMATIC PRODUCTS/ IBUPROFEN/ Ibuprofen	29-SEP-2018/ 12:00/ 29-SEP-2018/ 12:00	24/ 24	17/ 17	400	mg	ORAL	ONCE	Migraine
		16	ANTIINFLAMMATORY AND ANTIRHEUMATIC PRODUCTS/ IBUPROFEN/ Ibuprofen	02-OCT-2018/ 07:55/ 02-OCT-2018/ 07:55	27/ 27	20/ 20	400	mg	ORAL	ONCE	Migraine
		17	ANALGESICS/ PARACETAMOL/ Paracetamol	04-OCT-2018/ 12:00/ 16-OCT-2018/ 10:00	29/ 41	22/ 34	1000	mg	ORAL	PRN	Intermittent migraine
		18	ANALGESICS/ RIZATRIPTAN/ Rizatriptan	04-OCT-2018/ 15:00/ 10-OCT-2018/ 09:00	29/ 35	22/ 28	10	mg	ORAL	QD	Intermittent migraine

Notes: Medications were coded to preferred drug name and ATC class using WHO DD version 2015:03
Day relative to inoculum is number of days relative to day of administration of the inoculum.
Day relative to IP is the number of days relative to day of administration of Artesunate, where the day of dose = 1.
Concomitant medications are defined as those medications taken following the administration of inoculum or medication which has continued from prior medications. CM. No. = Concomitant medication number; IP = Artesunate; UN = Unknown; UNK = Unknown.
QD = Daily; BID = Twice per day; PRN = As needed.

Listing 16.2.4.12b
Concomitant Medications

Rando. Number	Challenge Agent	CM. No.	ATC Class/ Preferred Name/ Medication Name Verbatim	Start Date/ Start Time / End Date/ End Time	Start Day/ End Day Relative to Inoculum	Start Day/ End Day Relative to IP	Dose	Unit	Route	Frequency	Indication
R306	3D7	1	ANTIINFLAMMATORY AND ANTIRHEUMATIC PRODUCTS/ IBUPROFEN/ Ibuprofen	14-SEP-2018/ 13:00/ 14-SEP-2018/ 13:00	9/ 9	2/ 2	400	mg	ORAL	ONCE	Headache
		2	ANTIINFLAMMATORY AND ANTIRHEUMATIC PRODUCTS/ IBUPROFEN/ Ibuprofen	16-SEP-2018/ 13:00/ 16-SEP-2018/ 13:00	11/ 11	4/ 4	400	mg	ORAL	ONCE	Intermittent lower back ache
		3	ANTIINFLAMMATORY AND ANTIRHEUMATIC PRODUCTS/ IBUPROFEN/ Ibuprofen	20-SEP-2018/ 17:00/ 20-SEP-2018/ 17:00	15/ 15	8/ 8	400	mg	ORAL	ONCE	Fever, myalgia lower back
R307	3D7	2	ANTIINFLAMMATORY AND ANTIRHEUMATIC PRODUCTS/ IBUPROFEN/ Ibuprofen	13-SEP-2018/ 19:58/ 13-SEP-2018/ 19:58	8/ 8	1/ 1	400	mg	ORAL	ONCE	Fever
		3	ANALGESICS/ PARACETAMOL/ Paracetamol	13-SEP-2018/ 21:53/ 13-SEP-2018/ 21:53	8/ 8	1/ 1	1000	mg	ORAL	ONCE	Fever
		4	ANTIINFLAMMATORY AND ANTIRHEUMATIC PRODUCTS/ IBUPROFEN/ Ibuprofen	14-SEP-2018/ 05:52/ 14-SEP-2018/ 05:52	9/ 9	2/ 2	400	mg	ORAL	ONCE	Fever

Notes: Medications were coded to preferred drug name and ATC class using WHO DD version 2015:03
Day relative to inoculum is number of days relative to day of administration of the inoculum.
Day relative to IP is the number of days relative to day of administration of Artesunate, where the day of dose = 1.
Concomitant medications are defined as those medications taken following the administration of inoculum or medication which has continued from prior medications. CM. No. = Concomitant medication number; IP = Artesunate; UN = Unknown; UNK = Unknown.
QD = Daily; BID = Twice per day; PRN = As needed.

Listing 16.2.4.12b
Concomitant Medications

Rando. Number	Challenge Agent	CM. No.	ATC Class/ Preferred Name/ Medication Name Verbatim	Start Date/ Start Time / End Date/ End Time	Start Day/ End Day Relative to Inoculum	Start Day/ End Day Relative to IP	Dose	Unit	Route	Frequency	Indication
R307	3D7	5	ANTIINFLAMMATORY AND ANTIRHEUMATIC PRODUCTS/ IBUPROFEN/ Ibuprofen	14-SEP-2018/ 13:57/ 14-SEP-2018/ 13:57	9/ 9	2/ 2	400	mg	ORAL	ONCE	Headache, Myalgia
		6	ANALGESICS/ PARACETAMOL/ Paracetamol	14-SEP-2018/ 16:30/ 14-SEP-2018/ 16:30	9/ 9	2/ 2	1000	mg	ORAL	ONCE	Intermittent fever
		7	ANALGESICS/ PARACETAMOL/ Paracetamol	15-SEP-2018/ 05:56/ 15-SEP-2018/ 05:56	10/ 10	3/ 3	1000	mg	ORAL	ONCE	Intermittent fever
		8	ANTIINFLAMMATORY AND ANTIRHEUMATIC PRODUCTS/ IBUPROFEN/ Ibuprofen	20-SEP-2018/ 17:30/ 20-SEP-2018/ 17:30	15/ 15	8/ 8	400	mg	ORAL	ONCE	Fever, muscle ache, headache

Notes: Medications were coded to preferred drug name and ATC class using WHO DD version 2015:03
Day relative to inoculum is number of days relative to day of administration of the inoculum.
Day relative to IP is the number of days relative to day of administration of Artesunate, where the day of dose = 1.
Concomitant medications are defined as those medications taken following the administration of inoculum or medication which has continued from prior medications. CM. No. = Concomitant medication number; IP = Artesunate; UN = Unknown; UNK = Unknown.
QD = Daily; BID = Twice per day; PRN = As needed.

16.2.5 Compliance and drug concentration data

16.2.5.1 Malaria Inoculum Administration

Listing 16.2.5.1
Malaria Inoculum Administration

Randomisation Number	Challenge Agent	Visit	Date Administered	Time Administered	Day Relative to Inoculum	Day Relative to IP	Was Inoculum Administered as Per the Protocol?
R103	P. falciparum isolate K13	Inoculum	14-NOV-2017	10:38	0	-9	Yes
R104	P. falciparum isolate K13	Inoculum	14-NOV-2017	10:42	0	-9	Yes
R105	P. falciparum isolate K13	Inoculum	14-NOV-2017	10:46	0	-9	Yes
R106	P. falciparum isolate 3D7	Inoculum	15-NOV-2017	10:25	0	-8	Yes
R107	P. falciparum isolate 3D7	Inoculum	15-NOV-2017	10:30	0	-8	Yes
R108	P. falciparum isolate 3D7	Inoculum	15-NOV-2017	10:34	0	-8	Yes
R201	P. falciparum isolate K13	Inoculum	19-FEB-2018	10:23	0	-9	Yes
R202	P. falciparum isolate K13	Inoculum	19-FEB-2018	10:27	0	-9	Yes
R203	P. falciparum isolate K13	Inoculum	19-FEB-2018	10:31	0	-9	Yes
R204	P. falciparum isolate K13	Inoculum	19-FEB-2018	10:34	0	-9	Yes
R205	P. falciparum isolate K13	Inoculum	19-FEB-2018	10:37	0	-9	Yes
R206	P. falciparum isolate K13	Inoculum	19-FEB-2018	10:40	0	-9	Yes

Notes: P. falciparum = Plasmodium falciparum.
Day relative to inoculum is the number of days relative to day of administration of the inoculum, where the day of administration = 0.
The inoculum was administered on Study Day 0 for K13-infected subjects and Study Day 1 for 3D7-infected subjects.
Day relative to IP is the number of days relative to day of administration of Artesunate, where the day of dose = 1.

Listing 16.2.5.1
Malaria Inoculum Administration

Randomisation Number	Challenge Agent	Visit	Date Administered	Time Administered	Day Relative to Inoculum	Day Relative to IP	Was Inoculum Administered as Per the Protocol?
R207	P. falciparum isolate K13	Inoculum	19-FEB-2018	10:42	0	-9	Yes
R208	P. falciparum isolate 3D7	Inoculum	20-FEB-2018	10:18	0	-8	Yes
R209	P. falciparum isolate 3D7	Inoculum	20-FEB-2018	10:21	0	-8	Yes
R210	P. falciparum isolate 3D7	Inoculum	20-FEB-2018	10:25	0	-8	Yes
R302	P. falciparum isolate K13	Inoculum	04-SEP-2018	10:26	0	-9	Yes
R303	P. falciparum isolate K13	Inoculum	04-SEP-2018	10:31	0	-9	Yes
R304	P. falciparum isolate K13	Inoculum	04-SEP-2018	10:36	0	-9	Yes
R305	P. falciparum isolate 3D7	Inoculum	05-SEP-2018	11:25	0	-8	Yes
R306	P. falciparum isolate 3D7	Inoculum	05-SEP-2018	11:19	0	-8	Yes
R307	P. falciparum isolate 3D7	Inoculum	05-SEP-2018	11:30	0	-8	Yes

Notes: P. falciparum = Plasmodium falciparum.
Day relative to inoculum is the number of days relative to day of administration of the inoculum, where the day of administration = 0.
The inoculum was administered on Study Day 0 for K13-infected subjects and Study Day 1 for 3D7-infected subjects.
Day relative to IP is the number of days relative to day of administration of Artesunate, where the day of dose = 1.

16.2.5.2 Artesunate Administration

Listing 16.2.5.2
Artesunate Administration

Rando. Number	Challenge Agent	Treatment Name	Lot Number	Dose Amount (Unit)	Route of Administration	Date of Administered	Time of Administered	Day Relative to Inoculum	Day Relative to IP	Was Drug Administered as Per the Protocol?	Was IV Administration of Artesunate required?
R103	K13	Artesunate	AS160501P	100 (mg)	ORAL	23-NOV-2017	10:50	9	1	Yes	No
R104	K13	Artesunate	AS160501P	200 (mg)	ORAL	23-NOV-2017	10:40	9	1	Yes	No
R105	K13	Artesunate	AS160501P	150 (mg)	ORAL	23-NOV-2017	10:30	9	1	Yes	No
R106	3D7	Artesunate	AS160501P	150 (mg)	ORAL	23-NOV-2017	10:20	8	1	Yes	No
R107	3D7	Artesunate	AS160501P	150 (mg)	ORAL	23-NOV-2017	10:10	8	1	Yes	No
R108	3D7	Artesunate	AS160501P	150 (mg)	ORAL	23-NOV-2017	10:01	8	1	Yes	No
R201	K13	Artesunate	AS160501P	150 (mg)	ORAL	28-FEB-2018	13:22	9	1	Yes	No
R202	K13	Artesunate	AS160501P	100 (mg)	ORAL	28-FEB-2018	13:03	9	1	Yes	No
R203	K13	Artesunate	AS160501P	100 (mg)	ORAL	28-FEB-2018	11:40	9	1	Yes	No
R204	K13	Artesunate	AS160501P	150 (mg)	ORAL	28-FEB-2018	13:15	9	1	Yes	No
R205	K13	Artesunate	AS160501P	150 (mg)	ORAL	28-FEB-2018	13:10	9	1	Yes	No
R206	K13	Artesunate	AS160501P	100 (mg)	ORAL	28-FEB-2018	13:26	9	1	Yes	No
R207	K13	Artesunate	AS160501P	150 (mg)	ORAL	28-FEB-2018	12:58	9	1	Yes	No

Notes: Day relative to inoculum is the number of days relative to day of administration of the inoculum, where the day of administration = 0.
The inoculum was administered on Study Day 0 for K13-infected subjects and Study Day 1 for 3D7-infected subjects.
Day relative to IP is the number of days relative to day of administration of Artesunate, where the day of dose = 1.
Rando = Randomisation; IP = Artesunate.

Printed: 24FEB2020 (Final)

Listing 16.2.5.2
Artesunate Administration

Rando. Number	Challenge Agent	Treatment Name	Lot Number	Dose Amount (Unit)	Route of Administration	Date of Administered	Time of Administered	Day Relative to Inoculum	Day Relative to IP	Was Drug Administered as Per the Protocol?	Was IV Administration of Artesunate required?
R208	3D7	Artesunate	AS160501P	150 (mg)	ORAL	28-FEB-2018	11:35	8	1	Yes	No
R209	3D7	Artesunate	AS160501P	150 (mg)	ORAL	28-FEB-2018	15:45	8	1	Yes	No
R210	3D7	Artesunate	AS160501P	100 (mg)	ORAL	28-FEB-2018	11:45	8	1	Yes	No
R302	K13	Artesunate	PPP.18.983	150 (mg)	ORAL	13-SEP-2018	09:05	9	1	Yes	No
R303	K13	Artesunate	PPP.18.983	100 (mg)	ORAL	13-SEP-2018	11:18	9	1	Yes	No
R304	K13	Artesunate	PPP.18.983	100 (mg)	ORAL	13-SEP-2018	09:22	9	1	Yes	No
R305	3D7	Artesunate	PPP.18.983	150 (mg)	ORAL	13-SEP-2018	09:35	8	1	Yes	No
R306	3D7	Artesunate	PPP.18.983	100 (mg)	ORAL	13-SEP-2018	09:45	8	1	Yes	No
R307	3D7	Artesunate	PPP.18.983	150 (mg)	ORAL	13-SEP-2018	09:55	8	1	Yes	No

Notes: Day relative to inoculum is the number of days relative to day of administration of the inoculum, where the day of administration = 0.
The inoculum was administered on Study Day 0 for K13-infected subjects and Study Day 1 for 3D7-infected subjects.
Day relative to IP is the number of days relative to day of administration of Artesunate, where the day of dose = 1.
Rando = Randomisation; IP = Artesunate.

16.2.5.3 Piperaquine Phosphate Administration

Listing 16.2.5.3
Piperaquine Phosphate Administration

Randomisation Number	Treatment Name	Challenge Agent	Lot Number	Dose Amount (Unit)	Route of Administration	Date of Administered	Time of Administered	Day relative to Inoculum	Day Relative to IP
R103	Piperaquine	K13	014536	960 (mg)	ORAL	25-NOV-2017	18:05	11	3
R104	Piperaquine	K13	014536	960 (mg)	ORAL	25-NOV-2017	18:02	11	3
R105	Piperaquine	K13	014536	960 (mg)	ORAL	25-NOV-2017	17:59	11	3
R106	Piperaquine	3D7	014536	960 (mg)	ORAL	01-DEC-2017	08:40	16	9
R107	Piperaquine	3D7	014536	960 (mg)	ORAL	04-DEC-2017	UN:UN	19	12
R108	Piperaquine	3D7	014536	960 (mg)	ORAL	01-DEC-2017	07:34	16	9
R201	Piperaquine	K13	014536	960 (mg)	ORAL	02-MAR-2018	13:45	11	3
R202	Piperaquine	K13	014536	960 (mg)	ORAL	02-MAR-2018	13:58	11	3
R203	Piperaquine	K13	014536	960 (mg)	ORAL	02-MAR-2018	13:35	11	3
R204	Piperaquine	K13	014536	960 (mg)	ORAL	02-MAR-2018	13:52	11	3
R205	Piperaquine	K13	014536	960 (mg)	ORAL	02-MAR-2018	13:48	11	3
R206	Piperaquine	K13	014536	960 (mg)	ORAL	02-MAR-2018	13:40	11	3
R207	Piperaquine	K13	014536	960 (mg)	ORAL	02-MAR-2018	13:58	11	3

Notes: Day relative to inoculum is the number of days relative to day of administration of the inoculum, where the day of administration = 0.
The inoculum was administered on Study Day 0 for K13-infected subjects and Study Day 1 for 3D7-infected subjects.
Day relative to IP is the number of days relative to day of administration of Artesunate, where the day of dose = 1.
UN = Unknown.

Listing 16.2.5.3
Piperaquine Phosphate Administration

Randomisation Number	Treatment Name	Challenge Agent	Lot Number	Dose Amount (Unit)	Route of Administration	Date of Administered	Time of Administered	Day relative to Inoculum	Day Relative to IP
R208	Piperaquine	3D7	014536	960 (mg)	ORAL	10-MAR-2018	08:03	18	11
R209	Piperaquine	3D7	014536	960 (mg)	ORAL	08-MAR-2018	08:49	16	9
R210	Piperaquine	3D7	014536	960 (mg)	ORAL	08-MAR-2018	07:51	16	9
R302	Piperaquine	K13	014536	960 (mg)	ORAL	15-SEP-2018	09:10	11	3
R303	Piperaquine	K13	014536	960 (mg)	ORAL	15-SEP-2018	11:25	11	3
R304	Piperaquine	K13	014536	960 (mg)	ORAL	15-SEP-2018	09:33	11	3
R305	Piperaquine	3D7	014536	960 (mg)	ORAL	19-SEP-2018	UN:UN	14	7
R306	Piperaquine	3D7	014536	960 (mg)	ORAL	19-SEP-2018	UN:UN	14	7
R307	Piperaquine	3D7	014536	960 (mg)	ORAL	19-SEP-2018	UN:UN	14	7

Notes: Day relative to inoculum is the number of days relative to day of administration of the inoculum, where the day of administration = 0.
The inoculum was administered on Study Day 0 for K13-infected subjects and Study Day 1 for 3D7-infected subjects.
Day relative to IP is the number of days relative to day of administration of Artesunate, where the day of dose = 1.
UN = Unknown.

Printed: 24FEB2020 (Final)

16.2.5.4 Rescue Medications (Malarone® and Primacin™) Administration

Listing 16.2.5.4
Rescue Medications (Malarone® and Primacin™) Administration

Randomisation Number	Treatment Name	Challenge Agent	Dose Amount (Unit)	Route of Administration	Date of Administered	Time of Administered	Day relative to Inoculum	Day Relative to IP	Was Drug Administered as Per the Protocol?
R103	Malarone	K13	4 (Tablet)	ORAL	04-DEC-2017	09:35	20	12	Yes
		K13	4 (Tablet)	ORAL	05-DEC-2017	07:25	21	13	Yes
		K13	4 (Tablet)	ORAL	06-DEC-2017	08:10	22	14	Yes
	Primacin	K13	45 (mg)	ORAL	12-DEC-2017	09:37	28	20	Yes
R104	Malarone	K13	4 (Tablet)	ORAL	04-DEC-2017	12:52	20	12	Yes
		K13	4 (Tablet)	ORAL	05-DEC-2017	12:00	21	13	Yes
		K13	4 (Tablet)	ORAL	06-DEC-2017	12:30	22	14	Yes
	Primacin	K13	45 (mg)	ORAL	12-DEC-2017	08:05	28	20	Yes
R105	Malarone	K13	4 (Tablet)	ORAL	30-NOV-2017	07:35	16	8	Yes
		K13	4 (Tablet)	ORAL	01-DEC-2017	05:15	17	9	Yes
		K13	4 (Tablet)	ORAL	02-DEC-2017	07:05	18	10	Yes
	Primacin	K13	45 (mg)	ORAL	12-DEC-2017	07:30	28	20	Yes
R106	Malarone	3D7	4 (Tablet)	ORAL	11-DEC-2017	08:12	26	19	Yes
		3D7	4 (Tablet)	ORAL	12-DEC-2017	07:50	27	20	Yes
		3D7	4 (Tablet)	ORAL	13-DEC-2017	08:05	28	21	Yes
	Primacin	3D7	45 (mg)	ORAL	19-DEC-2017	08:59	34	27	Yes
R107	Malarone	3D7	4 (Tablet)	ORAL	11-DEC-2017	09:56	26	19	Yes
		3D7	4 (Tablet)	ORAL	12-DEC-2017	10:01	27	20	Yes
		3D7	4 (Tablet)	ORAL	13-DEC-2017	10:00	28	21	Yes
	Primacin	3D7	45 (mg)	ORAL	15-DEC-2017	09:41	30	23	Yes

Notes: Day relative to inoculum is the number of days relative to day of administration of the inoculum, where the day of administration = 0.
The inoculum was administered on Study Day 0 for K13-infected subjects and Study Day 1 for 3D7-infected subjects.
Day relative to IP is the number of days relative to day of administration of Artesunate, where the day of dose = 1.
Primacin 45 mg is 6 tablets of Primacin containing a total of 45 mg of the active ingredient primaquine.
UN = Unknown.

Listing 16.2.5.4
Rescue Medications (Malarone® and Primacin™) Administration

Randomisation Number	Treatment Name	Challenge Agent	Dose Amount (Unit)	Route of Administration	Date of Administered	Time of Administered	Day relative to Inoculum	Day Relative to IP	Was Drug Administered as Per the Protocol?
R108	Malarone	3D7	4 (Tablet)	ORAL	11-DEC-2017	09:00	26	19	Yes
		3D7	4 (Tablet)	ORAL	12-DEC-2017	08:20	27	20	Yes
		3D7	4 (Tablet)	ORAL	13-DEC-2017	08:30	28	21	Yes
	Primacin	3D7	45 (mg)	ORAL	19-DEC-2017	07:45	34	27	Yes
R201	Malarone	K13	4 (Tablet)	ORAL	19-MAR-2018	08:25	28	20	Yes
		K13	4 (Tablet)	ORAL	20-MAR-2018	08:09	29	21	Yes
		K13	4 (Tablet)	ORAL	21-MAR-2018	08:18	30	22	Yes
	Primacin	K13	45 (mg)	ORAL	14-MAR-2018	09:13	23	15	Yes
R202	Malarone	K13	4 (Tablet)	ORAL	12-MAR-2018	10:02	21	13	Yes
		K13	4 (Tablet)	ORAL	13-MAR-2018	10:02	22	14	Yes
		K13	4 (Tablet)	ORAL	14-MAR-2018	09:07	23	15	Yes
	Primacin	K13	45 (mg)	ORAL	14-MAR-2018	09:00	23	15	Yes
R203	Malarone	K13	4 (Tablet)	ORAL	10-MAR-2018	08:53	19	11	Yes
		K13	4 (Tablet)	ORAL	11-MAR-2018	08:50	20	12	Yes
		K13	4 (Tablet)	ORAL	12-MAR-2018	08:30	21	13	Yes
	Primacin	K13	45 (mg)	ORAL	14-MAR-2018	10:23	23	15	Yes
R204	Malarone	K13	4 (Tablet)	ORAL	19-MAR-2018	08:37	28	20	Yes
		K13	4 (Tablet)	ORAL	20-MAR-2018	08:53	29	21	Yes
		K13	4 (Tablet)	ORAL	21-MAR-2018	08:15	30	22	Yes
	Primacin	K13	45 (mg)	ORAL	14-MAR-2018	10:08	23	15	Yes

Notes: Day relative to inoculum is the number of days relative to day of administration of the inoculum, where the day of administration = 0.
The inoculum was administered on Study Day 0 for K13-infected subjects and Study Day 1 for 3D7-infected subjects.
Day relative to IP is the number of days relative to day of administration of Artesunate, where the day of dose = 1.
Primacin 45 mg is 6 tablets of Primacin containing a total of 45 mg of the active ingredient primaquine.
UN = Unknown.

Listing 16.2.5.4
Rescue Medications (Malarone® and Primacin™) Administration

Randomisation Number	Treatment Name	Challenge Agent	Dose Amount (Unit)	Route of Administration	Date of Administered	Time of Administered	Day relative to Inoculum	Day Relative to IP	Was Drug Administered as Per the Protocol?
R205	Malarone	K13	4 (Tablet)	ORAL	19-MAR-2018	07:39	28	20	Yes
		K13	4 (Tablet)	ORAL	20-MAR-2018	UN:UN	29	21	Yes
		K13	4 (Tablet)	ORAL	21-MAR-2018	UN:UN	30	22	Yes
	Primacin	K13	45 (mg)	ORAL	14-MAR-2018	08:57	23	15	Yes
R206	Malarone	K13	4 (Tablet)	ORAL	19-MAR-2018	09:51	28	20	Yes
		K13	4 (Tablet)	ORAL	20-MAR-2018	14:00	29	21	Yes
		K13	4 (Tablet)	ORAL	21-MAR-2018	14:00	30	22	Yes
	Primacin	K13	45 (mg)	ORAL	14-MAR-2018	10:24	23	15	Yes
R207	Malarone	K13	4 (Tablet)	ORAL	19-MAR-2018	09:22	28	20	Yes
		K13	4 (Tablet)	ORAL	20-MAR-2018	09:30	29	21	Yes
		K13	4 (Tablet)	ORAL	21-MAR-2018	09:45	30	22	Yes
	Primacin	K13	45 (mg)	ORAL	14-MAR-2018	09:19	23	15	Yes
R208	Malarone	3D7	4 (Tablet)	ORAL	19-MAR-2018	07:08	27	20	Yes
		3D7	4 (Tablet)	ORAL	20-MAR-2018	07:07	28	21	Yes
		3D7	4 (Tablet)	ORAL	21-MAR-2018	UN:UN	29	22	Yes
	Primacin	3D7	45 (mg)	ORAL	14-MAR-2018	07:23	22	15	Yes
R209	Malarone	3D7	4 (Tablet)	ORAL	19-MAR-2018	07:57	27	20	Yes
		3D7	4 (Tablet)	ORAL	20-MAR-2018	06:45	28	21	Yes
		3D7	4 (Tablet)	ORAL	21-MAR-2018	06:40	29	22	Yes
	Primacin	3D7	45 (mg)	ORAL	14-MAR-2018	07:44	22	15	Yes

Notes: Day relative to inoculum is the number of days relative to day of administration of the inoculum, where the day of administration = 0.
The inoculum was administered on Study Day 0 for K13-infected subjects and Study Day 1 for 3D7-infected subjects.
Day relative to IP is the number of days relative to day of administration of Artesunate, where the day of dose = 1.
Primacin 45 mg is 6 tablets of Primacin containing a total of 45 mg of the active ingredient primaquine.
UN = Unknown.

Listing 16.2.5.4
Rescue Medications (Malarone® and Primacin™) Administration

Randomisation Number	Treatment Name	Challenge Agent	Dose Amount (Unit)	Route of Administration	Date of Administered	Time of Administered	Day relative to Inoculum	Day Relative to IP	Was Drug Administered as Per the Protocol?
R210	Malarone	3D7	4 (Tablet)	ORAL	19-MAR-2018	07:49	27	20	Yes
		3D7	4 (Tablet)	ORAL	20-MAR-2018	07:20	28	21	Yes
		3D7	4 (Tablet)	ORAL	21-MAR-2018	07:20	29	22	Yes
	Primacin	3D7	45 (mg)	ORAL	14-MAR-2018	07:32	22	15	Yes
R302	Malarone	K13	4 (Tablet)	ORAL	25-SEP-2018	08:47	21	13	Yes
		K13	4 (Tablet)	ORAL	26-SEP-2018	09:00	22	14	Yes
		K13	4 (Tablet)	ORAL	27-SEP-2018	08:30	23	15	Yes
	Primacin	K13	45 (mg)	ORAL	28-SEP-2018	08:34	24	16	Yes
R303	Malarone	K13	4 (Tablet)	ORAL	25-SEP-2018	08:30	21	13	Yes
		K13	4 (Tablet)	ORAL	26-SEP-2018	09:00	22	14	Yes
		K13	4 (Tablet)	ORAL	27-SEP-2018	09:00	23	15	Yes
	Primacin	K13	45 (mg)	ORAL	28-SEP-2018	08:28	24	16	Yes
R304	Malarone	K13	4 (Tablet)	ORAL	25-SEP-2018	08:43	21	13	Yes
		K13	4 (Tablet)	ORAL	26-SEP-2018	08:00	22	14	Yes
		K13	4 (Tablet)	ORAL	27-SEP-2018	09:00	23	15	Yes
	Primacin	K13	45 (mg)	ORAL	28-SEP-2018	08:43	24	16	Yes
R305	Malarone	3D7	4 (Tablet)	ORAL	25-SEP-2018	08:37	20	13	Yes
		3D7	4 (Tablet)	ORAL	26-SEP-2018	10:00	21	14	Yes
		3D7	4 (Tablet)	ORAL	27-SEP-2018	10:00	22	15	Yes
	Primacin	3D7	45 (mg)	ORAL	28-SEP-2018	07:30	23	16	Yes

Notes: Day relative to inoculum is the number of days relative to day of administration of the inoculum, where the day of administration = 0.
The inoculum was administered on Study Day 0 for K13-infected subjects and Study Day 1 for 3D7-infected subjects.
Day relative to IP is the number of days relative to day of administration of Artesunate, where the day of dose = 1.
Primacin 45 mg is 6 tablets of Primacin containing a total of 45 mg of the active ingredient primaquine.
UN = Unknown.

Listing 16.2.5.4
Rescue Medications (Malarone® and Primacin™) Administration

Randomisation Number	Treatment Name	Challenge Agent	Dose Amount (Unit)	Route of Administration	Date of Administered	Time of Administered	Day relative to Inoculum	Day Relative to IP	Was Drug Administered as Per the Protocol?
R306	Malarone	3D7	4 (Tablet)	ORAL	25-SEP-2018	07:47	20	13	Yes
		3D7	4 (Tablet)	ORAL	26-SEP-2018	07:50	21	14	Yes
		3D7	4 (Tablet)	ORAL	27-SEP-2018	08:30	22	15	Yes
	Primacin	3D7	45 (mg)	ORAL	28-SEP-2018	07:17	23	16	Yes
R307	Malarone	3D7	4 (Tablet)	ORAL	25-SEP-2018	07:51	20	13	Yes
		3D7	4 (Tablet)	ORAL	26-SEP-2018	07:45	21	14	Yes
		3D7	4 (Tablet)	ORAL	27-SEP-2018	08:30	22	15	Yes
	Primacin	3D7	45 (mg)	ORAL	28-SEP-2018	07:44	23	16	Yes

Notes: Day relative to inoculum is the number of days relative to day of administration of the inoculum, where the day of administration = 0.
The inoculum was administered on Study Day 0 for K13-infected subjects and Study Day 1 for 3D7-infected subjects.
Day relative to IP is the number of days relative to day of administration of Artesunate, where the day of dose = 1.
Primacin 45 mg is 6 tablets of Primacin containing a total of 45 mg of the active ingredient primaquine.
UN = Unknown.

16.2.5.5 Plasma Pharmacokinetic Sample Collection Time and Concentration For Artesunate

Listing 16.2.5.5
Plasma Pharmacokinetic Sample Collection Time And Concentration For Artesunate

Randomisation Number	Challenge Agent	Scheduled Timepoint	Collection Date	Collection Time	Day Relative to Inoculum	Day Relative to IP	Actual Time Post Artesunate Dosing (hours)	Plasma Concentration (ug/L)
R103	K13	Pre-dose	23-NOV-2017	09:50	9	1	-1.00	0.00
		0.25 Hrs Post-dose	23-NOV-2017	11:05	9	1	0.25	21.89
		0.5 Hrs Post-dose	23-NOV-2017	11:20	9	1	0.50	37.36
		1 Hrs Post-dose	23-NOV-2017	11:50	9	1	1.00	74.30
		1.5 Hrs Post-dose	23-NOV-2017	12:20	9	1	1.50	35.80
		2 Hrs Post-dose	23-NOV-2017	12:50	9	1	2.00	32.87
		2.5 Hrs Post-dose	23-NOV-2017	13:20	9	1	2.50	15.87
		3 Hrs Post-dose	23-NOV-2017	13:50	9	1	3.00	23.74
		4 Hrs Post-dose	23-NOV-2017	14:50	9	1	4.00	16.96
		6 Hrs Post-dose	23-NOV-2017	16:56	9	1	6.10	0.00
		8 Hrs Post-dose	23-NOV-2017	18:50	9	1	8.00	0.00
		10 Hrs Post-dose	23-NOV-2017	20:50	9	1	10.00	0.00
		12 Hrs Post-dose	23-NOV-2017	22:50	9	1	12.00	0.00

Notes: Day relative to inoculum is the number of days relative to day of administration of the inoculum, where the day of administration = 0.
The inoculum was administered on Study Day 0 for K13-infected subjects and Study Day 1 for 3D7-infected subjects.
Day relative to IP is the number of days relative to day of administration of Artesunate, where the day of dose = 1.
Pre-dose = Pre Artesunate dose

Listing 16.2.5.5
Plasma Pharmacokinetic Sample Collection Time And Concentration For Artesunate

Randomisation Number	Challenge Agent	Scheduled Timepoint	Collection Date	Collection Time	Day Relative to Inoculum	Day Relative to IP	Actual Time Post Artesunate Dosing (hours)	Plasma Concentration (ug/L)
R104	K13	Pre-dose	23-NOV-2017	09:44	9	1	-0.93	0.00
		0.25 Hrs Post-dose	23-NOV-2017	10:55	9	1	0.25	89.69
		0.5 Hrs Post-dose	23-NOV-2017	11:10	9	1	0.50	157.69
		1 Hrs Post-dose	23-NOV-2017	11:40	9	1	1.00	133.77
		1.5 Hrs Post-dose	23-NOV-2017	12:10	9	1	1.50	110.60
		2 Hrs Post-dose	23-NOV-2017	12:40	9	1	2.00	53.79
		2.5 Hrs Post-dose	23-NOV-2017	13:10	9	1	2.50	22.41
		3 Hrs Post-dose	23-NOV-2017	13:40	9	1	3.00	14.83
		4 Hrs Post-dose	23-NOV-2017	14:40	9	1	4.00	9.20
		6 Hrs Post-dose	23-NOV-2017	16:41	9	1	6.02	0.70
		8 Hrs Post-dose	23-NOV-2017	18:40	9	1	8.00	0.50
		10 Hrs Post-dose	23-NOV-2017	20:40	9	1	10.00	0.00
		12 Hrs Post-dose	23-NOV-2017	22:40	9	1	12.00	0.00

Notes: Day relative to inoculum is the number of days relative to day of administration of the inoculum, where the day of administration = 0.
The inoculum was administered on Study Day 0 for K13-infected subjects and Study Day 1 for 3D7-infected subjects.
Day relative to IP is the number of days relative to day of administration of Artesunate, where the day of dose = 1.
Pre-dose = Pre Artesunate dose

Listing 16.2.5.5
Plasma Pharmacokinetic Sample Collection Time And Concentration For Artesunate

Randomisation Number	Challenge Agent	Scheduled Timepoint	Collection Date	Collection Time	Day Relative to Inoculum	Day Relative to IP	Actual Time Post Artesunate Dosing (hours)	Plasma Concentration (ug/L)
R105	K13	Pre-dose	23-NOV-2017	09:36	9	1	-0.90	0.00
		0.25 Hrs Post-dose	23-NOV-2017	10:45	9	1	0.25	7.92
		0.5 Hrs Post-dose	23-NOV-2017	11:00	9	1	0.50	122.45
		1 Hrs Post-dose	23-NOV-2017	11:30	9	1	1.00	56.35
		1.5 Hrs Post-dose	23-NOV-2017	12:00	9	1	1.50	46.27
		2 Hrs Post-dose	23-NOV-2017	12:31	9	1	2.02	22.54
		2.5 Hrs Post-dose	23-NOV-2017	13:00	9	1	2.50	0.31
		3 Hrs Post-dose	23-NOV-2017	13:30	9	1	3.00	0.00
		4 Hrs Post-dose	23-NOV-2017	14:30	9	1	4.00	0.00
		6 Hrs Post-dose	23-NOV-2017	16:31	9	1	6.02	0.00
		8 Hrs Post-dose	23-NOV-2017	18:30	9	1	8.00	0.00
		10 Hrs Post-dose	23-NOV-2017	20:30	9	1	10.00	0.00
		12 Hrs Post-dose	23-NOV-2017	22:31	9	1	12.02	0.00

Notes: Day relative to inoculum is the number of days relative to day of administration of the inoculum, where the day of administration = 0.
The inoculum was administered on Study Day 0 for K13-infected subjects and Study Day 1 for 3D7-infected subjects.
Day relative to IP is the number of days relative to day of administration of Artesunate, where the day of dose = 1.
Pre-dose = Pre Artesunate dose

Printed: 24FEB2020 (Final)

Listing 16.2.5.5
Plasma Pharmacokinetic Sample Collection Time And Concentration For Artesunate

Randomisation Number	Challenge Agent	Scheduled Timepoint	Collection Date	Collection Time	Day Relative to Inoculum	Day Relative to IP	Actual Time Post Artesunate Dosing (hours)	Plasma Concentration (ug/L)
R106	3D7	Pre-dose	23-NOV-2017	09:33	8	1	-0.78	0.00
		0.25 Hrs Post-dose	23-NOV-2017	10:35	8	1	0.25	46.03
		0.5 Hrs Post-dose	23-NOV-2017	10:50	8	1	0.50	65.09
		1 Hrs Post-dose	23-NOV-2017	11:20	8	1	1.00	107.61
		1.5 Hrs Post-dose	23-NOV-2017	11:50	8	1	1.50	40.89
		2 Hrs Post-dose	23-NOV-2017	12:20	8	1	2.00	171.92
		2.5 Hrs Post-dose	23-NOV-2017	12:50	8	1	2.50	47.68
		3 Hrs Post-dose	23-NOV-2017	13:20	8	1	3.00	20.55
		4 Hrs Post-dose	23-NOV-2017	14:20	8	1	4.00	19.45
		6 Hrs Post-dose	23-NOV-2017	16:22	8	1	6.03	0.00
		8 Hrs Post-dose	23-NOV-2017	18:20	8	1	8.00	0.00
		10 Hrs Post-dose	23-NOV-2017	20:20	8	1	10.00	0.00
		12 Hrs Post-dose	23-NOV-2017	22:32	8	1	12.20	0.00

Notes: Day relative to inoculum is the number of days relative to day of administration of the inoculum, where the day of administration = 0.
The inoculum was administered on Study Day 0 for K13-infected subjects and Study Day 1 for 3D7-infected subjects.
Day relative to IP is the number of days relative to day of administration of Artesunate, where the day of dose = 1.
Pre-dose = Pre Artesunate dose

Printed: 24FEB2020 (Final)

Listing 16.2.5.5
Plasma Pharmacokinetic Sample Collection Time And Concentration For Artesunate

Randomisation Number	Challenge Agent	Scheduled Timepoint	Collection Date	Collection Time	Day Relative to Inoculum	Day Relative to IP	Actual Time Post Artesunate Dosing (hours)	Plasma Concentration (ug/L)
R107	3D7	Pre-dose	23-NOV-2017	09:35	8	1	-0.58	0.00
		0.25 Hrs Post-dose	23-NOV-2017	10:25	8	1	0.25	116.14
		0.5 Hrs Post-dose	23-NOV-2017	10:40	8	1	0.50	378.57
		1 Hrs Post-dose	23-NOV-2017	11:10	8	1	1.00	6.50
		1.5 Hrs Post-dose	23-NOV-2017	11:40	8	1	1.50	2.20
		2 Hrs Post-dose	23-NOV-2017	12:10	8	1	2.00	0.00
		2.5 Hrs Post-dose	23-NOV-2017	12:40	8	1	2.50	0.00
		3 Hrs Post-dose	23-NOV-2017	13:11	8	1	3.02	0.00
		4 Hrs Post-dose	23-NOV-2017	14:10	8	1	4.00	0.00
		6 Hrs Post-dose	23-NOV-2017	16:10	8	1	6.00	0.00
		8 Hrs Post-dose	23-NOV-2017	18:10	8	1	8.00	0.00
		10 Hrs Post-dose	23-NOV-2017	20:10	8	1	10.00	0.00
		12 Hrs Post-dose	23-NOV-2017	22:10	8	1	12.00	0.00

Notes: Day relative to inoculum is the number of days relative to day of administration of the inoculum, where the day of administration = 0.
The inoculum was administered on Study Day 0 for K13-infected subjects and Study Day 1 for 3D7-infected subjects.
Day relative to IP is the number of days relative to day of administration of Artesunate, where the day of dose = 1.
Pre-dose = Pre Artesunate dose

Listing 16.2.5.5
Plasma Pharmacokinetic Sample Collection Time And Concentration For Artesunate

Randomisation Number	Challenge Agent	Scheduled Timepoint	Collection Date	Collection Time	Day Relative to Inoculum	Day Relative to IP	Actual Time Post Artesunate Dosing (hours)	Plasma Concentration (ug/L)
R108	3D7	Pre-dose	23-NOV-2017	09:17	8	1	-0.73	0.00
		0.25 Hrs Post-dose	23-NOV-2017	10:16	8	1	0.25	21.81
		0.5 Hrs Post-dose	23-NOV-2017	10:31	8	1	0.50	65.41
		1 Hrs Post-dose	23-NOV-2017	11:01	8	1	1.00	195.67
		1.5 Hrs Post-dose	23-NOV-2017	11:31	8	1	1.50	38.15
		2 Hrs Post-dose	23-NOV-2017	12:05	8	1	2.07	28.96
		2.5 Hrs Post-dose	23-NOV-2017	12:31	8	1	2.50	10.23
		3 Hrs Post-dose	23-NOV-2017	13:03	8	1	3.03	0.65
		4 Hrs Post-dose	23-NOV-2017	14:01	8	1	4.00	0.10
		6 Hrs Post-dose	23-NOV-2017	16:12	8	1	6.18	0.00
		8 Hrs Post-dose	23-NOV-2017	18:02	8	1	8.02	0.00
		10 Hrs Post-dose	23-NOV-2017	20:01	8	1	10.00	0.00
		12 Hrs Post-dose	23-NOV-2017	22:02	8	1	12.02	0.00

Notes: Day relative to inoculum is the number of days relative to day of administration of the inoculum, where the day of administration = 0.
The inoculum was administered on Study Day 0 for K13-infected subjects and Study Day 1 for 3D7-infected subjects.
Day relative to IP is the number of days relative to day of administration of Artesunate, where the day of dose = 1.
Pre-dose = Pre Artesunate dose

Printed: 24FEB2020 (Final)

Listing 16.2.5.5
Plasma Pharmacokinetic Sample Collection Time And Concentration For Artesunate

Randomisation Number	Challenge Agent	Scheduled Timepoint	Collection Date	Collection Time	Day Relative to Inoculum	Day Relative to IP	Actual Time Post Artesunate Dosing (hours)	Plasma Concentration (ug/L)
R201	K13	Pre-dose	28-FEB-2018	12:39	9	1	-0.72	0.00
		0.25 Hrs Post-dose	28-FEB-2018	13:37	9	1	0.25	44.80
		0.5 Hrs Post-dose	28-FEB-2018	13:52	9	1	0.50	45.71
		1 Hrs Post-dose	28-FEB-2018	14:22	9	1	1.00	38.24
		1.5 Hrs Post-dose	28-FEB-2018	14:52	9	1	1.50	373.93
		2 Hrs Post-dose	28-FEB-2018	15:23	9	1	2.02	3.28
		2.5 Hrs Post-dose	28-FEB-2018	15:52	9	1	2.50	1.36
		3 Hrs Post-dose	28-FEB-2018	16:22	9	1	3.00	0.00
		4 Hrs Post-dose	28-FEB-2018	17:22	9	1	4.00	0.00
		6 Hrs Post-dose	28-FEB-2018	19:22	9	1	6.00	0.00
		8 Hrs Post-dose	28-FEB-2018	21:22	9	1	8.00	0.00
		10 Hrs Post-dose	28-FEB-2018	23:28	9	1	10.10	0.00
		12 Hrs Post-dose	01-MAR-2018	01:28	10	2	12.10	0.00

Notes: Day relative to inoculum is the number of days relative to day of administration of the inoculum, where the day of administration = 0.
The inoculum was administered on Study Day 0 for K13-infected subjects and Study Day 1 for 3D7-infected subjects.
Day relative to IP is the number of days relative to day of administration of Artesunate, where the day of dose = 1.
Pre-dose = Pre Artesunate dose

Printed: 24FEB2020 (Final)

Listing 16.2.5.5
Plasma Pharmacokinetic Sample Collection Time And Concentration For Artesunate

Randomisation Number	Challenge Agent	Scheduled Timepoint	Collection Date	Collection Time	Day Relative to Inoculum	Day Relative to IP	Actual Time Post Artesunate Dosing (hours)	Plasma Concentration (ug/L)
R202	K13	Pre-dose	28-FEB-2018	12:05	9	1	-0.97	0.00
		0.25 Hrs Post-dose	28-FEB-2018	13:18	9	1	0.25	265.67
		0.5 Hrs Post-dose	28-FEB-2018	13:33	9	1	0.50	92.92
		1 Hrs Post-dose	28-FEB-2018	14:03	9	1	1.00	33.09
		1.5 Hrs Post-dose	28-FEB-2018	14:33	9	1	1.50	5.23
		2 Hrs Post-dose	28-FEB-2018	15:04	9	1	2.02	1.03
		2.5 Hrs Post-dose	28-FEB-2018	15:33	9	1	2.50	1.10
		3 Hrs Post-dose	28-FEB-2018	16:03	9	1	3.00	0.00
		4 Hrs Post-dose	28-FEB-2018	17:03	9	1	4.00	0.00
		6 Hrs Post-dose	28-FEB-2018	19:03	9	1	6.00	0.00
		8 Hrs Post-dose	28-FEB-2018	21:03	9	1	8.00	0.00
		10 Hrs Post-dose	28-FEB-2018	23:03	9	1	10.00	0.00
		12 Hrs Post-dose	01-MAR-2018	01:03	10	2	12.00	0.00

Notes: Day relative to inoculum is the number of days relative to day of administration of the inoculum, where the day of administration = 0.
The inoculum was administered on Study Day 0 for K13-infected subjects and Study Day 1 for 3D7-infected subjects.
Day relative to IP is the number of days relative to day of administration of Artesunate, where the day of dose = 1.
Pre-dose = Pre Artesunate dose

Listing 16.2.5.5
Plasma Pharmacokinetic Sample Collection Time And Concentration For Artesunate

Randomisation Number	Challenge Agent	Scheduled Timepoint	Collection Date	Collection Time	Day Relative to Inoculum	Day Relative to IP	Actual Time Post Artesunate Dosing (hours)	Plasma Concentration (ug/L)
R203	K13	Pre-dose	28-FEB-2018	11:14	9	1	-0.43	0.00
		0.25 Hrs Post-dose	28-FEB-2018	11:55	9	1	0.25	72.75
		0.5 Hrs Post-dose	28-FEB-2018	12:10	9	1	0.50	57.26
		1 Hrs Post-dose	28-FEB-2018	12:40	9	1	1.00	2.20
		1.5 Hrs Post-dose	28-FEB-2018	13:10	9	1	1.50	0.00
		2 Hrs Post-dose	28-FEB-2018	13:40	9	1	2.00	0.00
		2.5 Hrs Post-dose	28-FEB-2018	14:10	9	1	2.50	0.00
		3 Hrs Post-dose	28-FEB-2018	14:40	9	1	3.00	0.00
		4 Hrs Post-dose	28-FEB-2018	15:40	9	1	4.00	0.00
		6 Hrs Post-dose	28-FEB-2018	17:41	9	1	6.02	0.00
		8 Hrs Post-dose	28-FEB-2018	19:40	9	1	8.00	0.00
		10 Hrs Post-dose	28-FEB-2018	21:44	9	1	10.07	0.00
		12 Hrs Post-dose	28-FEB-2018	23:40	9	1	12.00	0.00

Notes: Day relative to inoculum is the number of days relative to day of administration of the inoculum, where the day of administration = 0.
The inoculum was administered on Study Day 0 for K13-infected subjects and Study Day 1 for 3D7-infected subjects.
Day relative to IP is the number of days relative to day of administration of Artesunate, where the day of dose = 1.
Pre-dose = Pre Artesunate dose

Listing 16.2.5.5
Plasma Pharmacokinetic Sample Collection Time And Concentration For Artesunate

Randomisation Number	Challenge Agent	Scheduled Timepoint	Collection Date	Collection Time	Day Relative to Inoculum	Day Relative to IP	Actual Time Post Artesunate Dosing (hours)	Plasma Concentration (ug/L)
R204	K13	Pre-dose	28-FEB-2018	12:29	9	1	-0.77	0.00
		0.25 Hrs Post-dose	28-FEB-2018	13:30	9	1	0.25	108.60
		0.5 Hrs Post-dose	28-FEB-2018	13:45	9	1	0.50	425.91
		1 Hrs Post-dose	28-FEB-2018	14:15	9	1	1.00	32.48
		1.5 Hrs Post-dose	28-FEB-2018	14:45	9	1	1.50	12.41
		2 Hrs Post-dose	28-FEB-2018	15:15	9	1	2.00	5.01
		2.5 Hrs Post-dose	28-FEB-2018	15:45	9	1	2.50	1.68
		3 Hrs Post-dose	28-FEB-2018	16:15	9	1	3.00	0.44
		4 Hrs Post-dose	28-FEB-2018	17:15	9	1	4.00	0.31
		6 Hrs Post-dose	28-FEB-2018	19:15	9	1	6.00	0.00
		8 Hrs Post-dose	28-FEB-2018	21:17	9	1	8.03	0.00
		10 Hrs Post-dose	28-FEB-2018	23:15	9	1	10.00	0.00
		12 Hrs Post-dose	01-MAR-2018	01:15	10	2	12.00	0.00

Notes: Day relative to inoculum is the number of days relative to day of administration of the inoculum, where the day of administration = 0.
The inoculum was administered on Study Day 0 for K13-infected subjects and Study Day 1 for 3D7-infected subjects.
Day relative to IP is the number of days relative to day of administration of Artesunate, where the day of dose = 1.
Pre-dose = Pre Artesunate dose

Listing 16.2.5.5
Plasma Pharmacokinetic Sample Collection Time And Concentration For Artesunate

Randomisation Number	Challenge Agent	Scheduled Timepoint	Collection Date	Collection Time	Day Relative to Inoculum	Day Relative to IP	Actual Time Post Artesunate Dosing (hours)	Plasma Concentration (ug/L)
R205	K13	Pre-dose	28-FEB-2018	12:19	9	1	-0.85	0.00
		0.25 Hrs Post-dose	28-FEB-2018	13:25	9	1	0.25	1.97
		0.5 Hrs Post-dose	28-FEB-2018	13:40	9	1	0.50	24.12
		1 Hrs Post-dose	28-FEB-2018	14:10	9	1	1.00	138.35
		1.5 Hrs Post-dose	28-FEB-2018	14:40	9	1	1.50	23.12
		2 Hrs Post-dose	28-FEB-2018	15:11	9	1	2.02	5.64
		2.5 Hrs Post-dose	28-FEB-2018	15:40	9	1	2.50	1.51
		3 Hrs Post-dose	28-FEB-2018	16:10	9	1	3.00	1.17
		4 Hrs Post-dose	28-FEB-2018	17:10	9	1	4.00	0.48
		6 Hrs Post-dose	28-FEB-2018	19:11	9	1	6.02	0.00
		8 Hrs Post-dose	28-FEB-2018	21:10	9	1	8.00	0.00
		10 Hrs Post-dose	28-FEB-2018	23:10	9	1	10.00	0.00
		12 Hrs Post-dose	01-MAR-2018	01:10	10	2	12.00	0.00

Notes: Day relative to inoculum is the number of days relative to day of administration of the inoculum, where the day of administration = 0.
The inoculum was administered on Study Day 0 for K13-infected subjects and Study Day 1 for 3D7-infected subjects.
Day relative to IP is the number of days relative to day of administration of Artesunate, where the day of dose = 1.
Pre-dose = Pre Artesunate dose

Listing 16.2.5.5
Plasma Pharmacokinetic Sample Collection Time And Concentration For Artesunate

Randomisation Number	Challenge Agent	Scheduled Timepoint	Collection Date	Collection Time	Day Relative to Inoculum	Day Relative to IP	Actual Time Post Artesunate Dosing (hours)	Plasma Concentration (ug/L)
R206	K13	Pre-dose	28-FEB-2018	12:48	9	1	-0.63	0.00
		0.25 Hrs Post-dose	28-FEB-2018	13:41	9	1	0.25	206.47
		0.5 Hrs Post-dose	28-FEB-2018	13:56	9	1	0.50	109.90
		1 Hrs Post-dose	28-FEB-2018	14:26	9	1	1.00	41.53
		1.5 Hrs Post-dose	28-FEB-2018	14:56	9	1	1.50	24.25
		2 Hrs Post-dose	28-FEB-2018	15:26	9	1	2.00	4.82
		2.5 Hrs Post-dose	28-FEB-2018	15:56	9	1	2.50	1.96
		3 Hrs Post-dose	28-FEB-2018	16:26	9	1	3.00	0.64
		4 Hrs Post-dose	28-FEB-2018	17:27	9	1	4.02	0.49
		6 Hrs Post-dose	28-FEB-2018	19:27	9	1	6.02	0.00
		8 Hrs Post-dose	28-FEB-2018	21:28	9	1	8.03	0.00
		10 Hrs Post-dose	28-FEB-2018	23:27	9	1	10.02	0.00
		12 Hrs Post-dose	01-MAR-2018	01:47	10	2	12.35	0.00

Notes: Day relative to inoculum is the number of days relative to day of administration of the inoculum, where the day of administration = 0.
The inoculum was administered on Study Day 0 for K13-infected subjects and Study Day 1 for 3D7-infected subjects.
Day relative to IP is the number of days relative to day of administration of Artesunate, where the day of dose = 1.
Pre-dose = Pre Artesunate dose

Listing 16.2.5.5
Plasma Pharmacokinetic Sample Collection Time And Concentration For Artesunate

Randomisation Number	Challenge Agent	Scheduled Timepoint	Collection Date	Collection Time	Day Relative to Inoculum	Day Relative to IP	Actual Time Post Artesunate Dosing (hours)	Plasma Concentration (ug/L)
R207	K13	Pre-dose	28-FEB-2018	12:00	9	1	-0.97	0.00
		0.25 Hrs Post-dose	28-FEB-2018	13:13	9	1	0.25	92.94
		0.5 Hrs Post-dose	28-FEB-2018	13:28	9	1	0.50	106.56
		1 Hrs Post-dose	28-FEB-2018	13:58	9	1	1.00	157.80
		1.5 Hrs Post-dose	28-FEB-2018	14:28	9	1	1.50	18.88
		2 Hrs Post-dose	28-FEB-2018	14:58	9	1	2.00	1.86
		2.5 Hrs Post-dose	28-FEB-2018	15:28	9	1	2.50	1.11
		3 Hrs Post-dose	28-FEB-2018	15:58	9	1	3.00	0.58
		4 Hrs Post-dose	28-FEB-2018	16:59	9	1	4.02	0.00
		6 Hrs Post-dose	28-FEB-2018	18:58	9	1	6.00	0.00
		8 Hrs Post-dose	28-FEB-2018	20:58	9	1	8.00	0.00
		10 Hrs Post-dose	28-FEB-2018	22:58	9	1	10.00	0.00
		12 Hrs Post-dose	01-MAR-2018	00:58	10	2	12.00	0.00

Notes: Day relative to inoculum is the number of days relative to day of administration of the inoculum, where the day of administration = 0.
The inoculum was administered on Study Day 0 for K13-infected subjects and Study Day 1 for 3D7-infected subjects.
Day relative to IP is the number of days relative to day of administration of Artesunate, where the day of dose = 1.
Pre-dose = Pre Artesunate dose

Listing 16.2.5.5
Plasma Pharmacokinetic Sample Collection Time And Concentration For Artesunate

Randomisation Number	Challenge Agent	Scheduled Timepoint	Collection Date	Collection Time	Day Relative to Inoculum	Day Relative to IP	Actual Time Post Artesunate Dosing (hours)	Plasma Concentration (ug/L)
R208	3D7	Pre-dose	28-FEB-2018	11:13	8	1	-0.37	0.00
		0.25 Hrs Post-dose	28-FEB-2018	11:50	8	1	0.25	19.15
		0.5 Hrs Post-dose	28-FEB-2018	12:05	8	1	0.50	624.63
		1 Hrs Post-dose	28-FEB-2018	12:35	8	1	1.00	91.90
		1.5 Hrs Post-dose	28-FEB-2018	13:05	8	1	1.50	14.91
		2 Hrs Post-dose	28-FEB-2018	13:36	8	1	2.02	5.04
		2.5 Hrs Post-dose	28-FEB-2018	14:05	8	1	2.50	1.20
		3 Hrs Post-dose	28-FEB-2018	14:35	8	1	3.00	0.32
		4 Hrs Post-dose	28-FEB-2018	15:35	8	1	4.00	0.00
		6 Hrs Post-dose	28-FEB-2018	17:35	8	1	6.00	0.00
		8 Hrs Post-dose	28-FEB-2018	19:35	8	1	8.00	0.00
		10 Hrs Post-dose	28-FEB-2018	21:46	8	1	10.18	0.00
		12 Hrs Post-dose	28-FEB-2018	23:35	8	1	12.00	0.00

Notes: Day relative to inoculum is the number of days relative to day of administration of the inoculum, where the day of administration = 0.
The inoculum was administered on Study Day 0 for K13-infected subjects and Study Day 1 for 3D7-infected subjects.
Day relative to IP is the number of days relative to day of administration of Artesunate, where the day of dose = 1.
Pre-dose = Pre Artesunate dose

Listing 16.2.5.5
Plasma Pharmacokinetic Sample Collection Time And Concentration For Artesunate

Randomisation Number	Challenge Agent	Scheduled Timepoint	Collection Date	Collection Time	Day Relative to Inoculum	Day Relative to IP	Actual Time Post Artesunate Dosing (hours)	Plasma Concentration (ug/L)
R209	3D7	Pre-dose	28-FEB-2018	14:42	8	1	-1.05	0.00
		0.25 Hrs Post-dose	28-FEB-2018	16:00	8	1	0.25	53.64
		0.5 Hrs Post-dose	28-FEB-2018	16:15	8	1	0.50	218.24
		1 Hrs Post-dose	28-FEB-2018	16:45	8	1	1.00	116.59
		1.5 Hrs Post-dose	28-FEB-2018	17:15	8	1	1.50	8.04
		2 Hrs Post-dose	28-FEB-2018	17:46	8	1	2.02	1.88
		2.5 Hrs Post-dose	28-FEB-2018	18:15	8	1	2.50	0.98
		3 Hrs Post-dose	28-FEB-2018	18:45	8	1	3.00	0.00
		4 Hrs Post-dose	28-FEB-2018	19:45	8	1	4.00	0.00
		6 Hrs Post-dose	28-FEB-2018	21:46	8	1	6.02	0.00
		8 Hrs Post-dose	28-FEB-2018	23:57	8	1	8.20	0.00
		10 Hrs Post-dose	01-MAR-2018	01:48	9	2	10.05	0.00
		12 Hrs Post-dose	01-MAR-2018	03:48	9	2	12.05	0.00

Notes: Day relative to inoculum is the number of days relative to day of administration of the inoculum, where the day of administration = 0.
The inoculum was administered on Study Day 0 for K13-infected subjects and Study Day 1 for 3D7-infected subjects.
Day relative to IP is the number of days relative to day of administration of Artesunate, where the day of dose = 1.
Pre-dose = Pre Artesunate dose

Listing 16.2.5.5
Plasma Pharmacokinetic Sample Collection Time And Concentration For Artesunate

Randomisation Number	Challenge Agent	Scheduled Timepoint	Collection Date	Collection Time	Day Relative to Inoculum	Day Relative to IP	Actual Time Post Artesunate Dosing (hours)	Plasma Concentration (ug/L)
R210	3D7	Pre-dose	28-FEB-2018	11:21	8	1	-0.40	0.00
		0.25 Hrs Post-dose	28-FEB-2018	12:00	8	1	0.25	14.84
		0.5 Hrs Post-dose	28-FEB-2018	12:15	8	1	0.50	19.77
		1 Hrs Post-dose	28-FEB-2018	12:45	8	1	1.00	27.66
		1.5 Hrs Post-dose	28-FEB-2018	13:15	8	1	1.50	63.60
		2 Hrs Post-dose	28-FEB-2018	13:45	8	1	2.00	32.62
		2.5 Hrs Post-dose	28-FEB-2018	14:15	8	1	2.50	29.39
		3 Hrs Post-dose	28-FEB-2018	14:45	8	1	3.00	13.87
		4 Hrs Post-dose	28-FEB-2018	15:45	8	1	4.00	7.39
		6 Hrs Post-dose	28-FEB-2018	17:45	8	1	6.00	5.34
		8 Hrs Post-dose	28-FEB-2018	19:45	8	1	8.00	1.97
		10 Hrs Post-dose	28-FEB-2018	21:47	8	1	10.03	0.00
		12 Hrs Post-dose	28-FEB-2018	23:45	8	1	12.00	0.00

Notes: Day relative to inoculum is the number of days relative to day of administration of the inoculum, where the day of administration = 0.
The inoculum was administered on Study Day 0 for K13-infected subjects and Study Day 1 for 3D7-infected subjects.
Day relative to IP is the number of days relative to day of administration of Artesunate, where the day of dose = 1.
Pre-dose = Pre Artesunate dose

Listing 16.2.5.5
Plasma Pharmacokinetic Sample Collection Time And Concentration For Artesunate

Randomisation Number	Challenge Agent	Scheduled Timepoint	Collection Date	Collection Time	Day Relative to Inoculum	Day Relative to IP	Actual Time Post Artesunate Dosing (hours)	Plasma Concentration (ug/L)
R302	K13	Pre-dose	13-SEP-2018	08:57	9	1	-0.13	0.00
		0.25 Hrs Post-dose	13-SEP-2018	09:20	9	1	0.25	38.10
		0.5 Hrs Post-dose	13-SEP-2018	09:35	9	1	0.50	143.80
		1 Hrs Post-dose	13-SEP-2018	10:05	9	1	1.00	226.90
		1.5 Hrs Post-dose	13-SEP-2018	10:35	9	1	1.50	6.20
		2 Hrs Post-dose	13-SEP-2018	11:05	9	1	2.00	0.20
		2.5 Hrs Post-dose	13-SEP-2018	11:35	9	1	2.50	0.00
		3 Hrs Post-dose	13-SEP-2018	12:06	9	1	3.02	0.00
		4 Hrs Post-dose	13-SEP-2018	13:05	9	1	4.00	0.00
		6 Hrs Post-dose	13-SEP-2018	15:05	9	1	6.00	0.00
		8 Hrs Post-dose	13-SEP-2018	17:06	9	1	8.02	0.00
		10 Hrs Post-dose	13-SEP-2018	19:05	9	1	10.00	0.00
		12 Hrs Post-dose	13-SEP-2018	21:05	9	1	12.00	0.00

Notes: Day relative to inoculum is the number of days relative to day of administration of the inoculum, where the day of administration = 0.
The inoculum was administered on Study Day 0 for K13-infected subjects and Study Day 1 for 3D7-infected subjects.
Day relative to IP is the number of days relative to day of administration of Artesunate, where the day of dose = 1.
Pre-dose = Pre Artesunate dose

Listing 16.2.5.5
Plasma Pharmacokinetic Sample Collection Time And Concentration For Artesunate

Randomisation Number	Challenge Agent	Scheduled Timepoint	Collection Date	Collection Time	Day Relative to Inoculum	Day Relative to IP	Actual Time Post Artesunate Dosing (hours)	Plasma Concentration (ug/L)
R303	K13	Pre-dose	13-SEP-2018	11:17	9	1	-0.02	0.00
		0.25 Hrs Post-dose	13-SEP-2018	11:36	9	1	0.30	124.20
		0.5 Hrs Post-dose	13-SEP-2018	11:49	9	1	0.52	134.50
		1 Hrs Post-dose	13-SEP-2018	12:19	9	1	1.02	634.80
		1.5 Hrs Post-dose	13-SEP-2018	12:48	9	1	1.50	13.20
		2 Hrs Post-dose	13-SEP-2018	13:18	9	1	2.00	0.50
		2.5 Hrs Post-dose	13-SEP-2018	13:48	9	1	2.50	0.00
		3 Hrs Post-dose	13-SEP-2018	14:18	9	1	3.00	0.00
		4 Hrs Post-dose	13-SEP-2018	15:20	9	1	4.03	0.00
		6 Hrs Post-dose	13-SEP-2018	17:21	9	1	6.05	0.00
		8 Hrs Post-dose	13-SEP-2018	19:18	9	1	8.00	0.00
		10 Hrs Post-dose	13-SEP-2018	21:18	9	1	10.00	0.00
		12 Hrs Post-dose	13-SEP-2018	23:19	9	1	12.02	0.00

Notes: Day relative to inoculum is the number of days relative to day of administration of the inoculum, where the day of administration = 0.
The inoculum was administered on Study Day 0 for K13-infected subjects and Study Day 1 for 3D7-infected subjects.
Day relative to IP is the number of days relative to day of administration of Artesunate, where the day of dose = 1.
Pre-dose = Pre Artesunate dose

Listing 16.2.5.5
Plasma Pharmacokinetic Sample Collection Time And Concentration For Artesunate

Randomisation Number	Challenge Agent	Scheduled Timepoint	Collection Date	Collection Time	Day Relative to Inoculum	Day Relative to IP	Actual Time Post Artesunate Dosing (hours)	Plasma Concentration (ug/L)
R304	K13	Pre-dose	13-SEP-2018	09:16	9	1	-0.10	0.00
		0.25 Hrs Post-dose	13-SEP-2018	09:37	9	1	0.25	22.70
		0.5 Hrs Post-dose	13-SEP-2018	09:52	9	1	0.50	75.20
		1 Hrs Post-dose	13-SEP-2018	10:22	9	1	1.00	110.30
		1.5 Hrs Post-dose	13-SEP-2018	10:54	9	1	1.53	4.80
		2 Hrs Post-dose	13-SEP-2018	11:22	9	1	2.00	0.10
		2.5 Hrs Post-dose	13-SEP-2018	11:52	9	1	2.50	0.00
		3 Hrs Post-dose	13-SEP-2018	12:22	9	1	3.00	0.00
		4 Hrs Post-dose	13-SEP-2018	13:22	9	1	4.00	0.00
		6 Hrs Post-dose	13-SEP-2018	15:23	9	1	6.02	0.00
		8 Hrs Post-dose	13-SEP-2018	17:23	9	1	8.02	0.00
		10 Hrs Post-dose	13-SEP-2018	19:23	9	1	10.02	0.00
		12 Hrs Post-dose	13-SEP-2018	21:23	9	1	12.02	0.00

Notes: Day relative to inoculum is the number of days relative to day of administration of the inoculum, where the day of administration = 0.
The inoculum was administered on Study Day 0 for K13-infected subjects and Study Day 1 for 3D7-infected subjects.
Day relative to IP is the number of days relative to day of administration of Artesunate, where the day of dose = 1.
Pre-dose = Pre Artesunate dose

Listing 16.2.5.5
Plasma Pharmacokinetic Sample Collection Time And Concentration For Artesunate

Randomisation Number	Challenge Agent	Scheduled Timepoint	Collection Date	Collection Time	Day Relative to Inoculum	Day Relative to IP	Actual Time Post Artesunate Dosing (hours)	Plasma Concentration (ug/L)
R305	3D7	Pre-dose	13-SEP-2018	09:29	8	1	-0.10	0.00
		0.25 Hrs Post-dose	13-SEP-2018	09:50	8	1	0.25	358.20
		0.5 Hrs Post-dose	13-SEP-2018	10:05	8	1	0.50	583.50
		1 Hrs Post-dose	13-SEP-2018	10:35	8	1	1.00	10.90
		1.5 Hrs Post-dose	13-SEP-2018	11:05	8	1	1.50	0.50
		2 Hrs Post-dose	13-SEP-2018	11:35	8	1	2.00	0.00
		2.5 Hrs Post-dose	13-SEP-2018	12:05	8	1	2.50	0.00
		3 Hrs Post-dose	13-SEP-2018	12:35	8	1	3.00	0.00
		4 Hrs Post-dose	13-SEP-2018	13:35	8	1	4.00	0.00
		6 Hrs Post-dose	13-SEP-2018	15:35	8	1	6.00	0.00
		8 Hrs Post-dose	13-SEP-2018	17:35	8	1	8.00	0.00
		10 Hrs Post-dose	13-SEP-2018	19:38	8	1	10.05	0.00
		12 Hrs Post-dose	13-SEP-2018	21:37	8	1	12.03	0.00

Notes: Day relative to inoculum is the number of days relative to day of administration of the inoculum, where the day of administration = 0.
The inoculum was administered on Study Day 0 for K13-infected subjects and Study Day 1 for 3D7-infected subjects.
Day relative to IP is the number of days relative to day of administration of Artesunate, where the day of dose = 1.
Pre-dose = Pre Artesunate dose

Listing 16.2.5.5
Plasma Pharmacokinetic Sample Collection Time And Concentration For Artesunate

Randomisation Number	Challenge Agent	Scheduled Timepoint	Collection Date	Collection Time	Day Relative to Inoculum	Day Relative to IP	Actual Time Post Artesunate Dosing (hours)	Plasma Concentration (ug/L)
R306	3D7	Pre-dose	13-SEP-2018	09:41	8	1	-0.07	0.00
		0.25 Hrs Post-dose	13-SEP-2018	10:00	8	1	0.25	56.70
		0.5 Hrs Post-dose	13-SEP-2018	10:15	8	1	0.50	161.70
		1 Hrs Post-dose	13-SEP-2018	10:45	8	1	1.00	201.90
		1.5 Hrs Post-dose	13-SEP-2018	11:15	8	1	1.50	33.60
		2 Hrs Post-dose	13-SEP-2018	11:45	8	1	2.00	5.48
		2.5 Hrs Post-dose	13-SEP-2018	12:15	8	1	2.50	5.40
		3 Hrs Post-dose	13-SEP-2018	12:45	8	1	3.00	2.10
		4 Hrs Post-dose	13-SEP-2018	13:45	8	1	4.00	0.60
		6 Hrs Post-dose	13-SEP-2018	15:45	8	1	6.00	0.00
		8 Hrs Post-dose	13-SEP-2018	17:45	8	1	8.00	0.00
		10 Hrs Post-dose	13-SEP-2018	19:45	8	1	10.00	0.00
		12 Hrs Post-dose	13-SEP-2018	21:45	8	1	12.00	0.00

Notes: Day relative to inoculum is the number of days relative to day of administration of the inoculum, where the day of administration = 0.
The inoculum was administered on Study Day 0 for K13-infected subjects and Study Day 1 for 3D7-infected subjects.
Day relative to IP is the number of days relative to day of administration of Artesunate, where the day of dose = 1.
Pre-dose = Pre Artesunate dose

Listing 16.2.5.5
Plasma Pharmacokinetic Sample Collection Time And Concentration For Artesunate

Randomisation Number	Challenge Agent	Scheduled Timepoint	Collection Date	Collection Time	Day Relative to Inoculum	Day Relative to IP	Actual Time Post Artesunate Dosing (hours)	Plasma Concentration (ug/L)
R307	3D7	Pre-dose	13-SEP-2018	09:51	8	1	-0.07	0.00
		0.25 Hrs Post-dose	13-SEP-2018	10:10	8	1	0.25	83.20
		0.5 Hrs Post-dose	13-SEP-2018	10:25	8	1	0.50	133.60
		1 Hrs Post-dose	13-SEP-2018	10:55	8	1	1.00	122.70
		1.5 Hrs Post-dose	13-SEP-2018	11:25	8	1	1.50	41.30
		2 Hrs Post-dose	13-SEP-2018	11:55	8	1	2.00	11.20
		2.5 Hrs Post-dose	13-SEP-2018	12:25	8	1	2.50	2.00
		3 Hrs Post-dose	13-SEP-2018	12:55	8	1	3.00	0.80
		4 Hrs Post-dose	13-SEP-2018	13:55	8	1	4.00	0.00
		6 Hrs Post-dose	13-SEP-2018	15:55	8	1	6.00	0.00
		8 Hrs Post-dose	13-SEP-2018	17:55	8	1	8.00	0.00
		10 Hrs Post-dose	13-SEP-2018	19:55	8	1	10.00	0.00
		12 Hrs Post-dose	13-SEP-2018	21:56	8	1	12.02	0.00

Notes: Day relative to inoculum is the number of days relative to day of administration of the inoculum, where the day of administration = 0.
The inoculum was administered on Study Day 0 for K13-infected subjects and Study Day 1 for 3D7-infected subjects.
Day relative to IP is the number of days relative to day of administration of Artesunate, where the day of dose = 1.
Pre-dose = Pre Artesunate dose

16.2.5.6 Plasma Pharmacokinetic Sample Collection Time and Concentration For Dihydroartemisinin

Listing 16.2.5.6
Plasma Pharmacokinetic Sample Collection Time And Concentration For Dihydroartemisinin

Randomisation Number	Challenge Agent	Scheduled Timepoint	Collection Date	Collection Time	Day Relative to Inoculum	Day Relative to IP	Actual Time Post Artesunate Dosing (hours)	Plasma Concentration (ug/L)
R103	K13	Pre-dose	23-NOV-2017	09:50	9	1	-1.00	0.00
		0.25 Hrs Post-dose	23-NOV-2017	11:05	9	1	0.25	13.28
		0.5 Hrs Post-dose	23-NOV-2017	11:20	9	1	0.50	53.21
		1 Hrs Post-dose	23-NOV-2017	11:50	9	1	1.00	109.22
		1.5 Hrs Post-dose	23-NOV-2017	12:20	9	1	1.50	111.22
		2 Hrs Post-dose	23-NOV-2017	12:50	9	1	2.00	183.68
		2.5 Hrs Post-dose	23-NOV-2017	13:20	9	1	2.50	103.34
		3 Hrs Post-dose	23-NOV-2017	13:50	9	1	3.00	85.61
		4 Hrs Post-dose	23-NOV-2017	14:50	9	1	4.00	92.44
		6 Hrs Post-dose	23-NOV-2017	16:56	9	1	6.10	28.01
		8 Hrs Post-dose	23-NOV-2017	18:50	9	1	8.00	23.30
		10 Hrs Post-dose	23-NOV-2017	20:50	9	1	10.00	14.84
		12 Hrs Post-dose	23-NOV-2017	22:50	9	1	12.00	5.22

Notes: Day relative to inoculum is the number of days relative to day of administration of the inoculum, where the day of administration = 0.
The inoculum was administered on Study Day 0 for K13-infected subjects and Study Day 1 for 3D7-infected subjects.
Day relative to IP is the number of days relative to day of administration of Artesunate, where the day of dose = 1.
Pre-dose = Pre Artesunate dose

Printed: 24FEB2020 (Final)

Listing 16.2.5.6
Plasma Pharmacokinetic Sample Collection Time And Concentration For Dihydroartemisinin

Randomisation Number	Challenge Agent	Scheduled Timepoint	Collection Date	Collection Time	Day Relative to Inoculum	Day Relative to IP	Actual Time Post Artesunate Dosing (hours)	Plasma Concentration (ug/L)
R104	K13	Pre-dose	23-NOV-2017	09:44	9	1	-0.93	0.00
		0.25 Hrs Post-dose	23-NOV-2017	10:55	9	1	0.25	18.54
		0.5 Hrs Post-dose	23-NOV-2017	11:10	9	1	0.50	80.29
		1 Hrs Post-dose	23-NOV-2017	11:40	9	1	1.00	293.36
		1.5 Hrs Post-dose	23-NOV-2017	12:10	9	1	1.50	272.65
		2 Hrs Post-dose	23-NOV-2017	12:40	9	1	2.00	250.30
		2.5 Hrs Post-dose	23-NOV-2017	13:10	9	1	2.50	141.28
		3 Hrs Post-dose	23-NOV-2017	13:40	9	1	3.00	111.75
		4 Hrs Post-dose	23-NOV-2017	14:40	9	1	4.00	66.45
		6 Hrs Post-dose	23-NOV-2017	16:41	9	1	6.02	42.80
		8 Hrs Post-dose	23-NOV-2017	18:40	9	1	8.00	28.46
		10 Hrs Post-dose	23-NOV-2017	20:40	9	1	10.00	25.37
		12 Hrs Post-dose	23-NOV-2017	22:40	9	1	12.00	4.93

Notes: Day relative to inoculum is the number of days relative to day of administration of the inoculum, where the day of administration = 0.
The inoculum was administered on Study Day 0 for K13-infected subjects and Study Day 1 for 3D7-infected subjects.
Day relative to IP is the number of days relative to day of administration of Artesunate, where the day of dose = 1.
Pre-dose = Pre Artesunate dose

Listing 16.2.5.6
Plasma Pharmacokinetic Sample Collection Time And Concentration For Dihydroartemisinin

Randomisation Number	Challenge Agent	Scheduled Timepoint	Collection Date	Collection Time	Day Relative to Inoculum	Day Relative to IP	Actual Time Post Artesunate Dosing (hours)	Plasma Concentration (ug/L)
R105	K13	Pre-dose	23-NOV-2017	09:36	9	1	-0.90	0.00
		0.25 Hrs Post-dose	23-NOV-2017	10:45	9	1	0.25	18.56
		0.5 Hrs Post-dose	23-NOV-2017	11:00	9	1	0.50	64.04
		1 Hrs Post-dose	23-NOV-2017	11:30	9	1	1.00	414.41
		1.5 Hrs Post-dose	23-NOV-2017	12:00	9	1	1.50	326.49
		2 Hrs Post-dose	23-NOV-2017	12:31	9	1	2.02	671.72
		2.5 Hrs Post-dose	23-NOV-2017	13:00	9	1	2.50	278.12
		3 Hrs Post-dose	23-NOV-2017	13:30	9	1	3.00	133.61
		4 Hrs Post-dose	23-NOV-2017	14:30	9	1	4.00	88.92
		6 Hrs Post-dose	23-NOV-2017	16:31	9	1	6.02	54.20
		8 Hrs Post-dose	23-NOV-2017	18:30	9	1	8.00	33.43
		10 Hrs Post-dose	23-NOV-2017	20:30	9	1	10.00	19.36
		12 Hrs Post-dose	23-NOV-2017	22:31	9	1	12.02	13.69

Notes: Day relative to inoculum is the number of days relative to day of administration of the inoculum, where the day of administration = 0.
The inoculum was administered on Study Day 0 for K13-infected subjects and Study Day 1 for 3D7-infected subjects.
Day relative to IP is the number of days relative to day of administration of Artesunate, where the day of dose = 1.
Pre-dose = Pre Artesunate dose

Listing 16.2.5.6
Plasma Pharmacokinetic Sample Collection Time And Concentration For Dihydroartemisinin

Randomisation Number	Challenge Agent	Scheduled Timepoint	Collection Date	Collection Time	Day Relative to Inoculum	Day Relative to IP	Actual Time Post Artesunate Dosing (hours)	Plasma Concentration (ug/L)
R106	3D7	Pre-dose	23-NOV-2017	09:33	8	1	-0.78	0.00
		0.25 Hrs Post-dose	23-NOV-2017	10:35	8	1	0.25	27.72
		0.5 Hrs Post-dose	23-NOV-2017	10:50	8	1	0.50	45.15
		1 Hrs Post-dose	23-NOV-2017	11:20	8	1	1.00	101.23
		1.5 Hrs Post-dose	23-NOV-2017	11:50	8	1	1.50	78.88
		2 Hrs Post-dose	23-NOV-2017	12:20	8	1	2.00	560.32
		2.5 Hrs Post-dose	23-NOV-2017	12:50	8	1	2.50	148.21
		3 Hrs Post-dose	23-NOV-2017	13:20	8	1	3.00	96.58
		4 Hrs Post-dose	23-NOV-2017	14:20	8	1	4.00	78.08
		6 Hrs Post-dose	23-NOV-2017	16:22	8	1	6.03	40.40
		8 Hrs Post-dose	23-NOV-2017	18:20	8	1	8.00	26.17
		10 Hrs Post-dose	23-NOV-2017	20:20	8	1	10.00	21.45
		12 Hrs Post-dose	23-NOV-2017	22:32	8	1	12.20	10.21

Notes: Day relative to inoculum is the number of days relative to day of administration of the inoculum, where the day of administration = 0.
The inoculum was administered on Study Day 0 for K13-infected subjects and Study Day 1 for 3D7-infected subjects.
Day relative to IP is the number of days relative to day of administration of Artesunate, where the day of dose = 1.
Pre-dose = Pre Artesunate dose

Listing 16.2.5.6
Plasma Pharmacokinetic Sample Collection Time And Concentration For Dihydroartemisinin

Randomisation Number	Challenge Agent	Scheduled Timepoint	Collection Date	Collection Time	Day Relative to Inoculum	Day Relative to IP	Actual Time Post Artesunate Dosing (hours)	Plasma Concentration (ug/L)
R107	3D7	Pre-dose	23-NOV-2017	09:35	8	1	-0.58	0.00
		0.25 Hrs Post-dose	23-NOV-2017	10:25	8	1	0.25	31.97
		0.5 Hrs Post-dose	23-NOV-2017	10:40	8	1	0.50	858.00
		1 Hrs Post-dose	23-NOV-2017	11:10	8	1	1.00	523.87
		1.5 Hrs Post-dose	23-NOV-2017	11:40	8	1	1.50	328.55
		2 Hrs Post-dose	23-NOV-2017	12:10	8	1	2.00	263.90
		2.5 Hrs Post-dose	23-NOV-2017	12:40	8	1	2.50	131.65
		3 Hrs Post-dose	23-NOV-2017	13:11	8	1	3.02	101.57
		4 Hrs Post-dose	23-NOV-2017	14:10	8	1	4.00	66.51
		6 Hrs Post-dose	23-NOV-2017	16:10	8	1	6.00	46.27
		8 Hrs Post-dose	23-NOV-2017	18:10	8	1	8.00	32.94
		10 Hrs Post-dose	23-NOV-2017	20:10	8	1	10.00	25.76
		12 Hrs Post-dose	23-NOV-2017	22:10	8	1	12.00	17.20

Notes: Day relative to inoculum is the number of days relative to day of administration of the inoculum, where the day of administration = 0.
The inoculum was administered on Study Day 0 for K13-infected subjects and Study Day 1 for 3D7-infected subjects.
Day relative to IP is the number of days relative to day of administration of Artesunate, where the day of dose = 1.
Pre-dose = Pre Artesunate dose

Listing 16.2.5.6
Plasma Pharmacokinetic Sample Collection Time And Concentration For Dihydroartemisinin

Randomisation Number	Challenge Agent	Scheduled Timepoint	Collection Date	Collection Time	Day Relative to Inoculum	Day Relative to IP	Actual Time Post Artesunate Dosing (hours)	Plasma Concentration (ug/L)
R108	3D7	Pre-dose	23-NOV-2017	09:17	8	1	-0.73	0.00
		0.25 Hrs Post-dose	23-NOV-2017	10:16	8	1	0.25	23.52
		0.5 Hrs Post-dose	23-NOV-2017	10:31	8	1	0.50	60.64
		1 Hrs Post-dose	23-NOV-2017	11:01	8	1	1.00	735.36
		1.5 Hrs Post-dose	23-NOV-2017	11:31	8	1	1.50	387.68
		2 Hrs Post-dose	23-NOV-2017	12:05	8	1	2.07	311.95
		2.5 Hrs Post-dose	23-NOV-2017	12:31	8	1	2.50	241.09
		3 Hrs Post-dose	23-NOV-2017	13:03	8	1	3.03	119.23
		4 Hrs Post-dose	23-NOV-2017	14:01	8	1	4.00	76.11
		6 Hrs Post-dose	23-NOV-2017	16:12	8	1	6.18	47.16
		8 Hrs Post-dose	23-NOV-2017	18:02	8	1	8.02	39.78
		10 Hrs Post-dose	23-NOV-2017	20:01	8	1	10.00	27.86
		12 Hrs Post-dose	23-NOV-2017	22:02	8	1	12.02	15.96

Notes: Day relative to inoculum is the number of days relative to day of administration of the inoculum, where the day of administration = 0.
The inoculum was administered on Study Day 0 for K13-infected subjects and Study Day 1 for 3D7-infected subjects.
Day relative to IP is the number of days relative to day of administration of Artesunate, where the day of dose = 1.
Pre-dose = Pre Artesunate dose

Listing 16.2.5.6

Plasma Pharmacokinetic Sample Collection Time And Concentration For Dihydroartemisinin

Randomisation Number	Challenge Agent	Scheduled Timepoint	Collection Date	Collection Time	Day Relative to Inoculum	Day Relative to IP	Actual Time Post Artesunate Dosing (hours)	Plasma Concentration (ug/L)
R201	K13	Pre-dose	28-FEB-2018	12:39	9	1	-0.72	0.00
		0.25 Hrs Post-dose	28-FEB-2018	13:37	9	1	0.25	19.69
		0.5 Hrs Post-dose	28-FEB-2018	13:52	9	1	0.50	100.07
		1 Hrs Post-dose	28-FEB-2018	14:22	9	1	1.00	165.11
		1.5 Hrs Post-dose	28-FEB-2018	14:52	9	1	1.50	1247.95
		2 Hrs Post-dose	28-FEB-2018	15:23	9	1	2.02	673.60
		2.5 Hrs Post-dose	28-FEB-2018	15:52	9	1	2.50	298.10
		3 Hrs Post-dose	28-FEB-2018	16:22	9	1	3.00	264.57
		4 Hrs Post-dose	28-FEB-2018	17:22	9	1	4.00	102.95
		6 Hrs Post-dose	28-FEB-2018	19:22	9	1	6.00	64.16
		8 Hrs Post-dose	28-FEB-2018	21:22	9	1	8.00	36.64
		10 Hrs Post-dose	28-FEB-2018	23:28	9	1	10.10	33.86
		12 Hrs Post-dose	01-MAR-2018	01:28	10	2	12.10	3.45

Notes: Day relative to inoculum is the number of days relative to day of administration of the inoculum, where the day of administration = 0.
The inoculum was administered on Study Day 0 for K13-infected subjects and Study Day 1 for 3D7-infected subjects.
Day relative to IP is the number of days relative to day of administration of Artesunate, where the day of dose = 1.
Pre-dose = Pre Artesunate dose

Printed: 24FEB2020 (Final)

Listing 16.2.5.6
Plasma Pharmacokinetic Sample Collection Time And Concentration For Dihydroartemisinin

Randomisation Number	Challenge Agent	Scheduled Timepoint	Collection Date	Collection Time	Day Relative to Inoculum	Day Relative to IP	Actual Time Post Artesunate Dosing (hours)	Plasma Concentration (ug/L)
R202	K13	Pre-dose	28-FEB-2018	12:05	9	1	-0.97	0.00
		0.25 Hrs Post-dose	28-FEB-2018	13:18	9	1	0.25	48.08
		0.5 Hrs Post-dose	28-FEB-2018	13:33	9	1	0.50	428.64
		1 Hrs Post-dose	28-FEB-2018	14:03	9	1	1.00	254.01
		1.5 Hrs Post-dose	28-FEB-2018	14:33	9	1	1.50	151.10
		2 Hrs Post-dose	28-FEB-2018	15:04	9	1	2.02	68.44
		2.5 Hrs Post-dose	28-FEB-2018	15:33	9	1	2.50	31.65
		3 Hrs Post-dose	28-FEB-2018	16:03	9	1	3.00	10.57
		4 Hrs Post-dose	28-FEB-2018	17:03	9	1	4.00	2.89
		6 Hrs Post-dose	28-FEB-2018	19:03	9	1	6.00	1.23
		8 Hrs Post-dose	28-FEB-2018	21:03	9	1	8.00	0.71
		10 Hrs Post-dose	28-FEB-2018	23:03	9	1	10.00	0.00
		12 Hrs Post-dose	01-MAR-2018	01:03	10	2	12.00	0.00

Notes: Day relative to inoculum is the number of days relative to day of administration of the inoculum, where the day of administration = 0.
The inoculum was administered on Study Day 0 for K13-infected subjects and Study Day 1 for 3D7-infected subjects.
Day relative to IP is the number of days relative to day of administration of Artesunate, where the day of dose = 1.
Pre-dose = Pre Artesunate dose

Listing 16.2.5.6
Plasma Pharmacokinetic Sample Collection Time And Concentration For Dihydroartemisinin

Randomisation Number	Challenge Agent	Scheduled Timepoint	Collection Date	Collection Time	Day Relative to Inoculum	Day Relative to IP	Actual Time Post Artesunate Dosing (hours)	Plasma Concentration (ug/L)
R203	K13	Pre-dose	28-FEB-2018	11:14	9	1	-0.43	0.00
		0.25 Hrs Post-dose	28-FEB-2018	11:55	9	1	0.25	68.53
		0.5 Hrs Post-dose	28-FEB-2018	12:10	9	1	0.50	285.41
		1 Hrs Post-dose	28-FEB-2018	12:40	9	1	1.00	293.10
		1.5 Hrs Post-dose	28-FEB-2018	13:10	9	1	1.50	150.52
		2 Hrs Post-dose	28-FEB-2018	13:40	9	1	2.00	76.92
		2.5 Hrs Post-dose	28-FEB-2018	14:10	9	1	2.50	55.24
		3 Hrs Post-dose	28-FEB-2018	14:40	9	1	3.00	45.84
		4 Hrs Post-dose	28-FEB-2018	15:40	9	1	4.00	23.68
		6 Hrs Post-dose	28-FEB-2018	17:41	9	1	6.02	7.78
		8 Hrs Post-dose	28-FEB-2018	19:40	9	1	8.00	0.00
		10 Hrs Post-dose	28-FEB-2018	21:44	9	1	10.07	0.00
		12 Hrs Post-dose	28-FEB-2018	23:40	9	1	12.00	0.00

Notes: Day relative to inoculum is the number of days relative to day of administration of the inoculum, where the day of administration = 0.
The inoculum was administered on Study Day 0 for K13-infected subjects and Study Day 1 for 3D7-infected subjects.
Day relative to IP is the number of days relative to day of administration of Artesunate, where the day of dose = 1.
Pre-dose = Pre Artesunate dose

Listing 16.2.5.6
Plasma Pharmacokinetic Sample Collection Time And Concentration For Dihydroartemisinin

Randomisation Number	Challenge Agent	Scheduled Timepoint	Collection Date	Collection Time	Day Relative to Inoculum	Day Relative to IP	Actual Time Post Artesunate Dosing (hours)	Plasma Concentration (ug/L)
R204	K13	Pre-dose	28-FEB-2018	12:29	9	1	-0.77	0.00
		0.25 Hrs Post-dose	28-FEB-2018	13:30	9	1	0.25	16.11
		0.5 Hrs Post-dose	28-FEB-2018	13:45	9	1	0.50	510.90
		1 Hrs Post-dose	28-FEB-2018	14:15	9	1	1.00	417.27
		1.5 Hrs Post-dose	28-FEB-2018	14:45	9	1	1.50	211.97
		2 Hrs Post-dose	28-FEB-2018	15:15	9	1	2.00	142.50
		2.5 Hrs Post-dose	28-FEB-2018	15:45	9	1	2.50	78.36
		3 Hrs Post-dose	28-FEB-2018	16:15	9	1	3.00	54.09
		4 Hrs Post-dose	28-FEB-2018	17:15	9	1	4.00	28.02
		6 Hrs Post-dose	28-FEB-2018	19:15	9	1	6.00	6.61
		8 Hrs Post-dose	28-FEB-2018	21:17	9	1	8.03	4.29
		10 Hrs Post-dose	28-FEB-2018	23:15	9	1	10.00	0.00
		12 Hrs Post-dose	01-MAR-2018	01:15	10	2	12.00	0.00

Notes: Day relative to inoculum is the number of days relative to day of administration of the inoculum, where the day of administration = 0.
The inoculum was administered on Study Day 0 for K13-infected subjects and Study Day 1 for 3D7-infected subjects.
Day relative to IP is the number of days relative to day of administration of Artesunate, where the day of dose = 1.
Pre-dose = Pre Artesunate dose

Listing 16.2.5.6
Plasma Pharmacokinetic Sample Collection Time And Concentration For Dihydroartemisinin

Randomisation Number	Challenge Agent	Scheduled Timepoint	Collection Date	Collection Time	Day Relative to Inoculum	Day Relative to IP	Actual Time Post Artesunate Dosing (hours)	Plasma Concentration (ug/L)
R205	K13	Pre-dose	28-FEB-2018	12:19	9	1	-0.85	0.00
		0.25 Hrs Post-dose	28-FEB-2018	13:25	9	1	0.25	1.35
		0.5 Hrs Post-dose	28-FEB-2018	13:40	9	1	0.50	19.62
		1 Hrs Post-dose	28-FEB-2018	14:10	9	1	1.00	368.88
		1.5 Hrs Post-dose	28-FEB-2018	14:40	9	1	1.50	295.89
		2 Hrs Post-dose	28-FEB-2018	15:11	9	1	2.02	212.44
		2.5 Hrs Post-dose	28-FEB-2018	15:40	9	1	2.50	115.53
		3 Hrs Post-dose	28-FEB-2018	16:10	9	1	3.00	128.00
		4 Hrs Post-dose	28-FEB-2018	17:10	9	1	4.00	26.22
		6 Hrs Post-dose	28-FEB-2018	19:11	9	1	6.02	6.29
		8 Hrs Post-dose	28-FEB-2018	21:10	9	1	8.00	1.78
		10 Hrs Post-dose	28-FEB-2018	23:10	9	1	10.00	0.00
		12 Hrs Post-dose	01-MAR-2018	01:10	10	2	12.00	0.00

Notes: Day relative to inoculum is the number of days relative to day of administration of the inoculum, where the day of administration = 0.
The inoculum was administered on Study Day 0 for K13-infected subjects and Study Day 1 for 3D7-infected subjects.
Day relative to IP is the number of days relative to day of administration of Artesunate, where the day of dose = 1.
Pre-dose = Pre Artesunate dose

Printed: 24FEB2020 (Final)

Listing 16.2.5.6
Plasma Pharmacokinetic Sample Collection Time And Concentration For Dihydroartemisinin

Randomisation Number	Challenge Agent	Scheduled Timepoint	Collection Date	Collection Time	Day Relative to Inoculum	Day Relative to IP	Actual Time Post Artesunate Dosing (hours)	Plasma Concentration (ug/L)
R206	K13	Pre-dose	28-FEB-2018	12:48	9	1	-0.63	0.00
		0.25 Hrs Post-dose	28-FEB-2018	13:41	9	1	0.25	48.22
		0.5 Hrs Post-dose	28-FEB-2018	13:56	9	1	0.50	277.46
		1 Hrs Post-dose	28-FEB-2018	14:26	9	1	1.00	265.51
		1.5 Hrs Post-dose	28-FEB-2018	14:56	9	1	1.50	270.13
		2 Hrs Post-dose	28-FEB-2018	15:26	9	1	2.00	113.58
		2.5 Hrs Post-dose	28-FEB-2018	15:56	9	1	2.50	58.38
		3 Hrs Post-dose	28-FEB-2018	16:26	9	1	3.00	23.54
		4 Hrs Post-dose	28-FEB-2018	17:27	9	1	4.02	22.67
		6 Hrs Post-dose	28-FEB-2018	19:27	9	1	6.02	0.46
		8 Hrs Post-dose	28-FEB-2018	21:28	9	1	8.03	0.00
		10 Hrs Post-dose	28-FEB-2018	23:27	9	1	10.02	0.00
		12 Hrs Post-dose	01-MAR-2018	01:47	10	2	12.35	0.00

Notes: Day relative to inoculum is the number of days relative to day of administration of the inoculum, where the day of administration = 0.
The inoculum was administered on Study Day 0 for K13-infected subjects and Study Day 1 for 3D7-infected subjects.
Day relative to IP is the number of days relative to day of administration of Artesunate, where the day of dose = 1.
Pre-dose = Pre Artesunate dose

Listing 16.2.5.6
Plasma Pharmacokinetic Sample Collection Time And Concentration For Dihydroartemisinin

Randomisation Number	Challenge Agent	Scheduled Timepoint	Collection Date	Collection Time	Day Relative to Inoculum	Day Relative to IP	Actual Time Post Artesunate Dosing (hours)	Plasma Concentration (ug/L)
R207	K13	Pre-dose	28-FEB-2018	12:00	9	1	-0.97	0.00
		0.25 Hrs Post-dose	28-FEB-2018	13:13	9	1	0.25	18.08
		0.5 Hrs Post-dose	28-FEB-2018	13:28	9	1	0.50	117.26
		1 Hrs Post-dose	28-FEB-2018	13:58	9	1	1.00	402.42
		1.5 Hrs Post-dose	28-FEB-2018	14:28	9	1	1.50	648.20
		2 Hrs Post-dose	28-FEB-2018	14:58	9	1	2.00	253.58
		2.5 Hrs Post-dose	28-FEB-2018	15:28	9	1	2.50	119.62
		3 Hrs Post-dose	28-FEB-2018	15:58	9	1	3.00	70.51
		4 Hrs Post-dose	28-FEB-2018	16:59	9	1	4.02	28.20
		6 Hrs Post-dose	28-FEB-2018	18:58	9	1	6.00	10.61
		8 Hrs Post-dose	28-FEB-2018	20:58	9	1	8.00	2.94
		10 Hrs Post-dose	28-FEB-2018	22:58	9	1	10.00	0.00
		12 Hrs Post-dose	01-MAR-2018	00:58	10	2	12.00	0.00

Notes: Day relative to inoculum is the number of days relative to day of administration of the inoculum, where the day of administration = 0.
The inoculum was administered on Study Day 0 for K13-infected subjects and Study Day 1 for 3D7-infected subjects.
Day relative to IP is the number of days relative to day of administration of Artesunate, where the day of dose = 1.
Pre-dose = Pre Artesunate dose

Listing 16.2.5.6
Plasma Pharmacokinetic Sample Collection Time And Concentration For Dihydroartemisinin

Randomisation Number	Challenge Agent	Scheduled Timepoint	Collection Date	Collection Time	Day Relative to Inoculum	Day Relative to IP	Actual Time Post Artesunate Dosing (hours)	Plasma Concentration (ug/L)
R208	3D7	Pre-dose	28-FEB-2018	11:13	8	1	-0.37	0.00
		0.25 Hrs Post-dose	28-FEB-2018	11:50	8	1	0.25	8.38
		0.5 Hrs Post-dose	28-FEB-2018	12:05	8	1	0.50	346.34
		1 Hrs Post-dose	28-FEB-2018	12:35	8	1	1.00	955.10
		1.5 Hrs Post-dose	28-FEB-2018	13:05	8	1	1.50	511.42
		2 Hrs Post-dose	28-FEB-2018	13:36	8	1	2.02	254.46
		2.5 Hrs Post-dose	28-FEB-2018	14:05	8	1	2.50	106.31
		3 Hrs Post-dose	28-FEB-2018	14:35	8	1	3.00	104.27
		4 Hrs Post-dose	28-FEB-2018	15:35	8	1	4.00	36.33
		6 Hrs Post-dose	28-FEB-2018	17:35	8	1	6.00	5.76
		8 Hrs Post-dose	28-FEB-2018	19:35	8	1	8.00	2.46
		10 Hrs Post-dose	28-FEB-2018	21:46	8	1	10.18	0.44
		12 Hrs Post-dose	28-FEB-2018	23:35	8	1	12.00	0.00

Notes: Day relative to inoculum is the number of days relative to day of administration of the inoculum, where the day of administration = 0.
The inoculum was administered on Study Day 0 for K13-infected subjects and Study Day 1 for 3D7-infected subjects.
Day relative to IP is the number of days relative to day of administration of Artesunate, where the day of dose = 1.
Pre-dose = Pre Artesunate dose

Listing 16.2.5.6
Plasma Pharmacokinetic Sample Collection Time And Concentration For Dihydroartemisinin

Randomisation Number	Challenge Agent	Scheduled Timepoint	Collection Date	Collection Time	Day Relative to Inoculum	Day Relative to IP	Actual Time Post Artesunate Dosing (hours)	Plasma Concentration (ug/L)
R209	3D7	Pre-dose	28-FEB-2018	14:42	8	1	-1.05	0.00
		0.25 Hrs Post-dose	28-FEB-2018	16:00	8	1	0.25	0.00
		0.5 Hrs Post-dose	28-FEB-2018	16:15	8	1	0.50	48.07
		1 Hrs Post-dose	28-FEB-2018	16:45	8	1	1.00	580.45
		1.5 Hrs Post-dose	28-FEB-2018	17:15	8	1	1.50	394.43
		2 Hrs Post-dose	28-FEB-2018	17:46	8	1	2.02	258.82
		2.5 Hrs Post-dose	28-FEB-2018	18:15	8	1	2.50	158.92
		3 Hrs Post-dose	28-FEB-2018	18:45	8	1	3.00	102.93
		4 Hrs Post-dose	28-FEB-2018	19:45	8	1	4.00	28.12
		6 Hrs Post-dose	28-FEB-2018	21:46	8	1	6.02	20.93
		8 Hrs Post-dose	28-FEB-2018	23:57	8	1	8.20	0.00
		10 Hrs Post-dose	01-MAR-2018	01:48	9	2	10.05	1.72
		12 Hrs Post-dose	01-MAR-2018	03:48	9	2	12.05	0.00

Notes: Day relative to inoculum is the number of days relative to day of administration of the inoculum, where the day of administration = 0.
The inoculum was administered on Study Day 0 for K13-infected subjects and Study Day 1 for 3D7-infected subjects.
Day relative to IP is the number of days relative to day of administration of Artesunate, where the day of dose = 1.
Pre-dose = Pre Artesunate dose

Listing 16.2.5.6
Plasma Pharmacokinetic Sample Collection Time And Concentration For Dihydroartemisinin

**Randomisation Number**	**Challenge Agent**	**Scheduled Timepoint**	**Collection Date**	**Collection Time**	**Day Relative to Inoculum**	**Day Relative to IP**	**Actual Time Post Artesunate Dosing (hours)**	**Plasma Concentration (ug/L)**
R210	3D7	Pre-dose	28-FEB-2018	11:21	8	1	-0.40	0.00
		0.25 Hrs Post-dose	28-FEB-2018	12:00	8	1	0.25	1.61
		0.5 Hrs Post-dose	28-FEB-2018	12:15	8	1	0.50	3.40
		1 Hrs Post-dose	28-FEB-2018	12:45	8	1	1.00	7.64
		1.5 Hrs Post-dose	28-FEB-2018	13:15	8	1	1.50	39.06
		2 Hrs Post-dose	28-FEB-2018	13:45	8	1	2.00	19.75
		2.5 Hrs Post-dose	28-FEB-2018	14:15	8	1	2.50	17.16
		3 Hrs Post-dose	28-FEB-2018	14:45	8	1	3.00	21.35
		4 Hrs Post-dose	28-FEB-2018	15:45	8	1	4.00	4.50
		6 Hrs Post-dose	28-FEB-2018	17:45	8	1	6.00	7.10
		8 Hrs Post-dose	28-FEB-2018	19:45	8	1	8.00	0.00
		10 Hrs Post-dose	28-FEB-2018	21:47	8	1	10.03	0.00
		12 Hrs Post-dose	28-FEB-2018	23:45	8	1	12.00	0.00

Notes: Day relative to inoculum is the number of days relative to day of administration of the inoculum, where the day of administration = 0.
The inoculum was administered on Study Day 0 for K13-infected subjects and Study Day 1 for 3D7-infected subjects.
Day relative to IP is the number of days relative to day of administration of Artesunate, where the day of dose = 1.
Pre-dose = Pre Artesunate dose

Listing 16.2.5.6
Plasma Pharmacokinetic Sample Collection Time And Concentration For Dihydroartemisinin

Randomisation Number	Challenge Agent	Scheduled Timepoint	Collection Date	Collection Time	Day Relative to Inoculum	Day Relative to IP	Actual Time Post Artesunate Dosing (hours)	Plasma Concentration (ug/L)
R302	K13	Pre-dose	13-SEP-2018	08:57	9	1	-0.13	0.00
		0.25 Hrs Post-dose	13-SEP-2018	09:20	9	1	0.25	0.00
		0.5 Hrs Post-dose	13-SEP-2018	09:35	9	1	0.50	50.50
		1 Hrs Post-dose	13-SEP-2018	10:05	9	1	1.00	1133.80
		1.5 Hrs Post-dose	13-SEP-2018	10:35	9	1	1.50	420.30
		2 Hrs Post-dose	13-SEP-2018	11:05	9	1	2.00	154.60
		2.5 Hrs Post-dose	13-SEP-2018	11:35	9	1	2.50	105.90
		3 Hrs Post-dose	13-SEP-2018	12:06	9	1	3.02	56.70
		4 Hrs Post-dose	13-SEP-2018	13:05	9	1	4.00	26.20
		6 Hrs Post-dose	13-SEP-2018	15:05	9	1	6.00	16.60
		8 Hrs Post-dose	13-SEP-2018	17:06	9	1	8.02	4.30
		10 Hrs Post-dose	13-SEP-2018	19:05	9	1	10.00	0.00
		12 Hrs Post-dose	13-SEP-2018	21:05	9	1	12.00	0.00

Notes: Day relative to inoculum is the number of days relative to day of administration of the inoculum, where the day of administration = 0.
The inoculum was administered on Study Day 0 for K13-infected subjects and Study Day 1 for 3D7-infected subjects.
Day relative to IP is the number of days relative to day of administration of Artesunate, where the day of dose = 1.
Pre-dose = Pre Artesunate dose

Listing 16.2.5.6
Plasma Pharmacokinetic Sample Collection Time And Concentration For Dihydroartemisinin

Randomisation Number	Challenge Agent	Scheduled Timepoint	Collection Date	Collection Time	Day Relative to Inoculum	Day Relative to IP	Actual Time Post Artesunate Dosing (hours)	Plasma Concentration (ug/L)
R303	K13	Pre-dose	13-SEP-2018	11:17	9	1	-0.02	0.00
		0.25 Hrs Post-dose	13-SEP-2018	11:36	9	1	0.30	4.60
		0.5 Hrs Post-dose	13-SEP-2018	11:49	9	1	0.52	47.70
		1 Hrs Post-dose	13-SEP-2018	12:19	9	1	1.02	361.50
		1.5 Hrs Post-dose	13-SEP-2018	12:48	9	1	1.50	281.60
		2 Hrs Post-dose	13-SEP-2018	13:18	9	1	2.00	95.20
		2.5 Hrs Post-dose	13-SEP-2018	13:48	9	1	2.50	51.70
		3 Hrs Post-dose	13-SEP-2018	14:18	9	1	3.00	35.36
		4 Hrs Post-dose	13-SEP-2018	15:20	9	1	4.03	18.80
		6 Hrs Post-dose	13-SEP-2018	17:21	9	1	6.05	0.00
		8 Hrs Post-dose	13-SEP-2018	19:18	9	1	8.00	0.00
		10 Hrs Post-dose	13-SEP-2018	21:18	9	1	10.00	0.00
		12 Hrs Post-dose	13-SEP-2018	23:19	9	1	12.02	0.00

Notes: Day relative to inoculum is the number of days relative to day of administration of the inoculum, where the day of administration = 0.
The inoculum was administered on Study Day 0 for K13-infected subjects and Study Day 1 for 3D7-infected subjects.
Day relative to IP is the number of days relative to day of administration of Artesunate, where the day of dose = 1.
Pre-dose = Pre Artesunate dose

Listing 16.2.5.6
Plasma Pharmacokinetic Sample Collection Time And Concentration For Dihydroartemisinin

Randomisation Number	Challenge Agent	Scheduled Timepoint	Collection Date	Collection Time	Day Relative to Inoculum	Day Relative to IP	Actual Time Post Artesunate Dosing (hours)	Plasma Concentration (ug/L)
R304	K13	Pre-dose	13-SEP-2018	09:16	9	1	-0.10	0.00
		0.25 Hrs Post-dose	13-SEP-2018	09:37	9	1	0.25	4.10
		0.5 Hrs Post-dose	13-SEP-2018	09:52	9	1	0.50	38.40
		1 Hrs Post-dose	13-SEP-2018	10:22	9	1	1.00	304.10
		1.5 Hrs Post-dose	13-SEP-2018	10:54	9	1	1.53	363.60
		2 Hrs Post-dose	13-SEP-2018	11:22	9	1	2.00	84.10
		2.5 Hrs Post-dose	13-SEP-2018	11:52	9	1	2.50	51.40
		3 Hrs Post-dose	13-SEP-2018	12:22	9	1	3.00	20.05
		4 Hrs Post-dose	13-SEP-2018	13:22	9	1	4.00	1.30
		6 Hrs Post-dose	13-SEP-2018	15:23	9	1	6.02	0.00
		8 Hrs Post-dose	13-SEP-2018	17:23	9	1	8.02	0.00
		10 Hrs Post-dose	13-SEP-2018	19:23	9	1	10.02	0.00
		12 Hrs Post-dose	13-SEP-2018	21:23	9	1	12.02	0.00

Notes: Day relative to inoculum is the number of days relative to day of administration of the inoculum, where the day of administration = 0.
The inoculum was administered on Study Day 0 for K13-infected subjects and Study Day 1 for 3D7-infected subjects.
Day relative to IP is the number of days relative to day of administration of Artesunate, where the day of dose = 1.
Pre-dose = Pre Artesunate dose

Listing 16.2.5.6
Plasma Pharmacokinetic Sample Collection Time And Concentration For Dihydroartemisinin

Randomisation Number	Challenge Agent	Scheduled Timepoint	Collection Date	Collection Time	Day Relative to Inoculum	Day Relative to IP	Actual Time Post Artesunate Dosing (hours)	Plasma Concentration (ug/L)
R305	3D7	Pre-dose	13-SEP-2018	09:29	8	1	-0.10	0.00
		0.25 Hrs Post-dose	13-SEP-2018	09:50	8	1	0.25	46.50
		0.5 Hrs Post-dose	13-SEP-2018	10:05	8	1	0.50	341.10
		1 Hrs Post-dose	13-SEP-2018	10:35	8	1	1.00	249.40
		1.5 Hrs Post-dose	13-SEP-2018	11:05	8	1	1.50	90.70
		2 Hrs Post-dose	13-SEP-2018	11:35	8	1	2.00	34.30
		2.5 Hrs Post-dose	13-SEP-2018	12:05	8	1	2.50	28.20
		3 Hrs Post-dose	13-SEP-2018	12:35	8	1	3.00	6.17
		4 Hrs Post-dose	13-SEP-2018	13:35	8	1	4.00	0.20
		6 Hrs Post-dose	13-SEP-2018	15:35	8	1	6.00	0.00
		8 Hrs Post-dose	13-SEP-2018	17:35	8	1	8.00	0.00
		10 Hrs Post-dose	13-SEP-2018	19:38	8	1	10.05	0.00
		12 Hrs Post-dose	13-SEP-2018	21:37	8	1	12.03	0.00

Notes: Day relative to inoculum is the number of days relative to day of administration of the inoculum, where the day of administration = 0.
The inoculum was administered on Study Day 0 for K13-infected subjects and Study Day 1 for 3D7-infected subjects.
Day relative to IP is the number of days relative to day of administration of Artesunate, where the day of dose = 1.
Pre-dose = Pre Artesunate dose

Printed: 24FEB2020 (Final)

Listing 16.2.5.6
Plasma Pharmacokinetic Sample Collection Time And Concentration For Dihydroartemisinin

Randomisation Number	Challenge Agent	Scheduled Timepoint	Collection Date	Collection Time	Day Relative to Inoculum	Day Relative to IP	Actual Time Post Artesunate Dosing (hours)	Plasma Concentration (ug/L)
R306	3D7	Pre-dose	13-SEP-2018	09:41	8	1	-0.07	0.00
		0.25 Hrs Post-dose	13-SEP-2018	10:00	8	1	0.25	0.00
		0.5 Hrs Post-dose	13-SEP-2018	10:15	8	1	0.50	87.10
		1 Hrs Post-dose	13-SEP-2018	10:45	8	1	1.00	177.80
		1.5 Hrs Post-dose	13-SEP-2018	11:15	8	1	1.50	277.50
		2 Hrs Post-dose	13-SEP-2018	11:45	8	1	2.00	98.80
		2.5 Hrs Post-dose	13-SEP-2018	12:15	8	1	2.50	45.87
		3 Hrs Post-dose	13-SEP-2018	12:45	8	1	3.00	24.20
		4 Hrs Post-dose	13-SEP-2018	13:45	8	1	4.00	4.00
		6 Hrs Post-dose	13-SEP-2018	15:45	8	1	6.00	0.00
		8 Hrs Post-dose	13-SEP-2018	17:45	8	1	8.00	0.00
		10 Hrs Post-dose	13-SEP-2018	19:45	8	1	10.00	0.00
		12 Hrs Post-dose	13-SEP-2018	21:45	8	1	12.00	0.00

Notes: Day relative to inoculum is the number of days relative to day of administration of the inoculum, where the day of administration = 0.
The inoculum was administered on Study Day 0 for K13-infected subjects and Study Day 1 for 3D7-infected subjects.
Day relative to IP is the number of days relative to day of administration of Artesunate, where the day of dose = 1.
Pre-dose = Pre Artesunate dose

Listing 16.2.5.6
Plasma Pharmacokinetic Sample Collection Time And Concentration For Dihydroartemisinin

Randomisation Number	Challenge Agent	Scheduled Timepoint	Collection Date	Collection Time	Day Relative to Inoculum	Day Relative to IP	Actual Time Post Artesunate Dosing (hours)	Plasma Concentration (ug/L)
R307	3D7	Pre-dose	13-SEP-2018	09:51	8	1	-0.07	0.00
		0.25 Hrs Post-dose	13-SEP-2018	10:10	8	1	0.25	4.90
		0.5 Hrs Post-dose	13-SEP-2018	10:25	8	1	0.50	197.70
		1 Hrs Post-dose	13-SEP-2018	10:55	8	1	1.00	265.50
		1.5 Hrs Post-dose	13-SEP-2018	11:25	8	1	1.50	817.10
		2 Hrs Post-dose	13-SEP-2018	11:55	8	1	2.00	419.70
		2.5 Hrs Post-dose	13-SEP-2018	12:25	8	1	2.50	178.90
		3 Hrs Post-dose	13-SEP-2018	12:55	8	1	3.00	92.09
		4 Hrs Post-dose	13-SEP-2018	13:55	8	1	4.00	50.90
		6 Hrs Post-dose	13-SEP-2018	15:55	8	1	6.00	14.90
		8 Hrs Post-dose	13-SEP-2018	17:55	8	1	8.00	0.00
		10 Hrs Post-dose	13-SEP-2018	19:55	8	1	10.00	0.00
		12 Hrs Post-dose	13-SEP-2018	21:56	8	1	12.02	0.00

Notes: Day relative to inoculum is the number of days relative to day of administration of the inoculum, where the day of administration = 0.
The inoculum was administered on Study Day 0 for K13-infected subjects and Study Day 1 for 3D7-infected subjects.
Day relative to IP is the number of days relative to day of administration of Artesunate, where the day of dose = 1.
Pre-dose = Pre Artesunate dose

16.2.6 Parasitaemia and parasite lifecycle stage qPCR/qRT-PCR data

16.2.6.1 Feeding Assay

Listing 16.2.6.1
Feeding Assay

Randomisation Number	Challenge Agent	Was Feeding Done?	Feeding Date	Type of Feeding	Site of Feeding	Start Time	End Time	Day Relative to Inoculum	Day Relative to IP
R103	K13	Yes	07-DEC-2017	DFA	Right Forearm	7:27	7:42	23	15
R104	K13	Yes	07-DEC-2017	DFA	Left Forearm	7:27	7:43	23	15
R105	K13	Yes	07-DEC-2017	DFA	Left Forearm	7:27	7:43	23	15
R201	K13	Yes	14-MAR-2018	DFA	Right Forearm	8:07	8:22	23	15
		Yes	14-MAR-2018	MFA		9:06		23	15
R202	K13	Yes	14-MAR-2018	DFA	Left Forearm	8:06	8:21	23	15
		Yes	14-MAR-2018	MFA		8:51		23	15
R203	K13	Yes	14-MAR-2018	DFA	Right Forearm	9:25	9:40	23	15
		Yes	14-MAR-2018	MFA		10:15		23	15
R204	K13	Yes	14-MAR-2018	DFA	Left Forearm	9:25	9:40	23	15
		Yes	14-MAR-2018	MFA		9:59		23	15
R205	K13	Yes	14-MAR-2018	DFA	Left Forearm	8:07	8:22	23	15
		Yes	14-MAR-2018	MFA		8:46		23	15
R206	K13	Yes	14-MAR-2018	DFA	Left Forearm	9:25	9:40	23	15
		Yes	14-MAR-2018	MFA		10:05		23	15

Notes: Day relative to inoculum is the number of days relative to day of administration of the inoculum, where the day of administration = 0.
The inoculum was administered on Study Day 0 for K13-infected subjects and Study Day 1 for 3D7-infected subjects.
Day relative to IP is the number of days relative to day of administration of Artesunate, where the day of dose = 1.
MFA start time is the time of sample collection; DFA = Direct Feeding Assay; MFA = Membrane Feeding Assay.

Listing 16.2.6.1
Feeding Assay

Randomisation Number	Challenge Agent	Was Feeding Done?	Feeding Date	Type of Feeding	Site of Feeding	Start Time	End Time	Day Relative to Inoculum	Day Relative to IP
R207	K13	Yes	14-MAR-2018	DFA	Right Forearm	8:06	8:21	23	15
		Yes	14-MAR-2018	MFA		9:10		23	15
R302	K13	Yes	25-SEP-2018	DFA	Right Forearm	7:33	7:48	21	13
		Yes	25-SEP-2018	MFA		8:13		21	13
		Yes	28-SEP-2018	DFA	Left Forearm	7:35	7:50	24	16
		Yes	28-SEP-2018	MFA		8:22		24	16
R303	K13	Yes	25-SEP-2018	DFA	Right Forearm	7:33	7:48	21	13
		Yes	25-SEP-2018	MFA		8:20		21	13
		Yes	28-SEP-2018	DFA	Left Forearm	7:35	7:50	24	16
		Yes	28-SEP-2018	MFA		8:19		24	16
R304	K13	Yes	25-SEP-2018	DFA	Left Forearm	7:33	7:48	21	13
		Yes	25-SEP-2018	MFA		8:11		21	13
		Yes	28-SEP-2018	MFA		8:17		24	16
R305	3D7	Yes	25-SEP-2018	DFA	Left Forearm	7:33	7:48	20	13
		Yes	25-SEP-2018	MFA		8:19		20	13

Notes: Day relative to inoculum is the number of days relative to day of administration of the inoculum, where the day of administration = 0.
The inoculum was administered on Study Day 0 for K13-infected subjects and Study Day 1 for 3D7-infected subjects.
Day relative to IP is the number of days relative to day of administration of Artesunate, where the day of dose = 1.
MFA start time is the time of sample collection; DFA = Direct Feeding Assay; MFA = Membrane Feeding Assay.

16.2.6.3 Malaria 18S qPCR

Listing 16.2.6.3
Malaria 18S qPCR

Randomisation Number	Challenge Agent	Visit	Timepoint	Date of Measurement	Time of Measurement	Day Relative to Inoculum	Day Relative to Artesunate	Assay	Result (parasites/500µL) of packed RBCs
R103	K13	Inoculum		14-NOV-2017	10:04	0	-9	Replicate 1	ND
								Replicate 2	ND
								Replicate 3	ND
								Geometric Mean	1.0000
		Malaria Monitoring		18-NOV-2017	09:06	4	-5	Replicate 1	158
								Replicate 2	271
								Replicate 3	39
								Geometric Mean	118.6398
				19-NOV-2017	08:40	5	-4	Replicate 1	299
								Replicate 2	279
								Replicate 3	228
								Geometric Mean	266.9337
				20-NOV-2017	08:36	6	-3	Replicate 1	2126
								Replicate 2	2763
								Replicate 3	2708
								Geometric Mean	2514.9591

Notes: Day relative to inoculum is the number of days relative to day of administration of the inoculum, where the day of administration = 0.
The inoculum was administered on Study Day 0 for K13-infected subjects and Study Day 1 for 3D7-infected subjects.
Day relative to IP is the number of days relative to day of administration of Artesunate, where the day of dose = 1.
qPCR = quantitative Polymerase Chain Reaction; IP = Artesunate; ND = Not Detected; RBCs: red blood cells.

Listing 16.2.6.3
Malaria 18S qPCR

Randomisation Number	Challenge Agent	Visit	Timepoint	Date of Measurement	Time of Measurement	Day Relative to Inoculum	Day Relative to Artesunate	Assay	Result (parasites/500µL) of packed RBCs
R103	K13	Malaria Monitoring		20-NOV-2017	20:25	6	-3	Replicate 1	3057
								Replicate 2	3699
								Replicate 3	2129
								Geometric Mean	2887.4766
				21-NOV-2017	09:08	7	-2	Replicate 1	13020
								Replicate 2	13100
								Replicate 3	14251
								Geometric Mean	13445.4664
				22-NOV-2017	09:17	8	-1	Replicate 1	77292
								Replicate 2	74532
								Replicate 3	70090
								Geometric Mean	73911.3486
				22-NOV-2017	19:27	8	-1	Replicate 1	68944
								Replicate 2	73445
								Replicate 3	70678
								Geometric Mean	70998.2517

Notes: Day relative to inoculum is the number of days relative to day of administration of the inoculum, where the day of administration = 0.
The inoculum was administered on Study Day 0 for K13-infected subjects and Study Day 1 for 3D7-infected subjects.
Day relative to IP is the number of days relative to day of administration of Artesunate, where the day of dose = 1.
qPCR = quantitative Polymerase Chain Reaction; IP = Artesunate; ND = Not Detected; RBCs: red blood cells.

Listing 16.2.6.3
Malaria 18S qPCR

Randomisation Number	Challenge Agent	Visit	Timepoint	Date of Measurement	Time of Measurement	Day Relative to Inoculum	Day Relative to Artesunate	Assay	Result (parasites/500µL) of packed RBCs
R103	K13	Admission	Pre-dose	23-NOV-2017	09:50	9	1	Replicate 1	304117
								Replicate 2	290552
								Replicate 3	247273
								Geometric Mean	279563.4695
			2 Hrs Post-dose	23-NOV-2017	12:50	9	1	Replicate 1	431556
								Replicate 2	337367
								Replicate 3	471939
								Geometric Mean	409582.9554
			4 Hrs Post-dose	23-NOV-2017	14:50	9	1	Replicate 1	453339
								Replicate 2	423444
								Replicate 3	450888
								Geometric Mean	442346.4854
			6 Hrs Post-dose	23-NOV-2017	16:56	9	1	Replicate 1	543952
								Replicate 2	567807
								Replicate 3	503284
								Geometric Mean	537681.1542

Notes: Day relative to inoculum is the number of days relative to day of administration of the inoculum, where the day of administration = 0.
The inoculum was administered on Study Day 0 for K13-infected subjects and Study Day 1 for 3D7-infected subjects.
Day relative to IP is the number of days relative to day of administration of Artesunate, where the day of dose = 1.
qPCR = quantitative Polymerase Chain Reaction; IP = Artesunate; ND = Not Detected; RBCs: red blood cells.

Listing 16.2.6.3
Malaria 18S qPCR

Randomisation Number	Challenge Agent	Visit	Timepoint	Date of Measurement	Time of Measurement	Day Relative to Inoculum	Day Relative to Artesunate	Assay	Result (parasites/500µL) of packed RBCs
R103	K13	Admission	8 Hrs Post-dose	23-NOV-2017	18:50	9	1	Replicate 1	340122
								Replicate 2	339661
								Replicate 3	355680
								Geometric Mean	345074.8314
			12 Hrs Post-dose	23-NOV-2017	22:50	9	1	Replicate 1	332425
								Replicate 2	329286
								Replicate 3	304392
								Geometric Mean	321785.6894
		Admission 1	16 Hrs Post-dose	24-NOV-2017	02:50	10	2	Replicate 1	211251
								Replicate 2	197811
								Replicate 3	202696
								Geometric Mean	203844.0926
			20 Hrs Post-dose	24-NOV-2017	06:50	10	2	Replicate 1	158529
								Replicate 2	150095
								Replicate 3	163106
								Geometric Mean	157150.2082

Notes: Day relative to inoculum is the number of days relative to day of administration of the inoculum, where the day of administration = 0.
The inoculum was administered on Study Day 0 for K13-infected subjects and Study Day 1 for 3D7-infected subjects.
Day relative to IP is the number of days relative to day of administration of Artesunate, where the day of dose = 1.
qPCR = quantitative Polymerase Chain Reaction; IP = Artesunate; ND = Not Detected; RBCs: red blood cells.

Listing 16.2.6.3
Malaria 18S qPCR

Randomisation Number	Challenge Agent	Visit	Timepoint	Date of Measurement	Time of Measurement	Day Relative to Inoculum	Day Relative to Artesunate	Assay	Result (parasites/500µL) of packed RBCs
R103	K13	Admission 1	24 Hrs Post-dose	24-NOV-2017	10:51	10	2	Replicate 1	72163
								Replicate 2	73978
								Replicate 3	66197
								Geometric Mean	70699.8498
			28 Hrs Post-dose	24-NOV-2017	14:50	10	2	Replicate 1	33274
								Replicate 2	25912
								Replicate 3	30787
								Geometric Mean	29830.3104
			32 Hrs Post-dose	24-NOV-2017	18:52	10	2	Replicate 1	20330
								Replicate 2	19742
								Replicate 3	20349
								Geometric Mean	20138.3491
			36 Hrs Post-dose	24-NOV-2017	22:51	10	2	Replicate 1	8672
								Replicate 2	9341
								Replicate 3	10564
								Geometric Mean	9493.9516

Notes: Day relative to inoculum is the number of days relative to day of administration of the inoculum, where the day of administration = 0.
The inoculum was administered on Study Day 0 for K13-infected subjects and Study Day 1 for 3D7-infected subjects.
Day relative to IP is the number of days relative to day of administration of Artesunate, where the day of dose = 1.
qPCR = quantitative Polymerase Chain Reaction; IP = Artesunate; ND = Not Detected; RBCs: red blood cells.

Listing 16.2.6.3
Malaria 18S qPCR

Randomisation Number	Challenge Agent	Visit	Timepoint	Date of Measurement	Time of Measurement	Day Relative to Inoculum	Day Relative to Artesunate	Assay	Result (parasites/500µL) of packed RBCs
R103	K13	Admission 2	48 Hrs Post-dose	25-NOV-2017	10:50	11	3	Replicate 1	118188
								Replicate 2	142174
								Replicate 3	140896
								Geometric Mean	133279.7215
				25-NOV-2017	17:52	11	3	Replicate 1	1489948
								Replicate 2	1469890
								Replicate 3	1490958
								Geometric Mean	1483566.8442
			60 Hrs Post-dose	25-NOV-2017	22:50	11	3	Replicate 1	1036143
								Replicate 2	1352641
								Replicate 3	1325718
								Geometric Mean	1229375.2507
		Admission 3	72 Hrs Post-dose	26-NOV-2017	10:53	12	4	Replicate 1	187147
								Replicate 2	178516
								Replicate 3	179122
								Geometric Mean	181552.8129

Notes: Day relative to inoculum is the number of days relative to day of administration of the inoculum, where the day of administration = 0.
The inoculum was administered on Study Day 0 for K13-infected subjects and Study Day 1 for 3D7-infected subjects.
Day relative to IP is the number of days relative to day of administration of Artesunate, where the day of dose = 1.
qPCR = quantitative Polymerase Chain Reaction; IP = Artesunate; ND = Not Detected; RBCs: red blood cells.

Listing 16.2.6.3
Malaria 18S qPCR

Randomisation Number	Challenge Agent	Visit	Timepoint	Date of Measurement	Time of Measurement	Day Relative to Inoculum	Day Relative to Artesunate	Assay	Result (parasites/500µL) of packed RBCs
R103	K13	Admission 3		26-NOV-2017	19:28	12	4	Replicate 1	164760
								Replicate 2	171055
								Replicate 3	173625
								Geometric Mean	169772.2112
		Safety Monitoring		27-NOV-2017	11:01	13	5	Replicate 1	18327
								Replicate 2	18485
								Replicate 3	18493
								Geometric Mean	18434.8412
				27-NOV-2017	19:26	13	5	Replicate 1	5098
								Replicate 2	4924
								Replicate 3	5119
								Geometric Mean	5046.2373
				28-NOV-2017	09:25	14	6	Replicate 1	2525
								Replicate 2	2149
								Replicate 3	2835
								Geometric Mean	2487.0449

Notes: Day relative to inoculum is the number of days relative to day of administration of the inoculum, where the day of administration = 0.
The inoculum was administered on Study Day 0 for K13-infected subjects and Study Day 1 for 3D7-infected subjects.
Day relative to IP is the number of days relative to day of administration of Artesunate, where the day of dose = 1.
qPCR = quantitative Polymerase Chain Reaction; IP = Artesunate; ND = Not Detected; RBCs: red blood cells.

Listing 16.2.6.3
Malaria 18S qPCR

Randomisation Number	Challenge Agent	Visit	Timepoint	Date of Measurement	Time of Measurement	Day Relative to Inoculum	Day Relative to Artesunate	Assay	Result (parasites/500µL) of packed RBCs
R103	K13	Safety Monitoring		28-NOV-2017	20:19	14	6	Replicate 1	548
								Replicate 2	408
								Replicate 3	739
								Geometric Mean	548.7338
				29-NOV-2017	06:58	15	7	Replicate 1	322
								Replicate 2	350
								Replicate 3	115
								Geometric Mean	234.8951
				30-NOV-2017	10:09	16	8	Replicate 1	197
								Replicate 2	ND
								Replicate 3	392
								Geometric Mean	42.5844
				01-DEC-2017	10:41	17	9	Replicate 1	1146
								Replicate 2	1122
								Replicate 3	1150
								Geometric Mean	1139.2659

Notes: Day relative to inoculum is the number of days relative to day of administration of the inoculum, where the day of administration = 0.
The inoculum was administered on Study Day 0 for K13-infected subjects and Study Day 1 for 3D7-infected subjects.
Day relative to IP is the number of days relative to day of administration of Artesunate, where the day of dose = 1.
qPCR = quantitative Polymerase Chain Reaction; IP = Artesunate; ND = Not Detected; RBCs: red blood cells.

Listing 16.2.6.3
Malaria 18S qPCR

Randomisation Number	Challenge Agent	Visit	Timepoint	Date of Measurement	Time of Measurement	Day Relative to Inoculum	Day Relative to Artesunate	Assay	Result (parasites/500µL) of packed RBCs
R103	K13	Safety Monitoring		03-DEC-2017	10:45	19	11	Replicate 1	9475
								Replicate 2	8596
								Replicate 3	8478
								Geometric Mean	8838.7262
				04-DEC-2017	09:27	20	12	Replicate 1	30583
								Replicate 2	35912
								Replicate 3	31183
								Geometric Mean	32474.7531
				05-DEC-2017	08:07	21	13	Replicate 1	13797
								Replicate 2	14792
								Replicate 3	14668
								Geometric Mean	14412.1073
				07-DEC-2017	08:41	23	15	Replicate 1	11089
								Replicate 2	9952
								Replicate 3	8634
								Geometric Mean	9840.2228

Notes: Day relative to inoculum is the number of days relative to day of administration of the inoculum, where the day of administration = 0.
The inoculum was administered on Study Day 0 for K13-infected subjects and Study Day 1 for 3D7-infected subjects.
Day relative to IP is the number of days relative to day of administration of Artesunate, where the day of dose = 1.
qPCR = quantitative Polymerase Chain Reaction; IP = Artesunate; ND = Not Detected; RBCs: red blood cells.

Listing 16.2.6.3
Malaria 18S qPCR

Randomisation Number	Challenge Agent	Visit	Timepoint	Date of Measurement	Time of Measurement	Day Relative to Inoculum	Day Relative to Artesunate	Assay	Result (parasites/500µL) of packed RBCs
R103	K13	Safety Monitoring		11-DEC-2017	10:17	27	19	Replicate 1	4263
								Replicate 2	3934
								Replicate 3	4379
								Geometric Mean	4187.6934
				12-DEC-2017	09:30	28	20	Replicate 1	1144
								Replicate 2	1440
								Replicate 3	1651
								Geometric Mean	1395.8707
		End of Study		14-DEC-2017	09:37	30	22	Replicate 1	455
								Replicate 2	325
								Replicate 3	379
								Geometric Mean	382.6878
		Unscheduled		18-DEC-2017	07:20	34	26	Replicate 1	152
								Replicate 2	133
								Replicate 3	140
								Geometric Mean	141.4516

Notes: Day relative to inoculum is the number of days relative to day of administration of the inoculum, where the day of administration = 0.
The inoculum was administered on Study Day 0 for K13-infected subjects and Study Day 1 for 3D7-infected subjects.
Day relative to IP is the number of days relative to day of administration of Artesunate, where the day of dose = 1.
qPCR = quantitative Polymerase Chain Reaction; IP = Artesunate; ND = Not Detected; RBCs: red blood cells.

Listing 16.2.6.3
Malaria 18S qPCR

Randomisation Number	Challenge Agent	Visit	Timepoint	Date of Measurement	Time of Measurement	Day Relative to Inoculum	Day Relative to Artesunate	Assay	Result (parasites/500µL) of packed RBCs
R103	K13	Unscheduled		22-DEC-2017	08:19	38	30	Replicate 1	ND
								Replicate 2	55
								Replicate 3	ND
								Geometric Mean	3.8030
				27-DEC-2017	10:14	43	35	Replicate 1	ND
								Replicate 2	ND
								Replicate 3	ND
								Geometric Mean	1.0000
R104	K13	Inoculum		14-NOV-2017	10:11	0	-9	Replicate 1	ND
								Replicate 2	ND
								Replicate 3	ND
								Geometric Mean	1.0000
		Malaria Monitoring		18-NOV-2017	09:12	4	-5	Replicate 1	92
								Replicate 2	33
								Replicate 3	89
								Geometric Mean	64.6493

Notes: Day relative to inoculum is the number of days relative to day of administration of the inoculum, where the day of administration = 0.
The inoculum was administered on Study Day 0 for K13-infected subjects and Study Day 1 for 3D7-infected subjects.
Day relative to IP is the number of days relative to day of administration of Artesunate, where the day of dose = 1.
qPCR = quantitative Polymerase Chain Reaction; IP = Artesunate; ND = Not Detected; RBCs: red blood cells.

Listing 16.2.6.3
Malaria 18S qPCR

Randomisation Number	Challenge Agent	Visit	Timepoint	Date of Measurement	Time of Measurement	Day Relative to Inoculum	Day Relative to Artesunate	Assay	Result (parasites/500µL) of packed RBCs
R104	K13	Malaria Monitoring		19-NOV-2017	09:15	5	-4	Replicate 1	116
								Replicate 2	44
								Replicate 3	148
								Geometric Mean	91.0732
				20-NOV-2017	07:25	6	-3	Replicate 1	1701
								Replicate 2	1813
								Replicate 3	1598
								Geometric Mean	1701.7390
				20-NOV-2017	20:05	6	-3	Replicate 1	2217
								Replicate 2	1965
								Replicate 3	2243
								Geometric Mean	2137.8924
				21-NOV-2017	08:37	7	-2	Replicate 1	6417
								Replicate 2	6726
								Replicate 3	7524
								Geometric Mean	6873.5205

Notes: Day relative to inoculum is the number of days relative to day of administration of the inoculum, where the day of administration = 0.
The inoculum was administered on Study Day 0 for K13-infected subjects and Study Day 1 for 3D7-infected subjects.
Day relative to IP is the number of days relative to day of administration of Artesunate, where the day of dose = 1.
qPCR = quantitative Polymerase Chain Reaction; IP = Artesunate; ND = Not Detected; RBCs: red blood cells.

Listing 16.2.6.3
Malaria 18S qPCR

Randomisation Number	Challenge Agent	Visit	Timepoint	Date of Measurement	Time of Measurement	Day Relative to Inoculum	Day Relative to Artesunate	Assay	Result (parasites/500µL) of packed RBCs
R104	K13	Malaria Monitoring		21-NOV-2017	19:49	7	-2	Replicate 1	40519
								Replicate 2	40172
								Replicate 3	40887
								Geometric Mean	40524.9486
				22-NOV-2017	07:48	8	-1	Replicate 1	40236
								Replicate 2	44996
								Replicate 3	41660
								Geometric Mean	42250.9026
				22-NOV-2017	20:12	8	-1	Replicate 1	46139
								Replicate 2	55805
								Replicate 3	42382
								Geometric Mean	47786.7593
		Admission	Pre-dose	23-NOV-2017	09:44	9	1	Replicate 1	174503
								Replicate 2	148476
								Replicate 3	158672
								Geometric Mean	160196.5470

Notes: Day relative to inoculum is the number of days relative to day of administration of the inoculum, where the day of administration = 0.
The inoculum was administered on Study Day 0 for K13-infected subjects and Study Day 1 for 3D7-infected subjects.
Day relative to IP is the number of days relative to day of administration of Artesunate, where the day of dose = 1.
qPCR = quantitative Polymerase Chain Reaction; IP = Artesunate; ND = Not Detected; RBCs: red blood cells.

Listing 16.2.6.3
Malaria 18S qPCR

Randomisation Number	Challenge Agent	Visit	Timepoint	Date of Measurement	Time of Measurement	Day Relative to Inoculum	Day Relative to Artesunate	Assay	Result (parasites/500µL) of packed RBCs
R104	K13	Admission	2 Hrs Post-dose	23-NOV-2017	12:40	9	1	Replicate 1	263855
								Replicate 2	232607
								Replicate 3	261482
								Geometric Mean	252237.6833
			4 Hrs Post-dose	23-NOV-2017	14:40	9	1	Replicate 1	292351
								Replicate 2	239973
								Replicate 3	245677
								Geometric Mean	258311.5477
			6 Hrs Post-dose	23-NOV-2017	16:41	9	1	Replicate 1	170156
								Replicate 2	175385
								Replicate 3	166130
								Geometric Mean	170515.0015
			8 Hrs Post-dose	23-NOV-2017	18:40	9	1	Replicate 1	200344
								Replicate 2	171700
								Replicate 3	198049
								Geometric Mean	189571.5218

Notes: Day relative to inoculum is the number of days relative to day of administration of the inoculum, where the day of administration = 0.
The inoculum was administered on Study Day 0 for K13-infected subjects and Study Day 1 for 3D7-infected subjects.
Day relative to IP is the number of days relative to day of administration of Artesunate, where the day of dose = 1.
qPCR = quantitative Polymerase Chain Reaction; IP = Artesunate; ND = Not Detected; RBCs: red blood cells.

Listing 16.2.6.3
Malaria 18S qPCR

Randomisation Number	Challenge Agent	Visit	Timepoint	Date of Measurement	Time of Measurement	Day Relative to Inoculum	Day Relative to Artesunate	Assay	Result (parasites/500µL) of packed RBCs
R104	K13	Admission	12 Hrs Post-dose	23-NOV-2017	22:40	9	1	Replicate 1	147740
								Replicate 2	142142
								Replicate 3	160547
								Geometric Mean	149948.0781
		Admission 1	16 Hrs Post-dose	24-NOV-2017	02:40	10	2	Replicate 1	143335
								Replicate 2	135037
								Replicate 3	151048
								Geometric Mean	142990.3306
			20 Hrs Post-dose	24-NOV-2017	06:40	10	2	Replicate 1	98426
								Replicate 2	93400
								Replicate 3	105852
								Geometric Mean	99095.0422
			24 Hrs Post-dose	24-NOV-2017	10:40	10	2	Replicate 1	36528
								Replicate 2	39964
								Replicate 3	34320
								Geometric Mean	36864.9833

Notes: Day relative to inoculum is the number of days relative to day of administration of the inoculum, where the day of administration = 0.
The inoculum was administered on Study Day 0 for K13-infected subjects and Study Day 1 for 3D7-infected subjects.
Day relative to IP is the number of days relative to day of administration of Artesunate, where the day of dose = 1.
qPCR = quantitative Polymerase Chain Reaction; IP = Artesunate; ND = Not Detected; RBCs: red blood cells.

Listing 16.2.6.3
Malaria 18S qPCR

Randomisation Number	Challenge Agent	Visit	Timepoint	Date of Measurement	Time of Measurement	Day Relative to Inoculum	Day Relative to Artesunate	Assay	Result (parasites/500µL) of packed RBCs
R104	K13	Admission 1	28 Hrs Post-dose	24-NOV-2017	14:41	10	2	Replicate 1	17906
								Replicate 2	19631
								Replicate 3	19015
								Geometric Mean	18837.0306
			32 Hrs Post-dose	24-NOV-2017	18:40	10	2	Replicate 1	21948
								Replicate 2	21580
								Replicate 3	22751
								Geometric Mean	22087.6220
			36 Hrs Post-dose	24-NOV-2017	22:40	10	2	Replicate 1	12329
								Replicate 2	11602
								Replicate 3	10111
								Geometric Mean	11308.8456
		Admission 2	48 Hrs Post-dose	25-NOV-2017	10:41	11	3	Replicate 1	48086
								Replicate 2	48852
								Replicate 3	60724
								Geometric Mean	52250.0998

Notes: Day relative to inoculum is the number of days relative to day of administration of the inoculum, where the day of administration = 0.
The inoculum was administered on Study Day 0 for K13-infected subjects and Study Day 1 for 3D7-infected subjects.
Day relative to IP is the number of days relative to day of administration of Artesunate, where the day of dose = 1.
qPCR = quantitative Polymerase Chain Reaction; IP = Artesunate; ND = Not Detected; RBCs: red blood cells.

Listing 16.2.6.3
Malaria 18S qPCR

Randomisation Number	Challenge Agent	Visit	Timepoint	Date of Measurement	Time of Measurement	Day Relative to Inoculum	Day Relative to Artesunate	Assay	Result (parasites/500µL) of packed RBCs
R104	K13	Admission 2		25-NOV-2017	17:43	11	3	Replicate 1	729400
								Replicate 2	704939
								Replicate 3	784432
								Geometric Mean	738852.1357
			60 Hrs Post-dose	25-NOV-2017	22:40	11	3	Replicate 1	647098
								Replicate 2	653857
								Replicate 3	571883
								Geometric Mean	623141.4979
		Admission 3	72 Hrs Post-dose	26-NOV-2017	10:41	12	4	Replicate 1	277696
								Replicate 2	311534
								Replicate 3	271494
								Geometric Mean	286381.5952
				26-NOV-2017	19:28	12	4	Replicate 1	91369
								Replicate 2	94518
								Replicate 3	91410
								Geometric Mean	92420.6534

Notes: Day relative to inoculum is the number of days relative to day of administration of the inoculum, where the day of administration = 0.
The inoculum was administered on Study Day 0 for K13-infected subjects and Study Day 1 for 3D7-infected subjects.
Day relative to IP is the number of days relative to day of administration of Artesunate, where the day of dose = 1.
qPCR = quantitative Polymerase Chain Reaction; IP = Artesunate; ND = Not Detected; RBCs: red blood cells.

Listing 16.2.6.3
Malaria 18S qPCR

Randomisation Number	Challenge Agent	Visit	Timepoint	Date of Measurement	Time of Measurement	Day Relative to Inoculum	Day Relative to Artesunate	Assay	Result (parasites/500µL) of packed RBCs
R104	K13	Safety Monitoring		27-NOV-2017	08:18	13	5	Replicate 1	19200
								Replicate 2	15874
								Replicate 3	14975
								Geometric Mean	16587.6480
				27-NOV-2017	19:34	13	5	Replicate 1	8976
								Replicate 2	7119
								Replicate 3	9033
								Geometric Mean	8326.1574
				28-NOV-2017	08:13	14	6	Replicate 1	3009
								Replicate 2	2946
								Replicate 3	2792
								Geometric Mean	2914.2275
				28-NOV-2017	19:05	14	6	Replicate 1	739
								Replicate 2	1006
								Replicate 3	647
								Geometric Mean	783.5179

Notes: Day relative to inoculum is the number of days relative to day of administration of the inoculum, where the day of administration = 0.
The inoculum was administered on Study Day 0 for K13-infected subjects and Study Day 1 for 3D7-infected subjects.
Day relative to IP is the number of days relative to day of administration of Artesunate, where the day of dose = 1.
qPCR = quantitative Polymerase Chain Reaction; IP = Artesunate; ND = Not Detected; RBCs: red blood cells.

Listing 16.2.6.3
Malaria 18S qPCR

Randomisation Number	Challenge Agent	Visit	Timepoint	Date of Measurement	Time of Measurement	Day Relative to Inoculum	Day Relative to Artesunate	Assay	Result (parasites/500µL) of packed RBCs
R104	K13	Safety Monitoring		29-NOV-2017	08:39	15	7	Replicate 1	341
								Replicate 2	378
								Replicate 3	391
								Geometric Mean	369.3808
				30-NOV-2017	08:43	16	8	Replicate 1	240
								Replicate 2	349
								Replicate 3	65
								Geometric Mean	175.9206
				01-DEC-2017	08:25	17	9	Replicate 1	873
								Replicate 2	847
								Replicate 3	585
								Geometric Mean	756.2833
				03-DEC-2017	09:13	19	11	Replicate 1	18816
								Replicate 2	21334
								Replicate 3	18672
								Geometric Mean	19570.2697

Notes: Day relative to inoculum is the number of days relative to day of administration of the inoculum, where the day of administration = 0.
The inoculum was administered on Study Day 0 for K13-infected subjects and Study Day 1 for 3D7-infected subjects.
Day relative to IP is the number of days relative to day of administration of Artesunate, where the day of dose = 1.
qPCR = quantitative Polymerase Chain Reaction; IP = Artesunate; ND = Not Detected; RBCs: red blood cells.

Listing 16.2.6.3
Malaria 18S qPCR

Randomisation Number	Challenge Agent	Visit	Timepoint	Date of Measurement	Time of Measurement	Day Relative to Inoculum	Day Relative to Artesunate	Assay	Result (parasites/500µL) of packed RBCs
R104	K13	Safety Monitoring		04-DEC-2017	08:05	20	12	Replicate 1	29752
								Replicate 2	29833
								Replicate 3	29232
								Geometric Mean	29604.4644
				04-DEC-2017	12:45	20	12	Replicate 1	24902
								Replicate 2	23350
								Replicate 3	24368
								Geometric Mean	24198.0480
				05-DEC-2017	08:30	21	13	Replicate 1	26600
								Replicate 2	23910
								Replicate 3	25650
								Geometric Mean	25361.9628
				07-DEC-2017	08:35	23	15	Replicate 1	28650
								Replicate 2	25498
								Replicate 3	24693
								Geometric Mean	26226.1697

Notes: Day relative to inoculum is the number of days relative to day of administration of the inoculum, where the day of administration = 0.
The inoculum was administered on Study Day 0 for K13-infected subjects and Study Day 1 for 3D7-infected subjects.
Day relative to IP is the number of days relative to day of administration of Artesunate, where the day of dose = 1.
qPCR = quantitative Polymerase Chain Reaction; IP = Artesunate; ND = Not Detected; RBCs: red blood cells.

Listing 16.2.6.3
Malaria 18S qPCR

Randomisation Number	Challenge Agent	Visit	Timepoint	Date of Measurement	Time of Measurement	Day Relative to Inoculum	Day Relative to Artesunate	Assay	Result (parasites/500µL) of packed RBCs
R104	K13	Safety Monitoring		08-DEC-2017	08:12	24	16	Replicate 1	11407
								Replicate 2	11891
								Replicate 3	10188
								Geometric Mean	11138.4883
				10-DEC-2017	10:33	26	18	Replicate 1	16851
								Replicate 2	17538
								Replicate 3	16620
								Geometric Mean	16998.5648
				11-DEC-2017	08:01	27	19	Replicate 1	4571
								Replicate 2	4554
								Replicate 3	4765
								Geometric Mean	4629.0199
				12-DEC-2017	07:57	28	20	Replicate 1	5111
								Replicate 2	4480
								Replicate 3	4473
								Geometric Mean	4678.7258

Notes: Day relative to inoculum is the number of days relative to day of administration of the inoculum, where the day of administration = 0.
The inoculum was administered on Study Day 0 for K13-infected subjects and Study Day 1 for 3D7-infected subjects.
Day relative to IP is the number of days relative to day of administration of Artesunate, where the day of dose = 1.
qPCR = quantitative Polymerase Chain Reaction; IP = Artesunate; ND = Not Detected; RBCs: red blood cells.

Listing 16.2.6.3
Malaria 18S qPCR

Randomisation Number	Challenge Agent	Visit	Timepoint	Date of Measurement	Time of Measurement	Day Relative to Inoculum	Day Relative to Artesunate	Assay	Result (parasites/500µL) of packed RBCs
R104	K13	End of Study		14-DEC-2017	08:42	30	22	Replicate 1	2087
								Replicate 2	2417
								Replicate 3	2674
								Geometric Mean	2380.4195
		Unscheduled		18-DEC-2017	08:14	34	26	Replicate 1	303
								Replicate 2	199
								Replicate 3	284
								Geometric Mean	257.7536
				21-DEC-2017	07:35	37	29	Replicate 1	68
								Replicate 2	41
								Replicate 3	65
								Geometric Mean	56.5894
				29-DEC-2017	10:15	45	37	Replicate 1	ND
								Replicate 2	ND
								Replicate 3	ND
								Geometric Mean	1.0000

Notes: Day relative to inoculum is the number of days relative to day of administration of the inoculum, where the day of administration = 0.
The inoculum was administered on Study Day 0 for K13-infected subjects and Study Day 1 for 3D7-infected subjects.
Day relative to IP is the number of days relative to day of administration of Artesunate, where the day of dose = 1.
qPCR = quantitative Polymerase Chain Reaction; IP = Artesunate; ND = Not Detected; RBCs: red blood cells.

Listing 16.2.6.3
Malaria 18S qPCR

Randomisation Number	Challenge Agent	Visit	Timepoint	Date of Measurement	Time of Measurement	Day Relative to Inoculum	Day Relative to Artesunate	Assay	Result (parasites/500µL) of packed RBCs
R105	K13	Inoculum		14-NOV-2017	09:53	0	-9	Replicate 1	ND
								Replicate 2	ND
								Replicate 3	ND
								Geometric Mean	1.0000
		Malaria Monitoring		18-NOV-2017	08:51	4	-5	Replicate 1	81
								Replicate 2	72
								Replicate 3	75
								Geometric Mean	75.9089
				19-NOV-2017	08:05	5	-4	Replicate 1	239
								Replicate 2	107
								Replicate 3	22
								Geometric Mean	82.5534
				20-NOV-2017	08:54	6	-3	Replicate 1	1254
								Replicate 2	1168
								Replicate 3	1359
								Geometric Mean	1257.9207

Notes: Day relative to inoculum is the number of days relative to day of administration of the inoculum, where the day of administration = 0.
The inoculum was administered on Study Day 0 for K13-infected subjects and Study Day 1 for 3D7-infected subjects.
Day relative to IP is the number of days relative to day of administration of Artesunate, where the day of dose = 1.
qPCR = quantitative Polymerase Chain Reaction; IP = Artesunate; ND = Not Detected; RBCs: red blood cells.

Listing 16.2.6.3
Malaria 18S qPCR

Randomisation Number	Challenge Agent	Visit	Timepoint	Date of Measurement	Time of Measurement	Day Relative to Inoculum	Day Relative to Artesunate	Assay	Result (parasites/500µL) of packed RBCs
R105	K13	Malaria Monitoring		20-NOV-2017	19:28	6	-3	Replicate 1	1197
								Replicate 2	1172
								Replicate 3	847
								Geometric Mean	1059.1766
				21-NOV-2017	08:07	7	-2	Replicate 1	2812
								Replicate 2	3001
								Replicate 3	3899
								Geometric Mean	3204.3862
				21-NOV-2017	19:29	7	-2	Replicate 1	20138
								Replicate 2	18641
								Replicate 3	18282
								Geometric Mean	19003.6234
				22-NOV-2017	08:11	8	-1	Replicate 1	18415
								Replicate 2	18073
								Replicate 3	19380
								Geometric Mean	18614.5231

Notes: Day relative to inoculum is the number of days relative to day of administration of the inoculum, where the day of administration = 0.
The inoculum was administered on Study Day 0 for K13-infected subjects and Study Day 1 for 3D7-infected subjects.
Day relative to IP is the number of days relative to day of administration of Artesunate, where the day of dose = 1.
qPCR = quantitative Polymerase Chain Reaction; IP = Artesunate; ND = Not Detected; RBCs: red blood cells.

Listing 16.2.6.3
Malaria 18S qPCR

Randomisation Number	Challenge Agent	Visit	Timepoint	Date of Measurement	Time of Measurement	Day Relative to Inoculum	Day Relative to Artesunate	Assay	Result (parasites/500µL) of packed RBCs
R105	K13	Malaria Monitoring		22-NOV-2017	19:19	8	-1	Replicate 1	18878
								Replicate 2	17996
								Replicate 3	19600
								Geometric Mean	18813.2003
		Admission	Pre-dose	23-NOV-2017	09:36	9	1	Replicate 1	57741
								Replicate 2	70710
								Replicate 3	63301
								Geometric Mean	63697.9178
			2 Hrs Post-dose	23-NOV-2017	12:31	9	1	Replicate 1	86639
								Replicate 2	109236
								Replicate 3	102541
								Geometric Mean	99005.4091
			4 Hrs Post-dose	23-NOV-2017	14:30	9	1	Replicate 1	117584
								Replicate 2	104931
								Replicate 3	103799
								Geometric Mean	108596.3105

Notes: Day relative to inoculum is the number of days relative to day of administration of the inoculum, where the day of administration = 0.
The inoculum was administered on Study Day 0 for K13-infected subjects and Study Day 1 for 3D7-infected subjects.
Day relative to IP is the number of days relative to day of administration of Artesunate, where the day of dose = 1.
qPCR = quantitative Polymerase Chain Reaction; IP = Artesunate; ND = Not Detected; RBCs: red blood cells.

Listing 16.2.6.3
Malaria 18S qPCR

Randomisation Number	Challenge Agent	Visit	Timepoint	Date of Measurement	Time of Measurement	Day Relative to Inoculum	Day Relative to Artesunate	Assay	Result (parasites/500µL) of packed RBCs
R105	K13	Admission	6 Hrs Post-dose	23-NOV-2017	16:31	9	1	Replicate 1	109979
								Replicate 2	115530
								Replicate 3	126028
								Geometric Mean	116992.2773
			8 Hrs Post-dose	23-NOV-2017	18:30	9	1	Replicate 1	105860
								Replicate 2	106700
								Replicate 3	101252
								Geometric Mean	104576.3083
			12 Hrs Post-dose	23-NOV-2017	22:31	9	1	Replicate 1	97628
								Replicate 2	105660
								Replicate 3	98070
								Geometric Mean	100386.1329
		Admission 1	16 Hrs Post-dose	24-NOV-2017	02:30	10	2	Replicate 1	52526
								Replicate 2	52812
								Replicate 3	52042
								Geometric Mean	52459.0362

Notes: Day relative to inoculum is the number of days relative to day of administration of the inoculum, where the day of administration = 0.
The inoculum was administered on Study Day 0 for K13-infected subjects and Study Day 1 for 3D7-infected subjects.
Day relative to IP is the number of days relative to day of administration of Artesunate, where the day of dose = 1.
qPCR = quantitative Polymerase Chain Reaction; IP = Artesunate; ND = Not Detected; RBCs: red blood cells.

Listing 16.2.6.3
Malaria 18S qPCR

Randomisation Number	Challenge Agent	Visit	Timepoint	Date of Measurement	Time of Measurement	Day Relative to Inoculum	Day Relative to Artesunate	Assay	Result (parasites/500μL) of packed RBCs
R105	K13	Admission 1	20 Hrs Post-dose	24-NOV-2017	06:30	10	2	Replicate 1	23434
								Replicate 2	24264
								Replicate 3	25357
								Geometric Mean	24338.9720
			24 Hrs Post-dose	24-NOV-2017	10:30	10	2	Replicate 1	23835
								Replicate 2	18810
								Replicate 3	16613
								Geometric Mean	19529.1799
			28 Hrs Post-dose	24-NOV-2017	14:30	10	2	Replicate 1	5827
								Replicate 2	6381
								Replicate 3	5452
								Geometric Mean	5874.3958
			32 Hrs Post-dose	24-NOV-2017	18:31	10	2	Replicate 1	6000
								Replicate 2	5430
								Replicate 3	6733
								Geometric Mean	6030.9616

Notes: Day relative to inoculum is the number of days relative to day of administration of the inoculum, where the day of administration = 0.
The inoculum was administered on Study Day 0 for K13-infected subjects and Study Day 1 for 3D7-infected subjects.
Day relative to IP is the number of days relative to day of administration of Artesunate, where the day of dose = 1.
qPCR = quantitative Polymerase Chain Reaction; IP = Artesunate; ND = Not Detected; RBCs: red blood cells.

Listing 16.2.6.3
Malaria 18S qPCR

Randomisation Number	Challenge Agent	Visit	Timepoint	Date of Measurement	Time of Measurement	Day Relative to Inoculum	Day Relative to Artesunate	Assay	Result (parasites/500µL) of packed RBCs
R105	K13	Admission 1	36 Hrs Post-dose	24-NOV-2017	22:30	10	2	Replicate 1	3444
								Replicate 2	2634
								Replicate 3	2855
								Geometric Mean	2958.6596
		Admission 2	48 Hrs Post-dose	25-NOV-2017	10:30	11	3	Replicate 1	13642
								Replicate 2	12783
								Replicate 3	14792
								Geometric Mean	13714.4768
				25-NOV-2017	17:42	11	3	Replicate 1	174963
								Replicate 2	172102
								Replicate 3	174529
								Geometric Mean	173860.0945
			60 Hrs Post-dose	25-NOV-2017	22:30	11	3	Replicate 1	196815
								Replicate 2	190301
								Replicate 3	202498
								Geometric Mean	196474.7151

Notes: Day relative to inoculum is the number of days relative to day of administration of the inoculum, where the day of administration = 0.
The inoculum was administered on Study Day 0 for K13-infected subjects and Study Day 1 for 3D7-infected subjects.
Day relative to IP is the number of days relative to day of administration of Artesunate, where the day of dose = 1.
qPCR = quantitative Polymerase Chain Reaction; IP = Artesunate; ND = Not Detected; RBCs: red blood cells.

Listing 16.2.6.3
Malaria 18S qPCR

Randomisation Number	Challenge Agent	Visit	Timepoint	Date of Measurement	Time of Measurement	Day Relative to Inoculum	Day Relative to Artesunate	Assay	Result (parasites/500µL) of packed RBCs
R105	K13	Admission 3	72 Hrs Post-dose	26-NOV-2017	10:31	12	4	Replicate 1	69110
								Replicate 2	69864
								Replicate 3	71702
								Geometric Mean	70216.9353
				26-NOV-2017	19:05	12	4	Replicate 1	32794
								Replicate 2	30002
								Replicate 3	32735
								Geometric Mean	31816.4935
		Safety Monitoring		27-NOV-2017	07:22	13	5	Replicate 1	6777
								Replicate 2	6527
								Replicate 3	8053
								Geometric Mean	7088.7490
				27-NOV-2017	19:05	13	5	Replicate 1	9289
								Replicate 2	10623
								Replicate 3	9514
								Geometric Mean	9791.7375

Notes: Day relative to inoculum is the number of days relative to day of administration of the inoculum, where the day of administration = 0.
The inoculum was administered on Study Day 0 for K13-infected subjects and Study Day 1 for 3D7-infected subjects.
Day relative to IP is the number of days relative to day of administration of Artesunate, where the day of dose = 1.
qPCR = quantitative Polymerase Chain Reaction; IP = Artesunate; ND = Not Detected; RBCs: red blood cells.

Listing 16.2.6.3
Malaria 18S qPCR

Randomisation Number	Challenge Agent	Visit	Timepoint	Date of Measurement	Time of Measurement	Day Relative to Inoculum	Day Relative to Artesunate	Assay	Result (parasites/500µL) of packed RBCs
R105	K13	Safety Monitoring		28-NOV-2017	07:34	14	6	Replicate 1	3641
								Replicate 2	3755
								Replicate 3	4591
								Geometric Mean	3974.1657
				28-NOV-2017	19:07	14	6	Replicate 1	1039
								Replicate 2	1145
								Replicate 3	1017
								Geometric Mean	1065.5668
				29-NOV-2017	10:55	15	7	Replicate 1	17237
								Replicate 2	19927
								Replicate 3	16875
								Geometric Mean	17963.1485
				30-NOV-2017	07:08	16	8	Replicate 1	6130
								Replicate 2	5942
								Replicate 3	7068
								Geometric Mean	6361.5540

Notes: Day relative to inoculum is the number of days relative to day of administration of the inoculum, where the day of administration = 0.
The inoculum was administered on Study Day 0 for K13-infected subjects and Study Day 1 for 3D7-infected subjects.
Day relative to IP is the number of days relative to day of administration of Artesunate, where the day of dose = 1.
qPCR = quantitative Polymerase Chain Reaction; IP = Artesunate; ND = Not Detected; RBCs: red blood cells.

Listing 16.2.6.3
Malaria 18S qPCR

Randomisation Number	Challenge Agent	Visit	Timepoint	Date of Measurement	Time of Measurement	Day Relative to Inoculum	Day Relative to Artesunate	Assay	Result (parasites/500µL) of packed RBCs
R105	K13	Safety Monitoring		01-DEC-2017	13:20	17	9	Replicate 1	1233
								Replicate 2	1250
								Replicate 3	1750
								Geometric Mean	1391.9930
				03-DEC-2017	07:45	19	11	Replicate 1	896
								Replicate 2	1132
								Replicate 3	1350
								Geometric Mean	1110.4425
				04-DEC-2017	07:22	20	12	Replicate 1	1096
								Replicate 2	1434
								Replicate 3	1688
								Geometric Mean	1384.3441
				05-DEC-2017	07:24	21	13	Replicate 1	1593
								Replicate 2	1679
								Replicate 3	1364
								Geometric Mean	1539.4312

Notes: Day relative to inoculum is the number of days relative to day of administration of the inoculum, where the day of administration = 0.
The inoculum was administered on Study Day 0 for K13-infected subjects and Study Day 1 for 3D7-infected subjects.
Day relative to IP is the number of days relative to day of administration of Artesunate, where the day of dose = 1.
qPCR = quantitative Polymerase Chain Reaction; IP = Artesunate; ND = Not Detected; RBCs: red blood cells.

Listing 16.2.6.3
Malaria 18S qPCR

Randomisation Number	Challenge Agent	Visit	Timepoint	Date of Measurement	Time of Measurement	Day Relative to Inoculum	Day Relative to Artesunate	Assay	Result (parasites/500µL) of packed RBCs
R105	K13	Safety Monitoring		07-DEC-2017	08:37	23	15	Replicate 1	2217
								Replicate 2	2027
								Replicate 3	1393
								Geometric Mean	1842.9923
				11-DEC-2017	08:44	27	19	Replicate 1	636
								Replicate 2	241
								Replicate 3	413
								Geometric Mean	398.5426
				12-DEC-2017	07:23	28	20	Replicate 1	470
								Replicate 2	311
								Replicate 3	144
								Geometric Mean	276.1046
		End of Study		13-DEC-2017	08:54	29	21	Replicate 1	338
								Replicate 2	383
								Replicate 3	252
								Geometric Mean	319.5254

Notes: Day relative to inoculum is the number of days relative to day of administration of the inoculum, where the day of administration = 0.
The inoculum was administered on Study Day 0 for K13-infected subjects and Study Day 1 for 3D7-infected subjects.
Day relative to IP is the number of days relative to day of administration of Artesunate, where the day of dose = 1.
qPCR = quantitative Polymerase Chain Reaction; IP = Artesunate; ND = Not Detected; RBCs: red blood cells.

Listing 16.2.6.3
Malaria 18S qPCR

Randomisation Number	Challenge Agent	Visit	Timepoint	Date of Measurement	Time of Measurement	Day Relative to Inoculum	Day Relative to Artesunate	Assay	Result (parasites/500µL) of packed RBCs
R105	K13	Unscheduled		18-DEC-2017	08:50	34	26	Replicate 1	86
								Replicate 2	34
								Replicate 3	51
								Geometric Mean	53.0293
				21-DEC-2017	09:15	37	29	Replicate 1	ND
								Replicate 2	ND
								Replicate 3	ND
								Geometric Mean	1.0000
R106	3D7	Inoculum		15-NOV-2017	09:41	0	-8	Replicate 1	ND
								Replicate 2	ND
								Replicate 3	ND
								Geometric Mean	1.0000
		Malaria Monitoring		19-NOV-2017	09:32	4	-4	Replicate 1	39
								Replicate 2	ND
								Replicate 3	44
								Geometric Mean	11.9722

Notes: Day relative to inoculum is the number of days relative to day of administration of the inoculum, where the day of administration = 0.
The inoculum was administered on Study Day 0 for K13-infected subjects and Study Day 1 for 3D7-infected subjects.
Day relative to IP is the number of days relative to day of administration of Artesunate, where the day of dose = 1.
qPCR = quantitative Polymerase Chain Reaction; IP = Artesunate; ND = Not Detected; RBCs: red blood cells.

Listing 16.2.6.3
Malaria 18S qPCR

Randomisation Number	Challenge Agent	Visit	Timepoint	Date of Measurement	Time of Measurement	Day Relative to Inoculum	Day Relative to Artesunate	Assay	Result (parasites/500µL) of packed RBCs
R106	3D7	Malaria Monitoring		20-NOV-2017	07:23	5	-3	Replicate 1	916
								Replicate 2	549
								Replicate 3	747
								Geometric Mean	721.5440
				20-NOV-2017	19:28	5	-3	Replicate 1	1204
								Replicate 2	988
								Replicate 3	813
								Geometric Mean	988.9128
				21-NOV-2017	08:42	6	-2	Replicate 1	161
								Replicate 2	165
								Replicate 3	120
								Geometric Mean	147.1737
				21-NOV-2017	19:47	6	-2	Replicate 1	4445
								Replicate 2	4920
								Replicate 3	4847
								Geometric Mean	4732.6381

Notes: Day relative to inoculum is the number of days relative to day of administration of the inoculum, where the day of administration = 0.
The inoculum was administered on Study Day 0 for K13-infected subjects and Study Day 1 for 3D7-infected subjects.
Day relative to IP is the number of days relative to day of administration of Artesunate, where the day of dose = 1.
qPCR = quantitative Polymerase Chain Reaction; IP = Artesunate; ND = Not Detected; RBCs: red blood cells.

Listing 16.2.6.3
Malaria 18S qPCR

Randomisation Number	Challenge Agent	Visit	Timepoint	Date of Measurement	Time of Measurement	Day Relative to Inoculum	Day Relative to Artesunate	Assay	Result (parasites/500μL) of packed RBCs
R106	3D7	Malaria Monitoring		22-NOV-2017	07:38	7	-1	Replicate 1	12549
								Replicate 2	13850
								Replicate 3	12905
								Geometric Mean	13089.9795
				22-NOV-2017	20:03	7	-1	Replicate 1	4625
								Replicate 2	4239
								Replicate 3	4723
								Geometric Mean	4524.0879
		Admission	Pre-dose	23-NOV-2017	09:33	8	1	Replicate 1	28184
								Replicate 2	32767
								Replicate 3	33523
								Geometric Mean	31399.8372
			2 Hrs Post-dose	23-NOV-2017	12:20	8	1	Replicate 1	35546
								Replicate 2	37290
								Replicate 3	43344
								Geometric Mean	38586.6761

Notes: Day relative to inoculum is the number of days relative to day of administration of the inoculum, where the day of administration = 0.
The inoculum was administered on Study Day 0 for K13-infected subjects and Study Day 1 for 3D7-infected subjects.
Day relative to IP is the number of days relative to day of administration of Artesunate, where the day of dose = 1.
qPCR = quantitative Polymerase Chain Reaction; IP = Artesunate; ND = Not Detected; RBCs: red blood cells.

Listing 16.2.6.3
Malaria 18S qPCR

Randomisation Number	Challenge Agent	Visit	Timepoint	Date of Measurement	Time of Measurement	Day Relative to Inoculum	Day Relative to Artesunate	Assay	Result (parasites/500µL) of packed RBCs
R106	3D7	Admission	4 Hrs Post-dose	23-NOV-2017	14:20	8	1	Replicate 1	45634
								Replicate 2	48068
								Replicate 3	44474
								Geometric Mean	46034.5173
			6 Hrs Post-dose	23-NOV-2017	16:22	8	1	Replicate 1	34160
								Replicate 2	39545
								Replicate 3	31464
								Geometric Mean	34898.5829
			8 Hrs Post-dose	23-NOV-2017	18:20	8	1	Replicate 1	17435
								Replicate 2	15475
								Replicate 3	17777
								Geometric Mean	16864.3793
			12 Hrs Post-dose	23-NOV-2017	22:32	8	1	Replicate 1	3980
								Replicate 2	4897
								Replicate 3	4057
								Geometric Mean	4292.1286

Notes: Day relative to inoculum is the number of days relative to day of administration of the inoculum, where the day of administration = 0.
The inoculum was administered on Study Day 0 for K13-infected subjects and Study Day 1 for 3D7-infected subjects.
Day relative to IP is the number of days relative to day of administration of Artesunate, where the day of dose = 1.
qPCR = quantitative Polymerase Chain Reaction; IP = Artesunate; ND = Not Detected; RBCs: red blood cells.

Listing 16.2.6.3
Malaria 18S qPCR

Randomisation Number	Challenge Agent	Visit	Timepoint	Date of Measurement	Time of Measurement	Day Relative to Inoculum	Day Relative to Artesunate	Assay	Result (parasites/500µL) of packed RBCs
R106	3D7	Admission 1	16 Hrs Post-dose	24-NOV-2017	02:20	9	2	Replicate 1	427
								Replicate 2	473
								Replicate 3	270
								Geometric Mean	379.2139
			20 Hrs Post-dose	24-NOV-2017	06:25	9	2	Replicate 1	ND
								Replicate 2	224
								Replicate 3	103
								Geometric Mean	28.4683
			24 Hrs Post-dose	24-NOV-2017	10:27	9	2	Replicate 1	ND
								Replicate 2	ND
								Replicate 3	ND
								Geometric Mean	1.0000
			28 Hrs Post-dose	24-NOV-2017	14:21	9	2	Replicate 1	94
								Replicate 2	ND
								Replicate 3	ND
								Geometric Mean	4.5468

Notes: Day relative to inoculum is the number of days relative to day of administration of the inoculum, where the day of administration = 0.
The inoculum was administered on Study Day 0 for K13-infected subjects and Study Day 1 for 3D7-infected subjects.
Day relative to IP is the number of days relative to day of administration of Artesunate, where the day of dose = 1.
qPCR = quantitative Polymerase Chain Reaction; IP = Artesunate; ND = Not Detected; RBCs: red blood cells.

Listing 16.2.6.3
Malaria 18S qPCR

Randomisation Number	Challenge Agent	Visit	Timepoint	Date of Measurement	Time of Measurement	Day Relative to Inoculum	Day Relative to Artesunate	Assay	Result (parasites/500µL) of packed RBCs
R106	3D7	Admission 1	32 Hrs Post-dose	24-NOV-2017	18:20	9	2	Replicate 1	ND
								Replicate 2	106
								Replicate 3	ND
								Geometric Mean	4.7326
			36 Hrs Post-dose	24-NOV-2017	22:20	9	2	Replicate 1	ND
								Replicate 2	ND
								Replicate 3	ND
								Geometric Mean	1.0000
		Admission 2	48 Hrs Post-dose	25-NOV-2017	10:20	10	3	Replicate 1	ND
								Replicate 2	ND
								Replicate 3	44
								Geometric Mean	3.5303
			60 Hrs Post-dose	25-NOV-2017	22:20	10	3	Replicate 1	ND
								Replicate 2	ND
								Replicate 3	ND
								Geometric Mean	1.0000

Notes: Day relative to inoculum is the number of days relative to day of administration of the inoculum, where the day of administration = 0.
The inoculum was administered on Study Day 0 for K13-infected subjects and Study Day 1 for 3D7-infected subjects.
Day relative to IP is the number of days relative to day of administration of Artesunate, where the day of dose = 1.
qPCR = quantitative Polymerase Chain Reaction; IP = Artesunate; ND = Not Detected; RBCs: red blood cells.

Listing 16.2.6.3
Malaria 18S qPCR

Randomisation Number	Challenge Agent	Visit	Timepoint	Date of Measurement	Time of Measurement	Day Relative to Inoculum	Day Relative to Artesunate	Assay	Result (parasites/500µL) of packed RBCs
R106	3D7	Admission 3	72 Hrs Post-dose	26-NOV-2017	10:20	11	4	Replicate 1	ND
								Replicate 2	ND
								Replicate 3	ND
								Geometric Mean	1.0000
				26-NOV-2017	19:17	11	4	Replicate 1	ND
								Replicate 2	47
								Replicate 3	65
								Geometric Mean	14.5101
		Safety Monitoring		27-NOV-2017	08:28	12	5	Replicate 1	ND
								Replicate 2	ND
								Replicate 3	77
								Geometric Mean	4.2543
				28-NOV-2017	08:13	13	6	Replicate 1	64
								Replicate 2	ND
								Replicate 3	27
								Geometric Mean	12.0000

Notes: Day relative to inoculum is the number of days relative to day of administration of the inoculum, where the day of administration = 0.
The inoculum was administered on Study Day 0 for K13-infected subjects and Study Day 1 for 3D7-infected subjects.
Day relative to IP is the number of days relative to day of administration of Artesunate, where the day of dose = 1.
qPCR = quantitative Polymerase Chain Reaction; IP = Artesunate; ND = Not Detected; RBCs: red blood cells.

Listing 16.2.6.3
Malaria 18S qPCR

Randomisation Number	Challenge Agent	Visit	Timepoint	Date of Measurement	Time of Measurement	Day Relative to Inoculum	Day Relative to Artesunate	Assay	Result (parasites/500µL) of packed RBCs
R106	3D7	Safety Monitoring		29-NOV-2017	08:35	14	7	Replicate 1	594
								Replicate 2	664
								Replicate 3	760
								Geometric Mean	669.2515
				30-NOV-2017	08:46	15	8	Replicate 1	4044
								Replicate 2	5659
								Replicate 3	4192
								Geometric Mean	4577.8059
				01-DEC-2017	08:27	16	9	Replicate 1	8420
								Replicate 2	7483
								Replicate 3	7850
								Geometric Mean	7908.3491
				03-DEC-2017	09:39	18	11	Replicate 1	ND
								Replicate 2	ND
								Replicate 3	ND
								Geometric Mean	1.0000

Notes: Day relative to inoculum is the number of days relative to day of administration of the inoculum, where the day of administration = 0.
The inoculum was administered on Study Day 0 for K13-infected subjects and Study Day 1 for 3D7-infected subjects.
Day relative to IP is the number of days relative to day of administration of Artesunate, where the day of dose = 1.
qPCR = quantitative Polymerase Chain Reaction; IP = Artesunate; ND = Not Detected; RBCs: red blood cells.

Listing 16.2.6.3
Malaria 18S qPCR

Randomisation Number	Challenge Agent	Visit	Timepoint	Date of Measurement	Time of Measurement	Day Relative to Inoculum	Day Relative to Artesunate	Assay	Result (parasites/500μL) of packed RBCs
R106	3D7	Safety Monitoring		04-DEC-2017	08:10	19	12	Replicate 1	ND
								Replicate 2	ND
								Replicate 3	ND
								Geometric Mean	1.0000
				05-DEC-2017	08:35	20	13	Replicate 1	ND
								Replicate 2	ND
								Replicate 3	ND
								Geometric Mean	1.0000
				07-DEC-2017	07:57	22	15	Replicate 1	43
								Replicate 2	ND
								Replicate 3	45
								Geometric Mean	12.4612
				11-DEC-2017	08:04	26	19	Replicate 1	ND
								Replicate 2	72
								Replicate 3	ND
								Geometric Mean	4.1602

Notes: Day relative to inoculum is the number of days relative to day of administration of the inoculum, where the day of administration = 0.
The inoculum was administered on Study Day 0 for K13-infected subjects and Study Day 1 for 3D7-infected subjects.
Day relative to IP is the number of days relative to day of administration of Artesunate, where the day of dose = 1.
qPCR = quantitative Polymerase Chain Reaction; IP = Artesunate; ND = Not Detected; RBCs: red blood cells.

Listing 16.2.6.3
Malaria 18S qPCR

Randomisation Number	Challenge Agent	Visit	Timepoint	Date of Measurement	Time of Measurement	Day Relative to Inoculum	Day Relative to Artesunate	Assay	Result (parasites/500µL) of packed RBCs
R106	3D7	End of Study		14-DEC-2017	08:53	29	22	Replicate 1	ND
								Replicate 2	32
								Replicate 3	35
								Geometric Mean	10.3850
		Unscheduled		18-DEC-2017	08:03	33	26	Replicate 1	ND
								Replicate 2	80
								Replicate 3	ND
								Geometric Mean	4.3089
				21-DEC-2017	07:23	36	29	Replicate 1	ND
								Replicate 2	ND
								Replicate 3	80
								Geometric Mean	4.3089
				29-DEC-2017	10:14	44	37	Replicate 1	ND
								Replicate 2	ND
								Replicate 3	ND
								Geometric Mean	1.0000

Notes: Day relative to inoculum is the number of days relative to day of administration of the inoculum, where the day of administration = 0.
The inoculum was administered on Study Day 0 for K13-infected subjects and Study Day 1 for 3D7-infected subjects.
Day relative to IP is the number of days relative to day of administration of Artesunate, where the day of dose = 1.
qPCR = quantitative Polymerase Chain Reaction; IP = Artesunate; ND = Not Detected; RBCs: red blood cells.

Listing 16.2.6.3
Malaria 18S qPCR

Randomisation Number	Challenge Agent	Visit	Timepoint	Date of Measurement	Time of Measurement	Day Relative to Inoculum	Day Relative to Artesunate	Assay	Result (parasites/500µL) of packed RBCs
R107	3D7	Inoculum		15-NOV-2017	10:01	0	-8	Replicate 1	ND
								Replicate 2	ND
								Replicate 3	ND
								Geometric Mean	1.0000
		Malaria Monitoring		19-NOV-2017	09:19	4	-4	Replicate 1	40
								Replicate 2	41
								Replicate 3	ND
								Geometric Mean	11.7927
				20-NOV-2017	07:50	5	-3	Replicate 1	182
								Replicate 2	363
								Replicate 3	207
								Geometric Mean	239.1385
				20-NOV-2017	20:26	5	-3	Replicate 1	554
								Replicate 2	683
								Replicate 3	588
								Geometric Mean	605.9488

Notes: Day relative to inoculum is the number of days relative to day of administration of the inoculum, where the day of administration = 0.
The inoculum was administered on Study Day 0 for K13-infected subjects and Study Day 1 for 3D7-infected subjects.
Day relative to IP is the number of days relative to day of administration of Artesunate, where the day of dose = 1.
qPCR = quantitative Polymerase Chain Reaction; IP = Artesunate; ND = Not Detected; RBCs: red blood cells.

Listing 16.2.6.3
Malaria 18S qPCR

Randomisation Number	Challenge Agent	Visit	Timepoint	Date of Measurement	Time of Measurement	Day Relative to Inoculum	Day Relative to Artesunate	Assay	Result (parasites/500µL) of packed RBCs
R107	3D7	Malaria Monitoring		21-NOV-2017	08:09	6	-2	Replicate 1	125
								Replicate 2	236
								Replicate 3	221
								Geometric Mean	186.8120
				21-NOV-2017	21:23	6	-2	Replicate 1	2212
								Replicate 2	2417
								Replicate 3	2253
								Geometric Mean	2292.3151
				22-NOV-2017	07:13	7	-1	Replicate 1	5444
								Replicate 2	4724
								Replicate 3	4729
								Geometric Mean	4954.4917
				22-NOV-2017	20:50	7	-1	Replicate 1	1908
								Replicate 2	2098
								Replicate 3	1938
								Geometric Mean	1979.6080

Notes: Day relative to inoculum is the number of days relative to day of administration of the inoculum, where the day of administration = 0.
The inoculum was administered on Study Day 0 for K13-infected subjects and Study Day 1 for 3D7-infected subjects.
Day relative to IP is the number of days relative to day of administration of Artesunate, where the day of dose = 1.
qPCR = quantitative Polymerase Chain Reaction; IP = Artesunate; ND = Not Detected; RBCs: red blood cells.

Listing 16.2.6.3
Malaria 18S qPCR

Randomisation Number	Challenge Agent	Visit	Timepoint	Date of Measurement	Time of Measurement	Day Relative to Inoculum	Day Relative to Artesunate	Assay	Result (parasites/500µL) of packed RBCs
R107	3D7	Admission	Pre-dose	23-NOV-2017	09:35	8	1	Replicate 1	11241
								Replicate 2	10009
								Replicate 3	10660
								Geometric Mean	10624.7229
			2 Hrs Post-dose	23-NOV-2017	12:10	8	1	Replicate 1	15271
								Replicate 2	13300
								Replicate 3	17695
								Geometric Mean	15317.5708
			4 Hrs Post-dose	23-NOV-2017	14:10	8	1	Replicate 1	17727
								Replicate 2	12615
								Replicate 3	24189
								Geometric Mean	17554.1635
			6 Hrs Post-dose	23-NOV-2017	16:10	8	1	Replicate 1	24887
								Replicate 2	16498
								Replicate 3	24156
								Geometric Mean	21485.3759

Notes: Day relative to inoculum is the number of days relative to day of administration of the inoculum, where the day of administration = 0.
The inoculum was administered on Study Day 0 for K13-infected subjects and Study Day 1 for 3D7-infected subjects.
Day relative to IP is the number of days relative to day of administration of Artesunate, where the day of dose = 1.
qPCR = quantitative Polymerase Chain Reaction; IP = Artesunate; ND = Not Detected; RBCs: red blood cells.

Listing 16.2.6.3
Malaria 18S qPCR

Randomisation Number	Challenge Agent	Visit	Timepoint	Date of Measurement	Time of Measurement	Day Relative to Inoculum	Day Relative to Artesunate	Assay	Result (parasites/500µL) of packed RBCs
R107	3D7	Admission	8 Hrs Post-dose	23-NOV-2017	18:10	8	1	Replicate 1	16234
								Replicate 2	12893
								Replicate 3	16794
								Geometric Mean	15204.7012
			12 Hrs Post-dose	23-NOV-2017	22:10	8	1	Replicate 1	12876
								Replicate 2	16411
								Replicate 3	13636
								Geometric Mean	14229.8723
		Admission 1	16 Hrs Post-dose	24-NOV-2017	02:10	9	2	Replicate 1	6875
								Replicate 2	6724
								Replicate 3	6965
								Geometric Mean	6853.9433
			20 Hrs Post-dose	24-NOV-2017	06:10	9	2	Replicate 1	2386
								Replicate 2	2297
								Replicate 3	2966
								Geometric Mean	2533.1886

Notes: Day relative to inoculum is the number of days relative to day of administration of the inoculum, where the day of administration = 0.
The inoculum was administered on Study Day 0 for K13-infected subjects and Study Day 1 for 3D7-infected subjects.
Day relative to IP is the number of days relative to day of administration of Artesunate, where the day of dose = 1.
qPCR = quantitative Polymerase Chain Reaction; IP = Artesunate; ND = Not Detected; RBCs: red blood cells.

Listing 16.2.6.3
Malaria 18S qPCR

Randomisation Number	Challenge Agent	Visit	Timepoint	Date of Measurement	Time of Measurement	Day Relative to Inoculum	Day Relative to Artesunate	Assay	Result (parasites/500µL) of packed RBCs
R107	3D7	Admission 1	24 Hrs Post-dose	24-NOV-2017	10:10	9	2	Replicate 1	584
								Replicate 2	613
								Replicate 3	504
								Geometric Mean	565.0687
			28 Hrs Post-dose	24-NOV-2017	14:10	9	2	Replicate 1	157
								Replicate 2	216
								Replicate 3	235
								Geometric Mean	199.7440
			32 Hrs Post-dose	24-NOV-2017	18:10	9	2	Replicate 1	49
								Replicate 2	135
								Replicate 3	157
								Geometric Mean	101.2690
			36 Hrs Post-dose	24-NOV-2017	22:11	9	2	Replicate 1	104
								Replicate 2	40
								Replicate 3	103
								Geometric Mean	75.3894

Notes: Day relative to inoculum is the number of days relative to day of administration of the inoculum, where the day of administration = 0.
The inoculum was administered on Study Day 0 for K13-infected subjects and Study Day 1 for 3D7-infected subjects.
Day relative to IP is the number of days relative to day of administration of Artesunate, where the day of dose = 1.
qPCR = quantitative Polymerase Chain Reaction; IP = Artesunate; ND = Not Detected; RBCs: red blood cells.

Listing 16.2.6.3
Malaria 18S qPCR

Randomisation Number	Challenge Agent	Visit	Timepoint	Date of Measurement	Time of Measurement	Day Relative to Inoculum	Day Relative to Artesunate	Assay	Result (parasites/500µL) of packed RBCs
R107	3D7	Admission 2	48 Hrs Post-dose	25-NOV-2017	10:10	10	3	Replicate 1	ND
								Replicate 2	ND
								Replicate 3	104
								Geometric Mean	4.7027
			60 Hrs Post-dose	25-NOV-2017	22:10	10	3	Replicate 1	26
								Replicate 2	ND
								Replicate 3	34
								Geometric Mean	9.5973
		Admission 3	72 Hrs Post-dose	26-NOV-2017	10:11	11	4	Replicate 1	ND
								Replicate 2	66
								Replicate 3	35
								Geometric Mean	13.2192
				26-NOV-2017	19:06	11	4	Replicate 1	ND
								Replicate 2	53
								Replicate 3	ND
								Geometric Mean	3.7563

Notes: Day relative to inoculum is the number of days relative to day of administration of the inoculum, where the day of administration = 0.
The inoculum was administered on Study Day 0 for K13-infected subjects and Study Day 1 for 3D7-infected subjects.
Day relative to IP is the number of days relative to day of administration of Artesunate, where the day of dose = 1.
qPCR = quantitative Polymerase Chain Reaction; IP = Artesunate; ND = Not Detected; RBCs: red blood cells.

Listing 16.2.6.3
Malaria 18S qPCR

Randomisation Number	Challenge Agent	Visit	Timepoint	Date of Measurement	Time of Measurement	Day Relative to Inoculum	Day Relative to Artesunate	Assay	Result (parasites/500µL) of packed RBCs
R107	3D7	Safety Monitoring		27-NOV-2017	07:22	12	5	Replicate 1	17
								Replicate 2	ND
								Replicate 3	ND
								Geometric Mean	2.5713
				28-NOV-2017	07:28	13	6	Replicate 1	ND
								Replicate 2	ND
								Replicate 3	ND
								Geometric Mean	1.0000
				29-NOV-2017	07:37	14	7	Replicate 1	57
								Replicate 2	86
								Replicate 3	225
								Geometric Mean	103.3202
				30-NOV-2017	07:30	15	8	Replicate 1	534
								Replicate 2	465
								Replicate 3	428
								Geometric Mean	473.6738

Notes: Day relative to inoculum is the number of days relative to day of administration of the inoculum, where the day of administration = 0.
The inoculum was administered on Study Day 0 for K13-infected subjects and Study Day 1 for 3D7-infected subjects.
Day relative to IP is the number of days relative to day of administration of Artesunate, where the day of dose = 1.
qPCR = quantitative Polymerase Chain Reaction; IP = Artesunate; ND = Not Detected; RBCs: red blood cells.

Listing 16.2.6.3
Malaria 18S qPCR

Randomisation Number	Challenge Agent	Visit	Timepoint	Date of Measurement	Time of Measurement	Day Relative to Inoculum	Day Relative to Artesunate	Assay	Result (parasites/500µL) of packed RBCs
R107	3D7	Safety Monitoring		01-DEC-2017	07:43	16	9	Replicate 1	1624
								Replicate 2	1345
								Replicate 3	1420
								Geometric Mean	1458.3627
				03-DEC-2017	08:29	18	11	Replicate 1	9087
								Replicate 2	9188
								Replicate 3	9749
								Geometric Mean	9336.8532
				04-DEC-2017	08:48	19	12	Replicate 1	134783
								Replicate 2	150220
								Replicate 3	151583
								Geometric Mean	145324.1500
				05-DEC-2017	07:41	20	13	Replicate 1	8884
								Replicate 2	10254
								Replicate 3	9693
								Geometric Mean	9593.7123

Notes: Day relative to inoculum is the number of days relative to day of administration of the inoculum, where the day of administration = 0.
The inoculum was administered on Study Day 0 for K13-infected subjects and Study Day 1 for 3D7-infected subjects.
Day relative to IP is the number of days relative to day of administration of Artesunate, where the day of dose = 1.
qPCR = quantitative Polymerase Chain Reaction; IP = Artesunate; ND = Not Detected; RBCs: red blood cells.

Listing 16.2.6.3
Malaria 18S qPCR

Randomisation Number	Challenge Agent	Visit	Timepoint	Date of Measurement	Time of Measurement	Day Relative to Inoculum	Day Relative to Artesunate	Assay	Result (parasites/500µL) of packed RBCs
R107	3D7	Safety Monitoring		07-DEC-2017	09:15	22	15	Replicate 1	117
								Replicate 2	137
								Replicate 3	ND
								Geometric Mean	25.2136
				11-DEC-2017	09:29	26	19	Replicate 1	1885
								Replicate 2	1182
								Replicate 3	1445
								Geometric Mean	1476.6092
		End of Study		14-DEC-2017	09:31	29	22	Replicate 1	4033
								Replicate 2	4114
								Replicate 3	4056
								Geometric Mean	4067.5243
		Unscheduled		15-DEC-2017	09:33	30	23	Replicate 1	4592
								Replicate 2	4095
								Replicate 3	3567
								Geometric Mean	4063.0575

Notes: Day relative to inoculum is the number of days relative to day of administration of the inoculum, where the day of administration = 0.
The inoculum was administered on Study Day 0 for K13-infected subjects and Study Day 1 for 3D7-infected subjects.
Day relative to IP is the number of days relative to day of administration of Artesunate, where the day of dose = 1.
qPCR = quantitative Polymerase Chain Reaction; IP = Artesunate; ND = Not Detected; RBCs: red blood cells.

Listing 16.2.6.3
Malaria 18S qPCR

Randomisation Number	Challenge Agent	Visit	Timepoint	Date of Measurement	Time of Measurement	Day Relative to Inoculum	Day Relative to Artesunate	Assay	Result (parasites/500µL) of packed RBCs
R107	3D7	Unscheduled		18-DEC-2017	09:15	33	26	Replicate 1	2591
								Replicate 2	2029
								Replicate 3	1756
								Geometric Mean	2097.7706
				21-DEC-2017	09:26	36	29	Replicate 1	1882
								Replicate 2	1568
								Replicate 3	1642
								Geometric Mean	1692.1789
				27-DEC-2017	11:23	42	35	Replicate 1	37
								Replicate 2	68
								Replicate 3	77
								Geometric Mean	57.8629
				03-JAN-2018	09:01	49	42	Replicate 1	ND
								Replicate 2	ND
								Replicate 3	ND
								Geometric Mean	1.0000

Notes: Day relative to inoculum is the number of days relative to day of administration of the inoculum, where the day of administration = 0.
The inoculum was administered on Study Day 0 for K13-infected subjects and Study Day 1 for 3D7-infected subjects.
Day relative to IP is the number of days relative to day of administration of Artesunate, where the day of dose = 1.
qPCR = quantitative Polymerase Chain Reaction; IP = Artesunate; ND = Not Detected; RBCs: red blood cells.

Listing 16.2.6.3
Malaria 18S qPCR

Randomisation Number	Challenge Agent	Visit	Timepoint	Date of Measurement	Time of Measurement	Day Relative to Inoculum	Day Relative to Artesunate	Assay	Result (parasites/500µL) of packed RBCs
R108	3D7	Inoculum		15-NOV-2017	10:14	0	-8	Replicate 1	ND
								Replicate 2	ND
								Replicate 3	ND
								Geometric Mean	1.0000
		Malaria Monitoring		19-NOV-2017	08:40	4	-4	Replicate 1	ND
								Replicate 2	ND
								Replicate 3	51
								Geometric Mean	3.7084
				20-NOV-2017	07:55	5	-3	Replicate 1	460
								Replicate 2	629
								Replicate 3	463
								Geometric Mean	511.6777
				20-NOV-2017	20:01	5	-3	Replicate 1	934
								Replicate 2	1227
								Replicate 3	1010
								Geometric Mean	1049.9556

Notes: Day relative to inoculum is the number of days relative to day of administration of the inoculum, where the day of administration = 0.
The inoculum was administered on Study Day 0 for K13-infected subjects and Study Day 1 for 3D7-infected subjects.
Day relative to IP is the number of days relative to day of administration of Artesunate, where the day of dose = 1.
qPCR = quantitative Polymerase Chain Reaction; IP = Artesunate; ND = Not Detected; RBCs: red blood cells.

Listing 16.2.6.3
Malaria 18S qPCR

Randomisation Number	Challenge Agent	Visit	Timepoint	Date of Measurement	Time of Measurement	Day Relative to Inoculum	Day Relative to Artesunate	Assay	Result (parasites/500µL) of packed RBCs
R108	3D7	Malaria Monitoring		21-NOV-2017	09:31	6	-2	Replicate 1	331
								Replicate 2	272
								Replicate 3	199
								Geometric Mean	261.6676
				21-NOV-2017	19:26	6	-2	Replicate 1	2358
								Replicate 2	1879
								Replicate 3	2629
								Geometric Mean	2266.8375
				22-NOV-2017	09:08	7	-1	Replicate 1	12318
								Replicate 2	12772
								Replicate 3	13377
								Geometric Mean	12815.0197
				22-NOV-2017	20:38	7	-1	Replicate 1	3962
								Replicate 2	4480
								Replicate 3	4160
								Geometric Mean	4195.2895

Notes: Day relative to inoculum is the number of days relative to day of administration of the inoculum, where the day of administration = 0.
The inoculum was administered on Study Day 0 for K13-infected subjects and Study Day 1 for 3D7-infected subjects.
Day relative to IP is the number of days relative to day of administration of Artesunate, where the day of dose = 1.
qPCR = quantitative Polymerase Chain Reaction; IP = Artesunate; ND = Not Detected; RBCs: red blood cells.

Listing 16.2.6.3
Malaria 18S qPCR

Randomisation Number	Challenge Agent	Visit	Timepoint	Date of Measurement	Time of Measurement	Day Relative to Inoculum	Day Relative to Artesunate	Assay	Result (parasites/500µL) of packed RBCs
R108	3D7	Admission	Pre-dose	23-NOV-2017	09:17	8	1	Replicate 1	15665
								Replicate 2	13453
								Replicate 3	13748
								Geometric Mean	14255.9563
			2 Hrs Post-dose	23-NOV-2017	12:05	8	1	Replicate 1	36424
								Replicate 2	43757
								Replicate 3	46846
								Geometric Mean	42108.4475
			4 Hrs Post-dose	23-NOV-2017	14:01	8	1	Replicate 1	42587
								Replicate 2	54921
								Replicate 3	53428
								Geometric Mean	49995.1797
			6 Hrs Post-dose	23-NOV-2017	16:12	8	1	Replicate 1	36096
								Replicate 2	34331
								Replicate 3	30333
								Geometric Mean	33498.1096

Notes: Day relative to inoculum is the number of days relative to day of administration of the inoculum, where the day of administration = 0.
The inoculum was administered on Study Day 0 for K13-infected subjects and Study Day 1 for 3D7-infected subjects.
Day relative to IP is the number of days relative to day of administration of Artesunate, where the day of dose = 1.
qPCR = quantitative Polymerase Chain Reaction; IP = Artesunate; ND = Not Detected; RBCs: red blood cells.

Listing 16.2.6.3
Malaria 18S qPCR

Randomisation Number	Challenge Agent	Visit	Timepoint	Date of Measurement	Time of Measurement	Day Relative to Inoculum	Day Relative to Artesunate	Assay	Result (parasites/500µL) of packed RBCs
R108	3D7	Admission	8 Hrs Post-dose	23-NOV-2017	18:02	8	1	Replicate 1	30530
								Replicate 2	31108
								Replicate 3	26781
								Geometric Mean	29408.6594
			12 Hrs Post-dose	23-NOV-2017	22:02	8	1	Replicate 1	9599
								Replicate 2	11173
								Replicate 3	9031
								Geometric Mean	9894.1209
		Admission 1	16 Hrs Post-dose	24-NOV-2017	02:01	9	2	Replicate 1	2081
								Replicate 2	1593
								Replicate 3	1388
								Geometric Mean	1663.2560
			20 Hrs Post-dose	24-NOV-2017	06:01	9	2	Replicate 1	129
								Replicate 2	373
								Replicate 3	191
								Geometric Mean	209.4646

Notes: Day relative to inoculum is the number of days relative to day of administration of the inoculum, where the day of administration = 0.
The inoculum was administered on Study Day 0 for K13-infected subjects and Study Day 1 for 3D7-infected subjects.
Day relative to IP is the number of days relative to day of administration of Artesunate, where the day of dose = 1.
qPCR = quantitative Polymerase Chain Reaction; IP = Artesunate; ND = Not Detected; RBCs: red blood cells.

Listing 16.2.6.3
Malaria 18S qPCR

Randomisation Number	Challenge Agent	Visit	Timepoint	Date of Measurement	Time of Measurement	Day Relative to Inoculum	Day Relative to Artesunate	Assay	Result (parasites/500µL) of packed RBCs
R108	3D7	Admission 1	24 Hrs Post-dose	24-NOV-2017	10:08	9	2	Replicate 1	153
								Replicate 2	167
								Replicate 3	116
								Geometric Mean	143.6444
			28 Hrs Post-dose	24-NOV-2017	14:04	9	2	Replicate 1	48
								Replicate 2	ND
								Replicate 3	ND
								Geometric Mean	3.6342
			32 Hrs Post-dose	24-NOV-2017	18:01	9	2	Replicate 1	75
								Replicate 2	52
								Replicate 3	38
								Geometric Mean	52.9195
			36 Hrs Post-dose	24-NOV-2017	22:04	9	2	Replicate 1	56
								Replicate 2	ND
								Replicate 3	ND
								Geometric Mean	3.8259

Notes: Day relative to inoculum is the number of days relative to day of administration of the inoculum, where the day of administration = 0.
The inoculum was administered on Study Day 0 for K13-infected subjects and Study Day 1 for 3D7-infected subjects.
Day relative to IP is the number of days relative to day of administration of Artesunate, where the day of dose = 1.
qPCR = quantitative Polymerase Chain Reaction; IP = Artesunate; ND = Not Detected; RBCs: red blood cells.

Listing 16.2.6.3
Malaria 18S qPCR

Randomisation Number	Challenge Agent	Visit	Timepoint	Date of Measurement	Time of Measurement	Day Relative to Inoculum	Day Relative to Artesunate	Assay	Result (parasites/500µL) of packed RBCs
R108	3D7	Admission 2	48 Hrs Post-dose	25-NOV-2017	10:01	10	3	Replicate 1	ND
								Replicate 2	ND
								Replicate 3	ND
								Geometric Mean	1.0000
			60 Hrs Post-dose	25-NOV-2017	22:03	10	3	Replicate 1	54
								Replicate 2	48
								Replicate 3	ND
								Geometric Mean	13.7366
		Admission 3	72 Hrs Post-dose	26-NOV-2017	10:02	11	4	Replicate 1	ND
								Replicate 2	ND
								Replicate 3	ND
								Geometric Mean	1.0000
				26-NOV-2017	19:05	11	4	Replicate 1	ND
								Replicate 2	ND
								Replicate 3	47
								Geometric Mean	3.6088

Notes: Day relative to inoculum is the number of days relative to day of administration of the inoculum, where the day of administration = 0.
The inoculum was administered on Study Day 0 for K13-infected subjects and Study Day 1 for 3D7-infected subjects.
Day relative to IP is the number of days relative to day of administration of Artesunate, where the day of dose = 1.
qPCR = quantitative Polymerase Chain Reaction; IP = Artesunate; ND = Not Detected; RBCs: red blood cells.

Listing 16.2.6.3
Malaria 18S qPCR

Randomisation Number	Challenge Agent	Visit	Timepoint	Date of Measurement	Time of Measurement	Day Relative to Inoculum	Day Relative to Artesunate	Assay	Result (parasites/500µL) of packed RBCs
R108	3D7	Safety Monitoring		28-NOV-2017	08:50	13	6	Replicate 1	115
								Replicate 2	69
								Replicate 3	24
								Geometric Mean	57.5333
				29-NOV-2017	07:06	14	7	Replicate 1	1269
								Replicate 2	1285
								Replicate 3	1568
								Geometric Mean	1367.4253
				30-NOV-2017	06:57	15	8	Replicate 1	4250
								Replicate 2	5341
								Replicate 3	4232
								Geometric Mean	4579.8620
				01-DEC-2017	07:14	16	9	Replicate 1	14110
								Replicate 2	15269
								Replicate 3	15376
								Geometric Mean	14907.1170

Notes: Day relative to inoculum is the number of days relative to day of administration of the inoculum, where the day of administration = 0.
The inoculum was administered on Study Day 0 for K13-infected subjects and Study Day 1 for 3D7-infected subjects.
Day relative to IP is the number of days relative to day of administration of Artesunate, where the day of dose = 1.
qPCR = quantitative Polymerase Chain Reaction; IP = Artesunate; ND = Not Detected; RBCs: red blood cells.

Listing 16.2.6.3
Malaria 18S qPCR

Randomisation Number	Challenge Agent	Visit	Timepoint	Date of Measurement	Time of Measurement	Day Relative to Inoculum	Day Relative to Artesunate	Assay	Result (parasites/500µL) of packed RBCs
R108	3D7	Safety Monitoring		03-DEC-2017	08:50	18	11	Replicate 1	ND
								Replicate 2	ND
								Replicate 3	ND
								Geometric Mean	1.0000
				04-DEC-2017	07:10	19	12	Replicate 1	ND
								Replicate 2	ND
								Replicate 3	ND
								Geometric Mean	1.0000
				05-DEC-2017	07:47	20	13	Replicate 1	57
								Replicate 2	ND
								Replicate 3	ND
								Geometric Mean	3.8485
				07-DEC-2017	06:54	22	15	Replicate 1	122
								Replicate 2	37
								Replicate 3	128
								Geometric Mean	83.2895

Notes: Day relative to inoculum is the number of days relative to day of administration of the inoculum, where the day of administration = 0.
The inoculum was administered on Study Day 0 for K13-infected subjects and Study Day 1 for 3D7-infected subjects.
Day relative to IP is the number of days relative to day of administration of Artesunate, where the day of dose = 1.
qPCR = quantitative Polymerase Chain Reaction; IP = Artesunate; ND = Not Detected; RBCs: red blood cells.

Listing 16.2.6.3
Malaria 18S qPCR

Randomisation Number	Challenge Agent	Visit	Timepoint	Date of Measurement	Time of Measurement	Day Relative to Inoculum	Day Relative to Artesunate	Assay	Result (parasites/500µL) of packed RBCs
R108	3D7	Safety Monitoring		11-DEC-2017	08:54	26	19	Replicate 1	82
								Replicate 2	81
								Replicate 3	82
								Geometric Mean	81.6653
		End of Study		14-DEC-2017	08:37	29	22	Replicate 1	132
								Replicate 2	300
								Replicate 3	204
								Geometric Mean	200.6512
		Unscheduled		18-DEC-2017	07:48	33	26	Replicate 1	137
								Replicate 2	129
								Replicate 3	47
								Geometric Mean	94.0018
				21-DEC-2017	07:53	36	29	Replicate 1	ND
								Replicate 2	ND
								Replicate 3	ND
								Geometric Mean	1.0000

Notes: Day relative to inoculum is the number of days relative to day of administration of the inoculum, where the day of administration = 0.
The inoculum was administered on Study Day 0 for K13-infected subjects and Study Day 1 for 3D7-infected subjects.
Day relative to IP is the number of days relative to day of administration of Artesunate, where the day of dose = 1.
qPCR = quantitative Polymerase Chain Reaction; IP = Artesunate; ND = Not Detected; RBCs: red blood cells.

Listing 16.2.6.3
Malaria 18S qPCR

Randomisation Number	Challenge Agent	Visit	Timepoint	Date of Measurement	Time of Measurement	Day Relative to Inoculum	Day Relative to Artesunate	Assay	Result (parasites/500µL) of packed RBCs
R201	K13	Inoculum		19-FEB-2018	10:05	0	-9	Replicate 1	ND
								Replicate 2	ND
								Replicate 3	ND
								Geometric Mean	1.0000
		Malaria Monitoring		23-FEB-2018	07:45	4	-5	Replicate 1	41
								Replicate 2	155
								Replicate 3	35
								Geometric Mean	60.5891
				24-FEB-2018	07:47	5	-4	Replicate 1	ND
								Replicate 2	99
								Replicate 3	34
								Geometric Mean	14.9867
				24-FEB-2018	19:28	5	-4	Replicate 1	544
								Replicate 2	535
								Replicate 3	816
								Geometric Mean	619.2713

Notes: Day relative to inoculum is the number of days relative to day of administration of the inoculum, where the day of administration = 0.
The inoculum was administered on Study Day 0 for K13-infected subjects and Study Day 1 for 3D7-infected subjects.
Day relative to IP is the number of days relative to day of administration of Artesunate, where the day of dose = 1.
qPCR = quantitative Polymerase Chain Reaction; IP = Artesunate; ND = Not Detected; RBCs: red blood cells.

Listing 16.2.6.3
Malaria 18S qPCR

Randomisation Number	Challenge Agent	Visit	Timepoint	Date of Measurement	Time of Measurement	Day Relative to Inoculum	Day Relative to Artesunate	Assay	Result (parasites/500µL) of packed RBCs
R201	K13	Malaria Monitoring		25-FEB-2018	08:30	6	-3	Replicate 1	818
								Replicate 2	1009
								Replicate 3	658
								Geometric Mean	815.8747
				25-FEB-2018	20:02	6	-3	Replicate 1	1298
								Replicate 2	1004
								Replicate 3	1177
								Geometric Mean	1153.2628
				26-FEB-2018	08:04	7	-2	Replicate 1	1806
								Replicate 2	2168
								Replicate 3	2275
								Geometric Mean	2072.9372
				26-FEB-2018	20:20	7	-2	Replicate 1	11236
								Replicate 2	14692
								Replicate 3	13037
								Geometric Mean	12910.9076

Notes: Day relative to inoculum is the number of days relative to day of administration of the inoculum, where the day of administration = 0.
The inoculum was administered on Study Day 0 for K13-infected subjects and Study Day 1 for 3D7-infected subjects.
Day relative to IP is the number of days relative to day of administration of Artesunate, where the day of dose = 1.
qPCR = quantitative Polymerase Chain Reaction; IP = Artesunate; ND = Not Detected; RBCs: red blood cells.

Listing 16.2.6.3
Malaria 18S qPCR

Randomisation Number	Challenge Agent	Visit	Timepoint	Date of Measurement	Time of Measurement	Day Relative to Inoculum	Day Relative to Artesunate	Assay	Result (parasites/500µL) of packed RBCs
R201	K13	Malaria Monitoring		27-FEB-2018	08:53	8	-1	Replicate 1	19903
								Replicate 2	17286
								Replicate 3	19327
								Geometric Mean	18804.3864
				27-FEB-2018	19:58	8	-1	Replicate 1	19265
								Replicate 2	17481
								Replicate 3	19174
								Geometric Mean	18621.5575
		Admission	Pre-dose	28-FEB-2018	12:39	9	1	Replicate 1	59140
								Replicate 2	55116
								Replicate 3	58186
								Geometric Mean	57454.7366
			2 Hrs Post-dose	28-FEB-2018	15:23	9	1	Replicate 1	71854
								Replicate 2	73761
								Replicate 3	77101
								Geometric Mean	74207.1784

Notes: Day relative to inoculum is the number of days relative to day of administration of the inoculum, where the day of administration = 0.
The inoculum was administered on Study Day 0 for K13-infected subjects and Study Day 1 for 3D7-infected subjects.
Day relative to IP is the number of days relative to day of administration of Artesunate, where the day of dose = 1.
qPCR = quantitative Polymerase Chain Reaction; IP = Artesunate; ND = Not Detected; RBCs: red blood cells.

Listing 16.2.6.3
Malaria 18S qPCR

Randomisation Number	Challenge Agent	Visit	Timepoint	Date of Measurement	Time of Measurement	Day Relative to Inoculum	Day Relative to Artesunate	Assay	Result (parasites/500µL) of packed RBCs
R201	K13	Admission	4 Hrs Post-dose	28-FEB-2018	17:22	9	1	Replicate 1	127911
								Replicate 2	134672
								Replicate 3	134004
								Geometric Mean	132160.2918
			6 Hrs Post-dose	28-FEB-2018	19:22	9	1	Replicate 1	127162
								Replicate 2	121134
								Replicate 3	108957
								Geometric Mean	118839.1441
			8 Hrs Post-dose	28-FEB-2018	21:22	9	1	Replicate 1	127594
								Replicate 2	93400
								Replicate 3	87775
								Geometric Mean	101511.6738
		Admission 1	16 Hrs Post-dose	01-MAR-2018	05:23	10	2	Replicate 1	36768
								Replicate 2	41915
								Replicate 3	39730
								Geometric Mean	39414.1968

Notes: Day relative to inoculum is the number of days relative to day of administration of the inoculum, where the day of administration = 0.
The inoculum was administered on Study Day 0 for K13-infected subjects and Study Day 1 for 3D7-infected subjects.
Day relative to IP is the number of days relative to day of administration of Artesunate, where the day of dose = 1.
qPCR = quantitative Polymerase Chain Reaction; IP = Artesunate; ND = Not Detected; RBCs: red blood cells.

Listing 16.2.6.3
Malaria 18S qPCR

Randomisation Number	Challenge Agent	Visit	Timepoint	Date of Measurement	Time of Measurement	Day Relative to Inoculum	Day Relative to Artesunate	Assay	Result (parasites/500µL) of packed RBCs
R201	K13	Admission 1	20 Hrs Post-dose	01-MAR-2018	09:22	10	2	Replicate 1	19872
								Replicate 2	20257
								Replicate 3	21575
								Geometric Mean	20555.2377
			24 Hrs Post-dose	01-MAR-2018	13:26	10	2	Replicate 1	18994
								Replicate 2	18747
								Replicate 3	18968
								Geometric Mean	18902.6743
			28 Hrs Post-dose	01-MAR-2018	17:29	10	2	Replicate 1	12936
								Replicate 2	12962
								Replicate 3	13506
								Geometric Mean	13132.0620
			32 Hrs Post-dose	01-MAR-2018	22:40	10	2	Replicate 1	3887
								Replicate 2	3773
								Replicate 3	3172
								Geometric Mean	3596.4844

Notes: Day relative to inoculum is the number of days relative to day of administration of the inoculum, where the day of administration = 0.
The inoculum was administered on Study Day 0 for K13-infected subjects and Study Day 1 for 3D7-infected subjects.
Day relative to IP is the number of days relative to day of administration of Artesunate, where the day of dose = 1.
qPCR = quantitative Polymerase Chain Reaction; IP = Artesunate; ND = Not Detected; RBCs: red blood cells.

Listing 16.2.6.3
Malaria 18S qPCR

Randomisation Number	Challenge Agent	Visit	Timepoint	Date of Measurement	Time of Measurement	Day Relative to Inoculum	Day Relative to Artesunate	Assay	Result (parasites/500µL) of packed RBCs
R201	K13	Admission 2	48 Hrs Post-dose	02-MAR-2018	13:24	11	3	Replicate 1	3978
								Replicate 2	4074
								Replicate 3	6303
								Geometric Mean	4674.5991
			52 Hrs Post-dose	02-MAR-2018	17:36	11	3	Replicate 1	23578
								Replicate 2	24545
								Replicate 3	31263
								Geometric Mean	26252.2713
			56 Hrs Post-dose	02-MAR-2018	21:28	11	3	Replicate 1	37515
								Replicate 2	33721
								Replicate 3	34757
								Geometric Mean	35295.2200
		Admission 3	60 Hrs Post-dose	03-MAR-2018	01:24	12	4	Replicate 1	33433
								Replicate 2	34585
								Replicate 3	32173
								Geometric Mean	33382.4517

Notes: Day relative to inoculum is the number of days relative to day of administration of the inoculum, where the day of administration = 0.
The inoculum was administered on Study Day 0 for K13-infected subjects and Study Day 1 for 3D7-infected subjects.
Day relative to IP is the number of days relative to day of administration of Artesunate, where the day of dose = 1.
qPCR = quantitative Polymerase Chain Reaction; IP = Artesunate; ND = Not Detected; RBCs: red blood cells.

Listing 16.2.6.3
Malaria 18S qPCR

Randomisation Number	Challenge Agent	Visit	Timepoint	Date of Measurement	Time of Measurement	Day Relative to Inoculum	Day Relative to Artesunate	Assay	Result (parasites/500µL) of packed RBCs
R201	K13	Admission 3	72 Hrs Post-dose	03-MAR-2018	13:27	12	4	Replicate 1	18851
								Replicate 2	21058
								Replicate 3	20146
								Geometric Mean	19997.7030
				03-MAR-2018	21:14	12	4	Replicate 1	4416
								Replicate 2	6262
								Replicate 3	4666
								Geometric Mean	5053.1511
		Safety Monitoring		04-MAR-2018	08:46	13	5	Replicate 1	441
								Replicate 2	686
								Replicate 3	352
								Geometric Mean	473.9892
				04-MAR-2018	19:16	13	5	Replicate 1	148
								Replicate 2	167
								Replicate 3	295
								Geometric Mean	193.9099

Notes: Day relative to inoculum is the number of days relative to day of administration of the inoculum, where the day of administration = 0.
The inoculum was administered on Study Day 0 for K13-infected subjects and Study Day 1 for 3D7-infected subjects.
Day relative to IP is the number of days relative to day of administration of Artesunate, where the day of dose = 1.
qPCR = quantitative Polymerase Chain Reaction; IP = Artesunate; ND = Not Detected; RBCs: red blood cells.

Listing 16.2.6.3
Malaria 18S qPCR

Randomisation Number	Challenge Agent	Visit	Timepoint	Date of Measurement	Time of Measurement	Day Relative to Inoculum	Day Relative to Artesunate	Assay	Result (parasites/500µL) of packed RBCs
R201	K13	Safety Monitoring		05-MAR-2018	08:54	14	6	Replicate 1	35
								Replicate 2	120
								Replicate 3	41
								Geometric Mean	55.6345
				06-MAR-2018	09:29	15	7	Replicate 1	ND
								Replicate 2	ND
								Replicate 3	ND
								Geometric Mean	1.0000
				07-MAR-2018	07:34	16	8	Replicate 1	82
								Replicate 2	41
								Replicate 3	38
								Geometric Mean	50.3648
				08-MAR-2018	08:59	17	9	Replicate 1	111
								Replicate 2	133
								Replicate 3	374
								Geometric Mean	176.7457

Notes: Day relative to inoculum is the number of days relative to day of administration of the inoculum, where the day of administration = 0.
The inoculum was administered on Study Day 0 for K13-infected subjects and Study Day 1 for 3D7-infected subjects.
Day relative to IP is the number of days relative to day of administration of Artesunate, where the day of dose = 1.
qPCR = quantitative Polymerase Chain Reaction; IP = Artesunate; ND = Not Detected; RBCs: red blood cells.

Listing 16.2.6.3
Malaria 18S qPCR

Randomisation Number	Challenge Agent	Visit	Timepoint	Date of Measurement	Time of Measurement	Day Relative to Inoculum	Day Relative to Artesunate	Assay	Result (parasites/500µL) of packed RBCs
R201	K13	Safety Monitoring		09-MAR-2018	09:59	18	10	Replicate 1	116
								Replicate 2	207
								Replicate 3	173
								Geometric Mean	160.7527
				12-MAR-2018	08:28	21	13	Replicate 1	266
								Replicate 2	524
								Replicate 3	280
								Geometric Mean	339.2009
				14-MAR-2018	09:06	23	15	Replicate 1	141
								Replicate 2	155
								Replicate 3	84
								Geometric Mean	122.4457
				16-MAR-2018	08:48	25	17	Replicate 1	99
								Replicate 2	ND
								Replicate 3	72
								Geometric Mean	19.2452

Notes: Day relative to inoculum is the number of days relative to day of administration of the inoculum, where the day of administration = 0.
The inoculum was administered on Study Day 0 for K13-infected subjects and Study Day 1 for 3D7-infected subjects.
Day relative to IP is the number of days relative to day of administration of Artesunate, where the day of dose = 1.
qPCR = quantitative Polymerase Chain Reaction; IP = Artesunate; ND = Not Detected; RBCs: red blood cells.

Listing 16.2.6.3
Malaria 18S qPCR

Randomisation Number	Challenge Agent	Visit	Timepoint	Date of Measurement	Time of Measurement	Day Relative to Inoculum	Day Relative to Artesunate	Assay	Result (parasites/500µL) of packed RBCs
R201	K13	Safety Monitoring		19-MAR-2018	08:19	28	20	Replicate 1	256
								Replicate 2	42
								Replicate 3	88
								Geometric Mean	98.1727
		End of Study		22-MAR-2018	08:31	31	23	Replicate 1	ND
								Replicate 2	ND
								Replicate 3	ND
								Geometric Mean	1.0000
R202	K13	Inoculum		19-FEB-2018	10:06	0	-9	Replicate 1	ND
								Replicate 2	ND
								Replicate 3	ND
								Geometric Mean	1.0000
		Malaria Monitoring		23-FEB-2018	07:17	4	-5	Replicate 1	50
								Replicate 2	125
								Replicate 3	91
								Geometric Mean	82.8528

Notes: Day relative to inoculum is the number of days relative to day of administration of the inoculum, where the day of administration = 0.
The inoculum was administered on Study Day 0 for K13-infected subjects and Study Day 1 for 3D7-infected subjects.
Day relative to IP is the number of days relative to day of administration of Artesunate, where the day of dose = 1.
qPCR = quantitative Polymerase Chain Reaction; IP = Artesunate; ND = Not Detected; RBCs: red blood cells.

Listing 16.2.6.3
Malaria 18S qPCR

Randomisation Number	Challenge Agent	Visit	Timepoint	Date of Measurement	Time of Measurement	Day Relative to Inoculum	Day Relative to Artesunate	Assay	Result (parasites/500µL) of packed RBCs
R202	K13	Malaria Monitoring		24-FEB-2018	09:03	5	-4	Replicate 1	67
								Replicate 2	158
								Replicate 3	44
								Geometric Mean	77.5166
				24-FEB-2018	20:37	5	-4	Replicate 1	395
								Replicate 2	814
								Replicate 3	949
								Geometric Mean	673.2286
				25-FEB-2018	08:47	6	-3	Replicate 1	1302
								Replicate 2	721
								Replicate 3	1038
								Geometric Mean	991.3976
				25-FEB-2018	20:40	6	-3	Replicate 1	1180
								Replicate 2	1271
								Replicate 3	1354
								Geometric Mean	1266.3354

Notes: Day relative to inoculum is the number of days relative to day of administration of the inoculum, where the day of administration = 0.
The inoculum was administered on Study Day 0 for K13-infected subjects and Study Day 1 for 3D7-infected subjects.
Day relative to IP is the number of days relative to day of administration of Artesunate, where the day of dose = 1.
qPCR = quantitative Polymerase Chain Reaction; IP = Artesunate; ND = Not Detected; RBCs: red blood cells.

Listing 16.2.6.3
Malaria 18S qPCR

Randomisation Number	Challenge Agent	Visit	Timepoint	Date of Measurement	Time of Measurement	Day Relative to Inoculum	Day Relative to Artesunate	Assay	Result (parasites/500µL) of packed RBCs
R202	K13	Malaria Monitoring		26-FEB-2018	08:54	7	-2	Replicate 1	3020
								Replicate 2	3337
								Replicate 3	2704
								Geometric Mean	3009.2385
				26-FEB-2018	20:16	7	-2	Replicate 1	19271
								Replicate 2	19706
								Replicate 3	20303
								Geometric Mean	19755.4828
				27-FEB-2018	09:12	8	-1	Replicate 1	23626
								Replicate 2	23754
								Replicate 3	19415
								Geometric Mean	22169.4430
				27-FEB-2018	20:39	8	-1	Replicate 1	22342
								Replicate 2	26538
								Replicate 3	24114
								Geometric Mean	24270.9345

Notes: Day relative to inoculum is the number of days relative to day of administration of the inoculum, where the day of administration = 0.
The inoculum was administered on Study Day 0 for K13-infected subjects and Study Day 1 for 3D7-infected subjects.
Day relative to IP is the number of days relative to day of administration of Artesunate, where the day of dose = 1.
qPCR = quantitative Polymerase Chain Reaction; IP = Artesunate; ND = Not Detected; RBCs: red blood cells.

Listing 16.2.6.3
Malaria 18S qPCR

Randomisation Number	Challenge Agent	Visit	Timepoint	Date of Measurement	Time of Measurement	Day Relative to Inoculum	Day Relative to Artesunate	Assay	Result (parasites/500µL) of packed RBCs
R202	K13	Admission	Pre-dose	28-FEB-2018	12:05	9	1	Replicate 1	118911
								Replicate 2	113915
								Replicate 3	121435
								Geometric Mean	118045.3655
			2 Hrs Post-dose	28-FEB-2018	15:04	9	1	Replicate 1	264791
								Replicate 2	148376
								Replicate 3	224536
								Geometric Mean	206625.6757
			4 Hrs Post-dose	28-FEB-2018	17:03	9	1	Replicate 1	233379
								Replicate 2	242954
								Replicate 3	259286
								Geometric Mean	244974.9243
			6 Hrs Post-dose	28-FEB-2018	19:03	9	1	Replicate 1	284962
								Replicate 2	337876
								Replicate 3	306600
								Geometric Mean	309057.1639

Notes: Day relative to inoculum is the number of days relative to day of administration of the inoculum, where the day of administration = 0.
The inoculum was administered on Study Day 0 for K13-infected subjects and Study Day 1 for 3D7-infected subjects.
Day relative to IP is the number of days relative to day of administration of Artesunate, where the day of dose = 1.
qPCR = quantitative Polymerase Chain Reaction; IP = Artesunate; ND = Not Detected; RBCs: red blood cells.

Listing 16.2.6.3
Malaria 18S qPCR

Randomisation Number	Challenge Agent	Visit	Timepoint	Date of Measurement	Time of Measurement	Day Relative to Inoculum	Day Relative to Artesunate	Assay	Result (parasites/500µL) of packed RBCs
R202	K13	Admission	8 Hrs Post-dose	28-FEB-2018	21:03	9	1	Replicate 1	268040
								Replicate 2	268889
								Replicate 3	273422
								Geometric Mean	270106.7062
		Admission 1	12 Hrs Post-dose	01-MAR-2018	01:03	10	2	Replicate 1	171068
								Replicate 2	210489
								Replicate 3	183104
								Geometric Mean	187513.2689
			16 Hrs Post-dose	01-MAR-2018	05:04	10	2	Replicate 1	120888
								Replicate 2	120452
								Replicate 3	121271
								Geometric Mean	120869.8702
			20 Hrs Post-dose	01-MAR-2018	09:03	10	2	Replicate 1	86632
								Replicate 2	86437
								Replicate 3	89194
								Geometric Mean	87412.0332

Notes: Day relative to inoculum is the number of days relative to day of administration of the inoculum, where the day of administration = 0.
The inoculum was administered on Study Day 0 for K13-infected subjects and Study Day 1 for 3D7-infected subjects.
Day relative to IP is the number of days relative to day of administration of Artesunate, where the day of dose = 1.
qPCR = quantitative Polymerase Chain Reaction; IP = Artesunate; ND = Not Detected; RBCs: red blood cells.

Listing 16.2.6.3
Malaria 18S qPCR

Randomisation Number	Challenge Agent	Visit	Timepoint	Date of Measurement	Time of Measurement	Day Relative to Inoculum	Day Relative to Artesunate	Assay	Result (parasites/500μL) of packed RBCs
R202	K13	Admission 1	24 Hrs Post-dose	01-MAR-2018	13:04	10	2	Replicate 1	79650
								Replicate 2	82469
								Replicate 3	67434
								Geometric Mean	76228.6936
			28 Hrs Post-dose	01-MAR-2018	17:09	10	2	Replicate 1	35928
								Replicate 2	35790
								Replicate 3	37733
								Geometric Mean	36473.0452
			32 Hrs Post-dose	01-MAR-2018	21:07	10	2	Replicate 1	7273
								Replicate 2	8363
								Replicate 3	7346
								Geometric Mean	7644.9656
		Admission 2	36 Hrs Post-dose	02-MAR-2018	01:03	11	3	Replicate 1	4076
								Replicate 2	3260
								Replicate 3	4733
								Geometric Mean	3976.7604

Notes: Day relative to inoculum is the number of days relative to day of administration of the inoculum, where the day of administration = 0.
The inoculum was administered on Study Day 0 for K13-infected subjects and Study Day 1 for 3D7-infected subjects.
Day relative to IP is the number of days relative to day of administration of Artesunate, where the day of dose = 1.
qPCR = quantitative Polymerase Chain Reaction; IP = Artesunate; ND = Not Detected; RBCs: red blood cells.

Listing 16.2.6.3
Malaria 18S qPCR

Randomisation Number	Challenge Agent	Visit	Timepoint	Date of Measurement	Time of Measurement	Day Relative to Inoculum	Day Relative to Artesunate	Assay	Result (parasites/500µL) of packed RBCs
R202	K13	Admission 2	48 Hrs Post-dose	02-MAR-2018	13:03	11	3	Replicate 1	396206
								Replicate 2	457523
								Replicate 3	369994
								Geometric Mean	406296.3183
			52 Hrs Post-dose	02-MAR-2018	17:04	11	3	Replicate 1	946055
								Replicate 2	846160
								Replicate 3	1040678
								Geometric Mean	940939.6079
			56 Hrs Post-dose	02-MAR-2018	21:03	11	3	Replicate 1	1111134
								Replicate 2	1087789
								Replicate 3	1080442
								Geometric Mean	1093043.6846
		Admission 3	60 Hrs Post-dose	03-MAR-2018	01:03	12	4	Replicate 1	1149948
								Replicate 2	1131396
								Replicate 3	1021578
								Geometric Mean	1099482.0014

Notes: Day relative to inoculum is the number of days relative to day of administration of the inoculum, where the day of administration = 0.
The inoculum was administered on Study Day 0 for K13-infected subjects and Study Day 1 for 3D7-infected subjects.
Day relative to IP is the number of days relative to day of administration of Artesunate, where the day of dose = 1.
qPCR = quantitative Polymerase Chain Reaction; IP = Artesunate; ND = Not Detected; RBCs: red blood cells.

Listing 16.2.6.3
Malaria 18S qPCR

Randomisation Number	Challenge Agent	Visit	Timepoint	Date of Measurement	Time of Measurement	Day Relative to Inoculum	Day Relative to Artesunate	Assay	Result (parasites/500µL) of packed RBCs
R202	K13	Admission 3	72 Hrs Post-dose	03-MAR-2018	13:05	12	4	Replicate 1	492784
								Replicate 2	420806
								Replicate 3	461120
								Geometric Mean	457282.0566
				03-MAR-2018	20:07	12	4	Replicate 1	112882
								Replicate 2	123864
								Replicate 3	113778
								Geometric Mean	116737.2246
		Safety Monitoring		04-MAR-2018	09:38	13	5	Replicate 1	14397
								Replicate 2	17583
								Replicate 3	13088
								Geometric Mean	14907.7725
				04-MAR-2018	20:38	13	5	Replicate 1	2885
								Replicate 2	3545
								Replicate 3	3279
								Geometric Mean	3224.7879

Notes: Day relative to inoculum is the number of days relative to day of administration of the inoculum, where the day of administration = 0.
The inoculum was administered on Study Day 0 for K13-infected subjects and Study Day 1 for 3D7-infected subjects.
Day relative to IP is the number of days relative to day of administration of Artesunate, where the day of dose = 1.
qPCR = quantitative Polymerase Chain Reaction; IP = Artesunate; ND = Not Detected; RBCs: red blood cells.

Listing 16.2.6.3
Malaria 18S qPCR

Randomisation Number	Challenge Agent	Visit	Timepoint	Date of Measurement	Time of Measurement	Day Relative to Inoculum	Day Relative to Artesunate	Assay	Result (parasites/500µL) of packed RBCs
R202	K13	Safety Monitoring		05-MAR-2018	09:59	14	6	Replicate 1	2070
								Replicate 2	1565
								Replicate 3	2242
								Geometric Mean	1936.6008
				06-MAR-2018	09:51	15	7	Replicate 1	342
								Replicate 2	412
								Replicate 3	216
								Geometric Mean	312.2188
				07-MAR-2018	09:07	16	8	Replicate 1	552
								Replicate 2	703
								Replicate 3	304
								Geometric Mean	490.4439
				08-MAR-2018	09:17	17	9	Replicate 1	404
								Replicate 2	463
								Replicate 3	547
								Geometric Mean	467.7171

Notes: Day relative to inoculum is the number of days relative to day of administration of the inoculum, where the day of administration = 0.
The inoculum was administered on Study Day 0 for K13-infected subjects and Study Day 1 for 3D7-infected subjects.
Day relative to IP is the number of days relative to day of administration of Artesunate, where the day of dose = 1.
qPCR = quantitative Polymerase Chain Reaction; IP = Artesunate; ND = Not Detected; RBCs: red blood cells.

Listing 16.2.6.3
Malaria 18S qPCR

Randomisation Number	Challenge Agent	Visit	Timepoint	Date of Measurement	Time of Measurement	Day Relative to Inoculum	Day Relative to Artesunate	Assay	Result (parasites/500µL) of packed RBCs
R202	K13	Safety Monitoring		09-MAR-2018	09:32	18	10	Replicate 1	1128
								Replicate 2	933
								Replicate 3	1379
								Geometric Mean	1132.1875
				10-MAR-2018	08:52	19	11	Replicate 1	3937
								Replicate 2	3391
								Replicate 3	3884
								Geometric Mean	3728.9865
				11-MAR-2018	08:52	20	12	Replicate 1	1988
								Replicate 2	3294
								Replicate 3	2255
								Geometric Mean	2453.3650
				12-MAR-2018	09:51	21	13	Replicate 1	1599
								Replicate 2	1971
								Replicate 3	1626
								Geometric Mean	1724.0580

Notes: Day relative to inoculum is the number of days relative to day of administration of the inoculum, where the day of administration = 0.
The inoculum was administered on Study Day 0 for K13-infected subjects and Study Day 1 for 3D7-infected subjects.
Day relative to IP is the number of days relative to day of administration of Artesunate, where the day of dose = 1.
qPCR = quantitative Polymerase Chain Reaction; IP = Artesunate; ND = Not Detected; RBCs: red blood cells.

Listing 16.2.6.3
Malaria 18S qPCR

Randomisation Number	Challenge Agent	Visit	Timepoint	Date of Measurement	Time of Measurement	Day Relative to Inoculum	Day Relative to Artesunate	Assay	Result (parasites/500µL) of packed RBCs
R202	K13	Safety Monitoring		14-MAR-2018	08:51	23	15	Replicate 1	1545
								Replicate 2	1085
								Replicate 3	1621
								Geometric Mean	1395.4483
				16-MAR-2018	09:35	25	17	Replicate 1	628
								Replicate 2	580
								Replicate 3	412
								Geometric Mean	531.4082
				19-MAR-2018	09:12	28	20	Replicate 1	477
								Replicate 2	343
								Replicate 3	590
								Geometric Mean	458.7276
		End of Study		22-MAR-2018	10:00	31	23	Replicate 1	236
								Replicate 2	243
								Replicate 3	255
								Geometric Mean	244.5416

Notes: Day relative to inoculum is the number of days relative to day of administration of the inoculum, where the day of administration = 0.
The inoculum was administered on Study Day 0 for K13-infected subjects and Study Day 1 for 3D7-infected subjects.
Day relative to IP is the number of days relative to day of administration of Artesunate, where the day of dose = 1.
qPCR = quantitative Polymerase Chain Reaction; IP = Artesunate; ND = Not Detected; RBCs: red blood cells.

Listing 16.2.6.3
Malaria 18S qPCR

Randomisation Number	Challenge Agent	Visit	Timepoint	Date of Measurement	Time of Measurement	Day Relative to Inoculum	Day Relative to Artesunate	Assay	Result (parasites/500µL) of packed RBCs
R202	K13	Unscheduled		27-MAR-2018	09:00	36	28	Replicate 1	44
								Replicate 2	45
								Replicate 3	ND
								Geometric Mean	12.5571
				03-APR-2018	09:10	43	35	Replicate 1	ND
								Replicate 2	ND
								Replicate 3	ND
								Geometric Mean	1.0000
R203	K13	Inoculum		19-FEB-2018	10:14	0	-9	Replicate 1	ND
								Replicate 2	ND
								Replicate 3	ND
								Geometric Mean	1.0000
		Malaria Monitoring		23-FEB-2018	09:09	4	-5	Replicate 1	90
								Replicate 2	100
								Replicate 3	172
								Geometric Mean	115.6797

Notes: Day relative to inoculum is the number of days relative to day of administration of the inoculum, where the day of administration = 0.
The inoculum was administered on Study Day 0 for K13-infected subjects and Study Day 1 for 3D7-infected subjects.
Day relative to IP is the number of days relative to day of administration of Artesunate, where the day of dose = 1.
qPCR = quantitative Polymerase Chain Reaction; IP = Artesunate; ND = Not Detected; RBCs: red blood cells.

Listing 16.2.6.3
Malaria 18S qPCR

Randomisation Number	Challenge Agent	Visit	Timepoint	Date of Measurement	Time of Measurement	Day Relative to Inoculum	Day Relative to Artesunate	Assay	Result (parasites/500µL) of packed RBCs
R203	K13	Malaria Monitoring		24-FEB-2018	08:54	5	-4	Replicate 1	297
								Replicate 2	125
								Replicate 3	355
								Geometric Mean	236.2100
				24-FEB-2018	19:41	5	-4	Replicate 1	2214
								Replicate 2	1837
								Replicate 3	1606
								Geometric Mean	1869.2933
				25-FEB-2018	09:03	6	-3	Replicate 1	2130
								Replicate 2	2629
								Replicate 3	2614
								Geometric Mean	2446.1998
				25-FEB-2018	21:32	6	-3	Replicate 1	2632
								Replicate 2	2858
								Replicate 3	3932
								Geometric Mean	3092.5771

Notes: Day relative to inoculum is the number of days relative to day of administration of the inoculum, where the day of administration = 0.
The inoculum was administered on Study Day 0 for K13-infected subjects and Study Day 1 for 3D7-infected subjects.
Day relative to IP is the number of days relative to day of administration of Artesunate, where the day of dose = 1.
qPCR = quantitative Polymerase Chain Reaction; IP = Artesunate; ND = Not Detected; RBCs: red blood cells.

Listing 16.2.6.3
Malaria 18S qPCR

Randomisation Number	Challenge Agent	Visit	Timepoint	Date of Measurement	Time of Measurement	Day Relative to Inoculum	Day Relative to Artesunate	Assay	Result (parasites/500µL) of packed RBCs
R203	K13	Malaria Monitoring		26-FEB-2018	09:14	7	-2	Replicate 1	10825
								Replicate 2	11827
								Replicate 3	11133
								Geometric Mean	11253.9480
				26-FEB-2018	20:35	7	-2	Replicate 1	50673
								Replicate 2	51621
								Replicate 3	52007
								Geometric Mean	51430.6024
				27-FEB-2018	08:52	8	-1	Replicate 1	81561
								Replicate 2	75805
								Replicate 3	65601
								Geometric Mean	74022.4777
				27-FEB-2018	19:52	8	-1	Replicate 1	105915
								Replicate 2	101351
								Replicate 3	105557
								Geometric Mean	104253.5492

Notes: Day relative to inoculum is the number of days relative to day of administration of the inoculum, where the day of administration = 0.
The inoculum was administered on Study Day 0 for K13-infected subjects and Study Day 1 for 3D7-infected subjects.
Day relative to IP is the number of days relative to day of administration of Artesunate, where the day of dose = 1.
qPCR = quantitative Polymerase Chain Reaction; IP = Artesunate; ND = Not Detected; RBCs: red blood cells.

Listing 16.2.6.3
Malaria 18S qPCR

Randomisation Number	Challenge Agent	Visit	Timepoint	Date of Measurement	Time of Measurement	Day Relative to Inoculum	Day Relative to Artesunate	Assay	Result (parasites/500µL) of packed RBCs
R203	K13	Admission	Pre-dose	28-FEB-2018	11:14	9	1	Replicate 1	348574
								Replicate 2	329733
								Replicate 3	361322
								Geometric Mean	346298.4943
			2 Hrs Post-dose	28-FEB-2018	13:40	9	1	Replicate 1	488388
								Replicate 2	504194
								Replicate 3	483558
								Geometric Mean	491968.2512
			4 Hrs Post-dose	28-FEB-2018	15:40	9	1	Replicate 1	751363
								Replicate 2	744941
								Replicate 3	702766
								Geometric Mean	732702.1651
			6 Hrs Post-dose	28-FEB-2018	17:41	9	1	Replicate 1	859064
								Replicate 2	750345
								Replicate 3	796454
								Geometric Mean	800723.1846

Notes: Day relative to inoculum is the number of days relative to day of administration of the inoculum, where the day of administration = 0.
The inoculum was administered on Study Day 0 for K13-infected subjects and Study Day 1 for 3D7-infected subjects.
Day relative to IP is the number of days relative to day of administration of Artesunate, where the day of dose = 1.
qPCR = quantitative Polymerase Chain Reaction; IP = Artesunate; ND = Not Detected; RBCs: red blood cells.

Listing 16.2.6.3
Malaria 18S qPCR

Randomisation Number	Challenge Agent	Visit	Timepoint	Date of Measurement	Time of Measurement	Day Relative to Inoculum	Day Relative to Artesunate	Assay	Result (parasites/500µL) of packed RBCs
R203	K13	Admission	8 Hrs Post-dose	28-FEB-2018	19:40	9	1	Replicate 1	769050
								Replicate 2	663579
								Replicate 3	752892
								Geometric Mean	726986.9766
			12 Hrs Post-dose	28-FEB-2018	23:40	9	1	Replicate 1	529047
								Replicate 2	555501
								Replicate 3	530004
								Geometric Mean	538046.0112
		Admission 1	16 Hrs Post-dose	01-MAR-2018	03:41	10	2	Replicate 1	350232
								Replicate 2	316380
								Replicate 3	353491
								Geometric Mean	339610.4685
			20 Hrs Post-dose	01-MAR-2018	07:44	10	2	Replicate 1	268040
								Replicate 2	268525
								Replicate 3	267194
								Geometric Mean	267919.1019

Notes: Day relative to inoculum is the number of days relative to day of administration of the inoculum, where the day of administration = 0.
The inoculum was administered on Study Day 0 for K13-infected subjects and Study Day 1 for 3D7-infected subjects.
Day relative to IP is the number of days relative to day of administration of Artesunate, where the day of dose = 1.
qPCR = quantitative Polymerase Chain Reaction; IP = Artesunate; ND = Not Detected; RBCs: red blood cells.

Listing 16.2.6.3
Malaria 18S qPCR

Randomisation Number	Challenge Agent	Visit	Timepoint	Date of Measurement	Time of Measurement	Day Relative to Inoculum	Day Relative to Artesunate	Assay	Result (parasites/500µL) of packed RBCs
R203	K13	Admission 1	24 Hrs Post-dose	01-MAR-2018	11:40	10	2	Replicate 1	160801
								Replicate 2	157103
								Replicate 3	154185
								Geometric Mean	157339.7478
			28 Hrs Post-dose	01-MAR-2018	15:40	10	2	Replicate 1	106901
								Replicate 2	106853
								Replicate 3	99581
								Geometric Mean	104387.4584
			32 Hrs Post-dose	01-MAR-2018	19:40	10	2	Replicate 1	37166
								Replicate 2	34426
								Replicate 3	39352
								Geometric Mean	36926.0680
			36 Hrs Post-dose	01-MAR-2018	23:40	10	2	Replicate 1	13706
								Replicate 2	14112
								Replicate 3	13045
								Geometric Mean	13613.8552

Notes: Day relative to inoculum is the number of days relative to day of administration of the inoculum, where the day of administration = 0.
The inoculum was administered on Study Day 0 for K13-infected subjects and Study Day 1 for 3D7-infected subjects.
Day relative to IP is the number of days relative to day of administration of Artesunate, where the day of dose = 1.
qPCR = quantitative Polymerase Chain Reaction; IP = Artesunate; ND = Not Detected; RBCs: red blood cells.

Listing 16.2.6.3
Malaria 18S qPCR

Randomisation Number	Challenge Agent	Visit	Timepoint	Date of Measurement	Time of Measurement	Day Relative to Inoculum	Day Relative to Artesunate	Assay	Result (parasites/500µL) of packed RBCs
R203	K13	Admission 2	48 Hrs Post-dose	02-MAR-2018	11:49	11	3	Replicate 1	660140
								Replicate 2	683355
								Replicate 3	584464
								Geometric Mean	641229.3569
			52 Hrs Post-dose	02-MAR-2018	15:42	11	3	Replicate 1	1413023
								Replicate 2	1308561
								Replicate 3	1317756
								Geometric Mean	1345631.2633
			56 Hrs Post-dose	02-MAR-2018	19:41	11	3	Replicate 1	1088280
								Replicate 2	1186896
								Replicate 3	1325818
								Geometric Mean	1196407.3572
			60 Hrs Post-dose	02-MAR-2018	23:42	11	3	Replicate 1	1331821
								Replicate 2	1359175
								Replicate 3	1420063
								Geometric Mean	1369860.3101

Notes: Day relative to inoculum is the number of days relative to day of administration of the inoculum, where the day of administration = 0.
The inoculum was administered on Study Day 0 for K13-infected subjects and Study Day 1 for 3D7-infected subjects.
Day relative to IP is the number of days relative to day of administration of Artesunate, where the day of dose = 1.
qPCR = quantitative Polymerase Chain Reaction; IP = Artesunate; ND = Not Detected; RBCs: red blood cells.

Listing 16.2.6.3
Malaria 18S qPCR

Randomisation Number	Challenge Agent	Visit	Timepoint	Date of Measurement	Time of Measurement	Day Relative to Inoculum	Day Relative to Artesunate	Assay	Result (parasites/500µL) of packed RBCs
R203	K13	Admission 3	72 Hrs Post-dose	03-MAR-2018	11:50	12	4	Replicate 1	264950
								Replicate 2	244091
								Replicate 3	317788
								Geometric Mean	273916.2063
				03-MAR-2018	20:57	12	4	Replicate 1	75526
								Replicate 2	83532
								Replicate 3	85575
								Geometric Mean	81426.4356
		Safety Monitoring		04-MAR-2018	09:08	13	5	Replicate 1	7255
								Replicate 2	8049
								Replicate 3	6985
								Geometric Mean	7416.2083
				04-MAR-2018	20:20	13	5	Replicate 1	3277
								Replicate 2	2438
								Replicate 3	2902
								Geometric Mean	2851.4725

Notes: Day relative to inoculum is the number of days relative to day of administration of the inoculum, where the day of administration = 0.
The inoculum was administered on Study Day 0 for K13-infected subjects and Study Day 1 for 3D7-infected subjects.
Day relative to IP is the number of days relative to day of administration of Artesunate, where the day of dose = 1.
qPCR = quantitative Polymerase Chain Reaction; IP = Artesunate; ND = Not Detected; RBCs: red blood cells.

Listing 16.2.6.3
Malaria 18S qPCR

Randomisation Number	Challenge Agent	Visit	Timepoint	Date of Measurement	Time of Measurement	Day Relative to Inoculum	Day Relative to Artesunate	Assay	Result (parasites/500µL) of packed RBCs
R203	K13	Safety Monitoring		05-MAR-2018	09:40	14	6	Replicate 1	1400
								Replicate 2	1910
								Replicate 3	1369
								Geometric Mean	1541.1856
				06-MAR-2018	09:29	15	7	Replicate 1	446
								Replicate 2	387
								Replicate 3	288
								Geometric Mean	367.6880
				07-MAR-2018	10:04	16	8	Replicate 1	689
								Replicate 2	422
								Replicate 3	625
								Geometric Mean	566.4182
				08-MAR-2018	10:00	17	9	Replicate 1	956
								Replicate 2	1251
								Replicate 3	1160
								Geometric Mean	1115.2984

Notes: Day relative to inoculum is the number of days relative to day of administration of the inoculum, where the day of administration = 0.
The inoculum was administered on Study Day 0 for K13-infected subjects and Study Day 1 for 3D7-infected subjects.
Day relative to IP is the number of days relative to day of administration of Artesunate, where the day of dose = 1.
qPCR = quantitative Polymerase Chain Reaction; IP = Artesunate; ND = Not Detected; RBCs: red blood cells.

Listing 16.2.6.3
Malaria 18S qPCR

Randomisation Number	Challenge Agent	Visit	Timepoint	Date of Measurement	Time of Measurement	Day Relative to Inoculum	Day Relative to Artesunate	Assay	Result (parasites/500µL) of packed RBCs
R203	K13	Safety Monitoring		09-MAR-2018	10:14	18	10	Replicate 1	3903
								Replicate 2	4597
								Replicate 3	4031
								Geometric Mean	4166.4095
				10-MAR-2018	08:37	19	11	Replicate 1	10120
								Replicate 2	8983
								Replicate 3	10742
								Geometric Mean	9921.1577
				12-MAR-2018	09:17	21	13	Replicate 1	6144
								Replicate 2	6259
								Replicate 3	4645
								Geometric Mean	5631.8009
				13-MAR-2018	09:41	22	14	Replicate 1	6014
								Replicate 2	6003
								Replicate 3	6868
								Geometric Mean	6282.3291

Notes: Day relative to inoculum is the number of days relative to day of administration of the inoculum, where the day of administration = 0.
The inoculum was administered on Study Day 0 for K13-infected subjects and Study Day 1 for 3D7-infected subjects.
Day relative to IP is the number of days relative to day of administration of Artesunate, where the day of dose = 1.
qPCR = quantitative Polymerase Chain Reaction; IP = Artesunate; ND = Not Detected; RBCs: red blood cells.

Listing 16.2.6.3
Malaria 18S qPCR

Randomisation Number	Challenge Agent	Visit	Timepoint	Date of Measurement	Time of Measurement	Day Relative to Inoculum	Day Relative to Artesunate	Assay	Result (parasites/500µL) of packed RBCs
R203	K13	Safety Monitoring		14-MAR-2018	10:15	23	15	Replicate 1	3294
								Replicate 2	3361
								Replicate 3	4641
								Geometric Mean	3717.6448
				16-MAR-2018	09:03	25	17	Replicate 1	3409
								Replicate 2	2920
								Replicate 3	3354
								Geometric Mean	3220.0131
				19-MAR-2018	10:10	28	20	Replicate 1	221
								Replicate 2	277
								Replicate 3	128
								Geometric Mean	198.6220
		End of Study		22-MAR-2018	10:14	31	23	Replicate 1	497
								Replicate 2	454
								Replicate 3	444
								Geometric Mean	464.4423

Notes: Day relative to inoculum is the number of days relative to day of administration of the inoculum, where the day of administration = 0.
The inoculum was administered on Study Day 0 for K13-infected subjects and Study Day 1 for 3D7-infected subjects.
Day relative to IP is the number of days relative to day of administration of Artesunate, where the day of dose = 1.
qPCR = quantitative Polymerase Chain Reaction; IP = Artesunate; ND = Not Detected; RBCs: red blood cells.

Listing 16.2.6.3
Malaria 18S qPCR

Randomisation Number	Challenge Agent	Visit	Timepoint	Date of Measurement	Time of Measurement	Day Relative to Inoculum	Day Relative to Artesunate	Assay	Result (parasites/500µL) of packed RBCs
R203	K13	Unscheduled		28-MAR-2018	09:53	37	29	Replicate 1	158
								Replicate 2	ND
								Replicate 3	ND
								Geometric Mean	5.4061
				02-APR-2018	09:18	42	34	Replicate 1	ND
								Replicate 2	ND
								Replicate 3	ND
								Geometric Mean	1.0000
R204	K13	Inoculum		19-FEB-2018	10:30	0	-9	Replicate 1	ND
								Replicate 2	ND
								Replicate 3	ND
								Geometric Mean	1.0000
		Malaria Monitoring		23-FEB-2018	08:49	4	-5	Replicate 1	21
								Replicate 2	37
								Replicate 3	ND
								Geometric Mean	9.1933

Notes: Day relative to inoculum is the number of days relative to day of administration of the inoculum, where the day of administration = 0.
The inoculum was administered on Study Day 0 for K13-infected subjects and Study Day 1 for 3D7-infected subjects.
Day relative to IP is the number of days relative to day of administration of Artesunate, where the day of dose = 1.
qPCR = quantitative Polymerase Chain Reaction; IP = Artesunate; ND = Not Detected; RBCs: red blood cells.

Listing 16.2.6.3
Malaria 18S qPCR

Randomisation Number	Challenge Agent	Visit	Timepoint	Date of Measurement	Time of Measurement	Day Relative to Inoculum	Day Relative to Artesunate	Assay	Result (parasites/500µL) of packed RBCs
R204	K13	Malaria Monitoring		24-FEB-2018	07:28	5	-4	Replicate 1	40
								Replicate 2	ND
								Replicate 3	ND
								Geometric Mean	3.4200
				24-FEB-2018	19:31	5	-4	Replicate 1	195
								Replicate 2	224
								Replicate 3	146
								Geometric Mean	185.4436
				25-FEB-2018	08:38	6	-3	Replicate 1	156
								Replicate 2	266
								Replicate 3	237
								Geometric Mean	214.2487
				25-FEB-2018	20:08	6	-3	Replicate 1	483
								Replicate 2	699
								Replicate 3	592
								Geometric Mean	584.6761

Notes: Day relative to inoculum is the number of days relative to day of administration of the inoculum, where the day of administration = 0.
The inoculum was administered on Study Day 0 for K13-infected subjects and Study Day 1 for 3D7-infected subjects.
Day relative to IP is the number of days relative to day of administration of Artesunate, where the day of dose = 1.
qPCR = quantitative Polymerase Chain Reaction; IP = Artesunate; ND = Not Detected; RBCs: red blood cells.

Listing 16.2.6.3
Malaria 18S qPCR

Randomisation Number	Challenge Agent	Visit	Timepoint	Date of Measurement	Time of Measurement	Day Relative to Inoculum	Day Relative to Artesunate	Assay	Result (parasites/500µL) of packed RBCs
R204	K13	Malaria Monitoring		26-FEB-2018	08:14	7	-2	Replicate 1	962
								Replicate 2	1227
								Replicate 3	1453
								Geometric Mean	1197.0026
				26-FEB-2018	19:42	7	-2	Replicate 1	2884
								Replicate 2	3646
								Replicate 3	3411
								Geometric Mean	3297.8524
				27-FEB-2018	08:03	8	-1	Replicate 1	8719
								Replicate 2	7851
								Replicate 3	8609
								Geometric Mean	8383.9392
				27-FEB-2018	19:36	8	-1	Replicate 1	6303
								Replicate 2	7050
								Replicate 3	7088
								Geometric Mean	6803.8288

Notes: Day relative to inoculum is the number of days relative to day of administration of the inoculum, where the day of administration = 0.
The inoculum was administered on Study Day 0 for K13-infected subjects and Study Day 1 for 3D7-infected subjects.
Day relative to IP is the number of days relative to day of administration of Artesunate, where the day of dose = 1.
qPCR = quantitative Polymerase Chain Reaction; IP = Artesunate; ND = Not Detected; RBCs: red blood cells.

Listing 16.2.6.3
Malaria 18S qPCR

Randomisation Number	Challenge Agent	Visit	Timepoint	Date of Measurement	Time of Measurement	Day Relative to Inoculum	Day Relative to Artesunate	Assay	Result (parasites/500µL) of packed RBCs
R204	K13	Admission	Pre-dose	28-FEB-2018	12:29	9	1	Replicate 1	35640
								Replicate 2	32752
								Replicate 3	31454
								Geometric Mean	33236.6416
			2 Hrs Post-dose	28-FEB-2018	15:15	9	1	Replicate 1	37915
								Replicate 2	38799
								Replicate 3	35375
								Geometric Mean	37334.4147
			4 Hrs Post-dose	28-FEB-2018	17:15	9	1	Replicate 1	36093
								Replicate 2	35874
								Replicate 3	35407
								Geometric Mean	35790.1874
			6 Hrs Post-dose	28-FEB-2018	19:15	9	1	Replicate 1	61581
								Replicate 2	58728
								Replicate 3	57054
								Geometric Mean	59091.6362

Notes: Day relative to inoculum is the number of days relative to day of administration of the inoculum, where the day of administration = 0.
The inoculum was administered on Study Day 0 for K13-infected subjects and Study Day 1 for 3D7-infected subjects.
Day relative to IP is the number of days relative to day of administration of Artesunate, where the day of dose = 1.
qPCR = quantitative Polymerase Chain Reaction; IP = Artesunate; ND = Not Detected; RBCs: red blood cells.

Listing 16.2.6.3
Malaria 18S qPCR

Randomisation Number	Challenge Agent	Visit	Timepoint	Date of Measurement	Time of Measurement	Day Relative to Inoculum	Day Relative to Artesunate	Assay	Result (parasites/500µL) of packed RBCs
R204	K13	Admission	8 Hrs Post-dose	28-FEB-2018	21:17	9	1	Replicate 1	28089
								Replicate 2	28945
								Replicate 3	32619
								Geometric Mean	29821.3174
		Admission 1	12 Hrs Post-dose	01-MAR-2018	01:15	10	2	Replicate 1	24880
								Replicate 2	21117
								Replicate 3	24330
								Geometric Mean	23381.6363
			16 Hrs Post-dose	01-MAR-2018	05:16	10	2	Replicate 1	12958
								Replicate 2	14084
								Replicate 3	11509
								Geometric Mean	12806.6005
			20 Hrs Post-dose	01-MAR-2018	09:16	10	2	Replicate 1	11608
								Replicate 2	9404
								Replicate 3	10691
								Geometric Mean	10528.4099

Notes: Day relative to inoculum is the number of days relative to day of administration of the inoculum, where the day of administration = 0.
The inoculum was administered on Study Day 0 for K13-infected subjects and Study Day 1 for 3D7-infected subjects.
Day relative to IP is the number of days relative to day of administration of Artesunate, where the day of dose = 1.
qPCR = quantitative Polymerase Chain Reaction; IP = Artesunate; ND = Not Detected; RBCs: red blood cells.

Listing 16.2.6.3
Malaria 18S qPCR

Randomisation Number	Challenge Agent	Visit	Timepoint	Date of Measurement	Time of Measurement	Day Relative to Inoculum	Day Relative to Artesunate	Assay	Result (parasites/500µL) of packed RBCs
R204	K13	Admission 1	24 Hrs Post-dose	01-MAR-2018	13:21	10	2	Replicate 1	4581
								Replicate 2	6729
								Replicate 3	5631
								Geometric Mean	5578.2604
			28 Hrs Post-dose	01-MAR-2018	17:33	10	2	Replicate 1	3201
								Replicate 2	3311
								Replicate 3	2931
								Geometric Mean	3143.5489
			32 Hrs Post-dose	01-MAR-2018	21:27	10	2	Replicate 1	816
								Replicate 2	925
								Replicate 3	1707
								Geometric Mean	1088.1493
		Admission 2	48 Hrs Post-dose	02-MAR-2018	13:17	11	3	Replicate 1	38078
								Replicate 2	37329
								Replicate 3	35761
								Geometric Mean	37043.3322

Notes: Day relative to inoculum is the number of days relative to day of administration of the inoculum, where the day of administration = 0.
The inoculum was administered on Study Day 0 for K13-infected subjects and Study Day 1 for 3D7-infected subjects.
Day relative to IP is the number of days relative to day of administration of Artesunate, where the day of dose = 1.
qPCR = quantitative Polymerase Chain Reaction; IP = Artesunate; ND = Not Detected; RBCs: red blood cells.

Listing 16.2.6.3
Malaria 18S qPCR

Randomisation Number	Challenge Agent	Visit	Timepoint	Date of Measurement	Time of Measurement	Day Relative to Inoculum	Day Relative to Artesunate	Assay	Result (parasites/500µL) of packed RBCs
R204	K13	Admission 2	52 Hrs Post-dose	02-MAR-2018	17:18	11	3	Replicate 1	50206
								Replicate 2	49530
								Replicate 3	47772
								Geometric Mean	49158.5568
			56 Hrs Post-dose	02-MAR-2018	21:22	11	3	Replicate 1	73479
								Replicate 2	67801
								Replicate 3	73945
								Geometric Mean	71686.2809
		Admission 3	60 Hrs Post-dose	03-MAR-2018	01:27	12	4	Replicate 1	55128
								Replicate 2	58305
								Replicate 3	73048
								Geometric Mean	61691.9913
			72 Hrs Post-dose	03-MAR-2018	13:21	12	4	Replicate 1	14153
								Replicate 2	14272
								Replicate 3	15537
								Geometric Mean	14640.8684

Notes: Day relative to inoculum is the number of days relative to day of administration of the inoculum, where the day of administration = 0.
The inoculum was administered on Study Day 0 for K13-infected subjects and Study Day 1 for 3D7-infected subjects.
Day relative to IP is the number of days relative to day of administration of Artesunate, where the day of dose = 1.
qPCR = quantitative Polymerase Chain Reaction; IP = Artesunate; ND = Not Detected; RBCs: red blood cells.

Listing 16.2.6.3
Malaria 18S qPCR

Randomisation Number	Challenge Agent	Visit	Timepoint	Date of Measurement	Time of Measurement	Day Relative to Inoculum	Day Relative to Artesunate	Assay	Result (parasites/500µL) of packed RBCs
R204	K13	Admission 3		03-MAR-2018	20:45	12	4	Replicate 1	3066
								Replicate 2	3782
								Replicate 3	3634
								Geometric Mean	3479.8421
		Safety Monitoring		04-MAR-2018	09:59	13	5	Replicate 1	599
								Replicate 2	293
								Replicate 3	446
								Geometric Mean	427.7695
				04-MAR-2018	19:39	13	5	Replicate 1	124
								Replicate 2	44
								Replicate 3	72
								Geometric Mean	73.2379
				05-MAR-2018	08:37	14	6	Replicate 1	105
								Replicate 2	109
								Replicate 3	ND
								Geometric Mean	22.5357

Notes: Day relative to inoculum is the number of days relative to day of administration of the inoculum, where the day of administration = 0.
The inoculum was administered on Study Day 0 for K13-infected subjects and Study Day 1 for 3D7-infected subjects.
Day relative to IP is the number of days relative to day of administration of Artesunate, where the day of dose = 1.
qPCR = quantitative Polymerase Chain Reaction; IP = Artesunate; ND = Not Detected; RBCs: red blood cells.

Listing 16.2.6.3
Malaria 18S qPCR

Randomisation Number	Challenge Agent	Visit	Timepoint	Date of Measurement	Time of Measurement	Day Relative to Inoculum	Day Relative to Artesunate	Assay	Result (parasites/500µL) of packed RBCs
R204	K13	Safety Monitoring		06-MAR-2018	09:04	15	7	Replicate 1	ND
								Replicate 2	ND
								Replicate 3	ND
								Geometric Mean	1.0000
				07-MAR-2018	09:32	16	8	Replicate 1	ND
								Replicate 2	ND
								Replicate 3	21
								Geometric Mean	2.7589
				08-MAR-2018	08:20	17	9	Replicate 1	ND
								Replicate 2	ND
								Replicate 3	35
								Geometric Mean	3.2711
				09-MAR-2018	09:00	18	10	Replicate 1	ND
								Replicate 2	ND
								Replicate 3	ND
								Geometric Mean	1.0000

Notes: Day relative to inoculum is the number of days relative to day of administration of the inoculum, where the day of administration = 0.
The inoculum was administered on Study Day 0 for K13-infected subjects and Study Day 1 for 3D7-infected subjects.
Day relative to IP is the number of days relative to day of administration of Artesunate, where the day of dose = 1.
qPCR = quantitative Polymerase Chain Reaction; IP = Artesunate; ND = Not Detected; RBCs: red blood cells.

Listing 16.2.6.3
Malaria 18S qPCR

Randomisation Number	Challenge Agent	Visit	Timepoint	Date of Measurement	Time of Measurement	Day Relative to Inoculum	Day Relative to Artesunate	Assay	Result (parasites/500µL) of packed RBCs
R204	K13	Safety Monitoring		12-MAR-2018	09:10	21	13	Replicate 1	87
								Replicate 2	119
								Replicate 3	44
								Geometric Mean	76.9437
				14-MAR-2018	09:59	23	15	Replicate 1	124
								Replicate 2	43
								Replicate 3	37
								Geometric Mean	58.2144
				16-MAR-2018	07:15	25	17	Replicate 1	41
								Replicate 2	41
								Replicate 3	121
								Geometric Mean	58.8100
				19-MAR-2018	08:24	28	20	Replicate 1	883
								Replicate 2	921
								Replicate 3	971
								Geometric Mean	924.3005

Notes: Day relative to inoculum is the number of days relative to day of administration of the inoculum, where the day of administration = 0.
The inoculum was administered on Study Day 0 for K13-infected subjects and Study Day 1 for 3D7-infected subjects.
Day relative to IP is the number of days relative to day of administration of Artesunate, where the day of dose = 1.
qPCR = quantitative Polymerase Chain Reaction; IP = Artesunate; ND = Not Detected; RBCs: red blood cells.

Listing 16.2.6.3
Malaria 18S qPCR

Randomisation Number	Challenge Agent	Visit	Timepoint	Date of Measurement	Time of Measurement	Day Relative to Inoculum	Day Relative to Artesunate	Assay	Result (parasites/500µL) of packed RBCs
R204	K13	End of Study		22-MAR-2018	08:43	31	23	Replicate 1	ND
								Replicate 2	ND
								Replicate 3	ND
								Geometric Mean	1.0000
R205	K13	Inoculum		19-FEB-2018	10:31	0	-9	Replicate 1	ND
								Replicate 2	ND
								Replicate 3	ND
								Geometric Mean	1.0000
		Malaria Monitoring		23-FEB-2018	07:40	4	-5	Replicate 1	36
								Replicate 2	44
								Replicate 3	46
								Geometric Mean	41.7674
				24-FEB-2018	07:17	5	-4	Replicate 1	45
								Replicate 2	71
								Replicate 3	35
								Geometric Mean	48.1777

Notes: Day relative to inoculum is the number of days relative to day of administration of the inoculum, where the day of administration = 0.
The inoculum was administered on Study Day 0 for K13-infected subjects and Study Day 1 for 3D7-infected subjects.
Day relative to IP is the number of days relative to day of administration of Artesunate, where the day of dose = 1.
qPCR = quantitative Polymerase Chain Reaction; IP = Artesunate; ND = Not Detected; RBCs: red blood cells.

Listing 16.2.6.3
Malaria 18S qPCR

Randomisation Number	Challenge Agent	Visit	Timepoint	Date of Measurement	Time of Measurement	Day Relative to Inoculum	Day Relative to Artesunate	Assay	Result (parasites/500µL) of packed RBCs
R205	K13	Malaria Monitoring		24-FEB-2018	19:11	5	-4	Replicate 1	561
								Replicate 2	336
								Replicate 3	616
								Geometric Mean	487.8590
				25-FEB-2018	07:45	6	-3	Replicate 1	917
								Replicate 2	1064
								Replicate 3	640
								Geometric Mean	854.7327
				25-FEB-2018	19:32	6	-3	Replicate 1	1568
								Replicate 2	1075
								Replicate 3	985
								Geometric Mean	1184.1233
				26-FEB-2018	07:40	7	-2	Replicate 1	1770
								Replicate 2	2071
								Replicate 3	1998
								Geometric Mean	1942.0017

Notes: Day relative to inoculum is the number of days relative to day of administration of the inoculum, where the day of administration = 0.
The inoculum was administered on Study Day 0 for K13-infected subjects and Study Day 1 for 3D7-infected subjects.
Day relative to IP is the number of days relative to day of administration of Artesunate, where the day of dose = 1.
qPCR = quantitative Polymerase Chain Reaction; IP = Artesunate; ND = Not Detected; RBCs: red blood cells.

Listing 16.2.6.3
Malaria 18S qPCR

Randomisation Number	Challenge Agent	Visit	Timepoint	Date of Measurement	Time of Measurement	Day Relative to Inoculum	Day Relative to Artesunate	Assay	Result (parasites/500µL) of packed RBCs
R205	K13	Malaria Monitoring		26-FEB-2018	19:21	7	-2	Replicate 1	11100
								Replicate 2	10660
								Replicate 3	10588
								Geometric Mean	10780.3130
				27-FEB-2018	07:15	8	-1	Replicate 1	27461
								Replicate 2	27880
								Replicate 3	27842
								Geometric Mean	27727.0191
				27-FEB-2018	19:20	8	-1	Replicate 1	22121
								Replicate 2	20821
								Replicate 3	22453
								Geometric Mean	21786.8063
		Admission	Pre-dose	28-FEB-2018	12:19	9	1	Replicate 1	64499
								Replicate 2	62013
								Replicate 3	72146
								Geometric Mean	66081.9211

Notes: Day relative to inoculum is the number of days relative to day of administration of the inoculum, where the day of administration = 0.
The inoculum was administered on Study Day 0 for K13-infected subjects and Study Day 1 for 3D7-infected subjects.
Day relative to IP is the number of days relative to day of administration of Artesunate, where the day of dose = 1.
qPCR = quantitative Polymerase Chain Reaction; IP = Artesunate; ND = Not Detected; RBCs: red blood cells.

Listing 16.2.6.3
Malaria 18S qPCR

Randomisation Number	Challenge Agent	Visit	Timepoint	Date of Measurement	Time of Measurement	Day Relative to Inoculum	Day Relative to Artesunate	Assay	Result (parasites/500µL) of packed RBCs
R205	K13	Admission	2 Hrs Post-dose	28-FEB-2018	15:11	9	1	Replicate 1	97960
								Replicate 2	92414
								Replicate 3	98670
								Geometric Mean	96306.8602
			4 Hrs Post-dose	28-FEB-2018	17:10	9	1	Replicate 1	131011
								Replicate 2	125961
								Replicate 3	122815
								Geometric Mean	126550.8572
			6 Hrs Post-dose	28-FEB-2018	19:11	9	1	Replicate 1	105279
								Replicate 2	104994
								Replicate 3	91976
								Geometric Mean	100552.6646
			8 Hrs Post-dose	28-FEB-2018	21:10	9	1	Replicate 1	111497
								Replicate 2	110269
								Replicate 3	109105
								Geometric Mean	110286.0098

Notes: Day relative to inoculum is the number of days relative to day of administration of the inoculum, where the day of administration = 0.
The inoculum was administered on Study Day 0 for K13-infected subjects and Study Day 1 for 3D7-infected subjects.
Day relative to IP is the number of days relative to day of administration of Artesunate, where the day of dose = 1.
qPCR = quantitative Polymerase Chain Reaction; IP = Artesunate; ND = Not Detected; RBCs: red blood cells.

Listing 16.2.6.3
Malaria 18S qPCR

Randomisation Number	Challenge Agent	Visit	Timepoint	Date of Measurement	Time of Measurement	Day Relative to Inoculum	Day Relative to Artesunate	Assay	Result (parasites/500µL) of packed RBCs
R205	K13	Admission 1	12 Hrs Post-dose	01-MAR-2018	01:10	10	2	Replicate 1	82507
								Replicate 2	75857
								Replicate 3	76718
								Geometric Mean	78305.9166
			16 Hrs Post-dose	01-MAR-2018	05:11	10	2	Replicate 1	41258
								Replicate 2	39036
								Replicate 3	43438
								Geometric Mean	41204.7959
			20 Hrs Post-dose	01-MAR-2018	09:09	10	2	Replicate 1	44551
								Replicate 2	39919
								Replicate 3	39063
								Geometric Mean	41108.7465
			24 Hrs Post-dose	01-MAR-2018	13:12	10	2	Replicate 1	29221
								Replicate 2	27424
								Replicate 3	30427
								Geometric Mean	28997.5743

Notes: Day relative to inoculum is the number of days relative to day of administration of the inoculum, where the day of administration = 0.
The inoculum was administered on Study Day 0 for K13-infected subjects and Study Day 1 for 3D7-infected subjects.
Day relative to IP is the number of days relative to day of administration of Artesunate, where the day of dose = 1.
qPCR = quantitative Polymerase Chain Reaction; IP = Artesunate; ND = Not Detected; RBCs: red blood cells.

Listing 16.2.6.3
Malaria 18S qPCR

Randomisation Number	Challenge Agent	Visit	Timepoint	Date of Measurement	Time of Measurement	Day Relative to Inoculum	Day Relative to Artesunate	Assay	Result (parasites/500µL) of packed RBCs
R205	K13	Admission 1	28 Hrs Post-dose	01-MAR-2018	17:13	10	2	Replicate 1	16596
								Replicate 2	16755
								Replicate 3	16134
								Geometric Mean	16492.8852
			32 Hrs Post-dose	01-MAR-2018	21:20	10	2	Replicate 1	7886
								Replicate 2	6814
								Replicate 3	7641
								Geometric Mean	7432.5248
		Admission 2	36 Hrs Post-dose	02-MAR-2018	01:10	11	3	Replicate 1	3983
								Replicate 2	3687
								Replicate 3	4161
								Geometric Mean	3938.7679
			48 Hrs Post-dose	02-MAR-2018	13:11	11	3	Replicate 1	54410
								Replicate 2	46035
								Replicate 3	51913
								Geometric Mean	50661.8448

Notes: Day relative to inoculum is the number of days relative to day of administration of the inoculum, where the day of administration = 0.
The inoculum was administered on Study Day 0 for K13-infected subjects and Study Day 1 for 3D7-infected subjects.
Day relative to IP is the number of days relative to day of administration of Artesunate, where the day of dose = 1.
qPCR = quantitative Polymerase Chain Reaction; IP = Artesunate; ND = Not Detected; RBCs: red blood cells.

Listing 16.2.6.3
Malaria 18S qPCR

Randomisation Number	Challenge Agent	Visit	Timepoint	Date of Measurement	Time of Measurement	Day Relative to Inoculum	Day Relative to Artesunate	Assay	Result (parasites/500µL) of packed RBCs
R205	K13	Admission 2	52 Hrs Post-dose	02-MAR-2018	17:10	11	3	Replicate 1	156996
								Replicate 2	148208
								Replicate 3	152625
								Geometric Mean	152567.4791
			56 Hrs Post-dose	02-MAR-2018	21:10	11	3	Replicate 1	138717
								Replicate 2	148544
								Replicate 3	150843
								Geometric Mean	145938.5369
		Admission 3	60 Hrs Post-dose	03-MAR-2018	01:12	12	4	Replicate 1	78951
								Replicate 2	76011
								Replicate 3	73761
								Geometric Mean	76211.4619
			72 Hrs Post-dose	03-MAR-2018	13:15	12	4	Replicate 1	46052
								Replicate 2	36270
								Replicate 3	39880
								Geometric Mean	40536.8876

Notes: Day relative to inoculum is the number of days relative to day of administration of the inoculum, where the day of administration = 0.
The inoculum was administered on Study Day 0 for K13-infected subjects and Study Day 1 for 3D7-infected subjects.
Day relative to IP is the number of days relative to day of administration of Artesunate, where the day of dose = 1.
qPCR = quantitative Polymerase Chain Reaction; IP = Artesunate; ND = Not Detected; RBCs: red blood cells.

Listing 16.2.6.3
Malaria 18S qPCR

Randomisation Number	Challenge Agent	Visit	Timepoint	Date of Measurement	Time of Measurement	Day Relative to Inoculum	Day Relative to Artesunate	Assay	Result (parasites/500µL) of packed RBCs
R205	K13	Admission 3		03-MAR-2018	20:27	12	4	Replicate 1	8954
								Replicate 2	8849
								Replicate 3	10812
								Geometric Mean	9497.4228
		Safety Monitoring		04-MAR-2018	08:32	13	5	Replicate 1	1709
								Replicate 2	1114
								Replicate 3	1555
								Geometric Mean	1435.8835
				04-MAR-2018	20:54	13	5	Replicate 1	526
								Replicate 2	638
								Replicate 3	342
								Geometric Mean	485.9715
				05-MAR-2018	09:13	14	6	Replicate 1	135
								Replicate 2	138
								Replicate 3	343
								Geometric Mean	185.5677

Notes: Day relative to inoculum is the number of days relative to day of administration of the inoculum, where the day of administration = 0.
The inoculum was administered on Study Day 0 for K13-infected subjects and Study Day 1 for 3D7-infected subjects.
Day relative to IP is the number of days relative to day of administration of Artesunate, where the day of dose = 1.
qPCR = quantitative Polymerase Chain Reaction; IP = Artesunate; ND = Not Detected; RBCs: red blood cells.

Listing 16.2.6.3
Malaria 18S qPCR

Randomisation Number	Challenge Agent	Visit	Timepoint	Date of Measurement	Time of Measurement	Day Relative to Inoculum	Day Relative to Artesunate	Assay	Result (parasites/500µL) of packed RBCs
R205	K13	Safety Monitoring		06-MAR-2018	07:20	15	7	Replicate 1	26
								Replicate 2	39
								Replicate 3	ND
								Geometric Mean	10.0465
				07-MAR-2018	07:54	16	8	Replicate 1	192
								Replicate 2	ND
								Replicate 3	142
								Geometric Mean	30.0975
				08-MAR-2018	07:33	17	9	Replicate 1	133
								Replicate 2	72
								Replicate 3	32
								Geometric Mean	67.4183
				09-MAR-2018	07:52	18	10	Replicate 1	122
								Replicate 2	243
								Replicate 3	359
								Geometric Mean	219.9650

Notes: Day relative to inoculum is the number of days relative to day of administration of the inoculum, where the day of administration = 0.
The inoculum was administered on Study Day 0 for K13-infected subjects and Study Day 1 for 3D7-infected subjects.
Day relative to IP is the number of days relative to day of administration of Artesunate, where the day of dose = 1.
qPCR = quantitative Polymerase Chain Reaction; IP = Artesunate; ND = Not Detected; RBCs: red blood cells.

Listing 16.2.6.3
Malaria 18S qPCR

Randomisation Number	Challenge Agent	Visit	Timepoint	Date of Measurement	Time of Measurement	Day Relative to Inoculum	Day Relative to Artesunate	Assay	Result (parasites/500µL) of packed RBCs
R205	K13	Safety Monitoring		12-MAR-2018	07:48	21	13	Replicate 1	610
								Replicate 2	547
								Replicate 3	715
								Geometric Mean	620.2133
				14-MAR-2018	08:46	23	15	Replicate 1	928
								Replicate 2	646
								Replicate 3	565
								Geometric Mean	697.0699
				16-MAR-2018	07:36	25	17	Replicate 1	365
								Replicate 2	242
								Replicate 3	531
								Geometric Mean	360.6348
				19-MAR-2018	07:29	28	20	Replicate 1	43
								Replicate 2	ND
								Replicate 3	ND
								Geometric Mean	3.5034

Notes: Day relative to inoculum is the number of days relative to day of administration of the inoculum, where the day of administration = 0.
The inoculum was administered on Study Day 0 for K13-infected subjects and Study Day 1 for 3D7-infected subjects.
Day relative to IP is the number of days relative to day of administration of Artesunate, where the day of dose = 1.
qPCR = quantitative Polymerase Chain Reaction; IP = Artesunate; ND = Not Detected; RBCs: red blood cells.

Listing 16.2.6.3
Malaria 18S qPCR

Randomisation Number	Challenge Agent	Visit	Timepoint	Date of Measurement	Time of Measurement	Day Relative to Inoculum	Day Relative to Artesunate	Assay	Result (parasites/500µL) of packed RBCs
R205	K13	End of Study		22-MAR-2018	07:30	31	23	Replicate 1	ND
								Replicate 2	ND
								Replicate 3	ND
								Geometric Mean	1.0000
R206	K13	Inoculum		19-FEB-2018	10:18	0	-9	Replicate 1	ND
								Replicate 2	ND
								Replicate 3	ND
								Geometric Mean	1.0000
		Malaria Monitoring		23-FEB-2018	08:20	4	-5	Replicate 1	84
								Replicate 2	130
								Replicate 3	87
								Geometric Mean	98.3061
				24-FEB-2018	08:15	5	-4	Replicate 1	122
								Replicate 2	42
								Replicate 3	61
								Geometric Mean	67.8651

Notes: Day relative to inoculum is the number of days relative to day of administration of the inoculum, where the day of administration = 0.
The inoculum was administered on Study Day 0 for K13-infected subjects and Study Day 1 for 3D7-infected subjects.
Day relative to IP is the number of days relative to day of administration of Artesunate, where the day of dose = 1.
qPCR = quantitative Polymerase Chain Reaction; IP = Artesunate; ND = Not Detected; RBCs: red blood cells.

Listing 16.2.6.3
Malaria 18S qPCR

Randomisation Number	Challenge Agent	Visit	Timepoint	Date of Measurement	Time of Measurement	Day Relative to Inoculum	Day Relative to Artesunate	Assay	Result (parasites/500µL) of packed RBCs
R206	K13	Malaria Monitoring		24-FEB-2018	19:47	5	-4	Replicate 1	754
								Replicate 2	526
								Replicate 3	476
								Geometric Mean	573.6601
				25-FEB-2018	08:16	6	-3	Replicate 1	1510
								Replicate 2	832
								Replicate 3	981
								Geometric Mean	1072.1522
				25-FEB-2018	21:06	6	-3	Replicate 1	1107
								Replicate 2	1587
								Replicate 3	1601
								Geometric Mean	1411.5793
				26-FEB-2018	09:12	7	-2	Replicate 1	6320
								Replicate 2	6474
								Replicate 3	6313
								Geometric Mean	6368.5689

Notes: Day relative to inoculum is the number of days relative to day of administration of the inoculum, where the day of administration = 0.
The inoculum was administered on Study Day 0 for K13-infected subjects and Study Day 1 for 3D7-infected subjects.
Day relative to IP is the number of days relative to day of administration of Artesunate, where the day of dose = 1.
qPCR = quantitative Polymerase Chain Reaction; IP = Artesunate; ND = Not Detected; RBCs: red blood cells.

Listing 16.2.6.3
Malaria 18S qPCR

Randomisation Number	Challenge Agent	Visit	Timepoint	Date of Measurement	Time of Measurement	Day Relative to Inoculum	Day Relative to Artesunate	Assay	Result (parasites/500µL) of packed RBCs
R206	K13	Malaria Monitoring		26-FEB-2018	19:55	7	-2	Replicate 1	22766
								Replicate 2	21663
								Replicate 3	21313
								Geometric Mean	21905.3387
				27-FEB-2018	08:11	8	-1	Replicate 1	24335
								Replicate 2	26727
								Replicate 3	24084
								Geometric Mean	25020.9269
				27-FEB-2018	19:02	8	-1	Replicate 1	25770
								Replicate 2	30015
								Replicate 3	32477
								Geometric Mean	29287.0901
		Admission	Pre-dose	28-FEB-2018	12:48	9	1	Replicate 1	184724
								Replicate 2	186907
								Replicate 3	183063
								Geometric Mean	184891.3054

Notes: Day relative to inoculum is the number of days relative to day of administration of the inoculum, where the day of administration = 0.
The inoculum was administered on Study Day 0 for K13-infected subjects and Study Day 1 for 3D7-infected subjects.
Day relative to IP is the number of days relative to day of administration of Artesunate, where the day of dose = 1.
qPCR = quantitative Polymerase Chain Reaction; IP = Artesunate; ND = Not Detected; RBCs: red blood cells.

Listing 16.2.6.3
Malaria 18S qPCR

Randomisation Number	Challenge Agent	Visit	Timepoint	Date of Measurement	Time of Measurement	Day Relative to Inoculum	Day Relative to Artesunate	Assay	Result (parasites/500µL) of packed RBCs
R206	K13	Admission	2 Hrs Post-dose	28-FEB-2018	15:26	9	1	Replicate 1	258642
								Replicate 2	254240
								Replicate 3	230446
								Geometric Mean	247459.4451
			4 Hrs Post-dose	28-FEB-2018	17:27	9	1	Replicate 1	334081
								Replicate 2	309104
								Replicate 3	324853
								Geometric Mean	322513.3982
			6 Hrs Post-dose	28-FEB-2018	19:27	9	1	Replicate 1	416487
								Replicate 2	402339
								Replicate 3	380161
								Geometric Mean	399380.6761
			8 Hrs Post-dose	28-FEB-2018	21:28	9	1	Replicate 1	244828
								Replicate 2	268829
								Replicate 3	270229
								Geometric Mean	261029.5763

Notes: Day relative to inoculum is the number of days relative to day of administration of the inoculum, where the day of administration = 0.
The inoculum was administered on Study Day 0 for K13-infected subjects and Study Day 1 for 3D7-infected subjects.
Day relative to IP is the number of days relative to day of administration of Artesunate, where the day of dose = 1.
qPCR = quantitative Polymerase Chain Reaction; IP = Artesunate; ND = Not Detected; RBCs: red blood cells.

Listing 16.2.6.3
Malaria 18S qPCR

Randomisation Number	Challenge Agent	Visit	Timepoint	Date of Measurement	Time of Measurement	Day Relative to Inoculum	Day Relative to Artesunate	Assay	Result (parasites/500µL) of packed RBCs
R206	K13	Admission 1	12 Hrs Post-dose	01-MAR-2018	01:47	10	2	Replicate 1	154115
								Replicate 2	167058
								Replicate 3	161310
								Geometric Mean	160740.1959
			16 Hrs Post-dose	01-MAR-2018	05:41	10	2	Replicate 1	114949
								Replicate 2	112179
								Replicate 3	100243
								Geometric Mean	108932.3666
			20 Hrs Post-dose	01-MAR-2018	09:26	10	2	Replicate 1	80083
								Replicate 2	86907
								Replicate 3	86632
								Geometric Mean	84480.7598
			24 Hrs Post-dose	01-MAR-2018	13:30	10	2	Replicate 1	60903
								Replicate 2	57741
								Replicate 3	53087
								Geometric Mean	57152.7401

Notes: Day relative to inoculum is the number of days relative to day of administration of the inoculum, where the day of administration = 0.
The inoculum was administered on Study Day 0 for K13-infected subjects and Study Day 1 for 3D7-infected subjects.
Day relative to IP is the number of days relative to day of administration of Artesunate, where the day of dose = 1.
qPCR = quantitative Polymerase Chain Reaction; IP = Artesunate; ND = Not Detected; RBCs: red blood cells.

Listing 16.2.6.3
Malaria 18S qPCR

Randomisation Number	Challenge Agent	Visit	Timepoint	Date of Measurement	Time of Measurement	Day Relative to Inoculum	Day Relative to Artesunate	Assay	Result (parasites/500µL) of packed RBCs
R206	K13	Admission 1	28 Hrs Post-dose	01-MAR-2018	17:26	10	2	Replicate 1	32654
								Replicate 2	35007
								Replicate 3	31752
								Geometric Mean	33109.6144
			32 Hrs Post-dose	01-MAR-2018	21:30	10	2	Replicate 1	9813
								Replicate 2	12157
								Replicate 3	10751
								Geometric Mean	10864.8990
		Admission 2	36 Hrs Post-dose	02-MAR-2018	01:28	11	3	Replicate 1	8344
								Replicate 2	7634
								Replicate 3	8640
								Geometric Mean	8194.9580
			48 Hrs Post-dose	02-MAR-2018	13:29	11	3	Replicate 1	92832
								Replicate 2	73015
								Replicate 3	70615
								Geometric Mean	78223.2015

Notes: Day relative to inoculum is the number of days relative to day of administration of the inoculum, where the day of administration = 0.
The inoculum was administered on Study Day 0 for K13-infected subjects and Study Day 1 for 3D7-infected subjects.
Day relative to IP is the number of days relative to day of administration of Artesunate, where the day of dose = 1.
qPCR = quantitative Polymerase Chain Reaction; IP = Artesunate; ND = Not Detected; RBCs: red blood cells.

Listing 16.2.6.3
Malaria 18S qPCR

Randomisation Number	Challenge Agent	Visit	Timepoint	Date of Measurement	Time of Measurement	Day Relative to Inoculum	Day Relative to Artesunate	Assay	Result (parasites/500µL) of packed RBCs
R206	K13	Admission 2	52 Hrs Post-dose	02-MAR-2018	17:27	11	3	Replicate 1	295850
								Replicate 2	280872
								Replicate 3	291999
								Geometric Mean	289503.4842
			56 Hrs Post-dose	02-MAR-2018	21:28	11	3	Replicate 1	252466
								Replicate 2	258935
								Replicate 3	244386
								Geometric Mean	251858.5275
		Admission 3	60 Hrs Post-dose	03-MAR-2018	01:31	12	4	Replicate 1	226523
								Replicate 2	200284
								Replicate 3	189972
								Geometric Mean	205029.3581
			72 Hrs Post-dose	03-MAR-2018	13:39	12	4	Replicate 1	90384
								Replicate 2	95419
								Replicate 3	101994
								Geometric Mean	95815.0390

Notes: Day relative to inoculum is the number of days relative to day of administration of the inoculum, where the day of administration = 0.
The inoculum was administered on Study Day 0 for K13-infected subjects and Study Day 1 for 3D7-infected subjects.
Day relative to IP is the number of days relative to day of administration of Artesunate, where the day of dose = 1.
qPCR = quantitative Polymerase Chain Reaction; IP = Artesunate; ND = Not Detected; RBCs: red blood cells.

Listing 16.2.6.3
Malaria 18S qPCR

Randomisation Number	Challenge Agent	Visit	Timepoint	Date of Measurement	Time of Measurement	Day Relative to Inoculum	Day Relative to Artesunate	Assay	Result (parasites/500µL) of packed RBCs
R206	K13	Admission 3		03-MAR-2018	19:55	12	4	Replicate 1	20996
								Replicate 2	26455
								Replicate 3	23491
								Geometric Mean	23542.2849
		Safety Monitoring		04-MAR-2018	08:21	13	5	Replicate 1	3183
								Replicate 2	4049
								Replicate 3	3909
								Geometric Mean	3693.3179
				04-MAR-2018	19:25	13	5	Replicate 1	690
								Replicate 2	708
								Replicate 3	714
								Geometric Mean	703.9258
				05-MAR-2018	09:30	14	6	Replicate 1	359
								Replicate 2	73
								Replicate 3	245
								Geometric Mean	185.8637

Notes: Day relative to inoculum is the number of days relative to day of administration of the inoculum, where the day of administration = 0.
The inoculum was administered on Study Day 0 for K13-infected subjects and Study Day 1 for 3D7-infected subjects.
Day relative to IP is the number of days relative to day of administration of Artesunate, where the day of dose = 1.
qPCR = quantitative Polymerase Chain Reaction; IP = Artesunate; ND = Not Detected; RBCs: red blood cells.

Listing 16.2.6.3
Malaria 18S qPCR

Randomisation Number	Challenge Agent	Visit	Timepoint	Date of Measurement	Time of Measurement	Day Relative to Inoculum	Day Relative to Artesunate	Assay	Result (parasites/500µL) of packed RBCs
R206	K13	Safety Monitoring		06-MAR-2018	08:31	15	7	Replicate 1	39
								Replicate 2	39
								Replicate 3	160
								Geometric Mean	62.4333
				07-MAR-2018	09:34	16	8	Replicate 1	301
								Replicate 2	122
								Replicate 3	370
								Geometric Mean	238.6214
				08-MAR-2018	08:37	17	9	Replicate 1	417
								Replicate 2	500
								Replicate 3	117
								Geometric Mean	290.0218
				09-MAR-2018	08:58	18	10	Replicate 1	320
								Replicate 2	227
								Replicate 3	482
								Geometric Mean	327.1455

Notes: Day relative to inoculum is the number of days relative to day of administration of the inoculum, where the day of administration = 0.
The inoculum was administered on Study Day 0 for K13-infected subjects and Study Day 1 for 3D7-infected subjects.
Day relative to IP is the number of days relative to day of administration of Artesunate, where the day of dose = 1.
qPCR = quantitative Polymerase Chain Reaction; IP = Artesunate; ND = Not Detected; RBCs: red blood cells.

Listing 16.2.6.3
Malaria 18S qPCR

Randomisation Number	Challenge Agent	Visit	Timepoint	Date of Measurement	Time of Measurement	Day Relative to Inoculum	Day Relative to Artesunate	Assay	Result (parasites/500µL) of packed RBCs
R206	K13	Safety Monitoring		12-MAR-2018	08:44	21	13	Replicate 1	1726
								Replicate 2	2121
								Replicate 3	2147
								Geometric Mean	1988.2508
				14-MAR-2018	10:05	23	15	Replicate 1	719
								Replicate 2	666
								Replicate 3	857
								Geometric Mean	743.1240
				16-MAR-2018	08:01	25	17	Replicate 1	1524
								Replicate 2	1652
								Replicate 3	1450
								Geometric Mean	1539.7646
				19-MAR-2018	09:44	28	20	Replicate 1	ND
								Replicate 2	ND
								Replicate 3	38
								Geometric Mean	3.3620

Notes: Day relative to inoculum is the number of days relative to day of administration of the inoculum, where the day of administration = 0.
The inoculum was administered on Study Day 0 for K13-infected subjects and Study Day 1 for 3D7-infected subjects.
Day relative to IP is the number of days relative to day of administration of Artesunate, where the day of dose = 1.
qPCR = quantitative Polymerase Chain Reaction; IP = Artesunate; ND = Not Detected; RBCs: red blood cells.

Listing 16.2.6.3
Malaria 18S qPCR

Randomisation Number	Challenge Agent	Visit	Timepoint	Date of Measurement	Time of Measurement	Day Relative to Inoculum	Day Relative to Artesunate	Assay	Result (parasites/500µL) of packed RBCs
R206	K13	End of Study		22-MAR-2018	09:19	31	23	Replicate 1	ND
								Replicate 2	35
								Replicate 3	ND
								Geometric Mean	3.2711
		Unscheduled		28-MAR-2018	10:02	37	29	Replicate 1	ND
								Replicate 2	ND
								Replicate 3	ND
								Geometric Mean	1.0000
R207	K13	Inoculum		19-FEB-2018	10:37	0	-9	Replicate 1	ND
								Replicate 2	ND
								Replicate 3	ND
								Geometric Mean	1.0000
		Malaria Monitoring		23-FEB-2018	09:09	4	-5	Replicate 1	44
								Replicate 2	44
								Replicate 3	87
								Geometric Mean	55.2257

Notes: Day relative to inoculum is the number of days relative to day of administration of the inoculum, where the day of administration = 0.
The inoculum was administered on Study Day 0 for K13-infected subjects and Study Day 1 for 3D7-infected subjects.
Day relative to IP is the number of days relative to day of administration of Artesunate, where the day of dose = 1.
qPCR = quantitative Polymerase Chain Reaction; IP = Artesunate; ND = Not Detected; RBCs: red blood cells.

Listing 16.2.6.3
Malaria 18S qPCR

Randomisation Number	Challenge Agent	Visit	Timepoint	Date of Measurement	Time of Measurement	Day Relative to Inoculum	Day Relative to Artesunate	Assay	Result (parasites/500µL) of packed RBCs
R207	K13	Malaria Monitoring		24-FEB-2018	09:06	5	-4	Replicate 1	43
								Replicate 2	ND
								Replicate 3	150
								Geometric Mean	18.6146
				24-FEB-2018	19:14	5	-4	Replicate 1	552
								Replicate 2	419
								Replicate 3	657
								Geometric Mean	533.6291
				25-FEB-2018	08:56	6	-3	Replicate 1	279
								Replicate 2	378
								Replicate 3	408
								Geometric Mean	350.4172
				25-FEB-2018	20:29	6	-3	Replicate 1	427
								Replicate 2	238
								Replicate 3	375
								Geometric Mean	336.5209

Notes: Day relative to inoculum is the number of days relative to day of administration of the inoculum, where the day of administration = 0.
The inoculum was administered on Study Day 0 for K13-infected subjects and Study Day 1 for 3D7-infected subjects.
Day relative to IP is the number of days relative to day of administration of Artesunate, where the day of dose = 1.
qPCR = quantitative Polymerase Chain Reaction; IP = Artesunate; ND = Not Detected; RBCs: red blood cells.

Listing 16.2.6.3
Malaria 18S qPCR

Randomisation Number	Challenge Agent	Visit	Timepoint	Date of Measurement	Time of Measurement	Day Relative to Inoculum	Day Relative to Artesunate	Assay	Result (parasites/500µL) of packed RBCs
R207	K13	Malaria Monitoring		26-FEB-2018	08:53	7	-2	Replicate 1	914
								Replicate 2	816
								Replicate 3	673
								Geometric Mean	794.7255
				26-FEB-2018	20:00	7	-2	Replicate 1	11524
								Replicate 2	9115
								Replicate 3	10567
								Geometric Mean	10353.8979
				27-FEB-2018	09:15	8	-1	Replicate 1	10845
								Replicate 2	9612
								Replicate 3	10111
								Geometric Mean	10176.8290
				27-FEB-2018	19:34	8	-1	Replicate 1	6834
								Replicate 2	6027
								Replicate 3	8608
								Geometric Mean	7077.7107

Notes: Day relative to inoculum is the number of days relative to day of administration of the inoculum, where the day of administration = 0.
The inoculum was administered on Study Day 0 for K13-infected subjects and Study Day 1 for 3D7-infected subjects.
Day relative to IP is the number of days relative to day of administration of Artesunate, where the day of dose = 1.
qPCR = quantitative Polymerase Chain Reaction; IP = Artesunate; ND = Not Detected; RBCs: red blood cells.

Listing 16.2.6.3
Malaria 18S qPCR

Randomisation Number	Challenge Agent	Visit	Timepoint	Date of Measurement	Time of Measurement	Day Relative to Inoculum	Day Relative to Artesunate	Assay	Result (parasites/500µL) of packed RBCs
R207	K13	Admission	Pre-dose	28-FEB-2018	12:00	9	1	Replicate 1	18772
								Replicate 2	17104
								Replicate 3	17011
								Geometric Mean	17610.8111
			2 Hrs Post-dose	28-FEB-2018	14:58	9	1	Replicate 1	70154
								Replicate 2	65958
								Replicate 3	67419
								Geometric Mean	67821.5027
			4 Hrs Post-dose	28-FEB-2018	16:59	9	1	Replicate 1	81635
								Replicate 2	80682
								Replicate 3	45600
								Geometric Mean	66968.8177
			6 Hrs Post-dose	28-FEB-2018	18:58	9	1	Replicate 1	70487
								Replicate 2	71902
								Replicate 3	65424
								Geometric Mean	69214.2786

Notes: Day relative to inoculum is the number of days relative to day of administration of the inoculum, where the day of administration = 0.
The inoculum was administered on Study Day 0 for K13-infected subjects and Study Day 1 for 3D7-infected subjects.
Day relative to IP is the number of days relative to day of administration of Artesunate, where the day of dose = 1.
qPCR = quantitative Polymerase Chain Reaction; IP = Artesunate; ND = Not Detected; RBCs: red blood cells.

Listing 16.2.6.3
Malaria 18S qPCR

Randomisation Number	Challenge Agent	Visit	Timepoint	Date of Measurement	Time of Measurement	Day Relative to Inoculum	Day Relative to Artesunate	Assay	Result (parasites/500µL) of packed RBCs
R207	K13	Admission	8 Hrs Post-dose	28-FEB-2018	20:58	9	1	Replicate 1	68866
								Replicate 2	63002
								Replicate 3	73230
								Geometric Mean	68236.3953
		Admission 1	12 Hrs Post-dose	01-MAR-2018	00:58	10	2	Replicate 1	31405
								Replicate 2	32723
								Replicate 3	37405
								Geometric Mean	33748.9700
			16 Hrs Post-dose	01-MAR-2018	04:58	10	2	Replicate 1	23466
								Replicate 2	19831
								Replicate 3	20575
								Geometric Mean	21234.4577
			20 Hrs Post-dose	01-MAR-2018	09:00	10	2	Replicate 1	16334
								Replicate 2	14949
								Replicate 3	15326
								Geometric Mean	15525.4610

Notes: Day relative to inoculum is the number of days relative to day of administration of the inoculum, where the day of administration = 0.
The inoculum was administered on Study Day 0 for K13-infected subjects and Study Day 1 for 3D7-infected subjects.
Day relative to IP is the number of days relative to day of administration of Artesunate, where the day of dose = 1.
qPCR = quantitative Polymerase Chain Reaction; IP = Artesunate; ND = Not Detected; RBCs: red blood cells.

Listing 16.2.6.3
Malaria 18S qPCR

Randomisation Number	Challenge Agent	Visit	Timepoint	Date of Measurement	Time of Measurement	Day Relative to Inoculum	Day Relative to Artesunate	Assay	Result (parasites/500µL) of packed RBCs
R207	K13	Admission 1	24 Hrs Post-dose	01-MAR-2018	13:04	10	2	Replicate 1	13407
								Replicate 2	11211
								Replicate 3	12495
								Geometric Mean	12337.7910
			28 Hrs Post-dose	01-MAR-2018	16:58	10	2	Replicate 1	5834
								Replicate 2	5572
								Replicate 3	5513
								Geometric Mean	5637.9581
			32 Hrs Post-dose	01-MAR-2018	20:58	10	2	Replicate 1	2957
								Replicate 2	3039
								Replicate 3	3498
								Geometric Mean	3155.9778
		Admission 2	36 Hrs Post-dose	02-MAR-2018	00:58	11	3	Replicate 1	1043
								Replicate 2	1256
								Replicate 3	1086
								Geometric Mean	1124.6946

Notes: Day relative to inoculum is the number of days relative to day of administration of the inoculum, where the day of administration = 0.
The inoculum was administered on Study Day 0 for K13-infected subjects and Study Day 1 for 3D7-infected subjects.
Day relative to IP is the number of days relative to day of administration of Artesunate, where the day of dose = 1.
qPCR = quantitative Polymerase Chain Reaction; IP = Artesunate; ND = Not Detected; RBCs: red blood cells.

Listing 16.2.6.3
Malaria 18S qPCR

Randomisation Number	Challenge Agent	Visit	Timepoint	Date of Measurement	Time of Measurement	Day Relative to Inoculum	Day Relative to Artesunate	Assay	Result (parasites/500µL) of packed RBCs
R207	K13	Admission 2	48 Hrs Post-dose	02-MAR-2018	12:59	11	3	Replicate 1	8366
								Replicate 2	8625
								Replicate 3	7674
								Geometric Mean	8211.6971
			52 Hrs Post-dose	02-MAR-2018	16:53	11	3	Replicate 1	26083
								Replicate 2	27987
								Replicate 3	10810
								Geometric Mean	19908.8662
			56 Hrs Post-dose	02-MAR-2018	21:01	11	3	Replicate 1	38808
								Replicate 2	38372
								Replicate 3	42728
								Geometric Mean	39922.3491
		Admission 3	60 Hrs Post-dose	03-MAR-2018	00:58	12	4	Replicate 1	33132
								Replicate 2	36976
								Replicate 3	37583
								Geometric Mean	35841.5136

Notes: Day relative to inoculum is the number of days relative to day of administration of the inoculum, where the day of administration = 0.
The inoculum was administered on Study Day 0 for K13-infected subjects and Study Day 1 for 3D7-infected subjects.
Day relative to IP is the number of days relative to day of administration of Artesunate, where the day of dose = 1.
qPCR = quantitative Polymerase Chain Reaction; IP = Artesunate; ND = Not Detected; RBCs: red blood cells.

Listing 16.2.6.3
Malaria 18S qPCR

Randomisation Number	Challenge Agent	Visit	Timepoint	Date of Measurement	Time of Measurement	Day Relative to Inoculum	Day Relative to Artesunate	Assay	Result (parasites/500µL) of packed RBCs
R207	K13	Admission 3	72 Hrs Post-dose	03-MAR-2018	13:00	12	4	Replicate 1	15356
								Replicate 2	14700
								Replicate 3	16555
								Geometric Mean	15518.2070
				03-MAR-2018	20:33	12	4	Replicate 1	2239
								Replicate 2	1872
								Replicate 3	2384
								Geometric Mean	2153.8828
		Safety Monitoring		04-MAR-2018	10:14	13	5	Replicate 1	461
								Replicate 2	298
								Replicate 3	153
								Geometric Mean	275.9749
				04-MAR-2018	19:55	13	5	Replicate 1	104
								Replicate 2	146
								Replicate 3	112
								Geometric Mean	119.3625

Notes: Day relative to inoculum is the number of days relative to day of administration of the inoculum, where the day of administration = 0.
The inoculum was administered on Study Day 0 for K13-infected subjects and Study Day 1 for 3D7-infected subjects.
Day relative to IP is the number of days relative to day of administration of Artesunate, where the day of dose = 1.
qPCR = quantitative Polymerase Chain Reaction; IP = Artesunate; ND = Not Detected; RBCs: red blood cells.

Listing 16.2.6.3
Malaria 18S qPCR

Randomisation Number	Challenge Agent	Visit	Timepoint	Date of Measurement	Time of Measurement	Day Relative to Inoculum	Day Relative to Artesunate	Assay	Result (parasites/500µL) of packed RBCs
R207	K13	Safety Monitoring		05-MAR-2018	10:01	14	6	Replicate 1	79
								Replicate 2	93
								Replicate 3	118
								Geometric Mean	95.3522
				06-MAR-2018	09:52	15	7	Replicate 1	ND
								Replicate 2	ND
								Replicate 3	ND
								Geometric Mean	1.0000
				07-MAR-2018	09:11	16	8	Replicate 1	ND
								Replicate 2	ND
								Replicate 3	ND
								Geometric Mean	1.0000
				08-MAR-2018	09:35	17	9	Replicate 1	ND
								Replicate 2	ND
								Replicate 3	ND
								Geometric Mean	1.0000

Notes: Day relative to inoculum is the number of days relative to day of administration of the inoculum, where the day of administration = 0.
The inoculum was administered on Study Day 0 for K13-infected subjects and Study Day 1 for 3D7-infected subjects.
Day relative to IP is the number of days relative to day of administration of Artesunate, where the day of dose = 1.
qPCR = quantitative Polymerase Chain Reaction; IP = Artesunate; ND = Not Detected; RBCs: red blood cells.

Listing 16.2.6.3
Malaria 18S qPCR

Randomisation Number	Challenge Agent	Visit	Timepoint	Date of Measurement	Time of Measurement	Day Relative to Inoculum	Day Relative to Artesunate	Assay	Result (parasites/500µL) of packed RBCs
R207	K13	Safety Monitoring		09-MAR-2018	09:33	18	10	Replicate 1	51
								Replicate 2	ND
								Replicate 3	ND
								Geometric Mean	3.7084
				12-MAR-2018	09:34	21	13	Replicate 1	66
								Replicate 2	73
								Replicate 3	41
								Geometric Mean	58.2394
				14-MAR-2018	09:10	23	15	Replicate 1	ND
								Replicate 2	41
								Replicate 3	ND
								Geometric Mean	3.4482
				16-MAR-2018	09:26	25	17	Replicate 1	ND
								Replicate 2	ND
								Replicate 3	ND
								Geometric Mean	1.0000

Notes: Day relative to inoculum is the number of days relative to day of administration of the inoculum, where the day of administration = 0.
The inoculum was administered on Study Day 0 for K13-infected subjects and Study Day 1 for 3D7-infected subjects.
Day relative to IP is the number of days relative to day of administration of Artesunate, where the day of dose = 1.
qPCR = quantitative Polymerase Chain Reaction; IP = Artesunate; ND = Not Detected; RBCs: red blood cells.

Listing 16.2.6.3
Malaria 18S qPCR

Randomisation Number	Challenge Agent	Visit	Timepoint	Date of Measurement	Time of Measurement	Day Relative to Inoculum	Day Relative to Artesunate	Assay	Result (parasites/500µL) of packed RBCs
R207	K13	Safety Monitoring		19-MAR-2018	09:12	28	20	Replicate 1	ND
								Replicate 2	ND
								Replicate 3	ND
								Geometric Mean	1.0000
		End of Study		22-MAR-2018	09:32	31	23	Replicate 1	ND
								Replicate 2	ND
								Replicate 3	ND
								Geometric Mean	1.0000
R208	3D7	Inoculum		20-FEB-2018	09:55	0	-8	Replicate 1	ND
								Replicate 2	ND
								Replicate 3	ND
								Geometric Mean	1.0000
		Malaria Monitoring		24-FEB-2018	07:19	4	-4	Replicate 1	40
								Replicate 2	ND
								Replicate 3	39
								Geometric Mean	11.5978

Notes: Day relative to inoculum is the number of days relative to day of administration of the inoculum, where the day of administration = 0.
The inoculum was administered on Study Day 0 for K13-infected subjects and Study Day 1 for 3D7-infected subjects.
Day relative to IP is the number of days relative to day of administration of Artesunate, where the day of dose = 1.
qPCR = quantitative Polymerase Chain Reaction; IP = Artesunate; ND = Not Detected; RBCs: red blood cells.

Listing 16.2.6.3
Malaria 18S qPCR

Randomisation Number	Challenge Agent	Visit	Timepoint	Date of Measurement	Time of Measurement	Day Relative to Inoculum	Day Relative to Artesunate	Assay	Result (parasites/500µL) of packed RBCs
R208	3D7	Malaria Monitoring		25-FEB-2018	07:21	5	-3	Replicate 1	272
								Replicate 2	331
								Replicate 3	461
								Geometric Mean	346.2310
				25-FEB-2018	19:08	5	-3	Replicate 1	1426
								Replicate 2	1759
								Replicate 3	1129
								Geometric Mean	1414.7937
				26-FEB-2018	07:03	6	-2	Replicate 1	143
								Replicate 2	170
								Replicate 3	182
								Geometric Mean	164.1668
				26-FEB-2018	19:05	6	-2	Replicate 1	1529
								Replicate 2	1792
								Replicate 3	2522
								Geometric Mean	1904.7158

Notes: Day relative to inoculum is the number of days relative to day of administration of the inoculum, where the day of administration = 0.
The inoculum was administered on Study Day 0 for K13-infected subjects and Study Day 1 for 3D7-infected subjects.
Day relative to IP is the number of days relative to day of administration of Artesunate, where the day of dose = 1.
qPCR = quantitative Polymerase Chain Reaction; IP = Artesunate; ND = Not Detected; RBCs: red blood cells.

Listing 16.2.6.3
Malaria 18S qPCR

Randomisation Number	Challenge Agent	Visit	Timepoint	Date of Measurement	Time of Measurement	Day Relative to Inoculum	Day Relative to Artesunate	Assay	Result (parasites/500µL) of packed RBCs
R208	3D7	Malaria Monitoring		27-FEB-2018	07:18	7	-1	Replicate 1	11158
								Replicate 2	8770
								Replicate 3	10070
								Geometric Mean	9951.1164
				27-FEB-2018	19:01	7	-1	Replicate 1	5038
								Replicate 2	4563
								Replicate 3	4209
								Geometric Mean	4590.8791
		Admission	Pre-dose	28-FEB-2018	11:13	8	1	Replicate 1	18155
								Replicate 2	17598
								Replicate 3	19067
								Geometric Mean	18263.3585
			2 Hrs Post-dose	28-FEB-2018	13:36	8	1	Replicate 1	25877
								Replicate 2	25237
								Replicate 3	26896
								Geometric Mean	25994.3991

Notes: Day relative to inoculum is the number of days relative to day of administration of the inoculum, where the day of administration = 0.
The inoculum was administered on Study Day 0 for K13-infected subjects and Study Day 1 for 3D7-infected subjects.
Day relative to IP is the number of days relative to day of administration of Artesunate, where the day of dose = 1.
qPCR = quantitative Polymerase Chain Reaction; IP = Artesunate; ND = Not Detected; RBCs: red blood cells.

Listing 16.2.6.3
Malaria 18S qPCR

Randomisation Number	Challenge Agent	Visit	Timepoint	Date of Measurement	Time of Measurement	Day Relative to Inoculum	Day Relative to Artesunate	Assay	Result (parasites/500µL) of packed RBCs
R208	3D7	Admission	4 Hrs Post-dose	28-FEB-2018	15:35	8	1	Replicate 1	16916
								Replicate 2	16779
								Replicate 3	16916
								Geometric Mean	16870.2095
			6 Hrs Post-dose	28-FEB-2018	17:35	8	1	Replicate 1	27530
								Replicate 2	23759
								Replicate 3	25391
								Geometric Mean	25513.5794
			8 Hrs Post-dose	28-FEB-2018	19:35	8	1	Replicate 1	11143
								Replicate 2	11900
								Replicate 3	12005
								Geometric Mean	11676.2599
			12 Hrs Post-dose	28-FEB-2018	23:35	8	1	Replicate 1	1091
								Replicate 2	475
								Replicate 3	1026
								Geometric Mean	810.1310

Notes: Day relative to inoculum is the number of days relative to day of administration of the inoculum, where the day of administration = 0.
The inoculum was administered on Study Day 0 for K13-infected subjects and Study Day 1 for 3D7-infected subjects.
Day relative to IP is the number of days relative to day of administration of Artesunate, where the day of dose = 1.
qPCR = quantitative Polymerase Chain Reaction; IP = Artesunate; ND = Not Detected; RBCs: red blood cells.

Listing 16.2.6.3
Malaria 18S qPCR

Randomisation Number	Challenge Agent	Visit	Timepoint	Date of Measurement	Time of Measurement	Day Relative to Inoculum	Day Relative to Artesunate	Assay	Result (parasites/500µL) of packed RBCs
R208	3D7	Admission 1	16 Hrs Post-dose	01-MAR-2018	03:35	9	2	Replicate 1	204
								Replicate 2	280
								Replicate 3	98
								Geometric Mean	177.5571
			20 Hrs Post-dose	01-MAR-2018	07:39	9	2	Replicate 1	154
								Replicate 2	130
								Replicate 3	52
								Geometric Mean	101.3497
			24 Hrs Post-dose	01-MAR-2018	11:35	9	2	Replicate 1	19
								Replicate 2	20
								Replicate 3	ND
								Geometric Mean	7.2432
			28 Hrs Post-dose	01-MAR-2018	15:35	9	2	Replicate 1	83
								Replicate 2	ND
								Replicate 3	89
								Geometric Mean	19.4755

Notes: Day relative to inoculum is the number of days relative to day of administration of the inoculum, where the day of administration = 0.
The inoculum was administered on Study Day 0 for K13-infected subjects and Study Day 1 for 3D7-infected subjects.
Day relative to IP is the number of days relative to day of administration of Artesunate, where the day of dose = 1.
qPCR = quantitative Polymerase Chain Reaction; IP = Artesunate; ND = Not Detected; RBCs: red blood cells.

Listing 16.2.6.3
Malaria 18S qPCR

Randomisation Number	Challenge Agent	Visit	Timepoint	Date of Measurement	Time of Measurement	Day Relative to Inoculum	Day Relative to Artesunate	Assay	Result (parasites/500µL) of packed RBCs
R208	3D7	Admission 1	32 Hrs Post-dose	01-MAR-2018	19:35	9	2	Replicate 1	40
								Replicate 2	40
								Replicate 3	ND
								Geometric Mean	11.6961
		Admission 2	36 Hrs Post-dose	02-MAR-2018	00:03	10	3	Replicate 1	150
								Replicate 2	ND
								Replicate 3	ND
								Geometric Mean	5.3133
			48 Hrs Post-dose	02-MAR-2018	11:37	10	3	Replicate 1	62
								Replicate 2	ND
								Replicate 3	ND
								Geometric Mean	3.9579
			60 Hrs Post-dose	02-MAR-2018	23:39	10	3	Replicate 1	ND
								Replicate 2	ND
								Replicate 3	ND
								Geometric Mean	1.0000

Notes: Day relative to inoculum is the number of days relative to day of administration of the inoculum, where the day of administration = 0.
The inoculum was administered on Study Day 0 for K13-infected subjects and Study Day 1 for 3D7-infected subjects.
Day relative to IP is the number of days relative to day of administration of Artesunate, where the day of dose = 1.
qPCR = quantitative Polymerase Chain Reaction; IP = Artesunate; ND = Not Detected; RBCs: red blood cells.

Listing 16.2.6.3
Malaria 18S qPCR

Randomisation Number	Challenge Agent	Visit	Timepoint	Date of Measurement	Time of Measurement	Day Relative to Inoculum	Day Relative to Artesunate	Assay	Result (parasites/500μL) of packed RBCs
R208	3D7	Admission 3	72 Hrs Post-dose	03-MAR-2018	11:36	11	4	Replicate 1	50
								Replicate 2	ND
								Replicate 3	47
								Geometric Mean	13.2950
		Safety Monitoring		04-MAR-2018	09:41	12	5	Replicate 1	46
								Replicate 2	82
								Replicate 3	ND
								Geometric Mean	15.5665
				05-MAR-2018	07:07	13	6	Replicate 1	39
								Replicate 2	41
								Replicate 3	ND
								Geometric Mean	11.6936
				07-MAR-2018	07:14	15	8	Replicate 1	148
								Replicate 2	243
								Replicate 3	271
								Geometric Mean	213.6055

Notes: Day relative to inoculum is the number of days relative to day of administration of the inoculum, where the day of administration = 0.
The inoculum was administered on Study Day 0 for K13-infected subjects and Study Day 1 for 3D7-infected subjects.
Day relative to IP is the number of days relative to day of administration of Artesunate, where the day of dose = 1.
qPCR = quantitative Polymerase Chain Reaction; IP = Artesunate; ND = Not Detected; RBCs: red blood cells.

Listing 16.2.6.3
Malaria 18S qPCR

Randomisation Number	Challenge Agent	Visit	Timepoint	Date of Measurement	Time of Measurement	Day Relative to Inoculum	Day Relative to Artesunate	Assay	Result (parasites/500µL) of packed RBCs
R208	3D7	Safety Monitoring		08-MAR-2018	07:10	16	9	Replicate 1	1378
								Replicate 2	1086
								Replicate 3	1151
								Geometric Mean	1198.7210
				09-MAR-2018	07:19	17	10	Replicate 1	8496
								Replicate 2	9489
								Replicate 3	9491
								Geometric Mean	9146.3744
				10-MAR-2018	07:52	18	11	Replicate 1	3816
								Replicate 2	4647
								Replicate 3	4798
								Geometric Mean	4398.2552
				12-MAR-2018	07:11	20	13	Replicate 1	ND
								Replicate 2	105
								Replicate 3	ND
								Geometric Mean	4.7177

Notes: Day relative to inoculum is the number of days relative to day of administration of the inoculum, where the day of administration = 0.
The inoculum was administered on Study Day 0 for K13-infected subjects and Study Day 1 for 3D7-infected subjects.
Day relative to IP is the number of days relative to day of administration of Artesunate, where the day of dose = 1.
qPCR = quantitative Polymerase Chain Reaction; IP = Artesunate; ND = Not Detected; RBCs: red blood cells.

Listing 16.2.6.3
Malaria 18S qPCR

Randomisation Number	Challenge Agent	Visit	Timepoint	Date of Measurement	Time of Measurement	Day Relative to Inoculum	Day Relative to Artesunate	Assay	Result (parasites/500µL) of packed RBCs
R208	3D7	Safety Monitoring		13-MAR-2018	07:22	21	14	Replicate 1	ND
								Replicate 2	ND
								Replicate 3	ND
								Geometric Mean	1.0000
				14-MAR-2018	07:14	22	15	Replicate 1	ND
								Replicate 2	ND
								Replicate 3	ND
								Geometric Mean	1.0000
				16-MAR-2018	07:11	24	17	Replicate 1	ND
								Replicate 2	ND
								Replicate 3	ND
								Geometric Mean	1.0000
				19-MAR-2018	07:01	27	20	Replicate 1	ND
								Replicate 2	ND
								Replicate 3	ND
								Geometric Mean	1.0000

Notes: Day relative to inoculum is the number of days relative to day of administration of the inoculum, where the day of administration = 0.
The inoculum was administered on Study Day 0 for K13-infected subjects and Study Day 1 for 3D7-infected subjects.
Day relative to IP is the number of days relative to day of administration of Artesunate, where the day of dose = 1.
qPCR = quantitative Polymerase Chain Reaction; IP = Artesunate; ND = Not Detected; RBCs: red blood cells.

Listing 16.2.6.3
Malaria 18S qPCR

Randomisation Number	Challenge Agent	Visit	Timepoint	Date of Measurement	Time of Measurement	Day Relative to Inoculum	Day Relative to Artesunate	Assay	Result (parasites/500µL) of packed RBCs
R208	3D7	End of Study		22-MAR-2018	07:50	30	23	Replicate 1	ND
								Replicate 2	ND
								Replicate 3	ND
								Geometric Mean	1.0000
R209	3D7	Inoculum		20-FEB-2018	09:44	0	-8	Replicate 1	ND
								Replicate 2	ND
								Replicate 3	ND
								Geometric Mean	1.0000
		Malaria Monitoring		24-FEB-2018	07:42	4	-4	Replicate 1	40
								Replicate 2	35
								Replicate 3	73
								Geometric Mean	46.7538
				25-FEB-2018	07:45	5	-3	Replicate 1	494
								Replicate 2	688
								Replicate 3	589
								Geometric Mean	584.9834

Notes: Day relative to inoculum is the number of days relative to day of administration of the inoculum, where the day of administration = 0.
The inoculum was administered on Study Day 0 for K13-infected subjects and Study Day 1 for 3D7-infected subjects.
Day relative to IP is the number of days relative to day of administration of Artesunate, where the day of dose = 1.
qPCR = quantitative Polymerase Chain Reaction; IP = Artesunate; ND = Not Detected; RBCs: red blood cells.

Listing 16.2.6.3
Malaria 18S qPCR

Randomisation Number	Challenge Agent	Visit	Timepoint	Date of Measurement	Time of Measurement	Day Relative to Inoculum	Day Relative to Artesunate	Assay	Result (parasites/500µL) of packed RBCs
R209	3D7	Malaria Monitoring		25-FEB-2018	19:38	5	-3	Replicate 1	1700
								Replicate 2	971
								Replicate 3	1714
								Geometric Mean	1414.3590
				26-FEB-2018	07:42	6	-2	Replicate 1	535
								Replicate 2	129
								Replicate 3	302
								Geometric Mean	275.2011
				26-FEB-2018	19:18	6	-2	Replicate 1	3866
								Replicate 2	3174
								Replicate 3	3655
								Geometric Mean	3552.9196
				27-FEB-2018	07:40	7	-1	Replicate 1	17443
								Replicate 2	20029
								Replicate 3	15994
								Geometric Mean	17745.1298

Notes: Day relative to inoculum is the number of days relative to day of administration of the inoculum, where the day of administration = 0.
The inoculum was administered on Study Day 0 for K13-infected subjects and Study Day 1 for 3D7-infected subjects.
Day relative to IP is the number of days relative to day of administration of Artesunate, where the day of dose = 1.
qPCR = quantitative Polymerase Chain Reaction; IP = Artesunate; ND = Not Detected; RBCs: red blood cells.

Listing 16.2.6.3
Malaria 18S qPCR

Randomisation Number	Challenge Agent	Visit	Timepoint	Date of Measurement	Time of Measurement	Day Relative to Inoculum	Day Relative to Artesunate	Assay	Result (parasites/500µL) of packed RBCs
R209	3D7	Malaria Monitoring		27-FEB-2018	19:18	7	-1	Replicate 1	7743
								Replicate 2	5795
								Replicate 3	6323
								Geometric Mean	6570.9570
		Admission	Pre-dose	28-FEB-2018	14:42	8	1	Replicate 1	164996
								Replicate 2	172933
								Replicate 3	173716
								Geometric Mean	170502.3506
			2 Hrs Post-dose	28-FEB-2018	17:46	8	1	Replicate 1	217137
								Replicate 2	228717
								Replicate 3	195177
								Geometric Mean	213216.2525
			4 Hrs Post-dose	28-FEB-2018	19:45	8	1	Replicate 1	208925
								Replicate 2	186359
								Replicate 3	188051
								Geometric Mean	194180.4381

Notes: Day relative to inoculum is the number of days relative to day of administration of the inoculum, where the day of administration = 0.
The inoculum was administered on Study Day 0 for K13-infected subjects and Study Day 1 for 3D7-infected subjects.
Day relative to IP is the number of days relative to day of administration of Artesunate, where the day of dose = 1.
qPCR = quantitative Polymerase Chain Reaction; IP = Artesunate; ND = Not Detected; RBCs: red blood cells.

Listing 16.2.6.3
Malaria 18S qPCR

Randomisation Number	Challenge Agent	Visit	Timepoint	Date of Measurement	Time of Measurement	Day Relative to Inoculum	Day Relative to Artesunate	Assay	Result (parasites/500µL) of packed RBCs
R209	3D7	Admission	6 Hrs Post-dose	28-FEB-2018	21:46	8	1	Replicate 1	233379
								Replicate 2	219125
								Replicate 3	246827
								Geometric Mean	232835.2815
			8 Hrs Post-dose	28-FEB-2018	23:57	8	1	Replicate 1	204490
								Replicate 2	186612
								Replicate 3	173168
								Geometric Mean	187654.8361
		Admission 1	12 Hrs Post-dose	01-MAR-2018	03:48	9	2	Replicate 1	58741
								Replicate 2	60205
								Replicate 3	55981
								Geometric Mean	58282.5014
			16 Hrs Post-dose	01-MAR-2018	07:47	9	2	Replicate 1	9165
								Replicate 2	8491
								Replicate 3	8729
								Geometric Mean	8790.6017

Notes: Day relative to inoculum is the number of days relative to day of administration of the inoculum, where the day of administration = 0.
The inoculum was administered on Study Day 0 for K13-infected subjects and Study Day 1 for 3D7-infected subjects.
Day relative to IP is the number of days relative to day of administration of Artesunate, where the day of dose = 1.
qPCR = quantitative Polymerase Chain Reaction; IP = Artesunate; ND = Not Detected; RBCs: red blood cells.

Listing 16.2.6.3
Malaria 18S qPCR

Randomisation Number	Challenge Agent	Visit	Timepoint	Date of Measurement	Time of Measurement	Day Relative to Inoculum	Day Relative to Artesunate	Assay	Result (parasites/500µL) of packed RBCs
R209	3D7	Admission 1	20 Hrs Post-dose	01-MAR-2018	11:47	9	2	Replicate 1	2161
								Replicate 2	2337
								Replicate 3	1868
								Geometric Mean	2112.9868
			24 Hrs Post-dose	01-MAR-2018	15:45	9	2	Replicate 1	445
								Replicate 2	533
								Replicate 3	500
								Geometric Mean	491.3064
			28 Hrs Post-dose	01-MAR-2018	19:45	9	2	Replicate 1	299
								Replicate 2	81
								Replicate 3	132
								Geometric Mean	147.3138
			32 Hrs Post-dose	01-MAR-2018	23:45	9	2	Replicate 1	120
								Replicate 2	257
								Replicate 3	73
								Geometric Mean	131.0627

Notes: Day relative to inoculum is the number of days relative to day of administration of the inoculum, where the day of administration = 0.
The inoculum was administered on Study Day 0 for K13-infected subjects and Study Day 1 for 3D7-infected subjects.
Day relative to IP is the number of days relative to day of administration of Artesunate, where the day of dose = 1.
qPCR = quantitative Polymerase Chain Reaction; IP = Artesunate; ND = Not Detected; RBCs: red blood cells.

Listing 16.2.6.3
Malaria 18S qPCR

Randomisation Number	Challenge Agent	Visit	Timepoint	Date of Measurement	Time of Measurement	Day Relative to Inoculum	Day Relative to Artesunate	Assay	Result (parasites/500µL) of packed RBCs
R209	3D7	Admission 2	36 Hrs Post-dose	02-MAR-2018	03:45	10	3	Replicate 1	122
								Replicate 2	ND
								Replicate 3	ND
								Geometric Mean	4.9597
			48 Hrs Post-dose	02-MAR-2018	15:48	10	3	Replicate 1	ND
								Replicate 2	50
								Replicate 3	43
								Geometric Mean	12.9066
		Admission 3	60 Hrs Post-dose	03-MAR-2018	03:45	11	4	Replicate 1	34
								Replicate 2	ND
								Replicate 3	73
								Geometric Mean	13.5394
			72 Hrs Post-dose	03-MAR-2018	15:47	11	4	Replicate 1	ND
								Replicate 2	ND
								Replicate 3	44
								Geometric Mean	3.5303

Notes: Day relative to inoculum is the number of days relative to day of administration of the inoculum, where the day of administration = 0.
The inoculum was administered on Study Day 0 for K13-infected subjects and Study Day 1 for 3D7-infected subjects.
Day relative to IP is the number of days relative to day of administration of Artesunate, where the day of dose = 1.
qPCR = quantitative Polymerase Chain Reaction; IP = Artesunate; ND = Not Detected; RBCs: red blood cells.

Listing 16.2.6.3
Malaria 18S qPCR

Randomisation Number	Challenge Agent	Visit	Timepoint	Date of Measurement	Time of Measurement	Day Relative to Inoculum	Day Relative to Artesunate	Assay	Result (parasites/500µL) of packed RBCs
R209	3D7	Safety Monitoring		04-MAR-2018	08:08	12	5	Replicate 1	43
								Replicate 2	48
								Replicate 3	41
								Geometric Mean	43.9034
				05-MAR-2018	07:41	13	6	Replicate 1	71
								Replicate 2	113
								Replicate 3	56
								Geometric Mean	76.5905
				07-MAR-2018	07:48	15	8	Replicate 1	2721
								Replicate 2	2587
								Replicate 3	2722
								Geometric Mean	2675.9071
				08-MAR-2018	08:42	16	9	Replicate 1	29961
								Replicate 2	29035
								Replicate 3	27552
								Geometric Mean	28832.1540

Notes: Day relative to inoculum is the number of days relative to day of administration of the inoculum, where the day of administration = 0.
The inoculum was administered on Study Day 0 for K13-infected subjects and Study Day 1 for 3D7-infected subjects.
Day relative to IP is the number of days relative to day of administration of Artesunate, where the day of dose = 1.
qPCR = quantitative Polymerase Chain Reaction; IP = Artesunate; ND = Not Detected; RBCs: red blood cells.

Listing 16.2.6.3
Malaria 18S qPCR

Randomisation Number	Challenge Agent	Visit	Timepoint	Date of Measurement	Time of Measurement	Day Relative to Inoculum	Day Relative to Artesunate	Assay	Result (parasites/500µL) of packed RBCs
R209	3D7	Safety Monitoring		09-MAR-2018	08:34	17	10	Replicate 1	546
								Replicate 2	1047
								Replicate 3	253
								Geometric Mean	524.9121
				12-MAR-2018	08:12	20	13	Replicate 1	ND
								Replicate 2	ND
								Replicate 3	38
								Geometric Mean	3.3620
				14-MAR-2018	07:40	22	15	Replicate 1	ND
								Replicate 2	69
								Replicate 3	89
								Geometric Mean	18.3124
				16-MAR-2018	07:27	24	17	Replicate 1	39
								Replicate 2	83
								Replicate 3	208
								Geometric Mean	87.6467

Notes: Day relative to inoculum is the number of days relative to day of administration of the inoculum, where the day of administration = 0.
The inoculum was administered on Study Day 0 for K13-infected subjects and Study Day 1 for 3D7-infected subjects.
Day relative to IP is the number of days relative to day of administration of Artesunate, where the day of dose = 1.
qPCR = quantitative Polymerase Chain Reaction; IP = Artesunate; ND = Not Detected; RBCs: red blood cells.

Listing 16.2.6.3
Malaria 18S qPCR

Randomisation Number	Challenge Agent	Visit	Timepoint	Date of Measurement	Time of Measurement	Day Relative to Inoculum	Day Relative to Artesunate	Assay	Result (parasites/500µL) of packed RBCs
R209	3D7	Safety Monitoring		19-MAR-2018	07:48	27	20	Replicate 1	ND
								Replicate 2	39
								Replicate 3	ND
								Geometric Mean	3.3912
		End of Study		22-MAR-2018	08:16	30	23	Replicate 1	ND
								Replicate 2	ND
								Replicate 3	ND
								Geometric Mean	1.0000
R210	3D7	Inoculum		20-FEB-2018	10:09	0	-8	Replicate 1	ND
								Replicate 2	ND
								Replicate 3	ND
								Geometric Mean	1.0000
		Malaria Monitoring		24-FEB-2018	08:23	4	-4	Replicate 1	ND
								Replicate 2	35
								Replicate 3	ND
								Geometric Mean	3.2711

Notes: Day relative to inoculum is the number of days relative to day of administration of the inoculum, where the day of administration = 0.
The inoculum was administered on Study Day 0 for K13-infected subjects and Study Day 1 for 3D7-infected subjects.
Day relative to IP is the number of days relative to day of administration of Artesunate, where the day of dose = 1.
qPCR = quantitative Polymerase Chain Reaction; IP = Artesunate; ND = Not Detected; RBCs: red blood cells.

Listing 16.2.6.3
Malaria 18S qPCR

Randomisation Number	Challenge Agent	Visit	Timepoint	Date of Measurement	Time of Measurement	Day Relative to Inoculum	Day Relative to Artesunate	Assay	Result (parasites/500µL) of packed RBCs
R210	3D7	Malaria Monitoring		25-FEB-2018	07:25	5	-3	Replicate 1	645
								Replicate 2	951
								Replicate 3	366
								Geometric Mean	607.7717
				25-FEB-2018	21:14	5	-3	Replicate 1	623
								Replicate 2	683
								Replicate 3	965
								Geometric Mean	743.2679
				26-FEB-2018	07:18	6	-2	Replicate 1	206
								Replicate 2	303
								Replicate 3	96
								Geometric Mean	181.6326
				26-FEB-2018	20:40	6	-2	Replicate 1	7087
								Replicate 2	6285
								Replicate 3	5872
								Geometric Mean	6395.1576

Notes: Day relative to inoculum is the number of days relative to day of administration of the inoculum, where the day of administration = 0.
The inoculum was administered on Study Day 0 for K13-infected subjects and Study Day 1 for 3D7-infected subjects.
Day relative to IP is the number of days relative to day of administration of Artesunate, where the day of dose = 1.
qPCR = quantitative Polymerase Chain Reaction; IP = Artesunate; ND = Not Detected; RBCs: red blood cells.

Listing 16.2.6.3
Malaria 18S qPCR

Randomisation Number	Challenge Agent	Visit	Timepoint	Date of Measurement	Time of Measurement	Day Relative to Inoculum	Day Relative to Artesunate	Assay	Result (parasites/500µL) of packed RBCs
R210	3D7	Malaria Monitoring		27-FEB-2018	07:32	7	-1	Replicate 1	16052
								Replicate 2	13302
								Replicate 3	12349
								Geometric Mean	13815.2674
				27-FEB-2018	20:20	7	-1	Replicate 1	2122
								Replicate 2	1817
								Replicate 3	2399
								Geometric Mean	2099.1502
		Admission	Pre-dose	28-FEB-2018	11:21	8	1	Replicate 1	60724
								Replicate 2	62350
								Replicate 3	60368
								Geometric Mean	61141.2818
			2 Hrs Post-dose	28-FEB-2018	13:45	8	1	Replicate 1	94099
								Replicate 2	97408
								Replicate 3	95211
								Geometric Mean	95562.8119

Notes: Day relative to inoculum is the number of days relative to day of administration of the inoculum, where the day of administration = 0.
The inoculum was administered on Study Day 0 for K13-infected subjects and Study Day 1 for 3D7-infected subjects.
Day relative to IP is the number of days relative to day of administration of Artesunate, where the day of dose = 1.
qPCR = quantitative Polymerase Chain Reaction; IP = Artesunate; ND = Not Detected; RBCs: red blood cells.

Listing 16.2.6.3
Malaria 18S qPCR

Randomisation Number	Challenge Agent	Visit	Timepoint	Date of Measurement	Time of Measurement	Day Relative to Inoculum	Day Relative to Artesunate	Assay	Result (parasites/500µL) of packed RBCs
R210	3D7	Admission	4 Hrs Post-dose	28-FEB-2018	15:45	8	1	Replicate 1	120561
								Replicate 2	137097
								Replicate 3	120452
								Geometric Mean	125800.6648
			6 Hrs Post-dose	28-FEB-2018	17:45	8	1	Replicate 1	106523
								Replicate 2	102835
								Replicate 3	104003
								Geometric Mean	104442.3743
			8 Hrs Post-dose	28-FEB-2018	19:45	8	1	Replicate 1	60124
								Replicate 2	74481
								Replicate 3	67924
								Geometric Mean	67252.0519
			12 Hrs Post-dose	28-FEB-2018	23:45	8	1	Replicate 1	17424
								Replicate 2	18586
								Replicate 3	18883
								Geometric Mean	18286.6827

Notes: Day relative to inoculum is the number of days relative to day of administration of the inoculum, where the day of administration = 0.
The inoculum was administered on Study Day 0 for K13-infected subjects and Study Day 1 for 3D7-infected subjects.
Day relative to IP is the number of days relative to day of administration of Artesunate, where the day of dose = 1.
qPCR = quantitative Polymerase Chain Reaction; IP = Artesunate; ND = Not Detected; RBCs: red blood cells.

Listing 16.2.6.3
Malaria 18S qPCR

Randomisation Number	Challenge Agent	Visit	Timepoint	Date of Measurement	Time of Measurement	Day Relative to Inoculum	Day Relative to Artesunate	Assay	Result (parasites/500µL) of packed RBCs
R210	3D7	Admission 1	16 Hrs Post-dose	01-MAR-2018	03:46	9	2	Replicate 1	1058
								Replicate 2	1291
								Replicate 3	1542
								Geometric Mean	1281.8349
			20 Hrs Post-dose	01-MAR-2018	07:45	9	2	Replicate 1	958
								Replicate 2	526
								Replicate 3	380
								Geometric Mean	576.3836
			24 Hrs Post-dose	01-MAR-2018	11:45	9	2	Replicate 1	317
								Replicate 2	294
								Replicate 3	238
								Geometric Mean	280.9708
			28 Hrs Post-dose	01-MAR-2018	15:45	9	2	Replicate 1	89
								Replicate 2	91
								Replicate 3	84
								Geometric Mean	87.9502

Notes: Day relative to inoculum is the number of days relative to day of administration of the inoculum, where the day of administration = 0.
The inoculum was administered on Study Day 0 for K13-infected subjects and Study Day 1 for 3D7-infected subjects.
Day relative to IP is the number of days relative to day of administration of Artesunate, where the day of dose = 1.
qPCR = quantitative Polymerase Chain Reaction; IP = Artesunate; ND = Not Detected; RBCs: red blood cells.

Listing 16.2.6.3
Malaria 18S qPCR

Randomisation Number	Challenge Agent	Visit	Timepoint	Date of Measurement	Time of Measurement	Day Relative to Inoculum	Day Relative to Artesunate	Assay	Result (parasites/500µL) of packed RBCs
R210	3D7	Admission 1	32 Hrs Post-dose	01-MAR-2018	19:45	9	2	Replicate 1	100
								Replicate 2	43
								Replicate 3	76
								Geometric Mean	68.8801
			36 Hrs Post-dose	01-MAR-2018	23:46	9	2	Replicate 1	35
								Replicate 2	ND
								Replicate 3	ND
								Geometric Mean	3.2711
		Admission 2	48 Hrs Post-dose	02-MAR-2018	11:58	10	3	Replicate 1	ND
								Replicate 2	46
								Replicate 3	ND
								Geometric Mean	3.5830
			60 Hrs Post-dose	02-MAR-2018	23:46	10	3	Replicate 1	ND
								Replicate 2	ND
								Replicate 3	ND
								Geometric Mean	1.0000

Notes: Day relative to inoculum is the number of days relative to day of administration of the inoculum, where the day of administration = 0.
The inoculum was administered on Study Day 0 for K13-infected subjects and Study Day 1 for 3D7-infected subjects.
Day relative to IP is the number of days relative to day of administration of Artesunate, where the day of dose = 1.
qPCR = quantitative Polymerase Chain Reaction; IP = Artesunate; ND = Not Detected; RBCs: red blood cells.

Listing 16.2.6.3
Malaria 18S qPCR

Randomisation Number	Challenge Agent	Visit	Timepoint	Date of Measurement	Time of Measurement	Day Relative to Inoculum	Day Relative to Artesunate	Assay	Result (parasites/500µL) of packed RBCs
R210	3D7	Admission 3	72 Hrs Post-dose	03-MAR-2018	12:01	11	4	Replicate 1	72
								Replicate 2	95
								Replicate 3	ND
								Geometric Mean	18.9824
		Safety Monitoring		04-MAR-2018	07:10	12	5	Replicate 1	44
								Replicate 2	75
								Replicate 3	48
								Geometric Mean	54.1068
				05-MAR-2018	07:40	13	6	Replicate 1	115
								Replicate 2	71
								Replicate 3	90
								Geometric Mean	90.2401
				07-MAR-2018	07:26	15	8	Replicate 1	8863
								Replicate 2	8275
								Replicate 3	10122
								Geometric Mean	9054.6506

Notes: Day relative to inoculum is the number of days relative to day of administration of the inoculum, where the day of administration = 0.
The inoculum was administered on Study Day 0 for K13-infected subjects and Study Day 1 for 3D7-infected subjects.
Day relative to IP is the number of days relative to day of administration of Artesunate, where the day of dose = 1.
qPCR = quantitative Polymerase Chain Reaction; IP = Artesunate; ND = Not Detected; RBCs: red blood cells.

Listing 16.2.6.3
Malaria 18S qPCR

Randomisation Number	Challenge Agent	Visit	Timepoint	Date of Measurement	Time of Measurement	Day Relative to Inoculum	Day Relative to Artesunate	Assay	Result (parasites/500µL) of packed RBCs
R210	3D7	Safety Monitoring		08-MAR-2018	07:40	16	9	Replicate 1	18002
								Replicate 2	20826
								Replicate 3	20260
								Geometric Mean	19657.2165
				09-MAR-2018	09:45	17	10	Replicate 1	87
								Replicate 2	263
								Replicate 3	247
								Geometric Mean	178.1246
				12-MAR-2018	07:37	20	13	Replicate 1	ND
								Replicate 2	ND
								Replicate 3	ND
								Geometric Mean	1.0000
				14-MAR-2018	07:17	22	15	Replicate 1	83
								Replicate 2	42
								Replicate 3	ND
								Geometric Mean	15.1627

Notes: Day relative to inoculum is the number of days relative to day of administration of the inoculum, where the day of administration = 0.
The inoculum was administered on Study Day 0 for K13-infected subjects and Study Day 1 for 3D7-infected subjects.
Day relative to IP is the number of days relative to day of administration of Artesunate, where the day of dose = 1.
qPCR = quantitative Polymerase Chain Reaction; IP = Artesunate; ND = Not Detected; RBCs: red blood cells.

Listing 16.2.6.3
Malaria 18S qPCR

Randomisation Number	Challenge Agent	Visit	Timepoint	Date of Measurement	Time of Measurement	Day Relative to Inoculum	Day Relative to Artesunate	Assay	Result (parasites/500µL) of packed RBCs
R210	3D7	Safety Monitoring		16-MAR-2018	07:38	24	17	Replicate 1	113
								Replicate 2	32
								Replicate 3	85
								Geometric Mean	67.4863
				19-MAR-2018	07:39	27	20	Replicate 1	44
								Replicate 2	ND
								Replicate 3	ND
								Geometric Mean	3.5303
		End of Study		22-MAR-2018	07:34	30	23	Replicate 1	ND
								Replicate 2	ND
								Replicate 3	ND
								Geometric Mean	1.0000
R302	K13	Inoculum		04-SEP-2018	09:46	0	-9	Replicate 1	ND
								Replicate 2	ND
								Replicate 3	ND
								Geometric Mean	1.0000

Notes: Day relative to inoculum is the number of days relative to day of administration of the inoculum, where the day of administration = 0.
The inoculum was administered on Study Day 0 for K13-infected subjects and Study Day 1 for 3D7-infected subjects.
Day relative to IP is the number of days relative to day of administration of Artesunate, where the day of dose = 1.
qPCR = quantitative Polymerase Chain Reaction; IP = Artesunate; ND = Not Detected; RBCs: red blood cells.

Listing 16.2.6.3
Malaria 18S qPCR

Randomisation Number	Challenge Agent	Visit	Timepoint	Date of Measurement	Time of Measurement	Day Relative to Inoculum	Day Relative to Artesunate	Assay	Result (parasites/500µL) of packed RBCs
R302	K13	Malaria Monitoring		08-SEP-2018	07:15	4	-5	Replicate 1	37
								Replicate 2	ND
								Replicate 3	ND
								Geometric Mean	3.3322
				09-SEP-2018	08:19	5	-4	Replicate 1	34
								Replicate 2	34
								Replicate 3	ND
								Geometric Mean	10.4951
				09-SEP-2018	19:19	5	-4	Replicate 1	992
								Replicate 2	769
								Replicate 3	803
								Geometric Mean	849.2806
				10-SEP-2018	07:26	6	-3	Replicate 1	1142
								Replicate 2	1024
								Replicate 3	1355
								Geometric Mean	1165.8297

Notes: Day relative to inoculum is the number of days relative to day of administration of the inoculum, where the day of administration = 0.
The inoculum was administered on Study Day 0 for K13-infected subjects and Study Day 1 for 3D7-infected subjects.
Day relative to IP is the number of days relative to day of administration of Artesunate, where the day of dose = 1.
qPCR = quantitative Polymerase Chain Reaction; IP = Artesunate; ND = Not Detected; RBCs: red blood cells.

Listing 16.2.6.3
Malaria 18S qPCR

Randomisation Number	Challenge Agent	Visit	Timepoint	Date of Measurement	Time of Measurement	Day Relative to Inoculum	Day Relative to Artesunate	Assay	Result (parasites/500µL) of packed RBCs
R302	K13	Malaria Monitoring		10-SEP-2018	19:27	6	-3	Replicate 1	910
								Replicate 2	681
								Replicate 3	957
								Geometric Mean	840.1693
				11-SEP-2018	07:24	7	-2	Replicate 1	2937
								Replicate 2	1934
								Replicate 3	2000
								Geometric Mean	2248.0026
				11-SEP-2018	19:27	7	-2	Replicate 1	16284
								Replicate 2	13199
								Replicate 3	16120
								Geometric Mean	15131.7464
				12-SEP-2018	07:16	8	-1	Replicate 1	17246
								Replicate 2	16634
								Replicate 3	19519
								Geometric Mean	17757.4615

Notes: Day relative to inoculum is the number of days relative to day of administration of the inoculum, where the day of administration = 0.
The inoculum was administered on Study Day 0 for K13-infected subjects and Study Day 1 for 3D7-infected subjects.
Day relative to IP is the number of days relative to day of administration of Artesunate, where the day of dose = 1.
qPCR = quantitative Polymerase Chain Reaction; IP = Artesunate; ND = Not Detected; RBCs: red blood cells.

Listing 16.2.6.3
Malaria 18S qPCR

Randomisation Number	Challenge Agent	Visit	Timepoint	Date of Measurement	Time of Measurement	Day Relative to Inoculum	Day Relative to Artesunate	Assay	Result (parasites/500µL) of packed RBCs
R302	K13	Malaria Monitoring		12-SEP-2018	19:24	8	-1	Replicate 1	18426
								Replicate 2	15939
								Replicate 3	16766
								Geometric Mean	17012.7244
		Admission	Pre-dose	13-SEP-2018	08:57	9	1	Replicate 1	74493
								Replicate 2	70625
								Replicate 3	69266
								Geometric Mean	71427.3731
			4 Hrs Post-dose	13-SEP-2018	13:05	9	1	Replicate 1	93752
								Replicate 2	96765
								Replicate 3	106587
								Geometric Mean	98885.8977
			8 Hrs Post-dose	13-SEP-2018	17:06	9	1	Replicate 1	91493
								Replicate 2	73673
								Replicate 3	96329
								Geometric Mean	86593.3268

Notes: Day relative to inoculum is the number of days relative to day of administration of the inoculum, where the day of administration = 0.
The inoculum was administered on Study Day 0 for K13-infected subjects and Study Day 1 for 3D7-infected subjects.
Day relative to IP is the number of days relative to day of administration of Artesunate, where the day of dose = 1.
qPCR = quantitative Polymerase Chain Reaction; IP = Artesunate; ND = Not Detected; RBCs: red blood cells.

Listing 16.2.6.3
Malaria 18S qPCR

Randomisation Number	Challenge Agent	Visit	Timepoint	Date of Measurement	Time of Measurement	Day Relative to Inoculum	Day Relative to Artesunate	Assay	Result (parasites/500µL) of packed RBCs
R302	K13	Admission	12 Hrs Post-dose	13-SEP-2018	21:05	9	1	Replicate 1	45576
								Replicate 2	41357
								Replicate 3	47264
								Geometric Mean	44662.0414
		Admission 1	16 Hrs Post-dose	14-SEP-2018	01:05	10	2	Replicate 1	32984
								Replicate 2	30366
								Replicate 3	35376
								Geometric Mean	32844.7942
			20 Hrs Post-dose	14-SEP-2018	05:05	10	2	Replicate 1	21585
								Replicate 2	22395
								Replicate 3	22324
								Geometric Mean	22098.2752
			24 Hrs Post-dose	14-SEP-2018	09:15	10	2	Replicate 1	15588
								Replicate 2	16516
								Replicate 3	15659
								Geometric Mean	15915.4809

Notes: Day relative to inoculum is the number of days relative to day of administration of the inoculum, where the day of administration = 0.
The inoculum was administered on Study Day 0 for K13-infected subjects and Study Day 1 for 3D7-infected subjects.
Day relative to IP is the number of days relative to day of administration of Artesunate, where the day of dose = 1.
qPCR = quantitative Polymerase Chain Reaction; IP = Artesunate; ND = Not Detected; RBCs: red blood cells.

Listing 16.2.6.3
Malaria 18S qPCR

Randomisation Number	Challenge Agent	Visit	Timepoint	Date of Measurement	Time of Measurement	Day Relative to Inoculum	Day Relative to Artesunate	Assay	Result (parasites/500µL) of packed RBCs
R302	K13	Admission 1	28 Hrs Post-dose	14-SEP-2018	13:05	10	2	Replicate 1	7463
								Replicate 2	6173
								Replicate 3	7535
								Geometric Mean	7027.9877
			32 Hrs Post-dose	14-SEP-2018	17:05	10	2	Replicate 1	6076
								Replicate 2	4716
								Replicate 3	5729
								Geometric Mean	5475.4959
			36 Hrs Post-dose	14-SEP-2018	21:07	10	2	Replicate 1	2489
								Replicate 2	2248
								Replicate 3	2988
								Geometric Mean	2557.0189
		Admission 2		15-SEP-2018	01:06	11	3	Replicate 1	1537
								Replicate 2	1019
								Replicate 3	2164
								Geometric Mean	1502.1101

Notes: Day relative to inoculum is the number of days relative to day of administration of the inoculum, where the day of administration = 0.
The inoculum was administered on Study Day 0 for K13-infected subjects and Study Day 1 for 3D7-infected subjects.
Day relative to IP is the number of days relative to day of administration of Artesunate, where the day of dose = 1.
qPCR = quantitative Polymerase Chain Reaction; IP = Artesunate; ND = Not Detected; RBCs: red blood cells.

Listing 16.2.6.3
Malaria 18S qPCR

Randomisation Number	Challenge Agent	Visit	Timepoint	Date of Measurement	Time of Measurement	Day Relative to Inoculum	Day Relative to Artesunate	Assay	Result (parasites/500µL) of packed RBCs
R302	K13	Admission 2		15-SEP-2018	05:06	11	3	Replicate 1	2482
								Replicate 2	4676
								Replicate 3	3535
								Geometric Mean	3448.9632
			48 Hrs Post-dose	15-SEP-2018	09:06	11	3	Replicate 1	11464
								Replicate 2	11142
								Replicate 3	10006
								Geometric Mean	10852.2538
			52 Hrs Post-dose	15-SEP-2018	13:05	11	3	Replicate 1	32755
								Replicate 2	31485
								Replicate 3	33337
								Geometric Mean	32516.4111
			56 Hrs Post-dose	15-SEP-2018	17:05	11	3	Replicate 1	37509
								Replicate 2	39048
								Replicate 3	39832
								Geometric Mean	38784.2560

Notes: Day relative to inoculum is the number of days relative to day of administration of the inoculum, where the day of administration = 0.
The inoculum was administered on Study Day 0 for K13-infected subjects and Study Day 1 for 3D7-infected subjects.
Day relative to IP is the number of days relative to day of administration of Artesunate, where the day of dose = 1.
qPCR = quantitative Polymerase Chain Reaction; IP = Artesunate; ND = Not Detected; RBCs: red blood cells.

Listing 16.2.6.3
Malaria 18S qPCR

Randomisation Number	Challenge Agent	Visit	Timepoint	Date of Measurement	Time of Measurement	Day Relative to Inoculum	Day Relative to Artesunate	Assay	Result (parasites/500µL) of packed RBCs
R302	K13	Admission 2	60 Hrs Post-dose	15-SEP-2018	21:05	11	3	Replicate 1	30998
								Replicate 2	32963
								Replicate 3	33549
								Geometric Mean	32484.7849
		Admission 3		16-SEP-2018	01:06	12	4	Replicate 1	24275
								Replicate 2	23035
								Replicate 3	28679
								Geometric Mean	25217.6040
				16-SEP-2018	05:06	12	4	Replicate 1	18069
								Replicate 2	16760
								Replicate 3	14062
								Geometric Mean	16208.8328
			72 Hrs Post-dose	16-SEP-2018	09:05	12	4	Replicate 1	11892
								Replicate 2	13293
								Replicate 3	14758
								Geometric Mean	13262.7975

Notes: Day relative to inoculum is the number of days relative to day of administration of the inoculum, where the day of administration = 0.
The inoculum was administered on Study Day 0 for K13-infected subjects and Study Day 1 for 3D7-infected subjects.
Day relative to IP is the number of days relative to day of administration of Artesunate, where the day of dose = 1.
qPCR = quantitative Polymerase Chain Reaction; IP = Artesunate; ND = Not Detected; RBCs: red blood cells.

Listing 16.2.6.3
Malaria 18S qPCR

Randomisation Number	Challenge Agent	Visit	Timepoint	Date of Measurement	Time of Measurement	Day Relative to Inoculum	Day Relative to Artesunate	Assay	Result (parasites/500µL) of packed RBCs
R302	K13	Admission 3		16-SEP-2018	20:00	12	4	Replicate 1	1646
								Replicate 2	1282
								Replicate 3	1830
								Geometric Mean	1568.8797
		Safety Monitoring		17-SEP-2018	08:00	13	5	Replicate 1	551
								Replicate 2	712
								Replicate 3	718
								Geometric Mean	655.5191
				18-SEP-2018	08:04	14	6	Replicate 1	120
								Replicate 2	177
								Replicate 3	170
								Geometric Mean	153.4150
				19-SEP-2018	07:31	15	7	Replicate 1	34
								Replicate 2	ND
								Replicate 3	20
								Geometric Mean	8.7937

Notes: Day relative to inoculum is the number of days relative to day of administration of the inoculum, where the day of administration = 0.
The inoculum was administered on Study Day 0 for K13-infected subjects and Study Day 1 for 3D7-infected subjects.
Day relative to IP is the number of days relative to day of administration of Artesunate, where the day of dose = 1.
qPCR = quantitative Polymerase Chain Reaction; IP = Artesunate; ND = Not Detected; RBCs: red blood cells.

Listing 16.2.6.3
Malaria 18S qPCR

Randomisation Number	Challenge Agent	Visit	Timepoint	Date of Measurement	Time of Measurement	Day Relative to Inoculum	Day Relative to Artesunate	Assay	Result (parasites/500µL) of packed RBCs
R302	K13	Safety Monitoring		20-SEP-2018	07:25	16	8	Replicate 1	ND
								Replicate 2	ND
								Replicate 3	13
								Geometric Mean	2.3513
				22-SEP-2018	07:56	18	10	Replicate 1	193
								Replicate 2	369
								Replicate 3	263
								Geometric Mean	265.5705
				24-SEP-2018	07:22	20	12	Replicate 1	2103
								Replicate 2	2211
								Replicate 3	1777
								Geometric Mean	2021.6462
				25-SEP-2018	08:13	21	13	Replicate 1	5639
								Replicate 2	5517
								Replicate 3	6443
								Geometric Mean	5852.3605

Notes: Day relative to inoculum is the number of days relative to day of administration of the inoculum, where the day of administration = 0.
The inoculum was administered on Study Day 0 for K13-infected subjects and Study Day 1 for 3D7-infected subjects.
Day relative to IP is the number of days relative to day of administration of Artesunate, where the day of dose = 1.
qPCR = quantitative Polymerase Chain Reaction; IP = Artesunate; ND = Not Detected; RBCs: red blood cells.

Listing 16.2.6.3
Malaria 18S qPCR

Randomisation Number	Challenge Agent	Visit	Timepoint	Date of Measurement	Time of Measurement	Day Relative to Inoculum	Day Relative to Artesunate	Assay	Result (parasites/500µL) of packed RBCs
R302	K13	Safety Monitoring		27-SEP-2018	07:19	23	15	Replicate 1	750
								Replicate 2	538
								Replicate 3	515
								Geometric Mean	592.3116
				28-SEP-2018	08:22	24	16	Replicate 1	103
								Replicate 2	68
								Replicate 3	92
								Geometric Mean	86.3730
		End of Study		02-OCT-2018	08:19	28	20	Replicate 1	27
								Replicate 2	25
								Replicate 3	25
								Geometric Mean	25.6496
		Unscheduled		05-OCT-2018	07:20	31	23	Replicate 1	ND
								Replicate 2	21
								Replicate 3	25
								Geometric Mean	8.0671

Notes: Day relative to inoculum is the number of days relative to day of administration of the inoculum, where the day of administration = 0.
The inoculum was administered on Study Day 0 for K13-infected subjects and Study Day 1 for 3D7-infected subjects.
Day relative to IP is the number of days relative to day of administration of Artesunate, where the day of dose = 1.
qPCR = quantitative Polymerase Chain Reaction; IP = Artesunate; ND = Not Detected; RBCs: red blood cells.

Listing 16.2.6.3
Malaria 18S qPCR

Randomisation Number	Challenge Agent	Visit	Timepoint	Date of Measurement	Time of Measurement	Day Relative to Inoculum	Day Relative to Artesunate	Assay	Result (parasites/500µL) of packed RBCs
R302	K13	Unscheduled		11-OCT-2018	07:17	37	29	Replicate 1	ND
								Replicate 2	ND
								Replicate 3	ND
								Geometric Mean	1.0000
R303	K13	Inoculum		04-SEP-2018	09:53	0	-9	Replicate 1	ND
								Replicate 2	ND
								Replicate 3	ND
								Geometric Mean	1.0000
		Malaria Monitoring		08-SEP-2018	08:08	4	-5	Replicate 1	98
								Replicate 2	ND
								Replicate 3	33
								Geometric Mean	14.7881
				09-SEP-2018	08:00	5	-4	Replicate 1	ND
								Replicate 2	58
								Replicate 3	112
								Geometric Mean	18.6587

Notes: Day relative to inoculum is the number of days relative to day of administration of the inoculum, where the day of administration = 0.
The inoculum was administered on Study Day 0 for K13-infected subjects and Study Day 1 for 3D7-infected subjects.
Day relative to IP is the number of days relative to day of administration of Artesunate, where the day of dose = 1.
qPCR = quantitative Polymerase Chain Reaction; IP = Artesunate; ND = Not Detected; RBCs: red blood cells.

Printed: 05MAR2020 (Final)

Listing 16.2.6.3
Malaria 18S qPCR

Randomisation Number	Challenge Agent	Visit	Timepoint	Date of Measurement	Time of Measurement	Day Relative to Inoculum	Day Relative to Artesunate	Assay	Result (parasites/500µL) of packed RBCs
R303	K13	Malaria Monitoring		09-SEP-2018	19:03	5	-4	Replicate 1	1267
								Replicate 2	1111
								Replicate 3	1235
								Geometric Mean	1202.4099
				10-SEP-2018	07:00	6	-3	Replicate 1	1478
								Replicate 2	2383
								Replicate 3	1881
								Geometric Mean	1878.1449
				10-SEP-2018	18:57	6	-3	Replicate 1	1697
								Replicate 2	1770
								Replicate 3	1476
								Geometric Mean	1642.7841
				11-SEP-2018	07:01	7	-2	Replicate 1	4473
								Replicate 2	4819
								Replicate 3	4347
								Geometric Mean	4542.0142

Notes: Day relative to inoculum is the number of days relative to day of administration of the inoculum, where the day of administration = 0.
The inoculum was administered on Study Day 0 for K13-infected subjects and Study Day 1 for 3D7-infected subjects.
Day relative to IP is the number of days relative to day of administration of Artesunate, where the day of dose = 1.
qPCR = quantitative Polymerase Chain Reaction; IP = Artesunate; ND = Not Detected; RBCs: red blood cells.

Listing 16.2.6.3
Malaria 18S qPCR

Randomisation Number	Challenge Agent	Visit	Timepoint	Date of Measurement	Time of Measurement	Day Relative to Inoculum	Day Relative to Artesunate	Assay	Result (parasites/500µL) of packed RBCs
R303	K13	Malaria Monitoring		11-SEP-2018	18:54	7	-2	Replicate 1	23908
								Replicate 2	21163
								Replicate 3	21125
								Geometric Mean	22027.8646
				12-SEP-2018	08:17	8	-1	Replicate 1	32846
								Replicate 2	34912
								Replicate 3	34676
								Geometric Mean	34132.0262
				12-SEP-2018	20:06	8	-1	Replicate 1	35213
								Replicate 2	38866
								Replicate 3	38761
								Geometric Mean	37574.1601
		Admission	Pre-dose	13-SEP-2018	11:17	9	1	Replicate 1	178718
								Replicate 2	160498
								Replicate 3	204059
								Geometric Mean	180217.8862

Notes: Day relative to inoculum is the number of days relative to day of administration of the inoculum, where the day of administration = 0.
The inoculum was administered on Study Day 0 for K13-infected subjects and Study Day 1 for 3D7-infected subjects.
Day relative to IP is the number of days relative to day of administration of Artesunate, where the day of dose = 1.
qPCR = quantitative Polymerase Chain Reaction; IP = Artesunate; ND = Not Detected; RBCs: red blood cells.

Listing 16.2.6.3
Malaria 18S qPCR

Randomisation Number	Challenge Agent	Visit	Timepoint	Date of Measurement	Time of Measurement	Day Relative to Inoculum	Day Relative to Artesunate	Assay	Result (parasites/500µL) of packed RBCs
R303	K13	Admission	4 Hrs Post-dose	13-SEP-2018	15:20	9	1	Replicate 1	210283
								Replicate 2	279521
								Replicate 3	249330
								Geometric Mean	244717.1357
			8 Hrs Post-dose	13-SEP-2018	19:18	9	1	Replicate 1	229649
								Replicate 2	227789
								Replicate 3	236172
								Geometric Mean	231175.5527
			12 Hrs Post-dose	13-SEP-2018	23:19	9	1	Replicate 1	117301
								Replicate 2	143487
								Replicate 3	134115
								Geometric Mean	131178.8667
		Admission 1	16 Hrs Post-dose	14-SEP-2018	03:22	10	2	Replicate 1	123766
								Replicate 2	114746
								Replicate 3	116627
								Geometric Mean	118316.6882

Notes: Day relative to inoculum is the number of days relative to day of administration of the inoculum, where the day of administration = 0.
The inoculum was administered on Study Day 0 for K13-infected subjects and Study Day 1 for 3D7-infected subjects.
Day relative to IP is the number of days relative to day of administration of Artesunate, where the day of dose = 1.
qPCR = quantitative Polymerase Chain Reaction; IP = Artesunate; ND = Not Detected; RBCs: red blood cells.

Listing 16.2.6.3
Malaria 18S qPCR

Randomisation Number	Challenge Agent	Visit	Timepoint	Date of Measurement	Time of Measurement	Day Relative to Inoculum	Day Relative to Artesunate	Assay	Result (parasites/500µL) of packed RBCs
R303	K13	Admission 1	20 Hrs Post-dose	14-SEP-2018	07:18	10	2	Replicate 1	79061
								Replicate 2	87106
								Replicate 3	81270
								Geometric Mean	82410.0564
			24 Hrs Post-dose	14-SEP-2018	11:18	10	2	Replicate 1	67330
								Replicate 2	69274
								Replicate 3	63691
								Geometric Mean	66724.5493
			28 Hrs Post-dose	14-SEP-2018	15:19	10	2	Replicate 1	33579
								Replicate 2	37189
								Replicate 3	31174
								Geometric Mean	33891.5629
			32 Hrs Post-dose	14-SEP-2018	19:22	10	2	Replicate 1	18379
								Replicate 2	12932
								Replicate 3	18978
								Geometric Mean	16522.6361

Notes: Day relative to inoculum is the number of days relative to day of administration of the inoculum, where the day of administration = 0.
The inoculum was administered on Study Day 0 for K13-infected subjects and Study Day 1 for 3D7-infected subjects.
Day relative to IP is the number of days relative to day of administration of Artesunate, where the day of dose = 1.
qPCR = quantitative Polymerase Chain Reaction; IP = Artesunate; ND = Not Detected; RBCs: red blood cells.

Listing 16.2.6.3
Malaria 18S qPCR

Randomisation Number	Challenge Agent	Visit	Timepoint	Date of Measurement	Time of Measurement	Day Relative to Inoculum	Day Relative to Artesunate	Assay	Result (parasites/500µL) of packed RBCs
R303	K13	Admission 1	36 Hrs Post-dose	14-SEP-2018	23:19	10	2	Replicate 1	8412
								Replicate 2	10119
								Replicate 3	9873
								Geometric Mean	9436.8851
		Admission 2		15-SEP-2018	03:22	11	3	Replicate 1	7912
								Replicate 2	9650
								Replicate 3	11895
								Geometric Mean	9684.1018
				15-SEP-2018	07:18	11	3	Replicate 1	10671
								Replicate 2	9638
								Replicate 3	10751
								Geometric Mean	10340.6293
			48 Hrs Post-dose	15-SEP-2018	11:18	11	3	Replicate 1	17499
								Replicate 2	15552
								Replicate 3	16308
								Geometric Mean	16433.6258

Notes: Day relative to inoculum is the number of days relative to day of administration of the inoculum, where the day of administration = 0.
The inoculum was administered on Study Day 0 for K13-infected subjects and Study Day 1 for 3D7-infected subjects.
Day relative to IP is the number of days relative to day of administration of Artesunate, where the day of dose = 1.
qPCR = quantitative Polymerase Chain Reaction; IP = Artesunate; ND = Not Detected; RBCs: red blood cells.

Listing 16.2.6.3
Malaria 18S qPCR

Randomisation Number	Challenge Agent	Visit	Timepoint	Date of Measurement	Time of Measurement	Day Relative to Inoculum	Day Relative to Artesunate	Assay	Result (parasites/500µL) of packed RBCs
R303	K13	Admission 2	52 Hrs Post-dose	15-SEP-2018	15:20	11	3	Replicate 1	88418
								Replicate 2	86913
								Replicate 3	81292
								Geometric Mean	85485.2289
			56 Hrs Post-dose	15-SEP-2018	19:25	11	3	Replicate 1	119377
								Replicate 2	123296
								Replicate 3	127488
								Geometric Mean	123342.5678
			60 Hrs Post-dose	15-SEP-2018	23:20	11	3	Replicate 1	125886
								Replicate 2	141544
								Replicate 3	128065
								Geometric Mean	131653.7147
		Admission 3		16-SEP-2018	03:19	12	4	Replicate 1	94227
								Replicate 2	76424
								Replicate 3	86613
								Geometric Mean	85440.2945

Notes: Day relative to inoculum is the number of days relative to day of administration of the inoculum, where the day of administration = 0.
The inoculum was administered on Study Day 0 for K13-infected subjects and Study Day 1 for 3D7-infected subjects.
Day relative to IP is the number of days relative to day of administration of Artesunate, where the day of dose = 1.
qPCR = quantitative Polymerase Chain Reaction; IP = Artesunate; ND = Not Detected; RBCs: red blood cells.

Listing 16.2.6.3
Malaria 18S qPCR

Randomisation Number	Challenge Agent	Visit	Timepoint	Date of Measurement	Time of Measurement	Day Relative to Inoculum	Day Relative to Artesunate	Assay	Result (parasites/500µL) of packed RBCs
R303	K13	Admission 3		16-SEP-2018	07:20	12	4	Replicate 1	67328
								Replicate 2	67985
								Replicate 3	70059
								Geometric Mean	68447.4989
			72 Hrs Post-dose	16-SEP-2018	11:18	12	4	Replicate 1	49530
								Replicate 2	44763
								Replicate 3	48326
								Geometric Mean	47495.8999
				16-SEP-2018	19:49	12	4	Replicate 1	12296
								Replicate 2	13419
								Replicate 3	11642
								Geometric Mean	12430.9371
		Safety Monitoring		17-SEP-2018	07:50	13	5	Replicate 1	2279
								Replicate 2	2567
								Replicate 3	2481
								Geometric Mean	2439.3025

Notes: Day relative to inoculum is the number of days relative to day of administration of the inoculum, where the day of administration = 0.
The inoculum was administered on Study Day 0 for K13-infected subjects and Study Day 1 for 3D7-infected subjects.
Day relative to IP is the number of days relative to day of administration of Artesunate, where the day of dose = 1.
qPCR = quantitative Polymerase Chain Reaction; IP = Artesunate; ND = Not Detected; RBCs: red blood cells.

Listing 16.2.6.3
Malaria 18S qPCR

Randomisation Number	Challenge Agent	Visit	Timepoint	Date of Measurement	Time of Measurement	Day Relative to Inoculum	Day Relative to Artesunate	Assay	Result (parasites/500µL) of packed RBCs
R303	K13	Safety Monitoring		18-SEP-2018	07:29	14	6	Replicate 1	41
								Replicate 2	343
								Replicate 3	442
								Geometric Mean	183.8654
				19-SEP-2018	07:10	15	7	Replicate 1	78
								Replicate 2	55
								Replicate 3	58
								Geometric Mean	62.8968
				20-SEP-2018	08:16	16	8	Replicate 1	332
								Replicate 2	327
								Replicate 3	440
								Geometric Mean	362.8381
				22-SEP-2018	08:40	18	10	Replicate 1	713
								Replicate 2	1008
								Replicate 3	907
								Geometric Mean	867.0666

Notes: Day relative to inoculum is the number of days relative to day of administration of the inoculum, where the day of administration = 0.
The inoculum was administered on Study Day 0 for K13-infected subjects and Study Day 1 for 3D7-infected subjects.
Day relative to IP is the number of days relative to day of administration of Artesunate, where the day of dose = 1.
qPCR = quantitative Polymerase Chain Reaction; IP = Artesunate; ND = Not Detected; RBCs: red blood cells.

Listing 16.2.6.3
Malaria 18S qPCR

Randomisation Number	Challenge Agent	Visit	Timepoint	Date of Measurement	Time of Measurement	Day Relative to Inoculum	Day Relative to Artesunate	Assay	Result (parasites/500µL) of packed RBCs
R303	K13	Safety Monitoring		24-SEP-2018	07:01	20	12	Replicate 1	2013
								Replicate 2	2197
								Replicate 3	2488
								Geometric Mean	2224.2046
				25-SEP-2018	08:20	21	13	Replicate 1	2840
								Replicate 2	2822
								Replicate 3	2548
								Geometric Mean	2733.3270
				27-SEP-2018	07:00	23	15	Replicate 1	258
								Replicate 2	295
								Replicate 3	196
								Geometric Mean	246.1686
				28-SEP-2018	08:19	24	16	Replicate 1	768
								Replicate 2	801
								Replicate 3	906
								Geometric Mean	822.9510

Notes: Day relative to inoculum is the number of days relative to day of administration of the inoculum, where the day of administration = 0.
The inoculum was administered on Study Day 0 for K13-infected subjects and Study Day 1 for 3D7-infected subjects.
Day relative to IP is the number of days relative to day of administration of Artesunate, where the day of dose = 1.
qPCR = quantitative Polymerase Chain Reaction; IP = Artesunate; ND = Not Detected; RBCs: red blood cells.

Listing 16.2.6.3
Malaria 18S qPCR

Randomisation Number	Challenge Agent	Visit	Timepoint	Date of Measurement	Time of Measurement	Day Relative to Inoculum	Day Relative to Artesunate	Assay	Result (parasites/500µL) of packed RBCs
R303	K13	End of Study		02-OCT-2018	09:05	28	20	Replicate 1	111
								Replicate 2	349
								Replicate 3	293
								Geometric Mean	224.7357
		Unscheduled		05-OCT-2018	07:01	31	23	Replicate 1	141
								Replicate 2	108
								Replicate 3	108
								Geometric Mean	118.0381
				11-OCT-2018	06:45	37	29	Replicate 1	ND
								Replicate 2	26
								Replicate 3	ND
								Geometric Mean	2.9625
				18-OCT-2018	11:20	44	36	Replicate 1	ND
								Replicate 2	ND
								Replicate 3	ND
								Geometric Mean	1.0000

Notes: Day relative to inoculum is the number of days relative to day of administration of the inoculum, where the day of administration = 0.
The inoculum was administered on Study Day 0 for K13-infected subjects and Study Day 1 for 3D7-infected subjects.
Day relative to IP is the number of days relative to day of administration of Artesunate, where the day of dose = 1.
qPCR = quantitative Polymerase Chain Reaction; IP = Artesunate; ND = Not Detected; RBCs: red blood cells.

Listing 16.2.6.3
Malaria 18S qPCR

Randomisation Number	Challenge Agent	Visit	Timepoint	Date of Measurement	Time of Measurement	Day Relative to Inoculum	Day Relative to Artesunate	Assay	Result (parasites/500µL) of packed RBCs
R304	K13	Inoculum		04-SEP-2018	10:01	0	-9	Replicate 1	ND
								Replicate 2	ND
								Replicate 3	ND
								Geometric Mean	1.0000
		Malaria Monitoring		08-SEP-2018	08:35	4	-5	Replicate 1	25
								Replicate 2	72
								Replicate 3	81
								Geometric Mean	52.6323
				09-SEP-2018	08:34	5	-4	Replicate 1	68
								Replicate 2	42
								Replicate 3	ND
								Geometric Mean	14.1879
				09-SEP-2018	19:27	5	-4	Replicate 1	636
								Replicate 2	579
								Replicate 3	480
								Geometric Mean	561.2103

Notes: Day relative to inoculum is the number of days relative to day of administration of the inoculum, where the day of administration = 0.
The inoculum was administered on Study Day 0 for K13-infected subjects and Study Day 1 for 3D7-infected subjects.
Day relative to IP is the number of days relative to day of administration of Artesunate, where the day of dose = 1.
qPCR = quantitative Polymerase Chain Reaction; IP = Artesunate; ND = Not Detected; RBCs: red blood cells.

Listing 16.2.6.3
Malaria 18S qPCR

Randomisation Number	Challenge Agent	Visit	Timepoint	Date of Measurement	Time of Measurement	Day Relative to Inoculum	Day Relative to Artesunate	Assay	Result (parasites/500µL) of packed RBCs
R304	K13	Malaria Monitoring		10-SEP-2018	08:21	6	-3	Replicate 1	972
								Replicate 2	370
								Replicate 3	473
								Geometric Mean	554.0850
				10-SEP-2018	19:43	6	-3	Replicate 1	846
								Replicate 2	641
								Replicate 3	663
								Geometric Mean	711.0726
				11-SEP-2018	07:58	7	-2	Replicate 1	1458
								Replicate 2	1006
								Replicate 3	1442
								Geometric Mean	1283.6312
				11-SEP-2018	19:25	7	-2	Replicate 1	9695
								Replicate 2	9551
								Replicate 3	9586
								Geometric Mean	9610.4715

Notes: Day relative to inoculum is the number of days relative to day of administration of the inoculum, where the day of administration = 0.
The inoculum was administered on Study Day 0 for K13-infected subjects and Study Day 1 for 3D7-infected subjects.
Day relative to IP is the number of days relative to day of administration of Artesunate, where the day of dose = 1.
qPCR = quantitative Polymerase Chain Reaction; IP = Artesunate; ND = Not Detected; RBCs: red blood cells.

Listing 16.2.6.3
Malaria 18S qPCR

Randomisation Number	Challenge Agent	Visit	Timepoint	Date of Measurement	Time of Measurement	Day Relative to Inoculum	Day Relative to Artesunate	Assay	Result (parasites/500µL) of packed RBCs
R304	K13	Malaria Monitoring		12-SEP-2018	08:01	8	-1	Replicate 1	14099
								Replicate 2	14401
								Replicate 3	15074
								Geometric Mean	14518.9928
				12-SEP-2018	19:46	8	-1	Replicate 1	15745
								Replicate 2	12393
								Replicate 3	13322
								Geometric Mean	13749.7937
		Admission	Pre-dose	13-SEP-2018	09:16	9	1	Replicate 1	29752
								Replicate 2	30826
								Replicate 3	28042
								Geometric Mean	29517.5818
			4 Hrs Post-dose	13-SEP-2018	13:22	9	1	Replicate 1	46125
								Replicate 2	40617
								Replicate 3	50555
								Geometric Mean	45582.9868

Notes: Day relative to inoculum is the number of days relative to day of administration of the inoculum, where the day of administration = 0.
The inoculum was administered on Study Day 0 for K13-infected subjects and Study Day 1 for 3D7-infected subjects.
Day relative to IP is the number of days relative to day of administration of Artesunate, where the day of dose = 1.
qPCR = quantitative Polymerase Chain Reaction; IP = Artesunate; ND = Not Detected; RBCs: red blood cells.

Listing 16.2.6.3
Malaria 18S qPCR

Randomisation Number	Challenge Agent	Visit	Timepoint	Date of Measurement	Time of Measurement	Day Relative to Inoculum	Day Relative to Artesunate	Assay	Result (parasites/500µL) of packed RBCs
R304	K13	Admission	8 Hrs Post-dose	13-SEP-2018	17:23	9	1	Replicate 1	28137
								Replicate 2	30336
								Replicate 3	30411
								Geometric Mean	29608.8997
			12 Hrs Post-dose	13-SEP-2018	21:23	9	1	Replicate 1	24131
								Replicate 2	21369
								Replicate 3	24131
								Geometric Mean	23172.7875
		Admission 1	16 Hrs Post-dose	14-SEP-2018	01:22	10	2	Replicate 1	17952
								Replicate 2	15223
								Replicate 3	14679
								Geometric Mean	15889.2422
			20 Hrs Post-dose	14-SEP-2018	05:25	10	2	Replicate 1	13067
								Replicate 2	13058
								Replicate 3	12492
								Geometric Mean	12869.4951

Notes: Day relative to inoculum is the number of days relative to day of administration of the inoculum, where the day of administration = 0.
The inoculum was administered on Study Day 0 for K13-infected subjects and Study Day 1 for 3D7-infected subjects.
Day relative to IP is the number of days relative to day of administration of Artesunate, where the day of dose = 1.
qPCR = quantitative Polymerase Chain Reaction; IP = Artesunate; ND = Not Detected; RBCs: red blood cells.

Listing 16.2.6.3
Malaria 18S qPCR

Randomisation Number	Challenge Agent	Visit	Timepoint	Date of Measurement	Time of Measurement	Day Relative to Inoculum	Day Relative to Artesunate	Assay	Result (parasites/500µL) of packed RBCs
R304	K13	Admission 1	24 Hrs Post-dose	14-SEP-2018	09:22	10	2	Replicate 1	7695
								Replicate 2	6878
								Replicate 3	6299
								Geometric Mean	6933.9515
			28 Hrs Post-dose	14-SEP-2018	13:22	10	2	Replicate 1	1843
								Replicate 2	1143
								Replicate 3	1252
								Geometric Mean	1381.6307
			32 Hrs Post-dose	14-SEP-2018	17:23	10	2	Replicate 1	874
								Replicate 2	1173
								Replicate 3	1228
								Geometric Mean	1079.7816
			36 Hrs Post-dose	14-SEP-2018	21:23	10	2	Replicate 1	1524
								Replicate 2	942
								Replicate 3	1099
								Geometric Mean	1164.1560

Notes: Day relative to inoculum is the number of days relative to day of administration of the inoculum, where the day of administration = 0.
The inoculum was administered on Study Day 0 for K13-infected subjects and Study Day 1 for 3D7-infected subjects.
Day relative to IP is the number of days relative to day of administration of Artesunate, where the day of dose = 1.
qPCR = quantitative Polymerase Chain Reaction; IP = Artesunate; ND = Not Detected; RBCs: red blood cells.

Listing 16.2.6.3
Malaria 18S qPCR

Randomisation Number	Challenge Agent	Visit	Timepoint	Date of Measurement	Time of Measurement	Day Relative to Inoculum	Day Relative to Artesunate	Assay	Result (parasites/500µL) of packed RBCs
R304	K13	Admission 2		15-SEP-2018	01:23	11	3	Replicate 1	2687
								Replicate 2	2235
								Replicate 3	1945
								Geometric Mean	2268.9326
				15-SEP-2018	05:23	11	3	Replicate 1	8433
								Replicate 2	9478
								Replicate 3	9599
								Geometric Mean	9154.6469
			48 Hrs Post-dose	15-SEP-2018	09:22	11	3	Replicate 1	51396
								Replicate 2	49225
								Replicate 3	43229
								Geometric Mean	47822.2580
			52 Hrs Post-dose	15-SEP-2018	13:24	11	3	Replicate 1	99125
								Replicate 2	94660
								Replicate 3	97086
								Geometric Mean	96939.7963

Notes: Day relative to inoculum is the number of days relative to day of administration of the inoculum, where the day of administration = 0.
The inoculum was administered on Study Day 0 for K13-infected subjects and Study Day 1 for 3D7-infected subjects.
Day relative to IP is the number of days relative to day of administration of Artesunate, where the day of dose = 1.
qPCR = quantitative Polymerase Chain Reaction; IP = Artesunate; ND = Not Detected; RBCs: red blood cells.

Listing 16.2.6.3
Malaria 18S qPCR

Randomisation Number	Challenge Agent	Visit	Timepoint	Date of Measurement	Time of Measurement	Day Relative to Inoculum	Day Relative to Artesunate	Assay	Result (parasites/500µL) of packed RBCs
R304	K13	Admission 2	56 Hrs Post-dose	15-SEP-2018	17:24	11	3	Replicate 1	88818
								Replicate 2	91094
								Replicate 3	92463
								Geometric Mean	90779.1783
			60 Hrs Post-dose	15-SEP-2018	21:26	11	3	Replicate 1	66999
								Replicate 2	59386
								Replicate 3	71325
								Geometric Mean	65715.0322
		Admission 3		16-SEP-2018	01:24	12	4	Replicate 1	44260
								Replicate 2	45672
								Replicate 3	47525
								Geometric Mean	45799.5476
				16-SEP-2018	05:23	12	4	Replicate 1	29573
								Replicate 2	28717
								Replicate 3	31228
								Geometric Mean	29821.2750

Notes: Day relative to inoculum is the number of days relative to day of administration of the inoculum, where the day of administration = 0.
The inoculum was administered on Study Day 0 for K13-infected subjects and Study Day 1 for 3D7-infected subjects.
Day relative to IP is the number of days relative to day of administration of Artesunate, where the day of dose = 1.
qPCR = quantitative Polymerase Chain Reaction; IP = Artesunate; ND = Not Detected; RBCs: red blood cells.

Listing 16.2.6.3
Malaria 18S qPCR

Randomisation Number	Challenge Agent	Visit	Timepoint	Date of Measurement	Time of Measurement	Day Relative to Inoculum	Day Relative to Artesunate	Assay	Result (parasites/500µL) of packed RBCs
R304	K13	Admission 3	72 Hrs Post-dose	16-SEP-2018	09:22	12	4	Replicate 1	23905
								Replicate 2	22364
								Replicate 3	23948
								Geometric Mean	23393.8927
				16-SEP-2018	20:18	12	4	Replicate 1	1360
								Replicate 2	774
								Replicate 3	948
								Geometric Mean	999.3004
		Safety Monitoring		17-SEP-2018	08:15	13	5	Replicate 1	364
								Replicate 2	241
								Replicate 3	510
								Geometric Mean	355.0010
				18-SEP-2018	08:36	14	6	Replicate 1	86
								Replicate 2	44
								Replicate 3	82
								Geometric Mean	67.6999

Notes: Day relative to inoculum is the number of days relative to day of administration of the inoculum, where the day of administration = 0.
The inoculum was administered on Study Day 0 for K13-infected subjects and Study Day 1 for 3D7-infected subjects.
Day relative to IP is the number of days relative to day of administration of Artesunate, where the day of dose = 1.
qPCR = quantitative Polymerase Chain Reaction; IP = Artesunate; ND = Not Detected; RBCs: red blood cells.

Listing 16.2.6.3
Malaria 18S qPCR

Randomisation Number	Challenge Agent	Visit	Timepoint	Date of Measurement	Time of Measurement	Day Relative to Inoculum	Day Relative to Artesunate	Assay	Result (parasites/500µL) of packed RBCs
R304	K13	Safety Monitoring		19-SEP-2018	08:24	15	7	Replicate 1	ND
								Replicate 2	ND
								Replicate 3	43
								Geometric Mean	3.5034
				20-SEP-2018	08:09	16	8	Replicate 1	186
								Replicate 2	214
								Replicate 3	142
								Geometric Mean	178.1305
				22-SEP-2018	09:25	18	10	Replicate 1	407
								Replicate 2	297
								Replicate 3	654
								Geometric Mean	429.1834
				24-SEP-2018	07:58	20	12	Replicate 1	925
								Replicate 2	942
								Replicate 3	901
								Geometric Mean	922.5130

Notes: Day relative to inoculum is the number of days relative to day of administration of the inoculum, where the day of administration = 0.
The inoculum was administered on Study Day 0 for K13-infected subjects and Study Day 1 for 3D7-infected subjects.
Day relative to IP is the number of days relative to day of administration of Artesunate, where the day of dose = 1.
qPCR = quantitative Polymerase Chain Reaction; IP = Artesunate; ND = Not Detected; RBCs: red blood cells.

Listing 16.2.6.3
Malaria 18S qPCR

Randomisation Number	Challenge Agent	Visit	Timepoint	Date of Measurement	Time of Measurement	Day Relative to Inoculum	Day Relative to Artesunate	Assay	Result (parasites/500µL) of packed RBCs
R304	K13	Safety Monitoring		25-SEP-2018	08:11	21	13	Replicate 1	495
								Replicate 2	396
								Replicate 3	417
								Geometric Mean	433.9891
				27-SEP-2018	08:20	23	15	Replicate 1	290
								Replicate 2	293
								Replicate 3	535
								Geometric Mean	356.8945
				28-SEP-2018	08:17	24	16	Replicate 1	219
								Replicate 2	238
								Replicate 3	254
								Geometric Mean	236.5656
		End of Study		02-OCT-2018	08:46	28	20	Replicate 1	99
								Replicate 2	85
								Replicate 3	99
								Geometric Mean	94.0943

Notes: Day relative to inoculum is the number of days relative to day of administration of the inoculum, where the day of administration = 0.
The inoculum was administered on Study Day 0 for K13-infected subjects and Study Day 1 for 3D7-infected subjects.
Day relative to IP is the number of days relative to day of administration of Artesunate, where the day of dose = 1.
qPCR = quantitative Polymerase Chain Reaction; IP = Artesunate; ND = Not Detected; RBCs: red blood cells.

Listing 16.2.6.3
Malaria 18S qPCR

Randomisation Number	Challenge Agent	Visit	Timepoint	Date of Measurement	Time of Measurement	Day Relative to Inoculum	Day Relative to Artesunate	Assay	Result (parasites/500µL) of packed RBCs
R304	K13	Unscheduled		05-OCT-2018	08:20	31	23	Replicate 1	ND
								Replicate 2	69
								Replicate 3	38
								Geometric Mean	13.7894
				11-OCT-2018	09:27	37	29	Replicate 1	ND
								Replicate 2	ND
								Replicate 3	ND
								Geometric Mean	1.0000
R305	3D7	Inoculum		05-SEP-2018	10:27	0	-8	Replicate 1	ND
								Replicate 2	ND
								Replicate 3	ND
								Geometric Mean	1.0000
		Malaria Monitoring		09-SEP-2018	07:50	4	-4	Replicate 1	36
								Replicate 2	37
								Replicate 3	143
								Geometric Mean	57.5369

Notes: Day relative to inoculum is the number of days relative to day of administration of the inoculum, where the day of administration = 0.
The inoculum was administered on Study Day 0 for K13-infected subjects and Study Day 1 for 3D7-infected subjects.
Day relative to IP is the number of days relative to day of administration of Artesunate, where the day of dose = 1.
qPCR = quantitative Polymerase Chain Reaction; IP = Artesunate; ND = Not Detected; RBCs: red blood cells.

Listing 16.2.6.3
Malaria 18S qPCR

Randomisation Number	Challenge Agent	Visit	Timepoint	Date of Measurement	Time of Measurement	Day Relative to Inoculum	Day Relative to Artesunate	Assay	Result (parasites/500µL) of packed RBCs
R305	3D7	Malaria Monitoring		10-SEP-2018	07:30	5	-3	Replicate 1	1242
								Replicate 2	1138
								Replicate 3	1026
								Geometric Mean	1131.8887
				10-SEP-2018	19:18	5	-3	Replicate 1	3685
								Replicate 2	2964
								Replicate 3	3602
								Geometric Mean	3401.1031
				11-SEP-2018	07:08	6	-2	Replicate 1	1091
								Replicate 2	1435
								Replicate 3	1111
								Geometric Mean	1202.6250
				11-SEP-2018	18:53	6	-2	Replicate 1	3958
								Replicate 2	4180
								Replicate 3	4170
								Geometric Mean	4101.3740

Notes: Day relative to inoculum is the number of days relative to day of administration of the inoculum, where the day of administration = 0.
The inoculum was administered on Study Day 0 for K13-infected subjects and Study Day 1 for 3D7-infected subjects.
Day relative to IP is the number of days relative to day of administration of Artesunate, where the day of dose = 1.
qPCR = quantitative Polymerase Chain Reaction; IP = Artesunate; ND = Not Detected; RBCs: red blood cells.

Listing 16.2.6.3
Malaria 18S qPCR

Randomisation Number	Challenge Agent	Visit	Timepoint	Date of Measurement	Time of Measurement	Day Relative to Inoculum	Day Relative to Artesunate	Assay	Result (parasites/500µL) of packed RBCs
R305	3D7	Malaria Monitoring		12-SEP-2018	06:50	7	-1	Replicate 1	53251
								Replicate 2	55675
								Replicate 3	50236
								Geometric Mean	53007.1371
				12-SEP-2018	19:04	7	-1	Replicate 1	38691
								Replicate 2	42331
								Replicate 3	39700
								Geometric Mean	40211.7521
		Admission	Pre-dose	13-SEP-2018	09:29	8	1	Replicate 1	62755
								Replicate 2	63254
								Replicate 3	65434
								Geometric Mean	63803.8080
			4 Hrs Post-dose	13-SEP-2018	13:35	8	1	Replicate 1	129384
								Replicate 2	133662
								Replicate 3	127067
								Geometric Mean	130009.1050

Notes: Day relative to inoculum is the number of days relative to day of administration of the inoculum, where the day of administration = 0.
The inoculum was administered on Study Day 0 for K13-infected subjects and Study Day 1 for 3D7-infected subjects.
Day relative to IP is the number of days relative to day of administration of Artesunate, where the day of dose = 1.
qPCR = quantitative Polymerase Chain Reaction; IP = Artesunate; ND = Not Detected; RBCs: red blood cells.

Listing 16.2.6.3
Malaria 18S qPCR

Randomisation Number	Challenge Agent	Visit	Timepoint	Date of Measurement	Time of Measurement	Day Relative to Inoculum	Day Relative to Artesunate	Assay	Result (parasites/500μL) of packed RBCs
R305	3D7	Admission	8 Hrs Post-dose	13-SEP-2018	17:35	8	1	Replicate 1	97599
								Replicate 2	115200
								Replicate 3	105224
								Geometric Mean	105763.9230
			12 Hrs Post-dose	13-SEP-2018	21:37	8	1	Replicate 1	59646
								Replicate 2	51991
								Replicate 3	50327
								Geometric Mean	53839.8093
		Admission 1	16 Hrs Post-dose	14-SEP-2018	01:42	9	2	Replicate 1	20819
								Replicate 2	20796
								Replicate 3	21698
								Geometric Mean	21100.1946
			20 Hrs Post-dose	14-SEP-2018	05:38	9	2	Replicate 1	7243
								Replicate 2	7697
								Replicate 3	7225
								Geometric Mean	7385.1492

Notes: Day relative to inoculum is the number of days relative to day of administration of the inoculum, where the day of administration = 0.
The inoculum was administered on Study Day 0 for K13-infected subjects and Study Day 1 for 3D7-infected subjects.
Day relative to IP is the number of days relative to day of administration of Artesunate, where the day of dose = 1.
qPCR = quantitative Polymerase Chain Reaction; IP = Artesunate; ND = Not Detected; RBCs: red blood cells.

Listing 16.2.6.3
Malaria 18S qPCR

Randomisation Number	Challenge Agent	Visit	Timepoint	Date of Measurement	Time of Measurement	Day Relative to Inoculum	Day Relative to Artesunate	Assay	Result (parasites/500µL) of packed RBCs
R305	3D7	Admission 1	24 Hrs Post-dose	14-SEP-2018	09:37	9	2	Replicate 1	3293
								Replicate 2	2708
								Replicate 3	3166
								Geometric Mean	3044.9753
			28 Hrs Post-dose	14-SEP-2018	13:37	9	2	Replicate 1	931
								Replicate 2	731
								Replicate 3	637
								Geometric Mean	756.8367
			32 Hrs Post-dose	14-SEP-2018	17:37	9	2	Replicate 1	131
								Replicate 2	164
								Replicate 3	322
								Geometric Mean	190.5418
			36 Hrs Post-dose	14-SEP-2018	21:36	9	2	Replicate 1	656
								Replicate 2	384
								Replicate 3	75
								Geometric Mean	266.3374

Notes: Day relative to inoculum is the number of days relative to day of administration of the inoculum, where the day of administration = 0.
The inoculum was administered on Study Day 0 for K13-infected subjects and Study Day 1 for 3D7-infected subjects.
Day relative to IP is the number of days relative to day of administration of Artesunate, where the day of dose = 1.
qPCR = quantitative Polymerase Chain Reaction; IP = Artesunate; ND = Not Detected; RBCs: red blood cells.

Listing 16.2.6.3
Malaria 18S qPCR

Randomisation Number	Challenge Agent	Visit	Timepoint	Date of Measurement	Time of Measurement	Day Relative to Inoculum	Day Relative to Artesunate	Assay	Result (parasites/500µL) of packed RBCs
R305	3D7	Admission 2		15-SEP-2018	01:35	10	3	Replicate 1	153
								Replicate 2	150
								Replicate 3	93
								Geometric Mean	128.7524
				15-SEP-2018	05:35	10	3	Replicate 1	77
								Replicate 2	179
								Replicate 3	440
								Geometric Mean	182.3611
			48 Hrs Post-dose	15-SEP-2018	09:35	10	3	Replicate 1	368
								Replicate 2	192
								Replicate 3	217
								Geometric Mean	248.4294
			52 Hrs Post-dose	15-SEP-2018	13:36	10	3	Replicate 1	173
								Replicate 2	199
								Replicate 3	315
								Geometric Mean	221.3451

Notes: Day relative to inoculum is the number of days relative to day of administration of the inoculum, where the day of administration = 0.
The inoculum was administered on Study Day 0 for K13-infected subjects and Study Day 1 for 3D7-infected subjects.
Day relative to IP is the number of days relative to day of administration of Artesunate, where the day of dose = 1.
qPCR = quantitative Polymerase Chain Reaction; IP = Artesunate; ND = Not Detected; RBCs: red blood cells.

Listing 16.2.6.3
Malaria 18S qPCR

Randomisation Number	Challenge Agent	Visit	Timepoint	Date of Measurement	Time of Measurement	Day Relative to Inoculum	Day Relative to Artesunate	Assay	Result (parasites/500μL) of packed RBCs
R305	3D7	Admission 2	56 Hrs Post-dose	15-SEP-2018	17:37	10	3	Replicate 1	461
								Replicate 2	232
								Replicate 3	304
								Geometric Mean	319.1691
			60 Hrs Post-dose	15-SEP-2018	21:35	10	3	Replicate 1	379
								Replicate 2	367
								Replicate 3	171
								Geometric Mean	287.5856
		Admission 3		16-SEP-2018	01:36	11	4	Replicate 1	659
								Replicate 2	187
								Replicate 3	395
								Geometric Mean	365.1248
				16-SEP-2018	05:36	11	4	Replicate 1	254
								Replicate 2	433
								Replicate 3	181
								Geometric Mean	271.0192

Notes: Day relative to inoculum is the number of days relative to day of administration of the inoculum, where the day of administration = 0.
The inoculum was administered on Study Day 0 for K13-infected subjects and Study Day 1 for 3D7-infected subjects.
Day relative to IP is the number of days relative to day of administration of Artesunate, where the day of dose = 1.
qPCR = quantitative Polymerase Chain Reaction; IP = Artesunate; ND = Not Detected; RBCs: red blood cells.

Listing 16.2.6.3
Malaria 18S qPCR

Randomisation Number	Challenge Agent	Visit	Timepoint	Date of Measurement	Time of Measurement	Day Relative to Inoculum	Day Relative to Artesunate	Assay	Result (parasites/500µL) of packed RBCs
R305	3D7	Admission 3	72 Hrs Post-dose	16-SEP-2018	09:36	11	4	Replicate 1	193
								Replicate 2	220
								Replicate 3	199
								Geometric Mean	203.6781
				16-SEP-2018	19:04	11	4	Replicate 1	225
								Replicate 2	78
								Replicate 3	142
								Geometric Mean	135.5778
		Safety Monitoring		17-SEP-2018	06:56	12	5	Replicate 1	1439
								Replicate 2	1573
								Replicate 3	1264
								Geometric Mean	1419.6421
				17-SEP-2018	19:05	12	5	Replicate 1	4310
								Replicate 2	3992
								Replicate 3	3549
								Geometric Mean	3937.8388

Notes: Day relative to inoculum is the number of days relative to day of administration of the inoculum, where the day of administration = 0.
The inoculum was administered on Study Day 0 for K13-infected subjects and Study Day 1 for 3D7-infected subjects.
Day relative to IP is the number of days relative to day of administration of Artesunate, where the day of dose = 1.
qPCR = quantitative Polymerase Chain Reaction; IP = Artesunate; ND = Not Detected; RBCs: red blood cells.

Listing 16.2.6.3
Malaria 18S qPCR

Randomisation Number	Challenge Agent	Visit	Timepoint	Date of Measurement	Time of Measurement	Day Relative to Inoculum	Day Relative to Artesunate	Assay	Result (parasites/500µL) of packed RBCs
R305	3D7	Safety Monitoring		18-SEP-2018	07:16	13	6	Replicate 1	2384
								Replicate 2	2834
								Replicate 3	3718
								Geometric Mean	2928.6794
				18-SEP-2018	19:05	13	6	Replicate 1	31864
								Replicate 2	31606
								Replicate 3	31125
								Geometric Mean	31530.1761
				19-SEP-2018	07:24	14	7	Replicate 1	88698
								Replicate 2	88059
								Replicate 3	92087
								Geometric Mean	89597.3780
				19-SEP-2018	19:23	14	7	Replicate 1	64528
								Replicate 2	62534
								Replicate 3	63316
								Geometric Mean	63454.0425

Notes: Day relative to inoculum is the number of days relative to day of administration of the inoculum, where the day of administration = 0.
The inoculum was administered on Study Day 0 for K13-infected subjects and Study Day 1 for 3D7-infected subjects.
Day relative to IP is the number of days relative to day of administration of Artesunate, where the day of dose = 1.
qPCR = quantitative Polymerase Chain Reaction; IP = Artesunate; ND = Not Detected; RBCs: red blood cells.

Listing 16.2.6.3
Malaria 18S qPCR

Randomisation Number	Challenge Agent	Visit	Timepoint	Date of Measurement	Time of Measurement	Day Relative to Inoculum	Day Relative to Artesunate	Assay	Result (parasites/500µL) of packed RBCs
R305	3D7	Safety Monitoring		20-SEP-2018	07:23	15	8	Replicate 1	40209
								Replicate 2	39400
								Replicate 3	41896
								Geometric Mean	40488.4065
				21-SEP-2018	07:03	16	9	Replicate 1	639
								Replicate 2	775
								Replicate 3	496
								Geometric Mean	626.2697
				22-SEP-2018	07:53	17	10	Replicate 1	68
								Replicate 2	ND
								Replicate 3	78
								Geometric Mean	17.4395
				24-SEP-2018	07:04	19	12	Replicate 1	153
								Replicate 2	117
								Replicate 3	75
								Geometric Mean	110.3180

Notes: Day relative to inoculum is the number of days relative to day of administration of the inoculum, where the day of administration = 0.
The inoculum was administered on Study Day 0 for K13-infected subjects and Study Day 1 for 3D7-infected subjects.
Day relative to IP is the number of days relative to day of administration of Artesunate, where the day of dose = 1.
qPCR = quantitative Polymerase Chain Reaction; IP = Artesunate; ND = Not Detected; RBCs: red blood cells.

Listing 16.2.6.3
Malaria 18S qPCR

Randomisation Number	Challenge Agent	Visit	Timepoint	Date of Measurement	Time of Measurement	Day Relative to Inoculum	Day Relative to Artesunate	Assay	Result (parasites/500µL) of packed RBCs
R305	3D7	Safety Monitoring		25-SEP-2018	08:19	20	13	Replicate 1	243
								Replicate 2	111
								Replicate 3	172
								Geometric Mean	166.7833
				28-SEP-2018	07:23	23	16	Replicate 1	418
								Replicate 2	306
								Replicate 3	377
								Geometric Mean	363.9818
		End of Study		02-OCT-2018	07:50	27	20	Replicate 1	1159
								Replicate 2	784
								Replicate 3	637
								Geometric Mean	833.3862
		Unscheduled		04-OCT-2018	09:35	29	22	Replicate 1	595
								Replicate 2	634
								Replicate 3	515
								Geometric Mean	579.1679

Notes: Day relative to inoculum is the number of days relative to day of administration of the inoculum, where the day of administration = 0.
The inoculum was administered on Study Day 0 for K13-infected subjects and Study Day 1 for 3D7-infected subjects.
Day relative to IP is the number of days relative to day of administration of Artesunate, where the day of dose = 1.
qPCR = quantitative Polymerase Chain Reaction; IP = Artesunate; ND = Not Detected; RBCs: red blood cells.

Listing 16.2.6.3
Malaria 18S qPCR

Randomisation Number	Challenge Agent	Visit	Timepoint	Date of Measurement	Time of Measurement	Day Relative to Inoculum	Day Relative to Artesunate	Assay	Result (parasites/500µL) of packed RBCs
R305	3D7	Unscheduled		10-OCT-2018	08:12	35	28	Replicate 1	104
								Replicate 2	90
								Replicate 3	166
								Geometric Mean	115.8230
				17-OCT-2018	09:41	42	35	Replicate 1	ND
								Replicate 2	ND
								Replicate 3	ND
								Geometric Mean	1.0000
R306	3D7	Inoculum		05-SEP-2018	10:46	0	-8	Replicate 1	ND
								Replicate 2	ND
								Replicate 3	ND
								Geometric Mean	1.0000
		Malaria Monitoring		09-SEP-2018	07:21	4	-4	Replicate 1	39
								Replicate 2	ND
								Replicate 3	ND
								Geometric Mean	3.3912

Notes: Day relative to inoculum is the number of days relative to day of administration of the inoculum, where the day of administration = 0.
The inoculum was administered on Study Day 0 for K13-infected subjects and Study Day 1 for 3D7-infected subjects.
Day relative to IP is the number of days relative to day of administration of Artesunate, where the day of dose = 1.
qPCR = quantitative Polymerase Chain Reaction; IP = Artesunate; ND = Not Detected; RBCs: red blood cells.

Listing 16.2.6.3
Malaria 18S qPCR

Randomisation Number	Challenge Agent	Visit	Timepoint	Date of Measurement	Time of Measurement	Day Relative to Inoculum	Day Relative to Artesunate	Assay	Result (parasites/500µL) of packed RBCs
R306	3D7	Malaria Monitoring		10-SEP-2018	07:03	5	-3	Replicate 1	663
								Replicate 2	860
								Replicate 3	525
								Geometric Mean	668.9450
				10-SEP-2018	19:08	5	-3	Replicate 1	1249
								Replicate 2	1527
								Replicate 3	746
								Geometric Mean	1124.7261
				11-SEP-2018	07:07	6	-2	Replicate 1	265
								Replicate 2	320
								Replicate 3	217
								Geometric Mean	264.0089
				11-SEP-2018	19:08	6	-2	Replicate 1	3242
								Replicate 2	2290
								Replicate 3	2776
								Geometric Mean	2741.7172

Notes: Day relative to inoculum is the number of days relative to day of administration of the inoculum, where the day of administration = 0.
The inoculum was administered on Study Day 0 for K13-infected subjects and Study Day 1 for 3D7-infected subjects.
Day relative to IP is the number of days relative to day of administration of Artesunate, where the day of dose = 1.
qPCR = quantitative Polymerase Chain Reaction; IP = Artesunate; ND = Not Detected; RBCs: red blood cells.

Listing 16.2.6.3
Malaria 18S qPCR

Randomisation Number	Challenge Agent	Visit	Timepoint	Date of Measurement	Time of Measurement	Day Relative to Inoculum	Day Relative to Artesunate	Assay	Result (parasites/500µL) of packed RBCs
R306	3D7	Malaria Monitoring		12-SEP-2018	06:59	7	-1	Replicate 1	15074
								Replicate 2	15863
								Replicate 3	17660
								Geometric Mean	16163.4791
				12-SEP-2018	19:09	7	-1	Replicate 1	7171
								Replicate 2	5883
								Replicate 3	6418
								Geometric Mean	6469.3318
		Admission	Pre-dose	13-SEP-2018	09:41	8	1	Replicate 1	23443
								Replicate 2	21858
								Replicate 3	22463
								Geometric Mean	22578.6058
			4 Hrs Post-dose	13-SEP-2018	13:45	8	1	Replicate 1	43103
								Replicate 2	37233
								Replicate 3	46292
								Geometric Mean	42038.4948

Notes: Day relative to inoculum is the number of days relative to day of administration of the inoculum, where the day of administration = 0.
The inoculum was administered on Study Day 0 for K13-infected subjects and Study Day 1 for 3D7-infected subjects.
Day relative to IP is the number of days relative to day of administration of Artesunate, where the day of dose = 1.
qPCR = quantitative Polymerase Chain Reaction; IP = Artesunate; ND = Not Detected; RBCs: red blood cells.

Listing 16.2.6.3
Malaria 18S qPCR

Randomisation Number	Challenge Agent	Visit	Timepoint	Date of Measurement	Time of Measurement	Day Relative to Inoculum	Day Relative to Artesunate	Assay	Result (parasites/500µL) of packed RBCs
R306	3D7	Admission	8 Hrs Post-dose	13-SEP-2018	17:45	8	1	Replicate 1	32839
								Replicate 2	30425
								Replicate 3	38674
								Geometric Mean	33807.5113
			12 Hrs Post-dose	13-SEP-2018	21:45	8	1	Replicate 1	11623
								Replicate 2	9273
								Replicate 3	10876
								Geometric Mean	10543.9313
		Admission 1	16 Hrs Post-dose	14-SEP-2018	01:46	9	2	Replicate 1	3003
								Replicate 2	2820
								Replicate 3	2602
								Geometric Mean	2803.5216
			20 Hrs Post-dose	14-SEP-2018	05:45	9	2	Replicate 1	745
								Replicate 2	1019
								Replicate 3	1092
								Geometric Mean	939.4010

Notes: Day relative to inoculum is the number of days relative to day of administration of the inoculum, where the day of administration = 0.
The inoculum was administered on Study Day 0 for K13-infected subjects and Study Day 1 for 3D7-infected subjects.
Day relative to IP is the number of days relative to day of administration of Artesunate, where the day of dose = 1.
qPCR = quantitative Polymerase Chain Reaction; IP = Artesunate; ND = Not Detected; RBCs: red blood cells.

Listing 16.2.6.3
Malaria 18S qPCR

Randomisation Number	Challenge Agent	Visit	Timepoint	Date of Measurement	Time of Measurement	Day Relative to Inoculum	Day Relative to Artesunate	Assay	Result (parasites/500µL) of packed RBCs
R306	3D7	Admission 1	24 Hrs Post-dose	14-SEP-2018	09:46	9	2	Replicate 1	368
								Replicate 2	419
								Replicate 3	381
								Geometric Mean	388.7427
			28 Hrs Post-dose	14-SEP-2018	13:46	9	2	Replicate 1	179
								Replicate 2	83
								Replicate 3	92
								Geometric Mean	110.9787
			32 Hrs Post-dose	14-SEP-2018	17:46	9	2	Replicate 1	176
								Replicate 2	153
								Replicate 3	81
								Geometric Mean	129.6870
			36 Hrs Post-dose	14-SEP-2018	21:45	9	2	Replicate 1	101
								Replicate 2	ND
								Replicate 3	107
								Geometric Mean	22.1090

Notes: Day relative to inoculum is the number of days relative to day of administration of the inoculum, where the day of administration = 0.
The inoculum was administered on Study Day 0 for K13-infected subjects and Study Day 1 for 3D7-infected subjects.
Day relative to IP is the number of days relative to day of administration of Artesunate, where the day of dose = 1.
qPCR = quantitative Polymerase Chain Reaction; IP = Artesunate; ND = Not Detected; RBCs: red blood cells.

Listing 16.2.6.3
Malaria 18S qPCR

Randomisation Number	Challenge Agent	Visit	Timepoint	Date of Measurement	Time of Measurement	Day Relative to Inoculum	Day Relative to Artesunate	Assay	Result (parasites/500µL) of packed RBCs
R306	3D7	Admission 2		15-SEP-2018	01:45	10	3	Replicate 1	ND
								Replicate 2	60
								Replicate 3	91
								Geometric Mean	17.6088
				15-SEP-2018	05:45	10	3	Replicate 1	45
								Replicate 2	91
								Replicate 3	80
								Geometric Mean	68.9363
			48 Hrs Post-dose	15-SEP-2018	09:45	10	3	Replicate 1	109
								Replicate 2	167
								Replicate 3	48
								Geometric Mean	95.6008
			52 Hrs Post-dose	15-SEP-2018	13:46	10	3	Replicate 1	62
								Replicate 2	141
								Replicate 3	57
								Geometric Mean	79.2797

Notes: Day relative to inoculum is the number of days relative to day of administration of the inoculum, where the day of administration = 0.
The inoculum was administered on Study Day 0 for K13-infected subjects and Study Day 1 for 3D7-infected subjects.
Day relative to IP is the number of days relative to day of administration of Artesunate, where the day of dose = 1.
qPCR = quantitative Polymerase Chain Reaction; IP = Artesunate; ND = Not Detected; RBCs: red blood cells.

Listing 16.2.6.3
Malaria 18S qPCR

Randomisation Number	Challenge Agent	Visit	Timepoint	Date of Measurement	Time of Measurement	Day Relative to Inoculum	Day Relative to Artesunate	Assay	Result (parasites/500µL) of packed RBCs
R306	3D7	Admission 2	56 Hrs Post-dose	15-SEP-2018	17:45	10	3	Replicate 1	113
								Replicate 2	143
								Replicate 3	138
								Geometric Mean	130.6465
			60 Hrs Post-dose	15-SEP-2018	21:45	10	3	Replicate 1	52
								Replicate 2	168
								Replicate 3	85
								Geometric Mean	90.5546
		Admission 3		16-SEP-2018	01:46	11	4	Replicate 1	178
								Replicate 2	204
								Replicate 3	90
								Geometric Mean	148.3990
				16-SEP-2018	05:45	11	4	Replicate 1	272
								Replicate 2	167
								Replicate 3	208
								Geometric Mean	211.4055

Notes: Day relative to inoculum is the number of days relative to day of administration of the inoculum, where the day of administration = 0.
The inoculum was administered on Study Day 0 for K13-infected subjects and Study Day 1 for 3D7-infected subjects.
Day relative to IP is the number of days relative to day of administration of Artesunate, where the day of dose = 1.
qPCR = quantitative Polymerase Chain Reaction; IP = Artesunate; ND = Not Detected; RBCs: red blood cells.

Listing 16.2.6.3
Malaria 18S qPCR

Randomisation Number	Challenge Agent	Visit	Timepoint	Date of Measurement	Time of Measurement	Day Relative to Inoculum	Day Relative to Artesunate	Assay	Result (parasites/500µL) of packed RBCs
R306	3D7	Admission 3	72 Hrs Post-dose	16-SEP-2018	09:45	11	4	Replicate 1	36
								Replicate 2	101
								Replicate 3	30
								Geometric Mean	47.7802
				16-SEP-2018	19:14	11	4	Replicate 1	ND
								Replicate 2	91
								Replicate 3	ND
								Geometric Mean	4.4979
		Safety Monitoring		17-SEP-2018	06:57	12	5	Replicate 1	530
								Replicate 2	748
								Replicate 3	623
								Geometric Mean	627.4154
				17-SEP-2018	19:10	12	5	Replicate 1	1204
								Replicate 2	1360
								Replicate 3	1093
								Geometric Mean	1214.1207

Notes: Day relative to inoculum is the number of days relative to day of administration of the inoculum, where the day of administration = 0.
The inoculum was administered on Study Day 0 for K13-infected subjects and Study Day 1 for 3D7-infected subjects.
Day relative to IP is the number of days relative to day of administration of Artesunate, where the day of dose = 1.
qPCR = quantitative Polymerase Chain Reaction; IP = Artesunate; ND = Not Detected; RBCs: red blood cells.

Listing 16.2.6.3
Malaria 18S qPCR

Randomisation Number	Challenge Agent	Visit	Timepoint	Date of Measurement	Time of Measurement	Day Relative to Inoculum	Day Relative to Artesunate	Assay	Result (parasites/500µL) of packed RBCs
R306	3D7	Safety Monitoring		18-SEP-2018	08:04	13	6	Replicate 1	384
								Replicate 2	371
								Replicate 3	576
								Geometric Mean	434.5528
				18-SEP-2018	19:29	13	6	Replicate 1	8643
								Replicate 2	8957
								Replicate 3	7449
								Geometric Mean	8323.5454
				19-SEP-2018	07:00	14	7	Replicate 1	18782
								Replicate 2	15438
								Replicate 3	17121
								Geometric Mean	17059.0169
				19-SEP-2018	20:08	14	7	Replicate 1	8116
								Replicate 2	9541
								Replicate 3	8468
								Geometric Mean	8687.7156

Notes: Day relative to inoculum is the number of days relative to day of administration of the inoculum, where the day of administration = 0.
The inoculum was administered on Study Day 0 for K13-infected subjects and Study Day 1 for 3D7-infected subjects.
Day relative to IP is the number of days relative to day of administration of Artesunate, where the day of dose = 1.
qPCR = quantitative Polymerase Chain Reaction; IP = Artesunate; ND = Not Detected; RBCs: red blood cells.

Listing 16.2.6.3
Malaria 18S qPCR

Randomisation Number	Challenge Agent	Visit	Timepoint	Date of Measurement	Time of Measurement	Day Relative to Inoculum	Day Relative to Artesunate	Assay	Result (parasites/500µL) of packed RBCs
R306	3D7	Safety Monitoring		20-SEP-2018	07:01	15	8	Replicate 1	5553
								Replicate 2	5496
								Replicate 3	4073
								Geometric Mean	4990.7169
				21-SEP-2018	07:32	16	9	Replicate 1	55
								Replicate 2	181
								Replicate 3	189
								Geometric Mean	123.4528
				22-SEP-2018	07:34	17	10	Replicate 1	ND
								Replicate 2	ND
								Replicate 3	ND
								Geometric Mean	1.0000
				24-SEP-2018	07:01	19	12	Replicate 1	ND
								Replicate 2	27
								Replicate 3	ND
								Geometric Mean	3.0000

Notes: Day relative to inoculum is the number of days relative to day of administration of the inoculum, where the day of administration = 0.
The inoculum was administered on Study Day 0 for K13-infected subjects and Study Day 1 for 3D7-infected subjects.
Day relative to IP is the number of days relative to day of administration of Artesunate, where the day of dose = 1.
qPCR = quantitative Polymerase Chain Reaction; IP = Artesunate; ND = Not Detected; RBCs: red blood cells.

Listing 16.2.6.3
Malaria 18S qPCR

Randomisation Number	Challenge Agent	Visit	Timepoint	Date of Measurement	Time of Measurement	Day Relative to Inoculum	Day Relative to Artesunate	Assay	Result (parasites/500µL) of packed RBCs
R306	3D7	Safety Monitoring		25-SEP-2018	07:30	20	13	Replicate 1	25
								Replicate 2	22
								Replicate 3	150
								Geometric Mean	43.5329
				28-SEP-2018	07:11	23	16	Replicate 1	49
								Replicate 2	45
								Replicate 3	116
								Geometric Mean	63.4778
		End of Study		02-OCT-2018	08:20	27	20	Replicate 1	83
								Replicate 2	63
								Replicate 3	77
								Geometric Mean	73.8419
		Unscheduled		08-OCT-2018	07:07	33	26	Replicate 1	ND
								Replicate 2	ND
								Replicate 3	ND
								Geometric Mean	1.0000

Notes: Day relative to inoculum is the number of days relative to day of administration of the inoculum, where the day of administration = 0.
The inoculum was administered on Study Day 0 for K13-infected subjects and Study Day 1 for 3D7-infected subjects.
Day relative to IP is the number of days relative to day of administration of Artesunate, where the day of dose = 1.
qPCR = quantitative Polymerase Chain Reaction; IP = Artesunate; ND = Not Detected; RBCs: red blood cells.

Listing 16.2.6.3
Malaria 18S qPCR

Randomisation Number	Challenge Agent	Visit	Timepoint	Date of Measurement	Time of Measurement	Day Relative to Inoculum	Day Relative to Artesunate	Assay	Result (parasites/500µL) of packed RBCs
R307	3D7	Inoculum		05-SEP-2018	10:59	0	-8	Replicate 1	ND
								Replicate 2	ND
								Replicate 3	ND
								Geometric Mean	1.0000
		Malaria Monitoring		09-SEP-2018	07:34	4	-4	Replicate 1	ND
								Replicate 2	68
								Replicate 3	36
								Geometric Mean	13.4773
				10-SEP-2018	08:22	5	-3	Replicate 1	3029
								Replicate 2	3484
								Replicate 3	3348
								Geometric Mean	3281.3631
				10-SEP-2018	20:16	5	-3	Replicate 1	3408
								Replicate 2	3730
								Replicate 3	2716
								Geometric Mean	3256.2124

Notes: Day relative to inoculum is the number of days relative to day of administration of the inoculum, where the day of administration = 0.
The inoculum was administered on Study Day 0 for K13-infected subjects and Study Day 1 for 3D7-infected subjects.
Day relative to IP is the number of days relative to day of administration of Artesunate, where the day of dose = 1.
qPCR = quantitative Polymerase Chain Reaction; IP = Artesunate; ND = Not Detected; RBCs: red blood cells.

Listing 16.2.6.3
Malaria 18S qPCR

Randomisation Number	Challenge Agent	Visit	Timepoint	Date of Measurement	Time of Measurement	Day Relative to Inoculum	Day Relative to Artesunate	Assay	Result (parasites/500µL) of packed RBCs
R307	3D7	Malaria Monitoring		11-SEP-2018	08:01	6	-2	Replicate 1	530
								Replicate 2	580
								Replicate 3	464
								Geometric Mean	522.4851
				11-SEP-2018	19:12	6	-2	Replicate 1	20643
								Replicate 2	23143
								Replicate 3	22821
								Geometric Mean	22173.9151
				12-SEP-2018	07:11	7	-1	Replicate 1	67612
								Replicate 2	72648
								Replicate 3	61632
								Geometric Mean	67145.6531
				12-SEP-2018	19:56	7	-1	Replicate 1	15838
								Replicate 2	16004
								Replicate 3	16979
								Geometric Mean	16265.9836

Notes: Day relative to inoculum is the number of days relative to day of administration of the inoculum, where the day of administration = 0.
The inoculum was administered on Study Day 0 for K13-infected subjects and Study Day 1 for 3D7-infected subjects.
Day relative to IP is the number of days relative to day of administration of Artesunate, where the day of dose = 1.
qPCR = quantitative Polymerase Chain Reaction; IP = Artesunate; ND = Not Detected; RBCs: red blood cells.

Listing 16.2.6.3
Malaria 18S qPCR

Randomisation Number	Challenge Agent	Visit	Timepoint	Date of Measurement	Time of Measurement	Day Relative to Inoculum	Day Relative to Artesunate	Assay	Result (parasites/500µL) of packed RBCs
R307	3D7	Admission	Pre-dose	13-SEP-2018	09:51	8	1	Replicate 1	393145
								Replicate 2	383496
								Replicate 3	404678
								Geometric Mean	393677.9254
			4 Hrs Post-dose	13-SEP-2018	13:55	8	1	Replicate 1	494010
								Replicate 2	529964
								Replicate 3	486150
								Geometric Mean	503018.7032
			8 Hrs Post-dose	13-SEP-2018	17:55	8	1	Replicate 1	379018
								Replicate 2	422425
								Replicate 3	369215
								Geometric Mean	389549.7485
			12 Hrs Post-dose	13-SEP-2018	21:56	8	1	Replicate 1	60843
								Replicate 2	58816
								Replicate 3	61340
								Geometric Mean	60323.0501

Notes: Day relative to inoculum is the number of days relative to day of administration of the inoculum, where the day of administration = 0.
The inoculum was administered on Study Day 0 for K13-infected subjects and Study Day 1 for 3D7-infected subjects.
Day relative to IP is the number of days relative to day of administration of Artesunate, where the day of dose = 1.
qPCR = quantitative Polymerase Chain Reaction; IP = Artesunate; ND = Not Detected; RBCs: red blood cells.

Listing 16.2.6.3
Malaria 18S qPCR

Randomisation Number	Challenge Agent	Visit	Timepoint	Date of Measurement	Time of Measurement	Day Relative to Inoculum	Day Relative to Artesunate	Assay	Result (parasites/500µL) of packed RBCs
R307	3D7	Admission 1	16 Hrs Post-dose	14-SEP-2018	01:55	9	2	Replicate 1	16140
								Replicate 2	16133
								Replicate 3	17099
								Geometric Mean	16451.1575
			20 Hrs Post-dose	14-SEP-2018	05:55	9	2	Replicate 1	7809
								Replicate 2	5698
								Replicate 3	6149
								Geometric Mean	6491.9344
			24 Hrs Post-dose	14-SEP-2018	09:55	9	2	Replicate 1	2579
								Replicate 2	2327
								Replicate 3	2483
								Geometric Mean	2460.7915
			28 Hrs Post-dose	14-SEP-2018	13:56	9	2	Replicate 1	858
								Replicate 2	757
								Replicate 3	1026
								Geometric Mean	873.4610

Notes: Day relative to inoculum is the number of days relative to day of administration of the inoculum, where the day of administration = 0.
The inoculum was administered on Study Day 0 for K13-infected subjects and Study Day 1 for 3D7-infected subjects.
Day relative to IP is the number of days relative to day of administration of Artesunate, where the day of dose = 1.
qPCR = quantitative Polymerase Chain Reaction; IP = Artesunate; ND = Not Detected; RBCs: red blood cells.

Listing 16.2.6.3
Malaria 18S qPCR

Randomisation Number	Challenge Agent	Visit	Timepoint	Date of Measurement	Time of Measurement	Day Relative to Inoculum	Day Relative to Artesunate	Assay	Result (parasites/500µL) of packed RBCs
R307	3D7	Admission 1	32 Hrs Post-dose	14-SEP-2018	17:56	9	2	Replicate 1	690
								Replicate 2	332
								Replicate 3	162
								Geometric Mean	333.5550
			36 Hrs Post-dose	14-SEP-2018	21:56	9	2	Replicate 1	155
								Replicate 2	177
								Replicate 3	306
								Geometric Mean	203.2398
		Admission 2		15-SEP-2018	01:55	10	3	Replicate 1	288
								Replicate 2	253
								Replicate 3	304
								Geometric Mean	280.8421
				15-SEP-2018	05:55	10	3	Replicate 1	71
								Replicate 2	144
								Replicate 3	264
								Geometric Mean	139.2328

Notes: Day relative to inoculum is the number of days relative to day of administration of the inoculum, where the day of administration = 0.
The inoculum was administered on Study Day 0 for K13-infected subjects and Study Day 1 for 3D7-infected subjects.
Day relative to IP is the number of days relative to day of administration of Artesunate, where the day of dose = 1.
qPCR = quantitative Polymerase Chain Reaction; IP = Artesunate; ND = Not Detected; RBCs: red blood cells.

Listing 16.2.6.3
Malaria 18S qPCR

Randomisation Number	Challenge Agent	Visit	Timepoint	Date of Measurement	Time of Measurement	Day Relative to Inoculum	Day Relative to Artesunate	Assay	Result (parasites/500µL) of packed RBCs
R307	3D7	Admission 2	48 Hrs Post-dose	15-SEP-2018	09:54	10	3	Replicate 1	203
								Replicate 2	307
								Replicate 3	76
								Geometric Mean	167.9381
			52 Hrs Post-dose	15-SEP-2018	13:56	10	3	Replicate 1	95
								Replicate 2	92
								Replicate 3	104
								Geometric Mean	96.8683
			56 Hrs Post-dose	15-SEP-2018	17:57	10	3	Replicate 1	130
								Replicate 2	175
								Replicate 3	410
								Geometric Mean	210.5014
			60 Hrs Post-dose	15-SEP-2018	21:56	10	3	Replicate 1	144
								Replicate 2	173
								Replicate 3	183
								Geometric Mean	165.8135

Notes: Day relative to inoculum is the number of days relative to day of administration of the inoculum, where the day of administration = 0.
The inoculum was administered on Study Day 0 for K13-infected subjects and Study Day 1 for 3D7-infected subjects.
Day relative to IP is the number of days relative to day of administration of Artesunate, where the day of dose = 1.
qPCR = quantitative Polymerase Chain Reaction; IP = Artesunate; ND = Not Detected; RBCs: red blood cells.

Listing 16.2.6.3
Malaria 18S qPCR

Randomisation Number	Challenge Agent	Visit	Timepoint	Date of Measurement	Time of Measurement	Day Relative to Inoculum	Day Relative to Artesunate	Assay	Result (parasites/500µL) of packed RBCs
R307	3D7	Admission 3		16-SEP-2018	01:55	11	4	Replicate 1	67
								Replicate 2	34
								Replicate 3	33
								Geometric Mean	42.2042
				16-SEP-2018	05:55	11	4	Replicate 1	34
								Replicate 2	66
								Replicate 3	30
								Geometric Mean	40.6800
			72 Hrs Post-dose	16-SEP-2018	09:55	11	4	Replicate 1	ND
								Replicate 2	69
								Replicate 3	85
								Geometric Mean	18.0339
				16-SEP-2018	19:27	11	4	Replicate 1	174
								Replicate 2	73
								Replicate 3	75
								Geometric Mean	98.3961

Notes: Day relative to inoculum is the number of days relative to day of administration of the inoculum, where the day of administration = 0.
The inoculum was administered on Study Day 0 for K13-infected subjects and Study Day 1 for 3D7-infected subjects.
Day relative to IP is the number of days relative to day of administration of Artesunate, where the day of dose = 1.
qPCR = quantitative Polymerase Chain Reaction; IP = Artesunate; ND = Not Detected; RBCs: red blood cells.

Listing 16.2.6.3
Malaria 18S qPCR

Randomisation Number	Challenge Agent	Visit	Timepoint	Date of Measurement	Time of Measurement	Day Relative to Inoculum	Day Relative to Artesunate	Assay	Result (parasites/500µL) of packed RBCs
R307	3D7	Safety Monitoring		17-SEP-2018	07:35	12	5	Replicate 1	315
								Replicate 2	327
								Replicate 3	293
								Geometric Mean	311.3451
				17-SEP-2018	20:36	12	5	Replicate 1	546
								Replicate 2	591
								Replicate 3	988
								Geometric Mean	683.1442
				18-SEP-2018	08:08	13	6	Replicate 1	375
								Replicate 2	247
								Replicate 3	534
								Geometric Mean	367.0764
				18-SEP-2018	19:17	13	6	Replicate 1	7997
								Replicate 2	7650
								Replicate 3	7616
								Geometric Mean	7752.4413

Notes: Day relative to inoculum is the number of days relative to day of administration of the inoculum, where the day of administration = 0.
The inoculum was administered on Study Day 0 for K13-infected subjects and Study Day 1 for 3D7-infected subjects.
Day relative to IP is the number of days relative to day of administration of Artesunate, where the day of dose = 1.
qPCR = quantitative Polymerase Chain Reaction; IP = Artesunate; ND = Not Detected; RBCs: red blood cells.

Listing 16.2.6.3
Malaria 18S qPCR

Randomisation Number	Challenge Agent	Visit	Timepoint	Date of Measurement	Time of Measurement	Day Relative to Inoculum	Day Relative to Artesunate	Assay	Result (parasites/500µL) of packed RBCs
R307	3D7	Safety Monitoring		19-SEP-2018	07:49	14	7	Replicate 1	18280
								Replicate 2	18001
								Replicate 3	18500
								Geometric Mean	18259.1904
				19-SEP-2018	19:23	14	7	Replicate 1	8109
								Replicate 2	7899
								Replicate 3	7821
								Geometric Mean	7942.0735
				20-SEP-2018	08:01	15	8	Replicate 1	5839
								Replicate 2	5174
								Replicate 3	5506
								Geometric Mean	5499.6329
				21-SEP-2018	07:54	16	9	Replicate 1	45
								Replicate 2	44
								Replicate 3	13
								Geometric Mean	29.5259

Notes: Day relative to inoculum is the number of days relative to day of administration of the inoculum, where the day of administration = 0.
The inoculum was administered on Study Day 0 for K13-infected subjects and Study Day 1 for 3D7-infected subjects.
Day relative to IP is the number of days relative to day of administration of Artesunate, where the day of dose = 1.
qPCR = quantitative Polymerase Chain Reaction; IP = Artesunate; ND = Not Detected; RBCs: red blood cells.

Listing 16.2.6.3
Malaria 18S qPCR

Randomisation Number	Challenge Agent	Visit	Timepoint	Date of Measurement	Time of Measurement	Day Relative to Inoculum	Day Relative to Artesunate	Assay	Result (parasites/500µL) of packed RBCs
R307	3D7	Safety Monitoring		22-SEP-2018	08:38	17	10	Replicate 1	26
								Replicate 2	ND
								Replicate 3	26
								Geometric Mean	8.7764
				24-SEP-2018	07:38	19	12	Replicate 1	29
								Replicate 2	ND
								Replicate 3	ND
								Geometric Mean	3.0723
				25-SEP-2018	07:36	20	13	Replicate 1	24
								Replicate 2	ND
								Replicate 3	21
								Geometric Mean	7.9581
				28-SEP-2018	07:40	23	16	Replicate 1	14
								Replicate 2	15
								Replicate 3	23
								Geometric Mean	16.9037

Notes: Day relative to inoculum is the number of days relative to day of administration of the inoculum, where the day of administration = 0.
The inoculum was administered on Study Day 0 for K13-infected subjects and Study Day 1 for 3D7-infected subjects.
Day relative to IP is the number of days relative to day of administration of Artesunate, where the day of dose = 1.
qPCR = quantitative Polymerase Chain Reaction; IP = Artesunate; ND = Not Detected; RBCs: red blood cells.

Listing 16.2.6.3
Malaria 18S qPCR

Randomisation Number	Challenge Agent	Visit	Timepoint	Date of Measurement	Time of Measurement	Day Relative to Inoculum	Day Relative to Artesunate	Assay	Result (parasites/500µL) of packed RBCs
R307	3D7	End of Study		02-OCT-2018	07:21	27	20	Replicate 1	25
								Replicate 2	124
								Replicate 3	52
								Geometric Mean	54.4237
		Unscheduled		05-OCT-2018	07:55	30	23	Replicate 1	ND
								Replicate 2	27
								Replicate 3	28
								Geometric Mean	9.1098
				11-OCT-2018	08:33	36	29	Replicate 1	30
								Replicate 2	ND
								Replicate 3	ND
								Geometric Mean	3.1072
				18-OCT-2018	07:56	43	36	Replicate 1	ND
								Replicate 2	ND
								Replicate 3	ND
								Geometric Mean	1.0000

Notes: Day relative to inoculum is the number of days relative to day of administration of the inoculum, where the day of administration = 0.
The inoculum was administered on Study Day 0 for K13-infected subjects and Study Day 1 for 3D7-infected subjects.
Day relative to IP is the number of days relative to day of administration of Artesunate, where the day of dose = 1.
qPCR = quantitative Polymerase Chain Reaction; IP = Artesunate; ND = Not Detected; RBCs: red blood cells.

16.2.6.4 qRT-PCR *pfs25* Daily

Listing 16.2.6.4
qRT-PCR pfs25 Daily

Randomisation Number	Challenge Agent	Visit	Timepoint	Date of Measurement	Time of Measurement	Day Relative to Inoculum	Day Relative to Artesunate	Assay	Result (copies of transcripts/500 µL packed RBCs)
R103	K13	Safety Monitoring		30-NOV-2017	10:09	16	8	Replicate 1	29292
								Replicate 2	22638
				05-DEC-2017	08:07	21	13	Replicate 1	1005576
								Replicate 2	977586
				07-DEC-2017	08:41	23	15	Replicate 1	1548364
								Replicate 2	1395688
R104	K13	Safety Monitoring		30-NOV-2017	08:43	16	8	Replicate 1	8646
								Replicate 2	8060
				05-DEC-2017	08:30	21	13	Replicate 1	1849234
								Replicate 2	1706826
				07-DEC-2017	08:35	23	15	Replicate 1	4601170
								Replicate 2	3634450
R105	K13	Safety Monitoring		30-NOV-2017	07:08	16	8	Replicate 1	14850
								Replicate 2	14824
				05-DEC-2017	07:24	21	13	Replicate 1	230004

Notes: Day relative to inoculum is the number of days relative to day of administration of the inoculum, where the day of administration = 0.
The inoculum was administered on Study Day 0 for K13-infected subjects and Study Day 1 for 3D7-infected subjects.
Day relative to IP is the number of days relative to day of administration of Artesunate, where the day of dose = 1.
qRT-PCR = quantitative reverse transcription polymerase chain reaction; IP = Artesunate; ND = Not Detected

Listing 16.2.6.4
qRT-PCR pfs25 Daily

Randomisation Number	Challenge Agent	Visit	Timepoint	Date of Measurement	Time of Measurement	Day Relative to Inoculum	Day Relative to Artesunate	Assay	Result (copies of transcripts/500 µL packed RBCs)
R105	K13	Safety Monitoring		05-DEC-2017	07:24	21	13	Replicate 2	203582
				07-DEC-2017	08:37	23	15	Replicate 1	472244
								Replicate 2	539394
R106	3D7	Safety Monitoring		30-NOV-2017	08:46	15	8	Replicate 1	ND
								Replicate 2	ND
				05-DEC-2017	08:35	20	13	Replicate 1	ND
								Replicate 2	ND
R107	3D7	Safety Monitoring		30-NOV-2017	07:30	15	8	Replicate 1	ND
								Replicate 2	ND
				05-DEC-2017	07:41	20	13	Replicate 1	298
								Replicate 2	ND
R108	3D7	Safety Monitoring		30-NOV-2017	06:57	15	8	Replicate 1	ND
								Replicate 2	ND
R201	K13	Safety Monitoring		12-MAR-2018	08:28	21	13	Replicate 1	180860
								Replicate 2	240940

Notes: Day relative to inoculum is the number of days relative to day of administration of the inoculum, where the day of administration = 0.
The inoculum was administered on Study Day 0 for K13-infected subjects and Study Day 1 for 3D7-infected subjects.
Day relative to IP is the number of days relative to day of administration of Artesunate, where the day of dose = 1.
qRT-PCR = quantitative reverse transcription polymerase chain reaction; IP = Artesunate; ND = Not Detected

Listing 16.2.6.4
qRT-PCR pfs25 Daily

Randomisation Number	Challenge Agent	Visit	Timepoint	Date of Measurement	Time of Measurement	Day Relative to Inoculum	Day Relative to Artesunate	Assay	Result (copies of transcripts/500 µL packed RBCs)
R201	K13	Safety Monitoring		14-MAR-2018	09:06	23	15	Replicate 1	109800
								Replicate 2	132216
R202	K13	Safety Monitoring		12-MAR-2018	09:51	21	13	Replicate 1	1111520
								Replicate 2	1648780
				14-MAR-2018	08:51	23	15	Replicate 1	363964
								Replicate 2	475696
R203	K13	Safety Monitoring		12-MAR-2018	09:17	21	13	Replicate 1	3488520
								Replicate 2	3324160
				14-MAR-2018	10:15	23	15	Replicate 1	983838
								Replicate 2	1079598
R204	K13	Safety Monitoring		12-MAR-2018	09:10	21	13	Replicate 1	10600
								Replicate 2	17460
				14-MAR-2018	09:59	23	15	Replicate 1	27560
								Replicate 2	30630
R205	K13	Safety Monitoring		12-MAR-2018	07:48	21	13	Replicate 1	528700

Notes: Day relative to inoculum is the number of days relative to day of administration of the inoculum, where the day of administration = 0.
The inoculum was administered on Study Day 0 for K13-infected subjects and Study Day 1 for 3D7-infected subjects.
Day relative to IP is the number of days relative to day of administration of Artesunate, where the day of dose = 1.
qRT-PCR = quantitative reverse transcription polymerase chain reaction; IP = Artesunate; ND = Not Detected

Listing 16.2.6.4
qRT-PCR pfs25 Daily

Randomisation Number	Challenge Agent	Visit	Timepoint	Date of Measurement	Time of Measurement	Day Relative to Inoculum	Day Relative to Artesunate	Assay	Result (copies of transcripts/500 μL packed RBCs)
R205	K13	Safety Monitoring		12-MAR-2018	07:48	21	13	Replicate 2	157340
				14-MAR-2018	08:46	23	15	Replicate 1	435876
								Replicate 2	452458
R206	K13	Safety Monitoring		12-MAR-2018	08:44	21	13	Replicate 1	1591240
								Replicate 2	1263800
				14-MAR-2018	10:05	23	15	Replicate 1	451634
								Replicate 2	613294
R207	K13	Safety Monitoring		12-MAR-2018	09:34	21	13	Replicate 1	55160
								Replicate 2	68700
				14-MAR-2018	09:10	23	15	Replicate 1	11300
								Replicate 2	13858
R208	3D7	Safety Monitoring		12-MAR-2018	07:11	20	13	Replicate 1	ND
								Replicate 2	ND
				14-MAR-2018	07:14	22	15	Replicate 1	1960
								Replicate 2	2106

Notes: Day relative to inoculum is the number of days relative to day of administration of the inoculum, where the day of administration = 0.
The inoculum was administered on Study Day 0 for K13-infected subjects and Study Day 1 for 3D7-infected subjects.
Day relative to IP is the number of days relative to day of administration of Artesunate, where the day of dose = 1.
qRT-PCR = quantitative reverse transcription polymerase chain reaction; IP = Artesunate; ND = Not Detected

Listing 16.2.6.4
qRT-PCR pfs25 Daily

Randomisation Number	Challenge Agent	Visit	Timepoint	Date of Measurement	Time of Measurement	Day Relative to Inoculum	Day Relative to Artesunate	Assay	Result (copies of transcripts/500 µL packed RBCs)
R209	3D7	Safety Monitoring		12-MAR-2018	08:12	20	13	Replicate 1	100
								Replicate 2	ND
				14-MAR-2018	07:40	22	15	Replicate 1	35176
								Replicate 2	31852
R210	3D7	Safety Monitoring		12-MAR-2018	07:37	20	13	Replicate 1	12480
								Replicate 2	3840
				14-MAR-2018	07:17	22	15	Replicate 1	28842
								Replicate 2	25460
R302	K13	Safety Monitoring		20-SEP-2018	07:25	16	8	Replicate 1	17600
								Replicate 2	20600
R303	K13	Safety Monitoring		20-SEP-2018	08:16	16	8	Replicate 1	622200
								Replicate 2	748600
R304	K13	Safety Monitoring		20-SEP-2018	08:09	16	8	Replicate 1	98800
								Replicate 2	128400
R305	3D7	Safety Monitoring		18-SEP-2018	07:16	13	6	Replicate 1	800

Notes: Day relative to inoculum is the number of days relative to day of administration of the inoculum, where the day of administration = 0.
The inoculum was administered on Study Day 0 for K13-infected subjects and Study Day 1 for 3D7-infected subjects.
Day relative to IP is the number of days relative to day of administration of Artesunate, where the day of dose = 1.
qRT-PCR = quantitative reverse transcription polymerase chain reaction; IP = Artesunate; ND = Not Detected

Listing 16.2.6.4
qRT-PCR pfs25 Daily

Randomisation Number	Challenge Agent	Visit	Timepoint	Date of Measurement	Time of Measurement	Day Relative to Inoculum	Day Relative to Artesunate	Assay	Result (copies of transcripts/500 µL packed RBCs)
R305	3D7	Safety Monitoring		18-SEP-2018	07:16	13	6	Replicate 2	2000
				21-SEP-2018	07:03	16	9	Replicate 1	13000
								Replicate 2	29400
R306	3D7	Safety Monitoring		21-SEP-2018	07:32	16	9	Replicate 1	14400
								Replicate 2	14400
R307	3D7	Safety Monitoring		21-SEP-2018	07:54	16	9	Replicate 1	4400
								Replicate 2	7400

Notes: Day relative to inoculum is the number of days relative to day of administration of the inoculum, where the day of administration = 0.
The inoculum was administered on Study Day 0 for K13-infected subjects and Study Day 1 for 3D7-infected subjects.
Day relative to IP is the number of days relative to day of administration of Artesunate, where the day of dose = 1.
qRT-PCR = quantitative reverse transcription polymerase chain reaction; IP = Artesunate; ND = Not Detected

16.2.6.5 qRT-PCR *pfs25* Retrospective

Listing 16.2.6.5
qRT-PCR pfs25 Retrospective

Randomisation Number	Challenge Agent	Visit	Timepoint	Date of Measurement	Time of Measurement	Day Relative to Inoculum	Day Relative to Artesunate	Assay	Result (copies of transcripts/500 µL packed RBCs)
R103	K13	Admission	Pre-dose	23-NOV-2017	09:50	9	1	Replicate 1	1500
								Replicate 2	880
		Admission 1	24 Hrs Post-dose	24-NOV-2017	10:51	10	2	Replicate 1	2028
								Replicate 2	282
		Admission 2	48 Hrs Post-dose	25-NOV-2017	10:50	11	3	Replicate 1	654
								Replicate 2	436
		Safety Monitoring		30-NOV-2017	10:09	16	8	Replicate 1	42280
								Replicate 2	65998
				01-DEC-2017	10:41	17	9	Replicate 1	1216384
								Replicate 2	431532
				05-DEC-2017	08:07	21	13	Replicate 1	3336288
								Replicate 2	2889200
				07-DEC-2017	08:41	23	15	Replicate 1	641864
								Replicate 2	2176622
				11-DEC-2017	10:17	27	19	Replicate 1	2269734
								Replicate 2	1829136

Notes: Day relative to inoculum is the number of days relative to day of administration of the inoculum, where the day of administration = 0.
The inoculum was administered on Study Day 0 for K13-infected subjects and Study Day 1 for 3D7-infected subjects.
Day relative to IP is the number of days relative to day of administration of Artesunate, where the day of dose = 1.
qRT-PCR = quantitative reverse transcription polymerase chain reaction; IP = Artesunate; ND = Not Detected; RBCs: red blood cells.
Method of analysis for all samples was Quantitative (RNAprotect Cell Reagent with Rneasy Plus Mini kit).

Listing 16.2.6.5
qRT-PCR pfs25 Retrospective

Randomisation Number	Challenge Agent	Visit	Timepoint	Date of Measurement	Time of Measurement	Day Relative to Inoculum	Day Relative to Artesunate	Assay	Result (copies of transcripts/500 µL packed RBCs)
R103	K13	End of Study		14-DEC-2017	09:37	30	22	Replicate 1	377122
								Replicate 2	358372
R104	K13	Admission	Pre-dose	23-NOV-2017	09:44	9	1	Replicate 1	802
								Replicate 2	822
		Admission 1	24 Hrs Post-dose	24-NOV-2017	10:40	10	2	Replicate 1	1188
								Replicate 2	1006
		Admission 2	48 Hrs Post-dose	25-NOV-2017	10:41	11	3	Replicate 1	376
								Replicate 2	232
		Safety Monitoring		30-NOV-2017	08:43	16	8	Replicate 1	6420
								Replicate 2	18244
				01-DEC-2017	08:25	17	9	Replicate 1	574374
								Replicate 2	442274
				05-DEC-2017	08:30	21	13	Replicate 1	6575016
								Replicate 2	7960638
				07-DEC-2017	08:35	23	15	Replicate 1	6887576
								Replicate 2	7287628

Notes: Day relative to inoculum is the number of days relative to day of administration of the inoculum, where the day of administration = 0.
The inoculum was administered on Study Day 0 for K13-infected subjects and Study Day 1 for 3D7-infected subjects.
Day relative to IP is the number of days relative to day of administration of Artesunate, where the day of dose = 1.
qRT-PCR = quantitative reverse transcription polymerase chain reaction; IP = Artesunate; ND = Not Detected; RBCs: red blood cells.
Method of analysis for all samples was Quantitative (RNAprotect Cell Reagent with Rneasy Plus Mini kit).

Listing 16.2.6.5
qRT-PCR pfs25 Retrospective

Randomisation Number	Challenge Agent	Visit	Timepoint	Date of Measurement	Time of Measurement	Day Relative to Inoculum	Day Relative to Artesunate	Assay	Result (copies of transcripts/500 µL packed RBCs)
R104	K13	Safety Monitoring		11-DEC-2017	08:01	27	19	Replicate 1	1147518
								Replicate 2	1426526
		End of Study		14-DEC-2017	08:42	30	22	Replicate 1	1556846
								Replicate 2	1498428
R105	K13	Admission	Pre-dose	23-NOV-2017	09:36	9	1	Replicate 1	286
								Replicate 2	514
		Admission 1	24 Hrs Post-dose	24-NOV-2017	10:30	10	2	Replicate 1	270
								Replicate 2	72
		Admission 2	48 Hrs Post-dose	25-NOV-2017	10:30	11	3	Replicate 1	96
								Replicate 2	ND
		Safety Monitoring		30-NOV-2017	07:08	16	8	Replicate 1	15856
								Replicate 2	15444
				01-DEC-2017	13:20	17	9	Replicate 1	139260
								Replicate 2	81746
				05-DEC-2017	07:24	21	13	Replicate 1	783526
								Replicate 2	831304

Notes: Day relative to inoculum is the number of days relative to day of administration of the inoculum, where the day of administration = 0.
The inoculum was administered on Study Day 0 for K13-infected subjects and Study Day 1 for 3D7-infected subjects.
Day relative to IP is the number of days relative to day of administration of Artesunate, where the day of dose = 1.
qRT-PCR = quantitative reverse transcription polymerase chain reaction; IP = Artesunate; ND = Not Detected; RBCs: red blood cells.
Method of analysis for all samples was Quantitative (RNAprotect Cell Reagent with Rneasy Plus Mini kit).

Listing 16.2.6.5
qRT-PCR pfs25 Retrospective

Randomisation Number	Challenge Agent	Visit	Timepoint	Date of Measurement	Time of Measurement	Day Relative to Inoculum	Day Relative to Artesunate	Assay	Result (copies of transcripts/500 µL packed RBCs)
R105	K13	Safety Monitoring		07-DEC-2017	08:37	23	15	Replicate 1	763104
								Replicate 2	906422
				11-DEC-2017	08:44	27	19	Replicate 1	297888
								Replicate 2	360008
		End of Study		13-DEC-2017	08:54	29	21	Replicate 1	182842
								Replicate 2	231896
R106	3D7	Admission	Pre-dose	23-NOV-2017	09:33	8	1	Replicate 1	144
								Replicate 2	ND
		Admission 1	28 Hrs Post-dose	24-NOV-2017	14:21	9	2	Replicate 1	ND
								Replicate 2	ND
		Admission 2	48 Hrs Post-dose	25-NOV-2017	10:20	10	3	Replicate 1	ND
								Replicate 2	ND
		Admission 3		26-NOV-2017	19:17	11	4	Replicate 1	ND
								Replicate 2	ND
		Safety Monitoring		28-NOV-2017	08:13	13	6	Replicate 1	ND
								Replicate 2	ND

Notes: Day relative to inoculum is the number of days relative to day of administration of the inoculum, where the day of administration = 0.
The inoculum was administered on Study Day 0 for K13-infected subjects and Study Day 1 for 3D7-infected subjects.
Day relative to IP is the number of days relative to day of administration of Artesunate, where the day of dose = 1.
qRT-PCR = quantitative reverse transcription polymerase chain reaction; IP = Artesunate; ND = Not Detected; RBCs: red blood cells.
Method of analysis for all samples was Quantitative (RNAprotect Cell Reagent with Rneasy Plus Mini kit).

Listing 16.2.6.5
qRT-PCR pfs25 Retrospective

Randomisation Number	Challenge Agent	Visit	Timepoint	Date of Measurement	Time of Measurement	Day Relative to Inoculum	Day Relative to Artesunate	Assay	Result (copies of transcripts/500 µL packed RBCs)
R106	3D7	Safety Monitoring		29-NOV-2017	08:35	14	7	Replicate 1	ND
								Replicate 2	ND
				30-NOV-2017	08:46	15	8	Replicate 1	ND
								Replicate 2	ND
				01-DEC-2017	08:27	16	9	Replicate 1	64
								Replicate 2	140
				07-DEC-2017	07:57	22	15	Replicate 1	7026
								Replicate 2	8942
				11-DEC-2017	08:04	26	19	Replicate 1	2112
								Replicate 2	1590
		End of Study		14-DEC-2017	08:53	29	22	Replicate 1	5514
								Replicate 2	18444
R107	3D7	Admission	Pre-dose	23-NOV-2017	09:35	8	1	Replicate 1	ND
								Replicate 2	ND
		Admission 1	28 Hrs Post-dose	24-NOV-2017	14:10	9	2	Replicate 1	ND
								Replicate 2	ND

Notes: Day relative to inoculum is the number of days relative to day of administration of the inoculum, where the day of administration = 0.
The inoculum was administered on Study Day 0 for K13-infected subjects and Study Day 1 for 3D7-infected subjects.
Day relative to IP is the number of days relative to day of administration of Artesunate, where the day of dose = 1.
qRT-PCR = quantitative reverse transcription polymerase chain reaction; IP = Artesunate; ND = Not Detected; RBCs: red blood cells.
Method of analysis for all samples was Quantitative (RNAprotect Cell Reagent with Rneasy Plus Mini kit).

Listing 16.2.6.5
qRT-PCR pfs25 Retrospective

Randomisation Number	Challenge Agent	Visit	Timepoint	Date of Measurement	Time of Measurement	Day Relative to Inoculum	Day Relative to Artesunate	Assay	Result (copies of transcripts/500 µL packed RBCs)
R107	3D7	Admission 2	48 Hrs Post-dose	25-NOV-2017	10:10	10	3	Replicate 1	ND
								Replicate 2	ND
		Admission 3		26-NOV-2017	19:06	11	4	Replicate 1	ND
								Replicate 2	ND
		Safety Monitoring		29-NOV-2017	07:37	14	7	Replicate 1	ND
								Replicate 2	ND
				30-NOV-2017	07:30	15	8	Replicate 1	ND
								Replicate 2	ND
				01-DEC-2017	07:43	16	9	Replicate 1	ND
								Replicate 2	ND
				05-DEC-2017	07:41	20	13	Replicate 1	ND
								Replicate 2	300
				07-DEC-2017	09:15	22	15	Replicate 1	12640
								Replicate 2	10450
				11-DEC-2017	09:29	26	19	Replicate 1	1928342
								Replicate 2	1855982

Notes: Day relative to inoculum is the number of days relative to day of administration of the inoculum, where the day of administration = 0.
The inoculum was administered on Study Day 0 for K13-infected subjects and Study Day 1 for 3D7-infected subjects.
Day relative to IP is the number of days relative to day of administration of Artesunate, where the day of dose = 1.
qRT-PCR = quantitative reverse transcription polymerase chain reaction; IP = Artesunate; ND = Not Detected; RBCs: red blood cells.
Method of analysis for all samples was Quantitative (RNAprotect Cell Reagent with Rneasy Plus Mini kit).

Listing 16.2.6.5
qRT-PCR pfs25 Retrospective

Randomisation Number	Challenge Agent	Visit	Timepoint	Date of Measurement	Time of Measurement	Day Relative to Inoculum	Day Relative to Artesunate	Assay	Result (copies of transcripts/500 µL packed RBCs)
R107	3D7	End of Study		14-DEC-2017	09:31	29	22	Replicate 1	2104506
								Replicate 2	2373306
R108	3D7	Admission	Pre-dose	23-NOV-2017	09:17	8	1	Replicate 1	ND
								Replicate 2	ND
		Admission 1	28 Hrs Post-dose	24-NOV-2017	14:04	9	2	Replicate 1	ND
								Replicate 2	ND
		Admission 3		26-NOV-2017	19:05	11	4	Replicate 1	ND
								Replicate 2	ND
		Safety Monitoring		28-NOV-2017	08:50	13	6	Replicate 1	ND
								Replicate 2	ND
				29-NOV-2017	07:06	14	7	Replicate 1	ND
								Replicate 2	ND
				30-NOV-2017	06:57	15	8	Replicate 1	ND
								Replicate 2	ND
				01-DEC-2017	07:14	16	9	Replicate 1	142
								Replicate 2	158

Notes: Day relative to inoculum is the number of days relative to day of administration of the inoculum, where the day of administration = 0.
The inoculum was administered on Study Day 0 for K13-infected subjects and Study Day 1 for 3D7-infected subjects.
Day relative to IP is the number of days relative to day of administration of Artesunate, where the day of dose = 1.
qRT-PCR = quantitative reverse transcription polymerase chain reaction; IP = Artesunate; ND = Not Detected; RBCs: red blood cells.
Method of analysis for all samples was Quantitative (RNAprotect Cell Reagent with Rneasy Plus Mini kit).

Listing 16.2.6.5
qRT-PCR pfs25 Retrospective

Randomisation Number	Challenge Agent	Visit	Timepoint	Date of Measurement	Time of Measurement	Day Relative to Inoculum	Day Relative to Artesunate	Assay	Result (copies of transcripts/500 µL packed RBCs)
R108	3D7	Safety Monitoring		07-DEC-2017	06:54	22	15	Replicate 1	49856
								Replicate 2	67456
				11-DEC-2017	08:54	26	19	Replicate 1	48690
								Replicate 2	43412
		End of Study		14-DEC-2017	08:37	29	22	Replicate 1	108114
								Replicate 2	145482
R201	K13	Malaria Monitoring		25-FEB-2018	08:30	6	-3	Replicate 1	106
								Replicate 2	ND
		Admission	Pre-dose	28-FEB-2018	12:39	9	1	Replicate 1	632
								Replicate 2	392
			8 Hrs Post-dose	28-FEB-2018	21:22	9	1	Replicate 1	164
								Replicate 2	484
		Admission 3	72 Hrs Post-dose	03-MAR-2018	13:27	12	4	Replicate 1	ND
								Replicate 2	102
		Safety Monitoring		06-MAR-2018	09:29	15	7	Replicate 1	8382
								Replicate 2	8466

Notes: Day relative to inoculum is the number of days relative to day of administration of the inoculum, where the day of administration = 0.
The inoculum was administered on Study Day 0 for K13-infected subjects and Study Day 1 for 3D7-infected subjects.
Day relative to IP is the number of days relative to day of administration of Artesunate, where the day of dose = 1.
qRT-PCR = quantitative reverse transcription polymerase chain reaction; IP = Artesunate; ND = Not Detected; RBCs: red blood cells.
Method of analysis for all samples was Quantitative (RNAprotect Cell Reagent with Rneasy Plus Mini kit).

Listing 16.2.6.5
qRT-PCR pfs25 Retrospective

Randomisation Number	Challenge Agent	Visit	Timepoint	Date of Measurement	Time of Measurement	Day Relative to Inoculum	Day Relative to Artesunate	Assay	Result (copies of transcripts/500 µL packed RBCs)
R201	K13	Safety Monitoring		07-MAR-2018	07:34	16	8	Replicate 1	30854
								Replicate 2	26236
				08-MAR-2018	08:59	17	9	Replicate 1	196480
								Replicate 2	133426
				09-MAR-2018	09:59	18	10	Replicate 1	98166
								Replicate 2	131736
				14-MAR-2018	09:06	23	15	Replicate 1	116600
								Replicate 2	139200
				16-MAR-2018	08:48	25	17	Replicate 1	29940
								Replicate 2	33094
R202	K13	Malaria Monitoring		25-FEB-2018	08:47	6	-3	Replicate 1	ND
								Replicate 2	ND
		Admission	Pre-dose	28-FEB-2018	12:05	9	1	Replicate 1	700
								Replicate 2	1224
			8 Hrs Post-dose	28-FEB-2018	21:03	9	1	Replicate 1	556
								Replicate 2	688

Notes: Day relative to inoculum is the number of days relative to day of administration of the inoculum, where the day of administration = 0.
The inoculum was administered on Study Day 0 for K13-infected subjects and Study Day 1 for 3D7-infected subjects.
Day relative to IP is the number of days relative to day of administration of Artesunate, where the day of dose = 1.
qRT-PCR = quantitative reverse transcription polymerase chain reaction; IP = Artesunate; ND = Not Detected; RBCs: red blood cells.
Method of analysis for all samples was Quantitative (RNAprotect Cell Reagent with Rneasy Plus Mini kit).

Listing 16.2.6.5
qRT-PCR pfs25 Retrospective

Randomisation Number	Challenge Agent	Visit	Timepoint	Date of Measurement	Time of Measurement	Day Relative to Inoculum	Day Relative to Artesunate	Assay	Result (copies of transcripts/500 µL packed RBCs)
R202	K13	Admission 3	72 Hrs Post-dose	03-MAR-2018	13:05	12	4	Replicate 1	13694
								Replicate 2	9830
		Safety Monitoring		06-MAR-2018	09:51	15	7	Replicate 1	23202
								Replicate 2	32586
				07-MAR-2018	09:07	16	8	Replicate 1	68572
								Replicate 2	103396
				08-MAR-2018	09:17	17	9	Replicate 1	571244
								Replicate 2	587600
				09-MAR-2018	09:32	18	10	Replicate 1	158628
								Replicate 2	125186
				10-MAR-2018	08:52	19	11	Replicate 1	5076686
								Replicate 2	2062764
				14-MAR-2018	08:51	23	15	Replicate 1	766600
								Replicate 2	516400
				16-MAR-2018	09:35	25	17	Replicate 1	861356
								Replicate 2	723188

Notes: Day relative to inoculum is the number of days relative to day of administration of the inoculum, where the day of administration = 0.
The inoculum was administered on Study Day 0 for K13-infected subjects and Study Day 1 for 3D7-infected subjects.
Day relative to IP is the number of days relative to day of administration of Artesunate, where the day of dose = 1.
qRT-PCR = quantitative reverse transcription polymerase chain reaction; IP = Artesunate; ND = Not Detected; RBCs: red blood cells.
Method of analysis for all samples was Quantitative (RNAprotect Cell Reagent with Rneasy Plus Mini kit).

Listing 16.2.6.5
qRT-PCR pfs25 Retrospective

Randomisation Number	Challenge Agent	Visit	Timepoint	Date of Measurement	Time of Measurement	Day Relative to Inoculum	Day Relative to Artesunate	Assay	Result (copies of transcripts/500 µL packed RBCs)
R203	K13	Malaria Monitoring		25-FEB-2018	09:03	6	-3	Replicate 1	ND
								Replicate 2	ND
		Admission	Pre-dose	28-FEB-2018	11:14	9	1	Replicate 1	4136
								Replicate 2	5384
			8 Hrs Post-dose	28-FEB-2018	19:40	9	1	Replicate 1	3342
								Replicate 2	1984
		Admission 3	72 Hrs Post-dose	03-MAR-2018	11:50	12	4	Replicate 1	25692
								Replicate 2	15972
		Safety Monitoring		06-MAR-2018	09:29	15	7	Replicate 1	76628
								Replicate 2	64454
				07-MAR-2018	10:04	16	8	Replicate 1	469242
								Replicate 2	559914
				08-MAR-2018	10:00	17	9	Replicate 1	2652176
								Replicate 2	2311450
				09-MAR-2018	10:14	18	10	Replicate 1	1921330
								Replicate 2	2661854

Notes: Day relative to inoculum is the number of days relative to day of administration of the inoculum, where the day of administration = 0.
The inoculum was administered on Study Day 0 for K13-infected subjects and Study Day 1 for 3D7-infected subjects.
Day relative to IP is the number of days relative to day of administration of Artesunate, where the day of dose = 1.
qRT-PCR = quantitative reverse transcription polymerase chain reaction; IP = Artesunate; ND = Not Detected; RBCs: red blood cells.
Method of analysis for all samples was Quantitative (RNAprotect Cell Reagent with Rneasy Plus Mini kit).

Listing 16.2.6.5
qRT-PCR pfs25 Retrospective

Randomisation Number	Challenge Agent	Visit	Timepoint	Date of Measurement	Time of Measurement	Day Relative to Inoculum	Day Relative to Artesunate	Assay	Result (copies of transcripts/500 µL packed RBCs)
R203	K13	Safety Monitoring		10-MAR-2018	08:37	19	11	Replicate 1	8114336
								Replicate 2	11344622
				14-MAR-2018	10:15	23	15	Replicate 1	4380400
								Replicate 2	4412400
				16-MAR-2018	09:03	25	17	Replicate 1	3388338
								Replicate 2	2529514
R204	K13	Malaria Monitoring		25-FEB-2018	08:38	6	-3	Replicate 1	ND
								Replicate 2	ND
		Admission	Pre-dose	28-FEB-2018	12:29	9	1	Replicate 1	370
								Replicate 2	144
			8 Hrs Post-dose	28-FEB-2018	21:17	9	1	Replicate 1	88
								Replicate 2	236
		Admission 3	72 Hrs Post-dose	03-MAR-2018	13:21	12	4	Replicate 1	350
								Replicate 2	382
		Safety Monitoring		06-MAR-2018	09:04	15	7	Replicate 1	2964
								Replicate 2	3428

Notes: Day relative to inoculum is the number of days relative to day of administration of the inoculum, where the day of administration = 0.
The inoculum was administered on Study Day 0 for K13-infected subjects and Study Day 1 for 3D7-infected subjects.
Day relative to IP is the number of days relative to day of administration of Artesunate, where the day of dose = 1.
qRT-PCR = quantitative reverse transcription polymerase chain reaction; IP = Artesunate; ND = Not Detected; RBCs: red blood cells.
Method of analysis for all samples was Quantitative (RNAprotect Cell Reagent with Rneasy Plus Mini kit).

Listing 16.2.6.5
qRT-PCR pfs25 Retrospective

Randomisation Number	Challenge Agent	Visit	Timepoint	Date of Measurement	Time of Measurement	Day Relative to Inoculum	Day Relative to Artesunate	Assay	Result (copies of transcripts/500 µL packed RBCs)
R204	K13	Safety Monitoring		07-MAR-2018	09:32	16	8	Replicate 1	1966
								Replicate 2	1776
				08-MAR-2018	08:20	17	9	Replicate 1	22700
								Replicate 2	21162
				14-MAR-2018	09:59	23	15	Replicate 1	48200
								Replicate 2	30200
				16-MAR-2018	07:15	25	17	Replicate 1	ND
								Replicate 2	ND
R205	K13	Malaria Monitoring		25-FEB-2018	07:45	6	-3	Replicate 1	ND
								Replicate 2	ND
		Admission	Pre-dose	28-FEB-2018	12:19	9	1	Replicate 1	792
								Replicate 2	448
			8 Hrs Post-dose	28-FEB-2018	21:10	9	1	Replicate 1	208
								Replicate 2	534
		Admission 3	72 Hrs Post-dose	03-MAR-2018	13:15	12	4	Replicate 1	290
								Replicate 2	182

Notes: Day relative to inoculum is the number of days relative to day of administration of the inoculum, where the day of administration = 0.
The inoculum was administered on Study Day 0 for K13-infected subjects and Study Day 1 for 3D7-infected subjects.
Day relative to IP is the number of days relative to day of administration of Artesunate, where the day of dose = 1.
qRT-PCR = quantitative reverse transcription polymerase chain reaction; IP = Artesunate; ND = Not Detected; RBCs: red blood cells.
Method of analysis for all samples was Quantitative (RNAprotect Cell Reagent with Rneasy Plus Mini kit).

Listing 16.2.6.5
qRT-PCR pfs25 Retrospective

Randomisation Number	Challenge Agent	Visit	Timepoint	Date of Measurement	Time of Measurement	Day Relative to Inoculum	Day Relative to Artesunate	Assay	Result (copies of transcripts/500 µL packed RBCs)
R205	K13	Safety Monitoring		06-MAR-2018	07:20	15	7	Replicate 1	1694
								Replicate 2	2286
				07-MAR-2018	07:54	16	8	Replicate 1	31736
								Replicate 2	56738
				08-MAR-2018	07:33	17	9	Replicate 1	325398
								Replicate 2	362970
				09-MAR-2018	07:52	18	10	Replicate 1	55512
								Replicate 2	55010
				14-MAR-2018	08:46	23	15	Replicate 1	1431800
								Replicate 2	1416200
				16-MAR-2018	07:36	25	17	Replicate 1	119722
								Replicate 2	93626
R206	K13	Malaria Monitoring		25-FEB-2018	08:16	6	-3	Replicate 1	ND
								Replicate 2	ND
		Admission	Pre-dose	28-FEB-2018	12:48	9	1	Replicate 1	764
								Replicate 2	954

Notes: Day relative to inoculum is the number of days relative to day of administration of the inoculum, where the day of administration = 0.
The inoculum was administered on Study Day 0 for K13-infected subjects and Study Day 1 for 3D7-infected subjects.
Day relative to IP is the number of days relative to day of administration of Artesunate, where the day of dose = 1.
qRT-PCR = quantitative reverse transcription polymerase chain reaction; IP = Artesunate; ND = Not Detected; RBCs: red blood cells.
Method of analysis for all samples was Quantitative (RNAprotect Cell Reagent with Rneasy Plus Mini kit).

Listing 16.2.6.5
qRT-PCR pfs25 Retrospective

Randomisation Number	Challenge Agent	Visit	Timepoint	Date of Measurement	Time of Measurement	Day Relative to Inoculum	Day Relative to Artesunate	Assay	Result (copies of transcripts/500 µL packed RBCs)
R206	K13	Admission	8 Hrs Post-dose	28-FEB-2018	21:28	9	1	Replicate 1	682
								Replicate 2	1526
		Admission 3	72 Hrs Post-dose	03-MAR-2018	13:39	12	4	Replicate 1	1862
								Replicate 2	1144
		Safety Monitoring		06-MAR-2018	08:31	15	7	Replicate 1	7708
								Replicate 2	19694
				07-MAR-2018	09:34	16	8	Replicate 1	175500
								Replicate 2	236590
				08-MAR-2018	08:37	17	9	Replicate 1	1589798
								Replicate 2	1183602
				09-MAR-2018	08:58	18	10	Replicate 1	273698
								Replicate 2	217576
				14-MAR-2018	10:05	23	15	Replicate 1	2332600
								Replicate 2	2133400
				16-MAR-2018	08:01	25	17	Replicate 1	2104506
								Replicate 2	2166734

Notes: Day relative to inoculum is the number of days relative to day of administration of the inoculum, where the day of administration = 0.
The inoculum was administered on Study Day 0 for K13-infected subjects and Study Day 1 for 3D7-infected subjects.
Day relative to IP is the number of days relative to day of administration of Artesunate, where the day of dose = 1.
qRT-PCR = quantitative reverse transcription polymerase chain reaction; IP = Artesunate; ND = Not Detected; RBCs: red blood cells.
Method of analysis for all samples was Quantitative (RNAprotect Cell Reagent with Rneasy Plus Mini kit).

Listing 16.2.6.5
qRT-PCR pfs25 Retrospective

Randomisation Number	Challenge Agent	Visit	Timepoint	Date of Measurement	Time of Measurement	Day Relative to Inoculum	Day Relative to Artesunate	Assay	Result (copies of transcripts/500 µL packed RBCs)
R207	K13	Admission	Pre-dose	28-FEB-2018	12:00	9	1	Replicate 1	466
								Replicate 2	296
			8 Hrs Post-dose	28-FEB-2018	20:58	9	1	Replicate 1	348
								Replicate 2	142
		Admission 3	72 Hrs Post-dose	03-MAR-2018	13:00	12	4	Replicate 1	172
								Replicate 2	148
		Safety Monitoring		06-MAR-2018	09:52	15	7	Replicate 1	ND
								Replicate 2	ND
				14-MAR-2018	09:10	23	15	Replicate 1	68600
								Replicate 2	63800
				16-MAR-2018	09:26	25	17	Replicate 1	16946
								Replicate 2	16414
R208	3D7	Malaria Monitoring		25-FEB-2018	07:21	5	-3	Replicate 1	ND
								Replicate 2	ND
		Admission	Pre-dose	28-FEB-2018	11:13	8	1	Replicate 1	154
								Replicate 2	218

Notes: Day relative to inoculum is the number of days relative to day of administration of the inoculum, where the day of administration = 0.
The inoculum was administered on Study Day 0 for K13-infected subjects and Study Day 1 for 3D7-infected subjects.
Day relative to IP is the number of days relative to day of administration of Artesunate, where the day of dose = 1.
qRT-PCR = quantitative reverse transcription polymerase chain reaction; IP = Artesunate; ND = Not Detected; RBCs: red blood cells.
Method of analysis for all samples was Quantitative (RNAprotect Cell Reagent with Rneasy Plus Mini kit).

Listing 16.2.6.5
qRT-PCR pfs25 Retrospective

Randomisation Number	Challenge Agent	Visit	Timepoint	Date of Measurement	Time of Measurement	Day Relative to Inoculum	Day Relative to Artesunate	Assay	Result (copies of transcripts/500 µL packed RBCs)
R208	3D7	Admission	8 Hrs Post-dose	28-FEB-2018	19:35	8	1	Replicate 1	ND
								Replicate 2	144
		Admission 2	48 Hrs Post-dose	02-MAR-2018	11:37	10	3	Replicate 1	ND
								Replicate 2	ND
		Admission 3	72 Hrs Post-dose	03-MAR-2018	11:36	11	4	Replicate 1	ND
								Replicate 2	ND
		Safety Monitoring		04-MAR-2018	09:41	12	5	Replicate 1	ND
								Replicate 2	ND
				05-MAR-2018	07:07	13	6	Replicate 1	ND
								Replicate 2	ND
				07-MAR-2018	07:14	15	8	Replicate 1	102
								Replicate 2	ND
				08-MAR-2018	07:10	16	9	Replicate 1	ND
								Replicate 2	ND
				09-MAR-2018	07:19	17	10	Replicate 1	70
								Replicate 2	270

Notes: Day relative to inoculum is the number of days relative to day of administration of the inoculum, where the day of administration = 0.
The inoculum was administered on Study Day 0 for K13-infected subjects and Study Day 1 for 3D7-infected subjects.
Day relative to IP is the number of days relative to day of administration of Artesunate, where the day of dose = 1.
qRT-PCR = quantitative reverse transcription polymerase chain reaction; IP = Artesunate; ND = Not Detected; RBCs: red blood cells.
Method of analysis for all samples was Quantitative (RNAprotect Cell Reagent with Rneasy Plus Mini kit).

Listing 16.2.6.5
qRT-PCR pfs25 Retrospective

Randomisation Number	Challenge Agent	Visit	Timepoint	Date of Measurement	Time of Measurement	Day Relative to Inoculum	Day Relative to Artesunate	Assay	Result (copies of transcripts/500 µL packed RBCs)
R208	3D7	Safety Monitoring		14-MAR-2018	07:14	22	15	Replicate 1	15600
								Replicate 2	17200
				16-MAR-2018	07:11	24	17	Replicate 1	28374
								Replicate 2	23844
R209	3D7	Malaria Monitoring		25-FEB-2018	07:45	5	-3	Replicate 1	ND
								Replicate 2	ND
		Admission	Pre-dose	28-FEB-2018	14:42	8	1	Replicate 1	626
								Replicate 2	730
			8 Hrs Post-dose	28-FEB-2018	23:57	8	1	Replicate 1	216
								Replicate 2	108
		Admission 2	48 Hrs Post-dose	02-MAR-2018	15:48	10	3	Replicate 1	ND
								Replicate 2	ND
		Admission 3	72 Hrs Post-dose	03-MAR-2018	15:47	11	4	Replicate 1	ND
								Replicate 2	ND
		Safety Monitoring		04-MAR-2018	08:08	12	5	Replicate 1	ND
								Replicate 2	ND

Notes: Day relative to inoculum is the number of days relative to day of administration of the inoculum, where the day of administration = 0.
The inoculum was administered on Study Day 0 for K13-infected subjects and Study Day 1 for 3D7-infected subjects.
Day relative to IP is the number of days relative to day of administration of Artesunate, where the day of dose = 1.
qRT-PCR = quantitative reverse transcription polymerase chain reaction; IP = Artesunate; ND = Not Detected; RBCs: red blood cells.
Method of analysis for all samples was Quantitative (RNAprotect Cell Reagent with Rneasy Plus Mini kit).

Listing 16.2.6.5
qRT-PCR pfs25 Retrospective

Randomisation Number	Challenge Agent	Visit	Timepoint	Date of Measurement	Time of Measurement	Day Relative to Inoculum	Day Relative to Artesunate	Assay	Result (copies of transcripts/500 µL packed RBCs)
R209	3D7	Safety Monitoring		05-MAR-2018	07:41	13	6	Replicate 1	ND
								Replicate 2	ND
				07-MAR-2018	07:48	15	8	Replicate 1	ND
								Replicate 2	ND
				08-MAR-2018	08:42	16	9	Replicate 1	356
								Replicate 2	700
				09-MAR-2018	08:34	17	10	Replicate 1	ND
								Replicate 2	ND
				12-MAR-2018	08:12	20	13	Replicate 1	868
								Replicate 2	736
				14-MAR-2018	07:40	22	15	Replicate 1	96000
								Replicate 2	92400
				16-MAR-2018	07:27	24	17	Replicate 1	114706
								Replicate 2	93370
R210	3D7	Malaria Monitoring		25-FEB-2018	07:25	5	-3	Replicate 1	ND
								Replicate 2	ND

Notes: Day relative to inoculum is the number of days relative to day of administration of the inoculum, where the day of administration = 0.
The inoculum was administered on Study Day 0 for K13-infected subjects and Study Day 1 for 3D7-infected subjects.
Day relative to IP is the number of days relative to day of administration of Artesunate, where the day of dose = 1.
qRT-PCR = quantitative reverse transcription polymerase chain reaction; IP = Artesunate; ND = Not Detected; RBCs: red blood cells.
Method of analysis for all samples was Quantitative (RNAprotect Cell Reagent with Rneasy Plus Mini kit).

Listing 16.2.6.5
qRT-PCR pfs25 Retrospective

Randomisation Number	Challenge Agent	Visit	Timepoint	Date of Measurement	Time of Measurement	Day Relative to Inoculum	Day Relative to Artesunate	Assay	Result (copies of transcripts/500 µL packed RBCs)
R210	3D7	Admission	Pre-dose	28-FEB-2018	11:21	8	1	Replicate 1	318
								Replicate 2	322
			8 Hrs Post-dose	28-FEB-2018	19:45	8	1	Replicate 1	314
								Replicate 2	258
		Admission 2	48 Hrs Post-dose	02-MAR-2018	11:58	10	3	Replicate 1	ND
								Replicate 2	ND
		Admission 3	72 Hrs Post-dose	03-MAR-2018	12:01	11	4	Replicate 1	ND
								Replicate 2	ND
		Safety Monitoring		04-MAR-2018	07:10	12	5	Replicate 1	ND
								Replicate 2	ND
				05-MAR-2018	07:40	13	6	Replicate 1	ND
								Replicate 2	ND
				07-MAR-2018	07:26	15	8	Replicate 1	ND
								Replicate 2	34
				08-MAR-2018	07:40	16	9	Replicate 1	5034
								Replicate 2	3150

Notes: Day relative to inoculum is the number of days relative to day of administration of the inoculum, where the day of administration = 0.
The inoculum was administered on Study Day 0 for K13-infected subjects and Study Day 1 for 3D7-infected subjects.
Day relative to IP is the number of days relative to day of administration of Artesunate, where the day of dose = 1.
qRT-PCR = quantitative reverse transcription polymerase chain reaction; IP = Artesunate; ND = Not Detected; RBCs: red blood cells.
Method of analysis for all samples was Quantitative (RNAprotect Cell Reagent with Rneasy Plus Mini kit).

Listing 16.2.6.5
qRT-PCR pfs25 Retrospective

Randomisation Number	Challenge Agent	Visit	Timepoint	Date of Measurement	Time of Measurement	Day Relative to Inoculum	Day Relative to Artesunate	Assay	Result (copies of transcripts/500 µL packed RBCs)
R210	3D7	Safety Monitoring		09-MAR-2018	09:45	17	10	Replicate 1	4120
								Replicate 2	1962
				14-MAR-2018	07:17	22	15	Replicate 1	76600
								Replicate 2	134200
				16-MAR-2018	07:38	24	17	Replicate 1	149784
								Replicate 2	139514
R302	K13	Malaria Monitoring		10-SEP-2018	07:26	6	-3	Replicate 1	ND
								Replicate 2	ND
		Admission	Pre-dose	13-SEP-2018	08:57	9	1	Replicate 1	1472
								Replicate 2	650
			8 Hrs Post-dose	13-SEP-2018	17:06	9	1	Replicate 1	1584
								Replicate 2	528
		Admission 2	48 Hrs Post-dose	15-SEP-2018	09:06	11	3	Replicate 1	7908
								Replicate 2	4732
		Admission 3	72 Hrs Post-dose	16-SEP-2018	09:05	12	4	Replicate 1	292
								Replicate 2	84

Notes: Day relative to inoculum is the number of days relative to day of administration of the inoculum, where the day of administration = 0.
The inoculum was administered on Study Day 0 for K13-infected subjects and Study Day 1 for 3D7-infected subjects.
Day relative to IP is the number of days relative to day of administration of Artesunate, where the day of dose = 1.
qRT-PCR = quantitative reverse transcription polymerase chain reaction; IP = Artesunate; ND = Not Detected; RBCs: red blood cells.
Method of analysis for all samples was Quantitative (RNAprotect Cell Reagent with Rneasy Plus Mini kit).

Listing 16.2.6.5
qRT-PCR pfs25 Retrospective

Randomisation Number	Challenge Agent	Visit	Timepoint	Date of Measurement	Time of Measurement	Day Relative to Inoculum	Day Relative to Artesunate	Assay	Result (copies of transcripts/500 µL packed RBCs)
R302	K13	Safety Monitoring		18-SEP-2018	08:04	14	6	Replicate 1	ND
								Replicate 2	ND
				20-SEP-2018	07:25	16	8	Replicate 1	9274
								Replicate 2	10186
				22-SEP-2018	07:56	18	10	Replicate 1	40178
								Replicate 2	36782
				24-SEP-2018	07:22	20	12	Replicate 1	300886
								Replicate 2	278222
				25-SEP-2018	08:13	21	13	Replicate 1	257030
								Replicate 2	377466
				27-SEP-2018	07:19	23	15	Replicate 1	120818
								Replicate 2	145746
				28-SEP-2018	08:22	24	16	Replicate 1	160664
								Replicate 2	181184
		End of Study		02-OCT-2018	08:19	28	20	Replicate 1	56584
								Replicate 2	66480

Notes: Day relative to inoculum is the number of days relative to day of administration of the inoculum, where the day of administration = 0.
The inoculum was administered on Study Day 0 for K13-infected subjects and Study Day 1 for 3D7-infected subjects.
Day relative to IP is the number of days relative to day of administration of Artesunate, where the day of dose = 1.
qRT-PCR = quantitative reverse transcription polymerase chain reaction; IP = Artesunate; ND = Not Detected; RBCs: red blood cells.
Method of analysis for all samples was Quantitative (RNAprotect Cell Reagent with Rneasy Plus Mini kit).

Listing 16.2.6.5
qRT-PCR pfs25 Retrospective

Randomisation Number	Challenge Agent	Visit	Timepoint	Date of Measurement	Time of Measurement	Day Relative to Inoculum	Day Relative to Artesunate	Assay	Result (copies of transcripts/500 µL packed RBCs)
R302	K13	Unscheduled		05-OCT-2018	07:20	31	23	Replicate 1	44940
								Replicate 2	39814
				11-OCT-2018	07:17	37	29	Replicate 1	ND
								Replicate 2	ND
R303	K13	Inoculum		04-SEP-2018	09:53	0	-9	Replicate 1	ND
								Replicate 2	ND
		Admission	Pre-dose	13-SEP-2018	11:17	9	1	Replicate 1	3048
								Replicate 2	4024
			8 Hrs Post-dose	13-SEP-2018	19:18	9	1	Replicate 1	2220
								Replicate 2	3690
		Admission 2	48 Hrs Post-dose	15-SEP-2018	11:18	11	3	Replicate 1	244
								Replicate 2	566
		Admission 3	72 Hrs Post-dose	16-SEP-2018	11:18	12	4	Replicate 1	1580
								Replicate 2	1968
		Safety Monitoring		18-SEP-2018	07:29	14	6	Replicate 1	232
								Replicate 2	150

Notes: Day relative to inoculum is the number of days relative to day of administration of the inoculum, where the day of administration = 0.
The inoculum was administered on Study Day 0 for K13-infected subjects and Study Day 1 for 3D7-infected subjects.
Day relative to IP is the number of days relative to day of administration of Artesunate, where the day of dose = 1.
qRT-PCR = quantitative reverse transcription polymerase chain reaction; IP = Artesunate; ND = Not Detected; RBCs: red blood cells.
Method of analysis for all samples was Quantitative (RNAprotect Cell Reagent with Rneasy Plus Mini kit).

Listing 16.2.6.5
qRT-PCR pfs25 Retrospective

Randomisation Number	Challenge Agent	Visit	Timepoint	Date of Measurement	Time of Measurement	Day Relative to Inoculum	Day Relative to Artesunate	Assay	Result (copies of transcripts/500 µL packed RBCs)
R303	K13	Safety Monitoring		20-SEP-2018	08:16	16	8	Replicate 1	483118
								Replicate 2	619468
				22-SEP-2018	08:40	18	10	Replicate 1	2114110
								Replicate 2	1855982
				24-SEP-2018	07:01	20	12	Replicate 1	2552654
								Replicate 2	2708310
				25-SEP-2018	08:20	21	13	Replicate 1	3687792
								Replicate 2	2773198
				27-SEP-2018	07:00	23	15	Replicate 1	891684
								Replicate 2	1103454
				28-SEP-2018	08:19	24	16	Replicate 1	1692896
								Replicate 2	2110262
		End of Study		02-OCT-2018	09:05	28	20	Replicate 1	616092
								Replicate 2	950376
		Unscheduled		05-OCT-2018	07:01	31	23	Replicate 1	546314
								Replicate 2	549308

Notes: Day relative to inoculum is the number of days relative to day of administration of the inoculum, where the day of administration = 0.
The inoculum was administered on Study Day 0 for K13-infected subjects and Study Day 1 for 3D7-infected subjects.
Day relative to IP is the number of days relative to day of administration of Artesunate, where the day of dose = 1.
qRT-PCR = quantitative reverse transcription polymerase chain reaction; IP = Artesunate; ND = Not Detected; RBCs: red blood cells.
Method of analysis for all samples was Quantitative (RNAprotect Cell Reagent with Rneasy Plus Mini kit).

Listing 16.2.6.5
qRT-PCR pfs25 Retrospective

Randomisation Number	Challenge Agent	Visit	Timepoint	Date of Measurement	Time of Measurement	Day Relative to Inoculum	Day Relative to Artesunate	Assay	Result (copies of transcripts/500 µL packed RBCs)
R303	K13	Unscheduled		11-OCT-2018	06:45	37	29	Replicate 1	13972
								Replicate 2	18714
				18-OCT-2018	11:20	44	36	Replicate 1	ND
								Replicate 2	ND
R304	K13	Inoculum		04-SEP-2018	10:01	0	-9	Replicate 1	ND
								Replicate 2	ND
		Admission	Pre-dose	13-SEP-2018	09:16	9	1	Replicate 1	858
								Replicate 2	1184
			8 Hrs Post-dose	13-SEP-2018	17:23	9	1	Replicate 1	810
								Replicate 2	ND
		Admission 2	48 Hrs Post-dose	15-SEP-2018	09:22	11	3	Replicate 1	292
								Replicate 2	ND
		Admission 3	72 Hrs Post-dose	16-SEP-2018	09:22	12	4	Replicate 1	1010
								Replicate 2	706
		Safety Monitoring		18-SEP-2018	08:36	14	6	Replicate 1	7626
								Replicate 2	12674

Notes: Day relative to inoculum is the number of days relative to day of administration of the inoculum, where the day of administration = 0.
The inoculum was administered on Study Day 0 for K13-infected subjects and Study Day 1 for 3D7-infected subjects.
Day relative to IP is the number of days relative to day of administration of Artesunate, where the day of dose = 1.
qRT-PCR = quantitative reverse transcription polymerase chain reaction; IP = Artesunate; ND = Not Detected; RBCs: red blood cells.
Method of analysis for all samples was Quantitative (RNAprotect Cell Reagent with Rneasy Plus Mini kit).

Listing 16.2.6.5
qRT-PCR pfs25 Retrospective

Randomisation Number	Challenge Agent	Visit	Timepoint	Date of Measurement	Time of Measurement	Day Relative to Inoculum	Day Relative to Artesunate	Assay	Result (copies of transcripts/500 µL packed RBCs)
R304	K13	Safety Monitoring		20-SEP-2018	08:09	16	8	Replicate 1	182674
								Replicate 2	178400
				22-SEP-2018	09:25	18	10	Replicate 1	1012006
								Replicate 2	1132982
				24-SEP-2018	07:58	20	12	Replicate 1	2021850
								Replicate 2	1542734
				25-SEP-2018	08:11	21	13	Replicate 1	952976
								Replicate 2	540376
				27-SEP-2018	08:20	23	15	Replicate 1	963446
								Replicate 2	963446
				28-SEP-2018	08:17	24	16	Replicate 1	761716
								Replicate 2	922240
		End of Study		02-OCT-2018	08:46	28	20	Replicate 1	308656
								Replicate 2	378154
		Unscheduled		05-OCT-2018	08:20	31	23	Replicate 1	107428
								Replicate 2	143116

Notes: Day relative to inoculum is the number of days relative to day of administration of the inoculum, where the day of administration = 0.
The inoculum was administered on Study Day 0 for K13-infected subjects and Study Day 1 for 3D7-infected subjects.
Day relative to IP is the number of days relative to day of administration of Artesunate, where the day of dose = 1.
qRT-PCR = quantitative reverse transcription polymerase chain reaction; IP = Artesunate; ND = Not Detected; RBCs: red blood cells.
Method of analysis for all samples was Quantitative (RNAprotect Cell Reagent with Rneasy Plus Mini kit).

Listing 16.2.6.5
qRT-PCR pfs25 Retrospective

Randomisation Number	Challenge Agent	Visit	Timepoint	Date of Measurement	Time of Measurement	Day Relative to Inoculum	Day Relative to Artesunate	Assay	Result (copies of transcripts/500 µL packed RBCs)
R304	K13	Unscheduled		11-OCT-2018	09:27	37	29	Replicate 1	14398
								Replicate 2	15486
R305	3D7	Malaria Monitoring		10-SEP-2018	07:30	5	-3	Replicate 1	ND
								Replicate 2	ND
		Admission	Pre-dose	13-SEP-2018	09:29	8	1	Replicate 1	254
								Replicate 2	1074
			8 Hrs Post-dose	13-SEP-2018	17:35	8	1	Replicate 1	136
								Replicate 2	576
		Admission 1	24 Hrs Post-dose	14-SEP-2018	09:37	9	2	Replicate 1	256
								Replicate 2	ND
			36 Hrs Post-dose	14-SEP-2018	21:36	9	2	Replicate 1	ND
								Replicate 2	ND
		Admission 2	48 Hrs Post-dose	15-SEP-2018	09:35	10	3	Replicate 1	ND
								Replicate 2	ND
		Admission 3	72 Hrs Post-dose	16-SEP-2018	09:36	11	4	Replicate 1	ND
								Replicate 2	ND

Notes: Day relative to inoculum is the number of days relative to day of administration of the inoculum, where the day of administration = 0.
The inoculum was administered on Study Day 0 for K13-infected subjects and Study Day 1 for 3D7-infected subjects.
Day relative to IP is the number of days relative to day of administration of Artesunate, where the day of dose = 1.
qRT-PCR = quantitative reverse transcription polymerase chain reaction; IP = Artesunate; ND = Not Detected; RBCs: red blood cells.
Method of analysis for all samples was Quantitative (RNAprotect Cell Reagent with Rneasy Plus Mini kit).

Listing 16.2.6.5
qRT-PCR pfs25 Retrospective

Randomisation Number	Challenge Agent	Visit	Timepoint	Date of Measurement	Time of Measurement	Day Relative to Inoculum	Day Relative to Artesunate	Assay	Result (copies of transcripts/500 µL packed RBCs)
R305	3D7	Safety Monitoring		18-SEP-2018	07:16	13	6	Replicate 1	ND
								Replicate 2	ND
				20-SEP-2018	07:23	15	8	Replicate 1	26022
								Replicate 2	18078
				22-SEP-2018	07:53	17	10	Replicate 1	14424
								Replicate 2	17242
				24-SEP-2018	07:04	19	12	Replicate 1	200820
								Replicate 2	220770
				25-SEP-2018	08:19	20	13	Replicate 1	832818
								Replicate 2	834336
				28-SEP-2018	07:23	23	16	Replicate 1	1270730
								Replicate 2	1209756
		End of Study		02-OCT-2018	07:50	27	20	Replicate 1	2116036
								Replicate 2	2313556
		Unscheduled		10-OCT-2018	08:12	35	28	Replicate 1	556862
								Replicate 2	580684

Notes: Day relative to inoculum is the number of days relative to day of administration of the inoculum, where the day of administration = 0.
The inoculum was administered on Study Day 0 for K13-infected subjects and Study Day 1 for 3D7-infected subjects.
Day relative to IP is the number of days relative to day of administration of Artesunate, where the day of dose = 1.
qRT-PCR = quantitative reverse transcription polymerase chain reaction; IP = Artesunate; ND = Not Detected; RBCs: red blood cells.
Method of analysis for all samples was Quantitative (RNAprotect Cell Reagent with Rneasy Plus Mini kit).

Listing 16.2.6.5
qRT-PCR pfs25 Retrospective

Randomisation Number	Challenge Agent	Visit	Timepoint	Date of Measurement	Time of Measurement	Day Relative to Inoculum	Day Relative to Artesunate	Assay	Result (copies of transcripts/500 µL packed RBCs)
R305	3D7	Unscheduled		17-OCT-2018	09:41	42	35	Replicate 1	ND
								Replicate 2	ND
R306	3D7	Malaria Monitoring		10-SEP-2018	07:03	5	-3	Replicate 1	ND
								Replicate 2	ND
		Admission	Pre-dose	13-SEP-2018	09:41	8	1	Replicate 1	400
								Replicate 2	ND
			8 Hrs Post-dose	13-SEP-2018	17:45	8	1	Replicate 1	200
								Replicate 2	400
		Admission 1	24 Hrs Post-dose	14-SEP-2018	09:46	9	2	Replicate 1	ND
								Replicate 2	ND
			36 Hrs Post-dose	14-SEP-2018	21:45	9	2	Replicate 1	ND
								Replicate 2	ND
		Admission 2	48 Hrs Post-dose	15-SEP-2018	09:45	10	3	Replicate 1	ND
								Replicate 2	ND
		Admission 3	72 Hrs Post-dose	16-SEP-2018	09:45	11	4	Replicate 1	ND
								Replicate 2	ND

Notes: Day relative to inoculum is the number of days relative to day of administration of the inoculum, where the day of administration = 0.
The inoculum was administered on Study Day 0 for K13-infected subjects and Study Day 1 for 3D7-infected subjects.
Day relative to IP is the number of days relative to day of administration of Artesunate, where the day of dose = 1.
qRT-PCR = quantitative reverse transcription polymerase chain reaction; IP = Artesunate; ND = Not Detected; RBCs: red blood cells.
Method of analysis for all samples was Quantitative (RNAprotect Cell Reagent with Rneasy Plus Mini kit).

Listing 16.2.6.5
qRT-PCR pfs25 Retrospective

Randomisation Number	Challenge Agent	Visit	Timepoint	Date of Measurement	Time of Measurement	Day Relative to Inoculum	Day Relative to Artesunate	Assay	Result (copies of transcripts/500 µL packed RBCs)
R306	3D7	Safety Monitoring		18-SEP-2018	08:04	13	6	Replicate 1	ND
								Replicate 2	ND
				20-SEP-2018	07:01	15	8	Replicate 1	200
								Replicate 2	ND
				22-SEP-2018	07:34	17	10	Replicate 1	14200
								Replicate 2	18800
				24-SEP-2018	07:01	19	12	Replicate 1	33800
								Replicate 2	35400
				25-SEP-2018	07:30	20	13	Replicate 1	102000
								Replicate 2	83000
				28-SEP-2018	07:11	23	16	Replicate 1	264800
								Replicate 2	255000
		End of Study		02-OCT-2018	08:20	27	20	Replicate 1	250800
								Replicate 2	207000
		Unscheduled		08-OCT-2018	07:07	33	26	Replicate 1	130800
								Replicate 2	138200

Notes: Day relative to inoculum is the number of days relative to day of administration of the inoculum, where the day of administration = 0.
The inoculum was administered on Study Day 0 for K13-infected subjects and Study Day 1 for 3D7-infected subjects.
Day relative to IP is the number of days relative to day of administration of Artesunate, where the day of dose = 1.
qRT-PCR = quantitative reverse transcription polymerase chain reaction; IP = Artesunate; ND = Not Detected; RBCs: red blood cells.
Method of analysis for all samples was Quantitative (RNAprotect Cell Reagent with Rneasy Plus Mini kit).

Listing 16.2.6.5
qRT-PCR pfs25 Retrospective

Randomisation Number	Challenge Agent	Visit	Timepoint	Date of Measurement	Time of Measurement	Day Relative to Inoculum	Day Relative to Artesunate	Assay	Result (copies of transcripts/500 µL packed RBCs)
R307	3D7	Inoculum		05-SEP-2018	10:59	0	-8	Replicate 1	ND
								Replicate 2	ND
		Malaria Monitoring		10-SEP-2018	08:22	5	-3	Replicate 1	ND
								Replicate 2	ND
		Admission	Pre-dose	13-SEP-2018	09:51	8	1	Replicate 1	1330
								Replicate 2	1272
			8 Hrs Post-dose	13-SEP-2018	17:55	8	1	Replicate 1	1926
								Replicate 2	1138
		Admission 1	24 Hrs Post-dose	14-SEP-2018	09:55	9	2	Replicate 1	188
								Replicate 2	ND
			36 Hrs Post-dose	14-SEP-2018	21:56	9	2	Replicate 1	ND
								Replicate 2	ND
		Admission 2	48 Hrs Post-dose	15-SEP-2018	09:54	10	3	Replicate 1	ND
								Replicate 2	ND
		Admission 3	72 Hrs Post-dose	16-SEP-2018	09:55	11	4	Replicate 1	ND
								Replicate 2	ND

Notes: Day relative to inoculum is the number of days relative to day of administration of the inoculum, where the day of administration = 0.
The inoculum was administered on Study Day 0 for K13-infected subjects and Study Day 1 for 3D7-infected subjects.
Day relative to IP is the number of days relative to day of administration of Artesunate, where the day of dose = 1.
qRT-PCR = quantitative reverse transcription polymerase chain reaction; IP = Artesunate; ND = Not Detected; RBCs: red blood cells.
Method of analysis for all samples was Quantitative (RNAprotect Cell Reagent with Rneasy Plus Mini kit).

Listing 16.2.6.5
qRT-PCR pfs25 Retrospective

Randomisation Number	Challenge Agent	Visit	Timepoint	Date of Measurement	Time of Measurement	Day Relative to Inoculum	Day Relative to Artesunate	Assay	Result (copies of transcripts/500 µL packed RBCs)
R307	3D7	Safety Monitoring		18-SEP-2018	08:08	13	6	Replicate 1	ND
								Replicate 2	ND
				20-SEP-2018	08:01	15	8	Replicate 1	186
								Replicate 2	214
				22-SEP-2018	08:38	17	10	Replicate 1	192
								Replicate 2	652
				24-SEP-2018	07:38	19	12	Replicate 1	53430
								Replicate 2	49630
				25-SEP-2018	07:36	20	13	Replicate 1	153372
								Replicate 2	99154
				28-SEP-2018	07:40	23	16	Replicate 1	260092
								Replicate 2	234018
		End of Study		02-OCT-2018	07:21	27	20	Replicate 1	211906
								Replicate 2	320108
		Unscheduled		05-OCT-2018	07:55	30	23	Replicate 1	191708
								Replicate 2	247160

Notes: Day relative to inoculum is the number of days relative to day of administration of the inoculum, where the day of administration = 0.
The inoculum was administered on Study Day 0 for K13-infected subjects and Study Day 1 for 3D7-infected subjects.
Day relative to IP is the number of days relative to day of administration of Artesunate, where the day of dose = 1.
qRT-PCR = quantitative reverse transcription polymerase chain reaction; IP = Artesunate; ND = Not Detected; RBCs: red blood cells.
Method of analysis for all samples was Quantitative (RNAprotect Cell Reagent with Rneasy Plus Mini kit).

Listing 16.2.6.5
qRT-PCR pfs25 Retrospective

Randomisation Number	Challenge Agent	Visit	Timepoint	Date of Measurement	Time of Measurement	Day Relative to Inoculum	Day Relative to Artesunate	Assay	Result (copies of transcripts/500 µL packed RBCs)
R307	3D7	Unscheduled		11-OCT-2018	08:33	36	29	Replicate 1	11990
								Replicate 2	8254
				18-OCT-2018	07:56	43	36	Replicate 1	ND
								Replicate 2	ND

Notes: Day relative to inoculum is the number of days relative to day of administration of the inoculum, where the day of administration = 0.
The inoculum was administered on Study Day 0 for K13-infected subjects and Study Day 1 for 3D7-infected subjects.
Day relative to IP is the number of days relative to day of administration of Artesunate, where the day of dose = 1.
qRT-PCR = quantitative reverse transcription polymerase chain reaction; IP = Artesunate; ND = Not Detected; RBCs: red blood cells.
Method of analysis for all samples was Quantitative (RNAprotect Cell Reagent with Rneasy Plus Mini kit).

16.2.7 Adverse events

16.2.7.1 Adverse Events

Listing 16.2.7.1
Adverse Events

Rando Number	Challenge Agent	AE No	System Organ Class/ Preferred Term/ Adverse Event Verbatim	Onset Date/ Onset Time/ (d) End Date/ End Time/ (d)	TEAE/ SAE	Severity/ Action Taken/ Outcome	Treatment Directly Related to AE (Inoculum/ Artesunate/ Piperaquine/ Malarone/ Primacin/ Mosquito)	Time of onset Relative to Inoculum/ Time of onset Relative to Artesunate/ Time of onset Relative to PP	Duration (hours)	Dis
R103	K13	1	Nervous system disorders/ Lethargy/ Lethargy	19-NOV-2017/ 17:00/ (-4)/ 19-NOV-2017/ 19:00/ (-4)	No/ No	MILD/ NONE/ RECOVERED/ RESOLVED	Possible/ Unrelated/ Unrelated/ Unrelated/ Unrelated/ Unrelated	5.3 days post/ -3.7 days pre/ -6 days pre	2.00	No
		2	Blood and lymphatic system disorders/ Splenomegaly/ Splenomegaly	23-NOV-2017/ 09:16/ (1)/ 26-NOV-2017/ 09:03/ (4)	No/ No	MILD/ NONE/ RECOVERED/ RESOLVED	Probable/ Unrelated/ Unrelated/ Unrelated/ Unlikely/ Unrelated	8.9 days post/ -0.1 days pre/ -2.4 days pre	71.78	No
		3	General disorders and administration site conditions/ Fatigue/ Fatigue	23-NOV-2017/ 18:30/ (1)/ 24-NOV-2017/ 10:00/ (2)	Yes/ No	MILD/ NONE/ RECOVERED/ RESOLVED	Probable/ Unrelated/ Unrelated/ Unrelated/ Unrelated/ Unrelated	9.3 days post/ 0.3 days post/ -2 days pre	15.50	No
		4	Nervous system disorders/ Headache/ Headache	23-NOV-2017/ 20:15/ (1)/ 24-NOV-2017/ 11:50/ (2)	Yes/ No	MILD/ MEDICATION: ibuprofen/ RECOVERED/ RESOLVED	Probable/ Unrelated/ Unrelated/ Unrelated/ Unrelated/ Unrelated	9.4 days post/ 0.4 days post/ -1.9 days pre	15.58	No

Notes: Adverse events were coded using the Medical Dictionary for Regulatory Activities (MedDRA) Version 20.1.
A treatment-emergent adverse event is defined as an AE that occurred or worsened following first administration of study drug.
Rando = Randomisation; PP = Piperaquine phosphate; (d) = Day from IP dose.
AE No. = Adverse Event Number; TEAE = A treatment-emergent adverse event; SAE = Serious adverse event; UN = Unknown; Dis = Discontinuation

Listing 16.2.7.1
Adverse Events

Rando Number	Challenge Agent	AE No	System Organ Class/ Preferred Term/ Adverse Event Verbatim	Onset Date/ Onset Time/ (d) End Date/ End Time/ (d)	TEAE/ SAE	Severity/ Action Taken/ Outcome	Treatment Directly Related to AE (Inoculum/ Artesunate/ Piperaquine/ Malarone/ Primacin/ Mosquito)	Time of onset Relative to Inoculum/ Time of onset Relative to Artesunate/ Time of onset Relative to PP	Duration (hours)	Dis
R103	K13	5	Musculoskeletal and connective tissue disorders/ Myalgia/ Myalgia	23-NOV-2017/ 21:01/ (1)/ 24-NOV-2017/ 11:30/ (2)	Yes/ No	MILD/ NONE/ RECOVERED/ RESOLVED	Probable/ Unrelated/ Unrelated/ Unrelated/ Unrelated/ Unrelated	9.4 days post/ 0.4 days post/ -1.9 days pre	14.48	No
		6	Gastrointestinal disorders/ Abdominal tenderness/ Abdominal Tenderness	24-NOV-2017/ 08:00/ (2)/ 24-NOV-2017/ 13:30/ (2)	Yes/ No	MILD/ NONE/ RECOVERED/ RESOLVED	Probable/ Unrelated/ Unrelated/ Unrelated/ Unrelated/ Unrelated	9.9 days post/ 0.9 days post/ -1.4 days pre	5.50	No
		7	General disorders and administration site conditions/ Chills/ Chills	24-NOV-2017/ 12:00/ (2)/ 25-NOV-2017/ 07:00/ (3)	Yes/ No	MILD/ NONE/ RECOVERED/ RESOLVED	Probable/ Unrelated/ Unrelated/ Unrelated/ Unrelated/ Unrelated	10.1 days post/ 1 days post/ -1.3 days pre	19.00	No
		8	General disorders and administration site conditions/ Fatigue/ Fatigue	24-NOV-2017/ 18:00/ (2)/ 25-NOV-2017/ 07:00/ (3)	Yes/ No	MILD/ NONE/ RECOVERED/ RESOLVED	Probable/ Unrelated/ Unrelated/ Unrelated/ Unrelated/ Unrelated	10.3 days post/ 1.3 days post/ -1 days pre	13.00	No

Notes: Adverse events were coded using the Medical Dictionary for Regulatory Activities (MedDRA) Version 20.1.
A treatment-emergent adverse event is defined as an AE that occurred or worsened following first administration of study drug.
Rando = Randomisation; PP = Piperaquine phosphate; (d) = Day from IP dose.
AE No. = Adverse Event Number; TEAE = A treatment-emergent adverse event; SAE = Serious adverse event; UN = Unknown; Dis = Discontinuation

Listing 16.2.7.1
Adverse Events

Rando Number	Challenge Agent	AE No	System Organ Class/ Preferred Term/ Adverse Event Verbatim	Onset Date/ Onset Time/ (d) End Date/ End Time/ (d)	TEAE/ SAE	Severity/ Action Taken/ Outcome	Treatment Directly Related to AE (Inoculum/ Artesunate/ Piperaquine/ Malarone/ Primacin/ Mosquito)	Time of onset Relative to Inoculum/ Time of onset Relative to Artesunate/ Time of onset Relative to PP	Duration (hours)	Dis
R103	K13	9	General disorders and administration site conditions/ Malaise/ Malaise	24-NOV-2017/ 18:00/ (2)/ 25-NOV-2017/ 07:00/ (3)	Yes/ No	MILD/ NONE/ RECOVERED/ RESOLVED	Probable/ Unrelated/ Unrelated/ Unrelated/ Unrelated/ Unrelated	10.3 days post/ 1.3 days post/ -1 days pre	13.00	No
		10	General disorders and administration site conditions/ Pyrexia/ Intermittent fever	24-NOV-2017/ 18:00/ (2)/ 26-NOV-2017/ 19:23/ (4)	Yes/ No	MILD/ NONE/ RECOVERED/ RESOLVED	Probable/ Unrelated/ Unrelated/ Unrelated/ Unrelated/ Unrelated	10.3 days post/ 1.3 days post/ -1 days pre	49.38	No
		11	Musculoskeletal and connective tissue disorders/ Myalgia/ Myalgia	24-NOV-2017/ 18:00/ (2)/ 26-NOV-2017/ 19:24/ (4)	Yes/ No	MILD/ MEDICATION: Ibuprofen, Panadol/ RECOVERED/ RESOLVED	Probable/ Unrelated/ Unrelated/ Unrelated/ Unrelated/ Unrelated	10.3 days post/ 1.3 days post/ -1 days pre	49.40	No
		12	Cardiac disorders/ Tachycardia/ tachycardia	24-NOV-2017/ 20:23/ (2)/ 24-NOV-2017/ 20:24/ (2)	Yes/ No	MILD/ NONE/ RECOVERED/ RESOLVED	Probable/ Unrelated/ Unrelated/ Unrelated/ Unrelated/ Unrelated	10.4 days post/ 1.4 days post/ -0.9 days pre	0.02	No

Notes: Adverse events were coded using the Medical Dictionary for Regulatory Activities (MedDRA) Version 20.1.
A treatment-emergent adverse event is defined as an AE that occurred or worsened following first administration of study drug.
Rando = Randomisation; PP = Piperaquine phosphate; (d) = Day from IP dose.
AE No. = Adverse Event Number; TEAE = A treatment-emergent adverse event; SAE = Serious adverse event; UN = Unknown; Dis = Discontinuation

Listing 16.2.7.1
Adverse Events

Rando Number	Challenge Agent	AE No	System Organ Class/ Preferred Term/ Adverse Event Verbatim	Onset Date/ Onset Time/ (d) End Date/ End Time/ (d)	TEAE/ SAE	Severity/ Action Taken/ Outcome	Treatment Directly Related to AE (Inoculum/ Artesunate/ Piperaquine/ Malarone/ Primacin/ Mosquito)	Time of onset Relative to Inoculum/ Time of onset Relative to Artesunate/ Time of onset Relative to PP	Duration (hours)	Dis
R103	K13	13	Nervous system disorders/ Headache/ Intermittent headache	25-NOV-2017/ 19:30/ (3)/ 26-NOV-2017/ 19:24/ (4)	Yes/ No	MILD/ MEDICATION: Ibuprofen/ RECOVERED/ RESOLVED	Probable/ Unrelated/ Unrelated/ Unrelated/ Unrelated/ Unrelated	11.4 days post/ 2.4 days post/ 0.1 days post	23.90	No
		14	Investigations/ Lymphocyte count decreased/ lymphocyctes count decreased	26-NOV-2017/ 10:53/ (4)/ 30-NOV-2017/ 10:09/ (8)	Yes/ No	SEVERE/ RECOVERED/ RESOLVED	Probable/ Unrelated/ Unrelated/ Unrelated/ Unrelated/ Unrelated	12 days post/ 3 days post/ 0.7 days post	95.27	No
		15	Nervous system disorders/ Dizziness/ dizziness	26-NOV-2017/ 22:00/ (4)/ 27-NOV-2017/ 08:45/ (5)	Yes/ No	MILD/ NONE/ RECOVERED/ RESOLVED	Probable/ Unrelated/ Unrelated/ Unrelated/ Unrelated/ Unrelated	12.5 days post/ 3.5 days post/ 1.2 days post	10.75	No
		16	General disorders and administration site conditions/ Pyrexia/ fever	26-NOV-2017/ 22:00/ (4)/ 27-NOV-2017/ 08:45/ (5)	Yes/ No	MILD/ NONE/ RECOVERED/ RESOLVED	Probable/ Unrelated/ Unrelated/ Unrelated/ Unrelated/ Unrelated	12.5 days post/ 3.5 days post/ 1.2 days post	10.75	No

Notes: Adverse events were coded using the Medical Dictionary for Regulatory Activities (MedDRA) Version 20.1.
A treatment-emergent adverse event is defined as an AE that occurred or worsened following first administration of study drug.
Rando = Randomisation; PP = Piperaquine phosphate; (d) = Day from IP dose.
AE No. = Adverse Event Number; TEAE = A treatment-emergent adverse event; SAE = Serious adverse event; UN = Unknown; Dis = Discontinuation

Listing 16.2.7.1
Adverse Events

Rando Number	Challenge Agent	AE No	System Organ Class/ Preferred Term/ Adverse Event Verbatim	Onset Date/ Onset Time/ (d) End Date/ End Time/ (d)	TEAE/ SAE	Severity/ Action Taken/ Outcome	Treatment Directly Related to AE (Inoculum/ Artesunate/ Piperaquine/ Malarone/ Primacin/ Mosquito)	Time of onset Relative to Inoculum/ Time of onset Relative to Artesunate/ Time of onset Relative to PP	Duration (hours)	Dis
R103	K13	17	Nervous system disorders/ Headache/ intermittent headache	27-NOV-2017/ 08:45/ (5)/ 29-NOV-2017/ 04:50/ (7)	Yes/ No	MILD/ MEDICATION: Ibuprofen/ RECOVERED/ RESOLVED	Probable/ Unrelated/ Unrelated/ Unrelated/ Unrelated/ Unrelated	12.9 days post/ 3.9 days post/ 1.6 days post	44.08	No
		18	Nervous system disorders/ Headache/ headache	01-DEC-2017/ 14:30/ (9)/ 02-DEC-2017/ 07:00/ (10)	Yes/ No	MILD/ MEDICATION: Ibuprofen/ RECOVERED/ RESOLVED	Probable/ Unrelated/ Unrelated/ Unrelated/ Unrelated/ Unrelated	17.2 days post/ 8.2 days post/ 5.9 days post	16.50	No
R104	K13	1	General disorders and administration site conditions/ Fatigue/ Intermittent fatigue	22-NOV-2017/ 06:00/ (-1)/ 23-NOV-2017/ 15:00/ (1)	No/ No	MILD/ NONE/ RECOVERED/ RESOLVED	Probable/ Unrelated/ Unrelated/ Unrelated/ Unrelated/ Unrelated	7.8 days post/ -1.2 days pre/ -3.5 days pre	33.00	No
		2	Musculoskeletal and connective tissue disorders/ Arthralgia/ Arthralgia	22-NOV-2017/ 09:00/ (-1)/ 22-NOV-2017/ 15:00/ (-1)	No/ No	MILD/ MEDICATION: Ibuprofen/ RECOVERED/ RESOLVED	Probable/ Unrelated/ Unrelated/ Unrelated/ Unrelated/ Unrelated	7.9 days post/ -1.1 days pre/ -3.4 days pre	6.00	No

Notes: Adverse events were coded using the Medical Dictionary for Regulatory Activities (MedDRA) Version 20.1.
A treatment-emergent adverse event is defined as an AE that occurred or worsened following first administration of study drug.
Rando = Randomisation; PP = Piperaquine phosphate; (d) = Day from IP dose.
AE No. = Adverse Event Number; TEAE = A treatment-emergent adverse event; SAE = Serious adverse event; UN = Unknown; Dis = Discontinuation

Listing 16.2.7.1
Adverse Events

Rando Number	Challenge Agent	AE No	System Organ Class/ Preferred Term/ Adverse Event Verbatim	Onset Date/ Onset Time/ (d) End Date/ End Time/ (d)	TEAE/ SAE	Severity/ Action Taken/ Outcome	Treatment Directly Related to AE (Inoculum/ Artesunate/ Piperaquine/ Malarone/ Primacin/ Mosquito)	Time of onset Relative to Inoculum/ Time of onset Relative to Artesunate/ Time of onset Relative to PP	Duration (hours)	Dis
R104	K13	3	Nervous system disorders/ Headache/ Headache	23-NOV-2017/ 06:00/ (1)/ 23-NOV-2017/ UN:UN/ (1)	No/ No	MILD/ MEDICATION: Ibuprofen/ RECOVERED/ RESOLVED	Probable/ Unrelated/ Unrelated/ Unrelated/ Unrelated/ Unrelated	8.8 days post/ -0.2 days pre/ -2.5 days pre		No
		4	Musculoskeletal and connective tissue disorders/ Arthralgia/ Arthralgia	23-NOV-2017/ 06:00/ (1)/ 24-NOV-2017/ 20:27/ (2)	No/ No	MILD/ MEDICATION: Ibuprofen/ RECOVERED/ RESOLVED	Probable/ Unrelated/ Unrelated/ Unrelated/ Unrelated/ Unrelated	8.8 days post/ -0.2 days pre/ -2.5 days pre	38.45	No
		5	Infections and infestations/ Upper respiratory tract infection/ Upper respiratory tract infection	23-NOV-2017/ 06:00/ (1)/ 30-NOV-2017/ UN:UN/ (8)	No/ No	MILD/ NONE/ RECOVERED/ RESOLVED	Unrelated/ Unrelated/ Unrelated/ Unrelated/ Unrelated/ Unrelated	8.8 days post/ -0.2 days pre/ -2.5 days pre		No
		6	General disorders and administration site conditions/ Chills/ Chills	23-NOV-2017/ 15:00/ (1)/ 24-NOV-2017/ 23:00/ (2)	Yes/ No	MILD/ NONE/ RECOVERED/ RESOLVED	Probable/ Unrelated/ Unrelated/ Unrelated/ Unrelated/ Unrelated	9.2 days post/ 0.2 days post/ -2.1 days pre	32.00	No

Notes: Adverse events were coded using the Medical Dictionary for Regulatory Activities (MedDRA) Version 20.1.
A treatment-emergent adverse event is defined as an AE that occurred or worsened following first administration of study drug.
Rando = Randomisation; PP = Piperaquine phosphate; (d) = Day from IP dose.
AE No. = Adverse Event Number; TEAE = A treatment-emergent adverse event; SAE = Serious adverse event; UN = Unknown; Dis = Discontinuation

Listing 16.2.7.1
Adverse Events

Rando Number	Challenge Agent	AE No	System Organ Class/ Preferred Term/ Adverse Event Verbatim	Onset Date/ Onset Time/ (d) End Date/ End Time/ (d)	TEAE/ SAE	Severity/ Action Taken/ Outcome	Treatment Directly Related to AE (Inoculum/ Artesunate/ Piperaquine/ Malarone/ Primacin/ Mosquito)	Time of onset Relative to Inoculum/ Time of onset Relative to Artesunate/ Time of onset Relative to PP	Duration (hours)	Dis
R104	K13	7	Metabolism and nutrition disorders/ Decreased appetite/ Anorexia	23-NOV-2017/ 18:35/ (1)/ 24-NOV-2017/ 08:15/ (2)	Yes/ No	MILD/ NONE/ RECOVERED/ RESOLVED	Probable/ Unrelated/ Unrelated/ Unrelated/ Unrelated/ Unrelated	9.3 days post/ 0.3 days post/ -2 days pre	13.67	No
		8	General disorders and administration site conditions/ Pyrexia/ Fever	23-NOV-2017/ 20:19/ (1)/ 23-NOV-2017/ 23:22/ (1)	Yes/ No	MILD/ MEDICATION: Ibuprofen/ RECOVERED/ RESOLVED	Probable/ Unrelated/ Unrelated/ Unrelated/ Unrelated/ Unrelated	9.4 days post/ 0.4 days post/ -1.9 days pre	3.05	No
		9	Cardiac disorders/ Tachycardia/ Tachycardia	23-NOV-2017/ 20:19/ (1)/ 24-NOV-2017/ 09:29/ (2)	Yes/ No	MILD/ NONE/ RECOVERED/ RESOLVED	Probable/ Unrelated/ Unrelated/ Unrelated/ Unrelated/ Unrelated	9.4 days post/ 0.4 days post/ -1.9 days pre	13.17	No
		10	Musculoskeletal and connective tissue disorders/ Myalgia/ Myalgia	24-NOV-2017/ 05:30/ (2)/ 24-NOV-2017/ 23:00/ (2)	Yes/ No	MILD/ MEDICATION: Ibuprofen, Paracetamol/ RECOVERED/ RESOLVED	Probable/ Unrelated/ Unrelated/ Unrelated/ Unrelated/ Unrelated	9.8 days post/ 0.8 days post/ -1.5 days pre	17.50	No

Notes: Adverse events were coded using the Medical Dictionary for Regulatory Activities (MedDRA) Version 20.1.
A treatment-emergent adverse event is defined as an AE that occurred or worsened following first administration of study drug.
Rando = Randomisation; PP = Piperaquine phosphate; (d) = Day from IP dose.
AE No. = Adverse Event Number; TEAE = A treatment-emergent adverse event; SAE = Serious adverse event; UN = Unknown; Dis = Discontinuation

Listing 16.2.7.1
Adverse Events

Rando Number	Challenge Agent	AE No	System Organ Class/ Preferred Term/ Adverse Event Verbatim	Onset Date/ Onset Time/ (d) End Date/ End Time/ (d)	TEAE/ SAE	Severity/ Action Taken/ Outcome	Treatment Directly Related to AE (Inoculum/ Artesunate/ Piperaquine/ Malarone/ Primacin/ Mosquito)	Time of onset Relative to Inoculum/ Time of onset Relative to Artesunate/ Time of onset Relative to PP	Duration (hours)	Dis
R104	K13	11	Nervous system disorders/ Headache/ Intermittent headache	24-NOV-2017/ 05:30/ (2)/ 25-NOV-2017/ 20:04/ (3)	Yes/ No	MILD/ MEDICATION: Ibuprofen, paracetamol/ RECOVERED/ RESOLVED	Probable/ Unrelated/ Unrelated/ Unrelated/ Unrelated/ Unrelated	9.8 days post/ 0.8 days post/ -1.5 days pre	38.57	No
		12	General disorders and administration site conditions/ Pyrexia/ Fever	24-NOV-2017/ 06:44/ (2)/ 24-NOV-2017/ 21:33/ (2)	Yes/ No	MILD/ MEDICATION: Ibuprofen/ RECOVERED/ RESOLVED	Probable/ Unrelated/ Unrelated/ Unrelated/ Unrelated/ Unrelated	9.8 days post/ 0.8 days post/ -1.5 days pre	14.82	No
		13	General disorders and administration site conditions/ Malaise/ Malaise	24-NOV-2017/ 19:00/ (2)/ 24-NOV-2017/ 23:00/ (2)	Yes/ No	MODERATE/ NONE/ RECOVERED/ RESOLVED	Probable/ Unrelated/ Unrelated/ Unrelated/ Unrelated/ Unrelated	10.3 days post/ 1.3 days post/ -1 days pre	4.00	No
		14	Musculoskeletal and connective tissue disorders/ Arthralgia/ Intermittent arthralgia	24-NOV-2017/ 20:27/ (2)/ 25-NOV-2017/ 09:19/ (3)	Yes/ No	MODERATE/ MEDICATION: Ibuprofen/ RECOVERED/ RESOLVED	Probable/ Unrelated/ Unrelated/ Unrelated/ Unrelated/ Unrelated	10.4 days post/ 1.4 days post/ -0.9 days pre	12.87	No

Notes: Adverse events were coded using the Medical Dictionary for Regulatory Activities (MedDRA) Version 20.1.
A treatment-emergent adverse event is defined as an AE that occurred or worsened following first administration of study drug.
Rando = Randomisation; PP = Piperaquine phosphate; (d) = Day from IP dose.
AE No. = Adverse Event Number; TEAE = A treatment-emergent adverse event; SAE = Serious adverse event; UN = Unknown; Dis = Discontinuation

Listing 16.2.7.1
Adverse Events

Rando Number	Challenge Agent	AE No	System Organ Class/ Preferred Term/ Adverse Event Verbatim	Onset Date/ Onset Time/ (d) End Date/ End Time/ (d)	TEAE/ SAE	Severity/ Action Taken/ Outcome	Treatment Directly Related to AE (Inoculum/ Artesunate/ Piperaquine/ Malarone/ Primacin/ Mosquito)	Time of onset Relative to Inoculum/ Time of onset Relative to Artesunate/ Time of onset Relative to PP	Duration (hours)	Dis
R104	K13	15	Musculoskeletal and connective tissue disorders/ Arthralgia/ Arthralgia intermittent	25-NOV-2017/ 16:59/ (3)/ 28-NOV-2017/ 08:28/ (6)	Yes/ No	MILD/ MEDICATION: Ibuprofen/ RECOVERED/ RESOLVED	Probable/ Unrelated/ Unrelated/ Unrelated/ Unrelated/ Unrelated	11.3 days post/ 2.3 days post/ 0 days post	63.48	No
		16	Investigations/ Neutrophil count decreased/ Neutrophil count decreased	25-NOV-2017/ 17:43/ (3)/ 26-NOV-2017/ 10:41/ (4)	Yes/ No	SEVERE/ NONE/ RECOVERED/ RESOLVED	Probable/ Unrelated/ Unrelated/ Unrelated/ Unrelated/ Unrelated	11.3 days post/ 2.3 days post/ 0 days post	16.97	No
		17	Investigations/ White blood cell count decreased/ White cell count decreased	25-NOV-2017/ 17:43/ (3)/ 30-NOV-2017/ 08:43/ (8)	Yes/ No	MODERATE/ NONE/ RECOVERED/ RESOLVED	Probable/ Unrelated/ Unrelated/ Unrelated/ Unrelated/ Unrelated	11.3 days post/ 2.3 days post/ 0 days post	111.00	No
		18	Musculoskeletal and connective tissue disorders/ Neck pain/ Neck pain	25-NOV-2017/ 18:00/ (3)/ 27-NOV-2017/ 08:00/ (5)	Yes/ No	MODERATE/ NONE/ RECOVERED/ RESOLVED	Possible/ Unrelated/ Unrelated/ Unrelated/ Unrelated/ Unrelated	11.3 days post/ 2.3 days post/ 0 days post	38.00	No

Notes: Adverse events were coded using the Medical Dictionary for Regulatory Activities (MedDRA) Version 20.1.
A treatment-emergent adverse event is defined as an AE that occurred or worsened following first administration of study drug.
Rando = Randomisation; PP = Piperaquine phosphate; (d) = Day from IP dose.
AE No. = Adverse Event Number; TEAE = A treatment-emergent adverse event; SAE = Serious adverse event; UN = Unknown; Dis = Discontinuation

Listing 16.2.7.1
Adverse Events

Rando Number	Challenge Agent	AE No	System Organ Class/ Preferred Term/ Adverse Event Verbatim	Onset Date/ Onset Time/ (d) End Date/ End Time/ (d)	TEAE/ SAE	Severity/ Action Taken/ Outcome	Treatment Directly Related to AE (Inoculum/ Artesunate/ Piperaquine/ Malarone/ Primacin/ Mosquito)	Time of onset Relative to Inoculum/ Time of onset Relative to Artesunate/ Time of onset Relative to PP	Duration (hours)	Dis
R104	K13	19	Nervous system disorders/ Headache/ Headache	25-NOV-2017/ 20:04/ (3)/ 26-NOV-2017/ UN:UN/ (4)	Yes/ No	MODERATE/ NONE/ RECOVERED/ RESOLVED	Probable/ Unrelated/ Unrelated/ Unrelated/ Unrelated/ Unrelated	11.4 days post/ 2.4 days post/ 0.1 days post		No
		20	Investigations/ Lymphocyte count decreased/ Lymphocyte count decreased	26-NOV-2017/ 10:41/ (4)/ 30-NOV-2017/ 08:43/ (8)	Yes/ No	MODERATE/ NONE/ RECOVERED/ RESOLVED	Probable/ Unrelated/ Unrelated/ Unrelated/ Unrelated/ Unrelated	12 days post/ 3 days post/ 0.7 days post	94.03	No
		21	Investigations/ Neutrophil count decreased/ Neutrophil count decreased	26-NOV-2017/ 10:41/ (4)/ 30-NOV-2017/ 08:43/ (8)	Yes/ No	MODERATE/ NONE/ RECOVERED/ RESOLVED	Probable/ Unrelated/ Unrelated/ Unrelated/ Unrelated/ Unrelated	12 days post/ 3 days post/ 0.7 days post	94.03	No
		22	Investigations/ Alanine aminotransferase increased/ Alanine Transaminase count increased	30-NOV-2017/ 08:43/ (8)/ 03-DEC-2017/ 09:13/ (11)	Yes/ No	SEVERE/ NONE/ RECOVERED/ RESOLVED	Probable/ Unrelated/ Unrelated/ Unrelated/ Unrelated/ Unrelated	15.9 days post/ 6.9 days post/ 4.6 days post	72.50	No

Notes: Adverse events were coded using the Medical Dictionary for Regulatory Activities (MedDRA) Version 20.1.
A treatment-emergent adverse event is defined as an AE that occurred or worsened following first administration of study drug.
Rando = Randomisation; PP = Piperaquine phosphate; (d) = Day from IP dose.
AE No. = Adverse Event Number; TEAE = A treatment-emergent adverse event; SAE = Serious adverse event; UN = Unknown; Dis = Discontinuation

Listing 16.2.7.1
Adverse Events

Rando Number	Challenge Agent	AE No	System Organ Class/ Preferred Term/ Adverse Event Verbatim	Onset Date/ Onset Time/ (d) End Date/ End Time/ (d)	TEAE/ SAE	Severity/ Action Taken/ Outcome	Treatment Directly Related to AE (Inoculum/ Artesunate/ Piperaquine/ Malarone/ Primacin/ Mosquito)	Time of onset Relative to Inoculum/ Time of onset Relative to Artesunate/ Time of onset Relative to PP	Duration (hours)	Dis
R104	K13	23	Investigations/ Alanine aminotransferase increased/ Alanine transaminase count increased	03-DEC-2017/ 09:13/ (11)/ 04-DEC-2017/ 12:45/ (12)	Yes/ No	MODERATE/ NONE/ RECOVERED/ RESOLVED	Probable/ Unrelated/ Unrelated/ Unrelated/ Unrelated/ Unrelated	18.9 days post/ 9.9 days post/ 7.6 days post	27.53	No
		24	Infections and infestations/ Upper respiratory tract infection/ Upper respiratory tract infection	05-DEC-2017/ 07:00/ (13)/ 08-DEC-2017/ 07:00/ (16)	Yes/ No	MILD/ NONE/ RECOVERED/ RESOLVED	Unrelated/ Unrelated/ Unrelated/ Unrelated/ Unrelated/ Unrelated	20.8 days post/ 11.8 days post/ 9.5 days post	72.00	No
R105	K13	1	Gastrointestinal disorders/ Nausea/ Nausea	19-NOV-2017/ 13:42/ (-4)/ 19-NOV-2017/ 14:00/ (-4)	No/ No	MILD/ NONE/ RECOVERED/ RESOLVED	Possible/ Unrelated/ Unrelated/ Unrelated/ Unrelated/ Unrelated	5.1 days post/ -3.9 days pre/ -6.2 days pre	0.30	No
		2	Nervous system disorders/ Headache/ Headache	20-NOV-2017/ 19:00/ (-3)/ 20-NOV-2017/ 20:00/ (-3)	No/ No	MILD/ NONE/ RECOVERED/ RESOLVED	Unlikely/ Unrelated/ Unrelated/ Unrelated/ Unrelated/ Unrelated	6.3 days post/ -2.6 days pre/ -5 days pre	1.00	No

Notes: Adverse events were coded using the Medical Dictionary for Regulatory Activities (MedDRA) Version 20.1.
A treatment-emergent adverse event is defined as an AE that occurred or worsened following first administration of study drug.
Rando = Randomisation; PP = Piperaquine phosphate; (d) = Day from IP dose.
AE No. = Adverse Event Number; TEAE = A treatment-emergent adverse event; SAE = Serious adverse event; UN = Unknown; Dis = Discontinuation

Listing 16.2.7.1
Adverse Events

Rando Number	Challenge Agent	AE No	System Organ Class/ Preferred Term/ Adverse Event Verbatim	Onset Date/ Onset Time/ (d) End Date/ End Time/ (d)	TEAE/ SAE	Severity/ Action Taken/ Outcome	Treatment Directly Related to AE (Inoculum/ Artesunate/ Piperaquine/ Malarone/ Primacin/ Mosquito)	Time of onset Relative to Inoculum/ Time of onset Relative to Artesunate/ Time of onset Relative to PP	Duration (hours)	Dis
R105	K13	3	Cardiac disorders/ Tachycardia/ Tachycardia	20-NOV-2017/ 19:15/ (-3)/ 21-NOV-2017/ 07:47/ (-2)	No/ No	MILD/ NONE/ RECOVERED/ RESOLVED	Possible/ Unrelated/ Unrelated/ Unrelated/ Unrelated/ Unrelated	6.4 days post/ -2.6 days pre/ -4.9 days pre	12.53	No
		4	Cardiac disorders/ Tachycardia/ Intermittent tachycardia	24-NOV-2017/ 20:10/ (2)/ 26-NOV-2017/ 18:55/ (4)	Yes/ No	MILD/ NONE/ RECOVERED/ RESOLVED	Probable/ Unrelated/ Unrelated/ Unrelated/ Unrelated/ Unrelated	10.4 days post/ 1.4 days post/ -0.9 days pre	46.75	No
		5	Nervous system disorders/ Headache/ Intermittent headache	27-NOV-2017/ 16:50/ (5)/ 28-NOV-2017/ 19:01/ (6)	Yes/ No	MILD/ NONE/ RECOVERED/ RESOLVED	Probable/ Unrelated/ Unrelated/ Unrelated/ Unrelated/ Unrelated	13.3 days post/ 4.3 days post/ 2 days post	26.18	No
R106	3D7	1	Musculoskeletal and connective tissue disorders/ Myalgia/ Intermittent myalgia	18-NOV-2017/ 12:00/ (-5)/ 19-NOV-2017/ 21:00/ (-4)	No/ No	MILD/ NONE/ RECOVERED/ RESOLVED	Unlikely/ Unrelated/ Unrelated/ Unrelated/ Unrelated/ Unrelated	3.1 days post/ -4.9 days pre/ -12.9 days pre	33.00	No

Notes: Adverse events were coded using the Medical Dictionary for Regulatory Activities (MedDRA) Version 20.1.
A treatment-emergent adverse event is defined as an AE that occurred or worsened following first administration of study drug.
Rando = Randomisation; PP = Piperaquine phosphate; (d) = Day from IP dose.
AE No. = Adverse Event Number; TEAE = A treatment-emergent adverse event; SAE = Serious adverse event; UN = Unknown; Dis = Discontinuation

Listing 16.2.7.1
Adverse Events

Rando Number	Challenge Agent	AE No	System Organ Class/ Preferred Term/ Adverse Event Verbatim	Onset Date/ Onset Time/ (d) End Date/ End Time/ (d)	TEAE/ SAE	Severity/ Action Taken/ Outcome	Treatment Directly Related to AE (Inoculum/ Artesunate/ Piperaquine/ Malarone/ Primacin/ Mosquito)	Time of onset Relative to Inoculum/ Time of onset Relative to Artesunate/ Time of onset Relative to PP	Duration (hours)	Dis
R106	3D7	2	Nervous system disorders/ Headache/ Headache	19-NOV-2017/ 21:30/ (-4)/ 19-NOV-2017/ 22:00/ (-4)	No/ No	MILD/ MEDICATION: Ibuprofen/ RECOVERED/ RESOLVED	Unlikely/ Unrelated/ Unrelated/ Unrelated/ Unrelated/ Unrelated	4.5 days post/ -3.5 days pre/ -11.5 days pre	0.50	No
		3	Musculoskeletal and connective tissue disorders/ Myalgia/ Myalgia	24-NOV-2017/ 07:00/ (2)/ 24-NOV-2017/ 17:00/ (2)	Yes/ No	MILD/ MEDICATION: Ibuprofen/ RECOVERED/ RESOLVED	Probable/ Unrelated/ Unrelated/ Unrelated/ Unrelated/ Unrelated	8.9 days post/ 0.9 days post/ -7.1 days pre	10.00	No
		4	Nervous system disorders/ Headache/ Intermittent headache	24-NOV-2017/ 09:40/ (2)/ 24-NOV-2017/ 17:00/ (2)	Yes/ No	MILD/ MEDICATION: Ibuprofen/ RECOVERED/ RESOLVED	Probable/ Unrelated/ Unrelated/ Unrelated/ Unrelated/ Unrelated	9 days post/ 1 days post/ -7 days pre	7.33	No
		5	General disorders and administration site conditions/ Fatigue/ Fatigue	24-NOV-2017/ 11:00/ (2)/ 24-NOV-2017/ 17:00/ (2)	Yes/ No	MILD/ NONE/ RECOVERED/ RESOLVED	Probable/ Unrelated/ Unrelated/ Unrelated/ Unrelated/ Unrelated	9 days post/ 1 days post/ -6.9 days pre	6.00	No

Notes: Adverse events were coded using the Medical Dictionary for Regulatory Activities (MedDRA) Version 20.1.
A treatment-emergent adverse event is defined as an AE that occurred or worsened following first administration of study drug.
Rando = Randomisation; PP = Piperaquine phosphate; (d) = Day from IP dose.
AE No. = Adverse Event Number; TEAE = A treatment-emergent adverse event; SAE = Serious adverse event; UN = Unknown; Dis = Discontinuation

Listing 16.2.7.1
Adverse Events

Rando Number	Challenge Agent	AE No	System Organ Class/ Preferred Term/ Adverse Event Verbatim	Onset Date/ Onset Time/ (d) End Date/ End Time/ (d)	TEAE/ SAE	Severity/ Action Taken/ Outcome	Treatment Directly Related to AE (Inoculum/ Artesunate/ Piperaquine/ Malarone/ Primacin/ Mosquito)	Time of onset Relative to Inoculum/ Time of onset Relative to Artesunate/ Time of onset Relative to PP	Duration (hours)	Dis
R106	3D7	6	Skin and subcutaneous tissue disorders/ Hyperhidrosis/ Sweats	24-NOV-2017/ 12:30/ (2)/ 24-NOV-2017/ 14:00/ (2)	Yes/ No	MILD/ NONE/ RECOVERED/ RESOLVED	Probable/ Unrelated/ Unrelated/ Unrelated/ Unrelated/ Unrelated	9.1 days post/ 1.1 days post/ -6.8 days pre	1.50	No
		7	General disorders and administration site conditions/ Malaise/ Malaise	24-NOV-2017/ 13:47/ (2)/ 24-NOV-2017/ 20:10/ (2)	Yes/ No	MILD/ NONE/ RECOVERED/ RESOLVED	Probable/ Unrelated/ Unrelated/ Unrelated/ Unrelated/ Unrelated	9.1 days post/ 1.1 days post/ -6.8 days pre	6.38	No
		8	Nervous system disorders/ Headache/ Headache	25-NOV-2017/ 18:00/ (3)/ 25-NOV-2017/ 20:15/ (3)	Yes/ No	MILD/ MEDICATION: Ibuprofen/ RECOVERED/ RESOLVED	Probable/ Unrelated/ Unrelated/ Unrelated/ Unrelated/ Unrelated	10.3 days post/ 2.3 days post/ -5.6 days pre	2.25	No
		9	Nervous system disorders/ Headache/ Headache	26-NOV-2017/ 11:30/ (4)/ 26-NOV-2017/ 12:30/ (4)	Yes/ No	MILD/ MEDICATION: Ibuprofen/ RECOVERED/ RESOLVED	Probable/ Unrelated/ Unrelated/ Unrelated/ Unrelated/ Unrelated	11 days post/ 3 days post/ -4.9 days pre	1.00	No

Notes: Adverse events were coded using the Medical Dictionary for Regulatory Activities (MedDRA) Version 20.1.
A treatment-emergent adverse event is defined as an AE that occurred or worsened following first administration of study drug.
Rando = Randomisation; PP = Piperaquine phosphate; (d) = Day from IP dose.
AE No. = Adverse Event Number; TEAE = A treatment-emergent adverse event; SAE = Serious adverse event; UN = Unknown; Dis = Discontinuation

Listing 16.2.7.1
Adverse Events

Rando Number	Challenge Agent	AE No	System Organ Class/ Preferred Term/ Adverse Event Verbatim	Onset Date/ Onset Time/ (d) End Date/ End Time/ (d)	TEAE/ SAE	Severity/ Action Taken/ Outcome	Treatment Directly Related to AE (Inoculum/ Artesunate/ Piperaquine/ Malarone/ Primacin/ Mosquito)	Time of onset Relative to Inoculum/ Time of onset Relative to Artesunate/ Time of onset Relative to PP	Duration (hours)	Dis
R106	3D7	10	General disorders and administration site conditions/ Fatigue/ Fatigue	26-NOV-2017/ 18:55/ (4)/ 27-NOV-2017/ 16:00/ (5)	Yes/ No	MILD/ NONE/ RECOVERED/ RESOLVED	Unrelated/ Unrelated/ Unrelated/ Unrelated/ Unrelated/ Unrelated	11.4 days post/ 3.4 days post/ -4.6 days pre	21.08	No
		11	Musculoskeletal and connective tissue disorders/ Back pain/ Back pain	27-NOV-2017/ 07:30/ (5)/ 27-NOV-2017/ 10:00/ (5)	Yes/ No	MILD/ MEDICATION: Ibuprofen/ RECOVERED/ RESOLVED	Unrelated/ Unrelated/ Unrelated/ Unrelated/ Unrelated/ Unrelated	11.9 days post/ 3.9 days post/ -4 days pre	2.50	No
		12	Reproductive system and breast disorders/ Dysmenorrhoea/ Dysmenorrhea	28-NOV-2017/ 22:00/ (6)/ 28-NOV-2017/ 23:00/ (6)	Yes/ No	MILD/ MEDICATION: ibuprofen/ RECOVERED/ RESOLVED	Unrelated/ Unrelated/ Unrelated/ Unrelated/ Unrelated/ Unrelated	13.5 days post/ 5.5 days post/ -2.4 days pre	1.00	No
		13	Reproductive system and breast disorders/ Dysmenorrhoea/ Dysmenorrhea	29-NOV-2017/ 16:00/ (7)/ 29-NOV-2017/ 17:00/ (7)	Yes/ No	MILD/ MEDICATION: Ibuprofen/ RECOVERED/ RESOLVED	Unrelated/ Unrelated/ Unrelated/ Unrelated/ Unrelated/ Unrelated	14.2 days post/ 6.2 days post/ -1.7 days pre	1.00	No

Notes: Adverse events were coded using the Medical Dictionary for Regulatory Activities (MedDRA) Version 20.1.
A treatment-emergent adverse event is defined as an AE that occurred or worsened following first administration of study drug.
Rando = Randomisation; PP = Piperaquine phosphate; (d) = Day from IP dose.
AE No. = Adverse Event Number; TEAE = A treatment-emergent adverse event; SAE = Serious adverse event; UN = Unknown; Dis = Discontinuation

Listing 16.2.7.1
Adverse Events

Rando Number	Challenge Agent	AE No	System Organ Class/ Preferred Term/ Adverse Event Verbatim	Onset Date/ Onset Time/ (d) End Date/ End Time/ (d)	TEAE/ SAE	Severity/ Action Taken/ Outcome	Treatment Directly Related to AE (Inoculum/ Artesunate/ Piperaquine/ Malarone/ Primacin/ Mosquito)	Time of onset Relative to Inoculum/ Time of onset Relative to Artesunate/ Time of onset Relative to PP	Duration (hours)	Dis
R106	3D7	14	Nervous system disorders/ Headache/ Intermittent headache	30-NOV-2017/ 11:00/ (8)/ 03-DEC-2017/ 21:30/ (11)	Yes/ No	MILD/ MEDICATION: Ibuprofen/ RECOVERED/ RESOLVED	Probable/ Unrelated/ Unrelated/ Unrelated/ Unrelated/ Unrelated	15 days post/ 7 days post/ -0.9 days pre	82.50	No
		15	Investigations/ Lymphocyte count decreased/ Lymphocytes count decreased	01-DEC-2017/ 08:27/ (9)/ 03-DEC-2017/ 09:39/ (11)	Yes/ No	MODERATE/ NONE/ RECOVERED/ RESOLVED	Probable/ Unrelated/ Unrelated/ Unrelated/ Unrelated/ Unrelated	15.9 days post/ 7.9 days post/ 0 days post	49.20	No
		16	General disorders and administration site conditions/ Malaise/ Malaise	01-DEC-2017/ 08:30/ (9)/ 01-DEC-2017/ 15:00/ (9)	Yes/ No	MILD/ NONE/ RECOVERED/ RESOLVED	Probable/ Unrelated/ Unrelated/ Unrelated/ Unrelated/ Unrelated	15.9 days post/ 7.9 days post/ 0 days post	6.50	No
		17	General disorders and administration site conditions/ Pyrexia/ fever	01-DEC-2017/ 20:00/ (9)/ 01-DEC-2017/ 21:30/ (9)	Yes/ No	MILD/ MEDICATION: Ibuprofen/ RECOVERED/ RESOLVED	Probable/ Unrelated/ Unrelated/ Unrelated/ Unrelated/ Unrelated	16.4 days post/ 8.4 days post/ 0.5 days post	1.50	No

Notes: Adverse events were coded using the Medical Dictionary for Regulatory Activities (MedDRA) Version 20.1.
A treatment-emergent adverse event is defined as an AE that occurred or worsened following first administration of study drug.
Rando = Randomisation; PP = Piperaquine phosphate; (d) = Day from IP dose.
AE No. = Adverse Event Number; TEAE = A treatment-emergent adverse event; SAE = Serious adverse event; UN = Unknown; Dis = Discontinuation

Listing 16.2.7.1
Adverse Events

Rando Number	Challenge Agent	AE No	System Organ Class/ Preferred Term/ Adverse Event Verbatim	Onset Date/ Onset Time/ (d) End Date/ End Time/ (d)	TEAE/ SAE	Severity/ Action Taken/ Outcome	Treatment Directly Related to AE (Inoculum/ Artesunate/ Piperaquine/ Malarone/ Primacin/ Mosquito)	Time of onset Relative to Inoculum/ Time of onset Relative to Artesunate/ Time of onset Relative to PP	Duration (hours)	Dis
R106	3D7	18	Infections and infestations/ Upper respiratory tract infection/ Upper respiratory tract infection	07-DEC-2017/ 06:30/ (15)/ 08-DEC-2017/ 06:30/ (16)	Yes/ No	MILD/ NONE/ RECOVERED/ RESOLVED	Unrelated/ Unrelated/ Unrelated/ Unrelated/ Unrelated/ Unrelated	21.8 days post/ 13.8 days post/ 5.9 days post	24.00	No
		19	Investigations/ Blood phosphorus decreased/ Phosphate count decreased	14-DEC-2017/ 08:53/ (22)/ 18-DEC-2017/ 08:03/ (26)	Yes/ No	MODERATE/ NONE/ RECOVERED/ RESOLVED	Unlikely/ Unrelated/ Unrelated/ Unrelated/ Unrelated/ Unrelated	28.9 days post/ 20.9 days post/ 13 days post	95.17	No
R107	3D7	1	Gastrointestinal disorders/ Nausea/ Nausea	20-NOV-2017/ 23:30/ (-3)/ 24-NOV-2017/ 06:10/ (2)	No/ No	MILD/ MEDICATION: Ondansetron/ RECOVERED/ RESOLVED	Probable/ Unrelated/ Unrelated/ Unrelated/ Unrelated/ Unrelated	5.5 days post	78.67	No
		2	Gastrointestinal disorders/ Abdominal tenderness/ Abdominal tenderness	21-NOV-2017/ 07:40/ (-2)/ 26-NOV-2017/ 08:59/ (4)	No/ No	MILD/ NONE/ RECOVERED/ RESOLVED	Probable/ Unrelated/ Unrelated/ Unrelated/ Unrelated/ Unrelated	5.9 days post	121.32	No

Notes: Adverse events were coded using the Medical Dictionary for Regulatory Activities (MedDRA) Version 20.1.
A treatment-emergent adverse event is defined as an AE that occurred or worsened following first administration of study drug.
Rando = Randomisation; PP = Piperaquine phosphate; (d) = Day from IP dose.
AE No. = Adverse Event Number; TEAE = A treatment-emergent adverse event; SAE = Serious adverse event; UN = Unknown; Dis = Discontinuation

Listing 16.2.7.1
Adverse Events

Rando Number	Challenge Agent	AE No	System Organ Class/ Preferred Term/ Adverse Event Verbatim	Onset Date/ Onset Time/ (d) End Date/ End Time/ (d)	TEAE/ SAE	Severity/ Action Taken/ Outcome	Treatment Directly Related to AE (Inoculum/ Artesunate/ Piperaquine/ Malarone/ Primacin/ Mosquito)	Time of onset Relative to Inoculum/ Time of onset Relative to Artesunate/ Time of onset Relative to PP	Duration (hours)	Dis
R107	3D7	3	General disorders and administration site conditions/ Fatigue/ Fatigue	21-NOV-2017/ 07:55/ (-2)/ 24-NOV-2017/ 08:15/ (2)	No/ No	MILD/ NONE/ RECOVERED/ RESOLVED	Probable/ Unrelated/ Unrelated/ Unrelated/ Unrelated/ Unrelated	5.9 days post	72.33	No
		4	Metabolism and nutrition disorders/ Decreased appetite/ Anorexia	21-NOV-2017/ 11:30/ (-2)/ 22-NOV-2017/ 16:00/ (-1)	No/ No	MILD/ NONE/ RECOVERED/ RESOLVED	Probable/ Unrelated/ Unrelated/ Unrelated/ Unrelated/ Unrelated	6 days post	28.50	No
		5	Musculoskeletal and connective tissue disorders/ Arthralgia/ Intermittent arthralgia	21-NOV-2017/ 12:00/ (-2)/ 23-NOV-2017/ 20:45/ (1)	No/ No	MILD/ NONE/ RECOVERED/ RESOLVED	Probable/ Unrelated/ Unrelated/ Unrelated/ Unrelated/ Unrelated	6.1 days post	56.75	No
		6	Nervous system disorders/ Headache/ Headache	22-NOV-2017/ 14:00/ (-1)/ 23-NOV-2017/ 07:00/ (1)	No/ No	MILD/ NONE/ RECOVERED/ RESOLVED	Probable/ Unrelated/ Unrelated/ Unrelated/ Unrelated/ Unrelated	7.1 days post	17.00	No

Notes: Adverse events were coded using the Medical Dictionary for Regulatory Activities (MedDRA) Version 20.1.
A treatment-emergent adverse event is defined as an AE that occurred or worsened following first administration of study drug.
Rando = Randomisation; PP = Piperaquine phosphate; (d) = Day from IP dose.
AE No. = Adverse Event Number; TEAE = A treatment-emergent adverse event; SAE = Serious adverse event; UN = Unknown; Dis = Discontinuation

Listing 16.2.7.1
Adverse Events

Rando Number	Challenge Agent	AE No	System Organ Class/ Preferred Term/ Adverse Event Verbatim	Onset Date/ Onset Time/ (d) End Date/ End Time/ (d)	TEAE/ SAE	Severity/ Action Taken/ Outcome	Treatment Directly Related to AE (Inoculum/ Artesunate/ Piperaquine/ Malarone/ Primacin/ Mosquito)	Time of onset Relative to Inoculum/ Time of onset Relative to Artesunate/ Time of onset Relative to PP	Duration (hours)	Dis
R107	3D7	7	Gastrointestinal disorders/ Abdominal discomfort/ Abdominal discomfort intermittent	26-NOV-2017/ 12:00/ (4)/ 01-DEC-2017/ 21:00/ (9)	Yes/ No	MILD/ NONE/ RECOVERED/ RESOLVED	Probable/ Unrelated/ Unrelated/ Unrelated/ Unrelated/ Unrelated	11.1 days post	129.00	No
		8	Musculoskeletal and connective tissue disorders/ Myalgia/ Myalgia	01-DEC-2017/ 19:00/ (9)/ 04-DEC-2017/ 12:00/ (12)	Yes/ No	MODERATE/ MEDICATION: Ondansetron/ RECOVERED/ RESOLVED	Probable/ Unrelated/ Unrelated/ Unrelated/ Unrelated/ Unrelated	16.4 days post	65.00	No
		9	Gastrointestinal disorders/ Abdominal tenderness/ Abdominal tenderness	04-DEC-2017/ 08:44/ (12)/ 05-DEC-2017/ 07:57/ (13)	Yes/ No	MILD/ NONE/ RECOVERED/ RESOLVED	Probable/ Unrelated/ Unrelated/ Unrelated/ Unrelated/ Unrelated	18.9 days post	23.22	No
		10	Nervous system disorders/ Headache/ Headache	04-DEC-2017/ 13:00/ (12)/ 04-DEC-2017/ 22:15/ (12)	Yes/ No	MILD/ MEDICATION: Ibuprofen/ RECOVERED/ RESOLVED	Probable/ Unrelated/ Unrelated/ Unrelated/ Unrelated/ Unrelated	19.1 days post	9.25	No

Notes: Adverse events were coded using the Medical Dictionary for Regulatory Activities (MedDRA) Version 20.1.
A treatment-emergent adverse event is defined as an AE that occurred or worsened following first administration of study drug.
Rando = Randomisation; PP = Piperaquine phosphate; (d) = Day from IP dose.
AE No. = Adverse Event Number; TEAE = A treatment-emergent adverse event; SAE = Serious adverse event; UN = Unknown; Dis = Discontinuation

Listing 16.2.7.1
Adverse Events

Rando Number	Challenge Agent	AE No	System Organ Class/ Preferred Term/ Adverse Event Verbatim	Onset Date/ Onset Time/ (d) End Date/ End Time/ (d)	TEAE/ SAE	Severity/ Action Taken/ Outcome	Treatment Directly Related to AE (Inoculum/ Artesunate/ Piperaquine/ Malarone/ Primacin/ Mosquito)	Time of onset Relative to Inoculum/ Time of onset Relative to Artesunate/ Time of onset Relative to PP	Duration (hours)	Dis
R107	3D7	11	Nervous system disorders/ Dizziness/ Dizziness	05-DEC-2017/ 05:00/ (13)/ 06-DEC-2017/ 09:00/ (14)	Yes/ No	MILD/ NONE/ RECOVERED/ RESOLVED	Probable/ Unrelated/ Unrelated/ Unrelated/ Unrelated/ Unrelated	19.8 days post	28.00	No
R108	3D7	1	Nervous system disorders/ Headache/ Headache	22-NOV-2017/ 22:00/ (-1)/ 23-NOV-2017/ 06:00/ (1)	No/ No	MILD/ MEDICATION: Paracetamol/ RECOVERED/ RESOLVED	Probable/ Unrelated/ Unrelated/ Unrelated/ Unrelated/ Unrelated	7.5 days post/ -0.5 days pre/ -8.4 days pre	8.00	No
		2	General disorders and administration site conditions/ Fatigue/ Fatigue	23-NOV-2017/ 06:00/ (1)/ 23-NOV-2017/ 10:00/ (1)	No/ No	MILD/ NONE/ RECOVERED/ RESOLVED	Probable/ Unrelated/ Unrelated/ Unrelated/ Unrelated/ Unrelated	7.8 days post/ -0.2 days pre/ -8.1 days pre	4.00	No
		3	Investigations/ Urobilinogen urine/ Urobilinogenuria	23-NOV-2017/ 08:03/ (1)/ 28-NOV-2017/ 08:54/ (6)	No/ No	MODERATE/ NONE/ RECOVERED/ RESOLVED	Probable/ Unrelated/ Unrelated/ Unrelated/ Unrelated/ Unrelated	7.9 days post/ -0.1 days pre/ -8 days pre	120.85	No

Notes: Adverse events were coded using the Medical Dictionary for Regulatory Activities (MedDRA) Version 20.1.
A treatment-emergent adverse event is defined as an AE that occurred or worsened following first administration of study drug.
Rando = Randomisation; PP = Piperaquine phosphate; (d) = Day from IP dose.
AE No. = Adverse Event Number; TEAE = A treatment-emergent adverse event; SAE = Serious adverse event; UN = Unknown; Dis = Discontinuation

Listing 16.2.7.1
Adverse Events

Rando Number	Challenge Agent	AE No	System Organ Class/ Preferred Term/ Adverse Event Verbatim	Onset Date/ Onset Time/ (d) End Date/ End Time/ (d)	TEAE/ SAE	Severity/ Action Taken/ Outcome	Treatment Directly Related to AE (Inoculum/ Artesunate/ Piperaquine/ Malarone/ Primacin/ Mosquito)	Time of onset Relative to Inoculum/ Time of onset Relative to Artesunate/ Time of onset Relative to PP	Duration (hours)	Dis
R108	3D7	4	Nervous system disorders/ Headache/ Intermittent headache	23-NOV-2017/ 17:00/ (1)/ 25-NOV-2017/ 21:00/ (3)	Yes/ No	MILD/ MEDICATION: Ibuprofen/ RECOVERED/ RESOLVED	Probable/ Unrelated/ Unrelated/ Unrelated/ Unrelated/ Unrelated	8.3 days post/ 0.3 days post/ -7.6 days pre	52.00	No
		5	General disorders and administration site conditions/ Fatigue/ Intermittent fatigue	23-NOV-2017/ 18:30/ (1)/ 24-NOV-2017/ 08:23/ (2)	Yes/ No	MODERATE/ NONE/ RECOVERED/ RESOLVED	Probable/ Unrelated/ Unrelated/ Unrelated/ Unrelated/ Unrelated	8.3 days post/ 0.4 days post/ -7.5 days pre	13.88	No
		6	General disorders and administration site conditions/ Pyrexia/ Fever	23-NOV-2017/ 19:55/ (1)/ 23-NOV-2017/ 21:54/ (1)	Yes/ No	MILD/ NONE/ NOT RECOVERED/ NOT RESOLVED	Probable/ Unrelated/ Unrelated/ Unrelated/ Unrelated/ Unrelated	8.4 days post/ 0.4 days post/ -7.5 days pre	1.98	No
		7	General disorders and administration site conditions/ Pyrexia/ fever	23-NOV-2017/ 21:54/ (1)/ 23-NOV-2017/ 23:14/ (1)	Yes/ No	MODERATE/ NONE/ RECOVERED/ RESOLVED	Probable/ Unrelated/ Unrelated/ Unrelated/ Unrelated/ Unrelated	8.5 days post/ 0.5 days post/ -7.4 days pre	1.33	No

Notes: Adverse events were coded using the Medical Dictionary for Regulatory Activities (MedDRA) Version 20.1.
A treatment-emergent adverse event is defined as an AE that occurred or worsened following first administration of study drug.
Rando = Randomisation; PP = Piperaquine phosphate; (d) = Day from IP dose.
AE No. = Adverse Event Number; TEAE = A treatment-emergent adverse event; SAE = Serious adverse event; UN = Unknown; Dis = Discontinuation

Listing 16.2.7.1
Adverse Events

Rando Number	Challenge Agent	AE No	System Organ Class/ Preferred Term/ Adverse Event Verbatim	Onset Date/ Onset Time/ (d) End Date/ End Time/ (d)	TEAE/ SAE	Severity/ Action Taken/ Outcome	Treatment Directly Related to AE (Inoculum/ Artesunate/ Piperaquine/ Malarone/ Primacin/ Mosquito)	Time of onset Relative to Inoculum/ Time of onset Relative to Artesunate/ Time of onset Relative to PP	Duration (hours)	Dis
R108	3D7	8	General disorders and administration site conditions/ Pyrexia/ intermittent fever	23-NOV-2017/ 23:14/ (1)/ 24-NOV-2017/ 18:55/ (2)	Yes/ No	MILD/ MEDICATION: Ibuprofen/ RECOVERED/ RESOLVED	Probable/ Unrelated/ Unrelated/ Unrelated/ Unrelated/ Unrelated	8.5 days post/ 0.6 days post/ -7.3 days pre	19.68	No
		9	Gastrointestinal disorders/ Abdominal discomfort/ Intermittent abdominal discomfort	24-NOV-2017/ 05:00/ (2)/ 24-NOV-2017/ 19:51/ (2)	Yes/ No	MODERATE/ MEDICATION: Ibuprofen/ RECOVERED/ RESOLVED	Probable/ Unrelated/ Unrelated/ Unrelated/ Unrelated/ Unrelated	8.8 days post/ 0.8 days post/ -7.1 days pre	14.85	No
		10	General disorders and administration site conditions/ Vessel puncture site bruise/ Bruising left cubital fossa due to venepuncture/cannulation	24-NOV-2017/ 06:00/ (2)/ 11-DEC-2017/ 06:00/ (19)	Yes/ No	MILD/ NONE/ RECOVERED/ RESOLVED	Unrelated/ Unrelated/ Unrelated/ Unrelated/ Unrelated/ Unrelated	8.8 days post/ 0.8 days post/ -7.1 days pre	408.00	No
		11	General disorders and administration site conditions/ Vessel puncture site bruise/ Bruising right cubital fossa due to venepuncture/cannulation	24-NOV-2017/ 06:00/ (2)/ 11-DEC-2017/ 06:00/ (19)	Yes/ No	MILD/ NONE/ RECOVERED/ RESOLVED	Unrelated/ Unrelated/ Unrelated/ Unrelated/ Unrelated/ Unrelated	8.8 days post/ 0.8 days post/ -7.1 days pre	408.00	No

Notes: Adverse events were coded using the Medical Dictionary for Regulatory Activities (MedDRA) Version 20.1.
A treatment-emergent adverse event is defined as an AE that occurred or worsened following first administration of study drug.
Rando = Randomisation; PP = Piperaquine phosphate; (d) = Day from IP dose.
AE No. = Adverse Event Number; TEAE = A treatment-emergent adverse event; SAE = Serious adverse event; UN = Unknown; Dis = Discontinuation

Listing 16.2.7.1
Adverse Events

Rando Number	Challenge Agent	AE No	System Organ Class/ Preferred Term/ Adverse Event Verbatim	Onset Date/ Onset Time/ (d) End Date/ End Time/ (d)	TEAE/ SAE	Severity/ Action Taken/ Outcome	Treatment Directly Related to AE (Inoculum/ Artesunate/ Piperaquine/ Malarone/ Primacin/ Mosquito)	Time of onset Relative to Inoculum/ Time of onset Relative to Artesunate/ Time of onset Relative to PP	Duration (hours)	Dis
R108	3D7	12	General disorders and administration site conditions/ Chills/ chills	24-NOV-2017/ 08:33/ (2)/ 24-NOV-2017/ 13:30/ (2)	Yes/ No	MILD/ NONE/ RECOVERED/ RESOLVED	Probable/ Unrelated/ Unrelated/ Unrelated/ Unrelated/ Unrelated	8.9 days post/ 0.9 days post/ -7 days pre	4.95	No
		13	Skin and subcutaneous tissue disorders/ Hyperhidrosis/ sweating	24-NOV-2017/ 08:33/ (2)/ 24-NOV-2017/ 13:30/ (2)	Yes/ No	MILD/ NONE/ RECOVERED/ RESOLVED	Probable/ Unrelated/ Unrelated/ Unrelated/ Unrelated/ Unrelated	8.9 days post/ 0.9 days post/ -7 days pre	4.95	No
		14	Cardiac disorders/ Tachycardia/ Tachycardia	24-NOV-2017/ 12:43/ (2)/ 24-NOV-2017/ 19:47/ (2)	Yes/ No	MILD/ NONE/ RECOVERED/ RESOLVED	Probable/ Unrelated/ Unrelated/ Unrelated/ Unrelated/ Unrelated	9.1 days post/ 1.1 days post/ -6.8 days pre	7.07	No
		15	General disorders and administration site conditions/ Malaise/ Malaise	24-NOV-2017/ 13:00/ (2)/ 24-NOV-2017/ 19:51/ (2)	Yes/ No	MILD/ NONE/ RECOVERED/ RESOLVED	Probable/ Unrelated/ Unrelated/ Unrelated/ Unrelated/ Unrelated	9.1 days post/ 1.1 days post/ -6.8 days pre	6.85	No

Notes: Adverse events were coded using the Medical Dictionary for Regulatory Activities (MedDRA) Version 20.1.
A treatment-emergent adverse event is defined as an AE that occurred or worsened following first administration of study drug.
Rando = Randomisation; PP = Piperaquine phosphate; (d) = Day from IP dose.
AE No. = Adverse Event Number; TEAE = A treatment-emergent adverse event; SAE = Serious adverse event; UN = Unknown; Dis = Discontinuation

Listing 16.2.7.1
Adverse Events

Rando Number	Challenge Agent	AE No	System Organ Class/ Preferred Term/ Adverse Event Verbatim	Onset Date/ Onset Time/ (d) End Date/ End Time/ (d)	TEAE/ SAE	Severity/ Action Taken/ Outcome	Treatment Directly Related to AE (Inoculum/ Artesunate/ Piperaquine/ Malarone/ Primacin/ Mosquito)	Time of onset Relative to Inoculum/ Time of onset Relative to Artesunate/ Time of onset Relative to PP	Duration (hours)	Dis
R108	3D7	16	Gastrointestinal disorders/ Nausea/ Nausea	24-NOV-2017/ 13:00/ (2)/ 24-NOV-2017/ 19:51/ (2)	Yes/ No	MILD/ NONE/ RECOVERED/ RESOLVED	Probable/ Unrelated/ Unrelated/ Unrelated/ Unrelated/ Unrelated	9.1 days post/ 1.1 days post/ -6.8 days pre	6.85	No
		17	General disorders and administration site conditions/ Fatigue/ fatigue	24-NOV-2017/ 13:30/ (2)/ 25-NOV-2017/ 12:08/ (3)	Yes/ No	MILD/ NONE/ RECOVERED/ RESOLVED	Probable/ Unrelated/ Unrelated/ Unrelated/ Unrelated/ Unrelated	9.1 days post/ 1.1 days post/ -6.8 days pre	22.63	No
		18	Investigations/ Alanine aminotransferase increased/ Increased alanine aminotransferase	30-NOV-2017/ 06:57/ (8)/ 01-DEC-2017/ 07:14/ (9)	Yes/ No	MODERATE/ NONE/ RECOVERED/ RESOLVED	Probable/ Unrelated/ Unrelated/ Unrelated/ Unrelated/ Unrelated	14.8 days post/ 6.9 days post/ -1 days pre	24.28	No
		19	Nervous system disorders/ Headache/ headache	01-DEC-2017/ 09:20/ (9)/ 01-DEC-2017/ 12:30/ (9)	Yes/ No	MODERATE/ MEDICATION: ibuprofen/ RECOVERED/ RESOLVED	Probable/ Unrelated/ Unrelated/ Unrelated/ Unrelated/ Unrelated	15.9 days post/ 8 days post/ 0.1 days post	3.17	No

Notes: Adverse events were coded using the Medical Dictionary for Regulatory Activities (MedDRA) Version 20.1.
A treatment-emergent adverse event is defined as an AE that occurred or worsened following first administration of study drug.
Rando = Randomisation; PP = Piperaquine phosphate; (d) = Day from IP dose.
AE No. = Adverse Event Number; TEAE = A treatment-emergent adverse event; SAE = Serious adverse event; UN = Unknown; Dis = Discontinuation

Listing 16.2.7.1
Adverse Events

Rando Number	Challenge Agent	AE No	System Organ Class/ Preferred Term/ Adverse Event Verbatim	Onset Date/ Onset Time/ (d) End Date/ End Time/ (d)	TEAE/ SAE	Severity/ Action Taken/ Outcome	Treatment Directly Related to AE (Inoculum/ Artesunate/ Piperaquine/ Malarone/ Primacin/ Mosquito)	Time of onset Relative to Inoculum/ Time of onset Relative to Artesunate/ Time of onset Relative to PP	Duration (hours)	Dis
R108	3D7	20	General disorders and administration site conditions/ Fatigue/ Fatigue	01-DEC-2017/ 09:20/ (9)/ 02-DEC-2017/ 15:00/ (10)	Yes/ No	MILD/ NONE/ RECOVERED/ RESOLVED	Probable/ Unrelated/ Unrelated/ Unrelated/ Unrelated/ Unrelated	15.9 days post/ 8 days post/ 0.1 days post	29.67	No
		21	General disorders and administration site conditions/ Malaise/ Malaise	02-DEC-2017/ 03:00/ (10)/ 02-DEC-2017/ 15:00/ (10)	Yes/ No	MILD/ NONE/ RECOVERED/ RESOLVED	Probable/ Unrelated/ Unrelated/ Unrelated/ Unrelated/ Unrelated	16.7 days post/ 8.7 days post/ 0.8 days post	12.00	No
		22	Nervous system disorders/ Lethargy/ lethargy	02-DEC-2017/ 07:00/ (10)/ 02-DEC-2017/ 15:00/ (10)	Yes/ No	MILD/ NONE/ RECOVERED/ RESOLVED	Probable/ Unrelated/ Unrelated/ Unrelated/ Unrelated/ Unrelated	16.9 days post/ 8.9 days post/ 1 days post	8.00	No
		23	Musculoskeletal and connective tissue disorders/ Musculoskeletal pain/ Right shoulder pain	03-DEC-2017/ 16:00/ (11)/ 03-DEC-2017/ 21:00/ (11)	Yes/ No	MILD/ MEDICATION: Paracetamol/ RECOVERED/ RESOLVED	Unlikely/ Unrelated/ Unrelated/ Unrelated/ Unrelated/ Unrelated	18.2 days post/ 10.2 days post/ 2.4 days post	5.00	No

Notes: Adverse events were coded using the Medical Dictionary for Regulatory Activities (MedDRA) Version 20.1.
A treatment-emergent adverse event is defined as an AE that occurred or worsened following first administration of study drug.
Rando = Randomisation; PP = Piperaquine phosphate; (d) = Day from IP dose.
AE No. = Adverse Event Number; TEAE = A treatment-emergent adverse event; SAE = Serious adverse event; UN = Unknown; Dis = Discontinuation

Listing 16.2.7.1
Adverse Events

Rando Number	Challenge Agent	AE No	System Organ Class/ Preferred Term/ Adverse Event Verbatim	Onset Date/ Onset Time/ (d) End Date/ End Time/ (d)	TEAE/ SAE	Severity/ Action Taken/ Outcome	Treatment Directly Related to AE (Inoculum/ Artesunate/ Piperaquine/ Malarone/ Primacin/ Mosquito)	Time of onset Relative to Inoculum/ Time of onset Relative to Artesunate/ Time of onset Relative to PP	Duration (hours)	Dis
R108	3D7	24	Gastrointestinal disorders/ Nausea/ intermittent nausea	12-DEC-2017/ 09:00/ (20)/ 13-DEC-2017/ 14:00/ (21)	Yes/ No	MILD/ NONE/ RECOVERED/ RESOLVED	Probable/ Unrelated/ Unrelated/ Unrelated/ Unrelated/ Unrelated	26.9 days post/ 19 days post/ 11.1 days post	29.00	No
		25	Metabolism and nutrition disorders/ Decreased appetite/ Anorexia	13-DEC-2017/ 10:00/ (21)/ 13-DEC-2017/ 14:00/ (21)	Yes/ No	MILD/ NONE/ RECOVERED/ RESOLVED	Unrelated/ Unrelated/ Unrelated/ Probable/ Unrelated/ Unrelated	28 days post/ 20 days post/ 12.1 days post	4.00	No
		26	Investigations/ Eosinophil count increased/ eosinophil count increased	14-DEC-2017/ 08:37/ (22)/ 21-DEC-2017/ 07:53/ (29)	Yes/ No	MODERATE/ NONE/ RECOVERED/ RESOLVED	Unrelated/ Unrelated/ Probable/ Probable/ Unrelated/ Unrelated	28.9 days post/ 20.9 days post/ 13 days post	167.27	No
R201	K13	1	Respiratory, thoracic and mediastinal disorders/ Oropharyngeal pain/ Sore throat	01-MAR-2018/ 16:50/ (2)/ 01-MAR-2018/ 19:00/ (2)	Yes/ No	MILD/ MEDICATION: ibuprofen/ RECOVERED/ RESOLVED	Probable/ Unrelated/ Unrelated/ Unrelated/ Unrelated/ Unrelated	10.3 days post/ 1.1 days post/ -0.9 days pre	2.17	No

Notes: Adverse events were coded using the Medical Dictionary for Regulatory Activities (MedDRA) Version 20.1.
A treatment-emergent adverse event is defined as an AE that occurred or worsened following first administration of study drug.
Rando = Randomisation; PP = Piperaquine phosphate; (d) = Day from IP dose.
AE No. = Adverse Event Number; TEAE = A treatment-emergent adverse event; SAE = Serious adverse event; UN = Unknown; Dis = Discontinuation

Listing 16.2.7.1
Adverse Events

Rando Number	Challenge Agent	AE No	System Organ Class/ Preferred Term/ Adverse Event Verbatim	Onset Date/ Onset Time/ (d) End Date/ End Time/ (d)	TEAE/ SAE	Severity/ Action Taken/ Outcome	Treatment Directly Related to AE (Inoculum/ Artesunate/ Piperaquine/ Malarone/ Primacin/ Mosquito)	Time of onset Relative to Inoculum/ Time of onset Relative to Artesunate/ Time of onset Relative to PP	Duration (hours)	Dis
R201	K13	2	Nervous system disorders/ Headache/ headache	01-MAR-2018/ 16:50/ (2)/ 01-MAR-2018/ 19:00/ (2)	Yes/ No	MODERATE/ MEDICATION: ibuprofen/ RECOVERED/ RESOLVED	Probable/ Unrelated/ Unrelated/ Unrelated/ Unrelated/ Unrelated	10.3 days post/ 1.1 days post/ -0.9 days pre	2.17	No
		3	Investigations/ White blood cell count decreased/ white cell count decreased	02-MAR-2018/ 13:24/ (3)/ 03-MAR-2018/ 13:27/ (4)	Yes/ No	MODERATE/ NONE/ RECOVERED/ RESOLVED	Probable/ Unrelated/ Unrelated/ Unrelated/ Unrelated/ Unrelated	11.1 days post/ 2 days post/ 0 days post	24.05	No
		4	Investigations/ Neutrophil count decreased/ neutrophil count decreased	02-MAR-2018/ 13:24/ (3)/ 05-MAR-2018/ 08:54/ (6)	Yes/ No	MODERATE/ NONE/ RECOVERED/ RESOLVED	Probable/ Unrelated/ Unrelated/ Unrelated/ Unrelated/ Unrelated	11.1 days post/ 2 days post/ 0 days post	67.50	No
		5	Nervous system disorders/ Headache/ headache	12-MAR-2018/ 20:00/ (13)/ 13-MAR-2018/ 08:00/ (14)	Yes/ No	MILD/ MEDICATION: ibuprofen/ RECOVERED/ RESOLVED	Unrelated/ Unrelated/ Unrelated/ Unrelated/ Unrelated/ Unrelated	21.4 days post/ 12.3 days post/ 10.3 days post	12.00	No

Notes: Adverse events were coded using the Medical Dictionary for Regulatory Activities (MedDRA) Version 20.1.
A treatment-emergent adverse event is defined as an AE that occurred or worsened following first administration of study drug.
Rando = Randomisation; PP = Piperaquine phosphate; (d) = Day from IP dose.
AE No. = Adverse Event Number; TEAE = A treatment-emergent adverse event; SAE = Serious adverse event; UN = Unknown; Dis = Discontinuation

Listing 16.2.7.1
Adverse Events

Rando Number	Challenge Agent	AE No	System Organ Class/ Preferred Term/ Adverse Event Verbatim	Onset Date/ Onset Time/ (d) End Date/ End Time/ (d)	TEAE/ SAE	Severity/ Action Taken/ Outcome	Treatment Directly Related to AE (Inoculum/ Artesunate/ Piperaquine/ Malarone/ Primacin/ Mosquito)	Time of onset Relative to Inoculum/ Time of onset Relative to Artesunate/ Time of onset Relative to PP	Duration (hours)	Dis
R201	K13	6	Gastrointestinal disorders/ Vomiting/ vomiting	21-MAR-2018/ 09:05/ (22)/ 21-MAR-2018/ 09:10/ (22)	Yes/ No	MODERATE/ NONE/ RECOVERED/ RESOLVED	Unrelated/ Unrelated/ Unrelated/ Probable/ Unrelated/ Unrelated	29.9 days post/ 20.8 days post/ 18.8 days post	0.08	No
		7	Gastrointestinal disorders/ Nausea/ nausea	21-MAR-2018/ 09:05/ (22)/ 22-MAR-2018/ 07:00/ (23)	Yes/ No	MILD/ NONE/ RECOVERED/ RESOLVED	Unrelated/ Unrelated/ Unrelated/ Probable/ Unrelated/ Unrelated	29.9 days post/ 20.8 days post/ 18.8 days post	21.92	No
R202	K13	1	Musculoskeletal and connective tissue disorders/ Arthralgia/ arthralgia	02-MAR-2018/ 23:00/ (3)/ 03-MAR-2018/ 08:00/ (4)	Yes/ No	MILD/ NONE/ RECOVERED/ RESOLVED	Probable/ Unrelated/ Unrelated/ Unrelated/ Unrelated/ Unrelated	11.5 days post/ 2.4 days post/ 0.4 days post	9.00	No
		2	General disorders and administration site conditions/ Feeling hot/ Feeling hot	03-MAR-2018/ UN:UN/ (4)/ 04-MAR-2018/ UN:UN/ (5)	Yes/ No	MILD/ MEDICATION: Ibuprofen/ RECOVERED/ RESOLVED	Probable/ Unrelated/ Unrelated/ Unrelated/ Unrelated/ Unrelated	11.6 days post/ 2.5 days post/ 0.4 days post		No

Notes: Adverse events were coded using the Medical Dictionary for Regulatory Activities (MedDRA) Version 20.1.
A treatment-emergent adverse event is defined as an AE that occurred or worsened following first administration of study drug.
Rando = Randomisation; PP = Piperaquine phosphate; (d) = Day from IP dose.
AE No. = Adverse Event Number; TEAE = A treatment-emergent adverse event; SAE = Serious adverse event; UN = Unknown; Dis = Discontinuation

Listing 16.2.7.1
Adverse Events

Rando Number	Challenge Agent	AE No	System Organ Class/ Preferred Term/ Adverse Event Verbatim	Onset Date/ Onset Time/ (d) End Date/ End Time/ (d)	TEAE/ SAE	Severity/ Action Taken/ Outcome	Treatment Directly Related to AE (Inoculum/ Artesunate/ Piperaquine/ Malarone/ Primacin/ Mosquito)	Time of onset Relative to Inoculum/ Time of onset Relative to Artesunate/ Time of onset Relative to PP	Duration (hours)	Dis
R202	K13	3	General disorders and administration site conditions/ Pyrexia/ fever	03-MAR-2018/ 09:42/ (4)/ 03-MAR-2018/ 11:06/ (4)	Yes/ No	MILD/ MEDICATION: ibuprofen/ RECOVERED/ RESOLVED	Probable/ Unrelated/ Unrelated/ Unrelated/ Unrelated/ Unrelated	12 days post/ 2.9 days post/ 0.8 days post	1.40	No
		4	Investigations/ Alanine aminotransferase increased/ alanine transaminase increased	09-MAR-2018/ 09:32/ (10)/ 12-MAR-2018/ 09:51/ (13)	Yes/ No	MODERATE/ NONE/ RECOVERED/ RESOLVED	Probable/ Unrelated/ Unrelated/ Unrelated/ Unrelated/ Unrelated	18 days post/ 8.9 days post/ 6.8 days post	72.32	No
		5	Injury, poisoning and procedural complications/ Arthropod bite/ Mosquito bite reaction	14-MAR-2018/ UN:UN/ (15)/ 18-MAR-2018/ UN:UN/ (19)	Yes/ No	MILD/ MEDICATION: Mometasone/ RECOVERED/ RESOLVED	Unrelated/ Unrelated/ Unrelated/ Unrelated/ Unrelated/ Probable	22.6 days post/ 13.5 days post/ 11.4 days post		No
R203	K13	1	Nervous system disorders/ Headache/ Intermittent headache	01-MAR-2018/ 07:00/ (2)/ 11-MAR-2018/ 07:00/ (12)	Yes/ No	MILD/ MEDICATION: ibuprofen and paracetamol/ RECOVERED/ RESOLVED	Probable/ Unrelated/ Unrelated/ Unrelated/ Unrelated/ Unrelated	9.9 days post/ 0.8 days post/ -1.3 days pre	240.00	No

Notes: Adverse events were coded using the Medical Dictionary for Regulatory Activities (MedDRA) Version 20.1.
A treatment-emergent adverse event is defined as an AE that occurred or worsened following first administration of study drug.
Rando = Randomisation; PP = Piperaquine phosphate; (d) = Day from IP dose.
AE No. = Adverse Event Number; TEAE = A treatment-emergent adverse event; SAE = Serious adverse event; UN = Unknown; Dis = Discontinuation

Listing 16.2.7.1
Adverse Events

Rando Number	Challenge Agent	AE No	System Organ Class/ Preferred Term/ Adverse Event Verbatim	Onset Date/ Onset Time/ (d) End Date/ End Time/ (d)	TEAE/ SAE	Severity/ Action Taken/ Outcome	Treatment Directly Related to AE (Inoculum/ Artesunate/ Piperaquine/ Malarone/ Primacin/ Mosquito)	Time of onset Relative to Inoculum/ Time of onset Relative to Artesunate/ Time of onset Relative to PP	Duration (hours)	Dis
R203	K13	2	Gastrointestinal disorders/ Nausea/ nausea	01-MAR-2018/ 09:10/ (2)/ 01-MAR-2018/ 12:00/ (2)	Yes/ No	MILD/ MEDICATION: ondansetron/ RECOVERED/ RESOLVED	Probable/ Unrelated/ Unrelated/ Unrelated/ Unrelated/ Unrelated	9.9 days post/ 0.9 days post/ -1.2 days pre	2.83	No
		3	Musculoskeletal and connective tissue disorders/ Myalgia/ Myalgia	03-MAR-2018/ 07:00/ (4)/ 03-MAR-2018/ 22:00/ (4)	Yes/ No	MILD/ MEDICATION: ibuprofen and paracetamol/ RECOVERED/ RESOLVED	Probable/ Unrelated/ Unrelated/ Unrelated/ Unrelated/ Unrelated	11.9 days post/ 2.8 days post/ 0.7 days post	15.00	No
		4	Gastrointestinal disorders/ Nausea/ Intermittent nausea	03-MAR-2018/ 12:30/ (4)/ 06-MAR-2018/ 06:30/ (7)	Yes/ No	MILD/ MEDICATION: ondansetron/ RECOVERED/ RESOLVED	Probable/ Unrelated/ Unrelated/ Unrelated/ Unrelated/ Unrelated	12.1 days post/ 3 days post/ 1 days post	66.00	No
		5	General disorders and administration site conditions/ Vessel puncture site bruise/ bruising right forearm due to venepuncture	03-MAR-2018/ 15:00/ (4)/ 19-MAR-2018/ 07:00/ (20)	Yes/ No	MILD/ NONE/ RECOVERED/ RESOLVED	Unrelated/ Unrelated/ Unrelated/ Unrelated/ Unrelated/ Unrelated	12.2 days post/ 3.1 days post/ 1.1 days post	376.00	No

Notes: Adverse events were coded using the Medical Dictionary for Regulatory Activities (MedDRA) Version 20.1.
A treatment-emergent adverse event is defined as an AE that occurred or worsened following first administration of study drug.
Rando = Randomisation; PP = Piperaquine phosphate; (d) = Day from IP dose.
AE No. = Adverse Event Number; TEAE = A treatment-emergent adverse event; SAE = Serious adverse event; UN = Unknown; Dis = Discontinuation

Listing 16.2.7.1
Adverse Events

Rando Number	Challenge Agent	AE No	System Organ Class/ Preferred Term/ Adverse Event Verbatim	Onset Date/ Onset Time/ (d) End Date/ End Time/ (d)	TEAE/ SAE	Severity/ Action Taken/ Outcome	Treatment Directly Related to AE (Inoculum/ Artesunate/ Piperaquine/ Malarone/ Primacin/ Mosquito)	Time of onset Relative to Inoculum/ Time of onset Relative to Artesunate/ Time of onset Relative to PP	Duration (hours)	Dis
R203	K13	6	General disorders and administration site conditions/ Chills/ chills	03-MAR-2018/ 17:00/ (4)/ 04-MAR-2018/ 06:00/ (5)	Yes/ No	MILD/ MEDICATION: ibuprfen and paracetamol/ RECOVERED/ RESOLVED	Probable/ Unrelated/ Unrelated/ Unrelated/ Unrelated/ Unrelated	12.3 days post/ 3.2 days post/ 1.1 days post	13.00	No
		7	Nervous system disorders/ Paraesthesia/ Paresthesia left fingers	03-MAR-2018/ 17:00/ (4)/ 04-MAR-2018/ 14:00/ (5)	Yes/ No	MILD/ NONE/ RECOVERED/ RESOLVED	Unrelated/ Unrelated/ Unrelated/ Unrelated/ Unrelated/ Unrelated	12.3 days post/ 3.2 days post/ 1.1 days post	21.00	No
		8	General disorders and administration site conditions/ Pyrexia/ fever	03-MAR-2018/ 20:54/ (4)/ 04-MAR-2018/ 08:45/ (5)	Yes/ No	MILD/ MEDICATION: ibuprofen and paracetamol/ RECOVERED/ RESOLVED	Probable/ Unrelated/ Unrelated/ Unrelated/ Unrelated/ Unrelated	12.4 days post/ 3.4 days post/ 1.3 days post	11.85	No
		9	General disorders and administration site conditions/ Malaise/ Malaise	04-MAR-2018/ 06:00/ (5)/ 06-MAR-2018/ 06:30/ (7)	Yes/ No	MILD/ MEDICATION: Ibuprofen, paracetamol/ RECOVERED/ RESOLVED	Probable/ Unrelated/ Unrelated/ Unrelated/ Unrelated/ Unrelated	12.8 days post/ 3.8 days post/ 1.7 days post	48.50	No

Notes: Adverse events were coded using the Medical Dictionary for Regulatory Activities (MedDRA) Version 20.1.
A treatment-emergent adverse event is defined as an AE that occurred or worsened following first administration of study drug.
Rando = Randomisation; PP = Piperaquine phosphate; (d) = Day from IP dose.
AE No. = Adverse Event Number; TEAE = A treatment-emergent adverse event; SAE = Serious adverse event; UN = Unknown; Dis = Discontinuation

Listing 16.2.7.1
Adverse Events

Rando Number	Challenge Agent	AE No	System Organ Class/ Preferred Term/ Adverse Event Verbatim	Onset Date/ Onset Time/ (d) End Date/ End Time/ (d)	TEAE/ SAE	Severity/ Action Taken/ Outcome	Treatment Directly Related to AE (Inoculum/ Artesunate/ Piperaquine/ Malarone/ Primacin/ Mosquito)	Time of onset Relative to Inoculum/ Time of onset Relative to Artesunate/ Time of onset Relative to PP	Duration (hours)	Dis
R203	K13	10	Musculoskeletal and connective tissue disorders/ Myalgia/ Intermittent Myalgia	04-MAR-2018/ 08:50/ (5)/ 11-MAR-2018/ 07:00/ (12)	Yes/ No	MILD/ MEDICATION: Ibuprofen, paracetamol/ RECOVERED/ RESOLVED	Probable/ Unrelated/ Unrelated/ Unrelated/ Unrelated/ Unrelated	12.9 days post/ 3.9 days post/ 1.8 days post	166.17	No
		11	General disorders and administration site conditions/ Pyrexia/ Fever	04-MAR-2018/ 12:15/ (5)/ 04-MAR-2018/ 20:11/ (5)	Yes/ No	MILD/ NONE/ RECOVERED/ RESOLVED	Probable/ Unrelated/ Unrelated/ Unrelated/ Unrelated/ Unrelated	13.1 days post/ 4 days post/ 1.9 days post	7.93	No
		12	Musculoskeletal and connective tissue disorders/ Arthralgia/ arthralgia	04-MAR-2018/ 20:13/ (5)/ 05-MAR-2018/ 09:22/ (6)	Yes/ No	MILD/ NONE/ RECOVERED/ RESOLVED	Probable/ Unrelated/ Unrelated/ Unrelated/ Unrelated/ Unrelated	13.4 days post/ 4.4 days post/ 2.3 days post	13.15	No
		13	Investigations/ Neutrophil count decreased/ Neutrophil count decreased	05-MAR-2018/ 09:40/ (6)/ 09-MAR-2018/ 10:14/ (10)	Yes/ No	MODERATE/ NONE/ RECOVERED/ RESOLVED	Probable/ Unrelated/ Unrelated/ Unrelated/ Unrelated/ Unrelated	14 days post/ 4.9 days post/ 2.8 days post	96.57	No

Notes: Adverse events were coded using the Medical Dictionary for Regulatory Activities (MedDRA) Version 20.1.
A treatment-emergent adverse event is defined as an AE that occurred or worsened following first administration of study drug.
Rando = Randomisation; PP = Piperaquine phosphate; (d) = Day from IP dose.
AE No. = Adverse Event Number; TEAE = A treatment-emergent adverse event; SAE = Serious adverse event; UN = Unknown; Dis = Discontinuation

Listing 16.2.7.1
Adverse Events

Rando Number	Challenge Agent	AE No	System Organ Class/ Preferred Term/ Adverse Event Verbatim	Onset Date/ Onset Time/ (d) End Date/ End Time/ (d)	TEAE/ SAE	Severity/ Action Taken/ Outcome	Treatment Directly Related to AE (Inoculum/ Artesunate/ Piperaquine/ Malarone/ Primacin/ Mosquito)	Time of onset Relative to Inoculum/ Time of onset Relative to Artesunate/ Time of onset Relative to PP	Duration (hours)	Dis
R203	K13	14	Investigations/ Eosinophil count increased/ eosinophil count increased	02-APR-2018/ 09:18/ (34)/ 14-APR-2018/ 10:00/ (46)	Yes/ No	MILD/ NONE/ RECOVERED/ RESOLVED	Unlikely/ Unrelated/ Unrelated/ Probable/ Probable/ Unrelated	41.9 days post/ 32.9 days post/ 30.8 days post	288.70	No
R204	K13	1	General disorders and administration site conditions/ Vessel puncture site bruise/ Bruise left arm due to venepuncture	20-FEB-2018/ 06:00/ (-8)/ 19-MAR-2018/ 07:00/ (20)	No/ No	MILD/ NONE/ RECOVERED/ RESOLVED	Unrelated/ Unrelated/ Unrelated/ Unrelated/ Unrelated/ Unrelated	0.8 days post/ -8.3 days pre/ -10.3 days pre	649.00	No
		2	General disorders and administration site conditions/ Vessel puncture site bruise/ Bruise right arm due to venepuncture	25-FEB-2018/ 08:00/ (-3)/ 19-MAR-2018/ 08:10/ (20)	No/ No	MILD/ NONE/ RECOVERED/ RESOLVED	Unrelated/ Unrelated/ Unrelated/ Unrelated/ Unrelated/ Unrelated	5.9 days post/ -3.2 days pre/ -5.2 days pre	528.17	No
		3	Nervous system disorders/ Headache/ intermittent headache	01-MAR-2018/ 10:30/ (2)/ 01-MAR-2018/ 13:30/ (2)	Yes/ No	MILD/ MEDICATION: ibuprofen/ RECOVERED/ RESOLVED	Probable/ Unrelated/ Unrelated/ Unrelated/ Unrelated/ Unrelated	10 days post/ 0.9 days post/ -1.1 days pre	3.00	No

Notes: Adverse events were coded using the Medical Dictionary for Regulatory Activities (MedDRA) Version 20.1.
A treatment-emergent adverse event is defined as an AE that occurred or worsened following first administration of study drug.
Rando = Randomisation; PP = Piperaquine phosphate; (d) = Day from IP dose.
AE No. = Adverse Event Number; TEAE = A treatment-emergent adverse event; SAE = Serious adverse event; UN = Unknown; Dis = Discontinuation

Listing 16.2.7.1
Adverse Events

Rando Number	Challenge Agent	AE No	System Organ Class/ Preferred Term/ Adverse Event Verbatim	Onset Date/ Onset Time/ (d) End Date/ End Time/ (d)	TEAE/ SAE	Severity/ Action Taken/ Outcome	Treatment Directly Related to AE (Inoculum/ Artesunate/ Piperaquine/ Malarone/ Primacin/ Mosquito)	Time of onset Relative to Inoculum/ Time of onset Relative to Artesunate/ Time of onset Relative to PP	Duration (hours)	Dis
R204	K13	4	General disorders and administration site conditions/ Vessel puncture site haematoma/ haematoma left cubital fossa	03-MAR-2018/ 01:20/ (4)/ 19-MAR-2018/ 07:00/ (20)	Yes/ No	MILD/ NONE/ RECOVERED/ RESOLVED	Unrelated/ Unrelated/ Unrelated/ Unrelated/ Unrelated/ Unrelated	11.6 days post/ 2.5 days post/ 0.5 days post	389.67	No
		5	General disorders and administration site conditions/ Vessel puncture site haematoma/ haematoma right cubital fossa	03-MAR-2018/ 01:20/ (4)/ 21-MAR-2018/ 08:00/ (22)	Yes/ No	MILD/ NONE/ RECOVERED/ RESOLVED	Unrelated/ Unrelated/ Unrelated/ Unrelated/ Unrelated/ Unrelated	11.6 days post/ 2.5 days post/ 0.5 days post	438.67	No
		6	Investigations/ Lymphocyte count decreased/ Lymphocyte count decreased	03-MAR-2018/ 13:21/ (4)/ 09-MAR-2018/ 09:00/ (10)	Yes/ No	MODERATE/ NONE/ RECOVERED/ RESOLVED	Probable/ Unrelated/ Unrelated/ Unrelated/ Unrelated/ Unrelated	12.1 days post/ 3 days post/ 1 days post	139.65	No
		7	Investigations/ White blood cell count decreased/ White cell count decreased	03-MAR-2018/ 13:21/ (4)/ 09-MAR-2018/ 09:00/ (10)	Yes/ No	MODERATE/ NONE/ RECOVERED/ RESOLVED	Probable/ Unrelated/ Unrelated/ Unrelated/ Unrelated/ Unrelated	12.1 days post/ 3 days post/ 1 days post	139.65	No

Notes: Adverse events were coded using the Medical Dictionary for Regulatory Activities (MedDRA) Version 20.1.
A treatment-emergent adverse event is defined as an AE that occurred or worsened following first administration of study drug.
Rando = Randomisation; PP = Piperaquine phosphate; (d) = Day from IP dose.
AE No. = Adverse Event Number; TEAE = A treatment-emergent adverse event; SAE = Serious adverse event; UN = Unknown; Dis = Discontinuation

Listing 16.2.7.1
Adverse Events

Rando Number	Challenge Agent	AE No	System Organ Class/ Preferred Term/ Adverse Event Verbatim	Onset Date/ Onset Time/ (d) End Date/ End Time/ (d)	TEAE/ SAE	Severity/ Action Taken/ Outcome	Treatment Directly Related to AE (Inoculum/ Artesunate/ Piperaquine/ Malarone/ Primacin/ Mosquito)	Time of onset Relative to Inoculum/ Time of onset Relative to Artesunate/ Time of onset Relative to PP	Duration (hours)	Dis
R204	K13	8	Nervous system disorders/ Headache/ Intermittent headache	04-MAR-2018/ 13:30/ (5)/ 06-MAR-2018/ 05:30/ (7)	Yes/ No	MILD/ MEDICATION: ibuprofen/ RECOVERED/ RESOLVED	Probable/ Unrelated/ Unrelated/ Unrelated/ Unrelated/ Unrelated	13.1 days post/ 4 days post/ 2 days post	40.00	No
		9	Investigations/ Neutrophil count decreased/ Neutrophil count decreased	05-MAR-2018/ 08:37/ (6)/ 09-MAR-2018/ 09:00/ (10)	Yes/ No	MODERATE/ NONE/ RECOVERED/ RESOLVED	Probable/ Unrelated/ Unrelated/ Unrelated/ Unrelated/ Unrelated	13.9 days post/ 4.8 days post/ 2.8 days post	96.38	No
		10	Metabolism and nutrition disorders/ Decreased appetite/ loss of appetite	19-MAR-2018/ 15:00/ (20)/ 25-MAR-2018/ 19:00/ (26)	Yes/ No	MILD/ NONE/ RECOVERED/ RESOLVED	Unrelated/ Unrelated/ Unrelated/ Probable/ Unrelated/ Unrelated	28.2 days post/ 19.1 days post/ 17 days post	148.00	No
		11	Psychiatric disorders/ Insomnia/ insomnia	19-MAR-2018/ 19:00/ (20)/ 25-MAR-2018/ 19:00/ (26)	Yes/ No	MILD/ NONE/ RECOVERED/ RESOLVED	Unrelated/ Unrelated/ Unrelated/ Probable/ Unrelated/ Unrelated	28.4 days post/ 19.2 days post/ 17.2 days post	144.00	No

Notes: Adverse events were coded using the Medical Dictionary for Regulatory Activities (MedDRA) Version 20.1.
A treatment-emergent adverse event is defined as an AE that occurred or worsened following first administration of study drug.
Rando = Randomisation; PP = Piperaquine phosphate; (d) = Day from IP dose.
AE No. = Adverse Event Number; TEAE = A treatment-emergent adverse event; SAE = Serious adverse event; UN = Unknown; Dis = Discontinuation

Listing 16.2.7.1
Adverse Events

Rando Number	Challenge Agent	AE No	System Organ Class/ Preferred Term/ Adverse Event Verbatim	Onset Date/ Onset Time/ (d) End Date/ End Time/ (d)	TEAE/ SAE	Severity/ Action Taken/ Outcome	Treatment Directly Related to AE (Inoculum/ Artesunate/ Piperaquine/ Malarone/ Primacin/ Mosquito)	Time of onset Relative to Inoculum/ Time of onset Relative to Artesunate/ Time of onset Relative to PP	Duration (hours)	Dis
R205	K13	1	Musculoskeletal and connective tissue disorders/ Musculoskeletal pain/ Bilateral shoulder pain	19-FEB-2018/ 18:00/ (-9)/ 20-FEB-2018/ 06:00/ (-8)	No/ No	MILD/ NONE/ RECOVERED/ RESOLVED	Unrelated/ Unrelated/ Unrelated/ Unrelated/ Unrelated/ Unrelated	0.3 days post/ -8.8 days pre/ -10.8 days pre	12.00	No
		2	Musculoskeletal and connective tissue disorders/ Back pain/ lower back pain	28-FEB-2018/ 22:00/ (1)/ 02-MAR-2018/ 10:00/ (3)	Yes/ No	MILD/ MEDICATION: ibuprofen/ RECOVERED/ RESOLVED	Probable/ Unrelated/ Unrelated/ Unrelated/ Unrelated/ Unrelated	9.5 days post/ 0.4 days post/ -1.7 days pre	36.00	No
		3	General disorders and administration site conditions/ Pyrexia/ fever	01-MAR-2018/ 13:49/ (2)/ 01-MAR-2018/ 13:51/ (2)	Yes/ No	MILD/ NONE/ RECOVERED/ RESOLVED	Probable/ Unrelated/ Unrelated/ Unrelated/ Unrelated/ Unrelated	10.1 days post/ 1 days post/ -1 days pre	0.03	No
		4	Nervous system disorders/ Headache/ intermittent headache	01-MAR-2018/ 17:18/ (2)/ 02-MAR-2018/ 10:00/ (3)	Yes/ No	MILD/ MEDICATION: paracetamol/ RECOVERED/ RESOLVED	Probable/ Unrelated/ Unrelated/ Unrelated/ Unrelated/ Unrelated	10.3 days post/ 1.2 days post/ -0.9 days pre	16.70	No

Notes: Adverse events were coded using the Medical Dictionary for Regulatory Activities (MedDRA) Version 20.1.
A treatment-emergent adverse event is defined as an AE that occurred or worsened following first administration of study drug.
Rando = Randomisation; PP = Piperaquine phosphate; (d) = Day from IP dose.
AE No. = Adverse Event Number; TEAE = A treatment-emergent adverse event; SAE = Serious adverse event; UN = Unknown; Dis = Discontinuation

Listing 16.2.7.1
Adverse Events

Rando Number	Challenge Agent	AE No	System Organ Class/ Preferred Term/ Adverse Event Verbatim	Onset Date/ Onset Time/ (d) End Date/ End Time/ (d)	TEAE/ SAE	Severity/ Action Taken/ Outcome	Treatment Directly Related to AE (Inoculum/ Artesunate/ Piperaquine/ Malarone/ Primacin/ Mosquito)	Time of onset Relative to Inoculum/ Time of onset Relative to Artesunate/ Time of onset Relative to PP	Duration (hours)	Dis
R205	K13	5	Skin and subcutaneous tissue disorders/ Hyperhidrosis/ sweats	01-MAR-2018/ 18:30/ (2)/ 02-MAR-2018/ 10:00/ (3)	Yes/ No	MILD/ NONE/ RECOVERED/ RESOLVED	Probable/ Unrelated/ Unrelated/ Unrelated/ Unrelated/ Unrelated	10.3 days post/ 1.2 days post/ -0.8 days pre	15.50	No
		6	Investigations/ Neutrophil count decreased/ neutrophil count decreased	02-MAR-2018/ 13:11/ (3)/ 09-MAR-2018/ 07:52/ (10)	Yes/ No	MODERATE/ NONE/ RECOVERED/ RESOLVED	Probable/ Unrelated/ Unrelated/ Unrelated/ Unrelated/ Unrelated	11.1 days post/ 2 days post/ 0 days post	162.68	No
		7	Investigations/ White blood cell count decreased/ white cell count decreased	02-MAR-2018/ 13:11/ (3)/ 09-MAR-2018/ 07:52/ (10)	Yes/ No	MODERATE/ NONE/ RECOVERED/ RESOLVED	Probable/ Unrelated/ Unrelated/ Unrelated/ Unrelated/ Unrelated	11.1 days post/ 2 days post/ 0 days post	162.68	No
		8	Skin and subcutaneous tissue disorders/ Hyperhidrosis/ intermittent sweats	04-MAR-2018/ 00:00/ (5)/ 06-MAR-2018/ 20:00/ (7)	Yes/ No	MILD/ NONE/ RECOVERED/ RESOLVED	Probable/ Unrelated/ Unrelated/ Unrelated/ Unrelated/ Unrelated	12.6 days post/ 3.5 days post/ 1.4 days post	68.00	No

Notes: Adverse events were coded using the Medical Dictionary for Regulatory Activities (MedDRA) Version 20.1.
A treatment-emergent adverse event is defined as an AE that occurred or worsened following first administration of study drug.
Rando = Randomisation; PP = Piperaquine phosphate; (d) = Day from IP dose.
AE No. = Adverse Event Number; TEAE = A treatment-emergent adverse event; SAE = Serious adverse event; UN = Unknown; Dis = Discontinuation

Listing 16.2.7.1
Adverse Events

Rando Number	Challenge Agent	AE No	System Organ Class/ Preferred Term/ Adverse Event Verbatim	Onset Date/ Onset Time/ (d) End Date/ End Time/ (d)	TEAE/ SAE	Severity/ Action Taken/ Outcome	Treatment Directly Related to AE (Inoculum/ Artesunate/ Piperaquine/ Malarone/ Primacin/ Mosquito)	Time of onset Relative to Inoculum/ Time of onset Relative to Artesunate/ Time of onset Relative to PP	Duration (hours)	Dis
R205	K13	9	Injury, poisoning and procedural complications/ Arthropod bite/ Mosquito bites reaction	14-MAR-2018/ UN:UN/ (15)/ 14-MAR-2018/ UN:UN/ (15)	Yes/ No	MILD/ MEDICATION: Mometasone/ RECOVERED/ RESOLVED	Unrelated/ Unrelated/ Unrelated/ Unrelated/ Unrelated/ Probable	22.6 days post/ 13.5 days post/ 11.4 days post		No
R206	K13	1	Nervous system disorders/ Headache/ headache	19-FEB-2018/ 12:00/ (-9)/ 19-FEB-2018/ 12:10/ (-9)	No/ No	MILD/ NONE/ RECOVERED/ RESOLVED	Unrelated/ Unrelated/ Unrelated/ Unrelated/ Unrelated/ Unrelated	0.1 days post/ -9.1 days pre/ -11.1 days pre	0.17	No
		2	Infections and infestations/ Upper respiratory tract infection/ Upper respiratory tract infection	26-FEB-2018/ 12:17/ (-2)/ 28-FEB-2018/ 06:00/ (1)	No/ No	MODERATE/ MEDICATION: Ibuprofen/ RECOVERED/ RESOLVED	Unrelated/ Unrelated/ Unrelated/ Unrelated/ Unrelated/ Unrelated	7.1 days post/ -2 days pre/ -4.1 days pre	41.72	No
		3	Musculoskeletal and connective tissue disorders/ Myalgia/ intermittent myalgia	28-FEB-2018/ 09:30/ (1)/ 03-MAR-2018/ 07:00/ (4)	No/ No	MILD/ MEDICATION: Ibuprofen, paracetamol/ RECOVERED/ RESOLVED	Probable/ Unrelated/ Unrelated/ Unrelated/ Unrelated/ Unrelated	9 days post/ -0.2 days pre/ -2.2 days pre	69.50	No

Notes: Adverse events were coded using the Medical Dictionary for Regulatory Activities (MedDRA) Version 20.1.
A treatment-emergent adverse event is defined as an AE that occurred or worsened following first administration of study drug.
Rando = Randomisation; PP = Piperaquine phosphate; (d) = Day from IP dose.
AE No. = Adverse Event Number; TEAE = A treatment-emergent adverse event; SAE = Serious adverse event; UN = Unknown; Dis = Discontinuation

Listing 16.2.7.1
Adverse Events

Rando Number	Challenge Agent	AE No	System Organ Class/ Preferred Term/ Adverse Event Verbatim	Onset Date/ Onset Time/ (d) End Date/ End Time/ (d)	TEAE/ SAE	Severity/ Action Taken/ Outcome	Treatment Directly Related to AE (Inoculum/ Artesunate/ Piperaquine/ Malarone/ Primacin/ Mosquito)	Time of onset Relative to Inoculum/ Time of onset Relative to Artesunate/ Time of onset Relative to PP	Duration (hours)	Dis
R206	K13	4	Nervous system disorders/ Headache/ intermittent headache	28-FEB-2018/ 09:46/ (1)/ 19-MAR-2018/ 07:00/ (20)	No/ No	MILD/ MEDICATION: Ibuprofen, paracetamol/ RECOVERED/ RESOLVED	Probable/ Unrelated/ Unrelated/ Unrelated/ Unrelated/ Unrelated	9 days post/ -0.2 days pre/ -2.2 days pre	453.23	No
		5	Investigations/ Lymphocyte count decreased/ lymphocyte count decreased	28-FEB-2018/ 12:48/ (1)/ 05-MAR-2018/ 09:30/ (6)	No/ No	MODERATE/ NONE/ RECOVERED/ RESOLVED	Probable/ Unrelated/ Unrelated/ Unrelated/ Unrelated/ Unrelated	9.1 days post/ 0 days post/ -2 days pre	116.70	No
		6	General disorders and administration site conditions/ Pyrexia/ Fever	01-MAR-2018/ 09:38/ (2)/ 01-MAR-2018/ 09:39/ (2)	Yes/ No	MILD/ NONE/ RECOVERED/ RESOLVED	Probable/ Unrelated/ Unrelated/ Unrelated/ Unrelated/ Unrelated	10 days post/ 0.8 days post/ -1.2 days pre	0.02	No
		7	Investigations/ Neutrophil count decreased/ neutrophil count decreased	02-MAR-2018/ 13:29/ (3)/ 05-MAR-2018/ 09:30/ (6)	Yes/ No	SEVERE/ NONE/ RECOVERED/ RESOLVED	Probable/ Unlikely/ Unrelated/ Unrelated/ Unrelated/ Unrelated	11.1 days post/ 2 days post/ 0 days post	68.02	No

Notes: Adverse events were coded using the Medical Dictionary for Regulatory Activities (MedDRA) Version 20.1.
A treatment-emergent adverse event is defined as an AE that occurred or worsened following first administration of study drug.
Rando = Randomisation; PP = Piperaquine phosphate; (d) = Day from IP dose.
AE No. = Adverse Event Number; TEAE = A treatment-emergent adverse event; SAE = Serious adverse event; UN = Unknown; Dis = Discontinuation

Listing 16.2.7.1
Adverse Events

Rando Number	Challenge Agent	AE No	System Organ Class/ Preferred Term/ Adverse Event Verbatim	Onset Date/ Onset Time/ (d) End Date/ End Time/ (d)	TEAE/ SAE	Severity/ Action Taken/ Outcome	Treatment Directly Related to AE (Inoculum/ Artesunate/ Piperaquine/ Malarone/ Primacin/ Mosquito)	Time of onset Relative to Inoculum/ Time of onset Relative to Artesunate/ Time of onset Relative to PP	Duration (hours)	Dis
R206	K13	8	Investigations/ White blood cell count decreased/ white cell count decreased	02-MAR-2018/ 13:29/ (3)/ 05-MAR-2018/ 09:30/ (6)	Yes/ No	SEVERE/ NONE/ RECOVERED/ RESOLVED	Probable/ Unrelated/ Unrelated/ Unrelated/ Unrelated/ Unrelated	11.1 days post/ 2 days post/ 0 days post	68.02	No
		9	Gastrointestinal disorders/ Nausea/ Nausea	02-MAR-2018/ 14:00/ (3)/ 02-MAR-2018/ 19:00/ (3)	Yes/ No	MILD/ NONE/ RECOVERED/ RESOLVED	Probable/ Unrelated/ Unrelated/ Unrelated/ Unrelated/ Unrelated	11.1 days post/ 2 days post/ 0 days post	5.00	No
		10	Gastrointestinal disorders/ Abdominal discomfort/ intermittent abdominal discomfort	02-MAR-2018/ 21:00/ (3)/ 03-MAR-2018/ 21:00/ (4)	Yes/ No	MILD/ NONE/ RECOVERED/ RESOLVED	Probable/ Unrelated/ Unrelated/ Unrelated/ Unrelated/ Unrelated	11.4 days post/ 2.3 days post/ 0.3 days post	24.00	No
		11	Musculoskeletal and connective tissue disorders/ Myalgia/ intermittent myalgia	11-MAR-2018/ 20:00/ (12)/ 13-MAR-2018/ 06:00/ (14)	Yes/ No	MILD/ MEDICATION: ibuprofen/ RECOVERED/ RESOLVED	Probable/ Unrelated/ Unrelated/ Unrelated/ Unrelated/ Unrelated	20.4 days post/ 11.3 days post/ 9.3 days post	34.00	No

Notes: Adverse events were coded using the Medical Dictionary for Regulatory Activities (MedDRA) Version 20.1.
A treatment-emergent adverse event is defined as an AE that occurred or worsened following first administration of study drug.
Rando = Randomisation; PP = Piperaquine phosphate; (d) = Day from IP dose.
AE No. = Adverse Event Number; TEAE = A treatment-emergent adverse event; SAE = Serious adverse event; UN = Unknown; Dis = Discontinuation

Listing 16.2.7.1
Adverse Events

Rando Number	Challenge Agent	AE No	System Organ Class/ Preferred Term/ Adverse Event Verbatim	Onset Date/ Onset Time/ (d) End Date/ End Time/ (d)	TEAE/ SAE	Severity/ Action Taken/ Outcome	Treatment Directly Related to AE (Inoculum/ Artesunate/ Piperaquine/ Malarone/ Primacin/ Mosquito)	Time of onset Relative to Inoculum/ Time of onset Relative to Artesunate/ Time of onset Relative to PP	Duration (hours)	Dis
R206	K13	12	Injury, poisoning and procedural complications/ Arthropod bite/ Mosquito bite reaction - itchiness	14-MAR-2018/ 20:00/ (15)/ 02-APR-2018/ 20:00/ (34)	Yes/ No	MILD/ MEDICATION: Mometasone 0.1%/ RECOVERED/ RESOLVED	Unrelated/ Unrelated/ Unrelated/ Unrelated/ Unrelated/ Probable	23.4 days post/ 14.3 days post/ 12.3 days post	456.00	No
		13	Gastrointestinal disorders/ Nausea/ intermittent nausea	19-MAR-2018/ 10:20/ (20)/ 21-MAR-2018/ 08:00/ (22)	Yes/ No	MILD/ NONE/ RECOVERED/ RESOLVED	Unrelated/ Unrelated/ Unrelated/ Probable/ Unrelated/ Unrelated	28 days post/ 18.9 days post/ 16.9 days post	45.67	No
		14	Gastrointestinal disorders/ Diarrhoea/ diarrhoea	20-MAR-2018/ 19:00/ (21)/ 20-MAR-2018/ 21:00/ (21)	Yes/ No	MILD/ NONE/ RECOVERED/ RESOLVED	Unrelated/ Unrelated/ Unrelated/ Probable/ Unrelated/ Unrelated	29.3 days post/ 20.2 days post/ 18.2 days post	2.00	No
R207	K13	1	General disorders and administration site conditions/ Vessel puncture site bruise/ Bruise left forearm due to venepuncture	20-FEB-2018/ 07:00/ (-8)/ 11-MAR-2018/ 08:00/ (12)	No/ No	MILD/ NONE/ RECOVERED/ RESOLVED	Unrelated/ Unrelated/ Unrelated/ Unrelated/ Unrelated/ Unrelated	0.8 days post/ -8.2 days pre/ -10.3 days pre	457.00	No

Notes: Adverse events were coded using the Medical Dictionary for Regulatory Activities (MedDRA) Version 20.1.
A treatment-emergent adverse event is defined as an AE that occurred or worsened following first administration of study drug.
Rando = Randomisation; PP = Piperaquine phosphate; (d) = Day from IP dose.
AE No. = Adverse Event Number; TEAE = A treatment-emergent adverse event; SAE = Serious adverse event; UN = Unknown; Dis = Discontinuation

Listing 16.2.7.1
Adverse Events

Rando Number	Challenge Agent	AE No	System Organ Class/ Preferred Term/ Adverse Event Verbatim	Onset Date/ Onset Time/ (d) End Date/ End Time/ (d)	TEAE/ SAE	Severity/ Action Taken/ Outcome	Treatment Directly Related to AE (Inoculum/ Artesunate/ Piperaquine/ Malarone/ Primacin/ Mosquito)	Time of onset Relative to Inoculum/ Time of onset Relative to Artesunate/ Time of onset Relative to PP	Duration (hours)	Dis
R207	K13	2	Nervous system disorders/ Headache/ intermittent headache	01-MAR-2018/ 07:00/ (2)/ 03-MAR-2018/ 16:15/ (4)	Yes/ No	MILD/ MEDICATION: ibuprofen/ RECOVERED/ RESOLVED	Probable/ Unrelated/ Unrelated/ Unrelated/ Unrelated/ Unrelated	9.8 days post/ 0.8 days post/ -1.3 days pre	57.25	No
		3	General disorders and administration site conditions/ Pyrexia/ fever	01-MAR-2018/ 08:53/ (2)/ 01-MAR-2018/ 08:55/ (2)	Yes/ No	MILD/ NONE/ RECOVERED/ RESOLVED	Probable/ Unrelated/ Unrelated/ Unrelated/ Unrelated/ Unrelated	9.9 days post/ 0.8 days post/ -1.2 days pre	0.03	No
		4	Investigations/ Lymphocyte count decreased/ lymphocyte count decreased	02-MAR-2018/ 12:59/ (3)/ 03-MAR-2018/ 13:00/ (4)	Yes/ No	MODERATE/ NONE/ RECOVERED/ RESOLVED	Probable/ Unrelated/ Unrelated/ Unrelated/ Unrelated/ Unrelated	11.1 days post/ 2 days post/ 0 days post	24.02	No
		5	Musculoskeletal and connective tissue disorders/ Arthralgia/ arthralgia	03-MAR-2018/ 09:15/ (4)/ 03-MAR-2018/ 16:00/ (4)	Yes/ No	MILD/ NONE/ RECOVERED/ RESOLVED	Probable/ Unrelated/ Unrelated/ Unrelated/ Unrelated/ Unrelated	11.9 days post/ 2.8 days post/ 0.8 days post	6.75	No

Notes: Adverse events were coded using the Medical Dictionary for Regulatory Activities (MedDRA) Version 20.1.
A treatment-emergent adverse event is defined as an AE that occurred or worsened following first administration of study drug.
Rando = Randomisation; PP = Piperaquine phosphate; (d) = Day from IP dose.
AE No. = Adverse Event Number; TEAE = A treatment-emergent adverse event; SAE = Serious adverse event; UN = Unknown; Dis = Discontinuation

Listing 16.2.7.1
Adverse Events

Rando Number	Challenge Agent	AE No	System Organ Class/ Preferred Term/ Adverse Event Verbatim	Onset Date/ Onset Time/ (d) End Date/ End Time/ (d)	TEAE/ SAE	Severity/ Action Taken/ Outcome	Treatment Directly Related to AE (Inoculum/ Artesunate/ Piperaquine/ Malarone/ Primacin/ Mosquito)	Time of onset Relative to Inoculum/ Time of onset Relative to Artesunate/ Time of onset Relative to PP	Duration (hours)	Dis
R207	K13	6	Injury, poisoning and procedural complications/ Arthropod bite/ mosquito bites - itchiness	15-MAR-2018/ 20:00/ (16)/ 16-MAR-2018/ 08:00/ (17)	Yes/ No	MILD/ MEDICATION: mometasome cream/ RECOVERED/ RESOLVED	Unrelated/ Unrelated/ Unrelated/ Unrelated/ Unrelated/ Probable	24.4 days post/ 15.3 days post/ 13.3 days post	12.00	No
R208	3D7	1	General disorders and administration site conditions/ Pyrexia/ fever	28-FEB-2018/ 19:59/ (1)/ 28-FEB-2018/ 20:01/ (1)	Yes/ No	MILD/ NONE/ RECOVERED/ RESOLVED	Probable/ Unrelated/ Unrelated/ Unrelated/ Unrelated/ Unrelated	8.4 days post/ 0.4 days post/ -9.5 days pre	0.03	No
		2	Investigations/ Alanine aminotransferase increased/ alanine transaminase increased	03-MAR-2018/ 11:36/ (4)/ 05-MAR-2018/ 07:07/ (6)	Yes/ No	MODERATE/ NONE/ NOT RECOVERED/ NOT RESOLVED	Probable/ Unrelated/ Unrelated/ Unrelated/ Unrelated/ Unrelated	11.1 days post/ 3 days post/ -6.9 days pre	43.52	No
		3	Investigations/ Aspartate aminotransferase increased/ Aspartate transaminase increased	05-MAR-2018/ 07:07/ (6)/ 09-MAR-2018/ 07:19/ (10)	Yes/ No	MODERATE/ NONE/ RECOVERED/ RESOLVED	Probable/ Unrelated/ Unrelated/ Unrelated/ Unrelated/ Unrelated	12.9 days post/ 4.8 days post/ -5 days pre	96.20	No

Notes: Adverse events were coded using the Medical Dictionary for Regulatory Activities (MedDRA) Version 20.1.
A treatment-emergent adverse event is defined as an AE that occurred or worsened following first administration of study drug.
Rando = Randomisation; PP = Piperaquine phosphate; (d) = Day from IP dose.
AE No. = Adverse Event Number; TEAE = A treatment-emergent adverse event; SAE = Serious adverse event; UN = Unknown; Dis = Discontinuation

Listing 16.2.7.1
Adverse Events

Rando Number	Challenge Agent	AE No	System Organ Class/ Preferred Term/ Adverse Event Verbatim	Onset Date/ Onset Time/ (d) End Date/ End Time/ (d)	TEAE/ SAE	Severity/ Action Taken/ Outcome	Treatment Directly Related to AE (Inoculum/ Artesunate/ Piperaquine/ Malarone/ Primacin/ Mosquito)	Time of onset Relative to Inoculum/ Time of onset Relative to Artesunate/ Time of onset Relative to PP	Duration (hours)	Dis
R208	3D7	4	Investigations/ Alanine aminotransferase increased/ Alanine transaminase increased	05-MAR-2018/ 07:07/ (6)/ 19-MAR-2018/ 07:50/ (20)	Yes/ No	SEVERE/ NONE/ RECOVERED/ RESOLVED	Probable/ Unrelated/ Unrelated/ Unrelated/ Unrelated/ Unrelated	12.9 days post/ 4.8 days post/ -5 days pre	336.72	No
		5	Investigations/ Aspartate aminotransferase increased/ aspartate transaminase increased	10-MAR-2018/ 07:52/ (11)/ 13-MAR-2018/ 07:22/ (14)	Yes/ No	MODERATE/ NONE/ RECOVERED/ RESOLVED	Probable/ Unrelated/ Unrelated/ Unrelated/ Unrelated/ Unrelated	17.9 days post/ 9.8 days post/ 0 days post	71.50	No
		6	Investigations/ Alanine aminotransferase increased/ Alanine transaminase increased	19-MAR-2018/ 07:01/ (20)/ 22-MAR-2018/ 07:50/ (23)	Yes/ No	MODERATE/ NONE/ RECOVERED/ RESOLVED	Probable/ Unrelated/ Unrelated/ Unrelated/ Unrelated/ Unrelated	26.9 days post/ 18.8 days post/ 9 days post	72.82	No
R209	3D7	1	Cardiac disorders/ Ventricular extrasystoles/ Ventricular bigeminy	28-FEB-2018/ 09:51/ Ongoing	No/ No	MODERATE/ NONE/ RECOVERING/ RESOLVING	Possible/ Unrelated/ Unrelated/ Unrelated/ Unrelated/ Unrelated	8 days post/ -0.2 days pre/ -8 days pre		No

Notes: Adverse events were coded using the Medical Dictionary for Regulatory Activities (MedDRA) Version 20.1.
A treatment-emergent adverse event is defined as an AE that occurred or worsened following first administration of study drug.
Rando = Randomisation; PP = Piperaquine phosphate; (d) = Day from IP dose.
AE No. = Adverse Event Number; TEAE = A treatment-emergent adverse event; SAE = Serious adverse event; UN = Unknown; Dis = Discontinuation

Listing 16.2.7.1
Adverse Events

Rando Number	Challenge Agent	AE No	System Organ Class/ Preferred Term/ Adverse Event Verbatim	Onset Date/ Onset Time/ (d) End Date/ End Time/ (d)	TEAE/ SAE	Severity/ Action Taken/ Outcome	Treatment Directly Related to AE (Inoculum/ Artesunate/ Piperaquine/ Malarone/ Primacin/ Mosquito)	Time of onset Relative to Inoculum/ Time of onset Relative to Artesunate/ Time of onset Relative to PP	Duration (hours)	Dis
R209	3D7	2	Gastrointestinal disorders/ Nausea/ Intermittent nausea	28-FEB-2018/ 19:30/ (1)/ 02-MAR-2018/ 15:00/ (3)	Yes/ No	MILD/ NONE/ RECOVERED/ RESOLVED	Probable/ Unrelated/ Unrelated/ Unrelated/ Unrelated/ Unrelated	8.4 days post/ 0.2 days post/ -7.6 days pre	43.50	No
		3	Cardiac disorders/ Tachycardia/ tachycardia	28-FEB-2018/ 23:21/ (1)/ 28-FEB-2018/ 23:22/ (1)	Yes/ No	MODERATE/ NONE/ RECOVERED/ RESOLVED	Possible/ Unrelated/ Unrelated/ Unrelated/ Unrelated/ Unrelated	8.5 days post/ 0.3 days post/ -7.4 days pre	0.02	No
		4	Nervous system disorders/ Headache/ Intermittent headache	28-FEB-2018/ 23:30/ (1)/ 01-MAR-2018/ 12:19/ (2)	Yes/ No	MILD/ MEDICATION: Ibuprofen, paracetamol/ NOT RECOVERED/ NOT RESOLVED	Probable/ Unrelated/ Unrelated/ Unrelated/ Unrelated/ Unrelated	8.5 days post/ 0.3 days post/ -7.4 days pre	12.82	No
		5	Nervous system disorders/ Headache/ headache	01-MAR-2018/ 12:19/ (2)/ 01-MAR-2018/ 20:06/ (2)	Yes/ No	MODERATE/ NONE/ RECOVERING/ RESOLVING	Probable/ Unrelated/ Unrelated/ Unrelated/ Unrelated/ Unrelated	9.1 days post/ 0.9 days post/ -6.9 days pre	7.78	No

Notes: Adverse events were coded using the Medical Dictionary for Regulatory Activities (MedDRA) Version 20.1.
A treatment-emergent adverse event is defined as an AE that occurred or worsened following first administration of study drug.
Rando = Randomisation; PP = Piperaquine phosphate; (d) = Day from IP dose.
AE No. = Adverse Event Number; TEAE = A treatment-emergent adverse event; SAE = Serious adverse event; UN = Unknown; Dis = Discontinuation

Listing 16.2.7.1
Adverse Events

Rando Number	Challenge Agent	AE No	System Organ Class/ Preferred Term/ Adverse Event Verbatim	Onset Date/ Onset Time/ (d) End Date/ End Time/ (d)	TEAE/ SAE	Severity/ Action Taken/ Outcome	Treatment Directly Related to AE (Inoculum/ Artesunate/ Piperaquine/ Malarone/ Primacin/ Mosquito)	Time of onset Relative to Inoculum/ Time of onset Relative to Artesunate/ Time of onset Relative to PP	Duration (hours)	Dis
R209	3D7	6	Cardiac disorders/ Tachycardia/ tachycardia	01-MAR-2018/ 12:20/ (2)/ 01-MAR-2018/ 14:32/ (2)	Yes/ No	MODERATE/ NONE/ RECOVERED/ RESOLVED	Probable/ Unrelated/ Unrelated/ Unrelated/ Unrelated/ Unrelated	9.1 days post/ 0.9 days post/ -6.9 days pre	2.20	No
		7	Nervous system disorders/ Headache/ intermittent headache	01-MAR-2018/ 20:06/ (2)/ 13-MAR-2018/ 09:00/ (14)	Yes/ No	MILD/ MEDICATION: ibuprofen/ RECOVERED/ RESOLVED	Probable/ Unrelated/ Unrelated/ Unrelated/ Unrelated/ Unrelated	9.4 days post/ 1.2 days post/ -6.5 days pre	276.90	No
		8	Skin and subcutaneous tissue disorders/ Hyperhidrosis/ Sweats	02-MAR-2018/ 04:00/ (3)/ 02-MAR-2018/ 20:04/ (3)	Yes/ No	MILD/ NONE/ RECOVERED/ RESOLVED	Probable/ Unrelated/ Unrelated/ Unrelated/ Unrelated/ Unrelated	9.7 days post/ 1.5 days post/ -6.2 days pre	16.07	No
		9	Musculoskeletal and connective tissue disorders/ Myalgia/ Intermittent myalgia	02-MAR-2018/ 04:00/ (3)/ 13-MAR-2018/ 09:00/ (14)	Yes/ No	MILD/ MEDICATION: Ibuprofen, deep heat metholatum regular relief/ RECOVERED/ RESOLVED	Probable/ Unrelated/ Unrelated/ Unrelated/ Unrelated/ Unrelated	9.7 days post/ 1.5 days post/ -6.2 days pre	269.00	No

Notes: Adverse events were coded using the Medical Dictionary for Regulatory Activities (MedDRA) Version 20.1.
A treatment-emergent adverse event is defined as an AE that occurred or worsened following first administration of study drug.
Rando = Randomisation; PP = Piperaquine phosphate; (d) = Day from IP dose.
AE No. = Adverse Event Number; TEAE = A treatment-emergent adverse event; SAE = Serious adverse event; UN = Unknown; Dis = Discontinuation

Listing 16.2.7.1
Adverse Events

Rando Number	Challenge Agent	AE No	System Organ Class/ Preferred Term/ Adverse Event Verbatim	Onset Date/ Onset Time/ (d) End Date/ End Time/ (d)	TEAE/ SAE	Severity/ Action Taken/ Outcome	Treatment Directly Related to AE (Inoculum/ Artesunate/ Piperaquine/ Malarone/ Primacin/ Mosquito)	Time of onset Relative to Inoculum/ Time of onset Relative to Artesunate/ Time of onset Relative to PP	Duration (hours)	Dis
R209	3D7	10	General disorders and administration site conditions/ Pyrexia/ fever	02-MAR-2018/ 07:21/ (3)/ 02-MAR-2018/ 14:56/ (3)	Yes/ No	MILD/ NONE/ RECOVERED/ RESOLVED	Probable/ Unrelated/ Unrelated/ Unrelated/ Unrelated/ Unrelated	9.9 days post/ 1.7 days post/ -6.1 days pre	7.58	No
		11	Investigations/ Alanine aminotransferase increased/ Alanine transaminase count increased	08-MAR-2018/ 08:42/ (9)/ 12-MAR-2018/ 08:12/ (13)	Yes/ No	MODERATE/ NONE/ RECOVERED/ RESOLVED	Probable/ Unrelated/ Unrelated/ Unrelated/ Unrelated/ Unrelated	15.9 days post/ 7.7 days post/ 0 days post	95.50	No
R210	3D7	1	Musculoskeletal and connective tissue disorders/ Myalgia/ Myalgia	23-FEB-2018/ 13:00/ (-5)/ 25-FEB-2018/ 06:00/ (-3)	No/ No	MILD/ MEDICATION: ibuprofen/ RECOVERED/ RESOLVED	Unrelated/ Unrelated/ Unrelated/ Unrelated/ Unrelated/ Unrelated	3.1 days post/ -4.9 days pre/ -12.8 days pre	41.00	No
		2	Musculoskeletal and connective tissue disorders/ Myalgia/ Intermittent myalgia	28-FEB-2018/ 23:00/ (1)/ 02-MAR-2018/ 18:00/ (3)	Yes/ No	MILD/ MEDICATION: Ibuprofen/ RECOVERED/ RESOLVED	Probable/ Unrelated/ Unrelated/ Unrelated/ Unrelated/ Unrelated	8.5 days post/ 0.5 days post/ -7.4 days pre	43.00	No

Notes: Adverse events were coded using the Medical Dictionary for Regulatory Activities (MedDRA) Version 20.1.
A treatment-emergent adverse event is defined as an AE that occurred or worsened following first administration of study drug.
Rando = Randomisation; PP = Piperaquine phosphate; (d) = Day from IP dose.
AE No. = Adverse Event Number; TEAE = A treatment-emergent adverse event; SAE = Serious adverse event; UN = Unknown; Dis = Discontinuation

Listing 16.2.7.1
Adverse Events

Rando Number	Challenge Agent	AE No	System Organ Class/ Preferred Term/ Adverse Event Verbatim	Onset Date/ Onset Time/ (d) End Date/ End Time/ (d)	TEAE/ SAE	Severity/ Action Taken/ Outcome	Treatment Directly Related to AE (Inoculum/ Artesunate/ Piperaquine/ Malarone/ Primacin/ Mosquito)	Time of onset Relative to Inoculum/ Time of onset Relative to Artesunate/ Time of onset Relative to PP	Duration (hours)	Dis
R210	3D7	3	Nervous system disorders/ Headache/ intermittent headache	01-MAR-2018/ 10:00/ (2)/ 16-MAR-2018/ 08:00/ (17)	Yes/ No	MILD/ MEDICATION: ibuprofen/ RECOVERED/ RESOLVED	Probable/ Unrelated/ Unrelated/ Unrelated/ Unrelated/ Unrelated	9 days post/ 0.9 days post/ -6.9 days pre	358.00	No
		4	Gastrointestinal disorders/ Nausea/ Nausea	01-MAR-2018/ 12:30/ (2)/ 01-MAR-2018/ 20:30/ (2)	Yes/ No	MILD/ NONE/ RECOVERED/ RESOLVED	Probable/ Unrelated/ Unrelated/ Unrelated/ Unrelated/ Unrelated	9.1 days post/ 1 days post/ -6.8 days pre	8.00	No
		5	General disorders and administration site conditions/ Pyrexia/ Fever	01-MAR-2018/ 20:05/ (2)/ 01-MAR-2018/ 20:07/ (2)	Yes/ No	MILD/ NONE/ RECOVERED/ RESOLVED	Probable/ Unrelated/ Unrelated/ Unrelated/ Unrelated/ Unrelated	9.4 days post/ 1.3 days post/ -6.5 days pre	0.03	No
		6	Gastrointestinal disorders/ Nausea/ nausea	04-MAR-2018/ 11:00/ (5)/ 04-MAR-2018/ 21:00/ (5)	Yes/ No	MILD/ MEDICATION: ibuprofen/ RECOVERED/ RESOLVED	Probable/ Unrelated/ Unrelated/ Unrelated/ Unrelated/ Unrelated	12 days post/ 4 days post/ -3.9 days pre	10.00	No

Notes: Adverse events were coded using the Medical Dictionary for Regulatory Activities (MedDRA) Version 20.1.
A treatment-emergent adverse event is defined as an AE that occurred or worsened following first administration of study drug.
Rando = Randomisation; PP = Piperaquine phosphate; (d) = Day from IP dose.
AE No. = Adverse Event Number; TEAE = A treatment-emergent adverse event; SAE = Serious adverse event; UN = Unknown; Dis = Discontinuation

Listing 16.2.7.1
Adverse Events

Rando Number	Challenge Agent	AE No	System Organ Class/ Preferred Term/ Adverse Event Verbatim	Onset Date/ Onset Time/ (d) End Date/ End Time/ (d)	TEAE/ SAE	Severity/ Action Taken/ Outcome	Treatment Directly Related to AE (Inoculum/ Artesunate/ Piperaquine/ Malarone/ Primacin/ Mosquito)	Time of onset Relative to Inoculum/ Time of onset Relative to Artesunate/ Time of onset Relative to PP	Duration (hours)	Dis
R210	3D7	7	Respiratory, thoracic and mediastinal disorders/ Oropharyngeal pain/ sore throat	05-MAR-2018/ 11:00/ (6)/ 07-MAR-2018/ 06:30/ (8)	Yes/ No	MILD/ MEDICATION: ibuprofen/ RECOVERED/ RESOLVED	Probable/ Unrelated/ Unrelated/ Unrelated/ Unrelated/ Unrelated	13 days post/ 5 days post/ -2.9 days pre	43.50	No
		8	Skin and subcutaneous tissue disorders/ Hyperhidrosis/ intermittent sweats	08-MAR-2018/ 12:00/ (9)/ 10-MAR-2018/ 06:30/ (11)	Yes/ No	MILD/ MEDICATION: ibuprofen/ RECOVERED/ RESOLVED	Probable/ Unrelated/ Unrelated/ Unrelated/ Unrelated/ Unrelated	16.1 days post/ 8 days post/ 0.2 days post	42.50	No
		9	Musculoskeletal and connective tissue disorders/ Myalgia/ myalgia	08-MAR-2018/ 12:00/ (9)/ 10-MAR-2018/ 06:30/ (11)	Yes/ No	MILD/ MEDICATION: ibuprofen/ RECOVERED/ RESOLVED	Probable/ Unrelated/ Unrelated/ Unrelated/ Unrelated/ Unrelated	16.1 days post/ 8 days post/ 0.2 days post	42.50	No
		10	General disorders and administration site conditions/ Fatigue/ fatigue	09-MAR-2018/ 14:00/ (10)/ 12-MAR-2018/ 07:15/ (13)	Yes/ No	MILD/ NONE/ RECOVERED/ RESOLVED	Probable/ Unrelated/ Unrelated/ Unrelated/ Unrelated/ Unrelated	17.1 days post/ 9.1 days post/ 1.3 days post	65.25	No

Notes: Adverse events were coded using the Medical Dictionary for Regulatory Activities (MedDRA) Version 20.1.
A treatment-emergent adverse event is defined as an AE that occurred or worsened following first administration of study drug.
Rando = Randomisation; PP = Piperaquine phosphate; (d) = Day from IP dose.
AE No. = Adverse Event Number; TEAE = A treatment-emergent adverse event; SAE = Serious adverse event; UN = Unknown; Dis = Discontinuation

Listing 16.2.7.1
Adverse Events

Rando Number	Challenge Agent	AE No	System Organ Class/ Preferred Term/ Adverse Event Verbatim	Onset Date/ Onset Time/ (d) End Date/ End Time/ (d)	TEAE/ SAE	Severity/ Action Taken/ Outcome	Treatment Directly Related to AE (Inoculum/ Artesunate/ Piperaquine/ Malarone/ Primacin/ Mosquito)	Time of onset Relative to Inoculum/ Time of onset Relative to Artesunate/ Time of onset Relative to PP	Duration (hours)	Dis
R302	K13	1	Nervous system disorders/ Headache/ Headache intermittent	14-SEP-2018/ 07:15/ (2)/ 16-SEP-2018/ 08:00/ (4)	Yes/ No	MILD/ MEDICATION: Ibuprofen/ RECOVERED/ RESOLVED	Probable/ Unrelated/ Unrelated/ Unrelated/ Unrelated/ Unrelated	9.9 days post/ 0.9 days post/ -1.1 days pre	48.75	No
		2	Musculoskeletal and connective tissue disorders/ Myalgia/ Intermittent myalgia	14-SEP-2018/ 11:00/ (2)/ 15-SEP-2018/ 12:30/ (3)	Yes/ No	MILD/ MEDICATION: Ibuprofen/ RECOVERED/ RESOLVED	Probable/ Unlikely/ Unrelated/ Unrelated/ Unrelated/ Unrelated	10 days post/ 1.1 days post/ -0.9 days pre	25.50	No
		3	General disorders and administration site conditions/ Pyrexia/ Fever	14-SEP-2018/ 20:55/ (2)/ 14-SEP-2018/ 21:00/ (2)	Yes/ No	MILD/ NONE/ RECOVERED/ RESOLVED	Probable/ Unrelated/ Unrelated/ Unrelated/ Unrelated/ Unrelated	10.4 days post/ 1.5 days post/ -0.5 days pre	0.08	No
		4	Cardiac disorders/ Tachycardia/ Tachycardia	14-SEP-2018/ 20:55/ (2)/ 15-SEP-2018/ 08:19/ (3)	Yes/ No	MILD/ NONE/ RECOVERED/ RESOLVED	Unlikely/ Unrelated/ Unrelated/ Unrelated/ Unrelated/ Unrelated	10.4 days post/ 1.5 days post/ -0.5 days pre	11.40	No

Notes: Adverse events were coded using the Medical Dictionary for Regulatory Activities (MedDRA) Version 20.1.
A treatment-emergent adverse event is defined as an AE that occurred or worsened following first administration of study drug.
Rando = Randomisation; PP = Piperaquine phosphate; (d) = Day from IP dose.
AE No. = Adverse Event Number; TEAE = A treatment-emergent adverse event; SAE = Serious adverse event; UN = Unknown; Dis = Discontinuation

Listing 16.2.7.1
Adverse Events

Rando Number	Challenge Agent	AE No	System Organ Class/ Preferred Term/ Adverse Event Verbatim	Onset Date/ Onset Time/ (d) End Date/ End Time/ (d)	TEAE/ SAE	Severity/ Action Taken/ Outcome	Treatment Directly Related to AE (Inoculum/ Artesunate/ Piperaquine/ Malarone/ Primacin/ Mosquito)	Time of onset Relative to Inoculum/ Time of onset Relative to Artesunate/ Time of onset Relative to PP	Duration (hours)	Dis
R302	K13	5	General disorders and administration site conditions/ Fatigue/ Fatigue	14-SEP-2018/ 20:55/ (2)/ 15-SEP-2018/ 08:21/ (3)	Yes/ No	MILD/ NONE/ RECOVERED/ RESOLVED	Unlikely/ Unrelated/ Unrelated/ Unrelated/ Unrelated/ Unrelated	10.4 days post/ 1.5 days post/ -0.5 days pre	11.43	No
		6	General disorders and administration site conditions/ Pyrexia/ Intermittent Fever	15-SEP-2018/ 12:43/ (3)/ 15-SEP-2018/ 19:41/ (3)	Yes/ No	MILD/ NONE/ RECOVERED/ RESOLVED	Probable/ Unrelated/ Unrelated/ Unrelated/ Unrelated/ Unrelated	11.1 days post/ 2.2 days post/ 0.1 days post	6.97	No
		7	Investigations/ Lymphocyte count decreased/ Lymphocyte count decreased	16-SEP-2018/ 09:05/ (4)/ 18-SEP-2018/ 08:04/ (6)	Yes/ No	MODERATE/ NONE/ RECOVERED/ RESOLVED	Unlikely/ Unrelated/ Unrelated/ Unrelated/ Unrelated/ Unrelated	11.9 days post/ 3 days post/ 1 days post	46.98	No
		8	Nervous system disorders/ Headache/ Intermittent headache	16-SEP-2018/ 18:00/ (4)/ 19-SEP-2018/ 06:00/ (7)	Yes/ No	MILD/ NONE/ RECOVERED/ RESOLVED	Probable/ Unrelated/ Unrelated/ Unrelated/ Unrelated/ Unrelated	12.3 days post/ 3.4 days post/ 1.4 days post	60.00	No

Notes: Adverse events were coded using the Medical Dictionary for Regulatory Activities (MedDRA) Version 20.1.
A treatment-emergent adverse event is defined as an AE that occurred or worsened following first administration of study drug.
Rando = Randomisation; PP = Piperaquine phosphate; (d) = Day from IP dose.
AE No. = Adverse Event Number; TEAE = A treatment-emergent adverse event; SAE = Serious adverse event; UN = Unknown; Dis = Discontinuation

Listing 16.2.7.1
Adverse Events

Rando Number	Challenge Agent	AE No	System Organ Class/ Preferred Term/ Adverse Event Verbatim	Onset Date/ Onset Time/ (d) End Date/ End Time/ (d)	TEAE/ SAE	Severity/ Action Taken/ Outcome	Treatment Directly Related to AE (Inoculum/ Artesunate/ Piperaquine/ Malarone/ Primacin/ Mosquito)	Time of onset Relative to Inoculum/ Time of onset Relative to Artesunate/ Time of onset Relative to PP	Duration (hours)	Dis
R302	K13	9	Injury, poisoning and procedural complications/ Arthropod bite/ Mosquito bites	25-SEP-2018/ 10:00/ (13)/ 11-OCT-2018/ 07:15/ (29)	Yes/ No	MILD/ MEDICATION: Novasone cream/ RECOVERED/ RESOLVED	Unrelated/ Unrelated/ Unrelated/ Unrelated/ Unrelated/ Probable	21 days post/ 12 days post/ 10 days post	381.25	No
R303	K13	1	Nervous system disorders/ Headache/ headache	09-SEP-2018/ 11:00/ (-4)/ 09-SEP-2018/ 11:10/ (-4)	No/ No	MILD/ NONE/ RECOVERED/ RESOLVED	Probable/ Unrelated/ Unrelated/ Unrelated/ Unrelated/ Unrelated	5 days post/ -4 days pre/ -6 days pre	0.17	No
		2	Infections and infestations/ Upper respiratory tract infection/ Upper respiratory tract infection	09-SEP-2018/ 11:00/ (-4)/ 11-SEP-2018/ 09:00/ (-2)	No/ No	MILD/ NONE/ RECOVERED/ RESOLVED	Probable/ Unrelated/ Unrelated/ Unrelated/ Unrelated/ Unrelated	5 days post/ -4 days pre/ -6 days pre	46.00	No
		3	General disorders and administration site conditions/ Malaise/ Intermittent Malaise	09-SEP-2018/ 19:01/ (-4)/ 12-SEP-2018/ 08:10/ (-1)	No/ No	MILD/ NONE/ RECOVERED/ RESOLVED	Probable/ Unrelated/ Unrelated/ Unrelated/ Unrelated/ Unrelated	5.4 days post/ -3.7 days pre/ -5.7 days pre	61.15	No

Notes: Adverse events were coded using the Medical Dictionary for Regulatory Activities (MedDRA) Version 20.1.
A treatment-emergent adverse event is defined as an AE that occurred or worsened following first administration of study drug.
Rando = Randomisation; PP = Piperaquine phosphate; (d) = Day from IP dose.
AE No. = Adverse Event Number; TEAE = A treatment-emergent adverse event; SAE = Serious adverse event; UN = Unknown; Dis = Discontinuation

Listing 16.2.7.1
Adverse Events

Rando Number	Challenge Agent	AE No	System Organ Class/ Preferred Term/ Adverse Event Verbatim	Onset Date/ Onset Time/ (d) End Date/ End Time/ (d)	TEAE/ SAE	Severity/ Action Taken/ Outcome	Treatment Directly Related to AE (Inoculum/ Artesunate/ Piperaquine/ Malarone/ Primacin/ Mosquito)	Time of onset Relative to Inoculum/ Time of onset Relative to Artesunate/ Time of onset Relative to PP	Duration (hours)	Dis
R303	K13	4	Skin and subcutaneous tissue disorders/ Dermatitis contact/ Rash in cubital fossa both arms	10-SEP-2018/ 08:00/ (-3)/ 22-SEP-2018/ 08:23/ (10)	No/ No	MILD/ NONE/ RECOVERED/ RESOLVED	Unrelated/ Unrelated/ Unrelated/ Unrelated/ Unrelated/ Unrelated	5.9 days post/ -3.1 days pre/ -5.1 days pre	288.38	No
		5	Gastrointestinal disorders/ Nausea/ Nausea	11-SEP-2018/ 14:00/ (-2)/ 11-SEP-2018/ 19:00/ (-2)	No/ No	MILD/ NONE/ RECOVERED/ RESOLVED	Probable/ Unrelated/ Unrelated/ Unrelated/ Unrelated/ Unrelated	7.1 days post/ -1.9 days pre/ -3.9 days pre	5.00	No
		6	Injury, poisoning and procedural complications/ Thermal burn/ Right hand, first two fingers scalded	12-SEP-2018/ 11:40/ (-1)/ 12-SEP-2018/ 20:00/ (-1)	No/ No	MILD/ NONE/ RECOVERED/ RESOLVED	Unrelated/ Unrelated/ Unrelated/ Unrelated/ Unrelated/ Unrelated	8 days post/ -1 days pre/ -3 days pre	8.33	No
		7	Cardiac disorders/ Ventricular extrasystoles/ Frequent ventricular ectopy	13-SEP-2018/ 08:52/ Ongoing	No/ No	MODERATE/ NONE/ RECOVERING/ RESOLVING	Possible/ Unrelated/ Unrelated/ Unrelated/ Unrelated/ Unrelated	8.9 days post/ -0.1 days pre/ -2.1 days pre		No

Notes: Adverse events were coded using the Medical Dictionary for Regulatory Activities (MedDRA) Version 20.1.
A treatment-emergent adverse event is defined as an AE that occurred or worsened following first administration of study drug.
Rando = Randomisation; PP = Piperaquine phosphate; (d) = Day from IP dose.
AE No. = Adverse Event Number; TEAE = A treatment-emergent adverse event; SAE = Serious adverse event; UN = Unknown; Dis = Discontinuation

Listing 16.2.7.1
Adverse Events

Rando Number	Challenge Agent	AE No	System Organ Class/ Preferred Term/ Adverse Event Verbatim	Onset Date/ Onset Time/ (d) End Date/ End Time/ (d)	TEAE/ SAE	Severity/ Action Taken/ Outcome	Treatment Directly Related to AE (Inoculum/ Artesunate/ Piperaquine/ Malarone/ Primacin/ Mosquito)	Time of onset Relative to Inoculum/ Time of onset Relative to Artesunate/ Time of onset Relative to PP	Duration (hours)	Dis
R303	K13	8	Gastrointestinal disorders/ Nausea/ Intermittent Nausea	13-SEP-2018/ 17:00/ (1)/ 16-SEP-2018/ 09:00/ (4)	Yes/ No	MILD/ MEDICATION: Ondansteron/ RECOVERED/ RESOLVED	Probable/ Unrelated/ Unrelated/ Unrelated/ Unrelated/ Unrelated	9.3 days post/ 0.2 days post/ -1.8 days pre	64.00	No
		9	General disorders and administration site conditions/ Pyrexia/ Intermittent fever	13-SEP-2018/ 17:04/ (1)/ 14-SEP-2018/ 17:28/ (2)	Yes/ No	MILD/ MEDICATION: Ibuprofen/ NOT RECOVERED/ NOT RESOLVED	Probable/ Unrelated/ Unrelated/ Unrelated/ Unrelated/ Unrelated	9.3 days post/ 0.2 days post/ -1.8 days pre	24.40	No
		10	Nervous system disorders/ Headache/ Intermittent headache	14-SEP-2018/ 01:30/ (2)/ 15-SEP-2018/ 07:00/ (3)	Yes/ No	MILD/ MEDICATION: Ibuprofen/ RECOVERED/ RESOLVED	Probable/ Unrelated/ Unrelated/ Unrelated/ Unrelated/ Unrelated	9.6 days post/ 0.6 days post/ -1.4 days pre	29.50	No
		11	General disorders and administration site conditions/ Chills/ Intermittent rigors	14-SEP-2018/ 09:55/ (2)/ 16-SEP-2018/ 20:18/ (4)	Yes/ No	MILD/ MEDICATION: Ibuprofen/ RECOVERED/ RESOLVED	Probable/ Unrelated/ Unrelated/ Unrelated/ Unrelated/ Unrelated	10 days post/ 0.9 days post/ -1.1 days pre	58.38	No

Notes: Adverse events were coded using the Medical Dictionary for Regulatory Activities (MedDRA) Version 20.1.
A treatment-emergent adverse event is defined as an AE that occurred or worsened following first administration of study drug.
Rando = Randomisation; PP = Piperaquine phosphate; (d) = Day from IP dose.
AE No. = Adverse Event Number; TEAE = A treatment-emergent adverse event; SAE = Serious adverse event; UN = Unknown; Dis = Discontinuation

Listing 16.2.7.1
Adverse Events

Rando Number	Challenge Agent	AE No	System Organ Class/ Preferred Term/ Adverse Event Verbatim	Onset Date/ Onset Time/ (d) End Date/ End Time/ (d)	TEAE/ SAE	Severity/ Action Taken/ Outcome	Treatment Directly Related to AE (Inoculum/ Artesunate/ Piperaquine/ Malarone/ Primacin/ Mosquito)	Time of onset Relative to Inoculum/ Time of onset Relative to Artesunate/ Time of onset Relative to PP	Duration (hours)	Dis
R303	K13	12	General disorders and administration site conditions/ Pyrexia/ Fever	14-SEP-2018/ 17:28/ (2)/ 14-SEP-2018/ 21:18/ (2)	Yes/ No	MODERATE/ MEDICATION: Ibuprofen/ RECOVERED/ RESOLVED	Probable/ Unrelated/ Unrelated/ Unrelated/ Unrelated/ Unrelated	10.3 days post/ 1.3 days post/ -0.7 days pre	3.83	No
		13	Skin and subcutaneous tissue disorders/ Hyperhidrosis/ Sweating	14-SEP-2018/ 21:16/ (2)/ 15-SEP-2018/ 07:25/ (3)	Yes/ No	MILD/ NONE/ RECOVERED/ RESOLVED	Probable/ Unrelated/ Unrelated/ Unrelated/ Unrelated/ Unrelated	10.4 days post/ 1.4 days post/ -0.6 days pre	10.15	No
		14	Investigations/ White blood cell count decreased/ decreased white cell count	15-SEP-2018/ 11:18/ (3)/ 18-SEP-2018/ 07:29/ (6)	Yes/ No	MODERATE/ NONE/ RECOVERED/ RESOLVED	Probable/ Unrelated/ Unrelated/ Unrelated/ Unrelated/ Unrelated	11 days post/ 2 days post/ 0 days post	68.18	No
		15	Investigations/ Neutrophil count decreased/ Neutrophil count decreased	15-SEP-2018/ 11:18/ (3)/ 22-SEP-2018/ 08:40/ (10)	Yes/ No	MODERATE/ NONE/ RECOVERED/ RESOLVED	Probable/ Unrelated/ Unrelated/ Unrelated/ Unrelated/ Unrelated	11 days post/ 2 days post/ 0 days post	165.37	No

Notes: Adverse events were coded using the Medical Dictionary for Regulatory Activities (MedDRA) Version 20.1.
A treatment-emergent adverse event is defined as an AE that occurred or worsened following first administration of study drug.
Rando = Randomisation; PP = Piperaquine phosphate; (d) = Day from IP dose.
AE No. = Adverse Event Number; TEAE = A treatment-emergent adverse event; SAE = Serious adverse event; UN = Unknown; Dis = Discontinuation

Listing 16.2.7.1
Adverse Events

Rando Number	Challenge Agent	AE No	System Organ Class/ Preferred Term/ Adverse Event Verbatim	Onset Date/ Onset Time/ (d) End Date/ End Time/ (d)	TEAE/ SAE	Severity/ Action Taken/ Outcome	Treatment Directly Related to AE (Inoculum/ Artesunate/ Piperaquine/ Malarone/ Primacin/ Mosquito)	Time of onset Relative to Inoculum/ Time of onset Relative to Artesunate/ Time of onset Relative to PP	Duration (hours)	Dis
R303	K13	16	Vascular disorders/ Thrombophlebitis superficial/ Left thigh superficial vein thrombophelitis	16-SEP-2018/ 19:00/ (4)/ 21-SEP-2018/ 09:00/ (9)	Yes/ No	MODERATE/ RECOVERED/ RESOLVED	Unrelated/ Unrelated/ Unrelated/ Unrelated/ Unrelated/ Unrelated	12.4 days post/ 3.3 days post/ 1.3 days post	110.00	No
		17	Nervous system disorders/ Headache/ Headache	17-SEP-2018/ 20:00/ (5)/ 18-SEP-2018/ 05:30/ (6)	Yes/ No	MILD/ MEDICATION: Ibuprofen/ RECOVERED/ RESOLVED	Probable/ Unrelated/ Unrelated/ Unrelated/ Unrelated/ Unrelated	13.4 days post/ 4.4 days post/ 2.4 days post	9.50	No
		18	Gastrointestinal disorders/ Nausea/ Intermittent Nausea	18-SEP-2018/ 06:10/ (6)/ 18-SEP-2018/ 13:00/ (6)	Yes/ No	MILD/ NONE/ RECOVERED/ RESOLVED	Probable/ Unrelated/ Unrelated/ Unrelated/ Unrelated/ Unrelated	13.8 days post/ 4.8 days post/ 2.8 days post	6.83	No
		19	Injury, poisoning and procedural complications/ Arthropod bite/ Mosquito bites reaction itching	25-SEP-2018/ 10:00/ (13)/ 02-OCT-2018/ 09:00/ (20)	Yes/ No	MILD/ MEDICATION: Novasone cream/ RECOVERED/ RESOLVED	Unrelated/ Unrelated/ Unrelated/ Unrelated/ Unrelated/ Probable	21 days post/ 11.9 days post/ 9.9 days post	167.00	No

Notes: Adverse events were coded using the Medical Dictionary for Regulatory Activities (MedDRA) Version 20.1.
A treatment-emergent adverse event is defined as an AE that occurred or worsened following first administration of study drug.
Rando = Randomisation; PP = Piperaquine phosphate; (d) = Day from IP dose.
AE No. = Adverse Event Number; TEAE = A treatment-emergent adverse event; SAE = Serious adverse event; UN = Unknown; Dis = Discontinuation

Listing 16.2.7.1
Adverse Events

Rando Number	Challenge Agent	AE No	System Organ Class/ Preferred Term/ Adverse Event Verbatim	Onset Date/ Onset Time/ (d) End Date/ End Time/ (d)	TEAE/ SAE	Severity/ Action Taken/ Outcome	Treatment Directly Related to AE (Inoculum/ Artesunate/ Piperaquine/ Malarone/ Primacin/ Mosquito)	Time of onset Relative to Inoculum/ Time of onset Relative to Artesunate/ Time of onset Relative to PP	Duration (hours)	Dis
R304	K13	1	Musculoskeletal and connective tissue disorders/ Myalgia/ Intermittent myalgia	10-SEP-2018/ 14:30/ (-3)/ 13-SEP-2018/ 12:00/ (1)	No/ No	MILD/ NONE/ RECOVERED/ RESOLVED	Probable/ Unrelated/ Unrelated/ Unrelated/ Unrelated/ Unrelated	6.2 days post/ -2.8 days pre/ -4.8 days pre	69.50	No
		2	General disorders and administration site conditions/ Malaise/ Intermittent Malaise	10-SEP-2018/ 14:30/ (-3)/ 15-SEP-2018/ 07:28/ (3)	No/ No	MILD/ NONE/ RECOVERED/ RESOLVED	Probable/ Unrelated/ Unrelated/ Unrelated/ Unrelated/ Unrelated	6.2 days post/ -2.8 days pre/ -4.8 days pre	112.97	No
		3	Nervous system disorders/ Headache/ intermittent headache	13-SEP-2018/ 15:00/ (1)/ 16-SEP-2018/ 07:00/ (4)	Yes/ No	MILD/ MEDICATION: Ibuprofen/ RECOVERED/ RESOLVED	Probable/ Unrelated/ Unrelated/ Unrelated/ Unrelated/ Unrelated	9.2 days post/ 0.2 days post/ -1.8 days pre	64.00	No
		4	Respiratory, thoracic and mediastinal disorders/ Oropharyngeal pain/ Sore throat	14-SEP-2018/ 15:30/ (2)/ 16-SEP-2018/ 07:00/ (4)	Yes/ No	MILD/ NONE/ RECOVERED/ RESOLVED	Probable/ Unrelated/ Unrelated/ Unrelated/ Unrelated/ Unrelated	10.2 days post/ 1.3 days post/ -0.8 days pre	39.50	No

Notes: Adverse events were coded using the Medical Dictionary for Regulatory Activities (MedDRA) Version 20.1.
A treatment-emergent adverse event is defined as an AE that occurred or worsened following first administration of study drug.
Rando = Randomisation; PP = Piperaquine phosphate; (d) = Day from IP dose.
AE No. = Adverse Event Number; TEAE = A treatment-emergent adverse event; SAE = Serious adverse event; UN = Unknown; Dis = Discontinuation

Listing 16.2.7.1
Adverse Events

Rando Number	Challenge Agent	AE No	System Organ Class/ Preferred Term/ Adverse Event Verbatim	Onset Date/ Onset Time/ (d) End Date/ End Time/ (d)	TEAE/ SAE	Severity/ Action Taken/ Outcome	Treatment Directly Related to AE (Inoculum/ Artesunate/ Piperaquine/ Malarone/ Primacin/ Mosquito)	Time of onset Relative to Inoculum/ Time of onset Relative to Artesunate/ Time of onset Relative to PP	Duration (hours)	Dis
R304	K13	5	General disorders and administration site conditions/ Pyrexia/ Fever	14-SEP-2018/ 16:35/ (2)/ 14-SEP-2018/ 21:44/ (2)	Yes/ No	MILD/ NONE/ RECOVERED/ RESOLVED	Probable/ Unrelated/ Unrelated/ Unrelated/ Unrelated/ Unrelated	10.2 days post/ 1.3 days post/ -0.7 days pre	5.15	No
		6	Infections and infestations/ Upper respiratory tract infection/ Upper respiratory tract infection	15-SEP-2018/ 07:00/ (3)/ 28-SEP-2018/ 08:30/ (16)	Yes/ No	MILD/ NONE/ RECOVERED/ RESOLVED	Unrelated/ Unrelated/ Unrelated/ Unrelated/ Unrelated/ Unrelated	10.9 days post/ 1.9 days post/ -0.1 days pre	313.50	No
		7	Metabolism and nutrition disorders/ Hypophosphataemia/ Hypophosphataemia	15-SEP-2018/ 09:22/ (3)/ 16-SEP-2018/ 09:22/ (4)	Yes/ No	MODERATE/ NONE/ RECOVERED/ RESOLVED	Probable/ Unrelated/ Unrelated/ Unrelated/ Unrelated/ Unrelated	10.9 days post/ 2 days post/ 0 days post	24.00	No
		8	Nervous system disorders/ Headache/ Intermittent headache	16-SEP-2018/ 09:02/ (4)/ 19-SEP-2018/ 19:00/ (7)	Yes/ No	MILD/ MEDICATION: Ibuprofen/ RECOVERED/ RESOLVED	Probable/ Unrelated/ Unrelated/ Unrelated/ Unrelated/ Unrelated	11.9 days post/ 3 days post/ 1 days post	81.97	No

Notes: Adverse events were coded using the Medical Dictionary for Regulatory Activities (MedDRA) Version 20.1.
A treatment-emergent adverse event is defined as an AE that occurred or worsened following first administration of study drug.
Rando = Randomisation; PP = Piperaquine phosphate; (d) = Day from IP dose.
AE No. = Adverse Event Number; TEAE = A treatment-emergent adverse event; SAE = Serious adverse event; UN = Unknown; Dis = Discontinuation

Listing 16.2.7.1
Adverse Events

Rando Number	Challenge Agent	AE No	System Organ Class/ Preferred Term/ Adverse Event Verbatim	Onset Date/ Onset Time/ (d) End Date/ End Time/ (d)	TEAE/ SAE	Severity/ Action Taken/ Outcome	Treatment Directly Related to AE (Inoculum/ Artesunate/ Piperaquine/ Malarone/ Primacin/ Mosquito)	Time of onset Relative to Inoculum/ Time of onset Relative to Artesunate/ Time of onset Relative to PP	Duration (hours)	Dis
R304	K13	9	Musculoskeletal and connective tissue disorders/ Myalgia/ Myalgia	16-SEP-2018/ 22:00/ (4)/ 17-SEP-2018/ 08:35/ (5)	Yes/ No	MILD/ MEDICATION: Ibuprofen/ RECOVERED/ RESOLVED	Probable/ Unrelated/ Unrelated/ Unrelated/ Unrelated/ Unrelated	12.5 days post/ 3.5 days post/ 1.5 days post	10.58	No
		10	Musculoskeletal and connective tissue disorders/ Myalgia/ Myalgia	23-SEP-2018/ UN:UN/ (11)/ 28-SEP-2018/ 15:00/ (16)	Yes/ No	MODERATE/ RECOVERED/ RESOLVED	Probable/ Unrelated/ Unrelated/ Unrelated/ Unrelated/ Unrelated	18.6 days post/ 9.6 days post/ 7.6 days post		No
R305	3D7	1	Nervous system disorders/ Headache/ Headache	10-SEP-2018/ 13:52/ (-3)/ 10-SEP-2018/ 17:00/ (-3)	No/ No	MILD/ MEDICATION: Ibuprofen/ RECOVERED/ RESOLVED	Probable/ Unrelated/ Unrelated/ Unrelated/ Unrelated/ Unrelated	5.1 days post	3.13	No
		2	Nervous system disorders/ Headache/ Intermittent headache	13-SEP-2018/ 12:00/ (1)/ 15-SEP-2018/ 14:00/ (3)	Yes/ No	MILD/ MEDICATION: Ibuprofen/ RECOVERED/ RESOLVED	Probable/ Unrelated/ Unrelated/ Unrelated/ Unrelated/ Unrelated	8 days post	50.00	No

Notes: Adverse events were coded using the Medical Dictionary for Regulatory Activities (MedDRA) Version 20.1.
A treatment-emergent adverse event is defined as an AE that occurred or worsened following first administration of study drug.
Rando = Randomisation; PP = Piperaquine phosphate; (d) = Day from IP dose.
AE No. = Adverse Event Number; TEAE = A treatment-emergent adverse event; SAE = Serious adverse event; UN = Unknown; Dis = Discontinuation

Listing 16.2.7.1
Adverse Events

Rando Number	Challenge Agent	AE No	System Organ Class/ Preferred Term/ Adverse Event Verbatim	Onset Date/ Onset Time/ (d) End Date/ End Time/ (d)	TEAE/ SAE	Severity/ Action Taken/ Outcome	Treatment Directly Related to AE (Inoculum/ Artesunate/ Piperaquine/ Malarone/ Primacin/ Mosquito)	Time of onset Relative to Inoculum/ Time of onset Relative to Artesunate/ Time of onset Relative to PP	Duration (hours)	Dis
R305	3D7	3	Musculoskeletal and connective tissue disorders/ Myalgia/ Intermittent myalgia	14-SEP-2018/ 08:18/ (2)/ 14-SEP-2018/ 16:00/ (2)	Yes/ No	MODERATE/ NONE/ RECOVERING/ RESOLVING	Probable/ Unrelated/ Unrelated/ Unrelated/ Unrelated/ Unrelated	8.9 days post	7.70	No
		4	Musculoskeletal and connective tissue disorders/ Myalgia/ Myalgia	14-SEP-2018/ 16:00/ (2)/ 15-SEP-2018/ 14:00/ (3)	Yes/ No	MILD/ MEDICATION: Ibuprofen/ RECOVERED/ RESOLVED	Probable/ Unrelated/ Unrelated/ Unrelated/ Unrelated/ Unrelated	9.2 days post	22.00	No
		5	Investigations/ Neutrophil count decreased/ Neutrophil count decreased	16-SEP-2018/ 09:36/ (4)/ 18-SEP-2018/ 07:16/ (6)	Yes/ No	SEVERE/ NONE/ RECOVERING/ RESOLVING	Probable/ Unrelated/ Unrelated/ Unrelated/ Unrelated/ Unrelated	10.9 days post	45.67	No
		6	Investigations/ White blood cell count decreased/ White cell count decreased	16-SEP-2018/ 09:36/ (4)/ 18-SEP-2018/ 07:16/ (6)	Yes/ No	MODERATE/ NONE/ RECOVERED/ RESOLVED	Probable/ Unrelated/ Unrelated/ Unrelated/ Unrelated/ Unrelated	10.9 days post	45.67	No

Notes: Adverse events were coded using the Medical Dictionary for Regulatory Activities (MedDRA) Version 20.1.
A treatment-emergent adverse event is defined as an AE that occurred or worsened following first administration of study drug.
Rando = Randomisation; PP = Piperaquine phosphate; (d) = Day from IP dose.
AE No. = Adverse Event Number; TEAE = A treatment-emergent adverse event; SAE = Serious adverse event; UN = Unknown; Dis = Discontinuation

Listing 16.2.7.1
Adverse Events

Rando Number	Challenge Agent	AE No	System Organ Class/ Preferred Term/ Adverse Event Verbatim	Onset Date/ Onset Time/ (d) End Date/ End Time/ (d)	TEAE/ SAE	Severity/ Action Taken/ Outcome	Treatment Directly Related to AE (Inoculum/ Artesunate/ Piperaquine/ Malarone/ Primacin/ Mosquito)	Time of onset Relative to Inoculum/ Time of onset Relative to Artesunate/ Time of onset Relative to PP	Duration (hours)	Dis
R305	3D7	7	General disorders and administration site conditions/ Malaise/ Malaise	16-SEP-2018/ 11:00/ (4)/ 16-SEP-2018/ 14:00/ (4)	Yes/ No	MILD/ MEDICATION: Ibuprofen/ RECOVERED/ RESOLVED	Probable/ Unrelated/ Unrelated/ Unrelated/ Unrelated/ Unrelated	11 days post	3.00	No
		8	Nervous system disorders/ Headache/ Headache	17-SEP-2018/ 06:30/ (5)/ 17-SEP-2018/ 09:00/ (5)	Yes/ No	MILD/ MEDICATION: Ibuprofen/ RECOVERED/ RESOLVED	Probable/ Unrelated/ Unrelated/ Unrelated/ Unrelated/ Unrelated	11.8 days post	2.50	No
		9	Investigations/ Neutrophil count decreased/ Decreased neutrophil count	18-SEP-2018/ 07:16/ (6)/ 19-SEP-2018/ 19:23/ (7)	Yes/ No	MODERATE/ NONE/ RECOVERED/ RESOLVED	Probable/ Unrelated/ Unrelated/ Unrelated/ Unrelated/ Unrelated	12.8 days post	36.12	No
		10	Musculoskeletal and connective tissue disorders/ Arthralgia/ Arthralgia	19-SEP-2018/ 21:00/ (7)/ 20-SEP-2018/ 19:00/ (8)	Yes/ No	MILD/ MEDICATION: Paracetamol/ RECOVERED/ RESOLVED	Probable/ Unrelated/ Unrelated/ Unrelated/ Unrelated/ Unrelated	14.4 days post	22.00	No

Notes: Adverse events were coded using the Medical Dictionary for Regulatory Activities (MedDRA) Version 20.1.
A treatment-emergent adverse event is defined as an AE that occurred or worsened following first administration of study drug.
Rando = Randomisation; PP = Piperaquine phosphate; (d) = Day from IP dose.
AE No. = Adverse Event Number; TEAE = A treatment-emergent adverse event; SAE = Serious adverse event; UN = Unknown; Dis = Discontinuation

Listing 16.2.7.1
Adverse Events

Rando Number	Challenge Agent	AE No	System Organ Class/ Preferred Term/ Adverse Event Verbatim	Onset Date/ Onset Time/ (d) End Date/ End Time/ (d)	TEAE/ SAE	Severity/ Action Taken/ Outcome	Treatment Directly Related to AE (Inoculum/ Artesunate/ Piperaquine/ Malarone/ Primacin/ Mosquito)	Time of onset Relative to Inoculum/ Time of onset Relative to Artesunate/ Time of onset Relative to PP	Duration (hours)	Dis
R305	3D7	11	Nervous system disorders/ Lethargy/ Lethargy	19-SEP-2018/ 21:00/ (7)/ 20-SEP-2018/ 19:00/ (8)	Yes/ No	MILD/ NONE/ RECOVERED/ RESOLVED	Probable/ Unrelated/ Unrelated/ Unrelated/ Unrelated/ Unrelated	14.4 days post	22.00	No
		12	General disorders and administration site conditions/ Malaise/ Malaise	19-SEP-2018/ 21:00/ (7)/ 20-SEP-2018/ 19:00/ (8)	Yes/ No	MILD/ MEDICATION: Paracetamol/ RECOVERED/ RESOLVED	Probable/ Unrelated/ Unrelated/ Unrelated/ Unrelated/ Unrelated	14.4 days post	22.00	No
		13	Gastrointestinal disorders/ Nausea/ Nausea	19-SEP-2018/ 21:00/ (7)/ 20-SEP-2018/ 19:00/ (8)	Yes/ No	MILD/ MEDICATION: Ondansentron/ RECOVERED/ RESOLVED	Probable/ Unrelated/ Unrelated/ Unrelated/ Unrelated/ Unrelated	14.4 days post	22.00	No
		14	Investigations/ Neutrophil count decreased/ Neutrophil count decreased	22-SEP-2018/ 07:53/ (10)/ 25-SEP-2018/ 08:19/ (13)	Yes/ No	MODERATE/ NONE/ RECOVERED/ RESOLVED	Probable/ Unrelated/ Unrelated/ Unrelated/ Unrelated/ Unrelated	16.9 days post	72.43	No

Notes: Adverse events were coded using the Medical Dictionary for Regulatory Activities (MedDRA) Version 20.1.
A treatment-emergent adverse event is defined as an AE that occurred or worsened following first administration of study drug.
Rando = Randomisation; PP = Piperaquine phosphate; (d) = Day from IP dose.
AE No. = Adverse Event Number; TEAE = A treatment-emergent adverse event; SAE = Serious adverse event; UN = Unknown; Dis = Discontinuation

Listing 16.2.7.1
Adverse Events

Rando Number	Challenge Agent	AE No	System Organ Class/ Preferred Term/ Adverse Event Verbatim	Onset Date/ Onset Time/ (d) End Date/ End Time/ (d)	TEAE/ SAE	Severity/ Action Taken/ Outcome	Treatment Directly Related to AE (Inoculum/ Artesunate/ Piperaquine/ Malarone/ Primacin/ Mosquito)	Time of onset Relative to Inoculum/ Time of onset Relative to Artesunate/ Time of onset Relative to PP	Duration (hours)	Dis
R305	3D7	15	Nervous system disorders/ Headache/ Intermittent headache	23-SEP-2018/ UN:UN/ (11)/ 23-SEP-2018/ 18:00/ (11)	Yes/ No	MILD/ NONE/ RECOVERED/ RESOLVED	Probable/ Unrelated/ Unrelated/ Unrelated/ Unrelated/ Unrelated	17.5 days post		No
		16	Injury, poisoning and procedural complications/ Arthropod bite/ Mosquito bites	25-SEP-2018/ 09:30/ (13)/ 25-SEP-2018/ 21:00/ (13)	Yes/ No	MILD/ MEDICATION: Mometasone cream/ RECOVERED/ RESOLVED	Unrelated/ Unrelated/ Unrelated/ Unrelated/ Unrelated/ Probable	19.9 days post	11.50	No
		17	Investigations/ Neutrophil count decreased/ Neutrophil count decreased	28-SEP-2018/ 07:23/ (16)/ 02-OCT-2018/ 07:50/ (20)	Yes/ No	MODERATE/ NONE/ RECOVERED/ RESOLVED	Probable/ Unrelated/ Unrelated/ Unrelated/ Unrelated/ Unrelated	22.8 days post	96.45	No
		18	Injury, poisoning and procedural complications/ Arthropod bite/ Lump right forearm	01-OCT-2018/ 09:00/ (19)/ 17-OCT-2018/ 09:45/ (35)	Yes/ No	MILD/ NONE/ RECOVERED/ RESOLVED	Unrelated/ Unrelated/ Unrelated/ Unrelated/ Unrelated/ Unrelated	25.9 days post	384.75	No

Notes: Adverse events were coded using the Medical Dictionary for Regulatory Activities (MedDRA) Version 20.1.
A treatment-emergent adverse event is defined as an AE that occurred or worsened following first administration of study drug.
Rando = Randomisation; PP = Piperaquine phosphate; (d) = Day from IP dose.
AE No. = Adverse Event Number; TEAE = A treatment-emergent adverse event; SAE = Serious adverse event; UN = Unknown; Dis = Discontinuation

Listing 16.2.7.1
Adverse Events

Rando Number	Challenge Agent	AE No	System Organ Class/ Preferred Term/ Adverse Event Verbatim	Onset Date/ Onset Time/ (d) End Date/ End Time/ (d)	TEAE/ SAE	Severity/ Action Taken/ Outcome	Treatment Directly Related to AE (Inoculum/ Artesunate/ Piperaquine/ Malarone/ Primacin/ Mosquito)	Time of onset Relative to Inoculum/ Time of onset Relative to Artesunate/ Time of onset Relative to PP	Duration (hours)	Dis
R306	3D7	1	Musculoskeletal and connective tissue disorders/ Back pain/ Intermittent back pain	14-SEP-2018/ 05:00/ (2)/ 17-SEP-2018/ 09:00/ (5)	Yes/ No	MILD/ MEDICATION: Ibuprofen/ RECOVERED/ RESOLVED	Unrelated/ Unrelated/ Unrelated/ Unrelated/ Unrelated/ Unrelated	8.7 days post	76.00	No
		2	Nervous system disorders/ Headache/ Headache	14-SEP-2018/ 12:50/ (2)/ 14-SEP-2018/ 21:37/ (2)	Yes/ No	MILD/ MEDICATION: Ibuprofen/ RECOVERED/ RESOLVED	Probable/ Unrelated/ Unrelated/ Unrelated/ Unrelated/ Unrelated	9.1 days post	8.78	No
		3	Cardiac disorders/ Tachycardia/ Tachycardia	20-SEP-2018/ 06:55/ (8)/ 20-SEP-2018/ 06:58/ (8)	Yes/ No	MILD/ NONE/ RECOVERED/ RESOLVED	Probable/ Unrelated/ Unrelated/ Unrelated/ Unrelated/ Unrelated	14.8 days post	0.05	No
		4	Musculoskeletal and connective tissue disorders/ Myalgia/ Myalgia lower back	20-SEP-2018/ 13:00/ (8)/ 20-SEP-2018/ 20:30/ (8)	Yes/ No	MILD/ MEDICATION: Ibuprofen/ RECOVERED/ RESOLVED	Probable/ Unrelated/ Unrelated/ Unrelated/ Unrelated/ Unrelated	15.1 days post	7.50	No

Notes: Adverse events were coded using the Medical Dictionary for Regulatory Activities (MedDRA) Version 20.1.
A treatment-emergent adverse event is defined as an AE that occurred or worsened following first administration of study drug.
Rando = Randomisation; PP = Piperaquine phosphate; (d) = Day from IP dose.
AE No. = Adverse Event Number; TEAE = A treatment-emergent adverse event; SAE = Serious adverse event; UN = Unknown; Dis = Discontinuation

Listing 16.2.7.1
Adverse Events

Rando Number	Challenge Agent	AE No	System Organ Class/ Preferred Term/ Adverse Event Verbatim	Onset Date/ Onset Time/ (d) End Date/ End Time/ (d)	TEAE/ SAE	Severity/ Action Taken/ Outcome	Treatment Directly Related to AE (Inoculum/ Artesunate/ Piperaquine/ Malarone/ Primacin/ Mosquito)	Time of onset Relative to Inoculum/ Time of onset Relative to Artesunate/ Time of onset Relative to PP	Duration (hours)	Dis
R306	3D7	5	General disorders and administration site conditions/ Chills/ Rigors	20-SEP-2018/ 14:30/ (8)/ 20-SEP-2018/ 20:30/ (8)	Yes/ No	MILD/ NONE/ RECOVERED/ RESOLVED	Probable/ Unrelated/ Unrelated/ Unrelated/ Unrelated/ Unrelated	15.1 days post	6.00	No
		6	General disorders and administration site conditions/ Pyrexia/ Fever	20-SEP-2018/ 17:00/ (8)/ 20-SEP-2018/ 20:30/ (8)	Yes/ No	MODERATE/ MEDICATION: Ibuprofen/ RECOVERED/ RESOLVED	Probable/ Unrelated/ Unrelated/ Unrelated/ Unrelated/ Unrelated	15.2 days post	3.50	No
R307	3D7	1	Musculoskeletal and connective tissue disorders/ Myalgia/ Myalgia	13-SEP-2018/ 13:30/ (1)/ 15-SEP-2018/ 08:00/ (3)	Yes/ No	MILD/ MEDICATION: Ibuprofen/ RECOVERED/ RESOLVED	Probable/ Unrelated/ Unrelated/ Unrelated/ Unrelated/ Unrelated	8.1 days post	42.50	No
		2	Nervous system disorders/ Headache/ Intermittent Headache	13-SEP-2018/ 16:00/ (1)/ 15-SEP-2018/ 08:00/ (3)	Yes/ No	MILD/ MEDICATION: Ibuprofen/ RECOVERED/ RESOLVED	Probable/ Unrelated/ Unrelated/ Unrelated/ Unrelated/ Unrelated	8.2 days post	40.00	No

Notes: Adverse events were coded using the Medical Dictionary for Regulatory Activities (MedDRA) Version 20.1.
A treatment-emergent adverse event is defined as an AE that occurred or worsened following first administration of study drug.
Rando = Randomisation; PP = Piperaquine phosphate; (d) = Day from IP dose.
AE No. = Adverse Event Number; TEAE = A treatment-emergent adverse event; SAE = Serious adverse event; UN = Unknown; Dis = Discontinuation

Listing 16.2.7.1
Adverse Events

Rando Number	Challenge Agent	AE No	System Organ Class/ Preferred Term/ Adverse Event Verbatim	Onset Date/ Onset Time/ (d) End Date/ End Time/ (d)	TEAE/ SAE	Severity/ Action Taken/ Outcome	Treatment Directly Related to AE (Inoculum/ Artesunate/ Piperaquine/ Malarone/ Primacin/ Mosquito)	Time of onset Relative to Inoculum/ Time of onset Relative to Artesunate/ Time of onset Relative to PP	Duration (hours)	Dis
R307	3D7	3	General disorders and administration site conditions/ Pyrexia/ Fever	13-SEP-2018/ 19:57/ (1)/ 13-SEP-2018/ 21:51/ (1)	Yes/ No	MODERATE/ MEDICATION: Ibuprofen/ RECOVERING/ RESOLVING	Probable/ Unrelated/ Unrelated/ Unrelated/ Unrelated/ Unrelated	8.4 days post	1.90	No
		4	General disorders and administration site conditions/ Pyrexia/ Fever	13-SEP-2018/ 21:51/ (1)/ 14-SEP-2018/ 01:51/ (2)	Yes/ No	MILD/ MEDICATION: Ibuprofen/ RECOVERED/ RESOLVED	Probable/ Unrelated/ Unrelated/ Unrelated/ Unrelated/ Unrelated	8.4 days post	4.00	No
		5	Gastrointestinal disorders/ Nausea/ Nausea	14-SEP-2018/ 04:30/ (2)/ 16-SEP-2018/ 08:00/ (4)	Yes/ No	MILD/ NONE/ RECOVERED/ RESOLVED	Probable/ Unrelated/ Unrelated/ Unrelated/ Unrelated/ Unrelated	8.7 days post	51.50	No
		6	General disorders and administration site conditions/ Chills/ Shivering	14-SEP-2018/ 04:30/ (2)/ 16-SEP-2018/ 08:00/ (4)	Yes/ No	MILD/ NONE/ RECOVERED/ RESOLVED	Probable/ Unrelated/ Unrelated/ Unrelated/ Unrelated/ Unrelated	8.7 days post	51.50	No

Notes: Adverse events were coded using the Medical Dictionary for Regulatory Activities (MedDRA) Version 20.1.
A treatment-emergent adverse event is defined as an AE that occurred or worsened following first administration of study drug.
Rando = Randomisation; PP = Piperaquine phosphate; (d) = Day from IP dose.
AE No. = Adverse Event Number; TEAE = A treatment-emergent adverse event; SAE = Serious adverse event; UN = Unknown; Dis = Discontinuation

Listing 16.2.7.1
Adverse Events

Rando Number	Challenge Agent	AE No	System Organ Class/ Preferred Term/ Adverse Event Verbatim	Onset Date/ Onset Time/ (d) End Date/ End Time/ (d)	TEAE/ SAE	Severity/ Action Taken/ Outcome	Treatment Directly Related to AE (Inoculum/ Artesunate/ Piperaquine/ Malarone/ Primacin/ Mosquito)	Time of onset Relative to Inoculum/ Time of onset Relative to Artesunate/ Time of onset Relative to PP	Duration (hours)	Dis
R307	3D7	7	General disorders and administration site conditions/ Pyrexia/ Fever	14-SEP-2018/ 05:49/ (2)/ 14-SEP-2018/ 08:36/ (2)	Yes/ No	MODERATE/ MEDICATION: Ibuprofen/ RECOVERED/ RESOLVED	Probable/ Unrelated/ Unrelated/ Unrelated/ Unrelated/ Unrelated	8.8 days post	2.78	No
		8	General disorders and administration site conditions/ Pyrexia/ Intermittent fever	14-SEP-2018/ 13:14/ (2)/ 16-SEP-2018/ 09:21/ (4)	Yes/ No	MILD/ MEDICATION: Paracetamol/ RECOVERED/ RESOLVED	Probable/ Unrelated/ Unrelated/ Unrelated/ Unrelated/ Unrelated	9.1 days post	44.12	No
		9	Musculoskeletal and connective tissue disorders/ Myalgia/ Myalgia	15-SEP-2018/ 18:30/ (3)/ 16-SEP-2018/ 08:00/ (4)	Yes/ No	MILD/ NONE/ RECOVERED/ RESOLVED	Probable/ Unrelated/ Unrelated/ Unrelated/ Unrelated/ Unrelated	10.3 days post	13.50	No
		10	Investigations/ Neutrophil count decreased/ Neutrophil count decreased	16-SEP-2018/ 09:55/ (4)/ 18-SEP-2018/ 08:08/ (6)	Yes/ No	MODERATE/ NONE/ RECOVERED/ RESOLVED	Probable/ Unrelated/ Unrelated/ Unrelated/ Unrelated/ Unrelated	10.9 days post	46.22	No

Notes: Adverse events were coded using the Medical Dictionary for Regulatory Activities (MedDRA) Version 20.1.
A treatment-emergent adverse event is defined as an AE that occurred or worsened following first administration of study drug.
Rando = Randomisation; PP = Piperaquine phosphate; (d) = Day from IP dose.
AE No. = Adverse Event Number; TEAE = A treatment-emergent adverse event; SAE = Serious adverse event; UN = Unknown; Dis = Discontinuation

Listing 16.2.7.1
Adverse Events

Rando Number	Challenge Agent	AE No	System Organ Class/ Preferred Term/ Adverse Event Verbatim	Onset Date/ Onset Time/ (d) End Date/ End Time/ (d)	TEAE/ SAE	Severity/ Action Taken/ Outcome	Treatment Directly Related to AE (Inoculum/ Artesunate/ Piperaquine/ Malarone/ Primacin/ Mosquito)	Time of onset Relative to Inoculum/ Time of onset Relative to Artesunate/ Time of onset Relative to PP	Duration (hours)	Dis
R307	3D7	11	Nervous system disorders/ Headache/ Intermittent headache	16-SEP-2018/ 12:00/ (4)/ 17-SEP-2018/ 06:04/ (5)	Yes/ No	MILD/ NONE/ RECOVERED/ RESOLVED	Probable/ Unrelated/ Unrelated/ Unrelated/ Unrelated/ Unrelated	11 days post	18.07	No
		12	Investigations/ Aspartate aminotransferase increased/ Aspartate aminotransferase increased	18-SEP-2018/ 08:08/ (6)/ 22-SEP-2018/ 08:38/ (10)	Yes/ No	SEVERE/ NONE/ RECOVERING/ RESOLVING	Probable/ Unrelated/ Unrelated/ Unrelated/ Unrelated/ Unrelated	12.9 days post	96.50	No
		13	Investigations/ Alanine aminotransferase increased/ Alanine aminotransferase increased	18-SEP-2018/ 08:08/ (6)/ 25-SEP-2018/ 07:36/ (13)	Yes/ No	SEVERE/ NONE/ RECOVERING/ RESOLVING	Probable/ Unrelated/ Unrelated/ Unrelated/ Unrelated/ Unrelated	12.9 days post	167.47	No
		14	Respiratory, thoracic and mediastinal disorders/ Nasal congestion/ Nasal congestion	18-SEP-2018/ 11:30/ (6)/ 29-SEP-2018/ UN:UN/ (17)	Yes/ No	MILD/ NONE/ RECOVERED/ RESOLVED	Unrelated/ Unrelated/ Unrelated/ Unrelated/ Unrelated/ Unrelated	13 days post		No

Notes: Adverse events were coded using the Medical Dictionary for Regulatory Activities (MedDRA) Version 20.1.
A treatment-emergent adverse event is defined as an AE that occurred or worsened following first administration of study drug.
Rando = Randomisation; PP = Piperaquine phosphate; (d) = Day from IP dose.
AE No. = Adverse Event Number; TEAE = A treatment-emergent adverse event; SAE = Serious adverse event; UN = Unknown; Dis = Discontinuation

Listing 16.2.7.1
Adverse Events

Rando Number	Challenge Agent	AE No	System Organ Class/ Preferred Term/ Adverse Event Verbatim	Onset Date/ Onset Time/ (d) End Date/ End Time/ (d)	TEAE/ SAE	Severity/ Action Taken/ Outcome	Treatment Directly Related to AE (Inoculum/ Artesunate/ Piperaquine/ Malarone/ Primacin/ Mosquito)	Time of onset Relative to Inoculum/ Time of onset Relative to Artesunate/ Time of onset Relative to PP	Duration (hours)	Dis
R307	3D7	15	Nervous system disorders/ Headache/ Headache	20-SEP-2018/ 14:30/ (8)/ 20-SEP-2018/ 21:00/ (8)	Yes/ No	MILD/ MEDICATION: Ibuprofen/ RECOVERED/ RESOLVED	Probable/ Unrelated/ Unrelated/ Unrelated/ Unrelated/ Unrelated	15.1 days post	6.50	No
		16	Musculoskeletal and connective tissue disorders/ Myalgia/ Muscle ache	20-SEP-2018/ 14:30/ (8)/ 20-SEP-2018/ 21:00/ (8)	Yes/ No	MILD/ MEDICATION: Ibuprofen/ RECOVERED/ RESOLVED	Probable/ Unrelated/ Unrelated/ Unrelated/ Unrelated/ Unrelated	15.1 days post	6.50	No
		17	General disorders and administration site conditions/ Pyrexia/ Fever	20-SEP-2018/ 17:30/ (8)/ 20-SEP-2018/ 21:00/ (8)	Yes/ No	MILD/ MEDICATION: Ibuprofen/ RECOVERED/ RESOLVED	Probable/ Unrelated/ Unrelated/ Unrelated/ Unrelated/ Unrelated	15.3 days post	3.50	No
		18	Investigations/ Aspartate aminotransferase increased/ Aspartate transaminase increased	22-SEP-2018/ 08:38/ (10)/ 25-SEP-2018/ 07:36/ (13)	Yes/ No	MODERATE/ NONE/ RECOVERED/ RESOLVED	Probable/ Unrelated/ Unrelated/ Unrelated/ Unrelated/ Unrelated	16.9 days post	70.97	No

Notes: Adverse events were coded using the Medical Dictionary for Regulatory Activities (MedDRA) Version 20.1.
A treatment-emergent adverse event is defined as an AE that occurred or worsened following first administration of study drug.
Rando = Randomisation; PP = Piperaquine phosphate; (d) = Day from IP dose.
AE No. = Adverse Event Number; TEAE = A treatment-emergent adverse event; SAE = Serious adverse event; UN = Unknown; Dis = Discontinuation

Listing 16.2.7.1
Adverse Events

Rando Number	Challenge Agent	AE No	System Organ Class/ Preferred Term/ Adverse Event Verbatim	Onset Date/ Onset Time/ (d) End Date/ End Time/ (d)	TEAE/ SAE	Severity/ Action Taken/ Outcome	Treatment Directly Related to AE (Inoculum/ Artesunate/ Piperaquine/ Malarone/ Primacin/ Mosquito)	Time of onset Relative to Inoculum/ Time of onset Relative to Artesunate/ Time of onset Relative to PP	Duration (hours)	Dis
R307	3D7	19	Investigations/ Alanine aminotransferase increased/ Alanine aminotransferase increased	25-SEP-2018/ 07:36/ (13)/ 28-SEP-2018/ 07:40/ (16)	Yes/ No	MODERATE/ NONE/ RECOVERED/ RESOLVED	Probable/ Unrelated/ Unrelated/ Unrelated/ Unrelated/ Unrelated	19.8 days post	72.07	No

Notes: Adverse events were coded using the Medical Dictionary for Regulatory Activities (MedDRA) Version 20.1.
A treatment-emergent adverse event is defined as an AE that occurred or worsened following first administration of study drug.
Rando = Randomisation; PP = Piperaquine phosphate; (d) = Day from IP dose.
AE No. = Adverse Event Number; TEAE = A treatment-emergent adverse event; SAE = Serious adverse event; UN = Unknown; Dis = Discontinuation

16.2.7.1a Adverse Events Related to Inoculum Only

Listing 16.2.7.1a
Adverse Events Related to Inoculum Only

Rando Number	Challenge Agent	AE No	System Organ Class/ Preferred Term/ Adverse Event Verbatim	Onset Date/ Onset Time / End Date/ End Time	TEAE/ SAE	Severity/ Action Taken/ Outcome	Relationship to Inoculum	Time of onset Relative to Inoculum	Duration (hours)	Dis.
R103	K13	1	Nervous system disorders/ Lethargy/ Lethargy	19-NOV-2017/ 17:00/ 19-NOV-2017/ 19:00	No/ No	MILD/ NONE/ RECOVERED/RESOLVED	Possible	5.3 days post	2.00	No
		2	Blood and lymphatic system disorders/ Splenomegaly/ Splenomegaly	23-NOV-2017/ 09:16/ 26-NOV-2017/ 09:03	No/ No	MILD/ NONE/ RECOVERED/RESOLVED	Probable	8.9 days post	71.78	No
		3	General disorders and administration site conditions/ Fatigue/ Fatigue	23-NOV-2017/ 18:30/ 24-NOV-2017/ 10:00	Yes/ No	MILD/ NONE/ RECOVERED/RESOLVED	Probable	9.3 days post	15.50	No
		4	Nervous system disorders/ Headache/ Headache	23-NOV-2017/ 20:15/ 24-NOV-2017/ 11:50	Yes/ No	MILD/ MEDICATION: ibuprofen/ RECOVERED/RESOLVED	Probable	9.4 days post	15.58	No
		5	Musculoskeletal and connective tissue disorders/ Myalgia/ Myalgia	23-NOV-2017/ 21:01/ 24-NOV-2017/ 11:30	Yes/ No	MILD/ NONE/ RECOVERED/RESOLVED	Probable	9.4 days post	14.48	No
		6	Gastrointestinal disorders/ Abdominal tenderness/ Abdominal Tenderness	24-NOV-2017/ 08:00/ 24-NOV-2017/ 13:30	Yes/ No	MILD/ NONE/ RECOVERED/RESOLVED	Probable	9.9 days post	5.50	No
		7	General disorders and administration site conditions/ Chills/ Chills	24-NOV-2017/ 12:00/ 25-NOV-2017/ 07:00	Yes/ No	MILD/ NONE/ RECOVERED/RESOLVED	Probable	10.1 days post	19.00	No

Notes: Adverse events were coded using the Medical Dictionary for Regulatory Activities (MedDRA) Version 20.1
A treatment-emergent adverse event is defined as an AE that occurred or worsened following first administration of study drug.
Rando = Randomisation.
AE = Adverse Event; TEAE = A treatment-emergent adverse event; SAE = Serious adverse event; UN = Unknown; Dis. = Discontinuation

Listing 16.2.7.1a
Adverse Events Related to Inoculum Only

Rando Number	Challenge Agent	AE No	System Organ Class/ Preferred Term/ Adverse Event Verbatim	Onset Date/ Onset Time / End Date/ End Time	TEAE/ SAE	Severity/ Action Taken/ Outcome	Relationship to Inoculum	Time of onset Relative to Inoculum	Duration (hours)	Dis.
R103	K13	8	General disorders and administration site conditions/ Fatigue/ Fatigue	24-NOV-2017/ 18:00/ 25-NOV-2017/ 07:00	Yes/ No	MILD/ NONE/ RECOVERED/RESOLVED	Probable	10.3 days post	13.00	No
		9	General disorders and administration site conditions/ Malaise/ Malaise	24-NOV-2017/ 18:00/ 25-NOV-2017/ 07:00	Yes/ No	MILD/ NONE/ RECOVERED/RESOLVED	Probable	10.3 days post	13.00	No
		10	General disorders and administration site conditions/ Pyrexia/ Intermittent fever	24-NOV-2017/ 18:00/ 26-NOV-2017/ 19:23	Yes/ No	MILD/ NONE/ RECOVERED/RESOLVED	Probable	10.3 days post	49.38	No
		11	Musculoskeletal and connective tissue disorders/ Myalgia/ Myalgia	24-NOV-2017/ 18:00/ 26-NOV-2017/ 19:24	Yes/ No	MILD/ MEDICATION: Ibuprofen, Panadol/ RECOVERED/RESOLVED	Probable	10.3 days post	49.40	No
		12	Cardiac disorders/ Tachycardia/ tachycardia	24-NOV-2017/ 20:23/ 24-NOV-2017/ 20:24	Yes/ No	MILD/ NONE/ RECOVERED/RESOLVED	Probable	10.4 days post	0.02	No
		13	Nervous system disorders/ Headache/ Intermittent headache	25-NOV-2017/ 19:30/ 26-NOV-2017/ 19:24	Yes/ No	MILD/ MEDICATION: Ibuprofen/ RECOVERED/RESOLVED	Probable	11.4 days post	23.90	No
		14	Investigations/ Lymphocyte count decreased/ lymphocyctes count decreased	26-NOV-2017/ 10:53/ 30-NOV-2017/ 10:09	Yes/ No	SEVERE/ RECOVERED/RESOLVED	Probable	12 days post	95.27	No

Notes: Adverse events were coded using the Medical Dictionary for Regulatory Activities (MedDRA) Version 20.1
A treatment-emergent adverse event is defined as an AE that occurred or worsened following first administration of study drug.
Rando = Randomisation.
AE = Adverse Event; TEAE = A treatment-emergent adverse event; SAE = Serious adverse event; UN = Unknown; Dis. = Discontinuation

Listing 16.2.7.1a
Adverse Events Related to Inoculum Only

Rando Number	Challenge Agent	AE No	System Organ Class/ Preferred Term/ Adverse Event Verbatim	Onset Date/ Onset Time / End Date/ End Time	TEAE/ SAE	Severity/ Action Taken/ Outcome	Relationship to Inoculum	Time of onset Relative to Inoculum	Duration (hours)	Dis.
R103	K13	15	Nervous system disorders/ Dizziness/ dizziness	26-NOV-2017/ 22:00/ 27-NOV-2017/ 08:45	Yes/ No	MILD/ NONE/ RECOVERED/RESOLVED	Probable	12.5 days post	10.75	No
		16	General disorders and administration site conditions/ Pyrexia/ fever	26-NOV-2017/ 22:00/ 27-NOV-2017/ 08:45	Yes/ No	MILD/ NONE/ RECOVERED/RESOLVED	Probable	12.5 days post	10.75	No
		17	Nervous system disorders/ Headache/ intermittent headache	27-NOV-2017/ 08:45/ 29-NOV-2017/ 04:50	Yes/ No	MILD/ MEDICATION: Ibuprofen/ RECOVERED/RESOLVED	Probable	12.9 days post	44.08	No
		18	Nervous system disorders/ Headache/ headache	01-DEC-2017/ 14:30/ 02-DEC-2017/ 07:00	Yes/ No	MILD/ MEDICATION: Ibuprofen/ RECOVERED/RESOLVED	Probable	17.2 days post	16.50	No
R104	K13	1	General disorders and administration site conditions/ Fatigue/ Intermittent fatigue	22-NOV-2017/ 06:00/ 23-NOV-2017/ 15:00	No/ No	MILD/ NONE/ RECOVERED/RESOLVED	Probable	7.8 days post	33.00	No
		2	Musculoskeletal and connective tissue disorders/ Arthralgia/ Arthralgia	22-NOV-2017/ 09:00/ 22-NOV-2017/ 15:00	No/ No	MILD/ MEDICATION: Ibuprofen/ RECOVERED/RESOLVED	Probable	7.9 days post	6.00	No
		3	Nervous system disorders/ Headache/ Headache	23-NOV-2017/ 06:00/ 23-NOV-2017/ UN:UN	No/ No	MILD/ MEDICATION: Ibuprofen/ RECOVERED/RESOLVED	Probable	8.8 days post		No

Notes: Adverse events were coded using the Medical Dictionary for Regulatory Activities (MedDRA) Version 20.1
A treatment-emergent adverse event is defined as an AE that occurred or worsened following first administration of study drug.
Rando = Randomisation.
AE = Adverse Event; TEAE = A treatment-emergent adverse event; SAE = Serious adverse event; UN = Unknown; Dis. = Discontinuation

Listing 16.2.7.1a
Adverse Events Related to Inoculum Only

Rando Number	Challenge Agent	AE No	System Organ Class/ Preferred Term/ Adverse Event Verbatim	Onset Date/ Onset Time / End Date/ End Time	TEAE/ SAE	Severity/ Action Taken/ Outcome	Relationship to Inoculum	Time of onset Relative to Inoculum	Duration (hours)	Dis.
R104	K13	4	Musculoskeletal and connective tissue disorders/ Arthralgia/ Arthralgia	23-NOV-2017/ 06:00/ 24-NOV-2017/ 20:27	No/ No	MILD/ MEDICATION: Ibuprofen/ RECOVERED/RESOLVED	Probable	8.8 days post	38.45	No
		6	General disorders and administration site conditions/ Chills/ Chills	23-NOV-2017/ 15:00/ 24-NOV-2017/ 23:00	Yes/ No	MILD/ NONE/ RECOVERED/RESOLVED	Probable	9.2 days post	32.00	No
		7	Metabolism and nutrition disorders/ Decreased appetite/ Anorexia	23-NOV-2017/ 18:35/ 24-NOV-2017/ 08:15	Yes/ No	MILD/ NONE/ RECOVERED/RESOLVED	Probable	9.3 days post	13.67	No
		8	General disorders and administration site conditions/ Pyrexia/ Fever	23-NOV-2017/ 20:19/ 23-NOV-2017/ 23:22	Yes/ No	MILD/ MEDICATION: Ibuprofen/ RECOVERED/RESOLVED	Probable	9.4 days post	3.05	No
		9	Cardiac disorders/ Tachycardia/ Tachycardia	23-NOV-2017/ 20:19/ 24-NOV-2017/ 09:29	Yes/ No	MILD/ NONE/ RECOVERED/RESOLVED	Probable	9.4 days post	13.17	No
		10	Musculoskeletal and connective tissue disorders/ Myalgia/ Myalgia	24-NOV-2017/ 05:30/ 24-NOV-2017/ 23:00	Yes/ No	MILD/ MEDICATION: Ibuprofen, Paracetamol/ RECOVERED/RESOLVED	Probable	9.8 days post	17.50	No
		11	Nervous system disorders/ Headache/ Intermittent headache	24-NOV-2017/ 05:30/ 25-NOV-2017/ 20:04	Yes/ No	MILD/ MEDICATION: Ibuprofen, paracetamol/ RECOVERED/RESOLVED	Probable	9.8 days post	38.57	No

Notes: Adverse events were coded using the Medical Dictionary for Regulatory Activities (MedDRA) Version 20.1
A treatment-emergent adverse event is defined as an AE that occurred or worsened following first administration of study drug.
Rando = Randomisation.
AE = Adverse Event; TEAE = A treatment-emergent adverse event; SAE = Serious adverse event; UN = Unknown; Dis. = Discontinuation

Listing 16.2.7.1a
Adverse Events Related to Inoculum Only

Rando Number	Challenge Agent	AE No	System Organ Class/ Preferred Term/ Adverse Event Verbatim	Onset Date/ Onset Time / End Date/ End Time	TEAE/ SAE	Severity/ Action Taken/ Outcome	Relationship to Inoculum	Time of onset Relative to Inoculum	Duration (hours)	Dis.
R104	K13	12	General disorders and administration site conditions/ Pyrexia/ Fever	24-NOV-2017/ 06:44/ 24-NOV-2017/ 21:33	Yes/ No	MILD/ MEDICATION: Ibuprofen/ RECOVERED/RESOLVED	Probable	9.8 days post	14.82	No
		13	General disorders and administration site conditions/ Malaise/ Malaise	24-NOV-2017/ 19:00/ 24-NOV-2017/ 23:00	Yes/ No	MODERATE/ NONE/ RECOVERED/RESOLVED	Probable	10.3 days post	4.00	No
		14	Musculoskeletal and connective tissue disorders/ Arthralgia/ Intermittent arthralgia	24-NOV-2017/ 20:27/ 25-NOV-2017/ 09:19	Yes/ No	MODERATE/ MEDICATION: Ibuprofen/ RECOVERED/RESOLVED	Probable	10.4 days post	12.87	No
		15	Musculoskeletal and connective tissue disorders/ Arthralgia/ Arthralgia intermittent	25-NOV-2017/ 16:59/ 28-NOV-2017/ 08:28	Yes/ No	MILD/ MEDICATION: Ibuprofen/ RECOVERED/RESOLVED	Probable	11.3 days post	63.48	No
		16	Investigations/ Neutrophil count decreased/ Neutrophil count decreased	25-NOV-2017/ 17:43/ 26-NOV-2017/ 10:41	Yes/ No	SEVERE/ NONE/ RECOVERED/RESOLVED	Probable	11.3 days post	16.97	No
		17	Investigations/ White blood cell count decreased/ White cell count decreased	25-NOV-2017/ 17:43/ 30-NOV-2017/ 08:43	Yes/ No	MODERATE/ NONE/ RECOVERED/RESOLVED	Probable	11.3 days post	111.00	No
		18	Musculoskeletal and connective tissue disorders/ Neck pain/ Neck pain	25-NOV-2017/ 18:00/ 27-NOV-2017/ 08:00	Yes/ No	MODERATE/ NONE/ RECOVERED/RESOLVED	Possible	11.3 days post	38.00	No

Notes: Adverse events were coded using the Medical Dictionary for Regulatory Activities (MedDRA) Version 20.1
A treatment-emergent adverse event is defined as an AE that occurred or worsened following first administration of study drug.
Rando = Randomisation.
AE = Adverse Event; TEAE = A treatment-emergent adverse event; SAE = Serious adverse event; UN = Unknown; Dis. = Discontinuation

Listing 16.2.7.1a
Adverse Events Related to Inoculum Only

Rando Number	Challenge Agent	AE No	System Organ Class/ Preferred Term/ Adverse Event Verbatim	Onset Date/ Onset Time / End Date/ End Time	TEAE/ SAE	Severity/ Action Taken/ Outcome	Relationship to Inoculum	Time of onset Relative to Inoculum	Duration (hours)	Dis.
R104	K13	19	Nervous system disorders/ Headache/ Headache	25-NOV-2017/ 20:04/ 26-NOV-2017/ UN:UN	Yes/ No	MODERATE/ NONE/ RECOVERED/RESOLVED	Probable	11.4 days post		No
		20	Investigations/ Lymphocyte count decreased/ Lymphocyte count decreased	26-NOV-2017/ 10:41/ 30-NOV-2017/ 08:43	Yes/ No	MODERATE/ NONE/ RECOVERED/RESOLVED	Probable	12 days post	94.03	No
		21	Investigations/ Neutrophil count decreased/ Neutrophil count decreased	26-NOV-2017/ 10:41/ 30-NOV-2017/ 08:43	Yes/ No	MODERATE/ NONE/ RECOVERED/RESOLVED	Probable	12 days post	94.03	No
		22	Investigations/ Alanine aminotransferase increased/ Alanine Transaminase count increased	30-NOV-2017/ 08:43/ 03-DEC-2017/ 09:13	Yes/ No	SEVERE/ NONE/ RECOVERED/RESOLVED	Probable	15.9 days post	72.50	No
		23	Investigations/ Alanine aminotransferase increased/ Alanine transaminase count increased	03-DEC-2017/ 09:13/ 04-DEC-2017/ 12:45	Yes/ No	MODERATE/ NONE/ RECOVERED/RESOLVED	Probable	18.9 days post	27.53	No
R105	K13	1	Gastrointestinal disorders/ Nausea/ Nausea	19-NOV-2017/ 13:42/ 19-NOV-2017/ 14:00	No/ No	MILD/ NONE/ RECOVERED/RESOLVED	Possible	5.1 days post	0.30	No
		3	Cardiac disorders/ Tachycardia/ Tachycardia	20-NOV-2017/ 19:15/ 21-NOV-2017/ 07:47	No/ No	MILD/ NONE/ RECOVERED/RESOLVED	Possible	6.4 days post	12.53	No

Notes: Adverse events were coded using the Medical Dictionary for Regulatory Activities (MedDRA) Version 20.1
A treatment-emergent adverse event is defined as an AE that occurred or worsened following first administration of study drug.
Rando = Randomisation.
AE = Adverse Event; TEAE = A treatment-emergent adverse event; SAE = Serious adverse event; UN = Unknown; Dis. = Discontinuation

Listing 16.2.7.1a
Adverse Events Related to Inoculum Only

Rando Number	Challenge Agent	AE No	System Organ Class/ Preferred Term/ Adverse Event Verbatim	Onset Date/ Onset Time / End Date/ End Time	TEAE/ SAE	Severity/ Action Taken/ Outcome	Relationship to Inoculum	Time of onset Relative to Inoculum	Duration (hours)	Dis.
R105	K13	4	Cardiac disorders/ Tachycardia/ Intermittent tachycardia	24-NOV-2017/ 20:10/ 26-NOV-2017/ 18:55	Yes/ No	MILD/ NONE/ RECOVERED/RESOLVED	Probable	10.4 days post	46.75	No
		5	Nervous system disorders/ Headache/ Intermittent headache	27-NOV-2017/ 16:50/ 28-NOV-2017/ 19:01	Yes/ No	MILD/ NONE/ RECOVERED/RESOLVED	Probable	13.3 days post	26.18	No
R106	3D7	3	Musculoskeletal and connective tissue disorders/ Myalgia/ Myalgia	24-NOV-2017/ 07:00/ 24-NOV-2017/ 17:00	Yes/ No	MILD/ MEDICATION: Ibuprofen/ RECOVERED/RESOLVED	Probable	8.9 days post	10.00	No
		4	Nervous system disorders/ Headache/ Intermittent headache	24-NOV-2017/ 09:40/ 24-NOV-2017/ 17:00	Yes/ No	MILD/ MEDICATION: Ibuprofen/ RECOVERED/RESOLVED	Probable	9 days post	7.33	No
		5	General disorders and administration site conditions/ Fatigue/ Fatigue	24-NOV-2017/ 11:00/ 24-NOV-2017/ 17:00	Yes/ No	MILD/ NONE/ RECOVERED/RESOLVED	Probable	9 days post	6.00	No
		6	Skin and subcutaneous tissue disorders/ Hyperhidrosis/ Sweats	24-NOV-2017/ 12:30/ 24-NOV-2017/ 14:00	Yes/ No	MILD/ NONE/ RECOVERED/RESOLVED	Probable	9.1 days post	1.50	No
		7	General disorders and administration site conditions/ Malaise/ Malaise	24-NOV-2017/ 13:47/ 24-NOV-2017/ 20:10	Yes/ No	MILD/ NONE/ RECOVERED/RESOLVED	Probable	9.1 days post	6.38	No

Notes: Adverse events were coded using the Medical Dictionary for Regulatory Activities (MedDRA) Version 20.1
A treatment-emergent adverse event is defined as an AE that occurred or worsened following first administration of study drug.
Rando = Randomisation.
AE = Adverse Event; TEAE = A treatment-emergent adverse event; SAE = Serious adverse event; UN = Unknown; Dis. = Discontinuation

Listing 16.2.7.1a
Adverse Events Related to Inoculum Only

Rando Number	Challenge Agent	AE No	System Organ Class/ Preferred Term/ Adverse Event Verbatim	Onset Date/ Onset Time / End Date/ End Time	TEAE/ SAE	Severity/ Action Taken/ Outcome	Relationship to Inoculum	Time of onset Relative to Inoculum	Duration (hours)	Dis.
R106	3D7	8	Nervous system disorders/ Headache/ Headache	25-NOV-2017/ 18:00/ 25-NOV-2017/ 20:15	Yes/ No	MILD/ MEDICATION: Ibuprofen/ RECOVERED/RESOLVED	Probable	10.3 days post	2.25	No
		9	Nervous system disorders/ Headache/ Headache	26-NOV-2017/ 11:30/ 26-NOV-2017/ 12:30	Yes/ No	MILD/ MEDICATION: Ibuprofen/ RECOVERED/RESOLVED	Probable	11 days post	1.00	No
		14	Nervous system disorders/ Headache/ Intermittent headache	30-NOV-2017/ 11:00/ 03-DEC-2017/ 21:30	Yes/ No	MILD/ MEDICATION: Ibuprofen/ RECOVERED/RESOLVED	Probable	15 days post	82.50	No
		15	Investigations/ Lymphocyte count decreased/ Lymphocytes count decreased	01-DEC-2017/ 08:27/ 03-DEC-2017/ 09:39	Yes/ No	MODERATE/ NONE/ RECOVERED/RESOLVED	Probable	15.9 days post	49.20	No
		16	General disorders and administration site conditions/ Malaise/ Malaise	01-DEC-2017/ 08:30/ 01-DEC-2017/ 15:00	Yes/ No	MILD/ NONE/ RECOVERED/RESOLVED	Probable	15.9 days post	6.50	No
		17	General disorders and administration site conditions/ Pyrexia/ fever	01-DEC-2017/ 20:00/ 01-DEC-2017/ 21:30	Yes/ No	MILD/ MEDICATION: Ibuprofen/ RECOVERED/RESOLVED	Probable	16.4 days post	1.50	No
R107	3D7	1	Gastrointestinal disorders/ Nausea/ Nausea	20-NOV-2017/ 23:30/ 24-NOV-2017/ 06:10	No/ No	MILD/ MEDICATION: Ondansetron/ RECOVERED/RESOLVED	Probable	5.5 days post	78.67	No

Notes: Adverse events were coded using the Medical Dictionary for Regulatory Activities (MedDRA) Version 20.1
A treatment-emergent adverse event is defined as an AE that occurred or worsened following first administration of study drug.
Rando = Randomisation.
AE = Adverse Event; TEAE = A treatment-emergent adverse event; SAE = Serious adverse event; UN = Unknown; Dis. = Discontinuation

Listing 16.2.7.1a
Adverse Events Related to Inoculum Only

Rando Number	Challenge Agent	AE No	System Organ Class/ Preferred Term/ Adverse Event Verbatim	Onset Date/ Onset Time / End Date/ End Time	TEAE/ SAE	Severity/ Action Taken/ Outcome	Relationship to Inoculum	Time of onset Relative to Inoculum	Duration (hours)	Dis.
R107	3D7	2	Gastrointestinal disorders/ Abdominal tenderness/ Abdominal tenderness	21-NOV-2017/ 07:40/ 26-NOV-2017/ 08:59	No/ No	MILD/ NONE/ RECOVERED/RESOLVED	Probable	5.9 days post	121.32	No
		3	General disorders and administration site conditions/ Fatigue/ Fatigue	21-NOV-2017/ 07:55/ 24-NOV-2017/ 08:15	No/ No	MILD/ NONE/ RECOVERED/RESOLVED	Probable	5.9 days post	72.33	No
		4	Metabolism and nutrition disorders/ Decreased appetite/ Anorexia	21-NOV-2017/ 11:30/ 22-NOV-2017/ 16:00	No/ No	MILD/ NONE/ RECOVERED/RESOLVED	Probable	6 days post	28.50	No
		5	Musculoskeletal and connective tissue disorders/ Arthralgia/ Intermittent arthralgia	21-NOV-2017/ 12:00/ 23-NOV-2017/ 20:45	No/ No	MILD/ NONE/ RECOVERED/RESOLVED	Probable	6.1 days post	56.75	No
		6	Nervous system disorders/ Headache/ Headache	22-NOV-2017/ 14:00/ 23-NOV-2017/ 07:00	No/ No	MILD/ NONE/ RECOVERED/RESOLVED	Probable	7.1 days post	17.00	No
		7	Gastrointestinal disorders/ Abdominal discomfort/ Abdominal discomfort intermittent	26-NOV-2017/ 12:00/ 01-DEC-2017/ 21:00	Yes/ No	MILD/ NONE/ RECOVERED/RESOLVED	Probable	11.1 days post	129.00	No
		8	Musculoskeletal and connective tissue disorders/ Myalgia/ Myalgia	01-DEC-2017/ 19:00/ 04-DEC-2017/ 12:00	Yes/ No	MODERATE/ MEDICATION: Ondansetron/ RECOVERED/RESOLVED	Probable	16.4 days post	65.00	No

Notes: Adverse events were coded using the Medical Dictionary for Regulatory Activities (MedDRA) Version 20.1
A treatment-emergent adverse event is defined as an AE that occurred or worsened following first administration of study drug.
Rando = Randomisation.
AE = Adverse Event; TEAE = A treatment-emergent adverse event; SAE = Serious adverse event; UN = Unknown; Dis. = Discontinuation

Listing 16.2.7.1a
Adverse Events Related to Inoculum Only

Rando Number	Challenge Agent	AE No	System Organ Class/ Preferred Term/ Adverse Event Verbatim	Onset Date/ Onset Time / End Date/ End Time	TEAE/ SAE	Severity/ Action Taken/ Outcome	Relationship to Inoculum	Time of onset Relative to Inoculum	Duration (hours)	Dis.
R107	3D7	9	Gastrointestinal disorders/ Abdominal tenderness/ Abdominal tenderness	04-DEC-2017/ 08:44/ 05-DEC-2017/ 07:57	Yes/ No	MILD/ NONE/ RECOVERED/RESOLVED	Probable	18.9 days post	23.22	No
		10	Nervous system disorders/ Headache/ Headache	04-DEC-2017/ 13:00/ 04-DEC-2017/ 22:15	Yes/ No	MILD/ MEDICATION: Ibuprofen/ RECOVERED/RESOLVED	Probable	19.1 days post	9.25	No
		11	Nervous system disorders/ Dizziness/ Dizziness	05-DEC-2017/ 05:00/ 06-DEC-2017/ 09:00	Yes/ No	MILD/ NONE/ RECOVERED/RESOLVED	Probable	19.8 days post	28.00	No
R108	3D7	1	Nervous system disorders/ Headache/ Headache	22-NOV-2017/ 22:00/ 23-NOV-2017/ 06:00	No/ No	MILD/ MEDICATION: Paracetamol/ RECOVERED/RESOLVED	Probable	7.5 days post	8.00	No
		2	General disorders and administration site conditions/ Fatigue/ Fatigue	23-NOV-2017/ 06:00/ 23-NOV-2017/ 10:00	No/ No	MILD/ NONE/ RECOVERED/RESOLVED	Probable	7.8 days post	4.00	No
		3	Investigations/ Urobilinogen urine/ Urobilinogenuria	23-NOV-2017/ 08:03/ 28-NOV-2017/ 08:54	No/ No	MODERATE/ NONE/ RECOVERED/RESOLVED	Probable	7.9 days post	120.85	No
		4	Nervous system disorders/ Headache/ Intermittent headache	23-NOV-2017/ 17:00/ 25-NOV-2017/ 21:00	Yes/ No	MILD/ MEDICATION: Ibuprofen/ RECOVERED/RESOLVED	Probable	8.3 days post	52.00	No

Notes: Adverse events were coded using the Medical Dictionary for Regulatory Activities (MedDRA) Version 20.1
A treatment-emergent adverse event is defined as an AE that occurred or worsened following first administration of study drug.
Rando = Randomisation.
AE = Adverse Event; TEAE = A treatment-emergent adverse event; SAE = Serious adverse event; UN = Unknown; Dis. = Discontinuation

Listing 16.2.7.1a
Adverse Events Related to Inoculum Only

Rando Number	Challenge Agent	AE No	System Organ Class/ Preferred Term/ Adverse Event Verbatim	Onset Date/ Onset Time / End Date/ End Time	TEAE/ SAE	Severity/ Action Taken/ Outcome	Relationship to Inoculum	Time of onset Relative to Inoculum	Duration (hours)	Dis.
R108	3D7	5	General disorders and administration site conditions/ Fatigue/ Intermittent fatigue	23-NOV-2017/ 18:30/ 24-NOV-2017/ 08:23	Yes/ No	MODERATE/ NONE/ RECOVERED/RESOLVED	Probable	8.3 days post	13.88	No
		6	General disorders and administration site conditions/ Pyrexia/ Fever	23-NOV-2017/ 19:55/ 23-NOV-2017/ 21:54	Yes/ No	MILD/ NONE/ NOT RECOVERED/ NOT RESOLVED	Probable	8.4 days post	1.98	No
		7	General disorders and administration site conditions/ Pyrexia/ fever	23-NOV-2017/ 21:54/ 23-NOV-2017/ 23:14	Yes/ No	MODERATE/ NONE/ RECOVERED/RESOLVED	Probable	8.5 days post	1.33	No
		8	General disorders and administration site conditions/ Pyrexia/ intermittent fever	23-NOV-2017/ 23:14/ 24-NOV-2017/ 18:55	Yes/ No	MILD/ MEDICATION: Ibuprofen/ RECOVERED/RESOLVED	Probable	8.5 days post	19.68	No
		9	Gastrointestinal disorders/ Abdominal discomfort/ Intermittent abdominal discomfort	24-NOV-2017/ 05:00/ 24-NOV-2017/ 19:51	Yes/ No	MODERATE/ MEDICATION: Ibuprofen/ RECOVERED/RESOLVED	Probable	8.8 days post	14.85	No
		12	General disorders and administration site conditions/ Chills/ chills	24-NOV-2017/ 08:33/ 24-NOV-2017/ 13:30	Yes/ No	MILD/ NONE/ RECOVERED/RESOLVED	Probable	8.9 days post	4.95	No
		13	Skin and subcutaneous tissue disorders/ Hyperhidrosis/ sweating	24-NOV-2017/ 08:33/ 24-NOV-2017/ 13:30	Yes/ No	MILD/ NONE/ RECOVERED/RESOLVED	Probable	8.9 days post	4.95	No

Notes: Adverse events were coded using the Medical Dictionary for Regulatory Activities (MedDRA) Version 20.1
A treatment-emergent adverse event is defined as an AE that occurred or worsened following first administration of study drug.
Rando = Randomisation.
AE = Adverse Event; TEAE = A treatment-emergent adverse event; SAE = Serious adverse event; UN = Unknown; Dis. = Discontinuation

Listing 16.2.7.1a
Adverse Events Related to Inoculum Only

Rando Number	Challenge Agent	AE No	System Organ Class/ Preferred Term/ Adverse Event Verbatim	Onset Date/ Onset Time / End Date/ End Time	TEAE/ SAE	Severity/ Action Taken/ Outcome	Relationship to Inoculum	Time of onset Relative to Inoculum	Duration (hours)	Dis.
R108	3D7	14	Cardiac disorders/ Tachycardia/ Tachycardia	24-NOV-2017/ 12:43/ 24-NOV-2017/ 19:47	Yes/ No	MILD/ NONE/ RECOVERED/RESOLVED	Probable	9.1 days post	7.07	No
		15	General disorders and administration site conditions/ Malaise/ Malaise	24-NOV-2017/ 13:00/ 24-NOV-2017/ 19:51	Yes/ No	MILD/ NONE/ RECOVERED/RESOLVED	Probable	9.1 days post	6.85	No
		16	Gastrointestinal disorders/ Nausea/ Nausea	24-NOV-2017/ 13:00/ 24-NOV-2017/ 19:51	Yes/ No	MILD/ NONE/ RECOVERED/RESOLVED	Probable	9.1 days post	6.85	No
		17	General disorders and administration site conditions/ Fatigue/ fatigue	24-NOV-2017/ 13:30/ 25-NOV-2017/ 12:08	Yes/ No	MILD/ NONE/ RECOVERED/RESOLVED	Probable	9.1 days post	22.63	No
		18	Investigations/ Alanine aminotransferase increased/ Increased alanine aminotransferase	30-NOV-2017/ 06:57/ 01-DEC-2017/ 07:14	Yes/ No	MODERATE/ NONE/ RECOVERED/RESOLVED	Probable	14.8 days post	24.28	No
		19	Nervous system disorders/ Headache/ headache	01-DEC-2017/ 09:20/ 01-DEC-2017/ 12:30	Yes/ No	MODERATE/ MEDICATION: ibuprofen/ RECOVERED/RESOLVED	Probable	15.9 days post	3.17	No
		20	General disorders and administration site conditions/ Fatigue/ Fatigue	01-DEC-2017/ 09:20/ 02-DEC-2017/ 15:00	Yes/ No	MILD/ NONE/ RECOVERED/RESOLVED	Probable	15.9 days post	29.67	No

Notes: Adverse events were coded using the Medical Dictionary for Regulatory Activities (MedDRA) Version 20.1
A treatment-emergent adverse event is defined as an AE that occurred or worsened following first administration of study drug.
Rando = Randomisation.
AE = Adverse Event; TEAE = A treatment-emergent adverse event; SAE = Serious adverse event; UN = Unknown; Dis. = Discontinuation

Listing 16.2.7.1a
Adverse Events Related to Inoculum Only

Rando Number	Challenge Agent	AE No	System Organ Class/ Preferred Term/ Adverse Event Verbatim	Onset Date/ Onset Time / End Date/ End Time	TEAE/ SAE	Severity/ Action Taken/ Outcome	Relationship to Inoculum	Time of onset Relative to Inoculum	Duration (hours)	Dis.
R108	3D7	21	General disorders and administration site conditions/ Malaise/ Malaise	02-DEC-2017/ 03:00/ 02-DEC-2017/ 15:00	Yes/ No	MILD/ NONE/ RECOVERED/RESOLVED	Probable	16.7 days post	12.00	No
		22	Nervous system disorders/ Lethargy/ lethargy	02-DEC-2017/ 07:00/ 02-DEC-2017/ 15:00	Yes/ No	MILD/ NONE/ RECOVERED/RESOLVED	Probable	16.9 days post	8.00	No
		24	Gastrointestinal disorders/ Nausea/ intermittent nausea	12-DEC-2017/ 09:00/ 13-DEC-2017/ 14:00	Yes/ No	MILD/ NONE/ RECOVERED/RESOLVED	Probable	26.9 days post	29.00	No
R201	K13	1	Respiratory, thoracic and mediastinal disorders/ Oropharyngeal pain/ Sore throat	01-MAR-2018/ 16:50/ 01-MAR-2018/ 19:00	Yes/ No	MILD/ MEDICATION: ibuprofen/ RECOVERED/RESOLVED	Probable	10.3 days post	2.17	No
		2	Nervous system disorders/ Headache/ headache	01-MAR-2018/ 16:50/ 01-MAR-2018/ 19:00	Yes/ No	MODERATE/ MEDICATION: ibuprofen/ RECOVERED/RESOLVED	Probable	10.3 days post	2.17	No
		3	Investigations/ White blood cell count decreased/ white cell count decreased	02-MAR-2018/ 13:24/ 03-MAR-2018/ 13:27	Yes/ No	MODERATE/ NONE/ RECOVERED/RESOLVED	Probable	11.1 days post	24.05	No
		4	Investigations/ Neutrophil count decreased/ neutrophil count decreased	02-MAR-2018/ 13:24/ 05-MAR-2018/ 08:54	Yes/ No	MODERATE/ NONE/ RECOVERED/RESOLVED	Probable	11.1 days post	67.50	No

Notes: Adverse events were coded using the Medical Dictionary for Regulatory Activities (MedDRA) Version 20.1
A treatment-emergent adverse event is defined as an AE that occurred or worsened following first administration of study drug.
Rando = Randomisation.
AE = Adverse Event; TEAE = A treatment-emergent adverse event; SAE = Serious adverse event; UN = Unknown; Dis. = Discontinuation

Listing 16.2.7.1a
Adverse Events Related to Inoculum Only

Rando Number	Challenge Agent	AE No	System Organ Class/ Preferred Term/ Adverse Event Verbatim	Onset Date/ Onset Time / End Date/ End Time	TEAE/ SAE	Severity/ Action Taken/ Outcome	Relationship to Inoculum	Time of onset Relative to Inoculum	Duration (hours)	Dis.
R202	K13	1	Musculoskeletal and connective tissue disorders/ Arthralgia/ arthralgia	02-MAR-2018/ 23:00/ 03-MAR-2018/ 08:00	Yes/ No	MILD/ NONE/ RECOVERED/RESOLVED	Probable	11.5 days post	9.00	No
		2	General disorders and administration site conditions/ Feeling hot/ Feeling hot	03-MAR-2018/ UN:UN/ 04-MAR-2018/ UN:UN	Yes/ No	MILD/ MEDICATION: Ibuprofen/ RECOVERED/RESOLVED	Probable	11.6 days post		No
		3	General disorders and administration site conditions/ Pyrexia/ fever	03-MAR-2018/ 09:42/ 03-MAR-2018/ 11:06	Yes/ No	MILD/ MEDICATION: ibuprofen/ RECOVERED/RESOLVED	Probable	12 days post	1.40	No
		4	Investigations/ Alanine aminotransferase increased/ alanine transaminase increased	09-MAR-2018/ 09:32/ 12-MAR-2018/ 09:51	Yes/ No	MODERATE/ NONE/ RECOVERED/RESOLVED	Probable	18 days post	72.32	No
R203	K13	1	Nervous system disorders/ Headache/ Intermittent headache	01-MAR-2018/ 07:00/ 11-MAR-2018/ 07:00	Yes/ No	MILD/ MEDICATION: ibuprofen and paracetamol/ RECOVERED/RESOLVED	Probable	9.9 days post	240.00	No
		2	Gastrointestinal disorders/ Nausea/ nausea	01-MAR-2018/ 09:10/ 01-MAR-2018/ 12:00	Yes/ No	MILD/ MEDICATION: ondansetron/ RECOVERED/RESOLVED	Probable	9.9 days post	2.83	No
		3	Musculoskeletal and connective tissue disorders/ Myalgia/ Myalgia	03-MAR-2018/ 07:00/ 03-MAR-2018/ 22:00	Yes/ No	MILD/ MEDICATION: ibuprofen and paracetamol/ RECOVERED/RESOLVED	Probable	11.9 days post	15.00	No

Notes: Adverse events were coded using the Medical Dictionary for Regulatory Activities (MedDRA) Version 20.1
A treatment-emergent adverse event is defined as an AE that occurred or worsened following first administration of study drug.
Rando = Randomisation.
AE = Adverse Event; TEAE = A treatment-emergent adverse event; SAE = Serious adverse event; UN = Unknown; Dis. = Discontinuation

Listing 16.2.7.1a
Adverse Events Related to Inoculum Only

Rando Number	Challenge Agent	AE No	System Organ Class/ Preferred Term/ Adverse Event Verbatim	Onset Date/ Onset Time / End Date/ End Time	TEAE/ SAE	Severity/ Action Taken/ Outcome	Relationship to Inoculum	Time of onset Relative to Inoculum	Duration (hours)	Dis.
R203	K13	4	Gastrointestinal disorders/ Nausea/ Intermittent nausea	03-MAR-2018/ 12:30/ 06-MAR-2018/ 06:30	Yes/ No	MILD/ MEDICATION: ondansetron/ RECOVERED/RESOLVED	Probable	12.1 days post	66.00	No
		6	General disorders and administration site conditions/ Chills/ chills	03-MAR-2018/ 17:00/ 04-MAR-2018/ 06:00	Yes/ No	MILD/ MEDICATION: ibuprfen and paracetamol/ RECOVERED/RESOLVED	Probable	12.3 days post	13.00	No
		8	General disorders and administration site conditions/ Pyrexia/ fever	03-MAR-2018/ 20:54/ 04-MAR-2018/ 08:45	Yes/ No	MILD/ MEDICATION: ibuprofen and paracetamol/ RECOVERED/RESOLVED	Probable	12.4 days post	11.85	No
		9	General disorders and administration site conditions/ Malaise/ Malaise	04-MAR-2018/ 06:00/ 06-MAR-2018/ 06:30	Yes/ No	MILD/ MEDICATION: Ibuprofen, paracetamol/ RECOVERED/RESOLVED	Probable	12.8 days post	48.50	No
		10	Musculoskeletal and connective tissue disorders/ Myalgia/ Intermittent Myalgia	04-MAR-2018/ 08:50/ 11-MAR-2018/ 07:00	Yes/ No	MILD/ MEDICATION: Ibuprofen, paracetamol/ RECOVERED/RESOLVED	Probable	12.9 days post	166.17	No
		11	General disorders and administration site conditions/ Pyrexia/ Fever	04-MAR-2018/ 12:15/ 04-MAR-2018/ 20:11	Yes/ No	MILD/ NONE/ RECOVERED/RESOLVED	Probable	13.1 days post	7.93	No
		12	Musculoskeletal and connective tissue disorders/ Arthralgia/ arthralgia	04-MAR-2018/ 20:13/ 05-MAR-2018/ 09:22	Yes/ No	MILD/ NONE/ RECOVERED/RESOLVED	Probable	13.4 days post	13.15	No

Notes: Adverse events were coded using the Medical Dictionary for Regulatory Activities (MedDRA) Version 20.1
A treatment-emergent adverse event is defined as an AE that occurred or worsened following first administration of study drug.
Rando = Randomisation.
AE = Adverse Event; TEAE = A treatment-emergent adverse event; SAE = Serious adverse event; UN = Unknown; Dis. = Discontinuation

Listing 16.2.7.1a
Adverse Events Related to Inoculum Only

Rando Number	Challenge Agent	AE No	System Organ Class/ Preferred Term/ Adverse Event Verbatim	Onset Date/ Onset Time / End Date/ End Time	TEAE/ SAE	Severity/ Action Taken/ Outcome	Relationship to Inoculum	Time of onset Relative to Inoculum	Duration (hours)	Dis.
R203	K13	13	Investigations/ Neutrophil count decreased/ Neutrophil count decreased	05-MAR-2018/ 09:40/ 09-MAR-2018/ 10:14	Yes/ No	MODERATE/ NONE/ RECOVERED/RESOLVED	Probable	14 days post	96.57	No
R204	K13	3	Nervous system disorders/ Headache/ intermittent headache	01-MAR-2018/ 10:30/ 01-MAR-2018/ 13:30	Yes/ No	MILD/ MEDICATION: ibuprofen/ RECOVERED/RESOLVED	Probable	10 days post	3.00	No
		6	Investigations/ Lymphocyte count decreased/ Lymphocyte count decreased	03-MAR-2018/ 13:21/ 09-MAR-2018/ 09:00	Yes/ No	MODERATE/ NONE/ RECOVERED/RESOLVED	Probable	12.1 days post	139.65	No
		7	Investigations/ White blood cell count decreased/ White cell count decreased	03-MAR-2018/ 13:21/ 09-MAR-2018/ 09:00	Yes/ No	MODERATE/ NONE/ RECOVERED/RESOLVED	Probable	12.1 days post	139.65	No
		8	Nervous system disorders/ Headache/ Intermittent headache	04-MAR-2018/ 13:30/ 06-MAR-2018/ 05:30	Yes/ No	MILD/ MEDICATION: ibuprofen/ RECOVERED/RESOLVED	Probable	13.1 days post	40.00	No
		9	Investigations/ Neutrophil count decreased/ Neutrophil count decreased	05-MAR-2018/ 08:37/ 09-MAR-2018/ 09:00	Yes/ No	MODERATE/ NONE/ RECOVERED/RESOLVED	Probable	13.9 days post	96.38	No
R205	K13	2	Musculoskeletal and connective tissue disorders/ Back pain/ lower back pain	28-FEB-2018/ 22:00/ 02-MAR-2018/ 10:00	Yes/ No	MILD/ MEDICATION: ibuprofen/ RECOVERED/RESOLVED	Probable	9.5 days post	36.00	No

Notes: Adverse events were coded using the Medical Dictionary for Regulatory Activities (MedDRA) Version 20.1
A treatment-emergent adverse event is defined as an AE that occurred or worsened following first administration of study drug.
Rando = Randomisation.
AE = Adverse Event; TEAE = A treatment-emergent adverse event; SAE = Serious adverse event; UN = Unknown; Dis. = Discontinuation

Listing 16.2.7.1a
Adverse Events Related to Inoculum Only

Rando Number	Challenge Agent	AE No	System Organ Class/ Preferred Term/ Adverse Event Verbatim	Onset Date/ Onset Time / End Date/ End Time	TEAE/ SAE	Severity/ Action Taken/ Outcome	Relationship to Inoculum	Time of onset Relative to Inoculum	Duration (hours)	Dis.
R205	K13	3	General disorders and administration site conditions/ Pyrexia/ fever	01-MAR-2018/ 13:49/ 01-MAR-2018/ 13:51	Yes/ No	MILD/ NONE/ RECOVERED/RESOLVED	Probable	10.1 days post	0.03	No
		4	Nervous system disorders/ Headache/ intermittent headache	01-MAR-2018/ 17:18/ 02-MAR-2018/ 10:00	Yes/ No	MILD/ MEDICATION: paracetamol/ RECOVERED/RESOLVED	Probable	10.3 days post	16.70	No
		5	Skin and subcutaneous tissue disorders/ Hyperhidrosis/ sweats	01-MAR-2018/ 18:30/ 02-MAR-2018/ 10:00	Yes/ No	MILD/ NONE/ RECOVERED/RESOLVED	Probable	10.3 days post	15.50	No
		6	Investigations/ Neutrophil count decreased/ neutrophil count decreased	02-MAR-2018/ 13:11/ 09-MAR-2018/ 07:52	Yes/ No	MODERATE/ NONE/ RECOVERED/RESOLVED	Probable	11.1 days post	162.68	No
		7	Investigations/ White blood cell count decreased/ white cell count decreased	02-MAR-2018/ 13:11/ 09-MAR-2018/ 07:52	Yes/ No	MODERATE/ NONE/ RECOVERED/RESOLVED	Probable	11.1 days post	162.68	No
		8	Skin and subcutaneous tissue disorders/ Hyperhidrosis/ intermittent sweats	04-MAR-2018/ 00:00/ 06-MAR-2018/ 20:00	Yes/ No	MILD/ NONE/ RECOVERED/RESOLVED	Probable	12.6 days post	68.00	No
R206	K13	3	Musculoskeletal and connective tissue disorders/ Myalgia/ intermittent myalgia	28-FEB-2018/ 09:30/ 03-MAR-2018/ 07:00	No/ No	MILD/ MEDICATION: Ibuprofen, paracetamol/ RECOVERED/RESOLVED	Probable	9 days post	69.50	No

Notes: Adverse events were coded using the Medical Dictionary for Regulatory Activities (MedDRA) Version 20.1
A treatment-emergent adverse event is defined as an AE that occurred or worsened following first administration of study drug.
Rando = Randomisation.
AE = Adverse Event; TEAE = A treatment-emergent adverse event; SAE = Serious adverse event; UN = Unknown; Dis. = Discontinuation

Listing 16.2.7.1a
Adverse Events Related to Inoculum Only

Rando Number	Challenge Agent	AE No	System Organ Class/ Preferred Term/ Adverse Event Verbatim	Onset Date/ Onset Time / End Date/ End Time	TEAE/ SAE	Severity/ Action Taken/ Outcome	Relationship to Inoculum	Time of onset Relative to Inoculum	Duration (hours)	Dis.
R206	K13	4	Nervous system disorders/ Headache/ intermittent headache	28-FEB-2018/ 09:46/ 19-MAR-2018/ 07:00	No/ No	MILD/ MEDICATION: Ibuprofen, paracetamol/ RECOVERED/RESOLVED	Probable	9 days post	453.23	No
		5	Investigations/ Lymphocyte count decreased/ lymphocyte count decreased	28-FEB-2018/ 12:48/ 05-MAR-2018/ 09:30	No/ No	MODERATE/ NONE/ RECOVERED/RESOLVED	Probable	9.1 days post	116.70	No
		6	General disorders and administration site conditions/ Pyrexia/ Fever	01-MAR-2018/ 09:38/ 01-MAR-2018/ 09:39	Yes/ No	MILD/ NONE/ RECOVERED/RESOLVED	Probable	10 days post	0.02	No
		7	Investigations/ Neutrophil count decreased/ neutrophil count decreased	02-MAR-2018/ 13:29/ 05-MAR-2018/ 09:30	Yes/ No	SEVERE/ NONE/ RECOVERED/RESOLVED	Probable	11.1 days post	68.02	No
		8	Investigations/ White blood cell count decreased/ white cell count decreased	02-MAR-2018/ 13:29/ 05-MAR-2018/ 09:30	Yes/ No	SEVERE/ NONE/ RECOVERED/RESOLVED	Probable	11.1 days post	68.02	No
		9	Gastrointestinal disorders/ Nausea/ Nausea	02-MAR-2018/ 14:00/ 02-MAR-2018/ 19:00	Yes/ No	MILD/ NONE/ RECOVERED/RESOLVED	Probable	11.1 days post	5.00	No
		10	Gastrointestinal disorders/ Abdominal discomfort/ intermittent abdominal discomfort	02-MAR-2018/ 21:00/ 03-MAR-2018/ 21:00	Yes/ No	MILD/ NONE/ RECOVERED/RESOLVED	Probable	11.4 days post	24.00	No

Notes: Adverse events were coded using the Medical Dictionary for Regulatory Activities (MedDRA) Version 20.1
A treatment-emergent adverse event is defined as an AE that occurred or worsened following first administration of study drug.
Rando = Randomisation.
AE = Adverse Event; TEAE = A treatment-emergent adverse event; SAE = Serious adverse event; UN = Unknown; Dis. = Discontinuation

Listing 16.2.7.1a
Adverse Events Related to Inoculum Only

Rando Number	Challenge Agent	AE No	System Organ Class/ Preferred Term/ Adverse Event Verbatim	Onset Date/ Onset Time / End Date/ End Time	TEAE/ SAE	Severity/ Action Taken/ Outcome	Relationship to Inoculum	Time of onset Relative to Inoculum	Duration (hours)	Dis.
R206	K13	11	Musculoskeletal and connective tissue disorders/ Myalgia/ intermittent myalgia	11-MAR-2018/ 20:00/ 13-MAR-2018/ 06:00	Yes/ No	MILD/ MEDICATION: ibuprofen/ RECOVERED/RESOLVED	Probable	20.4 days post	34.00	No
R207	K13	2	Nervous system disorders/ Headache/ intermittent headache	01-MAR-2018/ 07:00/ 03-MAR-2018/ 16:15	Yes/ No	MILD/ MEDICATION: ibuprofen/ RECOVERED/RESOLVED	Probable	9.8 days post	57.25	No
		3	General disorders and administration site conditions/ Pyrexia/ fever	01-MAR-2018/ 08:53/ 01-MAR-2018/ 08:55	Yes/ No	MILD/ NONE/ RECOVERED/RESOLVED	Probable	9.9 days post	0.03	No
		4	Investigations/ Lymphocyte count decreased/ lymphocyte count decreased	02-MAR-2018/ 12:59/ 03-MAR-2018/ 13:00	Yes/ No	MODERATE/ NONE/ RECOVERED/RESOLVED	Probable	11.1 days post	24.02	No
		5	Musculoskeletal and connective tissue disorders/ Arthralgia/ arthralgia	03-MAR-2018/ 09:15/ 03-MAR-2018/ 16:00	Yes/ No	MILD/ NONE/ RECOVERED/RESOLVED	Probable	11.9 days post	6.75	No
R208	3D7	1	General disorders and administration site conditions/ Pyrexia/ fever	28-FEB-2018/ 19:59/ 28-FEB-2018/ 20:01	Yes/ No	MILD/ NONE/ RECOVERED/RESOLVED	Probable	8.4 days post	0.03	No
		2	Investigations/ Alanine aminotransferase increased/ alanine transaminase increased	03-MAR-2018/ 11:36/ 05-MAR-2018/ 07:07	Yes/ No	MODERATE/ NONE/ NOT RECOVERED/ NOT RESOLVED	Probable	11.1 days post	43.52	No

Notes: Adverse events were coded using the Medical Dictionary for Regulatory Activities (MedDRA) Version 20.1
A treatment-emergent adverse event is defined as an AE that occurred or worsened following first administration of study drug.
Rando = Randomisation.
AE = Adverse Event; TEAE = A treatment-emergent adverse event; SAE = Serious adverse event; UN = Unknown; Dis. = Discontinuation

Listing 16.2.7.1a
Adverse Events Related to Inoculum Only

Rando Number	Challenge Agent	AE No	System Organ Class/ Preferred Term/ Adverse Event Verbatim	Onset Date/ Onset Time / End Date/ End Time	TEAE/ SAE	Severity/ Action Taken/ Outcome	Relationship to Inoculum	Time of onset Relative to Inoculum	Duration (hours)	Dis.
R208	3D7	3	Investigations/ Aspartate aminotransferase increased/ Aspartate transaminase increased	05-MAR-2018/ 07:07/ 09-MAR-2018/ 07:19	Yes/ No	MODERATE/ NONE/ RECOVERED/RESOLVED	Probable	12.9 days post	96.20	No
		4	Investigations/ Alanine aminotransferase increased/ Alanine transaminase increased	05-MAR-2018/ 07:07/ 19-MAR-2018/ 07:50	Yes/ No	SEVERE/ NONE/ RECOVERED/RESOLVED	Probable	12.9 days post	336.72	No
		5	Investigations/ Aspartate aminotransferase increased/ aspartate transaminase increased	10-MAR-2018/ 07:52/ 13-MAR-2018/ 07:22	Yes/ No	MODERATE/ NONE/ RECOVERED/RESOLVED	Probable	17.9 days post	71.50	No
		6	Investigations/ Alanine aminotransferase increased/ Alanine transaminase increased	19-MAR-2018/ 07:01/ 22-MAR-2018/ 07:50	Yes/ No	MODERATE/ NONE/ RECOVERED/RESOLVED	Probable	26.9 days post	72.82	No
R209	3D7	1	Cardiac disorders/ Ventricular extrasystoles/ Ventricular bigeminy	28-FEB-2018/ 09:51/ Ongoing	No/ No	MODERATE/ NONE/ RECOVERING/RESOLVING	Possible	8 days post		No
		2	Gastrointestinal disorders/ Nausea/ Intermittent nausea	28-FEB-2018/ 19:30/ 02-MAR-2018/ 15:00	Yes/ No	MILD/ NONE/ RECOVERED/RESOLVED	Probable	8.4 days post	43.50	No
		3	Cardiac disorders/ Tachycardia/ tachycardia	28-FEB-2018/ 23:21/ 28-FEB-2018/ 23:22	Yes/ No	MODERATE/ NONE/ RECOVERED/RESOLVED	Possible	8.5 days post	0.02	No

Notes: Adverse events were coded using the Medical Dictionary for Regulatory Activities (MedDRA) Version 20.1
A treatment-emergent adverse event is defined as an AE that occurred or worsened following first administration of study drug.
Rando = Randomisation.
AE = Adverse Event; TEAE = A treatment-emergent adverse event; SAE = Serious adverse event; UN = Unknown; Dis. = Discontinuation

Listing 16.2.7.1a
Adverse Events Related to Inoculum Only

Rando Number	Challenge Agent	AE No	System Organ Class/ Preferred Term/ Adverse Event Verbatim	Onset Date/ Onset Time / End Date/ End Time	TEAE/ SAE	Severity/ Action Taken/ Outcome	Relationship to Inoculum	Time of onset Relative to Inoculum	Duration (hours)	Dis.
R209	3D7	4	Nervous system disorders/ Headache/ Intermittent headache	28-FEB-2018/ 23:30/ 01-MAR-2018/ 12:19	Yes/ No	MILD/ MEDICATION: Ibuprofen, paracetamol/ NOT RECOVERED/ NOT RESOLVED	Probable	8.5 days post	12.82	No
		5	Nervous system disorders/ Headache/ headache	01-MAR-2018/ 12:19/ 01-MAR-2018/ 20:06	Yes/ No	MODERATE/ NONE/ RECOVERING/RESOLVING	Probable	9.1 days post	7.78	No
		6	Cardiac disorders/ Tachycardia/ tachycardia	01-MAR-2018/ 12:20/ 01-MAR-2018/ 14:32	Yes/ No	MODERATE/ NONE/ RECOVERED/RESOLVED	Probable	9.1 days post	2.20	No
		7	Nervous system disorders/ Headache/ intermittent headache	01-MAR-2018/ 20:06/ 13-MAR-2018/ 09:00	Yes/ No	MILD/ MEDICATION: ibuprofen/ RECOVERED/RESOLVED	Probable	9.4 days post	276.90	No
		8	Skin and subcutaneous tissue disorders/ Hyperhidrosis/ Sweats	02-MAR-2018/ 04:00/ 02-MAR-2018/ 20:04	Yes/ No	MILD/ NONE/ RECOVERED/RESOLVED	Probable	9.7 days post	16.07	No
		9	Musculoskeletal and connective tissue disorders/ Myalgia/ Intermittent myalgia	02-MAR-2018/ 04:00/ 13-MAR-2018/ 09:00	Yes/ No	MILD/ MEDICATION: Ibuprofen, deep heat metholatum regular relief/ RECOVERED/RESOLVED	Probable	9.7 days post	269.00	No
		10	General disorders and administration site conditions/ Pyrexia/ fever	02-MAR-2018/ 07:21/ 02-MAR-2018/ 14:56	Yes/ No	MILD/ NONE/ RECOVERED/RESOLVED	Probable	9.9 days post	7.58	No

Notes: Adverse events were coded using the Medical Dictionary for Regulatory Activities (MedDRA) Version 20.1
A treatment-emergent adverse event is defined as an AE that occurred or worsened following first administration of study drug.
Rando = Randomisation.
AE = Adverse Event; TEAE = A treatment-emergent adverse event; SAE = Serious adverse event; UN = Unknown; Dis. = Discontinuation

Listing 16.2.7.1a
Adverse Events Related to Inoculum Only

Rando Number	Challenge Agent	AE No	System Organ Class/ Preferred Term/ Adverse Event Verbatim	Onset Date/ Onset Time / End Date/ End Time	TEAE/ SAE	Severity/ Action Taken/ Outcome	Relationship to Inoculum	Time of onset Relative to Inoculum	Duration (hours)	Dis.
R209	3D7	11	Investigations/ Alanine aminotransferase increased/ Alanine transaminase count increased	08-MAR-2018/ 08:42/ 12-MAR-2018/ 08:12	Yes/ No	MODERATE/ NONE/ RECOVERED/RESOLVED	Probable	15.9 days post	95.50	No
R210	3D7	2	Musculoskeletal and connective tissue disorders/ Myalgia/ Intermittent myalgia	28-FEB-2018/ 23:00/ 02-MAR-2018/ 18:00	Yes/ No	MILD/ MEDICATION: Ibuprofen/ RECOVERED/RESOLVED	Probable	8.5 days post	43.00	No
		3	Nervous system disorders/ Headache/ intermittent headache	01-MAR-2018/ 10:00/ 16-MAR-2018/ 08:00	Yes/ No	MILD/ MEDICATION: ibuprofen/ RECOVERED/RESOLVED	Probable	9 days post	358.00	No
		4	Gastrointestinal disorders/ Nausea/ Nausea	01-MAR-2018/ 12:30/ 01-MAR-2018/ 20:30	Yes/ No	MILD/ NONE/ RECOVERED/RESOLVED	Probable	9.1 days post	8.00	No
		5	General disorders and administration site conditions/ Pyrexia/ Fever	01-MAR-2018/ 20:05/ 01-MAR-2018/ 20:07	Yes/ No	MILD/ NONE/ RECOVERED/RESOLVED	Probable	9.4 days post	0.03	No
		6	Gastrointestinal disorders/ Nausea/ nausea	04-MAR-2018/ 11:00/ 04-MAR-2018/ 21:00	Yes/ No	MILD/ MEDICATION: ibuprofen/ RECOVERED/RESOLVED	Probable	12 days post	10.00	No
		7	Respiratory, thoracic and mediastinal disorders/ Oropharyngeal pain/ sore throat	05-MAR-2018/ 11:00/ 07-MAR-2018/ 06:30	Yes/ No	MILD/ MEDICATION: ibuprofen/ RECOVERED/RESOLVED	Probable	13 days post	43.50	No

Notes: Adverse events were coded using the Medical Dictionary for Regulatory Activities (MedDRA) Version 20.1
A treatment-emergent adverse event is defined as an AE that occurred or worsened following first administration of study drug.
Rando = Randomisation.
AE = Adverse Event; TEAE = A treatment-emergent adverse event; SAE = Serious adverse event; UN = Unknown; Dis. = Discontinuation

Listing 16.2.7.1a
Adverse Events Related to Inoculum Only

Rando Number	Challenge Agent	AE No	System Organ Class/ Preferred Term/ Adverse Event Verbatim	Onset Date/ Onset Time / End Date/ End Time	TEAE/ SAE	Severity/ Action Taken/ Outcome	Relationship to Inoculum	Time of onset Relative to Inoculum	Duration (hours)	Dis.
R210	3D7	8	Skin and subcutaneous tissue disorders/ Hyperhidrosis/ intermittent sweats	08-MAR-2018/ 12:00/ 10-MAR-2018/ 06:30	Yes/ No	MILD/ MEDICATION: ibuprofen/ RECOVERED/RESOLVED	Probable	16.1 days post	42.50	No
		9	Musculoskeletal and connective tissue disorders/ Myalgia/ myalgia	08-MAR-2018/ 12:00/ 10-MAR-2018/ 06:30	Yes/ No	MILD/ MEDICATION: ibuprofen/ RECOVERED/RESOLVED	Probable	16.1 days post	42.50	No
		10	General disorders and administration site conditions/ Fatigue/ fatigue	09-MAR-2018/ 14:00/ 12-MAR-2018/ 07:15	Yes/ No	MILD/ NONE/ RECOVERED/RESOLVED	Probable	17.1 days post	65.25	No
R302	K13	1	Nervous system disorders/ Headache/ Headache intermittent	14-SEP-2018/ 07:15/ 16-SEP-2018/ 08:00	Yes/ No	MILD/ MEDICATION: Ibuprofen/ RECOVERED/RESOLVED	Probable	9.9 days post	48.75	No
		2	Musculoskeletal and connective tissue disorders/ Myalgia/ Intermittent myalgia	14-SEP-2018/ 11:00/ 15-SEP-2018/ 12:30	Yes/ No	MILD/ MEDICATION: Ibuprofen/ RECOVERED/RESOLVED	Probable	10 days post	25.50	No
		3	General disorders and administration site conditions/ Pyrexia/ Fever	14-SEP-2018/ 20:55/ 14-SEP-2018/ 21:00	Yes/ No	MILD/ NONE/ RECOVERED/RESOLVED	Probable	10.4 days post	0.08	No
		6	General disorders and administration site conditions/ Pyrexia/ Intermittent Fever	15-SEP-2018/ 12:43/ 15-SEP-2018/ 19:41	Yes/ No	MILD/ NONE/ RECOVERED/RESOLVED	Probable	11.1 days post	6.97	No

Notes: Adverse events were coded using the Medical Dictionary for Regulatory Activities (MedDRA) Version 20.1
A treatment-emergent adverse event is defined as an AE that occurred or worsened following first administration of study drug.
Rando = Randomisation.
AE = Adverse Event; TEAE = A treatment-emergent adverse event; SAE = Serious adverse event; UN = Unknown; Dis. = Discontinuation

Listing 16.2.7.1a
Adverse Events Related to Inoculum Only

Rando Number	Challenge Agent	AE No	System Organ Class/ Preferred Term/ Adverse Event Verbatim	Onset Date/ Onset Time / End Date/ End Time	TEAE/ SAE	Severity/ Action Taken/ Outcome	Relationship to Inoculum	Time of onset Relative to Inoculum	Duration (hours)	Dis.
R302	K13	8	Nervous system disorders/ Headache/ Intermittent headache	16-SEP-2018/ 18:00/ 19-SEP-2018/ 06:00	Yes/ No	MILD/ NONE/ RECOVERED/RESOLVED	Probable	12.3 days post	60.00	No
R303	K13	1	Nervous system disorders/ Headache/ headache	09-SEP-2018/ 11:00/ 09-SEP-2018/ 11:10	No/ No	MILD/ NONE/ RECOVERED/RESOLVED	Probable	5 days post	0.17	No
		2	Infections and infestations/ Upper respiratory tract infection/ Upper respiratory tract infection	09-SEP-2018/ 11:00/ 11-SEP-2018/ 09:00	No/ No	MILD/ NONE/ RECOVERED/RESOLVED	Probable	5 days post	46.00	No
		3	General disorders and administration site conditions/ Malaise/ Intermittent Malaise	09-SEP-2018/ 19:01/ 12-SEP-2018/ 08:10	No/ No	MILD/ NONE/ RECOVERED/RESOLVED	Probable	5.4 days post	61.15	No
		5	Gastrointestinal disorders/ Nausea/ Nausea	11-SEP-2018/ 14:00/ 11-SEP-2018/ 19:00	No/ No	MILD/ NONE/ RECOVERED/RESOLVED	Probable	7.1 days post	5.00	No
		7	Cardiac disorders/ Ventricular extrasystoles/ Frequent ventricular ectopy	13-SEP-2018/ 08:52/ Ongoing	No/ No	MODERATE/ NONE/ RECOVERING/RESOLVING	Possible	8.9 days post		No
		8	Gastrointestinal disorders/ Nausea/ Intermittent Nausea	13-SEP-2018/ 17:00/ 16-SEP-2018/ 09:00	Yes/ No	MILD/ MEDICATION: Ondansteron/ RECOVERED/RESOLVED	Probable	9.3 days post	64.00	No

Notes: Adverse events were coded using the Medical Dictionary for Regulatory Activities (MedDRA) Version 20.1
A treatment-emergent adverse event is defined as an AE that occurred or worsened following first administration of study drug.
Rando = Randomisation.
AE = Adverse Event; TEAE = A treatment-emergent adverse event; SAE = Serious adverse event; UN = Unknown; Dis. = Discontinuation

Listing 16.2.7.1a
Adverse Events Related to Inoculum Only

Rando Number	Challenge Agent	AE No	System Organ Class/ Preferred Term/ Adverse Event Verbatim	Onset Date/ Onset Time / End Date/ End Time	TEAE/ SAE	Severity/ Action Taken/ Outcome	Relationship to Inoculum	Time of onset Relative to Inoculum	Duration (hours)	Dis.
R303	K13	9	General disorders and administration site conditions/ Pyrexia/ Intermittent fever	13-SEP-2018/ 17:04/ 14-SEP-2018/ 17:28	Yes/ No	MILD/ MEDICATION: Ibuprofen/ NOT RECOVERED/ NOT RESOLVED	Probable	9.3 days post	24.40	No
		10	Nervous system disorders/ Headache/ Intermittent headache	14-SEP-2018/ 01:30/ 15-SEP-2018/ 07:00	Yes/ No	MILD/ MEDICATION: Ibuprofen/ RECOVERED/RESOLVED	Probable	9.6 days post	29.50	No
		11	General disorders and administration site conditions/ Chills/ Intermittent rigors	14-SEP-2018/ 09:55/ 16-SEP-2018/ 20:18	Yes/ No	MILD/ MEDICATION: Ibuprofen/ RECOVERED/RESOLVED	Probable	10 days post	58.38	No
		12	General disorders and administration site conditions/ Pyrexia/ Fever	14-SEP-2018/ 17:28/ 14-SEP-2018/ 21:18	Yes/ No	MODERATE/ MEDICATION: Ibuprofen/ RECOVERED/RESOLVED	Probable	10.3 days post	3.83	No
		13	Skin and subcutaneous tissue disorders/ Hyperhidrosis/ Sweating	14-SEP-2018/ 21:16/ 15-SEP-2018/ 07:25	Yes/ No	MILD/ NONE/ RECOVERED/RESOLVED	Probable	10.4 days post	10.15	No
		14	Investigations/ White blood cell count decreased/ decreased white cell count	15-SEP-2018/ 11:18/ 18-SEP-2018/ 07:29	Yes/ No	MODERATE/ NONE/ RECOVERED/RESOLVED	Probable	11 days post	68.18	No
		15	Investigations/ Neutrophil count decreased/ Neutrophil count decreased	15-SEP-2018/ 11:18/ 22-SEP-2018/ 08:40	Yes/ No	MODERATE/ NONE/ RECOVERED/RESOLVED	Probable	11 days post	165.37	No

Notes: Adverse events were coded using the Medical Dictionary for Regulatory Activities (MedDRA) Version 20.1
A treatment-emergent adverse event is defined as an AE that occurred or worsened following first administration of study drug.
Rando = Randomisation.
AE = Adverse Event; TEAE = A treatment-emergent adverse event; SAE = Serious adverse event; UN = Unknown; Dis. = Discontinuation

Listing 16.2.7.1a
Adverse Events Related to Inoculum Only

Rando Number	Challenge Agent	AE No	System Organ Class/ Preferred Term/ Adverse Event Verbatim	Onset Date/ Onset Time / End Date/ End Time	TEAE/ SAE	Severity/ Action Taken/ Outcome	Relationship to Inoculum	Time of onset Relative to Inoculum	Duration (hours)	Dis.
R303	K13	17	Nervous system disorders/ Headache/ Headache	17-SEP-2018/ 20:00/ 18-SEP-2018/ 05:30	Yes/ No	MILD/ MEDICATION: Ibuprofen/ RECOVERED/RESOLVED	Probable	13.4 days post	9.50	No
		18	Gastrointestinal disorders/ Nausea/ Intermittent Nausea	18-SEP-2018/ 06:10/ 18-SEP-2018/ 13:00	Yes/ No	MILD/ NONE/ RECOVERED/RESOLVED	Probable	13.8 days post	6.83	No
R304	K13	1	Musculoskeletal and connective tissue disorders/ Myalgia/ Intermittent myalgia	10-SEP-2018/ 14:30/ 13-SEP-2018/ 12:00	No/ No	MILD/ NONE/ RECOVERED/RESOLVED	Probable	6.2 days post	69.50	No
		2	General disorders and administration site conditions/ Malaise/ Intermittent Malaise	10-SEP-2018/ 14:30/ 15-SEP-2018/ 07:28	No/ No	MILD/ NONE/ RECOVERED/RESOLVED	Probable	6.2 days post	112.97	No
		3	Nervous system disorders/ Headache/ intermittent headache	13-SEP-2018/ 15:00/ 16-SEP-2018/ 07:00	Yes/ No	MILD/ MEDICATION: Ibuprofen/ RECOVERED/RESOLVED	Probable	9.2 days post	64.00	No
		4	Respiratory, thoracic and mediastinal disorders/ Oropharyngeal pain/ Sore throat	14-SEP-2018/ 15:30/ 16-SEP-2018/ 07:00	Yes/ No	MILD/ NONE/ RECOVERED/RESOLVED	Probable	10.2 days post	39.50	No
		5	General disorders and administration site conditions/ Pyrexia/ Fever	14-SEP-2018/ 16:35/ 14-SEP-2018/ 21:44	Yes/ No	MILD/ NONE/ RECOVERED/RESOLVED	Probable	10.2 days post	5.15	No

Notes: Adverse events were coded using the Medical Dictionary for Regulatory Activities (MedDRA) Version 20.1
A treatment-emergent adverse event is defined as an AE that occurred or worsened following first administration of study drug.
Rando = Randomisation.
AE = Adverse Event; TEAE = A treatment-emergent adverse event; SAE = Serious adverse event; UN = Unknown; Dis. = Discontinuation

Listing 16.2.7.1a
Adverse Events Related to Inoculum Only

Rando Number	Challenge Agent	AE No	System Organ Class/ Preferred Term/ Adverse Event Verbatim	Onset Date/ Onset Time / End Date/ End Time	TEAE/ SAE	Severity/ Action Taken/ Outcome	Relationship to Inoculum	Time of onset Relative to Inoculum	Duration (hours)	Dis.
R304	K13	7	Metabolism and nutrition disorders/ Hypophosphataemia/ Hypophosphataemia	15-SEP-2018/ 09:22/ 16-SEP-2018/ 09:22	Yes/ No	MODERATE/ NONE/ RECOVERED/RESOLVED	Probable	10.9 days post	24.00	No
		8	Nervous system disorders/ Headache/ Intermittent headache	16-SEP-2018/ 09:02/ 19-SEP-2018/ 19:00	Yes/ No	MILD/ MEDICATION: Ibuprofen/ RECOVERED/RESOLVED	Probable	11.9 days post	81.97	No
		9	Musculoskeletal and connective tissue disorders/ Myalgia/ Myalgia	16-SEP-2018/ 22:00/ 17-SEP-2018/ 08:35	Yes/ No	MILD/ MEDICATION: Ibuprofen/ RECOVERED/RESOLVED	Probable	12.5 days post	10.58	No
		10	Musculoskeletal and connective tissue disorders/ Myalgia/ Myalgia	23-SEP-2018/ UN:UN/ 28-SEP-2018/ 15:00	Yes/ No	MODERATE/ RECOVERED/RESOLVED	Probable	18.6 days post		No
R305	3D7	1	Nervous system disorders/ Headache/ Headache	10-SEP-2018/ 13:52/ 10-SEP-2018/ 17:00	No/ No	MILD/ MEDICATION: Ibuprofen/ RECOVERED/RESOLVED	Probable	5.1 days post	3.13	No
		2	Nervous system disorders/ Headache/ Intermittent headache	13-SEP-2018/ 12:00/ 15-SEP-2018/ 14:00	Yes/ No	MILD/ MEDICATION: Ibuprofen/ RECOVERED/RESOLVED	Probable	8 days post	50.00	No
		3	Musculoskeletal and connective tissue disorders/ Myalgia/ Intermittent myalgia	14-SEP-2018/ 08:18/ 14-SEP-2018/ 16:00	Yes/ No	MODERATE/ NONE/ RECOVERING/RESOLVING	Probable	8.9 days post	7.70	No

Notes: Adverse events were coded using the Medical Dictionary for Regulatory Activities (MedDRA) Version 20.1
A treatment-emergent adverse event is defined as an AE that occurred or worsened following first administration of study drug.
Rando = Randomisation.
AE = Adverse Event; TEAE = A treatment-emergent adverse event; SAE = Serious adverse event; UN = Unknown; Dis. = Discontinuation

Listing 16.2.7.1a
Adverse Events Related to Inoculum Only

Rando Number	Challenge Agent	AE No	System Organ Class/ Preferred Term/ Adverse Event Verbatim	Onset Date/ Onset Time / End Date/ End Time	TEAE/ SAE	Severity/ Action Taken/ Outcome	Relationship to Inoculum	Time of onset Relative to Inoculum	Duration (hours)	Dis.
R305	3D7	4	Musculoskeletal and connective tissue disorders/ Myalgia/ Myalgia	14-SEP-2018/ 16:00/ 15-SEP-2018/ 14:00	Yes/ No	MILD/ MEDICATION: Ibuprofen/ RECOVERED/RESOLVED	Probable	9.2 days post	22.00	No
		5	Investigations/ Neutrophil count decreased/ Neutrophil count decreased	16-SEP-2018/ 09:36/ 18-SEP-2018/ 07:16	Yes/ No	SEVERE/ NONE/ RECOVERING/RESOLVING	Probable	10.9 days post	45.67	No
		6	Investigations/ White blood cell count decreased/ White cell count decreased	16-SEP-2018/ 09:36/ 18-SEP-2018/ 07:16	Yes/ No	MODERATE/ NONE/ RECOVERED/RESOLVED	Probable	10.9 days post	45.67	No
		7	General disorders and administration site conditions/ Malaise/ Malaise	16-SEP-2018/ 11:00/ 16-SEP-2018/ 14:00	Yes/ No	MILD/ MEDICATION: Ibuprofen/ RECOVERED/RESOLVED	Probable	11 days post	3.00	No
		8	Nervous system disorders/ Headache/ Headache	17-SEP-2018/ 06:30/ 17-SEP-2018/ 09:00	Yes/ No	MILD/ MEDICATION: Ibuprofen/ RECOVERED/RESOLVED	Probable	11.8 days post	2.50	No
		9	Investigations/ Neutrophil count decreased/ Decreased neutrophil count	18-SEP-2018/ 07:16/ 19-SEP-2018/ 19:23	Yes/ No	MODERATE/ NONE/ RECOVERED/RESOLVED	Probable	12.8 days post	36.12	No
		10	Musculoskeletal and connective tissue disorders/ Arthralgia/ Arthralgia	19-SEP-2018/ 21:00/ 20-SEP-2018/ 19:00	Yes/ No	MILD/ MEDICATION: Paracetamol/ RECOVERED/RESOLVED	Probable	14.4 days post	22.00	No

Notes: Adverse events were coded using the Medical Dictionary for Regulatory Activities (MedDRA) Version 20.1
A treatment-emergent adverse event is defined as an AE that occurred or worsened following first administration of study drug.
Rando = Randomisation.
AE = Adverse Event; TEAE = A treatment-emergent adverse event; SAE = Serious adverse event; UN = Unknown; Dis. = Discontinuation

Listing 16.2.7.1a
Adverse Events Related to Inoculum Only

Rando Number	Challenge Agent	AE No	System Organ Class/ Preferred Term/ Adverse Event Verbatim	Onset Date/ Onset Time / End Date/ End Time	TEAE/ SAE	Severity/ Action Taken/ Outcome	Relationship to Inoculum	Time of onset Relative to Inoculum	Duration (hours)	Dis.
R305	3D7	11	Nervous system disorders/ Lethargy/ Lethargy	19-SEP-2018/ 21:00/ 20-SEP-2018/ 19:00	Yes/ No	MILD/ NONE/ RECOVERED/RESOLVED	Probable	14.4 days post	22.00	No
		12	General disorders and administration site conditions/ Malaise/ Malaise	19-SEP-2018/ 21:00/ 20-SEP-2018/ 19:00	Yes/ No	MILD/ MEDICATION: Paracetamol/ RECOVERED/RESOLVED	Probable	14.4 days post	22.00	No
		13	Gastrointestinal disorders/ Nausea/ Nausea	19-SEP-2018/ 21:00/ 20-SEP-2018/ 19:00	Yes/ No	MILD/ MEDICATION: Ondansentron/ RECOVERED/RESOLVED	Probable	14.4 days post	22.00	No
		14	Investigations/ Neutrophil count decreased/ Neutrophil count decreased	22-SEP-2018/ 07:53/ 25-SEP-2018/ 08:19	Yes/ No	MODERATE/ NONE/ RECOVERED/RESOLVED	Probable	16.9 days post	72.43	No
		15	Nervous system disorders/ Headache/ Intermittent headache	23-SEP-2018/ UN:UN/ 23-SEP-2018/ 18:00	Yes/ No	MILD/ NONE/ RECOVERED/RESOLVED	Probable	17.5 days post		No
		17	Investigations/ Neutrophil count decreased/ Neutrophil count decreased	28-SEP-2018/ 07:23/ 02-OCT-2018/ 07:50	Yes/ No	MODERATE/ NONE/ RECOVERED/RESOLVED	Probable	22.8 days post	96.45	No
R306	3D7	2	Nervous system disorders/ Headache/ Headache	14-SEP-2018/ 12:50/ 14-SEP-2018/ 21:37	Yes/ No	MILD/ MEDICATION: Ibuprofen/ RECOVERED/RESOLVED	Probable	9.1 days post	8.78	No

Notes: Adverse events were coded using the Medical Dictionary for Regulatory Activities (MedDRA) Version 20.1
A treatment-emergent adverse event is defined as an AE that occurred or worsened following first administration of study drug.
Rando = Randomisation.
AE = Adverse Event; TEAE = A treatment-emergent adverse event; SAE = Serious adverse event; UN = Unknown; Dis. = Discontinuation

Listing 16.2.7.1a
Adverse Events Related to Inoculum Only

Rando Number	Challenge Agent	AE No	System Organ Class/ Preferred Term/ Adverse Event Verbatim	Onset Date/ Onset Time / End Date/ End Time	TEAE/ SAE	Severity/ Action Taken/ Outcome	Relationship to Inoculum	Time of onset Relative to Inoculum	Duration (hours)	Dis.
R306	3D7	3	Cardiac disorders/ Tachycardia/ Tachycardia	20-SEP-2018/ 06:55/ 20-SEP-2018/ 06:58	Yes/ No	MILD/ NONE/ RECOVERED/RESOLVED	Probable	14.8 days post	0.05	No
		4	Musculoskeletal and connective tissue disorders/ Myalgia/ Myalgia lower back	20-SEP-2018/ 13:00/ 20-SEP-2018/ 20:30	Yes/ No	MILD/ MEDICATION: Ibuprofen/ RECOVERED/RESOLVED	Probable	15.1 days post	7.50	No
		5	General disorders and administration site conditions/ Chills/ Rigors	20-SEP-2018/ 14:30/ 20-SEP-2018/ 20:30	Yes/ No	MILD/ NONE/ RECOVERED/RESOLVED	Probable	15.1 days post	6.00	No
		6	General disorders and administration site conditions/ Pyrexia/ Fever	20-SEP-2018/ 17:00/ 20-SEP-2018/ 20:30	Yes/ No	MODERATE/ MEDICATION: Ibuprofen/ RECOVERED/RESOLVED	Probable	15.2 days post	3.50	No
R307	3D7	1	Musculoskeletal and connective tissue disorders/ Myalgia/ Myalgia	13-SEP-2018/ 13:30/ 15-SEP-2018/ 08:00	Yes/ No	MILD/ MEDICATION: Ibuprofen/ RECOVERED/RESOLVED	Probable	8.1 days post	42.50	No
		2	Nervous system disorders/ Headache/ Intermittent Headache	13-SEP-2018/ 16:00/ 15-SEP-2018/ 08:00	Yes/ No	MILD/ MEDICATION: Ibuprofen/ RECOVERED/RESOLVED	Probable	8.2 days post	40.00	No
		3	General disorders and administration site conditions/ Pyrexia/ Fever	13-SEP-2018/ 19:57/ 13-SEP-2018/ 21:51	Yes/ No	MODERATE/ MEDICATION: Ibuprofen/ RECOVERING/RESOLVING	Probable	8.4 days post	1.90	No

Notes: Adverse events were coded using the Medical Dictionary for Regulatory Activities (MedDRA) Version 20.1
A treatment-emergent adverse event is defined as an AE that occurred or worsened following first administration of study drug.
Rando = Randomisation.
AE = Adverse Event; TEAE = A treatment-emergent adverse event; SAE = Serious adverse event; UN = Unknown; Dis. = Discontinuation

Listing 16.2.7.1a
Adverse Events Related to Inoculum Only

Rando Number	Challenge Agent	AE No	System Organ Class/ Preferred Term/ Adverse Event Verbatim	Onset Date/ Onset Time / End Date/ End Time	TEAE/ SAE	Severity/ Action Taken/ Outcome	Relationship to Inoculum	Time of onset Relative to Inoculum	Duration (hours)	Dis.
R307	3D7	4	General disorders and administration site conditions/ Pyrexia/ Fever	13-SEP-2018/ 21:51/ 14-SEP-2018/ 01:51	Yes/ No	MILD/ MEDICATION: Ibuprofen/ RECOVERED/RESOLVED	Probable	8.4 days post	4.00	No
		5	Gastrointestinal disorders/ Nausea/ Nausea	14-SEP-2018/ 04:30/ 16-SEP-2018/ 08:00	Yes/ No	MILD/ NONE/ RECOVERED/RESOLVED	Probable	8.7 days post	51.50	No
		6	General disorders and administration site conditions/ Chills/ Shivering	14-SEP-2018/ 04:30/ 16-SEP-2018/ 08:00	Yes/ No	MILD/ NONE/ RECOVERED/RESOLVED	Probable	8.7 days post	51.50	No
		7	General disorders and administration site conditions/ Pyrexia/ Fever	14-SEP-2018/ 05:49/ 14-SEP-2018/ 08:36	Yes/ No	MODERATE/ MEDICATION: Ibuprofen/ RECOVERED/RESOLVED	Probable	8.8 days post	2.78	No
		8	General disorders and administration site conditions/ Pyrexia/ Intermittent fever	14-SEP-2018/ 13:14/ 16-SEP-2018/ 09:21	Yes/ No	MILD/ MEDICATION: Paracetamol/ RECOVERED/RESOLVED	Probable	9.1 days post	44.12	No
		9	Musculoskeletal and connective tissue disorders/ Myalgia/ Myalgia	15-SEP-2018/ 18:30/ 16-SEP-2018/ 08:00	Yes/ No	MILD/ NONE/ RECOVERED/RESOLVED	Probable	10.3 days post	13.50	No
		10	Investigations/ Neutrophil count decreased/ Neutrophil count decreased	16-SEP-2018/ 09:55/ 18-SEP-2018/ 08:08	Yes/ No	MODERATE/ NONE/ RECOVERED/RESOLVED	Probable	10.9 days post	46.22	No

Notes: Adverse events were coded using the Medical Dictionary for Regulatory Activities (MedDRA) Version 20.1
A treatment-emergent adverse event is defined as an AE that occurred or worsened following first administration of study drug.
Rando = Randomisation.
AE = Adverse Event; TEAE = A treatment-emergent adverse event; SAE = Serious adverse event; UN = Unknown; Dis. = Discontinuation

Listing 16.2.7.1a
Adverse Events Related to Inoculum Only

Rando Number	Challenge Agent	AE No	System Organ Class/ Preferred Term/ Adverse Event Verbatim	Onset Date/ Onset Time / End Date/ End Time	TEAE/ SAE	Severity/ Action Taken/ Outcome	Relationship to Inoculum	Time of onset Relative to Inoculum	Duration (hours)	Dis.
R307	3D7	11	Nervous system disorders/ Headache/ Intermittent headache	16-SEP-2018/ 12:00/ 17-SEP-2018/ 06:04	Yes/ No	MILD/ NONE/ RECOVERED/RESOLVED	Probable	11 days post	18.07	No
		12	Investigations/ Aspartate aminotransferase increased/ Aspartate aminotransferase increased	18-SEP-2018/ 08:08/ 22-SEP-2018/ 08:38	Yes/ No	SEVERE/ NONE/ RECOVERING/RESOLVING	Probable	12.9 days post	96.50	No
		13	Investigations/ Alanine aminotransferase increased/ Alanine aminotransferase increased	18-SEP-2018/ 08:08/ 25-SEP-2018/ 07:36	Yes/ No	SEVERE/ NONE/ RECOVERING/RESOLVING	Probable	12.9 days post	167.47	No
		15	Nervous system disorders/ Headache/ Headache	20-SEP-2018/ 14:30/ 20-SEP-2018/ 21:00	Yes/ No	MILD/ MEDICATION: Ibuprofen/ RECOVERED/RESOLVED	Probable	15.1 days post	6.50	No
		16	Musculoskeletal and connective tissue disorders/ Myalgia/ Muscle ache	20-SEP-2018/ 14:30/ 20-SEP-2018/ 21:00	Yes/ No	MILD/ MEDICATION: Ibuprofen/ RECOVERED/RESOLVED	Probable	15.1 days post	6.50	No
		17	General disorders and administration site conditions/ Pyrexia/ Fever	20-SEP-2018/ 17:30/ 20-SEP-2018/ 21:00	Yes/ No	MILD/ MEDICATION: Ibuprofen/ RECOVERED/RESOLVED	Probable	15.3 days post	3.50	No
		18	Investigations/ Aspartate aminotransferase increased/ Aspartate transaminase increased	22-SEP-2018/ 08:38/ 25-SEP-2018/ 07:36	Yes/ No	MODERATE/ NONE/ RECOVERED/RESOLVED	Probable	16.9 days post	70.97	No

Notes: Adverse events were coded using the Medical Dictionary for Regulatory Activities (MedDRA) Version 20.1
A treatment-emergent adverse event is defined as an AE that occurred or worsened following first administration of study drug.
Rando = Randomisation.
AE = Adverse Event; TEAE = A treatment-emergent adverse event; SAE = Serious adverse event; UN = Unknown; Dis. = Discontinuation

Listing 16.2.7.1a
Adverse Events Related to Inoculum Only

Rando Number	Challenge Agent	AE No	System Organ Class/ Preferred Term/ Adverse Event Verbatim	Onset Date/ Onset Time / End Date/ End Time	TEAE/ SAE	Severity/ Action Taken/ Outcome	Relationship to Inoculum	Time of onset Relative to Inoculum	Duration (hours)	Dis.
R307	3D7	19	Investigations/ Alanine aminotransferase increased/ Alanine aminotransferase increased	25-SEP-2018/ 07:36/ 28-SEP-2018/ 07:40	Yes/ No	MODERATE/ NONE/ RECOVERED/RESOLVED	Probable	19.8 days post	72.07	No

Notes: Adverse events were coded using the Medical Dictionary for Regulatory Activities (MedDRA) Version 20.1
A treatment-emergent adverse event is defined as an AE that occurred or worsened following first administration of study drug.
Rando = Randomisation.
AE = Adverse Event; TEAE = A treatment-emergent adverse event; SAE = Serious adverse event; UN = Unknown; Dis. = Discontinuation

16.2.7.1b Treatment Emergent Adverse Events Related to Artesunate Only

Listing 16.2.7.1b
Treatment-Emergent Adverse Events Related to Artesunate Only

Rando Number	Challenge Agent	AE No	System Organ Class/ Preferred Term/ Adverse Event Verbatim	Onset Date/ Onset Time / End Date/ End Time	TEAE/ SAE	Severity/ Action Taken/ Outcome	Relationship to Artesunate	Time of onset Relative to Artesunate	Duration (hours)	Dis.

No Treatment-Emergent Adverse Events Related to Artesunate Only Reported

16.2.7.1c Treatment Emergent Adverse Events Related to Piperaquine Only

Listing 16.2.7.1c
Treatment-Emergent Adverse Events Related to Piperaquine

Rando Number	Challenge Agent	AE No	System Organ Class/ Preferred Term/ Adverse Event Verbatim	Onset Date/ Onset Time / End Date/ End Time	TEAE/ SAE	Severity/ Action Taken/ Outcome	Relationship to Piperaquine	Time of onset Relative to Piperaquine	Duration (hours)	Dis.
R108	3D7	26	Investigations/ Eosinophil count increased/ eosinophil count increased	14-DEC-2017/ 08:37/ 21-DEC-2017/ 07:53	Yes/ No	MODERATE/ NONE/ RECOVERED/RESOLVED	Probable	13 days post	167.27	No

Notes: Adverse events were coded using the Medical Dictionary for Regulatory Activities (MedDRA) Version 20.1
A treatment-emergent adverse event is defined as an AE that occurred or worsened following first administration of study drug.
Rando = Randomisation.
AE = Adverse Event; TEAE = A treatment-emergent adverse event; SAE = Serious adverse event; UN = Unknown; Dis. = Discontinuation

Printed: 24FEB2020 (Final)

16.2.7.2 Serious Adverse Events

Listing 16.2.7.2
Serious Adverse Events

Rando Number	Challenge Agent	AE No	System Organ Class/ Preferred Term/ Adverse Event Verbatim	Onset Date/ Onset Time/ End Date/ End Time	Death	Life Threatening	Initial or Prolonged Hospitalization	Persistent or Significant Disability or Incapacity	Congenital Anomaly or Birth Defect	Occurred with Overdose	AE involves Cancer	Other Medically Important Event

No Serious Adverse Event Reported

16.2.7.3 Adverse Events Leading to Discontinuation or Withdrawal

Listing 16.2.7.3

Adverse Events leading to discontinuation or withdrawal

Rando Number	Challenge Agent	AE No	System Organ Class/ Preferred Term/ Adverse Event Verbatim	Onset Date/ Onset Time/ End Date/ End Time	Duration (Hours)	TEAE/ SAE	Severity	Relationship to Artesunate	Action Taken with Study Drug/ Other	Disc.	Outcome

No Adverse Events Leading to Discontinuation or Withdrawal

16.2.7.4 Deaths

Listing 16.2.7.4
Deaths

Randomisation Number	Challenge Agent	Day relative to Inoculum	Day relative to Artesunate	Date of Death	Primary Cause of Death	Autopsy Performed

No Deaths Reported

16.2.8 Clinical laboratory measurements

16.2.8.1a Individual Haematology Results

Listing 16.2.8.1a
Individual Haematology Results

Rando Number	Challenge Agent	Parameter (unit) [Normal Range]	Visit	Date of Assessment	Time of Assessment	Day Relative to Inoculum	Day Relative to IP	Result	Range Indicator	Clinical Assessment
R103	K13	Basophils (10^9/L) [0 – 0.15]	Screening	01-NOV-2017	12:16	-13	-22	0.06		
			Safety Visit	13-NOV-2017	08:44	-1	-10	0.04		
			Admission	23-NOV-2017	09:50	9	1	0.04		
			Safety Monitoring	25-NOV-2017	17:52	11	3	0.03		
			Admission 3	26-NOV-2017	10:53	12	4	0.02		
			Safety Monitoring	30-NOV-2017	10:09	16	8	0.04		
			Safety Monitoring	03-DEC-2017	10:45	19	11	0.05		
			Safety Monitoring	04-DEC-2017	09:27	20	12	0.06		
			Safety Monitoring	07-DEC-2017	08:41	23	15	0.08		
			End of Study	14-DEC-2017	09:37	30	22	0.07		

Notes: Day relative to inoculum is the number of days relative to day of administration of the inoculum, where the day of administration = 0.
The inoculum was administered on Study Day 0 for K13-infected subjects and Study Day 1 for 3D7-infected subjects.
Day relative to IP is the number of days relative to day of administration of Artesunate, where the day of dose = 1.
Rando = Randomisation; IP = Artesunate; H = High; L = Low; NCS = Not Clinically Significant; CS = Clinically Significant.

Listing 16.2.8.1a
Individual Haematology Results

Rando Number	Challenge Agent	Parameter (unit) [Normal Range]	Visit	Date of Assessment	Time of Assessment	Day Relative to Inoculum	Day Relative to IP	Result	Range Indicator	Clinical Assessment
R103	K13	Eosinophils (10^9/L) [0 – 0.6]	Screening	01-NOV-2017	12:16	-13	-22	0.27		
			Safety Visit	13-NOV-2017	08:44	-1	-10	0.21		
			Admission	23-NOV-2017	09:50	9	1	0.14		
			Safety Monitoring	25-NOV-2017	17:52	11	3	0.08		
			Admission 3	26-NOV-2017	10:53	12	4	0.08		
			Safety Monitoring	30-NOV-2017	10:09	16	8	0.16		
			Safety Monitoring	03-DEC-2017	10:45	19	11	0.15		
			Safety Monitoring	04-DEC-2017	09:27	20	12	0.19		
			Safety Monitoring	07-DEC-2017	08:41	23	15	0.30		
			End of Study	14-DEC-2017	09:37	30	22	0.23		

Notes: Day relative to inoculum is the number of days relative to day of administration of the inoculum, where the day of administration = 0.
The inoculum was administered on Study Day 0 for K13-infected subjects and Study Day 1 for 3D7-infected subjects.
Day relative to IP is the number of days relative to day of administration of Artesunate, where the day of dose = 1.
Rando = Randomisation; IP = Artesunate; H = High; L = Low; NCS = Not Clinically Significant; CS = Clinically Significant.

Listing 16.2.8.1a
Individual Haematology Results

Rando Number	Challenge Agent	Parameter (unit) [Normal Range]	Visit	Date of Assessment	Time of Assessment	Day Relative to Inoculum	Day Relative to IP	Result	Range Indicator	Clinical Assessment
R103	K13	Ery. Mean Corpuscular Volume (fL) [80 – 100]	Screening	01-NOV-2017	12:16	-13	-22	85		
			Safety Visit	13-NOV-2017	08:44	-1	-10	87		
			Admission	23-NOV-2017	09:50	9	1	83		
			Safety Monitoring	25-NOV-2017	17:52	11	3	85		
			Admission 3	26-NOV-2017	10:53	12	4	84		
			Safety Monitoring	30-NOV-2017	10:09	16	8	85		
			Safety Monitoring	03-DEC-2017	10:45	19	11	85		
			Safety Monitoring	04-DEC-2017	09:27	20	12	83		
			Safety Monitoring	07-DEC-2017	08:41	23	15	85		
			End of Study	14-DEC-2017	09:37	30	22	84		

Notes: Day relative to inoculum is the number of days relative to day of administration of the inoculum, where the day of administration = 0.
The inoculum was administered on Study Day 0 for K13-infected subjects and Study Day 1 for 3D7-infected subjects.
Day relative to IP is the number of days relative to day of administration of Artesunate, where the day of dose = 1.
Rando = Randomisation; IP = Artesunate; H = High; L = Low; NCS = Not Clinically Significant; CS = Clinically Significant.

Listing 16.2.8.1a
Individual Haematology Results

Rando Number	Challenge Agent	Parameter (unit) [Normal Range]	Visit	Date of Assessment	Time of Assessment	Day Relative to Inoculum	Day Relative to IP	Result	Range Indicator	Clinical Assessment
R103	K13	Erythrocytes (10^12/L) [4.5 – 6.5]	Screening	01-NOV-2017	12:16	-13	-22	5.4		
			Safety Visit	13-NOV-2017	08:44	-1	-10	5.1		
			Admission	23-NOV-2017	09:50	9	1	5.3		
			Safety Monitoring	25-NOV-2017	17:52	11	3	5.2		
			Admission 3	26-NOV-2017	10:53	12	4	5.1		
			Safety Monitoring	30-NOV-2017	10:09	16	8	4.6		
			Safety Monitoring	03-DEC-2017	10:45	19	11	5.1		
			Safety Monitoring	04-DEC-2017	09:27	20	12	4.7		
			Safety Monitoring	07-DEC-2017	08:41	23	15	4.9		
			End of Study	14-DEC-2017	09:37	30	22	4.7		

Notes: Day relative to inoculum is the number of days relative to day of administration of the inoculum, where the day of administration = 0.
The inoculum was administered on Study Day 0 for K13-infected subjects and Study Day 1 for 3D7-infected subjects.
Day relative to IP is the number of days relative to day of administration of Artesunate, where the day of dose = 1.
Rando = Randomisation; IP = Artesunate; H = High; L = Low; NCS = Not Clinically Significant; CS = Clinically Significant.

Listing 16.2.8.1a
Individual Haematology Results

Rando Number	Challenge Agent	Parameter (unit) [Normal Range]	Visit	Date of Assessment	Time of Assessment	Day Relative to Inoculum	Day Relative to IP	Result	Range Indicator	Clinical Assessment
R103	K13	Hematocrit [0.4 – 0.54]	Screening	01-NOV-2017	12:16	-13	-22	0.45		
			Safety Visit	13-NOV-2017	08:44	-1	-10	0.45		
			Admission	23-NOV-2017	09:50	9	1	0.44		
			Safety Monitoring	25-NOV-2017	17:52	11	3	0.44		
			Admission 3	26-NOV-2017	10:53	12	4	0.43		
			Safety Monitoring	30-NOV-2017	10:09	16	8	0.39	L	NCS
			Safety Monitoring	03-DEC-2017	10:45	19	11	0.44		
			Safety Monitoring	04-DEC-2017	09:27	20	12	0.39	L	NCS
			Safety Monitoring	07-DEC-2017	08:41	23	15	0.41		
			End of Study	14-DEC-2017	09:37	30	22	0.39	L	NCS

Notes: Day relative to inoculum is the number of days relative to day of administration of the inoculum, where the day of administration = 0.
The inoculum was administered on Study Day 0 for K13-infected subjects and Study Day 1 for 3D7-infected subjects.
Day relative to IP is the number of days relative to day of administration of Artesunate, where the day of dose = 1.
Rando = Randomisation; IP = Artesunate; H = High; L = Low; NCS = Not Clinically Significant; CS = Clinically Significant.

Listing 16.2.8.1a
Individual Haematology Results

Rando Number	Challenge Agent	Parameter (unit) [Normal Range]	Visit	Date of Assessment	Time of Assessment	Day Relative to Inoculum	Day Relative to IP	Result	Range Indicator	Clinical Assessment
R103	K13	Hemoglobin (g/L) [135 – 175]	Screening	01-NOV-2017	12:16	-13	-22	150		
			Safety Visit	13-NOV-2017	08:44	-1	-10	146		
			Admission	23-NOV-2017	09:50	9	1	152		
			Safety Monitoring	25-NOV-2017	17:52	11	3	151		
			Admission 3	26-NOV-2017	10:53	12	4	146		
			Safety Monitoring	30-NOV-2017	10:09	16	8	127	L	NCS
			Safety Monitoring	03-DEC-2017	10:45	19	11	144		
			Safety Monitoring	04-DEC-2017	09:27	20	12	129	L	NCS
			Safety Monitoring	07-DEC-2017	08:41	23	15	137		
			End of Study	14-DEC-2017	09:37	30	22	133	L	NCS

Notes: Day relative to inoculum is the number of days relative to day of administration of the inoculum, where the day of administration = 0.
The inoculum was administered on Study Day 0 for K13-infected subjects and Study Day 1 for 3D7-infected subjects.
Day relative to IP is the number of days relative to day of administration of Artesunate, where the day of dose = 1.
Rando = Randomisation; IP = Artesunate; H = High; L = Low; NCS = Not Clinically Significant; CS = Clinically Significant.

Listing 16.2.8.1a
Individual Haematology Results

Rando Number	Challenge Agent	Parameter (unit) [Normal Range]	Visit	Date of Assessment	Time of Assessment	Day Relative to Inoculum	Day Relative to IP	Result	Range Indicator	Clinical Assessment
R103	K13	Leukocytes (10^9/L) [3.5 – 10]	Screening	01-NOV-2017	12:16	-13	-22	6.9		
			Safety Visit	13-NOV-2017	08:44	-1	-10	5.2		
			Admission	23-NOV-2017	09:50	9	1	4.8		
			Safety Monitoring	25-NOV-2017	17:52	11	3	3.8		
			Admission 3	26-NOV-2017	10:53	12	4	3.4	L	NCS
			Safety Monitoring	30-NOV-2017	10:09	16	8	4.4		
			Safety Monitoring	03-DEC-2017	10:45	19	11	5.4		
			Safety Monitoring	04-DEC-2017	09:27	20	12	5.2		
			Safety Monitoring	07-DEC-2017	08:41	23	15	6.4		
			End of Study	14-DEC-2017	09:37	30	22	7.3		

Notes: Day relative to inoculum is the number of days relative to day of administration of the inoculum, where the day of administration = 0.
The inoculum was administered on Study Day 0 for K13-infected subjects and Study Day 1 for 3D7-infected subjects.
Day relative to IP is the number of days relative to day of administration of Artesunate, where the day of dose = 1.
Rando = Randomisation; IP = Artesunate; H = High; L = Low; NCS = Not Clinically Significant; CS = Clinically Significant.

Listing 16.2.8.1a
Individual Haematology Results

Rando Number	Challenge Agent	Parameter (unit) [Normal Range]	Visit	Date of Assessment	Time of Assessment	Day Relative to Inoculum	Day Relative to IP	Result	Range Indicator	Clinical Assessment
R103	K13	Lymphocytes (10^9/L) [1 – 4]	Screening	01-NOV-2017	12:16	-13	-22	2.25		
			Safety Visit	13-NOV-2017	08:44	-1	-10	1.98		
			Admission	23-NOV-2017	09:50	9	1	1.39		
			Safety Monitoring	25-NOV-2017	17:52	11	3	0.80	L	NCS
			Admission 3	26-NOV-2017	10:53	12	4	0.49	L	CS
			Safety Monitoring	30-NOV-2017	10:09	16	8	2.32		
			Safety Monitoring	03-DEC-2017	10:45	19	11	2.46		
			Safety Monitoring	04-DEC-2017	09:27	20	12	2.03		
			Safety Monitoring	07-DEC-2017	08:41	23	15	2.33		
			End of Study	14-DEC-2017	09:37	30	22	1.82		

Notes: Day relative to inoculum is the number of days relative to day of administration of the inoculum, where the day of administration = 0.
The inoculum was administered on Study Day 0 for K13-infected subjects and Study Day 1 for 3D7-infected subjects.
Day relative to IP is the number of days relative to day of administration of Artesunate, where the day of dose = 1.
Rando = Randomisation; IP = Artesunate; H = High; L = Low; NCS = Not Clinically Significant; CS = Clinically Significant.

Listing 16.2.8.1a
Individual Haematology Results

Rando Number	Challenge Agent	Parameter (unit) [Normal Range]	Visit	Date of Assessment	Time of Assessment	Day Relative to Inoculum	Day Relative to IP	Result	Range Indicator	Clinical Assessment
R103	K13	Monocytes (10^9/L) [0 – 0.9]	Screening	01-NOV-2017	12:16	-13	-22	0.72		
			Safety Visit	13-NOV-2017	08:44	-1	-10	0.55		
			Admission	23-NOV-2017	09:50	9	1	0.60		
			Safety Monitoring	25-NOV-2017	17:52	11	3	0.52		
			Admission 3	26-NOV-2017	10:53	12	4	0.26		
			Safety Monitoring	30-NOV-2017	10:09	16	8	0.34		
			Safety Monitoring	03-DEC-2017	10:45	19	11	0.48		
			Safety Monitoring	04-DEC-2017	09:27	20	12	0.50		
			Safety Monitoring	07-DEC-2017	08:41	23	15	0.63		
			End of Study	14-DEC-2017	09:37	30	22	0.73		

Notes: Day relative to inoculum is the number of days relative to day of administration of the inoculum, where the day of administration = 0.
The inoculum was administered on Study Day 0 for K13-infected subjects and Study Day 1 for 3D7-infected subjects.
Day relative to IP is the number of days relative to day of administration of Artesunate, where the day of dose = 1.
Rando = Randomisation; IP = Artesunate; H = High; L = Low; NCS = Not Clinically Significant; CS = Clinically Significant.

Listing 16.2.8.1a
Individual Haematology Results

Rando Number	Challenge Agent	Parameter (unit) [Normal Range]	Visit	Date of Assessment	Time of Assessment	Day Relative to Inoculum	Day Relative to IP	Result	Range Indicator	Clinical Assessment
R103	K13	Neutrophils (10^9/L) [1.5 – 6.5]	Screening	01-NOV-2017	12:16	-13	-22	3.61		
			Safety Visit	13-NOV-2017	08:44	-1	-10	2.44		
			Admission	23-NOV-2017	09:50	9	1	2.65		
			Safety Monitoring	25-NOV-2017	17:52	11	3	2.39		
			Admission 3	26-NOV-2017	10:53	12	4	2.50		
			Safety Monitoring	30-NOV-2017	10:09	16	8	1.56		
			Safety Monitoring	03-DEC-2017	10:45	19	11	2.30		
			Safety Monitoring	04-DEC-2017	09:27	20	12	2.38		
			Safety Monitoring	07-DEC-2017	08:41	23	15	3.04		
			End of Study	14-DEC-2017	09:37	30	22	4.41		

Notes: Day relative to inoculum is the number of days relative to day of administration of the inoculum, where the day of administration = 0.
The inoculum was administered on Study Day 0 for K13-infected subjects and Study Day 1 for 3D7-infected subjects.
Day relative to IP is the number of days relative to day of administration of Artesunate, where the day of dose = 1.
Rando = Randomisation; IP = Artesunate; H = High; L = Low; NCS = Not Clinically Significant; CS = Clinically Significant.

Listing 16.2.8.1a
Individual Haematology Results

Rando Number	Challenge Agent	Parameter (unit) [Normal Range]	Visit	Date of Assessment	Time of Assessment	Day Relative to Inoculum	Day Relative to IP	Result	Range Indicator	Clinical Assessment
R103	K13	Platelets (10^9/L) [150 – 400]	Screening	01-NOV-2017	12:16	-13	-22	232		
			Safety Visit	13-NOV-2017	08:44	-1	-10	209		
			Admission	23-NOV-2017	09:50	9	1	202		
			Safety Monitoring	25-NOV-2017	17:52	11	3	136	L	NCS
			Admission 3	26-NOV-2017	10:53	12	4	114	L	NCS
			Safety Monitoring	30-NOV-2017	10:09	16	8	132	L	NCS
			Safety Monitoring	03-DEC-2017	10:45	19	11	247		
			Safety Monitoring	04-DEC-2017	09:27	20	12	242		
			Safety Monitoring	07-DEC-2017	08:41	23	15	276		
			End of Study	14-DEC-2017	09:37	30	22	199		
		Reticulocytes (10^9/L) [25 – 120]	Safety Visit	13-NOV-2017	08:44	-1	-10	52		
			End of Study	14-DEC-2017	09:37	30	22	72		

Notes: Day relative to inoculum is the number of days relative to day of administration of the inoculum, where the day of administration = 0.
The inoculum was administered on Study Day 0 for K13-infected subjects and Study Day 1 for 3D7-infected subjects.
Day relative to IP is the number of days relative to day of administration of Artesunate, where the day of dose = 1.
Rando = Randomisation; IP = Artesunate; H = High; L = Low; NCS = Not Clinically Significant; CS = Clinically Significant.

Listing 16.2.8.1a
Individual Haematology Results

Rando Number	Challenge Agent	Parameter (unit) [Normal Range]	Visit	Date of Assessment	Time of Assessment	Day Relative to Inoculum	Day Relative to IP	Result	Range Indicator	Clinical Assessment
R104	K13	Basophils (10^9/L) [0 – 0.15]	Screening	09-NOV-2017	10:32	-5	-14	0.05		
			Safety Visit	13-NOV-2017	07:54	-1	-10	0.05		
			Admission	23-NOV-2017	09:44	9	1	0.04		
			Safety Monitoring	25-NOV-2017	17:43	11	3	0.03		
			Admission 3	26-NOV-2017	10:41	12	4	0.04		
			Safety Monitoring	30-NOV-2017	08:43	16	8	0.04		
			Safety Monitoring	03-DEC-2017	09:13	19	11	0.05		
			Safety Monitoring	04-DEC-2017	12:45	20	12	0.06		
			Safety Monitoring	07-DEC-2017	08:35	23	15	0.05		
			End of Study	14-DEC-2017	08:42	30	22	0.04		
			Unscheduled	18-DEC-2017	08:14	34	26	0.04		
			Unscheduled	21-DEC-2017	07:35	37	29	0.04		
			Unscheduled	29-DEC-2017	10:15	45	37	0.04		

Notes: Day relative to inoculum is the number of days relative to day of administration of the inoculum, where the day of administration = 0.
The inoculum was administered on Study Day 0 for K13-infected subjects and Study Day 1 for 3D7-infected subjects.
Day relative to IP is the number of days relative to day of administration of Artesunate, where the day of dose = 1.
Rando = Randomisation; IP = Artesunate; H = High; L = Low; NCS = Not Clinically Significant; CS = Clinically Significant.

Listing 16.2.8.1a
Individual Haematology Results

Rando Number	Challenge Agent	Parameter (unit) [Normal Range]	Visit	Date of Assessment	Time of Assessment	Day Relative to Inoculum	Day Relative to IP	Result	Range Indicator	Clinical Assessment
R104	K13	Eosinophils (10^9/L) [0 – 0.6]	Screening	09-NOV-2017	10:32	-5	-14	0.27		
			Safety Visit	13-NOV-2017	07:54	-1	-10	0.37		
			Admission	23-NOV-2017	09:44	9	1	0.10		
			Safety Monitoring	25-NOV-2017	17:43	11	3	0.03		
			Admission 3	26-NOV-2017	10:41	12	4	0.02		
			Safety Monitoring	30-NOV-2017	08:43	16	8	0.15		
			Safety Monitoring	03-DEC-2017	09:13	19	11	0.16		
			Safety Monitoring	04-DEC-2017	12:45	20	12	0.22		
			Safety Monitoring	07-DEC-2017	08:35	23	15	0.14		
			End of Study	14-DEC-2017	08:42	30	22	0.13		
			Unscheduled	18-DEC-2017	08:14	34	26	0.20		
			Unscheduled	21-DEC-2017	07:35	37	29	0.20		
			Unscheduled	29-DEC-2017	10:15	45	37	0.26		

Notes: Day relative to inoculum is the number of days relative to day of administration of the inoculum, where the day of administration = 0.
The inoculum was administered on Study Day 0 for K13-infected subjects and Study Day 1 for 3D7-infected subjects.
Day relative to IP is the number of days relative to day of administration of Artesunate, where the day of dose = 1.
Rando = Randomisation; IP = Artesunate; H = High; L = Low; NCS = Not Clinically Significant; CS = Clinically Significant.

Listing 16.2.8.1a
Individual Haematology Results

Rando Number	Challenge Agent	Parameter (unit) [Normal Range]	Visit	Date of Assessment	Time of Assessment	Day Relative to Inoculum	Day Relative to IP	Result	Range Indicator	Clinical Assessment
R104	K13	Ery. Mean Corpuscular Volume (fL) [80 – 100]	Screening	09-NOV-2017	10:32	-5	-14	87		
			Safety Visit	13-NOV-2017	07:54	-1	-10	88		
			Admission	23-NOV-2017	09:44	9	1	84		
			Safety Monitoring	25-NOV-2017	17:43	11	3	86		
			Admission 3	26-NOV-2017	10:41	12	4	87		
			Safety Monitoring	30-NOV-2017	08:43	16	8	86		
			Safety Monitoring	03-DEC-2017	09:13	19	11	86		
			Safety Monitoring	04-DEC-2017	12:45	20	12	84		
			Safety Monitoring	07-DEC-2017	08:35	23	15	85		
			End of Study	14-DEC-2017	08:42	30	22	85		
			Unscheduled	18-DEC-2017	08:14	34	26	86		
			Unscheduled	21-DEC-2017	07:35	37	29	87		
			Unscheduled	29-DEC-2017	10:15	45	37	86		

Notes: Day relative to inoculum is the number of days relative to day of administration of the inoculum, where the day of administration = 0.
The inoculum was administered on Study Day 0 for K13-infected subjects and Study Day 1 for 3D7-infected subjects.
Day relative to IP is the number of days relative to day of administration of Artesunate, where the day of dose = 1.
Rando = Randomisation; IP = Artesunate; H = High; L = Low; NCS = Not Clinically Significant; CS = Clinically Significant.

Listing 16.2.8.1a
Individual Haematology Results

Rando Number	Challenge Agent	Parameter (unit) [Normal Range]	Visit	Date of Assessment	Time of Assessment	Day Relative to Inoculum	Day Relative to IP	Result	Range Indicator	Clinical Assessment
R104	K13	Erythrocytes (10^12/L) [4.5 – 6.5]	Screening	09-NOV-2017	10:32	-5	-14	4.9		
			Safety Visit	13-NOV-2017	07:54	-1	-10	4.9		
			Admission	23-NOV-2017	09:44	9	1	4.7		
			Safety Monitoring	25-NOV-2017	17:43	11	3	4.8		
			Admission 3	26-NOV-2017	10:41	12	4	4.8		
			Safety Monitoring	30-NOV-2017	08:43	16	8	4.6		
			Safety Monitoring	03-DEC-2017	09:13	19	11	4.5		
			Safety Monitoring	04-DEC-2017	12:45	20	12	4.4	L	NCS
			Safety Monitoring	07-DEC-2017	08:35	23	15	4.3	L	NCS
			End of Study	14-DEC-2017	08:42	30	22	4.2	L	NCS
			Unscheduled	18-DEC-2017	08:14	34	26	4.1	L	NCS
			Unscheduled	21-DEC-2017	07:35	37	29	4.2	L	NCS
			Unscheduled	29-DEC-2017	10:15	45	37	4.6		

Notes: Day relative to inoculum is the number of days relative to day of administration of the inoculum, where the day of administration = 0.
The inoculum was administered on Study Day 0 for K13-infected subjects and Study Day 1 for 3D7-infected subjects.
Day relative to IP is the number of days relative to day of administration of Artesunate, where the day of dose = 1.
Rando = Randomisation; IP = Artesunate; H = High; L = Low; NCS = Not Clinically Significant; CS = Clinically Significant.

Listing 16.2.8.1a
Individual Haematology Results

Rando Number	Challenge Agent	Parameter (unit) [Normal Range]	Visit	Date of Assessment	Time of Assessment	Day Relative to Inoculum	Day Relative to IP	Result	Range Indicator	Clinical Assessment
R104	K13	Hematocrit [0.4 – 0.54]	Screening	09-NOV-2017	10:32	-5	-14	0.43		
			Safety Visit	13-NOV-2017	07:54	-1	-10	0.43		
			Admission	23-NOV-2017	09:44	9	1	0.39	L	NCS
			Safety Monitoring	25-NOV-2017	17:43	11	3	0.42		
			Admission 3	26-NOV-2017	10:41	12	4	0.42		
			Safety Monitoring	30-NOV-2017	08:43	16	8	0.40		
			Safety Monitoring	03-DEC-2017	09:13	19	11	0.39	L	NCS
			Safety Monitoring	04-DEC-2017	12:45	20	12	0.37	L	NCS
			Safety Monitoring	07-DEC-2017	08:35	23	15	0.37	L	NCS
			End of Study	14-DEC-2017	08:42	30	22	0.36	L	NCS
			Unscheduled	18-DEC-2017	08:14	34	26	0.35	L	NCS
			Unscheduled	21-DEC-2017	07:35	37	29	0.37	L	NCS
			Unscheduled	29-DEC-2017	10:15	45	37	0.39	L	NCS

Notes: Day relative to inoculum is the number of days relative to day of administration of the inoculum, where the day of administration = 0.
The inoculum was administered on Study Day 0 for K13-infected subjects and Study Day 1 for 3D7-infected subjects.
Day relative to IP is the number of days relative to day of administration of Artesunate, where the day of dose = 1.
Rando = Randomisation; IP = Artesunate; H = High; L = Low; NCS = Not Clinically Significant; CS = Clinically Significant.

Listing 16.2.8.1a
Individual Haematology Results

Rando Number	Challenge Agent	Parameter (unit) [Normal Range]	Visit	Date of Assessment	Time of Assessment	Day Relative to Inoculum	Day Relative to IP	Result	Range Indicator	Clinical Assessment
R104	K13	Hemoglobin (g/L) [135 – 175]	Screening	09-NOV-2017	10:32	-5	-14	141		
			Safety Visit	13-NOV-2017	07:54	-1	-10	145		
			Admission	23-NOV-2017	09:44	9	1	135		
			Safety Monitoring	25-NOV-2017	17:43	11	3	142		
			Admission 3	26-NOV-2017	10:41	12	4	142		
			Safety Monitoring	30-NOV-2017	08:43	16	8	135		
			Safety Monitoring	03-DEC-2017	09:13	19	11	132	L	NCS
			Safety Monitoring	04-DEC-2017	12:45	20	12	127	L	NCS
			Safety Monitoring	07-DEC-2017	08:35	23	15	125	L	NCS
			End of Study	14-DEC-2017	08:42	30	22	123	L	NCS
			Unscheduled	18-DEC-2017	08:14	34	26	120	L	NCS
			Unscheduled	21-DEC-2017	07:35	37	29	123	L	NCS
			Unscheduled	29-DEC-2017	10:15	45	37	133	L	NCS

Notes: Day relative to inoculum is the number of days relative to day of administration of the inoculum, where the day of administration = 0.
The inoculum was administered on Study Day 0 for K13-infected subjects and Study Day 1 for 3D7-infected subjects.
Day relative to IP is the number of days relative to day of administration of Artesunate, where the day of dose = 1.
Rando = Randomisation; IP = Artesunate; H = High; L = Low; NCS = Not Clinically Significant; CS = Clinically Significant.

Listing 16.2.8.1a
Individual Haematology Results

Rando Number	Challenge Agent	Parameter (unit) [Normal Range]	Visit	Date of Assessment	Time of Assessment	Day Relative to Inoculum	Day Relative to IP	Result	Range Indicator	Clinical Assessment
R104	K13	Leukocytes (10^9/L) [3.5 – 10]	Screening	09-NOV-2017	10:32	-5	-14	5.1		
			Safety Visit	13-NOV-2017	07:54	-1	-10	5.5		
			Admission	23-NOV-2017	09:44	9	1	3.9		
			Safety Monitoring	25-NOV-2017	17:43	11	3	2.2	L	CS
			Admission 3	26-NOV-2017	10:41	12	4	2.2	L	CS
			Safety Monitoring	30-NOV-2017	08:43	16	8	4.1		
			Safety Monitoring	03-DEC-2017	09:13	19	11	5.2		
			Safety Monitoring	04-DEC-2017	12:45	20	12	5.8		
			Safety Monitoring	07-DEC-2017	08:35	23	15	5.4		
			End of Study	14-DEC-2017	08:42	30	22	4.4		
			Unscheduled	18-DEC-2017	08:14	34	26	4.4		
			Unscheduled	21-DEC-2017	07:35	37	29	4.4		
			Unscheduled	29-DEC-2017	10:15	45	37	6.0		

Notes: Day relative to inoculum is the number of days relative to day of administration of the inoculum, where the day of administration = 0.
The inoculum was administered on Study Day 0 for K13-infected subjects and Study Day 1 for 3D7-infected subjects.
Day relative to IP is the number of days relative to day of administration of Artesunate, where the day of dose = 1.
Rando = Randomisation; IP = Artesunate; H = High; L = Low; NCS = Not Clinically Significant; CS = Clinically Significant.

Listing 16.2.8.1a
Individual Haematology Results

Rando Number	Challenge Agent	Parameter (unit) [Normal Range]	Visit	Date of Assessment	Time of Assessment	Day Relative to Inoculum	Day Relative to IP	Result	Range Indicator	Clinical Assessment
R104	K13	Lymphocytes (10^9/L) [1 – 4]	Screening	09-NOV-2017	10:32	-5	-14	1.90		
			Safety Visit	13-NOV-2017	07:54	-1	-10	1.74		
			Admission	23-NOV-2017	09:44	9	1	0.81	L	NCS
			Safety Monitoring	25-NOV-2017	17:43	11	3	0.84	L	NCS
			Admission 3	26-NOV-2017	10:41	12	4	0.77	L	CS
			Safety Monitoring	30-NOV-2017	08:43	16	8	1.91		
			Safety Monitoring	03-DEC-2017	09:13	19	11	1.89		
			Safety Monitoring	04-DEC-2017	12:45	20	12	2.11		
			Safety Monitoring	07-DEC-2017	08:35	23	15	1.96		
			End of Study	14-DEC-2017	08:42	30	22	1.66		
			Unscheduled	18-DEC-2017	08:14	34	26	1.99		
			Unscheduled	21-DEC-2017	07:35	37	29	1.93		
			Unscheduled	29-DEC-2017	10:15	45	37	2.65		

Notes: Day relative to inoculum is the number of days relative to day of administration of the inoculum, where the day of administration = 0.
The inoculum was administered on Study Day 0 for K13-infected subjects and Study Day 1 for 3D7-infected subjects.
Day relative to IP is the number of days relative to day of administration of Artesunate, where the day of dose = 1.
Rando = Randomisation; IP = Artesunate; H = High; L = Low; NCS = Not Clinically Significant; CS = Clinically Significant.

Listing 16.2.8.1a
Individual Haematology Results

Rando Number	Challenge Agent	Parameter (unit) [Normal Range]	Visit	Date of Assessment	Time of Assessment	Day Relative to Inoculum	Day Relative to IP	Result	Range Indicator	Clinical Assessment
R104	K13	Monocytes (10^9/L) [0 – 0.9]	Screening	09-NOV-2017	10:32	-5	-14	0.28		
			Safety Visit	13-NOV-2017	07:54	-1	-10	0.44		
			Admission	23-NOV-2017	09:44	9	1	0.50		
			Safety Monitoring	25-NOV-2017	17:43	11	3	0.32		
			Admission 3	26-NOV-2017	10:41	12	4	0.35		
			Safety Monitoring	30-NOV-2017	08:43	16	8	0.37		
			Safety Monitoring	03-DEC-2017	09:13	19	11	0.40		
			Safety Monitoring	04-DEC-2017	12:45	20	12	0.47		
			Safety Monitoring	07-DEC-2017	08:35	23	15	0.56		
			End of Study	14-DEC-2017	08:42	30	22	0.35		
			Unscheduled	18-DEC-2017	08:14	34	26	0.37		
			Unscheduled	21-DEC-2017	07:35	37	29	0.35		
			Unscheduled	29-DEC-2017	10:15	45	37	0.34		

Notes: Day relative to inoculum is the number of days relative to day of administration of the inoculum, where the day of administration = 0.
The inoculum was administered on Study Day 0 for K13-infected subjects and Study Day 1 for 3D7-infected subjects.
Day relative to IP is the number of days relative to day of administration of Artesunate, where the day of dose = 1.
Rando = Randomisation; IP = Artesunate; H = High; L = Low; NCS = Not Clinically Significant; CS = Clinically Significant.

Listing 16.2.8.1a
Individual Haematology Results

Rando Number	Challenge Agent	Parameter (unit) [Normal Range]	Visit	Date of Assessment	Time of Assessment	Day Relative to Inoculum	Day Relative to IP	Result	Range Indicator	Clinical Assessment
R104	K13	Neutrophils (10^9/L) [1.5 – 6.5]	Screening	09-NOV-2017	10:32	-5	-14	2.56		
			Safety Visit	13-NOV-2017	07:54	-1	-10	2.91		
			Admission	23-NOV-2017	09:44	9	1	2.49		
			Safety Monitoring	25-NOV-2017	17:43	11	3	0.99	L	CS
			Admission 3	26-NOV-2017	10:41	12	4	1.05	L	CS
			Safety Monitoring	30-NOV-2017	08:43	16	8	1.59		
			Safety Monitoring	03-DEC-2017	09:13	19	11	2.70		
			Safety Monitoring	04-DEC-2017	12:45	20	12	2.93		
			Safety Monitoring	07-DEC-2017	08:35	23	15	2.68		
			End of Study	14-DEC-2017	08:42	30	22	2.20		
			Unscheduled	18-DEC-2017	08:14	34	26	1.78		
			Unscheduled	21-DEC-2017	07:35	37	29	1.84		
			Unscheduled	29-DEC-2017	10:15	45	37	2.68		

Notes: Day relative to inoculum is the number of days relative to day of administration of the inoculum, where the day of administration = 0.
The inoculum was administered on Study Day 0 for K13-infected subjects and Study Day 1 for 3D7-infected subjects.
Day relative to IP is the number of days relative to day of administration of Artesunate, where the day of dose = 1.
Rando = Randomisation; IP = Artesunate; H = High; L = Low; NCS = Not Clinically Significant; CS = Clinically Significant.

Listing 16.2.8.1a
Individual Haematology Results

Rando Number	Challenge Agent	Parameter (unit) [Normal Range]	Visit	Date of Assessment	Time of Assessment	Day Relative to Inoculum	Day Relative to IP	Result	Range Indicator	Clinical Assessment
R104	K13	Platelets (10^9/L) [150 – 400]	Screening	09-NOV-2017	10:32	-5	-14	234		
			Safety Visit	13-NOV-2017	07:54	-1	-10	245		
			Admission	23-NOV-2017	09:44	9	1	218		
			Safety Monitoring	25-NOV-2017	17:43	11	3	181		
			Admission 3	26-NOV-2017	10:41	12	4	169		
			Safety Monitoring	30-NOV-2017	08:43	16	8	264		
			Safety Monitoring	03-DEC-2017	09:13	19	11	375		
			Safety Monitoring	04-DEC-2017	12:45	20	12	358		
			Safety Monitoring	07-DEC-2017	08:35	23	15	319		
			End of Study	14-DEC-2017	08:42	30	22	251		
			Unscheduled	18-DEC-2017	08:14	34	26	234		
			Unscheduled	21-DEC-2017	07:35	37	29	225		
			Unscheduled	29-DEC-2017	10:15	45	37	245		
		Reticulocytes (10^9/L) [25 – 120]	Safety Visit	13-NOV-2017	07:54	-1	-10	58		
			End of Study	14-DEC-2017	08:42	30	22	91		

Notes: Day relative to inoculum is the number of days relative to day of administration of the inoculum, where the day of administration = 0.
The inoculum was administered on Study Day 0 for K13-infected subjects and Study Day 1 for 3D7-infected subjects.
Day relative to IP is the number of days relative to day of administration of Artesunate, where the day of dose = 1.
Rando = Randomisation; IP = Artesunate; H = High; L = Low; NCS = Not Clinically Significant; CS = Clinically Significant.

Listing 16.2.8.1a
Individual Haematology Results

Rando Number	Challenge Agent	Parameter (unit) [Normal Range]	Visit	Date of Assessment	Time of Assessment	Day Relative to Inoculum	Day Relative to IP	Result	Range Indicator	Clinical Assessment
R105	K13	Basophils (10^9/L) [0 – 0.15]	Screening	08-NOV-2017	12:46	-6	-15	0.03		
			Safety Visit	13-NOV-2017	08:06	-1	-10	0.03		
			Admission	23-NOV-2017	09:36	9	1	0.02		
			Safety Monitoring	25-NOV-2017	17:42	11	3	0.02		
			Admission 3	26-NOV-2017	10:31	12	4	0.02		
			Safety Monitoring	30-NOV-2017	07:05	16	8	0.03		
			Safety Monitoring	03-DEC-2017	07:45	19	11	0.03		
			End of Study	13-DEC-2017	08:54	29	21	0.03		
		Eosinophils (10^9/L) [0 – 0.6]	Screening	08-NOV-2017	12:46	-6	-15	0.06		
			Safety Visit	13-NOV-2017	08:06	-1	-10	0.13		
			Admission	23-NOV-2017	09:36	9	1	0.13		
			Safety Monitoring	25-NOV-2017	17:42	11	3	0.12		
			Admission 3	26-NOV-2017	10:31	12	4	0.06		
			Safety Monitoring	30-NOV-2017	07:05	16	8	0.11		
			Safety Monitoring	03-DEC-2017	07:45	19	11	0.13		
			End of Study	13-DEC-2017	08:54	29	21	0.08		

Notes: Day relative to inoculum is the number of days relative to day of administration of the inoculum, where the day of administration = 0.
The inoculum was administered on Study Day 0 for K13-infected subjects and Study Day 1 for 3D7-infected subjects.
Day relative to IP is the number of days relative to day of administration of Artesunate, where the day of dose = 1.
Rando = Randomisation; IP = Artesunate; H = High; L = Low; NCS = Not Clinically Significant; CS = Clinically Significant.

Listing 16.2.8.1a
Individual Haematology Results

Rando Number	Challenge Agent	Parameter (unit) [Normal Range]	Visit	Date of Assessment	Time of Assessment	Day Relative to Inoculum	Day Relative to IP	Result	Range Indicator	Clinical Assessment
R105	K13	Ery. Mean Corpuscular Volume (fL) [80 – 100]	Screening	08-NOV-2017	12:46	-6	-15	88		
			Safety Visit	13-NOV-2017	08:06	-1	-10	89		
			Admission	23-NOV-2017	09:36	9	1	86		
			Safety Monitoring	25-NOV-2017	17:42	11	3	89		
			Admission 3	26-NOV-2017	10:31	12	4	88		
			Safety Monitoring	30-NOV-2017	07:05	16	8	90		
			Safety Monitoring	03-DEC-2017	07:45	19	11	89		
			End of Study	13-DEC-2017	08:54	29	21	88		
		Erythrocytes (10^12/L) [4.5 – 6.5]	Screening	08-NOV-2017	12:46	-6	-15	5.2		
			Safety Visit	13-NOV-2017	08:06	-1	-10	5.2		
			Admission	23-NOV-2017	09:36	9	1	4.6		
			Safety Monitoring	25-NOV-2017	17:42	11	3	5.1		
			Admission 3	26-NOV-2017	10:31	12	4	4.8		
			Safety Monitoring	30-NOV-2017	07:05	16	8	4.4	L	NCS
			Safety Monitoring	03-DEC-2017	07:45	19	11	4.7		
			End of Study	13-DEC-2017	08:54	29	21	4.7		

Notes: Day relative to inoculum is the number of days relative to day of administration of the inoculum, where the day of administration = 0.
The inoculum was administered on Study Day 0 for K13-infected subjects and Study Day 1 for 3D7-infected subjects.
Day relative to IP is the number of days relative to day of administration of Artesunate, where the day of dose = 1.
Rando = Randomisation; IP = Artesunate; H = High; L = Low; NCS = Not Clinically Significant; CS = Clinically Significant.

Listing 16.2.8.1a
Individual Haematology Results

Rando Number	Challenge Agent	Parameter (unit) [Normal Range]	Visit	Date of Assessment	Time of Assessment	Day Relative to Inoculum	Day Relative to IP	Result	Range Indicator	Clinical Assessment
R105	K13	Hematocrit [0.4 – 0.54]	Screening	08-NOV-2017	12:46	-6	-15	0.46		
			Safety Visit	13-NOV-2017	08:06	-1	-10	0.46		
			Admission	23-NOV-2017	09:36	9	1	0.40		
			Safety Monitoring	25-NOV-2017	17:42	11	3	0.45		
			Admission 3	26-NOV-2017	10:31	12	4	0.43		
			Safety Monitoring	30-NOV-2017	07:05	16	8	0.40		
			Safety Monitoring	03-DEC-2017	07:45	19	11	0.42		
			End of Study	13-DEC-2017	08:54	29	21	0.42		
		Hemoglobin (g/L) [135 – 175]	Screening	08-NOV-2017	12:46	-6	-15	154		
			Safety Visit	13-NOV-2017	08:06	-1	-10	157		
			Admission	23-NOV-2017	09:36	9	1	138		
			Safety Monitoring	25-NOV-2017	17:42	11	3	153		
			Admission 3	26-NOV-2017	10:31	12	4	148		
			Safety Monitoring	30-NOV-2017	07:05	16	8	132	L	NCS
			Safety Monitoring	03-DEC-2017	07:45	19	11	140		
			End of Study	13-DEC-2017	08:54	29	21	141		

Notes: Day relative to inoculum is the number of days relative to day of administration of the inoculum, where the day of administration = 0.
The inoculum was administered on Study Day 0 for K13-infected subjects and Study Day 1 for 3D7-infected subjects.
Day relative to IP is the number of days relative to day of administration of Artesunate, where the day of dose = 1.
Rando = Randomisation; IP = Artesunate; H = High; L = Low; NCS = Not Clinically Significant; CS = Clinically Significant.

Listing 16.2.8.1a
Individual Haematology Results

Rando Number	Challenge Agent	Parameter (unit) [Normal Range]	Visit	Date of Assessment	Time of Assessment	Day Relative to Inoculum	Day Relative to IP	Result	Range Indicator	Clinical Assessment
R105	K13	Leukocytes (10^9/L) [3.5 – 10]	Screening	08-NOV-2017	12:46	-6	-15	7.9		
			Safety Visit	13-NOV-2017	08:06	-1	-10	8.5		
			Admission	23-NOV-2017	09:36	9	1	7.7		
			Safety Monitoring	25-NOV-2017	17:42	11	3	7.2		
			Admission 3	26-NOV-2017	10:31	12	4	5.4		
			Safety Monitoring	30-NOV-2017	07:05	16	8	6.1		
			Safety Monitoring	03-DEC-2017	07:45	19	11	7.7		
			End of Study	13-DEC-2017	08:54	29	21	7.1		
		Lymphocytes (10^9/L) [1 – 4]	Screening	08-NOV-2017	12:46	-6	-15	2.56		
			Safety Visit	13-NOV-2017	08:06	-1	-10	3.02		
			Admission	23-NOV-2017	09:36	9	1	2.58		
			Safety Monitoring	25-NOV-2017	17:42	11	3	2.37		
			Admission 3	26-NOV-2017	10:31	12	4	1.40		
			Safety Monitoring	30-NOV-2017	07:05	16	8	2.54		
			Safety Monitoring	03-DEC-2017	07:45	19	11	2.46		
			End of Study	13-DEC-2017	08:54	29	21	2.56		

Notes: Day relative to inoculum is the number of days relative to day of administration of the inoculum, where the day of administration = 0.
The inoculum was administered on Study Day 0 for K13-infected subjects and Study Day 1 for 3D7-infected subjects.
Day relative to IP is the number of days relative to day of administration of Artesunate, where the day of dose = 1.
Rando = Randomisation; IP = Artesunate; H = High; L = Low; NCS = Not Clinically Significant; CS = Clinically Significant.

Listing 16.2.8.1a
Individual Haematology Results

Rando Number	Challenge Agent	Parameter (unit) [Normal Range]	Visit	Date of Assessment	Time of Assessment	Day Relative to Inoculum	Day Relative to IP	Result	Range Indicator	Clinical Assessment
R105	K13	Monocytes (10^9/L) [0 – 0.9]	Screening	08-NOV-2017	12:46	-6	-15	0.63		
			Safety Visit	13-NOV-2017	08:06	-1	-10	0.69		
			Admission	23-NOV-2017	09:36	9	1	0.69		
			Safety Monitoring	25-NOV-2017	17:42	11	3	0.84		
			Admission 3	26-NOV-2017	10:31	12	4	0.69		
			Safety Monitoring	30-NOV-2017	07:05	16	8	0.57		
			Safety Monitoring	03-DEC-2017	07:45	19	11	0.63		
			End of Study	13-DEC-2017	08:54	29	21	0.57		
		Neutrophils (10^9/L) [1.5 – 6.5]	Screening	08-NOV-2017	12:46	-6	-15	4.59		
			Safety Visit	13-NOV-2017	08:06	-1	-10	4.58		
			Admission	23-NOV-2017	09:36	9	1	4.26		
			Safety Monitoring	25-NOV-2017	17:42	11	3	3.82		
			Admission 3	26-NOV-2017	10:31	12	4	3.24		
			Safety Monitoring	30-NOV-2017	07:05	16	8	2.81		
			Safety Monitoring	03-DEC-2017	07:45	19	11	4.41		
			End of Study	13-DEC-2017	08:54	29	21	3.89		

Notes: Day relative to inoculum is the number of days relative to day of administration of the inoculum, where the day of administration = 0.
The inoculum was administered on Study Day 0 for K13-infected subjects and Study Day 1 for 3D7-infected subjects.
Day relative to IP is the number of days relative to day of administration of Artesunate, where the day of dose = 1.
Rando = Randomisation; IP = Artesunate; H = High; L = Low; NCS = Not Clinically Significant; CS = Clinically Significant.

Listing 16.2.8.1a
Individual Haematology Results

Rando Number	Challenge Agent	Parameter (unit) [Normal Range]	Visit	Date of Assessment	Time of Assessment	Day Relative to Inoculum	Day Relative to IP	Result	Range Indicator	Clinical Assessment
R105	K13	Platelets (10^9/L) [150 – 400]	Screening	08-NOV-2017	12:46	-6	-15	291		
			Safety Visit	13-NOV-2017	08:06	-1	-10	313		
			Admission	23-NOV-2017	09:36	9	1	260		
			Safety Monitoring	25-NOV-2017	17:42	11	3	270		
			Admission 3	26-NOV-2017	10:31	12	4	240		
			Safety Monitoring	30-NOV-2017	07:05	16	8	243		
			Safety Monitoring	03-DEC-2017	07:45	19	11	332		
			End of Study	13-DEC-2017	08:54	29	21	278		
		Reticulocytes (10^9/L) [25 – 120]	Safety Visit	13-NOV-2017	08:06	-1	-10	65		
			End of Study	13-DEC-2017	08:54	29	21	84		

Notes: Day relative to inoculum is the number of days relative to day of administration of the inoculum, where the day of administration = 0.
The inoculum was administered on Study Day 0 for K13-infected subjects and Study Day 1 for 3D7-infected subjects.
Day relative to IP is the number of days relative to day of administration of Artesunate, where the day of dose = 1.
Rando = Randomisation; IP = Artesunate; H = High; L = Low; NCS = Not Clinically Significant; CS = Clinically Significant.

Listing 16.2.8.1a
Individual Haematology Results

Rando Number	Challenge Agent	Parameter (unit) [Normal Range]	Visit	Date of Assessment	Time of Assessment	Day Relative to Inoculum	Day Relative to IP	Result	Range Indicator	Clinical Assessment
R106	3D7	Basophils (10^9/L) [0 – 0.15]	Screening	09-NOV-2017	10:57	-6	-14	0.03		
			Safety Visit	13-NOV-2017	07:45	-2	-10	0.04		
			Admission	23-NOV-2017	09:33	8	1	0.04		
			Admission 3	26-NOV-2017	10:20	11	4	0.03		
			Safety Monitoring	30-NOV-2017	08:46	15	8	0.04		
			Safety Monitoring	01-DEC-2017	08:27	16	9	0.01		
			Safety Monitoring	03-DEC-2017	09:39	18	11	0.05		
			Safety Monitoring	11-DEC-2017	08:04	26	19	0.05		
			End of Study	14-DEC-2017	08:53	29	22	0.04		
R106		Eosinophils (10^9/L) [0 – 0.6]	Screening	09-NOV-2017	10:57	-6	-14	0.19		
			Safety Visit	13-NOV-2017	07:45	-2	-10	0.37		
			Admission	23-NOV-2017	09:33	8	1	0.11		
			Admission 3	26-NOV-2017	10:20	11	4	0.12		
			Safety Monitoring	30-NOV-2017	08:46	15	8	0.27		
			Safety Monitoring	01-DEC-2017	08:27	16	9	0.18		
			Safety Monitoring	03-DEC-2017	09:39	18	11	0.18		
			Safety Monitoring	11-DEC-2017	08:04	26	19	0.23		
			End of Study	14-DEC-2017	08:53	29	22	0.53		

Notes: Day relative to inoculum is the number of days relative to day of administration of the inoculum, where the day of administration = 0.
The inoculum was administered on Study Day 0 for K13-infected subjects and Study Day 1 for 3D7-infected subjects.
Day relative to IP is the number of days relative to day of administration of Artesunate, where the day of dose = 1.
Rando = Randomisation; IP = Artesunate; H = High; L = Low; NCS = Not Clinically Significant; CS = Clinically Significant.

Listing 16.2.8.1a
Individual Haematology Results

Rando Number	Challenge Agent	Parameter (unit) [Normal Range]	Visit	Date of Assessment	Time of Assessment	Day Relative to Inoculum	Day Relative to IP	Result	Range Indicator	Clinical Assessment
R106	3D7	Ery. Mean Corpuscular Volume (fL) [80 – 100]	Screening	09-NOV-2017	10:57	-6	-14	85		
			Safety Visit	13-NOV-2017	07:45	-2	-10	86		
			Admission	23-NOV-2017	09:33	8	1	83		
			Admission 3	26-NOV-2017	10:20	11	4	85		
			Safety Monitoring	30-NOV-2017	08:46	15	8	84		
			Safety Monitoring	01-DEC-2017	08:27	16	9	83		
			Safety Monitoring	03-DEC-2017	09:39	18	11	85		
			Safety Monitoring	11-DEC-2017	08:04	26	19	84		
			End of Study	14-DEC-2017	08:53	29	22	84		
R106		Erythrocytes (10^12/L) [3.9 – 5.6]	Screening	09-NOV-2017	10:57	-6	-14	4.5		
			Safety Visit	13-NOV-2017	07:45	-2	-10	4.7		
			Admission	23-NOV-2017	09:33	8	1	4.3		
			Admission 3	26-NOV-2017	10:20	11	4	4.4		
			Safety Monitoring	30-NOV-2017	08:46	15	8	4.4		
			Safety Monitoring	01-DEC-2017	08:27	16	9	4.2		
			Safety Monitoring	03-DEC-2017	09:39	18	11	4.2		
			Safety Monitoring	11-DEC-2017	08:04	26	19	4.0		
			End of Study	14-DEC-2017	08:53	29	22	3.9		

Notes: Day relative to inoculum is the number of days relative to day of administration of the inoculum, where the day of administration = 0.
The inoculum was administered on Study Day 0 for K13-infected subjects and Study Day 1 for 3D7-infected subjects.
Day relative to IP is the number of days relative to day of administration of Artesunate, where the day of dose = 1.
Rando = Randomisation; IP = Artesunate; H = High; L = Low; NCS = Not Clinically Significant; CS = Clinically Significant.

Listing 16.2.8.1a
Individual Haematology Results

Rando Number	Challenge Agent	Parameter (unit) [Normal Range]	Visit	Date of Assessment	Time of Assessment	Day Relative to Inoculum	Day Relative to IP	Result	Range Indicator	Clinical Assessment
R106	3D7	Hematocrit [0.35 – 0.47]	Screening	09-NOV-2017	10:57	-6	-14	0.38		
			Safety Visit	13-NOV-2017	07:45	-2	-10	0.40		
			Admission	23-NOV-2017	09:33	8	1	0.35		
			Admission 3	26-NOV-2017	10:20	11	4	0.37		
			Safety Monitoring	30-NOV-2017	08:46	15	8	0.37		
			Safety Monitoring	01-DEC-2017	08:27	16	9	0.35		
			Safety Monitoring	03-DEC-2017	09:39	18	11	0.35		
			Safety Monitoring	11-DEC-2017	08:04	26	19	0.33	L	NCS
			End of Study	14-DEC-2017	08:53	29	22	0.33	L	NCS
		Hemoglobin (g/L) [115 – 165]	Screening	09-NOV-2017	10:57	-6	-14	137		
			Safety Visit	13-NOV-2017	07:45	-2	-10	139		
			Admission	23-NOV-2017	09:33	8	1	128		
			Admission 3	26-NOV-2017	10:20	11	4	133		
			Safety Monitoring	30-NOV-2017	08:46	15	8	128		
			Safety Monitoring	01-DEC-2017	08:27	16	9	121		
			Safety Monitoring	03-DEC-2017	09:39	18	11	125		
			Safety Monitoring	11-DEC-2017	08:04	26	19	116		
			End of Study	14-DEC-2017	08:53	29	22	117		

Notes: Day relative to inoculum is the number of days relative to day of administration of the inoculum, where the day of administration = 0.
The inoculum was administered on Study Day 0 for K13-infected subjects and Study Day 1 for 3D7-infected subjects.
Day relative to IP is the number of days relative to day of administration of Artesunate, where the day of dose = 1.
Rando = Randomisation; IP = Artesunate; H = High; L = Low; NCS = Not Clinically Significant; CS = Clinically Significant.

Listing 16.2.8.1a
Individual Haematology Results

Rando Number	Challenge Agent	Parameter (unit) [Normal Range]	Visit	Date of Assessment	Time of Assessment	Day Relative to Inoculum	Day Relative to IP	Result	Range Indicator	Clinical Assessment
R106	3D7	Leukocytes (10^9/L) [3.5 – 12]	Screening	09-NOV-2017	10:57	-6	-14	6.1		
			Safety Visit	13-NOV-2017	07:45	-2	-10	5.7		
			Admission	23-NOV-2017	09:33	8	1	5.8		
			Admission 3	26-NOV-2017	10:20	11	4	3.1	L	NCS
			Safety Monitoring	30-NOV-2017	08:46	15	8	4.9		
			Safety Monitoring	01-DEC-2017	08:27	16	9	4.0		
			Safety Monitoring	03-DEC-2017	09:39	18	11	3.8		
			Safety Monitoring	11-DEC-2017	08:04	26	19	5.4		
			End of Study	14-DEC-2017	08:53	29	22	5.6		
R106		Lymphocytes (10^9/L) [1 – 4]	Screening	09-NOV-2017	10:57	-6	-14	1.81		
			Safety Visit	13-NOV-2017	07:45	-2	-10	1.70		
			Admission	23-NOV-2017	09:33	8	1	1.12		
			Admission 3	26-NOV-2017	10:20	11	4	1.03		
			Safety Monitoring	30-NOV-2017	08:46	15	8	1.47		
			Safety Monitoring	01-DEC-2017	08:27	16	9	0.78	L	CS
			Safety Monitoring	03-DEC-2017	09:39	18	11	1.55		
			Safety Monitoring	11-DEC-2017	08:04	26	19	1.60		
			End of Study	14-DEC-2017	08:53	29	22	1.27		

Notes: Day relative to inoculum is the number of days relative to day of administration of the inoculum, where the day of administration = 0.
The inoculum was administered on Study Day 0 for K13-infected subjects and Study Day 1 for 3D7-infected subjects.
Day relative to IP is the number of days relative to day of administration of Artesunate, where the day of dose = 1.
Rando = Randomisation; IP = Artesunate; H = High; L = Low; NCS = Not Clinically Significant; CS = Clinically Significant.

Listing 16.2.8.1a
Individual Haematology Results

Rando Number	Challenge Agent	Parameter (unit) [Normal Range]	Visit	Date of Assessment	Time of Assessment	Day Relative to Inoculum	Day Relative to IP	Result	Range Indicator	Clinical Assessment
R106	3D7	Monocytes (10^9/L) [0 – 0.9]	Screening	09-NOV-2017	10:57	-6	-14	0.40		
			Safety Visit	13-NOV-2017	07:45	-2	-10	0.38		
			Admission	23-NOV-2017	09:33	8	1	0.40		
			Admission 3	26-NOV-2017	10:20	11	4	0.39		
			Safety Monitoring	30-NOV-2017	08:46	15	8	0.37		
			Safety Monitoring	01-DEC-2017	08:27	16	9	0.36		
			Safety Monitoring	03-DEC-2017	09:39	18	11	0.39		
			Safety Monitoring	11-DEC-2017	08:04	26	19	0.37		
			End of Study	14-DEC-2017	08:53	29	22	0.34		
		Neutrophils (10^9/L) [1.5 – 8]	Screening	09-NOV-2017	10:57	-6	-14	3.70		
			Safety Visit	13-NOV-2017	07:45	-2	-10	3.23		
			Admission	23-NOV-2017	09:33	8	1	4.12		
			Admission 3	26-NOV-2017	10:20	11	4	1.57		
			Safety Monitoring	30-NOV-2017	08:46	15	8	2.73		
			Safety Monitoring	01-DEC-2017	08:27	16	9	2.64		
			Safety Monitoring	03-DEC-2017	09:39	18	11	1.61		
			Safety Monitoring	11-DEC-2017	08:04	26	19	3.15		
			End of Study	14-DEC-2017	08:53	29	22	3.42		

Notes: Day relative to inoculum is the number of days relative to day of administration of the inoculum, where the day of administration = 0.
The inoculum was administered on Study Day 0 for K13-infected subjects and Study Day 1 for 3D7-infected subjects.
Day relative to IP is the number of days relative to day of administration of Artesunate, where the day of dose = 1.
Rando = Randomisation; IP = Artesunate; H = High; L = Low; NCS = Not Clinically Significant; CS = Clinically Significant.

Listing 16.2.8.1a
Individual Haematology Results

Rando Number	Challenge Agent	Parameter (unit) [Normal Range]	Visit	Date of Assessment	Time of Assessment	Day Relative to Inoculum	Day Relative to IP	Result	Range Indicator	Clinical Assessment
R106	3D7	Platelets (10^9/L) [150 – 400]	Screening	09-NOV-2017	10:57	-6	-14	211		
			Safety Visit	13-NOV-2017	07:45	-2	-10	196		
			Admission	23-NOV-2017	09:33	8	1	195		
			Admission 3	26-NOV-2017	10:20	11	4	165		
			Safety Monitoring	30-NOV-2017	08:46	15	8	193		
			Safety Monitoring	01-DEC-2017	08:27	16	9	174		
			Safety Monitoring	03-DEC-2017	09:39	18	11	201		
			Safety Monitoring	11-DEC-2017	08:04	26	19	235		
			End of Study	14-DEC-2017	08:53	29	22	204		
		Reticulocytes (10^9/L) [25 – 120]	Safety Visit	13-NOV-2017	07:45	-2	-10	71		
			End of Study	14-DEC-2017	08:53	29	22	87		

Notes: Day relative to inoculum is the number of days relative to day of administration of the inoculum, where the day of administration = 0.
The inoculum was administered on Study Day 0 for K13-infected subjects and Study Day 1 for 3D7-infected subjects.
Day relative to IP is the number of days relative to day of administration of Artesunate, where the day of dose = 1.
Rando = Randomisation; IP = Artesunate; H = High; L = Low; NCS = Not Clinically Significant; CS = Clinically Significant.

Listing 16.2.8.1a
Individual Haematology Results

Rando Number	Challenge Agent	Parameter (unit) [Normal Range]	Visit	Date of Assessment	Time of Assessment	Day Relative to Inoculum	Day Relative to IP	Result	Range Indicator	Clinical Assessment
R107	3D7	Basophils (10^9/L) [0 – 0.15]	Screening	03-NOV-2017	11:25	-12	-20	0.02		
			Safety Visit	13-NOV-2017	08:17	-2	-10	0.03		
			Admission	23-NOV-2017	09:35	8	1	0.03		
			Admission 3	26-NOV-2017	10:11	11	4	0.02		
			Safety Monitoring	30-NOV-2017	07:30	15	8	0.03		
			Safety Monitoring	03-DEC-2017	08:29	18	11	0.02		
			Safety Monitoring	04-DEC-2017	08:48	19	12	0.03		
			Safety Monitoring	11-DEC-2017	09:29	26	19	0.03		
			End of Study	14-DEC-2017	09:31	29	22	0.04		
		Eosinophils (10^9/L) [0 – 0.6]	Screening	03-NOV-2017	11:25	-12	-20	0.15		
			Safety Visit	13-NOV-2017	08:17	-2	-10	0.14		
			Admission	23-NOV-2017	09:35	8	1	0.14		
			Admission 3	26-NOV-2017	10:11	11	4	0.10		
			Safety Monitoring	30-NOV-2017	07:30	15	8	0.12		
			Safety Monitoring	03-DEC-2017	08:29	18	11	0.05		
			Safety Monitoring	04-DEC-2017	08:48	19	12	0.06		
			Safety Monitoring	11-DEC-2017	09:29	26	19	0.13		
			End of Study	14-DEC-2017	09:31	29	22	0.11		

Notes: Day relative to inoculum is the number of days relative to day of administration of the inoculum, where the day of administration = 0.
The inoculum was administered on Study Day 0 for K13-infected subjects and Study Day 1 for 3D7-infected subjects.
Day relative to IP is the number of days relative to day of administration of Artesunate, where the day of dose = 1.
Rando = Randomisation; IP = Artesunate; H = High; L = Low; NCS = Not Clinically Significant; CS = Clinically Significant.

Listing 16.2.8.1a
Individual Haematology Results

Rando Number	Challenge Agent	Parameter (unit) [Normal Range]	Visit	Date of Assessment	Time of Assessment	Day Relative to Inoculum	Day Relative to IP	Result	Range Indicator	Clinical Assessment
R107	3D7	Ery. Mean Corpuscular Volume (fL) [80 – 100]	Screening	03-NOV-2017	11:25	-12	-20	86		
			Safety Visit	13-NOV-2017	08:17	-2	-10	88		
			Admission	23-NOV-2017	09:35	8	1	86		
			Admission 3	26-NOV-2017	10:11	11	4	89		
			Safety Monitoring	30-NOV-2017	07:30	15	8	88		
			Safety Monitoring	03-DEC-2017	08:29	18	11	88		
			Safety Monitoring	04-DEC-2017	08:48	19	12	87		
			Safety Monitoring	11-DEC-2017	09:29	26	19	86		
			End of Study	14-DEC-2017	09:31	29	22	87		
R107		Erythrocytes (10^12/L) [4.5 – 6.5]	Screening	03-NOV-2017	11:25	-12	-20	5.5		
			Safety Visit	13-NOV-2017	08:17	-2	-10	5.7		
			Admission	23-NOV-2017	09:35	8	1	5.1		
			Admission 3	26-NOV-2017	10:11	11	4	5.4		
			Safety Monitoring	30-NOV-2017	07:30	15	8	5.1		
			Safety Monitoring	03-DEC-2017	08:29	18	11	5.4		
			Safety Monitoring	04-DEC-2017	08:48	19	12	5.6		
			Safety Monitoring	11-DEC-2017	09:29	26	19	5.3		
			End of Study	14-DEC-2017	09:31	29	22	5.2		

Notes: Day relative to inoculum is the number of days relative to day of administration of the inoculum, where the day of administration = 0.
The inoculum was administered on Study Day 0 for K13-infected subjects and Study Day 1 for 3D7-infected subjects.
Day relative to IP is the number of days relative to day of administration of Artesunate, where the day of dose = 1.
Rando = Randomisation; IP = Artesunate; H = High; L = Low; NCS = Not Clinically Significant; CS = Clinically Significant.

Listing 16.2.8.1a
Individual Haematology Results

Rando Number	Challenge Agent	Parameter (unit) [Normal Range]	Visit	Date of Assessment	Time of Assessment	Day Relative to Inoculum	Day Relative to IP	Result	Range Indicator	Clinical Assessment
R107	3D7	Hematocrit [0.4 – 0.54]	Screening	03-NOV-2017	11:25	-12	-20	0.48		
			Safety Visit	13-NOV-2017	08:17	-2	-10	0.50		
			Admission	23-NOV-2017	09:35	8	1	0.43		
			Admission 3	26-NOV-2017	10:11	11	4	0.48		
			Safety Monitoring	30-NOV-2017	07:30	15	8	0.45		
			Safety Monitoring	03-DEC-2017	08:29	18	11	0.48		
			Safety Monitoring	04-DEC-2017	08:48	19	12	0.49		
			Safety Monitoring	11-DEC-2017	09:29	26	19	0.46		
			End of Study	14-DEC-2017	09:31	29	22	0.46		
		Hemoglobin (g/L) [135 – 175]	Screening	03-NOV-2017	11:25	-12	-20	161		
			Safety Visit	13-NOV-2017	08:17	-2	-10	162		
			Admission	23-NOV-2017	09:35	8	1	146		
			Admission 3	26-NOV-2017	10:11	11	4	156		
			Safety Monitoring	30-NOV-2017	07:30	15	8	145		
			Safety Monitoring	03-DEC-2017	08:29	18	11	160		
			Safety Monitoring	04-DEC-2017	08:48	19	12	160		
			Safety Monitoring	11-DEC-2017	09:29	26	19	150		
			End of Study	14-DEC-2017	09:31	29	22	150		

Notes: Day relative to inoculum is the number of days relative to day of administration of the inoculum, where the day of administration = 0.
The inoculum was administered on Study Day 0 for K13-infected subjects and Study Day 1 for 3D7-infected subjects.
Day relative to IP is the number of days relative to day of administration of Artesunate, where the day of dose = 1.
Rando = Randomisation; IP = Artesunate; H = High; L = Low; NCS = Not Clinically Significant; CS = Clinically Significant.

Listing 16.2.8.1a
Individual Haematology Results

Rando Number	Challenge Agent	Parameter (unit) [Normal Range]	Visit	Date of Assessment	Time of Assessment	Day Relative to Inoculum	Day Relative to IP	Result	Range Indicator	Clinical Assessment
R107	3D7	Leukocytes (10^9/L) [3.5 – 10]	Screening	03-NOV-2017	11:25	-12	-20	7.8		
			Safety Visit	13-NOV-2017	08:17	-2	-10	7.4		
			Admission	23-NOV-2017	09:35	8	1	6.6		
			Admission 3	26-NOV-2017	10:11	11	4	6.0		
			Safety Monitoring	30-NOV-2017	07:30	15	8	6.5		
			Safety Monitoring	03-DEC-2017	08:29	18	11	5.9		
			Safety Monitoring	04-DEC-2017	08:48	19	12	5.0		
			Safety Monitoring	11-DEC-2017	09:29	26	19	7.0		
			End of Study	14-DEC-2017	09:31	29	22	6.5		
R107		Lymphocytes (10^9/L) [1 – 4]	Screening	03-NOV-2017	11:25	-12	-20	2.95		
			Safety Visit	13-NOV-2017	08:17	-2	-10	3.18		
			Admission	23-NOV-2017	09:35	8	1	2.59		
			Admission 3	26-NOV-2017	10:11	11	4	1.99		
			Safety Monitoring	30-NOV-2017	07:30	15	8	2.50		
			Safety Monitoring	03-DEC-2017	08:29	18	11	1.23		
			Safety Monitoring	04-DEC-2017	08:48	19	12	1.12		
			Safety Monitoring	11-DEC-2017	09:29	26	19	2.38		
			End of Study	14-DEC-2017	09:31	29	22	2.25		

Notes: Day relative to inoculum is the number of days relative to day of administration of the inoculum, where the day of administration = 0.
The inoculum was administered on Study Day 0 for K13-infected subjects and Study Day 1 for 3D7-infected subjects.
Day relative to IP is the number of days relative to day of administration of Artesunate, where the day of dose = 1.
Rando = Randomisation; IP = Artesunate; H = High; L = Low; NCS = Not Clinically Significant; CS = Clinically Significant.

Listing 16.2.8.1a
Individual Haematology Results

Rando Number	Challenge Agent	Parameter (unit) [Normal Range]	Visit	Date of Assessment	Time of Assessment	Day Relative to Inoculum	Day Relative to IP	Result	Range Indicator	Clinical Assessment
R107	3D7	Monocytes (10^9/L) [0 – 0.9]	Screening	03-NOV-2017	11:25	-12	-20	0.57		
			Safety Visit	13-NOV-2017	08:17	-2	-10	0.61		
			Admission	23-NOV-2017	09:35	8	1	0.59		
			Admission 3	26-NOV-2017	10:11	11	4	0.51		
			Safety Monitoring	30-NOV-2017	07:30	15	8	0.59		
			Safety Monitoring	03-DEC-2017	08:29	18	11	0.62		
			Safety Monitoring	04-DEC-2017	08:48	19	12	0.54		
			Safety Monitoring	11-DEC-2017	09:29	26	19	0.50		
			End of Study	14-DEC-2017	09:31	29	22	0.41		
		Neutrophils (10^9/L) [1.5 – 6.5]	Screening	03-NOV-2017	11:25	-12	-20	4.12		
			Safety Visit	13-NOV-2017	08:17	-2	-10	3.45		
			Admission	23-NOV-2017	09:35	8	1	3.21		
			Admission 3	26-NOV-2017	10:11	11	4	3.33		
			Safety Monitoring	30-NOV-2017	07:30	15	8	3.21		
			Safety Monitoring	03-DEC-2017	08:29	18	11	3.97		
			Safety Monitoring	04-DEC-2017	08:48	19	12	3.21		
			Safety Monitoring	11-DEC-2017	09:29	26	19	3.98		
			End of Study	14-DEC-2017	09:31	29	22	3.65		

Notes: Day relative to inoculum is the number of days relative to day of administration of the inoculum, where the day of administration = 0.
The inoculum was administered on Study Day 0 for K13-infected subjects and Study Day 1 for 3D7-infected subjects.
Day relative to IP is the number of days relative to day of administration of Artesunate, where the day of dose = 1.
Rando = Randomisation; IP = Artesunate; H = High; L = Low; NCS = Not Clinically Significant; CS = Clinically Significant.

Listing 16.2.8.1a
Individual Haematology Results

Rando Number	Challenge Agent	Parameter (unit) [Normal Range]	Visit	Date of Assessment	Time of Assessment	Day Relative to Inoculum	Day Relative to IP	Result	Range Indicator	Clinical Assessment
R107	3D7	Platelets (10^9/L) [150 – 400]	Screening	03-NOV-2017	11:25	-12	-20	293		
			Safety Visit	13-NOV-2017	08:17	-2	-10	286		
			Admission	23-NOV-2017	09:35	8	1	141	L	NCS
			Admission 3	26-NOV-2017	10:11	11	4	307		
			Safety Monitoring	30-NOV-2017	07:30	15	8	289		
			Safety Monitoring	03-DEC-2017	08:29	18	11	291		
			Safety Monitoring	04-DEC-2017	08:48	19	12	239		
			Safety Monitoring	11-DEC-2017	09:29	26	19	306		
			End of Study	14-DEC-2017	09:31	29	22	319		
		Reticulocytes (10^9/L) [25 – 120]	Safety Visit	13-NOV-2017	08:17	-2	-10	87		
			End of Study	14-DEC-2017	09:31	29	22	107		

Notes: Day relative to inoculum is the number of days relative to day of administration of the inoculum, where the day of administration = 0.
The inoculum was administered on Study Day 0 for K13-infected subjects and Study Day 1 for 3D7-infected subjects.
Day relative to IP is the number of days relative to day of administration of Artesunate, where the day of dose = 1.
Rando = Randomisation; IP = Artesunate; H = High; L = Low; NCS = Not Clinically Significant; CS = Clinically Significant.

Listing 16.2.8.1a
Individual Haematology Results

Rando Number	Challenge Agent	Parameter (unit) [Normal Range]	Visit	Date of Assessment	Time of Assessment	Day Relative to Inoculum	Day Relative to IP	Result	Range Indicator	Clinical Assessment
R108	3D7	Basophils (10^9/L) [0 – 0.15]	Screening	26-OCT-2017	09:30	-20	-28	0.08		
			Safety Visit	13-NOV-2017	07:27	-2	-10	0.06		
			Admission	23-NOV-2017	09:17	8	1	0.05		
			Admission 3	26-NOV-2017	10:02	11	4	0.03		
			Safety Monitoring	30-NOV-2017	06:57	15	8	0.06		
			Safety Monitoring	01-DEC-2017	07:14	16	9	0.06		
			Safety Monitoring	03-DEC-2017	08:50	18	11	0.07		
			Safety Monitoring	11-DEC-2017	08:54	26	19	0.08		
			End of Study	14-DEC-2017	08:37	29	22	0.08		
			Unscheduled	18-DEC-2017	07:48	33	26	0.09		
			Unscheduled	21-DEC-2017	07:53	36	29	0.12		
			Unscheduled	08-JAN-2018	08:06	54	47	0.06		

Notes: Day relative to inoculum is the number of days relative to day of administration of the inoculum, where the day of administration = 0.
The inoculum was administered on Study Day 0 for K13-infected subjects and Study Day 1 for 3D7-infected subjects.
Day relative to IP is the number of days relative to day of administration of Artesunate, where the day of dose = 1.
Rando = Randomisation; IP = Artesunate; H = High; L = Low; NCS = Not Clinically Significant; CS = Clinically Significant.

Listing 16.2.8.1a
Individual Haematology Results

Rando Number	Challenge Agent	Parameter (unit) [Normal Range]	Visit	Date of Assessment	Time of Assessment	Day Relative to Inoculum	Day Relative to IP	Result	Range Indicator	Clinical Assessment
R108	3D7	Eosinophils (10^9/L) [0 – 0.6]	Screening	26-OCT-2017	09:30	-20	-28	0.53		
			Safety Visit	13-NOV-2017	07:27	-2	-10	0.58		
			Admission	23-NOV-2017	09:17	8	1	0.30		
			Admission 3	26-NOV-2017	10:02	11	4	0.22		
			Safety Monitoring	30-NOV-2017	06:57	15	8	0.64	H	NCS
			Safety Monitoring	01-DEC-2017	07:14	16	9	0.50		
			Safety Monitoring	03-DEC-2017	08:50	18	11	0.39		
			Safety Monitoring	11-DEC-2017	08:54	26	19	0.54		
			End of Study	14-DEC-2017	08:37	29	22	1.07	H	CS
			Unscheduled	18-DEC-2017	07:48	33	26	1.02	H	CS
			Unscheduled	21-DEC-2017	07:53	36	29	0.72	H	NCS
			Unscheduled	08-JAN-2018	08:06	54	47	0.74	H	NCS

Notes: Day relative to inoculum is the number of days relative to day of administration of the inoculum, where the day of administration = 0.
The inoculum was administered on Study Day 0 for K13-infected subjects and Study Day 1 for 3D7-infected subjects.
Day relative to IP is the number of days relative to day of administration of Artesunate, where the day of dose = 1.
Rando = Randomisation; IP = Artesunate; H = High; L = Low; NCS = Not Clinically Significant; CS = Clinically Significant.

Listing 16.2.8.1a
Individual Haematology Results

Rando Number	Challenge Agent	Parameter (unit) [Normal Range]	Visit	Date of Assessment	Time of Assessment	Day Relative to Inoculum	Day Relative to IP	Result	Range Indicator	Clinical Assessment
R108	3D7	Ery. Mean Corpuscular Volume (fL) [80 – 100]	Screening	26-OCT-2017	09:30	-20	-28	85		
			Safety Visit	13-NOV-2017	07:27	-2	-10	87		
			Admission	23-NOV-2017	09:17	8	1	81		
			Admission 3	26-NOV-2017	10:02	11	4	83		
			Safety Monitoring	30-NOV-2017	06:57	15	8	84		
			Safety Monitoring	01-DEC-2017	07:14	16	9	83		
			Safety Monitoring	03-DEC-2017	08:50	18	11	84		
			Safety Monitoring	11-DEC-2017	08:54	26	19	85		
			End of Study	14-DEC-2017	08:37	29	22	84		
			Unscheduled	18-DEC-2017	07:48	33	26	84		
			Unscheduled	21-DEC-2017	07:53	36	29	85		
			Unscheduled	08-JAN-2018	08:06	54	47	84		

Notes: Day relative to inoculum is the number of days relative to day of administration of the inoculum, where the day of administration = 0.
The inoculum was administered on Study Day 0 for K13-infected subjects and Study Day 1 for 3D7-infected subjects.
Day relative to IP is the number of days relative to day of administration of Artesunate, where the day of dose = 1.
Rando = Randomisation; IP = Artesunate; H = High; L = Low; NCS = Not Clinically Significant; CS = Clinically Significant.

Listing 16.2.8.1a
Individual Haematology Results

Rando Number	Challenge Agent	Parameter (unit) [Normal Range]	Visit	Date of Assessment	Time of Assessment	Day Relative to Inoculum	Day Relative to IP	Result	Range Indicator	Clinical Assessment
R108	3D7	Erythrocytes (10^12/L) [4.5 – 6.5]	Screening	26-OCT-2017	09:30	-20	-28	5.7		
			Safety Visit	13-NOV-2017	07:27	-2	-10	5.6		
			Admission	23-NOV-2017	09:17	8	1	5.5		
			Admission 3	26-NOV-2017	10:02	11	4	5.1		
			Safety Monitoring	30-NOV-2017	06:57	15	8	5.0		
			Safety Monitoring	01-DEC-2017	07:14	16	9	5.0		
			Safety Monitoring	03-DEC-2017	08:50	18	11	5.1		
			Safety Monitoring	11-DEC-2017	08:54	26	19	5.0		
			End of Study	14-DEC-2017	08:37	29	22	5.0		
			Unscheduled	18-DEC-2017	07:48	33	26	5.5		
			Unscheduled	21-DEC-2017	07:53	36	29	5.2		
			Unscheduled	08-JAN-2018	08:06	54	47	5.4		

Notes: Day relative to inoculum is the number of days relative to day of administration of the inoculum, where the day of administration = 0.
The inoculum was administered on Study Day 0 for K13-infected subjects and Study Day 1 for 3D7-infected subjects.
Day relative to IP is the number of days relative to day of administration of Artesunate, where the day of dose = 1.
Rando = Randomisation; IP = Artesunate; H = High; L = Low; NCS = Not Clinically Significant; CS = Clinically Significant.

Listing 16.2.8.1a
Individual Haematology Results

Rando Number	Challenge Agent	Parameter (unit) [Normal Range]	Visit	Date of Assessment	Time of Assessment	Day Relative to Inoculum	Day Relative to IP	Result	Range Indicator	Clinical Assessment
R108	3D7	Hematocrit [0.4 – 0.54]	Screening	26-OCT-2017	09:30	-20	-28	0.48		
			Safety Visit	13-NOV-2017	07:27	-2	-10	0.49		
			Admission	23-NOV-2017	09:17	8	1	0.44		
			Admission 3	26-NOV-2017	10:02	11	4	0.43		
			Safety Monitoring	30-NOV-2017	06:57	15	8	0.43		
			Safety Monitoring	01-DEC-2017	07:14	16	9	0.42		
			Safety Monitoring	03-DEC-2017	08:50	18	11	0.43		
			Safety Monitoring	11-DEC-2017	08:54	26	19	0.42		
			End of Study	14-DEC-2017	08:37	29	22	0.42		
			Unscheduled	18-DEC-2017	07:48	33	26	0.46		
			Unscheduled	21-DEC-2017	07:53	36	29	0.44		
			Unscheduled	08-JAN-2018	08:06	54	47	0.46		

Notes: Day relative to inoculum is the number of days relative to day of administration of the inoculum, where the day of administration = 0.
The inoculum was administered on Study Day 0 for K13-infected subjects and Study Day 1 for 3D7-infected subjects.
Day relative to IP is the number of days relative to day of administration of Artesunate, where the day of dose = 1.
Rando = Randomisation; IP = Artesunate; H = High; L = Low; NCS = Not Clinically Significant; CS = Clinically Significant.

Listing 16.2.8.1a
Individual Haematology Results

Rando Number	Challenge Agent	Parameter (unit) [Normal Range]	Visit	Date of Assessment	Time of Assessment	Day Relative to Inoculum	Day Relative to IP	Result	Range Indicator	Clinical Assessment
R108	3D7	Hemoglobin (g/L) [135 – 175]	Screening	26-OCT-2017	09:30	-20	-28	160		
			Safety Visit	13-NOV-2017	07:27	-2	-10	161		
			Admission	23-NOV-2017	09:17	8	1	150		
			Admission 3	26-NOV-2017	10:02	11	4	145		
			Safety Monitoring	30-NOV-2017	06:57	15	8	141		
			Safety Monitoring	01-DEC-2017	07:14	16	9	141		
			Safety Monitoring	03-DEC-2017	08:50	18	11	145		
			Safety Monitoring	11-DEC-2017	08:54	26	19	143		
			End of Study	14-DEC-2017	08:37	29	22	142		
			Unscheduled	18-DEC-2017	07:48	33	26	151		
			Unscheduled	21-DEC-2017	07:53	36	29	145		
			Unscheduled	08-JAN-2018	08:06	54	47	149		

Notes: Day relative to inoculum is the number of days relative to day of administration of the inoculum, where the day of administration = 0.
The inoculum was administered on Study Day 0 for K13-infected subjects and Study Day 1 for 3D7-infected subjects.
Day relative to IP is the number of days relative to day of administration of Artesunate, where the day of dose = 1.
Rando = Randomisation; IP = Artesunate; H = High; L = Low; NCS = Not Clinically Significant; CS = Clinically Significant.

Listing 16.2.8.1a
Individual Haematology Results

Rando Number	Challenge Agent	Parameter (unit) [Normal Range]	Visit	Date of Assessment	Time of Assessment	Day Relative to Inoculum	Day Relative to IP	Result	Range Indicator	Clinical Assessment
R108	3D7	Leukocytes (10^9/L) [3.5 – 10]	Screening	26-OCT-2017	09:30	-20	-28	8.3		
			Safety Visit	13-NOV-2017	07:27	-2	-10	7.8		
			Admission	23-NOV-2017	09:17	8	1	4.5		
			Admission 3	26-NOV-2017	10:02	11	4	3.9		
			Safety Monitoring	30-NOV-2017	06:57	15	8	8.4		
			Safety Monitoring	01-DEC-2017	07:14	16	9	6.3		
			Safety Monitoring	03-DEC-2017	08:50	18	11	5.4		
			Safety Monitoring	11-DEC-2017	08:54	26	19	6.1		
			End of Study	14-DEC-2017	08:37	29	22	9.2		
			Unscheduled	18-DEC-2017	07:48	33	26	8.1		
			Unscheduled	21-DEC-2017	07:53	36	29	9.3		
			Unscheduled	08-JAN-2018	08:06	54	47	7.8		

Notes: Day relative to inoculum is the number of days relative to day of administration of the inoculum, where the day of administration = 0.
The inoculum was administered on Study Day 0 for K13-infected subjects and Study Day 1 for 3D7-infected subjects.
Day relative to IP is the number of days relative to day of administration of Artesunate, where the day of dose = 1.
Rando = Randomisation; IP = Artesunate; H = High; L = Low; NCS = Not Clinically Significant; CS = Clinically Significant.

Listing 16.2.8.1a
Individual Haematology Results

Rando Number	Challenge Agent	Parameter (unit) [Normal Range]	Visit	Date of Assessment	Time of Assessment	Day Relative to Inoculum	Day Relative to IP	Result	Range Indicator	Clinical Assessment
R108	3D7	Lymphocytes (10^9/L) [1 – 4]	Screening	26-OCT-2017	09:30	-20	-28	2.41		
			Safety Visit	13-NOV-2017	07:27	-2	-10	3.16		
			Admission	23-NOV-2017	09:17	8	1	0.97	L	NCS
			Admission 3	26-NOV-2017	10:02	11	4	1.30		
			Safety Monitoring	30-NOV-2017	06:57	15	8	3.04		
			Safety Monitoring	01-DEC-2017	07:14	16	9	1.74		
			Safety Monitoring	03-DEC-2017	08:50	18	11	2.10		
			Safety Monitoring	11-DEC-2017	08:54	26	19	2.17		
			End of Study	14-DEC-2017	08:37	29	22	1.92		
			Unscheduled	18-DEC-2017	07:48	33	26	2.86		
			Unscheduled	21-DEC-2017	07:53	36	29	3.18		
			Unscheduled	08-JAN-2018	08:06	54	47	2.95		

Notes: Day relative to inoculum is the number of days relative to day of administration of the inoculum, where the day of administration = 0.
The inoculum was administered on Study Day 0 for K13-infected subjects and Study Day 1 for 3D7-infected subjects.
Day relative to IP is the number of days relative to day of administration of Artesunate, where the day of dose = 1.
Rando = Randomisation; IP = Artesunate; H = High; L = Low; NCS = Not Clinically Significant; CS = Clinically Significant.

Listing 16.2.8.1a
Individual Haematology Results

Rando Number	Challenge Agent	Parameter (unit) [Normal Range]	Visit	Date of Assessment	Time of Assessment	Day Relative to Inoculum	Day Relative to IP	Result	Range Indicator	Clinical Assessment
R108	3D7	Monocytes (10^9/L) [0 – 0.9]	Screening	26-OCT-2017	09:30	-20	-28	0.98	H	NCS
			Safety Visit	13-NOV-2017	07:27	-2	-10	0.82		
			Admission	23-NOV-2017	09:17	8	1	0.66		
			Admission 3	26-NOV-2017	10:02	11	4	0.73		
			Safety Monitoring	30-NOV-2017	06:57	15	8	0.82		
			Safety Monitoring	01-DEC-2017	07:14	16	9	0.65		
			Safety Monitoring	03-DEC-2017	08:50	18	11	0.77		
			Safety Monitoring	11-DEC-2017	08:54	26	19	0.58		
			End of Study	14-DEC-2017	08:37	29	22	0.96	H	NCS
			Unscheduled	18-DEC-2017	07:48	33	26	0.77		
			Unscheduled	21-DEC-2017	07:53	36	29	0.92	H	NCS
			Unscheduled	08-JAN-2018	08:06	54	47	0.84		

Notes: Day relative to inoculum is the number of days relative to day of administration of the inoculum, where the day of administration = 0.
The inoculum was administered on Study Day 0 for K13-infected subjects and Study Day 1 for 3D7-infected subjects.
Day relative to IP is the number of days relative to day of administration of Artesunate, where the day of dose = 1.
Rando = Randomisation; IP = Artesunate; H = High; L = Low; NCS = Not Clinically Significant; CS = Clinically Significant.

Listing 16.2.8.1a
Individual Haematology Results

Rando Number	Challenge Agent	Parameter (unit) [Normal Range]	Visit	Date of Assessment	Time of Assessment	Day Relative to Inoculum	Day Relative to IP	Result	Range Indicator	Clinical Assessment
R108	3D7	Neutrophils (10^9/L) [1.5 – 6.5]	Screening	26-OCT-2017	09:30	-20	-28	4.33		
			Safety Visit	13-NOV-2017	07:27	-2	-10	3.15		
			Admission	23-NOV-2017	09:17	8	1	2.54		
			Admission 3	26-NOV-2017	10:02	11	4	1.60		
			Safety Monitoring	30-NOV-2017	06:57	15	8	3.87		
			Safety Monitoring	01-DEC-2017	07:14	16	9	3.34		
			Safety Monitoring	03-DEC-2017	08:50	18	11	2.07		
			Safety Monitoring	11-DEC-2017	08:54	26	19	2.71		
			End of Study	14-DEC-2017	08:37	29	22	5.16		
			Unscheduled	18-DEC-2017	07:48	33	26	3.38		
			Unscheduled	21-DEC-2017	07:53	36	29	4.40		
			Unscheduled	08-JAN-2018	08:06	54	47	3.22		

Notes: Day relative to inoculum is the number of days relative to day of administration of the inoculum, where the day of administration = 0.
The inoculum was administered on Study Day 0 for K13-infected subjects and Study Day 1 for 3D7-infected subjects.
Day relative to IP is the number of days relative to day of administration of Artesunate, where the day of dose = 1.
Rando = Randomisation; IP = Artesunate; H = High; L = Low; NCS = Not Clinically Significant; CS = Clinically Significant.

Listing 16.2.8.1a
Individual Haematology Results

Rando Number	Challenge Agent	Parameter (unit) [Normal Range]	Visit	Date of Assessment	Time of Assessment	Day Relative to Inoculum	Day Relative to IP	Result	Range Indicator	Clinical Assessment
R108	3D7	Platelets (10^9/L) [150 – 400]	Screening	26-OCT-2017	09:30	-20	-28	285		
			Safety Visit	13-NOV-2017	07:27	-2	-10	261		
			Admission	23-NOV-2017	09:17	8	1	193		
			Admission 3	26-NOV-2017	10:02	11	4	165		
			Safety Monitoring	30-NOV-2017	06:57	15	8	252		
			Safety Monitoring	01-DEC-2017	07:14	16	9	225		
			Safety Monitoring	03-DEC-2017	08:50	18	11	256		
			Safety Monitoring	11-DEC-2017	08:54	26	19	265		
			End of Study	14-DEC-2017	08:37	29	22	253		
			Unscheduled	18-DEC-2017	07:48	33	26	271		
			Unscheduled	21-DEC-2017	07:53	36	29	275		
			Unscheduled	08-JAN-2018	08:06	54	47	289		
		Reticulocytes (10^9/L) [25 – 120]	Safety Visit	13-NOV-2017	07:27	-2	-10	82		
			End of Study	14-DEC-2017	08:37	29	22	115		

Notes: Day relative to inoculum is the number of days relative to day of administration of the inoculum, where the day of administration = 0.
The inoculum was administered on Study Day 0 for K13-infected subjects and Study Day 1 for 3D7-infected subjects.
Day relative to IP is the number of days relative to day of administration of Artesunate, where the day of dose = 1.
Rando = Randomisation; IP = Artesunate; H = High; L = Low; NCS = Not Clinically Significant; CS = Clinically Significant.

Listing 16.2.8.1a
Individual Haematology Results

Rando Number	Challenge Agent	Parameter (unit) [Normal Range]	Visit	Date of Assessment	Time of Assessment	Day Relative to Inoculum	Day Relative to IP	Result	Range Indicator	Clinical Assessment
R201	K13	Basophils (10^9/L) [0 – 0.15]	Screening	08-FEB-2018	12:01	-11	-20	0.02		
			Safety Visit	16-FEB-2018	08:12	-3	-12	0.03		
			Admission	28-FEB-2018	12:39	9	1	0.04		
			Safety Monitoring	02-MAR-2018	13:24	11	3	0.02		
			Admission 3	03-MAR-2018	13:27	12	4	0.02		
			Safety Monitoring	05-MAR-2018	08:54	14	6	0.02		
			Safety Monitoring	09-MAR-2018	09:59	18	10	0.02		
			Safety Monitoring	19-MAR-2018	08:19	28	20	0.04		
			End of Study	22-MAR-2018	08:31	31	23	0.04		
		Eosinophils (10^9/L) [0 – 0.6]	Screening	08-FEB-2018	12:01	-11	-20	0.00		
			Safety Visit	16-FEB-2018	08:12	-3	-12	0.00		
			Admission	28-FEB-2018	12:39	9	1	0.01		
			Safety Monitoring	02-MAR-2018	13:24	11	3	0.00		
			Admission 3	03-MAR-2018	13:27	12	4	0.00		
			Safety Monitoring	05-MAR-2018	08:54	14	6	0.00		
			Safety Monitoring	09-MAR-2018	09:59	18	10	0.00		
			Safety Monitoring	19-MAR-2018	08:19	28	20	0.22		
			End of Study	22-MAR-2018	08:31	31	23	0.01		

Notes: Day relative to inoculum is the number of days relative to day of administration of the inoculum, where the day of administration = 0.
The inoculum was administered on Study Day 0 for K13-infected subjects and Study Day 1 for 3D7-infected subjects.
Day relative to IP is the number of days relative to day of administration of Artesunate, where the day of dose = 1.
Rando = Randomisation; IP = Artesunate; H = High; L = Low; NCS = Not Clinically Significant; CS = Clinically Significant.

Listing 16.2.8.1a
Individual Haematology Results

Rando Number	Challenge Agent	Parameter (unit) [Normal Range]	Visit	Date of Assessment	Time of Assessment	Day Relative to Inoculum	Day Relative to IP	Result	Range Indicator	Clinical Assessment
R201	K13	Ery. Mean Corpuscular Volume (fL) [80 – 100]	Screening	08-FEB-2018	12:01	-11	-20	85		
			Safety Visit	16-FEB-2018	08:12	-3	-12	85		
			Admission	28-FEB-2018	12:39	9	1	87		
			Safety Monitoring	02-MAR-2018	13:24	11	3	86		
			Admission 3	03-MAR-2018	13:27	12	4	84		
			Safety Monitoring	05-MAR-2018	08:54	14	6	84		
			Safety Monitoring	09-MAR-2018	09:59	18	10	87		
			Safety Monitoring	19-MAR-2018	08:19	28	20	89		
			End of Study	22-MAR-2018	08:31	31	23	85		
		Erythrocytes (10^12/L) [3.9 – 5.6]	Screening	08-FEB-2018	12:01	-11	-20	4.6		
			Safety Visit	16-FEB-2018	08:12	-3	-12	4.4		
			Admission	28-FEB-2018	12:39	9	1	4.2		
			Safety Monitoring	02-MAR-2018	13:24	11	3	4.3		
			Admission 3	03-MAR-2018	13:27	12	4	4.3		
			Safety Monitoring	05-MAR-2018	08:54	14	6	3.8	L	NCS
			Safety Monitoring	09-MAR-2018	09:59	18	10	3.9		
			Safety Monitoring	19-MAR-2018	08:19	28	20	4.0		
			End of Study	22-MAR-2018	08:31	31	23	3.8	L	NCS

Notes: Day relative to inoculum is the number of days relative to day of administration of the inoculum, where the day of administration = 0.
The inoculum was administered on Study Day 0 for K13-infected subjects and Study Day 1 for 3D7-infected subjects.
Day relative to IP is the number of days relative to day of administration of Artesunate, where the day of dose = 1.
Rando = Randomisation; IP = Artesunate; H = High; L = Low; NCS = Not Clinically Significant; CS = Clinically Significant.

Listing 16.2.8.1a
Individual Haematology Results

Rando Number	Challenge Agent	Parameter (unit) [Normal Range]	Visit	Date of Assessment	Time of Assessment	Day Relative to Inoculum	Day Relative to IP	Result	Range Indicator	Clinical Assessment
R201	K13	Hematocrit [0.35 – 0.47]	Screening	08-FEB-2018	12:01	-11	-20	0.39		
			Safety Visit	16-FEB-2018	08:12	-3	-12	0.37		
			Admission	28-FEB-2018	12:39	9	1	0.36		
			Safety Monitoring	02-MAR-2018	13:24	11	3	0.37		
			Admission 3	03-MAR-2018	13:27	12	4	0.36		
			Safety Monitoring	05-MAR-2018	08:54	14	6	0.32	L	NCS
			Safety Monitoring	09-MAR-2018	09:59	18	10	0.34	L	NCS
			Safety Monitoring	19-MAR-2018	08:19	28	20	0.35		
			End of Study	22-MAR-2018	08:31	31	23	0.32	L	NCS
		Hemoglobin (g/L) [115 – 165]	Screening	08-FEB-2018	12:01	-11	-20	128		
			Safety Visit	16-FEB-2018	08:12	-3	-12	124		
			Admission	28-FEB-2018	12:39	9	1	117		
			Safety Monitoring	02-MAR-2018	13:24	11	3	122		
			Admission 3	03-MAR-2018	13:27	12	4	117		
			Safety Monitoring	05-MAR-2018	08:54	14	6	109	L	NCS
			Safety Monitoring	09-MAR-2018	09:59	18	10	116		
			Safety Monitoring	19-MAR-2018	08:19	28	20	112	L	NCS
			End of Study	22-MAR-2018	08:31	31	23	113	L	NCS

Notes: Day relative to inoculum is the number of days relative to day of administration of the inoculum, where the day of administration = 0.
The inoculum was administered on Study Day 0 for K13-infected subjects and Study Day 1 for 3D7-infected subjects.
Day relative to IP is the number of days relative to day of administration of Artesunate, where the day of dose = 1.
Rando = Randomisation; IP = Artesunate; H = High; L = Low; NCS = Not Clinically Significant; CS = Clinically Significant.

Listing 16.2.8.1a
Individual Haematology Results

Rando Number	Challenge Agent	Parameter (unit) [Normal Range]	Visit	Date of Assessment	Time of Assessment	Day Relative to Inoculum	Day Relative to IP	Result	Range Indicator	Clinical Assessment
R201	K13	Leukocytes (10^9/L) [3.5 – 12]	Screening	08-FEB-2018	12:01	-11	-20	8.8		
			Safety Visit	16-FEB-2018	08:12	-3	-12	7.2		
			Admission	28-FEB-2018	12:39	9	1	6.3		
			Safety Monitoring	02-MAR-2018	13:24	11	3	2.4	L	CS
			Admission 3	03-MAR-2018	13:27	12	4	3.4	L	NCS
			Safety Monitoring	05-MAR-2018	08:54	14	6	4.4		
			Safety Monitoring	09-MAR-2018	09:59	18	10	5.9		
			Safety Monitoring	19-MAR-2018	08:19	28	20	6.2		
			End of Study	22-MAR-2018	08:31	31	23	6.1		
		Lymphocytes (10^9/L) [1 – 4]	Screening	08-FEB-2018	12:01	-11	-20	2.62		
			Safety Visit	16-FEB-2018	08:12	-3	-12	2.57		
			Admission	28-FEB-2018	12:39	9	1	1.89		
			Safety Monitoring	02-MAR-2018	13:24	11	3	0.95	L	NCS
			Admission 3	03-MAR-2018	13:27	12	4	1.43		
			Safety Monitoring	05-MAR-2018	08:54	14	6	1.79		
			Safety Monitoring	09-MAR-2018	09:59	18	10	2.27		
			Safety Monitoring	19-MAR-2018	08:19	28	20	1.79		
			End of Study	22-MAR-2018	08:31	31	23	1.74		

Notes: Day relative to inoculum is the number of days relative to day of administration of the inoculum, where the day of administration = 0.
The inoculum was administered on Study Day 0 for K13-infected subjects and Study Day 1 for 3D7-infected subjects.
Day relative to IP is the number of days relative to day of administration of Artesunate, where the day of dose = 1.
Rando = Randomisation; IP = Artesunate; H = High; L = Low; NCS = Not Clinically Significant; CS = Clinically Significant.

Listing 16.2.8.1a
Individual Haematology Results

Rando Number	Challenge Agent	Parameter (unit) [Normal Range]	Visit	Date of Assessment	Time of Assessment	Day Relative to Inoculum	Day Relative to IP	Result	Range Indicator	Clinical Assessment
R201	K13	Monocytes (10^9/L) [0 – 0.9]	Screening	08-FEB-2018	12:01	-11	-20	0.66		
			Safety Visit	16-FEB-2018	08:12	-3	-12	0.56		
			Admission	28-FEB-2018	12:39	9	1	0.54		
			Safety Monitoring	02-MAR-2018	13:24	11	3	0.28		
			Admission 3	03-MAR-2018	13:27	12	4	0.57		
			Safety Monitoring	05-MAR-2018	08:54	14	6	0.54		
			Safety Monitoring	09-MAR-2018	09:59	18	10	0.39		
			Safety Monitoring	19-MAR-2018	08:19	28	20	0.50		
			End of Study	22-MAR-2018	08:31	31	23	0.52		
		Neutrophils (10^9/L) [1.5 – 8]	Screening	08-FEB-2018	12:01	-11	-20	5.51		
			Safety Visit	16-FEB-2018	08:12	-3	-12	4.04		
			Admission	28-FEB-2018	12:39	9	1	3.78		
			Safety Monitoring	02-MAR-2018	13:24	11	3	1.17	L	CS
			Admission 3	03-MAR-2018	13:27	12	4	1.38	L	CS
			Safety Monitoring	05-MAR-2018	08:54	14	6	2.07		
			Safety Monitoring	09-MAR-2018	09:59	18	10	3.22		
			Safety Monitoring	19-MAR-2018	08:19	28	20	3.60		
			End of Study	22-MAR-2018	08:31	31	23	3.78		

Notes: Day relative to inoculum is the number of days relative to day of administration of the inoculum, where the day of administration = 0.
The inoculum was administered on Study Day 0 for K13-infected subjects and Study Day 1 for 3D7-infected subjects.
Day relative to IP is the number of days relative to day of administration of Artesunate, where the day of dose = 1.
Rando = Randomisation; IP = Artesunate; H = High; L = Low; NCS = Not Clinically Significant; CS = Clinically Significant.

Listing 16.2.8.1a
Individual Haematology Results

Rando Number	Challenge Agent	Parameter (unit) [Normal Range]	Visit	Date of Assessment	Time of Assessment	Day Relative to Inoculum	Day Relative to IP	Result	Range Indicator	Clinical Assessment
R201	K13	Platelets (10^9/L) [150 – 400]	Screening	08-FEB-2018	12:01	-11	-20	248		
			Safety Visit	16-FEB-2018	08:12	-3	-12	260		
			Admission	28-FEB-2018	12:39	9	1	245		
			Safety Monitoring	02-MAR-2018	13:24	11	3	182		
			Admission 3	03-MAR-2018	13:27	12	4	199		
			Safety Monitoring	05-MAR-2018	08:54	14	6	186		
			Safety Monitoring	09-MAR-2018	09:59	18	10	257		
			Safety Monitoring	19-MAR-2018	08:19	28	20	272		
			End of Study	22-MAR-2018	08:31	31	23	254		
		Reticulocytes (10^9/L) [25 – 120]	Safety Visit	16-FEB-2018	08:12	-3	-12	76		
			End of Study	22-MAR-2018	08:31	31	23	86		

Notes: Day relative to inoculum is the number of days relative to day of administration of the inoculum, where the day of administration = 0.
The inoculum was administered on Study Day 0 for K13-infected subjects and Study Day 1 for 3D7-infected subjects.
Day relative to IP is the number of days relative to day of administration of Artesunate, where the day of dose = 1.
Rando = Randomisation; IP = Artesunate; H = High; L = Low; NCS = Not Clinically Significant; CS = Clinically Significant.

Listing 16.2.8.1a
Individual Haematology Results

Rando Number	Challenge Agent	Parameter (unit) [Normal Range]	Visit	Date of Assessment	Time of Assessment	Day Relative to Inoculum	Day Relative to IP	Result	Range Indicator	Clinical Assessment
R202	K13	Basophils (10^9/L) [0 – 0.15]	Screening	13-FEB-2018	11:20	-6	-15	0.04		
			Safety Visit	16-FEB-2018	08:57	-3	-12	0.06		
			Admission	28-FEB-2018	12:05	9	1	0.04		
			Safety Monitoring	02-MAR-2018	13:03	11	3	0.05		
			Admission 3	03-MAR-2018	13:05	12	4	0.03		
			Safety Monitoring	05-MAR-2018	09:59	14	6	0.03		
			Safety Monitoring	09-MAR-2018	09:32	18	10	0.05		
			Safety Monitoring	12-MAR-2018	09:51	21	13	0.06		
			Safety Monitoring	16-MAR-2018	09:35	25	17	0.07		
			End of Study	22-MAR-2018	10:00	31	23	0.06		
			Unscheduled	03-APR-2018	09:10	43	35	0.06		

Notes: Day relative to inoculum is the number of days relative to day of administration of the inoculum, where the day of administration = 0.
The inoculum was administered on Study Day 0 for K13-infected subjects and Study Day 1 for 3D7-infected subjects.
Day relative to IP is the number of days relative to day of administration of Artesunate, where the day of dose = 1.
Rando = Randomisation; IP = Artesunate; H = High; L = Low; NCS = Not Clinically Significant; CS = Clinically Significant.

Listing 16.2.8.1a
Individual Haematology Results

Rando Number	Challenge Agent	Parameter (unit) [Normal Range]	Visit	Date of Assessment	Time of Assessment	Day Relative to Inoculum	Day Relative to IP	Result	Range Indicator	Clinical Assessment
R202	K13	Eosinophils (10^9/L) [0 – 0.6]	Screening	13-FEB-2018	11:20	-6	-15	0.16		
			Safety Visit	16-FEB-2018	08:57	-3	-12	0.18		
			Admission	28-FEB-2018	12:05	9	1	0.12		
			Safety Monitoring	02-MAR-2018	13:03	11	3	0.11		
			Admission 3	03-MAR-2018	13:05	12	4	0.03		
			Safety Monitoring	05-MAR-2018	09:59	14	6	0.13		
			Safety Monitoring	09-MAR-2018	09:32	18	10	0.15		
			Safety Monitoring	12-MAR-2018	09:51	21	13	0.20		
			Safety Monitoring	16-MAR-2018	09:35	25	17	0.19		
			End of Study	22-MAR-2018	10:00	31	23	0.21		
			Unscheduled	03-APR-2018	09:10	43	35	0.25		

Notes: Day relative to inoculum is the number of days relative to day of administration of the inoculum, where the day of administration = 0.
The inoculum was administered on Study Day 0 for K13-infected subjects and Study Day 1 for 3D7-infected subjects.
Day relative to IP is the number of days relative to day of administration of Artesunate, where the day of dose = 1.
Rando = Randomisation; IP = Artesunate; H = High; L = Low; NCS = Not Clinically Significant; CS = Clinically Significant.

Listing 16.2.8.1a
Individual Haematology Results

Rando Number	Challenge Agent	Parameter (unit) [Normal Range]	Visit	Date of Assessment	Time of Assessment	Day Relative to Inoculum	Day Relative to IP	Result	Range Indicator	Clinical Assessment
R202	K13	Ery. Mean Corpuscular Volume (fL) [80 – 100]	Screening	13-FEB-2018	11:20	-6	-15	90		
			Safety Visit	16-FEB-2018	08:57	-3	-12	91		
			Admission	28-FEB-2018	12:05	9	1	92		
			Safety Monitoring	02-MAR-2018	13:03	11	3	92		
			Admission 3	03-MAR-2018	13:05	12	4	91		
			Safety Monitoring	05-MAR-2018	09:59	14	6	89		
			Safety Monitoring	09-MAR-2018	09:32	18	10	90		
			Safety Monitoring	12-MAR-2018	09:51	21	13	91		
			Safety Monitoring	16-MAR-2018	09:35	25	17	89		
			End of Study	22-MAR-2018	10:00	31	23	89		
			Unscheduled	03-APR-2018	09:10	43	35	90		

Notes: Day relative to inoculum is the number of days relative to day of administration of the inoculum, where the day of administration = 0.
The inoculum was administered on Study Day 0 for K13-infected subjects and Study Day 1 for 3D7-infected subjects.
Day relative to IP is the number of days relative to day of administration of Artesunate, where the day of dose = 1.
Rando = Randomisation; IP = Artesunate; H = High; L = Low; NCS = Not Clinically Significant; CS = Clinically Significant.

Listing 16.2.8.1a
Individual Haematology Results

Rando Number	Challenge Agent	Parameter (unit) [Normal Range]	Visit	Date of Assessment	Time of Assessment	Day Relative to Inoculum	Day Relative to IP	Result	Range Indicator	Clinical Assessment
R202	K13	Erythrocytes (10^12/L) [4.5 – 6.5]	Screening	13-FEB-2018	11:20	-6	-15	4.7		
			Safety Visit	16-FEB-2018	08:57	-3	-12	4.4	L	NCS
			Admission	28-FEB-2018	12:05	9	1	4.2	L	NCS
			Safety Monitoring	02-MAR-2018	13:03	11	3	4.6		
			Admission 3	03-MAR-2018	13:05	12	4	4.7		
			Safety Monitoring	05-MAR-2018	09:59	14	6	4.5		
			Safety Monitoring	09-MAR-2018	09:32	18	10	4.3	L	NCS
			Safety Monitoring	12-MAR-2018	09:51	21	13	4.2	L	NCS
			Safety Monitoring	16-MAR-2018	09:35	25	17	4.2	L	NCS
			End of Study	22-MAR-2018	10:00	31	23	4.1	L	NCS
			Unscheduled	03-APR-2018	09:10	43	35	4.5		

Notes: Day relative to inoculum is the number of days relative to day of administration of the inoculum, where the day of administration = 0.
The inoculum was administered on Study Day 0 for K13-infected subjects and Study Day 1 for 3D7-infected subjects.
Day relative to IP is the number of days relative to day of administration of Artesunate, where the day of dose = 1.
Rando = Randomisation; IP = Artesunate; H = High; L = Low; NCS = Not Clinically Significant; CS = Clinically Significant.

Listing 16.2.8.1a
Individual Haematology Results

Rando Number	Challenge Agent	Parameter (unit) [Normal Range]	Visit	Date of Assessment	Time of Assessment	Day Relative to Inoculum	Day Relative to IP	Result	Range Indicator	Clinical Assessment
R202	K13	Hematocrit [0.4 – 0.54]	Screening	13-FEB-2018	11:20	-6	-15	0.42		
			Safety Visit	16-FEB-2018	08:57	-3	-12	0.40		
			Unscheduled	17-FEB-2018	07:39	-2	-11	0.40		
			Admission	28-FEB-2018	12:05	9	1	0.39	L	NCS
			Safety Monitoring	02-MAR-2018	13:03	11	3	0.42		
			Admission 3	03-MAR-2018	13:05	12	4	0.43		
			Safety Monitoring	05-MAR-2018	09:59	14	6	0.40		
			Safety Monitoring	09-MAR-2018	09:32	18	10	0.39	L	NCS
			Safety Monitoring	12-MAR-2018	09:51	21	13	0.38	L	NCS
			Safety Monitoring	16-MAR-2018	09:35	25	17	0.37	L	NCS
			End of Study	22-MAR-2018	10:00	31	23	0.36	L	NCS
			Unscheduled	27-MAR-2018	09:00	36	28	0.37	L	NCS
			Unscheduled	03-APR-2018	09:10	43	35	0.40		

Notes: Day relative to inoculum is the number of days relative to day of administration of the inoculum, where the day of administration = 0.
The inoculum was administered on Study Day 0 for K13-infected subjects and Study Day 1 for 3D7-infected subjects.
Day relative to IP is the number of days relative to day of administration of Artesunate, where the day of dose = 1.
Rando = Randomisation; IP = Artesunate; H = High; L = Low; NCS = Not Clinically Significant; CS = Clinically Significant.

Listing 16.2.8.1a
Individual Haematology Results

Rando Number	Challenge Agent	Parameter (unit) [Normal Range]	Visit	Date of Assessment	Time of Assessment	Day Relative to Inoculum	Day Relative to IP	Result	Range Indicator	Clinical Assessment
R202	K13	Hemoglobin (g/L) [135 – 175]	Screening	13-FEB-2018	11:20	-6	-15	141		
			Safety Visit	16-FEB-2018	08:57	-3	-12	134	L	NCS
			Unscheduled	17-FEB-2018	07:39	-2	-11	135		
			Admission	28-FEB-2018	12:05	9	1	130	L	NCS
			Safety Monitoring	02-MAR-2018	13:03	11	3	140		
			Admission 3	03-MAR-2018	13:05	12	4	144		
			Safety Monitoring	05-MAR-2018	09:59	14	6	137		
			Safety Monitoring	09-MAR-2018	09:32	18	10	130	L	NCS
			Safety Monitoring	12-MAR-2018	09:51	21	13	130	L	NCS
			Safety Monitoring	16-MAR-2018	09:35	25	17	130	L	NCS
			End of Study	22-MAR-2018	10:00	31	23	127	L	NCS
			Unscheduled	27-MAR-2018	09:00	36	28	129	L	NCS
			Unscheduled	03-APR-2018	09:10	43	35	132	L	NCS

Notes: Day relative to inoculum is the number of days relative to day of administration of the inoculum, where the day of administration = 0.
The inoculum was administered on Study Day 0 for K13-infected subjects and Study Day 1 for 3D7-infected subjects.
Day relative to IP is the number of days relative to day of administration of Artesunate, where the day of dose = 1.
Rando = Randomisation; IP = Artesunate; H = High; L = Low; NCS = Not Clinically Significant; CS = Clinically Significant.

Listing 16.2.8.1a
Individual Haematology Results

Rando Number	Challenge Agent	Parameter (unit) [Normal Range]	Visit	Date of Assessment	Time of Assessment	Day Relative to Inoculum	Day Relative to IP	Result	Range Indicator	Clinical Assessment
R202	K13	Leukocytes (10^9/L) [3.5 – 10]	Screening	13-FEB-2018	11:20	-6	-15	5.5		
			Safety Visit	16-FEB-2018	08:57	-3	-12	6.2		
			Admission	28-FEB-2018	12:05	9	1	5.7		
			Safety Monitoring	02-MAR-2018	13:03	11	3	5.2		
			Admission 3	03-MAR-2018	13:05	12	4	4.4		
			Safety Monitoring	05-MAR-2018	09:59	14	6	4.3		
			Safety Monitoring	09-MAR-2018	09:32	18	10	6.1		
			Safety Monitoring	12-MAR-2018	09:51	21	13	6.5		
			Safety Monitoring	16-MAR-2018	09:35	25	17	7.6		
			End of Study	22-MAR-2018	10:00	31	23	6.2		
			Unscheduled	03-APR-2018	09:10	43	35	5.9		

Notes: Day relative to inoculum is the number of days relative to day of administration of the inoculum, where the day of administration = 0.
The inoculum was administered on Study Day 0 for K13-infected subjects and Study Day 1 for 3D7-infected subjects.
Day relative to IP is the number of days relative to day of administration of Artesunate, where the day of dose = 1.
Rando = Randomisation; IP = Artesunate; H = High; L = Low; NCS = Not Clinically Significant; CS = Clinically Significant.

Listing 16.2.8.1a
Individual Haematology Results

Rando Number	Challenge Agent	Parameter (unit) [Normal Range]	Visit	Date of Assessment	Time of Assessment	Day Relative to Inoculum	Day Relative to IP	Result	Range Indicator	Clinical Assessment
R202	K13	Lymphocytes (10^9/L) [1 – 4]	Screening	13-FEB-2018	11:20	-6	-15	1.80		
			Safety Visit	16-FEB-2018	08:57	-3	-12	2.19		
			Admission	28-FEB-2018	12:05	9	1	1.80		
			Safety Monitoring	02-MAR-2018	13:03	11	3	1.29		
			Admission 3	03-MAR-2018	13:05	12	4	1.01		
			Safety Monitoring	05-MAR-2018	09:59	14	6	1.69		
			Safety Monitoring	09-MAR-2018	09:32	18	10	2.31		
			Safety Monitoring	12-MAR-2018	09:51	21	13	2.54		
			Safety Monitoring	16-MAR-2018	09:35	25	17	2.31		
			End of Study	22-MAR-2018	10:00	31	23	2.23		
			Unscheduled	03-APR-2018	09:10	43	35	2.46		

Notes: Day relative to inoculum is the number of days relative to day of administration of the inoculum, where the day of administration = 0.
The inoculum was administered on Study Day 0 for K13-infected subjects and Study Day 1 for 3D7-infected subjects.
Day relative to IP is the number of days relative to day of administration of Artesunate, where the day of dose = 1.
Rando = Randomisation; IP = Artesunate; H = High; L = Low; NCS = Not Clinically Significant; CS = Clinically Significant.

Listing 16.2.8.1a
Individual Haematology Results

Rando Number	Challenge Agent	Parameter (unit) [Normal Range]	Visit	Date of Assessment	Time of Assessment	Day Relative to Inoculum	Day Relative to IP	Result	Range Indicator	Clinical Assessment
R202	K13	Monocytes (10^9/L) [0 – 0.9]	Screening	13-FEB-2018	11:20	-6	-15	0.40		
			Safety Visit	16-FEB-2018	08:57	-3	-12	0.51		
			Admission	28-FEB-2018	12:05	9	1	0.51		
			Safety Monitoring	02-MAR-2018	13:03	11	3	0.50		
			Admission 3	03-MAR-2018	13:05	12	4	0.37		
			Safety Monitoring	05-MAR-2018	09:59	14	6	0.49		
			Safety Monitoring	09-MAR-2018	09:32	18	10	0.39		
			Safety Monitoring	12-MAR-2018	09:51	21	13	0.40		
			Safety Monitoring	16-MAR-2018	09:35	25	17	0.64		
			End of Study	22-MAR-2018	10:00	31	23	0.44		
			Unscheduled	03-APR-2018	09:10	43	35	0.43		

Notes: Day relative to inoculum is the number of days relative to day of administration of the inoculum, where the day of administration = 0.
The inoculum was administered on Study Day 0 for K13-infected subjects and Study Day 1 for 3D7-infected subjects.
Day relative to IP is the number of days relative to day of administration of Artesunate, where the day of dose = 1.
Rando = Randomisation; IP = Artesunate; H = High; L = Low; NCS = Not Clinically Significant; CS = Clinically Significant.

Listing 16.2.8.1a
Individual Haematology Results

Rando Number	Challenge Agent	Parameter (unit) [Normal Range]	Visit	Date of Assessment	Time of Assessment	Day Relative to Inoculum	Day Relative to IP	Result	Range Indicator	Clinical Assessment
R202	K13	Neutrophils (10^9/L) [1.5 – 6.5]	Screening	13-FEB-2018	11:20	-6	-15	3.11		
			Safety Visit	16-FEB-2018	08:57	-3	-12	3.26		
			Admission	28-FEB-2018	12:05	9	1	3.27		
			Safety Monitoring	02-MAR-2018	13:03	11	3	3.29		
			Admission 3	03-MAR-2018	13:05	12	4	2.98		
			Safety Monitoring	05-MAR-2018	09:59	14	6	1.98		
			Safety Monitoring	09-MAR-2018	09:32	18	10	3.18		
			Safety Monitoring	12-MAR-2018	09:51	21	13	3.28		
			Safety Monitoring	16-MAR-2018	09:35	25	17	4.35		
			End of Study	22-MAR-2018	10:00	31	23	3.26		
			Unscheduled	03-APR-2018	09:10	43	35	2.66		

Notes: Day relative to inoculum is the number of days relative to day of administration of the inoculum, where the day of administration = 0.
The inoculum was administered on Study Day 0 for K13-infected subjects and Study Day 1 for 3D7-infected subjects.
Day relative to IP is the number of days relative to day of administration of Artesunate, where the day of dose = 1.
Rando = Randomisation; IP = Artesunate; H = High; L = Low; NCS = Not Clinically Significant; CS = Clinically Significant.

Listing 16.2.8.1a
Individual Haematology Results

Rando Number	Challenge Agent	Parameter (unit) [Normal Range]	Visit	Date of Assessment	Time of Assessment	Day Relative to Inoculum	Day Relative to IP	Result	Range Indicator	Clinical Assessment
R202	K13	Platelets (10^9/L) [150 – 400]	Screening	13-FEB-2018	11:20	-6	-15	256		
			Safety Visit	16-FEB-2018	08:57	-3	-12	235		
			Admission	28-FEB-2018	12:05	9	1	222		
			Safety Monitoring	02-MAR-2018	13:03	11	3	201		
			Admission 3	03-MAR-2018	13:05	12	4	181		
			Safety Monitoring	05-MAR-2018	09:59	14	6	135	L	NCS
			Safety Monitoring	09-MAR-2018	09:32	18	10	236		
			Safety Monitoring	12-MAR-2018	09:51	21	13	279		
			Safety Monitoring	16-MAR-2018	09:35	25	17	283		
			End of Study	22-MAR-2018	10:00	31	23	248		
			Unscheduled	03-APR-2018	09:10	43	35	221		
		Reticulocytes (10^9/L) [25 – 120]	Safety Visit	16-FEB-2018	08:57	-3	-12	59		
			End of Study	22-MAR-2018	10:00	31	23	66		

Notes: Day relative to inoculum is the number of days relative to day of administration of the inoculum, where the day of administration = 0.
The inoculum was administered on Study Day 0 for K13-infected subjects and Study Day 1 for 3D7-infected subjects.
Day relative to IP is the number of days relative to day of administration of Artesunate, where the day of dose = 1.
Rando = Randomisation; IP = Artesunate; H = High; L = Low; NCS = Not Clinically Significant; CS = Clinically Significant.

Listing 16.2.8.1a
Individual Haematology Results

Rando Number	Challenge Agent	Parameter (unit) [Normal Range]	Visit	Date of Assessment	Time of Assessment	Day Relative to Inoculum	Day Relative to IP	Result	Range Indicator	Clinical Assessment
R203	K13	Basophils (10^9/L) [0 – 0.15]	Screening	13-FEB-2018	10:18	-6	-15	0.08		
			Safety Visit	16-FEB-2018	08:22	-3	-12	0.09		
			Admission	28-FEB-2018	11:14	9	1	0.06		
			Safety Monitoring	02-MAR-2018	11:49	11	3	0.03		
			Admission 3	03-MAR-2018	11:50	12	4	0.03		
			Safety Monitoring	09-MAR-2018	10:14	18	10	0.06		
			Safety Monitoring	10-MAR-2018	08:37	19	11	0.08		
			Safety Monitoring	13-MAR-2018	09:41	22	14	0.09		
			End of Study	22-MAR-2018	10:14	31	23	0.11		
			Unscheduled	28-MAR-2018	09:53	37	29	0.07		
			Unscheduled	02-APR-2018	09:18	42	34	0.08		
			Unscheduled	14-APR-2018	10:00	54	46	0.07		

Notes: Day relative to inoculum is the number of days relative to day of administration of the inoculum, where the day of administration = 0.
The inoculum was administered on Study Day 0 for K13-infected subjects and Study Day 1 for 3D7-infected subjects.
Day relative to IP is the number of days relative to day of administration of Artesunate, where the day of dose = 1.
Rando = Randomisation; IP = Artesunate; H = High; L = Low; NCS = Not Clinically Significant; CS = Clinically Significant.

Listing 16.2.8.1a
Individual Haematology Results

Rando Number	Challenge Agent	Parameter (unit) [Normal Range]	Visit	Date of Assessment	Time of Assessment	Day Relative to Inoculum	Day Relative to IP	Result	Range Indicator	Clinical Assessment
R203	K13	Eosinophils (10^9/L) [0 – 0.6]	Screening	13-FEB-2018	10:18	-6	-15	0.28		
			Safety Visit	16-FEB-2018	08:22	-3	-12	0.32		
			Admission	28-FEB-2018	11:14	9	1	0.33		
			Safety Monitoring	02-MAR-2018	11:49	11	3	0.04		
			Admission 3	03-MAR-2018	11:50	12	4	0.01		
			Safety Monitoring	05-MAR-2018	09:40	14	6	0.04		
			Safety Monitoring	09-MAR-2018	10:14	18	10	0.12		
			Safety Monitoring	10-MAR-2018	08:37	19	11	0.28		
			Safety Monitoring	13-MAR-2018	09:41	22	14	0.37		
			End of Study	22-MAR-2018	10:14	31	23	0.61	H	NCS
			Unscheduled	28-MAR-2018	09:53	37	29	0.50		
			Unscheduled	02-APR-2018	09:18	42	34	0.69	H	CS
			Unscheduled	14-APR-2018	10:00	54	46	0.30		

Notes: Day relative to inoculum is the number of days relative to day of administration of the inoculum, where the day of administration = 0.
The inoculum was administered on Study Day 0 for K13-infected subjects and Study Day 1 for 3D7-infected subjects.
Day relative to IP is the number of days relative to day of administration of Artesunate, where the day of dose = 1.
Rando = Randomisation; IP = Artesunate; H = High; L = Low; NCS = Not Clinically Significant; CS = Clinically Significant.

Listing 16.2.8.1a
Individual Haematology Results

Rando Number	Challenge Agent	Parameter (unit) [Normal Range]	Visit	Date of Assessment	Time of Assessment	Day Relative to Inoculum	Day Relative to IP	Result	Range Indicator	Clinical Assessment
R203	K13	Ery. Mean Corpuscular Volume (fL) [80 – 100]	Screening	13-FEB-2018	10:18	-6	-15	82		
			Safety Visit	16-FEB-2018	08:22	-3	-12	81		
			Admission	28-FEB-2018	11:14	9	1	83		
			Safety Monitoring	02-MAR-2018	11:49	11	3	81		
			Admission 3	03-MAR-2018	11:50	12	4	78	L	NCS
			Safety Monitoring	05-MAR-2018	09:40	14	6	79	L	NCS
			Safety Monitoring	09-MAR-2018	10:14	18	10	79	L	NCS
			Safety Monitoring	10-MAR-2018	08:37	19	11	79	L	NCS
			Safety Monitoring	13-MAR-2018	09:41	22	14	80		
			End of Study	22-MAR-2018	10:14	31	23	80		
			Unscheduled	28-MAR-2018	09:53	37	29	78	L	NCS
			Unscheduled	02-APR-2018	09:18	42	34	80		
			Unscheduled	14-APR-2018	10:00	54	46	79	L	NCS

Notes: Day relative to inoculum is the number of days relative to day of administration of the inoculum, where the day of administration = 0.
The inoculum was administered on Study Day 0 for K13-infected subjects and Study Day 1 for 3D7-infected subjects.
Day relative to IP is the number of days relative to day of administration of Artesunate, where the day of dose = 1.
Rando = Randomisation; IP = Artesunate; H = High; L = Low; NCS = Not Clinically Significant; CS = Clinically Significant.

Listing 16.2.8.1a
Individual Haematology Results

Rando Number	Challenge Agent	Parameter (unit) [Normal Range]	Visit	Date of Assessment	Time of Assessment	Day Relative to Inoculum	Day Relative to IP	Result	Range Indicator	Clinical Assessment
R203	K13	Erythrocytes (10^12/L) [3.9 – 5.6]	Screening	13-FEB-2018	10:18	-6	-15	5.1		
			Safety Visit	16-FEB-2018	08:22	-3	-12	5.0		
			Admission	28-FEB-2018	11:14	9	1	4.5		
			Safety Monitoring	02-MAR-2018	11:49	11	3	4.7		
			Admission 3	03-MAR-2018	11:50	12	4	4.8		
			Safety Monitoring	05-MAR-2018	09:40	14	6	4.7		
			Safety Monitoring	09-MAR-2018	10:14	18	10	4.9		
			Safety Monitoring	10-MAR-2018	08:37	19	11	4.7		
			Safety Monitoring	13-MAR-2018	09:41	22	14	4.7		
			End of Study	22-MAR-2018	10:14	31	23	4.8		
			Unscheduled	28-MAR-2018	09:53	37	29	4.4		
			Unscheduled	02-APR-2018	09:18	42	34	4.8		
			Unscheduled	14-APR-2018	10:00	54	46	4.5		

Notes: Day relative to inoculum is the number of days relative to day of administration of the inoculum, where the day of administration = 0.
The inoculum was administered on Study Day 0 for K13-infected subjects and Study Day 1 for 3D7-infected subjects.
Day relative to IP is the number of days relative to day of administration of Artesunate, where the day of dose = 1.
Rando = Randomisation; IP = Artesunate; H = High; L = Low; NCS = Not Clinically Significant; CS = Clinically Significant.

Listing 16.2.8.1a
Individual Haematology Results

Rando Number	Challenge Agent	Parameter (unit) [Normal Range]	Visit	Date of Assessment	Time of Assessment	Day Relative to Inoculum	Day Relative to IP	Result	Range Indicator	Clinical Assessment
R203	K13	Hematocrit [0.35 – 0.47]	Screening	13-FEB-2018	10:18	-6	-15	0.42		
			Safety Visit	16-FEB-2018	08:22	-3	-12	0.40		
			Admission	28-FEB-2018	11:14	9	1	0.37		
			Safety Monitoring	02-MAR-2018	11:49	11	3	0.38		
			Admission 3	03-MAR-2018	11:50	12	4	0.37		
			Safety Monitoring	05-MAR-2018	09:40	14	6	0.37		
			Safety Monitoring	09-MAR-2018	10:14	18	10	0.39		
			Safety Monitoring	10-MAR-2018	08:37	19	11	0.37		
			Safety Monitoring	13-MAR-2018	09:41	22	14	0.37		
			End of Study	22-MAR-2018	10:14	31	23	0.39		
			Unscheduled	28-MAR-2018	09:53	37	29	0.35		
			Unscheduled	02-APR-2018	09:18	42	34	0.38		
			Unscheduled	14-APR-2018	10:00	54	46	0.36		

Notes: Day relative to inoculum is the number of days relative to day of administration of the inoculum, where the day of administration = 0.
The inoculum was administered on Study Day 0 for K13-infected subjects and Study Day 1 for 3D7-infected subjects.
Day relative to IP is the number of days relative to day of administration of Artesunate, where the day of dose = 1.
Rando = Randomisation; IP = Artesunate; H = High; L = Low; NCS = Not Clinically Significant; CS = Clinically Significant.

Listing 16.2.8.1a
Individual Haematology Results

Rando Number	Challenge Agent	Parameter (unit) [Normal Range]	Visit	Date of Assessment	Time of Assessment	Day Relative to Inoculum	Day Relative to IP	Result	Range Indicator	Clinical Assessment
R203	K13	Hemoglobin (g/L) [115 – 165]	Screening	13-FEB-2018	10:18	-6	-15	135		
			Safety Visit	16-FEB-2018	08:22	-3	-12	132		
			Admission	28-FEB-2018	11:14	9	1	119		
			Safety Monitoring	02-MAR-2018	11:49	11	3	121		
			Admission 3	03-MAR-2018	11:50	12	4	127		
			Safety Monitoring	05-MAR-2018	09:40	14	6	123		
			Safety Monitoring	09-MAR-2018	10:14	18	10	123		
			Safety Monitoring	10-MAR-2018	08:37	19	11	120		
			Safety Monitoring	13-MAR-2018	09:41	22	14	123		
			End of Study	22-MAR-2018	10:14	31	23	127		
			Unscheduled	28-MAR-2018	09:53	37	29	114	L	NCS
			Unscheduled	02-APR-2018	09:18	42	34	120		
			Unscheduled	14-APR-2018	10:00	54	46	116		

Notes: Day relative to inoculum is the number of days relative to day of administration of the inoculum, where the day of administration = 0.
The inoculum was administered on Study Day 0 for K13-infected subjects and Study Day 1 for 3D7-infected subjects.
Day relative to IP is the number of days relative to day of administration of Artesunate, where the day of dose = 1.
Rando = Randomisation; IP = Artesunate; H = High; L = Low; NCS = Not Clinically Significant; CS = Clinically Significant.

Listing 16.2.8.1a
Individual Haematology Results

Rando Number	Challenge Agent	Parameter (unit) [Normal Range]	Visit	Date of Assessment	Time of Assessment	Day Relative to Inoculum	Day Relative to IP	Result	Range Indicator	Clinical Assessment
R203	K13	Leukocytes (10^9/L) [3.5 – 12]	Screening	13-FEB-2018	10:18	-6	-15	6.9		
			Safety Visit	16-FEB-2018	08:22	-3	-12	7.8		
			Admission	28-FEB-2018	11:14	9	1	6.3		
			Safety Monitoring	02-MAR-2018	11:49	11	3	3.8		
			Admission 3	03-MAR-2018	11:50	12	4	3.5		
			Safety Monitoring	05-MAR-2018	09:40	14	6	3.7		
			Safety Monitoring	09-MAR-2018	10:14	18	10	5.8		
			Safety Monitoring	10-MAR-2018	08:37	19	11	7.1		
			Safety Monitoring	13-MAR-2018	09:41	22	14	7.4		
			End of Study	22-MAR-2018	10:14	31	23	6.5		
			Unscheduled	28-MAR-2018	09:53	37	29	5.8		
			Unscheduled	02-APR-2018	09:18	42	34	7.6		
			Unscheduled	14-APR-2018	10:00	54	46	6.7		

Notes: Day relative to inoculum is the number of days relative to day of administration of the inoculum, where the day of administration = 0.
The inoculum was administered on Study Day 0 for K13-infected subjects and Study Day 1 for 3D7-infected subjects.
Day relative to IP is the number of days relative to day of administration of Artesunate, where the day of dose = 1.
Rando = Randomisation; IP = Artesunate; H = High; L = Low; NCS = Not Clinically Significant; CS = Clinically Significant.

Listing 16.2.8.1a
Individual Haematology Results

Rando Number	Challenge Agent	Parameter (unit) [Normal Range]	Visit	Date of Assessment	Time of Assessment	Day Relative to Inoculum	Day Relative to IP	Result	Range Indicator	Clinical Assessment
R203	K13	Lymphocytes (10^9/L) [1 – 4]	Screening	13-FEB-2018	10:18	-6	-15	2.65		
			Safety Visit	16-FEB-2018	08:22	-3	-12	3.13		
			Admission	28-FEB-2018	11:14	9	1	2.77		
			Safety Monitoring	02-MAR-2018	11:49	11	3	1.09		
			Admission 3	03-MAR-2018	11:50	12	4	0.92	L	NCS
			Safety Monitoring	05-MAR-2018	09:40	14	6	1.97		
			Safety Monitoring	09-MAR-2018	10:14	18	10	3.07		
			Safety Monitoring	10-MAR-2018	08:37	19	11	3.49		
			Safety Monitoring	13-MAR-2018	09:41	22	14	4.05	H	NCS
			End of Study	22-MAR-2018	10:14	31	23	2.81		
			Unscheduled	28-MAR-2018	09:53	37	29	2.70		
			Unscheduled	02-APR-2018	09:18	42	34	3.69		
			Unscheduled	14-APR-2018	10:00	54	46	3.47		
		Lymphocytes Atypical (10^9/L) [0 – 0.01]	Safety Monitoring	05-MAR-2018	09:40	14	6	0.18	H	NCS
			Safety Monitoring	09-MAR-2018	10:14	18	10	0.29	H	NCS

Notes: Day relative to inoculum is the number of days relative to day of administration of the inoculum, where the day of administration = 0.
The inoculum was administered on Study Day 0 for K13-infected subjects and Study Day 1 for 3D7-infected subjects.
Day relative to IP is the number of days relative to day of administration of Artesunate, where the day of dose = 1.
Rando = Randomisation; IP = Artesunate; H = High; L = Low; NCS = Not Clinically Significant; CS = Clinically Significant.

Listing 16.2.8.1a
Individual Haematology Results

Rando Number	Challenge Agent	Parameter (unit) [Normal Range]	Visit	Date of Assessment	Time of Assessment	Day Relative to Inoculum	Day Relative to IP	Result	Range Indicator	Clinical Assessment
R203	K13	Monocytes (10^9/L) [0 – 0.9]	Screening	13-FEB-2018	10:18	-6	-15	0.53		
			Safety Visit	16-FEB-2018	08:22	-3	-12	0.67		
			Admission	28-FEB-2018	11:14	9	1	0.52		
			Safety Monitoring	02-MAR-2018	11:49	11	3	0.36		
			Admission 3	03-MAR-2018	11:50	12	4	0.32		
			Safety Monitoring	05-MAR-2018	09:40	14	6	0.29		
			Safety Monitoring	09-MAR-2018	10:14	18	10	0.35		
			Safety Monitoring	10-MAR-2018	08:37	19	11	0.64		
			Safety Monitoring	13-MAR-2018	09:41	22	14	0.44		
			End of Study	22-MAR-2018	10:14	31	23	0.67		
			Unscheduled	28-MAR-2018	09:53	37	29	0.41		
			Unscheduled	02-APR-2018	09:18	42	34	0.50		
			Unscheduled	14-APR-2018	10:00	54	46	0.52		

Notes: Day relative to inoculum is the number of days relative to day of administration of the inoculum, where the day of administration = 0.
The inoculum was administered on Study Day 0 for K13-infected subjects and Study Day 1 for 3D7-infected subjects.
Day relative to IP is the number of days relative to day of administration of Artesunate, where the day of dose = 1.
Rando = Randomisation; IP = Artesunate; H = High; L = Low; NCS = Not Clinically Significant; CS = Clinically Significant.

Listing 16.2.8.1a
Individual Haematology Results

Rando Number	Challenge Agent	Parameter (unit) [Normal Range]	Visit	Date of Assessment	Time of Assessment	Day Relative to Inoculum	Day Relative to IP	Result	Range Indicator	Clinical Assessment
R203	K13	Neutrophils (10^9/L) [1.5 – 8]	Screening	13-FEB-2018	10:18	-6	-15	3.40		
			Safety Visit	16-FEB-2018	08:22	-3	-12	3.56		
			Admission	28-FEB-2018	11:14	9	1	2.65		
			Safety Monitoring	02-MAR-2018	11:49	11	3	2.23		
			Admission 3	03-MAR-2018	11:50	12	4	2.20		
			Safety Monitoring	05-MAR-2018	09:40	14	6	1.17	L	CS
			Safety Monitoring	09-MAR-2018	10:14	18	10	1.91		
			Safety Monitoring	10-MAR-2018	08:37	19	11	2.56		
			Safety Monitoring	13-MAR-2018	09:41	22	14	2.44		
			End of Study	22-MAR-2018	10:14	31	23	2.29		
			Unscheduled	28-MAR-2018	09:53	37	29	2.09		
			Unscheduled	02-APR-2018	09:18	42	34	2.66		
			Unscheduled	14-APR-2018	10:00	54	46	2.32		

Notes: Day relative to inoculum is the number of days relative to day of administration of the inoculum, where the day of administration = 0.
The inoculum was administered on Study Day 0 for K13-infected subjects and Study Day 1 for 3D7-infected subjects.
Day relative to IP is the number of days relative to day of administration of Artesunate, where the day of dose = 1.
Rando = Randomisation; IP = Artesunate; H = High; L = Low; NCS = Not Clinically Significant; CS = Clinically Significant.

Listing 16.2.8.1a
Individual Haematology Results

Rando Number	Challenge Agent	Parameter (unit) [Normal Range]	Visit	Date of Assessment	Time of Assessment	Day Relative to Inoculum	Day Relative to IP	Result	Range Indicator	Clinical Assessment
R203	K13	Platelets (10^9/L) [150 – 400]	Screening	13-FEB-2018	10:18	-6	-15	238		
			Safety Visit	16-FEB-2018	08:22	-3	-12	219		
			Admission	28-FEB-2018	11:14	9	1	220		
			Safety Monitoring	02-MAR-2018	11:49	11	3	179		
			Admission 3	03-MAR-2018	11:50	12	4	140	L	NCS
			Safety Monitoring	05-MAR-2018	09:40	14	6	127	L	NCS
			Safety Monitoring	09-MAR-2018	10:14	18	10	261		
			Safety Monitoring	10-MAR-2018	08:37	19	11	306		
			Safety Monitoring	13-MAR-2018	09:41	22	14	319		
			End of Study	22-MAR-2018	10:14	31	23	263		
			Unscheduled	28-MAR-2018	09:53	37	29	205		
			Unscheduled	02-APR-2018	09:18	42	34	216		
			Unscheduled	14-APR-2018	10:00	54	46	254		
		Reticulocytes (10^9/L) [25 – 120]	Safety Visit	16-FEB-2018	08:22	-3	-12	52		
			End of Study	22-MAR-2018	10:14	31	23	76		

Notes: Day relative to inoculum is the number of days relative to day of administration of the inoculum, where the day of administration = 0.
The inoculum was administered on Study Day 0 for K13-infected subjects and Study Day 1 for 3D7-infected subjects.
Day relative to IP is the number of days relative to day of administration of Artesunate, where the day of dose = 1.
Rando = Randomisation; IP = Artesunate; H = High; L = Low; NCS = Not Clinically Significant; CS = Clinically Significant.

Listing 16.2.8.1a
Individual Haematology Results

Rando Number	Challenge Agent	Parameter (unit) [Normal Range]	Visit	Date of Assessment	Time of Assessment	Day Relative to Inoculum	Day Relative to IP	Result	Range Indicator	Clinical Assessment
R204	K13	Basophils (10^9/L) [0 – 0.15]	Screening	05-FEB-2018	10:46	-14	-23	0.02		
			Safety Visit	16-FEB-2018	08:44	-3	-12	0.02		
			Admission	28-FEB-2018	12:29	9	1	0.02		
			Safety Monitoring	02-MAR-2018	13:17	11	3	0.02		
			Safety Monitoring	05-MAR-2018	08:37	14	6	0.02		
			Safety Monitoring	09-MAR-2018	09:00	18	10	0.03		
			Safety Monitoring	19-MAR-2018	08:24	28	20	0.02		
			End of Study	22-MAR-2018	08:43	31	23	0.03		
		Eosinophils (10^9/L) [0 – 0.6]	Screening	05-FEB-2018	10:46	-14	-23	0.09		
			Safety Visit	16-FEB-2018	08:44	-3	-12	0.16		
			Admission	28-FEB-2018	12:29	9	1	0.15		
			Safety Monitoring	02-MAR-2018	13:17	11	3	0.18		
			Admission 3	03-MAR-2018	13:21	12	4	0.15		
			Safety Monitoring	05-MAR-2018	08:37	14	6	0.11		
			Safety Monitoring	09-MAR-2018	09:00	18	10	0.16		
			Safety Monitoring	19-MAR-2018	08:24	28	20	0.17		
			End of Study	22-MAR-2018	08:43	31	23	0.25		

Notes: Day relative to inoculum is the number of days relative to day of administration of the inoculum, where the day of administration = 0.
The inoculum was administered on Study Day 0 for K13-infected subjects and Study Day 1 for 3D7-infected subjects.
Day relative to IP is the number of days relative to day of administration of Artesunate, where the day of dose = 1.
Rando = Randomisation; IP = Artesunate; H = High; L = Low; NCS = Not Clinically Significant; CS = Clinically Significant.

Listing 16.2.8.1a
Individual Haematology Results

Rando Number	Challenge Agent	Parameter (unit) [Normal Range]	Visit	Date of Assessment	Time of Assessment	Day Relative to Inoculum	Day Relative to IP	Result	Range Indicator	Clinical Assessment
R204	K13	Ery. Mean Corpuscular Volume (fL) [80 – 100]	Screening	05-FEB-2018	10:46	-14	-23	94		
			Safety Visit	16-FEB-2018	08:44	-3	-12	91		
			Admission	28-FEB-2018	12:29	9	1	93		
			Safety Monitoring	02-MAR-2018	13:17	11	3	94		
			Admission 3	03-MAR-2018	13:21	12	4	92		
			Safety Monitoring	05-MAR-2018	08:37	14	6	92		
			Safety Monitoring	09-MAR-2018	09:00	18	10	94		
			Safety Monitoring	19-MAR-2018	08:24	28	20	93		
			End of Study	22-MAR-2018	08:43	31	23	93		
		Erythrocytes (10^12/L) [3.9 – 5.6]	Screening	05-FEB-2018	10:46	-14	-23	4.3		
			Safety Visit	16-FEB-2018	08:44	-3	-12	4.1		
			Admission	28-FEB-2018	12:29	9	1	4.2		
			Safety Monitoring	02-MAR-2018	13:17	11	3	4.3		
			Admission 3	03-MAR-2018	13:21	12	4	4.0		
			Safety Monitoring	05-MAR-2018	08:37	14	6	3.8	L	NCS
			Safety Monitoring	09-MAR-2018	09:00	18	10	3.7	L	NCS
			Safety Monitoring	19-MAR-2018	08:24	28	20	3.9		
			End of Study	22-MAR-2018	08:43	31	23	3.7	L	NCS

Notes: Day relative to inoculum is the number of days relative to day of administration of the inoculum, where the day of administration = 0.
The inoculum was administered on Study Day 0 for K13-infected subjects and Study Day 1 for 3D7-infected subjects.
Day relative to IP is the number of days relative to day of administration of Artesunate, where the day of dose = 1.
Rando = Randomisation; IP = Artesunate; H = High; L = Low; NCS = Not Clinically Significant; CS = Clinically Significant.

Listing 16.2.8.1a
Individual Haematology Results

Rando Number	Challenge Agent	Parameter (unit) [Normal Range]	Visit	Date of Assessment	Time of Assessment	Day Relative to Inoculum	Day Relative to IP	Result	Range Indicator	Clinical Assessment
R204	K13	Hematocrit [0.35 – 0.47]	Screening	05-FEB-2018	10:46	-14	-23	0.40		
			Safety Visit	16-FEB-2018	08:44	-3	-12	0.37		
			Admission	28-FEB-2018	12:29	9	1	0.38		
			Safety Monitoring	02-MAR-2018	13:17	11	3	0.41		
			Admission 3	03-MAR-2018	13:21	12	4	0.37		
			Safety Monitoring	05-MAR-2018	08:37	14	6	0.35		
			Safety Monitoring	09-MAR-2018	09:00	18	10	0.35		
			Safety Monitoring	19-MAR-2018	08:24	28	20	0.37		
			End of Study	22-MAR-2018	08:43	31	23	0.34	L	NCS
		Hemoglobin (g/L) [115 – 165]	Screening	05-FEB-2018	10:46	-14	-23	136		
			Safety Visit	16-FEB-2018	08:44	-3	-12	130		
			Admission	28-FEB-2018	12:29	9	1	134		
			Safety Monitoring	02-MAR-2018	13:17	11	3	138		
			Admission 3	03-MAR-2018	13:21	12	4	130		
			Safety Monitoring	05-MAR-2018	08:37	14	6	122		
			Safety Monitoring	09-MAR-2018	09:00	18	10	118		
			Safety Monitoring	19-MAR-2018	08:24	28	20	128		
			End of Study	22-MAR-2018	08:43	31	23	122		

Notes: Day relative to inoculum is the number of days relative to day of administration of the inoculum, where the day of administration = 0.
The inoculum was administered on Study Day 0 for K13-infected subjects and Study Day 1 for 3D7-infected subjects.
Day relative to IP is the number of days relative to day of administration of Artesunate, where the day of dose = 1.
Rando = Randomisation; IP = Artesunate; H = High; L = Low; NCS = Not Clinically Significant; CS = Clinically Significant.

Listing 16.2.8.1a
Individual Haematology Results

Rando Number	Challenge Agent	Parameter (unit) [Normal Range]	Visit	Date of Assessment	Time of Assessment	Day Relative to Inoculum	Day Relative to IP	Result	Range Indicator	Clinical Assessment
R204	K13	Leukocytes (10^9/L) [3.5 – 12]	Screening	05-FEB-2018	10:46	-14	-23	5.7		
			Safety Visit	16-FEB-2018	08:44	-3	-12	4.2		
			Admission	28-FEB-2018	12:29	9	1	5.9		
			Safety Monitoring	02-MAR-2018	13:17	11	3	3.9		
			Admission 3	03-MAR-2018	13:21	12	4	2.9	L	CS
			Safety Monitoring	05-MAR-2018	08:37	14	6	2.5	L	CS
			Safety Monitoring	09-MAR-2018	09:00	18	10	4.1		
			Safety Monitoring	19-MAR-2018	08:24	28	20	4.1		
			End of Study	22-MAR-2018	08:43	31	23	4.2		
		Lymphocytes (10^9/L) [1 – 4]	Screening	05-FEB-2018	10:46	-14	-23	1.45		
			Safety Visit	16-FEB-2018	08:44	-3	-12	1.64		
			Admission	28-FEB-2018	12:29	9	1	1.70		
			Safety Monitoring	02-MAR-2018	13:17	11	3	1.21		
			Admission 3	03-MAR-2018	13:21	12	4	0.64	L	CS
			Safety Monitoring	05-MAR-2018	08:37	14	6	0.66	L	CS
			Safety Monitoring	09-MAR-2018	09:00	18	10	1.54		
			Safety Monitoring	19-MAR-2018	08:24	28	20	1.26		
			End of Study	22-MAR-2018	08:43	31	23	1.19		

Notes: Day relative to inoculum is the number of days relative to day of administration of the inoculum, where the day of administration = 0.
The inoculum was administered on Study Day 0 for K13-infected subjects and Study Day 1 for 3D7-infected subjects.
Day relative to IP is the number of days relative to day of administration of Artesunate, where the day of dose = 1.
Rando = Randomisation; IP = Artesunate; H = High; L = Low; NCS = Not Clinically Significant; CS = Clinically Significant.

Listing 16.2.8.1a
Individual Haematology Results

Rando Number	Challenge Agent	Parameter (unit) [Normal Range]	Visit	Date of Assessment	Time of Assessment	Day Relative to Inoculum	Day Relative to IP	Result	Range Indicator	Clinical Assessment
R204	K13	Monocytes (10^9/L) [0 – 0.9]	Screening	05-FEB-2018	10:46	-14	-23	0.29		
			Safety Visit	16-FEB-2018	08:44	-3	-12	0.35		
			Admission	28-FEB-2018	12:29	9	1	0.31		
			Safety Monitoring	02-MAR-2018	13:17	11	3	0.52		
			Admission 3	03-MAR-2018	13:21	12	4	0.23		
			Safety Monitoring	05-MAR-2018	08:37	14	6	0.39		
			Safety Monitoring	09-MAR-2018	09:00	18	10	0.28		
			Safety Monitoring	19-MAR-2018	08:24	28	20	0.32		
			End of Study	22-MAR-2018	08:43	31	23	0.35		
		Neutrophils (10^9/L) [1.5 – 8]	Screening	05-FEB-2018	10:46	-14	-23	3.82		
			Safety Visit	16-FEB-2018	08:44	-3	-12	2.02		
			Admission	28-FEB-2018	12:29	9	1	3.75		
			Safety Monitoring	02-MAR-2018	13:17	11	3	1.96		
			Admission 3	03-MAR-2018	13:21	12	4	1.88		
			Safety Monitoring	05-MAR-2018	08:37	14	6	1.32	L	CS
			Safety Monitoring	09-MAR-2018	09:00	18	10	2.05		
			Safety Monitoring	19-MAR-2018	08:24	28	20	2.35		
			End of Study	22-MAR-2018	08:43	31	23	2.33		

Notes: Day relative to inoculum is the number of days relative to day of administration of the inoculum, where the day of administration = 0.
The inoculum was administered on Study Day 0 for K13-infected subjects and Study Day 1 for 3D7-infected subjects.
Day relative to IP is the number of days relative to day of administration of Artesunate, where the day of dose = 1.
Rando = Randomisation; IP = Artesunate; H = High; L = Low; NCS = Not Clinically Significant; CS = Clinically Significant.

Listing 16.2.8.1a
Individual Haematology Results

Rando Number	Challenge Agent	Parameter (unit) [Normal Range]	Visit	Date of Assessment	Time of Assessment	Day Relative to Inoculum	Day Relative to IP	Result	Range Indicator	Clinical Assessment
R204	K13	Platelets (10^9/L) [150 – 400]	Screening	05-FEB-2018	10:46	-14	-23	183		
			Safety Visit	16-FEB-2018	08:44	-3	-12	184		
			Admission	28-FEB-2018	12:29	9	1	206		
			Safety Monitoring	02-MAR-2018	13:17	11	3	199		
			Admission 3	03-MAR-2018	13:21	12	4	159		
			Safety Monitoring	05-MAR-2018	08:37	14	6	122	L	NCS
			Safety Monitoring	09-MAR-2018	09:00	18	10	166		
			Safety Monitoring	19-MAR-2018	08:24	28	20	165		
			End of Study	22-MAR-2018	08:43	31	23	165		
		Reticulocytes (10^9/L) [25 – 120]	Safety Visit	16-FEB-2018	08:44	-3	-12	48		
			End of Study	22-MAR-2018	08:43	31	23	100		

Notes: Day relative to inoculum is the number of days relative to day of administration of the inoculum, where the day of administration = 0.
The inoculum was administered on Study Day 0 for K13-infected subjects and Study Day 1 for 3D7-infected subjects.
Day relative to IP is the number of days relative to day of administration of Artesunate, where the day of dose = 1.
Rando = Randomisation; IP = Artesunate; H = High; L = Low; NCS = Not Clinically Significant; CS = Clinically Significant.

Listing 16.2.8.1a
Individual Haematology Results

Rando Number	Challenge Agent	Parameter (unit) [Normal Range]	Visit	Date of Assessment	Time of Assessment	Day Relative to Inoculum	Day Relative to IP	Result	Range Indicator	Clinical Assessment
R205	K13	Basophils (10^9/L) [0 – 0.15]	Screening	31-JAN-2018	10:01	-19	-28	0.03		
			Safety Visit	16-FEB-2018	07:45	-3	-12	0.02		
			Admission	28-FEB-2018	12:19	9	1	0.02		
			Safety Monitoring	02-MAR-2018	13:11	11	3	0.01		
			Admission 3	03-MAR-2018	13:15	12	4	0.02		
			Safety Monitoring	09-MAR-2018	07:52	18	10	0.04		
			Safety Monitoring	19-MAR-2018	07:29	28	20	0.05		
			End of Study	22-MAR-2018	07:30	31	23	0.06		
			Unscheduled	26-MAR-2018	07:26	35	27	0.04		
		Eosinophils (10^9/L) [0 – 0.6]	Screening	31-JAN-2018	10:01	-19	-28	0.08		
			Safety Visit	16-FEB-2018	07:45	-3	-12	0.07		
			Admission	28-FEB-2018	12:19	9	1	0.08		
			Safety Monitoring	02-MAR-2018	13:11	11	3	0.03		
			Admission 3	03-MAR-2018	13:15	12	4	0.04		
			Safety Monitoring	05-MAR-2018	09:13	14	6	0.12		
			Safety Monitoring	09-MAR-2018	07:52	18	10	0.14		
			Safety Monitoring	19-MAR-2018	07:29	28	20	0.25		
			End of Study	22-MAR-2018	07:30	31	23	0.30		
			Unscheduled	26-MAR-2018	07:26	35	27	0.12		

Notes: Day relative to inoculum is the number of days relative to day of administration of the inoculum, where the day of administration = 0.
The inoculum was administered on Study Day 0 for K13-infected subjects and Study Day 1 for 3D7-infected subjects.
Day relative to IP is the number of days relative to day of administration of Artesunate, where the day of dose = 1.
Rando = Randomisation; IP = Artesunate; H = High; L = Low; NCS = Not Clinically Significant; CS = Clinically Significant.

Listing 16.2.8.1a
Individual Haematology Results

Rando Number	Challenge Agent	Parameter (unit) [Normal Range]	Visit	Date of Assessment	Time of Assessment	Day Relative to Inoculum	Day Relative to IP	Result	Range Indicator	Clinical Assessment
R205	K13	Ery. Mean Corpuscular Volume (fL) [80 – 100]	Screening	31-JAN-2018	10:01	-19	-28	89		
			Safety Visit	16-FEB-2018	07:45	-3	-12	92		
			Admission	28-FEB-2018	12:19	9	1	91		
			Safety Monitoring	02-MAR-2018	13:11	11	3	90		
			Admission 3	03-MAR-2018	13:15	12	4	90		
			Safety Monitoring	05-MAR-2018	09:13	14	6	89		
			Safety Monitoring	09-MAR-2018	07:52	18	10	90		
			Safety Monitoring	19-MAR-2018	07:29	28	20	91		
			End of Study	22-MAR-2018	07:30	31	23	89		
			Unscheduled	26-MAR-2018	07:26	35	27	91		

Notes: Day relative to inoculum is the number of days relative to day of administration of the inoculum, where the day of administration = 0.
The inoculum was administered on Study Day 0 for K13-infected subjects and Study Day 1 for 3D7-infected subjects.
Day relative to IP is the number of days relative to day of administration of Artesunate, where the day of dose = 1.
Rando = Randomisation; IP = Artesunate; H = High; L = Low; NCS = Not Clinically Significant; CS = Clinically Significant.

Listing 16.2.8.1a
Individual Haematology Results

Rando Number	Challenge Agent	Parameter (unit) [Normal Range]	Visit	Date of Assessment	Time of Assessment	Day Relative to Inoculum	Day Relative to IP	Result	Range Indicator	Clinical Assessment
R205	K13	Erythrocytes (10^12/L) [3.9 – 5.6]	Screening	31-JAN-2018	10:01	-19	-28	4.0		
			Safety Visit	16-FEB-2018	07:45	-3	-12	3.6	L	NCS
			Admission	28-FEB-2018	12:19	9	1	3.5	L	NCS
			Safety Monitoring	02-MAR-2018	13:11	11	3	3.8	L	NCS
			Admission 3	03-MAR-2018	13:15	12	4	3.9		
			Safety Monitoring	05-MAR-2018	09:13	14	6	4.0		
			Safety Monitoring	09-MAR-2018	07:52	18	10	4.1		
			Safety Monitoring	19-MAR-2018	07:29	28	20	3.7	L	NCS
			End of Study	22-MAR-2018	07:30	31	23	3.6	L	NCS
			Unscheduled	26-MAR-2018	07:26	35	27	3.8	L	NCS

Notes: Day relative to inoculum is the number of days relative to day of administration of the inoculum, where the day of administration = 0.
The inoculum was administered on Study Day 0 for K13-infected subjects and Study Day 1 for 3D7-infected subjects.
Day relative to IP is the number of days relative to day of administration of Artesunate, where the day of dose = 1.
Rando = Randomisation; IP = Artesunate; H = High; L = Low; NCS = Not Clinically Significant; CS = Clinically Significant.

Listing 16.2.8.1a
Individual Haematology Results

Rando Number	Challenge Agent	Parameter (unit) [Normal Range]	Visit	Date of Assessment	Time of Assessment	Day Relative to Inoculum	Day Relative to IP	Result	Range Indicator	Clinical Assessment
R205	K13	Hematocrit [0.35 – 0.47]	Screening	31-JAN-2018	10:01	-19	-28	0.36		
			Safety Visit	16-FEB-2018	07:45	-3	-12	0.33	L	NCS
			Unscheduled	17-FEB-2018	07:35	-2	-11	0.38		
			Admission	28-FEB-2018	12:19	9	1	0.32	L	NCS
			Safety Monitoring	02-MAR-2018	13:11	11	3	0.34	L	NCS
			Admission 3	03-MAR-2018	13:15	12	4	0.36		
			Safety Monitoring	05-MAR-2018	09:13	14	6	0.35		
			Safety Monitoring	09-MAR-2018	07:52	18	10	0.37		
			Safety Monitoring	19-MAR-2018	07:29	28	20	0.34	L	NCS
			End of Study	22-MAR-2018	07:30	31	23	0.32	L	NCS
			Unscheduled	26-MAR-2018	07:26	35	27	0.34	L	NCS

Notes: Day relative to inoculum is the number of days relative to day of administration of the inoculum, where the day of administration = 0.
The inoculum was administered on Study Day 0 for K13-infected subjects and Study Day 1 for 3D7-infected subjects.
Day relative to IP is the number of days relative to day of administration of Artesunate, where the day of dose = 1.
Rando = Randomisation; IP = Artesunate; H = High; L = Low; NCS = Not Clinically Significant; CS = Clinically Significant.

Listing 16.2.8.1a
Individual Haematology Results

Rando Number	Challenge Agent	Parameter (unit) [Normal Range]	Visit	Date of Assessment	Time of Assessment	Day Relative to Inoculum	Day Relative to IP	Result	Range Indicator	Clinical Assessment
R205	K13	Hemoglobin (g/L) [115 – 165]	Screening	31-JAN-2018	10:01	-19	-28	122		
			Safety Visit	16-FEB-2018	07:45	-3	-12	109	L	NCS
			Unscheduled	17-FEB-2018	07:35	-2	-11	126		
			Admission	28-FEB-2018	12:19	9	1	108	L	NCS
			Safety Monitoring	02-MAR-2018	13:11	11	3	114	L	NCS
			Admission 3	03-MAR-2018	13:15	12	4	119		
			Safety Monitoring	05-MAR-2018	09:13	14	6	118		
			Safety Monitoring	09-MAR-2018	07:52	18	10	123		
			Safety Monitoring	19-MAR-2018	07:29	28	20	111	L	NCS
			End of Study	22-MAR-2018	07:30	31	23	111	L	NCS
			Unscheduled	26-MAR-2018	07:26	35	27	113	L	NCS

Notes: Day relative to inoculum is the number of days relative to day of administration of the inoculum, where the day of administration = 0.
The inoculum was administered on Study Day 0 for K13-infected subjects and Study Day 1 for 3D7-infected subjects.
Day relative to IP is the number of days relative to day of administration of Artesunate, where the day of dose = 1.
Rando = Randomisation; IP = Artesunate; H = High; L = Low; NCS = Not Clinically Significant; CS = Clinically Significant.

Listing 16.2.8.1a
Individual Haematology Results

Rando Number	Challenge Agent	Parameter (unit) [Normal Range]	Visit	Date of Assessment	Time of Assessment	Day Relative to Inoculum	Day Relative to IP	Result	Range Indicator	Clinical Assessment
R205	K13	Leukocytes (10^9/L) [3.5 – 12]	Screening	31-JAN-2018	10:01	-19	-28	5.3		
			Safety Visit	16-FEB-2018	07:45	-3	-12	3.6		
			Admission	28-FEB-2018	12:19	9	1	5.2		
			Safety Monitoring	02-MAR-2018	13:11	11	3	2.1	L	CS
			Admission 3	03-MAR-2018	13:15	12	4	2.3	L	CS
			Safety Monitoring	05-MAR-2018	09:13	14	6	2.9	L	CS
			Safety Monitoring	09-MAR-2018	07:52	18	10	5.9		
			Safety Monitoring	19-MAR-2018	07:29	28	20	5.0		
			End of Study	22-MAR-2018	07:30	31	23	4.4		
			Unscheduled	26-MAR-2018	07:26	35	27	4.3		

Notes: Day relative to inoculum is the number of days relative to day of administration of the inoculum, where the day of administration = 0.
The inoculum was administered on Study Day 0 for K13-infected subjects and Study Day 1 for 3D7-infected subjects.
Day relative to IP is the number of days relative to day of administration of Artesunate, where the day of dose = 1.
Rando = Randomisation; IP = Artesunate; H = High; L = Low; NCS = Not Clinically Significant; CS = Clinically Significant.

Listing 16.2.8.1a
Individual Haematology Results

Rando Number	Challenge Agent	Parameter (unit) [Normal Range]	Visit	Date of Assessment	Time of Assessment	Day Relative to Inoculum	Day Relative to IP	Result	Range Indicator	Clinical Assessment
R205	K13	Lymphocytes (10^9/L) [1 – 4]	Screening	31-JAN-2018	10:01	-19	-28	1.67		
			Safety Visit	16-FEB-2018	07:45	-3	-12	1.49		
			Admission	28-FEB-2018	12:19	9	1	1.74		
			Safety Monitoring	02-MAR-2018	13:11	11	3	0.81	L	NCS
			Admission 3	03-MAR-2018	13:15	12	4	0.82	L	NCS
			Safety Monitoring	05-MAR-2018	09:13	14	6	1.33		
			Safety Monitoring	09-MAR-2018	07:52	18	10	2.47		
			Safety Monitoring	19-MAR-2018	07:29	28	20	1.98		
			End of Study	22-MAR-2018	07:30	31	23	1.86		
			Unscheduled	26-MAR-2018	07:26	35	27	1.91		
		Lymphocytes Atypical (10^9/L) [0 – 0.01]	Safety Monitoring	05-MAR-2018	09:13	14	6	0.12	H	NCS

Notes: Day relative to inoculum is the number of days relative to day of administration of the inoculum, where the day of administration = 0.
The inoculum was administered on Study Day 0 for K13-infected subjects and Study Day 1 for 3D7-infected subjects.
Day relative to IP is the number of days relative to day of administration of Artesunate, where the day of dose = 1.
Rando = Randomisation; IP = Artesunate; H = High; L = Low; NCS = Not Clinically Significant; CS = Clinically Significant.

Listing 16.2.8.1a
Individual Haematology Results

Rando Number	Challenge Agent	Parameter (unit) [Normal Range]	Visit	Date of Assessment	Time of Assessment	Day Relative to Inoculum	Day Relative to IP	Result	Range Indicator	Clinical Assessment
R205	K13	Monocytes (10^9/L) [0 – 0.9]	Screening	31-JAN-2018	10:01	-19	-28	0.26		
			Safety Visit	16-FEB-2018	07:45	-3	-12	0.25		
			Admission	28-FEB-2018	12:19	9	1	0.40		
			Safety Monitoring	02-MAR-2018	13:11	11	3	0.12		
			Admission 3	03-MAR-2018	13:15	12	4	0.15		
			Safety Monitoring	05-MAR-2018	09:13	14	6	0.12		
			Safety Monitoring	09-MAR-2018	07:52	18	10	0.37		
			Safety Monitoring	19-MAR-2018	07:29	28	20	0.34		
			End of Study	22-MAR-2018	07:30	31	23	0.42		
			Unscheduled	26-MAR-2018	07:26	35	27	0.27		

Notes: Day relative to inoculum is the number of days relative to day of administration of the inoculum, where the day of administration = 0.
The inoculum was administered on Study Day 0 for K13-infected subjects and Study Day 1 for 3D7-infected subjects.
Day relative to IP is the number of days relative to day of administration of Artesunate, where the day of dose = 1.
Rando = Randomisation; IP = Artesunate; H = High; L = Low; NCS = Not Clinically Significant; CS = Clinically Significant.

Listing 16.2.8.1a
Individual Haematology Results

Rando Number	Challenge Agent	Parameter (unit) [Normal Range]	Visit	Date of Assessment	Time of Assessment	Day Relative to Inoculum	Day Relative to IP	Result	Range Indicator	Clinical Assessment
R205	K13	Neutrophils (10^9/L) [1.5 – 8]	Screening	31-JAN-2018	10:01	-19	-28	3.21		
			Safety Visit	16-FEB-2018	07:45	-3	-12	1.81		
			Admission	28-FEB-2018	12:19	9	1	2.92		
			Safety Monitoring	02-MAR-2018	13:11	11	3	1.08	L	CS
			Admission 3	03-MAR-2018	13:15	12	4	1.26	L	CS
			Safety Monitoring	05-MAR-2018	09:13	14	6	1.22	L	CS
			Safety Monitoring	09-MAR-2018	07:52	18	10	2.86		
			Safety Monitoring	19-MAR-2018	07:29	28	20	2.37		
			End of Study	22-MAR-2018	07:30	31	23	1.71		
			Unscheduled	26-MAR-2018	07:26	35	27	1.95		

Notes: Day relative to inoculum is the number of days relative to day of administration of the inoculum, where the day of administration = 0.
The inoculum was administered on Study Day 0 for K13-infected subjects and Study Day 1 for 3D7-infected subjects.
Day relative to IP is the number of days relative to day of administration of Artesunate, where the day of dose = 1.
Rando = Randomisation; IP = Artesunate; H = High; L = Low; NCS = Not Clinically Significant; CS = Clinically Significant.

Listing 16.2.8.1a
Individual Haematology Results

Rando Number	Challenge Agent	Parameter (unit) [Normal Range]	Visit	Date of Assessment	Time of Assessment	Day Relative to Inoculum	Day Relative to IP	Result	Range Indicator	Clinical Assessment
R205	K13	Platelets (10^9/L) [150 – 400]	Screening	31-JAN-2018	10:01	-19	-28	235		
			Safety Visit	16-FEB-2018	07:45	-3	-12	231		
			Admission	28-FEB-2018	12:19	9	1	223		
			Safety Monitoring	02-MAR-2018	13:11	11	3	168		
			Admission 3	03-MAR-2018	13:15	12	4	161		
			Safety Monitoring	05-MAR-2018	09:13	14	6	140	L	NCS
			Safety Monitoring	09-MAR-2018	07:52	18	10	298		
			Safety Monitoring	19-MAR-2018	07:29	28	20	295		
			End of Study	22-MAR-2018	07:30	31	23	269		
			Unscheduled	26-MAR-2018	07:26	35	27	266		
		Reticulocytes (10^9/L) [25 – 120]	Safety Visit	16-FEB-2018	07:45	-3	-12	29		
			End of Study	22-MAR-2018	07:30	31	23	57		

Notes: Day relative to inoculum is the number of days relative to day of administration of the inoculum, where the day of administration = 0.
The inoculum was administered on Study Day 0 for K13-infected subjects and Study Day 1 for 3D7-infected subjects.
Day relative to IP is the number of days relative to day of administration of Artesunate, where the day of dose = 1.
Rando = Randomisation; IP = Artesunate; H = High; L = Low; NCS = Not Clinically Significant; CS = Clinically Significant.

Listing 16.2.8.1a
Individual Haematology Results

Rando Number	Challenge Agent	Parameter (unit) [Normal Range]	Visit	Date of Assessment	Time of Assessment	Day Relative to Inoculum	Day Relative to IP	Result	Range Indicator	Clinical Assessment
R206	K13	Basophils (10^9/L) [0 – 0.15]	Screening	06-FEB-2018	13:32	-13	-22	0.04		
			Safety Visit	16-FEB-2018	08:00	-3	-12	0.04		
			Admission	28-FEB-2018	12:48	9	1	0.04		
			Safety Monitoring	02-MAR-2018	13:29	11	3	0.01		
			Admission 3	03-MAR-2018	13:39	12	4	0.01		
			Safety Monitoring	05-MAR-2018	09:30	14	6	0.02		
			Safety Monitoring	09-MAR-2018	08:58	18	10	0.03		
			Safety Monitoring	19-MAR-2018	09:44	28	20	0.03		
			End of Study	22-MAR-2018	09:19	31	23	0.02		
			Unscheduled	28-MAR-2018	10:02	37	29	0.04		

Notes: Day relative to inoculum is the number of days relative to day of administration of the inoculum, where the day of administration = 0.
The inoculum was administered on Study Day 0 for K13-infected subjects and Study Day 1 for 3D7-infected subjects.
Day relative to IP is the number of days relative to day of administration of Artesunate, where the day of dose = 1.
Rando = Randomisation; IP = Artesunate; H = High; L = Low; NCS = Not Clinically Significant; CS = Clinically Significant.

Listing 16.2.8.1a
Individual Haematology Results

Rando Number	Challenge Agent	Parameter (unit) [Normal Range]	Visit	Date of Assessment	Time of Assessment	Day Relative to Inoculum	Day Relative to IP	Result	Range Indicator	Clinical Assessment
R206	K13	Eosinophils (10^9/L) [0 – 0.6]	Screening	06-FEB-2018	13:32	-13	-22	0.12		
			Safety Visit	16-FEB-2018	08:00	-3	-12	0.15		
			Admission	28-FEB-2018	12:48	9	1	0.02		
			Safety Monitoring	02-MAR-2018	13:29	11	3	0.01		
			Admission 3	03-MAR-2018	13:39	12	4	0.02		
			Safety Monitoring	05-MAR-2018	09:30	14	6	0.01		
			Safety Monitoring	09-MAR-2018	08:58	18	10	0.05		
			Safety Monitoring	19-MAR-2018	09:44	28	20	0.01		
			End of Study	22-MAR-2018	09:19	31	23	0.03		
			Unscheduled	28-MAR-2018	10:02	37	29	0.03		

Notes: Day relative to inoculum is the number of days relative to day of administration of the inoculum, where the day of administration = 0.
The inoculum was administered on Study Day 0 for K13-infected subjects and Study Day 1 for 3D7-infected subjects.
Day relative to IP is the number of days relative to day of administration of Artesunate, where the day of dose = 1.
Rando = Randomisation; IP = Artesunate; H = High; L = Low; NCS = Not Clinically Significant; CS = Clinically Significant.

Listing 16.2.8.1a
Individual Haematology Results

Rando Number	Challenge Agent	Parameter (unit) [Normal Range]	Visit	Date of Assessment	Time of Assessment	Day Relative to Inoculum	Day Relative to IP	Result	Range Indicator	Clinical Assessment
R206	K13	Ery. Mean Corpuscular Volume (fL) [80 – 100]	Screening	06-FEB-2018	13:32	-13	-22	91		
			Safety Visit	16-FEB-2018	08:00	-3	-12	92		
			Admission	28-FEB-2018	12:48	9	1	91		
			Safety Monitoring	02-MAR-2018	13:29	11	3	91		
			Admission 3	03-MAR-2018	13:39	12	4	90		
			Safety Monitoring	05-MAR-2018	09:30	14	6	90		
			Safety Monitoring	09-MAR-2018	08:58	18	10	90		
			Safety Monitoring	19-MAR-2018	09:44	28	20	91		
			End of Study	22-MAR-2018	09:19	31	23	91		
			Unscheduled	28-MAR-2018	10:02	37	29	89		

Notes: Day relative to inoculum is the number of days relative to day of administration of the inoculum, where the day of administration = 0.
The inoculum was administered on Study Day 0 for K13-infected subjects and Study Day 1 for 3D7-infected subjects.
Day relative to IP is the number of days relative to day of administration of Artesunate, where the day of dose = 1.
Rando = Randomisation; IP = Artesunate; H = High; L = Low; NCS = Not Clinically Significant; CS = Clinically Significant.

Listing 16.2.8.1a
Individual Haematology Results

Rando Number	Challenge Agent	Parameter (unit) [Normal Range]	Visit	Date of Assessment	Time of Assessment	Day Relative to Inoculum	Day Relative to IP	Result	Range Indicator	Clinical Assessment
R206	K13	Erythrocytes (10^12/L) [3.9 – 5.6]	Screening	06-FEB-2018	13:32	-13	-22	4.5		
			Safety Visit	16-FEB-2018	08:00	-3	-12	4.1		
			Admission	28-FEB-2018	12:48	9	1	4.0		
			Safety Monitoring	02-MAR-2018	13:29	11	3	4.2		
			Admission 3	03-MAR-2018	13:39	12	4	4.4		
			Safety Monitoring	05-MAR-2018	09:30	14	6	4.1		
			Safety Monitoring	09-MAR-2018	08:58	18	10	4.0		
			Safety Monitoring	19-MAR-2018	09:44	28	20	4.0		
			End of Study	22-MAR-2018	09:19	31	23	3.4	L	NCS
			Unscheduled	28-MAR-2018	10:02	37	29	4.0		

Notes: Day relative to inoculum is the number of days relative to day of administration of the inoculum, where the day of administration = 0.
The inoculum was administered on Study Day 0 for K13-infected subjects and Study Day 1 for 3D7-infected subjects.
Day relative to IP is the number of days relative to day of administration of Artesunate, where the day of dose = 1.
Rando = Randomisation; IP = Artesunate; H = High; L = Low; NCS = Not Clinically Significant; CS = Clinically Significant.

Listing 16.2.8.1a
Individual Haematology Results

Rando Number	Challenge Agent	Parameter (unit) [Normal Range]	Visit	Date of Assessment	Time of Assessment	Day Relative to Inoculum	Day Relative to IP	Result	Range Indicator	Clinical Assessment
R206	K13	Hematocrit [0.35 – 0.47]	Screening	06-FEB-2018	13:32	-13	-22	0.41		
			Safety Visit	16-FEB-2018	08:00	-3	-12	0.38		
			Admission	28-FEB-2018	12:48	9	1	0.36		
			Safety Monitoring	02-MAR-2018	13:29	11	3	0.38		
			Admission 3	03-MAR-2018	13:39	12	4	0.40		
			Safety Monitoring	05-MAR-2018	09:30	14	6	0.37		
			Safety Monitoring	09-MAR-2018	08:58	18	10	0.36		
			Safety Monitoring	19-MAR-2018	09:44	28	20	0.37		
			End of Study	22-MAR-2018	09:19	31	23	0.31	L	NCS
			Unscheduled	28-MAR-2018	10:02	37	29	0.35		

Notes: Day relative to inoculum is the number of days relative to day of administration of the inoculum, where the day of administration = 0.
The inoculum was administered on Study Day 0 for K13-infected subjects and Study Day 1 for 3D7-infected subjects.
Day relative to IP is the number of days relative to day of administration of Artesunate, where the day of dose = 1.
Rando = Randomisation; IP = Artesunate; H = High; L = Low; NCS = Not Clinically Significant; CS = Clinically Significant.

Listing 16.2.8.1a
Individual Haematology Results

Rando Number	Challenge Agent	Parameter (unit) [Normal Range]	Visit	Date of Assessment	Time of Assessment	Day Relative to Inoculum	Day Relative to IP	Result	Range Indicator	Clinical Assessment
R206	K13	Hemoglobin (g/L) [115 – 165]	Screening	06-FEB-2018	13:32	-13	-22	136		
			Safety Visit	16-FEB-2018	08:00	-3	-12	128		
			Admission	28-FEB-2018	12:48	9	1	121		
			Safety Monitoring	02-MAR-2018	13:29	11	3	128		
			Admission 3	03-MAR-2018	13:39	12	4	131		
			Safety Monitoring	05-MAR-2018	09:30	14	6	123		
			Safety Monitoring	09-MAR-2018	08:58	18	10	120		
			Safety Monitoring	19-MAR-2018	09:44	28	20	120		
			End of Study	22-MAR-2018	09:19	31	23	110	L	NCS
			Unscheduled	28-MAR-2018	10:02	37	29	121		

Notes: Day relative to inoculum is the number of days relative to day of administration of the inoculum, where the day of administration = 0.
The inoculum was administered on Study Day 0 for K13-infected subjects and Study Day 1 for 3D7-infected subjects.
Day relative to IP is the number of days relative to day of administration of Artesunate, where the day of dose = 1.
Rando = Randomisation; IP = Artesunate; H = High; L = Low; NCS = Not Clinically Significant; CS = Clinically Significant.

Listing 16.2.8.1a
Individual Haematology Results

Rando Number	Challenge Agent	Parameter (unit) [Normal Range]	Visit	Date of Assessment	Time of Assessment	Day Relative to Inoculum	Day Relative to IP	Result	Range Indicator	Clinical Assessment
R206	K13	Leukocytes (10^9/L) [3.5 – 12]	Screening	06-FEB-2018	13:32	-13	-22	7.8		
			Safety Visit	16-FEB-2018	08:00	-3	-12	8.6		
			Admission	28-FEB-2018	12:48	9	1	3.7		
			Safety Monitoring	02-MAR-2018	13:29	11	3	1.8	L	CS
			Admission 3	03-MAR-2018	13:39	12	4	2.6	L	CS
			Safety Monitoring	05-MAR-2018	09:30	14	6	3.8		
			Safety Monitoring	09-MAR-2018	08:58	18	10	7.7		
			Safety Monitoring	19-MAR-2018	09:44	28	20	7.2		
			End of Study	22-MAR-2018	09:19	31	23	5.8		
			Unscheduled	28-MAR-2018	10:02	37	29	7.1		

Notes: Day relative to inoculum is the number of days relative to day of administration of the inoculum, where the day of administration = 0.
The inoculum was administered on Study Day 0 for K13-infected subjects and Study Day 1 for 3D7-infected subjects.
Day relative to IP is the number of days relative to day of administration of Artesunate, where the day of dose = 1.
Rando = Randomisation; IP = Artesunate; H = High; L = Low; NCS = Not Clinically Significant; CS = Clinically Significant.

Listing 16.2.8.1a
Individual Haematology Results

Rando Number	Challenge Agent	Parameter (unit) [Normal Range]	Visit	Date of Assessment	Time of Assessment	Day Relative to Inoculum	Day Relative to IP	Result	Range Indicator	Clinical Assessment
R206	K13	Lymphocytes (10^9/L) [1 – 4]	Screening	06-FEB-2018	13:32	-13	-22	2.13		
			Safety Visit	16-FEB-2018	08:00	-3	-12	1.55		
			Admission	28-FEB-2018	12:48	9	1	0.73	L	CS
			Safety Monitoring	02-MAR-2018	13:29	11	3	0.57	L	CS
			Admission 3	03-MAR-2018	13:39	12	4	0.78	L	CS
			Safety Monitoring	05-MAR-2018	09:30	14	6	1.10		
			Safety Monitoring	09-MAR-2018	08:58	18	10	2.21		
			Safety Monitoring	19-MAR-2018	09:44	28	20	1.30		
			End of Study	22-MAR-2018	09:19	31	23	1.36		
			Unscheduled	28-MAR-2018	10:02	37	29	1.77		

Notes: Day relative to inoculum is the number of days relative to day of administration of the inoculum, where the day of administration = 0.
The inoculum was administered on Study Day 0 for K13-infected subjects and Study Day 1 for 3D7-infected subjects.
Day relative to IP is the number of days relative to day of administration of Artesunate, where the day of dose = 1.
Rando = Randomisation; IP = Artesunate; H = High; L = Low; NCS = Not Clinically Significant; CS = Clinically Significant.

Listing 16.2.8.1a
Individual Haematology Results

Rando Number	Challenge Agent	Parameter (unit) [Normal Range]	Visit	Date of Assessment	Time of Assessment	Day Relative to Inoculum	Day Relative to IP	Result	Range Indicator	Clinical Assessment
R206	K13	Monocytes (10^9/L) [0 – 0.9]	Screening	06-FEB-2018	13:32	-13	-22	0.57		
			Safety Visit	16-FEB-2018	08:00	-3	-12	0.59		
			Admission	28-FEB-2018	12:48	9	1	0.40		
			Safety Monitoring	02-MAR-2018	13:29	11	3	0.29		
			Admission 3	03-MAR-2018	13:39	12	4	0.38		
			Safety Monitoring	05-MAR-2018	09:30	14	6	0.39		
			Safety Monitoring	09-MAR-2018	08:58	18	10	0.63		
			Safety Monitoring	19-MAR-2018	09:44	28	20	0.44		
			End of Study	22-MAR-2018	09:19	31	23	0.43		
			Unscheduled	28-MAR-2018	10:02	37	29	0.50		

Notes: Day relative to inoculum is the number of days relative to day of administration of the inoculum, where the day of administration = 0.
The inoculum was administered on Study Day 0 for K13-infected subjects and Study Day 1 for 3D7-infected subjects.
Day relative to IP is the number of days relative to day of administration of Artesunate, where the day of dose = 1.
Rando = Randomisation; IP = Artesunate; H = High; L = Low; NCS = Not Clinically Significant; CS = Clinically Significant.

Listing 16.2.8.1a
Individual Haematology Results

Rando Number	Challenge Agent	Parameter (unit) [Normal Range]	Visit	Date of Assessment	Time of Assessment	Day Relative to Inoculum	Day Relative to IP	Result	Range Indicator	Clinical Assessment
R206	K13	Neutrophils (10^9/L) [1.5 – 8]	Screening	06-FEB-2018	13:32	-13	-22	4.89		
			Safety Visit	16-FEB-2018	08:00	-3	-12	6.24		
			Admission	28-FEB-2018	12:48	9	1	2.46		
			Safety Monitoring	02-MAR-2018	13:29	11	3	0.95	L	CS
			Admission 3	03-MAR-2018	13:39	12	4	1.38	L	CS
			Safety Monitoring	05-MAR-2018	09:30	14	6	2.28		
			Safety Monitoring	09-MAR-2018	08:58	18	10	4.79		
			Safety Monitoring	19-MAR-2018	09:44	28	20	5.37		
			End of Study	22-MAR-2018	09:19	31	23	3.92		
			Unscheduled	28-MAR-2018	10:02	37	29	4.72		

Notes: Day relative to inoculum is the number of days relative to day of administration of the inoculum, where the day of administration = 0.
The inoculum was administered on Study Day 0 for K13-infected subjects and Study Day 1 for 3D7-infected subjects.
Day relative to IP is the number of days relative to day of administration of Artesunate, where the day of dose = 1.
Rando = Randomisation; IP = Artesunate; H = High; L = Low; NCS = Not Clinically Significant; CS = Clinically Significant.

Listing 16.2.8.1a
Individual Haematology Results

Rando Number	Challenge Agent	Parameter (unit) [Normal Range]	Visit	Date of Assessment	Time of Assessment	Day Relative to Inoculum	Day Relative to IP	Result	Range Indicator	Clinical Assessment
R206	K13	Platelets (10^9/L) [150 – 400]	Screening	06-FEB-2018	13:32	-13	-22	286		
			Safety Visit	16-FEB-2018	08:00	-3	-12	251		
			Admission	28-FEB-2018	12:48	9	1	232		
			Safety Monitoring	02-MAR-2018	13:29	11	3	164		
			Admission 3	03-MAR-2018	13:39	12	4	150		
			Safety Monitoring	05-MAR-2018	09:30	14	6	162		
			Safety Monitoring	09-MAR-2018	08:58	18	10	288		
			Safety Monitoring	19-MAR-2018	09:44	28	20	230		
			End of Study	22-MAR-2018	09:19	31	23	230		
			Unscheduled	28-MAR-2018	10:02	37	29	231		
		Reticulocytes (10^9/L) [25 – 120]	Safety Visit	16-FEB-2018	08:00	-3	-12	49		
			End of Study	22-MAR-2018	09:19	31	23	68		

Notes: Day relative to inoculum is the number of days relative to day of administration of the inoculum, where the day of administration = 0.
The inoculum was administered on Study Day 0 for K13-infected subjects and Study Day 1 for 3D7-infected subjects.
Day relative to IP is the number of days relative to day of administration of Artesunate, where the day of dose = 1.
Rando = Randomisation; IP = Artesunate; H = High; L = Low; NCS = Not Clinically Significant; CS = Clinically Significant.

Listing 16.2.8.1a
Individual Haematology Results

Rando Number	Challenge Agent	Parameter (unit) [Normal Range]	Visit	Date of Assessment	Time of Assessment	Day Relative to Inoculum	Day Relative to IP	Result	Range Indicator	Clinical Assessment
R207	K13	Basophils (10^9/L) [0 – 0.15]	Screening	14-FEB-2018	11:34	-5	-14	0.06		
			Safety Visit	16-FEB-2018	08:46	-3	-12	0.06		
			Admission	28-FEB-2018	12:00	9	1	0.05		
			Safety Monitoring	02-MAR-2018	12:59	11	3	0.02		
			Admission 3	03-MAR-2018	13:00	12	4	0.04		
			Safety Monitoring	05-MAR-2018	10:01	14	6	0.05		
			Safety Monitoring	19-MAR-2018	09:12	28	20	0.06		
			End of Study	22-MAR-2018	09:32	31	23	0.13		
		Eosinophils (10^9/L) [0 – 0.6]	Screening	14-FEB-2018	11:34	-5	-14	0.14		
			Safety Visit	16-FEB-2018	08:46	-3	-12	0.21		
			Admission	28-FEB-2018	12:00	9	1	0.14		
			Safety Monitoring	02-MAR-2018	12:59	11	3	0.08		
			Admission 3	03-MAR-2018	13:00	12	4	0.16		
			Safety Monitoring	05-MAR-2018	10:01	14	6	0.20		
			Safety Monitoring	09-MAR-2018	09:33	18	10	0.13		
			Safety Monitoring	19-MAR-2018	09:12	28	20	0.43		
			End of Study	22-MAR-2018	09:32	31	23	0.57		

Notes: Day relative to inoculum is the number of days relative to day of administration of the inoculum, where the day of administration = 0.
The inoculum was administered on Study Day 0 for K13-infected subjects and Study Day 1 for 3D7-infected subjects.
Day relative to IP is the number of days relative to day of administration of Artesunate, where the day of dose = 1.
Rando = Randomisation; IP = Artesunate; H = High; L = Low; NCS = Not Clinically Significant; CS = Clinically Significant.

Listing 16.2.8.1a
Individual Haematology Results

Rando Number	Challenge Agent	Parameter (unit) [Normal Range]	Visit	Date of Assessment	Time of Assessment	Day Relative to Inoculum	Day Relative to IP	Result	Range Indicator	Clinical Assessment
R207	K13	Ery. Mean Corpuscular Volume (fL) [80 – 100]	Screening	14-FEB-2018	11:34	-5	-14	87		
			Safety Visit	16-FEB-2018	08:46	-3	-12	87		
			Admission	28-FEB-2018	12:00	9	1	86		
			Safety Monitoring	02-MAR-2018	12:59	11	3	85		
			Admission 3	03-MAR-2018	13:00	12	4	87		
			Safety Monitoring	05-MAR-2018	10:01	14	6	86		
			Safety Monitoring	09-MAR-2018	09:33	18	10	87		
			Safety Monitoring	19-MAR-2018	09:12	28	20	87		
			End of Study	22-MAR-2018	09:32	31	23	86		
R207		Erythrocytes (10^12/L) [4.5 – 6.5]	Screening	14-FEB-2018	11:34	-5	-14	5.1		
			Safety Visit	16-FEB-2018	08:46	-3	-12	4.8		
			Admission	28-FEB-2018	12:00	9	1	5.0		
			Safety Monitoring	02-MAR-2018	12:59	11	3	5.2		
			Admission 3	03-MAR-2018	13:00	12	4	5.2		
			Safety Monitoring	05-MAR-2018	10:01	14	6	4.7		
			Safety Monitoring	09-MAR-2018	09:33	18	10	4.8		
			Safety Monitoring	19-MAR-2018	09:12	28	20	4.6		
			End of Study	22-MAR-2018	09:32	31	23	4.8		

Notes: Day relative to inoculum is the number of days relative to day of administration of the inoculum, where the day of administration = 0.
The inoculum was administered on Study Day 0 for K13-infected subjects and Study Day 1 for 3D7-infected subjects.
Day relative to IP is the number of days relative to day of administration of Artesunate, where the day of dose = 1.
Rando = Randomisation; IP = Artesunate; H = High; L = Low; NCS = Not Clinically Significant; CS = Clinically Significant.

Listing 16.2.8.1a
Individual Haematology Results

Rando Number	Challenge Agent	Parameter (unit) [Normal Range]	Visit	Date of Assessment	Time of Assessment	Day Relative to Inoculum	Day Relative to IP	Result	Range Indicator	Clinical Assessment
R207	K13	Hematocrit [0.4 – 0.54]	Screening	14-FEB-2018	11:34	-5	-14	0.45		
			Safety Visit	16-FEB-2018	08:46	-3	-12	0.42		
			Admission	28-FEB-2018	12:00	9	1	0.43		
			Safety Monitoring	02-MAR-2018	12:59	11	3	0.44		
			Admission 3	03-MAR-2018	13:00	12	4	0.45		
			Safety Monitoring	05-MAR-2018	10:01	14	6	0.41		
			Safety Monitoring	09-MAR-2018	09:33	18	10	0.42		
			Safety Monitoring	19-MAR-2018	09:12	28	20	0.40		
			End of Study	22-MAR-2018	09:32	31	23	0.41		
		Hemoglobin (g/L) [135 – 175]	Screening	14-FEB-2018	11:34	-5	-14	150		
			Safety Visit	16-FEB-2018	08:46	-3	-12	144		
			Admission	28-FEB-2018	12:00	9	1	147		
			Safety Monitoring	02-MAR-2018	12:59	11	3	154		
			Admission 3	03-MAR-2018	13:00	12	4	152		
			Safety Monitoring	05-MAR-2018	10:01	14	6	138		
			Safety Monitoring	09-MAR-2018	09:33	18	10	144		
			Safety Monitoring	19-MAR-2018	09:12	28	20	135		
			End of Study	22-MAR-2018	09:32	31	23	143		

Notes: Day relative to inoculum is the number of days relative to day of administration of the inoculum, where the day of administration = 0.
The inoculum was administered on Study Day 0 for K13-infected subjects and Study Day 1 for 3D7-infected subjects.
Day relative to IP is the number of days relative to day of administration of Artesunate, where the day of dose = 1.
Rando = Randomisation; IP = Artesunate; H = High; L = Low; NCS = Not Clinically Significant; CS = Clinically Significant.

Listing 16.2.8.1a
Individual Haematology Results

Rando Number	Challenge Agent	Parameter (unit) [Normal Range]	Visit	Date of Assessment	Time of Assessment	Day Relative to Inoculum	Day Relative to IP	Result	Range Indicator	Clinical Assessment
R207	K13	Leukocytes (10^9/L) [3.5 – 10]	Screening	14-FEB-2018	11:34	-5	-14	6.5		
			Safety Visit	16-FEB-2018	08:46	-3	-12	7.8		
			Admission	28-FEB-2018	12:00	9	1	5.7		
			Safety Monitoring	02-MAR-2018	12:59	11	3	3.5		
			Admission 3	03-MAR-2018	13:00	12	4	4.7		
			Safety Monitoring	05-MAR-2018	10:01	14	6	5.1		
			Safety Monitoring	09-MAR-2018	09:33	18	10	6.6		
			Safety Monitoring	19-MAR-2018	09:12	28	20	6.4		
			End of Study	22-MAR-2018	09:32	31	23	6.4		
		Lymphocytes (10^9/L) [1 – 4]	Screening	14-FEB-2018	11:34	-5	-14	1.54		
			Safety Visit	16-FEB-2018	08:46	-3	-12	2.08		
			Admission	28-FEB-2018	12:00	9	1	1.16		
			Safety Monitoring	02-MAR-2018	12:59	11	3	0.68	L	CS
			Admission 3	03-MAR-2018	13:00	12	4	0.97	L	NCS
			Safety Monitoring	05-MAR-2018	10:01	14	6	1.44		
			Safety Monitoring	09-MAR-2018	09:33	18	10	1.53		
			Safety Monitoring	19-MAR-2018	09:12	28	20	1.84		
			End of Study	22-MAR-2018	09:32	31	23	1.27		

Notes: Day relative to inoculum is the number of days relative to day of administration of the inoculum, where the day of administration = 0.
The inoculum was administered on Study Day 0 for K13-infected subjects and Study Day 1 for 3D7-infected subjects.
Day relative to IP is the number of days relative to day of administration of Artesunate, where the day of dose = 1.
Rando = Randomisation; IP = Artesunate; H = High; L = Low; NCS = Not Clinically Significant; CS = Clinically Significant.

Listing 16.2.8.1a
Individual Haematology Results

Rando Number	Challenge Agent	Parameter (unit) [Normal Range]	Visit	Date of Assessment	Time of Assessment	Day Relative to Inoculum	Day Relative to IP	Result	Range Indicator	Clinical Assessment
R207	K13	Metamyelocytes (10^9/L) [0 – 0.01]	Safety Monitoring	09-MAR-2018	09:33	18	10	0.20	H	NCS
			End of Study	22-MAR-2018	09:32	31	23	0.32	H	NCS
		Monocytes (10^9/L) [0 – 0.9]	Screening	14-FEB-2018	11:34	-5	-14	0.59		
			Safety Visit	16-FEB-2018	08:46	-3	-12	0.73		
			Admission	28-FEB-2018	12:00	9	1	0.59		
			Safety Monitoring	02-MAR-2018	12:59	11	3	0.48		
			Admission 3	03-MAR-2018	13:00	12	4	0.56		
			Safety Monitoring	05-MAR-2018	10:01	14	6	0.62		
			Safety Monitoring	09-MAR-2018	09:33	18	10	0.66		
			Safety Monitoring	19-MAR-2018	09:12	28	20	0.45		
			End of Study	22-MAR-2018	09:32	31	23	0.44		
		Myelocytes (10^9/L) [0 – 0.01]	Safety Monitoring	09-MAR-2018	09:33	18	10	0.07	H	NCS

Notes: Day relative to inoculum is the number of days relative to day of administration of the inoculum, where the day of administration = 0.
The inoculum was administered on Study Day 0 for K13-infected subjects and Study Day 1 for 3D7-infected subjects.
Day relative to IP is the number of days relative to day of administration of Artesunate, where the day of dose = 1.
Rando = Randomisation; IP = Artesunate; H = High; L = Low; NCS = Not Clinically Significant; CS = Clinically Significant.

Listing 16.2.8.1a
Individual Haematology Results

Rando Number	Challenge Agent	Parameter (unit) [Normal Range]	Visit	Date of Assessment	Time of Assessment	Day Relative to Inoculum	Day Relative to IP	Result	Range Indicator	Clinical Assessment
R207	K13	Neutrophils (10^9/L) [1.5 – 6.5]	Screening	14-FEB-2018	11:34	-5	-14	4.15		
			Safety Visit	16-FEB-2018	08:46	-3	-12	4.75		
			Admission	28-FEB-2018	12:00	9	1	3.76		
			Safety Monitoring	02-MAR-2018	12:59	11	3	2.28		
			Admission 3	03-MAR-2018	13:00	12	4	2.98		
			Safety Monitoring	05-MAR-2018	10:01	14	6	2.83		
			Safety Monitoring	09-MAR-2018	09:33	18	10	4.05		
			Safety Monitoring	19-MAR-2018	09:12	28	20	3.61		
			End of Study	22-MAR-2018	09:32	31	23	3.62		
R207		Platelets (10^9/L) [150 – 400]	Screening	14-FEB-2018	11:34	-5	-14	220		
			Safety Visit	16-FEB-2018	08:46	-3	-12	215		
			Admission	28-FEB-2018	12:00	9	1	199		
			Safety Monitoring	02-MAR-2018	12:59	11	3	162		
			Admission 3	03-MAR-2018	13:00	12	4	155		
			Safety Monitoring	05-MAR-2018	10:01	14	6	168		
			Safety Monitoring	09-MAR-2018	09:33	18	10	248		
			Safety Monitoring	19-MAR-2018	09:12	28	20	242		
			End of Study	22-MAR-2018	09:32	31	23	255		

Notes: Day relative to inoculum is the number of days relative to day of administration of the inoculum, where the day of administration = 0.
The inoculum was administered on Study Day 0 for K13-infected subjects and Study Day 1 for 3D7-infected subjects.
Day relative to IP is the number of days relative to day of administration of Artesunate, where the day of dose = 1.
Rando = Randomisation; IP = Artesunate; H = High; L = Low; NCS = Not Clinically Significant; CS = Clinically Significant.

Listing 16.2.8.1a
Individual Haematology Results

Rando Number	Challenge Agent	Parameter (unit) [Normal Range]	Visit	Date of Assessment	Time of Assessment	Day Relative to Inoculum	Day Relative to IP	Result	Range Indicator	Clinical Assessment
R207	K13	Reticulocytes (10^9/L) [25 – 120]	Safety Visit	16-FEB-2018	08:46	-3	-12	96		
			End of Study	22-MAR-2018	09:32	31	23	114		
R208	3D7	Basophils (10^9/L) [0 – 0.15]	Screening	01-FEB-2018	12:12	-19	-27	0.03		
			Safety Visit	16-FEB-2018	07:18	-4	-12	0.04		
			Admission	28-FEB-2018	11:13	8	1	0.01		
			Admission 3	03-MAR-2018	11:36	11	4	0.02		
			Safety Monitoring	05-MAR-2018	07:07	13	6	0.02		
			Safety Monitoring	09-MAR-2018	07:19	17	10	0.03		
			Safety Monitoring	10-MAR-2018	07:52	18	11	0.03		
			Safety Monitoring	13-MAR-2018	07:22	21	14	0.03		
			Safety Monitoring	19-MAR-2018	07:01	27	20	0.05		

Notes: Day relative to inoculum is the number of days relative to day of administration of the inoculum, where the day of administration = 0.
The inoculum was administered on Study Day 0 for K13-infected subjects and Study Day 1 for 3D7-infected subjects.
Day relative to IP is the number of days relative to day of administration of Artesunate, where the day of dose = 1.
Rando = Randomisation; IP = Artesunate; H = High; L = Low; NCS = Not Clinically Significant; CS = Clinically Significant.

Listing 16.2.8.1a
Individual Haematology Results

Rando Number	Challenge Agent	Parameter (unit) [Normal Range]	Visit	Date of Assessment	Time of Assessment	Day Relative to Inoculum	Day Relative to IP	Result	Range Indicator	Clinical Assessment
R208	3D7	Eosinophils (10^9/L) [0 – 0.6]	Screening	01-FEB-2018	12:12	-19	-27	0.16		
			Safety Visit	16-FEB-2018	07:18	-4	-12	0.23		
			Admission	28-FEB-2018	11:13	8	1	0.03		
			Admission 3	03-MAR-2018	11:36	11	4	0.14		
			Safety Monitoring	05-MAR-2018	07:07	13	6	0.18		
			Safety Monitoring	09-MAR-2018	07:19	17	10	0.17		
			Safety Monitoring	10-MAR-2018	07:52	18	11	0.08		
			Safety Monitoring	13-MAR-2018	07:22	21	14	0.11		
			Safety Monitoring	19-MAR-2018	07:01	27	20	0.16		
			End of Study	22-MAR-2018	07:50	30	23	0.29		

Notes: Day relative to inoculum is the number of days relative to day of administration of the inoculum, where the day of administration = 0.
The inoculum was administered on Study Day 0 for K13-infected subjects and Study Day 1 for 3D7-infected subjects.
Day relative to IP is the number of days relative to day of administration of Artesunate, where the day of dose = 1.
Rando = Randomisation; IP = Artesunate; H = High; L = Low; NCS = Not Clinically Significant; CS = Clinically Significant.

Listing 16.2.8.1a
Individual Haematology Results

Rando Number	Challenge Agent	Parameter (unit) [Normal Range]	Visit	Date of Assessment	Time of Assessment	Day Relative to Inoculum	Day Relative to IP	Result	Range Indicator	Clinical Assessment
R208	3D7	Ery. Mean Corpuscular Volume (fL) [80 – 100]	Screening	01-FEB-2018	12:12	-19	-27	90		
			Safety Visit	16-FEB-2018	07:18	-4	-12	91		
			Admission	28-FEB-2018	11:13	8	1	89		
			Admission 3	03-MAR-2018	11:36	11	4	87		
			Safety Monitoring	05-MAR-2018	07:07	13	6	89		
			Safety Monitoring	09-MAR-2018	07:19	17	10	90		
			Safety Monitoring	10-MAR-2018	07:52	18	11	89		
			Safety Monitoring	13-MAR-2018	07:22	21	14	90		
			Safety Monitoring	19-MAR-2018	07:01	27	20	91		
			End of Study	22-MAR-2018	07:50	30	23	89		

Notes: Day relative to inoculum is the number of days relative to day of administration of the inoculum, where the day of administration = 0.
The inoculum was administered on Study Day 0 for K13-infected subjects and Study Day 1 for 3D7-infected subjects.
Day relative to IP is the number of days relative to day of administration of Artesunate, where the day of dose = 1.
Rando = Randomisation; IP = Artesunate; H = High; L = Low; NCS = Not Clinically Significant; CS = Clinically Significant.

Listing 16.2.8.1a
Individual Haematology Results

Rando Number	Challenge Agent	Parameter (unit) [Normal Range]	Visit	Date of Assessment	Time of Assessment	Day Relative to Inoculum	Day Relative to IP	Result	Range Indicator	Clinical Assessment
R208	3D7	Erythrocytes (10^12/L) [3.9 – 5.6]	Screening	01-FEB-2018	12:12	-19	-27	4.6		
			Safety Visit	16-FEB-2018	07:18	-4	-12	4.5		
			Admission	28-FEB-2018	11:13	8	1	4.3		
			Admission 3	03-MAR-2018	11:36	11	4	3.8	L	NCS
			Safety Monitoring	05-MAR-2018	07:07	13	6	4.0		
			Safety Monitoring	09-MAR-2018	07:19	17	10	4.3		
			Safety Monitoring	10-MAR-2018	07:52	18	11	4.3		
			Safety Monitoring	13-MAR-2018	07:22	21	14	3.8	L	NCS
			Safety Monitoring	19-MAR-2018	07:01	27	20	4.0		
			End of Study	22-MAR-2018	07:50	30	23	4.2		

Notes: Day relative to inoculum is the number of days relative to day of administration of the inoculum, where the day of administration = 0.
The inoculum was administered on Study Day 0 for K13-infected subjects and Study Day 1 for 3D7-infected subjects.
Day relative to IP is the number of days relative to day of administration of Artesunate, where the day of dose = 1.
Rando = Randomisation; IP = Artesunate; H = High; L = Low; NCS = Not Clinically Significant; CS = Clinically Significant.

Listing 16.2.8.1a
Individual Haematology Results

Rando Number	Challenge Agent	Parameter (unit) [Normal Range]	Visit	Date of Assessment	Time of Assessment	Day Relative to Inoculum	Day Relative to IP	Result	Range Indicator	Clinical Assessment
R208	3D7	Hematocrit [0.35 – 0.47]	Screening	01-FEB-2018	12:12	-19	-27	0.42		
			Safety Visit	16-FEB-2018	07:18	-4	-12	0.41		
			Admission	28-FEB-2018	11:13	8	1	0.38		
			Admission 3	03-MAR-2018	11:36	11	4	0.33	L	NCS
			Safety Monitoring	05-MAR-2018	07:07	13	6	0.35		
			Safety Monitoring	09-MAR-2018	07:19	17	10	0.39		
			Safety Monitoring	10-MAR-2018	07:52	18	11	0.38		
			Safety Monitoring	13-MAR-2018	07:22	21	14	0.34	L	NCS
			Safety Monitoring	19-MAR-2018	07:01	27	20	0.36		
			End of Study	22-MAR-2018	07:50	30	23	0.37		

Notes: Day relative to inoculum is the number of days relative to day of administration of the inoculum, where the day of administration = 0.
The inoculum was administered on Study Day 0 for K13-infected subjects and Study Day 1 for 3D7-infected subjects.
Day relative to IP is the number of days relative to day of administration of Artesunate, where the day of dose = 1.
Rando = Randomisation; IP = Artesunate; H = High; L = Low; NCS = Not Clinically Significant; CS = Clinically Significant.

Listing 16.2.8.1a
Individual Haematology Results

Rando Number	Challenge Agent	Parameter (unit) [Normal Range]	Visit	Date of Assessment	Time of Assessment	Day Relative to Inoculum	Day Relative to IP	Result	Range Indicator	Clinical Assessment
R208	3D7	Hemoglobin (g/L) [115 – 165]	Screening	01-FEB-2018	12:12	-19	-27	144		
			Safety Visit	16-FEB-2018	07:18	-4	-12	141		
			Admission	28-FEB-2018	11:13	8	1	134		
			Admission 3	03-MAR-2018	11:36	11	4	115		
			Safety Monitoring	05-MAR-2018	07:07	13	6	123		
			Safety Monitoring	09-MAR-2018	07:19	17	10	131		
			Safety Monitoring	10-MAR-2018	07:52	18	11	129		
			Safety Monitoring	13-MAR-2018	07:22	21	14	115		
			Safety Monitoring	19-MAR-2018	07:01	27	20	125		
			End of Study	22-MAR-2018	07:50	30	23	131		

Notes: Day relative to inoculum is the number of days relative to day of administration of the inoculum, where the day of administration = 0.
The inoculum was administered on Study Day 0 for K13-infected subjects and Study Day 1 for 3D7-infected subjects.
Day relative to IP is the number of days relative to day of administration of Artesunate, where the day of dose = 1.
Rando = Randomisation; IP = Artesunate; H = High; L = Low; NCS = Not Clinically Significant; CS = Clinically Significant.

Listing 16.2.8.1a
Individual Haematology Results

Rando Number	Challenge Agent	Parameter (unit) [Normal Range]	Visit	Date of Assessment	Time of Assessment	Day Relative to Inoculum	Day Relative to IP	Result	Range Indicator	Clinical Assessment
R208	3D7	Leukocytes (10^9/L) [3.5 – 12]	Screening	01-FEB-2018	12:12	-19	-27	9.1		
			Safety Visit	16-FEB-2018	07:18	-4	-12	8.7		
			Admission	28-FEB-2018	11:13	8	1	4.6		
			Admission 3	03-MAR-2018	11:36	11	4	4.4		
			Safety Monitoring	05-MAR-2018	07:07	13	6	5.7		
			Safety Monitoring	09-MAR-2018	07:19	17	10	7.3		
			Safety Monitoring	10-MAR-2018	07:52	18	11	5.0		
			Safety Monitoring	13-MAR-2018	07:22	21	14	5.1		
			Safety Monitoring	19-MAR-2018	07:01	27	20	7.1		
			End of Study	22-MAR-2018	07:50	30	23	7.3		

Notes: Day relative to inoculum is the number of days relative to day of administration of the inoculum, where the day of administration = 0.
The inoculum was administered on Study Day 0 for K13-infected subjects and Study Day 1 for 3D7-infected subjects.
Day relative to IP is the number of days relative to day of administration of Artesunate, where the day of dose = 1.
Rando = Randomisation; IP = Artesunate; H = High; L = Low; NCS = Not Clinically Significant; CS = Clinically Significant.

Listing 16.2.8.1a
Individual Haematology Results

Rando Number	Challenge Agent	Parameter (unit) [Normal Range]	Visit	Date of Assessment	Time of Assessment	Day Relative to Inoculum	Day Relative to IP	Result	Range Indicator	Clinical Assessment
R208	3D7	Lymphocytes (10^9/L) [1 – 4]	Screening	01-FEB-2018	12:12	-19	-27	2.96		
			Safety Visit	16-FEB-2018	07:18	-4	-12	3.09		
			Admission	28-FEB-2018	11:13	8	1	1.34		
			Admission 3	03-MAR-2018	11:36	11	4	1.89		
			Safety Monitoring	05-MAR-2018	07:07	13	6	1.87		
			Safety Monitoring	09-MAR-2018	07:19	17	10	2.13		
			Safety Monitoring	10-MAR-2018	07:52	18	11	1.24		
			Safety Monitoring	13-MAR-2018	07:22	21	14	1.99		
			Safety Monitoring	19-MAR-2018	07:01	27	20	2.72		
			End of Study	22-MAR-2018	07:50	30	23	2.35		
		Metamyelocytes (10^9/L) [0 – 0.01]	End of Study	22-MAR-2018	07:50	30	23	0.22	H	NCS

Notes: Day relative to inoculum is the number of days relative to day of administration of the inoculum, where the day of administration = 0.
The inoculum was administered on Study Day 0 for K13-infected subjects and Study Day 1 for 3D7-infected subjects.
Day relative to IP is the number of days relative to day of administration of Artesunate, where the day of dose = 1.
Rando = Randomisation; IP = Artesunate; H = High; L = Low; NCS = Not Clinically Significant; CS = Clinically Significant.

Listing 16.2.8.1a
Individual Haematology Results

Rando Number	Challenge Agent	Parameter (unit) [Normal Range]	Visit	Date of Assessment	Time of Assessment	Day Relative to Inoculum	Day Relative to IP	Result	Range Indicator	Clinical Assessment
R208	3D7	Monocytes (10^9/L) [0 – 0.9]	Screening	01-FEB-2018	12:12	-19	-27	0.70		
			Safety Visit	16-FEB-2018	07:18	-4	-12	0.66		
			Admission	28-FEB-2018	11:13	8	1	0.52		
			Admission 3	03-MAR-2018	11:36	11	4	0.58		
			Safety Monitoring	05-MAR-2018	07:07	13	6	0.51		
			Safety Monitoring	09-MAR-2018	07:19	17	10	0.60		
			Safety Monitoring	10-MAR-2018	07:52	18	11	0.51		
			Safety Monitoring	13-MAR-2018	07:22	21	14	0.49		
			Safety Monitoring	19-MAR-2018	07:01	27	20	0.53		
			End of Study	22-MAR-2018	07:50	30	23	0.44		
		Myelocytes (10^9/L) [0 – 0.01]	End of Study	22-MAR-2018	07:50	30	23	0.15	H	NCS

Notes: Day relative to inoculum is the number of days relative to day of administration of the inoculum, where the day of administration = 0.
The inoculum was administered on Study Day 0 for K13-infected subjects and Study Day 1 for 3D7-infected subjects.
Day relative to IP is the number of days relative to day of administration of Artesunate, where the day of dose = 1.
Rando = Randomisation; IP = Artesunate; H = High; L = Low; NCS = Not Clinically Significant; CS = Clinically Significant.

Listing 16.2.8.1a
Individual Haematology Results

Rando Number	Challenge Agent	Parameter (unit) [Normal Range]	Visit	Date of Assessment	Time of Assessment	Day Relative to Inoculum	Day Relative to IP	Result	Range Indicator	Clinical Assessment
R208	3D7	Neutrophils (10^9/L) [1.5 – 8]	Screening	01-FEB-2018	12:12	-19	-27	5.24		
			Safety Visit	16-FEB-2018	07:18	-4	-12	4.71		
			Admission	28-FEB-2018	11:13	8	1	2.69		
			Admission 3	03-MAR-2018	11:36	11	4	1.80		
			Safety Monitoring	05-MAR-2018	07:07	13	6	3.13		
			Safety Monitoring	09-MAR-2018	07:19	17	10	4.34		
			Safety Monitoring	10-MAR-2018	07:52	18	11	3.09		
			Safety Monitoring	13-MAR-2018	07:22	21	14	2.51		
			Safety Monitoring	19-MAR-2018	07:01	27	20	3.62		
			End of Study	22-MAR-2018	07:50	30	23	3.89		

Notes: Day relative to inoculum is the number of days relative to day of administration of the inoculum, where the day of administration = 0.
The inoculum was administered on Study Day 0 for K13-infected subjects and Study Day 1 for 3D7-infected subjects.
Day relative to IP is the number of days relative to day of administration of Artesunate, where the day of dose = 1.
Rando = Randomisation; IP = Artesunate; H = High; L = Low; NCS = Not Clinically Significant; CS = Clinically Significant.

Listing 16.2.8.1a
Individual Haematology Results

Rando Number	Challenge Agent	Parameter (unit) [Normal Range]	Visit	Date of Assessment	Time of Assessment	Day Relative to Inoculum	Day Relative to IP	Result	Range Indicator	Clinical Assessment
R208	3D7	Platelets (10^9/L) [150 – 400]	Screening	01-FEB-2018	12:12	-19	-27	269		
			Safety Visit	16-FEB-2018	07:18	-4	-12	255		
			Admission	28-FEB-2018	11:13	8	1	185		
			Admission 3	03-MAR-2018	11:36	11	4	146	L	NCS
			Safety Monitoring	05-MAR-2018	07:07	13	6	195		
			Safety Monitoring	09-MAR-2018	07:19	17	10	268		
			Safety Monitoring	10-MAR-2018	07:52	18	11	236		
			Safety Monitoring	13-MAR-2018	07:22	21	14	208		
			Safety Monitoring	19-MAR-2018	07:01	27	20	261		
			End of Study	22-MAR-2018	07:50	30	23	276		
		Reticulocytes (10^9/L) [25 – 120]	Safety Visit	16-FEB-2018	07:18	-4	-12	102		
			End of Study	22-MAR-2018	07:50	30	23	126	H	NCS

Notes: Day relative to inoculum is the number of days relative to day of administration of the inoculum, where the day of administration = 0.
The inoculum was administered on Study Day 0 for K13-infected subjects and Study Day 1 for 3D7-infected subjects.
Day relative to IP is the number of days relative to day of administration of Artesunate, where the day of dose = 1.
Rando = Randomisation; IP = Artesunate; H = High; L = Low; NCS = Not Clinically Significant; CS = Clinically Significant.

Listing 16.2.8.1a
Individual Haematology Results

Rando Number	Challenge Agent	Parameter (unit) [Normal Range]	Visit	Date of Assessment	Time of Assessment	Day Relative to Inoculum	Day Relative to IP	Result	Range Indicator	Clinical Assessment
R209	3D7	Basophils (10^9/L) [0 – 0.15]	Screening	08-FEB-2018	10:36	-12	-20	0.04		
			Safety Visit	16-FEB-2018	07:42	-4	-12	0.05		
			Admission	28-FEB-2018	10:16	8	1	0.02		
			Admission	28-FEB-2018	14:42	8	1	0.04		
			Admission 3	03-MAR-2018	15:47	11	4	0.03		
			Safety Monitoring	05-MAR-2018	07:41	13	6	0.03		
			Safety Monitoring	08-MAR-2018	08:42	16	9	0.04		
			Safety Monitoring	12-MAR-2018	08:12	20	13	0.04		
			Safety Monitoring	19-MAR-2018	07:48	27	20	0.04		
			End of Study	22-MAR-2018	08:16	30	23	0.04		

Notes: Day relative to inoculum is the number of days relative to day of administration of the inoculum, where the day of administration = 0.
The inoculum was administered on Study Day 0 for K13-infected subjects and Study Day 1 for 3D7-infected subjects.
Day relative to IP is the number of days relative to day of administration of Artesunate, where the day of dose = 1.
Rando = Randomisation; IP = Artesunate; H = High; L = Low; NCS = Not Clinically Significant; CS = Clinically Significant.

Listing 16.2.8.1a
Individual Haematology Results

Rando Number	Challenge Agent	Parameter (unit) [Normal Range]	Visit	Date of Assessment	Time of Assessment	Day Relative to Inoculum	Day Relative to IP	Result	Range Indicator	Clinical Assessment
R209	3D7	Eosinophils (10^9/L) [0 – 0.6]	Screening	08-FEB-2018	10:36	-12	-20	0.08		
			Safety Visit	16-FEB-2018	07:42	-4	-12	0.17		
			Admission	28-FEB-2018	10:16	8	1	0.06		
			Admission	28-FEB-2018	14:42	8	1	0.07		
			Admission 3	03-MAR-2018	15:47	11	4	0.12		
			Safety Monitoring	05-MAR-2018	07:41	13	6	0.13		
			Safety Monitoring	08-MAR-2018	08:42	16	9	0.11		
			Safety Monitoring	12-MAR-2018	08:12	20	13	0.08		
			Safety Monitoring	19-MAR-2018	07:48	27	20	0.07		
			End of Study	22-MAR-2018	08:16	30	23	0.07		

Notes: Day relative to inoculum is the number of days relative to day of administration of the inoculum, where the day of administration = 0.
The inoculum was administered on Study Day 0 for K13-infected subjects and Study Day 1 for 3D7-infected subjects.
Day relative to IP is the number of days relative to day of administration of Artesunate, where the day of dose = 1.
Rando = Randomisation; IP = Artesunate; H = High; L = Low; NCS = Not Clinically Significant; CS = Clinically Significant.

Listing 16.2.8.1a
Individual Haematology Results

Rando Number	Challenge Agent	Parameter (unit) [Normal Range]	Visit	Date of Assessment	Time of Assessment	Day Relative to Inoculum	Day Relative to IP	Result	Range Indicator	Clinical Assessment
R209	3D7	Ery. Mean Corpuscular Volume (fL) [80 – 100]	Screening	08-FEB-2018	10:36	-12	-20	86		
			Safety Visit	16-FEB-2018	07:42	-4	-12	87		
			Admission	28-FEB-2018	10:16	8	1	84		
			Admission	28-FEB-2018	14:42	8	1	86		
			Admission 3	03-MAR-2018	15:47	11	4	86		
			Safety Monitoring	05-MAR-2018	07:41	13	6	86		
			Safety Monitoring	08-MAR-2018	08:42	16	9	87		
			Safety Monitoring	12-MAR-2018	08:12	20	13	88		
			Safety Monitoring	19-MAR-2018	07:48	27	20	88		
			End of Study	22-MAR-2018	08:16	30	23	87		

Notes: Day relative to inoculum is the number of days relative to day of administration of the inoculum, where the day of administration = 0.
The inoculum was administered on Study Day 0 for K13-infected subjects and Study Day 1 for 3D7-infected subjects.
Day relative to IP is the number of days relative to day of administration of Artesunate, where the day of dose = 1.
Rando = Randomisation; IP = Artesunate; H = High; L = Low; NCS = Not Clinically Significant; CS = Clinically Significant.

Listing 16.2.8.1a
Individual Haematology Results

Rando Number	Challenge Agent	Parameter (unit) [Normal Range]	Visit	Date of Assessment	Time of Assessment	Day Relative to Inoculum	Day Relative to IP	Result	Range Indicator	Clinical Assessment
R209	3D7	Erythrocytes (10^12/L) [4.5 – 6.5]	Screening	08-FEB-2018	10:36	-12	-20	5.2		
			Safety Visit	16-FEB-2018	07:42	-4	-12	5.2		
			Admission	28-FEB-2018	10:16	8	1	4.9		
			Admission	28-FEB-2018	14:42	8	1	5.1		
			Admission 3	03-MAR-2018	15:47	11	4	5.0		
			Safety Monitoring	05-MAR-2018	07:41	13	6	4.4	L	NCS
			Safety Monitoring	08-MAR-2018	08:42	16	9	4.6		
			Safety Monitoring	12-MAR-2018	08:12	20	13	4.5		
			Safety Monitoring	19-MAR-2018	07:48	27	20	4.5		
			End of Study	22-MAR-2018	08:16	30	23	4.5		

Notes: Day relative to inoculum is the number of days relative to day of administration of the inoculum, where the day of administration = 0.
The inoculum was administered on Study Day 0 for K13-infected subjects and Study Day 1 for 3D7-infected subjects.
Day relative to IP is the number of days relative to day of administration of Artesunate, where the day of dose = 1.
Rando = Randomisation; IP = Artesunate; H = High; L = Low; NCS = Not Clinically Significant; CS = Clinically Significant.

Listing 16.2.8.1a
Individual Haematology Results

Rando Number	Challenge Agent	Parameter (unit) [Normal Range]	Visit	Date of Assessment	Time of Assessment	Day Relative to Inoculum	Day Relative to IP	Result	Range Indicator	Clinical Assessment
R209	3D7	Hematocrit [0.4 – 0.54]	Screening	08-FEB-2018	10:36	-12	-20	0.45		
			Safety Visit	16-FEB-2018	07:42	-4	-12	0.45		
			Admission	28-FEB-2018	10:16	8	1	0.41		
			Admission	28-FEB-2018	14:42	8	1	0.44		
			Admission 3	03-MAR-2018	15:47	11	4	0.43		
			Safety Monitoring	05-MAR-2018	07:41	13	6	0.38	L	NCS
			Safety Monitoring	08-MAR-2018	08:42	16	9	0.40		
			Safety Monitoring	12-MAR-2018	08:12	20	13	0.39	L	NCS
			Safety Monitoring	19-MAR-2018	07:48	27	20	0.40		
			End of Study	22-MAR-2018	08:16	30	23	0.39	L	NCS

Notes: Day relative to inoculum is the number of days relative to day of administration of the inoculum, where the day of administration = 0.
The inoculum was administered on Study Day 0 for K13-infected subjects and Study Day 1 for 3D7-infected subjects.
Day relative to IP is the number of days relative to day of administration of Artesunate, where the day of dose = 1.
Rando = Randomisation; IP = Artesunate; H = High; L = Low; NCS = Not Clinically Significant; CS = Clinically Significant.

Listing 16.2.8.1a
Individual Haematology Results

Rando Number	Challenge Agent	Parameter (unit) [Normal Range]	Visit	Date of Assessment	Time of Assessment	Day Relative to Inoculum	Day Relative to IP	Result	Range Indicator	Clinical Assessment
R209	3D7	Hemoglobin (g/L) [135 – 175]	Screening	08-FEB-2018	10:36	-12	-20	155		
			Safety Visit	16-FEB-2018	07:42	-4	-12	153		
			Admission	28-FEB-2018	10:16	8	1	144		
			Admission	28-FEB-2018	14:42	8	1	151		
			Admission 3	03-MAR-2018	15:47	11	4	146		
			Safety Monitoring	05-MAR-2018	07:41	13	6	127	L	NCS
			Safety Monitoring	08-MAR-2018	08:42	16	9	134	L	NCS
			Safety Monitoring	12-MAR-2018	08:12	20	13	128	L	NCS
			Safety Monitoring	19-MAR-2018	07:48	27	20	134	L	NCS
			End of Study	22-MAR-2018	08:16	30	23	135		

Notes: Day relative to inoculum is the number of days relative to day of administration of the inoculum, where the day of administration = 0.
The inoculum was administered on Study Day 0 for K13-infected subjects and Study Day 1 for 3D7-infected subjects.
Day relative to IP is the number of days relative to day of administration of Artesunate, where the day of dose = 1.
Rando = Randomisation; IP = Artesunate; H = High; L = Low; NCS = Not Clinically Significant; CS = Clinically Significant.

Listing 16.2.8.1a
Individual Haematology Results

Rando Number	Challenge Agent	Parameter (unit) [Normal Range]	Visit	Date of Assessment	Time of Assessment	Day Relative to Inoculum	Day Relative to IP	Result	Range Indicator	Clinical Assessment
R209	3D7	Leukocytes (10^9/L) [3.5 – 10]	Screening	08-FEB-2018	10:36	-12	-20	7.0		
			Safety Visit	16-FEB-2018	07:42	-4	-12	8.1		
			Admission	28-FEB-2018	10:16	8	1	5.6		
			Admission	28-FEB-2018	14:42	8	1	5.7		
			Admission 3	03-MAR-2018	15:47	11	4	5.5		
			Safety Monitoring	05-MAR-2018	07:41	13	6	5.6		
			Safety Monitoring	08-MAR-2018	08:42	16	9	6.7		
			Safety Monitoring	12-MAR-2018	08:12	20	13	5.8		
			Safety Monitoring	19-MAR-2018	07:48	27	20	5.9		
			End of Study	22-MAR-2018	08:16	30	23	5.6		

Notes: Day relative to inoculum is the number of days relative to day of administration of the inoculum, where the day of administration = 0.
The inoculum was administered on Study Day 0 for K13-infected subjects and Study Day 1 for 3D7-infected subjects.
Day relative to IP is the number of days relative to day of administration of Artesunate, where the day of dose = 1.
Rando = Randomisation; IP = Artesunate; H = High; L = Low; NCS = Not Clinically Significant; CS = Clinically Significant.

Listing 16.2.8.1a
Individual Haematology Results

Rando Number	Challenge Agent	Parameter (unit) [Normal Range]	Visit	Date of Assessment	Time of Assessment	Day Relative to Inoculum	Day Relative to IP	Result	Range Indicator	Clinical Assessment
R209	3D7	Lymphocytes (10^9/L) [1 – 4]	Screening	08-FEB-2018	10:36	-12	-20	2.59		
			Safety Visit	16-FEB-2018	07:42	-4	-12	2.61		
			Admission	28-FEB-2018	10:16	8	1	1.34		
			Admission	28-FEB-2018	14:42	8	1	1.43		
			Admission 3	03-MAR-2018	15:47	11	4	1.98		
			Safety Monitoring	05-MAR-2018	07:41	13	6	2.46		
			Safety Monitoring	08-MAR-2018	08:42	16	9	1.31		
			Safety Monitoring	12-MAR-2018	08:12	20	13	2.58		
			Safety Monitoring	19-MAR-2018	07:48	27	20	2.33		
			End of Study	22-MAR-2018	08:16	30	23	2.43		

Notes: Day relative to inoculum is the number of days relative to day of administration of the inoculum, where the day of administration = 0.
The inoculum was administered on Study Day 0 for K13-infected subjects and Study Day 1 for 3D7-infected subjects.
Day relative to IP is the number of days relative to day of administration of Artesunate, where the day of dose = 1.
Rando = Randomisation; IP = Artesunate; H = High; L = Low; NCS = Not Clinically Significant; CS = Clinically Significant.

Listing 16.2.8.1a
Individual Haematology Results

Rando Number	Challenge Agent	Parameter (unit) [Normal Range]	Visit	Date of Assessment	Time of Assessment	Day Relative to Inoculum	Day Relative to IP	Result	Range Indicator	Clinical Assessment
R209	3D7	Monocytes (10^9/L) [0 – 0.9]	Screening	08-FEB-2018	10:36	-12	-20	0.56		
			Safety Visit	16-FEB-2018	07:42	-4	-12	0.72		
			Admission	28-FEB-2018	10:16	8	1	0.71		
			Admission	28-FEB-2018	14:42	8	1	0.84		
			Admission 3	03-MAR-2018	15:47	11	4	0.74		
			Safety Monitoring	05-MAR-2018	07:41	13	6	0.55		
			Safety Monitoring	08-MAR-2018	08:42	16	9	0.66		
			Safety Monitoring	12-MAR-2018	08:12	20	13	0.62		
			Safety Monitoring	19-MAR-2018	07:48	27	20	0.63		
			End of Study	22-MAR-2018	08:16	30	23	0.55		

Notes: Day relative to inoculum is the number of days relative to day of administration of the inoculum, where the day of administration = 0.
The inoculum was administered on Study Day 0 for K13-infected subjects and Study Day 1 for 3D7-infected subjects.
Day relative to IP is the number of days relative to day of administration of Artesunate, where the day of dose = 1.
Rando = Randomisation; IP = Artesunate; H = High; L = Low; NCS = Not Clinically Significant; CS = Clinically Significant.

Listing 16.2.8.1a
Individual Haematology Results

Rando Number	Challenge Agent	Parameter (unit) [Normal Range]	Visit	Date of Assessment	Time of Assessment	Day Relative to Inoculum	Day Relative to IP	Result	Range Indicator	Clinical Assessment
R209	3D7	Neutrophils (10^9/L) [1.5 – 6.5]	Screening	08-FEB-2018	10:36	-12	-20	3.70		
			Safety Visit	16-FEB-2018	07:42	-4	-12	4.51		
			Admission	28-FEB-2018	10:16	8	1	3.48		
			Admission	28-FEB-2018	14:42	8	1	3.30		
			Admission 3	03-MAR-2018	15:47	11	4	2.67		
			Safety Monitoring	05-MAR-2018	07:41	13	6	2.47		
			Safety Monitoring	08-MAR-2018	08:42	16	9	4.62		
			Safety Monitoring	12-MAR-2018	08:12	20	13	2.45		
			Safety Monitoring	19-MAR-2018	07:48	27	20	2.81		
			End of Study	22-MAR-2018	08:16	30	23	2.55		

Notes: Day relative to inoculum is the number of days relative to day of administration of the inoculum, where the day of administration = 0.
The inoculum was administered on Study Day 0 for K13-infected subjects and Study Day 1 for 3D7-infected subjects.
Day relative to IP is the number of days relative to day of administration of Artesunate, where the day of dose = 1.
Rando = Randomisation; IP = Artesunate; H = High; L = Low; NCS = Not Clinically Significant; CS = Clinically Significant.

Listing 16.2.8.1a
Individual Haematology Results

Rando Number	Challenge Agent	Parameter (unit) [Normal Range]	Visit	Date of Assessment	Time of Assessment	Day Relative to Inoculum	Day Relative to IP	Result	Range Indicator	Clinical Assessment
R209	3D7	Platelets (10^9/L) [150 – 400]	Screening	08-FEB-2018	10:36	-12	-20	245		
			Safety Visit	16-FEB-2018	07:42	-4	-12	253		
			Admission	28-FEB-2018	10:16	8	1	203		
			Admission	28-FEB-2018	14:42	8	1	235		
			Admission 3	03-MAR-2018	15:47	11	4	178		
			Safety Monitoring	05-MAR-2018	07:41	13	6	173		
			Safety Monitoring	08-MAR-2018	08:42	16	9	201		
			Safety Monitoring	12-MAR-2018	08:12	20	13	145	L	NCS
			Safety Monitoring	19-MAR-2018	07:48	27	20	194		
			End of Study	22-MAR-2018	08:16	30	23	229		
		Reticulocytes (10^9/L) [25 – 120]	Safety Visit	16-FEB-2018	07:42	-4	-12	44		
			End of Study	22-MAR-2018	08:16	30	23	69		

Notes: Day relative to inoculum is the number of days relative to day of administration of the inoculum, where the day of administration = 0.
The inoculum was administered on Study Day 0 for K13-infected subjects and Study Day 1 for 3D7-infected subjects.
Day relative to IP is the number of days relative to day of administration of Artesunate, where the day of dose = 1.
Rando = Randomisation; IP = Artesunate; H = High; L = Low; NCS = Not Clinically Significant; CS = Clinically Significant.

Listing 16.2.8.1a
Individual Haematology Results

Rando Number	Challenge Agent	Parameter (unit) [Normal Range]	Visit	Date of Assessment	Time of Assessment	Day Relative to Inoculum	Day Relative to IP	Result	Range Indicator	Clinical Assessment
R210	3D7	Basophils (10^9/L) [0 – 0.15]	Screening	12-FEB-2018	09:38	-8	-16	0.07		
			Safety Visit	16-FEB-2018	07:28	-4	-12	0.04		
			Admission	28-FEB-2018	11:21	8	1	0.05		
			Admission 3	03-MAR-2018	12:01	11	4	0.04		
			Safety Monitoring	05-MAR-2018	07:40	13	6	0.05		
			Safety Monitoring	08-MAR-2018	07:40	16	9	0.05		
			Safety Monitoring	12-MAR-2018	07:37	20	13	0.03		
			Safety Monitoring	19-MAR-2018	07:39	27	20	0.05		
			End of Study	22-MAR-2018	07:34	30	23	0.04		
		Eosinophils (10^9/L) [0 – 0.6]	Screening	12-FEB-2018	09:38	-8	-16	0.16		
			Safety Visit	16-FEB-2018	07:28	-4	-12	0.12		
			Admission	28-FEB-2018	11:21	8	1	0.15		
			Admission 3	03-MAR-2018	12:01	11	4	0.09		
			Safety Monitoring	05-MAR-2018	07:40	13	6	0.10		
			Safety Monitoring	08-MAR-2018	07:40	16	9	0.11		
			Safety Monitoring	12-MAR-2018	07:37	20	13	0.09		
			Safety Monitoring	19-MAR-2018	07:39	27	20	0.17		
			End of Study	22-MAR-2018	07:34	30	23	0.38		

Notes: Day relative to inoculum is the number of days relative to day of administration of the inoculum, where the day of administration = 0.
The inoculum was administered on Study Day 0 for K13-infected subjects and Study Day 1 for 3D7-infected subjects.
Day relative to IP is the number of days relative to day of administration of Artesunate, where the day of dose = 1.
Rando = Randomisation; IP = Artesunate; H = High; L = Low; NCS = Not Clinically Significant; CS = Clinically Significant.

Listing 16.2.8.1a
Individual Haematology Results

Rando Number	Challenge Agent	Parameter (unit) [Normal Range]	Visit	Date of Assessment	Time of Assessment	Day Relative to Inoculum	Day Relative to IP	Result	Range Indicator	Clinical Assessment
R210	3D7	Ery. Mean Corpuscular Volume (fL) [80 – 100]	Screening	12-FEB-2018	09:38	-8	-16	87		
			Safety Visit	16-FEB-2018	07:28	-4	-12	87		
			Admission	28-FEB-2018	11:21	8	1	88		
			Admission 3	03-MAR-2018	12:01	11	4	87		
			Safety Monitoring	05-MAR-2018	07:40	13	6	88		
			Safety Monitoring	08-MAR-2018	07:40	16	9	88		
			Safety Monitoring	12-MAR-2018	07:37	20	13	88		
			Safety Monitoring	19-MAR-2018	07:39	27	20	89		
			End of Study	22-MAR-2018	07:34	30	23	88		
R210		Erythrocytes (10^12/L) [3.9 – 5.6]	Screening	12-FEB-2018	09:38	-8	-16	4.2		
			Safety Visit	16-FEB-2018	07:28	-4	-12	3.9		
			Admission	28-FEB-2018	11:21	8	1	4.0		
			Admission 3	03-MAR-2018	12:01	11	4	4.1		
			Safety Monitoring	05-MAR-2018	07:40	13	6	4.1		
			Safety Monitoring	08-MAR-2018	07:40	16	9	4.0		
			Safety Monitoring	12-MAR-2018	07:37	20	13	4.0		
			Safety Monitoring	19-MAR-2018	07:39	27	20	3.8	L	NCS
			End of Study	22-MAR-2018	07:34	30	23	4.1		

Notes: Day relative to inoculum is the number of days relative to day of administration of the inoculum, where the day of administration = 0.
The inoculum was administered on Study Day 0 for K13-infected subjects and Study Day 1 for 3D7-infected subjects.
Day relative to IP is the number of days relative to day of administration of Artesunate, where the day of dose = 1.
Rando = Randomisation; IP = Artesunate; H = High; L = Low; NCS = Not Clinically Significant; CS = Clinically Significant.

Listing 16.2.8.1a
Individual Haematology Results

Rando Number	Challenge Agent	Parameter (unit) [Normal Range]	Visit	Date of Assessment	Time of Assessment	Day Relative to Inoculum	Day Relative to IP	Result	Range Indicator	Clinical Assessment
R210	3D7	Hematocrit [0.35 – 0.47]	Screening	12-FEB-2018	09:38	-8	-16	0.37		
			Safety Visit	16-FEB-2018	07:28	-4	-12	0.34	L	NCS
			Unscheduled	17-FEB-2018	08:15	-3	-11	0.37		
			Admission	28-FEB-2018	11:21	8	1	0.35		
			Admission 3	03-MAR-2018	12:01	11	4	0.36		
			Safety Monitoring	05-MAR-2018	07:40	13	6	0.36		
			Safety Monitoring	08-MAR-2018	07:40	16	9	0.35		
			Safety Monitoring	12-MAR-2018	07:37	20	13	0.35		
			Safety Monitoring	19-MAR-2018	07:39	27	20	0.34	L	NCS
			End of Study	22-MAR-2018	07:34	30	23	0.36		

Notes: Day relative to inoculum is the number of days relative to day of administration of the inoculum, where the day of administration = 0.
The inoculum was administered on Study Day 0 for K13-infected subjects and Study Day 1 for 3D7-infected subjects.
Day relative to IP is the number of days relative to day of administration of Artesunate, where the day of dose = 1.
Rando = Randomisation; IP = Artesunate; H = High; L = Low; NCS = Not Clinically Significant; CS = Clinically Significant.

Listing 16.2.8.1a
Individual Haematology Results

Rando Number	Challenge Agent	Parameter (unit) [Normal Range]	Visit	Date of Assessment	Time of Assessment	Day Relative to Inoculum	Day Relative to IP	Result	Range Indicator	Clinical Assessment
R210	3D7	Hemoglobin (g/L) [115 – 165]	Screening	12-FEB-2018	09:38	-8	-16	122		
			Safety Visit	16-FEB-2018	07:28	-4	-12	113	L	NCS
			Unscheduled	17-FEB-2018	08:15	-3	-11	122		
			Admission	28-FEB-2018	11:21	8	1	114	L	NCS
			Admission 3	03-MAR-2018	12:01	11	4	118		
			Safety Monitoring	05-MAR-2018	07:40	13	6	117		
			Safety Monitoring	08-MAR-2018	07:40	16	9	114	L	NCS
			Safety Monitoring	12-MAR-2018	07:37	20	13	113	L	NCS
			Safety Monitoring	19-MAR-2018	07:39	27	20	110	L	NCS
			End of Study	22-MAR-2018	07:34	30	23	120		
		Leukocytes (10^9/L) [3.5 – 12]	Screening	12-FEB-2018	09:38	-8	-16	6.4		
			Safety Visit	16-FEB-2018	07:28	-4	-12	5.3		
			Admission	28-FEB-2018	11:21	8	1	5.9		
			Admission 3	03-MAR-2018	12:01	11	4	3.8		
			Safety Monitoring	05-MAR-2018	07:40	13	6	4.8		
			Safety Monitoring	08-MAR-2018	07:40	16	9	5.0		
			Safety Monitoring	12-MAR-2018	07:37	20	13	5.3		
			Safety Monitoring	19-MAR-2018	07:39	27	20	6.3		
			End of Study	22-MAR-2018	07:34	30	23	5.8		

Notes: Day relative to inoculum is the number of days relative to day of administration of the inoculum, where the day of administration = 0.
The inoculum was administered on Study Day 0 for K13-infected subjects and Study Day 1 for 3D7-infected subjects.
Day relative to IP is the number of days relative to day of administration of Artesunate, where the day of dose = 1.
Rando = Randomisation; IP = Artesunate; H = High; L = Low; NCS = Not Clinically Significant; CS = Clinically Significant.

Listing 16.2.8.1a
Individual Haematology Results

Rando Number	Challenge Agent	Parameter (unit) [Normal Range]	Visit	Date of Assessment	Time of Assessment	Day Relative to Inoculum	Day Relative to IP	Result	Range Indicator	Clinical Assessment
R210	3D7	Lymphocytes (10^9/L) [1 – 4]	Screening	12-FEB-2018	09:38	-8	-16	2.31		
			Safety Visit	16-FEB-2018	07:28	-4	-12	1.87		
			Admission	28-FEB-2018	11:21	8	1	1.63		
			Admission 3	03-MAR-2018	12:01	11	4	1.57		
			Safety Monitoring	05-MAR-2018	07:40	13	6	2.06		
			Safety Monitoring	08-MAR-2018	07:40	16	9	1.12		
			Safety Monitoring	12-MAR-2018	07:37	20	13	2.13		
			Safety Monitoring	19-MAR-2018	07:39	27	20	1.97		
			End of Study	22-MAR-2018	07:34	30	23	1.73		
R210		Monocytes (10^9/L) [0 – 0.9]	Screening	12-FEB-2018	09:38	-8	-16	0.40		
			Safety Visit	16-FEB-2018	07:28	-4	-12	0.41		
			Admission	28-FEB-2018	11:21	8	1	0.40		
			Admission 3	03-MAR-2018	12:01	11	4	0.31		
			Safety Monitoring	05-MAR-2018	07:40	13	6	0.34		
			Safety Monitoring	08-MAR-2018	07:40	16	9	0.40		
			Safety Monitoring	12-MAR-2018	07:37	20	13	0.42		
			Safety Monitoring	19-MAR-2018	07:39	27	20	0.47		
			End of Study	22-MAR-2018	07:34	30	23	0.42		

Notes: Day relative to inoculum is the number of days relative to day of administration of the inoculum, where the day of administration = 0.
The inoculum was administered on Study Day 0 for K13-infected subjects and Study Day 1 for 3D7-infected subjects.
Day relative to IP is the number of days relative to day of administration of Artesunate, where the day of dose = 1.
Rando = Randomisation; IP = Artesunate; H = High; L = Low; NCS = Not Clinically Significant; CS = Clinically Significant.

Listing 16.2.8.1a
Individual Haematology Results

Rando Number	Challenge Agent	Parameter (unit) [Normal Range]	Visit	Date of Assessment	Time of Assessment	Day Relative to Inoculum	Day Relative to IP	Result	Range Indicator	Clinical Assessment
R210	3D7	Neutrophils (10^9/L) [1.5 – 8]	Screening	12-FEB-2018	09:38	-8	-16	3.41		
			Safety Visit	16-FEB-2018	07:28	-4	-12	2.87		
			Admission	28-FEB-2018	11:21	8	1	3.62		
			Admission 3	03-MAR-2018	12:01	11	4	1.80		
			Safety Monitoring	05-MAR-2018	07:40	13	6	2.27		
			Safety Monitoring	08-MAR-2018	07:40	16	9	3.34		
			Safety Monitoring	12-MAR-2018	07:37	20	13	2.61		
			Safety Monitoring	19-MAR-2018	07:39	27	20	3.66		
			End of Study	22-MAR-2018	07:34	30	23	3.19		
R210		Platelets (10^9/L) [150 – 400]	Screening	12-FEB-2018	09:38	-8	-16	269		
			Safety Visit	16-FEB-2018	07:28	-4	-12	235		
			Admission	28-FEB-2018	11:21	8	1	236		
			Admission 3	03-MAR-2018	12:01	11	4	196		
			Safety Monitoring	05-MAR-2018	07:40	13	6	226		
			Safety Monitoring	08-MAR-2018	07:40	16	9	211		
			Safety Monitoring	12-MAR-2018	07:37	20	13	247		
			Safety Monitoring	19-MAR-2018	07:39	27	20	242		
			End of Study	22-MAR-2018	07:34	30	23	269		

Notes: Day relative to inoculum is the number of days relative to day of administration of the inoculum, where the day of administration = 0.
The inoculum was administered on Study Day 0 for K13-infected subjects and Study Day 1 for 3D7-infected subjects.
Day relative to IP is the number of days relative to day of administration of Artesunate, where the day of dose = 1.
Rando = Randomisation; IP = Artesunate; H = High; L = Low; NCS = Not Clinically Significant; CS = Clinically Significant.

Listing 16.2.8.1a
Individual Haematology Results

Rando Number	Challenge Agent	Parameter (unit) [Normal Range]	Visit	Date of Assessment	Time of Assessment	Day Relative to Inoculum	Day Relative to IP	Result	Range Indicator	Clinical Assessment
R210	3D7	Reticulocytes (10^9/L) [25 – 120]	Safety Visit	16-FEB-2018	07:28	-4	-12	66		
			End of Study	22-MAR-2018	07:34	30	23	78		
R302	K13	Basophils (10^9/L) [0 – 0.15]	Screening	16-AUG-2018	09:36	-19	-28	0.04		
			Safety Visit	03-SEP-2018	08:18	-1	-10	0.04		
			Admission	13-SEP-2018	08:57	9	1	0.04		
			Safety Monitoring	15-SEP-2018	09:06	11	3	0.02		
			Admission 3	16-SEP-2018	09:05	12	4	0.01		
			Safety Monitoring	18-SEP-2018	08:04	14	6	0.05		
			Safety Monitoring	22-SEP-2018	07:56	18	10	0.05		
			Safety Monitoring	25-SEP-2018	08:13	21	13	0.05		
			Safety Monitoring	28-SEP-2018	08:22	24	16	0.04		
			End of Study	02-OCT-2018	08:19	28	20	0.06		

Notes: Day relative to inoculum is the number of days relative to day of administration of the inoculum, where the day of administration = 0.
The inoculum was administered on Study Day 0 for K13-infected subjects and Study Day 1 for 3D7-infected subjects.
Day relative to IP is the number of days relative to day of administration of Artesunate, where the day of dose = 1.
Rando = Randomisation; IP = Artesunate; H = High; L = Low; NCS = Not Clinically Significant; CS = Clinically Significant.

Listing 16.2.8.1a
Individual Haematology Results

Rando Number	Challenge Agent	Parameter (unit) [Normal Range]	Visit	Date of Assessment	Time of Assessment	Day Relative to Inoculum	Day Relative to IP	Result	Range Indicator	Clinical Assessment
R302	K13	Eosinophils (10^9/L) [0 – 0.6]	Screening	16-AUG-2018	09:36	-19	-28	0.05		
			Safety Visit	03-SEP-2018	08:18	-1	-10	0.11		
			Admission	13-SEP-2018	08:57	9	1	0.04		
			Safety Monitoring	15-SEP-2018	09:06	11	3	0.01		
			Admission 3	16-SEP-2018	09:05	12	4	0.02		
			Safety Monitoring	18-SEP-2018	08:04	14	6	0.06		
			Safety Monitoring	22-SEP-2018	07:56	18	10	0.07		
			Safety Monitoring	25-SEP-2018	08:13	21	13	0.05		
			Safety Monitoring	28-SEP-2018	08:22	24	16	0.06		
			End of Study	02-OCT-2018	08:19	28	20	0.08		

Notes: Day relative to inoculum is the number of days relative to day of administration of the inoculum, where the day of administration = 0.
The inoculum was administered on Study Day 0 for K13-infected subjects and Study Day 1 for 3D7-infected subjects.
Day relative to IP is the number of days relative to day of administration of Artesunate, where the day of dose = 1.
Rando = Randomisation; IP = Artesunate; H = High; L = Low; NCS = Not Clinically Significant; CS = Clinically Significant.

Listing 16.2.8.1a
Individual Haematology Results

Rando Number	Challenge Agent	Parameter (unit) [Normal Range]	Visit	Date of Assessment	Time of Assessment	Day Relative to Inoculum	Day Relative to IP	Result	Range Indicator	Clinical Assessment
R302	K13	Ery. Mean Corpuscular Volume (fL) [80 – 100]	Screening	16-AUG-2018	09:36	-19	-28	84		
			Safety Visit	03-SEP-2018	08:18	-1	-10	87		
			Admission	13-SEP-2018	08:57	9	1	83		
			Safety Monitoring	15-SEP-2018	09:06	11	3	81		
			Admission 3	16-SEP-2018	09:05	12	4	82		
			Safety Monitoring	18-SEP-2018	08:04	14	6	81		
			Safety Monitoring	22-SEP-2018	07:56	18	10	81		
			Safety Monitoring	25-SEP-2018	08:13	21	13	83		
			Safety Monitoring	28-SEP-2018	08:22	24	16	82		
			End of Study	02-OCT-2018	08:19	28	20	84		

Notes: Day relative to inoculum is the number of days relative to day of administration of the inoculum, where the day of administration = 0.
The inoculum was administered on Study Day 0 for K13-infected subjects and Study Day 1 for 3D7-infected subjects.
Day relative to IP is the number of days relative to day of administration of Artesunate, where the day of dose = 1.
Rando = Randomisation; IP = Artesunate; H = High; L = Low; NCS = Not Clinically Significant; CS = Clinically Significant.

Listing 16.2.8.1a
Individual Haematology Results

Rando Number	Challenge Agent	Parameter (unit) [Normal Range]	Visit	Date of Assessment	Time of Assessment	Day Relative to Inoculum	Day Relative to IP	Result	Range Indicator	Clinical Assessment
R302	K13	Erythrocytes (10^12/L) [4.5 – 6.5]	Screening	16-AUG-2018	09:36	-19	-28	5.1		
			Safety Visit	03-SEP-2018	08:18	-1	-10	4.7		
			Admission	13-SEP-2018	08:57	9	1	5.0		
			Safety Monitoring	15-SEP-2018	09:06	11	3	4.9		
			Admission 3	16-SEP-2018	09:05	12	4	4.9		
			Safety Monitoring	18-SEP-2018	08:04	14	6	4.5		
			Safety Monitoring	22-SEP-2018	07:56	18	10	4.4	L	NCS
			Safety Monitoring	25-SEP-2018	08:13	21	13	4.7		
			Safety Monitoring	28-SEP-2018	08:22	24	16	4.4	L	NCS
			End of Study	02-OCT-2018	08:19	28	20	4.7		

Notes: Day relative to inoculum is the number of days relative to day of administration of the inoculum, where the day of administration = 0.
The inoculum was administered on Study Day 0 for K13-infected subjects and Study Day 1 for 3D7-infected subjects.
Day relative to IP is the number of days relative to day of administration of Artesunate, where the day of dose = 1.
Rando = Randomisation; IP = Artesunate; H = High; L = Low; NCS = Not Clinically Significant; CS = Clinically Significant.

Listing 16.2.8.1a
Individual Haematology Results

Rando Number	Challenge Agent	Parameter (unit) [Normal Range]	Visit	Date of Assessment	Time of Assessment	Day Relative to Inoculum	Day Relative to IP	Result	Range Indicator	Clinical Assessment
R302	K13	Hematocrit [0.4 – 0.54]	Screening	16-AUG-2018	09:36	-19	-28	0.43		
			Safety Visit	03-SEP-2018	08:18	-1	-10	0.41		
			Admission	13-SEP-2018	08:57	9	1	0.41		
			Safety Monitoring	15-SEP-2018	09:06	11	3	0.40		
			Admission 3	16-SEP-2018	09:05	12	4	0.40		
			Safety Monitoring	18-SEP-2018	08:04	14	6	0.36	L	NCS
			Safety Monitoring	22-SEP-2018	07:56	18	10	0.36	L	NCS
			Safety Monitoring	25-SEP-2018	08:13	21	13	0.39	L	NCS
			Safety Monitoring	28-SEP-2018	08:22	24	16	0.37	L	NCS
			End of Study	02-OCT-2018	08:19	28	20	0.40		

Notes: Day relative to inoculum is the number of days relative to day of administration of the inoculum, where the day of administration = 0.
The inoculum was administered on Study Day 0 for K13-infected subjects and Study Day 1 for 3D7-infected subjects.
Day relative to IP is the number of days relative to day of administration of Artesunate, where the day of dose = 1.
Rando = Randomisation; IP = Artesunate; H = High; L = Low; NCS = Not Clinically Significant; CS = Clinically Significant.

Listing 16.2.8.1a
Individual Haematology Results

Rando Number	Challenge Agent	Parameter (unit) [Normal Range]	Visit	Date of Assessment	Time of Assessment	Day Relative to Inoculum	Day Relative to IP	Result	Range Indicator	Clinical Assessment
R302	K13	Hemoglobin (g/L) [135 – 175]	Screening	16-AUG-2018	09:36	-19	-28	151		
			Safety Visit	03-SEP-2018	08:18	-1	-10	141		
			Admission	13-SEP-2018	08:57	9	1	146		
			Safety Monitoring	15-SEP-2018	09:06	11	3	145		
			Admission 3	16-SEP-2018	09:05	12	4	144		
			Safety Monitoring	18-SEP-2018	08:04	14	6	131	L	NCS
			Safety Monitoring	22-SEP-2018	07:56	18	10	128	L	NCS
			Safety Monitoring	25-SEP-2018	08:13	21	13	137		
			Safety Monitoring	28-SEP-2018	08:22	24	16	131	L	NCS
			End of Study	02-OCT-2018	08:19	28	20	138		

Notes: Day relative to inoculum is the number of days relative to day of administration of the inoculum, where the day of administration = 0.
The inoculum was administered on Study Day 0 for K13-infected subjects and Study Day 1 for 3D7-infected subjects.
Day relative to IP is the number of days relative to day of administration of Artesunate, where the day of dose = 1.
Rando = Randomisation; IP = Artesunate; H = High; L = Low; NCS = Not Clinically Significant; CS = Clinically Significant.

Listing 16.2.8.1a
Individual Haematology Results

Rando Number	Challenge Agent	Parameter (unit) [Normal Range]	Visit	Date of Assessment	Time of Assessment	Day Relative to Inoculum	Day Relative to IP	Result	Range Indicator	Clinical Assessment
R302	K13	Leukocytes (10^9/L) [3.5 – 10]	Screening	16-AUG-2018	09:36	-19	-28	7.0		
			Safety Visit	03-SEP-2018	08:18	-1	-10	6.0		
			Admission	13-SEP-2018	08:57	9	1	5.3		
			Safety Monitoring	15-SEP-2018	09:06	11	3	3.9		
			Admission 3	16-SEP-2018	09:05	12	4	4.5		
			Safety Monitoring	18-SEP-2018	08:04	14	6	4.6		
			Safety Monitoring	22-SEP-2018	07:56	18	10	5.6		
			Safety Monitoring	25-SEP-2018	08:13	21	13	5.3		
			Safety Monitoring	28-SEP-2018	08:22	24	16	4.7		
			End of Study	02-OCT-2018	08:19	28	20	6.0		

Notes: Day relative to inoculum is the number of days relative to day of administration of the inoculum, where the day of administration = 0.
The inoculum was administered on Study Day 0 for K13-infected subjects and Study Day 1 for 3D7-infected subjects.
Day relative to IP is the number of days relative to day of administration of Artesunate, where the day of dose = 1.
Rando = Randomisation; IP = Artesunate; H = High; L = Low; NCS = Not Clinically Significant; CS = Clinically Significant.

Listing 16.2.8.1a
Individual Haematology Results

Rando Number	Challenge Agent	Parameter (unit) [Normal Range]	Visit	Date of Assessment	Time of Assessment	Day Relative to Inoculum	Day Relative to IP	Result	Range Indicator	Clinical Assessment
R302	K13	Lymphocytes (10^9/L) [1 – 4]	Screening	16-AUG-2018	09:36	-19	-28	1.82		
			Safety Visit	03-SEP-2018	08:18	-1	-10	2.42		
			Admission	13-SEP-2018	08:57	9	1	1.28		
			Safety Monitoring	15-SEP-2018	09:06	11	3	0.95	L	NCS
			Admission 3	16-SEP-2018	09:05	12	4	0.78	L	CS
			Safety Monitoring	18-SEP-2018	08:04	14	6	2.04		
			Safety Monitoring	22-SEP-2018	07:56	18	10	2.15		
			Safety Monitoring	25-SEP-2018	08:13	21	13	1.78		
			Safety Monitoring	28-SEP-2018	08:22	24	16	1.87		
			End of Study	02-OCT-2018	08:19	28	20	2.30		

Notes: Day relative to inoculum is the number of days relative to day of administration of the inoculum, where the day of administration = 0.
The inoculum was administered on Study Day 0 for K13-infected subjects and Study Day 1 for 3D7-infected subjects.
Day relative to IP is the number of days relative to day of administration of Artesunate, where the day of dose = 1.
Rando = Randomisation; IP = Artesunate; H = High; L = Low; NCS = Not Clinically Significant; CS = Clinically Significant.

Listing 16.2.8.1a
Individual Haematology Results

Rando Number	Challenge Agent	Parameter (unit) [Normal Range]	Visit	Date of Assessment	Time of Assessment	Day Relative to Inoculum	Day Relative to IP	Result	Range Indicator	Clinical Assessment
R302	K13	Monocytes (10^9/L) [0 – 0.9]	Screening	16-AUG-2018	09:36	-19	-28	0.48		
			Safety Visit	03-SEP-2018	08:18	-1	-10	0.48		
			Admission	13-SEP-2018	08:57	9	1	0.69		
			Safety Monitoring	15-SEP-2018	09:06	11	3	0.43		
			Admission 3	16-SEP-2018	09:05	12	4	0.44		
			Safety Monitoring	18-SEP-2018	08:04	14	6	0.58		
			Safety Monitoring	22-SEP-2018	07:56	18	10	0.38		
			Safety Monitoring	25-SEP-2018	08:13	21	13	0.46		
			Safety Monitoring	28-SEP-2018	08:22	24	16	0.41		
			End of Study	02-OCT-2018	08:19	28	20	0.44		

Notes: Day relative to inoculum is the number of days relative to day of administration of the inoculum, where the day of administration = 0.
The inoculum was administered on Study Day 0 for K13-infected subjects and Study Day 1 for 3D7-infected subjects.
Day relative to IP is the number of days relative to day of administration of Artesunate, where the day of dose = 1.
Rando = Randomisation; IP = Artesunate; H = High; L = Low; NCS = Not Clinically Significant; CS = Clinically Significant.

Printed: 24FEB2020 (Final)

Listing 16.2.8.1a
Individual Haematology Results

Rando Number	Challenge Agent	Parameter (unit) [Normal Range]	Visit	Date of Assessment	Time of Assessment	Day Relative to Inoculum	Day Relative to IP	Result	Range Indicator	Clinical Assessment
R302	K13	Neutrophils (10^9/L) [1.5 – 6.5]	Screening	16-AUG-2018	09:36	-19	-28	4.59		
			Safety Visit	03-SEP-2018	08:18	-1	-10	2.94		
			Admission	13-SEP-2018	08:57	9	1	3.25		
			Safety Monitoring	15-SEP-2018	09:06	11	3	2.52		
			Admission 3	16-SEP-2018	09:05	12	4	3.22		
			Safety Monitoring	18-SEP-2018	08:04	14	6	1.86		
			Safety Monitoring	22-SEP-2018	07:56	18	10	2.90		
			Safety Monitoring	25-SEP-2018	08:13	21	13	2.95		
			Safety Monitoring	28-SEP-2018	08:22	24	16	2.33		
			End of Study	02-OCT-2018	08:19	28	20	3.12		

Notes: Day relative to inoculum is the number of days relative to day of administration of the inoculum, where the day of administration = 0.
The inoculum was administered on Study Day 0 for K13-infected subjects and Study Day 1 for 3D7-infected subjects.
Day relative to IP is the number of days relative to day of administration of Artesunate, where the day of dose = 1.
Rando = Randomisation; IP = Artesunate; H = High; L = Low; NCS = Not Clinically Significant; CS = Clinically Significant.

Listing 16.2.8.1a
Individual Haematology Results

Rando Number	Challenge Agent	Parameter (unit) [Normal Range]	Visit	Date of Assessment	Time of Assessment	Day Relative to Inoculum	Day Relative to IP	Result	Range Indicator	Clinical Assessment
R302	K13	Platelets (10^9/L) [150 – 400]	Screening	16-AUG-2018	09:36	-19	-28	295		
			Safety Visit	03-SEP-2018	08:18	-1	-10	279		
			Admission	13-SEP-2018	08:57	9	1	273		
			Safety Monitoring	15-SEP-2018	09:06	11	3	203		
			Admission 3	16-SEP-2018	09:05	12	4	167		
			Safety Monitoring	18-SEP-2018	08:04	14	6	160		
			Safety Monitoring	22-SEP-2018	07:56	18	10	261		
			Safety Monitoring	25-SEP-2018	08:13	21	13	309		
			Safety Monitoring	28-SEP-2018	08:22	24	16	286		
			End of Study	02-OCT-2018	08:19	28	20	281		
		Reticulocytes (10^9/L) [25 – 120]	Safety Visit	03-SEP-2018	08:18	-1	-10	88		
			End of Study	02-OCT-2018	08:19	28	20	120		

Notes: Day relative to inoculum is the number of days relative to day of administration of the inoculum, where the day of administration = 0.
The inoculum was administered on Study Day 0 for K13-infected subjects and Study Day 1 for 3D7-infected subjects.
Day relative to IP is the number of days relative to day of administration of Artesunate, where the day of dose = 1.
Rando = Randomisation; IP = Artesunate; H = High; L = Low; NCS = Not Clinically Significant; CS = Clinically Significant.

Listing 16.2.8.1a
Individual Haematology Results

Rando Number	Challenge Agent	Parameter (unit) [Normal Range]	Visit	Date of Assessment	Time of Assessment	Day Relative to Inoculum	Day Relative to IP	Result	Range Indicator	Clinical Assessment
R303	K13	Basophils (10^9/L) [0 – 0.15]	Screening	09-AUG-2018	10:30	-26	-35	0.03		
			Safety Visit	03-SEP-2018	07:27	-1	-10	0.05		
			Unscheduled	13-SEP-2018	09:52	9	1	0.03		
			Admission	13-SEP-2018	11:17	9	1	0.04		
			Safety Monitoring	15-SEP-2018	11:18	11	3	0.02		
			Admission 3	16-SEP-2018	11:18	12	4	0.02		
			Safety Monitoring	18-SEP-2018	07:29	14	6	0.04		
			Safety Monitoring	22-SEP-2018	08:40	18	10	0.05		
			Safety Monitoring	25-SEP-2018	08:20	21	13	0.05		
			Safety Monitoring	28-SEP-2018	08:19	24	16	0.04		
			End of Study	02-OCT-2018	09:05	28	20	0.05		

Notes: Day relative to inoculum is the number of days relative to day of administration of the inoculum, where the day of administration = 0.
The inoculum was administered on Study Day 0 for K13-infected subjects and Study Day 1 for 3D7-infected subjects.
Day relative to IP is the number of days relative to day of administration of Artesunate, where the day of dose = 1.
Rando = Randomisation; IP = Artesunate; H = High; L = Low; NCS = Not Clinically Significant; CS = Clinically Significant.

Listing 16.2.8.1a
Individual Haematology Results

Rando Number	Challenge Agent	Parameter (unit) [Normal Range]	Visit	Date of Assessment	Time of Assessment	Day Relative to Inoculum	Day Relative to IP	Result	Range Indicator	Clinical Assessment
R303	K13	Eosinophils (10^9/L) [0 – 0.6]	Screening	09-AUG-2018	10:30	-26	-35	0.04		
			Safety Visit	03-SEP-2018	07:27	-1	-10	0.10		
			Unscheduled	13-SEP-2018	09:52	9	1	0.03		
			Admission	13-SEP-2018	11:17	9	1	0.03		
			Safety Monitoring	15-SEP-2018	11:18	11	3	0.01		
			Admission 3	16-SEP-2018	11:18	12	4	0.02		
			Safety Monitoring	18-SEP-2018	07:29	14	6	0.08		
			Safety Monitoring	22-SEP-2018	08:40	18	10	0.11		
			Safety Monitoring	25-SEP-2018	08:20	21	13	0.05		
			Safety Monitoring	28-SEP-2018	08:19	24	16	0.03		
			End of Study	02-OCT-2018	09:05	28	20	0.17		

Notes: Day relative to inoculum is the number of days relative to day of administration of the inoculum, where the day of administration = 0.
The inoculum was administered on Study Day 0 for K13-infected subjects and Study Day 1 for 3D7-infected subjects.
Day relative to IP is the number of days relative to day of administration of Artesunate, where the day of dose = 1.
Rando = Randomisation; IP = Artesunate; H = High; L = Low; NCS = Not Clinically Significant; CS = Clinically Significant.

Listing 16.2.8.1a
Individual Haematology Results

Rando Number	Challenge Agent	Parameter (unit) [Normal Range]	Visit	Date of Assessment	Time of Assessment	Day Relative to Inoculum	Day Relative to IP	Result	Range Indicator	Clinical Assessment
R303	K13	Ery. Mean Corpuscular Volume (fL) [80 – 100]	Screening	09-AUG-2018	10:30	-26	-35	84		
			Safety Visit	03-SEP-2018	07:27	-1	-10	85		
			Unscheduled	13-SEP-2018	09:52	9	1	82		
			Admission	13-SEP-2018	11:17	9	1	80		
			Safety Monitoring	15-SEP-2018	11:18	11	3	78	L	NCS
			Admission 3	16-SEP-2018	11:18	12	4	79	L	NCS
			Safety Monitoring	18-SEP-2018	07:29	14	6	80		
			Safety Monitoring	22-SEP-2018	08:40	18	10	79	L	NCS
			Safety Monitoring	25-SEP-2018	08:20	21	13	81		
			Safety Monitoring	28-SEP-2018	08:19	24	16	80		
			End of Study	02-OCT-2018	09:05	28	20	80		

Notes: Day relative to inoculum is the number of days relative to day of administration of the inoculum, where the day of administration = 0.
The inoculum was administered on Study Day 0 for K13-infected subjects and Study Day 1 for 3D7-infected subjects.
Day relative to IP is the number of days relative to day of administration of Artesunate, where the day of dose = 1.
Rando = Randomisation; IP = Artesunate; H = High; L = Low; NCS = Not Clinically Significant; CS = Clinically Significant.

Listing 16.2.8.1a
Individual Haematology Results

Rando Number	Challenge Agent	Parameter (unit) [Normal Range]	Visit	Date of Assessment	Time of Assessment	Day Relative to Inoculum	Day Relative to IP	Result	Range Indicator	Clinical Assessment
R303	K13	Erythrocytes (10^12/L) [3.9 – 5.6]	Screening	09-AUG-2018	10:30	-26	-35	4.9		
			Safety Visit	03-SEP-2018	07:27	-1	-10	4.6		
			Unscheduled	13-SEP-2018	09:52	9	1	4.6		
			Admission	13-SEP-2018	11:17	9	1	4.7		
			Safety Monitoring	15-SEP-2018	11:18	11	3	4.6		
			Admission 3	16-SEP-2018	11:18	12	4	4.6		
			Safety Monitoring	18-SEP-2018	07:29	14	6	4.5		
			Safety Monitoring	22-SEP-2018	08:40	18	10	4.2		
			Safety Monitoring	25-SEP-2018	08:20	21	13	4.2		
			Safety Monitoring	28-SEP-2018	08:19	24	16	4.0		
			End of Study	02-OCT-2018	09:05	28	20	4.3		

Notes: Day relative to inoculum is the number of days relative to day of administration of the inoculum, where the day of administration = 0.
The inoculum was administered on Study Day 0 for K13-infected subjects and Study Day 1 for 3D7-infected subjects.
Day relative to IP is the number of days relative to day of administration of Artesunate, where the day of dose = 1.
Rando = Randomisation; IP = Artesunate; H = High; L = Low; NCS = Not Clinically Significant; CS = Clinically Significant.

Listing 16.2.8.1a
Individual Haematology Results

Rando Number	Challenge Agent	Parameter (unit) [Normal Range]	Visit	Date of Assessment	Time of Assessment	Day Relative to Inoculum	Day Relative to IP	Result	Range Indicator	Clinical Assessment
R303	K13	Hematocrit [0.35 – 0.47]	Screening	09-AUG-2018	10:30	-26	-35	0.41		
			Safety Visit	03-SEP-2018	07:27	-1	-10	0.39		
			Unscheduled	13-SEP-2018	09:52	9	1	0.38		
			Admission	13-SEP-2018	11:17	9	1	0.38		
			Safety Monitoring	15-SEP-2018	11:18	11	3	0.36		
			Admission 3	16-SEP-2018	11:18	12	4	0.36		
			Safety Monitoring	18-SEP-2018	07:29	14	6	0.36		
			Safety Monitoring	22-SEP-2018	08:40	18	10	0.33	L	NCS
			Safety Monitoring	25-SEP-2018	08:20	21	13	0.34	L	NCS
			Safety Monitoring	28-SEP-2018	08:19	24	16	0.32	L	NCS
			End of Study	02-OCT-2018	09:05	28	20	0.35		

Notes: Day relative to inoculum is the number of days relative to day of administration of the inoculum, where the day of administration = 0.
The inoculum was administered on Study Day 0 for K13-infected subjects and Study Day 1 for 3D7-infected subjects.
Day relative to IP is the number of days relative to day of administration of Artesunate, where the day of dose = 1.
Rando = Randomisation; IP = Artesunate; H = High; L = Low; NCS = Not Clinically Significant; CS = Clinically Significant.

Listing 16.2.8.1a
Individual Haematology Results

Rando Number	Challenge Agent	Parameter (unit) [Normal Range]	Visit	Date of Assessment	Time of Assessment	Day Relative to Inoculum	Day Relative to IP	Result	Range Indicator	Clinical Assessment
R303	K13	Hemoglobin (g/L) [115 – 165]	Screening	09-AUG-2018	10:30	-26	-35	137		
			Safety Visit	03-SEP-2018	07:27	-1	-10	128		
			Unscheduled	13-SEP-2018	09:52	9	1	127		
			Admission	13-SEP-2018	11:17	9	1	127		
			Safety Monitoring	15-SEP-2018	11:18	11	3	127		
			Admission 3	16-SEP-2018	11:18	12	4	127		
			Safety Monitoring	18-SEP-2018	07:29	14	6	124		
			Safety Monitoring	22-SEP-2018	08:40	18	10	114	L	NCS
			Safety Monitoring	25-SEP-2018	08:20	21	13	114	L	NCS
			Safety Monitoring	28-SEP-2018	08:19	24	16	110	L	NCS
			End of Study	02-OCT-2018	09:05	28	20	120		

Notes: Day relative to inoculum is the number of days relative to day of administration of the inoculum, where the day of administration = 0.
The inoculum was administered on Study Day 0 for K13-infected subjects and Study Day 1 for 3D7-infected subjects.
Day relative to IP is the number of days relative to day of administration of Artesunate, where the day of dose = 1.
Rando = Randomisation; IP = Artesunate; H = High; L = Low; NCS = Not Clinically Significant; CS = Clinically Significant.

Listing 16.2.8.1a
Individual Haematology Results

Rando Number	Challenge Agent	Parameter (unit) [Normal Range]	Visit	Date of Assessment	Time of Assessment	Day Relative to Inoculum	Day Relative to IP	Result	Range Indicator	Clinical Assessment
R303	K13	Leukocytes (10^9/L) [3.5 – 12]	Screening	09-AUG-2018	10:30	-26	-35	6.7		
			Safety Visit	03-SEP-2018	07:27	-1	-10	5.3		
			Unscheduled	13-SEP-2018	09:52	9	1	4.2		
			Admission	13-SEP-2018	11:17	9	1	4.4		
			Safety Monitoring	15-SEP-2018	11:18	11	3	2.5	L	CS
			Admission 3	16-SEP-2018	11:18	12	4	2.9	L	CS
			Safety Monitoring	18-SEP-2018	07:29	14	6	4.6		
			Safety Monitoring	22-SEP-2018	08:40	18	10	7.4		
			Safety Monitoring	25-SEP-2018	08:20	21	13	6.5		
			Safety Monitoring	28-SEP-2018	08:19	24	16	5.1		
			End of Study	02-OCT-2018	09:05	28	20	6.1		

Notes: Day relative to inoculum is the number of days relative to day of administration of the inoculum, where the day of administration = 0.
The inoculum was administered on Study Day 0 for K13-infected subjects and Study Day 1 for 3D7-infected subjects.
Day relative to IP is the number of days relative to day of administration of Artesunate, where the day of dose = 1.
Rando = Randomisation; IP = Artesunate; H = High; L = Low; NCS = Not Clinically Significant; CS = Clinically Significant.

Listing 16.2.8.1a
Individual Haematology Results

Rando Number	Challenge Agent	Parameter (unit) [Normal Range]	Visit	Date of Assessment	Time of Assessment	Day Relative to Inoculum	Day Relative to IP	Result	Range Indicator	Clinical Assessment
R303	K13	Lymphocytes (10^9/L) [1 – 4]	Screening	09-AUG-2018	10:30	-26	-35	2.69		
			Safety Visit	03-SEP-2018	07:27	-1	-10	2.80		
			Unscheduled	13-SEP-2018	09:52	9	1	1.43		
			Admission	13-SEP-2018	11:17	9	1	1.62		
			Safety Monitoring	15-SEP-2018	11:18	11	3	1.22		
			Admission 3	16-SEP-2018	11:18	12	4	1.58		
			Safety Monitoring	18-SEP-2018	07:29	14	6	3.03		
			Safety Monitoring	22-SEP-2018	08:40	18	10	3.23		
			Safety Monitoring	25-SEP-2018	08:20	21	13	3.23		
			Safety Monitoring	28-SEP-2018	08:19	24	16	2.51		
			End of Study	02-OCT-2018	09:05	28	20	2.55		

Notes: Day relative to inoculum is the number of days relative to day of administration of the inoculum, where the day of administration = 0.
The inoculum was administered on Study Day 0 for K13-infected subjects and Study Day 1 for 3D7-infected subjects.
Day relative to IP is the number of days relative to day of administration of Artesunate, where the day of dose = 1.
Rando = Randomisation; IP = Artesunate; H = High; L = Low; NCS = Not Clinically Significant; CS = Clinically Significant.

Listing 16.2.8.1a
Individual Haematology Results

Rando Number	Challenge Agent	Parameter (unit) [Normal Range]	Visit	Date of Assessment	Time of Assessment	Day Relative to Inoculum	Day Relative to IP	Result	Range Indicator	Clinical Assessment
R303	K13	Monocytes (10^9/L) [0 – 0.9]	Screening	09-AUG-2018	10:30	-26	-35	0.38		
			Safety Visit	03-SEP-2018	07:27	-1	-10	0.36		
			Unscheduled	13-SEP-2018	09:52	9	1	0.31		
			Admission	13-SEP-2018	11:17	9	1	0.32		
			Safety Monitoring	15-SEP-2018	11:18	11	3	0.18		
			Admission 3	16-SEP-2018	11:18	12	4	0.25		
			Safety Monitoring	18-SEP-2018	07:29	14	6	0.33		
			Safety Monitoring	22-SEP-2018	08:40	18	10	0.37		
			Safety Monitoring	25-SEP-2018	08:20	21	13	0.42		
			Safety Monitoring	28-SEP-2018	08:19	24	16	0.46		
			End of Study	02-OCT-2018	09:05	28	20	0.35		

Notes: Day relative to inoculum is the number of days relative to day of administration of the inoculum, where the day of administration = 0.
The inoculum was administered on Study Day 0 for K13-infected subjects and Study Day 1 for 3D7-infected subjects.
Day relative to IP is the number of days relative to day of administration of Artesunate, where the day of dose = 1.
Rando = Randomisation; IP = Artesunate; H = High; L = Low; NCS = Not Clinically Significant; CS = Clinically Significant.

Listing 16.2.8.1a
Individual Haematology Results

Rando Number	Challenge Agent	Parameter (unit) [Normal Range]	Visit	Date of Assessment	Time of Assessment	Day Relative to Inoculum	Day Relative to IP	Result	Range Indicator	Clinical Assessment
R303	K13	Neutrophils (10^9/L) [1.5 – 8]	Screening	09-AUG-2018	10:30	-26	-35	3.56		
			Safety Visit	03-SEP-2018	07:27	-1	-10	2.01		
			Unscheduled	13-SEP-2018	09:52	9	1	2.44		
			Admission	13-SEP-2018	11:17	9	1	2.43		
			Safety Monitoring	15-SEP-2018	11:18	11	3	1.11	L	CS
			Admission 3	16-SEP-2018	11:18	12	4	1.01	L	CS
			Safety Monitoring	18-SEP-2018	07:29	14	6	1.12	L	CS
			Safety Monitoring	22-SEP-2018	08:40	18	10	3.63		
			Safety Monitoring	25-SEP-2018	08:20	21	13	2.70		
			Safety Monitoring	28-SEP-2018	08:19	24	16	2.07		
			End of Study	02-OCT-2018	09:05	28	20	2.93		

Notes: Day relative to inoculum is the number of days relative to day of administration of the inoculum, where the day of administration = 0.
The inoculum was administered on Study Day 0 for K13-infected subjects and Study Day 1 for 3D7-infected subjects.
Day relative to IP is the number of days relative to day of administration of Artesunate, where the day of dose = 1.
Rando = Randomisation; IP = Artesunate; H = High; L = Low; NCS = Not Clinically Significant; CS = Clinically Significant.

Listing 16.2.8.1a
Individual Haematology Results

Rando Number	Challenge Agent	Parameter (unit) [Normal Range]	Visit	Date of Assessment	Time of Assessment	Day Relative to Inoculum	Day Relative to IP	Result	Range Indicator	Clinical Assessment
R303	K13	Platelets (10^9/L) [150 – 400]	Screening	09-AUG-2018	10:30	-26	-35	363		
			Safety Visit	03-SEP-2018	07:27	-1	-10	348		
			Unscheduled	13-SEP-2018	09:52	9	1	258		
			Admission	13-SEP-2018	11:17	9	1	264		
			Safety Monitoring	15-SEP-2018	11:18	11	3	168		
			Admission 3	16-SEP-2018	11:18	12	4	162		
			Safety Monitoring	18-SEP-2018	07:29	14	6	169		
			Safety Monitoring	22-SEP-2018	08:40	18	10	384		
			Safety Monitoring	25-SEP-2018	08:20	21	13	412	H	NCS
			Safety Monitoring	28-SEP-2018	08:19	24	16	346		
			End of Study	02-OCT-2018	09:05	28	20	308		
		Reticulocytes (10^9/L) [25 – 120]	Safety Visit	03-SEP-2018	07:27	-1	-10	63		
			End of Study	02-OCT-2018	09:05	28	20	99		

Notes: Day relative to inoculum is the number of days relative to day of administration of the inoculum, where the day of administration = 0.
The inoculum was administered on Study Day 0 for K13-infected subjects and Study Day 1 for 3D7-infected subjects.
Day relative to IP is the number of days relative to day of administration of Artesunate, where the day of dose = 1.
Rando = Randomisation; IP = Artesunate; H = High; L = Low; NCS = Not Clinically Significant; CS = Clinically Significant.

Listing 16.2.8.1a
Individual Haematology Results

Rando Number	Challenge Agent	Parameter (unit) [Normal Range]	Visit	Date of Assessment	Time of Assessment	Day Relative to Inoculum	Day Relative to IP	Result	Range Indicator	Clinical Assessment
R304	K13	Basophils (10^9/L) [0 – 0.15]	Screening	13-AUG-2018	11:57	-22	-31	0.03		
			Safety Visit	03-SEP-2018	08:07	-1	-10	0.03		
			Admission	13-SEP-2018	09:16	9	1	0.02		
			Safety Monitoring	15-SEP-2018	09:22	11	3	0.02		
			Admission 3	16-SEP-2018	09:22	12	4	0.04		
			Safety Monitoring	18-SEP-2018	08:36	14	6	0.06		
			Safety Monitoring	22-SEP-2018	09:25	18	10	0.04		
			Safety Monitoring	25-SEP-2018	08:11	21	13	0.05		
			Safety Monitoring	28-SEP-2018	08:17	24	16	0.03		
			End of Study	02-OCT-2018	08:46	28	20	0.04		

Notes: Day relative to inoculum is the number of days relative to day of administration of the inoculum, where the day of administration = 0.
The inoculum was administered on Study Day 0 for K13-infected subjects and Study Day 1 for 3D7-infected subjects.
Day relative to IP is the number of days relative to day of administration of Artesunate, where the day of dose = 1.
Rando = Randomisation; IP = Artesunate; H = High; L = Low; NCS = Not Clinically Significant; CS = Clinically Significant.

Listing 16.2.8.1a
Individual Haematology Results

Rando Number	Challenge Agent	Parameter (unit) [Normal Range]	Visit	Date of Assessment	Time of Assessment	Day Relative to Inoculum	Day Relative to IP	Result	Range Indicator	Clinical Assessment
R304	K13	Eosinophils (10^9/L) [0 – 0.6]	Screening	13-AUG-2018	11:57	-22	-31	0.10		
			Safety Visit	03-SEP-2018	08:07	-1	-10	0.07		
			Admission	13-SEP-2018	09:16	9	1	0.05		
			Safety Monitoring	15-SEP-2018	09:22	11	3	0.02		
			Admission 3	16-SEP-2018	09:22	12	4	0.05		
			Safety Monitoring	18-SEP-2018	08:36	14	6	0.10		
			Safety Monitoring	22-SEP-2018	09:25	18	10	0.04		
			Safety Monitoring	25-SEP-2018	08:11	21	13	0.08		
			Safety Monitoring	28-SEP-2018	08:17	24	16	0.07		
			End of Study	02-OCT-2018	08:46	28	20	0.12		

Notes: Day relative to inoculum is the number of days relative to day of administration of the inoculum, where the day of administration = 0.
The inoculum was administered on Study Day 0 for K13-infected subjects and Study Day 1 for 3D7-infected subjects.
Day relative to IP is the number of days relative to day of administration of Artesunate, where the day of dose = 1.
Rando = Randomisation; IP = Artesunate; H = High; L = Low; NCS = Not Clinically Significant; CS = Clinically Significant.

Listing 16.2.8.1a
Individual Haematology Results

Rando Number	Challenge Agent	Parameter (unit) [Normal Range]	Visit	Date of Assessment	Time of Assessment	Day Relative to Inoculum	Day Relative to IP	Result	Range Indicator	Clinical Assessment
R304	K13	Ery. Mean Corpuscular Volume (fL) [80 – 100]	Screening	13-AUG-2018	11:57	-22	-31	87		
			Safety Visit	03-SEP-2018	08:07	-1	-10	87		
			Admission	13-SEP-2018	09:16	9	1	86		
			Safety Monitoring	15-SEP-2018	09:22	11	3	84		
			Admission 3	16-SEP-2018	09:22	12	4	85		
			Safety Monitoring	18-SEP-2018	08:36	14	6	86		
			Safety Monitoring	22-SEP-2018	09:25	18	10	84		
			Safety Monitoring	25-SEP-2018	08:11	21	13	84		
			Safety Monitoring	28-SEP-2018	08:17	24	16	84		
			End of Study	02-OCT-2018	08:46	28	20	85		

Notes: Day relative to inoculum is the number of days relative to day of administration of the inoculum, where the day of administration = 0.
The inoculum was administered on Study Day 0 for K13-infected subjects and Study Day 1 for 3D7-infected subjects.
Day relative to IP is the number of days relative to day of administration of Artesunate, where the day of dose = 1.
Rando = Randomisation; IP = Artesunate; H = High; L = Low; NCS = Not Clinically Significant; CS = Clinically Significant.

Listing 16.2.8.1a
Individual Haematology Results

Rando Number	Challenge Agent	Parameter (unit) [Normal Range]	Visit	Date of Assessment	Time of Assessment	Day Relative to Inoculum	Day Relative to IP	Result	Range Indicator	Clinical Assessment
R304	K13	Erythrocytes (10^12/L) [4.5 – 6.5]	Screening	13-AUG-2018	11:57	-22	-31	5.3		
			Safety Visit	03-SEP-2018	08:07	-1	-10	5.4		
			Admission	13-SEP-2018	09:16	9	1	5.3		
			Safety Monitoring	15-SEP-2018	09:22	11	3	5.4		
			Admission 3	16-SEP-2018	09:22	12	4	5.4		
			Safety Monitoring	18-SEP-2018	08:36	14	6	5.1		
			Safety Monitoring	22-SEP-2018	09:25	18	10	5.3		
			Safety Monitoring	25-SEP-2018	08:11	21	13	4.8		
			Safety Monitoring	28-SEP-2018	08:17	24	16	4.8		
			End of Study	02-OCT-2018	08:46	28	20	5.1		

Notes: Day relative to inoculum is the number of days relative to day of administration of the inoculum, where the day of administration = 0.
The inoculum was administered on Study Day 0 for K13-infected subjects and Study Day 1 for 3D7-infected subjects.
Day relative to IP is the number of days relative to day of administration of Artesunate, where the day of dose = 1.
Rando = Randomisation; IP = Artesunate; H = High; L = Low; NCS = Not Clinically Significant; CS = Clinically Significant.

Listing 16.2.8.1a
Individual Haematology Results

Rando Number	Challenge Agent	Parameter (unit) [Normal Range]	Visit	Date of Assessment	Time of Assessment	Day Relative to Inoculum	Day Relative to IP	Result	Range Indicator	Clinical Assessment
R304	K13	Hematocrit [0.4 – 0.54]	Screening	13-AUG-2018	11:57	-22	-31	0.46		
			Safety Visit	03-SEP-2018	08:07	-1	-10	0.47		
			Admission	13-SEP-2018	09:16	9	1	0.46		
			Safety Monitoring	15-SEP-2018	09:22	11	3	0.46		
			Admission 3	16-SEP-2018	09:22	12	4	0.46		
			Safety Monitoring	18-SEP-2018	08:36	14	6	0.44		
			Safety Monitoring	22-SEP-2018	09:25	18	10	0.44		
			Safety Monitoring	25-SEP-2018	08:11	21	13	0.41		
			Safety Monitoring	28-SEP-2018	08:17	24	16	0.41		
			End of Study	02-OCT-2018	08:46	28	20	0.44		

Notes: Day relative to inoculum is the number of days relative to day of administration of the inoculum, where the day of administration = 0.
The inoculum was administered on Study Day 0 for K13-infected subjects and Study Day 1 for 3D7-infected subjects.
Day relative to IP is the number of days relative to day of administration of Artesunate, where the day of dose = 1.
Rando = Randomisation; IP = Artesunate; H = High; L = Low; NCS = Not Clinically Significant; CS = Clinically Significant.

Listing 16.2.8.1a
Individual Haematology Results

Rando Number	Challenge Agent	Parameter (unit) [Normal Range]	Visit	Date of Assessment	Time of Assessment	Day Relative to Inoculum	Day Relative to IP	Result	Range Indicator	Clinical Assessment
R304	K13	Hemoglobin (g/L) [135 – 175]	Screening	13-AUG-2018	11:57	-22	-31	165		
			Safety Visit	03-SEP-2018	08:07	-1	-10	167		
			Admission	13-SEP-2018	09:16	9	1	165		
			Safety Monitoring	15-SEP-2018	09:22	11	3	169		
			Admission 3	16-SEP-2018	09:22	12	4	170		
			Safety Monitoring	18-SEP-2018	08:36	14	6	159		
			Safety Monitoring	22-SEP-2018	09:25	18	10	160		
			Safety Monitoring	25-SEP-2018	08:11	21	13	148		
			Safety Monitoring	28-SEP-2018	08:17	24	16	147		
			End of Study	02-OCT-2018	08:46	28	20	158		

Notes: Day relative to inoculum is the number of days relative to day of administration of the inoculum, where the day of administration = 0.
The inoculum was administered on Study Day 0 for K13-infected subjects and Study Day 1 for 3D7-infected subjects.
Day relative to IP is the number of days relative to day of administration of Artesunate, where the day of dose = 1.
Rando = Randomisation; IP = Artesunate; H = High; L = Low; NCS = Not Clinically Significant; CS = Clinically Significant.

Listing 16.2.8.1a
Individual Haematology Results

Rando Number	Challenge Agent	Parameter (unit) [Normal Range]	Visit	Date of Assessment	Time of Assessment	Day Relative to Inoculum	Day Relative to IP	Result	Range Indicator	Clinical Assessment
R304	K13	Leukocytes (10^9/L) [3.5 – 10]	Screening	13-AUG-2018	11:57	-22	-31	6.7		
			Safety Visit	03-SEP-2018	08:07	-1	-10	8.0		
			Admission	13-SEP-2018	09:16	9	1	6.3		
			Safety Monitoring	15-SEP-2018	09:22	11	3	4.6		
			Admission 3	16-SEP-2018	09:22	12	4	6.0		
			Safety Monitoring	18-SEP-2018	08:36	14	6	7.5		
			Safety Monitoring	22-SEP-2018	09:25	18	10	8.6		
			Safety Monitoring	25-SEP-2018	08:11	21	13	7.6		
			Safety Monitoring	28-SEP-2018	08:17	24	16	5.2		
			End of Study	02-OCT-2018	08:46	28	20	6.1		

Notes: Day relative to inoculum is the number of days relative to day of administration of the inoculum, where the day of administration = 0.
The inoculum was administered on Study Day 0 for K13-infected subjects and Study Day 1 for 3D7-infected subjects.
Day relative to IP is the number of days relative to day of administration of Artesunate, where the day of dose = 1.
Rando = Randomisation; IP = Artesunate; H = High; L = Low; NCS = Not Clinically Significant; CS = Clinically Significant.

Listing 16.2.8.1a
Individual Haematology Results

Rando Number	Challenge Agent	Parameter (unit) [Normal Range]	Visit	Date of Assessment	Time of Assessment	Day Relative to Inoculum	Day Relative to IP	Result	Range Indicator	Clinical Assessment
R304	K13	Lymphocytes (10^9/L) [1 – 4]	Screening	13-AUG-2018	11:57	-22	-31	2.24		
			Safety Visit	03-SEP-2018	08:07	-1	-10	2.28		
			Admission	13-SEP-2018	09:16	9	1	1.37		
			Safety Monitoring	15-SEP-2018	09:22	11	3	1.07		
			Admission 3	16-SEP-2018	09:22	12	4	1.21		
			Safety Monitoring	18-SEP-2018	08:36	14	6	2.69		
			Safety Monitoring	22-SEP-2018	09:25	18	10	2.96		
			Safety Monitoring	25-SEP-2018	08:11	21	13	2.40		
			Safety Monitoring	28-SEP-2018	08:17	24	16	1.93		
			End of Study	02-OCT-2018	08:46	28	20	1.85		

Notes: Day relative to inoculum is the number of days relative to day of administration of the inoculum, where the day of administration = 0.
The inoculum was administered on Study Day 0 for K13-infected subjects and Study Day 1 for 3D7-infected subjects.
Day relative to IP is the number of days relative to day of administration of Artesunate, where the day of dose = 1.
Rando = Randomisation; IP = Artesunate; H = High; L = Low; NCS = Not Clinically Significant; CS = Clinically Significant.

Listing 16.2.8.1a
Individual Haematology Results

Rando Number	Challenge Agent	Parameter (unit) [Normal Range]	Visit	Date of Assessment	Time of Assessment	Day Relative to Inoculum	Day Relative to IP	Result	Range Indicator	Clinical Assessment
R304	K13	Monocytes (10^9/L) [0 – 0.9]	Screening	13-AUG-2018	11:57	-22	-31	0.60		
			Safety Visit	03-SEP-2018	08:07	-1	-10	0.81		
			Admission	13-SEP-2018	09:16	9	1	0.94	H	NCS
			Safety Monitoring	15-SEP-2018	09:22	11	3	0.75		
			Admission 3	16-SEP-2018	09:22	12	4	0.89		
			Safety Monitoring	18-SEP-2018	08:36	14	6	0.76		
			Safety Monitoring	22-SEP-2018	09:25	18	10	0.62		
			Safety Monitoring	25-SEP-2018	08:11	21	13	0.67		
			Safety Monitoring	28-SEP-2018	08:17	24	16	0.48		
			End of Study	02-OCT-2018	08:46	28	20	0.42		

Notes: Day relative to inoculum is the number of days relative to day of administration of the inoculum, where the day of administration = 0.
The inoculum was administered on Study Day 0 for K13-infected subjects and Study Day 1 for 3D7-infected subjects.
Day relative to IP is the number of days relative to day of administration of Artesunate, where the day of dose = 1.
Rando = Randomisation; IP = Artesunate; H = High; L = Low; NCS = Not Clinically Significant; CS = Clinically Significant.

Listing 16.2.8.1a
Individual Haematology Results

Rando Number	Challenge Agent	Parameter (unit) [Normal Range]	Visit	Date of Assessment	Time of Assessment	Day Relative to Inoculum	Day Relative to IP	Result	Range Indicator	Clinical Assessment
R304	K13	Neutrophils (10^9/L) [1.5 – 6.5]	Screening	13-AUG-2018	11:57	-22	-31	3.74		
			Safety Visit	03-SEP-2018	08:07	-1	-10	4.83		
			Admission	13-SEP-2018	09:16	9	1	3.93		
			Safety Monitoring	15-SEP-2018	09:22	11	3	2.70		
			Admission 3	16-SEP-2018	09:22	12	4	3.79		
			Safety Monitoring	18-SEP-2018	08:36	14	6	3.89		
			Safety Monitoring	22-SEP-2018	09:25	18	10	4.90		
			Safety Monitoring	25-SEP-2018	08:11	21	13	4.36		
			Safety Monitoring	28-SEP-2018	08:17	24	16	2.70		
			End of Study	02-OCT-2018	08:46	28	20	3.63		

Notes: Day relative to inoculum is the number of days relative to day of administration of the inoculum, where the day of administration = 0.
The inoculum was administered on Study Day 0 for K13-infected subjects and Study Day 1 for 3D7-infected subjects.
Day relative to IP is the number of days relative to day of administration of Artesunate, where the day of dose = 1.
Rando = Randomisation; IP = Artesunate; H = High; L = Low; NCS = Not Clinically Significant; CS = Clinically Significant.

Listing 16.2.8.1a
Individual Haematology Results

Rando Number	Challenge Agent	Parameter (unit) [Normal Range]	Visit	Date of Assessment	Time of Assessment	Day Relative to Inoculum	Day Relative to IP	Result	Range Indicator	Clinical Assessment
R304	K13	Platelets (10^9/L) [150 – 400]	Screening	13-AUG-2018	11:57	-22	-31	202		
			Safety Visit	03-SEP-2018	08:07	-1	-10	223		
			Admission	13-SEP-2018	09:16	9	1	174		
			Safety Monitoring	15-SEP-2018	09:22	11	3	119	L	NCS
			Admission 3	16-SEP-2018	09:22	12	4	114	L	NCS
			Safety Monitoring	18-SEP-2018	08:36	14	6	126	L	NCS
			Safety Monitoring	22-SEP-2018	09:25	18	10	203		
			Safety Monitoring	25-SEP-2018	08:11	21	13	188		
			Safety Monitoring	28-SEP-2018	08:17	24	16	186		
			End of Study	02-OCT-2018	08:46	28	20	194		
		Reticulocytes (10^9/L) [25 – 120]	Safety Visit	03-SEP-2018	08:07	-1	-10	84		
			End of Study	02-OCT-2018	08:46	28	20	124	H	NCS

Notes: Day relative to inoculum is the number of days relative to day of administration of the inoculum, where the day of administration = 0.
The inoculum was administered on Study Day 0 for K13-infected subjects and Study Day 1 for 3D7-infected subjects.
Day relative to IP is the number of days relative to day of administration of Artesunate, where the day of dose = 1.
Rando = Randomisation; IP = Artesunate; H = High; L = Low; NCS = Not Clinically Significant; CS = Clinically Significant.

Listing 16.2.8.1a
Individual Haematology Results

Rando Number	Challenge Agent	Parameter (unit) [Normal Range]	Visit	Date of Assessment	Time of Assessment	Day Relative to Inoculum	Day Relative to IP	Result	Range Indicator	Clinical Assessment
R305	3D7	Basophils (10^9/L) [0 – 0.15]	Screening	13-AUG-2018	09:38	-23	-31	0.04		
			Safety Visit	03-SEP-2018	07:41	-2	-10	0.04		
			Admission	13-SEP-2018	09:29	8	1	0.02		
			Admission 3	16-SEP-2018	09:36	11	4	0.02		
			Safety Monitoring	18-SEP-2018	07:16	13	6	0.03		
			Safety Monitoring	19-SEP-2018	19:23	14	7	0.02		
			Safety Monitoring	22-SEP-2018	07:53	17	10	0.01		
			Safety Monitoring	25-SEP-2018	08:19	20	13	0.02		
			Safety Monitoring	28-SEP-2018	07:23	23	16	0.03		
			End of Study	02-OCT-2018	07:50	27	20	0.03		

Notes: Day relative to inoculum is the number of days relative to day of administration of the inoculum, where the day of administration = 0.
The inoculum was administered on Study Day 0 for K13-infected subjects and Study Day 1 for 3D7-infected subjects.
Day relative to IP is the number of days relative to day of administration of Artesunate, where the day of dose = 1.
Rando = Randomisation; IP = Artesunate; H = High; L = Low; NCS = Not Clinically Significant; CS = Clinically Significant.

Listing 16.2.8.1a
Individual Haematology Results

Rando Number	Challenge Agent	Parameter (unit) [Normal Range]	Visit	Date of Assessment	Time of Assessment	Day Relative to Inoculum	Day Relative to IP	Result	Range Indicator	Clinical Assessment
R305	3D7	Eosinophils (10^9/L) [0 – 0.6]	Screening	13-AUG-2018	09:38	-23	-31	0.18		
			Safety Visit	03-SEP-2018	07:41	-2	-10	0.18		
			Admission	13-SEP-2018	09:29	8	1	0.08		
			Admission 3	16-SEP-2018	09:36	11	4	0.18		
			Safety Monitoring	18-SEP-2018	07:16	13	6	0.12		
			Safety Monitoring	19-SEP-2018	19:23	14	7	0.16		
			Safety Monitoring	22-SEP-2018	07:53	17	10	0.13		
			Safety Monitoring	25-SEP-2018	08:19	20	13	0.07		
			Safety Monitoring	28-SEP-2018	07:23	23	16	0.14		
			End of Study	02-OCT-2018	07:50	27	20	0.24		

Notes: Day relative to inoculum is the number of days relative to day of administration of the inoculum, where the day of administration = 0.
The inoculum was administered on Study Day 0 for K13-infected subjects and Study Day 1 for 3D7-infected subjects.
Day relative to IP is the number of days relative to day of administration of Artesunate, where the day of dose = 1.
Rando = Randomisation; IP = Artesunate; H = High; L = Low; NCS = Not Clinically Significant; CS = Clinically Significant.

Listing 16.2.8.1a
Individual Haematology Results

Rando Number	Challenge Agent	Parameter (unit) [Normal Range]	Visit	Date of Assessment	Time of Assessment	Day Relative to Inoculum	Day Relative to IP	Result	Range Indicator	Clinical Assessment
R305	3D7	Ery. Mean Corpuscular Volume (fL) [80 – 100]	Screening	13-AUG-2018	09:38	-23	-31	91		
			Safety Visit	03-SEP-2018	07:41	-2	-10	92		
			Admission	13-SEP-2018	09:29	8	1	87		
			Admission 3	16-SEP-2018	09:36	11	4	87		
			Safety Monitoring	18-SEP-2018	07:16	13	6	86		
			Safety Monitoring	19-SEP-2018	19:23	14	7	85		
			Safety Monitoring	22-SEP-2018	07:53	17	10	87		
			Safety Monitoring	25-SEP-2018	08:19	20	13	86		
			Safety Monitoring	28-SEP-2018	07:23	23	16	86		
			End of Study	02-OCT-2018	07:50	27	20	86		

Notes: Day relative to inoculum is the number of days relative to day of administration of the inoculum, where the day of administration = 0.
The inoculum was administered on Study Day 0 for K13-infected subjects and Study Day 1 for 3D7-infected subjects.
Day relative to IP is the number of days relative to day of administration of Artesunate, where the day of dose = 1.
Rando = Randomisation; IP = Artesunate; H = High; L = Low; NCS = Not Clinically Significant; CS = Clinically Significant.

Listing 16.2.8.1a
Individual Haematology Results

Rando Number	Challenge Agent	Parameter (unit) [Normal Range]	Visit	Date of Assessment	Time of Assessment	Day Relative to Inoculum	Day Relative to IP	Result	Range Indicator	Clinical Assessment
R305	3D7	Erythrocytes (10^12/L) [4.5 – 6.5]	Screening	13-AUG-2018	09:38	-23	-31	4.9		
			Safety Visit	03-SEP-2018	07:41	-2	-10	5.2		
			Admission	13-SEP-2018	09:29	8	1	4.9		
			Admission 3	16-SEP-2018	09:36	11	4	4.9		
			Safety Monitoring	18-SEP-2018	07:16	13	6	4.6		
			Safety Monitoring	19-SEP-2018	19:23	14	7	4.5		
			Safety Monitoring	22-SEP-2018	07:53	17	10	4.7		
			Safety Monitoring	25-SEP-2018	08:19	20	13	4.7		
			Safety Monitoring	28-SEP-2018	07:23	23	16	4.6		
			End of Study	02-OCT-2018	07:50	27	20	5.0		

Notes: Day relative to inoculum is the number of days relative to day of administration of the inoculum, where the day of administration = 0.
The inoculum was administered on Study Day 0 for K13-infected subjects and Study Day 1 for 3D7-infected subjects.
Day relative to IP is the number of days relative to day of administration of Artesunate, where the day of dose = 1.
Rando = Randomisation; IP = Artesunate; H = High; L = Low; NCS = Not Clinically Significant; CS = Clinically Significant.

Listing 16.2.8.1a
Individual Haematology Results

Rando Number	Challenge Agent	Parameter (unit) [Normal Range]	Visit	Date of Assessment	Time of Assessment	Day Relative to Inoculum	Day Relative to IP	Result	Range Indicator	Clinical Assessment
R305	3D7	Hematocrit [0.4 – 0.54]	Screening	13-AUG-2018	09:38	-23	-31	0.45		
			Safety Visit	03-SEP-2018	07:41	-2	-10	0.47		
			Admission	13-SEP-2018	09:29	8	1	0.43		
			Admission 3	16-SEP-2018	09:36	11	4	0.43		
			Safety Monitoring	18-SEP-2018	07:16	13	6	0.39	L	NCS
			Safety Monitoring	19-SEP-2018	19:23	14	7	0.38	L	NCS
			Safety Monitoring	22-SEP-2018	07:53	17	10	0.41		
			Safety Monitoring	25-SEP-2018	08:19	20	13	0.41		
			Safety Monitoring	28-SEP-2018	07:23	23	16	0.39	L	NCS
			End of Study	02-OCT-2018	07:50	27	20	0.42		

Notes: Day relative to inoculum is the number of days relative to day of administration of the inoculum, where the day of administration = 0.
The inoculum was administered on Study Day 0 for K13-infected subjects and Study Day 1 for 3D7-infected subjects.
Day relative to IP is the number of days relative to day of administration of Artesunate, where the day of dose = 1.
Rando = Randomisation; IP = Artesunate; H = High; L = Low; NCS = Not Clinically Significant; CS = Clinically Significant.

Listing 16.2.8.1a
Individual Haematology Results

Rando Number	Challenge Agent	Parameter (unit) [Normal Range]	Visit	Date of Assessment	Time of Assessment	Day Relative to Inoculum	Day Relative to IP	Result	Range Indicator	Clinical Assessment
R305	3D7	Hemoglobin (g/L) [135 – 175]	Screening	13-AUG-2018	09:38	-23	-31	151		
			Safety Visit	03-SEP-2018	07:41	-2	-10	158		
			Admission	13-SEP-2018	09:29	8	1	151		
			Admission 3	16-SEP-2018	09:36	11	4	152		
			Safety Monitoring	18-SEP-2018	07:16	13	6	139		
			Safety Monitoring	19-SEP-2018	19:23	14	7	135		
			Safety Monitoring	22-SEP-2018	07:53	17	10	143		
			Safety Monitoring	25-SEP-2018	08:19	20	13	143		
			Safety Monitoring	28-SEP-2018	07:23	23	16	140		
			End of Study	02-OCT-2018	07:50	27	20	153		

Notes: Day relative to inoculum is the number of days relative to day of administration of the inoculum, where the day of administration = 0.
The inoculum was administered on Study Day 0 for K13-infected subjects and Study Day 1 for 3D7-infected subjects.
Day relative to IP is the number of days relative to day of administration of Artesunate, where the day of dose = 1.
Rando = Randomisation; IP = Artesunate; H = High; L = Low; NCS = Not Clinically Significant; CS = Clinically Significant.

Listing 16.2.8.1a
Individual Haematology Results

Rando Number	Challenge Agent	Parameter (unit) [Normal Range]	Visit	Date of Assessment	Time of Assessment	Day Relative to Inoculum	Day Relative to IP	Result	Range Indicator	Clinical Assessment
R305	3D7	Leukocytes (10^9/L) [3.5 – 10]	Screening	13-AUG-2018	09:38	-23	-31	3.9		
			Safety Visit	03-SEP-2018	07:41	-2	-10	3.6		
			Admission	13-SEP-2018	09:29	8	1	3.3	L	NCS
			Admission 3	16-SEP-2018	09:36	11	4	2.4	L	CS
			Safety Monitoring	18-SEP-2018	07:16	13	6	3.2	L	NCS
			Safety Monitoring	19-SEP-2018	19:23	14	7	3.6		
			Safety Monitoring	22-SEP-2018	07:53	17	10	3.1	L	NCS
			Safety Monitoring	25-SEP-2018	08:19	20	13	3.7		
			Safety Monitoring	28-SEP-2018	07:23	23	16	3.4	L	NCS
			End of Study	02-OCT-2018	07:50	27	20	4.7		

Notes: Day relative to inoculum is the number of days relative to day of administration of the inoculum, where the day of administration = 0.
The inoculum was administered on Study Day 0 for K13-infected subjects and Study Day 1 for 3D7-infected subjects.
Day relative to IP is the number of days relative to day of administration of Artesunate, where the day of dose = 1.
Rando = Randomisation; IP = Artesunate; H = High; L = Low; NCS = Not Clinically Significant; CS = Clinically Significant.

Listing 16.2.8.1a
Individual Haematology Results

Rando Number	Challenge Agent	Parameter (unit) [Normal Range]	Visit	Date of Assessment	Time of Assessment	Day Relative to Inoculum	Day Relative to IP	Result	Range Indicator	Clinical Assessment
R305	3D7	Lymphocytes (10^9/L) [1 – 4]	Screening	13-AUG-2018	09:38	-23	-31	1.80		
			Safety Visit	03-SEP-2018	07:41	-2	-10	1.61		
			Admission	13-SEP-2018	09:29	8	1	0.92	L	NCS
			Admission 3	16-SEP-2018	09:36	11	4	0.99	L	NCS
			Safety Monitoring	18-SEP-2018	07:16	13	6	1.59		
			Safety Monitoring	19-SEP-2018	19:23	14	7	1.42		
			Safety Monitoring	22-SEP-2018	07:53	17	10	1.47		
			Safety Monitoring	25-SEP-2018	08:19	20	13	1.81		
			Safety Monitoring	28-SEP-2018	07:23	23	16	1.67		
			End of Study	02-OCT-2018	07:50	27	20	1.34		

Notes: Day relative to inoculum is the number of days relative to day of administration of the inoculum, where the day of administration = 0.
The inoculum was administered on Study Day 0 for K13-infected subjects and Study Day 1 for 3D7-infected subjects.
Day relative to IP is the number of days relative to day of administration of Artesunate, where the day of dose = 1.
Rando = Randomisation; IP = Artesunate; H = High; L = Low; NCS = Not Clinically Significant; CS = Clinically Significant.

Listing 16.2.8.1a
Individual Haematology Results

Rando Number	Challenge Agent	Parameter (unit) [Normal Range]	Visit	Date of Assessment	Time of Assessment	Day Relative to Inoculum	Day Relative to IP	Result	Range Indicator	Clinical Assessment
R305	3D7	Monocytes (10^9/L) [0 – 0.9]	Screening	13-AUG-2018	09:38	-23	-31	0.26		
			Safety Visit	03-SEP-2018	07:41	-2	-10	0.26		
			Admission	13-SEP-2018	09:29	8	1	0.49		
			Admission 3	16-SEP-2018	09:36	11	4	0.36		
			Safety Monitoring	18-SEP-2018	07:16	13	6	0.36		
			Safety Monitoring	19-SEP-2018	19:23	14	7	0.36		
			Safety Monitoring	22-SEP-2018	07:53	17	10	0.39		
			Safety Monitoring	25-SEP-2018	08:19	20	13	0.30		
			Safety Monitoring	28-SEP-2018	07:23	23	16	0.23		
			End of Study	02-OCT-2018	07:50	27	20	0.31		

Notes: Day relative to inoculum is the number of days relative to day of administration of the inoculum, where the day of administration = 0.
The inoculum was administered on Study Day 0 for K13-infected subjects and Study Day 1 for 3D7-infected subjects.
Day relative to IP is the number of days relative to day of administration of Artesunate, where the day of dose = 1.
Rando = Randomisation; IP = Artesunate; H = High; L = Low; NCS = Not Clinically Significant; CS = Clinically Significant.

Listing 16.2.8.1a
Individual Haematology Results

Rando Number	Challenge Agent	Parameter (unit) [Normal Range]	Visit	Date of Assessment	Time of Assessment	Day Relative to Inoculum	Day Relative to IP	Result	Range Indicator	Clinical Assessment
R305	3D7	Neutrophils (10^9/L) [1.5 – 6.5]	Screening	13-AUG-2018	09:38	-23	-31	1.64		
			Safety Visit	03-SEP-2018	07:41	-2	-10	1.53		
			Admission	13-SEP-2018	09:29	8	1	1.82		
			Admission 3	16-SEP-2018	09:36	11	4	0.89	L	CS
			Safety Monitoring	18-SEP-2018	07:16	13	6	1.08	L	CS
			Safety Monitoring	19-SEP-2018	19:23	14	7	1.67		
			Safety Monitoring	22-SEP-2018	07:53	17	10	1.11	L	CS
			Safety Monitoring	25-SEP-2018	08:19	20	13	1.50		
			Safety Monitoring	28-SEP-2018	07:23	23	16	1.32	L	CS
			End of Study	02-OCT-2018	07:50	27	20	2.73		

Notes: Day relative to inoculum is the number of days relative to day of administration of the inoculum, where the day of administration = 0.
The inoculum was administered on Study Day 0 for K13-infected subjects and Study Day 1 for 3D7-infected subjects.
Day relative to IP is the number of days relative to day of administration of Artesunate, where the day of dose = 1.
Rando = Randomisation; IP = Artesunate; H = High; L = Low; NCS = Not Clinically Significant; CS = Clinically Significant.

Listing 16.2.8.1a
Individual Haematology Results

Rando Number	Challenge Agent	Parameter (unit) [Normal Range]	Visit	Date of Assessment	Time of Assessment	Day Relative to Inoculum	Day Relative to IP	Result	Range Indicator	Clinical Assessment
R305	3D7	Platelets (10^9/L) [150 – 400]	Screening	13-AUG-2018	09:38	-23	-31	218		
			Safety Visit	03-SEP-2018	07:41	-2	-10	207		
			Admission	13-SEP-2018	09:29	8	1	180		
			Admission 3	16-SEP-2018	09:36	11	4	141	L	NCS
			Safety Monitoring	18-SEP-2018	07:16	13	6	159		
			Safety Monitoring	19-SEP-2018	19:23	14	7	179		
			Safety Monitoring	22-SEP-2018	07:53	17	10	225		
			Safety Monitoring	25-SEP-2018	08:19	20	13	295		
			Safety Monitoring	28-SEP-2018	07:23	23	16	308		
			End of Study	02-OCT-2018	07:50	27	20	266		
		Reticulocytes (10^9/L) [25 – 120]	Safety Visit	03-SEP-2018	07:41	-2	-10	82		
			End of Study	02-OCT-2018	07:50	27	20	93		

Notes: Day relative to inoculum is the number of days relative to day of administration of the inoculum, where the day of administration = 0.
The inoculum was administered on Study Day 0 for K13-infected subjects and Study Day 1 for 3D7-infected subjects.
Day relative to IP is the number of days relative to day of administration of Artesunate, where the day of dose = 1.
Rando = Randomisation; IP = Artesunate; H = High; L = Low; NCS = Not Clinically Significant; CS = Clinically Significant.

Listing 16.2.8.1a
Individual Haematology Results

Rando Number	Challenge Agent	Parameter (unit) [Normal Range]	Visit	Date of Assessment	Time of Assessment	Day Relative to Inoculum	Day Relative to IP	Result	Range Indicator	Clinical Assessment
R306	3D7	Basophils (10^9/L) [0 – 0.15]	Screening	28-AUG-2018	11:39	-8	-16	0.07		
			Safety Visit	03-SEP-2018	07:31	-2	-10	0.07		
			Admission	13-SEP-2018	09:41	8	1	0.04		
			Admission 3	16-SEP-2018	09:45	11	4	0.04		
			Safety Monitoring	18-SEP-2018	08:04	13	6	0.07		
			Safety Monitoring	19-SEP-2018	20:08	14	7	0.06		
			Safety Monitoring	22-SEP-2018	07:34	17	10	0.05		
			Safety Monitoring	25-SEP-2018	07:30	20	13	0.07		
			Safety Monitoring	28-SEP-2018	07:11	23	16	0.10		
			End of Study	02-OCT-2018	08:20	27	20	0.07		

Notes: Day relative to inoculum is the number of days relative to day of administration of the inoculum, where the day of administration = 0.
The inoculum was administered on Study Day 0 for K13-infected subjects and Study Day 1 for 3D7-infected subjects.
Day relative to IP is the number of days relative to day of administration of Artesunate, where the day of dose = 1.
Rando = Randomisation; IP = Artesunate; H = High; L = Low; NCS = Not Clinically Significant; CS = Clinically Significant.

Listing 16.2.8.1a
Individual Haematology Results

Rando Number	Challenge Agent	Parameter (unit) [Normal Range]	Visit	Date of Assessment	Time of Assessment	Day Relative to Inoculum	Day Relative to IP	Result	Range Indicator	Clinical Assessment
R306	3D7	Eosinophils (10^9/L) [0 – 0.6]	Screening	28-AUG-2018	11:39	-8	-16	0.30		
			Safety Visit	03-SEP-2018	07:31	-2	-10	0.27		
			Admission	13-SEP-2018	09:41	8	1	0.30		
			Admission 3	16-SEP-2018	09:45	11	4	0.16		
			Safety Monitoring	18-SEP-2018	08:04	13	6	0.31		
			Safety Monitoring	19-SEP-2018	20:08	14	7	0.32		
			Safety Monitoring	22-SEP-2018	07:34	17	10	0.29		
			Safety Monitoring	25-SEP-2018	07:30	20	13	0.32		
			Safety Monitoring	28-SEP-2018	07:11	23	16	0.42		
			End of Study	02-OCT-2018	08:20	27	20	0.27		

Notes: Day relative to inoculum is the number of days relative to day of administration of the inoculum, where the day of administration = 0.
The inoculum was administered on Study Day 0 for K13-infected subjects and Study Day 1 for 3D7-infected subjects.
Day relative to IP is the number of days relative to day of administration of Artesunate, where the day of dose = 1.
Rando = Randomisation; IP = Artesunate; H = High; L = Low; NCS = Not Clinically Significant; CS = Clinically Significant.

Listing 16.2.8.1a
Individual Haematology Results

Rando Number	Challenge Agent	Parameter (unit) [Normal Range]	Visit	Date of Assessment	Time of Assessment	Day Relative to Inoculum	Day Relative to IP	Result	Range Indicator	Clinical Assessment
R306	3D7	Ery. Mean Corpuscular Volume (fL) [80 – 100]	Screening	28-AUG-2018	11:39	-8	-16	86		
			Safety Visit	03-SEP-2018	07:31	-2	-10	88		
			Admission	13-SEP-2018	09:41	8	1	86		
			Admission 3	16-SEP-2018	09:45	11	4	85		
			Safety Monitoring	18-SEP-2018	08:04	13	6	86		
			Safety Monitoring	19-SEP-2018	20:08	14	7	85		
			Safety Monitoring	22-SEP-2018	07:34	17	10	85		
			Safety Monitoring	25-SEP-2018	07:30	20	13	86		
			Safety Monitoring	28-SEP-2018	07:11	23	16	86		
			End of Study	02-OCT-2018	08:20	27	20	85		

Notes: Day relative to inoculum is the number of days relative to day of administration of the inoculum, where the day of administration = 0.
The inoculum was administered on Study Day 0 for K13-infected subjects and Study Day 1 for 3D7-infected subjects.
Day relative to IP is the number of days relative to day of administration of Artesunate, where the day of dose = 1.
Rando = Randomisation; IP = Artesunate; H = High; L = Low; NCS = Not Clinically Significant; CS = Clinically Significant.

Listing 16.2.8.1a
Individual Haematology Results

Rando Number	Challenge Agent	Parameter (unit) [Normal Range]	Visit	Date of Assessment	Time of Assessment	Day Relative to Inoculum	Day Relative to IP	Result	Range Indicator	Clinical Assessment
R306	3D7	Erythrocytes (10^12/L) [4.5 – 6.5]	Screening	28-AUG-2018	11:39	-8	-16	5.0		
			Safety Visit	03-SEP-2018	07:31	-2	-10	4.9		
			Admission	13-SEP-2018	09:41	8	1	4.8		
			Admission 3	16-SEP-2018	09:45	11	4	4.8		
			Safety Monitoring	18-SEP-2018	08:04	13	6	4.9		
			Safety Monitoring	19-SEP-2018	20:08	14	7	4.4	L	NCS
			Safety Monitoring	22-SEP-2018	07:34	17	10	4.5		
			Safety Monitoring	25-SEP-2018	07:30	20	13	4.6		
			Safety Monitoring	28-SEP-2018	07:11	23	16	4.8		
			End of Study	02-OCT-2018	08:20	27	20	4.6		

Notes: Day relative to inoculum is the number of days relative to day of administration of the inoculum, where the day of administration = 0.
The inoculum was administered on Study Day 0 for K13-infected subjects and Study Day 1 for 3D7-infected subjects.
Day relative to IP is the number of days relative to day of administration of Artesunate, where the day of dose = 1.
Rando = Randomisation; IP = Artesunate; H = High; L = Low; NCS = Not Clinically Significant; CS = Clinically Significant.

Listing 16.2.8.1a
Individual Haematology Results

Rando Number	Challenge Agent	Parameter (unit) [Normal Range]	Visit	Date of Assessment	Time of Assessment	Day Relative to Inoculum	Day Relative to IP	Result	Range Indicator	Clinical Assessment
R306	3D7	Hematocrit [0.4 – 0.54]	Screening	28-AUG-2018	11:39	-8	-16	0.43		
			Safety Visit	03-SEP-2018	07:31	-2	-10	0.43		
			Admission	13-SEP-2018	09:41	8	1	0.41		
			Admission 3	16-SEP-2018	09:45	11	4	0.41		
			Safety Monitoring	18-SEP-2018	08:04	13	6	0.42		
			Safety Monitoring	19-SEP-2018	20:08	14	7	0.38	L	NCS
			Safety Monitoring	22-SEP-2018	07:34	17	10	0.38	L	NCS
			Safety Monitoring	25-SEP-2018	07:30	20	13	0.39	L	NCS
			Safety Monitoring	28-SEP-2018	07:11	23	16	0.42		
			End of Study	02-OCT-2018	08:20	27	20	0.39	L	NCS

Notes: Day relative to inoculum is the number of days relative to day of administration of the inoculum, where the day of administration = 0.
The inoculum was administered on Study Day 0 for K13-infected subjects and Study Day 1 for 3D7-infected subjects.
Day relative to IP is the number of days relative to day of administration of Artesunate, where the day of dose = 1.
Rando = Randomisation; IP = Artesunate; H = High; L = Low; NCS = Not Clinically Significant; CS = Clinically Significant.

Listing 16.2.8.1a
Individual Haematology Results

Rando Number	Challenge Agent	Parameter (unit) [Normal Range]	Visit	Date of Assessment	Time of Assessment	Day Relative to Inoculum	Day Relative to IP	Result	Range Indicator	Clinical Assessment
R306	3D7	Hemoglobin (g/L) [135 – 175]	Screening	28-AUG-2018	11:39	-8	-16	155		
			Safety Visit	03-SEP-2018	07:31	-2	-10	148		
			Admission	13-SEP-2018	09:41	8	1	147		
			Admission 3	16-SEP-2018	09:45	11	4	146		
			Safety Monitoring	18-SEP-2018	08:04	13	6	150		
			Safety Monitoring	19-SEP-2018	20:08	14	7	135		
			Safety Monitoring	22-SEP-2018	07:34	17	10	136		
			Safety Monitoring	25-SEP-2018	07:30	20	13	138		
			Safety Monitoring	28-SEP-2018	07:11	23	16	145		
			End of Study	02-OCT-2018	08:20	27	20	141		

Notes: Day relative to inoculum is the number of days relative to day of administration of the inoculum, where the day of administration = 0.
The inoculum was administered on Study Day 0 for K13-infected subjects and Study Day 1 for 3D7-infected subjects.
Day relative to IP is the number of days relative to day of administration of Artesunate, where the day of dose = 1.
Rando = Randomisation; IP = Artesunate; H = High; L = Low; NCS = Not Clinically Significant; CS = Clinically Significant.

Listing 16.2.8.1a
Individual Haematology Results

Rando Number	Challenge Agent	Parameter (unit) [Normal Range]	Visit	Date of Assessment	Time of Assessment	Day Relative to Inoculum	Day Relative to IP	Result	Range Indicator	Clinical Assessment
R306	3D7	Leukocytes (10^9/L) [3.5 – 10]	Screening	28-AUG-2018	11:39	-8	-16	6.2		
			Safety Visit	03-SEP-2018	07:31	-2	-10	5.9		
			Admission	13-SEP-2018	09:41	8	1	5.4		
			Admission 3	16-SEP-2018	09:45	11	4	5.2		
			Safety Monitoring	18-SEP-2018	08:04	13	6	6.1		
			Safety Monitoring	19-SEP-2018	20:08	14	7	6.6		
			Safety Monitoring	22-SEP-2018	07:34	17	10	5.0		
			Safety Monitoring	25-SEP-2018	07:30	20	13	5.6		
			Safety Monitoring	28-SEP-2018	07:11	23	16	6.4		
			End of Study	02-OCT-2018	08:20	27	20	5.1		

Notes: Day relative to inoculum is the number of days relative to day of administration of the inoculum, where the day of administration = 0.
The inoculum was administered on Study Day 0 for K13-infected subjects and Study Day 1 for 3D7-infected subjects.
Day relative to IP is the number of days relative to day of administration of Artesunate, where the day of dose = 1.
Rando = Randomisation; IP = Artesunate; H = High; L = Low; NCS = Not Clinically Significant; CS = Clinically Significant.

Listing 16.2.8.1a
Individual Haematology Results

Rando Number	Challenge Agent	Parameter (unit) [Normal Range]	Visit	Date of Assessment	Time of Assessment	Day Relative to Inoculum	Day Relative to IP	Result	Range Indicator	Clinical Assessment
R306	3D7	Lymphocytes (10^9/L) [1 – 4]	Screening	28-AUG-2018	11:39	-8	-16	1.48		
			Safety Visit	03-SEP-2018	07:31	-2	-10	1.44		
			Admission	13-SEP-2018	09:41	8	1	1.36		
			Admission 3	16-SEP-2018	09:45	11	4	1.11		
			Safety Monitoring	18-SEP-2018	08:04	13	6	1.52		
			Safety Monitoring	19-SEP-2018	20:08	14	7	1.45		
			Safety Monitoring	22-SEP-2018	07:34	17	10	1.58		
			Safety Monitoring	25-SEP-2018	07:30	20	13	1.61		
			Safety Monitoring	28-SEP-2018	07:11	23	16	1.68		
			End of Study	02-OCT-2018	08:20	27	20	1.39		

Notes: Day relative to inoculum is the number of days relative to day of administration of the inoculum, where the day of administration = 0.
The inoculum was administered on Study Day 0 for K13-infected subjects and Study Day 1 for 3D7-infected subjects.
Day relative to IP is the number of days relative to day of administration of Artesunate, where the day of dose = 1.
Rando = Randomisation; IP = Artesunate; H = High; L = Low; NCS = Not Clinically Significant; CS = Clinically Significant.

Listing 16.2.8.1a
Individual Haematology Results

Rando Number	Challenge Agent	Parameter (unit) [Normal Range]	Visit	Date of Assessment	Time of Assessment	Day Relative to Inoculum	Day Relative to IP	Result	Range Indicator	Clinical Assessment
R306	3D7	Monocytes (10^9/L) [0 – 0.9]	Screening	28-AUG-2018	11:39	-8	-16	0.46		
			Safety Visit	03-SEP-2018	07:31	-2	-10	0.48		
			Admission	13-SEP-2018	09:41	8	1	0.56		
			Admission 3	16-SEP-2018	09:45	11	4	0.61		
			Safety Monitoring	18-SEP-2018	08:04	13	6	0.58		
			Safety Monitoring	19-SEP-2018	20:08	14	7	0.60		
			Safety Monitoring	22-SEP-2018	07:34	17	10	0.54		
			Safety Monitoring	25-SEP-2018	07:30	20	13	0.43		
			Safety Monitoring	28-SEP-2018	07:11	23	16	0.50		
			End of Study	02-OCT-2018	08:20	27	20	0.47		

Notes: Day relative to inoculum is the number of days relative to day of administration of the inoculum, where the day of administration = 0.
The inoculum was administered on Study Day 0 for K13-infected subjects and Study Day 1 for 3D7-infected subjects.
Day relative to IP is the number of days relative to day of administration of Artesunate, where the day of dose = 1.
Rando = Randomisation; IP = Artesunate; H = High; L = Low; NCS = Not Clinically Significant; CS = Clinically Significant.

Listing 16.2.8.1a
Individual Haematology Results

Rando Number	Challenge Agent	Parameter (unit) [Normal Range]	Visit	Date of Assessment	Time of Assessment	Day Relative to Inoculum	Day Relative to IP	Result	Range Indicator	Clinical Assessment
R306	3D7	Neutrophils (10^9/L) [1.5 – 6.5]	Screening	28-AUG-2018	11:39	-8	-16	3.87		
			Safety Visit	03-SEP-2018	07:31	-2	-10	3.65		
			Admission	13-SEP-2018	09:41	8	1	3.11		
			Admission 3	16-SEP-2018	09:45	11	4	3.28		
			Safety Monitoring	18-SEP-2018	08:04	13	6	3.59		
			Safety Monitoring	19-SEP-2018	20:08	14	7	4.14		
			Safety Monitoring	22-SEP-2018	07:34	17	10	2.54		
			Safety Monitoring	25-SEP-2018	07:30	20	13	3.14		
			Safety Monitoring	28-SEP-2018	07:11	23	16	3.68		
			End of Study	02-OCT-2018	08:20	27	20	2.88		

Notes: Day relative to inoculum is the number of days relative to day of administration of the inoculum, where the day of administration = 0.
The inoculum was administered on Study Day 0 for K13-infected subjects and Study Day 1 for 3D7-infected subjects.
Day relative to IP is the number of days relative to day of administration of Artesunate, where the day of dose = 1.
Rando = Randomisation; IP = Artesunate; H = High; L = Low; NCS = Not Clinically Significant; CS = Clinically Significant.

Listing 16.2.8.1a
Individual Haematology Results

Rando Number	Challenge Agent	Parameter (unit) [Normal Range]	Visit	Date of Assessment	Time of Assessment	Day Relative to Inoculum	Day Relative to IP	Result	Range Indicator	Clinical Assessment
R306	3D7	Platelets (10^9/L) [150 – 400]	Screening	28-AUG-2018	11:39	-8	-16	189		
			Safety Visit	03-SEP-2018	07:31	-2	-10	206		
			Admission	13-SEP-2018	09:41	8	1	209		
			Admission 3	16-SEP-2018	09:45	11	4	178		
			Safety Monitoring	18-SEP-2018	08:04	13	6	212		
			Safety Monitoring	19-SEP-2018	20:08	14	7	176		
			Safety Monitoring	22-SEP-2018	07:34	17	10	205		
			Safety Monitoring	25-SEP-2018	07:30	20	13	220		
			Safety Monitoring	28-SEP-2018	07:11	23	16	255		
			End of Study	02-OCT-2018	08:20	27	20	232		
		Reticulocytes (10^9/L) [25 – 120]	Safety Visit	03-SEP-2018	07:31	-2	-10	59		
			End of Study	02-OCT-2018	08:20	27	20	84		

Notes: Day relative to inoculum is the number of days relative to day of administration of the inoculum, where the day of administration = 0.
The inoculum was administered on Study Day 0 for K13-infected subjects and Study Day 1 for 3D7-infected subjects.
Day relative to IP is the number of days relative to day of administration of Artesunate, where the day of dose = 1.
Rando = Randomisation; IP = Artesunate; H = High; L = Low; NCS = Not Clinically Significant; CS = Clinically Significant.

Listing 16.2.8.1a
Individual Haematology Results

Rando Number	Challenge Agent	Parameter (unit) [Normal Range]	Visit	Date of Assessment	Time of Assessment	Day Relative to Inoculum	Day Relative to IP	Result	Range Indicator	Clinical Assessment
R307	3D7	Basophils (10^9/L) [0 – 0.15]	Screening	07-AUG-2018	12:31	-29	-37	0.03		
			Safety Visit	03-SEP-2018	07:53	-2	-10	0.04		
			Admission	13-SEP-2018	09:51	8	1	0.02		
			Admission 3	16-SEP-2018	09:55	11	4	0.01		
			Safety Monitoring	18-SEP-2018	08:08	13	6	0.03		
			Safety Monitoring	19-SEP-2018	19:23	14	7	0.04		
			Safety Monitoring	22-SEP-2018	08:38	17	10	0.03		
			Safety Monitoring	25-SEP-2018	07:36	20	13	0.02		
			Safety Monitoring	28-SEP-2018	07:40	23	16	0.03		
			End of Study	02-OCT-2018	07:21	27	20	0.04		

Notes: Day relative to inoculum is the number of days relative to day of administration of the inoculum, where the day of administration = 0.
The inoculum was administered on Study Day 0 for K13-infected subjects and Study Day 1 for 3D7-infected subjects.
Day relative to IP is the number of days relative to day of administration of Artesunate, where the day of dose = 1.
Rando = Randomisation; IP = Artesunate; H = High; L = Low; NCS = Not Clinically Significant; CS = Clinically Significant.

Listing 16.2.8.1a
Individual Haematology Results

Rando Number	Challenge Agent	Parameter (unit) [Normal Range]	Visit	Date of Assessment	Time of Assessment	Day Relative to Inoculum	Day Relative to IP	Result	Range Indicator	Clinical Assessment
R307	3D7	Eosinophils (10^9/L) [0 – 0.6]	Screening	07-AUG-2018	12:31	-29	-37	0.24		
			Safety Visit	03-SEP-2018	07:53	-2	-10	0.29		
			Admission	13-SEP-2018	09:51	8	1	0.05		
			Admission 3	16-SEP-2018	09:55	11	4	0.03		
			Safety Monitoring	18-SEP-2018	08:08	13	6	0.14		
			Safety Monitoring	19-SEP-2018	19:23	14	7	0.11		
			Safety Monitoring	22-SEP-2018	08:38	17	10	0.18		
			Safety Monitoring	25-SEP-2018	07:36	20	13	0.09		
			Safety Monitoring	28-SEP-2018	07:40	23	16	0.12		
			End of Study	02-OCT-2018	07:21	27	20	0.14		

Notes: Day relative to inoculum is the number of days relative to day of administration of the inoculum, where the day of administration = 0.
The inoculum was administered on Study Day 0 for K13-infected subjects and Study Day 1 for 3D7-infected subjects.
Day relative to IP is the number of days relative to day of administration of Artesunate, where the day of dose = 1.
Rando = Randomisation; IP = Artesunate; H = High; L = Low; NCS = Not Clinically Significant; CS = Clinically Significant.

Listing 16.2.8.1a
Individual Haematology Results

Rando Number	Challenge Agent	Parameter (unit) [Normal Range]	Visit	Date of Assessment	Time of Assessment	Day Relative to Inoculum	Day Relative to IP	Result	Range Indicator	Clinical Assessment
R307	3D7	Ery. Mean Corpuscular Volume (fL) [80 – 100]	Screening	07-AUG-2018	12:31	-29	-37	95		
			Safety Visit	03-SEP-2018	07:53	-2	-10	94		
			Admission	13-SEP-2018	09:51	8	1	90		
			Admission 3	16-SEP-2018	09:55	11	4	89		
			Safety Monitoring	18-SEP-2018	08:08	13	6	89		
			Safety Monitoring	19-SEP-2018	19:23	14	7	89		
			Safety Monitoring	22-SEP-2018	08:38	17	10	89		
			Safety Monitoring	25-SEP-2018	07:36	20	13	91		
			Safety Monitoring	28-SEP-2018	07:40	23	16	90		
			End of Study	02-OCT-2018	07:21	27	20	91		

Notes: Day relative to inoculum is the number of days relative to day of administration of the inoculum, where the day of administration = 0.
The inoculum was administered on Study Day 0 for K13-infected subjects and Study Day 1 for 3D7-infected subjects.
Day relative to IP is the number of days relative to day of administration of Artesunate, where the day of dose = 1.
Rando = Randomisation; IP = Artesunate; H = High; L = Low; NCS = Not Clinically Significant; CS = Clinically Significant.

Listing 16.2.8.1a
Individual Haematology Results

Rando Number	Challenge Agent	Parameter (unit) [Normal Range]	Visit	Date of Assessment	Time of Assessment	Day Relative to Inoculum	Day Relative to IP	Result	Range Indicator	Clinical Assessment
R307	3D7	Erythrocytes (10^12/L) [3.9 – 5.6]	Screening	07-AUG-2018	12:31	-29	-37	3.8	L	NCS
			Safety Visit	03-SEP-2018	07:53	-2	-10	4.3		
			Admission	13-SEP-2018	09:51	8	1	4.1		
			Admission 3	16-SEP-2018	09:55	11	4	3.9		
			Safety Monitoring	18-SEP-2018	08:08	13	6	3.7	L	NCS
			Safety Monitoring	19-SEP-2018	19:23	14	7	3.5	L	NCS
			Safety Monitoring	22-SEP-2018	08:38	17	10	3.6	L	NCS
			Safety Monitoring	25-SEP-2018	07:36	20	13	3.8	L	NCS
			Safety Monitoring	28-SEP-2018	07:40	23	16	3.6	L	NCS
			End of Study	02-OCT-2018	07:21	27	20	3.5	L	NCS

Notes: Day relative to inoculum is the number of days relative to day of administration of the inoculum, where the day of administration = 0.
The inoculum was administered on Study Day 0 for K13-infected subjects and Study Day 1 for 3D7-infected subjects.
Day relative to IP is the number of days relative to day of administration of Artesunate, where the day of dose = 1.
Rando = Randomisation; IP = Artesunate; H = High; L = Low; NCS = Not Clinically Significant; CS = Clinically Significant.

Listing 16.2.8.1a
Individual Haematology Results

Rando Number	Challenge Agent	Parameter (unit) [Normal Range]	Visit	Date of Assessment	Time of Assessment	Day Relative to Inoculum	Day Relative to IP	Result	Range Indicator	Clinical Assessment
R307	3D7	Hematocrit [0.35 – 0.47]	Screening	07-AUG-2018	12:31	-29	-37	0.36		
			Safety Visit	03-SEP-2018	07:53	-2	-10	0.40		
			Admission	13-SEP-2018	09:51	8	1	0.37		
			Admission 3	16-SEP-2018	09:55	11	4	0.35		
			Safety Monitoring	18-SEP-2018	08:08	13	6	0.33	L	NCS
			Safety Monitoring	19-SEP-2018	19:23	14	7	0.31	L	NCS
			Safety Monitoring	22-SEP-2018	08:38	17	10	0.32	L	NCS
			Safety Monitoring	25-SEP-2018	07:36	20	13	0.35		
			Safety Monitoring	28-SEP-2018	07:40	23	16	0.32	L	NCS
			End of Study	02-OCT-2018	07:21	27	20	0.31	L	NCS
			Unscheduled	05-OCT-2018	07:55	30	23	0.36		

Notes: Day relative to inoculum is the number of days relative to day of administration of the inoculum, where the day of administration = 0.
The inoculum was administered on Study Day 0 for K13-infected subjects and Study Day 1 for 3D7-infected subjects.
Day relative to IP is the number of days relative to day of administration of Artesunate, where the day of dose = 1.
Rando = Randomisation; IP = Artesunate; H = High; L = Low; NCS = Not Clinically Significant; CS = Clinically Significant.

Listing 16.2.8.1a
Individual Haematology Results

Rando Number	Challenge Agent	Parameter (unit) [Normal Range]	Visit	Date of Assessment	Time of Assessment	Day Relative to Inoculum	Day Relative to IP	Result	Range Indicator	Clinical Assessment
R307	3D7	Hemoglobin (g/L) [115 – 165]	Screening	07-AUG-2018	12:31	-29	-37	119		
			Safety Visit	03-SEP-2018	07:53	-2	-10	134		
			Admission	13-SEP-2018	09:51	8	1	124		
			Admission 3	16-SEP-2018	09:55	11	4	122		
			Safety Monitoring	18-SEP-2018	08:08	13	6	114	L	NCS
			Safety Monitoring	19-SEP-2018	19:23	14	7	109	L	NCS
			Safety Monitoring	22-SEP-2018	08:38	17	10	112	L	NCS
			Safety Monitoring	25-SEP-2018	07:36	20	13	117		
			Safety Monitoring	28-SEP-2018	07:40	23	16	108	L	NCS
			End of Study	02-OCT-2018	07:21	27	20	106	L	NCS
			Unscheduled	05-OCT-2018	07:55	30	23	119		

Notes: Day relative to inoculum is the number of days relative to day of administration of the inoculum, where the day of administration = 0.
The inoculum was administered on Study Day 0 for K13-infected subjects and Study Day 1 for 3D7-infected subjects.
Day relative to IP is the number of days relative to day of administration of Artesunate, where the day of dose = 1.
Rando = Randomisation; IP = Artesunate; H = High; L = Low; NCS = Not Clinically Significant; CS = Clinically Significant.

Listing 16.2.8.1a
Individual Haematology Results

Rando Number	Challenge Agent	Parameter (unit) [Normal Range]	Visit	Date of Assessment	Time of Assessment	Day Relative to Inoculum	Day Relative to IP	Result	Range Indicator	Clinical Assessment
R307	3D7	Leukocytes (10^9/L) [3.5 – 12]	Screening	07-AUG-2018	12:31	-29	-37	6.6		
			Safety Visit	03-SEP-2018	07:53	-2	-10	8.4		
			Admission	13-SEP-2018	09:51	8	1	4.7		
			Admission 3	16-SEP-2018	09:55	11	4	3.2	L	NCS
			Safety Monitoring	18-SEP-2018	08:08	13	6	5.8		
			Safety Monitoring	19-SEP-2018	19:23	14	7	5.5		
			Safety Monitoring	22-SEP-2018	08:38	17	10	5.4		
			Safety Monitoring	25-SEP-2018	07:36	20	13	5.5		
			Safety Monitoring	28-SEP-2018	07:40	23	16	5.2		
			End of Study	02-OCT-2018	07:21	27	20	5.7		

Notes: Day relative to inoculum is the number of days relative to day of administration of the inoculum, where the day of administration = 0.
The inoculum was administered on Study Day 0 for K13-infected subjects and Study Day 1 for 3D7-infected subjects.
Day relative to IP is the number of days relative to day of administration of Artesunate, where the day of dose = 1.
Rando = Randomisation; IP = Artesunate; H = High; L = Low; NCS = Not Clinically Significant; CS = Clinically Significant.

Listing 16.2.8.1a
Individual Haematology Results

Rando Number	Challenge Agent	Parameter (unit) [Normal Range]	Visit	Date of Assessment	Time of Assessment	Day Relative to Inoculum	Day Relative to IP	Result	Range Indicator	Clinical Assessment
R307	3D7	Lymphocytes (10^9/L) [1 – 4]	Screening	07-AUG-2018	12:31	-29	-37	2.33		
			Safety Visit	03-SEP-2018	07:53	-2	-10	3.48		
			Admission	13-SEP-2018	09:51	8	1	1.14		
			Admission 3	16-SEP-2018	09:55	11	4	1.37		
			Safety Monitoring	18-SEP-2018	08:08	13	6	2.89		
			Safety Monitoring	19-SEP-2018	19:23	14	7	2.40		
			Safety Monitoring	22-SEP-2018	08:38	17	10	2.20		
			Safety Monitoring	25-SEP-2018	07:36	20	13	2.07		
			Safety Monitoring	28-SEP-2018	07:40	23	16	2.02		
			End of Study	02-OCT-2018	07:21	27	20	2.48		

Notes: Day relative to inoculum is the number of days relative to day of administration of the inoculum, where the day of administration = 0.
The inoculum was administered on Study Day 0 for K13-infected subjects and Study Day 1 for 3D7-infected subjects.
Day relative to IP is the number of days relative to day of administration of Artesunate, where the day of dose = 1.
Rando = Randomisation; IP = Artesunate; H = High; L = Low; NCS = Not Clinically Significant; CS = Clinically Significant.

Listing 16.2.8.1a
Individual Haematology Results

Rando Number	Challenge Agent	Parameter (unit) [Normal Range]	Visit	Date of Assessment	Time of Assessment	Day Relative to Inoculum	Day Relative to IP	Result	Range Indicator	Clinical Assessment
R307	3D7	Monocytes (10^9/L) [0 – 0.9]	Screening	07-AUG-2018	12:31	-29	-37	0.49		
			Safety Visit	03-SEP-2018	07:53	-2	-10	0.81		
			Admission	13-SEP-2018	09:51	8	1	0.55		
			Admission 3	16-SEP-2018	09:55	11	4	0.40		
			Safety Monitoring	18-SEP-2018	08:08	13	6	0.68		
			Safety Monitoring	19-SEP-2018	19:23	14	7	0.73		
			Safety Monitoring	22-SEP-2018	08:38	17	10	0.64		
			Safety Monitoring	25-SEP-2018	07:36	20	13	0.50		
			Safety Monitoring	28-SEP-2018	07:40	23	16	0.70		
			End of Study	02-OCT-2018	07:21	27	20	0.53		

Notes: Day relative to inoculum is the number of days relative to day of administration of the inoculum, where the day of administration = 0.
The inoculum was administered on Study Day 0 for K13-infected subjects and Study Day 1 for 3D7-infected subjects.
Day relative to IP is the number of days relative to day of administration of Artesunate, where the day of dose = 1.
Rando = Randomisation; IP = Artesunate; H = High; L = Low; NCS = Not Clinically Significant; CS = Clinically Significant.

Listing 16.2.8.1a
Individual Haematology Results

Rando Number	Challenge Agent	Parameter (unit) [Normal Range]	Visit	Date of Assessment	Time of Assessment	Day Relative to Inoculum	Day Relative to IP	Result	Range Indicator	Clinical Assessment
R307	3D7	Neutrophils (10^9/L) [1.5 – 8]	Screening	07-AUG-2018	12:31	-29	-37	3.50		
			Safety Visit	03-SEP-2018	07:53	-2	-10	3.80		
			Admission	13-SEP-2018	09:51	8	1	2.95		
			Admission 3	16-SEP-2018	09:55	11	4	1.34	L	CS
			Safety Monitoring	18-SEP-2018	08:08	13	6	2.05		
			Safety Monitoring	19-SEP-2018	19:23	14	7	2.20		
			Safety Monitoring	22-SEP-2018	08:38	17	10	2.36		
			Safety Monitoring	25-SEP-2018	07:36	20	13	2.85		
			Safety Monitoring	28-SEP-2018	07:40	23	16	2.31		
			End of Study	02-OCT-2018	07:21	27	20	2.46		

Notes: Day relative to inoculum is the number of days relative to day of administration of the inoculum, where the day of administration = 0.
The inoculum was administered on Study Day 0 for K13-infected subjects and Study Day 1 for 3D7-infected subjects.
Day relative to IP is the number of days relative to day of administration of Artesunate, where the day of dose = 1.
Rando = Randomisation; IP = Artesunate; H = High; L = Low; NCS = Not Clinically Significant; CS = Clinically Significant.

Listing 16.2.8.1a
Individual Haematology Results

Rando Number	Challenge Agent	Parameter (unit) [Normal Range]	Visit	Date of Assessment	Time of Assessment	Day Relative to Inoculum	Day Relative to IP	Result	Range Indicator	Clinical Assessment
R307	3D7	Platelets (10^9/L) [150 – 400]	Screening	07-AUG-2018	12:31	-29	-37	272		
			Safety Visit	03-SEP-2018	07:53	-2	-10	328		
			Admission	13-SEP-2018	09:51	8	1	219		
			Admission 3	16-SEP-2018	09:55	11	4	168		
			Safety Monitoring	18-SEP-2018	08:08	13	6	212		
			Safety Monitoring	19-SEP-2018	19:23	14	7	254		
			Safety Monitoring	22-SEP-2018	08:38	17	10	337		
			Safety Monitoring	25-SEP-2018	07:36	20	13	419	H	NCS
			Safety Monitoring	28-SEP-2018	07:40	23	16	371		
			End of Study	02-OCT-2018	07:21	27	20	334		
		Reticulocytes (10^9/L) [25 – 120]	Safety Visit	03-SEP-2018	07:53	-2	-10	44		
			End of Study	02-OCT-2018	07:21	27	20	65		

Notes: Day relative to inoculum is the number of days relative to day of administration of the inoculum, where the day of administration = 0.
The inoculum was administered on Study Day 0 for K13-infected subjects and Study Day 1 for 3D7-infected subjects.
Day relative to IP is the number of days relative to day of administration of Artesunate, where the day of dose = 1.
Rando = Randomisation; IP = Artesunate; H = High; L = Low; NCS = Not Clinically Significant; CS = Clinically Significant.

16.2.8.1b Individual Abnormal Haematology Results

Listing 16.2.8.1b
Individual Abnormal Haematology Results

Rando Number	Challenge Agent	Parameter (unit) [Normal Range]	Visit	Date of Assessment	Time of Assessment	Day Relative to Inoculum	Day Relative to IP	Result	Range Indicator	Clinical Assessment
R103	K13	Hematocrit [0.4 – 0.54]	Safety Monitoring	30-NOV-2017	10:09	16	8	0.39	L	NCS
			Safety Monitoring	04-DEC-2017	09:27	20	12	0.39	L	NCS
			End of Study	14-DEC-2017	09:37	30	22	0.39	L	NCS
		Hemoglobin (g/L) [135 – 175]	Safety Monitoring	30-NOV-2017	10:09	16	8	127	L	NCS
			Safety Monitoring	04-DEC-2017	09:27	20	12	129	L	NCS
			End of Study	14-DEC-2017	09:37	30	22	133	L	NCS
		Leukocytes (10^9/L) [3.5 – 10]	Admission 3	26-NOV-2017	10:53	12	4	3.4	L	NCS
		Lymphocytes (10^9/L) [1 – 4]	Safety Monitoring	25-NOV-2017	17:52	11	3	0.80	L	NCS
			Admission 3	26-NOV-2017	10:53	12	4	0.49	L	CS
		Platelets (10^9/L) [150 – 400]	Safety Monitoring	25-NOV-2017	17:52	11	3	136	L	NCS
			Admission 3	26-NOV-2017	10:53	12	4	114	L	NCS
			Safety Monitoring	30-NOV-2017	10:09	16	8	132	L	NCS

Notes: Day relative to inoculum is the number of days relative to day of administration of the inoculum, where the day of administration = 0.
The inoculum was administered on Study Day 0 for K13-infected subjects and Study Day 1 for 3D7-infected subjects.
Day relative to IP is the number of days relative to day of administration of Artesunate, where the day of dose = 1.
Rando = Randomisation; IP = Artesunate; H = High; L = Low; NCS = Not Clinically Significant; CS = Clinically Significant.

Listing 16.2.8.1b
Individual Abnormal Haematology Results

Rando Number	Challenge Agent	Parameter (unit) [Normal Range]	Visit	Date of Assessment	Time of Assessment	Day Relative to Inoculum	Day Relative to IP	Result	Range Indicator	Clinical Assessment
R104	K13	Erythrocytes (10^12/L) [4.5 – 6.5]	Safety Monitoring	04-DEC-2017	12:45	20	12	4.4	L	NCS
			Safety Monitoring	07-DEC-2017	08:35	23	15	4.3	L	NCS
			End of Study	14-DEC-2017	08:42	30	22	4.2	L	NCS
			Unscheduled	18-DEC-2017	08:14	34	26	4.1	L	NCS
			Unscheduled	21-DEC-2017	07:35	37	29	4.2	L	NCS
		Hematocrit [0.4 – 0.54]	Admission	23-NOV-2017	09:44	9	1	0.39	L	NCS
			Safety Monitoring	03-DEC-2017	09:13	19	11	0.39	L	NCS
			Safety Monitoring	04-DEC-2017	12:45	20	12	0.37	L	NCS
			Safety Monitoring	07-DEC-2017	08:35	23	15	0.37	L	NCS
			End of Study	14-DEC-2017	08:42	30	22	0.36	L	NCS
			Unscheduled	18-DEC-2017	08:14	34	26	0.35	L	NCS
			Unscheduled	21-DEC-2017	07:35	37	29	0.37	L	NCS
			Unscheduled	29-DEC-2017	10:15	45	37	0.39	L	NCS

Notes: Day relative to inoculum is the number of days relative to day of administration of the inoculum, where the day of administration = 0.
The inoculum was administered on Study Day 0 for K13-infected subjects and Study Day 1 for 3D7-infected subjects.
Day relative to IP is the number of days relative to day of administration of Artesunate, where the day of dose = 1.
Rando = Randomisation; IP = Artesunate; H = High; L = Low; NCS = Not Clinically Significant; CS = Clinically Significant.

Listing 16.2.8.1b
Individual Abnormal Haematology Results

Rando Number	Challenge Agent	Parameter (unit) [Normal Range]	Visit	Date of Assessment	Time of Assessment	Day Relative to Inoculum	Day Relative to IP	Result	Range Indicator	Clinical Assessment
R104	K13	Hemoglobin (g/L) [135 – 175]	Safety Monitoring	03-DEC-2017	09:13	19	11	132	L	NCS
			Safety Monitoring	04-DEC-2017	12:45	20	12	127	L	NCS
			Safety Monitoring	07-DEC-2017	08:35	23	15	125	L	NCS
			End of Study	14-DEC-2017	08:42	30	22	123	L	NCS
			Unscheduled	18-DEC-2017	08:14	34	26	120	L	NCS
			Unscheduled	21-DEC-2017	07:35	37	29	123	L	NCS
			Unscheduled	29-DEC-2017	10:15	45	37	133	L	NCS
		Leukocytes (10^9/L) [3.5 – 10]	Safety Monitoring	25-NOV-2017	17:43	11	3	2.2	L	CS
			Admission 3	26-NOV-2017	10:41	12	4	2.2	L	CS
		Lymphocytes (10^9/L) [1 – 4]	Admission	23-NOV-2017	09:44	9	1	0.81	L	NCS
			Safety Monitoring	25-NOV-2017	17:43	11	3	0.84	L	NCS
			Admission 3	26-NOV-2017	10:41	12	4	0.77	L	CS
		Neutrophils (10^9/L) [1.5 – 6.5]	Safety Monitoring	25-NOV-2017	17:43	11	3	0.99	L	CS
			Admission 3	26-NOV-2017	10:41	12	4	1.05	L	CS
R105	K13	Erythrocytes (10^12/L) [4.5 – 6.5]	Safety Monitoring	30-NOV-2017	07:05	16	8	4.4	L	NCS

Notes: Day relative to inoculum is the number of days relative to day of administration of the inoculum, where the day of administration = 0.
The inoculum was administered on Study Day 0 for K13-infected subjects and Study Day 1 for 3D7-infected subjects.
Day relative to IP is the number of days relative to day of administration of Artesunate, where the day of dose = 1.
Rando = Randomisation; IP = Artesunate; H = High; L = Low; NCS = Not Clinically Significant; CS = Clinically Significant.

Listing 16.2.8.1b
Individual Abnormal Haematology Results

Rando Number	Challenge Agent	Parameter (unit) [Normal Range]	Visit	Date of Assessment	Time of Assessment	Day Relative to Inoculum	Day Relative to IP	Result	Range Indicator	Clinical Assessment
R105	K13	Hemoglobin (g/L) [135 – 175]	Safety Monitoring	30-NOV-2017	07:05	16	8	132	L	NCS
R106	3D7	Hematocrit [0.35 – 0.47]	Safety Monitoring	11-DEC-2017	08:04	26	19	0.33	L	NCS
			End of Study	14-DEC-2017	08:53	29	22	0.33	L	NCS
		Leukocytes (10^9/L) [3.5 – 12]	Admission 3	26-NOV-2017	10:20	11	4	3.1	L	NCS
		Lymphocytes (10^9/L) [1 – 4]	Safety Monitoring	01-DEC-2017	08:27	16	9	0.78	L	CS
R107	3D7	Platelets (10^9/L) [150 – 400]	Admission	23-NOV-2017	09:35	8	1	141	L	NCS
R108	3D7	Eosinophils (10^9/L) [0 – 0.6]	Safety Monitoring	30-NOV-2017	06:57	15	8	0.64	H	NCS
			End of Study	14-DEC-2017	08:37	29	22	1.07	H	CS
			Unscheduled	18-DEC-2017	07:48	33	26	1.02	H	CS
			Unscheduled	21-DEC-2017	07:53	36	29	0.72	H	NCS
			Unscheduled	08-JAN-2018	08:06	54	47	0.74	H	NCS
		Lymphocytes (10^9/L) [1 – 4]	Admission	23-NOV-2017	09:17	8	1	0.97	L	NCS

Notes: Day relative to inoculum is the number of days relative to day of administration of the inoculum, where the day of administration = 0.
The inoculum was administered on Study Day 0 for K13-infected subjects and Study Day 1 for 3D7-infected subjects.
Day relative to IP is the number of days relative to day of administration of Artesunate, where the day of dose = 1.
Rando = Randomisation; IP = Artesunate; H = High; L = Low; NCS = Not Clinically Significant; CS = Clinically Significant.

Listing 16.2.8.1b
Individual Abnormal Haematology Results

Rando Number	Challenge Agent	Parameter (unit) [Normal Range]	Visit	Date of Assessment	Time of Assessment	Day Relative to Inoculum	Day Relative to IP	Result	Range Indicator	Clinical Assessment
R108	3D7	Monocytes (10^9/L) [0 – 0.9]	Screening	26-OCT-2017	09:30	-20	-28	0.98	H	NCS
			End of Study	14-DEC-2017	08:37	29	22	0.96	H	NCS
			Unscheduled	21-DEC-2017	07:53	36	29	0.92	H	NCS
R201	K13	Erythrocytes (10^12/L) [3.9 – 5.6]	Safety Monitoring	05-MAR-2018	08:54	14	6	3.8	L	NCS
			End of Study	22-MAR-2018	08:31	31	23	3.8	L	NCS
		Hematocrit [0.35 – 0.47]	Safety Monitoring	05-MAR-2018	08:54	14	6	0.32	L	NCS
			Safety Monitoring	09-MAR-2018	09:59	18	10	0.34	L	NCS
			End of Study	22-MAR-2018	08:31	31	23	0.32	L	NCS
		Hemoglobin (g/L) [115 – 165]	Safety Monitoring	05-MAR-2018	08:54	14	6	109	L	NCS
			Safety Monitoring	19-MAR-2018	08:19	28	20	112	L	NCS
			End of Study	22-MAR-2018	08:31	31	23	113	L	NCS
		Leukocytes (10^9/L) [3.5 – 12]	Safety Monitoring	02-MAR-2018	13:24	11	3	2.4	L	CS
			Admission 3	03-MAR-2018	13:27	12	4	3.4	L	NCS
		Lymphocytes (10^9/L) [1 – 4]	Safety Monitoring	02-MAR-2018	13:24	11	3	0.95	L	NCS

Notes: Day relative to inoculum is the number of days relative to day of administration of the inoculum, where the day of administration = 0.
The inoculum was administered on Study Day 0 for K13-infected subjects and Study Day 1 for 3D7-infected subjects.
Day relative to IP is the number of days relative to day of administration of Artesunate, where the day of dose = 1.
Rando = Randomisation; IP = Artesunate; H = High; L = Low; NCS = Not Clinically Significant; CS = Clinically Significant.

Listing 16.2.8.1b
Individual Abnormal Haematology Results

Rando Number	Challenge Agent	Parameter (unit) [Normal Range]	Visit	Date of Assessment	Time of Assessment	Day Relative to Inoculum	Day Relative to IP	Result	Range Indicator	Clinical Assessment
R201	K13	Neutrophils (10^9/L) [1.5 – 8]	Safety Monitoring	02-MAR-2018	13:24	11	3	1.17	L	CS
			Admission 3	03-MAR-2018	13:27	12	4	1.38	L	CS
R202	K13	Erythrocytes (10^12/L) [4.5 – 6.5]	Safety Visit	16-FEB-2018	08:57	-3	-12	4.4	L	NCS
			Admission	28-FEB-2018	12:05	9	1	4.2	L	NCS
			Safety Monitoring	09-MAR-2018	09:32	18	10	4.3	L	NCS
			Safety Monitoring	12-MAR-2018	09:51	21	13	4.2	L	NCS
			Safety Monitoring	16-MAR-2018	09:35	25	17	4.2	L	NCS
			End of Study	22-MAR-2018	10:00	31	23	4.1	L	NCS
		Hematocrit [0.4 – 0.54]	Admission	28-FEB-2018	12:05	9	1	0.39	L	NCS
			Safety Monitoring	09-MAR-2018	09:32	18	10	0.39	L	NCS
			Safety Monitoring	12-MAR-2018	09:51	21	13	0.38	L	NCS
			Safety Monitoring	16-MAR-2018	09:35	25	17	0.37	L	NCS
			End of Study	22-MAR-2018	10:00	31	23	0.36	L	NCS
			Unscheduled	27-MAR-2018	09:00	36	28	0.37	L	NCS

Notes: Day relative to inoculum is the number of days relative to day of administration of the inoculum, where the day of administration = 0.
The inoculum was administered on Study Day 0 for K13-infected subjects and Study Day 1 for 3D7-infected subjects.
Day relative to IP is the number of days relative to day of administration of Artesunate, where the day of dose = 1.
Rando = Randomisation; IP = Artesunate; H = High; L = Low; NCS = Not Clinically Significant; CS = Clinically Significant.

Listing 16.2.8.1b
Individual Abnormal Haematology Results

Rando Number	Challenge Agent	Parameter (unit) [Normal Range]	Visit	Date of Assessment	Time of Assessment	Day Relative to Inoculum	Day Relative to IP	Result	Range Indicator	Clinical Assessment
R202	K13	Hemoglobin (g/L) [135 – 175]	Safety Visit	16-FEB-2018	08:57	-3	-12	134	L	NCS
			Admission	28-FEB-2018	12:05	9	1	130	L	NCS
			Safety Monitoring	09-MAR-2018	09:32	18	10	130	L	NCS
			Safety Monitoring	12-MAR-2018	09:51	21	13	130	L	NCS
			Safety Monitoring	16-MAR-2018	09:35	25	17	130	L	NCS
			End of Study	22-MAR-2018	10:00	31	23	127	L	NCS
			Unscheduled	27-MAR-2018	09:00	36	28	129	L	NCS
			Unscheduled	03-APR-2018	09:10	43	35	132	L	NCS
		Platelets (10^9/L) [150 – 400]	Safety Monitoring	05-MAR-2018	09:59	14	6	135	L	NCS
R203	K13	Eosinophils (10^9/L) [0 – 0.6]	End of Study	22-MAR-2018	10:14	31	23	0.61	H	NCS
			Unscheduled	02-APR-2018	09:18	42	34	0.69	H	CS

Notes: Day relative to inoculum is the number of days relative to day of administration of the inoculum, where the day of administration = 0.
The inoculum was administered on Study Day 0 for K13-infected subjects and Study Day 1 for 3D7-infected subjects.
Day relative to IP is the number of days relative to day of administration of Artesunate, where the day of dose = 1.
Rando = Randomisation; IP = Artesunate; H = High; L = Low; NCS = Not Clinically Significant; CS = Clinically Significant.

Listing 16.2.8.1b
Individual Abnormal Haematology Results

Rando Number	Challenge Agent	Parameter (unit) [Normal Range]	Visit	Date of Assessment	Time of Assessment	Day Relative to Inoculum	Day Relative to IP	Result	Range Indicator	Clinical Assessment
R203	K13	Ery. Mean Corpuscular Volume (fL) [80 – 100]	Admission 3	03-MAR-2018	11:50	12	4	78	L	NCS
			Safety Monitoring	05-MAR-2018	09:40	14	6	79	L	NCS
			Safety Monitoring	09-MAR-2018	10:14	18	10	79	L	NCS
			Safety Monitoring	10-MAR-2018	08:37	19	11	79	L	NCS
			Unscheduled	28-MAR-2018	09:53	37	29	78	L	NCS
			Unscheduled	14-APR-2018	10:00	54	46	79	L	NCS
		Hemoglobin (g/L) [115 – 165]	Unscheduled	28-MAR-2018	09:53	37	29	114	L	NCS
		Lymphocytes (10^9/L) [1 – 4]	Admission 3	03-MAR-2018	11:50	12	4	0.92	L	NCS
			Safety Monitoring	13-MAR-2018	09:41	22	14	4.05	H	NCS

Notes: Day relative to inoculum is the number of days relative to day of administration of the inoculum, where the day of administration = 0.
The inoculum was administered on Study Day 0 for K13-infected subjects and Study Day 1 for 3D7-infected subjects.
Day relative to IP is the number of days relative to day of administration of Artesunate, where the day of dose = 1.
Rando = Randomisation; IP = Artesunate; H = High; L = Low; NCS = Not Clinically Significant; CS = Clinically Significant.

Printed: 24FEB2020 (Final)

Listing 16.2.8.1b
Individual Abnormal Haematology Results

Rando Number	Challenge Agent	Parameter (unit) [Normal Range]	Visit	Date of Assessment	Time of Assessment	Day Relative to Inoculum	Day Relative to IP	Result	Range Indicator	Clinical Assessment
R203	K13	Lymphocytes Atypical (10^9/L) [0 – 0.01]	Safety Monitoring	05-MAR-2018	09:40	14	6	0.18	H	NCS
			Safety Monitoring	09-MAR-2018	10:14	18	10	0.29	H	NCS
		Neutrophils (10^9/L) [1.5 – 8]	Safety Monitoring	05-MAR-2018	09:40	14	6	1.17	L	CS
		Platelets (10^9/L) [150 – 400]	Admission 3	03-MAR-2018	11:50	12	4	140	L	NCS
			Safety Monitoring	05-MAR-2018	09:40	14	6	127	L	NCS
R204	K13	Erythrocytes (10^12/L) [3.9 – 5.6]	Safety Monitoring	05-MAR-2018	08:37	14	6	3.8	L	NCS
			Safety Monitoring	09-MAR-2018	09:00	18	10	3.7	L	NCS
			End of Study	22-MAR-2018	08:43	31	23	3.7	L	NCS
		Hematocrit [0.35 – 0.47]	End of Study	22-MAR-2018	08:43	31	23	0.34	L	NCS
		Leukocytes (10^9/L) [3.5 – 12]	Admission 3	03-MAR-2018	13:21	12	4	2.9	L	CS
			Safety Monitoring	05-MAR-2018	08:37	14	6	2.5	L	CS

Notes: Day relative to inoculum is the number of days relative to day of administration of the inoculum, where the day of administration = 0.
The inoculum was administered on Study Day 0 for K13-infected subjects and Study Day 1 for 3D7-infected subjects.
Day relative to IP is the number of days relative to day of administration of Artesunate, where the day of dose = 1.
Rando = Randomisation; IP = Artesunate; H = High; L = Low; NCS = Not Clinically Significant; CS = Clinically Significant.

Listing 16.2.8.1b
Individual Abnormal Haematology Results

Rando Number	Challenge Agent	Parameter (unit) [Normal Range]	Visit	Date of Assessment	Time of Assessment	Day Relative to Inoculum	Day Relative to IP	Result	Range Indicator	Clinical Assessment
R204	K13	Lymphocytes (10^9/L) [1 – 4]	Admission 3	03-MAR-2018	13:21	12	4	0.64	L	CS
			Safety Monitoring	05-MAR-2018	08:37	14	6	0.66	L	CS
		Neutrophils (10^9/L) [1.5 – 8]	Safety Monitoring	05-MAR-2018	08:37	14	6	1.32	L	CS
		Platelets (10^9/L) [150 – 400]	Safety Monitoring	05-MAR-2018	08:37	14	6	122	L	NCS
R205	K13	Erythrocytes (10^12/L) [3.9 – 5.6]	Safety Visit	16-FEB-2018	07:45	-3	-12	3.6	L	NCS
			Admission	28-FEB-2018	12:19	9	1	3.5	L	NCS
			Safety Monitoring	02-MAR-2018	13:11	11	3	3.8	L	NCS
			Safety Monitoring	19-MAR-2018	07:29	28	20	3.7	L	NCS
			End of Study	22-MAR-2018	07:30	31	23	3.6	L	NCS
			Unscheduled	26-MAR-2018	07:26	35	27	3.8	L	NCS

Notes: Day relative to inoculum is the number of days relative to day of administration of the inoculum, where the day of administration = 0.
The inoculum was administered on Study Day 0 for K13-infected subjects and Study Day 1 for 3D7-infected subjects.
Day relative to IP is the number of days relative to day of administration of Artesunate, where the day of dose = 1.
Rando = Randomisation; IP = Artesunate; H = High; L = Low; NCS = Not Clinically Significant; CS = Clinically Significant.

Listing 16.2.8.1b
Individual Abnormal Haematology Results

Rando Number	Challenge Agent	Parameter (unit) [Normal Range]	Visit	Date of Assessment	Time of Assessment	Day Relative to Inoculum	Day Relative to IP	Result	Range Indicator	Clinical Assessment
R205	K13	Hematocrit [0.35 – 0.47]	Safety Visit	16-FEB-2018	07:45	-3	-12	0.33	L	NCS
			Admission	28-FEB-2018	12:19	9	1	0.32	L	NCS
			Safety Monitoring	02-MAR-2018	13:11	11	3	0.34	L	NCS
			Safety Monitoring	19-MAR-2018	07:29	28	20	0.34	L	NCS
			End of Study	22-MAR-2018	07:30	31	23	0.32	L	NCS
			Unscheduled	26-MAR-2018	07:26	35	27	0.34	L	NCS
		Hemoglobin (g/L) [115 – 165]	Safety Visit	16-FEB-2018	07:45	-3	-12	109	L	NCS
			Admission	28-FEB-2018	12:19	9	1	108	L	NCS
			Safety Monitoring	02-MAR-2018	13:11	11	3	114	L	NCS
			Safety Monitoring	19-MAR-2018	07:29	28	20	111	L	NCS
			End of Study	22-MAR-2018	07:30	31	23	111	L	NCS
			Unscheduled	26-MAR-2018	07:26	35	27	113	L	NCS
		Leukocytes (10^9/L) [3.5 – 12]	Safety Monitoring	02-MAR-2018	13:11	11	3	2.1	L	CS
			Admission 3	03-MAR-2018	13:15	12	4	2.3	L	CS
			Safety Monitoring	05-MAR-2018	09:13	14	6	2.9	L	CS

Notes: Day relative to inoculum is the number of days relative to day of administration of the inoculum, where the day of administration = 0.
The inoculum was administered on Study Day 0 for K13-infected subjects and Study Day 1 for 3D7-infected subjects.
Day relative to IP is the number of days relative to day of administration of Artesunate, where the day of dose = 1.
Rando = Randomisation; IP = Artesunate; H = High; L = Low; NCS = Not Clinically Significant; CS = Clinically Significant.

Printed: 24FEB2020 (Final)

Listing 16.2.8.1b
Individual Abnormal Haematology Results

Rando Number	Challenge Agent	Parameter (unit) [Normal Range]	Visit	Date of Assessment	Time of Assessment	Day Relative to Inoculum	Day Relative to IP	Result	Range Indicator	Clinical Assessment
R205	K13	Lymphocytes (10^9/L) [1 – 4]	Safety Monitoring	02-MAR-2018	13:11	11	3	0.81	L	NCS
			Admission 3	03-MAR-2018	13:15	12	4	0.82	L	NCS
		Lymphocytes Atypical (10^9/L) [0 – 0.01]	Safety Monitoring	05-MAR-2018	09:13	14	6	0.12	H	NCS
		Neutrophils (10^9/L) [1.5 – 8]	Safety Monitoring	02-MAR-2018	13:11	11	3	1.08	L	CS
			Admission 3	03-MAR-2018	13:15	12	4	1.26	L	CS
			Safety Monitoring	05-MAR-2018	09:13	14	6	1.22	L	CS
		Platelets (10^9/L) [150 – 400]	Safety Monitoring	05-MAR-2018	09:13	14	6	140	L	NCS
R206	K13	Erythrocytes (10^12/L) [3.9 – 5.6]	End of Study	22-MAR-2018	09:19	31	23	3.4	L	NCS
		Hematocrit [0.35 – 0.47]	End of Study	22-MAR-2018	09:19	31	23	0.31	L	NCS
		Hemoglobin (g/L) [115 – 165]	End of Study	22-MAR-2018	09:19	31	23	110	L	NCS

Notes: Day relative to inoculum is the number of days relative to day of administration of the inoculum, where the day of administration = 0.
The inoculum was administered on Study Day 0 for K13-infected subjects and Study Day 1 for 3D7-infected subjects.
Day relative to IP is the number of days relative to day of administration of Artesunate, where the day of dose = 1.
Rando = Randomisation; IP = Artesunate; H = High; L = Low; NCS = Not Clinically Significant; CS = Clinically Significant.

Listing 16.2.8.1b
Individual Abnormal Haematology Results

Rando Number	Challenge Agent	Parameter (unit) [Normal Range]	Visit	Date of Assessment	Time of Assessment	Day Relative to Inoculum	Day Relative to IP	Result	Range Indicator	Clinical Assessment
R206	K13	Leukocytes (10^9/L) [3.5 – 12]	Safety Monitoring	02-MAR-2018	13:29	11	3	1.8	L	CS
			Admission 3	03-MAR-2018	13:39	12	4	2.6	L	CS
		Lymphocytes (10^9/L) [1 – 4]	Admission	28-FEB-2018	12:48	9	1	0.73	L	CS
			Safety Monitoring	02-MAR-2018	13:29	11	3	0.57	L	CS
			Admission 3	03-MAR-2018	13:39	12	4	0.78	L	CS
		Neutrophils (10^9/L) [1.5 – 8]	Safety Monitoring	02-MAR-2018	13:29	11	3	0.95	L	CS
			Admission 3	03-MAR-2018	13:39	12	4	1.38	L	CS
R207	K13	Lymphocytes (10^9/L) [1 – 4]	Safety Monitoring	02-MAR-2018	12:59	11	3	0.68	L	CS
			Admission 3	03-MAR-2018	13:00	12	4	0.97	L	NCS
		Metamyelocytes (10^9/L) [0 – 0.01]	Safety Monitoring	09-MAR-2018	09:33	18	10	0.20	H	NCS
			End of Study	22-MAR-2018	09:32	31	23	0.32	H	NCS
		Myelocytes (10^9/L) [0 – 0.01]	Safety Monitoring	09-MAR-2018	09:33	18	10	0.07	H	NCS

Notes: Day relative to inoculum is the number of days relative to day of administration of the inoculum, where the day of administration = 0.
The inoculum was administered on Study Day 0 for K13-infected subjects and Study Day 1 for 3D7-infected subjects.
Day relative to IP is the number of days relative to day of administration of Artesunate, where the day of dose = 1.
Rando = Randomisation; IP = Artesunate; H = High; L = Low; NCS = Not Clinically Significant; CS = Clinically Significant.

Listing 16.2.8.1b
Individual Abnormal Haematology Results

Rando Number	Challenge Agent	Parameter (unit) [Normal Range]	Visit	Date of Assessment	Time of Assessment	Day Relative to Inoculum	Day Relative to IP	Result	Range Indicator	Clinical Assessment
R208	3D7	Erythrocytes (10^12/L) [3.9 – 5.6]	Admission 3	03-MAR-2018	11:36	11	4	3.8	L	NCS
			Safety Monitoring	13-MAR-2018	07:22	21	14	3.8	L	NCS
		Hematocrit [0.35 – 0.47]	Admission 3	03-MAR-2018	11:36	11	4	0.33	L	NCS
			Safety Monitoring	13-MAR-2018	07:22	21	14	0.34	L	NCS
		Metamyelocytes (10^9/L) [0 – 0.01]	End of Study	22-MAR-2018	07:50	30	23	0.22	H	NCS
		Myelocytes (10^9/L) [0 – 0.01]	End of Study	22-MAR-2018	07:50	30	23	0.15	H	NCS
		Platelets (10^9/L) [150 – 400]	Admission 3	03-MAR-2018	11:36	11	4	146	L	NCS
		Reticulocytes (10^9/L) [25 – 120]	End of Study	22-MAR-2018	07:50	30	23	126	H	NCS
R209	3D7	Erythrocytes (10^12/L) [4.5 – 6.5]	Safety Monitoring	05-MAR-2018	07:41	13	6	4.4	L	NCS
		Hematocrit [0.4 – 0.54]	Safety Monitoring	05-MAR-2018	07:41	13	6	0.38	L	NCS
			Safety Monitoring	12-MAR-2018	08:12	20	13	0.39	L	NCS
			End of Study	22-MAR-2018	08:16	30	23	0.39	L	NCS

Notes: Day relative to inoculum is the number of days relative to day of administration of the inoculum, where the day of administration = 0.
The inoculum was administered on Study Day 0 for K13-infected subjects and Study Day 1 for 3D7-infected subjects.
Day relative to IP is the number of days relative to day of administration of Artesunate, where the day of dose = 1.
Rando = Randomisation; IP = Artesunate; H = High; L = Low; NCS = Not Clinically Significant; CS = Clinically Significant.

Listing 16.2.8.1b
Individual Abnormal Haematology Results

Rando Number	Challenge Agent	Parameter (unit) [Normal Range]	Visit	Date of Assessment	Time of Assessment	Day Relative to Inoculum	Day Relative to IP	Result	Range Indicator	Clinical Assessment
R209	3D7	Hemoglobin (g/L) [135 – 175]	Safety Monitoring	05-MAR-2018	07:41	13	6	127	L	NCS
			Safety Monitoring	08-MAR-2018	08:42	16	9	134	L	NCS
			Safety Monitoring	12-MAR-2018	08:12	20	13	128	L	NCS
			Safety Monitoring	19-MAR-2018	07:48	27	20	134	L	NCS
		Platelets (10^9/L) [150 – 400]	Safety Monitoring	12-MAR-2018	08:12	20	13	145	L	NCS
R210	3D7	Erythrocytes (10^12/L) [3.9 – 5.6]	Safety Monitoring	19-MAR-2018	07:39	27	20	3.8	L	NCS
		Hematocrit [0.35 – 0.47]	Safety Visit	16-FEB-2018	07:28	-4	-12	0.34	L	NCS
			Safety Monitoring	19-MAR-2018	07:39	27	20	0.34	L	NCS
		Hemoglobin (g/L) [115 – 165]	Safety Visit	16-FEB-2018	07:28	-4	-12	113	L	NCS
			Admission	28-FEB-2018	11:21	8	1	114	L	NCS
			Safety Monitoring	08-MAR-2018	07:40	16	9	114	L	NCS
			Safety Monitoring	12-MAR-2018	07:37	20	13	113	L	NCS
			Safety Monitoring	19-MAR-2018	07:39	27	20	110	L	NCS

Notes: Day relative to inoculum is the number of days relative to day of administration of the inoculum, where the day of administration = 0.
The inoculum was administered on Study Day 0 for K13-infected subjects and Study Day 1 for 3D7-infected subjects.
Day relative to IP is the number of days relative to day of administration of Artesunate, where the day of dose = 1.
Rando = Randomisation; IP = Artesunate; H = High; L = Low; NCS = Not Clinically Significant; CS = Clinically Significant.

Listing 16.2.8.1b
Individual Abnormal Haematology Results

Rando Number	Challenge Agent	Parameter (unit) [Normal Range]	Visit	Date of Assessment	Time of Assessment	Day Relative to Inoculum	Day Relative to IP	Result	Range Indicator	Clinical Assessment
R302	K13	Erythrocytes (10^12/L) [4.5 – 6.5]	Safety Monitoring	22-SEP-2018	07:56	18	10	4.4	L	NCS
			Safety Monitoring	28-SEP-2018	08:22	24	16	4.4	L	NCS
		Hematocrit [0.4 – 0.54]	Safety Monitoring	18-SEP-2018	08:04	14	6	0.36	L	NCS
			Safety Monitoring	22-SEP-2018	07:56	18	10	0.36	L	NCS
			Safety Monitoring	25-SEP-2018	08:13	21	13	0.39	L	NCS
			Safety Monitoring	28-SEP-2018	08:22	24	16	0.37	L	NCS
		Hemoglobin (g/L) [135 – 175]	Safety Monitoring	18-SEP-2018	08:04	14	6	131	L	NCS
			Safety Monitoring	22-SEP-2018	07:56	18	10	128	L	NCS
			Safety Monitoring	28-SEP-2018	08:22	24	16	131	L	NCS
		Lymphocytes (10^9/L) [1 – 4]	Safety Monitoring	15-SEP-2018	09:06	11	3	0.95	L	NCS
			Admission 3	16-SEP-2018	09:05	12	4	0.78	L	CS

Notes: Day relative to inoculum is the number of days relative to day of administration of the inoculum, where the day of administration = 0.
The inoculum was administered on Study Day 0 for K13-infected subjects and Study Day 1 for 3D7-infected subjects.
Day relative to IP is the number of days relative to day of administration of Artesunate, where the day of dose = 1.
Rando = Randomisation; IP = Artesunate; H = High; L = Low; NCS = Not Clinically Significant; CS = Clinically Significant.

Listing 16.2.8.1b
Individual Abnormal Haematology Results

Rando Number	Challenge Agent	Parameter (unit) [Normal Range]	Visit	Date of Assessment	Time of Assessment	Day Relative to Inoculum	Day Relative to IP	Result	Range Indicator	Clinical Assessment
R303	K13	Ery. Mean Corpuscular Volume (fL) [80 – 100]	Safety Monitoring	15-SEP-2018	11:18	11	3	78	L	NCS
			Admission 3	16-SEP-2018	11:18	12	4	79	L	NCS
			Safety Monitoring	22-SEP-2018	08:40	18	10	79	L	NCS
		Hematocrit [0.35 – 0.47]	Safety Monitoring	22-SEP-2018	08:40	18	10	0.33	L	NCS
			Safety Monitoring	25-SEP-2018	08:20	21	13	0.34	L	NCS
			Safety Monitoring	28-SEP-2018	08:19	24	16	0.32	L	NCS
		Hemoglobin (g/L) [115 – 165]	Safety Monitoring	22-SEP-2018	08:40	18	10	114	L	NCS
			Safety Monitoring	25-SEP-2018	08:20	21	13	114	L	NCS
			Safety Monitoring	28-SEP-2018	08:19	24	16	110	L	NCS
		Leukocytes (10^9/L) [3.5 – 12]	Safety Monitoring	15-SEP-2018	11:18	11	3	2.5	L	CS
			Admission 3	16-SEP-2018	11:18	12	4	2.9	L	CS

Notes: Day relative to inoculum is the number of days relative to day of administration of the inoculum, where the day of administration = 0.
The inoculum was administered on Study Day 0 for K13-infected subjects and Study Day 1 for 3D7-infected subjects.
Day relative to IP is the number of days relative to day of administration of Artesunate, where the day of dose = 1.
Rando = Randomisation; IP = Artesunate; H = High; L = Low; NCS = Not Clinically Significant; CS = Clinically Significant.

Listing 16.2.8.1b
Individual Abnormal Haematology Results

Rando Number	Challenge Agent	Parameter (unit) [Normal Range]	Visit	Date of Assessment	Time of Assessment	Day Relative to Inoculum	Day Relative to IP	Result	Range Indicator	Clinical Assessment
R303	K13	Neutrophils (10^9/L) [1.5 – 8]	Safety Monitoring	15-SEP-2018	11:18	11	3	1.11	L	CS
			Admission 3	16-SEP-2018	11:18	12	4	1.01	L	CS
			Safety Monitoring	18-SEP-2018	07:29	14	6	1.12	L	CS
		Platelets (10^9/L) [150 – 400]	Safety Monitoring	25-SEP-2018	08:20	21	13	412	H	NCS
R304	K13	Monocytes (10^9/L) [0 – 0.9]	Admission	13-SEP-2018	09:16	9	1	0.94	H	NCS
		Platelets (10^9/L) [150 – 400]	Safety Monitoring	15-SEP-2018	09:22	11	3	119	L	NCS
			Admission 3	16-SEP-2018	09:22	12	4	114	L	NCS
			Safety Monitoring	18-SEP-2018	08:36	14	6	126	L	NCS
		Reticulocytes (10^9/L) [25 – 120]	End of Study	02-OCT-2018	08:46	28	20	124	H	NCS
R305	3D7	Hematocrit [0.4 – 0.54]	Safety Monitoring	18-SEP-2018	07:16	13	6	0.39	L	NCS
			Safety Monitoring	19-SEP-2018	19:23	14	7	0.38	L	NCS
			Safety Monitoring	28-SEP-2018	07:23	23	16	0.39	L	NCS

Notes: Day relative to inoculum is the number of days relative to day of administration of the inoculum, where the day of administration = 0.
The inoculum was administered on Study Day 0 for K13-infected subjects and Study Day 1 for 3D7-infected subjects.
Day relative to IP is the number of days relative to day of administration of Artesunate, where the day of dose = 1.
Rando = Randomisation; IP = Artesunate; H = High; L = Low; NCS = Not Clinically Significant; CS = Clinically Significant.

Listing 16.2.8.1b
Individual Abnormal Haematology Results

Rando Number	Challenge Agent	Parameter (unit) [Normal Range]	Visit	Date of Assessment	Time of Assessment	Day Relative to Inoculum	Day Relative to IP	Result	Range Indicator	Clinical Assessment
R305	3D7	Leukocytes (10^9/L) [3.5 – 10]	Admission	13-SEP-2018	09:29	8	1	3.3	L	NCS
			Admission 3	16-SEP-2018	09:36	11	4	2.4	L	CS
			Safety Monitoring	18-SEP-2018	07:16	13	6	3.2	L	NCS
			Safety Monitoring	22-SEP-2018	07:53	17	10	3.1	L	NCS
			Safety Monitoring	28-SEP-2018	07:23	23	16	3.4	L	NCS
		Lymphocytes (10^9/L) [1 – 4]	Admission	13-SEP-2018	09:29	8	1	0.92	L	NCS
			Admission 3	16-SEP-2018	09:36	11	4	0.99	L	NCS
		Neutrophils (10^9/L) [1.5 – 6.5]	Admission 3	16-SEP-2018	09:36	11	4	0.89	L	CS
			Safety Monitoring	18-SEP-2018	07:16	13	6	1.08	L	CS
			Safety Monitoring	22-SEP-2018	07:53	17	10	1.11	L	CS
			Safety Monitoring	28-SEP-2018	07:23	23	16	1.32	L	CS
		Platelets (10^9/L) [150 – 400]	Admission 3	16-SEP-2018	09:36	11	4	141	L	NCS
R306	3D7	Erythrocytes (10^12/L) [4.5 – 6.5]	Safety Monitoring	19-SEP-2018	20:08	14	7	4.4	L	NCS

Notes: Day relative to inoculum is the number of days relative to day of administration of the inoculum, where the day of administration = 0.
The inoculum was administered on Study Day 0 for K13-infected subjects and Study Day 1 for 3D7-infected subjects.
Day relative to IP is the number of days relative to day of administration of Artesunate, where the day of dose = 1.
Rando = Randomisation; IP = Artesunate; H = High; L = Low; NCS = Not Clinically Significant; CS = Clinically Significant.

Listing 16.2.8.1b
Individual Abnormal Haematology Results

Rando Number	Challenge Agent	Parameter (unit) [Normal Range]	Visit	Date of Assessment	Time of Assessment	Day Relative to Inoculum	Day Relative to IP	Result	Range Indicator	Clinical Assessment
R306	3D7	Hematocrit [0.4 – 0.54]	Safety Monitoring	19-SEP-2018	20:08	14	7	0.38	L	NCS
			Safety Monitoring	22-SEP-2018	07:34	17	10	0.38	L	NCS
			Safety Monitoring	25-SEP-2018	07:30	20	13	0.39	L	NCS
			End of Study	02-OCT-2018	08:20	27	20	0.39	L	NCS
R307	3D7	Erythrocytes (10^12/L) [3.9 – 5.6]	Screening	07-AUG-2018	12:31	-29	-37	3.8	L	NCS
			Safety Monitoring	18-SEP-2018	08:08	13	6	3.7	L	NCS
			Safety Monitoring	19-SEP-2018	19:23	14	7	3.5	L	NCS
			Safety Monitoring	22-SEP-2018	08:38	17	10	3.6	L	NCS
			Safety Monitoring	25-SEP-2018	07:36	20	13	3.8	L	NCS
			Safety Monitoring	28-SEP-2018	07:40	23	16	3.6	L	NCS
			End of Study	02-OCT-2018	07:21	27	20	3.5	L	NCS
		Hematocrit [0.35 – 0.47]	Safety Monitoring	18-SEP-2018	08:08	13	6	0.33	L	NCS
			Safety Monitoring	19-SEP-2018	19:23	14	7	0.31	L	NCS
			Safety Monitoring	22-SEP-2018	08:38	17	10	0.32	L	NCS
			Safety Monitoring	28-SEP-2018	07:40	23	16	0.32	L	NCS
			End of Study	02-OCT-2018	07:21	27	20	0.31	L	NCS

Notes: Day relative to inoculum is the number of days relative to day of administration of the inoculum, where the day of administration = 0.
The inoculum was administered on Study Day 0 for K13-infected subjects and Study Day 1 for 3D7-infected subjects.
Day relative to IP is the number of days relative to day of administration of Artesunate, where the day of dose = 1.
Rando = Randomisation; IP = Artesunate; H = High; L = Low; NCS = Not Clinically Significant; CS = Clinically Significant.

Listing 16.2.8.1b
Individual Abnormal Haematology Results

Rando Number	Challenge Agent	Parameter (unit) [Normal Range]	Visit	Date of Assessment	Time of Assessment	Day Relative to Inoculum	Day Relative to IP	Result	Range Indicator	Clinical Assessment
R307	3D7	Hemoglobin (g/L) [115 – 165]	Safety Monitoring	18-SEP-2018	08:08	13	6	114	L	NCS
			Safety Monitoring	19-SEP-2018	19:23	14	7	109	L	NCS
			Safety Monitoring	22-SEP-2018	08:38	17	10	112	L	NCS
			Safety Monitoring	28-SEP-2018	07:40	23	16	108	L	NCS
			End of Study	02-OCT-2018	07:21	27	20	106	L	NCS
		Leukocytes (10^9/L) [3.5 – 12]	Admission 3	16-SEP-2018	09:55	11	4	3.2	L	NCS
		Neutrophils (10^9/L) [1.5 – 8]	Admission 3	16-SEP-2018	09:55	11	4	1.34	L	CS
		Platelets (10^9/L) [150 – 400]	Safety Monitoring	25-SEP-2018	07:36	20	13	419	H	NCS

Notes: Day relative to inoculum is the number of days relative to day of administration of the inoculum, where the day of administration = 0.
The inoculum was administered on Study Day 0 for K13-infected subjects and Study Day 1 for 3D7-infected subjects.
Day relative to IP is the number of days relative to day of administration of Artesunate, where the day of dose = 1.
Rando = Randomisation; IP = Artesunate; H = High; L = Low; NCS = Not Clinically Significant; CS = Clinically Significant.

16.2.8.2a Individual Biochemistry Results

Listing 16.2.8.2a
Individual Biochemistry Results

Rando Number	Challenge Agent	Parameter (unit) [Normal Range]	Visit	Date of Assessment	Time of Assessment	Day Relative to Inoculum	Day Relative to IP	Result	Range Indicator	Clinical Assessment
R103	K13	Alanine Aminotransferase (U/L) [5 – 40]	Screening	01-NOV-2017	12:16	-13	-22	11		
			Safety Visit	13-NOV-2017	08:44	-1	-10	12		
			Admission	23-NOV-2017	09:50	9	1	10		
			Safety Monitoring	25-NOV-2017	17:52	11	3	17		
			Admission 3	26-NOV-2017	10:53	12	4	15		
			Safety Monitoring	30-NOV-2017	10:09	16	8	37		
			Safety Monitoring	03-DEC-2017	10:45	19	11	33		
			Safety Monitoring	04-DEC-2017	09:27	20	12	31		
			Safety Monitoring	07-DEC-2017	08:41	23	15	20		
			End of Study	14-DEC-2017	09:37	30	22	15		
			Unscheduled	18-DEC-2017	07:20	34	26	14		

Notes: Day relative to inoculum is the number of days relative to day of administration of the inoculum, where the day of administration = 0.
The inoculum was administered on Study Day 0 for K13-infected subjects and Study Day 1 for 3D7-infected subjects.
Day relative to IP is the number of days relative to day of administration of Artesunate, where the day of dose = 1.
Rando = Randomisation; IP = Artesunate; H = High; L = Low; NCS = Not Clinically Significant; CS = Clinically Significant.

Listing 16.2.8.2a
Individual Biochemistry Results

Rando Number	Challenge Agent	Parameter (unit) [Normal Range]	Visit	Date of Assessment	Time of Assessment	Day Relative to Inoculum	Day Relative to IP	Result	Range Indicator	Clinical Assessment
R103	K13	Albumin (g/L) [35 – 48]	Screening	01-NOV-2017	12:16	-13	-22	43		
			Safety Visit	13-NOV-2017	08:44	-1	-10	40		
			Admission	23-NOV-2017	09:50	9	1	39		
			Safety Monitoring	25-NOV-2017	17:52	11	3	39		
			Admission 3	26-NOV-2017	10:53	12	4	38		
			Safety Monitoring	30-NOV-2017	10:09	16	8	36		
			Safety Monitoring	03-DEC-2017	10:45	19	11	45		
			Safety Monitoring	04-DEC-2017	09:27	20	12	38		
			Safety Monitoring	07-DEC-2017	08:41	23	15	37		
			End of Study	14-DEC-2017	09:37	30	22	41		
			Unscheduled	18-DEC-2017	07:20	34	26	40		

Notes: Day relative to inoculum is the number of days relative to day of administration of the inoculum, where the day of administration = 0.
The inoculum was administered on Study Day 0 for K13-infected subjects and Study Day 1 for 3D7-infected subjects.
Day relative to IP is the number of days relative to day of administration of Artesunate, where the day of dose = 1.
Rando = Randomisation; IP = Artesunate; H = High; L = Low; NCS = Not Clinically Significant; CS = Clinically Significant.

Listing 16.2.8.2a
Individual Biochemistry Results

Rando Number	Challenge Agent	Parameter (unit) [Normal Range]	Visit	Date of Assessment	Time of Assessment	Day Relative to Inoculum	Day Relative to IP	Result	Range Indicator	Clinical Assessment
R103	K13	Alkaline Phosphatase (U/L) [60 – 200]	Screening	01-NOV-2017	12:16	-13	-22	90		
			Safety Visit	13-NOV-2017	08:44	-1	-10	78		
			Admission	23-NOV-2017	09:50	9	1	77		
			Safety Monitoring	25-NOV-2017	17:52	11	3	76		
			Admission 3	26-NOV-2017	10:53	12	4	72		
			Safety Monitoring	30-NOV-2017	10:09	16	8	70		
			Safety Monitoring	03-DEC-2017	10:45	19	11	83		
			Safety Monitoring	04-DEC-2017	09:27	20	12	75		
			Safety Monitoring	07-DEC-2017	08:41	23	15	83		
			End of Study	14-DEC-2017	09:37	30	22	90		
			Unscheduled	18-DEC-2017	07:20	34	26	85		

Notes: Day relative to inoculum is the number of days relative to day of administration of the inoculum, where the day of administration = 0.
The inoculum was administered on Study Day 0 for K13-infected subjects and Study Day 1 for 3D7-infected subjects.
Day relative to IP is the number of days relative to day of administration of Artesunate, where the day of dose = 1.
Rando = Randomisation; IP = Artesunate; H = High; L = Low; NCS = Not Clinically Significant; CS = Clinically Significant.

Listing 16.2.8.2a
Individual Biochemistry Results

Rando Number	Challenge Agent	Parameter (unit) [Normal Range]	Visit	Date of Assessment	Time of Assessment	Day Relative to Inoculum	Day Relative to IP	Result	Range Indicator	Clinical Assessment
R103	K13	Aspartate Aminotransferase (U/L) [10 – 40]	Screening	01-NOV-2017	12:16	-13	-22	17		
			Safety Visit	13-NOV-2017	08:44	-1	-10	18		
			Admission	23-NOV-2017	09:50	9	1	17		
			Safety Monitoring	25-NOV-2017	17:52	11	3	23		
			Admission 3	26-NOV-2017	10:53	12	4	25		
			Safety Monitoring	30-NOV-2017	10:09	16	8	36		
			Safety Monitoring	03-DEC-2017	10:45	19	11	28		
			Safety Monitoring	04-DEC-2017	09:27	20	12	24		
			Safety Monitoring	07-DEC-2017	08:41	23	15	17		
			End of Study	14-DEC-2017	09:37	30	22	20		
			Unscheduled	18-DEC-2017	07:20	34	26	19		

Notes: Day relative to inoculum is the number of days relative to day of administration of the inoculum, where the day of administration = 0.
The inoculum was administered on Study Day 0 for K13-infected subjects and Study Day 1 for 3D7-infected subjects.
Day relative to IP is the number of days relative to day of administration of Artesunate, where the day of dose = 1.
Rando = Randomisation; IP = Artesunate; H = High; L = Low; NCS = Not Clinically Significant; CS = Clinically Significant.

Listing 16.2.8.2a
Individual Biochemistry Results

Rando Number	Challenge Agent	Parameter (unit) [Normal Range]	Visit	Date of Assessment	Time of Assessment	Day Relative to Inoculum	Day Relative to IP	Result	Range Indicator	Clinical Assessment
R103	K13	Bicarbonate (mmol/L) [20 – 32]	Screening	01-NOV-2017	12:16	-13	-22	30		
			Safety Visit	13-NOV-2017	08:44	-1	-10	30		
			Admission	23-NOV-2017	09:50	9	1	28		
			Safety Monitoring	25-NOV-2017	17:52	11	3	22		
			Admission 3	26-NOV-2017	10:53	12	4	23		
			Safety Monitoring	30-NOV-2017	10:09	16	8	27		
			Safety Monitoring	03-DEC-2017	10:45	19	11	26		
			Safety Monitoring	04-DEC-2017	09:27	20	12	29		
			Safety Monitoring	07-DEC-2017	08:41	23	15	27		
			End of Study	14-DEC-2017	09:37	30	22	27		
			Unscheduled	18-DEC-2017	07:20	34	26	27		

Notes: Day relative to inoculum is the number of days relative to day of administration of the inoculum, where the day of administration = 0.
The inoculum was administered on Study Day 0 for K13-infected subjects and Study Day 1 for 3D7-infected subjects.
Day relative to IP is the number of days relative to day of administration of Artesunate, where the day of dose = 1.
Rando = Randomisation; IP = Artesunate; H = High; L = Low; NCS = Not Clinically Significant; CS = Clinically Significant.

Listing 16.2.8.2a
Individual Biochemistry Results

Rando Number	Challenge Agent	Parameter (unit) [Normal Range]	Visit	Date of Assessment	Time of Assessment	Day Relative to Inoculum	Day Relative to IP	Result	Range Indicator	Clinical Assessment
R103	K13	Bilirubin (umol/L) [<21]	Screening	01-NOV-2017	12:16	-13	-22	23	H	NCS
			Unscheduled	09-NOV-2017	08:54	-5	-14	11		
			Safety Visit	13-NOV-2017	08:44	-1	-10	12		
			Admission	23-NOV-2017	09:50	9	1	13		
			Safety Monitoring	25-NOV-2017	17:52	11	3	9		
			Admission 3	26-NOV-2017	10:53	12	4	10		
			Safety Monitoring	30-NOV-2017	10:09	16	8	10		
			Safety Monitoring	03-DEC-2017	10:45	19	11	13		
			Safety Monitoring	04-DEC-2017	09:27	20	12	16		
			Safety Monitoring	07-DEC-2017	08:41	23	15	9		
			End of Study	14-DEC-2017	09:37	30	22	23	H	NCS
			Unscheduled	18-DEC-2017	07:20	34	26	12		

Notes: Day relative to inoculum is the number of days relative to day of administration of the inoculum, where the day of administration = 0.
The inoculum was administered on Study Day 0 for K13-infected subjects and Study Day 1 for 3D7-infected subjects.
Day relative to IP is the number of days relative to day of administration of Artesunate, where the day of dose = 1.
Rando = Randomisation; IP = Artesunate; H = High; L = Low; NCS = Not Clinically Significant; CS = Clinically Significant.

Listing 16.2.8.2a
Individual Biochemistry Results

Rando Number	Challenge Agent	Parameter (unit) [Normal Range]	Visit	Date of Assessment	Time of Assessment	Day Relative to Inoculum	Day Relative to IP	Result	Range Indicator	Clinical Assessment
R103	K13	Calcium Corrected (mmol/L) [2.1 – 2.6]	Screening	01-NOV-2017	12:16	-13	-22	2.45		
			Safety Visit	13-NOV-2017	08:44	-1	-10	2.42		
			Admission	23-NOV-2017	09:50	9	1	2.43		
			Safety Monitoring	25-NOV-2017	17:52	11	3	2.36		
			Admission 3	26-NOV-2017	10:53	12	4	2.33		
			Safety Monitoring	30-NOV-2017	10:09	16	8	2.40		
			Safety Monitoring	03-DEC-2017	10:45	19	11	2.41		
			Safety Monitoring	04-DEC-2017	09:27	20	12	2.45		
			Safety Monitoring	07-DEC-2017	08:41	23	15	2.34		
			End of Study	14-DEC-2017	09:37	30	22	2.45		
			Unscheduled	18-DEC-2017	07:20	34	26	2.37		

Notes: Day relative to inoculum is the number of days relative to day of administration of the inoculum, where the day of administration = 0.
The inoculum was administered on Study Day 0 for K13-infected subjects and Study Day 1 for 3D7-infected subjects.
Day relative to IP is the number of days relative to day of administration of Artesunate, where the day of dose = 1.
Rando = Randomisation; IP = Artesunate; H = High; L = Low; NCS = Not Clinically Significant; CS = Clinically Significant.

Listing 16.2.8.2a
Individual Biochemistry Results

Rando Number	Challenge Agent	Parameter (unit) [Normal Range]	Visit	Date of Assessment	Time of Assessment	Day Relative to Inoculum	Day Relative to IP	Result	Range Indicator	Clinical Assessment
R103	K13	Chloride (mmol/L) [95 – 110]	Screening	01-NOV-2017	12:16	-13	-22	104		
			Safety Visit	13-NOV-2017	08:44	-1	-10	106		
			Admission	23-NOV-2017	09:50	9	1	106		
			Safety Monitoring	25-NOV-2017	17:52	11	3	98		
			Admission 3	26-NOV-2017	10:53	12	4	100		
			Safety Monitoring	30-NOV-2017	10:09	16	8	109		
			Safety Monitoring	03-DEC-2017	10:45	19	11	100		
			Safety Monitoring	04-DEC-2017	09:27	20	12	105		
			Safety Monitoring	07-DEC-2017	08:41	23	15	106		
			End of Study	14-DEC-2017	09:37	30	22	104		
			Unscheduled	18-DEC-2017	07:20	34	26	106		
		Cholesterol (mmol/L) [3.9 – 5.5]	Screening	01-NOV-2017	12:16	-13	-22	4.3		
		Cholesterol/HDL-Cholesterol [0 – 4.5]	Screening	01-NOV-2017	12:16	-13	-22	2.9		

Notes: Day relative to inoculum is the number of days relative to day of administration of the inoculum, where the day of administration = 0.
The inoculum was administered on Study Day 0 for K13-infected subjects and Study Day 1 for 3D7-infected subjects.
Day relative to IP is the number of days relative to day of administration of Artesunate, where the day of dose = 1.
Rando = Randomisation; IP = Artesunate; H = High; L = Low; NCS = Not Clinically Significant; CS = Clinically Significant.

Listing 16.2.8.2a
Individual Biochemistry Results

Rando Number	Challenge Agent	Parameter (unit) [Normal Range]	Visit	Date of Assessment	Time of Assessment	Day Relative to Inoculum	Day Relative to IP	Result	Range Indicator	Clinical Assessment
R103	K13	Creatinine (umol/L) [60 – 110]	Screening	01-NOV-2017	12:16	-13	-22	88		
			Safety Visit	13-NOV-2017	08:44	-1	-10	77		
			Admission	23-NOV-2017	09:50	9	1	71		
			Safety Monitoring	25-NOV-2017	17:52	11	3	78		
			Admission 3	26-NOV-2017	10:53	12	4	82		
			Safety Monitoring	30-NOV-2017	10:09	16	8	79		
			Safety Monitoring	03-DEC-2017	10:45	19	11	93		
			Safety Monitoring	04-DEC-2017	09:27	20	12	81		
			Safety Monitoring	07-DEC-2017	08:41	23	15	75		
			End of Study	14-DEC-2017	09:37	30	22	75		
			Unscheduled	18-DEC-2017	07:20	34	26	77		

Notes: Day relative to inoculum is the number of days relative to day of administration of the inoculum, where the day of administration = 0.
The inoculum was administered on Study Day 0 for K13-infected subjects and Study Day 1 for 3D7-infected subjects.
Day relative to IP is the number of days relative to day of administration of Artesunate, where the day of dose = 1.
Rando = Randomisation; IP = Artesunate; H = High; L = Low; NCS = Not Clinically Significant; CS = Clinically Significant.

Listing 16.2.8.2a
Individual Biochemistry Results

Rando Number	Challenge Agent	Parameter (unit) [Normal Range]	Visit	Date of Assessment	Time of Assessment	Day Relative to Inoculum	Day Relative to IP	Result	Range Indicator	Clinical Assessment
R103	K13	Direct Bilirubin (umol/L) [<8]	Screening	01-NOV-2017	12:16	-13	-22	9	H	NCS
			Unscheduled	09-NOV-2017	08:54	-5	-14	5		
			Safety Visit	13-NOV-2017	08:44	-1	-10	5		
			Admission	23-NOV-2017	09:50	9	1	6		
			Safety Monitoring	25-NOV-2017	17:52	11	3	3		
			Admission 3	26-NOV-2017	10:53	12	4	<2		
			Safety Monitoring	30-NOV-2017	10:09	16	8	4		
			Safety Monitoring	03-DEC-2017	10:45	19	11	4		
			Safety Monitoring	04-DEC-2017	09:27	20	12	6		
			Safety Monitoring	07-DEC-2017	08:41	23	15	3		
			End of Study	14-DEC-2017	09:37	30	22	9	H	NCS
			Unscheduled	18-DEC-2017	07:20	34	26	5		
		Fasting Glucose (mmol/L) [3.6 – 6]	Screening	01-NOV-2017	12:16	-13	-22	4.5		
			Admission	23-NOV-2017	09:50	9	1	4.3		
			Safety Monitoring	25-NOV-2017	17:52	11	3	4.8		
			Safety Monitoring	03-DEC-2017	10:45	19	11	4.6		
			Safety Monitoring	04-DEC-2017	09:27	20	12	4.4		
			End of Study	14-DEC-2017	09:37	30	22	4.2		
			Unscheduled	18-DEC-2017	07:20	34	26	4.6		

Notes: Day relative to inoculum is the number of days relative to day of administration of the inoculum, where the day of administration = 0.
The inoculum was administered on Study Day 0 for K13-infected subjects and Study Day 1 for 3D7-infected subjects.
Day relative to IP is the number of days relative to day of administration of Artesunate, where the day of dose = 1.
Rando = Randomisation; IP = Artesunate; H = High; L = Low; NCS = Not Clinically Significant; CS = Clinically Significant.

Listing 16.2.8.2a
Individual Biochemistry Results

Rando Number	Challenge Agent	Parameter (unit) [Normal Range]	Visit	Date of Assessment	Time of Assessment	Day Relative to Inoculum	Day Relative to IP	Result	Range Indicator	Clinical Assessment
R103	K13	Globulin (g/L) [23 – 43]	Screening	01-NOV-2017	12:16	-13	-22	30		
			Safety Visit	13-NOV-2017	08:44	-1	-10	29		
			Admission	23-NOV-2017	09:50	9	1	28		
			Safety Monitoring	25-NOV-2017	17:52	11	3	29		
			Admission 3	26-NOV-2017	10:53	12	4	29		
			Safety Monitoring	30-NOV-2017	10:09	16	8	29		
			Safety Monitoring	03-DEC-2017	10:45	19	11	34		
			Safety Monitoring	04-DEC-2017	09:27	20	12	30		
			Safety Monitoring	07-DEC-2017	08:41	23	15	33		
			End of Study	14-DEC-2017	09:37	30	22	32		
			Unscheduled	18-DEC-2017	07:20	34	26	30		

Notes: Day relative to inoculum is the number of days relative to day of administration of the inoculum, where the day of administration = 0.
The inoculum was administered on Study Day 0 for K13-infected subjects and Study Day 1 for 3D7-infected subjects.
Day relative to IP is the number of days relative to day of administration of Artesunate, where the day of dose = 1.
Rando = Randomisation; IP = Artesunate; H = High; L = Low; NCS = Not Clinically Significant; CS = Clinically Significant.

Listing 16.2.8.2a
Individual Biochemistry Results

Rando Number	Challenge Agent	Parameter (unit) [Normal Range]	Visit	Date of Assessment	Time of Assessment	Day Relative to Inoculum	Day Relative to IP	Result	Range Indicator	Clinical Assessment
R103	K13	Glomerular Filtration Rate, Estimated [>59]	Screening	01-NOV-2017	12:16	-13	-22	>90		
			Safety Visit	13-NOV-2017	08:44	-1	-10	>90		
			Admission	23-NOV-2017	09:50	9	1	>90		
			Safety Monitoring	25-NOV-2017	17:52	11	3	>90		
			Admission 3	26-NOV-2017	10:53	12	4	>90		
			Safety Monitoring	30-NOV-2017	10:09	16	8	>90		
			Safety Monitoring	03-DEC-2017	10:45	19	11	>90		
			Safety Monitoring	04-DEC-2017	09:27	20	12	>90		
			Safety Monitoring	07-DEC-2017	08:41	23	15	>90		
			End of Study	14-DEC-2017	09:37	30	22	>90		
			Unscheduled	18-DEC-2017	07:20	34	26	>90		
		Glucose (mmol/L) [3.6 – 7.7]	Safety Visit	13-NOV-2017	08:44	-1	-10	4.4		
			Admission 3	26-NOV-2017	10:53	12	4	8.2	H	NCS
			Safety Monitoring	30-NOV-2017	10:09	16	8	4.5		
			Safety Monitoring	07-DEC-2017	08:41	23	15	4.1		
		HDL Cholesterol (mmol/L) [0.9 – 1.5]	Screening	01-NOV-2017	12:16	-13	-22	1.50		

Notes: Day relative to inoculum is the number of days relative to day of administration of the inoculum, where the day of administration = 0.
The inoculum was administered on Study Day 0 for K13-infected subjects and Study Day 1 for 3D7-infected subjects.
Day relative to IP is the number of days relative to day of administration of Artesunate, where the day of dose = 1.
Rando = Randomisation; IP = Artesunate; H = High; L = Low; NCS = Not Clinically Significant; CS = Clinically Significant.

Listing 16.2.8.2a
Individual Biochemistry Results

Rando Number	Challenge Agent	Parameter (unit) [Normal Range]	Visit	Date of Assessment	Time of Assessment	Day Relative to Inoculum	Day Relative to IP	Result	Range Indicator	Clinical Assessment
R103	K13	Hemolytic Index [0 – 40]	Screening	01-NOV-2017	12:16	-13	-22	5		
			Unscheduled	09-NOV-2017	08:54	-5	-14	7		
			Safety Visit	13-NOV-2017	08:44	-1	-10	8		
			Admission	23-NOV-2017	09:50	9	1	21		
			Safety Monitoring	25-NOV-2017	17:52	11	3	22		
			Safety Monitoring	30-NOV-2017	10:09	16	8	7		
			Safety Monitoring	03-DEC-2017	10:45	19	11	4		
			Safety Monitoring	04-DEC-2017	09:27	20	12	6		
			Safety Monitoring	07-DEC-2017	08:41	23	15	11		
			End of Study	14-DEC-2017	09:37	30	22	8		
			Unscheduled	18-DEC-2017	07:20	34	26	7		

Notes: Day relative to inoculum is the number of days relative to day of administration of the inoculum, where the day of administration = 0.
The inoculum was administered on Study Day 0 for K13-infected subjects and Study Day 1 for 3D7-infected subjects.
Day relative to IP is the number of days relative to day of administration of Artesunate, where the day of dose = 1.
Rando = Randomisation; IP = Artesunate; H = High; L = Low; NCS = Not Clinically Significant; CS = Clinically Significant.

Listing 16.2.8.2a
Individual Biochemistry Results

Rando Number	Challenge Agent	Parameter (unit) [Normal Range]	Visit	Date of Assessment	Time of Assessment	Day Relative to Inoculum	Day Relative to IP	Result	Range Indicator	Clinical Assessment
R103	K13	Icteric Index [0 – 200]	Screening	01-NOV-2017	12:16	-13	-22	27		
			Unscheduled	09-NOV-2017	08:54	-5	-14	16		
			Safety Visit	13-NOV-2017	08:44	-1	-10	17		
			Admission	23-NOV-2017	09:50	9	1	14		
			Safety Monitoring	25-NOV-2017	17:52	11	3	14		
			Admission 3	26-NOV-2017	10:53	12	4	11		
			Safety Monitoring	30-NOV-2017	10:09	16	8	16		
			Safety Monitoring	03-DEC-2017	10:45	19	11	18		
			Safety Monitoring	04-DEC-2017	09:27	20	12	18		
			Safety Monitoring	07-DEC-2017	08:41	23	15	13		
			End of Study	14-DEC-2017	09:37	30	22	24		
			Unscheduled	18-DEC-2017	07:20	34	26	17		
		LDL Cholesterol (mmol/L) [0 – 4]	Screening	01-NOV-2017	12:16	-13	-22	2.4		

Notes: Day relative to inoculum is the number of days relative to day of administration of the inoculum, where the day of administration = 0.
The inoculum was administered on Study Day 0 for K13-infected subjects and Study Day 1 for 3D7-infected subjects.
Day relative to IP is the number of days relative to day of administration of Artesunate, where the day of dose = 1.
Rando = Randomisation; IP = Artesunate; H = High; L = Low; NCS = Not Clinically Significant; CS = Clinically Significant.

Listing 16.2.8.2a
Individual Biochemistry Results

Rando Number	Challenge Agent	Parameter (unit) [Normal Range]	Visit	Date of Assessment	Time of Assessment	Day Relative to Inoculum	Day Relative to IP	Result	Range Indicator	Clinical Assessment
R103	K13	Lactate Dehydrogenase (U/L) [120 – 250]	Screening	01-NOV-2017	12:16	-13	-22	168		
			Safety Visit	13-NOV-2017	08:44	-1	-10	173		
			Admission	23-NOV-2017	09:50	9	1	174		
			Safety Monitoring	25-NOV-2017	17:52	11	3	199		
			Admission 3	26-NOV-2017	10:53	12	4	:SPU		
			Safety Monitoring	30-NOV-2017	10:09	16	8	226		
			Safety Monitoring	03-DEC-2017	10:45	19	11	221		
			Safety Monitoring	04-DEC-2017	09:27	20	12	191		
			Safety Monitoring	07-DEC-2017	08:41	23	15	188		
			End of Study	14-DEC-2017	09:37	30	22	180		
			Unscheduled	18-DEC-2017	07:20	34	26	167		

Notes: Day relative to inoculum is the number of days relative to day of administration of the inoculum, where the day of administration = 0.
The inoculum was administered on Study Day 0 for K13-infected subjects and Study Day 1 for 3D7-infected subjects.
Day relative to IP is the number of days relative to day of administration of Artesunate, where the day of dose = 1.
Rando = Randomisation; IP = Artesunate; H = High; L = Low; NCS = Not Clinically Significant; CS = Clinically Significant.

Listing 16.2.8.2a
Individual Biochemistry Results

Rando Number	Challenge Agent	Parameter (unit) [Normal Range]	Visit	Date of Assessment	Time of Assessment	Day Relative to Inoculum	Day Relative to IP	Result	Range Indicator	Clinical Assessment
R103	K13	Lipemic Index [0 – 10]	Screening	01-NOV-2017	12:16	-13	-22	0		
			Unscheduled	09-NOV-2017	08:54	-5	-14	0		
			Safety Visit	13-NOV-2017	08:44	-1	-10	0		
		Lipemic Index [<2]	Admission	23-NOV-2017	09:50	9	1	0		
			Safety Monitoring	25-NOV-2017	17:52	11	3	0		
			Admission 3	26-NOV-2017	10:53	12	4	0		
			Safety Monitoring	30-NOV-2017	10:09	16	8	0		
			Safety Monitoring	03-DEC-2017	10:45	19	11	0		
			Safety Monitoring	04-DEC-2017	09:27	20	12	0		
			Safety Monitoring	07-DEC-2017	08:41	23	15	1		
			End of Study	14-DEC-2017	09:37	30	22	0		
			Unscheduled	18-DEC-2017	07:20	34	26	0		
		Magnesium (mmol/L) [0.7 – 1.1]	Screening	01-NOV-2017	12:16	-13	-22	0.86		

Notes: Day relative to inoculum is the number of days relative to day of administration of the inoculum, where the day of administration = 0.
The inoculum was administered on Study Day 0 for K13-infected subjects and Study Day 1 for 3D7-infected subjects.
Day relative to IP is the number of days relative to day of administration of Artesunate, where the day of dose = 1.
Rando = Randomisation; IP = Artesunate; H = High; L = Low; NCS = Not Clinically Significant; CS = Clinically Significant.

Listing 16.2.8.2a
Individual Biochemistry Results

Rando Number	Challenge Agent	Parameter (unit) [Normal Range]	Visit	Date of Assessment	Time of Assessment	Day Relative to Inoculum	Day Relative to IP	Result	Range Indicator	Clinical Assessment
R103	K13	Phosphate (mmol/L) [0.8 – 1.5]	Screening	01-NOV-2017	12:16	-13	-22	1.24		
			Safety Visit	13-NOV-2017	08:44	-1	-10	0.98		
			Admission	23-NOV-2017	09:50	9	1	1.25		
			Safety Monitoring	25-NOV-2017	17:52	11	3	1.27		
			Admission 3	26-NOV-2017	10:53	12	4	1.05		
			Safety Monitoring	30-NOV-2017	10:09	16	8	1.09		
			Safety Monitoring	03-DEC-2017	10:45	19	11	1.21		
			Safety Monitoring	04-DEC-2017	09:27	20	12	1.06		
			Safety Monitoring	07-DEC-2017	08:41	23	15	1.09		
			End of Study	14-DEC-2017	09:37	30	22	1.14		
			Unscheduled	18-DEC-2017	07:20	34	26	1.23		

Notes: Day relative to inoculum is the number of days relative to day of administration of the inoculum, where the day of administration = 0.
The inoculum was administered on Study Day 0 for K13-infected subjects and Study Day 1 for 3D7-infected subjects.
Day relative to IP is the number of days relative to day of administration of Artesunate, where the day of dose = 1.
Rando = Randomisation; IP = Artesunate; H = High; L = Low; NCS = Not Clinically Significant; CS = Clinically Significant.

Listing 16.2.8.2a
Individual Biochemistry Results

Rando Number	Challenge Agent	Parameter (unit) [Normal Range]	Visit	Date of Assessment	Time of Assessment	Day Relative to Inoculum	Day Relative to IP	Result	Range Indicator	Clinical Assessment
R103	K13	Potassium (mmol/L) [3.5 – 5.5]	Screening	01-NOV-2017	12:16	-13	-22	4.7		
			Safety Visit	13-NOV-2017	08:44	-1	-10	4.0		
			Admission	23-NOV-2017	09:50	9	1	4.4		
			Safety Monitoring	25-NOV-2017	17:52	11	3	4.0		
			Admission 3	26-NOV-2017	10:53	12	4	4.6		
			Safety Monitoring	30-NOV-2017	10:09	16	8	4.5		
			Safety Monitoring	03-DEC-2017	10:45	19	11	4.7		
			Safety Monitoring	04-DEC-2017	09:27	20	12	4.2		
			Safety Monitoring	07-DEC-2017	08:41	23	15	4.1		
			End of Study	14-DEC-2017	09:37	30	22	3.8		
			Unscheduled	18-DEC-2017	07:20	34	26	4.0		

Notes: Day relative to inoculum is the number of days relative to day of administration of the inoculum, where the day of administration = 0.
The inoculum was administered on Study Day 0 for K13-infected subjects and Study Day 1 for 3D7-infected subjects.
Day relative to IP is the number of days relative to day of administration of Artesunate, where the day of dose = 1.
Rando = Randomisation; IP = Artesunate; H = High; L = Low; NCS = Not Clinically Significant; CS = Clinically Significant.

Listing 16.2.8.2a
Individual Biochemistry Results

Rando Number	Challenge Agent	Parameter (unit) [Normal Range]	Visit	Date of Assessment	Time of Assessment	Day Relative to Inoculum	Day Relative to IP	Result	Range Indicator	Clinical Assessment
R103	K13	Protein (g/L) [66 – 83]	Screening	01-NOV-2017	12:16	-13	-22	73		
			Safety Visit	13-NOV-2017	08:44	-1	-10	69		
			Admission	23-NOV-2017	09:50	9	1	67		
			Safety Monitoring	25-NOV-2017	17:52	11	3	68		
			Admission 3	26-NOV-2017	10:53	12	4	67		
			Safety Monitoring	30-NOV-2017	10:09	16	8	65	L	NCS
			Safety Monitoring	03-DEC-2017	10:45	19	11	79		
			Safety Monitoring	04-DEC-2017	09:27	20	12	68		
			Safety Monitoring	07-DEC-2017	08:41	23	15	70		
			End of Study	14-DEC-2017	09:37	30	22	73		
			Unscheduled	18-DEC-2017	07:20	34	26	70		

Notes: Day relative to inoculum is the number of days relative to day of administration of the inoculum, where the day of administration = 0.
The inoculum was administered on Study Day 0 for K13-infected subjects and Study Day 1 for 3D7-infected subjects.
Day relative to IP is the number of days relative to day of administration of Artesunate, where the day of dose = 1.
Rando = Randomisation; IP = Artesunate; H = High; L = Low; NCS = Not Clinically Significant; CS = Clinically Significant.

Listing 16.2.8.2a
Individual Biochemistry Results

Rando Number	Challenge Agent	Parameter (unit) [Normal Range]	Visit	Date of Assessment	Time of Assessment	Day Relative to Inoculum	Day Relative to IP	Result	Range Indicator	Clinical Assessment
R103	K13	Sodium (mmol/L) [135 – 145]	Screening	01-NOV-2017	12:16	-13	-22	141		
			Safety Visit	13-NOV-2017	08:44	-1	-10	141		
			Admission	23-NOV-2017	09:50	9	1	140		
			Safety Monitoring	25-NOV-2017	17:52	11	3	139		
			Admission 3	26-NOV-2017	10:53	12	4	138		
			Safety Monitoring	30-NOV-2017	10:09	16	8	144		
			Safety Monitoring	03-DEC-2017	10:45	19	11	142		
			Safety Monitoring	04-DEC-2017	09:27	20	12	141		
			Safety Monitoring	07-DEC-2017	08:41	23	15	139		
			End of Study	14-DEC-2017	09:37	30	22	139		
			Unscheduled	18-DEC-2017	07:20	34	26	140		
		Triglycerides (mmol/L) [0.6 – 2]	Screening	01-NOV-2017	12:16	-13	-22	0.9		

Notes: Day relative to inoculum is the number of days relative to day of administration of the inoculum, where the day of administration = 0.
The inoculum was administered on Study Day 0 for K13-infected subjects and Study Day 1 for 3D7-infected subjects.
Day relative to IP is the number of days relative to day of administration of Artesunate, where the day of dose = 1.
Rando = Randomisation; IP = Artesunate; H = High; L = Low; NCS = Not Clinically Significant; CS = Clinically Significant.

Listing 16.2.8.2a
Individual Biochemistry Results

Rando Number	Challenge Agent	Parameter (unit) [Normal Range]	Visit	Date of Assessment	Time of Assessment	Day Relative to Inoculum	Day Relative to IP	Result	Range Indicator	Clinical Assessment
R103	K13	Urate (mmol/L) [0.2 – 0.5]	Screening	01-NOV-2017	12:16	-13	-22	0.303		
			Safety Visit	13-NOV-2017	08:44	-1	-10	0.278		
			Admission	23-NOV-2017	09:50	9	1	0.246		
			Safety Monitoring	25-NOV-2017	17:52	11	3	0.256		
			Admission 3	26-NOV-2017	10:53	12	4	0.251		
			Safety Monitoring	30-NOV-2017	10:09	16	8	0.369		
			Safety Monitoring	03-DEC-2017	10:45	19	11	0.322		
			Safety Monitoring	04-DEC-2017	09:27	20	12	0.350		
			Safety Monitoring	07-DEC-2017	08:41	23	15	0.305		
			End of Study	14-DEC-2017	09:37	30	22	0.302		
			Unscheduled	18-DEC-2017	07:20	34	26	0.297		

Notes: Day relative to inoculum is the number of days relative to day of administration of the inoculum, where the day of administration = 0.
The inoculum was administered on Study Day 0 for K13-infected subjects and Study Day 1 for 3D7-infected subjects.
Day relative to IP is the number of days relative to day of administration of Artesunate, where the day of dose = 1.
Rando = Randomisation; IP = Artesunate; H = High; L = Low; NCS = Not Clinically Significant; CS = Clinically Significant.

Listing 16.2.8.2a
Individual Biochemistry Results

Rando Number	Challenge Agent	Parameter (unit) [Normal Range]	Visit	Date of Assessment	Time of Assessment	Day Relative to Inoculum	Day Relative to IP	Result	Range Indicator	Clinical Assessment
R103	K13	Urea Nitrogen (mmol/L) [3 – 7.5]	Screening	01-NOV-2017	12:16	-13	-22	4.4		
			Safety Visit	13-NOV-2017	08:44	-1	-10	4.9		
			Admission	23-NOV-2017	09:50	9	1	5.8		
			Safety Monitoring	25-NOV-2017	17:52	11	3	4.9		
			Admission 3	26-NOV-2017	10:53	12	4	4.9		
			Safety Monitoring	30-NOV-2017	10:09	16	8	6.1		
			Safety Monitoring	03-DEC-2017	10:45	19	11	6.4		
			Safety Monitoring	04-DEC-2017	09:27	20	12	6.5		
			Safety Monitoring	07-DEC-2017	08:41	23	15	6.6		
			End of Study	14-DEC-2017	09:37	30	22	6.4		
			Unscheduled	18-DEC-2017	07:20	34	26	6.2		

Notes: Day relative to inoculum is the number of days relative to day of administration of the inoculum, where the day of administration = 0.
The inoculum was administered on Study Day 0 for K13-infected subjects and Study Day 1 for 3D7-infected subjects.
Day relative to IP is the number of days relative to day of administration of Artesunate, where the day of dose = 1.
Rando = Randomisation; IP = Artesunate; H = High; L = Low; NCS = Not Clinically Significant; CS = Clinically Significant.

Listing 16.2.8.2a
Individual Biochemistry Results

Rando Number	Challenge Agent	Parameter (unit) [Normal Range]	Visit	Date of Assessment	Time of Assessment	Day Relative to Inoculum	Day Relative to IP	Result	Range Indicator	Clinical Assessment
R104	K13	Alanine Aminotransferase (U/L) [5 – 40]	Screening	09-NOV-2017	10:32	-5	-14	24		
			Safety Visit	13-NOV-2017	07:54	-1	-10	26		
			Admission	23-NOV-2017	09:44	9	1	24		
			Safety Monitoring	25-NOV-2017	17:43	11	3	84	H	NCS
			Admission 3	26-NOV-2017	10:41	12	4	80	H	NCS
			Safety Monitoring	30-NOV-2017	08:43	16	8	248	H	CS
			Safety Monitoring	03-DEC-2017	09:13	19	11	133	H	CS
			Safety Monitoring	04-DEC-2017	12:45	20	12	113	H	NCS
			Safety Monitoring	07-DEC-2017	08:35	23	15	69	H	NCS
			End of Study	14-DEC-2017	08:42	30	22	32		

Notes: Day relative to inoculum is the number of days relative to day of administration of the inoculum, where the day of administration = 0.
The inoculum was administered on Study Day 0 for K13-infected subjects and Study Day 1 for 3D7-infected subjects.
Day relative to IP is the number of days relative to day of administration of Artesunate, where the day of dose = 1.
Rando = Randomisation; IP = Artesunate; H = High; L = Low; NCS = Not Clinically Significant; CS = Clinically Significant.

Listing 16.2.8.2a
Individual Biochemistry Results

Rando Number	Challenge Agent	Parameter (unit) [Normal Range]	Visit	Date of Assessment	Time of Assessment	Day Relative to Inoculum	Day Relative to IP	Result	Range Indicator	Clinical Assessment
R104	K13	Albumin (g/L) [35 – 48]	Screening	09-NOV-2017	10:32	-5	-14	39		
			Safety Visit	13-NOV-2017	07:54	-1	-10	39		
			Admission	23-NOV-2017	09:44	9	1	37		
			Safety Monitoring	25-NOV-2017	17:43	11	3	37		
			Admission 3	26-NOV-2017	10:41	12	4	37		
			Safety Monitoring	30-NOV-2017	08:43	16	8	39		
			Safety Monitoring	03-DEC-2017	09:13	19	11	43		
			Safety Monitoring	04-DEC-2017	12:45	20	12	38		
			Safety Monitoring	07-DEC-2017	08:35	23	15	37		
			End of Study	14-DEC-2017	08:42	30	22	38		

Notes: Day relative to inoculum is the number of days relative to day of administration of the inoculum, where the day of administration = 0.
The inoculum was administered on Study Day 0 for K13-infected subjects and Study Day 1 for 3D7-infected subjects.
Day relative to IP is the number of days relative to day of administration of Artesunate, where the day of dose = 1.
Rando = Randomisation; IP = Artesunate; H = High; L = Low; NCS = Not Clinically Significant; CS = Clinically Significant.

Listing 16.2.8.2a
Individual Biochemistry Results

Rando Number	Challenge Agent	Parameter (unit) [Normal Range]	Visit	Date of Assessment	Time of Assessment	Day Relative to Inoculum	Day Relative to IP	Result	Range Indicator	Clinical Assessment
		Alkaline Phosphatase (U/L) [35 – 110]	Screening	09-NOV-2017	10:32	-5	-14	82		
			Safety Visit	13-NOV-2017	07:54	-1	-10	78		
			Admission	23-NOV-2017	09:44	9	1	79		
			Safety Monitoring	25-NOV-2017	17:43	11	3	104		
			Admission 3	26-NOV-2017	10:41	12	4	105		
			Safety Monitoring	30-NOV-2017	08:43	16	8	110		
			Safety Monitoring	03-DEC-2017	09:13	19	11	105		
			Safety Monitoring	04-DEC-2017	12:45	20	12	98		
			Safety Monitoring	07-DEC-2017	08:35	23	15	93		
			End of Study	14-DEC-2017	08:42	30	22	92		

Notes: Day relative to inoculum is the number of days relative to day of administration of the inoculum, where the day of administration = 0.
The inoculum was administered on Study Day 0 for K13-infected subjects and Study Day 1 for 3D7-infected subjects.
Day relative to IP is the number of days relative to day of administration of Artesunate, where the day of dose = 1.
[truncated: 340,117 more chars]
