# Supplementary material for: Safety and parasite clearance of artemisinin-resistant Plasmodium falciparum infection: A pilot and a randomised volunteer infection study in Australia
Source: PLoS Med. 2020 Aug 21;17(8):e1003203. doi: 10.1371/journal.pmed.1003203 (PMC7444516; doi:10.1371/journal.pmed.1003203)
Supplement: Attachment — Submitted filename: Watts_et_al_response_reviewers.pdf [file pmed.1003203.s027.pdf]

PLOS Medicine

Response to comments from Editors and Reviewers

Manuscript: "Safety and parasite clearance of artemisinin-resistant *Plasmodium falciparum* infection: A pilot and a randomised volunteer infection study in Australia"

(PMEDICINE-D-20-00295R1)

Note to editors and reviewers: page and lines numbers are as per clean copy of the submitted manuscript

Academic Editor comments

- This is a descriptive study but readers may be helped if the authors could formulate a clear research question which could be presented at the end of the introduction?

We have added to the end of the introduction a sentence describing the research question the study addressed (page 7 line 125–131):

"The aim of this study was to evaluate the safety and infectivity of artemisinin-resistant parasites and to compare the parasitological response of the artemisinin-resistant and artemisinin-sensitive parasites to artesunate in healthy participants inoculated in the IBSM model. We hypothesised that artesunate would clear parasitaemia from participants infected with artemisinin-resistant parasites at a slower rate compared to participants infected with artemisinin-sensitive parasites, as observed in field studies [2]."

Editor comments

- Title: Please revise your title according to PLOS Medicine's style. Would it be more accurate to call your study 2 phase 1 trials? Please add the country setting.

We have amended the title to add the two clinical trials: a pilot and a randomised volunteer infection study. Although both clinical trials are technically phase 1 studies, we believe having "volunteer infection study" in the title is more descriptive than phase 1 study. We have also added the country setting to the title (page 1, lines 1–2):

"Safety and parasite clearance of artemisinin-resistant Plasmodium falciparum infection: A pilot and a randomised volunteer infection study in Australia"

- Abstract – Please add summary demographic information including mean age; Please add information regarding country setting and towns and recruitment.

We have added summary demographic information to the abstract, including sex and median age (page 2, lines 34–40):

"In the pilot study, two participants were enrolled between April 27 and September 12, 2017, and included in final analyses (males n=2 [100%], mean age=26 years [range, 23–28 years]). In the comparative study, 25 participants were enrolled between October 26, 2017 and October 18, 2018, of whom 22 were inoculated and included in final analyses (artemisinin-resistant infected participants: males n=7 [53.8%], median age=22 years [range, 18–40 years]; artemisinin-sensitive infected participants: males n=5 [55.6%], median age=28 years [range, 22–35 years]"

We have added information regarding country setting and towns and recruitment to the abstract (page 2, lines 26–27):

"We conducted two sequential phase 1, single-centre, open-label clinical trials at Q-Pharm, Brisbane, Australia, ..."

- Main text – Please ensure refs are in square brackets before punctuation.

All references are now in square brackets before punctuation.

- Data – note an author cannot be a point of contact for data requests. Please provide another contact such as a data manager.

We have added our data manager at QIMR Berghofer as the point of contact for data request, together with the corresponding author (page 28, lines 538–544):

"Investigators who seek access to individual participant data will contact Prof. James S. McCarthy and the QIMR Berghofer Data Manager (Ria.Woo@qimrberghofer.edu.au), ..."

- Throughout the text there is a lack of information about the locations of these trials as well as where recruitment was. Please provide.

Both studies were single-centre conducted in Brisbane, Australia. This information is provided in the Study Design subheading in the Methods section (page 7, line 140):

"The studies were conducted at Q-Pharm Pty Ltd, Brisbane, Australia, ..."

We have now also added this information to the abstract (page 2, lines 26–27):

"We conducted two sequential phase 1, single-centre, open-label clinical trials at Q-Pharm, Brisbane, Australia, ..."

Please let us know if we should add this information elsewhere in the text.

- Please use sections and paragraphs in the CONSORT as page numbers can change during revision and formatting.

We have added sections and paragraphs to the CONSORT checklist (see updated CONSORT checklist in S1 Supporting Information).

- Did your study have a prospective protocol or analysis plan? Please state this (either way) early in the Methods section.
  a) If a prospective analysis plan (from your funding proposal, IRB or other ethics committee submission, study protocol, or other planning document written before analyzing the data) was used in designing the study, please include the relevant prospectively written document with your revised manuscript as a Supporting Information file to be published alongside your study, and cite it in the Methods section. A legend for this file should be included at the end of your manuscript.
  b) If no such document exists, please make sure that the Methods section transparently describes when analyses were planned, and when/why any data-driven changes to analyses took place.
  c) In either case, changes in the analysis – including those made in response to peer review comments – should be identified as such in the Methods section of the paper, with rationale.

Both studies had a clinical trial protocol, which were approved by the QIMR Berghofer and Australian Red Cross Blood Service Human Research Ethics Committees before study initiation.

- *The protocol of the pilot study described the methodology to analyse the primary and secondary objectives of the study.*
- *The protocol of the comparative study described the methodology to analyse the primary objectives of the study. In addition, the comparative study had a statistical analysis plan that was finalised before the data was locked for final analysis. The statistical analysis plan describes in more detail than the protocol how the demographics and safety data was to be analysed.*

We have submitted the protocol of both studies (S2 and S3 Supplementary Information) and the statistical analysis plan of the comparative study (S4 Supplementary Information), and added this information to the Methods section (pages 7–8, lines 143–146):

"Data was analysed according to the clinical trial protocols (S2 and S3 Supporting Information) and the statistical analysis plan (comparative study, S4 Supporting Information), which was finalised before the data was locked for analysis."

- Data – you say some restrictions will apply, then say all data is available in the MS and Supp Files. Please state what data is available and what is not and why.

All data presented in the manuscript will be available. The only restriction that will apply is that data will be shared upon request. The reason for this is because our data involves human research participant data. This data access restriction is acceptable according to PLOS Medicine guidelines for data sharing (from https://journals.plos.org/plosmedicine/s/data-availability):

"For studies involving human research participant data or other sensitive data, we encourage authors to share de-identified or anonymized data. However, when data cannot be publicly shared, we allow authors to make their data sets available upon request."

Please see wording in the Data Sharing statement in our manuscript and let us know if you need any further clarification (pages 28, lines 538–544):

"Data collected for this study will be made available immediately after article publication with no end date. De-identified datasets containing the variables analysed for the primary and secondary outcomes will be made available as well as other supporting documents (e.g., protocol and informed consent). Investigators who seek access to individual participant data will contact Prof. James S. McCarthy and the QIMR Berghofer Data Manager (Ria.Woo@qimrberghofer.edu.au), who will send them the data via a secure data sharing platform."

- Abstract – this should be formatted with 3 sections: Background, Method and Findings, Conclusion. Please format accordingly.

The abstract is currently formatted with these 3 sections: Background, Method and Findings and Conclusion. No change is required.

- Abstract – use months as well as years for recruitment and tell us the cities / regions where recruitment took place, if known; Please use 95% CIs as well as p values for all quantifiable

data (both here and throughout the manuscript); Please ensure summary demographic information is provided, including gender ratio and mean age and number of smokers, etc; Please provide – as the last sentence of the 'Methods and Findings' section a sentence on the limitations of the study;

The abstract includes months as well as years for recruitment. We have added summary demographic information, including gender ratio and median age (page 2, lines 34–40):

"In the pilot study, two participants were enrolled between April 27 and September 12, 2017, and included in final analyses (males n=2 [100%], mean age=26 years [range, 23–28 years]). In the comparative study, 25 participants were enrolled between October 26, 2017 and October 18, 2018, of whom 22 were inoculated and included in final analyses (artemisinin-resistant infected participants: males n=7 [53.8%], median age=22 years [range, 18–40 years]; artemisinin-sensitive infected participants: males n=5 [55.6%], median age=28 years [range, 22–35 years]."

We have added the city and country were the study took place (page 2, lines 26–27):

"We conducted two sequential phase 1, open-label clinical trials at Q-Pharm, Brisbane, Australia, …"

The abstract includes as the last sentence of the "Methods and Findings section a sentence on the limitations of the study (page 3, lines 47–48):

"The main limitation of this study was that the artemisinin-resistant and artemisinin-sensitive parasite strains did not share the same genetic background."

- At this stage, we ask that you include a short, non-technical Author Summary of your research to make findings accessible to a wide audience that includes both scientists and non-scientists. The Author Summary should immediately follow the Abstract in your revised manuscript. This text is subject to editorial change and should be distinct from the scientific abstract. Please see our author guidelines for more information: https://journals.plos.org/plosmedicine/s/revising-your-manuscript#loc-author-summary

We have included an Author Summary to the revised manuscript (pages 4–5, lines 59–93).

- Refs in main text, these need to be presented in square brackets and not using superscript.

References in main text are presented in square brackets. No changes needed.

- In the main text where you use 95% CIs, please also add p values.

The main text already presents p values where relevant. Currently, all the p values are difference between two groups. Where we are not comparing groups, a p value is not informative.

- Please include line numbers on your revised version.

We have added line numbers to the revised version of the manuscript.

- Please be careful of deriving cause where non can be drawn as this is not a trial: “Our data provide evidence…” and “In summary, our study provides evidence that obesity, even without metabolic syndrome…”

We have been careful of deriving cause where non can be drawn.

- Please use the "Vancouver" style for reference formatting, and see our website for other reference guidelines https://journals.plos.org/plosmedicine/s/submission-guidelines#loc-references (noting remove ital font specifically)

We have used the Vancouver style to format references and removed the italic font as suggested (pages 29–33, lines 546–642).

- Did your study have a prospective protocol or analysis plan? Please state this (either way) early in the Methods section.

  a) If a prospective analysis plan (from your funding proposal, IRB or other ethics committee submission, study protocol, or other planning document written before analyzing the data) was used in designing the study, please include the relevant prospectively written document with your revised manuscript as a Supporting Information file to be published alongside your study, and cite it in the Methods section. A legend for this file should be included at the end of your manuscript.

  b) If no such document exists, please make sure that the Methods section transparently describes when analyses were planned, and when/why any data-driven changes to analyses took place.

  c) In either case, changes in the analysis-- including those made in response to peer review comments-- should be identified as such in the Methods section of the paper, with rationale.

Both studies had a clinical trial protocol, which were approved by the QIMR Berghofer and Australian Red Cross Blood Service Human Research Ethics Committees before study initiation.

- *The protocol of the pilot study described the methodology to analyse the primary and secondary objectives of the study.*
- *The protocol of the comparative study described the methodology to analyse the primary objectives of the study. In addition, the comparative study had a statistical analysis plan that was finalised before the data was locked for final analysis. The statistical analysis plan described in more detail than the protocol how the demographics and safety data was to be analysed.*

We have submitted the protocol of both studies (S2 and S3 Supplementary Information) and the statistical analysis plan of the comparative study (S4 Supplementary Information), and added this information to the Methods section (pages 7–8, lines 143–146):

"Data was analysed according to the clinical trial protocols (S2 and S3 Supporting Information) and the statistical analysis plan (comparative study, S4 Supporting Information), which was finalised before the data was locked for analysis."

- Please ensure that the study is reported according to the STROBE guideline, and include the completed STROBE checklist as Supporting Information. Please add the following statement, or similar, to the Methods: "This study is reported as per the Strengthening the Reporting of Observational Studies in Epidemiology (STROBE) guideline (S1 Checklist)." The STROBE guideline can be found here: http://www.equator-network.org/reporting-guidelines/strobe/

  When completing the checklist, please use section and paragraph numbers, rather than page numbers.

STROBE guidelines are for observational studies, whereas this study is an interventional randomized clinical trial. We have reported the study according to CONSORT, which are the guidelines for randomized clinical trials.

Comments from the reviewers

Reviewer #1

To the authors,

The manuscript "Safety and parasite clearance of artemisinin-resistant *Plasmodium falciparum* infection: a randomised volunteer infection study" by Watts et al describes with full details the establishment of an induced blood stage malaria model in human volunteers and the results they obtain in two assays.

The manuscript is well written, the study is nicely designed and incorporates all the necessary prerequisites for the selection of the volunteers, the monitoring of the infections and the clinical management of the patient.

Some minor amendments / precisions could give some valuable points:

- A justification of why from the beginning IBSM was preferred to sporozoites induced infections that are more close to natural ones?

The artemisinin resistance phenotype is linked to delayed clearance of blood stage parasites after treatment with an artemisinin or an artemisinin combination therapy. Therefore, we used IBSM because it focuses on the drug effects on blood-stage parasites, which is the relevant stage for the phenotypic hallmark of artemisinin-resistance. We believe conducting these studies with sporozoites would not provide additional information, nor allow to address the main objectives of this study better.

- While assessing parasitaemia by 18S qPCR is very precise, a justification of why not making blood film concurrently could be useful particularly to correlate with stages present and their development.

Monitoring parasitemia by microscopy at such low levels (around the limit of detection by thick film) is extremely labour intensive, time-consuming, has a long turn-around time and is subject to stochastic errors around the limit of detection. We need to obtain results in timely manner (within 24 hours) to ensure the safety of participants. We use routinely 18S qPCR to measure parasitemia in the IBSM model and have accrued sufficient data to validate the 18S qPCR assay to reliably measure parasitaemia at submicroscopic levels. A manuscript presenting the results from this validation work is currently under review and may be able to be referenced if accepted for publication.

- The use of the name "K13 parasite" all along the study sounds inadequate and could be misleading since all *P. falciparum* (including the 3D7 use for comparison) harbour the K13 gene. The use another name or the real strain or isolate name could be preferable.

The authors agree with the reviewer. We have changed the wording in the manuscript to refer and to the parasites as artemisinin-resistant (K13^{R539T} strain) or artemisinin-sensitive (3D7 strain) parasites.

- The production of the master cell bank references the 3D7 strain. No precision is given for the production of ART-R parasite MCB and the study only mentioned as a limitation that both parasites have different genetic background. Was the same method (GAP) applied to the production of the MCB for the ART-R parasite? Which gene have been targeted? Could the authors give precision about ART-R parasite MCB such as parasite stages also represented by 96% of rings, presence of schizonts and gametocytes, parasitaemia?

The artemisinin-resistant bank was produced by expanding artemisinin-resistant parasites carrying the kelch 13 mutation R539T (K13^{R539T} strain) using the bioreactor system described in Pawliw et al., 2018, Malar J, 17:283. The bank expanded in the cited publication was a genetically attenuated parasite (GAP). However, no genetic manipulation was undertaken during the production of the artemisinin-resistant bank. The cited paper describes a methodology to expand parasites in vitro using a bioreactor system, regardless of whether the parasites are genetically modified or not.

We have amended the text in the results section of the manuscript to specify that the artemisinin-resistant master cell bank was manufactured using the bioreactor system as described by Pawliw et al, 2018 (page 8, lines 164–165). Furthermore, we have added information about the characterisation of the artemisinin-resistant master cell bank to the Appendix (see Text C in S1 Appendix):

"The artemisinin-resistant master cell bank was manufactured using a bioreactor system as previously described [10], and characterised at the time of release (Text C in S1 Appendix).".

- The ART-R strain used was R539T while the authors acknowledged that this strain have been outcompeted by some other such as the C580Y, they could also highlight that this strain is widely distributed in SEA with adequate reference.

We have added information to the discussion section about the geographic distribution of both strains (pages 25–26, lines 485–486):

"Furthermore, both the R539T and the C580Y strains are widely distributed across the Greater Mekong Subregion [31, 32]".

- The listed serological tests include: HBs Ag, anti-HBc Ab, anti-HCV Ab, anti-HIV1 and anti-HIV2 Ab. Despite stringent selection criteria, did any serological tests for Malaria were performed?

No serological test for malaria were performed in study participants. All participants were carefully questioned for a travel history to a malaria endemic area and therefore were considered malaria naïve.

- Gametocytaemia for both macro- and microgametocytes was tested by stage specific qRT-PCR and the results sounds promising for transmission experiments. Transmission experiments to mosquitoes were attempted as exploratory study but only very limited information is provided, and as stated "the results will be shared separately… With no need to provide the full details and results about these experiments it is highly desirable to state if the transmission was achieved or not, this will help validate the model presented.

We are currently preparing a manuscript describing the exploratory transmission studies conducted during the comparative study. We think that adding the results of the transmission studies will distract from the message of this manuscript.

- It would be good to present, in table format, the clinical biochemistry and haematology parameters recorded along the experiments with reference values and highlighting the abnormal ones.

The listings of the biochemistry and haematology parameters are presented in the appendix of the clinical study report. However, it is not common practice to present these listings in a publication – please note that the individual biochemistry listing is 450 pages long, and the individual haematology listing is 206 pages long. Nevertheless, we could include this information in the Supplementary Information if the reviewer considers it necessary. We would also be prepared to provide this information to interested readers via the data sharing agreement.

- Overall, it is an impressive study and an interesting manuscript that contribute to the development of safe and useful human malaria infection models. Thank you.

The authors thank the reviewer for the kind comment.

Reviewer #2

In the proposed document, the authors report the results of a human challenge experiment. I have restricted my comments to the statistical analyses. The methods require additional detail.

- The authors calculate 'The weighted mean of the parasite clearance slope', however, it is not clear what the parasite clearance slope is and what the weights are. This should be clarified.

We have clarified in the text that the parasite clearance slope is the slope of the relationship between log_{10} parasitemia and time, and that the weights are based on the inverse-variance method using the standard errors of the corresponding clearance slope (page 12, lines 254–258):

"The parasite clearance slope is the slope of the linear regression of log_{10} parasitemia over time after artesunate administration, derived from an iterative procedure [13]. The weighted mean of the parasite clearance slope was estimated by the inverse-variance method using the corresponding standard errors of the clearance slope, and presented as $log_{10}PRR_{48}$ and the parasite clearance half-life, as previously described [13]."

- The authors refer to an 'omnibus test', however no reference is made. A brief description of this test should be provided to help the non-specialist reader.

We explain in the text that the omnibus test is used to determine differences between the weighted mean parasite clearance slopes of artemisinin-resistant and artemisinin-sensitive parasites. We have added a reference for readers who may wish to learn more about the omnibus text (page 12, lines 258–260):

"An omnibus test [14] was used to determine differences between the weighted mean parasite clearance slopes of artemisinin-resistant and artemisinin-sensitive parasites."

- The authors conduct a 'sine-wave growth model' to model the viral titers (I believe). The authors should set out why this model was chosen (I presume for the sinusoidal nature of the dynamics). It would also be useful to demonstrate the fit of their model (e.g., plot the observed versus fitted average values).

We chose the sine-wave model to capture the oscillatory profile of parasite growth due to sequestration of the asexual parasites. We have added this information to the manuscript (page 12, lines 262–263):

“Parasite growth dynamics were estimated using a sine-wave model to capture the oscillatory nature of the parasite growth [15].”

The predicted fit of the sine-wave mixed-effects model for the growth of artemisinin-resistant and artemisinin-sensitive infected participants with the observed trajectories for each participant is presented in Fig C in S1 Appendix.

- The authors should clarify what the weights are for the sine-wave growth model.

We have clarified that the weights for the sine-wave growth model were based on the inverse-variance method as detailed in Text E S1 Appendix (page 12, lines 264–267):

“For the pilot study, the sine-wave growth model was estimated for each participant and the lifecycle duration of artemisinin-resistant parasites was estimated from the inverse-variance weighted mean of the sine-wave period estimates (Text E in S1 Appendix).”

- Similarly, the authors state that 'Pharmacokinetic parameters were determined by non-compartmental analysis, however, it is not clear what a non-compartmental analysis represents. The authors should describe what they mean by this and clarify exactly what parameters are being estimated.

Non-compartmental analysis is the standard methodology used to estimate pharmacokinetic parameters. We believe that this standard pharmacometric method does not need to be described in this manuscript.

The pharmacokinetic parameters estimated are described in the Outcomes subheading of the methods section (pages 11–12, lines 238–242):

“Both studies had the exploratory outcome to calculate the following pharmacokinetic parameters for artesunate and DHA: area under the concentration-time curve from time 0 to the last measurable concentration $AUC_{0\text{-}last}$); area under the concentration-time curve from time 0 to infinite time ($AUC_{0\text{-}\infty}$); maximum concentration (C_{max}); time of C_{max} (t_{max}); and elimination half-life ($t_{1/2}$).”

Reviewer #3

This is an impressive study of great importance. Human model for artemisinin resistant malaria could be valuable for a variety of reasons, including (but not limited to) controlled studies of new antimalarials. I have a few small comments, questions, and clarifications that should be addressed.

Major critique

- The limitations should be more comprehensively considered and explained. As I've already stated, this is an impressive amount of work. Pointing out potential limitations to this work does not detract from the quality of work.

We have added other limitations to the text (pages 26, lines 486–493):

"Another limitation of the study is that our results are difficult to generalize to other populations. All the participants in these studies were malaria naïve, and therefore their response to malaria infection may be different to patients in endemic areas, where patients may have different levels of immunity to malaria. In addition, levels of parasitaemia are lower in VIS than in clinical malaria, which further hinders generalisability. Nevertheless, despite these limitations, the delayed clearance profile of artemisinin-resistant parasites after artesunate observed in these studies is comparable to observations from field studies [1-3, 17]."

Currently, the only limitation that the authors discuss is related to the different genetic background between 3d7 and the k13 strain that they chose for this study. No explanation is given for why they chose the strain that they did. No comments are given about testing to see if their hypothesis (that they would have the same results if using a different strain) is true.

We used the parasite strain harbouring the kelch13 gene mutation R539T because this is the strain that was available to us from a naturally infected patient. We considered that this strain was adequate to perform these clinical trials. Although this strain has been out-competed in the Greater Mekong Subregion by the C580Y Kelch variant, it shows significant resistance in the ring-stage survival assay (RSA) (Straimer et al., Science, 2015, 347(6220):428-31).

- No discussion is given about sample sizes or generalizability/representativity of the study population to other populations.

The sample size of this study was the number of participants needed to observe a 30% mean difference in the parasite clearance slope between artemisinin-resistant and artemisinin-

sensitive parasites. We observed a larger difference than 30%, and therefore the study was adequately powered to detect this difference. However, the study was conducted in healthy malaria naïve adult participants and is difficult to generalize the results to other populations.

We have added information about the sample size to the discussion section (page 23, lines 426–428):

"The observed difference in parasite clearance between the artemisinin-resistant and artemisinin-sensitive was larger than the 30% difference in mean parasite clearance rates that the study was powered to detect."

Moreover, we have added information about the generalizability of the study to the discussion section (page 26, lines 486–493):

"Another limitation of the study is the that our results are difficult to generalize to other populations. All the participants in these studies were malaria naïve, and therefore their response to malaria infection may be different to patients in endemic areas, where patients may have different levels of immunity to malaria. In addition, levels of parasitaemia are lower in VIS than in clinical malaria, which further hinders generalisability. Nevertheless, despite these limitations, the delayed clearance profile of artemisinin-resistant parasites after artesunate observed in these studies is comparable to observations from field studies [1-3, 17]."

- On page 19: "However, our studies used malaria naïve participants given a single dose of antimalarial drug, which rules out these confounding effects." Some immunity is inherited rather than acquired, and immunity to malaria in general is not well understood. Were there any tests for blood cell variations among participants? Given that immunity is complex, and not perfectly understood, it is a bit too strong of a statement to suggest that having malaria naïve participants completely accounts for any possible confounding from immunity. Also, the inclusion criteria appears to limit potential malaria exposure to the last year. While some types of malaria immunity are short lived, immunological markers for malaria (msp, csp, etc.) can last for a very long time (years and possibly decades).

We accept that some immunity to malaria is innate rather than acquired, the best example being the haemoglobinopathies such sickle cell trait. Likewise, it is conceivable that one of the participants may have travelled to a malaria-endemic area in the distant past. Of note, however, there was little variation between subjects within groups in the pre-treatment parasite growth rate. Furthermore, the growth rates of the participants inoculated with artemisinin-

sensitive 3D7 in this study was 0.78 log_{10} parasites/day (95% CI: 0.69 – 0.87 log_{10} parasites/day), which is similar to the growth rate estimated using data from 177 participants from previous IBSM studies (0.71 log_{10}parasites/day, 95% CI: 0.67 – 0.74 log_{10}parasites/day) (Wockner et al., J Infect Dis, 2019;221 [6]:963-72).

Testing for malaria-specific antibodies is no longer available through our Red Cross. We do not have access to a validated serologic assay, and we are unaware that any of the other units performing volunteer infection studies do pre-enrolment serologic screening. Although exclusion criteria number 2 (page 31 in the protocol of the comparative study) states that participants must have not travelled or lived more than two weeks in a malaria-endemic region during the past 12 months, exclusion criteria number 1 in addition excludes participants with any history of malaria.

Minor critiques

- Title: Why is the term "volunteer infection study" used rather than "human challenge study"? Are they not the same? The latter is more commonly used and would be more clearly understood by most infectious disease specialists.

We use the term "volunteer infection study" to conform to the nomenclature adopted by Medicines for Malaria Venture. Please find MMV website in the following link: https://www.mmv.org/newsroom/infographics/volunteer-infection-studies

- Introduction: "This drug resistant phenotype is linked to specific mutations…" It would be a bit more accurate to describe this as mutations in a specific genome region, rather than specific mutations, since there are several different mutations in the kelch region that are associated with the phenotype of delayed parasite clearance.

We have changed the wording in the Introduction as suggested by the reviewer (page 6, lines 98–99):

"This drug-resistant phenotype is linked to a number of specific non-synonymous mutations in the Plasmodium falciparum kelch13 gene [3,4]."

- In the final paragraph of page 4, it would help if there was a sentence explaining that both the pilot and the comparative (second) study are being presented here. It becomes clear in the methods and results sections, but it would be good to state up front that this manuscript presents the results from both a pilot study and a follow-up study. A simple: "Here we present results from… " type statement here would work.

The authors agree with the reviewer. We have added the following sentence to the beginning of the last paragraph of the introduction (page 6, line 112):

"Here, we present the results from two clinical trials. First, we conducted a pilot study…"

- Why was the R539T mutation chosen? There are others of higher concern…

We used the parasite strain harbouring the kelch13 gene mutation R539T because this is the strain that was available to us from a naturally-infected patient. We considered that this strain was adequate to perform these clinical trials. As we note above, since Kelch mutations first emerged, parasites containing the C580Y mutation have outcompeted those carrying the R539T mutation. However, their RSA phenotype indicates artemisinin resistance.

- Page 6: It is a bit confusing to have the K13 inoculants on day 0 and 3d7 inoculants on day 1. Later you give a brief explanation for why, but it would add some clarity if it was explained here.

We have added to the methods a sentence explaining the difference in the inoculation day for artemisinin-resistant and artemisinin-sensitive infected participants (page 9, lines 175–177):

"Artemisinin-resistant infected participants were inoculated a day earlier than artemisinin-sensitive infected participants so that parasites were at a similar stage of development at the time of artesunate administration (see results section for more detail)."

- Page 9: The pharmacokinetic parameters for artesunate and DHA should be explained. Some readers will not immediately recognize the abbreviations.

We have explained the pharmacokinetic parameters as suggested for the reviewer (pages 11–12, lines 239–242):

"area under the concentration-time curve from time 0 to the last measurable concentration ($AUC_{0\text{-}last}$); area under the concentration-time curve from time 0 to infinite time ($AUC_{0\text{-}\infty}$); maximum concentration (C_{max}); time of C_{max} (t_{max}); and elimination half-life ($t_{1/2}$)."

- Page 12: Regarding the difference in parasite life cycle duration, which led to starting the K13 infected participants a day earlier… this deserves at least a statement or two in the conclusion. Also, if life stages are normally at different lengths, does this not influence the comparison of the 3d7 and the K13 resistant parasitemias? Or is this accounted for with the use of the mixed effects model? Please explain.

The sine-wave growth model was applied to the pilot study to estimate the parasite growth parameters of artemisinin-resistant parasites, including the parasite lifecycle duration. We compared the growth profile of artemisinin-resistant parasites with the growth profile of artemisinin-sensitive parasites. The growth profile of artemisinin-sensitive parasites was estimated using a sine-wave mixed effects model using data from previous studies inoculated with the 3D7 parasite, using a subset of data reported by Wockner et al., J Infect Dis, 2019;221 (6):963-72.

The observed difference in the growth profiles of the two parasite strains suggested that inoculating participants with artemisinin-resistant parasites a day earlier than participants with artemisinin-sensitive parasites should account for the different lengths of parasite lifecycle. Therefore, by inoculating participants with artemisinin-resistant parasites a day earlier, we have compensated for the differences in the lifecycle duration and ensured that artesunate was administered at the same lifecycle stage. Therefore, the difference in lifecycle duration are not expected to influence the comparison of the artemisinin-resistant and artemisinin-sensitive parasites.

We have added to following text to the discussion (page 24, lines 443–449):

"In the comparative study, we inoculated artemisinin-resistant infected participants a day earlier than artemisinin-sensitive infected participants to account for the longer duration of the parasite lifecycle of the artemisinin-resistant parasite, as estimated with the data from the pilot study. By inoculating artemisinin-resistant infected participants one day earlier, we aimed to administer artesunate at the same stage of parasite lifecycle for both strains. Therefore, the difference in lifecycle duration between the two strains is not expected to influence the comparison of parasite clearance."

- Page 12: Any explanation for the massive difference in parasitemia between the K13 lineage and 3D7 (with parasitemia over 2 times larger)?

The differences in parasitemia at time of artesunate administration is within the expected variability we observe between cohorts inoculated with different inocula preparations. This variability is also observed with the same parasite strain, as has previously been shown for 177 individuals inoculated with the artemisinin-sensitive strain in Wockner et al., J Infect Dis, 2019;221 (6):963-72 (see Figure 1 in the cited paper). Although the absolute difference in parasitaemia was high, a Mann-Whitney U test suggests that the difference in parasitaemia at the time of treatment was not significant (p=0.13). Furthermore, the range of the parasitemia

within each group are comparable, which suggests that the observed variability is comparable (pages 16–17, lines 318–321):

"Median parasitaemia prior to artesunate administration for artemisinin-resistant infected participants was 71427 parasites/mL (range 17611–346298 parasites/mL) and for artemisinin-sensitive infected participants 31400 parasites/mL (range 10625–393678 parasites/mL). "

- Page 14: This isn't a minor point, but consider giving a quantitative value rather than stating that clearance was "significantly longer" - for example, it looks like clearance was twice as long for the k13 resistant infected participants when compared to the 3d7 infected participants.

We have changed the text to provide a quantitative value (page 18, lines 352–355):

"In the comparative study, parasite clearance half-life in artemisinin-resistant infected participants after artesunate administration was twice as long than in artemisinin-sensitive infected participants (6.5 hours vs 3.2 hours; p<0.0001; Table 2, Table G in S1 Appendix)."
