# Supplementary material for: Safety and parasite clearance of artemisinin-resistant Plasmodium falciparum infection: A pilot and a randomised volunteer infection study in Australia
Source: PLoS Med. 2020 Aug 21;17(8):e1003203. doi: 10.1371/journal.pmed.1003203 (PMC7444516; doi:10.1371/journal.pmed.1003203)
Supplement: Attachment — Submitted filename: Watts_et_al_responses_17Jul20.docx [file pmed.1003203.s028.docx]

**PMEDICINE-D-20-00295R3: Response to comments**

**Response to comments: 12 June 2020**

FIGURES and GRAPHICS:

1. Please ensure that all main figure files are cited in ascending numerical order in the main text of the article.

*Response: We have confirmed that all main figures are cited in ascending numerical order.*

2. Please ensure that all main figures are referenced in the manuscript as Fig 1, Fig 2, etc. (including capitalization), rather than Figure 1, Figure 2, etc. Please note, however, that the file names themselves must not include the space, i.e. Fig1.tif, Fig2.tif, etc.

*Response: We have confirmed that all main figures are references correctly in the manuscript and the files are named correctly.*

3. Please ensure that all main figure legends are placed after the paragraph containing their first in-text citation.

*Response: We have confirmed that all main figure legends are placed after the paragraph containing their first in-text citation.*

4. Please note that your figures do not currently comply with our publication requirements. For further instructions to assist with your Figure revisions, please refer to our Figure Guidelines at: <http://journals.plos.org/plosmedicine/s/figures>.

If you need assistance, please contact us directly at [graphics@jjeditorial.com](mailto:graphics@jjeditorial.com) so we can further help with this matter. Please send any relevant source files and tell us what application(s) and what operating system you are using.

-Figures 1-3 have text that looks smaller than 8 points. The legibility of type at sizes smaller than 8 points in the published PDF is frequently compromised. We cannot assume responsibility for the legibility of Figures in the published PDF if there is type smaller than 8 point in them. Please note that this problem will not be detected or corrected by the PACE tool. We recommend that you print figures out to get a better idea of the text legibility. If you are happy with the way they look, we will publish them with the font as is.

-Figures 1 and 3 have been submitted in a layered TIF file format which we don’t accept for Figures. To ensure that your Figures publish properly, please flatten the layers in each Figure file (Layer→Flatten Image in Photoshop, Image→Flatten Image in GIMP) and re-upload. Make sure to save your TIF files with LZW Compression turned on.

-Please resave Figure 2 using LZW compression (loss-less) in order to reduce the file size by over 75% and re-upload.

In Photoshop: File→Save As. Save as TIFF, Image Compression set to LZW, Pixel Order set to Interleaved, Byte Order set to IBM PC.

In GIMP: File→Save As. Click the + sign next to Select File Type (By Extension). From the menu that appears, select TIFF. Click Save. Set Compression to LZW. If you're prompted about layers in the file, select Flatten Image.

*Response: The figures have been amended as requested.*

TABLES:

1. Please cite main tables in ascending numerical order in your main article file.

*Response: We have confirmed that all main tables are cited in ascending numerical order.*

2. Please ensure that all main tables in their entirety (including titles and footnotes), are placed after the paragraph containing their first in-text citation.

*Response: We have confirmed that all main tables are placed after the paragraph containing their first in-text citation.*

3. All tables must be numbered in whole numbers and not parts. Please either combine or renumber your lettered tables as appropriate.

*Response: We have confirmed that all tables are numbered in whole numbers and not parts.*

4. Tables 1 and 3 use returns to control text wrapping. This is indicated by a [¶ or ↵] symbol when "Show Paragraph Marks" is turned on in Word. Please either split those cells into separate cells to achieve alignment or remove the returns.

*Response: The tables have been amended as requested.*

METADATA:

1. Your co-author, Dr. Chalon, has declared the following competing interest in relation to this manuscript. Please include it in the main competing interest statement in our system, sticking as closely as possible to Dr. Chalon's wording, using only their initials.

"I am an employee of Medicines for Malaria Venture (which has provided funding for the study)"

*Response: The competing interests for Dr. Chalon and Dr. Möhrle in relation to their employment with Medicines for Malaria Venture has been declared in the main competing interest statement in the system.*

2. In accordance with PLOS' data policy, please ensure that your Data Availability Statement in the submission form clearly identifies how readers can access your data. Data cannot be available on request, or only accessible by contacting one of the authors. Note that your Data Availability Statement will be typeset as it is written, so please ensure it is in complete sentences and appears as you would like it to in the published version.

For details on our data policy, please see <http://journals.plos.org/plosmedicine/s/data-availability>

*Response: The Data Availability Statement has been modified as follows to comply with PLOS' data policy:*

*“All raw data obtained in the two clinical trials described are included in S1 Appendix (pilot study) and S2 Appendix (comparative study).”*

3. In the online submission form, you indicated that "Data collected for this study will be made available immediately after article publication with no end date. De-identified datasets containing the variables analysed for the primary and secondary outcomes will be made available as well as other supporting documents (e.g., protocol and informed consent). Investigators who seek access to individual participant data will contact Prof. James S. McCarthy and the QIMR Berghofer Data Manager ([Ria.Woo@qimrberghofer.edu.au](mailto:Ria.Woo@qimrberghofer.edu.au)), who will send them the data via a secure data sharing platform." All PLOS journals now require all data underlying the findings described in their manuscript to be freely available to other researchers, either 1. In a public repository, 2. Within the manuscript itself, or 3. Uploaded as supplementary information.

This policy applies to all data except where public deposition would breach compliance with the protocol approved by your research ethics board. If your data cannot be made publicly available for ethical or legal reasons (e.g., public availability would compromise patient privacy), please explain this by return email and staff will assist you with completing your Data Availability Statement.

At this time, we ask that you please explain, with as much detail as possible, why you are unable to make your data available in one of the three places mentioned above so we can further assess whether or not this complies with PLOS’ Data Policy and determine how we can assist you in this matter.

*Response: As above, we have now modified our Data Availability Statement to comply with PLOS' data policy. All raw data obtained in the two clinical trials described in the manuscript has been uploaded as supplementary information (S1 Appendix and S2 Appendix).*

ARTICLE FILE:

1. Please indicate the corresponding author in the byline by placing an asterisk (*) after their affiliation number.

*Response: The corresponding author has been indicated as requested.*

2. Please include the corresponding author's email address on the title page of your manuscript, indicated by an asterisk (*). Only an asterisk and the email address itself are required.

*Response: The corresponding author's email address* *has been included as requested.*

3. Please remove the Competing Interests section from your manuscript file. The published version is pulled automatically from the "Competing Interests" field in the submission form and must not be duplicated. Please therefore take this opportunity to check that the information in this field is correct and completed in full.

*Response: The* *competing Interests section has been removed as requested.*

4. Please remove your Author Contributions section from your manuscript file as this information is automatically pulled from the authors section of our submission form and must not be duplicated. Please therefore take this opportunity to check that the information in this field is correct and completed in full.

*Response: The* *author contributions section has been removed as requested.*

5. Please remove the data availability information from your manuscript file as this is automatically pulled from the "Data Availability" field in the submission form and must not be duplicated. Please therefore take this opportunity to check that the information in this field is correct and completed in full.

*Response: The* *data availability section has been removed as requested.*

6. Please ensure that your Ethics statement is available in the Methods section of your manuscript in its entirety.

*Response: We have confirmed that the ethics statement is included in the Methods section.*

REFERENCES:

1. Please ensure your references are in the style of PLOS Medicine (ICMJE/Vancouver style). The reference style guide can be found here: <http://journals.plos.org/plosmedicine/s/submission-guidelines#loc-references>. You can also find a downloadable EndNote file in these guidelines to assist you. Please do a manual check as well, as the EndNote file does not catch everything. Please note that the references will be typeset as they appear so please ensure the format is consistent.

*Response: We have confirmed that the references are in the style of PLOS Medicine.*

2. Please put all reference citations in square brackets separated by either commas or dashes with no spaces, e.g. [1,2,3] or [1-3].

*Response: We have confirmed that all references are cited* *in square brackets separated by either commas or dashes with no spaces.*

3. Please make sure all references are cited in ascending numerical order in the text.

*Response: We have confirmed that all references are cited in ascending numerical order in the text.*

SUPPORTING INFORMATION:

1. Please ensure that all Supporting Information files use the following format verbatim throughout the manuscript (including legends and all in-text citations): S1 Fig, S1 Table, S1 Text, S1 Data, etc. Each file type should start at 1 and increase numerically. Please note, however, that the file names themselves must use an underscore rather than a space, i.e. S1_Fig.tif, S1_Table.xlsx, S1_Text.doc, S1_Data.xlsx, S1_PRISMA_Checklist.docx, etc.

*Response: We have ensured that the supporting information files and in-text citations follow the naming convention specified.*

2. Please ensure that each supporting information file has a legend at the end of the manuscript file, after the Reference list.

*Response: We have added the legend for each supporting information file* *at the end of the manuscript file.*

3. Please note that your submission cannot contain any references to generic "supporting information" files and instead must refer to specific files using our naming convention (S1 Fig, S1 Table, S1 Text, S1 Data, S1 PRISMA Checklist, etc.)

*Response: We have included the supporting information as specific individual files as requested.*

**Response to comments: 19 June 2020**

1. Figure 1 is ready for publication.

-Please note that your figures do not currently comply with our publication requirements. For further instructions to assist with your Figure revisions, please refer to our Figure Guidelines at: <http://journals.plos.org/plosmedicine/s/figures>.

If you need assistance, please contact us directly at [graphics@jjeditorial.com](mailto:graphics@jjeditorial.com) so we can further help with this matter. Please send any relevant source files and tell us what application(s) and what operating system you are using.

-Figure 2 has text that looks smaller than 8 points. The legibility of type at sizes smaller than 8 points in the published PDF is frequently compromised. We cannot assume responsibility for the legibility of Figures in the published PDF if there is type smaller than 8 point in them. Please note that this problem will not be detected or corrected by the PACE tool. We recommend that you print figures out to get a better idea of the text legibility. If you are happy with the way they look, we will publish them with the font as is.

-Figure 3 looks like it was originally at a lower resolution and will appear blurry or pixelated if published. Text and images should appear crisp and clear. Please note that this problem will not be detected or corrected by the PACE tool, and this will not be fixed by simply increasing the resolution of your Figures in the image editing software. Please carefully review the PLoS Figure Guidelines and provide Figure files that were originally created at 300 pixels/inch (dpi) and at least 6.68cm wide, but less than 19.05cm and under 22.225cm tall. For further information, please see the Figure Guidelines (<http://journals.plos.org/plosmedicine/s/figures>).

1) Export a PDF from PowerPoint/Word/Excel (or other image source file). On a Mac, click on the 'PDF' button in the printer dialog box and select 'Save as PDF'. On a PC, send the file to print and select 'Adobe PDF' or 'PDFCreator' (it's freeware) from your list of available printers.

If you are using Adobe PDF, then go to Printer Properties, Default Settings and select Press Quality.

If you are using PDFCreator, Go to 'Printer Properties' and then click on 'Advanced' on the bottom right. Under 'Graphic', go to 'Print Quality' and select 600dpi.

2) PHOTOSHOP: When you open up the PDF, make sure that the 'Constrain Proportions' box is checked. Set the 'Resolution' to 300dpi (118.11 pixels/cm) and set the 'Bit Depth' to 8 bit. Use the crop tool (next to the eyedropper) to remove unnecessary white space. Go to - Image - Image Size. Make sure the 'Constrain Proportions' and 'Resample Image” boxes are checked.

To ensure that your Figures publish properly, please delete the alpha channels from each Figure file. In Photoshop, select it in the Channels Palette and click on the trash can icon. When you save the TIFFs make sure that the 'LZW' button is checked and 'Discard Layers and Save a Copy' button is checked.

3) GIMP: When you open up the PDF, set the resolution to 300. Make sure the chain link icon is joined together. Then change the size of your figure according to the pixel specifications mentioned in step 2) above. To ensure that your Figures publish properly, please delete the alpha channels from each Figure file. In GIMP, go to Layers - Transparency - Remove Alpha Channel. When you are done, 'SAVE AS' (or Export if GIMP 2.8). Select File Type - 1st TIFF option. Make sure to use LZW compression.

4) Print out your figures on a desktop printer. Text and graphics should appear clear and crisp with no pixelated or blurred edges.

5) If the resulting figures are still blurry, then your original source images are too low of a resolution. Please increase the resolution of your original source images, and repeat this process.

*Response: Figures 2 and 3 have been amended as requested.*

2. Please provide a point-by-point response to the following points from the editor's decision letter:

Requests from Editors:

In the attached protocol documents, some of the authors are listed as having positions with "Q Pharm", which is apparently a company (CRO). If they have been paid by Q Pharm, I think this should be included in the competing interest statement.

*Response: None of the authors received payment from Q-Pharm. Two authors (JMc and AO, the principal investigator and associate investigator respectively) were visiting medical officers at Q-Pharm during the study; they were not employed by Q-Pharm and received no financial compensation from Q-Pharm. There are no conflicts of interest to declare.*

The data statement needs to be updated to note how the authors will make the aggregate data available; also please remove the author contact as this is not permitted.

*Response: The data sharing statement has been modified as follows:*

*“All raw data obtained in the two clinical trials described are included in S1 Appendix (pilot study) and S2 Appendix (comparative study).”*

Line 51 and elsewhere please add in to our knowledge re "the first"

*Response: Lines 49, 84, 416, 496 have been amended to indicate that:*

*“We developed the first* ***(to our knowledge)*** *human model of artemisinin-resistant malaria”*

Line 146 – can you clarify what you mean by ‘which was finalised before the data was locked for analysis.’

*Response: The planned statistical analysis of the data was determined prior to availability of the final data listings.*

Please remove all trademark signs

*Response: All trademark signs have been removed as requested.*

It seems from the protocol that the volunteers received money; Please mention this in the methods section

*Response:* *The methods section (line 149) has been modified as follows:*

*All participants gave written informed consent before enrolment* ***and were compensated financially for their involvement in this study.***

If I understand correctly the authors cannot rule out a small risk of blood-borne pathogen transmission in this system, and they could mention this in the methods section.

*Response: WE have added the following sentence to the methods section (line 169):*

*“Although the master cell bank was screened for blood-borne pathogens, the risk of pathogen transmission cannot be completely excluded with any blood transfusion, although the risk should be considerably lower than a single unit blood transfusion where the volume transfused is >5 logs higher.”*

- At line 379, we learn that 7 participants had "severe adverse events related to malaria". This seems to have been missed in the abstract and author summary which note that there were "no serious adverse events". Please amend.

*Response: Severe adverse events are not equivalent to serious adverse events. Distinct criteria were pre-defined in the study protocol for what constitutes a serious adverse event (results in death, hospitalisation, is life threatening etc.). The severity of an adverse event was graded in accordance with Common Terminology Criteria for Adverse Events v4.03 (grade 1-5, where 1=mild, 2=moderate, 3=severe, 4=life-threatening, 5=death). Thus, it is possible to have “severe” adverse events that are not “serious”. The classification of adverse events in this manner is typical for clinical trials.*

Please ensure the abstract is accurate on safety events. Please quote the severe adverse events up-front (including the 2 cardiac events, incidentally) and noting that these were not clinically serious (in their assessment)

*Response: The following text has been added to the abstract (line 42):*

*“Seven participants experienced transient severe falls in white cell counts and/or elevations in liver transaminase levels which were considered related to malaria. Additionally, two participants developed ventricular extrasystoles that were attributed to unmasking of a predisposition to benign fever-induced tachyarrhythmia.”*

Where the authors claim that the method can be "safely used", they can perhaps modify this by acknowledging the small size of their study

*Response: We have modified the abstract (line 55) to state:*

*“****Although based on a relatively small sample size****, results indicate that this model can be safely used to assess new drugs against artemisinin-resistant P. falciparum.”*

*We have also modified the author summary (line 88) to state:*

*“We have developed the first (to our knowledge) human model of artemisinin-resistant malaria, the results of which,* ***although based on a relatively small sample size****, indicate potential to be safely used to assess the efficacy of new drugs against artemisinin-resistant parasites.”*

*Finally, we have modified the discussion (line 503) to state:*

*“****Although based on a relatively small sample size****, results indicate that this artemisinin-resistant IBSM model is safe and replicates the delayed parasite clearance phenotype documented in the field.”*

Please adjust statistics to style: p<0.0001 -> p<0.001 in abstract and at least one table

*Response: The statistics style has been adjusted as requested in the abstract (line 45) and results section (line 351 and Table 2).*

In the revised doc, when resubmitting, please add the marked up version as a Supp file instead of including with the clean doc in the main file.

*Response: The marked up version of the resubmitted manuscript has been uploaded by selecting the “Marked up Manuscript” in the drop down menu.*

Comments from Reviewers:

Reviewer #1: Dear authors and editors,

The revision of the manuscript now titled "Safety and parasite clearance of artemisinin-resistant Plasmodium falciparum infection: A pilot and a randomised volunteer infection study in Australia", presents an impressive piece of work based on a large amount of data and detailed studies.

Encompassing, detailed answers to the reviewers' comments and the editorial requests, this revision has definitely gains in clarity, precision and readability.

A single minor comment: since the study is based in Australia in the "theoretical risk zone" for autochthonous Babesia microti, did serology for Babesia was performed in the volunteers accounting that Babesia is often asymptomatic and some cross-protective immunity exists with Plasmodium? This could be mentioned.

*Response: We did not screen the volunteers for Babesia in this study given that transmission of B. microti is currently uncommon in Australia, as evidenced by the following study:*

*Faddy HM, et al., 2019. No Evidence for Widespread Babesia Microti Transmission in Australia. Transfusion 59:2368-2374.*

Overall, this a convincing study presenting a new and valuable tool in the field of Plasmodium falciparum artemisinin-resistance. This tool will definitely be useful for further studies and has the potential to accelerate the development and evaluation of new anti-malarial drugs efficient against ART-R parasites which urgently needed. I will be happy to it published.

**Response to comments: 16 July 2020**

1. Please ensure that all authors have at least one affiliation listed on the title page and that all listed affiliations are attributed to at least one author. Affiliations should be listed in ascending numerical order in the affiliation list and should match what is provided in the Authors Information in the submission form.

Affiliations should include an institution, a city, and a country (no postcode). US affiliations need the state as well, written out in full (with no abbreviations).

*Response: The affiliations list has been modified to ensure it meets these specifications.*

2. Figure 3 has text that looks smaller than 8 points. Specifically, the axis labels like "Study Day." The legibility of type at sizes smaller than 8 points in the published PDF is frequently compromised. We cannot assume responsibility for the legibility of Figures in the published PDF if there is type smaller than 8 point in them. Please note that this problem will not be detected or corrected by the PACE tool. We recommend that you print figures out to get a better idea of the text legibility. If you are happy with the way they look, we will publish them with the font as is.

*Response: Figure 3 has been modified to increase the text size of the axes. The figure has been printed and appears acceptable.*
